# Rapid high-resolution melting genotyping scheme for *Escherichia coli* based on MLST derived single nucleotide polymorphisms

**DOI:** 10.1038/s41598-021-96148-3

**Published:** 2021-08-16

**Authors:** Matej Bezdicek, Marketa Nykrynova, Karel Sedlar, Stanislava Kralova, Jana Hanslikova, Aja Komprdova, Helena Skutkova, Iva Kocmanova, Jiri Mayer, Martina Lengerova

**Affiliations:** 1 Department of Internal Medicine, Hematology and Oncology, University Hospital Brno, Brno, Czech Republic; 2 Department of Internal Medicine, Hematology and Oncology, Masaryk University, Brno, Czech Republic; 3 Department of Biomedical Engineering, Faculty of Electrical Engineering and Communication, Brno University of Technology, Brno, Czech Republic; 4 Department of Clinical Microbiology and Immunology, University Hospital Brno, Brno, Czech Republic; 5 Department of Experimental Biology, Czech Collection of Microorganisms, Faculty of Science, Masaryk University, Brno, Czech Republic

**Keywords:** Clinical microbiology, Infectious-disease epidemiology, Bacteriology

## Abstract

Routinely used typing methods including MLST, rep-PCR and whole genome sequencing (WGS) are time-consuming, costly, and often low throughput. Here, we describe a novel mini-MLST scheme for *Eschericha coli* as an alternative method for rapid genotyping. Using the proposed mini-MLST scheme, 10,946 existing STs were converted into 1,038 Melting Types (MelTs). To validate the new mini-MLST scheme, in silico analysis was performed on 73,704 strains retrieved from EnteroBase resulting in discriminatory power D = 0.9465 (CI 95% 0.9726–0.9736) for mini-MLST and D = 0.9731 (CI 95% 0.9726–0.9736) for MLST. Moreover, validation on clinical isolates was conducted with a significant concordance between MLST, rep-PCR and WGS. To conclude, the great portability, efficient processing, cost-effectiveness, and high throughput of mini-MLST represents immense benefits, even when accompanied with a slightly lower discriminatory power than other typing methods. This study proved mini-MLST is an ideal method to screen and subgroup large sets of isolates and/or quick strain typing during outbreaks. In addition, our results clearly showed its suitability for prospective surveillance monitoring of emergent and high-risk *E. coli* clones’.

## Introduction

*Escherichia coli* is a gram-negative bacteria commonly colonizing the human gastrointestinal tract as a harmless commensal microorganism. However, it is also one of the major human pathogens causing mainly urinary tract infections, bloodstream infections, neonatal sepses, skin structure infections and traveller’s diarrhea^1^. Together with the globally increasing rates of antibiotic resistance including *E. coli* strains, extraintestinal infections caused by this opportunistic pathogen represent one of the main public health problems.

Bacterial genotyping is a powerful tool for strain identification, outbreak investigation and surveillance applications. Electrophoretic techniques such as pulse-field gel electrophoresis (PFGE) or repetitive element sequence-based PCR (rep-PCR) have been considered the gold standard methods for bacterial strain tracking for many years^2^. Although PFGE is a highly discriminatory and robust method, its disadvantages such as extreme time and labor demands combined with potential resolution issues and inconsistencies in interpretation make it difficult to standardize between laboratories. In contrast, sequence-based typing such as multilocus sequence typing (MLST) provides a robust, portable, interlaboratory system based on sequencing 6–8 housekeeping genes^3^. Despite its costs and demands on time, MLST has become a typing technique used worldwide to track the spread of virulent and antibiotic-resistant strains on a local and global level. Recently, whole genome sequencing (WGS) has been introduced and has become a widely used sequence-based genotyping tool. Along with the rapid development, WGS technology has also become more accessible for routine applications. However, it is still impractical for prospective typing and/or screening typing larger numbers of strains. In particular, data processing and their evaluation are currently WGS’ main disadvantage, demanding both extra computational and human resources.

Another method that can be used for genotyping is high-resolution melting (HRM). HRM is a highly specific and sensitive method to discriminate variants in PCR amplicons^4^. It is based on dsDNA’s melting separation with monitoring via changes in fluorescence intensity, which correlates with the CG content and amplicon length. Bacterial strain typing using HRM detection of single nucleotide polymorphisms (SNPs), called mini-MLST or minim typing, has already been successfully applied to *Klebsiella pneumoniae*^5^, *Staphylococcus aureus*^6^, *Enterococcus faecium*^7^ and *Streptococcus pyogenes*^8^. These methods are based on detecting allelic specific SNPs derived from well-established MLST schemes used worldwide. Regarding *E. coli*, methods based on HRM were already used for multiple purposes such as species identification^9^, detecting and quantifying of enterotoxigenic virulence factors^10^, detecting of AmpC, ESBL and carbapenemase genes^11^ and in combination with ligation-mediated real-time PCR for molecular typing on a local level^12^.

Here we describe a novel mini-MLST scheme for *E. coli* as an alternative method for rapid genotyping suitable for routine clinical practice. We compared the proposed mini-MLST with commonly used molecular typing such as REP-PCR and MLST on an outbreak of *E. coli* at the Neonatal Department at the University Hospital Brno (UHB). In addition, our novel mini-MLST scheme was also compared to WGS on *E. coli* strains collected during a surveillance study at the Department of Hematology and Oncology (UHB).

## Results

### Method design

Firstly, all SNPs in the *E. coli* MLST^13^ loci were identified using Minimum SNPs software^14^. Consequently, the SNPs that do not represent a nucleotide exchange affecting the number of hydrogen bonds (A/T ↔ G/C) were excluded, because these are not commonly detectable by HRM. All SNPs without flanking conserved regions were also excluded from future analyses as those are crucial for primer design. Simpson’s Index of Diversity (D) was calculated. For all the remaining SNPs. Finally, a set of six regions containing highly informative SNP sets meeting our criteria was found in 6 different MLST loci. The regions were labelled as (locus name + first and last position of region of interest) *adk*288-368, *fumC*227-390, *gyrB*147-191, *icd*144-218, *purA*254-334 and *recA*82-143 (Table 1). No D-optimized SNPs in the *mdh* could be found to meet our mini-MLST design criteria. The amplified fragments sizes ranged between 85 to 152 bp. The total number of alleles (n) and D values were calculated to determine the discriminatory power of each loci containing the targeted SNPs as follows: *adk*288-368 (n = 161, D = 0.8784, CI 95% 0.8657–0.8911), *fumC*227-390 (n = 456, D = 0.9605, CI 95% 0.9561–0.9649), *gyrB*147-191 (n = 81, D = 0.7380, CI 95% 0.7111–0.7649), *icd*144-218 (n = 179, D = 0.8664, CI 95% 0.8536–0.8793), *purA*254-334 (n = 144, D = 0.8182, CI 95% 0.7956–0.8408) and *recA*82-143 (n = 131, D = 0.7175, CI 95% 0.6829–0.7520).

**Table 1:** Target SNP positions, primer information and predicted melting alleles for mini-MLST scheme.

Gene		Target region	Primer ID	Primer sequence 5′–3′	Amplicon length (bp)	Predicted alleles (based on GC content)
*adk*	288–368		adk322 FW	GGCATCAATGTTGATTACGTTC	122	43, **44**, **45**, **46**, **47**, 48, 49, 52
			adk322 RV	GGCGGATTGAATTTAACGT		
*fumC*	227–390		fumC327 FW	CTGCGCAAGCAACTCATTC	152	50, 51, 52, 55, 56, 57, **58**, 59, 60, **61**, **62**, **63**, 64, 65
			fumC327 RV	GCTACCCAGCCGGAAATCT		
*gyrB*	147–191		gyrB174 FW	GTTACCGGCGAGACTGAAAA	85	22, 23, **24**, **25**, **26**, **27**, 28
			gyrB174 RV	ATTCGAACTCGGTCACATTG		
*icd*	144–218		icd174 FW	TCTGATTCGTGAATATCGCG	116	41, 42, **43**, **44**, **45**, 46, **47**, 48
			icd174 RV	GCAGGCAGATGTAGAGATCCA		
*purA*	254–334		purA263 FW	CTGTTGCCGACATCCTGAC	120	**43**, **44**, **45**, **46**, **47**, 48, 53
			purA263 RV	GGTCGATATCCAGCAGCGTA		
*recA*	82–143		recA91 FW	CAGGCACTGGAAATCTGTGA	100	34, **38**, **39**, **40**, **41**, 42, 43, 44, 45, 46
			recA91 RV	CTTCGATTTCCGCTTTCG		

Allele prediction is based on the total number of GC bases between primers in the targeted region. Alleles captured in our sample set are shown in bold.

We used our in-house MLST2MELT software to predict mini-MLST alleles for all 6 regions (Table 1) and generate a MelT key to work as a conversion key between STs and MelTs. In 3/2021, 10,946 existing STs were converted into 1,038 MelTs. Out of a total of 10,946 STs, 39 STs were not assigned to a MelT. ST1942 and ST339’s MLST schemes contain alleles missing in the source database. Additionally, the following alleles carry a high polymorphism or deletion content in the primer binding region: *adk* 44; *fumC* 42, 255, 256, 624, 683, 684, 808, 809, 1012, 1021, 1030, 1050, 1216, 1273, 1594, 1664; *gyrB* 212, 213, 311, 331,354, 427, 428, 741, 747, 1024, *icd* 40, 1306 and *purA* 99, 428. In the Melt Key, STs containing these alleles are assigned to the MelT0 and appended with a note indicating that the certain region may fail to amplify, or the ST may carry an allele missing from the MLST database.

### Method validation

**Figure 1:**
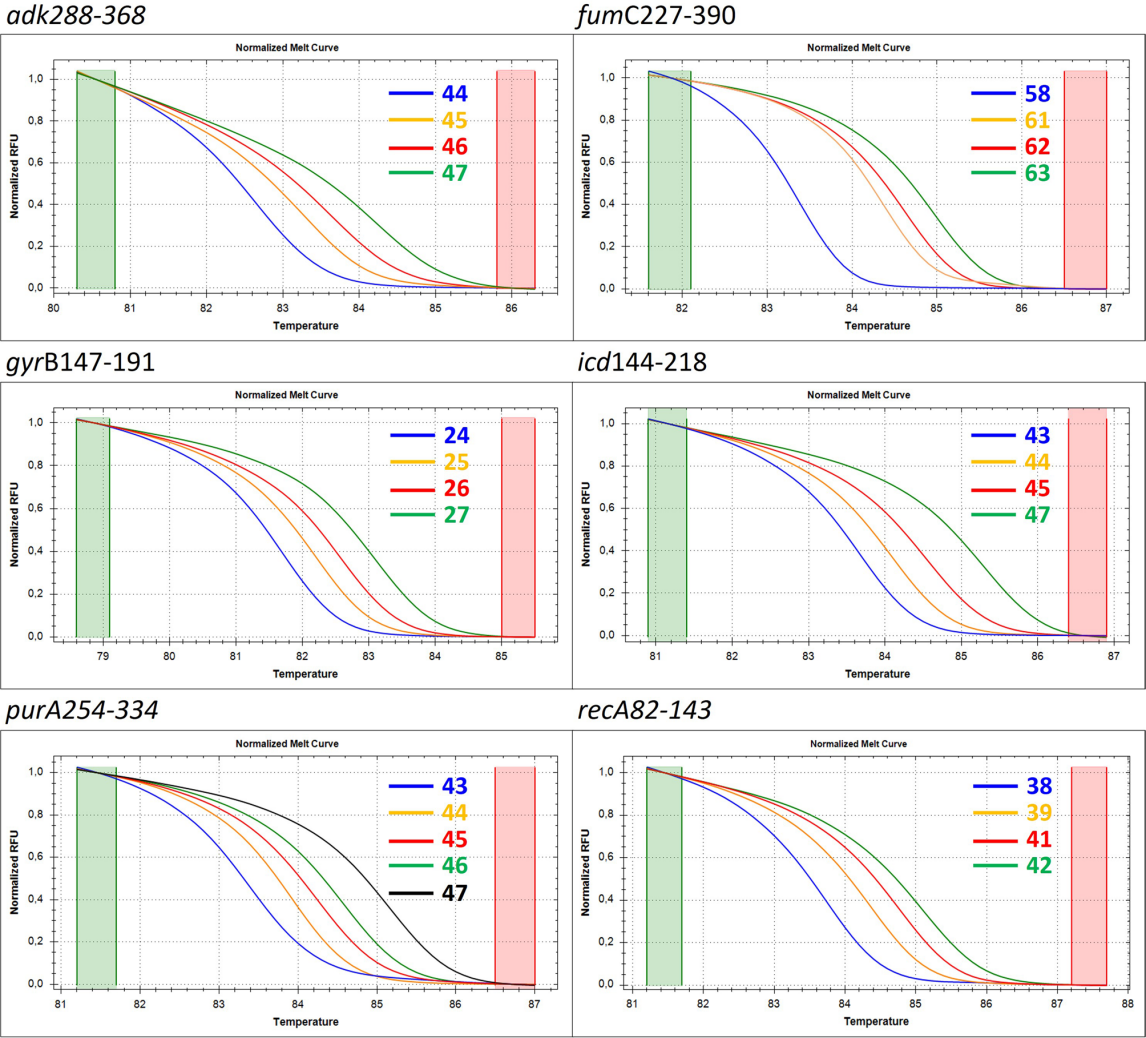
High-resolution melting curves for each of six mini-MLST loci. The curves are labeled with the GC content in corresponding mini-MLST loci. For locus *adk*288-368, four (44, 45, 46, 47) out of eight predicted alleles are displayed. For locus *fum*C227-390, four (58, 61, 62, 63) out of 14 predicted alleles are displayed. For locus *gyr*B147-191, four (24, 25, 26, 27) out of seven predicted alleles are displayed. For locus *icd*144-218, four (43, 44, 45, 47) out of eight predicted alleles are displayed. For locus *purA*254-334, five (43, 44, 45, 46, 47) out of seven predicted alleles are displayed. For locus *recA*82-143, four (38, 39, 40, 41) out of 10 predicted alleles are displayed.

**Figure 2:**
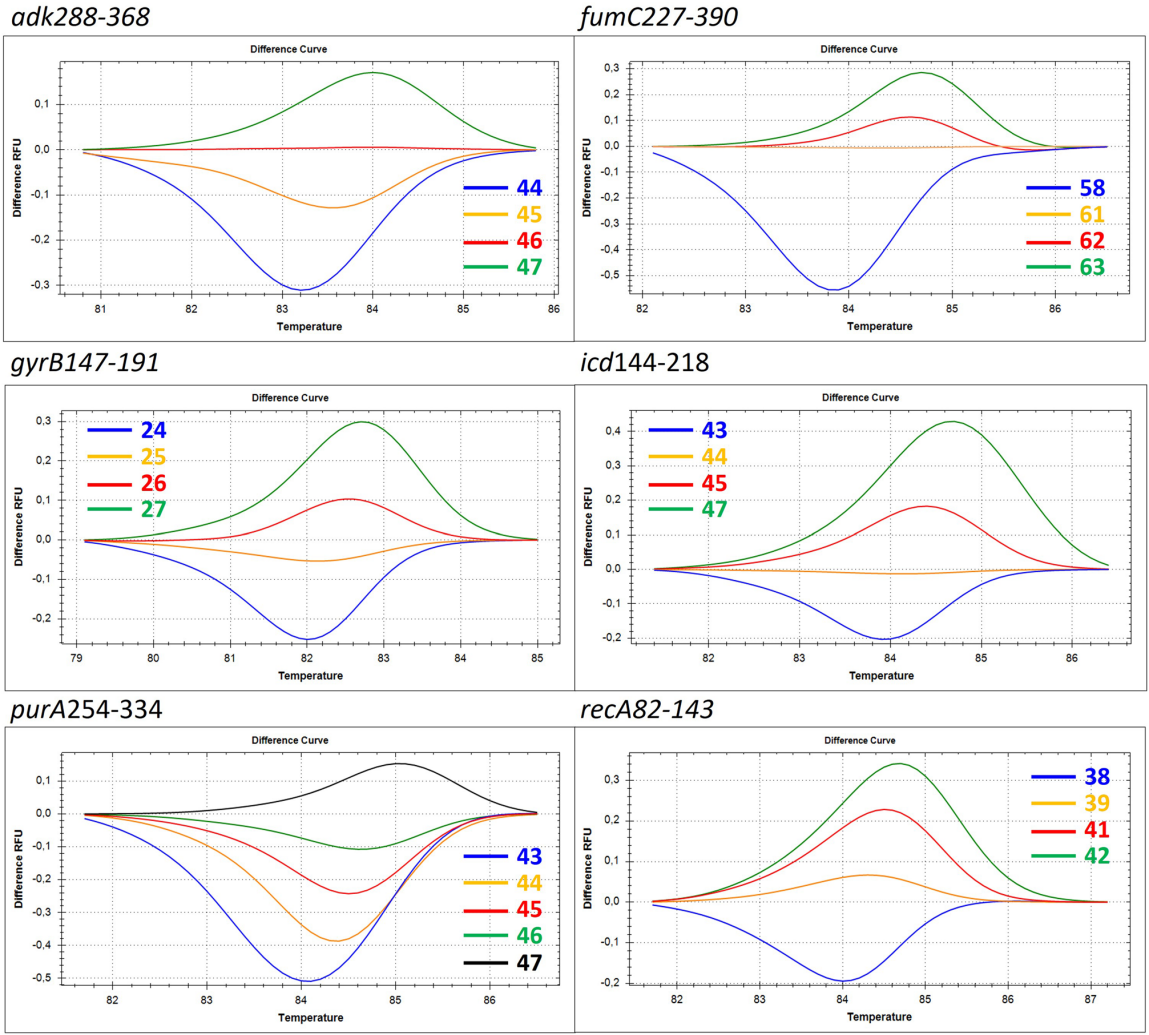
Difference graph for each of six mini-MLST loci. The difference curves are labeled with the GC content in corresponding mini-MLST loci. For locus *adk*288-368, four (44, 45, 46, 47) out of eight predicted alleles are displayed. For locus *fumC*227-390, four (58, 61, 62, 63) out of 14 predicted alleles are displayed. For locus *gyr*B147-191, four (24, 25, 26, 27) out of seven predicted alleles are displayed. For locus *icd*144-218, four (43, 44, 45, 47) out of eight predicted alleles are displayed. For locus *purA*254-334, five (43, 44, 45, 46, 47) out of seven predicted alleles are displayed. For locus *recA*82-143, four (38, 39, 40, 41) out of 10 predicted alleles are displayed.

The HRM curves for each mini-MLST loci were obtained for all 169 *E. coli* isolates and are presented in Fig. 1. The corresponding difference curves are shown in Fig. 2. The corresponding melting peaks can be found in Supplementary Fig. S1. Representatives from each obtained HRM curve were sequenced using MLST primers to determine the GC content in specific mini-MLST loci. The GC values were subsequently used to identify mini-MLST alleles. The melting temperature (Tm) values for each mini-MLST allele were calculated (Table 2). The HRM curves from four isolates had a non-standard shape and differed from the remaining HRM curves in at least one mini-MLST loci (an example of non-standard HRM curves can be found in Supplementary Fig. S2). Using Sanger sequencing, we found these isolates to be a mixture of at least two different strains and they were therefore excluded from further analyses.

**Table 2:** Confidence interval 95% (95% CI) of melting temperature (Tm), Tm absolute range and HRM normalization regions for obtained mini-MLST loci.

Mini-MLST loci	Allele (GC number)	Tm 95% CI (°C)	Tm absolute range (°C)	Distribution of captured alleles
adk288-368	44	82.61–82.73	82.6–82.8	20
	45	83.26–83.39	83.2–83.4	15
	46	83.56–83.68	83.5–83.8	125
	47	84.28–84.37	84.3–84.4	5
fumC227-390	58	83.29–83.39	83.3–83.4	8
	61	84.33–84.48	84.3–84.5	18
	62	84.57–84.66	84.5–84.7	73
	63	84.87–84.99	84.8–85.1	66
gyrB147-191	24	81.63–81.74	81.6–81.8	50
	25	82.20–82.31	82.2–82.4	67
	26	82.58–82.67	82.6–82.7	19
	27	82.94–83.07	82.9–83.1	29
icd144-218	43	83.60–83.76	83.5–83.8	41
	44	84.04–84.19	84.0–84.2	33
	45	84.49–84.61	84.4–84.7	87
	47	NA	85.3	1
purA254-334	43	NA	83.9	1
	44	84.19–84.38	84.2–84.5	50
	45	84.65–84.81	84.6–84.7	49
	46	84.91–85.02	84.7–84.9	64
	47	NA	85.3	1
recA82-143	38	NA	83.8	1
	39	84.23–84.41	84.1–84.5	63
	40	84.65–84.81	84.6–84.9	46
	41	85.14–85.28	85.1–85.3	55

MelTs were assigned to each isolate based on the acquired HRM curves and our MelT conversion key. From 165 isolates, 34 different MelTs were determined (Table 3). To correlate MelTs and STs, a subgroup of 110 isolates including at least one isolate of each obtained MelT was subjected to complete MLST. Those 110 isolates were divided into 45 STs and 34 MelTs, respectively. The mini-MST’s discriminatory power in our group was D = 0.9231 (CI 95% 0.8958–0.9504) against MLST’s D = 0.9364 (CI 95% 0.9077–0.9652) with the inter-rater agreement κ = 0.8979 (SD ± 0.0091).

**Table 3:** mini-MLST and MLST genotyping results of 110 selected *E. coli* isolates.

Melt	ST	Clonal complex	No. of isolates
71	93	ST168 cplx	2
76	48	ST10 cplx	1
156	590	ST590 cplx	2
160	88	ST23 cplx	1
166	410	ST23 cplx	1
175	1049	ST155 cplx	2
198	58	ST155 cplx	5
	399	ST399 cplx	1
	1423	None	1
	1429	None	2
234	297	None	1
359	684	ST648 cplx	1
380	1851	None	1
387	69	ST69 cplx	5
447	609	ST46 cplx	1
	6740	None	1
508	11,601	None	1
511	43	ST9 cplx	1
	617	ST101 cplx	1
512	44	ST10 cplx	1
592	117	None	1
623	961	ST12 cplx	1
626	12	ST12 cplx	2
644	141	None	3
653	131	ST131 cplx	23
669	38	ST38 cplx	3
696	100	ST100 cplx	3
	101	ST101 cplx	2
731	80	ST80 cplx	1
736	1618	ST73 cplx	1
737	73	ST73 cplx	11
	638	ST73 cplx	1
738	95	ST95 cplx	7
	127	None	2
	5484	ST95 cplx	1
740	144	None	1
744	429	ST95 cplx	1
756	1411	ST14 cplx	1
761	14	ST14 cplx	2
	404	ST14 cplx	6
900	2	None	1
923	405	ST405 cplx	1
	406	ST406 cplx	1
924	517	ST469 cplx	1
968	973	None	1

In silico analysis of 73,704 strains retrieved from EnteroBase^15^ was performed to validate the mini-MLST typing application on large sample sets. The discriminatory power on this substantial set of samples was D = 0.9465 (CI 95% 0.9726–0.9736) for mini-MLST and D = 0.9731 (CI 95% 0.9726–0.9736) for MLST.

### Mini-MLST as a tool for outbreak investigation

**Figure 3:**
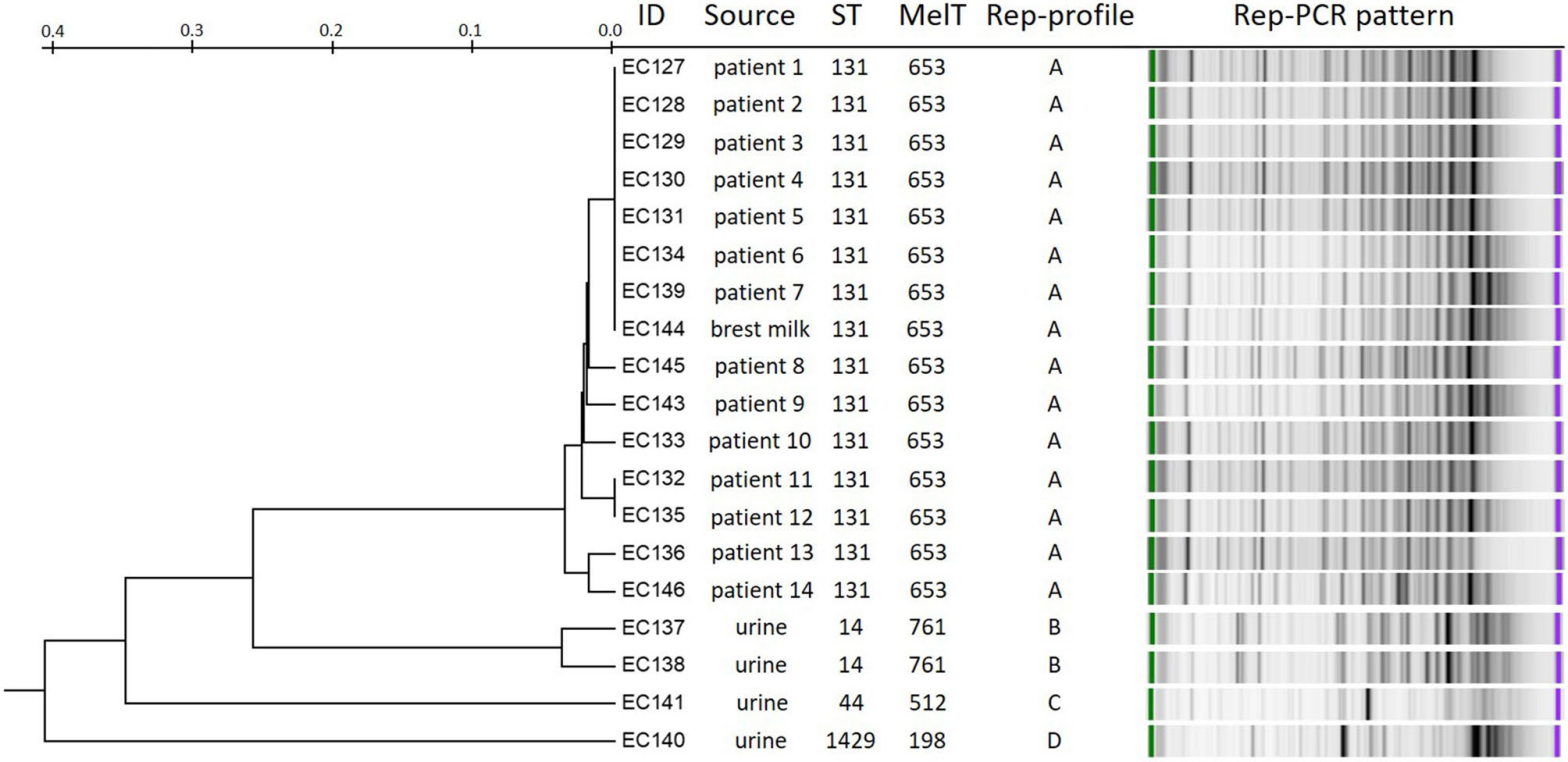
Concordance of rep-PCR, MLST and mini-MLST. Methods were compared with an ESBL *E. coli* isolate subset collected during a local outbreak at the Neonatal Department (UHB). Rep-profile are based on 95% similarity.

An increased incidence in extended-spectrum β-lactamases (ESBL) *E. coli* was observed in March and May 2016 at the Neonatal Department (UHB). In total, 15 ESBL *E. coli* isolates were isolated from blood cultures, rectal swabs, and neonates’ urine. Environment swabs (room, baths, incubators), health-worker swabs and breast milk samples (sterilized prior to administration to the neonate) were also tested. A single ESBL *E. coli* isolate was recovered from breast milk and none from the environmental and health-workers swabs. All isolates were subjected to molecular typing analysis using rep-PCR, MLST and mini-MLST. As a control, four ESBL *E. coli* urine isolates isolated from different departments at the UHB were added to our analyses. The isolates were differentiated into 4 rep-profiles, 4 STs and 4 MelTs (Fig. 3) indicating the concordance between methods was 100%, whereas no possible higher discriminatory power could be obtained from any of the used methods. Based on typing, the breast milk isolate was identical to the isolates recovered from the neonates. As a result, an immediate sterilizer inspection was performed, and crucial technical damage resulting in its impaired function was discovered. After replacing the sterilizer, no further ESBL *E. coli* cases in breast milk were observed.

### WGS typing, MLST, rep-PCR and mini-MLST concordance

**Figure 4:**
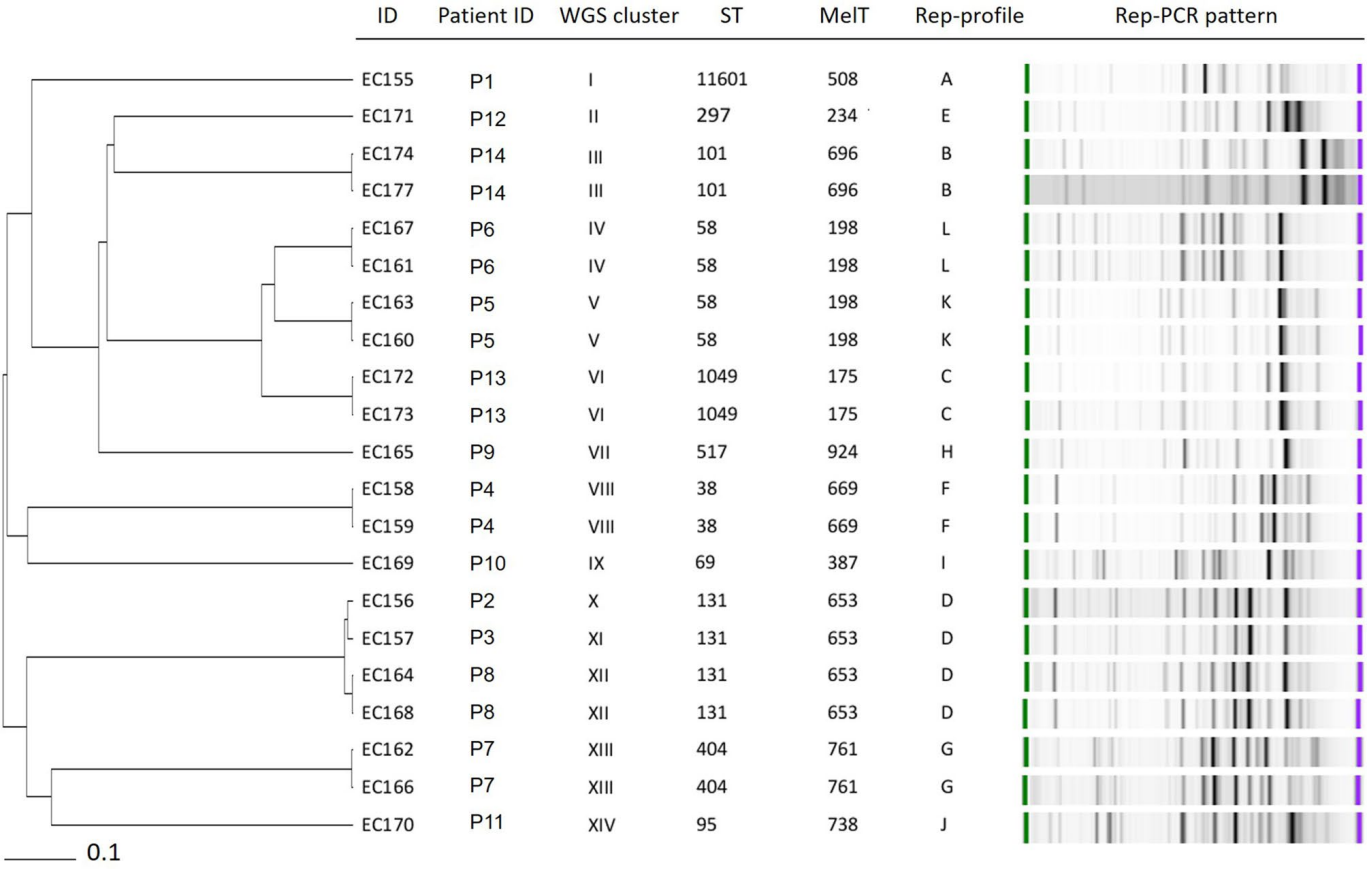
Concordance of wgMLST, MLST, mini-MLST and rep-PCR. Comparison is shown with a subset of 21 ESBL *E. coli* isolates collected between 5/2019 and 7/2019 at the Department of Internal Medicine—Hematology and Oncology, University Hospital Brno. The wgMLST clusters were defined by a maximum of 10 allele differences in a total of 4,637 analyzed genes.

The concordance of WGS, MLST, rep-PCR and mini-MLST and a comparison of their discriminatory power were tested on a subset of isolates obtained during a local epidemiological study conducted at the Department of Internal Medicine—Hematology and Oncology, UHB between 5/2019 and 7/2019. In total, 21 ESBL *E. coli* isolates were obtained from 14 patients. The 21 isolates were differentiated into 14 WGS clusters (cut-off for clustering isolates together was set to 10 allele differences in a total of 4,637 analyzed genes), 11 STs and 11 MelTs and 12 rep-profiles (Fig. 4). While the ST and mini-MLST results were in exact concordance, the rep-PCR and WGS data analysis divided samples belonging to ST58 into two different clusters. In addition, the WGS further sorted ST131 into three WGS clusters against one MelT and one rep-profile.

## Discussion

MLST and PFGE are still considered the gold standards for molecular typing. However, the development and application of WGS-based techniques is rising, along with a reduction in their costs. WGS has an unsurpassed discriminatory power over other typing methods but is also the most demanding approach in terms of difficulty and data analysis. In contrast, HRM-based methods are extremely cheap, fast and at the same time also very robust and easily portable between laboratories. HRM has already been effectively used to detect and identify antimicrobial resistance, to screen and identify target mutations, evaluate bacterial population structure and genetic diversity^4^. Mini-MLST typing schemes have already been successfully validated for *K. pneumoniae*^5^, *S. aureus*^6^, *E. faecium*^7^ and *S. pyogenes*^8^. Regarding *E.coli*, apart from a number of methods designated for species identification, use of HRM methods has been increasing over time and used to quantify virulence and resistance genes or are currently available^9–11^. In terms of molecular typing, there are two methods available. The first one is designed to identify ST131 as an internationally spread high-risk clone^16^. This method is only capable of distinguishing ST131 from non-ST131 strains, which is not sufficient in most cases. The second method is based on ligation-mediated real-time PCR followed by HRM^12^. While retaining the advantages of HRM (speed, cost, low labor intensity), the main disadvantage of this method remains the reproducibility and transferability due to the lack of support within a globally recognized scheme (e.g., MLST). Although this method may be used for direct isolate comparison during a local outbreak investigation, it is not suitable for comparing of large sample sets, long-term studies, and inter-laboratory studies.

The mini-MLST approach is based solely on the GC content of the target locus. Therefore, different sequences with the same GC content are practically indistinguishable using HRM analysis alone. Being specific, this means that hundreds of MLST alleles are converted into just a few mini-MLST alleles (typically 3–10), which is reflected in the lower mini-MLST against MLST discriminatory power. However, the major advantages of mini-MLST are cost-effectiveness, rapid performance, robustness, and great reproducibility accompanied with lower analytical complexity, resulting in straightforward interpretation (Table 4)^2,17,18^. The total price per isolate is approximately $5 which is significantly more cost effective than the majority of other typing methods (e.g., $50 per complete MLST, $150 per WGS). The whole analysis including results evaluation takes about 2.5 h (excluding DNA isolation). Moreover, during this study we proved that it is possible to optimize the reaction mixture and temperature to be identical for all existing mini-MLST schemes. This is a considerable advantage as it allows our laboratory to simultaneously type up to 48 isolates of different bacterial species (using 384-well plates). Considering mini-MLST’s portability, using our approach may accelerate typing in other laboratories, which is particularly suitable for larger laboratories with a significant number of isolates to be analyzed. Mini-MLST’s robustness is based on unique HRM curves produced by fragments with different CG content while using an optimal fragment length (70–200 bp). For longer fragments, the impact of GC content differences on Tm may be reduced and the HRM curves may not be clearly distinguishable^6^. Since mini-MLST is derived from globally recognized MLST, it has great portability and together with its high reproducibility, can be globally implemented into laboratories without substantial effort.

**Table 4:** Comparison of main Mini-MLST, MLST, Rep-PCR and WGS typing characteristics^2,17,18^.

	Mini-MLST	MLST	Rep-PCR	WGS
Discriminatory power	++	+++	++	+++++
Typability	+++	++++	+++	+++++
Reproducibility	++++	++++	++	++++
Throughput	+++++	++	++++	++
Labor Intensity	+	+++	++	++++
Analytical Complexity	+	+	+	+++++
Cost	$5	$50	$10	$150
Time required	+	+++	++	+++++

Individual characteristics are ranged from + (lowest) to +++++ (highest).

To compare the routinely used MLST and to validate mini-MLST for typing large sample sets, we performed an in silico analysis on 73,704 strains retrieved from EnteroBase database^15^. With a D value of 0.9465 (CI 95% 0.9726–0.9736) for mini-MLST, a D value of 0.9731 (CI 95% 0.9726–0.9736) for MLST, mini-MLST proved to have a comparable discrimination power to MLST and to be a suitable method with sufficient discriminatory power for large population studies and long-term screening. Mini-MLST’s validation in routine clinical practice was performed against rep-PCR, MLST and WGS on clinical isolates collected during the local outbreak and surveillance study at the UHB. During the local epidemiological study at the Department of Internal Medicine, all typing methods were compared not only with each other but also evaluated against WGS as it provides the highest currently achievable discriminatory potential. This comparison resulted 11 MelTs and 11 ST, 12 rep-profiles and 14 WGS clusters. Overall, proposed mini-MLST typing scheme showed great correlation with all three aforementioned methods which was further accompanied by the essential advantages mentioned above. In this case, however, a limited number of isolates need to be taken into account.

Due to the expanding use of next-generation sequencing (incl. WGS), new alleles and/or their combinations (new STs) are being discovered almost daily. To be able to type strains from the newest STs, the conversion key is updated monthly. The current version of the conversion key is available at http://www.cmbgt.cz/mini-mlst/t6353.

From the total number of 10,946 STs known as of 3/2021, only 39 STs are marked as MelT0 i.e., with no specified MelT. In most cases, this is caused by the absence of a specific allele in the source MLST database. In this case, we cannot predict the mini-MLST allele (number of GC bases) and thus determine the specific MelT. Even though the conversion key contains an indication of the missing allele (marked as -3 error in the conversion key) and the result is MelT0, the HRM curves still can be obtained. Thus, MelT0 does not necessarily mean it is impossible to type. If the particular allele was added to the source data, the change would be reflected in the conversion key after the update.

Mini-MLST can be used not only to resolve an acute epidemiological situation as a rapid typing method, but also for prospective monitoring of high-risk bacterial strains’ occurrence. This is possible due to the correlation between the biological properties and the ST/genotype, previously described for *E. coli* high-risk clones belonging to ST38 (MelT669), ST69 (MelT387), ST73 (MelT737), ST95 (MelT738), ST131 (MelT653), ST155 (MelT175), ST393 (MelT343), ST405 (MelT923), ST410 (MelT166) and ST648 (MelT359)^19–21^. At the same time, the better we know the local bacterial population structure and its properties, the better we can respond to the occurrence of new or emergent virulent and/or multidrug-resistant strains. A two-step approach using mini-MLST can be advantageously used for both prospective surveillance and retrospective molecular typing. Our results clearly showed that the isolates distinguished by mini-MLST are similarly distinguished by WGS-based typing. Therefore, only isolates from the same MelT should be processed to the next typing step during an outbreak investigation involving WGS. This will allow hospitals to concentrate focus and resources specifically on the outbreak strains and their subsequent in-depth typing. On the other hand, in studies characterizing a large bacterial population, it is advantageous both in terms of time and cost to select strains from different MelTs as it prevents sequencing identical strains and acquiring redundant information.

To conclude, mini-MLST has great portability, low labor intensity, great cost-efficiency and very high throughput, which represents immense benefits, even when those are accompanied with its slightly lower discriminatory power than other typing methods. Our results proved mini-MLST is a great method for rapid and cost-effective screening and subgrouping for large isolates sets and/or quick strain typing during outbreaks. In addition, it is also suitable for prospective surveillance monitoring of emergent and high-risk *E. coli* clones.

## Material and methods

### Target identification and primer design

The *E. coli* MLST scheme for 10,946 sequence types (STs) (data to 31/1/2021), including allele sequences for *adk, fumC, gyrB, icd, mdh, purA* and *recA* genes were downloaded from the EnteroBase website (http://enterobase.warwick.ac.uk/species/ecoli/download_7_gene)^13^. All genes were concatenated and aligned using MEGA7 software^22^.

The SNPs that change the percentage content of G + C were identified and selected for further analysis as the A ↔ T and C ↔ G nucleotide changes cannot be generally/commonly detected using HRM analysis. The *mdh* genes were excluded from further analyses as no significant SNPs were found within this gene.

The primer sets were designed using Primer3 v 0.4.0 (http://bioinfo.ut.ee/primer3-0.4.0/) and targeted conserved regions flanking previously identified SNPs (Table 1). From the available literature, we determined the optimal amplicon length for HRM analysis ranged between 50 to 200 bp^5–8^.

### Mini-MLST scheme design

Predicting the HRM curve and assigning the melting type (MelT) were carried out using our in-house MELT2MELT software. Each analyzed locus sequence was processed as follows. Specific forward and reverse primers for all loci were found in amplicons using a simple regex search. The number of G and C bases between a pair of primers in every gene in the selected sequence was counted and stored in a table containing all analyzed sequence types. The order of the rows in the table was rearranged according to the increasing number of G and C bases in every analyzed gene. Finally, a MelT number was assigned to each ST by assigning a number one to the first row of the table and increasing this number by one for every ST whose G and C base numbers were different from the previous ST.

### Discriminatory power calculation

To determine the discriminatory power of proposed mini-MLST scheme and MLST, Simpson’s Index of Diversity (D)^23^ was calculated using a freely accessible MATLAB (MathWorks, USA) code available at http://www.comparingpartitions.info/.

### Clinical Isolates

For mini-MLST validation, a total of 169 clinical *E. coli* isolates collected between 3/2016 and 7/2019 at the Department of Clinical microbiology (UHB) were used. All isolates were identified using Matrix-Assisted Laser Desorption/Ionization Time-of-Flight Mass Spectrometry (MALDI-TOF). Pure culture colonies were harvested and resuspended in 2 ml of sterile water (density corresponding to 5.0 McFarland standard) and stored at − 20 °C for further molecular analyses. All isolates were stored in cryotubes ITEST Kryobanka B (ITEST plus, Czech Republic). Out of all 169 *E. coli* isolates, 19 were collected during the local outbreak at the Neonatal Department, UHB in 3/2016–5/2016 and 21 isolates were collected during a surveillance study conducted at the Department of Hematology and Oncology (UHB) in 5/2019–7/2019.

### Sample set for in silico analysis

To validate the mini-MLST on a large sample set, the MLST data from 100,000 most recently uploaded *E. coli* strains were retrieved (http://enterobase.warwick.ac.uk/species/ecoli/search_strains). Out of 100,000 strains, only strains with specified ST (n = 73,704) were used to compare in silico mini-MLST and MLST. The complete list of all 73,704 strains is listed in the Supplementary Table S1.

### DNA isolation

Genomic DNA (gDNA) was isolated using Chelex 100 Resin (Bio-Rad, USA). The overnight bacterial cultures were homogenized in 100 μL of 5% w/v Chelex 100 Resin with vortex. The obtained suspensions were incubated for 10 min at 100 °C and then centrifuged for 2 min at 15,500 rcf. Each strain’s supernatant containing gDNA was transferred into a clean microtube. For WGS, gDNA was purified using GenElute Bacterial Genomic DNA Kit (SIGMA-ALDRICH, USA).

### Mini-MLST

Mini-MLST was performed on a Bio-Rad CFX96 platform (Bio-Rad, USA). The reaction mixture contained 10 μL 2 × SensiFAST HRM mix (Meridian Bioscience, UK), 0.4 μM of each primer, 1 μL of gDNA (30 ng/µL) and deionized water to a final volume of 20 μL. Thermo cycling parameters were: 95 °C for 3 min, 40 cycles of 95 °C for 5 s, 65 °C for 10 s and 72 °C for 20 s, followed by one cycle of 95 °C for 2 min and 50 °C for 20 s, terminated by HRM ramping from 72 to 88 °C, increasing by 0.1 °C at each step. The results were interpreted using the current version of our conversion key, which is available for free download at http://www.cmbgt.cz/mini-mlst/t6353. The conversion key is regularly updated on a monthly basis.

### Multilocus sequence typing

In total, 110 strains were selected for a full MLST sequencing scheme according to the protocol described by Wirth et al*.*^15^. The current version of the *E. coli* MSLT database is available on http://enterobase.warwick.ac.uk/species/ecoli/download_7_gene.

### Rep-PCR

To generate DNA fingerprint patterns, Rep-PCR primers REP1R and REP2I were used^24^, following the protocols described previously^25^. The PCR amplicons were analyzed using Agilent 2100 Bioanalyzer (Agilent Technologies, USA) and the previously described algorithm was used^26^ to determine the rep-profiles.

### Whole genome sequencing

Sequencing libraries were prepared using KAPA HyperPlus Kits (Roche, Switzerland). Sequencing was performed on the Illumina MiSeq platform using the MiSeq Reagent Kit v2 (500-cycles) (Illumina, USA). After quality control, the reads were trimmed via Trimmomatic^27^. Burrows-Wheeler Alignment MEM (v0.7.17-r1188)^28^ was used for reference-based assembly. *E. coli* NC_002695.2 was used as a reference genome. Samtools^29^ was used to remove low-quality and duplicated reads, followed by consensus sequence generation. The assembled genomes were analyzed using Ridom SeqSphere+ (Ridom GmbH, DE).

## Supplementary Information

Supplementary Information.

## Data Availability

All *E. coli* raw illumina sequence data from this study have been deposited in the Sequence Read Archive under the BioProject No. PRJNA695195. All MLST FASTA files are available from https://github.com/tysek3/ESCO-mini-MLST-Sup-files.git. The MelT conversion key is available to download from http://www.cmbgt.cz/mini-mlst/t6353.
