# Supplementary material for: Rapid high-resolution melting genotyping scheme for Escherichia coli based on MLST derived single nucleotide polymorphisms
Source: Sci Rep. 2021 Aug 16;11:16572. doi: 10.1038/s41598-021-96148-3 (PMC8368041; doi:10.1038/s41598-021-96148-3)
Supplement: Supplementary file 1 — Supplementary Information. [file 41598_2021_96148_MOESM1_ESM.pdf]

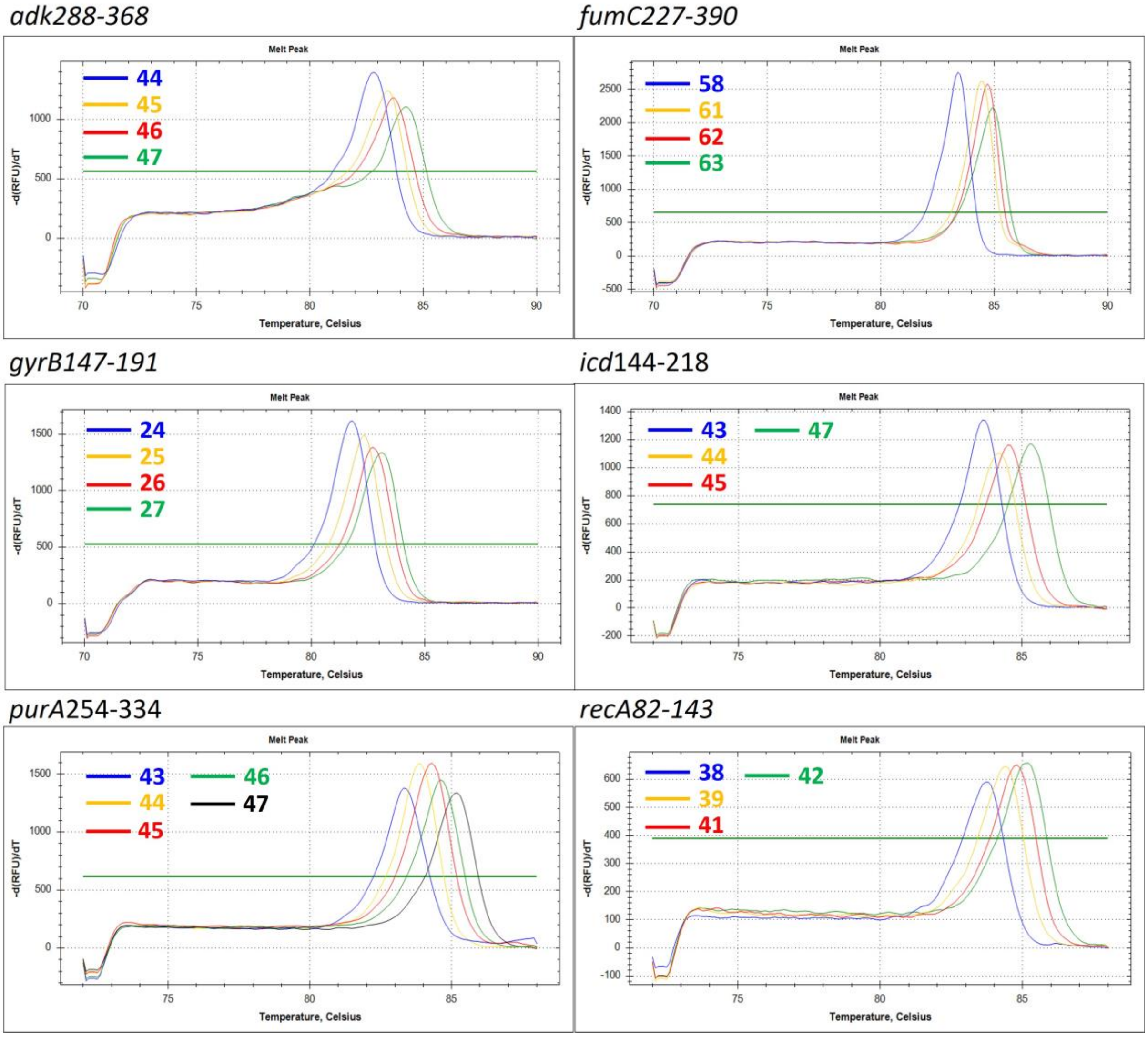

Fig. S1 Melting peaks curves for each of six mini-MLST loci. The peaks are labeled with the GC content in corresponding mini-MLST loci. For locus *adk*288-368, four (44,45,46,47) out of eight predicted alleles are displayed. For locus *fum*C227-390, four (58, 61, 62, 63) out of 14 predicted alleles are displayed. For locus *gyr*B147-191, four (24, 25, 26, 27) out of seven predicted alleles are displayed. For locus *icd*144-218, four (43, 44, 45, 47) out of eight predicted alleles are displayed. For locus *purA*254-334, five (43, 44, 45, 46, 47) out of seven predicted alleles are displayed. For locus *recA*82-143, four (38, 39, 40, 41) out of 10 predicted alleles are displayed.

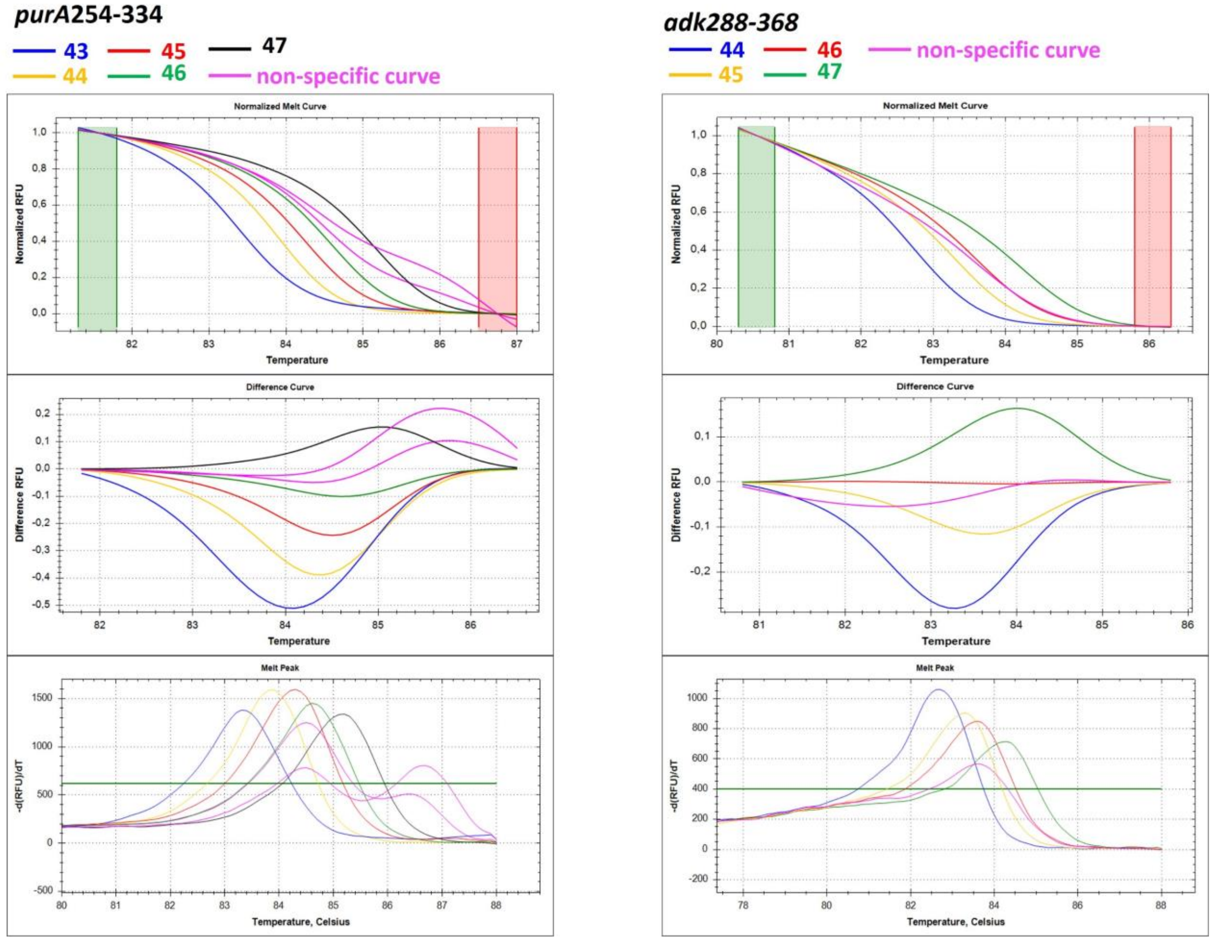

Fig. S2 Melting peaks curves for each of six mini-MLST loci. The peaks are labeled with the GC content in corresponding mini-MLST loci. The curves with non-standard shape are highlighted in violet.

Table S1 The complete list of all 73,704 *E. coli* strains used for the *in silico* comparison

Barcode	ST	MelT
ESC_UA9356AA	11	936
ESC_UA9355AA	301	307
ESC_UA9354AA	11	936
ESC_UA9353AA	167	512
ESC_UA9155AA	442	257
ESC_UA8922AA	152	257
ESC_UA8921AA	152	257
ESC_UA8920AA	152	257
ESC_UA8919AA	245	261
ESC_UA8918AA	152	257
ESC_UA8917AA	7384	257
ESC_UA8916AA	152	257
ESC_UA8915AA	10769	257
ESC_UA8914AA	245	261
ESC_UA8913AA	7384	257
ESC_UA8912AA	245	261
ESC_UA8911AA	152	257
ESC_UA8910AA	152	257
ESC_UA8909AA	152	257
ESC_UA8908AA	152	257
ESC_UA8907AA	152	257
ESC_UA8906AA	152	257
ESC_UA8905AA	152	257
ESC_UA8904AA	245	261
ESC_UA8903AA	245	261
ESC_UA8902AA	152	257
ESC_UA8901AA	245	261
ESC_UA8900AA	152	257
ESC_UA8899AA	152	257
ESC_UA8898AA	152	257
ESC_UA8880AA	245	261
ESC_UA8879AA	152	257
ESC_UA8878AA	245	261
ESC_UA8733AA	301	307
ESC_UA8643AA	6781	508
ESC_UA8642AA	167	512
ESC_UA8641AA	648	359
ESC_UA8640AA	744	512
ESC_UA8639AA	410	156
ESC_UA8638AA	11921	175
ESC_UA8637AA	167	512
ESC_UA8636AA	744	512
ESC_UA8635AA	162	924
ESC_UA8634AA	11920	111
ESC_UA8633AA	155	175
ESC_UA8632AA	156	175
ESC_UA8631AA	2197	511

ESC_UA8630AA	10	512
ESC_UA8629AA	617	511
ESC_UA8628AA	167	512
ESC_UA8627AA	167	512
ESC_UA8626AA	162	924
ESC_UA8625AA	1049	175
ESC_UA8624AA	602	257
ESC_UA8623AA	1249	257
ESC_UA8622AA	648	359
ESC_UA8621AA	648	359
ESC_UA8620AA	11919	789
ESC_UA8619AA	117	592
ESC_UA8618AA	1485	408
ESC_UA8617AA	371	50
ESC_UA8616AA	2325	447
ESC_UA8615AA	2197	511
ESC_UA8614AA	156	175
ESC_UA8613AA	11918	672
ESC_UA8612AA	38	669
ESC_UA8611AA	156	175
ESC_UA8265AA	301	307
ESC_UA8264AA	301	307
ESC_UA8263AA	21	156
ESC_UA8262AA	11	936
ESC_UA8261AA	11	936
ESC_UA6944AA	245	261
ESC_UA6943AA	152	257
ESC_UA6942AA	152	257
ESC_UA6941AA	152	257
ESC_UA6940AA	145	256
ESC_UA6939AA	11389	261
ESC_UA6938AA	152	257
ESC_UA6937AA	245	261
ESC_UA6936AA	152	257
ESC_UA6935AA	152	257
ESC_UA6934AA	152	257
ESC_UA6933AA	152	257
ESC_UA6932AA	245	261
ESC_UA6930AA	11917	512
ESC_UA6929AA	11917	512
ESC_UA6928AA	11917	512
ESC_UA6927AA	11917	512
ESC_UA6926AA	11917	512
ESC_UA6925AA	11916	515
ESC_UA6924AA	1431	257
ESC_UA6887AA	648	359
ESC_UA6886AA	648	359
ESC_UA6885AA	1193	761
ESC_UA6884AA	58	198
ESC_UA6883AA	617	511

ESC_UA6882AA	164	175
ESC_UA6881AA	58	198
ESC_UA6880AA	1246	198
ESC_UA6879AA	6448	175
ESC_UA6878AA	6448	175
ESC_UA6877AA	6448	175
ESC_UA6876AA	6448	175
ESC_UA6875AA	6448	175
ESC_UA6858AA	224	198
ESC_UA6857AA	224	198
ESC_UA6856AA	6448	175
ESC_UA6855AA	6448	175
ESC_UA6854AA	33	198
ESC_UA6853AA	641	935
ESC_UA6609AA	245	261
ESC_UA6608AA	1968	76
ESC_UA6607AA	3018	903
ESC_UA6606AA	3593	153
ESC_UA6605AA	1204	961
ESC_UA6604AA	10	512
ESC_UA6603AA	1589	156
ESC_UA6601AA	23	156
ESC_UA6600AA	6448	175
ESC_UA6599AA	6448	175
ESC_UA6598AA	224	198
ESC_UA6597AA	164	175
ESC_UA6596AA	3018	903
ESC_UA6595AA	11	936
ESC_UA6594AA	11915	504
ESC_UA6593AA	21	156
ESC_UA6592AA	21	156
ESC_UA6590AA	181	311
ESC_UA6559AA	1193	761
ESC_UA6558AA	245	261
ESC_UA6548AA	4977	71
ESC_UA6546AA	11912	257
ESC_UA6544AA	11911	854
ESC_UA6543AA	216	512
ESC_UA6540AA	11910	198
ESC_UA6531AA	11914	962
ESC_UA6527AA	11905	398
ESC_UA6526AA	11907	160
ESC_UA6525AA	11906	198
ESC_UA6524AA	11909	601
ESC_UA6521AA	3476	512
ESC_UA6520AA	11913	470
ESC_UA6518AA	11908	1038
ESC_UA6517AA	11904	1037
ESC_UA6515AA	11903	198
ESC_UA6514AA	11898	198

ESC_UA6513AA	11902	76
ESC_UA6512AA	11901	511
ESC_UA6511AA	11900	198
ESC_UA6510AA	11899	307
ESC_UA6509AA	95	738
ESC_UA6508AA	95	738
ESC_UA6507AA	1775	51
ESC_UA6506AA	38	669
ESC_UA6505AA	245	261
ESC_UA6504AA	152	257
ESC_UA6503AA	245	261
ESC_UA6502AA	245	261
ESC_UA6501AA	245	261
ESC_UA6500AA	6	445
ESC_UA6499AA	245	261
ESC_UA6498AA	152	257
ESC_UA6497AA	245	261
ESC_UA6496AA	152	257
ESC_UA6495AA	152	257
ESC_UA6494AA	152	257
ESC_UA6493AA	245	261
ESC_UA6492AA	245	261
ESC_UA6491AA	11897	198
ESC_UA6490AA	131	653
ESC_UA6489AA	131	653
ESC_UA6488AA	131	653
ESC_UA6487AA	131	653
ESC_UA6486AA	131	653
ESC_UA6485AA	131	653
ESC_UA6484AA	131	653
ESC_UA6483AA	131	653
ESC_UA6482AA	131	653
ESC_UA6481AA	131	653
ESC_UA6480AA	131	653
ESC_UA6479AA	131	653
ESC_UA6478AA	131	653
ESC_UA6477AA	131	653
ESC_UA6476AA	131	653
ESC_UA6475AA	131	653
ESC_UA6474AA	131	653
ESC_UA6473AA	131	653
ESC_UA6472AA	131	653
ESC_UA6471AA	131	653
ESC_UA6470AA	131	653
ESC_UA6469AA	131	653
ESC_UA6468AA	131	653
ESC_UA6467AA	131	653
ESC_UA6466AA	131	653
ESC_UA6465AA	131	653
ESC_UA6464AA	131	653

ESC_UA6463AA	131	653
ESC_UA6462AA	131	653
ESC_UA6461AA	131	653
ESC_UA6460AA	131	653
ESC_UA6459AA	131	653
ESC_UA6458AA	131	653
ESC_UA5008AA	11	936
ESC_UA5007AA	11	936
ESC_UA5006AA	11	936
ESC_UA5005AA	11	936
ESC_UA5004AA	11	936
ESC_UA5003AA	11	936
ESC_UA4985AA	11	936
ESC_UA4984AA	7006	936
ESC_UA4983AA	7006	936
ESC_UA4982AA	7006	936
ESC_UA4981AA	3268	669
ESC_UA4936AA	11895	400
ESC_UA4935AA	11894	515
ESC_UA4934AA	11896	512
ESC_UA4916AA	11893	261
ESC_UA4914AA	342	286
ESC_UA4913AA	583	768
ESC_UA4911AA	11891	654
ESC_UA4910AA	95	738
ESC_UA4907AA	1148	257
ESC_UA4906AA	2015	684
ESC_UA4905AA	646	738
ESC_UA4904AA	2015	684
ESC_UA4903AA	2015	684
ESC_UA4902AA	2015	684
ESC_UA4901AA	2015	684
ESC_UA4900AA	2015	684
ESC_UA4899AA	152	257
ESC_UA4898AA	245	261
ESC_UA4813AA	245	261
ESC_UA4812AA	245	261
ESC_UA4811AA	152	257
ESC_UA4810AA	245	261
ESC_UA4809AA	245	261
ESC_UA4808AA	152	257
ESC_UA4807AA	245	261
ESC_UA4806AA	6949	765
ESC_UA4805AA	6949	765
ESC_UA4661AA	131	653
ESC_UA4660AA	131	653
ESC_UA4659AA	131	653
ESC_UA4658AA	131	653
ESC_UA4657AA	131	653
ESC_UA4656AA	131	653

ESC_UA4655AA	131	653
ESC_UA4654AA	131	653
ESC_UA4653AA	131	653
ESC_UA4652AA	131	653
ESC_UA4651AA	131	653
ESC_UA4650AA	131	653
ESC_UA4649AA	131	653
ESC_UA4648AA	131	653
ESC_UA4647AA	245	261
ESC_UA4646AA	342	286
ESC_UA4645AA	121	537
ESC_UA4644AA	31	387
ESC_UA4643AA	58	198
ESC_UA4642AA	131	653
ESC_UA4641AA	131	653
ESC_UA4640AA	131	653
ESC_UA4639AA	131	653
ESC_UA4638AA	131	653
ESC_UA4637AA	131	653
ESC_UA4636AA	131	653
ESC_UA4635AA	131	653
ESC_UA4634AA	131	653
ESC_UA4633AA	131	653
ESC_UA4632AA	11887	512
ESC_UA4631AA	21	156
ESC_UA4629AA	10	512
ESC_UA4628AA	10	512
ESC_UA4389AA	152	257
ESC_UA4388AA	11885	669
ESC_UA4387AA	245	261
ESC_UA4386AA	245	261
ESC_UA4385AA	152	257
ESC_UA4384AA	245	261
ESC_UA4383AA	152	257
ESC_UA4382AA	245	261
ESC_UA4381AA	245	261
ESC_UA4380AA	152	257
ESC_UA4379AA	152	257
ESC_UA4378AA	152	257
ESC_UA4377AA	152	257
ESC_UA4376AA	245	261
ESC_UA4374AA	152	257
ESC_UA4373AA	152	257
ESC_UA4372AA	152	257
ESC_UA4371AA	152	257
ESC_UA4370AA	245	261
ESC_UA4369AA	152	257
ESC_UA4368AA	245	261
ESC_UA4367AA	245	261
ESC_UA4366AA	152	257

ESC_UA4365AA	152	257
ESC_UA4364AA	152	257
ESC_UA4363AA	21	156
ESC_UA4362AA	301	307
ESC_UA4361AA	11	936
ESC_UA4360AA	11	936
ESC_UA4359AA	7796	738
ESC_UA4358AA	127	738
ESC_UA4357AA	119	286
ESC_UA4356AA	152	257
ESC_UA4355AA	152	257
ESC_UA4354AA	152	257
ESC_UA4353AA	152	257
ESC_UA4352AA	152	257
ESC_UA3991AA	245	261
ESC_UA3989AA	152	257
ESC_UA3988AA	152	257
ESC_UA3987AA	245	261
ESC_UA3986AA	148	191
ESC_UA3985AA	245	261
ESC_UA3383AA	7006	936
ESC_UA3382AA	11874	626
ESC_UA3381AA	11873	512
ESC_UA3380AA	10	512
ESC_UA3379AA	11873	512
ESC_UA3378AA	11873	512
ESC_UA3377AA	58	198
ESC_UA3376AA	11869	234
ESC_UA3375AA	73	737
ESC_UA3374AA	11870	761
ESC_UA3373AA	11871	629
ESC_UA3372AA	11872	737
ESC_UA3346AA	11868	198
ESC_UA3345AA	11867	741
ESC_UA3344AA	11867	741
ESC_UA3343AA	538	629
ESC_UA3342AA	11866	789
ESC_UA3341AA	62	574
ESC_UA3340AA	11867	741
ESC_UA3339AA	1125	198
ESC_UA3338AA	141	644
ESC_UA3337AA	91	629
ESC_UA3336AA	998	644
ESC_UA3335AA	1426	160
ESC_UA3334AA	95	738
ESC_UA3333AA	69	387
ESC_UA3332AA	11865	390
ESC_UA3331AA	58	198
ESC_UA3330AA	73	737
ESC_UA3308AA	11864	936

ESC_UA3307AA	11863	1
ESC_UA3306AA	11862	191
ESC_UA3305AA	11	936
ESC_UA3304AA	11864	936
ESC_UA3303AA	11864	936
ESC_UA3302AA	245	261
ESC_UA3301AA	152	257
ESC_UA3300AA	152	257
ESC_UA3299AA	152	257
ESC_UA3298AA	152	257
ESC_UA3157AA	11	936
ESC_UA3156AA	11	936
ESC_UA3155AA	10	512
ESC_UA3154AA	11	936
ESC_UA3153AA	11	936
ESC_UA3152AA	11	936
ESC_UA3151AA	11	936
ESC_UA3150AA	11	936
ESC_UA3128AA	101	696
ESC_UA3127AA	718	257
ESC_UA3126AA	677	257
ESC_UA3125AA	10	512
ESC_UA3124AA	205	257
ESC_UA3123AA	448	257
ESC_UA3122AA	17	191
ESC_UA3121AA	657	592
ESC_UA3120AA	10	512
ESC_UA3119AA	677	257
ESC_UA3118AA	154	257
ESC_UA3117AA	2385	198
ESC_UA3116AA	718	257
ESC_UA3115AA	58	198
ESC_UA3114AA	101	696
ESC_UA3113AA	301	307
ESC_UA3062AA	245	261
ESC_UA3061AA	152	257
ESC_UA3055AA	11	936
ESC_UA3054AA	659	286
ESC_UA3053AA	11	936
ESC_UA3052AA	11	936
ESC_UA3030AA	10	512
ESC_UA3029AA	1423	198
ESC_UA3028AA	11841	737
ESC_UA3026AA	152	257
ESC_UA3025AA	152	257
ESC_UA3024AA	152	257
ESC_UA3023AA	152	257
ESC_UA3022AA	152	257
ESC_UA3021AA	152	257
ESC_UA3020AA	21	156

ESC_UA3019AA	11	936
ESC_UA3018AA	301	307
ESC_UA3017AA	6372	198
ESC_UA3016AA	11	936
ESC_UA3015AA	1431	257
ESC_UA3014AA	17	191
ESC_UA3013AA	7624	175
ESC_UA3011AA	2165	257
ESC_UA3009AA	2165	257
ESC_UA3008AA	69	387
ESC_UA3007AA	152	257
ESC_UA3006AA	152	257
ESC_UA3005AA	243	197
ESC_UA3004AA	245	261
ESC_UA3003AA	245	261
ESC_UA3001AA	245	261
ESC_UA3000AA	245	261
ESC_UA2999AA	245	261
ESC_UA2998AA	245	261
ESC_UA2997AA	152	257
ESC_UA2856AA	58	198
ESC_UA2855AA	1727	257
ESC_UA2854AA	48	76
ESC_UA2853AA	48	76
ESC_UA2852AA	1421	447
ESC_UA2851AA	361	512
ESC_UA2850AA	963	669
ESC_UA2849AA	58	198
ESC_UA2848AA	710	291
ESC_UA2847AA	10	512
ESC_UA2846AA	641	935
ESC_UA2845AA	2144	257
ESC_UA2844AA	12	626
ESC_UA2843AA	681	393
ESC_UA2842AA	394	400
ESC_UA2767AA	16	156
ESC_UA2766AA	152	257
ESC_UA2765AA	10	512
ESC_UA2764AA	21	156
ESC_UA2763AA	8356	198
ESC_UA2762AA	5292	738
ESC_UA2761AA	11	936
ESC_UA2760AA	301	307
ESC_UA2759AA	301	307
ESC_UA2758AA	21	156
ESC_UA2757AA	21	156
ESC_UA2653AA	245	261
ESC_UA2652AA	245	261
ESC_UA2651AA	245	261
ESC_UA2649AA	245	261

ESC_UA2647AA	245	261
ESC_UA2646AA	245	261
ESC_UA2645AA	245	261
ESC_UA2644AA	245	261
ESC_UA2638AA	245	261
ESC_UA2637AA	245	261
ESC_UA2636AA	152	257
ESC_UA2635AA	245	261
ESC_UA2634AA	245	261
ESC_UA2633AA	245	261
ESC_UA2632AA	23	156
ESC_UA2631AA	23	156
ESC_UA2630AA	23	156
ESC_UA2629AA	279	142
ESC_UA2628AA	270	204
ESC_UA2627AA	6	445
ESC_UA2626AA	270	204
ESC_UA2624AA	6	445
ESC_UA2623AA	6	445
ESC_UA2622AA	311	204
ESC_UA2620AA	270	204
ESC_UA2619AA	11926	204
ESC_UA2614AA	23	156
ESC_UA2613AA	2389	160
ESC_UA2612AA	6	445
ESC_UA2611AA	270	204
ESC_UA2610AA	270	204
ESC_UA2609AA	278	924
ESC_UA2608AA	280	142
ESC_UA2607AA	280	142
ESC_UA2606AA	281	355
ESC_UA2605AA	270	204
ESC_UA2604AA	270	204
ESC_UA2603AA	283	204
ESC_UA2602AA	284	204
ESC_UA2601AA	270	204
ESC_UA2600AA	280	142
ESC_UA2599AA	270	204
ESC_UA2598AA	270	204
ESC_UA2596AA	279	142
ESC_UA2595AA	6	445
ESC_UA2594AA	6	445
ESC_UA2593AA	6	445
ESC_UA2592AA	6	445
ESC_UA2591AA	11925	444
ESC_UA2590AA	6	445
ESC_UA2589AA	281	355
ESC_UA2588AA	4627	204
ESC_UA2587AA	641	935
ESC_UA2586AA	270	204

ESC_UA2585AA	273	102
ESC_UA2584AA	6	445
ESC_UA2583AA	281	355
ESC_UA2582AA	6	445
ESC_UA2581AA	131	653
ESC_UA2580AA	1434	512
ESC_UA2578AA	442	257
ESC_UA2577AA	8590	212
ESC_UA2575AA	602	257
ESC_UA2574AA	540	286
ESC_UA2573AA	11924	198
ESC_UA2572AA	2522	198
ESC_UA2571AA	68	386
ESC_UA2570AA	140	738
ESC_UA2569AA	11	936
ESC_UA2568AA	11	936
ESC_UA2567AA	11	936
ESC_UA2566AA	11	936
ESC_UA2565AA	11	936
ESC_UA2564AA	11	936
ESC_UA2563AA	69	387
ESC_UA2562AA	4253	512
ESC_UA2561AA	201	257
ESC_UA2560AA	2522	198
ESC_UA2559AA	2473	257
ESC_UA2558AA	3947	337
ESC_UA2556AA	48	76
ESC_UA2555AA	1611	198
ESC_UA2553AA	10	512
ESC_UA2552AA	58	198
ESC_UA2551AA	191	198
ESC_UA2550AA	38	669
ESC_UA2549AA	4085	791
ESC_UA2548AA	6726	493
ESC_UA2547AA	10	512
ESC_UA2546AA	69	387
ESC_UA2545AA	4086	842
ESC_UA2544AA	1642	198
ESC_UA2543AA	38	669
ESC_UA2542AA	11928	160
ESC_UA2541AA	48	76
ESC_UA2540AA	382	481
ESC_UA2539AA	569	738
ESC_UA2538AA	993	789
ESC_UA2537AA	993	789
ESC_UA2536AA	3075	701
ESC_UA2535AA	17	191
ESC_UA2534AA	17	191
ESC_UA2533AA	48	76
ESC_UA2532AA	196	257

ESC_UA2531AA	10	512
ESC_UA2530AA	898	108
ESC_UA2529AA	5786	513
ESC_UA2528AA	10	512
ESC_UA2527AA	162	924
ESC_UA2526AA	7616	257
ESC_UA2525AA	3602	234
ESC_UA2524AA	58	198
ESC_UA2523AA	10	512
ESC_UA2522AA	201	257
ESC_UA2521AA	657	592
ESC_UA2520AA	11923	900
ESC_UA2519AA	10	512
ESC_UA2518AA	23	156
ESC_UA2517AA	770	587
ESC_UA2516AA	410	156
ESC_UA2515AA	101	696
ESC_UA2514AA	8582	912
ESC_UA2513AA	348	175
ESC_UA2512AA	301	307
ESC_UA2511AA	542	824
ESC_UA2510AA	10774	512
ESC_UA2509AA	101	696
ESC_UA2508AA	11922	512
ESC_UA2507AA	3234	257
ESC_UA2506AA	117	592
ESC_UA2505AA	6841	160
ESC_UA2504AA	5765	234
ESC_UA2503AA	328	924
ESC_UA2502AA	716	795
ESC_UA2499AA	1122	307
ESC_UA2498AA	7324	257
ESC_UA2497AA	1125	198
ESC_UA2496AA	1771	868
ESC_UA2495AA	7008	900
ESC_UA2494AA	388	156
ESC_UA2493AA	6534	101
ESC_UA2492AA	101	696
ESC_UA2491AA	4151	971
ESC_UA2490AA	189	504
ESC_UA2489AA	131	653
ESC_UA2488AA	648	359
ESC_UA2487AA	155	175
ESC_UA2486AA	141	644
ESC_UA2485AA	127	738
ESC_UA2484AA	1735	737
ESC_UA2483AA	11927	175
ESC_UA2482AA	173	254
ESC_UA2481AA	173	254
ESC_UA2480AA	127	738

ESC_UA2479AA	372	424
ESC_UA2478AA	38	669
ESC_UA2477AA	681	393
ESC_UA2476AA	1670	438
ESC_UA2475AA	11463	868
ESC_UA2473AA	44	512
ESC_UA2472AA	1914	956
ESC_UA2471AA	410	156
ESC_UA2470AA	224	198
ESC_UA2469AA	372	424
ESC_UA2468AA	1193	761
ESC_UA2467AA	372	424
ESC_UA2466AA	224	198
ESC_UA2465AA	405	923
ESC_UA2464AA	73	737
ESC_UA2463AA	68	386
ESC_UA2462AA	372	424
ESC_UA2461AA	2558	782
ESC_UA2460AA	73	737
ESC_UA2459AA	372	424
ESC_UA2458AA	73	737
ESC_UA2457AA	963	669
ESC_UA2456AA	117	592
ESC_UA2455AA	1884	234
ESC_UA2454AA	73	737
ESC_UA2453AA	4166	936
ESC_UA2452AA	73	737
ESC_UA2451AA	453	935
ESC_UA2450AA	127	738
ESC_UA2448AA	679	257
ESC_UA2447AA	2966	936
ESC_UA2446AA	11	936
ESC_UA2445AA	73	737
ESC_UA2444AA	11	936
ESC_UA2443AA	23	156
ESC_UA2442AA	243	197
ESC_UA2441AA	152	257
ESC_UA2440AA	245	261
ESC_UA2439AA	245	261
ESC_UA2438AA	152	257
ESC_UA2437AA	718	257
ESC_UA2436AA	162	924
ESC_UA2435AA	10	512
ESC_UA2434AA	10	512
ESC_UA2433AA	2539	257
ESC_UA2432AA	720	900
ESC_UA2431AA	10	512
ESC_UA2430AA	3270	76
ESC_UA2429AA	718	257
ESC_UA2428AA	6527	629

ESC_UA2427AA	10	512
ESC_UA2426AA	720	900
ESC_UA2425AA	720	900
ESC_UA2424AA	2622	537
ESC_UA2423AA	11834	234
ESC_UA2422AA	1611	198
ESC_UA2421AA	162	924
ESC_UA2419AA	10955	234
ESC_UA2418AA	3056	174
ESC_UA2417AA	1623	891
ESC_UA2416AA	3056	174
ESC_UA2415AA	442	257
ESC_UA2414AA	10	512
ESC_UA2413AA	1611	198
ESC_UA2412AA	442	257
ESC_UA2411AA	6643	198
ESC_UA2410AA	297	234
ESC_UA2409AA	6527	629
ESC_UA2408AA	1246	198
ESC_UA2407AA	5973	92
ESC_UA2406AA	1611	198
ESC_UA2405AA	2622	537
ESC_UA2404AA	5973	92
ESC_UA2403AA	11527	80
ESC_UA2402AA	1611	198
ESC_UA2401AA	58	198
ESC_UA2400AA	154	257
ESC_UA2399AA	1611	198
ESC_UA2398AA	906	198
ESC_UA2397AA	394	400
ESC_UA2396AA	10	512
ESC_UA2395AA	11818	878
ESC_UA2393AA	155	175
ESC_UA2392AA	1800	924
ESC_UA2391AA	117	592
ESC_UA2390AA	6527	629
ESC_UA2389AA	906	198
ESC_UA2388AA	8463	198
ESC_UA2387AA	5973	92
ESC_UA2386AA	1125	198
ESC_UA2385AA	58	198
ESC_UA2384AA	156	175
ESC_UA2383AA	10	512
ESC_UA2381AA	5597	72
ESC_UA2380AA	5966	229
ESC_UA2379AA	162	924
ESC_UA2378AA	3056	174
ESC_UA2377AA	13	257
ESC_UA2376AA	1228	899
ESC_UA2375AA	2521	257

ESC_UA2374AA	10	512
ESC_UA2373AA	350	50
ESC_UA2372AA	1123	257
ESC_UA2371AA	642	924
ESC_UA2370AA	1123	257
ESC_UA2369AA	5973	92
ESC_UA2368AA	1992	257
ESC_UA2367AA	1623	891
ESC_UA2366AA	1611	198
ESC_UA2365AA	3246	900
ESC_UA2364AA	1623	891
ESC_UA2363AA	34	504
ESC_UA2362AA	1611	198
ESC_UA2361AA	5973	92
ESC_UA2360AA	1992	257
ESC_UA2359AA	10	512
ESC_UA2358AA	5597	72
ESC_UA2357AA	661	92
ESC_UA2356AA	5973	92
ESC_UA2355AA	11833	924
ESC_UA2354AA	392	234
ESC_UA2353AA	1246	198
ESC_UA2351AA	23	156
ESC_UA2350AA	5973	92
ESC_UA2349AA	10	512
ESC_UA2348AA	753	842
ESC_UA2347AA	5973	92
ESC_UA2346AA	5973	92
ESC_UA2345AA	11817	156
ESC_UA2344AA	23	156
ESC_UA2343AA	297	234
ESC_UA2342AA	1665	601
ESC_UA2341AA	3056	174
ESC_UA2340AA	1611	198
ESC_UA2339AA	10	512
ESC_UA2338AA	5973	92
ESC_UA2337AA	10	512
ESC_UA2336AA	5973	92
ESC_UA2335AA	67	257
ESC_UA2334AA	67	257
ESC_UA2333AA	8463	198
ESC_UA2332AA	11527	80
ESC_UA2331AA	336	878
ESC_UA2330AA	336	878
ESC_UA2329AA	1611	198
ESC_UA2328AA	442	257
ESC_UA2327AA	58	198
ESC_UA2326AA	13	257
ESC_UA2325AA	1084	180
ESC_UA2324AA	58	198

ESC_UA2323AA	11527	80
ESC_UA2322AA	58	198
ESC_UA2321AA	10	512
ESC_UA2320AA	1246	198
ESC_UA2319AA	6985	915
ESC_UA2318AA	297	234
ESC_UA2317AA	10	512
ESC_UA2316AA	337	257
ESC_UA2315AA	58	198
ESC_UA2314AA	6239	11
ESC_UA2313AA	154	257
ESC_UA2312AA	8852	213
ESC_UA2311AA	362	234
ESC_UA2310AA	155	175
ESC_UA2309AA	1611	198
ESC_UA2308AA	1172	234
ESC_UA2307AA	1172	234
ESC_UA2306AA	720	900
ESC_UA2305AA	906	198
ESC_UA2304AA	23	156
ESC_UA2303AA	10	512
ESC_UA2302AA	10	512
ESC_UA2301AA	155	175
ESC_UA2300AA	642	924
ESC_UA2298AA	4151	971
ESC_UA2297AA	4151	971
ESC_UA2295AA	3056	174
ESC_UA2294AA	10	512
ESC_UA2293AA	155	175
ESC_UA2292AA	10	512
ESC_UA2291AA	10	512
ESC_UA2290AA	1800	924
ESC_UA2289AA	10	512
ESC_UA2288AA	442	257
ESC_UA2286AA	10	512
ESC_UA2285AA	10	512
ESC_UA2284AA	10	512
ESC_UA2283AA	1800	924
ESC_UA2282AA	11832	257
ESC_UA2281AA	10	512
ESC_UA2280AA	13	257
ESC_UA2279AA	329	355
ESC_UA2278AA	9120	234
ESC_UA2277AA	58	198
ESC_UA2276AA	3519	101
ESC_UA2275AA	3519	101
ESC_UA2274AA	8852	213
ESC_UA2273AA	448	257
ESC_UA2272AA	11831	734
ESC_UA2271AA	3519	101

ESC_UA2270AA	1611	198
ESC_UA2269AA	11830	1036
ESC_UA2268AA	1611	198
ESC_UA2267AA	348	175
ESC_UA2266AA	328	924
ESC_UA2265AA	56	198
ESC_UA2264AA	56	198
ESC_UA2263AA	1817	842
ESC_UA2262AA	58	198
ESC_UA2261AA	10955	234
ESC_UA2260AA	345	175
ESC_UA2258AA	3056	174
ESC_UA2255AA	5973	92
ESC_UA2254AA	5973	92
ESC_UA2253AA	58	198
ESC_UA2252AA	10	512
ESC_UA2249AA	718	257
ESC_UA2248AA	23	156
ESC_UA2247AA	10	512
ESC_UA2246AA	58	198
ESC_UA2245AA	5966	229
ESC_UA2244AA	647	734
ESC_UA2243AA	10	512
ESC_UA2242AA	56	198
ESC_UA2239AA	8828	669
ESC_UA2238AA	11829	235
ESC_UA2237AA	43	512
ESC_UA2236AA	8828	669
ESC_UA2235AA	1623	891
ESC_UA2234AA	5973	92
ESC_UA2233AA	5973	92
ESC_UA2232AA	8463	198
ESC_UA2231AA	5597	72
ESC_UA2230AA	297	234
ESC_UA2229AA	8463	198
ESC_UA2228AA	7949	512
ESC_UA2227AA	4038	257
ESC_UA2226AA	10	512
ESC_UA2225AA	1611	198
ESC_UA2223AA	108	257
ESC_UA2222AA	348	175
ESC_UA2221AA	2308	257
ESC_UA2220AA	2308	257
ESC_UA2219AA	108	257
ESC_UA2218AA	56	198
ESC_UA2217AA	7949	512
ESC_UA2216AA	10	512
ESC_UA2215AA	5597	72
ESC_UA2214AA	1081	198
ESC_UA2213AA	1081	198

ESC_UA2212AA	11818	878
ESC_UA2211AA	6060	41
ESC_UA2210AA	155	175
ESC_UA2209AA	5934	257
ESC_UA2208AA	10	512
ESC_UA2207AA	1081	198
ESC_UA2206AA	1858	757
ESC_UA2205AA	441	198
ESC_UA2204AA	5825	924
ESC_UA2203AA	11825	333
ESC_UA2202AA	641	935
ESC_UA2201AA	446	257
ESC_UA2200AA	2521	257
ESC_UA2199AA	11828	924
ESC_UA2198AA	1081	198
ESC_UA2197AA	11818	878
ESC_UA2196AA	11817	156
ESC_UA2195AA	10	512
ESC_UA2194AA	2028	257
ESC_UA2193AA	10	512
ESC_UA2192AA	155	175
ESC_UA2191AA	1079	235
ESC_UA2190AA	1623	891
ESC_UA2189AA	54	256
ESC_UA2188AA	10	512
ESC_UA2187AA	11825	333
ESC_UA2186AA	2164	257
ESC_UA2185AA	3519	101
ESC_UA2184AA	155	175
ESC_UA2182AA	155	175
ESC_UA2181AA	11827	198
ESC_UA2180AA	2028	257
ESC_UA2179AA	2028	257
ESC_UA2178AA	56	198
ESC_UA2177AA	441	198
ESC_UA2176AA	56	198
ESC_UA2175AA	11825	333
ESC_UA2174AA	1403	924
ESC_UA2173AA	295	160
ESC_UA2172AA	57	50
ESC_UA2171AA	394	400
ESC_UA2170AA	4038	257
ESC_UA2169AA	155	175
ESC_UA2168AA	7348	331
ESC_UA2167AA	753	842
ESC_UA2166AA	7348	331
ESC_UA2165AA	162	924
ESC_UA2164AA	641	935
ESC_UA2163AA	1081	198
ESC_UA2162AA	5934	257

ESC_UA2161AA	1228	899
ESC_UA2160AA	155	175
ESC_UA2158AA	2436	179
ESC_UA2157AA	155	175
ESC_UA2155AA	11817	156
ESC_UA2154AA	10	512
ESC_UA2153AA	155	175
ESC_UA2152AA	1081	198
ESC_UA2151AA	720	900
ESC_UA2150AA	154	257
ESC_UA2149AA	345	175
ESC_UA2148AA	11823	421
ESC_UA2147AA	278	924
ESC_UA2146AA	7495	333
ESC_UA2145AA	7495	333
ESC_UA2144AA	155	175
ESC_UA2143AA	10	512
ESC_UA2142AA	11817	156
ESC_UA2141AA	2622	537
ESC_UA2140AA	164	175
ESC_UA2139AA	58	198
ESC_UA2138AA	8823	257
ESC_UA2137AA	5635	175
ESC_UA2136AA	10	512
ESC_UA2135AA	1308	257
ESC_UA2134AA	88	160
ESC_UA2133AA	1623	891
ESC_UA2132AA	155	175
ESC_UA2131AA	336	878
ESC_UA2130AA	1081	198
ESC_UA2129AA	2351	234
ESC_UA2128AA	6060	41
ESC_UA2127AA	155	175
ESC_UA2126AA	155	175
ESC_UA2125AA	11826	24
ESC_UA2124AA	141	644
ESC_UA2123AA	1665	601
ESC_UA2122AA	156	175
ESC_UA2121AA	10	512
ESC_UA2120AA	155	175
ESC_UA2119AA	154	257
ESC_UA2118AA	1623	891
ESC_UA2117AA	10	512
ESC_UA2116AA	124	254
ESC_UA2115AA	33	198
ESC_UA2114AA	156	175
ESC_UA2113AA	3519	101
ESC_UA2112AA	7348	331
ESC_UA2111AA	11823	421
ESC_UA2110AA	11823	421

ESC_UA2109AA	155	175
ESC_UA2108AA	11825	333
ESC_UA2107AA	10	512
ESC_UA2106AA	54	256
ESC_UA2105AA	1611	198
ESC_UA2104AA	661	92
ESC_UA2103AA	11825	333
ESC_UA2102AA	394	400
ESC_UA2101AA	162	924
ESC_UA2100AA	155	175
ESC_UA2099AA	33	198
ESC_UA2098AA	906	198
ESC_UA2097AA	11818	878
ESC_UA2096AA	155	175
ESC_UA2095AA	441	198
ESC_UA2094AA	99	198
ESC_UA2093AA	441	198
ESC_UA2092AA	5976	924
ESC_UA2091AA	101	696
ESC_UA2090AA	156	175
ESC_UA2089AA	11817	156
ESC_UA2088AA	1623	891
ESC_UA2087AA	10979	257
ESC_UA2086AA	154	257
ESC_UA2085AA	88	160
ESC_UA2084AA	5934	257
ESC_UA2083AA	1623	891
ESC_UA2082AA	164	175
ESC_UA2081AA	5825	924
ESC_UA2080AA	7495	333
ESC_UA2079AA	108	257
ESC_UA2078AA	5825	924
ESC_UA2077AA	1623	891
ESC_UA2076AA	753	842
ESC_UA2075AA	11824	312
ESC_UA2074AA	1718	269
ESC_UA2073AA	2785	175
ESC_UA2072AA	1623	891
ESC_UA2071AA	129	257
ESC_UA2069AA	10	512
ESC_UA2068AA	753	842
ESC_UA2065AA	33	198
ESC_UA2064AA	10	512
ESC_UA2063AA	675	234
ESC_UA2062AA	1205	198
ESC_UA2061AA	154	257
ESC_UA2060AA	155	175
ESC_UA2059AA	1623	891
ESC_UA2058AA	201	257
ESC_UA2057AA	108	257

ESC_UA2056AA	11817	156
ESC_UA2055AA	5825	924
ESC_UA2054AA	11817	156
ESC_UA2053AA	5597	72
ESC_UA2052AA	2785	175
ESC_UA2051AA	1228	899
ESC_UA2050AA	13	257
ESC_UA2049AA	162	924
ESC_UA2047AA	362	234
ESC_UA2046AA	297	234
ESC_UA2045AA	33	198
ESC_UA2044AA	5934	257
ESC_UA2043AA	1086	175
ESC_UA2042AA	8501	108
ESC_UA2041AA	43	512
ESC_UA2040AA	11822	198
ESC_UA2039AA	3519	101
ESC_UA2038AA	4577	257
ESC_UA2037AA	1623	891
ESC_UA2036AA	1609	198
ESC_UA2035AA	155	175
ESC_UA2034AA	3519	101
ESC_UA2033AA	155	175
ESC_UA2032AA	441	198
ESC_UA2031AA	1228	899
ESC_UA2030AA	11820	653
ESC_UA2029AA	394	400
ESC_UA2028AA	11820	653
ESC_UA2027AA	58	198
ESC_UA2026AA	11821	373
ESC_UA2025AA	7949	512
ESC_UA2024AA	201	257
ESC_UA2023AA	345	175
ESC_UA2022AA	1403	924
ESC_UA2021AA	345	175
ESC_UA2020AA	297	234
ESC_UA2019AA	906	198
ESC_UA2018AA	1081	198
ESC_UA2017AA	108	257
ESC_UA2016AA	641	935
ESC_UA2015AA	11819	373
ESC_UA2014AA	156	175
ESC_UA2013AA	129	257
ESC_UA2012AA	1246	198
ESC_UA2011AA	1623	891
ESC_UA2010AA	1161	764
ESC_UA2009AA	155	175
ESC_UA2008AA	3519	101
ESC_UA2007AA	162	924
ESC_UA2006AA	162	924

ESC_UA2005AA	5885	261
ESC_UA2004AA	3519	101
ESC_UA2003AA	753	842
ESC_UA2002AA	10	512
ESC_UA2001AA	10	512
ESC_UA2000AA	10	512
ESC_UA1998AA	641	935
ESC_UA1997AA	641	935
ESC_UA1996AA	13	257
ESC_UA1995AA	446	257
ESC_UA1994AA	162	924
ESC_UA1993AA	7495	333
ESC_UA1992AA	155	175
ESC_UA1991AA	11817	156
ESC_UA1990AA	753	842
ESC_UA1989AA	58	198
ESC_UA1988AA	7	842
ESC_UA1987AA	11820	653
ESC_UA1986AA	162	924
ESC_UA1985AA	108	257
ESC_UA1984AA	11819	373
ESC_UA1983AA	11819	373
ESC_UA1982AA	7348	331
ESC_UA1981AA	1125	198
ESC_UA1980AA	753	842
ESC_UA1979AA	11818	878
ESC_UA1978AA	5474	198
ESC_UA1977AA	11823	421
ESC_UA1976AA	164	175
ESC_UA1975AA	7804	369
ESC_UA1974AA	753	842
ESC_UA1973AA	6060	41
ESC_UA1972AA	1623	891
ESC_UA1971AA	1081	198
ESC_UA1970AA	11817	156
ESC_UA1969AA	11817	156
ESC_UA1968AA	10	512
ESC_UA1967AA	8463	198
ESC_UA1966AA	162	924
ESC_UA1965AA	1081	198
ESC_UA1964AA	33	198
ESC_UA1963AA	5934	257
ESC_UA1962AA	154	257
ESC_UA1961AA	441	198
ESC_UA1960AA	441	198
ESC_UA1959AA	441	198
ESC_UA1958AA	8823	257
ESC_UA1957AA	1170	629
ESC_UA1956AA	1161	764
ESC_UA1955AA	1170	629

ESC_UA1954AA	108	257
ESC_UA1953AA	753	842
ESC_UA1952AA	11818	878
ESC_UA1951AA	446	257
ESC_UA1950AA	57	50
ESC_UA1949AA	348	175
ESC_UA1948AA	5597	72
ESC_UA1947AA	5597	72
ESC_UA1946AA	10	512
ESC_UA1945AA	1623	891
ESC_UA1944AA	155	175
ESC_UA1943AA	1665	601
ESC_UA1942AA	3	198
ESC_UA1941AA	3	198
ESC_UA1940AA	206	286
ESC_UA1939AA	29	156
ESC_UA1938AA	3	198
ESC_UA1937AA	3	198
ESC_UA1936AA	3	198
ESC_UA1935AA	3	198
ESC_UA1934AA	3	198
ESC_UA1933AA	3	198
ESC_UA1932AA	3	198
ESC_UA1931AA	3	198
ESC_UA1930AA	3	198
ESC_UA1929AA	3	198
ESC_UA1928AA	3	198
ESC_UA1927AA	3	198
ESC_UA1926AA	3	198
ESC_UA1925AA	3	198
ESC_UA1924AA	3	198
ESC_UA1923AA	3	198
ESC_UA1922AA	3	198
ESC_UA1921AA	3	198
ESC_UA1920AA	3	198
ESC_UA1919AA	3	198
ESC_UA1918AA	3	198
ESC_UA1917AA	3	198
ESC_UA1916AA	3	198
ESC_UA1915AA	3	198
ESC_UA1914AA	3	198
ESC_UA1913AA	3	198
ESC_UA1912AA	3	198
ESC_UA1911AA	3	198
ESC_UA1910AA	3	198
ESC_UA1909AA	3	198
ESC_UA1908AA	3	198
ESC_UA1907AA	3	198
ESC_UA1906AA	3	198
ESC_UA1905AA	3	198

ESC_UA1904AA	11816	282
ESC_UA1903AA	3	198
ESC_UA1902AA	3	198
ESC_UA1901AA	3	198
ESC_UA1900AA	3	198
ESC_UA1899AA	3	198
ESC_UA1898AA	3	198
ESC_UA1897AA	3	198
ESC_UA1896AA	3	198
ESC_UA1895AA	3	198
ESC_UA1894AA	152	257
ESC_UA1893AA	3	198
ESC_UA1892AA	3	198
ESC_UA1891AA	3	198
ESC_UA1890AA	3	198
ESC_UA1889AA	3	198
ESC_UA1888AA	3	198
ESC_UA1887AA	3	198
ESC_UA1886AA	3	198
ESC_UA1885AA	3	198
ESC_UA1884AA	11815	198
ESC_UA1883AA	3	198
ESC_UA1882AA	3	198
ESC_UA1881AA	3	198
ESC_UA1880AA	3	198
ESC_UA1879AA	3	198
ESC_UA1878AA	3	198
ESC_UA1877AA	73	737
ESC_UA1875AA	127	738
ESC_UA1874AA	372	424
ESC_UA1873AA	68	386
ESC_UA1872AA	295	160
ESC_UA1871AA	1193	761
ESC_UA1870AA	420	741
ESC_UA1869AA	167	512
ESC_UA1868AA	5954	923
ESC_UA1867AA	361	512
ESC_UA1866AA	1193	761
ESC_UA1865AA	11021	512
ESC_UA1864AA	361	512
ESC_UA1863AA	108	257
ESC_UA1862AA	167	512
ESC_UA1861AA	131	653
ESC_UA1860AA	410	156
ESC_UA1859AA	11681	0
ESC_UA1858AA	410	156
ESC_UA1857AA	1193	761
ESC_UA1856AA	5909	76
ESC_UA1855AA	457	595
ESC_UA1854AA	127	738

ESC_UA1853AA	131	653
ESC_UA1852AA	245	261
ESC_UA1851AA	11814	261
ESC_UA1850AA	152	257
ESC_UA1849AA	152	257
ESC_UA1848AA	152	257
ESC_UA1847AA	152	257
ESC_UA1846AA	7384	257
ESC_UA1845AA	245	261
ESC_UA1844AA	245	261
ESC_UA1843AA	152	257
ESC_UA1842AA	152	257
ESC_UA1841AA	630	261
ESC_UA1840AA	245	261
ESC_UA1839AA	245	261
ESC_UA1838AA	630	261
ESC_UA1837AA	245	261
ESC_UA1836AA	152	257
ESC_UA1835AA	152	257
ESC_UA1834AA	152	257
ESC_UA1833AA	245	261
ESC_UA1832AA	245	261
ESC_UA1831AA	152	257
ESC_UA1830AA	245	261
ESC_UA1829AA	245	261
ESC_UA1828AA	245	261
ESC_UA1827AA	245	261
ESC_UA1826AA	152	257
ESC_UA1825AA	245	261
ESC_UA1824AA	152	257
ESC_UA1823AA	1753	261
ESC_UA1822AA	1753	261
ESC_UA1821AA	245	261
ESC_UA1820AA	542	824
ESC_UA1819AA	652	204
ESC_UA1818AA	316	257
ESC_UA1817AA	58	198
ESC_UA1815AA	48	76
ESC_UA1814AA	117	592
ESC_UA1813AA	1011	198
ESC_UA1812AA	297	234
ESC_UA1811AA	117	592
ESC_UA1810AA	429	654
ESC_UA1809AA	38	669
ESC_UA1808AA	1684	504
ESC_UA1807AA	3234	257
ESC_UA1806AA	58	198
ESC_UA1805AA	162	924
ESC_UA1804AA	101	696
ESC_UA1803AA	108	257

ESC_UA1802AA	355	764
ESC_UA1801AA	48	76
ESC_UA1800AA	744	512
ESC_UA1799AA	223	198
ESC_UA1798AA	162	924
ESC_UA1797AA	877	935
ESC_UA1796AA	10636	198
ESC_UA1795AA	109	257
ESC_UA1794AA	616	156
ESC_UA1793AA	10	512
ESC_UA1792AA	57	50
ESC_UA1791AA	602	257
ESC_UA1790AA	973	968
ESC_UA1789AA	189	504
ESC_UA1788AA	162	924
ESC_UA1787AA	2079	198
ESC_UA1786AA	6189	175
ESC_UA1785AA	212	198
ESC_UA1784AA	349	670
ESC_UA1783AA	752	512
ESC_UA1782AA	162	924
ESC_UA1781AA	10	512
ESC_UA1780AA	4597	247
ESC_UA1779AA	10	512
ESC_UA1778AA	5478	924
ESC_UA1777AA	10	512
ESC_UA1776AA	11892	789
ESC_UA1775AA	10	512
ESC_UA1774AA	270	204
ESC_UA1773AA	311	204
ESC_UA1772AA	3580	257
ESC_UA1771AA	359	696
ESC_UA1770AA	58	198
ESC_UA1769AA	115	669
ESC_UA1768AA	453	935
ESC_UA1767AA	1594	71
ESC_UA1766AA	453	935
ESC_UA1765AA	2178	924
ESC_UA1764AA	2178	924
ESC_UA1743AA	131	653
ESC_UA1741AA	131	653
ESC_UA1740AA	131	653
ESC_UA1739AA	131	653
ESC_UA1738AA	131	653
ESC_UA1737AA	131	653
ESC_UA1736AA	131	653
ESC_UA1735AA	131	653
ESC_UA1734AA	131	653
ESC_UA1733AA	131	653
ESC_UA1732AA	131	653

ESC_UA1731AA	131	653
ESC_UA1730AA	131	653
ESC_UA1729AA	131	653
ESC_UA1728AA	131	653
ESC_UA1727AA	131	653
ESC_UA1726AA	131	653
ESC_UA1725AA	131	653
ESC_UA1724AA	131	653
ESC_UA1723AA	131	653
ESC_UA1722AA	131	653
ESC_UA1721AA	131	653
ESC_UA1720AA	131	653
ESC_UA1719AA	131	653
ESC_UA1718AA	131	653
ESC_UA1717AA	131	653
ESC_UA1716AA	131	653
ESC_UA1715AA	131	653
ESC_UA1714AA	131	653
ESC_UA1713AA	131	653
ESC_UA1712AA	131	653
ESC_UA1711AA	131	653
ESC_UA1710AA	131	653
ESC_UA1709AA	131	653
ESC_UA1708AA	131	653
ESC_UA1707AA	131	653
ESC_UA1706AA	131	653
ESC_UA1705AA	131	653
ESC_UA1704AA	131	653
ESC_UA1703AA	131	653
ESC_UA1702AA	131	653
ESC_UA1701AA	131	653
ESC_UA1700AA	131	653
ESC_UA1699AA	131	653
ESC_UA1698AA	131	653
ESC_UA1697AA	131	653
ESC_UA1696AA	131	653
ESC_UA1695AA	131	653
ESC_UA1694AA	131	653
ESC_UA1693AA	131	653
ESC_UA1692AA	131	653
ESC_UA1691AA	131	653
ESC_UA1690AA	131	653
ESC_UA1689AA	131	653
ESC_UA1688AA	131	653
ESC_UA1687AA	131	653
ESC_UA1686AA	131	653
ESC_UA1685AA	131	653
ESC_UA1684AA	131	653
ESC_UA1683AA	131	653
ESC_UA1682AA	131	653

ESC_UA1681AA	131	653
ESC_UA1680AA	131	653
ESC_UA1679AA	131	653
ESC_UA1678AA	131	653
ESC_UA1677AA	131	653
ESC_UA1676AA	131	653
ESC_UA1675AA	131	653
ESC_UA1674AA	131	653
ESC_UA1673AA	131	653
ESC_UA1672AA	131	653
ESC_UA1671AA	131	653
ESC_UA1670AA	131	653
ESC_UA1669AA	131	653
ESC_UA1668AA	131	653
ESC_UA1667AA	131	653
ESC_UA1666AA	131	653
ESC_UA1665AA	131	653
ESC_UA1664AA	131	653
ESC_UA1663AA	131	653
ESC_UA1662AA	131	653
ESC_UA1661AA	131	653
ESC_UA1660AA	131	653
ESC_UA1659AA	131	653
ESC_UA1658AA	131	653
ESC_UA1657AA	131	653
ESC_UA1656AA	131	653
ESC_UA1655AA	131	653
ESC_UA1654AA	131	653
ESC_UA1653AA	131	653
ESC_UA1652AA	131	653
ESC_UA1651AA	131	653
ESC_UA1650AA	131	653
ESC_UA1649AA	131	653
ESC_UA1648AA	131	653
ESC_UA1647AA	131	653
ESC_UA1646AA	131	653
ESC_UA1645AA	131	653
ESC_UA1644AA	131	653
ESC_UA1643AA	131	653
ESC_UA1642AA	131	653
ESC_UA1641AA	131	653
ESC_UA1640AA	131	653
ESC_UA1639AA	131	653
ESC_UA1638AA	131	653
ESC_UA1637AA	131	653
ESC_UA1636AA	131	653
ESC_UA1635AA	131	653
ESC_UA1634AA	131	653
ESC_UA1633AA	131	653
ESC_UA1632AA	131	653

ESC_UA1631AA	131	653
ESC_UA1630AA	131	653
ESC_UA1629AA	131	653
ESC_UA1628AA	131	653
ESC_UA1627AA	131	653
ESC_UA1626AA	131	653
ESC_UA1625AA	131	653
ESC_UA1624AA	131	653
ESC_UA1623AA	131	653
ESC_UA1622AA	131	653
ESC_UA1621AA	131	653
ESC_UA1620AA	131	653
ESC_UA1619AA	131	653
ESC_UA1618AA	131	653
ESC_UA1617AA	131	653
ESC_UA1615AA	131	653
ESC_UA1614AA	131	653
ESC_UA1613AA	131	653
ESC_UA1612AA	131	653
ESC_UA1611AA	131	653
ESC_UA1610AA	131	653
ESC_UA1609AA	131	653
ESC_UA1608AA	131	653
ESC_UA1606AA	131	653
ESC_UA1605AA	131	653
ESC_UA1604AA	131	653
ESC_UA1603AA	131	653
ESC_UA1602AA	131	653
ESC_UA1601AA	131	653
ESC_UA1600AA	131	653
ESC_UA1599AA	131	653
ESC_UA1598AA	131	653
ESC_UA1597AA	131	653
ESC_UA1596AA	131	653
ESC_UA1595AA	131	653
ESC_UA1594AA	11890	380
ESC_UA1593AA	131	653
ESC_UA1592AA	131	653
ESC_UA1591AA	131	653
ESC_UA1590AA	131	653
ESC_UA1588AA	131	653
ESC_UA1587AA	131	653
ESC_UA1586AA	131	653
ESC_UA1585AA	131	653
ESC_UA1583AA	131	653
ESC_UA1582AA	131	653
ESC_UA1581AA	131	653
ESC_UA1580AA	131	653
ESC_UA1579AA	131	653
ESC_UA1578AA	131	653

ESC_UA1577AA	131	653
ESC_UA1576AA	131	653
ESC_UA1575AA	131	653
ESC_UA1574AA	131	653
ESC_UA1573AA	131	653
ESC_UA1572AA	131	653
ESC_UA1571AA	131	653
ESC_UA1570AA	131	653
ESC_UA1569AA	131	653
ESC_UA1568AA	131	653
ESC_UA1567AA	131	653
ESC_UA1566AA	131	653
ESC_UA1565AA	131	653
ESC_UA1564AA	131	653
ESC_UA1563AA	131	653
ESC_UA1562AA	131	653
ESC_UA1561AA	131	653
ESC_UA1560AA	131	653
ESC_UA1559AA	131	653
ESC_UA1558AA	131	653
ESC_UA1557AA	131	653
ESC_UA1556AA	131	653
ESC_UA1555AA	131	653
ESC_UA1554AA	131	653
ESC_UA1553AA	131	653
ESC_UA1552AA	131	653
ESC_UA1551AA	131	653
ESC_UA1550AA	131	653
ESC_UA1549AA	131	653
ESC_UA1548AA	131	653
ESC_UA1547AA	131	653
ESC_UA1546AA	131	653
ESC_UA1545AA	131	653
ESC_UA1544AA	131	653
ESC_UA1543AA	58	198
ESC_UA1541AA	131	653
ESC_UA1540AA	131	653
ESC_UA1539AA	6694	234
ESC_UA1538AA	366	198
ESC_UA1537AA	131	653
ESC_UA1536AA	515	1004
ESC_UA1535AA	69	387
ESC_UA1534AA	101	696
ESC_UA1533AA	1611	198
ESC_UA1531AA	388	156
ESC_UA1530AA	5748	924
ESC_UA1529AA	101	696
ESC_UA1528AA	118	985
ESC_UA1527AA	641	935
ESC_UA1526AA	201	257

ESC_UA1525AA	101	696
ESC_UA1524AA	925	387
ESC_UA1523AA	549	387
ESC_UA1522AA	4248	166
ESC_UA1521AA	192	696
ESC_UA1520AA	361	512
ESC_UA1519AA	69	387
ESC_UA1518AA	69	387
ESC_UA1517AA	2179	924
ESC_UA1516AA	131	653
ESC_UA1515AA	117	592
ESC_UA1514AA	117	592
ESC_UA1513AA	88	160
ESC_UA1512AA	131	653
ESC_UA1511AA	131	653
ESC_UA1510AA	1722	599
ESC_UA1509AA	46	447
ESC_UA1508AA	136	392
ESC_UA1507AA	58	198
ESC_UA1506AA	131	653
ESC_UA1505AA	69	387
ESC_UA1504AA	131	653
ESC_UA1503AA	131	653
ESC_UA1502AA	162	924
ESC_UA1501AA	88	160
ESC_UA1500AA	88	160
ESC_UA1499AA	38	669
ESC_UA1497AA	131	653
ESC_UA1496AA	2792	886
ESC_UA1495AA	131	653
ESC_UA1494AA	540	286
ESC_UA1493AA	3	198
ESC_UA1492AA	3	198
ESC_UA1491AA	3	198
ESC_UA1490AA	3	198
ESC_UA1489AA	3	198
ESC_UA1488AA	3	198
ESC_UA1487AA	3	198
ESC_UA1486AA	3	198
ESC_UA1485AA	11812	24
ESC_UA1484AA	3	198
ESC_UA1483AA	3	198
ESC_UA1482AA	3	198
ESC_UA1481AA	3	198
ESC_UA1480AA	3	198
ESC_UA1479AA	3	198
ESC_UA1478AA	3	198
ESC_UA1477AA	3	198
ESC_UA1476AA	3	198
ESC_UA1475AA	3	198

ESC_UA1474AA	3	198
ESC_UA1473AA	3	198
ESC_UA1472AA	21	156
ESC_UA1471AA	245	261
ESC_UA1470AA	152	257
ESC_UA1469AA	245	261
ESC_UA1468AA	245	261
ESC_UA1467AA	148	191
ESC_UA1466AA	678	257
ESC_UA1465AA	2389	160
ESC_UA1464AA	278	924
ESC_UA1463AA	2967	289
ESC_UA1462AA	201	257
ESC_UA1461AA	335	936
ESC_UA1460AA	301	307
ESC_UA1459AA	191	198
ESC_UA1458AA	401	142
ESC_UA1457AA	401	142
ESC_UA1456AA	191	198
ESC_UA1455AA	5493	198
ESC_UA1454AA	1324	156
ESC_UA1453AA	9734	142
ESC_UA1452AA	401	142
ESC_UA1451AA	9734	142
ESC_UA1450AA	9716	356
ESC_UA1449AA	245	261
ESC_UA1448AA	301	307
ESC_UA1447AA	301	307
ESC_UA1390AA	10	512
ESC_UA1389AA	10	512
ESC_UA1388AA	10	512
ESC_UA1387AA	10	512
ESC_UA1383AA	10	512
ESC_UA1382AA	10	512
ESC_UA1381AA	10	512
ESC_UA1380AA	10	512
ESC_UA1378AA	10	512
ESC_UA1376AA	10	512
ESC_UA1375AA	10	512
ESC_UA1374AA	10	512
ESC_UA1369AA	17	191
ESC_UA1368AA	119	286
ESC_UA1367AA	94	257
ESC_UA1366AA	316	257
ESC_UA1365AA	6872	601
ESC_UA1364AA	155	175
ESC_UA1363AA	4599	504
ESC_UA1361AA	1308	257
ESC_UA1359AA	21	156
ESC_UA1358AA	16	156

ESC_UA1357AA	88	160
ESC_UA1356AA	75	257
ESC_UA1354AA	131	653
ESC_UA1353AA	58	198
ESC_UA1352AA	58	198
ESC_UA1351AA	369	156
ESC_UA1350AA	88	160
ESC_UA1349AA	491	626
ESC_UA1348AA	58	198
ESC_UA1347AA	1079	235
ESC_UA1346AA	369	156
ESC_UA1345AA	4170	87
ESC_UA1344AA	21	156
ESC_UA1343AA	659	286
ESC_UA1342AA	17	191
ESC_UA1341AA	16	156
ESC_UA1340AA	659	286
ESC_UA1339AA	659	286
ESC_UA1338AA	5923	763
ESC_UA1337AA	21	156
ESC_UA1336AA	342	286
ESC_UA1335AA	21	156
ESC_UA1334AA	11	936
ESC_UA1333AA	17	191
ESC_UA1332AA	21	156
ESC_UA1331AA	32	270
ESC_UA1330AA	11877	156
ESC_UA1329AA	2101	108
ESC_UA1328AA	11	936
ESC_UA1327AA	11	936
ESC_UA1326AA	11	936
ESC_UA1325AA	11	936
ESC_UA1324AA	11	936
ESC_UA1323AA	11	936
ESC_UA1322AA	11	936
ESC_UA1321AA	11	936
ESC_UA1320AA	11	936
ESC_UA1319AA	16	156
ESC_UA1318AA	11	936
ESC_UA1317AA	297	234
ESC_UA1315AA	21	156
ESC_UA1314AA	16	156
ESC_UA1313AA	33	198
ESC_UA1312AA	342	286
ESC_UA1311AA	32	270
ESC_UA1310AA	11	936
ESC_UA1309AA	11	936
ESC_UA1308AA	11	936
ESC_UA1307AA	11	936
ESC_UA1306AA	11	936

ESC_UA1305AA	11	936
ESC_UA1304AA	11	936
ESC_UA1303AA	11	936
ESC_UA1302AA	11	936
ESC_UA1301AA	11879	696
ESC_UA1300AA	17	191
ESC_UA1299AA	21	156
ESC_UA1298AA	11	936
ESC_UA1297AA	11	936
ESC_UA1296AA	11	936
ESC_UA1295AA	11	936
ESC_UA1294AA	11	936
ESC_UA1293AA	11	936
ESC_UA1292AA	156	175
ESC_UA1291AA	11	936
ESC_UA1289AA	59	217
ESC_UA1288AA	11	936
ESC_UA1282AA	10	512
ESC_UA1281AA	10	512
ESC_UA1280AA	10	512
ESC_UA1279AA	11878	512
ESC_UA1278AA	10	512
ESC_UA1277AA	10	512
ESC_UA1276AA	10	512
ESC_UA1275AA	10	512
ESC_UA1274AA	10	512
ESC_UA1273AA	10	512
ESC_UA1272AA	10	512
ESC_UA1271AA	10	512
ESC_UA1270AA	10	512
ESC_UA1269AA	10	512
ESC_UA1268AA	10	512
ESC_UA1267AA	10	512
ESC_UA1266AA	10	512
ESC_UA1265AA	10	512
ESC_UA1264AA	10	512
ESC_UA1263AA	10	512
ESC_UA1262AA	10	512
ESC_UA1261AA	10	512
ESC_UA1260AA	10	512
ESC_UA1259AA	10	512
ESC_UA1258AA	10	512
ESC_UA1257AA	10	512
ESC_UA1256AA	10	512
ESC_UA1255AA	10	512
ESC_UA1254AA	10	512
ESC_UA1253AA	10	512
ESC_UA1252AA	10	512
ESC_UA1251AA	10	512
ESC_UA1250AA	10	512

ESC_UA1249AA	10	512
ESC_UA1248AA	10	512
ESC_UA1247AA	10	512
ESC_UA1246AA	10	512
ESC_UA1245AA	10	512
ESC_UA1244AA	10	512
ESC_UA1243AA	10	512
ESC_UA1242AA	10	512
ESC_UA1241AA	10	512
ESC_UA1240AA	10	512
ESC_UA1239AA	10	512
ESC_UA1238AA	10	512
ESC_UA1237AA	10	512
ESC_UA1236AA	10	512
ESC_UA1235AA	10	512
ESC_UA1234AA	10	512
ESC_UA1233AA	10	512
ESC_UA1232AA	10	512
ESC_UA1231AA	10	512
ESC_UA1230AA	10	512
ESC_UA1229AA	10	512
ESC_UA1228AA	10	512
ESC_UA1227AA	10	512
ESC_UA1226AA	10	512
ESC_UA1225AA	10	512
ESC_UA1224AA	10	512
ESC_UA1223AA	10	512
ESC_UA1222AA	10	512
ESC_UA1221AA	10	512
ESC_UA1220AA	10	512
ESC_UA1219AA	10	512
ESC_UA1218AA	10	512
ESC_UA1217AA	10	512
ESC_UA1216AA	10	512
ESC_UA1215AA	10	512
ESC_UA1214AA	10	512
ESC_UA1213AA	10	512
ESC_UA1212AA	10	512
ESC_UA1211AA	10	512
ESC_UA1207AA	11881	512
ESC_UA1206AA	10	512
ESC_UA1205AA	10	512
ESC_UA1202AA	11880	512
ESC_UA1201AA	10	512
ESC_UA1200AA	10	512
ESC_UA1199AA	10	512
ESC_UA1198AA	10	512
ESC_UA1197AA	10	512
ESC_UA1196AA	10	512
ESC_UA1195AA	10	512

ESC_UA1194AA	10	512
ESC_UA1193AA	10	512
ESC_UA1190AA	10	512
ESC_UA1189AA	10	512
ESC_UA1188AA	10	512
ESC_UA1187AA	10	512
ESC_UA1186AA	10	512
ESC_UA1185AA	10	512
ESC_UA1184AA	10	512
ESC_UA1183AA	10	512
ESC_UA1182AA	10	512
ESC_UA1181AA	10	512
ESC_UA1180AA	10	512
ESC_UA1179AA	10	512
ESC_UA1178AA	10	512
ESC_UA1177AA	10	512
ESC_UA1176AA	10	512
ESC_UA1175AA	10	512
ESC_UA1174AA	10	512
ESC_UA1173AA	10	512
ESC_UA1172AA	10	512
ESC_UA1171AA	10	512
ESC_UA1170AA	10	512
ESC_UA1169AA	10	512
ESC_UA1168AA	10	512
ESC_UA1167AA	10	512
ESC_UA1166AA	10	512
ESC_UA1165AA	10	512
ESC_UA1164AA	10	512
ESC_UA1163AA	10	512
ESC_UA1162AA	10	512
ESC_UA1161AA	10	512
ESC_UA1160AA	10	512
ESC_UA1159AA	10	512
ESC_UA1158AA	10	512
ESC_UA1157AA	10	512
ESC_UA1156AA	10	512
ESC_UA1155AA	10	512
ESC_UA1153AA	10	512
ESC_UA1152AA	10	512
ESC_UA1151AA	10	512
ESC_UA1150AA	10	512
ESC_UA1149AA	10	512
ESC_UA1148AA	10	512
ESC_UA1147AA	10	512
ESC_UA1146AA	10	512
ESC_UA1145AA	10	512
ESC_UA1144AA	10	512
ESC_UA1143AA	10	512
ESC_UA1142AA	10	512

ESC_UA1141AA	10	512
ESC_UA1140AA	10	512
ESC_UA1138AA	10	512
ESC_UA1137AA	10	512
ESC_UA1136AA	10	512
ESC_UA1135AA	10	512
ESC_UA1134AA	10	512
ESC_UA1133AA	10	512
ESC_UA1132AA	10	512
ESC_UA1131AA	10	512
ESC_UA1130AA	10	512
ESC_UA1129AA	10	512
ESC_UA1128AA	10	512
ESC_UA1127AA	10	512
ESC_UA1125AA	10	512
ESC_UA1124AA	10	512
ESC_UA1123AA	10	512
ESC_UA1122AA	10	512
ESC_UA1121AA	10	512
ESC_UA1120AA	10	512
ESC_UA1119AA	10	512
ESC_UA1118AA	10	512
ESC_UA1117AA	10	512
ESC_UA1116AA	10	512
ESC_UA1115AA	10	512
ESC_UA1114AA	10	512
ESC_UA1113AA	10	512
ESC_UA1112AA	10	512
ESC_UA1111AA	10	512
ESC_UA1110AA	10	512
ESC_UA1109AA	10	512
ESC_UA1108AA	10	512
ESC_UA1107AA	10	512
ESC_UA1106AA	10	512
ESC_UA1105AA	10	512
ESC_UA1101AA	10	512
ESC_UA1100AA	10	512
ESC_UA1099AA	10	512
ESC_UA1098AA	10	512
ESC_UA1097AA	10	512
ESC_UA1096AA	10	512
ESC_UA1095AA	10	512
ESC_UA1094AA	10	512
ESC_UA1093AA	10	512
ESC_UA1092AA	10	512
ESC_UA1091AA	10	512
ESC_UA1090AA	10	512
ESC_UA1088AA	10	512
ESC_UA1087AA	10	512
ESC_UA1086AA	10	512

ESC_UA1085AA	10	512
ESC_UA1084AA	10	512
ESC_UA1083AA	10	512
ESC_UA1082AA	10	512
ESC_UA1081AA	10	512
ESC_UA1080AA	10	512
ESC_UA1079AA	10	512
ESC_UA1078AA	10	512
ESC_UA1077AA	10	512
ESC_UA1076AA	10	512
ESC_UA1075AA	10	512
ESC_UA1074AA	10	512
ESC_UA1073AA	10	512
ESC_UA1072AA	10	512
ESC_UA1071AA	10	512
ESC_UA1070AA	10	512
ESC_UA1069AA	10	512
ESC_UA1067AA	10	512
ESC_UA1066AA	10	512
ESC_UA1065AA	10	512
ESC_UA1064AA	10	512
ESC_UA1063AA	10	512
ESC_UA1062AA	10	512
ESC_UA1061AA	10	512
ESC_UA1060AA	10	512
ESC_UA1059AA	10	512
ESC_UA1058AA	10	512
ESC_UA1056AA	10	512
ESC_UA1055AA	10	512
ESC_UA1054AA	10	512
ESC_UA1053AA	10	512
ESC_UA1052AA	10	512
ESC_UA1051AA	10	512
ESC_UA1049AA	10	512
ESC_UA1048AA	10	512
ESC_UA1047AA	10	512
ESC_UA1046AA	10	512
ESC_UA1045AA	10	512
ESC_UA1044AA	10	512
ESC_UA1043AA	10	512
ESC_UA1042AA	10	512
ESC_UA1041AA	10	512
ESC_UA1040AA	10	512
ESC_UA1039AA	10	512
ESC_UA1038AA	10	512
ESC_UA1037AA	10	512
ESC_UA1036AA	10	512
ESC_UA1035AA	10	512
ESC_UA1034AA	10	512
ESC_UA1033AA	10	512

ESC_UA1032AA	10	512
ESC_UA1031AA	10	512
ESC_UA1030AA	10	512
ESC_UA1029AA	10	512
ESC_UA1028AA	10	512
ESC_UA1027AA	10	512
ESC_UA1025AA	10	512
ESC_UA1024AA	10	512
ESC_UA1023AA	10	512
ESC_UA1022AA	10	512
ESC_UA1021AA	10	512
ESC_UA1020AA	10	512
ESC_UA1019AA	10	512
ESC_UA1018AA	10	512
ESC_UA1017AA	10	512
ESC_UA1016AA	10	512
ESC_UA1015AA	10	512
ESC_UA1014AA	10	512
ESC_UA1013AA	10	512
ESC_UA1012AA	10	512
ESC_UA1011AA	10	512
ESC_UA1010AA	10	512
ESC_UA1009AA	10	512
ESC_UA1008AA	10	512
ESC_UA1007AA	10	512
ESC_UA1006AA	10	512
ESC_UA1005AA	10	512
ESC_UA1004AA	10	512
ESC_UA1003AA	10	512
ESC_UA1002AA	10	512
ESC_UA1001AA	10	512
ESC_UA1000AA	10	512
ESC_UA0999AA	10	512
ESC_UA0998AA	10	512
ESC_UA0997AA	10	512
ESC_UA0996AA	10	512
ESC_UA0995AA	10	512
ESC_UA0994AA	10	512
ESC_UA0993AA	10	512
ESC_UA0992AA	10	512
ESC_UA0991AA	10	512
ESC_UA0990AA	10	512
ESC_UA0989AA	10	512
ESC_UA0988AA	10	512
ESC_UA0987AA	10	512
ESC_UA0986AA	10	512
ESC_UA0985AA	10	512
ESC_UA0984AA	10	512
ESC_UA0983AA	10	512
ESC_UA0982AA	10	512

ESC_UA0981AA	10	512
ESC_UA0980AA	10	512
ESC_UA0979AA	10	512
ESC_UA0978AA	10	512
ESC_UA0977AA	10	512
ESC_UA0976AA	10	512
ESC_UA0975AA	10	512
ESC_UA0974AA	10	512
ESC_UA0973AA	10	512
ESC_UA0972AA	10	512
ESC_UA0971AA	10	512
ESC_UA0970AA	10	512
ESC_UA0969AA	10	512
ESC_UA0968AA	10	512
ESC_UA0967AA	10	512
ESC_UA0966AA	10	512
ESC_UA0965AA	10	512
ESC_UA0964AA	10	512
ESC_UA0963AA	10	512
ESC_UA0962AA	10	512
ESC_UA0961AA	10	512
ESC_UA0960AA	10	512
ESC_UA0959AA	10	512
ESC_UA0958AA	10	512
ESC_UA0957AA	10	512
ESC_UA0956AA	10	512
ESC_UA0955AA	10	512
ESC_UA0954AA	10	512
ESC_UA0953AA	10	512
ESC_UA0952AA	10	512
ESC_UA0951AA	10	512
ESC_UA0950AA	10	512
ESC_UA0949AA	10	512
ESC_UA0948AA	10	512
ESC_UA0947AA	10	512
ESC_UA0946AA	10	512
ESC_UA0944AA	10	512
ESC_UA0943AA	10	512
ESC_UA0942AA	10	512
ESC_UA0941AA	10	512
ESC_UA0940AA	10	512
ESC_UA0939AA	10	512
ESC_UA0938AA	10	512
ESC_UA0936AA	10	512
ESC_UA0935AA	10	512
ESC_UA0934AA	10	512
ESC_UA0933AA	11884	512
ESC_UA0932AA	10	512
ESC_UA0931AA	10	512
ESC_UA0930AA	10	512

ESC_UA0929AA	10	512
ESC_UA0928AA	10	512
ESC_UA0927AA	10	512
ESC_UA0926AA	10	512
ESC_UA0925AA	11883	512
ESC_UA0924AA	10	512
ESC_UA0922AA	10	512
ESC_UA0921AA	10	512
ESC_UA0920AA	10	512
ESC_UA0919AA	10	512
ESC_UA0918AA	10	512
ESC_UA0916AA	10	512
ESC_UA0915AA	10	512
ESC_UA0914AA	10	512
ESC_UA0912AA	10	512
ESC_UA0911AA	10	512
ESC_UA0910AA	10	512
ESC_UA0909AA	10	512
ESC_UA0908AA	10	512
ESC_UA0907AA	11882	820
ESC_UA0906AA	10	512
ESC_UA0905AA	10	512
ESC_UA0904AA	10	512
ESC_UA0903AA	10	512
ESC_UA0902AA	10	512
ESC_UA0901AA	10	512
ESC_UA0900AA	10	512
ESC_UA0899AA	10	512
ESC_UA0898AA	10	512
ESC_UA0897AA	10	512
ESC_UA0896AA	10	512
ESC_UA0895AA	10	512
ESC_UA0894AA	10	512
ESC_UA0893AA	10	512
ESC_UA0892AA	10	512
ESC_UA0891AA	10	512
ESC_UA0890AA	10	512
ESC_UA0889AA	10	512
ESC_UA0888AA	10	512
ESC_UA0886AA	10	512
ESC_UA0885AA	10	512
ESC_UA0884AA	10	512
ESC_UA0883AA	10	512
ESC_UA0882AA	10	512
ESC_UA0881AA	10	512
ESC_UA0880AA	10	512
ESC_UA0879AA	10	512
ESC_UA0878AA	10	512
ESC_UA0877AA	10	512
ESC_UA0876AA	10	512

ESC_UA0875AA	10	512
ESC_UA0874AA	10	512
ESC_UA0873AA	10	512
ESC_UA0872AA	10	512
ESC_UA0871AA	10	512
ESC_UA0870AA	10	512
ESC_UA0869AA	10	512
ESC_UA0868AA	10	512
ESC_UA0867AA	10	512
ESC_UA0866AA	10	512
ESC_UA0865AA	10	512
ESC_UA0864AA	10	512
ESC_UA0863AA	10	512
ESC_UA0862AA	10	512
ESC_UA0861AA	10	512
ESC_UA0860AA	10	512
ESC_UA0859AA	10	512
ESC_UA0858AA	10	512
ESC_UA0857AA	10	512
ESC_UA0856AA	10	512
ESC_UA0855AA	10	512
ESC_UA0853AA	10	512
ESC_UA0852AA	10	512
ESC_UA0851AA	10	512
ESC_UA0850AA	10	512
ESC_UA0849AA	10	512
ESC_UA0848AA	10	512
ESC_UA0847AA	10	512
ESC_UA0846AA	10	512
ESC_UA0844AA	10	512
ESC_UA0843AA	10	512
ESC_UA0842AA	10	512
ESC_UA0841AA	10	512
ESC_UA0840AA	10	512
ESC_UA0839AA	10	512
ESC_UA0838AA	10	512
ESC_UA0837AA	10	512
ESC_UA0836AA	10	512
ESC_UA0835AA	10	512
ESC_UA0834AA	10	512
ESC_UA0833AA	10	512
ESC_UA0832AA	10	512
ESC_UA0831AA	10	512
ESC_UA0830AA	10	512
ESC_UA0828AA	10	512
ESC_UA0827AA	10	512
ESC_UA0826AA	10	512
ESC_UA0825AA	10	512
ESC_UA0824AA	10	512
ESC_UA0822AA	10	512

ESC_UA0821AA	10	512
ESC_UA0820AA	10	512
ESC_UA0819AA	10	512
ESC_UA0817AA	10	512
ESC_UA0816AA	10	512
ESC_UA0815AA	10	512
ESC_UA0814AA	10	512
ESC_UA0813AA	10	512
ESC_UA0812AA	10	512
ESC_UA0811AA	10	512
ESC_UA0810AA	10	512
ESC_UA0809AA	10	512
ESC_UA0808AA	10	512
ESC_UA0807AA	10	512
ESC_UA0806AA	10	512
ESC_UA0805AA	10	512
ESC_UA0804AA	10	512
ESC_UA0803AA	10	512
ESC_UA0802AA	10	512
ESC_UA0801AA	10	512
ESC_UA0800AA	10	512
ESC_UA0799AA	10	512
ESC_UA0798AA	10	512
ESC_UA0797AA	10	512
ESC_UA0796AA	10	512
ESC_UA0795AA	10	512
ESC_UA0794AA	10	512
ESC_UA0792AA	10	512
ESC_UA0791AA	10	512
ESC_UA0790AA	10	512
ESC_UA0789AA	10	512
ESC_UA0788AA	10	512
ESC_UA0787AA	10	512
ESC_UA0786AA	10	512
ESC_UA0785AA	10	512
ESC_UA0780AA	10	512
ESC_UA0774AA	10	512
ESC_UA0773AA	10	512
ESC_UA0772AA	10	512
ESC_UA0771AA	10	512
ESC_UA0770AA	10	512
ESC_UA0769AA	10	512
ESC_UA0768AA	10	512
ESC_UA0767AA	10	512
ESC_UA0766AA	10	512
ESC_UA0765AA	10	512
ESC_UA0764AA	10	512
ESC_UA0763AA	10	512
ESC_UA0762AA	10	512
ESC_UA0761AA	10	512

ESC_UA0760AA	10	512
ESC_UA0759AA	10	512
ESC_UA0758AA	10	512
ESC_UA0757AA	10	512
ESC_UA0756AA	10	512
ESC_UA0755AA	10	512
ESC_UA0753AA	10	512
ESC_UA0752AA	10	512
ESC_UA0751AA	10	512
ESC_UA0750AA	10	512
ESC_UA0749AA	10	512
ESC_UA0748AA	10	512
ESC_UA0747AA	10	512
ESC_UA0746AA	10	512
ESC_UA0745AA	10	512
ESC_UA0744AA	10	512
ESC_UA0743AA	10	512
ESC_UA0742AA	10	512
ESC_UA0741AA	10	512
ESC_UA0740AA	10	512
ESC_UA0739AA	10	512
ESC_UA0737AA	10	512
ESC_UA0736AA	10	512
ESC_UA0734AA	10	512
ESC_UA0733AA	10	512
ESC_UA0732AA	10	512
ESC_UA0731AA	10	512
ESC_UA0730AA	10	512
ESC_UA0728AA	10	512
ESC_UA0720AA	10	512
ESC_UA0719AA	10	512
ESC_UA0718AA	10	512
ESC_UA0716AA	10	512
ESC_UA0715AA	10	512
ESC_UA0714AA	10	512
ESC_UA0712AA	10	512
ESC_UA0711AA	10	512
ESC_UA0710AA	10	512
ESC_UA0709AA	10	512
ESC_UA0708AA	10	512
ESC_UA0707AA	10	512
ESC_UA0706AA	10	512
ESC_UA0705AA	10	512
ESC_UA0704AA	10	512
ESC_UA0703AA	10	512
ESC_UA0702AA	10	512
ESC_UA0701AA	10	512
ESC_UA0700AA	10	512
ESC_UA0699AA	10	512
ESC_UA0698AA	10	512

ESC_UA0697AA	10	512
ESC_UA0696AA	10	512
ESC_UA0695AA	10	512
ESC_UA0694AA	10	512
ESC_UA0693AA	10	512
ESC_UA0692AA	10	512
ESC_UA0691AA	10	512
ESC_UA0690AA	10	512
ESC_UA0689AA	10	512
ESC_UA0688AA	10	512
ESC_UA0687AA	10	512
ESC_UA0686AA	10	512
ESC_UA0685AA	10	512
ESC_UA0684AA	10	512
ESC_UA0683AA	10	512
ESC_UA0682AA	10	512
ESC_UA0681AA	10	512
ESC_UA0680AA	10	512
ESC_UA0679AA	10	512
ESC_UA0678AA	10	512
ESC_UA0677AA	10	512
ESC_UA0676AA	10	512
ESC_UA0675AA	10	512
ESC_UA0674AA	11886	820
ESC_UA0671AA	10	512
ESC_UA0670AA	10	512
ESC_UA0669AA	10	512
ESC_UA0668AA	10	512
ESC_UA0667AA	10	512
ESC_UA0666AA	10	512
ESC_UA0665AA	10	512
ESC_UA0664AA	10	512
ESC_UA0662AA	10	512
ESC_UA0661AA	10	512
ESC_UA0660AA	10	512
ESC_UA0659AA	10	512
ESC_UA0658AA	10	512
ESC_UA0657AA	10	512
ESC_UA0656AA	10	512
ESC_UA0655AA	10	512
ESC_UA0654AA	10	512
ESC_UA0652AA	10	512
ESC_UA0651AA	10	512
ESC_UA0649AA	10	512
ESC_UA0648AA	10	512
ESC_UA0647AA	10	512
ESC_UA0646AA	10	512
ESC_UA0645AA	10	512
ESC_UA0644AA	10	512
ESC_UA0642AA	10	512

ESC_UA0641AA	10	512
ESC_UA0640AA	10	512
ESC_UA0639AA	10	512
ESC_UA0638AA	10	512
ESC_UA0637AA	10	512
ESC_UA0636AA	10	512
ESC_UA0635AA	10	512
ESC_UA0634AA	10	512
ESC_UA0633AA	10	512
ESC_UA0632AA	10	512
ESC_UA0631AA	10	512
ESC_UA0630AA	10	512
ESC_UA0629AA	10	512
ESC_UA0628AA	10	512
ESC_UA0627AA	10	512
ESC_UA0626AA	10	512
ESC_UA0625AA	10	512
ESC_UA0624AA	10	512
ESC_UA0623AA	10	512
ESC_UA0622AA	10	512
ESC_UA0621AA	10	512
ESC_UA0620AA	10	512
ESC_UA0619AA	10	512
ESC_UA0617AA	10	512
ESC_UA0616AA	10	512
ESC_UA0615AA	10	512
ESC_UA0614AA	10	512
ESC_UA0613AA	10	512
ESC_UA0612AA	10	512
ESC_UA0611AA	10	512
ESC_UA0610AA	10	512
ESC_UA0609AA	10	512
ESC_UA0608AA	10	512
ESC_UA0607AA	10	512
ESC_UA0605AA	10	512
ESC_UA0603AA	10	512
ESC_UA0602AA	10	512
ESC_UA0601AA	10	512
ESC_UA0600AA	10	512
ESC_UA0599AA	10	512
ESC_UA0598AA	10	512
ESC_UA0597AA	10	512
ESC_UA0596AA	10	512
ESC_UA0595AA	10	512
ESC_UA0594AA	10	512
ESC_UA0593AA	10	512
ESC_UA0592AA	10	512
ESC_UA0591AA	10	512
ESC_UA0590AA	10	512
ESC_UA0589AA	10	512

ESC_UA0588AA	10	512
ESC_UA0587AA	10	512
ESC_UA0586AA	10	512
ESC_UA0585AA	10	512
ESC_UA0584AA	10	512
ESC_UA0583AA	10	512
ESC_UA0582AA	10	512
ESC_UA0581AA	10	512
ESC_UA0580AA	10	512
ESC_UA0579AA	10	512
ESC_UA0578AA	10	512
ESC_UA0577AA	10	512
ESC_UA0576AA	10	512
ESC_UA0575AA	10	512
ESC_UA0574AA	10	512
ESC_UA0573AA	10	512
ESC_UA0572AA	10	512
ESC_UA0571AA	10	512
ESC_UA0570AA	10	512
ESC_UA0569AA	10	512
ESC_UA0568AA	10	512
ESC_UA0567AA	10	512
ESC_UA0566AA	10	512
ESC_UA0565AA	10	512
ESC_UA0564AA	10	512
ESC_UA0563AA	10	512
ESC_UA0562AA	10	512
ESC_UA0561AA	10	512
ESC_UA0560AA	10	512
ESC_UA0559AA	10	512
ESC_UA0558AA	10	512
ESC_UA0557AA	10	512
ESC_UA0556AA	10	512
ESC_UA0555AA	10	512
ESC_UA0554AA	10	512
ESC_UA0553AA	10	512
ESC_UA0552AA	10	512
ESC_UA0551AA	10	512
ESC_UA0550AA	10	512
ESC_UA0549AA	10	512
ESC_UA0546AA	10	512
ESC_UA0545AA	10	512
ESC_UA0544AA	10	512
ESC_UA0543AA	10	512
ESC_UA0542AA	10	512
ESC_UA0541AA	10	512
ESC_UA0540AA	10	512
ESC_UA0539AA	10	512
ESC_UA0538AA	10	512
ESC_UA0537AA	10	512

ESC_UA0535AA	10	512
ESC_UA0534AA	10	512
ESC_UA0533AA	10	512
ESC_UA0532AA	10	512
ESC_UA0531AA	10	512
ESC_UA0530AA	10	512
ESC_UA0529AA	10	512
ESC_UA0528AA	10	512
ESC_UA0527AA	10	512
ESC_UA0526AA	10	512
ESC_UA0525AA	10	512
ESC_UA0524AA	10	512
ESC_UA0523AA	10	512
ESC_UA0521AA	10	512
ESC_UA0520AA	10	512
ESC_UA0519AA	10	512
ESC_UA0518AA	10	512
ESC_UA0516AA	10	512
ESC_UA0515AA	10	512
ESC_UA0514AA	10	512
ESC_UA0513AA	10	512
ESC_UA0512AA	10	512
ESC_UA0511AA	10	512
ESC_UA0510AA	10	512
ESC_UA0509AA	10	512
ESC_UA0508AA	10	512
ESC_UA0507AA	10	512
ESC_UA0506AA	10	512
ESC_UA0505AA	10	512
ESC_UA0504AA	10	512
ESC_UA0503AA	10	512
ESC_UA0502AA	10	512
ESC_UA0501AA	10	512
ESC_UA0500AA	10	512
ESC_UA0499AA	10	512
ESC_UA0498AA	10	512
ESC_UA0497AA	10	512
ESC_UA0496AA	10	512
ESC_UA0495AA	10	512
ESC_UA0494AA	10	512
ESC_UA0493AA	10	512
ESC_UA0492AA	10	512
ESC_UA0490AA	10	512
ESC_UA0489AA	10	512
ESC_UA0488AA	10	512
ESC_UA0487AA	10	512
ESC_UA0486AA	11888	512
ESC_UA0485AA	10	512
ESC_UA0484AA	10	512
ESC_UA0483AA	10	512

ESC_UA0482AA	10	512
ESC_UA0481AA	10	512
ESC_UA0480AA	10	512
ESC_UA0479AA	10	512
ESC_UA0478AA	10	512
ESC_UA0477AA	10	512
ESC_UA0476AA	10	512
ESC_UA0475AA	10	512
ESC_UA0474AA	10	512
ESC_UA0473AA	10	512
ESC_UA0472AA	10	512
ESC_UA0471AA	10	512
ESC_UA0470AA	10	512
ESC_UA0469AA	10	512
ESC_UA0468AA	10	512
ESC_UA0467AA	10	512
ESC_UA0466AA	10	512
ESC_UA0465AA	10	512
ESC_UA0464AA	10	512
ESC_UA0463AA	10	512
ESC_UA0462AA	10	512
ESC_UA0461AA	10	512
ESC_UA0460AA	10	512
ESC_UA0459AA	10	512
ESC_UA0458AA	10	512
ESC_UA0457AA	10	512
ESC_UA0456AA	10	512
ESC_UA0455AA	10	512
ESC_UA0454AA	10	512
ESC_UA0453AA	10	512
ESC_UA0452AA	10	512
ESC_UA0451AA	10	512
ESC_UA0450AA	10	512
ESC_UA0449AA	10	512
ESC_UA0448AA	10	512
ESC_UA0447AA	10	512
ESC_UA0446AA	10	512
ESC_UA0445AA	10	512
ESC_UA0444AA	10	512
ESC_UA0443AA	10	512
ESC_UA0442AA	10	512
ESC_UA0441AA	10	512
ESC_UA0440AA	10	512
ESC_UA0439AA	10	512
ESC_UA0438AA	10	512
ESC_UA0437AA	10	512
ESC_UA0436AA	10	512
ESC_UA0435AA	10	512
ESC_UA0434AA	10	512
ESC_UA0433AA	10	512

ESC_UA0432AA	10	512
ESC_UA0431AA	10	512
ESC_UA0430AA	10	512
ESC_UA0429AA	10	512
ESC_UA0428AA	10	512
ESC_UA0427AA	10	512
ESC_UA0426AA	10	512
ESC_UA0425AA	10	512
ESC_UA0424AA	10	512
ESC_UA0423AA	10	512
ESC_UA0422AA	10	512
ESC_UA0421AA	10	512
ESC_UA0420AA	10	512
ESC_UA0419AA	10	512
ESC_UA0418AA	10	512
ESC_UA0416AA	10	512
ESC_UA0415AA	10	512
ESC_UA0414AA	10	512
ESC_UA0413AA	10	512
ESC_UA0412AA	10	512
ESC_UA0411AA	10	512
ESC_UA0410AA	10	512
ESC_UA0409AA	10	512
ESC_UA0408AA	10	512
ESC_UA0407AA	10	512
ESC_UA0406AA	10	512
ESC_UA0405AA	10	512
ESC_UA0404AA	10	512
ESC_UA0403AA	10	512
ESC_UA0402AA	10	512
ESC_UA0401AA	10	512
ESC_UA0400AA	10	512
ESC_UA0399AA	10	512
ESC_UA0398AA	10	512
ESC_UA0397AA	10	512
ESC_UA0396AA	10	512
ESC_UA0395AA	10	512
ESC_UA0394AA	10	512
ESC_UA0393AA	10	512
ESC_UA0392AA	10	512
ESC_UA0391AA	10	512
ESC_UA0390AA	10	512
ESC_UA0389AA	10	512
ESC_UA0388AA	10	512
ESC_UA0387AA	10	512
ESC_UA0386AA	10	512
ESC_UA0385AA	10	512
ESC_UA0384AA	10	512
ESC_UA0383AA	10	512
ESC_UA0382AA	10	512

ESC_UA0381AA	10	512
ESC_UA0379AA	10	512
ESC_UA0378AA	10	512
ESC_UA0377AA	10	512
ESC_UA0376AA	10	512
ESC_UA0375AA	10	512
ESC_UA0374AA	10	512
ESC_UA0373AA	10	512
ESC_UA0372AA	10	512
ESC_UA0371AA	10	512
ESC_UA0370AA	10	512
ESC_UA0369AA	10	512
ESC_UA0368AA	10	512
ESC_UA0367AA	10	512
ESC_UA0366AA	10	512
ESC_UA0365AA	10	512
ESC_UA0364AA	10	512
ESC_UA0363AA	10	512
ESC_UA0362AA	11889	512
ESC_UA0360AA	10	512
ESC_UA0359AA	10	512
ESC_UA0357AA	10	512
ESC_UA0355AA	10	512
ESC_UA0354AA	10	512
ESC_UA0352AA	10	512
ESC_UA0351AA	10	512
ESC_UA0350AA	10	512
ESC_UA0349AA	10	512
ESC_UA0348AA	10	512
ESC_UA0347AA	10	512
ESC_UA0346AA	10	512
ESC_UA0345AA	10	512
ESC_UA0338AA	10	512
ESC_UA0336AA	10	512
ESC_UA0331AA	10	512
ESC_UA0327AA	10	512
ESC_UA0326AA	10	512
ESC_UA0325AA	10	512
ESC_UA0323AA	10	512
ESC_UA0322AA	10	512
ESC_UA0321AA	10	512
ESC_UA0320AA	10	512
ESC_UA0319AA	10	512
ESC_UA0318AA	10	512
ESC_UA0317AA	10	512
ESC_UA0316AA	10	512
ESC_UA0315AA	10	512
ESC_UA0314AA	10	512
ESC_UA0313AA	10	512
ESC_UA0312AA	10	512

ESC_UA0311AA	10	512
ESC_UA0310AA	10	512
ESC_UA0309AA	10	512
ESC_UA0308AA	10	512
ESC_UA0306AA	10	512
ESC_UA0305AA	10	512
ESC_UA0304AA	10	512
ESC_UA0303AA	10	512
ESC_UA0302AA	10	512
ESC_UA0301AA	10	512
ESC_UA0300AA	10	512
ESC_UA0299AA	10	512
ESC_UA0298AA	10	512
ESC_UA0297AA	10	512
ESC_UA0296AA	10	512
ESC_UA0295AA	10	512
ESC_UA0294AA	10	512
ESC_UA0293AA	10	512
ESC_UA0292AA	10	512
ESC_UA0291AA	10	512
ESC_UA0290AA	10	512
ESC_UA0289AA	10	512
ESC_UA0288AA	10	512
ESC_UA0287AA	10	512
ESC_UA0286AA	10	512
ESC_UA0285AA	10	512
ESC_UA0284AA	10	512
ESC_UA0283AA	10	512
ESC_UA0282AA	14	761
ESC_UA0281AA	245	261
ESC_UA0280AA	10	512
ESC_UA0279AA	10	512
ESC_UA0278AA	10	512
ESC_UA0277AA	10	512
ESC_UA0275AA	10	512
ESC_UA0274AA	10	512
ESC_UA0273AA	10	512
ESC_UA0270AA	10	512
ESC_UA0268AA	10	512
ESC_UA0267AA	10	512
ESC_UA0266AA	10	512
ESC_UA0265AA	10	512
ESC_UA0264AA	10	512
ESC_UA0263AA	10	512
ESC_UA0262AA	10	512
ESC_UA0261AA	10	512
ESC_UA0260AA	10	512
ESC_UA0258AA	10	512
ESC_UA0257AA	10	512
ESC_UA0256AA	10	512

ESC_UA0255AA	10	512
ESC_UA0254AA	10	512
ESC_UA0253AA	10	512
ESC_UA0252AA	10	512
ESC_UA0249AA	46	447
ESC_UA0248AA	1049	175
ESC_UA0247AA	405	923
ESC_UA0246AA	405	923
ESC_UA0245AA	11	936
ESC_UA0244AA	11	936
ESC_UA0243AA	11	936
ESC_UA0242AA	11	936
ESC_UA0241AA	11	936
ESC_UA0240AA	11	936
ESC_UA0239AA	11	936
ESC_UA0238AA	11	936
ESC_UA0237AA	11	936
ESC_UA0236AA	11	936
ESC_UA0235AA	11	936
ESC_UA0234AA	11	936
ESC_UA0233AA	11	936
ESC_UA0232AA	11	936
ESC_UA0231AA	11	936
ESC_UA0230AA	278	924
ESC_UA0229AA	13	257
ESC_UA0228AA	88	160
ESC_UA0227AA	2705	504
ESC_UA0226AA	101	696
ESC_UA0224AA	88	160
ESC_UA0223AA	23	156
ESC_UA0222AA	23	156
ESC_UA0221AA	4085	791
ESC_UA0220AA	349	670
ESC_UA0219AA	2522	198
ESC_UA0218AA	925	387
ESC_UA0217AA	106	387
ESC_UA0216AA	2522	198
ESC_UA0215AA	1114	504
ESC_UA0214AA	349	670
ESC_UA0213AA	540	286
ESC_UA0212AA	10610	512
ESC_UA0208AA	977	71
ESC_UA0207AA	88	160
ESC_UA0206AA	362	234
ESC_UA0205AA	34	504
ESC_UA0204AA	617	511
ESC_UA0203AA	10	512
ESC_UA0202AA	361	512
ESC_UA0201AA	10	512
ESC_UA0200AA	1163	579

ESC_UA0199AA	1163	579
ESC_UA0198AA	69	387
ESC_UA0197AA	744	512
ESC_UA0196AA	410	156
ESC_UA0195AA	117	592
ESC_UA0194AA	1114	504
ESC_UA0193AA	410	156
ESC_UA0192AA	1303	791
ESC_UA0191AA	8817	512
ESC_UA0190AA	117	592
ESC_UA0189AA	410	156
ESC_UA0188AA	744	512
ESC_UA0187AA	3889	291
ESC_UA0186AA	10	512
ESC_UA0185AA	3	198
ESC_UA0184AA	3	198
ESC_UA0183AA	3	198
ESC_UA0182AA	3	198
ESC_UA0181AA	19	774
ESC_UA0180AA	11792	766
ESC_UA0179AA	11792	766
ESC_UA0178AA	3	198
ESC_UA0177AA	3	198
ESC_UA0176AA	3	198
ESC_UA0175AA	3	198
ESC_UA0174AA	3	198
ESC_UA0173AA	3	198
ESC_UA0172AA	3	198
ESC_UA0171AA	3	198
ESC_UA0170AA	3	198
ESC_UA0169AA	3	198
ESC_UA0168AA	3	198
ESC_UA0167AA	3	198
ESC_UA0166AA	3	198
ESC_UA0165AA	3	198
ESC_UA0164AA	3	198
ESC_UA0163AA	3	198
ESC_UA0162AA	3	198
ESC_UA0161AA	3	198
ESC_UA0160AA	3	198
ESC_UA0159AA	3	198
ESC_UA0158AA	3	198
ESC_UA0157AA	3	198
ESC_UA0156AA	3	198
ESC_UA0155AA	3	198
ESC_UA0154AA	3	198
ESC_UA0153AA	3	198
ESC_UA0152AA	3	198
ESC_UA0151AA	3	198
ESC_UA0150AA	3	198

ESC_UA0149AA	3	198
ESC_UA0148AA	3	198
ESC_UA0147AA	3	198
ESC_UA0146AA	3	198
ESC_UA0145AA	3	198
ESC_UA0144AA	3	198
ESC_UA0143AA	3	198
ESC_UA0142AA	3	198
ESC_UA0141AA	3	198
ESC_UA0140AA	3	198
ESC_UA0139AA	3	198
ESC_UA0138AA	3	198
ESC_UA0137AA	3	198
ESC_UA0136AA	3	198
ESC_UA0135AA	3	198
ESC_UA0134AA	3	198
ESC_UA0133AA	3	198
ESC_UA0132AA	3	198
ESC_UA0131AA	3	198
ESC_UA0130AA	3	198
ESC_UA0129AA	3	198
ESC_UA0128AA	3	198
ESC_UA0127AA	3	198
ESC_UA0126AA	3	198
ESC_UA0125AA	28	537
ESC_UA0124AA	362	234
ESC_UA0123AA	29	156
ESC_UA0122AA	28	537
ESC_UA0121AA	29	156
ESC_UA0120AA	28	537
ESC_UA0119AA	2362	772
ESC_UA0118AA	7444	937
ESC_UA0117AA	3	198
ESC_UA0116AA	19	774
ESC_UA0115AA	3	198
ESC_UA0114AA	3	198
ESC_UA0113AA	28	537
ESC_UA0112AA	3	198
ESC_UA0111AA	20	198
ESC_UA0110AA	6355	625
ESC_UA0109AA	162	924
ESC_UA0108AA	2622	537
ESC_UA0107AA	46	447
ESC_UA0106AA	131	653
ESC_UA0105AA	354	15
ESC_UA0104AA	131	653
ESC_UA0103AA	295	160
ESC_UA0102AA	354	15
ESC_UA0101AA	141	644
ESC_UA0100AA	6355	625

ESC_UA0099AA	11810	740
ESC_UA0098AA	3285	257
ESC_UA0097AA	23	156
ESC_UA0096AA	2659	679
ESC_UA0095AA	1434	512
ESC_UA0094AA	3014	410
ESC_UA0093AA	40	198
ESC_UA0092AA	675	234
ESC_UA0091AA	34	504
ESC_UA0090AA	2509	257
ESC_UA0089AA	206	286
ESC_UA0088AA	635	141
ESC_UA0087AA	773	71
ESC_UA0086AA	1316	471
ESC_UA0085AA	3877	512
ESC_UA0084AA	5295	355
ESC_UA0083AA	5295	355
ESC_UA0082AA	8677	483
ESC_UA0081AA	9655	198
ESC_UA0080AA	3666	679
ESC_UA0079AA	3666	679
ESC_UA0078AA	135	653
ESC_UA0077AA	3666	679
ESC_UA0076AA	3666	679
ESC_UA0075AA	167	512
ESC_UA0074AA	424	234
ESC_UA0073AA	607	355
ESC_UA0072AA	8186	629
ESC_UA0071AA	6355	625
ESC_UA0070AA	2380	198
ESC_UA0069AA	162	924
ESC_UA0068AA	12	626
ESC_UA0067AA	4623	257
ESC_UA0066AA	6355	625
ESC_UA0065AA	83	740
ESC_UA0064AA	6355	625
ESC_UA0063AA	100	504
ESC_UA0062AA	100	504
ESC_UA0061AA	93	71
ESC_UA0060AA	11875	71
ESC_UA0059AA	100	504
ESC_UA0058AA	5786	513
ESC_UA0057AA	5786	513
ESC_UA0056AA	3057	672
ESC_UA0055AA	10	512
ESC_UA0054AA	100	504
ESC_UA0053AA	10	512
ESC_UA0052AA	29	156
ESC_UA0051AA	23	156
ESC_UA0050AA	46	447

ESC_UA0049AA	206	286
ESC_UA0048AA	216	512
ESC_UA0047AA	216	512
ESC_UA0046AA	398	445
ESC_UA0045AA	399	198
ESC_UA0044AA	409	504
ESC_UA0043AA	409	504
ESC_UA0042AA	450	71
ESC_UA0041AA	635	141
ESC_UA0040AA	635	141
ESC_UA0039AA	635	141
ESC_UA0038AA	635	141
ESC_UA0037AA	635	141
ESC_UA0036AA	635	141
ESC_UA0035AA	728	355
ESC_UA0034AA	101	696
ESC_UA0033AA	17	191
ESC_UA0031AA	10	512
ESC_UA0030AA	10	512
ESC_UA0029AA	10	512
ESC_UA0028AA	10	512
ESC_UA0027AA	152	257
ESC_UA0026AA	245	261
ESC_UA0025AA	301	307
ESC_UA0023AA	21	156
ESC_UA0022AA	278	924
ESC_UA0021AA	301	307
ESC_UA0020AA	33	198
ESC_UA0019AA	11	936
ESC_UA0018AA	73	737
ESC_UA0017AA	5292	738
ESC_UA0016AA	447	198
ESC_UA0015AA	7641	738
ESC_UA0014AA	10	512
ESC_UA0013AA	10	512
ESC_UA0012AA	10	512
ESC_UA0011AA	10	512
ESC_UA0010AA	10	512
ESC_UA0009AA	10	512
ESC_UA0008AA	10	512
ESC_UA0007AA	10	512
ESC_UA0006AA	10	512
ESC_UA0005AA	117	592
ESC_UA0004AA	131	653
ESC_UA0003AA	117	592
ESC_UA0002AA	1236	380
ESC_UA0001AA	131	653
ESC_UA0000AA	131	653
ESC_TA9999AA	11809	761
ESC_TA9998AA	95	738

ESC_TA9997AA	2179	924
ESC_TA9996AA	38	669
ESC_TA9995AA	48	76
ESC_TA9994AA	88	160
ESC_TA9993AA	457	595
ESC_TA9992AA	351	268
ESC_TA9991AA	131	653
ESC_TA9990AA	131	653
ESC_TA9989AA	155	175
ESC_TA9988AA	3052	669
ESC_TA9987AA	73	737
ESC_TA9986AA	1193	761
ESC_TA9985AA	6995	584
ESC_TA9984AA	1196	257
ESC_TA9983AA	70	670
ESC_TA9982AA	127	738
ESC_TA9980AA	11808	373
ESC_TA9979AA	101	696
ESC_TA9978AA	744	512
ESC_TA9977AA	355	764
ESC_TA9976AA	1946	726
ESC_TA9975AA	73	737
ESC_TA9974AA	131	653
ESC_TA9973AA	1061	406
ESC_TA9972AA	327	204
ESC_TA9971AA	212	198
ESC_TA9970AA	491	626
ESC_TA9969AA	1946	726
ESC_TA9968AA	6998	629
ESC_TA9963AA	491	626
ESC_TA9958AA	56	198
ESC_TA9956AA	718	257
ESC_TA9955AA	738	598
ESC_TA9950AA	223	198
ESC_TA9947AA	993	789
ESC_TA9946AA	33	198
ESC_TA9927AA	357	654
ESC_TA9926AA	8934	844
ESC_TA9919AA	31	387
ESC_TA9918AA	200	198
ESC_TA9917AA	10	512
ESC_TA9916AA	95	738
ESC_TA9915AA	58	198
ESC_TA9914AA	371	50
ESC_TA9913AA	226	512
ESC_TA9912AA	371	50
ESC_TA9911AA	371	50
ESC_TA9910AA	2003	669
ESC_TA9909AA	11807	670
ESC_TA9908AA	10	512

ESC_TA9907AA	10	512
ESC_TA9906AA	349	670
ESC_TA9905AA	155	175
ESC_TA9904AA	607	355
ESC_TA9903AA	38	669
ESC_TA9902AA	1193	761
ESC_TA9901AA	1286	474
ESC_TA9900AA	200	198
ESC_TA9899AA	607	355
ESC_TA9898AA	200	198
ESC_TA9897AA	539	257
ESC_TA9896AA	3580	257
ESC_TA9891AA	3570	156
ESC_TA9885AA	131	653
ESC_TA9883AA	2558	782
ESC_TA9882AA	83	740
ESC_TA9881AA	155	175
ESC_TA9880AA	1193	761
ESC_TA9879AA	2351	234
ESC_TA9878AA	38	669
ESC_TA9877AA	2973	234
ESC_TA9876AA	88	160
ESC_TA9875AA	12	626
ESC_TA9874AA	130	387
ESC_TA9873AA	191	198
ESC_TA9872AA	162	924
ESC_TA9871AA	11805	899
ESC_TA9867AA	152	257
ESC_TA9866AA	245	261
ESC_TA9865AA	152	257
ESC_TA9862AA	152	257
ESC_TA9860AA	152	257
ESC_TA9858AA	245	261
ESC_TA9857AA	152	257
ESC_TA9856AA	152	257
ESC_TA9855AA	152	257
ESC_TA9854AA	152	257
ESC_TA9849AA	152	257
ESC_TA9846AA	245	261
ESC_TA9845AA	245	261
ESC_TA9844AA	152	257
ESC_TA9833AA	630	261
ESC_TA9826AA	152	257
ESC_TA9825AA	245	261
ESC_TA9822AA	152	257
ESC_TA9819AA	152	257
ESC_TA9816AA	245	261
ESC_TA9811AA	152	257
ESC_TA9810AA	152	257
ESC_TA9807AA	245	261

ESC_TA9806AA	245	261
ESC_TA9803AA	152	257
ESC_TA9798AA	152	257
ESC_TA9797AA	152	257
ESC_TA9796AA	245	261
ESC_TA9793AA	245	261
ESC_TA9790AA	152	257
ESC_TA9778AA	40	198
ESC_TA9775AA	675	234
ESC_TA9774AA	1041	765
ESC_TA9767AA	11768	234
ESC_TA9766AA	6745	885
ESC_TA9765AA	95	738
ESC_TA9764AA	355	764
ESC_TA9763AA	164	175
ESC_TA9762AA	164	175
ESC_TA9761AA	11671	962
ESC_TA9760AA	11671	962
ESC_TA9759AA	11671	962
ESC_TA9758AA	1324	156
ESC_TA9757AA	404	761
ESC_TA9756AA	550	756
ESC_TA9755AA	73	737
ESC_TA9754AA	10	512
ESC_TA9753AA	48	76
ESC_TA9751AA	101	696
ESC_TA9750AA	165	504
ESC_TA9749AA	117	592
ESC_TA9748AA	56	198
ESC_TA9747AA	4054	198
ESC_TA9746AA	11856	198
ESC_TA9745AA	156	175
ESC_TA9744AA	2509	257
ESC_TA9743AA	23	156
ESC_TA9742AA	871	447
ESC_TA9741AA	154	257
ESC_TA9740AA	69	387
ESC_TA9739AA	88	160
ESC_TA9738AA	58	198
ESC_TA9737AA	215	512
ESC_TA9736AA	8577	445
ESC_TA9735AA	48	76
ESC_TA9734AA	10	512
ESC_TA9733AA	3321	935
ESC_TA9732AA	34	504
ESC_TA9731AA	111	175
ESC_TA9730AA	925	387
ESC_TA9729AA	227	504
ESC_TA9728AA	58	198
ESC_TA9727AA	11855	512

ESC_TA9726AA	10	512
ESC_TA9725AA	542	824
ESC_TA9724AA	8165	111
ESC_TA9723AA	6071	817
ESC_TA9722AA	362	234
ESC_TA9721AA	329	355
ESC_TA9720AA	46	447
ESC_TA9719AA	1494	180
ESC_TA9718AA	11854	198
ESC_TA9717AA	453	935
ESC_TA9716AA	10	512
ESC_TA9715AA	131	653
ESC_TA9714AA	542	824
ESC_TA9713AA	48	76
ESC_TA9712AA	405	923
ESC_TA9711AA	7345	377
ESC_TA9710AA	101	696
ESC_TA9709AA	167	512
ESC_TA9708AA	10	512
ESC_TA9707AA	129	257
ESC_TA9706AA	156	175
ESC_TA9705AA	75	257
ESC_TA9704AA	156	175
ESC_TA9703AA	11853	512
ESC_TA9702AA	23	156
ESC_TA9701AA	1196	257
ESC_TA9700AA	2325	447
ESC_TA9699AA	1431	257
ESC_TA9698AA	457	595
ESC_TA9697AA	11852	445
ESC_TA9696AA	641	935
ESC_TA9695AA	3631	71
ESC_TA9694AA	101	696
ESC_TA9693AA	165	504
ESC_TA9692AA	88	160
ESC_TA9691AA	48	76
ESC_TA9690AA	117	592
ESC_TA9689AA	11851	546
ESC_TA9688AA	88	160
ESC_TA9687AA	101	696
ESC_TA9686AA	10	512
ESC_TA9685AA	165	504
ESC_TA9684AA	57	50
ESC_TA9683AA	162	924
ESC_TA9682AA	88	160
ESC_TA9681AA	1611	198
ESC_TA9680AA	765	156
ESC_TA9679AA	117	592
ESC_TA9678AA	101	696
ESC_TA9677AA	11850	71

ESC_TA9676AA	58	198
ESC_TA9675AA	162	924
ESC_TA9674AA	117	592
ESC_TA9673AA	88	160
ESC_TA9672AA	11849	924
ESC_TA9671AA	75	257
ESC_TA9670AA	88	160
ESC_TA9669AA	683	175
ESC_TA9668AA	101	696
ESC_TA9667AA	567	740
ESC_TA9666AA	3014	410
ESC_TA9665AA	88	160
ESC_TA9664AA	117	592
ESC_TA9663AA	10	512
ESC_TA9662AA	69	387
ESC_TA9661AA	156	175
ESC_TA9660AA	648	359
ESC_TA9659AA	361	512
ESC_TA9658AA	10	512
ESC_TA9657AA	57	50
ESC_TA9656AA	48	76
ESC_TA9655AA	410	156
ESC_TA9654AA	453	935
ESC_TA9653AA	446	257
ESC_TA9652AA	398	445
ESC_TA9651AA	156	175
ESC_TA9650AA	641	935
ESC_TA9649AA	224	198
ESC_TA9648AA	44	512
ESC_TA9647AA	847	257
ESC_TA9646AA	156	175
ESC_TA9645AA	641	935
ESC_TA9644AA	410	156
ESC_TA9642AA	8047	213
ESC_TA9641AA	1433	601
ESC_TA9640AA	1001	257
ESC_TA9639AA	453	935
ESC_TA9638AA	57	50
ESC_TA9637AA	58	198
ESC_TA9636AA	1431	257
ESC_TA9635AA	101	696
ESC_TA9634AA	189	504
ESC_TA9633AA	58	198
ESC_TA9632AA	1196	257
ESC_TA9631AA	101	696
ESC_TA9630AA	11847	108
ESC_TA9629AA	93	71
ESC_TA9628AA	11846	924
ESC_TA9627AA	4994	584
ESC_TA9626AA	11046	924

ESC_TA9625AA	224	198
ESC_TA9624AA	58	198
ESC_TA9623AA	58	198
ESC_TA9622AA	11845	670
ESC_TA9621AA	542	824
ESC_TA9620AA	93	71
ESC_TA9619AA	117	592
ESC_TA9618AA	997	915
ESC_TA9617AA	394	400
ESC_TA9616AA	359	696
ESC_TA9615AA	648	359
ESC_TA9614AA	155	175
ESC_TA9613AA	1684	504
ESC_TA9612AA	117	592
ESC_TA9611AA	453	935
ESC_TA9610AA	1485	408
ESC_TA9609AA	2144	257
ESC_TA9608AA	101	696
ESC_TA9607AA	993	789
ESC_TA9606AA	101	696
ESC_TA9605AA	101	696
ESC_TA9604AA	1716	512
ESC_TA9603AA	349	670
ESC_TA9602AA	10	512
ESC_TA9601AA	58	198
ESC_TA9600AA	1421	447
ESC_TA9599AA	2179	924
ESC_TA9598AA	117	592
ESC_TA9597AA	101	696
ESC_TA9596AA	117	592
ESC_TA9595AA	58	198
ESC_TA9594AA	457	595
ESC_TA9593AA	361	512
ESC_TA9592AA	69	387
ESC_TA9591AA	1437	512
ESC_TA9590AA	3564	160
ESC_TA9589AA	11844	257
ESC_TA9588AA	5416	445
ESC_TA9587AA	297	234
ESC_TA9586AA	10	512
ESC_TA9585AA	155	175
ESC_TA9584AA	297	234
ESC_TA9583AA	165	504
ESC_TA9582AA	10	512
ESC_TA9581AA	57	50
ESC_TA9580AA	1112	504
ESC_TA9579AA	189	504
ESC_TA9578AA	1640	50
ESC_TA9577AA	10	512
ESC_TA9576AA	58	198

ESC_TA9575AA	212	198
ESC_TA9574AA	2496	789
ESC_TA9573AA	11843	160
ESC_TA9572AA	58	198
ESC_TA9571AA	58	198
ESC_TA9570AA	11843	160
ESC_TA9569AA	58	198
ESC_TA9568AA	58	198
ESC_TA9567AA	58	198
ESC_TA9566AA	101	696
ESC_TA9565AA	11842	696
ESC_TA9564AA	10	512
ESC_TA9563AA	1148	257
ESC_TA9562AA	1137	512
ESC_TA9561AA	6215	962
ESC_TA9560AA	6215	962
ESC_TA9559AA	4373	831
ESC_TA9558AA	20	198
ESC_TA9557AA	1079	235
ESC_TA9556AA	101	696
ESC_TA9555AA	5899	592
ESC_TA9554AA	2325	447
ESC_TA9553AA	661	92
ESC_TA9552AA	847	257
ESC_TA9551AA	10	512
ESC_TA9550AA	1722	599
ESC_TA9549AA	212	198
ESC_TA9548AA	1721	311
ESC_TA9547AA	9578	512
ESC_TA9546AA	1011	198
ESC_TA9545AA	162	924
ESC_TA9544AA	1011	198
ESC_TA9543AA	58	198
ESC_TA9542AA	48	76
ESC_TA9541AA	2040	198
ESC_TA9540AA	56	198
ESC_TA9539AA	491	626
ESC_TA9538AA	2541	878
ESC_TA9537AA	641	935
ESC_TA9536AA	88	160
ESC_TA9535AA	88	160
ESC_TA9534AA	11848	263
ESC_TA9533AA	11848	263
ESC_TA9532AA	88	160
ESC_TA9531AA	10	512
ESC_TA9530AA	11537	286
ESC_TA9529AA	361	512
ESC_TA9528AA	5175	198
ESC_TA9527AA	156	175
ESC_TA9526AA	678	257

ESC_TA9525AA	88	160
ESC_TA9524AA	10	512
ESC_TA9523AA	11857	156
ESC_TA9522AA	10	512
ESC_TA9521AA	101	696
ESC_TA9520AA	10	512
ESC_TA9519AA	7445	512
ESC_TA9518AA	9699	198
ESC_TA9517AA	88	160
ESC_TA9516AA	540	286
ESC_TA9515AA	75	257
ESC_TA9514AA	10	512
ESC_TA9513AA	9562	198
ESC_TA9512AA	10	512
ESC_TA9511AA	58	198
ESC_TA9510AA	410	156
ESC_TA9509AA	155	175
ESC_TA9508AA	6448	175
ESC_TA9507AA	10	512
ESC_TA9506AA	2496	789
ESC_TA9505AA	10	512
ESC_TA9504AA	56	198
ESC_TA9503AA	75	257
ESC_TA9502AA	1642	198
ESC_TA9498AA	1684	504
ESC_TA9496AA	2179	924
ESC_TA9495AA	515	1004
ESC_TA9494AA	410	156
ESC_TA9493AA	993	789
ESC_TA9492AA	648	359
ESC_TA9490AA	2197	511
ESC_TA9489AA	1684	504
ESC_TA9488AA	23	156
ESC_TA9487AA	11861	512
ESC_TA9486AA	93	71
ESC_TA9485AA	131	653
ESC_TA9484AA	457	595
ESC_TA9483AA	2179	924
ESC_TA9482AA	101	696
ESC_TA9481AA	1079	235
ESC_TA9480AA	11860	696
ESC_TA9479AA	666	257
ESC_TA9478AA	4580	878
ESC_TA9477AA	34	504
ESC_TA9476AA	3014	410
ESC_TA9475AA	77	286
ESC_TA9474AA	88	160
ESC_TA9472AA	744	512
ESC_TA9470AA	602	257
ESC_TA9469AA	410	156

ESC_TA9468AA	29	156
ESC_TA9467AA	101	696
ESC_TA9466AA	453	935
ESC_TA9464AA	34	504
ESC_TA9463AA	93	71
ESC_TA9462AA	744	512
ESC_TA9461AA	58	198
ESC_TA9460AA	117	592
ESC_TA9459AA	117	592
ESC_TA9458AA	11859	175
ESC_TA9457AA	88	160
ESC_TA9456AA	58	198
ESC_TA9455AA	88	160
ESC_TA9454AA	367	156
ESC_TA9453AA	5926	50
ESC_TA9452AA	101	696
ESC_TA9451AA	58	198
ESC_TA9450AA	101	696
ESC_TA9449AA	10	512
ESC_TA9448AA	5229	696
ESC_TA9447AA	93	71
ESC_TA9446AA	1083	203
ESC_TA9445AA	10	512
ESC_TA9443AA	23	156
ESC_TA9442AA	1629	924
ESC_TA9441AA	167	512
ESC_TA9440AA	117	592
ESC_TA9438AA	75	257
ESC_TA9437AA	88	160
ESC_TA9436AA	410	156
ESC_TA9435AA	164	175
ESC_TA9434AA	10	512
ESC_TA9433AA	109	257
ESC_TA9432AA	410	156
ESC_TA9431AA	167	512
ESC_TA9430AA	93	71
ESC_TA9429AA	90	156
ESC_TA9428AA	410	156
ESC_TA9427AA	101	696
ESC_TA9426AA	167	512
ESC_TA9425AA	2325	447
ESC_TA9424AA	1431	257
ESC_TA9423AA	744	512
ESC_TA9422AA	58	198
ESC_TA9421AA	11858	198
ESC_TA9420AA	2521	257
ESC_TA9419AA	361	512
ESC_TA9418AA	10	512
ESC_TA9417AA	744	512
ESC_TA9416AA	993	789

ESC_TA9415AA	48	76
ESC_TA9414AA	23	156
ESC_TA9411AA	38	669
ESC_TA9410AA	1722	599
ESC_TA9409AA	2599	257
ESC_TA9408AA	101	696
ESC_TA9407AA	410	156
ESC_TA9406AA	453	935
ESC_TA9405AA	3057	672
ESC_TA9404AA	648	359
ESC_TA9403AA	117	592
ESC_TA9402AA	48	76
ESC_TA9401AA	4496	924
ESC_TA9400AA	1304	257
ESC_TA9392AA	3	198
ESC_TA9391AA	3	198
ESC_TA9390AA	3	198
ESC_TA9389AA	3	198
ESC_TA9388AA	3	198
ESC_TA9387AA	3	198
ESC_TA9386AA	3	198
ESC_TA9385AA	3	198
ESC_TA9384AA	3	198
ESC_TA9383AA	3	198
ESC_TA9382AA	3	198
ESC_TA9381AA	3	198
ESC_TA9380AA	3	198
ESC_TA9379AA	3	198
ESC_TA9378AA	3	198
ESC_TA9377AA	3	198
ESC_TA9376AA	3	198
ESC_TA9375AA	7368	511
ESC_TA9374AA	10	512
ESC_TA9373AA	131	653
ESC_TA9372AA	11801	653
ESC_TA9371AA	12	626
ESC_TA9370AA	131	653
ESC_TA9369AA	3	198
ESC_TA9368AA	3	198
ESC_TA9367AA	131	653
ESC_TA9366AA	3	198
ESC_TA9365AA	3	198
ESC_TA9364AA	3	198
ESC_TA9363AA	69	387
ESC_TA9361AA	3	198
ESC_TA9360AA	3	198
ESC_TA9359AA	1907	766
ESC_TA9358AA	38	669
ESC_TA9357AA	4305	512
ESC_TA9356AA	40	198

ESC_TA9355AA	301	307
ESC_TA9354AA	301	307
ESC_TA9353AA	11800	198
ESC_TA9351AA	11302	261
ESC_TA9350AA	11701	261
ESC_TA9349AA	245	261
ESC_TA9348AA	11840	217
ESC_TA9347AA	1423	198
ESC_TA9346AA	11839	912
ESC_TA9345AA	973	968
ESC_TA9344AA	8576	212
ESC_TA9343AA	2606	198
ESC_TA9342AA	2329	924
ESC_TA9341AA	48	76
ESC_TA9340AA	9514	504
ESC_TA9339AA	399	198
ESC_TA9338AA	10	512
ESC_TA9337AA	7488	156
ESC_TA9336AA	2362	772
ESC_TA9335AA	10	512
ESC_TA9334AA	362	234
ESC_TA9333AA	21	156
ESC_TA9332AA	29	156
ESC_TA9331AA	21	156
ESC_TA9330AA	28	537
ESC_TA9329AA	29	156
ESC_TA9328AA	28	537
ESC_TA9327AA	28	537
ESC_TA9326AA	299	773
ESC_TA9325AA	299	773
ESC_TA9324AA	1283	766
ESC_TA9323AA	19	774
ESC_TA9322AA	19	774
ESC_TA9321AA	278	924
ESC_TA9320AA	162	924
ESC_TA9319AA	1326	198
ESC_TA9318AA	3631	71
ESC_TA9317AA	88	160
ESC_TA9316AA	7040	156
ESC_TA9315AA	349	670
ESC_TA9314AA	4935	445
ESC_TA9313AA	155	175
ESC_TA9312AA	117	592
ESC_TA9311AA	11838	512
ESC_TA9310AA	38	669
ESC_TA9309AA	877	935
ESC_TA9308AA	58	198
ESC_TA9307AA	720	900
ESC_TA9305AA	3234	257
ESC_TA9304AA	11837	789

ESC_TA9303AA	942	198
ESC_TA9302AA	2253	71
ESC_TA9301AA	155	175
ESC_TA9300AA	295	160
ESC_TA9299AA	1727	257
ESC_TA9298AA	278	924
ESC_TA9297AA	33	198
ESC_TA9296AA	11799	234
ESC_TA9294AA	101	696
ESC_TA9293AA	21	156
ESC_TA9292AA	11798	936
ESC_TA9291AA	5400	261
ESC_TA9290AA	33	198
ESC_TA9289AA	1819	808
ESC_TA9288AA	1819	808
ESC_TA9287AA	301	307
ESC_TA9286AA	381	156
ESC_TA9285AA	131	653
ESC_TA9284AA	517	924
ESC_TA9283AA	1011	198
ESC_TA9282AA	5891	878
ESC_TA9281AA	1633	880
ESC_TA9280AA	73	737
ESC_TA9279AA	224	198
ESC_TA9278AA	11767	180
ESC_TA9277AA	201	257
ESC_TA9276AA	297	234
ESC_TA9275AA	67	257
ESC_TA9274AA	73	737
ESC_TA9273AA	2773	257
ESC_TA9272AA	7624	175
ESC_TA9271AA	11742	160
ESC_TA9270AA	3594	924
ESC_TA9269AA	164	175
ESC_TA9268AA	131	653
ESC_TA9267AA	2602	257
ESC_TA9266AA	448	257
ESC_TA9265AA	21	156
ESC_TA9264AA	17	191
ESC_TA9263AA	17	191
ESC_TA9262AA	17	191
ESC_TA9261AA	21	156
ESC_TA9260AA	21	156
ESC_TA9259AA	17	191
ESC_TA9258AA	21	156
ESC_TA9257AA	17	191
ESC_TA9256AA	21	156
ESC_TA9255AA	17	191
ESC_TA9254AA	21	156
ESC_TA9253AA	191	198

ESC_TA9251AA	973	968
ESC_TA9249AA	10	512
ESC_TA9248AA	38	669
ESC_TA9247AA	1385	653
ESC_TA9246AA	399	198
ESC_TA9245AA	155	175
ESC_TA9244AA	11661	912
ESC_TA9243AA	11668	878
ESC_TA9242AA	1662	924
ESC_TA9241AA	297	234
ESC_TA9240AA	847	257
ESC_TA9239AA	642	924
ESC_TA9238AA	1722	599
ESC_TA9237AA	196	257
ESC_TA9236AA	714	318
ESC_TA9235AA	70	670
ESC_TA9234AA	11285	924
ESC_TA9233AA	11669	1032
ESC_TA9232AA	877	935
ESC_TA9231AA	69	387
ESC_TA9230AA	162	924
ESC_TA9229AA	162	924
ESC_TA9228AA	162	924
ESC_TA9227AA	164	175
ESC_TA9226AA	164	175
ESC_TA9225AA	164	175
ESC_TA9224AA	11671	962
ESC_TA9223AA	164	175
ESC_TA9222AA	446	257
ESC_TA9221AA	162	924
ESC_TA9219AA	162	924
ESC_TA9218AA	162	924
ESC_TA9216AA	162	924
ESC_TA9214AA	11741	652
ESC_TA9212AA	99	198
ESC_TA9211AA	761	512
ESC_TA9210AA	11158	235
ESC_TA9209AA	38	669
ESC_TA9208AA	40	198
ESC_TA9207AA	5295	355
ESC_TA9206AA	409	504
ESC_TA9204AA	2005	175
ESC_TA9203AA	607	355
ESC_TA9202AA	3595	822
ESC_TA9201AA	4379	878
ESC_TA9200AA	4379	878
ESC_TA9199AA	3595	822
ESC_TA9198AA	11796	504
ESC_TA9197AA	4408	286
ESC_TA9195AA	542	824

ESC_TA9194AA	542	824
ESC_TA9193AA	11795	76
ESC_TA9192AA	11795	76
ESC_TA9189AA	11599	144
ESC_TA9188AA	398	445
ESC_TA9187AA	11794	286
ESC_TA9186AA	10	512
ESC_TA9185AA	189	504
ESC_TA9184AA	542	824
ESC_TA9183AA	3595	822
ESC_TA9182AA	3595	822
ESC_TA9181AA	3595	822
ESC_TA9180AA	3595	822
ESC_TA9179AA	189	504
ESC_TA9178AA	58	198
ESC_TA9177AA	11	936
ESC_TA9176AA	372	424
ESC_TA9175AA	38	669
ESC_TA9174AA	457	595
ESC_TA9173AA	372	424
ESC_TA9172AA	1193	761
ESC_TA9171AA	10	512
ESC_TA9170AA	10	512
ESC_TA9169AA	10	512
ESC_TA9168AA	2179	924
ESC_TA9167AA	2179	924
ESC_TA9166AA	301	307
ESC_TA9165AA	21	156
ESC_TA9164AA	301	307
ESC_TA9163AA	442	257
ESC_TA9162AA	301	307
ESC_TA9161AA	301	307
ESC_TA9160AA	2283	817
ESC_TA9159AA	301	307
ESC_TA9158AA	19	774
ESC_TA9157AA	3	198
ESC_TA9156AA	7444	937
ESC_TA9155AA	1283	766
ESC_TA9154AA	19	774
ESC_TA9153AA	1283	766
ESC_TA9152AA	1283	766
ESC_TA9151AA	1283	766
ESC_TA9150AA	3	198
ESC_TA9149AA	1283	766
ESC_TA9148AA	11792	766
ESC_TA9147AA	3	198
ESC_TA9146AA	3	198
ESC_TA9145AA	3	198
ESC_TA9144AA	3	198
ESC_TA9143AA	29	156

ESC_TA9142AA	28	537
ESC_TA9141AA	3	198
ESC_TA9140AA	3	198
ESC_TA9139AA	28	537
ESC_TA9138AA	206	286
ESC_TA9137AA	3	198
ESC_TA9136AA	245	261
ESC_TA9134AA	11790	261
ESC_TA9133AA	1767	928
ESC_TA9132AA	1753	261
ESC_TA9131AA	11783	261
ESC_TA9130AA	11787	261
ESC_TA9129AA	1753	261
ESC_TA9128AA	1753	261
ESC_TA9127AA	11788	261
ESC_TA9126AA	11786	261
ESC_TA9125AA	11784	261
ESC_TA9111AA	4166	936
ESC_TA9110AA	80	731
ESC_TA9109AA	73	737
ESC_TA9108AA	372	424
ESC_TA9107AA	297	234
ESC_TA9106AA	12	626
ESC_TA9105AA	998	644
ESC_TA9104AA	127	738
ESC_TA9103AA	127	738
ESC_TA9102AA	372	424
ESC_TA9101AA	1273	256
ESC_TA9100AA	145	256
ESC_TA9099AA	1273	256
ESC_TA9098AA	245	261
ESC_TA9096AA	152	257
ESC_TA9095AA	245	261
ESC_TA9094AA	245	261
ESC_TA9093AA	245	261
ESC_TA9092AA	95	738
ESC_TA9091AA	144	740
ESC_TA9090AA	648	359
ESC_TA9089AA	442	257
ESC_TA9088AA	11836	268
ESC_TA9087AA	11835	198
ESC_TA9086AA	11	936
ESC_TA9085AA	6118	257
ESC_TA9084AA	131	653
ESC_TA9083AA	162	924
ESC_TA9082AA	4981	473
ESC_TA9080AA	44	512
ESC_TA9079AA	48	76
ESC_TA9078AA	38	669
ESC_TA9077AA	11813	286

ESC_TA9076AA	5086	286
ESC_TA9075AA	10	512
ESC_TA9074AA	5086	286
ESC_TA9073AA	10	512
ESC_TA9072AA	10	512
ESC_TA9071AA	10	512
ESC_TA9070AA	744	512
ESC_TA9069AA	10	512
ESC_TA9068AA	2005	175
ESC_TA9067AA	10	512
ESC_TA9066AA	11769	924
ESC_TA9065AA	10	512
ESC_TA9064AA	3944	512
ESC_TA9063AA	973	968
ESC_TA9062AA	1788	785
ESC_TA9061AA	3	198
ESC_TA9060AA	501	900
ESC_TA9059AA	3	198
ESC_TA9058AA	28	537
ESC_TA9057AA	28	537
ESC_TA9056AA	28	537
ESC_TA9055AA	245	261
ESC_TA9054AA	245	261
ESC_TA9053AA	25	234
ESC_TA9052AA	33	198
ESC_TA9051AA	3101	160
ESC_TA9050AA	6992	842
ESC_TA9049AA	1722	599
ESC_TA9048AA	73	737
ESC_TA9047AA	357	654
ESC_TA9046AA	7870	198
ESC_TA9045AA	131	653
ESC_TA9044AA	457	595
ESC_TA9043AA	399	198
ESC_TA9042AA	3576	175
ESC_TA9041AA	635	141
ESC_TA9040AA	95	738
ESC_TA9039AA	73	737
ESC_TA9038AA	216	512
ESC_TA9037AA	131	653
ESC_TA9036AA	457	595
ESC_TA9035AA	95	738
ESC_TA9034AA	10	512
ESC_TA9033AA	95	738
ESC_TA9032AA	5295	355
ESC_TA9031AA	420	741
ESC_TA9030AA	12	626
ESC_TA9029AA	21	156
ESC_TA9028AA	21	156
ESC_TA9027AA	21	156

ESC_TA9026AA	21	156
ESC_TA9025AA	21	156
ESC_TA9024AA	21	156
ESC_TA9023AA	21	156
ESC_TA9022AA	21	156
ESC_TA9021AA	21	156
ESC_TA9020AA	29	156
ESC_TA9019AA	152	257
ESC_TA9018AA	152	257
ESC_TA9017AA	11	936
ESC_TA9016AA	10	512
ESC_TA9015AA	10	512
ESC_TA9014AA	515	1004
ESC_TA9013AA	218	519
ESC_TA9012AA	218	519
ESC_TA9011AA	10	512
ESC_TA9010AA	361	512
ESC_TA9009AA	33	198
ESC_TA9008AA	38	669
ESC_TA9007AA	5150	661
ESC_TA9006AA	6196	223
ESC_TA9005AA	38	669
ESC_TA9004AA	1193	761
ESC_TA9003AA	3570	156
ESC_TA9002AA	131	653
ESC_TA9001AA	2155	387
ESC_TA9000AA	349	670
ESC_TA8999AA	38	669
ESC_TA8998AA	131	653
ESC_TA8997AA	847	257
ESC_TA8984AA	1146	198
ESC_TA8983AA	155	175
ESC_TA8982AA	10	512
ESC_TA8981AA	1423	198
ESC_TA8980AA	189	504
ESC_TA8979AA	10	512
ESC_TA8978AA	58	198
ESC_TA8977AA	501	900
ESC_TA8976AA	58	198
ESC_TA8975AA	349	670
ESC_TA8974AA	744	512
ESC_TA8973AA	155	175
ESC_TA8970AA	1324	156
ESC_TA8969AA	11671	962
ESC_TA8968AA	11671	962
ESC_TA8967AA	11671	962
ESC_TA8966AA	164	175
ESC_TA8965AA	164	175
ESC_TA8964AA	95	738
ESC_TA8963AA	355	764

ESC_TA8961AA	127	738
ESC_TA8960AA	6745	885
ESC_TA8959AA	11768	234
ESC_TA8958AA	95	738
ESC_TA8957AA	155	175
ESC_TA8956AA	129	257
ESC_TA8955AA	6701	535
ESC_TA8954AA	69	387
ESC_TA8953AA	5597	72
ESC_TA8952AA	720	900
ESC_TA8951AA	737	175
ESC_TA8950AA	357	654
ESC_TA8949AA	635	141
ESC_TA8948AA	11	936
ESC_TA8947AA	11	936
ESC_TA8946AA	11	936
ESC_TA8945AA	11	936
ESC_TA8944AA	11	936
ESC_TA8943AA	11	936
ESC_TA8942AA	11	936
ESC_TA8941AA	11	936
ESC_TA8940AA	11	936
ESC_TA8939AA	11	936
ESC_TA8938AA	11	936
ESC_TA8937AA	11	936
ESC_TA8936AA	11	936
ESC_TA8935AA	11	936
ESC_TA8934AA	11	936
ESC_TA8933AA	11	936
ESC_TA8932AA	11	936
ESC_TA8931AA	11	936
ESC_TA8930AA	11	936
ESC_TA8929AA	11	936
ESC_TA8928AA	11	936
ESC_TA8927AA	11	936
ESC_TA8926AA	11	936
ESC_TA8925AA	11	936
ESC_TA8924AA	11	936
ESC_TA8923AA	11	936
ESC_TA8922AA	11	936
ESC_TA8921AA	11	936
ESC_TA8920AA	11	936
ESC_TA8919AA	11	936
ESC_TA8918AA	11	936
ESC_TA8917AA	11	936
ESC_TA8916AA	11	936
ESC_TA8915AA	11	936
ESC_TA8914AA	11	936
ESC_TA8913AA	11	936
ESC_TA8912AA	11	936

ESC_TA8911AA	11	936
ESC_TA8910AA	11	936
ESC_TA8909AA	11	936
ESC_TA8908AA	11	936
ESC_TA8907AA	11	936
ESC_TA8906AA	11	936
ESC_TA8905AA	11	936
ESC_TA8904AA	11	936
ESC_TA8903AA	11	936
ESC_TA8902AA	11	936
ESC_TA8901AA	11	936
ESC_TA8900AA	11	936
ESC_TA8899AA	11	936
ESC_TA8898AA	11	936
ESC_TA8897AA	11	936
ESC_TA8896AA	11	936
ESC_TA8895AA	11	936
ESC_TA8894AA	11	936
ESC_TA8893AA	11	936
ESC_TA8892AA	11	936
ESC_TA8891AA	11	936
ESC_TA8890AA	11	936
ESC_TA8889AA	11	936
ESC_TA8888AA	11	936
ESC_TA8887AA	11	936
ESC_TA8886AA	11	936
ESC_TA8885AA	11	936
ESC_TA8884AA	11	936
ESC_TA8883AA	11	936
ESC_TA8882AA	11	936
ESC_TA8881AA	11	936
ESC_TA8880AA	11	936
ESC_TA8879AA	11	936
ESC_TA8878AA	11	936
ESC_TA8877AA	11	936
ESC_TA8876AA	11	936
ESC_TA8875AA	11	936
ESC_TA8874AA	11	936
ESC_TA8873AA	11	936
ESC_TA8872AA	11	936
ESC_TA8871AA	11	936
ESC_TA8870AA	11	936
ESC_TA8869AA	11	936
ESC_TA8868AA	11	936
ESC_TA8867AA	11	936
ESC_TA8866AA	11	936
ESC_TA8865AA	11	936
ESC_TA8864AA	11	936
ESC_TA8863AA	11	936
ESC_TA8862AA	11	936

ESC_TA8861AA	11	936
ESC_TA8860AA	11	936
ESC_TA8859AA	11	936
ESC_TA8858AA	11	936
ESC_TA8857AA	11	936
ESC_TA8856AA	11	936
ESC_TA8855AA	11	936
ESC_TA8854AA	11	936
ESC_TA8853AA	11	936
ESC_TA8852AA	11	936
ESC_TA8851AA	11	936
ESC_TA8850AA	11	936
ESC_TA8849AA	11	936
ESC_TA8848AA	11	936
ESC_TA8847AA	11	936
ESC_TA8846AA	11	936
ESC_TA8845AA	11	936
ESC_TA8844AA	11	936
ESC_TA8843AA	11	936
ESC_TA8842AA	11	936
ESC_TA8841AA	11	936
ESC_TA8840AA	11	936
ESC_TA8839AA	11	936
ESC_TA8838AA	11	936
ESC_TA8837AA	11	936
ESC_TA8836AA	11	936
ESC_TA8835AA	11	936
ESC_TA8834AA	11	936
ESC_TA8833AA	11	936
ESC_TA8832AA	11	936
ESC_TA8831AA	11	936
ESC_TA8830AA	11	936
ESC_TA8829AA	11	936
ESC_TA8828AA	11	936
ESC_TA8827AA	11	936
ESC_TA8826AA	11811	936
ESC_TA8825AA	11	936
ESC_TA8824AA	11	936
ESC_TA8823AA	11	936
ESC_TA8822AA	11	936
ESC_TA8821AA	11	936
ESC_TA8820AA	11	936
ESC_TA8819AA	11	936
ESC_TA8818AA	11	936
ESC_TA8817AA	11	936
ESC_TA8816AA	11	936
ESC_TA8815AA	11	936
ESC_TA8814AA	11	936
ESC_TA8813AA	11	936
ESC_TA8812AA	11	936

ESC_TA8811AA	11	936
ESC_TA8810AA	11	936
ESC_TA8809AA	11	936
ESC_TA8808AA	11	936
ESC_TA8807AA	11	936
ESC_TA8806AA	11	936
ESC_TA8805AA	11	936
ESC_TA8804AA	11	936
ESC_TA8803AA	11	936
ESC_TA8802AA	11	936
ESC_TA8801AA	11	936
ESC_TA8800AA	11	936
ESC_TA8799AA	11	936
ESC_TA8798AA	11	936
ESC_TA8797AA	11	936
ESC_TA8796AA	11	936
ESC_TA8795AA	11	936
ESC_TA8794AA	11	936
ESC_TA8793AA	11	936
ESC_TA8792AA	11	936
ESC_TA8791AA	11	936
ESC_TA8790AA	11	936
ESC_TA8789AA	11	936
ESC_TA8788AA	11	936
ESC_TA8787AA	11	936
ESC_TA8786AA	11	936
ESC_TA8785AA	11	936
ESC_TA8784AA	11	936
ESC_TA8783AA	11	936
ESC_TA8782AA	11	936
ESC_TA8781AA	11	936
ESC_TA8780AA	11	936
ESC_TA8779AA	11	936
ESC_TA8778AA	11	936
ESC_TA8777AA	11	936
ESC_TA8776AA	11	936
ESC_TA8775AA	11	936
ESC_TA8774AA	11	936
ESC_TA8773AA	11	936
ESC_TA8772AA	11	936
ESC_TA8771AA	11	936
ESC_TA8770AA	11	936
ESC_TA8769AA	11	936
ESC_TA8768AA	11	936
ESC_TA8767AA	11	936
ESC_TA8766AA	11	936
ESC_TA8765AA	11	936
ESC_TA8764AA	11	936
ESC_TA8763AA	11	936
ESC_TA8762AA	11	936

ESC_TA8761AA	11	936
ESC_TA8760AA	11	936
ESC_TA8759AA	11	936
ESC_TA8758AA	11	936
ESC_TA8757AA	11	936
ESC_TA8756AA	11	936
ESC_TA8755AA	11	936
ESC_TA8754AA	11	936
ESC_TA8753AA	11	936
ESC_TA8752AA	164	175
ESC_TA8751AA	38	669
ESC_TA8750AA	1485	408
ESC_TA8749AA	117	592
ESC_TA8748AA	1079	235
ESC_TA8747AA	327	204
ESC_TA8746AA	1252	567
ESC_TA8745AA	38	669
ESC_TA8744AA	10	512
ESC_TA8743AA	162	924
ESC_TA8742AA	8611	422
ESC_TA8741AA	491	626
ESC_TA8739AA	301	307
ESC_TA8738AA	11756	537
ESC_TA8737AA	201	257
ESC_TA8736AA	73	737
ESC_TA8735AA	5891	878
ESC_TA8734AA	1011	198
ESC_TA8733AA	73	737
ESC_TA8732AA	67	257
ESC_TA8731AA	297	234
ESC_TA8730AA	11767	180
ESC_TA8729AA	224	198
ESC_TA8728AA	517	924
ESC_TA8727AA	1633	880
ESC_TA8726AA	131	653
ESC_TA8725AA	448	257
ESC_TA8724AA	2602	257
ESC_TA8723AA	131	653
ESC_TA8722AA	164	175
ESC_TA8721AA	3594	924
ESC_TA8720AA	7624	175
ESC_TA8719AA	11742	160
ESC_TA8718AA	2773	257
ESC_TA8717AA	1722	599
ESC_TA8716AA	70	670
ESC_TA8715AA	196	257
ESC_TA8714AA	714	318
ESC_TA8713AA	1662	924
ESC_TA8712AA	297	234
ESC_TA8711AA	847	257

ESC_TA8710AA	642	924
ESC_TA8709AA	99	198
ESC_TA8708AA	847	257
ESC_TA8707AA	761	512
ESC_TA8706AA	607	355
ESC_TA8705AA	2005	175
ESC_TA8704AA	409	504
ESC_TA8703AA	5295	355
ESC_TA8702AA	40	198
ESC_TA8701AA	38	669
ESC_TA8700AA	11741	652
ESC_TA8699AA	472	601
ESC_TA8698AA	95	738
ESC_TA8697AA	635	141
ESC_TA8696AA	399	198
ESC_TA8695AA	3576	175
ESC_TA8694AA	457	595
ESC_TA8693AA	131	653
ESC_TA8692AA	7870	198
ESC_TA8691AA	12	626
ESC_TA8690AA	357	654
ESC_TA8689AA	10	512
ESC_TA8688AA	5295	355
ESC_TA8687AA	420	741
ESC_TA8686AA	95	738
ESC_TA8685AA	95	738
ESC_TA8684AA	131	653
ESC_TA8683AA	457	595
ESC_TA8682AA	73	737
ESC_TA8681AA	1722	599
ESC_TA8680AA	877	935
ESC_TA8679AA	162	924
ESC_TA8678AA	216	512
ESC_TA8677AA	446	257
ESC_TA8676AA	69	387
ESC_TA8675AA	164	175
ESC_TA8674AA	164	175
ESC_TA8673AA	73	737
ESC_TA8672AA	164	175
ESC_TA8671AA	164	175
ESC_TA8670AA	162	924
ESC_TA8669AA	162	924
ESC_TA8668AA	162	924
ESC_TA8667AA	162	924
ESC_TA8666AA	162	924
ESC_TA8665AA	162	924
ESC_TA8664AA	162	924
ESC_TA8663AA	162	924
ESC_TA8662AA	6096	257
ESC_TA8660AA	11797	445

ESC_TA8659AA	117	592
ESC_TA8658AA	245	261
ESC_TA8657AA	152	257
ESC_TA8656AA	152	257
ESC_TA8655AA	152	257
ESC_TA8654AA	152	257
ESC_TA8653AA	245	261
ESC_TA8652AA	245	261
ESC_TA8651AA	245	261
ESC_TA8650AA	152	257
ESC_TA8649AA	145	256
ESC_TA8648AA	245	261
ESC_TA8647AA	245	261
ESC_TA8646AA	243	197
ESC_TA8645AA	11739	71
ESC_TA8644AA	11738	511
ESC_TA8643AA	11737	286
ESC_TA8642AA	11734	142
ESC_TA8641AA	11735	160
ESC_TA8640AA	11736	504
ESC_TA8639AA	152	257
ESC_TA8638AA	245	261
ESC_TA8637AA	245	261
ESC_TA8636AA	245	261
ESC_TA8635AA	1025	261
ESC_TA8634AA	245	261
ESC_TA8633AA	1877	773
ESC_TA8632AA	10	512
ESC_TA8631AA	442	257
ESC_TA8629AA	6130	268
ESC_TA8628AA	11	936
ESC_TA8627AA	245	261
ESC_TA8626AA	245	261
ESC_TA8625AA	245	261
ESC_TA8624AA	245	261
ESC_TA8623AA	295	160
ESC_TA8622AA	4774	198
ESC_TA8621AA	11793	512
ESC_TA8620AA	11780	696
ESC_TA8619AA	328	924
ESC_TA8618AA	2271	929
ESC_TA8617AA	131	653
ESC_TA8616AA	428	654
ESC_TA8614AA	162	924
ESC_TA8613AA	162	924
ESC_TA8612AA	7040	156
ESC_TA8611AA	1158	387
ESC_TA8610AA	399	198
ESC_TA8609AA	770	587
ESC_TA8608AA	38	669

ESC_TA8607AA	117	592
ESC_TA8606AA	1434	512
ESC_TA8605AA	1434	512
ESC_TA8604AA	1434	512
ESC_TA8603AA	1434	512
ESC_TA8602AA	1434	512
ESC_TA8601AA	1434	512
ESC_TA8600AA	1434	512
ESC_TA8585AA	4358	71
ESC_TA8583AA	1056	257
ESC_TA8582AA	69	387
ESC_TA8581AA	38	669
ESC_TA8580AA	1652	670
ESC_TA8579AA	131	653
ESC_TA8578AA	1236	380
ESC_TA8577AA	224	198
ESC_TA8576AA	940	257
ESC_TA8575AA	744	512
ESC_TA8574AA	127	738
ESC_TA8573AA	8346	234
ESC_TA8572AA	10	512
ESC_TA8571AA	38	669
ESC_TA8570AA	354	15
ESC_TA8569AA	38	669
ESC_TA8568AA	11362	380
ESC_TA8567AA	99	198
ESC_TA8566AA	297	234
ESC_TA8565AA	127	738
ESC_TA8564AA	205	257
ESC_TA8563AA	38	669
ESC_TA8562AA	1049	175
ESC_TA8561AA	349	670
ESC_TA8560AA	70	670
ESC_TA8559AA	10	512
ESC_TA8558AA	362	234
ESC_TA8557AA	940	257
ESC_TA8556AA	167	512
ESC_TA8555AA	38	669
ESC_TA8554AA	38	669
ESC_TA8553AA	69	387
ESC_TA8552AA	38	669
ESC_TA8551AA	410	156
ESC_TA8550AA	38	669
ESC_TA8549AA	5552	261
ESC_TA8548AA	38	669
ESC_TA8547AA	38	669
ESC_TA8546AA	46	447
ESC_TA8545AA	372	424
ESC_TA8544AA	361	512
ESC_TA8543AA	216	512

ESC_TA8542AA	127	738
ESC_TA8541AA	38	669
ESC_TA8540AA	38	669
ESC_TA8539AA	38	669
ESC_TA8538AA	38	669
ESC_TA8537AA	410	156
ESC_TA8536AA	38	669
ESC_TA8535AA	127	738
ESC_TA8534AA	540	286
ESC_TA8533AA	127	738
ESC_TA8532AA	127	738
ESC_TA8531AA	38	669
ESC_TA8530AA	227	504
ESC_TA8529AA	38	669
ESC_TA8528AA	963	669
ESC_TA8527AA	164	175
ESC_TA8526AA	10644	424
ESC_TA8525AA	224	198
ESC_TA8524AA	6262	76
ESC_TA8523AA	10	512
ESC_TA8522AA	38	669
ESC_TA8521AA	405	923
ESC_TA8520AA	38	669
ESC_TA8519AA	6691	878
ESC_TA8518AA	2521	257
ESC_TA8517AA	205	257
ESC_TA8516AA	939	198
ESC_TA8515AA	127	738
ESC_TA8514AA	10	512
ESC_TA8513AA	453	935
ESC_TA8512AA	1722	599
ESC_TA8511AA	44	512
ESC_TA8510AA	167	512
ESC_TA8509AA	38	669
ESC_TA8508AA	131	653
ESC_TA8507AA	131	653
ESC_TA8506AA	746	789
ESC_TA8505AA	372	424
ESC_TA8504AA	38	669
ESC_TA8503AA	345	175
ESC_TA8502AA	69	387
ESC_TA8501AA	38	669
ESC_TA8500AA	167	512
ESC_TA8499AA	23	156
ESC_TA8498AA	38	669
ESC_TA8497AA	38	669
ESC_TA8496AA	342	286
ESC_TA8495AA	11	936
ESC_TA8494AA	675	234
ESC_TA8493AA	3476	512

ESC_TA8492AA	58	198
ESC_TA8491AA	5869	257
ESC_TA8490AA	3476	512
ESC_TA8489AA	398	445
ESC_TA8488AA	3476	512
ESC_TA8487AA	58	198
ESC_TA8486AA	58	198
ESC_TA8485AA	5869	257
ESC_TA8484AA	5869	257
ESC_TA8483AA	1140	934
ESC_TA8482AA	5869	257
ESC_TA8481AA	300	286
ESC_TA8480AA	224	198
ESC_TA8479AA	58	198
ESC_TA8478AA	162	924
ESC_TA8477AA	3476	512
ESC_TA8476AA	58	198
ESC_TA8475AA	223	198
ESC_TA8474AA	58	198
ESC_TA8473AA	58	198
ESC_TA8472AA	446	257
ESC_TA8471AA	2226	512
ESC_TA8470AA	58	198
ESC_TA8469AA	131	653
ESC_TA8468AA	1594	71
ESC_TA8467AA	131	653
ESC_TA8466AA	973	968
ESC_TA8465AA	131	653
ESC_TA8464AA	38	669
ESC_TA8463AA	152	257
ESC_TA8462AA	245	261
ESC_TA8461AA	152	257
ESC_TA8460AA	11	936
ESC_TA8459AA	155	175
ESC_TA8458AA	567	740
ESC_TA8457AA	95	738
ESC_TA8456AA	73	737
ESC_TA8455AA	73	737
ESC_TA8454AA	73	737
ESC_TA8453AA	73	737
ESC_TA8452AA	69	387
ESC_TA8451AA	69	387
ESC_TA8450AA	131	653
ESC_TA8449AA	8137	198
ESC_TA8448AA	11802	626
ESC_TA8447AA	38	669
ESC_TA8446AA	5463	373
ESC_TA8445AA	12	626
ESC_TA8444AA	648	359
ESC_TA8442AA	69	387

ESC_TA8441AA	607	355
ESC_TA8440AA	537	756
ESC_TA8439AA	111	175
ESC_TA8437AA	12	626
ESC_TA8436AA	607	355
ESC_TA8435AA	73	737
ESC_TA8434AA	131	653
ESC_TA8433AA	5662	992
ESC_TA8432AA	453	935
ESC_TA8431AA	453	935
ESC_TA8430AA	453	935
ESC_TA8429AA	21	156
ESC_TA8427AA	73	737
ESC_TA8426AA	69	387
ESC_TA8425AA	131	653
ESC_TA8424AA	141	644
ESC_TA8423AA	131	653
ESC_TA8422AA	131	653
ESC_TA8421AA	73	737
ESC_TA8420AA	540	286
ESC_TA8419AA	95	738
ESC_TA8418AA	393	343
ESC_TA8417AA	69	387
ESC_TA8416AA	80	731
ESC_TA8415AA	10	512
ESC_TA8414AA	73	737
ESC_TA8413AA	12	626
ESC_TA8412AA	141	644
ESC_TA8411AA	1193	761
ESC_TA8410AA	73	737
ESC_TA8409AA	11763	537
ESC_TA8408AA	141	644
ESC_TA8407AA	38	669
ESC_TA8406AA	131	653
ESC_TA8405AA	357	654
ESC_TA8404AA	69	387
ESC_TA8403AA	69	387
ESC_TA8402AA	12	626
ESC_TA8401AA	117	592
ESC_TA8400AA	2015	684
ESC_TA8399AA	69	387
ESC_TA8398AA	131	653
ESC_TA8397AA	127	738
ESC_TA8396AA	73	737
ESC_TA8395AA	348	175
ESC_TA8394AA	5135	278
ESC_TA8393AA	59	217
ESC_TA8392AA	95	738
ESC_TA8391AA	2279	657
ESC_TA8390AA	131	653

ESC_TA8389AA	95	738
ESC_TA8388AA	95	738
ESC_TA8387AA	69	387
ESC_TA8386AA	4219	630
ESC_TA8385AA	1643	780
ESC_TA8384AA	69	387
ESC_TA8383AA	11762	669
ESC_TA8382AA	11761	400
ESC_TA8381AA	12	626
ESC_TA8380AA	2522	198
ESC_TA8379AA	349	670
ESC_TA8378AA	1722	599
ESC_TA8377AA	69	387
ESC_TA8376AA	69	387
ESC_TA8375AA	95	738
ESC_TA8374AA	117	592
ESC_TA8373AA	127	738
ESC_TA8372AA	40	198
ESC_TA8371AA	1193	761
ESC_TA8370AA	6321	541
ESC_TA8369AA	328	924
ESC_TA8368AA	12	626
ESC_TA8367AA	3290	528
ESC_TA8366AA	131	653
ESC_TA8364AA	404	761
ESC_TA8363AA	8371	898
ESC_TA8362AA	11760	626
ESC_TA8361AA	95	738
ESC_TA8360AA	28	537
ESC_TA8359AA	227	504
ESC_TA8358AA	410	156
ESC_TA8357AA	1643	780
ESC_TA8356AA	998	644
ESC_TA8355AA	1844	198
ESC_TA8354AA	58	198
ESC_TA8353AA	80	731
ESC_TA8352AA	131	653
ESC_TA8351AA	95	738
ESC_TA8350AA	95	738
ESC_TA8349AA	453	935
ESC_TA8348AA	10	512
ESC_TA8347AA	526	745
ESC_TA8346AA	517	924
ESC_TA8345AA	69	387
ESC_TA8344AA	69	387
ESC_TA8343AA	8371	898
ESC_TA8342AA	73	737
ESC_TA8341AA	73	737
ESC_TA8340AA	569	738
ESC_TA8339AA	404	761

ESC_TA8338AA	421	744
ESC_TA8337AA	10	512
ESC_TA8336AA	131	653
ESC_TA8335AA	136	392
ESC_TA8334AA	404	761
ESC_TA8333AA	80	731
ESC_TA8332AA	372	424
ESC_TA8330AA	998	644
ESC_TA8329AA	1123	257
ESC_TA8328AA	636	653
ESC_TA8327AA	73	737
ESC_TA8326AA	297	234
ESC_TA8325AA	141	644
ESC_TA8324AA	141	644
ESC_TA8323AA	10	512
ESC_TA8322AA	95	738
ESC_TA8321AA	69	387
ESC_TA8320AA	390	738
ESC_TA8319AA	69	387
ESC_TA8318AA	543	842
ESC_TA8317AA	2013	725
ESC_TA8316AA	404	761
ESC_TA8315AA	1159	381
ESC_TA8314AA	428	654
ESC_TA8313AA	131	653
ESC_TA8312AA	10	512
ESC_TA8311AA	3489	962
ESC_TA8310AA	95	738
ESC_TA8309AA	4219	630
ESC_TA8308AA	59	217
ESC_TA8307AA	357	654
ESC_TA8306AA	127	738
ESC_TA8305AA	1092	739
ESC_TA8304AA	127	738
ESC_TA8303AA	1193	761
ESC_TA8302AA	141	644
ESC_TA8301AA	517	924
ESC_TA8300AA	131	653
ESC_TA8299AA	69	387
ESC_TA8298AA	127	738
ESC_TA8297AA	421	744
ESC_TA8296AA	1431	257
ESC_TA8295AA	1722	599
ESC_TA8294AA	69	387
ESC_TA8293AA	59	217
ESC_TA8292AA	8098	257
ESC_TA8291AA	127	738
ESC_TA8290AA	127	738
ESC_TA8289AA	95	738
ESC_TA8288AA	69	387

ESC_TA8287AA	58	198
ESC_TA8286AA	95	738
ESC_TA8285AA	73	737
ESC_TA8284AA	73	737
ESC_TA8283AA	69	387
ESC_TA8282AA	11765	156
ESC_TA8281AA	93	71
ESC_TA8280AA	69	387
ESC_TA8279AA	10	512
ESC_TA8278AA	73	737
ESC_TA8277AA	69	387
ESC_TA8276AA	162	924
ESC_TA8275AA	297	234
ESC_TA8274AA	95	738
ESC_TA8273AA	95	738
ESC_TA8272AA	95	738
ESC_TA8271AA	69	387
ESC_TA8270AA	101	696
ESC_TA8269AA	131	653
ESC_TA8268AA	7614	160
ESC_TA8267AA	69	387
ESC_TA8266AA	69	387
ESC_TA8265AA	131	653
ESC_TA8264AA	73	737
ESC_TA8263AA	131	653
ESC_TA8262AA	2473	257
ESC_TA8261AA	144	740
ESC_TA8260AA	73	737
ESC_TA8259AA	1434	512
ESC_TA8258AA	404	761
ESC_TA8257AA	162	924
ESC_TA8256AA	95	738
ESC_TA8255AA	117	592
ESC_TA8254AA	1858	757
ESC_TA8253AA	6453	512
ESC_TA8252AA	73	737
ESC_TA8251AA	46	447
ESC_TA8250AA	11804	797
ESC_TA8249AA	396	153
ESC_TA8248AA	216	512
ESC_TA8247AA	58	198
ESC_TA8246AA	62	574
ESC_TA8245AA	73	737
ESC_TA8244AA	95	738
ESC_TA8243AA	141	644
ESC_TA8242AA	973	968
ESC_TA8241AA	10	512
ESC_TA8240AA	131	653
ESC_TA8239AA	2711	640
ESC_TA8238AA	569	738

ESC_TA8237AA	10	512
ESC_TA8236AA	968	737
ESC_TA8235AA	93	71
ESC_TA8234AA	69	387
ESC_TA8233AA	681	393
ESC_TA8232AA	12	626
ESC_TA8231AA	95	738
ESC_TA8230AA	162	924
ESC_TA8229AA	357	654
ESC_TA8228AA	80	731
ESC_TA8227AA	421	744
ESC_TA8226AA	11764	474
ESC_TA8225AA	95	738
ESC_TA8224AA	538	629
ESC_TA8223AA	457	595
ESC_TA8222AA	12	626
ESC_TA8221AA	457	595
ESC_TA8220AA	11766	71
ESC_TA8219AA	95	738
ESC_TA8218AA	141	644
ESC_TA8217AA	23	156
ESC_TA8216AA	127	738
ESC_TA8215AA	12	626
ESC_TA8214AA	10	512
ESC_TA8213AA	4456	654
ESC_TA8212AA	1193	761
ESC_TA8211AA	131	653
ESC_TA8210AA	28	537
ESC_TA8209AA	410	156
ESC_TA8208AA	69	387
ESC_TA8207AA	1434	512
ESC_TA8206AA	73	737
ESC_TA8205AA	404	761
ESC_TA8204AA	73	737
ESC_TA8203AA	80	731
ESC_TA8202AA	547	653
ESC_TA8201AA	69	387
ESC_TA8200AA	73	737
ESC_TA8199AA	569	738
ESC_TA8198AA	95	738
ESC_TA8197AA	372	424
ESC_TA8196AA	131	653
ESC_TA8195AA	131	653
ESC_TA8194AA	91	629
ESC_TA8193AA	141	644
ESC_TA8192AA	491	626
ESC_TA8191AA	11771	76
ESC_TA8190AA	11770	24
ESC_TA8189AA	2015	684
ESC_TA8188AA	69	387

ESC_TA8187AA	95	738
ESC_TA8186AA	681	393
ESC_TA8185AA	95	738
ESC_TA8184AA	58	198
ESC_TA8183AA	127	738
ESC_TA8182AA	73	737
ESC_TA8181AA	69	387
ESC_TA8180AA	69	387
ESC_TA8179AA	69	387
ESC_TA8178AA	7341	655
ESC_TA8177AA	58	198
ESC_TA8176AA	141	644
ESC_TA8175AA	141	644
ESC_TA8174AA	95	738
ESC_TA8173AA	95	738
ESC_TA8172AA	10	512
ESC_TA8171AA	127	738
ESC_TA8170AA	12	626
ESC_TA8169AA	1170	629
ESC_TA8168AA	80	731
ESC_TA8167AA	73	737
ESC_TA8166AA	73	737
ESC_TA8165AA	625	756
ESC_TA8164AA	73	737
ESC_TA8163AA	349	670
ESC_TA8162AA	73	737
ESC_TA8161AA	131	653
ESC_TA8160AA	1246	198
ESC_TA8159AA	1249	257
ESC_TA8158AA	88	160
ESC_TA8157AA	372	424
ESC_TA8156AA	62	574
ESC_TA8155AA	58	198
ESC_TA8154AA	117	592
ESC_TA8153AA	73	737
ESC_TA8152AA	104	737
ESC_TA8151AA	7340	629
ESC_TA8150AA	357	654
ESC_TA8149AA	1193	761
ESC_TA8148AA	636	653
ESC_TA8147AA	1056	257
ESC_TA8146AA	10	512
ESC_TA8145AA	40	198
ESC_TA8144AA	69	387
ESC_TA8143AA	73	737
ESC_TA8142AA	11777	734
ESC_TA8141AA	95	738
ESC_TA8140AA	12	626
ESC_TA8139AA	62	574
ESC_TA8138AA	357	654

ESC_TA8137AA	6375	737
ESC_TA8136AA	1670	438
ESC_TA8135AA	131	653
ESC_TA8134AA	80	731
ESC_TA8133AA	12	626
ESC_TA8132AA	3352	626
ESC_TA8131AA	28	537
ESC_TA8130AA	162	924
ESC_TA8129AA	95	738
ESC_TA8128AA	73	737
ESC_TA8127AA	11776	654
ESC_TA8126AA	69	387
ESC_TA8125AA	141	644
ESC_TA8124AA	69	387
ESC_TA8123AA	69	387
ESC_TA8122AA	12	626
ESC_TA8121AA	131	653
ESC_TA8120AA	405	923
ESC_TA8119AA	73	737
ESC_TA8118AA	1385	653
ESC_TA8117AA	80	731
ESC_TA8116AA	58	198
ESC_TA8115AA	69	387
ESC_TA8114AA	73	737
ESC_TA8113AA	140	738
ESC_TA8112AA	349	670
ESC_TA8111AA	69	387
ESC_TA8110AA	95	738
ESC_TA8109AA	38	669
ESC_TA8108AA	543	842
ESC_TA8107AA	58	198
ESC_TA8106AA	372	424
ESC_TA8105AA	69	387
ESC_TA8104AA	14	761
ESC_TA8103AA	80	731
ESC_TA8102AA	2959	914
ESC_TA8101AA	3672	649
ESC_TA8100AA	1334	627
ESC_TA8099AA	155	175
ESC_TA8098AA	1057	761
ESC_TA8097AA	11028	198
ESC_TA8096AA	14	761
ESC_TA8095AA	69	387
ESC_TA8094AA	69	387
ESC_TA8093AA	963	669
ESC_TA8092AA	95	738
ESC_TA8091AA	744	512
ESC_TA8090AA	11774	738
ESC_TA8089AA	144	740
ESC_TA8088AA	5784	448

ESC_TA8087AA	12	626
ESC_TA8086AA	1193	761
ESC_TA8085AA	1193	761
ESC_TA8084AA	537	756
ESC_TA8083AA	567	740
ESC_TA8082AA	126	740
ESC_TA8081AA	1486	71
ESC_TA8080AA	58	198
ESC_TA8079AA	73	737
ESC_TA8078AA	14	761
ESC_TA8077AA	1429	198
ESC_TA8076AA	80	731
ESC_TA8075AA	59	217
ESC_TA8074AA	141	644
ESC_TA8073AA	1670	438
ESC_TA8072AA	141	644
ESC_TA8071AA	131	653
ESC_TA8070AA	69	387
ESC_TA8069AA	95	738
ESC_TA8068AA	135	653
ESC_TA8067AA	5784	448
ESC_TA8066AA	10	512
ESC_TA8065AA	38	669
ESC_TA8064AA	567	740
ESC_TA8063AA	83	740
ESC_TA8062AA	88	160
ESC_TA8061AA	11773	737
ESC_TA8060AA	11772	156
ESC_TA8059AA	69	387
ESC_TA8058AA	8588	257
ESC_TA8057AA	95	738
ESC_TA8056AA	69	387
ESC_TA8055AA	131	653
ESC_TA8054AA	297	234
ESC_TA8053AA	80	731
ESC_TA8052AA	69	387
ESC_TA8051AA	357	654
ESC_TA8050AA	131	653
ESC_TA8049AA	69	387
ESC_TA8048AA	141	644
ESC_TA8047AA	362	234
ESC_TA8046AA	1434	512
ESC_TA8045AA	73	737
ESC_TA8044AA	569	738
ESC_TA8043AA	73	737
ESC_TA8042AA	2040	198
ESC_TA8041AA	33	198
ESC_TA8040AA	33	198
ESC_TA8039AA	33	198
ESC_TA8038AA	33	198

ESC_TA8037AA	33	198
ESC_TA8036AA	33	198
ESC_TA8035AA	1434	512
ESC_TA8034AA	12	626
ESC_TA8033AA	349	670
ESC_TA8032AA	69	387
ESC_TA8031AA	135	653
ESC_TA8030AA	404	761
ESC_TA8029AA	162	924
ESC_TA8028AA	131	653
ESC_TA8027AA	10	512
ESC_TA8026AA	69	387
ESC_TA8025AA	135	653
ESC_TA8024AA	648	359
ESC_TA8023AA	2013	725
ESC_TA8022AA	1193	761
ESC_TA8021AA	567	740
ESC_TA8020AA	355	764
ESC_TA8019AA	141	644
ESC_TA8018AA	69	387
ESC_TA8017AA	101	696
ESC_TA8016AA	69	387
ESC_TA8015AA	70	670
ESC_TA8014AA	2013	725
ESC_TA8013AA	453	935
ESC_TA8012AA	8125	512
ESC_TA8011AA	10	512
ESC_TA8010AA	2015	684
ESC_TA8009AA	155	175
ESC_TA8008AA	95	738
ESC_TA8007AA	95	738
ESC_TA8006AA	753	842
ESC_TA8005AA	80	731
ESC_TA8004AA	624	359
ESC_TA8003AA	80	731
ESC_TA8001AA	5617	204
ESC_TA8000AA	11771	76
ESC_TA7999AA	1727	257
ESC_TA7998AA	73	737
ESC_TA7997AA	968	737
ESC_TA7996AA	131	653
ESC_TA7995AA	69	387
ESC_TA7994AA	88	160
ESC_TA7993AA	1122	307
ESC_TA7992AA	335	936
ESC_TA7991AA	1147	198
ESC_TA7990AA	131	653
ESC_TA7989AA	617	511
ESC_TA7988AA	1777	198
ESC_TA7987AA	73	737

ESC_TA7986AA	73	737
ESC_TA7985AA	91	629
ESC_TA7983AA	69	387
ESC_TA7982AA	11775	725
ESC_TA7981AA	155	175
ESC_TA7980AA	73	737
ESC_TA7979AA	11806	738
ESC_TA7978AA	131	653
ESC_TA7977AA	1314	71
ESC_TA7976AA	648	359
ESC_TA7975AA	69	387
ESC_TA7974AA	95	738
ESC_TA7973AA	73	737
ESC_TA7972AA	10	512
ESC_TA7971AA	14	761
ESC_TA7969AA	10	512
ESC_TA7968AA	131	653
ESC_TA7967AA	968	737
ESC_TA7966AA	73	737
ESC_TA7965AA	117	592
ESC_TA7964AA	95	738
ESC_TA7963AA	1057	761
ESC_TA7962AA	95	738
ESC_TA7961AA	95	738
ESC_TA7960AA	1170	629
ESC_TA7959AA	540	286
ESC_TA7958AA	117	592
ESC_TA7957AA	404	761
ESC_TA7956AA	394	400
ESC_TA7955AA	10	512
ESC_TA7954AA	219	877
ESC_TA7953AA	1193	761
ESC_TA7952AA	95	738
ESC_TA7951AA	453	935
ESC_TA7950AA	998	644
ESC_TA7949AA	141	644
ESC_TA7948AA	416	738
ESC_TA7947AA	38	669
ESC_TA7946AA	131	653
ESC_TA7945AA	73	737
ESC_TA7944AA	135	653
ESC_TA7943AA	428	654
ESC_TA7942AA	5274	737
ESC_TA7941AA	69	387
ESC_TA7940AA	95	738
ESC_TA7939AA	95	738
ESC_TA7938AA	95	738
ESC_TA7937AA	11778	912
ESC_TA7936AA	706	422
ESC_TA7935AA	354	15

ESC_TA7934AA	10	512
ESC_TA7933AA	1193	761
ESC_TA7932AA	69	387
ESC_TA7931AA	141	644
ESC_TA7930AA	131	653
ESC_TA7929AA	491	626
ESC_TA7928AA	1276	241
ESC_TA7927AA	10	512
ESC_TA7926AA	14	761
ESC_TA7925AA	131	653
ESC_TA7924AA	91	629
ESC_TA7923AA	88	160
ESC_TA7922AA	13	257
ESC_TA7921AA	301	307
ESC_TA7920AA	301	307
ESC_TA7919AA	301	307
ESC_TA7918AA	301	307
ESC_TA7917AA	8691	118
ESC_TA7916AA	13	257
ESC_TA7915AA	767	198
ESC_TA7914AA	301	307
ESC_TA7913AA	656	789
ESC_TA7912AA	13	257
ESC_TA7911AA	301	307
ESC_TA7910AA	301	307
ESC_TA7909AA	301	307
ESC_TA7908AA	301	307
ESC_TA7907AA	88	160
ESC_TA7906AA	88	160
ESC_TA7905AA	301	307
ESC_TA7904AA	301	307
ESC_TA7903AA	33	198
ESC_TA7902AA	301	307
ESC_TA7901AA	301	307
ESC_TA7900AA	206	286
ESC_TA7899AA	206	286
ESC_TA7898AA	10	512
ESC_TA7897AA	4450	844
ESC_TA7896AA	3858	935
ESC_TA7895AA	131	653
ESC_TA7894AA	354	15
ESC_TA7893AA	131	653
ESC_TA7892AA	73	737
ESC_TA7891AA	73	737
ESC_TA7890AA	410	156
ESC_TA7889AA	648	359
ESC_TA7888AA	131	653
ESC_TA7887AA	131	653
ESC_TA7886AA	131	653
ESC_TA7885AA	710	291

ESC_TA7884AA	405	923
ESC_TA7883AA	131	653
ESC_TA7882AA	131	653
ESC_TA7881AA	131	653
ESC_TA7880AA	131	653
ESC_TA7879AA	131	653
ESC_TA7878AA	131	653
ESC_TA7877AA	131	653
ESC_TA7876AA	38	669
ESC_TA7875AA	131	653
ESC_TA7874AA	131	653
ESC_TA7873AA	88	160
ESC_TA7872AA	73	737
ESC_TA7871AA	69	387
ESC_TA7870AA	69	387
ESC_TA7869AA	10	512
ESC_TA7868AA	131	653
ESC_TA7867AA	73	737
ESC_TA7866AA	73	737
ESC_TA7865AA	131	653
ESC_TA7864AA	155	175
ESC_TA7863AA	95	738
ESC_TA7862AA	131	653
ESC_TA7861AA	38	669
ESC_TA7860AA	155	175
ESC_TA7859AA	1323	405
ESC_TA7858AA	93	71
ESC_TA7857AA	73	737
ESC_TA7856AA	73	737
ESC_TA7855AA	73	737
ESC_TA7854AA	378	504
ESC_TA7853AA	73	737
ESC_TA7852AA	349	670
ESC_TA7851AA	1638	519
ESC_TA7850AA	80	731
ESC_TA7849AA	216	512
ESC_TA7848AA	58	198
ESC_TA7847AA	537	756
ESC_TA7846AA	95	738
ESC_TA7845AA	59	217
ESC_TA7844AA	69	387
ESC_TA7843AA	69	387
ESC_TA7842AA	648	359
ESC_TA7841AA	95	738
ESC_TA7840AA	80	731
ESC_TA7839AA	131	653
ESC_TA7838AA	404	761
ESC_TA7837AA	131	653
ESC_TA7836AA	8158	372
ESC_TA7835AA	362	234

ESC_TA7834AA	1057	761
ESC_TA7833AA	648	359
ESC_TA7832AA	10	512
ESC_TA7831AA	216	512
ESC_TA7830AA	127	738
ESC_TA7829AA	95	738
ESC_TA7828AA	648	359
ESC_TA7827AA	10	512
ESC_TA7826AA	131	653
ESC_TA7825AA	1308	257
ESC_TA7824AA	69	387
ESC_TA7823AA	744	512
ESC_TA7822AA	95	738
ESC_TA7821AA	69	387
ESC_TA7820AA	216	512
ESC_TA7819AA	428	654
ESC_TA7818AA	847	257
ESC_TA7816AA	69	387
ESC_TA7815AA	69	387
ESC_TA7814AA	10	512
ESC_TA7813AA	127	738
ESC_TA7812AA	12	626
ESC_TA7811AA	69	387
ESC_TA7810AA	141	644
ESC_TA7809AA	127	738
ESC_TA7808AA	10898	453
ESC_TA7807AA	104	737
ESC_TA7806AA	80	731
ESC_TA7805AA	73	737
ESC_TA7804AA	120	180
ESC_TA7803AA	773	71
ESC_TA7802AA	1193	761
ESC_TA7801AA	117	592
ESC_TA7800AA	58	198
ESC_TA7799AA	73	737
ESC_TA7798AA	12	626
ESC_TA7797AA	1193	761
ESC_TA7796AA	69	387
ESC_TA7795AA	69	387
ESC_TA7794AA	59	217
ESC_TA7793AA	567	740
ESC_TA7792AA	69	387
ESC_TA7791AA	73	737
ESC_TA7790AA	58	198
ESC_TA7789AA	73	737
ESC_TA7788AA	1723	900
ESC_TA7787AA	73	737
ESC_TA7786AA	131	653
ESC_TA7785AA	448	257
ESC_TA7784AA	127	738

ESC_TA7783AA	69	387
ESC_TA7782AA	73	737
ESC_TA7781AA	1429	198
ESC_TA7780AA	1434	512
ESC_TA7779AA	10	512
ESC_TA7778AA	73	737
ESC_TA7777AA	131	653
ESC_TA7776AA	795	924
ESC_TA7775AA	73	737
ESC_TA7774AA	405	923
ESC_TA7773AA	69	387
ESC_TA7772AA	38	669
ESC_TA7771AA	11759	923
ESC_TA7770AA	1236	380
ESC_TA7769AA	538	629
ESC_TA7768AA	1858	757
ESC_TA7767AA	73	737
ESC_TA7766AA	38	669
ESC_TA7765AA	131	653
ESC_TA7764AA	2079	198
ESC_TA7763AA	69	387
ESC_TA7762AA	683	175
ESC_TA7761AA	91	629
ESC_TA7760AA	141	644
ESC_TA7759AA	2013	725
ESC_TA7758AA	88	160
ESC_TA7757AA	2015	684
ESC_TA7756AA	393	343
ESC_TA7755AA	1092	739
ESC_TA7754AA	388	156
ESC_TA7753AA	95	738
ESC_TA7752AA	73	737
ESC_TA7751AA	7011	512
ESC_TA7750AA	131	653
ESC_TA7749AA	11758	626
ESC_TA7748AA	69	387
ESC_TA7747AA	95	738
ESC_TA7746AA	567	740
ESC_TA7745AA	761	512
ESC_TA7744AA	357	654
ESC_TA7743AA	349	670
ESC_TA7742AA	73	737
ESC_TA7741AA	70	670
ESC_TA7740AA	11757	512
ESC_TA7739AA	80	731
ESC_TA7738AA	83	740
ESC_TA7737AA	131	653
ESC_TA7736AA	69	387
ESC_TA7735AA	33	198
ESC_TA7734AA	10	512

ESC_TA7733AA	73	737
ESC_TA7732AA	973	968
ESC_TA7731AA	162	924
ESC_TA7730AA	83	740
ESC_TA7729AA	95	738
ESC_TA7728AA	1828	738
ESC_TA7727AA	5936	662
ESC_TA7726AA	38	669
ESC_TA7725AA	1193	761
ESC_TA7724AA	69	387
ESC_TA7723AA	180	234
ESC_TA7722AA	648	359
ESC_TA7721AA	14	761
ESC_TA7720AA	73	737
ESC_TA7719AA	73	737
ESC_TA7718AA	405	923
ESC_TA7717AA	12	626
ESC_TA7716AA	69	387
ESC_TA7715AA	131	653
ESC_TA7714AA	448	257
ESC_TA7713AA	95	738
ESC_TA7712AA	968	737
ESC_TA7711AA	3568	801
ESC_TA7710AA	25	234
ESC_TA7709AA	12	626
ESC_TA7708AA	73	737
ESC_TA7707AA	95	738
ESC_TA7706AA	131	653
ESC_TA7705AA	357	654
ESC_TA7704AA	141	644
ESC_TA7703AA	8611	422
ESC_TA7701AA	1	667
ESC_TA7700AA	88	160
ESC_TA7699AA	10	512
ESC_TA7698AA	2705	504
ESC_TA7697AA	10	512
ESC_TA7696AA	1882	387
ESC_TA7695AA	457	595
ESC_TA7694AA	752	512
ESC_TA7693AA	10	512
ESC_TA7692AA	10	512
ESC_TA7691AA	10	512
ESC_TA7690AA	10	512
ESC_TA7689AA	5825	924
ESC_TA7688AA	117	592
ESC_TA7687AA	155	175
ESC_TA7686AA	48	76
ESC_TA7685AA	354	15
ESC_TA7684AA	354	15
ESC_TA7683AA	410	156

ESC_TA7682AA	6753	76
ESC_TA7681AA	57	50
ESC_TA7680AA	359	696
ESC_TA7679AA	57	50
ESC_TA7678AA	38	669
ESC_TA7677AA	10	512
ESC_TA7676AA	117	592
ESC_TA7675AA	57	50
ESC_TA7674AA	117	592
ESC_TA7673AA	1196	257
ESC_TA7672AA	624	359
ESC_TA7671AA	1011	198
ESC_TA7670AA	1011	198
ESC_TA7669AA	10	512
ESC_TA7668AA	93	71
ESC_TA7667AA	1771	868
ESC_TA7666AA	93	71
ESC_TA7665AA	48	76
ESC_TA7664AA	359	696
ESC_TA7663AA	1771	868
ESC_TA7662AA	48	76
ESC_TA7661AA	117	592
ESC_TA7660AA	1684	504
ESC_TA7659AA	1140	934
ESC_TA7658AA	4937	519
ESC_TA7657AA	1771	868
ESC_TA7656AA	1771	868
ESC_TA7655AA	8234	512
ESC_TA7654AA	1771	868
ESC_TA7653AA	93	71
ESC_TA7652AA	162	924
ESC_TA7651AA	4937	519
ESC_TA7650AA	48	76
ESC_TA7649AA	1276	241
ESC_TA7648AA	2040	198
ESC_TA7647AA	117	592
ESC_TA7646AA	1112	504
ESC_TA7645AA	93	71
ESC_TA7644AA	155	175
ESC_TA7643AA	359	696
ESC_TA7642AA	1140	934
ESC_TA7641AA	997	915
ESC_TA7640AA	1286	474
ESC_TA7639AA	359	696
ESC_TA7638AA	155	175
ESC_TA7637AA	1101	842
ESC_TA7636AA	354	15
ESC_TA7635AA	359	696
ESC_TA7634AA	1286	474
ESC_TA7633AA	1140	934

ESC_TA7632AA	359	696
ESC_TA7631AA	359	696
ESC_TA7630AA	1011	198
ESC_TA7629AA	93	71
ESC_TA7628AA	359	696
ESC_TA7627AA	2509	257
ESC_TA7626AA	359	696
ESC_TA7625AA	359	696
ESC_TA7624AA	1011	198
ESC_TA7623AA	155	175
ESC_TA7622AA	1140	934
ESC_TA7621AA	1011	198
ESC_TA7620AA	1101	842
ESC_TA7619AA	57	50
ESC_TA7618AA	1011	198
ESC_TA7617AA	155	175
ESC_TA7616AA	354	15
ESC_TA7615AA	359	696
ESC_TA7614AA	1101	842
ESC_TA7613AA	1101	842
ESC_TA7612AA	359	696
ESC_TA7611AA	354	15
ESC_TA7610AA	1011	198
ESC_TA7609AA	359	696
ESC_TA7608AA	1011	198
ESC_TA7607AA	1101	842
ESC_TA7606AA	1140	934
ESC_TA7605AA	1101	842
ESC_TA7604AA	1140	934
ESC_TA7603AA	359	696
ESC_TA7602AA	359	696
ESC_TA7601AA	155	175
ESC_TA7600AA	93	71
ESC_TA7599AA	1140	934
ESC_TA7598AA	155	175
ESC_TA7597AA	354	15
ESC_TA7596AA	1011	198
ESC_TA7595AA	1771	868
ESC_TA7594AA	354	15
ESC_TA7593AA	354	15
ESC_TA7592AA	359	696
ESC_TA7591AA	1011	198
ESC_TA7590AA	1011	198
ESC_TA7589AA	359	696
ESC_TA7588AA	156	175
ESC_TA7587AA	3994	512
ESC_TA7586AA	3994	512
ESC_TA7585AA	1196	257
ESC_TA7584AA	3640	257
ESC_TA7583AA	1011	198

ESC_TA7582AA	453	935
ESC_TA7581AA	453	935
ESC_TA7580AA	1196	257
ESC_TA7579AA	156	175
ESC_TA7578AA	57	50
ESC_TA7576AA	69	387
ESC_TA7575AA	86	935
ESC_TA7574AA	23	156
ESC_TA7573AA	10	512
ESC_TA7572AA	1	667
ESC_TA7571AA	10	512
ESC_TA7570AA	10	512
ESC_TA7569AA	1	667
ESC_TA7568AA	1	667
ESC_TA7567AA	118	985
ESC_TA7566AA	4379	878
ESC_TA7565AA	846	915
ESC_TA7564AA	4398	924
ESC_TA7563AA	846	915
ESC_TA7562AA	4398	924
ESC_TA7561AA	10	512
ESC_TA7560AA	4398	924
ESC_TA7559AA	2509	257
ESC_TA7558AA	2509	257
ESC_TA7557AA	2509	257
ESC_TA7556AA	1011	198
ESC_TA7555AA	345	175
ESC_TA7554AA	5281	886
ESC_TA7553AA	410	156
ESC_TA7552AA	2067	257
ESC_TA7551AA	2067	257
ESC_TA7550AA	2067	257
ESC_TA7549AA	342	286
ESC_TA7548AA	4398	924
ESC_TA7547AA	2509	257
ESC_TA7546AA	997	915
ESC_TA7545AA	10	512
ESC_TA7544AA	10	512
ESC_TA7543AA	1140	934
ESC_TA7542AA	1011	198
ESC_TA7541AA	2067	257
ESC_TA7540AA	4398	924
ESC_TA7539AA	4398	924
ESC_TA7538AA	4398	924
ESC_TA7537AA	846	915
ESC_TA7536AA	4398	924
ESC_TA7535AA	744	512
ESC_TA7534AA	10	512
ESC_TA7533AA	33	198
ESC_TA7532AA	11	936

ESC_TA7531AA	200	198
ESC_TA7530AA	88	160
ESC_TA7529AA	4748	234
ESC_TA7528AA	11	936
ESC_TA7527AA	4175	270
ESC_TA7526AA	2165	257
ESC_TA7525AA	9703	99
ESC_TA7524AA	1083	203
ESC_TA7523AA	4038	257
ESC_TA7522AA	2958	257
ESC_TA7521AA	2178	924
ESC_TA7520AA	11722	261
ESC_TA7519AA	2178	924
ESC_TA7518AA	152	257
ESC_TA7517AA	152	257
ESC_TA7516AA	152	257
ESC_TA7515AA	152	257
ESC_TA7514AA	152	257
ESC_TA7513AA	152	257
ESC_TA7512AA	245	261
ESC_TA7511AA	245	261
ESC_TA7510AA	152	257
ESC_TA7509AA	152	257
ESC_TA7508AA	152	257
ESC_TA7507AA	152	257
ESC_TA7506AA	152	257
ESC_TA7505AA	152	257
ESC_TA7504AA	245	261
ESC_TA7503AA	245	261
ESC_TA7502AA	245	261
ESC_TA7501AA	245	261
ESC_TA7500AA	245	261
ESC_TA7499AA	245	261
ESC_TA7498AA	245	261
ESC_TA7497AA	245	261
ESC_TA7496AA	245	261
ESC_TA7495AA	245	261
ESC_TA7494AA	245	261
ESC_TA7493AA	245	261
ESC_TA7492AA	245	261
ESC_TA7491AA	245	261
ESC_TA7490AA	9352	141
ESC_TA7489AA	152	257
ESC_TA7488AA	245	261
ESC_TA7487AA	245	261
ESC_TA7486AA	152	257
ESC_TA7485AA	245	261
ESC_TA7484AA	152	257
ESC_TA7483AA	245	261
ESC_TA7482AA	245	261

ESC_TA7481AA	152	257
ESC_TA7480AA	152	257
ESC_TA7479AA	245	261
ESC_TA7478AA	152	257
ESC_TA7477AA	152	257
ESC_TA7476AA	245	261
ESC_TA7475AA	152	257
ESC_TA7474AA	152	257
ESC_TA7473AA	152	257
ESC_TA7471AA	152	257
ESC_TA7469AA	152	257
ESC_TA7468AA	152	257
ESC_TA7467AA	245	261
ESC_TA7466AA	152	257
ESC_TA7465AA	245	261
ESC_TA7464AA	152	257
ESC_TA7463AA	152	257
ESC_TA7461AA	152	257
ESC_TA7460AA	245	261
ESC_TA7459AA	245	261
ESC_TA7458AA	152	257
ESC_TA7456AA	11721	257
ESC_TA7455AA	152	257
ESC_TA7454AA	245	261
ESC_TA7453AA	152	257
ESC_TA7452AA	1025	261
ESC_TA7451AA	152	257
ESC_TA7450AA	152	257
ESC_TA7449AA	152	257
ESC_TA7448AA	152	257
ESC_TA7447AA	152	257
ESC_TA7446AA	245	261
ESC_TA7445AA	2280	212
ESC_TA7444AA	117	592
ESC_TA7443AA	58	198
ESC_TA7442AA	714	318
ESC_TA7441AA	714	318
ESC_TA7440AA	7006	936
ESC_TA7439AA	9701	261
ESC_TA7438AA	301	307
ESC_TA7437AA	117	592
ESC_TA7436AA	2165	257
ESC_TA7435AA	9702	53
ESC_TA7434AA	2063	198
ESC_TA7433AA	2144	257
ESC_TA7432AA	226	512
ESC_TA7431AA	69	387
ESC_TA7430AA	2309	669
ESC_TA7429AA	48	76
ESC_TA7427AA	48	76

ESC_TA7426AA	4589	924
ESC_TA7425AA	10	512
ESC_TA7424AA	542	824
ESC_TA7423AA	226	512
ESC_TA7422AA	295	160
ESC_TA7421AA	115	669
ESC_TA7420AA	2732	234
ESC_TA7419AA	6256	76
ESC_TA7418AA	898	108
ESC_TA7417AA	542	824
ESC_TA7416AA	2973	234
ESC_TA7415AA	3634	71
ESC_TA7414AA	295	160
ESC_TA7413AA	117	592
ESC_TA7412AA	1266	210
ESC_TA7411AA	540	286
ESC_TA7410AA	189	504
ESC_TA7409AA	730	445
ESC_TA7408AA	115	669
ESC_TA7407AA	117	592
ESC_TA7406AA	1114	504
ESC_TA7405AA	5766	198
ESC_TA7404AA	1421	447
ESC_TA7403AA	3274	512
ESC_TA7402AA	542	824
ESC_TA7401AA	846	915
ESC_TA7400AA	117	592
ESC_TA7399AA	212	198
ESC_TA7398AA	8165	111
ESC_TA7397AA	3274	512
ESC_TA7396AA	117	592
ESC_TA7395AA	11747	234
ESC_TA7394AA	117	592
ESC_TA7393AA	189	504
ESC_TA7392AA	453	935
ESC_TA7391AA	2973	234
ESC_TA7390AA	117	592
ESC_TA7389AA	398	445
ESC_TA7388AA	2325	447
ESC_TA7387AA	1072	355
ESC_TA7386AA	117	592
ESC_TA7385AA	117	592
ESC_TA7384AA	1244	291
ESC_TA7383AA	156	175
ESC_TA7382AA	1607	844
ESC_TA7381AA	115	669
ESC_TA7380AA	1158	387
ESC_TA7379AA	2309	669
ESC_TA7378AA	212	198
ESC_TA7377AA	3258	592

ESC_TA7376AA	93	71
ESC_TA7375AA	11746	935
ESC_TA7374AA	48	76
ESC_TA7373AA	117	592
ESC_TA7372AA	189	504
ESC_TA7371AA	117	592
ESC_TA7370AA	117	592
ESC_TA7369AA	48	76
ESC_TA7368AA	155	175
ESC_TA7367AA	117	592
ESC_TA7366AA	752	512
ESC_TA7365AA	11745	587
ESC_TA7364AA	23	156
ESC_TA7363AA	212	198
ESC_TA7362AA	1158	387
ESC_TA7361AA	117	592
ESC_TA7360AA	11744	408
ESC_TA7359AA	117	592
ESC_TA7358AA	117	592
ESC_TA7357AA	162	924
ESC_TA7356AA	2973	234
ESC_TA7355AA	115	669
ESC_TA7354AA	156	175
ESC_TA7353AA	654	257
ESC_TA7352AA	11743	257
ESC_TA7351AA	4937	519
ESC_TA7350AA	354	15
ESC_TA7349AA	3776	175
ESC_TA7348AA	155	175
ESC_TA7347AA	162	924
ESC_TA7346AA	349	670
ESC_TA7345AA	295	160
ESC_TA7344AA	349	670
ESC_TA7343AA	345	175
ESC_TA7342AA	11754	504
ESC_TA7341AA	1114	504
ESC_TA7340AA	101	696
ESC_TA7339AA	3776	175
ESC_TA7338AA	34	504
ESC_TA7337AA	48	76
ESC_TA7336AA	6697	286
ESC_TA7335AA	48	76
ESC_TA7334AA	602	257
ESC_TA7333AA	752	512
ESC_TA7332AA	93	71
ESC_TA7331AA	398	445
ESC_TA7330AA	117	592
ESC_TA7329AA	654	257
ESC_TA7328AA	48	76
ESC_TA7327AA	871	447

ESC_TA7326AA	3776	175
ESC_TA7325AA	1158	387
ESC_TA7324AA	227	504
ESC_TA7323AA	57	50
ESC_TA7322AA	2223	76
ESC_TA7321AA	3014	410
ESC_TA7320AA	23	156
ESC_TA7319AA	1158	387
ESC_TA7318AA	345	175
ESC_TA7317AA	156	175
ESC_TA7316AA	117	592
ESC_TA7315AA	2309	669
ESC_TA7314AA	156	175
ESC_TA7312AA	58	198
ESC_TA7311AA	57	50
ESC_TA7310AA	3258	592
ESC_TA7309AA	3776	175
ESC_TA7308AA	457	595
ESC_TA7307AA	155	175
ESC_TA7306AA	117	592
ESC_TA7305AA	115	669
ESC_TA7304AA	3776	175
ESC_TA7303AA	38	669
ESC_TA7302AA	1011	198
ESC_TA7301AA	3634	71
ESC_TA7300AA	2973	234
ESC_TA7299AA	3258	592
ESC_TA7298AA	362	234
ESC_TA7297AA	2223	76
ESC_TA7296AA	602	257
ESC_TA7295AA	34	504
ESC_TA7294AA	752	512
ESC_TA7293AA	117	592
ESC_TA7292AA	117	592
ESC_TA7291AA	155	175
ESC_TA7290AA	351	268
ESC_TA7289AA	9578	512
ESC_TA7288AA	206	286
ESC_TA7287AA	3776	175
ESC_TA7286AA	88	160
ESC_TA7285AA	11753	288
ESC_TA7284AA	117	592
ESC_TA7283AA	10	512
ESC_TA7282AA	3576	175
ESC_TA7281AA	117	592
ESC_TA7280AA	117	592
ESC_TA7279AA	206	286
ESC_TA7278AA	3776	175
ESC_TA7277AA	871	447
ESC_TA7276AA	11752	512

ESC_TA7275AA	218	519
ESC_TA7274AA	48	76
ESC_TA7273AA	11751	175
ESC_TA7272AA	1551	40
ESC_TA7271AA	117	592
ESC_TA7270AA	10064	711
ESC_TA7269AA	88	160
ESC_TA7268AA	394	400
ESC_TA7267AA	7506	198
ESC_TA7266AA	4450	844
ESC_TA7265AA	11750	257
ESC_TA7264AA	11750	257
ESC_TA7263AA	93	71
ESC_TA7262AA	11749	592
ESC_TA7261AA	11749	592
ESC_TA7260AA	8261	592
ESC_TA7259AA	654	257
ESC_TA7258AA	117	592
ESC_TA7257AA	155	175
ESC_TA7256AA	156	175
ESC_TA7255AA	162	924
ESC_TA7254AA	155	175
ESC_TA7253AA	398	445
ESC_TA7252AA	117	592
ESC_TA7251AA	10	512
ESC_TA7250AA	101	696
ESC_TA7249AA	117	592
ESC_TA7248AA	191	198
ESC_TA7247AA	654	257
ESC_TA7246AA	746	789
ESC_TA7245AA	46	447
ESC_TA7244AA	117	592
ESC_TA7243AA	117	592
ESC_TA7242AA	10	512
ESC_TA7241AA	58	198
ESC_TA7240AA	11748	447
ESC_TA7239AA	542	824
ESC_TA7238AA	48	76
ESC_TA7237AA	457	595
ESC_TA7236AA	117	592
ESC_TA7235AA	156	175
ESC_TA7234AA	117	592
ESC_TA7233AA	2614	975
ESC_TA7232AA	58	198
ESC_TA7231AA	117	592
ESC_TA7230AA	11755	286
ESC_TA7229AA	654	257
ESC_TA7228AA	10	512
ESC_TA7227AA	34	504
ESC_TA7226AA	227	504

ESC_TA7225AA	1730	234
ESC_TA7224AA	654	257
ESC_TA7223AA	48	76
ESC_TA7222AA	3580	257
ESC_TA7221AA	155	175
ESC_TA7220AA	345	175
ESC_TA7219AA	2309	669
ESC_TA7218AA	295	160
ESC_TA7216AA	654	257
ESC_TA7215AA	746	789
ESC_TA7214AA	117	592
ESC_TA7213AA	23	156
ESC_TA7212AA	155	175
ESC_TA7211AA	345	175
ESC_TA7210AA	156	175
ESC_TA7209AA	2179	924
ESC_TA7208AA	117	592
ESC_TA7207AA	117	592
ESC_TA7206AA	58	198
ESC_TA7205AA	410	156
ESC_TA7204AA	410	156
ESC_TA7203AA	11740	175
ESC_TA7202AA	167	512
ESC_TA7201AA	167	512
ESC_TA7200AA	136	392
ESC_TA7199AA	73	737
ESC_TA7198AA	73	737
ESC_TA7197AA	131	653
ESC_TA7196AA	648	359
ESC_TA7195AA	69	387
ESC_TA7194AA	11717	175
ESC_TA7193AA	131	653
ESC_TA7192AA	155	175
ESC_TA7191AA	1193	761
ESC_TA7190AA	38	669
ESC_TA7189AA	145	256
ESC_TA7188AA	11389	261
ESC_TA7187AA	11389	261
ESC_TA7185AA	11	936
ESC_TA7184AA	11	936
ESC_TA7183AA	17	191
ESC_TA7182AA	11727	504
ESC_TA7181AA	8427	257
ESC_TA7180AA	8427	257
ESC_TA7179AA	93	71
ESC_TA7178AA	11704	512
ESC_TA7177AA	152	257
ESC_TA7176AA	245	261
ESC_TA7175AA	1025	261
ESC_TA7174AA	8515	261

ESC_TA7173AA	245	261
ESC_TA7172AA	25	234
ESC_TA7171AA	442	257
ESC_TA7170AA	25	234
ESC_TA7169AA	11724	217
ESC_TA7168AA	11723	156
ESC_TA7167AA	6880	726
ESC_TA7166AA	21	156
ESC_TA7165AA	33	198
ESC_TA7164AA	10	512
ESC_TA7163AA	10	512
ESC_TA7162AA	33	198
ESC_TA7161AA	677	257
ESC_TA7160AA	182	246
ESC_TA7159AA	1092	739
ESC_TA7158AA	10	512
ESC_TA7155AA	101	696
ESC_TA7154AA	761	512
ESC_TA7152AA	10	512
ESC_TA7151AA	46	447
ESC_TA7148AA	1408	445
ESC_TA7145AA	34	504
ESC_TA7143AA	48	76
ESC_TA7140AA	877	935
ESC_TA7139AA	877	935
ESC_TA7132AA	48	76
ESC_TA7131AA	101	696
ESC_TA7130AA	101	696
ESC_TA7129AA	410	156
ESC_TA7128AA	410	156
ESC_TA7127AA	410	156
ESC_TA7126AA	410	156
ESC_TA7125AA	453	935
ESC_TA7124AA	152	257
ESC_TA7121AA	394	400
ESC_TA7120AA	607	355
ESC_TA7118AA	1237	447
ESC_TA7117AA	1434	512
ESC_TA7116AA	1721	311
ESC_TA7115AA	1434	512
ESC_TA7114AA	10	512
ESC_TA7112AA	3168	261
ESC_TA7111AA	25	234
ESC_TA7110AA	162	924
ESC_TA7109AA	1877	773
ESC_TA7108AA	10	512
ESC_TA7107AA	131	653
ESC_TA7106AA	410	156
ESC_TA7105AA	11672	653
ESC_TA7104AA	648	359

ESC_TA7103AA	405	923
ESC_TA7102AA	410	156
ESC_TA7101AA	410	156
ESC_TA7100AA	68	386
ESC_TA7099AA	540	286
ESC_TA7098AA	399	198
ESC_TA7097AA	399	198
ESC_TA7096AA	336	878
ESC_TA7095AA	137	270
ESC_TA7094AA	409	504
ESC_TA7093AA	1720	935
ESC_TA7092AA	3580	257
ESC_TA7091AA	3580	257
ESC_TA7090AA	162	924
ESC_TA7089AA	21	156
ESC_TA7088AA	11341	156
ESC_TA7087AA	5429	156
ESC_TA7076AA	906	198
ESC_TA7075AA	11708	198
ESC_TA7074AA	3234	257
ESC_TA7073AA	10	512
ESC_TA7072AA	446	257
ESC_TA7071AA	906	198
ESC_TA7070AA	11230	512
ESC_TA7069AA	1670	438
ESC_TA7068AA	448	257
ESC_TA7067AA	10	512
ESC_TA7066AA	491	626
ESC_TA7065AA	491	626
ESC_TA7064AA	38	669
ESC_TA7063AA	141	644
ESC_TA7062AA	2622	537
ESC_TA7061AA	133	373
ESC_TA7060AA	133	373
ESC_TA7059AA	278	924
ESC_TA7058AA	162	924
ESC_TA7057AA	162	924
ESC_TA7056AA	133	373
ESC_TA7055AA	5825	924
ESC_TA7054AA	906	198
ESC_TA7053AA	641	935
ESC_TA7052AA	349	670
ESC_TA7051AA	5540	373
ESC_TA7050AA	5540	373
ESC_TA7049AA	1204	961
ESC_TA7048AA	10	512
ESC_TA7047AA	164	175
ESC_TA7046AA	501	900
ESC_TA7045AA	1308	257
ESC_TA7044AA	69	387

ESC_TA7043AA	1308	257
ESC_TA7042AA	1323	405
ESC_TA7041AA	11707	393
ESC_TA7040AA	681	393
ESC_TA7039AA	162	924
ESC_TA7038AA	10	512
ESC_TA7037AA	5976	924
ESC_TA7036AA	162	924
ESC_TA7035AA	162	924
ESC_TA7034AA	7495	333
ESC_TA7033AA	191	198
ESC_TA7032AA	11707	393
ESC_TA7031AA	6488	198
ESC_TA7030AA	1955	900
ESC_TA7029AA	58	198
ESC_TA7028AA	11707	393
ESC_TA7027AA	1727	257
ESC_TA7026AA	11710	860
ESC_TA7025AA	162	924
ESC_TA7024AA	154	257
ESC_TA7023AA	38	669
ESC_TA7022AA	154	257
ESC_TA7021AA	10	512
ESC_TA7020AA	973	968
ESC_TA7019AA	155	175
ESC_TA7018AA	4104	373
ESC_TA7017AA	3234	257
ESC_TA7016AA	441	198
ESC_TA7015AA	2028	257
ESC_TA7014AA	206	286
ESC_TA7013AA	642	924
ESC_TA7012AA	154	257
ESC_TA7011AA	154	257
ESC_TA7010AA	10	512
ESC_TA7009AA	11705	257
ESC_TA7008AA	297	234
ESC_TA7007AA	5540	373
ESC_TA7006AA	2521	257
ESC_TA7005AA	162	924
ESC_TA7004AA	2520	878
ESC_TA7003AA	11708	198
ESC_TA7002AA	2602	257
ESC_TA7001AA	11726	1034
ESC_TA7000AA	362	234
ESC_TA6999AA	10	512
ESC_TA6998AA	11707	393
ESC_TA6997AA	642	924
ESC_TA6996AA	5260	331
ESC_TA6995AA	349	670
ESC_TA6994AA	70	670

ESC_TA6993AA	446	257
ESC_TA6992AA	973	968
ESC_TA6991AA	154	257
ESC_TA6990AA	155	175
ESC_TA6989AA	216	512
ESC_TA6988AA	4429	820
ESC_TA6987AA	10987	204
ESC_TA6986AA	2602	257
ESC_TA6985AA	441	198
ESC_TA6984AA	154	257
ESC_TA6983AA	7348	331
ESC_TA6982AA	162	924
ESC_TA6981AA	362	234
ESC_TA6980AA	1011	198
ESC_TA6979AA	1286	474
ESC_TA6978AA	133	373
ESC_TA6977AA	11709	373
ESC_TA6976AA	154	257
ESC_TA6975AA	501	900
ESC_TA6974AA	351	268
ESC_TA6973AA	7495	333
ESC_TA6972AA	1386	629
ESC_TA6971AA	154	257
ESC_TA6970AA	2785	175
ESC_TA6969AA	10	512
ESC_TA6968AA	162	924
ESC_TA6967AA	410	156
ESC_TA6966AA	2602	257
ESC_TA6965AA	154	257
ESC_TA6964AA	2521	257
ESC_TA6963AA	6490	847
ESC_TA6962AA	973	968
ESC_TA6961AA	135	653
ESC_TA6960AA	942	198
ESC_TA6959AA	4624	387
ESC_TA6958AA	7348	331
ESC_TA6957AA	547	653
ESC_TA6956AA	602	257
ESC_TA6955AA	441	198
ESC_TA6954AA	8372	669
ESC_TA6953AA	5973	92
ESC_TA6952AA	164	175
ESC_TA6951AA	11708	198
ESC_TA6950AA	155	175
ESC_TA6949AA	164	175
ESC_TA6948AA	13	257
ESC_TA6947AA	101	696
ESC_TA6946AA	56	198
ESC_TA6945AA	162	924
ESC_TA6944AA	11714	175

ESC_TA6943AA	2559	373
ESC_TA6942AA	75	257
ESC_TA6941AA	10	512
ESC_TA6940AA	1170	629
ESC_TA6939AA	3303	537
ESC_TA6938AA	11707	393
ESC_TA6937AA	7236	336
ESC_TA6936AA	69	387
ESC_TA6935AA	5566	373
ESC_TA6934AA	2552	653
ESC_TA6933AA	5566	373
ESC_TA6932AA	1091	512
ESC_TA6931AA	11706	304
ESC_TA6930AA	122	768
ESC_TA6929AA	362	234
ESC_TA6928AA	2602	257
ESC_TA6927AA	11705	257
ESC_TA6926AA	162	924
ESC_TA6925AA	446	257
ESC_TA6924AA	10	512
ESC_TA6923AA	11705	257
ESC_TA6922AA	491	626
ESC_TA6921AA	602	257
ESC_TA6920AA	101	696
ESC_TA6919AA	162	924
ESC_TA6918AA	1122	307
ESC_TA6917AA	58	198
ESC_TA6916AA	9348	453
ESC_TA6915AA	681	393
ESC_TA6914AA	1308	257
ESC_TA6913AA	6490	847
ESC_TA6912AA	165	504
ESC_TA6911AA	2614	975
ESC_TA6910AA	154	257
ESC_TA6909AA	154	257
ESC_TA6908AA	10	512
ESC_TA6907AA	11725	257
ESC_TA6906AA	117	592
ESC_TA6905AA	3234	257
ESC_TA6904AA	11708	198
ESC_TA6903AA	162	924
ESC_TA6902AA	5540	373
ESC_TA6901AA	2614	975
ESC_TA6900AA	5566	373
ESC_TA6899AA	28	537
ESC_TA6898AA	11707	393
ESC_TA6897AA	162	924
ESC_TA6896AA	517	924
ESC_TA6895AA	162	924
ESC_TA6894AA	582	420

ESC_TA6893AA	547	653
ESC_TA6892AA	342	286
ESC_TA6891AA	5409	175
ESC_TA6890AA	162	924
ESC_TA6889AA	3234	257
ESC_TA6888AA	1308	257
ESC_TA6887AA	2602	257
ESC_TA6886AA	1011	198
ESC_TA6885AA	297	234
ESC_TA6884AA	7236	336
ESC_TA6883AA	327	204
ESC_TA6882AA	441	198
ESC_TA6881AA	1640	50
ESC_TA6880AA	602	257
ESC_TA6879AA	602	257
ESC_TA6878AA	75	257
ESC_TA6877AA	154	257
ESC_TA6876AA	165	504
ESC_TA6875AA	8891	113
ESC_TA6874AA	336	878
ESC_TA6873AA	441	198
ESC_TA6872AA	164	175
ESC_TA6871AA	327	204
ESC_TA6870AA	441	198
ESC_TA6869AA	75	257
ESC_TA6868AA	906	198
ESC_TA6867AA	1611	198
ESC_TA6866AA	11712	198
ESC_TA6865AA	441	198
ESC_TA6864AA	1611	198
ESC_TA6863AA	212	198
ESC_TA6862AA	5976	924
ESC_TA6861AA	88	160
ESC_TA6860AA	95	738
ESC_TA6859AA	75	257
ESC_TA6858AA	641	935
ESC_TA6857AA	1589	156
ESC_TA6856AA	355	764
ESC_TA6855AA	906	198
ESC_TA6854AA	28	537
ESC_TA6853AA	162	924
ESC_TA6852AA	38	669
ESC_TA6851AA	11711	653
ESC_TA6850AA	1011	198
ESC_TA6849AA	10	512
ESC_TA6848AA	8660	175
ESC_TA6847AA	547	653
ESC_TA6846AA	58	198
ESC_TA6845AA	11713	878
ESC_TA6844AA	278	924

ESC_TA6843AA	95	738
ESC_TA6842AA	906	198
ESC_TA6841AA	5540	373
ESC_TA6840AA	642	924
ESC_TA6839AA	5976	924
ESC_TA6660AA	410	156
ESC_TA6655AA	10	512
ESC_TA6653AA	10	512
ESC_TA6652AA	10	512
ESC_TA6651AA	56	198
ESC_TA6650AA	56	198
ESC_TA6649AA	442	257
ESC_TA6648AA	56	198
ESC_TA6647AA	11728	737
ESC_TA6646AA	7926	153
ESC_TA6645AA	388	156
ESC_TA6644AA	442	257
ESC_TA6643AA	56	198
ESC_TA6642AA	56	198
ESC_TA6641AA	56	198
ESC_TA6640AA	442	257
ESC_TA6639AA	56	198
ESC_TA6638AA	56	198
ESC_TA6637AA	442	257
ESC_TA6636AA	56	198
ESC_TA6635AA	746	789
ESC_TA6634AA	38	669
ESC_TA6633AA	10	512
ESC_TA6632AA	7025	288
ESC_TA6631AA	10	512
ESC_TA6630AA	678	257
ESC_TA6629AA	10	512
ESC_TA6628AA	34	504
ESC_TA6627AA	40	198
ESC_TA6626AA	11720	512
ESC_TA6625AA	38	669
ESC_TA6624AA	31	387
ESC_TA6623AA	10	512
ESC_TA6622AA	69	387
ESC_TA6621AA	636	653
ESC_TA6620AA	10	512
ESC_TA6619AA	6082	789
ESC_TA6618AA	131	653
ESC_TA6617AA	38	669
ESC_TA6616AA	69	387
ESC_TA6615AA	10	512
ESC_TA6614AA	10	512
ESC_TA6613AA	10	512
ESC_TA6612AA	10	512
ESC_TA6611AA	10	512

ESC_TA6610AA	10	512
ESC_TA6609AA	10	512
ESC_TA6608AA	10	512
ESC_TA6607AA	10	512
ESC_TA6606AA	10	512
ESC_TA6605AA	10	512
ESC_TA6604AA	10	512
ESC_TA6603AA	10	512
ESC_TA6602AA	10	512
ESC_TA6601AA	10	512
ESC_TA6600AA	10	512
ESC_TA6599AA	10	512
ESC_TA6598AA	10	512
ESC_TA6597AA	10	512
ESC_TA6596AA	10	512
ESC_TA6595AA	10	512
ESC_TA6594AA	10	512
ESC_TA6593AA	10	512
ESC_TA6592AA	10	512
ESC_TA6591AA	10	512
ESC_TA6590AA	10	512
ESC_TA6589AA	10	512
ESC_TA6588AA	10	512
ESC_TA6587AA	10	512
ESC_TA6586AA	10	512
ESC_TA6585AA	10	512
ESC_TA6584AA	10	512
ESC_TA6583AA	10	512
ESC_TA6582AA	10	512
ESC_TA6581AA	10	512
ESC_TA6580AA	10	512
ESC_TA6579AA	10	512
ESC_TA6578AA	10	512
ESC_TA6577AA	10	512
ESC_TA6576AA	10	512
ESC_TA6575AA	10	512
ESC_TA6574AA	10	512
ESC_TA6573AA	10	512
ESC_TA6572AA	10	512
ESC_TA6571AA	10	512
ESC_TA6569AA	23	156
ESC_TA6568AA	23	156
ESC_TA6567AA	23	156
ESC_TA6566AA	23	156
ESC_TA6565AA	23	156
ESC_TA6564AA	23	156
ESC_TA6563AA	23	156
ESC_TA6562AA	23	156
ESC_TA6561AA	23	156
ESC_TA6560AA	23	156

ESC_TA6559AA	23	156
ESC_TA6558AA	23	156
ESC_TA6556AA	1726	257
ESC_TA6555AA	67	257
ESC_TA6554AA	5277	101
ESC_TA6553AA	2101	108
ESC_TA6552AA	11732	924
ESC_TA6551AA	11731	924
ESC_TA6550AA	11716	673
ESC_TA6549AA	295	160
ESC_TA6548AA	1308	257
ESC_TA6547AA	101	696
ESC_TA6546AA	191	198
ESC_TA6545AA	2178	924
ESC_TA6544AA	11730	160
ESC_TA6543AA	2144	257
ESC_TA6542AA	11729	257
ESC_TA6541AA	2517	160
ESC_TA6540AA	295	160
ESC_TA6539AA	1020	924
ESC_TA6538AA	6	445
ESC_TA6537AA	6	445
ESC_TA6536AA	6	445
ESC_TA6535AA	6	445
ESC_TA6534AA	6	445
ESC_TA6533AA	11715	445
ESC_TA6532AA	6	445
ESC_TA6531AA	6	445
ESC_TA6530AA	6	445
ESC_TA6529AA	6	445
ESC_TA6528AA	6	445
ESC_TA6527AA	6	445
ESC_TA6526AA	6	445
ESC_TA6525AA	224	198
ESC_TA6524AA	1193	761
ESC_TA6523AA	95	738
ESC_TA6522AA	95	738
ESC_TA6521AA	1193	761
ESC_TA6520AA	963	669
ESC_TA6519AA	1193	761
ESC_TA6518AA	1193	761
ESC_TA6517AA	131	653
ESC_TA6516AA	131	653
ESC_TA6514AA	131	653
ESC_TA6513AA	1193	761
ESC_TA6512AA	648	359
ESC_TA6511AA	648	359
ESC_TA6510AA	1193	761
ESC_TA6509AA	1193	761
ESC_TA6508AA	1193	761

ESC_TA6507AA	1193	761
ESC_TA6506AA	648	359
ESC_TA6505AA	648	359
ESC_TA6504AA	131	653
ESC_TA6503AA	648	359
ESC_TA6502AA	648	359
ESC_TA6501AA	1193	761
ESC_TA6500AA	131	653
ESC_TA6499AA	648	359
ESC_TA6498AA	131	653
ESC_TA6497AA	1193	761
ESC_TA6496AA	162	924
ESC_TA6495AA	648	359
ESC_TA6494AA	648	359
ESC_TA6493AA	648	359
ESC_TA6492AA	1193	761
ESC_TA6491AA	11718	1035
ESC_TA6490AA	11718	1035
ESC_TA6489AA	1193	761
ESC_TA6488AA	1193	761
ESC_TA6487AA	1193	761
ESC_TA6486AA	1193	761
ESC_TA6485AA	1193	761
ESC_TA6484AA	393	343
ESC_TA6483AA	393	343
ESC_TA6482AA	393	343
ESC_TA6481AA	393	343
ESC_TA6480AA	131	653
ESC_TA6479AA	1193	761
ESC_TA6478AA	131	653
ESC_TA6477AA	131	653
ESC_TA6476AA	131	653
ESC_TA6475AA	131	653
ESC_TA6474AA	1193	761
ESC_TA6473AA	131	653
ESC_TA6472AA	131	653
ESC_TA6471AA	131	653
ESC_TA6470AA	131	653
ESC_TA6469AA	1193	761
ESC_TA6468AA	1193	761
ESC_TA6467AA	405	923
ESC_TA6466AA	405	923
ESC_TA6465AA	131	653
ESC_TA6464AA	131	653
ESC_TA6463AA	131	653
ESC_TA6462AA	1193	761
ESC_TA6461AA	131	653
ESC_TA6460AA	131	653
ESC_TA6459AA	1193	761
ESC_TA6458AA	131	653

ESC_TA6457AA	1193	761
ESC_TA6455AA	1193	761
ESC_TA6454AA	648	359
ESC_TA6453AA	1193	761
ESC_TA6452AA	9116	546
ESC_TA6451AA	648	359
ESC_TA6450AA	648	359
ESC_TA6449AA	648	359
ESC_TA6448AA	2178	924
ESC_TA6447AA	648	359
ESC_TA6446AA	648	359
ESC_TA6445AA	648	359
ESC_TA6444AA	1193	761
ESC_TA6443AA	1193	761
ESC_TA6442AA	1193	761
ESC_TA6441AA	1193	761
ESC_TA6440AA	131	653
ESC_TA6439AA	131	653
ESC_TA6438AA	224	198
ESC_TA6437AA	1193	761
ESC_TA6436AA	1193	761
ESC_TA6435AA	1193	761
ESC_TA6434AA	1193	761
ESC_TA6433AA	1193	761
ESC_TA6432AA	131	653
ESC_TA6431AA	131	653
ESC_TA6430AA	131	653
ESC_TA6429AA	1193	761
ESC_TA6428AA	1193	761
ESC_TA6427AA	1193	761
ESC_TA6426AA	131	653
ESC_TA6425AA	131	653
ESC_TA6424AA	131	653
ESC_TA6423AA	131	653
ESC_TA6422AA	131	653
ESC_TA6421AA	131	653
ESC_TA6420AA	131	653
ESC_TA6419AA	1193	761
ESC_TA6418AA	1049	175
ESC_TA6417AA	731	285
ESC_TA6416AA	1091	512
ESC_TA6415AA	1266	210
ESC_TA6414AA	10	512
ESC_TA6413AA	10	512
ESC_TA6412AA	10	512
ESC_TA6411AA	1091	512
ESC_TA6410AA	109	257
ESC_TA6409AA	641	935
ESC_TA6408AA	11024	256
ESC_TA6407AA	69	387

ESC_TA6406AA	1415	504
ESC_TA6405AA	398	445
ESC_TA6404AA	1415	504
ESC_TA6403AA	345	175
ESC_TA6402AA	1121	198
ESC_TA6401AA	731	285
ESC_TA6400AA	1121	198
ESC_TA6399AA	543	842
ESC_TA6398AA	10	512
ESC_TA6397AA	10	512
ESC_TA6396AA	540	286
ESC_TA6395AA	88	160
ESC_TA6394AA	295	160
ESC_TA6393AA	10	512
ESC_TA6392AA	1125	198
ESC_TA6391AA	1123	257
ESC_TA6390AA	10	512
ESC_TA6389AA	58	198
ESC_TA6388AA	731	285
ESC_TA6387AA	685	311
ESC_TA6386AA	11733	512
ESC_TA6385AA	227	504
ESC_TA6384AA	662	470
ESC_TA6383AA	88	160
ESC_TA6382AA	1049	175
ESC_TA6381AA	10	512
ESC_TA6380AA	58	198
ESC_TA6379AA	1421	447
ESC_TA6378AA	56	198
ESC_TA6377AA	10	512
ESC_TA6376AA	731	285
ESC_TA6375AA	5901	234
ESC_TA6374AA	1613	198
ESC_TA6373AA	10	512
ESC_TA6372AA	10	512
ESC_TA6371AA	10	512
ESC_TA6370AA	1495	311
ESC_TA6369AA	295	160
ESC_TA6368AA	1301	270
ESC_TA6367AA	9355	198
ESC_TA6366AA	69	387
ESC_TA6365AA	10	512
ESC_TA6364AA	1125	198
ESC_TA6363AA	10	512
ESC_TA6362AA	1121	198
ESC_TA6361AA	10	512
ESC_TA6360AA	297	234
ESC_TA6359AA	1121	198
ESC_TA6358AA	1123	257
ESC_TA6357AA	1125	198

ESC_TA6356AA	58	198
ESC_TA6355AA	1408	445
ESC_TA6354AA	1121	198
ESC_TA6353AA	548	512
ESC_TA6352AA	685	311
ESC_TA6351AA	1125	198
ESC_TA6350AA	11023	512
ESC_TA6349AA	10	512
ESC_TA6347AA	10	512
ESC_TA6346AA	1125	198
ESC_TA6345AA	10	512
ESC_TA6344AA	9315	504
ESC_TA6343AA	398	445
ESC_TA6341AA	10	512
ESC_TA6340AA	10	512
ESC_TA6339AA	398	445
ESC_TA6338AA	69	387
ESC_TA6337AA	2325	447
ESC_TA6336AA	58	198
ESC_TA6335AA	58	198
ESC_TA6334AA	58	198
ESC_TA6333AA	9334	257
ESC_TA6332AA	1125	198
ESC_TA6331AA	58	198
ESC_TA6330AA	88	160
ESC_TA6329AA	8076	504
ESC_TA6328AA	70	670
ESC_TA6327AA	48	76
ESC_TA6326AA	1091	512
ESC_TA6325AA	58	198
ESC_TA6324AA	10	512
ESC_TA6323AA	662	470
ESC_TA6322AA	162	924
ESC_TA6321AA	1125	198
ESC_TA6320AA	10	512
ESC_TA6319AA	10	512
ESC_TA6318AA	101	696
ESC_TA6317AA	1121	198
ESC_TA6316AA	88	160
ESC_TA6315AA	1146	198
ESC_TA6314AA	10	512
ESC_TA6313AA	1495	311
ESC_TA6312AA	10	512
ESC_TA6311AA	69	387
ESC_TA6310AA	662	470
ESC_TA6309AA	4087	512
ESC_TA6308AA	10	512
ESC_TA6307AA	1125	198
ESC_TA6306AA	4260	737
ESC_TA6305AA	10	512

ESC_TA6304AA	731	285
ESC_TA6303AA	1125	198
ESC_TA6302AA	731	285
ESC_TA6301AA	1125	198
ESC_TA6300AA	731	285
ESC_TA6299AA	11719	234
ESC_TA6298AA	548	512
ESC_TA6297AA	10	512
ESC_TA6296AA	88	160
ESC_TA6277AA	409	504
ESC_TA6276AA	359	696
ESC_TA6275AA	165	504
ESC_TA6274AA	409	504
ESC_TA6273AA	11511	373
ESC_TA6272AA	48	76
ESC_TA6271AA	359	696
ESC_TA6270AA	48	76
ESC_TA6269AA	359	696
ESC_TA6268AA	359	696
ESC_TA6267AA	359	696
ESC_TA6266AA	409	504
ESC_TA6265AA	409	504
ESC_TA6264AA	409	504
ESC_TA6263AA	409	504
ESC_TA6262AA	359	696
ESC_TA6261AA	11511	373
ESC_TA6260AA	165	504
ESC_TA6245AA	10	512
ESC_TA6244AA	95	738
ESC_TA6243AA	131	653
ESC_TA6242AA	2003	669
ESC_TA6241AA	162	924
ESC_TA6240AA	744	512
ESC_TA6239AA	2003	669
ESC_TA6238AA	2003	669
ESC_TA6237AA	69	387
ESC_TA6236AA	127	738
ESC_TA6235AA	410	156
ESC_TA6234AA	450	71
ESC_TA6233AA	354	15
ESC_TA6232AA	405	923
ESC_TA6231AA	10698	512
ESC_TA6230AA	648	359
ESC_TA6229AA	405	923
ESC_TA6228AA	224	198
ESC_TA6227AA	1193	761
ESC_TA6226AA	1193	761
ESC_TA6225AA	354	15
ESC_TA6224AA	224	198
ESC_TA6223AA	131	653

ESC_TA6222AA	218	519
ESC_TA6221AA	1722	599
ESC_TA6220AA	48	76
ESC_TA6219AA	11703	912
ESC_TA6218AA	1656	175
ESC_TA6217AA	3014	410
ESC_TA6216AA	131	653
ESC_TA6215AA	11702	504
ESC_TA6214AA	4985	254
ESC_TA6213AA	115	669
ESC_TA6212AA	1193	761
ESC_TA6211AA	58	198
ESC_TA6210AA	1193	761
ESC_TA6209AA	68	386
ESC_TA6208AA	131	653
ESC_TA6207AA	1702	512
ESC_TA6206AA	131	653
ESC_TA6205AA	410	156
ESC_TA6204AA	2003	669
ESC_TA6203AA	155	175
ESC_TA6202AA	4995	198
ESC_TA6201AA	710	291
ESC_TA6200AA	6588	233
ESC_TA6199AA	11	936
ESC_TA6198AA	245	261
ESC_TA6197AA	245	261
ESC_TA6196AA	21	156
ESC_TA6195AA	428	654
ESC_TA6194AA	245	261
ESC_TA6193AA	245	261
ESC_TA6192AA	245	261
ESC_TA6191AA	10	512
ESC_TA6190AA	2522	198
ESC_TA6189AA	730	445
ESC_TA6188AA	2522	198
ESC_TA6187AA	16	156
ESC_TA6186AA	16	156
ESC_TA6185AA	2522	198
ESC_TA6184AA	2522	198
ESC_TA6183AA	16	156
ESC_TA6182AA	16	156
ESC_TA6181AA	58	198
ESC_TA6180AA	3519	101
ESC_TA6179AA	16	156
ESC_TA6178AA	746	789
ESC_TA6177AA	29	156
ESC_TA6176AA	10	512
ESC_TA6175AA	10	512
ESC_TA6174AA	10	512
ESC_TA6173AA	10	512

ESC_TA6172AA	10	512
ESC_TA6171AA	10	512
ESC_TA6170AA	10	512
ESC_TA6169AA	10	512
ESC_TA6168AA	10	512
ESC_TA6167AA	10	512
ESC_TA6166AA	10	512
ESC_TA6165AA	10	512
ESC_TA6164AA	10	512
ESC_TA6163AA	10	512
ESC_TA6162AA	10	512
ESC_TA6161AA	10	512
ESC_TA6160AA	10	512
ESC_TA6159AA	10	512
ESC_TA6158AA	10	512
ESC_TA6156AA	10	512
ESC_TA6155AA	10	512
ESC_TA6154AA	10	512
ESC_TA6152AA	10	512
ESC_TA6151AA	10	512
ESC_TA6150AA	10	512
ESC_TA6149AA	10	512
ESC_TA6148AA	10	512
ESC_TA6147AA	10	512
ESC_TA6146AA	10	512
ESC_TA6145AA	10	512
ESC_TA6144AA	10	512
ESC_TA6143AA	10	512
ESC_TA6142AA	10	512
ESC_TA6141AA	10	512
ESC_TA6140AA	10	512
ESC_TA6139AA	10	512
ESC_TA6138AA	10	512
ESC_TA6137AA	10	512
ESC_TA6136AA	10	512
ESC_TA6135AA	10	512
ESC_TA6134AA	10	512
ESC_TA6133AA	10	512
ESC_TA6132AA	10	512
ESC_TA6131AA	10	512
ESC_TA6130AA	10	512
ESC_TA6129AA	10	512
ESC_TA6128AA	10	512
ESC_TA6127AA	10	512
ESC_TA6126AA	10	512
ESC_TA6125AA	10	512
ESC_TA6124AA	10	512
ESC_TA6123AA	10	512
ESC_TA6122AA	10	512
ESC_TA6121AA	10	512

ESC_TA6120AA	10	512
ESC_TA6119AA	10	512
ESC_TA6118AA	10	512
ESC_TA6117AA	10	512
ESC_TA6116AA	10	512
ESC_TA6115AA	10	512
ESC_TA6114AA	10	512
ESC_TA6113AA	10	512
ESC_TA6112AA	10	512
ESC_TA6111AA	10	512
ESC_TA6110AA	10	512
ESC_TA6109AA	10	512
ESC_TA6108AA	10	512
ESC_TA6107AA	10	512
ESC_TA6106AA	10	512
ESC_TA6105AA	10	512
ESC_TA6104AA	10	512
ESC_TA6103AA	10	512
ESC_TA6102AA	10	512
ESC_TA6101AA	10	512
ESC_TA6100AA	10	512
ESC_TA6099AA	10	512
ESC_TA6098AA	10	512
ESC_TA6097AA	10	512
ESC_TA6096AA	10	512
ESC_TA6095AA	10	512
ESC_TA6094AA	10	512
ESC_TA6093AA	10	512
ESC_TA6092AA	10	512
ESC_TA6091AA	10	512
ESC_TA6090AA	10	512
ESC_TA6089AA	10	512
ESC_TA6088AA	10	512
ESC_TA6087AA	10	512
ESC_TA6086AA	10	512
ESC_TA6085AA	10	512
ESC_TA6084AA	10	512
ESC_TA6082AA	10	512
ESC_TA6081AA	10	512
ESC_TA6080AA	10	512
ESC_TA6079AA	10	512
ESC_TA6078AA	10	512
ESC_TA6077AA	10	512
ESC_TA6076AA	10	512
ESC_TA6075AA	10	512
ESC_TA6074AA	10	512
ESC_TA6073AA	10	512
ESC_TA6072AA	10	512
ESC_TA6071AA	10	512
ESC_TA6070AA	10	512

ESC_TA6069AA	10	512
ESC_TA6068AA	10	512
ESC_TA6067AA	10	512
ESC_TA6066AA	10	512
ESC_TA6065AA	10	512
ESC_TA6064AA	10	512
ESC_TA6063AA	10	512
ESC_TA6062AA	10	512
ESC_TA6061AA	10	512
ESC_TA6060AA	10	512
ESC_TA6059AA	10	512
ESC_TA6058AA	10	512
ESC_TA6057AA	10	512
ESC_TA6056AA	10	512
ESC_TA6055AA	10	512
ESC_TA6054AA	10	512
ESC_TA6053AA	10	512
ESC_TA6052AA	10	512
ESC_TA6051AA	10	512
ESC_TA6050AA	10	512
ESC_TA6049AA	10	512
ESC_TA6048AA	10	512
ESC_TA6047AA	10	512
ESC_TA6046AA	10	512
ESC_TA6045AA	10	512
ESC_TA6044AA	10	512
ESC_TA6043AA	10	512
ESC_TA6042AA	10	512
ESC_TA6041AA	10	512
ESC_TA6040AA	10	512
ESC_TA6039AA	10	512
ESC_TA6038AA	10	512
ESC_TA6037AA	10	512
ESC_TA6036AA	10	512
ESC_TA6035AA	10	512
ESC_TA6034AA	10	512
ESC_TA6033AA	10	512
ESC_TA6032AA	10	512
ESC_TA6031AA	10	512
ESC_TA6030AA	10	512
ESC_TA6029AA	10	512
ESC_TA6028AA	10	512
ESC_TA6027AA	10	512
ESC_TA6026AA	10	512
ESC_TA6025AA	10	512
ESC_TA6024AA	10	512
ESC_TA6023AA	10	512
ESC_TA6022AA	10	512
ESC_TA6021AA	10	512
ESC_TA6020AA	10	512

ESC_TA6019AA	10	512
ESC_TA6018AA	10	512
ESC_TA6017AA	10	512
ESC_TA6016AA	10	512
ESC_TA6015AA	10	512
ESC_TA6014AA	10	512
ESC_TA6013AA	10	512
ESC_TA6012AA	10	512
ESC_TA6011AA	10	512
ESC_TA6010AA	10	512
ESC_TA6009AA	10	512
ESC_TA6008AA	10	512
ESC_TA6007AA	10	512
ESC_TA6006AA	10	512
ESC_TA6005AA	10	512
ESC_TA6004AA	10	512
ESC_TA6003AA	10	512
ESC_TA6002AA	10	512
ESC_TA6001AA	10	512
ESC_TA6000AA	10	512
ESC_TA5999AA	10	512
ESC_TA5998AA	10	512
ESC_TA5997AA	10	512
ESC_TA5996AA	10	512
ESC_TA5995AA	10	512
ESC_TA5994AA	10	512
ESC_TA5993AA	10	512
ESC_TA5992AA	10	512
ESC_TA5991AA	10	512
ESC_TA5990AA	10	512
ESC_TA5989AA	10	512
ESC_TA5988AA	10	512
ESC_TA5987AA	10	512
ESC_TA5986AA	10	512
ESC_TA5985AA	10	512
ESC_TA5984AA	10	512
ESC_TA5983AA	10	512
ESC_TA5982AA	10	512
ESC_TA5981AA	10	512
ESC_TA5980AA	10	512
ESC_TA5979AA	10	512
ESC_TA5978AA	10	512
ESC_TA5977AA	10	512
ESC_TA5976AA	10	512
ESC_TA5975AA	10	512
ESC_TA5974AA	10	512
ESC_TA5973AA	10	512
ESC_TA5972AA	10	512
ESC_TA5971AA	10	512
ESC_TA5970AA	10	512

ESC_TA5969AA	10	512
ESC_TA5968AA	10	512
ESC_TA5966AA	10	512
ESC_TA5965AA	10	512
ESC_TA5964AA	10	512
ESC_TA5963AA	10	512
ESC_TA5962AA	10	512
ESC_TA5961AA	10	512
ESC_TA5960AA	10	512
ESC_TA5959AA	10	512
ESC_TA5958AA	10	512
ESC_TA5957AA	10	512
ESC_TA5956AA	10	512
ESC_TA5955AA	10	512
ESC_TA5954AA	10	512
ESC_TA5953AA	10	512
ESC_TA5952AA	10	512
ESC_TA5951AA	10	512
ESC_TA5950AA	10	512
ESC_TA5949AA	10	512
ESC_TA5948AA	10	512
ESC_TA5947AA	10	512
ESC_TA5946AA	10	512
ESC_TA5945AA	10	512
ESC_TA5944AA	10	512
ESC_TA5943AA	10	512
ESC_TA5942AA	10	512
ESC_TA5941AA	10	512
ESC_TA5940AA	10	512
ESC_TA5939AA	10	512
ESC_TA5938AA	10	512
ESC_TA5937AA	10	512
ESC_TA5936AA	10	512
ESC_TA5935AA	10	512
ESC_TA5934AA	10	512
ESC_TA5933AA	10	512
ESC_TA5932AA	10	512
ESC_TA5931AA	10	512
ESC_TA5930AA	10	512
ESC_TA5929AA	10	512
ESC_TA5928AA	10	512
ESC_TA5927AA	10	512
ESC_TA5926AA	10	512
ESC_TA5925AA	10	512
ESC_TA5924AA	10	512
ESC_TA5923AA	10	512
ESC_TA5922AA	10	512
ESC_TA5921AA	10	512
ESC_TA5920AA	10	512
ESC_TA5919AA	10	512

ESC_TA5918AA	10	512
ESC_TA5917AA	10	512
ESC_TA5916AA	10	512
ESC_TA5915AA	10	512
ESC_TA5914AA	10	512
ESC_TA5913AA	10	512
ESC_TA5912AA	10	512
ESC_TA5911AA	10	512
ESC_TA5910AA	10	512
ESC_TA5909AA	10	512
ESC_TA5908AA	10	512
ESC_TA5907AA	10	512
ESC_TA5906AA	10	512
ESC_TA5905AA	10	512
ESC_TA5904AA	10	512
ESC_TA5903AA	10	512
ESC_TA5902AA	10	512
ESC_TA5901AA	10	512
ESC_TA5900AA	10	512
ESC_TA5899AA	10	512
ESC_TA5898AA	10	512
ESC_TA5897AA	10	512
ESC_TA5896AA	10	512
ESC_TA5895AA	10	512
ESC_TA5894AA	10	512
ESC_TA5893AA	10	512
ESC_TA5892AA	10	512
ESC_TA5891AA	10	512
ESC_TA5890AA	10	512
ESC_TA5889AA	10	512
ESC_TA5888AA	10	512
ESC_TA5887AA	10	512
ESC_TA5886AA	10	512
ESC_TA5885AA	10	512
ESC_TA5884AA	10	512
ESC_TA5883AA	10	512
ESC_TA5882AA	10	512
ESC_TA5881AA	10	512
ESC_TA5880AA	10	512
ESC_TA5879AA	10	512
ESC_TA5878AA	10	512
ESC_TA5877AA	10	512
ESC_TA5876AA	10	512
ESC_TA5875AA	10	512
ESC_TA5874AA	10	512
ESC_TA5873AA	10	512
ESC_TA5872AA	10	512
ESC_TA5871AA	10	512
ESC_TA5870AA	10	512
ESC_TA5869AA	10	512

ESC_TA5868AA	10	512
ESC_TA5867AA	10	512
ESC_TA5866AA	10	512
ESC_TA5865AA	10	512
ESC_TA5864AA	10	512
ESC_TA5863AA	10	512
ESC_TA5862AA	10	512
ESC_TA5861AA	10	512
ESC_TA5860AA	10	512
ESC_TA5859AA	10	512
ESC_TA5858AA	10	512
ESC_TA5857AA	10	512
ESC_TA5856AA	10	512
ESC_TA5855AA	10	512
ESC_TA5854AA	10	512
ESC_TA5853AA	10	512
ESC_TA5852AA	10	512
ESC_TA5851AA	10	512
ESC_TA5850AA	10	512
ESC_TA5849AA	10	512
ESC_TA5848AA	10	512
ESC_TA5847AA	10	512
ESC_TA5846AA	10	512
ESC_TA5845AA	10	512
ESC_TA5844AA	10	512
ESC_TA5843AA	10	512
ESC_TA5842AA	10	512
ESC_TA5841AA	10	512
ESC_TA5840AA	10	512
ESC_TA5839AA	10	512
ESC_TA5838AA	10	512
ESC_TA5837AA	10	512
ESC_TA5836AA	10	512
ESC_TA5835AA	10	512
ESC_TA5834AA	10	512
ESC_TA5833AA	10	512
ESC_TA5832AA	10	512
ESC_TA5831AA	10	512
ESC_TA5830AA	10	512
ESC_TA5829AA	10	512
ESC_TA5828AA	10	512
ESC_TA5827AA	10	512
ESC_TA5826AA	10	512
ESC_TA5825AA	10	512
ESC_TA5824AA	10	512
ESC_TA5823AA	10	512
ESC_TA5822AA	10	512
ESC_TA5821AA	10	512
ESC_TA5820AA	10	512
ESC_TA5819AA	10	512

ESC_TA5818AA	10	512
ESC_TA5817AA	10	512
ESC_TA5816AA	10	512
ESC_TA5815AA	10	512
ESC_TA5814AA	10	512
ESC_TA5813AA	10	512
ESC_TA5812AA	10	512
ESC_TA5811AA	10	512
ESC_TA5810AA	10	512
ESC_TA5809AA	10	512
ESC_TA5808AA	10	512
ESC_TA5807AA	10	512
ESC_TA5806AA	10	512
ESC_TA5805AA	10	512
ESC_TA5804AA	10	512
ESC_TA5803AA	10	512
ESC_TA5802AA	10	512
ESC_TA5801AA	10	512
ESC_TA5800AA	10	512
ESC_TA5799AA	10	512
ESC_TA5798AA	10	512
ESC_TA5797AA	10	512
ESC_TA5796AA	10	512
ESC_TA5795AA	10	512
ESC_TA5794AA	10	512
ESC_TA5793AA	10	512
ESC_TA5792AA	10	512
ESC_TA5791AA	10	512
ESC_TA5790AA	10	512
ESC_TA5789AA	10	512
ESC_TA5788AA	10	512
ESC_TA5787AA	10	512
ESC_TA5786AA	10	512
ESC_TA5785AA	10	512
ESC_TA5784AA	10	512
ESC_TA5783AA	10	512
ESC_TA5782AA	10	512
ESC_TA5781AA	10	512
ESC_TA5780AA	10	512
ESC_TA5779AA	10	512
ESC_TA5778AA	10	512
ESC_TA5777AA	10	512
ESC_TA5776AA	10	512
ESC_TA5775AA	10	512
ESC_TA5774AA	10	512
ESC_TA5773AA	10	512
ESC_TA5772AA	10	512
ESC_TA5771AA	10	512
ESC_TA5770AA	10	512
ESC_TA5769AA	10	512

ESC_TA5768AA	10	512
ESC_TA5767AA	10	512
ESC_TA5766AA	10	512
ESC_TA5765AA	10	512
ESC_TA5764AA	10	512
ESC_TA5763AA	10	512
ESC_TA5762AA	10	512
ESC_TA5761AA	10	512
ESC_TA5760AA	10	512
ESC_TA5759AA	10	512
ESC_TA5758AA	10	512
ESC_TA5757AA	10	512
ESC_TA5756AA	10	512
ESC_TA5755AA	10	512
ESC_TA5754AA	10	512
ESC_TA5753AA	10	512
ESC_TA5752AA	10	512
ESC_TA5751AA	10	512
ESC_TA5750AA	10	512
ESC_TA5749AA	10	512
ESC_TA5748AA	10	512
ESC_TA5747AA	10	512
ESC_TA5746AA	10	512
ESC_TA5745AA	10	512
ESC_TA5744AA	10	512
ESC_TA5743AA	10	512
ESC_TA5742AA	10	512
ESC_TA5741AA	10	512
ESC_TA5740AA	10	512
ESC_TA5739AA	10	512
ESC_TA5738AA	10	512
ESC_TA5737AA	10	512
ESC_TA5736AA	10	512
ESC_TA5735AA	10	512
ESC_TA5734AA	10	512
ESC_TA5733AA	10	512
ESC_TA5732AA	10	512
ESC_TA5731AA	10	512
ESC_TA5730AA	10	512
ESC_TA5729AA	10	512
ESC_TA5728AA	10	512
ESC_TA5727AA	10	512
ESC_TA5726AA	10	512
ESC_TA5725AA	10	512
ESC_TA5724AA	10	512
ESC_TA5723AA	10	512
ESC_TA5722AA	10	512
ESC_TA5721AA	10	512
ESC_TA5720AA	10	512
ESC_TA5719AA	10	512

ESC_TA5718AA	10	512
ESC_TA5717AA	10	512
ESC_TA5716AA	10	512
ESC_TA5715AA	10	512
ESC_TA5714AA	10	512
ESC_TA5713AA	10	512
ESC_TA5712AA	10	512
ESC_TA5711AA	10	512
ESC_TA5710AA	10	512
ESC_TA5709AA	10	512
ESC_TA5708AA	10	512
ESC_TA5707AA	10	512
ESC_TA5706AA	10	512
ESC_TA5705AA	10	512
ESC_TA5704AA	10	512
ESC_TA5703AA	10	512
ESC_TA5702AA	10	512
ESC_TA5701AA	10	512
ESC_TA5700AA	10	512
ESC_TA5699AA	10	512
ESC_TA5698AA	10	512
ESC_TA5697AA	10	512
ESC_TA5696AA	10	512
ESC_TA5695AA	10	512
ESC_TA5694AA	10	512
ESC_TA5693AA	10	512
ESC_TA5692AA	10	512
ESC_TA5691AA	10	512
ESC_TA5690AA	10	512
ESC_TA5689AA	10	512
ESC_TA5688AA	10	512
ESC_TA5687AA	10	512
ESC_TA5686AA	10	512
ESC_TA5685AA	10	512
ESC_TA5684AA	10	512
ESC_TA5683AA	10	512
ESC_TA5682AA	10	512
ESC_TA5681AA	10	512
ESC_TA5680AA	10	512
ESC_TA5679AA	10	512
ESC_TA5678AA	10	512
ESC_TA5677AA	10	512
ESC_TA5676AA	10	512
ESC_TA5675AA	10	512
ESC_TA5674AA	10	512
ESC_TA5673AA	10	512
ESC_TA5672AA	10	512
ESC_TA5671AA	10	512
ESC_TA5670AA	10	512
ESC_TA5669AA	10	512

ESC_TA5668AA	10	512
ESC_TA5667AA	10	512
ESC_TA5666AA	10	512
ESC_TA5665AA	10	512
ESC_TA5664AA	10	512
ESC_TA5663AA	10	512
ESC_TA5662AA	10	512
ESC_TA5661AA	10	512
ESC_TA5660AA	10	512
ESC_TA5659AA	10	512
ESC_TA5658AA	10	512
ESC_TA5657AA	10	512
ESC_TA5656AA	10	512
ESC_TA5655AA	10	512
ESC_TA5654AA	10	512
ESC_TA5653AA	10	512
ESC_TA5652AA	10	512
ESC_TA5651AA	10	512
ESC_TA5650AA	10	512
ESC_TA5649AA	10	512
ESC_TA5648AA	10	512
ESC_TA5647AA	10	512
ESC_TA5646AA	10	512
ESC_TA5645AA	10	512
ESC_TA5644AA	10	512
ESC_TA5643AA	10	512
ESC_TA5642AA	10	512
ESC_TA5641AA	10	512
ESC_TA5640AA	10	512
ESC_TA5639AA	10	512
ESC_TA5638AA	10	512
ESC_TA5637AA	10	512
ESC_TA5636AA	10	512
ESC_TA5635AA	10	512
ESC_TA5634AA	10	512
ESC_TA5633AA	10	512
ESC_TA5632AA	10	512
ESC_TA5631AA	10	512
ESC_TA5630AA	10	512
ESC_TA5629AA	10	512
ESC_TA5628AA	10	512
ESC_TA5627AA	10	512
ESC_TA5626AA	10	512
ESC_TA5625AA	10	512
ESC_TA5624AA	10	512
ESC_TA5622AA	10	512
ESC_TA5621AA	10	512
ESC_TA5620AA	10	512
ESC_TA5619AA	10	512
ESC_TA5618AA	10	512

ESC_TA5617AA	10	512
ESC_TA5616AA	10	512
ESC_TA5615AA	10	512
ESC_TA5614AA	10	512
ESC_TA5613AA	10	512
ESC_TA5612AA	10	512
ESC_TA5611AA	10	512
ESC_TA5610AA	10	512
ESC_TA5609AA	10	512
ESC_TA5608AA	10	512
ESC_TA5607AA	10	512
ESC_TA5606AA	10	512
ESC_TA5605AA	10	512
ESC_TA5604AA	10	512
ESC_TA5603AA	10	512
ESC_TA5602AA	10	512
ESC_TA5601AA	10	512
ESC_TA5600AA	10	512
ESC_TA5599AA	10	512
ESC_TA5598AA	10	512
ESC_TA5597AA	10	512
ESC_TA5596AA	10	512
ESC_TA5595AA	10	512
ESC_TA5594AA	10	512
ESC_TA5593AA	10	512
ESC_TA5592AA	10	512
ESC_TA5591AA	10	512
ESC_TA5590AA	10	512
ESC_TA5588AA	10	512
ESC_TA5587AA	10	512
ESC_TA5586AA	10	512
ESC_TA5585AA	10	512
ESC_TA5584AA	10	512
ESC_TA5582AA	10	512
ESC_TA5581AA	10	512
ESC_TA5580AA	10	512
ESC_TA5579AA	10	512
ESC_TA5578AA	10	512
ESC_TA5577AA	10	512
ESC_TA5576AA	10	512
ESC_TA5575AA	10	512
ESC_TA5574AA	10	512
ESC_TA5573AA	10	512
ESC_TA5572AA	10	512
ESC_TA5571AA	10	512
ESC_TA5570AA	10	512
ESC_TA5569AA	10	512
ESC_TA5568AA	10	512
ESC_TA5567AA	10	512
ESC_TA5566AA	10	512

ESC_TA5565AA	10	512
ESC_TA5564AA	10	512
ESC_TA5563AA	10	512
ESC_TA5562AA	10	512
ESC_TA5561AA	10	512
ESC_TA5560AA	10	512
ESC_TA5559AA	10	512
ESC_TA5558AA	10	512
ESC_TA5557AA	10	512
ESC_TA5556AA	10	512
ESC_TA5555AA	10	512
ESC_TA5554AA	10	512
ESC_TA5553AA	10	512
ESC_TA5552AA	10	512
ESC_TA5551AA	10	512
ESC_TA5550AA	10	512
ESC_TA5549AA	10	512
ESC_TA5548AA	10	512
ESC_TA5547AA	10	512
ESC_TA5546AA	10	512
ESC_TA5545AA	10	512
ESC_TA5544AA	10	512
ESC_TA5543AA	10	512
ESC_TA5542AA	10	512
ESC_TA5541AA	10	512
ESC_TA5540AA	10	512
ESC_TA5539AA	10	512
ESC_TA5538AA	10	512
ESC_TA5537AA	164	175
ESC_TA5536AA	1844	198
ESC_TA5535AA	69	387
ESC_TA5534AA	69	387
ESC_TA5533AA	10	512
ESC_TA5532AA	3580	257
ESC_TA5531AA	7616	257
ESC_TA5530AA	10	512
ESC_TA5529AA	1850	269
ESC_TA5528AA	652	204
ESC_TA5527AA	4421	257
ESC_TA5526AA	1704	257
ESC_TA5525AA	1844	198
ESC_TA5524AA	5876	847
ESC_TA5523AA	152	257
ESC_TA5522AA	245	261
ESC_TA5521AA	245	261
ESC_TA5520AA	11701	261
ESC_TA5519AA	245	261
ESC_TA5518AA	11	936
ESC_TA5517AA	11	936
ESC_TA5516AA	10	512

ESC_TA5515AA	33	198
ESC_TA5514AA	8304	286
ESC_TA5513AA	660	286
ESC_TA5512AA	25	234
ESC_TA5511AA	21	156
ESC_TA5510AA	4748	234
ESC_TA5509AA	25	234
ESC_TA5508AA	442	257
ESC_TA5507AA	4496	924
ESC_TA5506AA	4496	924
ESC_TA5505AA	58	198
ESC_TA5504AA	1237	447
ESC_TA5503AA	602	257
ESC_TA5502AA	201	257
ESC_TA5501AA	3234	257
ESC_TA5500AA	603	198
ESC_TA5499AA	162	924
ESC_TA5498AA	117	592
ESC_TA5497AA	2253	71
ESC_TA5496AA	4138	198
ESC_TA5495AA	1844	198
ESC_TA5494AA	3090	212
ESC_TA5493AA	3090	212
ESC_TA5492AA	205	257
ESC_TA5490AA	2207	474
ESC_TA5489AA	3014	410
ESC_TA5488AA	48	76
ESC_TA5486AA	155	175
ESC_TA5485AA	3998	198
ESC_TA5484AA	155	175
ESC_TA5483AA	10	512
ESC_TA5482AA	10	512
ESC_TA5480AA	4380	198
ESC_TA5479AA	4380	198
ESC_TA5478AA	4380	198
ESC_TA5475AA	4380	198
ESC_TA5474AA	642	924
ESC_TA5473AA	1426	160
ESC_TA5472AA	57	50
ESC_TA5471AA	4981	473
ESC_TA5470AA	2001	670
ESC_TA5469AA	2001	670
ESC_TA5468AA	2001	670
ESC_TA5467AA	388	156
ESC_TA5466AA	2280	212
ESC_TA5465AA	155	175
ESC_TA5464AA	10	512
ESC_TA5463AA	11700	257
ESC_TA5462AA	101	696
ESC_TA5460AA	4935	445

ESC_TA5459AA	11699	203
ESC_TA5458AA	58	198
ESC_TA5457AA	744	512
ESC_TA5453AA	949	175
ESC_TA5451AA	10292	512
ESC_TA5448AA	164	175
ESC_TA5447AA	2766	198
ESC_TA5445AA	641	935
ESC_TA5444AA	165	504
ESC_TA5443AA	1495	311
ESC_TA5442AA	38	669
ESC_TA5441AA	359	696
ESC_TA5440AA	2001	670
ESC_TA5439AA	162	924
ESC_TA5438AA	69	387
ESC_TA5437AA	847	257
ESC_TA5436AA	162	924
ESC_TA5435AA	2524	789
ESC_TA5434AA	602	257
ESC_TA5432AA	3014	410
ESC_TA5431AA	3014	410
ESC_TA5430AA	405	923
ESC_TA5429AA	131	653
ESC_TA5428AA	1193	761
ESC_TA5427AA	393	343
ESC_TA5426AA	1193	761
ESC_TA5425AA	131	653
ESC_TA5424AA	131	653
ESC_TA5423AA	1193	761
ESC_TA5422AA	131	653
ESC_TA5421AA	131	653
ESC_TA5420AA	405	923
ESC_TA5419AA	131	653
ESC_TA5418AA	131	653
ESC_TA5417AA	131	653
ESC_TA5416AA	131	653
ESC_TA5415AA	131	653
ESC_TA5414AA	11698	72
ESC_TA5413AA	349	670
ESC_TA5412AA	152	257
ESC_TA5411AA	245	261
ESC_TA5409AA	69	387
ESC_TA5408AA	48	76
ESC_TA5406AA	11697	653
ESC_TA5405AA	11696	669
ESC_TA5403AA	9580	286
ESC_TA5402AA	11695	738
ESC_TA5400AA	11694	257
ESC_TA5399AA	8492	234
ESC_TA5397AA	11693	798

ESC_TA5396AA	152	257
ESC_TA5395AA	152	257
ESC_TA5394AA	23	156
ESC_TA5393AA	152	257
ESC_TA5392AA	152	257
ESC_TA5391AA	245	261
ESC_TA5390AA	245	261
ESC_TA5389AA	301	307
ESC_TA5388AA	301	307
ESC_TA5387AA	301	307
ESC_TA5386AA	301	307
ESC_TA5385AA	23	156
ESC_TA5384AA	1485	408
ESC_TA5383AA	23	156
ESC_TA5250AA	297	234
ESC_TA5235AA	218	519
ESC_TA5157AA	245	261
ESC_TA5156AA	245	261
ESC_TA5155AA	11689	71
ESC_TA5154AA	23	156
ESC_TA5153AA	11691	261
ESC_TA5152AA	11	936
ESC_TA5151AA	11	936
ESC_TA5150AA	23	156
ESC_TA5149AA	744	512
ESC_TA5148AA	152	257
ESC_TA5147AA	11692	257
ESC_TA5146AA	21	156
ESC_TA5142AA	156	175
ESC_TA5141AA	223	198
ESC_TA5140AA	5876	847
ESC_TA5139AA	1079	235
ESC_TA5138AA	117	592
ESC_TA5137AA	399	198
ESC_TA5136AA	34	504
ESC_TA5135AA	117	592
ESC_TA5134AA	1423	198
ESC_TA5133AA	1385	653
ESC_TA5132AA	538	629
ESC_TA5131AA	6401	198
ESC_TA5130AA	10	512
ESC_TA5129AA	607	355
ESC_TA5128AA	162	924
ESC_TA5127AA	10	512
ESC_TA5126AA	58	198
ESC_TA5125AA	906	198
ESC_TA5123AA	1434	512
ESC_TA5122AA	38	669
ESC_TA5121AA	1434	512
ESC_TA5120AA	1246	198

ESC_TA5119AA	115	669
ESC_TA5118AA	641	935
ESC_TA5117AA	10	512
ESC_TA5116AA	10	512
ESC_TA5115AA	10	512
ESC_TA5114AA	10	512
ESC_TA5113AA	10	512
ESC_TA5112AA	10	512
ESC_TA5111AA	10	512
ESC_TA5110AA	10	512
ESC_TA5109AA	10	512
ESC_TA5108AA	10	512
ESC_TA5107AA	17	191
ESC_TA5106AA	442	257
ESC_TA5105AA	761	512
ESC_TA5104AA	32	270
ESC_TA5103AA	297	234
ESC_TA5102AA	131	653
ESC_TA5101AA	88	160
ESC_TA5100AA	245	261
ESC_TA5099AA	245	261
ESC_TA5098AA	245	261
ESC_TA5097AA	245	261
ESC_TA5096AA	245	261
ESC_TA5095AA	245	261
ESC_TA5094AA	245	261
ESC_TA5093AA	11691	261
ESC_TA5092AA	245	261
ESC_TA5091AA	245	261
ESC_TA5090AA	152	257
ESC_TA5089AA	245	261
ESC_TA5088AA	245	261
ESC_TA5087AA	245	261
ESC_TA5086AA	245	261
ESC_TA5085AA	11	936
ESC_TA5084AA	223	198
ESC_TA5083AA	223	198
ESC_TA5082AA	1819	808
ESC_TA5081AA	442	257
ESC_TA5080AA	11	936
ESC_TA5079AA	17	191
ESC_TA5078AA	29	156
ESC_TA5077AA	4553	408
ESC_TA5076AA	4553	408
ESC_TA5075AA	4553	408
ESC_TA5074AA	156	175
ESC_TA5073AA	38	669
ESC_TA5072AA	38	669
ESC_TA5071AA	648	359
ESC_TA5070AA	131	653

ESC_TA5068AA	131	653
ESC_TA5067AA	131	653
ESC_TA5066AA	21	156
ESC_TA5065AA	40	198
ESC_TA5064AA	5431	0
ESC_TA5063AA	69	387
ESC_TA5062AA	69	387
ESC_TA5061AA	752	512
ESC_TA5060AA	11	936
ESC_TA5059AA	11690	936
ESC_TA5058AA	11	936
ESC_TA5057AA	11	936
ESC_TA5056AA	1041	765
ESC_TA5055AA	446	257
ESC_TA5054AA	58	198
ESC_TA5053AA	446	257
ESC_TA5052AA	164	175
ESC_TA5051AA	164	175
ESC_TA5050AA	58	198
ESC_TA5049AA	164	175
ESC_TA5048AA	21	156
ESC_TA5047AA	200	198
ESC_TA5046AA	442	257
ESC_TA5045AA	3101	160
ESC_TA5044AA	442	257
ESC_TA5043AA	21	156
ESC_TA5042AA	25	234
ESC_TA5041AA	39	156
ESC_TA5040AA	442	257
ESC_TA5039AA	33	198
ESC_TA5038AA	59	217
ESC_TA5037AA	10	512
ESC_TA5036AA	10	512
ESC_TA5035AA	10	512
ESC_TA5034AA	10	512
ESC_TA5033AA	10	512
ESC_TA5032AA	10	512
ESC_TA5031AA	10	512
ESC_TA5030AA	677	257
ESC_TA5028AA	117	592
ESC_TA5027AA	1485	408
ESC_TA5026AA	23	156
ESC_TA5025AA	117	592
ESC_TA5024AA	11686	936
ESC_TA5023AA	21	156
ESC_TA5022AA	811	234
ESC_TA5021AA	11685	355
ESC_TA5020AA	410	156
ESC_TA5019AA	223	198
ESC_TA5018AA	223	198

ESC_TA5017AA	4942	36
ESC_TA5016AA	223	198
ESC_TA5015AA	223	198
ESC_TA5014AA	223	198
ESC_TA5013AA	223	198
ESC_TA5012AA	152	257
ESC_TA5011AA	152	257
ESC_TA5010AA	152	257
ESC_TA5009AA	152	257
ESC_TA5008AA	245	261
ESC_TA5007AA	245	261
ESC_TA5006AA	152	257
ESC_TA5005AA	245	261
ESC_TA5004AA	152	257
ESC_TA5003AA	152	257
ESC_TA5002AA	152	257
ESC_TA5001AA	152	257
ESC_TA5000AA	245	261
ESC_TA4999AA	442	257
ESC_TA4998AA	33	198
ESC_TA4997AA	811	234
ESC_TA4996AA	33	198
ESC_TA4995AA	10	512
ESC_TA4994AA	11	936
ESC_TA4993AA	93	71
ESC_TA4992AA	811	234
ESC_TA4991AA	3570	156
ESC_TA4990AA	752	512
ESC_TA4989AA	11	936
ESC_TA4988AA	11683	297
ESC_TA4987AA	21	156
ESC_TA4986AA	11682	234
ESC_TA4985AA	10	512
ESC_TA4984AA	10	512
ESC_TA4983AA	10	512
ESC_TA4982AA	29	156
ESC_TA4981AA	152	257
ESC_TA4980AA	152	257
ESC_TA4979AA	152	257
ESC_TA4978AA	145	256
ESC_TA4977AA	744	512
ESC_TA4975AA	88	160
ESC_TA4974AA	410	156
ESC_TA4973AA	2389	160
ESC_TA4972AA	297	234
ESC_TA4971AA	58	198
ESC_TA4970AA	223	198
ESC_TA4969AA	658	684
ESC_TA4968AA	679	257
ESC_TA4967AA	223	198

ESC_TA4965AA	10	512
ESC_TA4964AA	443	257
ESC_TA4963AA	2389	160
ESC_TA4962AA	641	935
ESC_TA4961AA	658	684
ESC_TA4960AA	297	234
ESC_TA4959AA	443	257
ESC_TA4958AA	297	234
ESC_TA4957AA	223	198
ESC_TA4956AA	7261	198
ESC_TA4955AA	58	198
ESC_TA4954AA	655	926
ESC_TA4953AA	11209	257
ESC_TA4952AA	446	257
ESC_TA4949AA	658	684
ESC_TA4948AA	3018	903
ESC_TA4947AA	297	234
ESC_TA4946AA	21	156
ESC_TA4945AA	297	234
ESC_TA4940AA	131	653
ESC_TA4939AA	252	190
ESC_TA4938AA	73	737
ESC_TA4937AA	2179	924
ESC_TA4936AA	167	512
ESC_TA4935AA	361	512
ESC_TA4934AA	617	511
ESC_TA4933AA	405	923
ESC_TA4932AA	38	669
ESC_TA4931AA	448	257
ESC_TA4930AA	648	359
ESC_TA4929AA	167	512
ESC_TA4928AA	648	359
ESC_TA4927AA	167	512
ESC_TA4926AA	167	512
ESC_TA4925AA	410	156
ESC_TA4924AA	940	257
ESC_TA4923AA	405	923
ESC_TA4922AA	46	447
ESC_TA4921AA	38	669
ESC_TA4920AA	46	447
ESC_TA4919AA	405	923
ESC_TA4918AA	361	512
ESC_TA4917AA	167	512
ESC_TA4916AA	11681	0
ESC_TA4915AA	361	512
ESC_TA4914AA	10	512
ESC_TA4913AA	491	626
ESC_TA4912AA	1193	761
ESC_TA4911AA	2659	679
ESC_TA4910AA	361	512

ESC_TA4909AA	410	156
ESC_TA4908AA	940	257
ESC_TA4906AA	69	387
ESC_TA4905AA	648	359
ESC_TA4904AA	361	512
ESC_TA4903AA	167	512
ESC_TA4902AA	167	512
ESC_TA4901AA	1284	604
ESC_TA4900AA	90	156
ESC_TA4899AA	448	257
ESC_TA4898AA	617	511
ESC_TA4897AA	1702	512
ESC_TA4896AA	12	626
ESC_TA4895AA	617	511
ESC_TA4894AA	648	359
ESC_TA4893AA	362	234
ESC_TA4892AA	1284	604
ESC_TA4891AA	648	359
ESC_TA4890AA	131	653
ESC_TA4889AA	10	512
ESC_TA4887AA	1284	604
ESC_TA4886AA	46	447
ESC_TA4885AA	167	512
ESC_TA4884AA	167	512
ESC_TA4883AA	405	923
ESC_TA4882AA	167	512
ESC_TA4881AA	1284	604
ESC_TA4880AA	167	512
ESC_TA4879AA	617	511
ESC_TA4878AA	940	257
ESC_TA4877AA	156	175
ESC_TA4876AA	167	512
ESC_TA4875AA	2659	679
ESC_TA4874AA	405	923
ESC_TA4873AA	167	512
ESC_TA4872AA	1284	604
ESC_TA4871AA	361	512
ESC_TA4870AA	361	512
ESC_TA4869AA	410	156
ESC_TA4867AA	69	387
ESC_TA4866AA	1284	604
ESC_TA4865AA	11680	387
ESC_TA4864AA	405	923
ESC_TA4863AA	11679	738
ESC_TA4862AA	11678	653
ESC_TA4861AA	131	653
ESC_TA4860AA	1640	50
ESC_TA4859AA	38	669
ESC_TA4858AA	11675	924
ESC_TA4857AA	11676	653

ESC_TA4856AA	11677	380
ESC_TA4855AA	11674	387
ESC_TA4854AA	131	653
ESC_TA4853AA	245	261
ESC_TA4852AA	152	257
ESC_TA4851AA	245	261
ESC_TA4850AA	152	257
ESC_TA4849AA	245	261
ESC_TA4848AA	152	257
ESC_TA4847AA	152	257
ESC_TA4846AA	152	257
ESC_TA4845AA	245	261
ESC_TA4844AA	152	257
ESC_TA4843AA	1025	261
ESC_TA4842AA	152	257
ESC_TA4841AA	245	261
ESC_TA4840AA	152	257
ESC_TA4839AA	245	261
ESC_TA4838AA	152	257
ESC_TA4837AA	245	261
ESC_TA4836AA	245	261
ESC_TA4835AA	152	257
ESC_TA4834AA	152	257
ESC_TA4833AA	152	257
ESC_TA4832AA	245	261
ESC_TA4831AA	152	257
ESC_TA4830AA	152	257
ESC_TA4829AA	152	257
ESC_TA4828AA	152	257
ESC_TA4827AA	245	261
ESC_TA4826AA	245	261
ESC_TA4825AA	152	257
ESC_TA4824AA	152	257
ESC_TA4823AA	152	257
ESC_TA4822AA	152	257
ESC_TA4821AA	152	257
ESC_TA4820AA	152	257
ESC_TA4819AA	152	257
ESC_TA4818AA	152	257
ESC_TA4817AA	152	257
ESC_TA4816AA	152	257
ESC_TA4815AA	152	257
ESC_TA4814AA	152	257
ESC_TA4813AA	152	257
ESC_TA4812AA	245	261
ESC_TA4811AA	152	257
ESC_TA4810AA	152	257
ESC_TA4809AA	152	257
ESC_TA4808AA	152	257
ESC_TA4807AA	152	257

ESC_TA4806AA	152	257
ESC_TA4805AA	152	257
ESC_TA4804AA	58	198
ESC_TA4803AA	11673	234
ESC_TA4802AA	738	598
ESC_TA4801AA	21	156
ESC_TA4800AA	152	257
ESC_TA4799AA	245	261
ESC_TA4798AA	245	261
ESC_TA4796AA	245	261
ESC_TA4795AA	152	257
ESC_TA4794AA	152	257
ESC_TA4793AA	245	261
ESC_TA4792AA	245	261
ESC_TA4791AA	245	261
ESC_TA4790AA	206	286
ESC_TA4789AA	10	512
ESC_TA4788AA	7416	372
ESC_TA4787AA	5651	803
ESC_TA4786AA	9287	732
ESC_TA4785AA	4392	234
ESC_TA4784AA	69	387
ESC_TA4783AA	10	512
ESC_TA4782AA	7416	372
ESC_TA4781AA	362	234
ESC_TA4780AA	711	935
ESC_TA4778AA	2307	198
ESC_TA4777AA	642	924
ESC_TA4776AA	4392	234
ESC_TA4775AA	1304	257
ESC_TA4774AA	131	653
ESC_TA4773AA	48	76
ESC_TA4772AA	155	175
ESC_TA4771AA	48	76
ESC_TA4770AA	1125	198
ESC_TA4769AA	88	160
ESC_TA4768AA	58	198
ESC_TA4767AA	155	175
ESC_TA4765AA	752	512
ESC_TA4764AA	11	936
ESC_TA4763AA	752	512
ESC_TA4762AA	131	653
ESC_TA4709AA	205	257
ESC_TA4708AA	8427	257
ESC_TA4707AA	297	234
ESC_TA4706AA	2385	198
ESC_TA4705AA	205	257
ESC_TA4704AA	205	257
ESC_TA4703AA	1114	504
ESC_TA4702AA	747	640

ESC_TA4701AA	2387	261
ESC_TA4700AA	641	935
ESC_TA4699AA	4175	270
ESC_TA4698AA	1114	504
ESC_TA4697AA	2520	878
ESC_TA4696AA	152	257
ESC_TA4695AA	245	261
ESC_TA4694AA	245	261
ESC_TA4693AA	28	537
ESC_TA4692AA	33	198
ESC_TA4691AA	152	257
ESC_TA4690AA	152	257
ESC_TA4689AA	245	261
ESC_TA4688AA	147	102
ESC_TA4687AA	152	257
ESC_TA4686AA	628	261
ESC_TA4685AA	152	257
ESC_TA4684AA	245	261
ESC_TA4683AA	8934	844
ESC_TA4682AA	11671	962
ESC_TA4659AA	11670	198
ESC_TA4658AA	569	738
ESC_TA4657AA	11605	627
ESC_TA4656AA	131	653
ESC_TA4655AA	11441	880
ESC_TA4654AA	11158	235
ESC_TA4653AA	11285	924
ESC_TA4652AA	11669	1032
ESC_TA4651AA	11668	878
ESC_TA4650AA	11672	653
ESC_TA4649AA	648	359
ESC_TA4648AA	73	737
ESC_TA4647AA	73	737
ESC_TA4646AA	73	737
ESC_TA4645AA	2008	257
ESC_TA4644AA	8427	257
ESC_TA4643AA	8427	257
ESC_TA4642AA	21	156
ESC_TA4641AA	205	257
ESC_TA4640AA	21	156
ESC_TA4639AA	11	936
ESC_TA4638AA	11	936
ESC_TA4637AA	205	257
ESC_TA4636AA	8427	257
ESC_TA4560AA	21	156
ESC_TA4559AA	1113	221
ESC_TA4558AA	205	257
ESC_TA4557AA	3476	512
ESC_TA4556AA	278	924
ESC_TA4555AA	1679	160

ESC_TA4554AA	10	512
ESC_TA4553AA	11666	74
ESC_TA4552AA	6015	234
ESC_TA4551AA	48	76
ESC_TA4550AA	11667	1031
ESC_TA4549AA	86	935
ESC_TA4548AA	2334	701
ESC_TA4547AA	58	198
ESC_TA4546AA	164	175
ESC_TA4545AA	48	76
ESC_TA4544AA	93	71
ESC_TA4543AA	683	175
ESC_TA4542AA	11	936
ESC_TA4541AA	11	936
ESC_TA4540AA	11	936
ESC_TA4539AA	453	935
ESC_TA4538AA	3580	257
ESC_TA4537AA	770	587
ESC_TA4536AA	5973	92
ESC_TA4535AA	38	669
ESC_TA4534AA	401	142
ESC_TA4533AA	355	764
ESC_TA4532AA	278	924
ESC_TA4531AA	131	653
ESC_TA4530AA	457	595
ESC_TA4529AA	117	592
ESC_TA4528AA	540	286
ESC_TA4524AA	405	923
ESC_TA4523AA	69	387
ESC_TA4522AA	11176	512
ESC_TA4521AA	11176	512
ESC_TA4518AA	405	923
ESC_TA4517AA	617	511
ESC_TA4511AA	617	511
ESC_TA4508AA	405	923
ESC_TA4507AA	69	387
ESC_TA4506AA	617	511
ESC_TA4502AA	131	653
ESC_TA4501AA	8767	669
ESC_TA4500AA	648	359
ESC_TA4497AA	131	653
ESC_TA4496AA	648	359
ESC_TA4495AA	131	653
ESC_TA4494AA	44	512
ESC_TA4492AA	131	653
ESC_TA4491AA	1193	761
ESC_TA4490AA	131	653
ESC_TA4489AA	131	653
ESC_TA4488AA	131	653
ESC_TA4487AA	131	653

ESC_TA4486AA	746	789
ESC_TA4485AA	738	598
ESC_TA4484AA	25	234
ESC_TA4483AA	5592	830
ESC_TA4482AA	5592	830
ESC_TA4481AA	5592	830
ESC_TA4480AA	5578	830
ESC_TA4479AA	5578	830
ESC_TA4478AA	5578	830
ESC_TA4477AA	6373	828
ESC_TA4476AA	5592	830
ESC_TA4475AA	152	257
ESC_TA4474AA	152	257
ESC_TA4473AA	152	257
ESC_TA4472AA	152	257
ESC_TA4471AA	11662	257
ESC_TA4470AA	152	257
ESC_TA4469AA	152	257
ESC_TA4468AA	245	261
ESC_TA4467AA	245	261
ESC_TA4466AA	152	257
ESC_TA4465AA	152	257
ESC_TA4464AA	152	257
ESC_TA4463AA	5592	830
ESC_TA4462AA	1025	261
ESC_TA4461AA	152	257
ESC_TA4460AA	152	257
ESC_TA4459AA	152	257
ESC_TA4458AA	152	257
ESC_TA4457AA	152	257
ESC_TA4456AA	152	257
ESC_TA4455AA	152	257
ESC_TA4454AA	152	257
ESC_TA4453AA	152	257
ESC_TA4452AA	152	257
ESC_TA4451AA	152	257
ESC_TA4450AA	152	257
ESC_TA4449AA	152	257
ESC_TA4448AA	152	257
ESC_TA4447AA	152	257
ESC_TA4446AA	152	257
ESC_TA4445AA	152	257
ESC_TA4444AA	152	257
ESC_TA4443AA	245	261
ESC_TA4442AA	5578	830
ESC_TA4441AA	5592	830
ESC_TA4440AA	413	121
ESC_TA4439AA	5592	830
ESC_TA4438AA	5592	830
ESC_TA4437AA	29	156

ESC_TA4436AA	11661	912
ESC_TA4435AA	17	191
ESC_TA4434AA	641	935
ESC_TA4433AA	641	935
ESC_TA4432AA	33	198
ESC_TA4431AA	33	198
ESC_TA4430AA	33	198
ESC_TA4429AA	33	198
ESC_TA4428AA	33	198
ESC_TA4427AA	33	198
ESC_TA4426AA	33	198
ESC_TA4425AA	33	198
ESC_TA4424AA	11663	198
ESC_TA4423AA	641	935
ESC_TA4422AA	33	198
ESC_TA4421AA	33	198
ESC_TA4420AA	33	198
ESC_TA4419AA	33	198
ESC_TA4418AA	33	198
ESC_TA4417AA	33	198
ESC_TA4416AA	33	198
ESC_TA4415AA	33	198
ESC_TA4414AA	33	198
ESC_TA4413AA	641	935
ESC_TA4412AA	33	198
ESC_TA4411AA	33	198
ESC_TA4410AA	33	198
ESC_TA4409AA	33	198
ESC_TA4408AA	442	257
ESC_TA4407AA	11664	198
ESC_TA4406AA	641	935
ESC_TA4405AA	33	198
ESC_TA4404AA	33	198
ESC_TA4403AA	641	935
ESC_TA4402AA	33	198
ESC_TA4401AA	11664	198
ESC_TA4400AA	33	198
ESC_TA4399AA	33	198
ESC_TA4398AA	33	198
ESC_TA4397AA	33	198
ESC_TA4396AA	33	198
ESC_TA4395AA	33	198
ESC_TA4394AA	33	198
ESC_TA4393AA	33	198
ESC_TA4392AA	33	198
ESC_TA4391AA	9700	198
ESC_TA4390AA	11664	198
ESC_TA4389AA	442	257
ESC_TA4388AA	33	198
ESC_TA4387AA	33	198

ESC_TA4386AA	21	156
ESC_TA4385AA	152	257
ESC_TA4384AA	245	261
ESC_TA4383AA	11660	261
ESC_TA4382AA	342	286
ESC_TA4381AA	152	257
ESC_TA4380AA	152	257
ESC_TA4379AA	245	261
ESC_TA4378AA	152	257
ESC_TA4377AA	152	257
ESC_TA4376AA	245	261
ESC_TA4375AA	245	261
ESC_TA4374AA	11	936
ESC_TA4373AA	7931	234
ESC_TA4372AA	33	198
ESC_TA4371AA	11	936
ESC_TA4370AA	137	270
ESC_TA4369AA	442	257
ESC_TA4368AA	10	512
ESC_TA4367AA	10	512
ESC_TA4366AA	10	512
ESC_TA4365AA	442	257
ESC_TA4364AA	11	936
ESC_TA4363AA	33	198
ESC_TA4362AA	675	234
ESC_TA4361AA	4748	234
ESC_TA4353AA	770	587
ESC_TA4352AA	73	737
ESC_TA4351AA	245	261
ESC_TA4350AA	10	512
ESC_TA4349AA	11659	198
ESC_TA4348AA	10	512
ESC_TA4347AA	167	512
ESC_TA4346AA	1284	604
ESC_TA4345AA	167	512
ESC_TA4344AA	167	512
ESC_TA4343AA	167	512
ESC_TA4342AA	405	923
ESC_TA4341AA	405	923
ESC_TA4340AA	167	512
ESC_TA4339AA	167	512
ESC_TA4338AA	354	15
ESC_TA4337AA	11	936
ESC_TA4336AA	32	270
ESC_TA4335AA	32	270
ESC_TA4333AA	300	286
ESC_TA4332AA	16	156
ESC_TA4331AA	16	156
ESC_TA4330AA	5973	92
ESC_TA4329AA	16	156

ESC_TA4328AA	5973	92
ESC_TA4327AA	5973	92
ESC_TA4326AA	301	307
ESC_TA4325AA	301	307
ESC_TA4324AA	21	156
ESC_TA4323AA	10	512
ESC_TA4322AA	11	936
ESC_TA4321AA	752	512
ESC_TA4320AA	11656	108
ESC_TA4319AA	11654	1033
ESC_TA4318AA	11651	55
ESC_TA4317AA	11649	923
ESC_TA4316AA	11653	118
ESC_TA4315AA	11652	257
ESC_TA4314AA	11650	204
ESC_TA4313AA	11580	935
ESC_TA4312AA	11648	808
ESC_TA4311AA	11647	908
ESC_TA4310AA	11581	1026
ESC_TA4309AA	11644	928
ESC_TA4308AA	11646	55
ESC_TA4306AA	11642	989
ESC_TA4305AA	8826	926
ESC_TA4304AA	11645	261
ESC_TA4303AA	11640	971
ESC_TA4302AA	11641	878
ESC_TA4301AA	11655	885
ESC_TA4300AA	11639	935
ESC_TA4299AA	11656	108
ESC_TA4298AA	11655	885
ESC_TA4297AA	11643	698
ESC_TA4296AA	1204	961
ESC_TA4289AA	354	15
ESC_TA4288AA	28	537
ESC_TA4287AA	10	512
ESC_TA4286AA	1444	768
ESC_TA4285AA	607	355
ESC_TA4284AA	7290	512
ESC_TA4283AA	6701	535
ESC_TA4282AA	156	175
ESC_TA4281AA	11	936
ESC_TA4280AA	11	936
ESC_TA4279AA	11	936
ESC_TA4278AA	43	512
ESC_TA4277AA	10	512
ESC_TA4276AA	93	71
ESC_TA4275AA	998	644
ESC_TA4274AA	14	761
ESC_TA4273AA	569	738
ESC_TA4272AA	420	741

ESC_TA4271AA	1278	797
ESC_TA4270AA	127	738
ESC_TA4269AA	127	738
ESC_TA4268AA	12	626
ESC_TA4267AA	12	626
ESC_TA4266AA	11657	512
ESC_TA4265AA	2178	924
ESC_TA4264AA	12	626
ESC_TA4263AA	2604	626
ESC_TA4262AA	12	626
ESC_TA4261AA	131	653
ESC_TA4260AA	131	653
ESC_TA4259AA	131	653
ESC_TA4258AA	141	644
ESC_TA4257AA	131	653
ESC_TA4256AA	457	595
ESC_TA4255AA	38	669
ESC_TA4254AA	38	669
ESC_TA4253AA	165	504
ESC_TA4252AA	11658	817
ESC_TA4251AA	131	653
ESC_TA4247AA	1011	198
ESC_TA4235AA	88	160
ESC_TA4127AA	245	261
ESC_TA4126AA	245	261
ESC_TA4125AA	245	261
ESC_TA4124AA	152	257
ESC_TA4123AA	245	261
ESC_TA4122AA	245	261
ESC_TA4121AA	245	261
ESC_TA4120AA	245	261
ESC_TA4119AA	11	936
ESC_TA4118AA	11	936
ESC_TA4117AA	442	257
ESC_TA4116AA	101	696
ESC_TA4115AA	1677	739
ESC_TA4114AA	11	936
ESC_TA4113AA	101	696
ESC_TA4112AA	421	744
ESC_TA4111AA	10	512
ESC_TA4110AA	127	738
ESC_TA4109AA	10	512
ESC_TA4108AA	131	653
ESC_TA4107AA	131	653
ESC_TA4106AA	62	574
ESC_TA4105AA	361	512
ESC_TA4104AA	2186	198
ESC_TA4103AA	73	737
ESC_TA4102AA	156	175
ESC_TA4101AA	1163	579

ESC_TA4100AA	131	653
ESC_TA4099AA	224	198
ESC_TA4098AA	48	76
ESC_TA4097AA	131	653
ESC_TA4096AA	73	737
ESC_TA4095AA	10	512
ESC_TA4094AA	998	644
ESC_TA4093AA	410	156
ESC_TA4092AA	131	653
ESC_TA4091AA	48	76
ESC_TA4090AA	542	824
ESC_TA4089AA	69	387
ESC_TA4088AA	10	512
ESC_TA4087AA	10995	257
ESC_TA4086AA	131	653
ESC_TA4085AA	405	923
ESC_TA4084AA	219	877
ESC_TA4083AA	224	198
ESC_TA4082AA	38	669
ESC_TA4081AA	131	653
ESC_TA4080AA	315	669
ESC_TA4079AA	450	71
ESC_TA4078AA	131	653
ESC_TA4077AA	8881	362
ESC_TA4076AA	3268	669
ESC_TA4075AA	38	669
ESC_TA4074AA	73	737
ESC_TA4073AA	131	653
ESC_TA4072AA	394	400
ESC_TA4071AA	38	669
ESC_TA4070AA	1695	504
ESC_TA4068AA	349	670
ESC_TA4067AA	354	15
ESC_TA4066AA	131	653
ESC_TA4065AA	38	669
ESC_TA4064AA	328	924
ESC_TA4063AA	181	311
ESC_TA4062AA	752	512
ESC_TA4061AA	405	923
ESC_TA4060AA	1193	761
ESC_TA4059AA	410	156
ESC_TA4058AA	69	387
ESC_TA4057AA	38	669
ESC_TA4056AA	540	286
ESC_TA4055AA	1727	257
ESC_TA4054AA	73	737
ESC_TA4053AA	38	669
ESC_TA4052AA	155	175
ESC_TA4051AA	131	653
ESC_TA4050AA	1193	761

ESC_TA4049AA	8881	362
ESC_TA4048AA	155	175
ESC_TA4047AA	131	653
ESC_TA4045AA	9269	198
ESC_TA4044AA	155	175
ESC_TA4043AA	141	644
ESC_TA4042AA	1312	76
ESC_TA4041AA	10	512
ESC_TA4040AA	648	359
ESC_TA4039AA	131	653
ESC_TA4038AA	2141	597
ESC_TA4036AA	131	653
ESC_TA4035AA	131	653
ESC_TA4034AA	2076	400
ESC_TA4033AA	500	71
ESC_TA4032AA	131	653
ESC_TA4031AA	28	537
ESC_TA4030AA	127	738
ESC_TA4029AA	1193	761
ESC_TA4028AA	1312	76
ESC_TA4027AA	131	653
ESC_TA4026AA	69	387
ESC_TA4025AA	648	359
ESC_TA4024AA	421	744
ESC_TA4023AA	131	653
ESC_TA4022AA	550	756
ESC_TA4021AA	68	386
ESC_TA4020AA	68	386
ESC_TA4019AA	131	653
ESC_TA4018AA	101	696
ESC_TA4017AA	10	512
ESC_TA4016AA	90	156
ESC_TA4015AA	38	669
ESC_TA4014AA	10	512
ESC_TA4013AA	131	653
ESC_TA4012AA	616	156
ESC_TA4011AA	131	653
ESC_TA4010AA	1193	761
ESC_TA4009AA	131	653
ESC_TA4008AA	10	512
ESC_TA4007AA	8881	362
ESC_TA4006AA	131	653
ESC_TA4005AA	2076	400
ESC_TA4004AA	131	653
ESC_TA4003AA	1193	761
ESC_TA4002AA	10	512
ESC_TA4001AA	131	653
ESC_TA4000AA	38	669
ESC_TA3999AA	38	669
ESC_TA3998AA	1193	761

ESC_TA3997AA	10	512
ESC_TA3996AA	501	900
ESC_TA3995AA	1193	761
ESC_TA3994AA	131	653
ESC_TA3993AA	131	653
ESC_TA3992AA	73	737
ESC_TA3991AA	10992	76
ESC_TA3990AA	1642	198
ESC_TA3989AA	515	1004
ESC_TA3988AA	38	669
ESC_TA3987AA	155	175
ESC_TA3986AA	58	198
ESC_TA3985AA	394	400
ESC_TA3984AA	636	653
ESC_TA3983AA	131	653
ESC_TA3982AA	10	512
ESC_TA3981AA	162	924
ESC_TA3980AA	38	669
ESC_TA3979AA	131	653
ESC_TA3978AA	2040	198
ESC_TA3977AA	405	923
ESC_TA3976AA	1193	761
ESC_TA3975AA	46	447
ESC_TA3974AA	181	311
ESC_TA3973AA	10993	738
ESC_TA3972AA	4981	473
ESC_TA3971AA	58	198
ESC_TA3970AA	227	504
ESC_TA3969AA	998	644
ESC_TA3968AA	88	160
ESC_TA3967AA	73	737
ESC_TA3966AA	450	71
ESC_TA3965AA	131	653
ESC_TA3964AA	46	447
ESC_TA3963AA	69	387
ESC_TA3962AA	38	669
ESC_TA3961AA	443	257
ESC_TA3960AA	10994	789
ESC_TA3959AA	73	737
ESC_TA3958AA	1727	257
ESC_TA3957AA	115	669
ESC_TA3956AA	648	359
ESC_TA3955AA	295	160
ESC_TA3954AA	4981	473
ESC_TA3953AA	3541	842
ESC_TA3952AA	278	924
ESC_TA3951AA	8881	362
ESC_TA3950AA	648	359
ESC_TA3949AA	10	512
ESC_TA3948AA	131	653

ESC_TA3947AA	10	512
ESC_TA3946AA	10996	76
ESC_TA3945AA	73	737
ESC_TA3944AA	2141	597
ESC_TA3943AA	69	387
ESC_TA3942AA	410	156
ESC_TA3941AA	1611	198
ESC_TA3940AA	484	71
ESC_TA3939AA	38	669
ESC_TA3938AA	131	653
ESC_TA3937AA	3346	592
ESC_TA3936AA	540	286
ESC_TA3935AA	131	653
ESC_TA3934AA	131	653
ESC_TA3933AA	10	512
ESC_TA3932AA	10	512
ESC_TA3931AA	448	257
ESC_TA3930AA	517	924
ESC_TA3929AA	155	175
ESC_TA3928AA	156	175
ESC_TA3927AA	10997	473
ESC_TA3926AA	8881	362
ESC_TA3925AA	10997	473
ESC_TA3924AA	131	653
ESC_TA3923AA	1294	512
ESC_TA3922AA	93	71
ESC_TA3921AA	1147	198
ESC_TA3920AA	95	738
ESC_TA3919AA	515	1004
ESC_TA3918AA	131	653
ESC_TA3917AA	501	900
ESC_TA3916AA	38	669
ESC_TA3915AA	58	198
ESC_TA3914AA	48	76
ESC_TA3913AA	1722	599
ESC_TA3912AA	38	669
ESC_TA3911AA	4684	257
ESC_TA3910AA	141	644
ESC_TA3909AA	6496	198
ESC_TA3908AA	2562	640
ESC_TA3907AA	442	257
ESC_TA3906AA	5614	257
ESC_TA3905AA	34	504
ESC_TA3904AA	10	512
ESC_TA3903AA	10994	789
ESC_TA3902AA	10	512
ESC_TA3901AA	38	669
ESC_TA3900AA	10	512
ESC_TA3899AA	131	653
ESC_TA3898AA	5584	640

ESC_TA3897AA	10998	670
ESC_TA3896AA	2089	276
ESC_TA3895AA	34	504
ESC_TA3894AA	501	900
ESC_TA3893AA	206	286
ESC_TA3892AA	773	71
ESC_TA3891AA	8881	362
ESC_TA3890AA	34	504
ESC_TA3889AA	38	669
ESC_TA3888AA	3873	175
ESC_TA3887AA	131	653
ESC_TA3886AA	38	669
ESC_TA3885AA	130	387
ESC_TA3884AA	10	512
ESC_TA3883AA	2076	400
ESC_TA3882AA	1193	761
ESC_TA3881AA	38	669
ESC_TA3880AA	6355	625
ESC_TA3879AA	515	1004
ESC_TA3878AA	131	653
ESC_TA3877AA	120	180
ESC_TA3876AA	167	512
ESC_TA3875AA	38	669
ESC_TA3874AA	1193	761
ESC_TA3873AA	38	669
ESC_TA3872AA	131	653
ESC_TA3871AA	4981	473
ESC_TA3870AA	46	447
ESC_TA3869AA	442	257
ESC_TA3868AA	90	156
ESC_TA3867AA	130	387
ESC_TA3866AA	442	257
ESC_TA3865AA	8149	311
ESC_TA3864AA	48	76
ESC_TA3863AA	450	71
ESC_TA3862AA	349	670
ESC_TA3861AA	131	653
ESC_TA3860AA	517	924
ESC_TA3859AA	131	653
ESC_TA3858AA	10	512
ESC_TA3857AA	10	512
ESC_TA3856AA	355	764
ESC_TA3855AA	372	424
ESC_TA3854AA	372	424
ESC_TA3853AA	372	424
ESC_TA3852AA	372	424
ESC_TA3851AA	372	424
ESC_TA3850AA	372	424
ESC_TA3849AA	372	424
ESC_TA3848AA	372	424

ESC_TA3847AA	372	424
ESC_TA3846AA	372	424
ESC_TA3845AA	372	424
ESC_TA3844AA	372	424
ESC_TA3843AA	372	424
ESC_TA3842AA	372	424
ESC_TA3841AA	372	424
ESC_TA3840AA	372	424
ESC_TA3839AA	372	424
ESC_TA3838AA	372	424
ESC_TA3837AA	372	424
ESC_TA3836AA	372	424
ESC_TA3835AA	372	424
ESC_TA3834AA	372	424
ESC_TA3833AA	372	424
ESC_TA3832AA	361	512
ESC_TA3831AA	940	257
ESC_TA3830AA	167	512
ESC_TA3829AA	167	512
ESC_TA3828AA	167	512
ESC_TA3827AA	1588	100
ESC_TA3826AA	167	512
ESC_TA3825AA	405	923
ESC_TA3824AA	405	923
ESC_TA3823AA	1284	604
ESC_TA3822AA	167	512
ESC_TA3821AA	167	512
ESC_TA3820AA	167	512
ESC_TA3819AA	1284	604
ESC_TA3818AA	167	512
ESC_TA3817AA	648	359
ESC_TA3816AA	361	512
ESC_TA3815AA	405	923
ESC_TA3814AA	354	15
ESC_TA3813AA	167	512
ESC_TA3812AA	1284	604
ESC_TA3811AA	354	15
ESC_TA3810AA	648	359
ESC_TA3809AA	1284	604
ESC_TA3808AA	405	923
ESC_TA3807AA	361	512
ESC_TA3806AA	10	512
ESC_TA3805AA	940	257
ESC_TA3804AA	940	257
ESC_TA3803AA	2851	156
ESC_TA3802AA	46	447
ESC_TA3801AA	167	512
ESC_TA3800AA	11638	447
ESC_TA3799AA	1718	269
ESC_TA3798AA	967	212

ESC_TA3797AA	10752	370
ESC_TA3796AA	2	900
ESC_TA3795AA	130	387
ESC_TA3791AA	410	156
ESC_TA3790AA	58	198
ESC_TA3789AA	101	696
ESC_TA3788AA	410	156
ESC_TA3787AA	1487	512
ESC_TA3786AA	617	511
ESC_TA3785AA	131	653
ESC_TA3783AA	38	669
ESC_TA3782AA	405	923
ESC_TA3780AA	38	669
ESC_TA3779AA	410	156
ESC_TA3778AA	167	512
ESC_TA3777AA	167	512
ESC_TA3776AA	410	156
ESC_TA3775AA	648	359
ESC_TA3774AA	648	359
ESC_TA3773AA	38	669
ESC_TA3772AA	8346	234
ESC_TA3771AA	361	512
ESC_TA3770AA	405	923
ESC_TA3769AA	38	669
ESC_TA3768AA	410	156
ESC_TA3767AA	10	512
ESC_TA3765AA	101	696
ESC_TA3764AA	10	512
ESC_TA3763AA	38	669
ESC_TA3762AA	1487	512
ESC_TA3761AA	940	257
ESC_TA3760AA	410	156
ESC_TA3759AA	410	156
ESC_TA3758AA	167	512
ESC_TA3757AA	410	156
ESC_TA3756AA	23	156
ESC_TA3755AA	1193	761
ESC_TA3754AA	617	511
ESC_TA3753AA	11	936
ESC_TA3752AA	11	936
ESC_TA3751AA	11	936
ESC_TA3750AA	11	936
ESC_TA3749AA	11	936
ESC_TA3748AA	11	936
ESC_TA3747AA	11	936
ESC_TA3746AA	11	936
ESC_TA3745AA	11	936
ESC_TA3744AA	11	936
ESC_TA3743AA	11	936
ESC_TA3742AA	11	936

ESC_TA3741AA	11	936
ESC_TA3740AA	11	936
ESC_TA3739AA	11	936
ESC_TA3738AA	11	936
ESC_TA3737AA	11	936
ESC_TA3736AA	11	936
ESC_TA3735AA	11	936
ESC_TA3734AA	11	936
ESC_TA3733AA	11	936
ESC_TA3732AA	11	936
ESC_TA3731AA	11	936
ESC_TA3730AA	11	936
ESC_TA3729AA	11	936
ESC_TA3728AA	11	936
ESC_TA3727AA	11	936
ESC_TA3726AA	11	936
ESC_TA3725AA	11	936
ESC_TA3724AA	11	936
ESC_TA3723AA	11	936
ESC_TA3722AA	11	936
ESC_TA3721AA	11	936
ESC_TA3720AA	11	936
ESC_TA3719AA	11	936
ESC_TA3718AA	11	936
ESC_TA3717AA	11	936
ESC_TA3716AA	11	936
ESC_TA3715AA	11	936
ESC_TA3714AA	11	936
ESC_TA3713AA	11	936
ESC_TA3712AA	11	936
ESC_TA3711AA	11	936
ESC_TA3710AA	11	936
ESC_TA3709AA	11	936
ESC_TA3708AA	11	936
ESC_TA3707AA	11	936
ESC_TA3706AA	11	936
ESC_TA3705AA	11	936
ESC_TA3704AA	11	936
ESC_TA3703AA	11	936
ESC_TA3702AA	11	936
ESC_TA3701AA	11	936
ESC_TA3700AA	11	936
ESC_TA3699AA	11	936
ESC_TA3698AA	11	936
ESC_TA3697AA	11	936
ESC_TA3696AA	11	936
ESC_TA3695AA	11	936
ESC_TA3694AA	11	936
ESC_TA3693AA	11	936
ESC_TA3692AA	11	936

ESC_TA3691AA	11	936
ESC_TA3690AA	11	936
ESC_TA3689AA	11	936
ESC_TA3688AA	11	936
ESC_TA3687AA	11	936
ESC_TA3686AA	11	936
ESC_TA3685AA	11	936
ESC_TA3684AA	11	936
ESC_TA3683AA	11	936
ESC_TA3682AA	11	936
ESC_TA3681AA	11	936
ESC_TA3680AA	11	936
ESC_TA3679AA	11	936
ESC_TA3678AA	11	936
ESC_TA3677AA	11	936
ESC_TA3676AA	11	936
ESC_TA3675AA	11	936
ESC_TA3674AA	11	936
ESC_TA3673AA	11	936
ESC_TA3672AA	11	936
ESC_TA3671AA	11	936
ESC_TA3670AA	11	936
ESC_TA3669AA	11	936
ESC_TA3668AA	11	936
ESC_TA3667AA	11	936
ESC_TA3666AA	11	936
ESC_TA3665AA	11	936
ESC_TA3664AA	11	936
ESC_TA3663AA	11	936
ESC_TA3662AA	11	936
ESC_TA3661AA	11	936
ESC_TA3660AA	11	936
ESC_TA3659AA	11	936
ESC_TA3658AA	11	936
ESC_TA3657AA	11	936
ESC_TA3656AA	11	936
ESC_TA3655AA	11	936
ESC_TA3654AA	11	936
ESC_TA3653AA	11	936
ESC_TA3652AA	11	936
ESC_TA3651AA	11	936
ESC_TA3650AA	11	936
ESC_TA3640AA	661	92
ESC_TA3639AA	3519	101
ESC_TA3638AA	661	92
ESC_TA3637AA	661	92
ESC_TA3636AA	661	92
ESC_TA3635AA	3519	101
ESC_TA3634AA	3519	101
ESC_TA3630AA	11	936

ESC_TA3629AA	23	156
ESC_TA3628AA	69	387
ESC_TA3627AA	88	160
ESC_TA3626AA	58	198
ESC_TA3625AA	10	512
ESC_TA3624AA	101	696
ESC_TA3623AA	38	669
ESC_TA3622AA	101	696
ESC_TA3621AA	11637	761
ESC_TA3620AA	446	257
ESC_TA3619AA	297	234
ESC_TA3618AA	297	234
ESC_TA3617AA	442	257
ESC_TA3616AA	11232	257
ESC_TA3615AA	297	234
ESC_TA3614AA	131	653
ESC_TA3613AA	648	359
ESC_TA3612AA	117	592
ESC_TA3611AA	10	512
ESC_TA3610AA	10	512
ESC_TA3531AA	442	257
ESC_TA3530AA	300	286
ESC_TA3529AA	5973	92
ESC_TA3528AA	5487	198
ESC_TA3527AA	5487	198
ESC_TA3526AA	345	175
ESC_TA3525AA	152	257
ESC_TA3524AA	152	257
ESC_TA3523AA	152	257
ESC_TA3522AA	152	257
ESC_TA3521AA	152	257
ESC_TA3520AA	145	256
ESC_TA3519AA	10596	311
ESC_TA3518AA	1304	257
ESC_TA3517AA	6917	261
ESC_TA3516AA	73	737
ESC_TA3515AA	11	936
ESC_TA3514AA	401	142
ESC_TA3513AA	25	234
ESC_TA3512AA	245	261
ESC_TA3511AA	245	261
ESC_TA3510AA	25	234
ESC_TA3509AA	25	234
ESC_TA3508AA	11636	198
ESC_TA3507AA	13	257
ESC_TA3506AA	129	257
ESC_TA3505AA	101	696
ESC_TA3504AA	681	393
ESC_TA3503AA	2562	640
ESC_TA3502AA	641	935

ESC_TA3501AA	603	198
ESC_TA3500AA	4663	257
ESC_TA3499AA	88	160
ESC_TA3498AA	3689	961
ESC_TA3497AA	2521	257
ESC_TA3496AA	48	76
ESC_TA3495AA	1727	257
ESC_TA3494AA	6031	108
ESC_TA3493AA	345	175
ESC_TA3492AA	1642	198
ESC_TA3491AA	38	669
ESC_TA3490AA	23	156
ESC_TA3489AA	17	191
ESC_TA3488AA	11	936
ESC_TA3487AA	11	936
ESC_TA3486AA	11	936
ESC_TA3485AA	2179	924
ESC_TA3484AA	44	512
ESC_TA3483AA	6738	76
ESC_TA3482AA	58	198
ESC_TA3481AA	1011	198
ESC_TA3480AA	3288	286
ESC_TA3478AA	746	789
ESC_TA3477AA	648	359
ESC_TA3476AA	224	198
ESC_TA3475AA	11634	512
ESC_TA3474AA	11634	512
ESC_TA3473AA	11021	512
ESC_TA3472AA	410	156
ESC_TA3471AA	410	156
ESC_TA3470AA	405	923
ESC_TA3469AA	167	512
ESC_TA3468AA	69	387
ESC_TA3467AA	410	156
ESC_TA3466AA	617	511
ESC_TA3465AA	131	653
ESC_TA3464AA	405	923
ESC_TA3463AA	410	156
ESC_TA3462AA	167	512
ESC_TA3461AA	744	512
ESC_TA3460AA	410	156
ESC_TA3459AA	405	923
ESC_TA3458AA	2659	679
ESC_TA3457AA	405	923
ESC_TA3456AA	405	923
ESC_TA3455AA	28	537
ESC_TA3454AA	448	257
ESC_TA3453AA	224	198
ESC_TA3452AA	410	156
ESC_TA3451AA	1113	221

ESC_TA3450AA	1113	221
ESC_TA3315AA	8427	257
ESC_TA3314AA	8427	257
ESC_TA3313AA	223	198
ESC_TA3312AA	205	257
ESC_TA3311AA	223	198
ESC_TA3310AA	223	198
ESC_TA3308AA	131	653
ESC_TA3307AA	38	669
ESC_TA3306AA	224	198
ESC_TA3305AA	11635	198
ESC_TA3304AA	3177	362
ESC_TA3303AA	5640	653
ESC_TA3302AA	69	387
ESC_TA3301AA	167	512
ESC_TA3300AA	405	923
ESC_TA3299AA	7366	824
ESC_TA3298AA	2521	257
ESC_TA3297AA	11021	512
ESC_TA3296AA	11021	512
ESC_TA3295AA	95	738
ESC_TA3294AA	1193	761
ESC_TA3293AA	11633	234
ESC_TA3292AA	245	261
ESC_TA3291AA	245	261
ESC_TA3290AA	245	261
ESC_TA3289AA	152	257
ESC_TA3288AA	7384	257
ESC_TA3287AA	245	261
ESC_TA3286AA	7384	257
ESC_TA3285AA	7384	257
ESC_TA3284AA	245	261
ESC_TA3283AA	245	261
ESC_TA3282AA	7384	257
ESC_TA3281AA	245	261
ESC_TA3280AA	245	261
ESC_TA3279AA	152	257
ESC_TA3278AA	245	261
ESC_TA3277AA	245	261
ESC_TA3276AA	245	261
ESC_TA3275AA	11060	198
ESC_TA3274AA	11	936
ESC_TA3273AA	675	234
ESC_TA3272AA	33	198
ESC_TA3271AA	21	156
ESC_TA3270AA	447	198
ESC_TA3269AA	398	445
ESC_TA3268AA	3101	160
ESC_TA3267AA	21	156
ESC_TA3266AA	3101	160

ESC_TA3265AA	342	286
ESC_TA3264AA	17	191
ESC_TA3263AA	101	696
ESC_TA3262AA	442	257
ESC_TA3261AA	657	592
ESC_TA3260AA	5487	198
ESC_TA3259AA	117	592
ESC_TA3258AA	2085	592
ESC_TA3257AA	2085	592
ESC_TA3256AA	2505	221
ESC_TA3255AA	23	156
ESC_TA3254AA	11632	198
ESC_TA3253AA	11632	198
ESC_TA3252AA	117	592
ESC_TA3251AA	11629	668
ESC_TA3250AA	117	592
ESC_TA3249AA	117	592
ESC_TA3248AA	117	592
ESC_TA3247AA	2847	212
ESC_TA3246AA	117	592
ESC_TA3243AA	23	156
ESC_TA3242AA	23	156
ESC_TA3241AA	2505	221
ESC_TA3240AA	224	198
ESC_TA3239AA	2505	221
ESC_TA3238AA	2505	221
ESC_TA3237AA	117	592
ESC_TA3236AA	93	71
ESC_TA3235AA	11631	156
ESC_TA3234AA	155	175
ESC_TA3233AA	23	156
ESC_TA3232AA	23	156
ESC_TA3231AA	95	738
ESC_TA3230AA	95	738
ESC_TA3229AA	11	936
ESC_TA3228AA	131	653
ESC_TA3227AA	44	512
ESC_TA3226AA	44	512
ESC_TA3225AA	1485	408
ESC_TA3224AA	410	156
ESC_TA3223AA	485	587
ESC_TA3222AA	2179	924
ESC_TA3221AA	44	512
ESC_TA3220AA	1011	198
ESC_TA3219AA	1589	156
ESC_TA3218AA	602	257
ESC_TA3217AA	602	257
ESC_TA3216AA	354	15
ESC_TA3215AA	155	175
ESC_TA3214AA	3941	71

ESC_TA3213AA	155	175
ESC_TA3212AA	1485	408
ESC_TA3211AA	405	923
ESC_TA3210AA	1431	257
ESC_TA3209AA	1431	257
ESC_TA3208AA	1431	257
ESC_TA3207AA	410	156
ESC_TA3206AA	162	924
ESC_TA3205AA	410	156
ESC_TA3204AA	410	156
ESC_TA3203AA	155	175
ESC_TA3202AA	3014	410
ESC_TA3201AA	48	76
ESC_TA3200AA	2179	924
ESC_TA3199AA	162	924
ESC_TA3198AA	2179	924
ESC_TA3197AA	11628	76
ESC_TA3196AA	602	257
ESC_TA3195AA	156	175
ESC_TA3194AA	410	156
ESC_TA3193AA	156	175
ESC_TA3192AA	354	15
ESC_TA3191AA	156	175
ESC_TA3190AA	602	257
ESC_TA3189AA	602	257
ESC_TA3188AA	602	257
ESC_TA3187AA	602	257
ESC_TA3186AA	602	257
ESC_TA3185AA	3944	512
ESC_TA3184AA	155	175
ESC_TA3183AA	354	15
ESC_TA3182AA	155	175
ESC_TA3181AA	602	257
ESC_TA3180AA	117	592
ESC_TA3179AA	165	504
ESC_TA3178AA	165	504
ESC_TA3177AA	8492	234
ESC_TA3176AA	224	198
ESC_TA3175AA	2491	512
ESC_TA3174AA	48	76
ESC_TA3173AA	155	175
ESC_TA3172AA	162	924
ESC_TA3171AA	224	198
ESC_TA3170AA	297	234
ESC_TA3169AA	658	684
ESC_TA3167AA	2385	198
ESC_TA3166AA	679	257
ESC_TA3165AA	3576	175
ESC_TA3164AA	297	234
ESC_TA3163AA	2387	261

ESC_TA3162AA	2505	221
ESC_TA3161AA	95	738
ESC_TA3160AA	359	696
ESC_TA3159AA	95	738
ESC_TA3158AA	3856	789
ESC_TA3157AA	2505	221
ESC_TA3156AA	162	924
ESC_TA3155AA	23	156
ESC_TA3154AA	2847	212
ESC_TA3153AA	2505	221
ESC_TA3152AA	155	175
ESC_TA3151AA	23	156
ESC_TA3150AA	23	156
ESC_TA3149AA	95	738
ESC_TA3148AA	23	156
ESC_TA3147AA	2847	212
ESC_TA3146AA	23	156
ESC_TA3145AA	23	156
ESC_TA3144AA	206	286
ESC_TA3143AA	117	592
ESC_TA3142AA	117	592
ESC_TA3141AA	2847	212
ESC_TA3140AA	2847	212
ESC_TA3139AA	2847	212
ESC_TA3138AA	117	592
ESC_TA3137AA	117	592
ESC_TA3136AA	117	592
ESC_TA3135AA	117	592
ESC_TA3134AA	117	592
ESC_TA3133AA	155	175
ESC_TA3131AA	224	198
ESC_TA3130AA	23	156
ESC_TA3129AA	117	592
ESC_TA3128AA	2309	669
ESC_TA3127AA	453	935
ESC_TA3126AA	23	156
ESC_TA3125AA	7153	263
ESC_TA3124AA	11630	175
ESC_TA3123AA	354	15
ESC_TA3122AA	410	156
ESC_TA3121AA	2505	221
ESC_TA3120AA	23	156
ESC_TA3119AA	2085	592
ESC_TA3118AA	2085	592
ESC_TA3117AA	117	592
ESC_TA3116AA	117	592
ESC_TA3115AA	23	156
ESC_TA3114AA	206	286
ESC_TA3113AA	23	156
ESC_TA3112AA	224	198

ESC_TA3111AA	648	359
ESC_TA3110AA	11629	668
ESC_TA3109AA	224	198
ESC_TA3108AA	224	198
ESC_TA3107AA	224	198
ESC_TA3106AA	224	198
ESC_TA3105AA	117	592
ESC_TA3104AA	156	175
ESC_TA3103AA	117	592
ESC_TA3102AA	2085	592
ESC_TA3101AA	2085	592
ESC_TA3100AA	2085	592
ESC_TA3099AA	224	198
ESC_TA3098AA	155	175
ESC_TA3097AA	155	175
ESC_TA3096AA	93	71
ESC_TA3095AA	117	592
ESC_TA3094AA	117	592
ESC_TA3093AA	2085	592
ESC_TA3092AA	2085	592
ESC_TA3091AA	95	738
ESC_TA3090AA	2505	221
ESC_TA3089AA	2505	221
ESC_TA3088AA	224	198
ESC_TA3087AA	6712	50
ESC_TA3086AA	2505	221
ESC_TA3085AA	2505	221
ESC_TA3084AA	224	198
ESC_TA3083AA	224	198
ESC_TA3082AA	224	198
ESC_TA3081AA	117	592
ESC_TA3080AA	224	198
ESC_TA3079AA	23	156
ESC_TA3078AA	23	156
ESC_TA3077AA	224	198
ESC_TA3076AA	131	653
ESC_TA3075AA	95	738
ESC_TA3074AA	117	592
ESC_TA3073AA	88	160
ESC_TA3072AA	48	76
ESC_TA3070AA	23	156
ESC_TA3069AA	23	156
ESC_TA3068AA	23	156
ESC_TA3067AA	963	669
ESC_TA3066AA	131	653
ESC_TA3065AA	2179	924
ESC_TA3064AA	48	76
ESC_TA3063AA	410	156
ESC_TA3062AA	2179	924
ESC_TA3061AA	2179	924

ESC_TA3060AA	359	696
ESC_TA3059AA	1431	257
ESC_TA3058AA	2179	924
ESC_TA3057AA	1642	198
ESC_TA3056AA	3107	512
ESC_TA3055AA	658	684
ESC_TA3054AA	1490	935
ESC_TA3053AA	205	257
ESC_TA3052AA	205	257
ESC_TA3051AA	205	257
ESC_TA3050AA	205	257
ESC_TA3049AA	8257	657
ESC_TA3048AA	11021	512
ESC_TA3047AA	3576	175
ESC_TA3046AA	679	257
ESC_TA3045AA	679	257
ESC_TA3044AA	5487	198
ESC_TA3043AA	5487	198
ESC_TA3042AA	679	257
ESC_TA3041AA	2385	198
ESC_TA3040AA	301	307
ESC_TA3039AA	205	257
ESC_TA3038AA	410	156
ESC_TA3037AA	301	307
ESC_TA3036AA	301	307
ESC_TA3035AA	301	307
ESC_TA3034AA	301	307
ESC_TA3033AA	301	307
ESC_TA3032AA	301	307
ESC_TA3031AA	301	307
ESC_TA3030AA	301	307
ESC_TA3029AA	301	307
ESC_TA3028AA	301	307
ESC_TA3027AA	301	307
ESC_TA3026AA	301	307
ESC_TA3025AA	301	307
ESC_TA3024AA	301	307
ESC_TA3023AA	245	261
ESC_TA3022AA	1236	380
ESC_TA3021AA	205	257
ESC_TA3020AA	297	234
ESC_TA3019AA	205	257
ESC_TA3018AA	21	156
ESC_TA3017AA	205	257
ESC_TA3016AA	205	257
ESC_TA3015AA	205	257
ESC_TA3014AA	205	257
ESC_TA3013AA	205	257
ESC_TA3012AA	205	257
ESC_TA3011AA	205	257

ESC_TA3010AA	10	512
ESC_TA3009AA	131	653
ESC_TA3008AA	8257	657
ESC_TA3007AA	11627	1019
ESC_TA3006AA	1193	761
ESC_TA3005AA	131	653
ESC_TA3004AA	2556	630
ESC_TA3003AA	131	653
ESC_TA3002AA	131	653
ESC_TA3001AA	11021	512
ESC_TA3000AA	301	307
ESC_TA2999AA	301	307
ESC_TA2998AA	301	307
ESC_TA2997AA	301	307
ESC_TA2996AA	658	684
ESC_TA2995AA	205	257
ESC_TA2994AA	11	936
ESC_TA2993AA	1804	935
ESC_TA2992AA	11	936
ESC_TA2991AA	11	936
ESC_TA2990AA	11	936
ESC_TA2989AA	11	936
ESC_TA2988AA	11	936
ESC_TA2987AA	11	936
ESC_TA2986AA	11	936
ESC_TA2985AA	11	936
ESC_TA2984AA	11	936
ESC_TA2983AA	11	936
ESC_TA2982AA	11626	257
ESC_TA2981AA	11625	1017
ESC_TA2980AA	48	76
ESC_TA2979AA	602	257
ESC_TA2978AA	301	307
ESC_TA2977AA	11	936
ESC_TA2976AA	11624	936
ESC_TA2975AA	152	257
ESC_TA2972AA	152	257
ESC_TA2971AA	152	257
ESC_TA2970AA	245	261
ESC_TA2969AA	245	261
ESC_TA2968AA	11623	1021
ESC_TA2967AA	245	261
ESC_TA2966AA	245	261
ESC_TA2965AA	5592	830
ESC_TA2964AA	1839	84
ESC_TA2963AA	300	286
ESC_TA2962AA	1839	84
ESC_TA2961AA	11471	121
ESC_TA2960AA	11608	85
ESC_TA2959AA	2682	87

ESC_TA2958AA	675	234
ESC_TA2957AA	6057	82
ESC_TA2956AA	1839	84
ESC_TA2955AA	11622	123
ESC_TA2954AA	1839	84
ESC_TA2953AA	442	257
ESC_TA2952AA	7851	463
ESC_TA2951AA	1839	84
ESC_TA2950AA	11608	85
ESC_TA2949AA	11608	85
ESC_TA2948AA	5399	87
ESC_TA2947AA	4633	118
ESC_TA2946AA	5399	87
ESC_TA2945AA	11387	653
ESC_TA2944AA	11380	653
ESC_TA2943AA	10	512
ESC_TA2942AA	679	257
ESC_TA2941AA	679	257
ESC_TA2940AA	679	257
ESC_TA2939AA	1431	257
ESC_TA2938AA	1431	257
ESC_TA2937AA	1431	257
ESC_TA2936AA	1431	257
ESC_TA2935AA	4038	257
ESC_TA2934AA	442	257
ESC_TA2933AA	152	257
ESC_TA2932AA	152	257
ESC_TA2931AA	152	257
ESC_TA2930AA	301	307
ESC_TA2929AA	301	307
ESC_TA2928AA	301	307
ESC_TA2927AA	301	307
ESC_TA2926AA	301	307
ESC_TA2925AA	301	307
ESC_TA2924AA	301	307
ESC_TA2923AA	301	307
ESC_TA2922AA	301	307
ESC_TA2921AA	301	307
ESC_TA2920AA	301	307
ESC_TA2919AA	11620	108
ESC_TA2918AA	3595	822
ESC_TA2917AA	11619	445
ESC_TA2916AA	542	824
ESC_TA2915AA	542	824
ESC_TA2914AA	137	270
ESC_TA2913AA	9965	445
ESC_TA2912AA	73	737
ESC_TA2911AA	409	504
ESC_TA2910AA	10	512
ESC_TA2909AA	69	387

ESC_TA2908AA	410	156
ESC_TA2907AA	405	923
ESC_TA2906AA	1284	604
ESC_TA2905AA	224	198
ESC_TA2904AA	410	156
ESC_TA2903AA	140	738
ESC_TA2902AA	10	512
ESC_TA2901AA	10634	257
ESC_TA2900AA	7616	257
ESC_TA2899AA	540	286
ESC_TA2898AA	428	654
ESC_TA2897AA	795	924
ESC_TA2896AA	69	387
ESC_TA2895AA	101	696
ESC_TA2894AA	117	592
ESC_TA2893AA	212	198
ESC_TA2892AA	2307	198
ESC_TA2891AA	11621	198
ESC_TA2890AA	398	445
ESC_TA2889AA	4198	878
ESC_TA2888AA	3595	822
ESC_TA2887AA	1	667
ESC_TA2886AA	20	198
ESC_TA2885AA	542	824
ESC_TA2884AA	871	447
ESC_TA2883AA	3595	822
ESC_TA2882AA	165	504
ESC_TA2881AA	993	789
ESC_TA2880AA	11618	985
ESC_TA2879AA	93	71
ESC_TA2878AA	993	789
ESC_TA2877AA	542	824
ESC_TA2876AA	10	512
ESC_TA2875AA	93	71
ESC_TA2874AA	10	512
ESC_TA2873AA	11617	71
ESC_TA2872AA	10	512
ESC_TA2871AA	763	878
ESC_TA2870AA	10	512
ESC_TA2869AA	100	504
ESC_TA2868AA	93	71
ESC_TA2867AA	542	824
ESC_TA2866AA	542	824
ESC_TA2865AA	542	824
ESC_TA2864AA	10	512
ESC_TA2863AA	23	156
ESC_TA2862AA	8421	261
ESC_TA2861AA	8421	261
ESC_TA2860AA	8421	261
ESC_TA2859AA	23	156

ESC_TA2858AA	23	156
ESC_TA2857AA	23	156
ESC_TA2856AA	23	156
ESC_TA2855AA	542	824
ESC_TA2854AA	542	824
ESC_TA2853AA	542	824
ESC_TA2852AA	10	512
ESC_TA2851AA	23	156
ESC_TA2850AA	10	512
ESC_TA2849AA	154	257
ESC_TA2848AA	10	512
ESC_TA2847AA	542	824
ESC_TA2846AA	448	257
ESC_TA2845AA	746	789
ESC_TA2844AA	189	504
ESC_TA2843AA	871	447
ESC_TA2842AA	542	824
ESC_TA2841AA	5995	504
ESC_TA2840AA	1081	198
ESC_TA2839AA	48	76
ESC_TA2838AA	11616	512
ESC_TA2837AA	10	512
ESC_TA2836AA	398	445
ESC_TA2834AA	410	156
ESC_TA2833AA	636	653
ESC_TA2832AA	410	156
ESC_TA2831AA	11613	886
ESC_TA2830AA	11613	886
ESC_TA2828AA	11612	519
ESC_TA2826AA	11611	512
ESC_TA2825AA	354	15
ESC_TA2824AA	131	653
ESC_TA2823AA	678	257
ESC_TA2822AA	131	653
ESC_TA2821AA	410	156
ESC_TA2820AA	131	653
ESC_TA2819AA	10	512
ESC_TA2818AA	410	156
ESC_TA2817AA	10	512
ESC_TA2816AA	405	923
ESC_TA2815AA	424	234
ESC_TA2814AA	361	512
ESC_TA2813AA	5229	696
ESC_TA2812AA	101	696
ESC_TA2811AA	657	592
ESC_TA2810AA	410	156
ESC_TA2809AA	361	512
ESC_TA2808AA	95	738
ESC_TA2806AA	410	156
ESC_TA2805AA	410	156

ESC_TA2804AA	648	359
ESC_TA2803AA	410	156
ESC_TA2802AA	405	923
ESC_TA2801AA	617	511
ESC_TA2800AA	131	653
ESC_TA2799AA	131	653
ESC_TA2798AA	131	653
ESC_TA2797AA	69	387
ESC_TA2796AA	131	653
ESC_TA2795AA	11615	923
ESC_TA2794AA	224	198
ESC_TA2793AA	117	592
ESC_TA2792AA	410	156
ESC_TA2791AA	410	156
ESC_TA2790AA	11	936
ESC_TA2789AA	167	512
ESC_TA2788AA	405	923
ESC_TA2787AA	457	595
ESC_TA2786AA	405	923
ESC_TA2785AA	167	512
ESC_TA2784AA	410	156
ESC_TA2783AA	131	653
ESC_TA2782AA	131	653
ESC_TA2781AA	131	653
ESC_TA2780AA	410	156
ESC_TA2779AA	131	653
ESC_TA2778AA	218	519
ESC_TA2777AA	131	653
ESC_TA2776AA	131	653
ESC_TA2775AA	648	359
ESC_TA2774AA	410	156
ESC_TA2773AA	131	653
ESC_TA2772AA	617	511
ESC_TA2771AA	131	653
ESC_TA2770AA	131	653
ESC_TA2769AA	410	156
ESC_TA2768AA	131	653
ESC_TA2767AA	155	175
ESC_TA2766AA	131	653
ESC_TA2765AA	410	156
ESC_TA2764AA	6332	153
ESC_TA2763AA	410	156
ESC_TA2762AA	2448	234
ESC_TA2760AA	405	923
ESC_TA2759AA	410	156
ESC_TA2758AA	405	923
ESC_TA2757AA	405	923
ESC_TA2756AA	448	257
ESC_TA2755AA	448	257
ESC_TA2754AA	2011	362

ESC_TA2753AA	2011	362
ESC_TA2752AA	405	923
ESC_TA2751AA	648	359
ESC_TA2750AA	167	512
ESC_TA2749AA	127	738
ESC_TA2748AA	131	653
ESC_TA2747AA	405	923
ESC_TA2746AA	448	257
ESC_TA2745AA	405	923
ESC_TA2744AA	2659	679
ESC_TA2743AA	38	669
ESC_TA2742AA	361	512
ESC_TA2741AA	131	653
ESC_TA2740AA	410	156
ESC_TA2739AA	405	923
ESC_TA2738AA	617	511
ESC_TA2737AA	405	923
ESC_TA2736AA	405	923
ESC_TA2735AA	405	923
ESC_TA2734AA	101	696
ESC_TA2630AA	152	257
ESC_TA2629AA	245	261
ESC_TA2628AA	7384	257
ESC_TA2627AA	245	261
ESC_TA2626AA	21	156
ESC_TA2625AA	21	156
ESC_TA2624AA	21	156
ESC_TA2623AA	301	307
ESC_TA2622AA	641	935
ESC_TA2621AA	641	935
ESC_TA2620AA	11610	156
ESC_TA2619AA	11471	121
ESC_TA2618AA	4736	87
ESC_TA2617AA	5268	86
ESC_TA2616AA	11608	85
ESC_TA2615AA	11608	85
ESC_TA2614AA	2682	87
ESC_TA2613AA	2967	289
ESC_TA2612AA	56	198
ESC_TA2611AA	11471	121
ESC_TA2610AA	11471	121
ESC_TA2609AA	10	512
ESC_TA2608AA	6048	87
ESC_TA2607AA	4748	234
ESC_TA2606AA	2967	289
ESC_TA2605AA	6048	87
ESC_TA2604AA	101	696
ESC_TA2603AA	401	142
ESC_TA2602AA	117	592
ESC_TA2601AA	1288	512

ESC_TA2600AA	11614	198
ESC_TA2599AA	973	968
ESC_TA2598AA	770	587
ESC_TA2597AA	906	198
ESC_TA2596AA	641	935
ESC_TA2559AA	11	936
ESC_TA2558AA	405	923
ESC_TA2557AA	167	512
ESC_TA2556AA	156	175
ESC_TA2555AA	156	175
ESC_TA2554AA	617	511
ESC_TA2553AA	10	512
ESC_TA2552AA	297	234
ESC_TA2551AA	297	234
ESC_TA2550AA	10	512
ESC_TA2549AA	1056	257
ESC_TA2548AA	11	936
ESC_TA2547AA	11231	198
ESC_TA2545AA	11231	198
ESC_TA2544AA	11231	198
ESC_TA2543AA	11231	198
ESC_TA2542AA	11231	198
ESC_TA2541AA	11231	198
ESC_TA2540AA	58	198
ESC_TA2539AA	58	198
ESC_TA2538AA	58	198
ESC_TA2537AA	54	256
ESC_TA2536AA	152	257
ESC_TA2535AA	152	257
ESC_TA2534AA	11606	261
ESC_TA2533AA	357	654
ESC_TA2532AA	11	936
ESC_TA2531AA	32	270
ESC_TA2530AA	32	270
ESC_TA2529AA	32	270
ESC_TA2528AA	32	270
ESC_TA2527AA	32	270
ESC_TA2526AA	32	270
ESC_TA2525AA	32	270
ESC_TA2524AA	32	270
ESC_TA2523AA	32	270
ESC_TA2522AA	32	270
ESC_TA2521AA	342	286
ESC_TA2520AA	32	270
ESC_TA2519AA	137	270
ESC_TA2518AA	32	270
ESC_TA2517AA	32	270
ESC_TA2516AA	32	270
ESC_TA2515AA	32	270
ESC_TA2514AA	342	286

ESC_TA2513AA	32	270
ESC_TA2512AA	32	270
ESC_TA2511AA	32	270
ESC_TA2510AA	342	286
ESC_TA2509AA	32	270
ESC_TA2508AA	32	270
ESC_TA2507AA	32	270
ESC_TA2506AA	342	286
ESC_TA2505AA	32	270
ESC_TA2504AA	32	270
ESC_TA2503AA	11605	627
ESC_TA2502AA	410	156
ESC_TA2501AA	245	261
ESC_TA2500AA	245	261
ESC_TA2499AA	245	261
ESC_TA2498AA	245	261
ESC_TA2497AA	245	261
ESC_TA2496AA	245	261
ESC_TA2495AA	245	261
ESC_TA2492AA	5292	738
ESC_TA2491AA	12	626
ESC_TA2490AA	442	257
ESC_TA2489AA	17	191
ESC_TA2488AA	11471	121
ESC_TA2487AA	4736	87
ESC_TA2486AA	11471	121
ESC_TA2485AA	4170	87
ESC_TA2484AA	25	234
ESC_TA2483AA	536	141
ESC_TA2482AA	144	740
ESC_TA2481AA	11609	3
ESC_TA2480AA	11471	121
ESC_TA2479AA	2703	87
ESC_TA2478AA	11608	85
ESC_TA2477AA	327	204
ESC_TA2476AA	11471	121
ESC_TA2475AA	11607	56
ESC_TA2474AA	11	936
ESC_TA2473AA	11471	121
ESC_TA2472AA	11471	121
ESC_TA2471AA	11471	121
ESC_TA2470AA	11608	85
ESC_TA2464AA	95	738
ESC_TA2459AA	906	198
ESC_TA2458AA	1721	311
ESC_TA2457AA	8246	696
ESC_TA2456AA	2531	198
ESC_TA2455AA	131	653
ESC_TA2454AA	538	629
ESC_TA2453AA	5891	878

ESC_TA2452AA	135	653
ESC_TA2451AA	95	738
ESC_TA2450AA	2726	175
ESC_TA2449AA	57	50
ESC_TA2448AA	297	234
ESC_TA2447AA	5971	979
ESC_TA2446AA	278	924
ESC_TA2445AA	155	175
ESC_TA2444AA	3580	257
ESC_TA2443AA	5378	197
ESC_TA2442AA	11604	936
ESC_TA2441AA	155	175
ESC_TA2440AA	1434	512
ESC_TA2439AA	355	764
ESC_TA2438AA	442	257
ESC_TA2437AA	145	256
ESC_TA2436AA	21	156
ESC_TA2435AA	11471	121
ESC_TA2434AA	4606	117
ESC_TA2433AA	11	936
ESC_TA2432AA	5967	87
ESC_TA2431AA	33	198
ESC_TA2430AA	11478	85
ESC_TA2429AA	4736	87
ESC_TA2428AA	11603	924
ESC_TA2427AA	11471	121
ESC_TA2426AA	11	936
ESC_TA2425AA	4736	87
ESC_TA2424AA	4748	234
ESC_TA2423AA	34	504
ESC_TA2422AA	21	156
ESC_TA2421AA	675	234
ESC_TA2420AA	33	198
ESC_TA2419AA	34	504
ESC_TA2418AA	21	156
ESC_TA2417AA	442	257
ESC_TA2416AA	33	198
ESC_TA2415AA	245	261
ESC_TA2414AA	148	191
ESC_TA2412AA	301	307
ESC_TA2411AA	301	307
ESC_TA2410AA	21	156
ESC_TA2409AA	399	198
ESC_TA2408AA	6354	447
ESC_TA2407AA	90	156
ESC_TA2406AA	90	156
ESC_TA2405AA	10	512
ESC_TA2404AA	86	935
ESC_TA2403AA	11231	198
ESC_TA2402AA	910	929

ESC_TA2401AA	86	935
ESC_TA2400AA	11600	257
ESC_TA2399AA	245	261
ESC_TA2398AA	245	261
ESC_TA2397AA	11602	645
ESC_TA2396AA	25	234
ESC_TA2395AA	442	257
ESC_TA2394AA	718	257
ESC_TA2393AA	342	286
ESC_TA2392AA	32	270
ESC_TA2391AA	25	234
ESC_TA2390AA	93	71
ESC_TA2389AA	93	71
ESC_TA2388AA	93	71
ESC_TA2387AA	93	71
ESC_TA2386AA	93	71
ESC_TA2385AA	93	71
ESC_TA2384AA	93	71
ESC_TA2383AA	93	71
ESC_TA2382AA	93	71
ESC_TA2381AA	93	71
ESC_TA2379AA	93	71
ESC_TA2377AA	93	71
ESC_TA2376AA	93	71
ESC_TA2375AA	93	71
ESC_TA2374AA	93	71
ESC_TA2373AA	93	71
ESC_TA2372AA	93	71
ESC_TA2371AA	10	512
ESC_TA2370AA	11599	144
ESC_TA2369AA	542	824
ESC_TA2368AA	4408	286
ESC_TA2367AA	11599	144
ESC_TA2366AA	10	512
ESC_TA2365AA	3595	822
ESC_TA2364AA	10	512
ESC_TA2363AA	10	512
ESC_TA2362AA	398	445
ESC_TA2361AA	542	824
ESC_TA2360AA	11416	504
ESC_TA2350AA	410	156
ESC_TA2349AA	117	592
ESC_TA2348AA	88	160
ESC_TA2347AA	11415	458
ESC_TA2346AA	3595	822
ESC_TA2345AA	4379	878
ESC_TA2344AA	4379	878
ESC_TA2343AA	4379	878
ESC_TA2342AA	10	512
ESC_TA2341AA	4379	878

ESC_TA2340AA	4379	878
ESC_TA2339AA	4379	878
ESC_TA2338AA	4379	878
ESC_TA2337AA	11416	504
ESC_TA2336AA	4379	878
ESC_TA2335AA	11416	504
ESC_TA2334AA	2514	504
ESC_TA2333AA	11416	504
ESC_TA2332AA	11416	504
ESC_TA2331AA	398	445
ESC_TA2330AA	398	445
ESC_TA2329AA	398	445
ESC_TA2328AA	208	76
ESC_TA2327AA	4379	878
ESC_TA2326AA	7329	175
ESC_TA2325AA	4379	878
ESC_TA2324AA	4379	878
ESC_TA2323AA	4379	878
ESC_TA2322AA	4379	878
ESC_TA2321AA	4379	878
ESC_TA2320AA	4379	878
ESC_TA2319AA	398	445
ESC_TA2318AA	10	512
ESC_TA2317AA	398	445
ESC_TA2316AA	398	445
ESC_TA2315AA	398	445
ESC_TA2314AA	398	445
ESC_TA2313AA	398	445
ESC_TA2312AA	4379	878
ESC_TA2311AA	4379	878
ESC_TA2310AA	4379	878
ESC_TA2309AA	4379	878
ESC_TA2308AA	4379	878
ESC_TA2307AA	3595	822
ESC_TA2306AA	4379	878
ESC_TA2305AA	772	76
ESC_TA2304AA	3595	822
ESC_TA2303AA	3595	822
ESC_TA2302AA	3595	822
ESC_TA2301AA	3595	822
ESC_TA2300AA	3595	822
ESC_TA2299AA	3595	822
ESC_TA2298AA	3595	822
ESC_TA2297AA	3595	822
ESC_TA2296AA	3595	822
ESC_TA2294AA	11601	512
ESC_TA2293AA	131	653
ESC_TA2292AA	517	924
ESC_TA2291AA	404	761
ESC_TA2290AA	58	198

ESC_TA2289AA	131	653
ESC_TA2288AA	69	387
ESC_TA2287AA	95	738
ESC_TA2286AA	297	234
ESC_TA2285AA	1049	175
ESC_TA2284AA	1049	175
ESC_TA2283AA	101	696
ESC_TA2282AA	131	653
ESC_TA2281AA	101	696
ESC_TA2280AA	38	669
ESC_TA2279AA	38	669
ESC_TA2278AA	58	198
ESC_TA2277AA	58	198
ESC_TA2276AA	404	761
ESC_TA2275AA	58	198
ESC_TA2274AA	131	653
ESC_TA2273AA	245	261
ESC_TA2272AA	245	261
ESC_TA2271AA	11	936
ESC_TA2270AA	11	936
ESC_TA2269AA	11	936
ESC_TA2268AA	21	156
ESC_TA2267AA	11	936
ESC_TA2266AA	11	936
ESC_TA2265AA	10988	283
ESC_TA2264AA	1041	765
ESC_TA2263AA	11	936
ESC_TA2262AA	152	257
ESC_TA2261AA	245	261
ESC_TA2260AA	245	261
ESC_TA2259AA	245	261
ESC_TA2258AA	245	261
ESC_TA2257AA	152	257
ESC_TA2256AA	245	261
ESC_TA2255AA	245	261
ESC_TA2254AA	245	261
ESC_TA2253AA	152	257
ESC_TA2252AA	152	257
ESC_TA2251AA	245	261
ESC_TA2250AA	8878	261
ESC_TA2249AA	301	307
ESC_TA2247AA	11590	587
ESC_TA2246AA	11590	587
ESC_TA2245AA	152	257
ESC_TA2244AA	245	261
ESC_TA2239AA	32	270
ESC_TA2238AA	442	257
ESC_TA2237AA	21	156
ESC_TA2236AA	11	936
ESC_TA2235AA	25	234

ESC_TA2234AA	11598	254
ESC_TA2233AA	583	768
ESC_TA2232AA	327	204
ESC_TA2231AA	21	156
ESC_TA2230AA	32	270
ESC_TA2229AA	11	936
ESC_TA2227AA	131	653
ESC_TA2226AA	131	653
ESC_TA2225AA	617	511
ESC_TA2224AA	131	653
ESC_TA2223AA	2599	257
ESC_TA2222AA	448	257
ESC_TA2221AA	410	156
ESC_TA2220AA	617	511
ESC_TA2219AA	648	359
ESC_TA2218AA	156	175
ESC_TA2217AA	716	795
ESC_TA2216AA	410	156
ESC_TA2215AA	131	653
ESC_TA2214AA	167	512
ESC_TA2213AA	354	15
ESC_TA2212AA	174	660
ESC_TA2211AA	6332	153
ESC_TA2210AA	6332	153
ESC_TA2209AA	648	359
ESC_TA2208AA	6359	473
ESC_TA2207AA	162	924
ESC_TA2206AA	410	156
ESC_TA2205AA	410	156
ESC_TA2204AA	2006	198
ESC_TA2203AA	38	669
ESC_TA2202AA	405	923
ESC_TA2201AA	155	175
ESC_TA2200AA	410	156
ESC_TA2197AA	354	15
ESC_TA2196AA	10	512
ESC_TA2195AA	73	737
ESC_TA2194AA	2006	198
ESC_TA2193AA	156	175
ESC_TA2192AA	410	156
ESC_TA2191AA	131	653
ESC_TA2190AA	450	71
ESC_TA2189AA	38	669
ESC_TA2188AA	205	257
ESC_TA2187AA	617	511
ESC_TA2186AA	156	175
ESC_TA2185AA	167	512
ESC_TA2184AA	131	653
ESC_TA2183AA	410	156
ESC_TA2182AA	648	359

ESC_TA2179AA	410	156
ESC_TA2178AA	410	156
ESC_TA2177AA	216	512
ESC_TA2176AA	10	512
ESC_TA2175AA	1136	108
ESC_TA2174AA	4166	936
ESC_TA2173AA	216	512
ESC_TA2172AA	410	156
ESC_TA2171AA	205	257
ESC_TA2170AA	410	156
ESC_TA2169AA	131	653
ESC_TA2168AA	617	511
ESC_TA2167AA	69	387
ESC_TA2165AA	127	738
ESC_TA2164AA	410	156
ESC_TA2163AA	648	359
ESC_TA2162AA	410	156
ESC_TA2161AA	998	644
ESC_TA2160AA	2521	257
ESC_TA2159AA	1162	394
ESC_TA2158AA	442	257
ESC_TA2157AA	5082	198
ESC_TA2156AA	223	198
ESC_TA2155AA	164	175
ESC_TA2154AA	906	198
ESC_TA2153AA	641	935
ESC_TA2152AA	21	156
ESC_TA2151AA	167	512
ESC_TA2150AA	86	935
ESC_TA2149AA	117	592
ESC_TA2148AA	603	198
ESC_TA2147AA	167	512
ESC_TA2146AA	29	156
ESC_TA2145AA	141	644
ESC_TA2144AA	56	198
ESC_TA2143AA	5082	198
ESC_TA2142AA	2521	257
ESC_TA2141AA	11594	198
ESC_TA2140AA	164	175
ESC_TA2139AA	677	257
ESC_TA2138AA	11589	198
ESC_TA2137AA	58	198
ESC_TA2136AA	1317	740
ESC_TA2135AA	973	968
ESC_TA2134AA	5082	198
ESC_TA2133AA	297	234
ESC_TA2132AA	196	257
ESC_TA2131AA	1967	191
ESC_TA2130AA	10	512
ESC_TA2129AA	83	740

ESC_TA2128AA	641	935
ESC_TA2127AA	1771	868
ESC_TA2126AA	446	257
ESC_TA2125AA	609	447
ESC_TA2124AA	8464	743
ESC_TA2123AA	10	512
ESC_TA2122AA	11	936
ESC_TA2121AA	10	512
ESC_TA2120AA	2217	62
ESC_TA2119AA	223	198
ESC_TA2118AA	10	512
ESC_TA2117AA	515	1004
ESC_TA2116AA	131	653
ESC_TA2115AA	162	924
ESC_TA2114AA	4516	445
ESC_TA2113AA	10	512
ESC_TA2112AA	648	359
ESC_TA2111AA	23	156
ESC_TA2110AA	4198	878
ESC_TA2109AA	11	936
ESC_TA2108AA	58	198
ESC_TA2107AA	648	359
ESC_TA2106AA	10	512
ESC_TA2105AA	156	175
ESC_TA2104AA	56	198
ESC_TA2103AA	410	156
ESC_TA2102AA	131	653
ESC_TA2101AA	23	156
ESC_TA2100AA	641	935
ESC_TA2099AA	23	156
ESC_TA2098AA	6579	156
ESC_TA2097AA	117	592
ESC_TA2096AA	48	76
ESC_TA2095AA	88	160
ESC_TA2094AA	1201	512
ESC_TA2093AA	2522	198
ESC_TA2092AA	88	160
ESC_TA2091AA	218	519
ESC_TA2090AA	10	512
ESC_TA2089AA	1685	257
ESC_TA2088AA	11	936
ESC_TA2087AA	3107	512
ESC_TA2086AA	2522	198
ESC_TA2085AA	58	198
ESC_TA2084AA	156	175
ESC_TA2083AA	131	653
ESC_TA2082AA	11	936
ESC_TA2081AA	446	257
ESC_TA2080AA	117	592
ESC_TA2079AA	9063	315

ESC_TA2078AA	69	387
ESC_TA2077AA	11593	198
ESC_TA2076AA	6610	175
ESC_TA2075AA	746	789
ESC_TA2074AA	355	764
ESC_TA2073AA	6841	160
ESC_TA2072AA	58	198
ESC_TA2071AA	1721	311
ESC_TA2070AA	34	504
ESC_TA2069AA	12	626
ESC_TA2068AA	2168	878
ESC_TA2066AA	10	512
ESC_TA2065AA	34	504
ESC_TA2064AA	162	924
ESC_TA2063AA	10	512
ESC_TA2062AA	3107	512
ESC_TA2061AA	48	76
ESC_TA2060AA	648	359
ESC_TA2059AA	11592	763
ESC_TA2058AA	117	592
ESC_TA2057AA	5309	286
ESC_TA2056AA	457	595
ESC_TA2055AA	155	175
ESC_TA2054AA	58	198
ESC_TA2053AA	657	592
ESC_TA2052AA	998	644
ESC_TA2051AA	657	592
ESC_TA2050AA	11591	1027
ESC_TA2049AA	10	512
ESC_TA2048AA	12	626
ESC_TA2047AA	10	512
ESC_TA2046AA	1252	567
ESC_TA2045AA	117	592
ESC_TA2044AA	11597	597
ESC_TA2043AA	847	257
ESC_TA2042AA	2035	113
ESC_TA2041AA	446	257
ESC_TA2040AA	6617	519
ESC_TA2039AA	963	669
ESC_TA2038AA	21	156
ESC_TA2037AA	117	592
ESC_TA2036AA	10	512
ESC_TA2035AA	973	968
ESC_TA2034AA	602	257
ESC_TA2033AA	224	198
ESC_TA2032AA	457	595
ESC_TA2031AA	86	935
ESC_TA2030AA	2346	435
ESC_TA2029AA	1252	567
ESC_TA2028AA	973	968

ESC_TA2027AA	11596	257
ESC_TA2026AA	11595	696
ESC_TA2025AA	162	924
ESC_TA2024AA	88	160
ESC_TA2023AA	442	257
ESC_TA2022AA	58	198
ESC_TA2021AA	10	512
ESC_TA2020AA	88	160
ESC_TA2019AA	1308	257
ESC_TA2018AA	2035	113
ESC_TA2017AA	388	156
ESC_TA2016AA	362	234
ESC_TA2015AA	10	512
ESC_TA2014AA	117	592
ESC_TA2013AA	58	198
ESC_TA2012AA	2449	234
ESC_TA2011AA	131	653
ESC_TA2010AA	131	653
ESC_TA2009AA	131	653
ESC_TA2008AA	131	653
ESC_TA2007AA	617	511
ESC_TA2006AA	155	175
ESC_TA2005AA	652	204
ESC_TA2004AA	131	653
ESC_TA2003AA	131	653
ESC_TA2002AA	131	653
ESC_TA2001AA	131	653
ESC_TA2000AA	131	653
ESC_TA1999AA	69	387
ESC_TA1998AA	167	512
ESC_TA1997AA	424	234
ESC_TA1996AA	2006	198
ESC_TA1995AA	131	653
ESC_TA1994AA	648	359
ESC_TA1993AA	131	653
ESC_TA1992AA	101	696
ESC_TA1991AA	131	653
ESC_TA1990AA	131	653
ESC_TA1989AA	131	653
ESC_TA1988AA	998	644
ESC_TA1987AA	2705	504
ESC_TA1986AA	410	156
ESC_TA1985AA	744	512
ESC_TA1984AA	3018	903
ESC_TA1983AA	2006	198
ESC_TA1982AA	101	696
ESC_TA1981AA	101	696
ESC_TA1980AA	410	156
ESC_TA1979AA	744	512
ESC_TA1978AA	101	696

ESC_TA1977AA	652	204
ESC_TA1976AA	131	653
ESC_TA1975AA	405	923
ESC_TA1974AA	131	653
ESC_TA1973AA	648	359
ESC_TA1972AA	648	359
ESC_TA1971AA	11	936
ESC_TA1970AA	747	640
ESC_TA1969AA	11	936
ESC_TA1968AA	11	936
ESC_TA1967AA	747	640
ESC_TA1966AA	11	936
ESC_TA1965AA	216	512
ESC_TA1964AA	154	257
ESC_TA1963AA	2425	257
ESC_TA1962AA	297	234
ESC_TA1961AA	1201	512
ESC_TA1960AA	401	142
ESC_TA1959AA	58	198
ESC_TA1958AA	538	629
ESC_TA1956AA	772	76
ESC_TA1955AA	355	764
ESC_TA1954AA	58	198
ESC_TA1953AA	7906	198
ESC_TA1952AA	11	936
ESC_TA1951AA	11	936
ESC_TA1950AA	11	936
ESC_TA1949AA	11	936
ESC_TA1948AA	11	936
ESC_TA1947AA	11	936
ESC_TA1946AA	11	936
ESC_TA1945AA	11	936
ESC_TA1944AA	11	936
ESC_TA1943AA	11	936
ESC_TA1942AA	11	936
ESC_TA1941AA	11	936
ESC_TA1940AA	11	936
ESC_TA1939AA	11	936
ESC_TA1938AA	11	936
ESC_TA1937AA	11	936
ESC_TA1936AA	11	936
ESC_TA1935AA	11	936
ESC_TA1934AA	11	936
ESC_TA1933AA	11	936
ESC_TA1932AA	11	936
ESC_TA1931AA	11	936
ESC_TA1930AA	11	936
ESC_TA1929AA	11	936
ESC_TA1928AA	11	936
ESC_TA1927AA	11	936

ESC_TA1926AA	11	936
ESC_TA1925AA	11	936
ESC_TA1924AA	224	198
ESC_TA1923AA	694	512
ESC_TA1922AA	6726	493
ESC_TA1921AA	245	261
ESC_TA1920AA	10	512
ESC_TA1919AA	32	270
ESC_TA1918AA	327	204
ESC_TA1917AA	6265	233
ESC_TA1916AA	590	156
ESC_TA1915AA	2569	107
ESC_TA1914AA	223	198
ESC_TA1913AA	75	257
ESC_TA1912AA	297	234
ESC_TA1911AA	297	234
ESC_TA1909AA	75	257
ESC_TA1901AA	11389	261
ESC_TA1894AA	11	936
ESC_TA1870AA	11	936
ESC_TA1866AA	11	936
ESC_TA1865AA	301	307
ESC_TA1864AA	11	936
ESC_TA1863AA	11	936
ESC_TA1862AA	11	936
ESC_TA1861AA	11	936
ESC_TA1860AA	21	156
ESC_TA1859AA	11	936
ESC_TA1858AA	21	156
ESC_TA1857AA	135	653
ESC_TA1854AA	135	653
ESC_TA1853AA	135	653
ESC_TA1852AA	135	653
ESC_TA1851AA	135	653
ESC_TA1850AA	135	653
ESC_TA1849AA	135	653
ESC_TA1848AA	135	653
ESC_TA1847AA	135	653
ESC_TA1846AA	135	653
ESC_TA1845AA	135	653
ESC_TA1844AA	135	653
ESC_TA1843AA	135	653
ESC_TA1842AA	135	653
ESC_TA1838AA	135	653
ESC_TA1837AA	135	653
ESC_TA1835AA	11	936
ESC_TA1834AA	21	156
ESC_TA1833AA	11	936
ESC_TA1832AA	17	191
ESC_TA1831AA	32	270

ESC_TA1830AA	17	191
ESC_TA1829AA	32	270
ESC_TA1828AA	32	270
ESC_TA1827AA	21	156
ESC_TA1825AA	10	512
ESC_TA1824AA	33	198
ESC_TA1823AA	1735	737
ESC_TA1822AA	1196	257
ESC_TA1821AA	646	738
ESC_TA1820AA	2558	782
ESC_TA1819AA	372	424
ESC_TA1818AA	372	424
ESC_TA1817AA	12	626
ESC_TA1816AA	11	936
ESC_TA1815AA	11	936
ESC_TA1814AA	11	936
ESC_TA1813AA	11	936
ESC_TA1812AA	11	936
ESC_TA1811AA	11	936
ESC_TA1810AA	11	936
ESC_TA1809AA	11	936
ESC_TA1808AA	11	936
ESC_TA1806AA	11588	512
ESC_TA1805AA	197	601
ESC_TA1804AA	216	512
ESC_TA1803AA	1972	406
ESC_TA1802AA	1972	406
ESC_TA1801AA	349	670
ESC_TA1800AA	224	198
ESC_TA1799AA	224	198
ESC_TA1798AA	1817	842
ESC_TA1797AA	6228	257
ESC_TA1796AA	641	935
ESC_TA1795AA	6228	257
ESC_TA1793AA	216	512
ESC_TA1792AA	216	512
ESC_TA1791AA	224	198
ESC_TA1790AA	1148	257
ESC_TA1789AA	155	175
ESC_TA1788AA	1148	257
ESC_TA1787AA	155	175
ESC_TA1786AA	1148	257
ESC_TA1785AA	1148	257
ESC_TA1784AA	155	175
ESC_TA1783AA	1049	175
ESC_TA1782AA	1148	257
ESC_TA1781AA	1148	257
ESC_TA1780AA	1148	257
ESC_TA1779AA	1148	257
ESC_TA1778AA	156	175

ESC_TA1777AA	1423	198
ESC_TA1776AA	155	175
ESC_TA1775AA	155	175
ESC_TA1774AA	200	198
ESC_TA1773AA	155	175
ESC_TA1772AA	155	175
ESC_TA1771AA	200	198
ESC_TA1770AA	200	198
ESC_TA1769AA	155	175
ESC_TA1768AA	155	175
ESC_TA1767AA	155	175
ESC_TA1766AA	155	175
ESC_TA1765AA	11587	924
ESC_TA1764AA	200	198
ESC_TA1763AA	1914	956
ESC_TA1762AA	2522	198
ESC_TA1761AA	2522	198
ESC_TA1760AA	1079	235
ESC_TA1759AA	11586	512
ESC_TA1758AA	11585	198
ESC_TA1757AA	2593	653
ESC_TA1756AA	11585	198
ESC_TA1754AA	2593	653
ESC_TA1753AA	2593	653
ESC_TA1752AA	2593	653
ESC_TA1751AA	2593	653
ESC_TA1750AA	2593	653
ESC_TA1749AA	2593	653
ESC_TA1748AA	7361	156
ESC_TA1747AA	2593	653
ESC_TA1746AA	2593	653
ESC_TA1745AA	2593	653
ESC_TA1744AA	2593	653
ESC_TA1743AA	2593	653
ESC_TA1742AA	2593	653
ESC_TA1741AA	11585	198
ESC_TA1740AA	154	257
ESC_TA1739AA	3606	204
ESC_TA1738AA	58	198
ESC_TA1736AA	58	198
ESC_TA1735AA	345	175
ESC_TA1734AA	58	198
ESC_TA1733AA	11584	981
ESC_TA1732AA	4382	900
ESC_TA1731AA	11583	55
ESC_TA1730AA	737	175
ESC_TA1728AA	4382	900
ESC_TA1727AA	4382	900
ESC_TA1726AA	11582	1013
ESC_TA1725AA	442	257

ESC_TA1724AA	442	257
ESC_TA1723AA	226	512
ESC_TA1722AA	442	257
ESC_TA1719AA	180	234
ESC_TA1718AA	226	512
ESC_TA1717AA	1079	235
ESC_TA1716AA	5523	445
ESC_TA1715AA	2280	212
ESC_TA1714AA	2076	400
ESC_TA1713AA	5474	198
ESC_TA1712AA	38	669
ESC_TA1710AA	362	234
ESC_TA1709AA	345	175
ESC_TA1708AA	11581	1026
ESC_TA1707AA	345	175
ESC_TA1706AA	180	234
ESC_TA1705AA	10	512
ESC_TA1704AA	11580	935
ESC_TA1703AA	11579	198
ESC_TA1702AA	301	307
ESC_TA1701AA	301	307
ESC_TA1700AA	11577	307
ESC_TA1699AA	301	307
ESC_TA1698AA	301	307
ESC_TA1697AA	301	307
ESC_TA1696AA	301	307
ESC_TA1695AA	301	307
ESC_TA1694AA	301	307
ESC_TA1693AA	301	307
ESC_TA1692AA	11575	926
ESC_TA1691AA	1250	926
ESC_TA1690AA	6984	156
ESC_TA1689AA	58	198
ESC_TA1688AA	4552	926
ESC_TA1687AA	11575	926
ESC_TA1686AA	11574	257
ESC_TA1685AA	4527	880
ESC_TA1684AA	826	926
ESC_TA1683AA	58	198
ESC_TA1682AA	11576	924
ESC_TA1681AA	2936	515
ESC_TA1680AA	11568	926
ESC_TA1679AA	362	234
ESC_TA1678AA	1250	926
ESC_TA1677AA	1250	926
ESC_TA1675AA	906	198
ESC_TA1674AA	11570	926
ESC_TA1673AA	11571	935
ESC_TA1672AA	826	926
ESC_TA1671AA	11573	925

ESC_TA1670AA	86	935
ESC_TA1669AA	11123	926
ESC_TA1668AA	1611	198
ESC_TA1667AA	826	926
ESC_TA1666AA	11572	926
ESC_TA1665AA	6986	926
ESC_TA1664AA	2035	113
ESC_TA1663AA	10	512
ESC_TA1662AA	11568	926
ESC_TA1661AA	1250	926
ESC_TA1660AA	1250	926
ESC_TA1659AA	826	926
ESC_TA1658AA	11569	926
ESC_TA1657AA	826	926
ESC_TA1656AA	10	512
ESC_TA1655AA	1250	926
ESC_TA1654AA	4552	926
ESC_TA1653AA	1245	270
ESC_TA1652AA	1250	926
ESC_TA1651AA	10	512
ESC_TA1650AA	1250	926
ESC_TA1649AA	1250	926
ESC_TA1648AA	1250	926
ESC_TA1647AA	1245	270
ESC_TA1646AA	1250	926
ESC_TA1645AA	1586	198
ESC_TA1643AA	11568	926
ESC_TA1642AA	32	270
ESC_TA1641AA	11	936
ESC_TA1640AA	11	936
ESC_TA1639AA	677	257
ESC_TA1638AA	11	936
ESC_TA1637AA	11	936
ESC_TA1636AA	752	512
ESC_TA1635AA	32	270
ESC_TA1634AA	11	936
ESC_TA1633AA	32	270
ESC_TA1632AA	11	936
ESC_TA1631AA	32	270
ESC_TA1630AA	11567	156
ESC_TA1629AA	11	936
ESC_TA1628AA	306	286
ESC_TA1627AA	11	936
ESC_TA1626AA	21	156
ESC_TA1625AA	17	191
ESC_TA1624AA	11	936
ESC_TA1623AA	11	936
ESC_TA1622AA	11	936
ESC_TA1621AA	11	936
ESC_TA1620AA	11	936

ESC_TA1619AA	11	936
ESC_TA1618AA	11	936
ESC_TA1617AA	11	936
ESC_TA1616AA	11	936
ESC_TA1615AA	11	936
ESC_TA1614AA	11	936
ESC_TA1613AA	583	768
ESC_TA1612AA	11	936
ESC_TA1611AA	11	936
ESC_TA1610AA	8412	696
ESC_TA1609AA	196	257
ESC_TA1608AA	156	175
ESC_TA1607AA	162	924
ESC_TA1606AA	297	234
ESC_TA1605AA	1125	198
ESC_TA1604AA	297	234
ESC_TA1603AA	162	924
ESC_TA1602AA	162	924
ESC_TA1601AA	162	924
ESC_TA1600AA	131	653
ESC_TA1599AA	131	653
ESC_TA1598AA	11	936
ESC_TA1597AA	10082	504
ESC_TA1596AA	10082	504
ESC_TA1595AA	141	644
ESC_TA1594AA	141	644
ESC_TA1593AA	141	644
ESC_TA1592AA	141	644
ESC_TA1591AA	141	644
ESC_TA1590AA	141	644
ESC_TA1589AA	70	670
ESC_TA1588AA	162	924
ESC_TA1587AA	17	191
ESC_TA1586AA	58	198
ESC_TA1585AA	10	512
ESC_TA1583AA	21	156
ESC_TA1580AA	127	738
ESC_TA1579AA	127	738
ESC_TA1578AA	245	261
ESC_TA1577AA	245	261
ESC_TA1576AA	245	261
ESC_TA1575AA	245	261
ESC_TA1574AA	152	257
ESC_TA1573AA	245	261
ESC_TA1572AA	152	257
ESC_TA1571AA	245	261
ESC_TA1570AA	245	261
ESC_TA1569AA	152	257
ESC_TA1568AA	245	261
ESC_TA1567AA	152	257

ESC_TA1566AA	245	261
ESC_TA1565AA	327	204
ESC_TA1564AA	21	156
ESC_TA1563AA	300	286
ESC_TA1557AA	38	669
ESC_TA1556AA	38	669
ESC_TA1555AA	38	669
ESC_TA1554AA	38	669
ESC_TA1553AA	69	387
ESC_TA1552AA	38	669
ESC_TA1551AA	69	387
ESC_TA1550AA	38	669
ESC_TA1549AA	38	669
ESC_TA1548AA	38	669
ESC_TA1547AA	38	669
ESC_TA1546AA	38	669
ESC_TA1545AA	38	669
ESC_TA1544AA	38	669
ESC_TA1543AA	38	669
ESC_TA1542AA	38	669
ESC_TA1541AA	38	669
ESC_TA1540AA	10	512
ESC_TA1539AA	10	512
ESC_TA1538AA	38	669
ESC_TA1537AA	38	669
ESC_TA1536AA	38	669
ESC_TA1535AA	38	669
ESC_TA1534AA	38	669
ESC_TA1533AA	38	669
ESC_TA1532AA	38	669
ESC_TA1531AA	38	669
ESC_TA1530AA	38	669
ESC_TA1529AA	38	669
ESC_TA1528AA	38	669
ESC_TA1527AA	38	669
ESC_TA1526AA	38	669
ESC_TA1525AA	1551	40
ESC_TA1524AA	38	669
ESC_TA1523AA	38	669
ESC_TA1522AA	38	669
ESC_TA1521AA	38	669
ESC_TA1520AA	38	669
ESC_TA1519AA	38	669
ESC_TA1518AA	38	669
ESC_TA1517AA	1594	71
ESC_TA1516AA	38	669
ESC_TA1515AA	38	669
ESC_TA1514AA	38	669
ESC_TA1513AA	10	512
ESC_TA1512AA	38	669

ESC_TA1511AA	38	669
ESC_TA1510AA	38	669
ESC_TA1509AA	38	669
ESC_TA1508AA	38	669
ESC_TA1507AA	38	669
ESC_TA1506AA	38	669
ESC_TA1505AA	38	669
ESC_TA1504AA	38	669
ESC_TA1503AA	38	669
ESC_TA1502AA	38	669
ESC_TA1501AA	38	669
ESC_TA1500AA	38	669
ESC_TA1499AA	38	669
ESC_TA1498AA	38	669
ESC_TA1497AA	1594	71
ESC_TA1496AA	115	669
ESC_TA1495AA	297	234
ESC_TA1494AA	117	592
ESC_TA1492AA	117	592
ESC_TA1491AA	117	592
ESC_TA1490AA	38	669
ESC_TA1489AA	1158	387
ESC_TA1488AA	38	669
ESC_TA1487AA	117	592
ESC_TA1486AA	297	234
ESC_TA1485AA	1158	387
ESC_TA1484AA	1158	387
ESC_TA1483AA	191	198
ESC_TA1482AA	1158	387
ESC_TA1481AA	38	669
ESC_TA1480AA	115	669
ESC_TA1479AA	38	669
ESC_TA1478AA	10	512
ESC_TA1477AA	1158	387
ESC_TA1476AA	115	669
ESC_TA1475AA	38	669
ESC_TA1474AA	38	669
ESC_TA1473AA	1158	387
ESC_TA1472AA	1158	387
ESC_TA1471AA	1944	934
ESC_TA1470AA	10	512
ESC_TA1469AA	115	669
ESC_TA1468AA	38	669
ESC_TA1467AA	38	669
ESC_TA1466AA	38	669
ESC_TA1465AA	1594	71
ESC_TA1464AA	429	654
ESC_TA1463AA	10	512
ESC_TA1462AA	429	654
ESC_TA1461AA	442	257

ESC_TA1460AA	2040	198
ESC_TA1459AA	2040	198
ESC_TA1458AA	2040	198
ESC_TA1457AA	2040	198
ESC_TA1456AA	2040	198
ESC_TA1455AA	2040	198
ESC_TA1454AA	2040	198
ESC_TA1453AA	2040	198
ESC_TA1452AA	2040	198
ESC_TA1451AA	2040	198
ESC_TA1450AA	11578	71
ESC_TA1449AA	1158	387
ESC_TA1448AA	117	592
ESC_TA1447AA	11578	71
ESC_TA1446AA	117	592
ESC_TA1445AA	38	669
ESC_TA1444AA	117	592
ESC_TA1443AA	11578	71
ESC_TA1442AA	38	669
ESC_TA1441AA	1158	387
ESC_TA1440AA	429	654
ESC_TA1439AA	2040	198
ESC_TA1438AA	38	669
ESC_TA1437AA	38	669
ESC_TA1436AA	38	669
ESC_TA1435AA	3249	156
ESC_TA1434AA	38	669
ESC_TA1433AA	1158	387
ESC_TA1432AA	38	669
ESC_TA1431AA	1158	387
ESC_TA1430AA	1158	387
ESC_TA1429AA	38	669
ESC_TA1428AA	38	669
ESC_TA1427AA	1158	387
ESC_TA1426AA	1158	387
ESC_TA1425AA	131	653
ESC_TA1424AA	38	669
ESC_TA1423AA	38	669
ESC_TA1422AA	57	50
ESC_TA1421AA	355	764
ESC_TA1420AA	355	764
ESC_TA1419AA	355	764
ESC_TA1418AA	355	764
ESC_TA1417AA	355	764
ESC_TA1416AA	2569	107
ESC_TA1415AA	6318	286
ESC_TA1414AA	40	198
ESC_TA1413AA	442	257
ESC_TA1412AA	12	626
ESC_TA1411AA	753	842

ESC_TA1410AA	11	936
ESC_TA1409AA	83	740
ESC_TA1408AA	32	270
ESC_TA1407AA	300	286
ESC_TA1406AA	11566	230
ESC_TA1405AA	5418	257
ESC_TA1404AA	5418	257
ESC_TA1399AA	675	234
ESC_TA1398AA	1819	808
ESC_TA1397AA	7641	738
ESC_TA1396AA	11	936
ESC_TA1395AA	11	936
ESC_TA1394AA	354	15
ESC_TA1393AA	10	512
ESC_TA1392AA	93	71
ESC_TA1391AA	10	512
ESC_TA1390AA	744	512
ESC_TA1389AA	1011	198
ESC_TA1388AA	117	592
ESC_TA1387AA	1485	408
ESC_TA1386AA	48	76
ESC_TA1385AA	93	71
ESC_TA1384AA	457	595
ESC_TA1383AA	457	595
ESC_TA1382AA	48	76
ESC_TA1381AA	770	587
ESC_TA1380AA	997	915
ESC_TA1379AA	7941	504
ESC_TA1378AA	457	595
ESC_TA1377AA	48	76
ESC_TA1376AA	457	595
ESC_TA1375AA	93	71
ESC_TA1374AA	1011	198
ESC_TA1373AA	10	512
ESC_TA1372AA	162	924
ESC_TA1371AA	93	71
ESC_TA1370AA	48	76
ESC_TA1369AA	48	76
ESC_TA1368AA	457	595
ESC_TA1367AA	746	789
ESC_TA1366AA	1011	198
ESC_TA1365AA	115	669
ESC_TA1364AA	115	669
ESC_TA1363AA	117	592
ESC_TA1362AA	10	512
ESC_TA1361AA	10	512
ESC_TA1360AA	6856	76
ESC_TA1359AA	117	592
ESC_TA1358AA	1011	198
ESC_TA1357AA	117	592

ESC_TA1356AA	359	696
ESC_TA1355AA	117	592
ESC_TA1354AA	349	670
ESC_TA1353AA	189	504
ESC_TA1352AA	162	924
ESC_TA1351AA	10	512
ESC_TA1350AA	206	286
ESC_TA1349AA	48	76
ESC_TA1348AA	11546	198
ESC_TA1347AA	206	286
ESC_TA1346AA	93	71
ESC_TA1345AA	115	669
ESC_TA1344AA	43	512
ESC_TA1343AA	5687	71
ESC_TA1341AA	69	387
ESC_TA1340AA	69	387
ESC_TA1339AA	2521	257
ESC_TA1338AA	1308	257
ESC_TA1337AA	58	198
ESC_TA1336AA	602	257
ESC_TA1335AA	58	198
ESC_TA1334AA	641	935
ESC_TA1333AA	223	198
ESC_TA1332AA	88	160
ESC_TA1331AA	88	160
ESC_TA1330AA	3057	672
ESC_TA1329AA	58	198
ESC_TA1328AA	29	156
ESC_TA1327AA	69	387
ESC_TA1326AA	533	198
ESC_TA1325AA	69	387
ESC_TA1324AA	106	387
ESC_TA1323AA	88	160
ESC_TA1322AA	174	660
ESC_TA1321AA	21	156
ESC_TA1320AA	69	387
ESC_TA1319AA	106	387
ESC_TA1318AA	362	234
ESC_TA1317AA	362	234
ESC_TA1316AA	322	453
ESC_TA1315AA	156	175
ESC_TA1314AA	11544	257
ESC_TA1313AA	10	512
ESC_TA1312AA	1011	198
ESC_TA1311AA	1011	198
ESC_TA1310AA	48	76
ESC_TA1309AA	48	76
ESC_TA1308AA	189	504
ESC_TA1307AA	302	850
ESC_TA1306AA	117	592

ESC_TA1305AA	354	15
ESC_TA1304AA	48	76
ESC_TA1303AA	10	512
ESC_TA1302AA	189	504
ESC_TA1301AA	359	696
ESC_TA1300AA	6448	175
ESC_TA1299AA	1968	76
ESC_TA1298AA	770	587
ESC_TA1297AA	9567	156
ESC_TA1296AA	457	595
ESC_TA1295AA	48	76
ESC_TA1294AA	457	595
ESC_TA1293AA	156	175
ESC_TA1292AA	10	512
ESC_TA1291AA	93	71
ESC_TA1290AA	117	592
ESC_TA1289AA	1140	934
ESC_TA1288AA	93	71
ESC_TA1287AA	354	15
ESC_TA1286AA	11545	592
ESC_TA1285AA	10	512
ESC_TA1284AA	354	15
ESC_TA1283AA	997	915
ESC_TA1282AA	746	789
ESC_TA1281AA	10	512
ESC_TA1280AA	48	76
ESC_TA1279AA	115	669
ESC_TA1278AA	93	71
ESC_TA1277AA	10	512
ESC_TA1276AA	189	504
ESC_TA1275AA	11544	257
ESC_TA1274AA	117	592
ESC_TA1273AA	117	592
ESC_TA1272AA	115	669
ESC_TA1271AA	162	924
ESC_TA1270AA	1011	198
ESC_TA1269AA	48	76
ESC_TA1268AA	117	592
ESC_TA1267AA	117	592
ESC_TA1266AA	6	445
ESC_TA1265AA	152	257
ESC_TA1264AA	152	257
ESC_TA1263AA	252	190
ESC_TA1262AA	152	257
ESC_TA1261AA	152	257
ESC_TA1260AA	152	257
ESC_TA1259AA	245	261
ESC_TA1258AA	245	261
ESC_TA1257AA	152	257
ESC_TA1256AA	152	257

ESC_TA1255AA	11	936
ESC_TA1254AA	301	307
ESC_TA1253AA	301	307
ESC_TA1252AA	301	307
ESC_TA1251AA	301	307
ESC_TA1250AA	21	156
ESC_TA1249AA	21	156
ESC_TA1248AA	10	512
ESC_TA1247AA	10	512
ESC_TA1246AA	10	512
ESC_TA1245AA	10	512
ESC_TA1244AA	10	512
ESC_TA1243AA	10	512
ESC_TA1242AA	10	512
ESC_TA1241AA	10	512
ESC_TA1240AA	10	512
ESC_TA1239AA	10	512
ESC_TA1238AA	10	512
ESC_TA1237AA	10	512
ESC_TA1236AA	10	512
ESC_TA1235AA	10	512
ESC_TA1234AA	10	512
ESC_TA1233AA	10	512
ESC_TA1232AA	10	512
ESC_TA1231AA	10	512
ESC_TA1230AA	10	512
ESC_TA1229AA	10	512
ESC_TA1228AA	10	512
ESC_TA1227AA	10	512
ESC_TA1226AA	10	512
ESC_TA1225AA	10	512
ESC_TA1224AA	10	512
ESC_TA1223AA	10	512
ESC_TA1222AA	10	512
ESC_TA1221AA	10	512
ESC_TA1220AA	10	512
ESC_TA1218AA	10	512
ESC_TA1217AA	10	512
ESC_TA1216AA	10	512
ESC_TA1215AA	10	512
ESC_TA1214AA	10	512
ESC_TA1213AA	10	512
ESC_TA1212AA	10	512
ESC_TA1211AA	10	512
ESC_TA1210AA	744	512
ESC_TA1208AA	10	512
ESC_TA1207AA	10	512
ESC_TA1206AA	11552	512
ESC_TA1205AA	10	512
ESC_TA1204AA	10	512

ESC_TA1203AA	10	512
ESC_TA1202AA	10	512
ESC_TA1201AA	10	512
ESC_TA1200AA	10	512
ESC_TA1199AA	11551	512
ESC_TA1198AA	10	512
ESC_TA1197AA	10	512
ESC_TA1196AA	10	512
ESC_TA1195AA	10	512
ESC_TA1194AA	10	512
ESC_TA1193AA	10	512
ESC_TA1192AA	10	512
ESC_TA1191AA	10	512
ESC_TA1190AA	10	512
ESC_TA1189AA	10	512
ESC_TA1188AA	10	512
ESC_TA1187AA	10	512
ESC_TA1186AA	10	512
ESC_TA1185AA	10	512
ESC_TA1184AA	10	512
ESC_TA1183AA	11550	512
ESC_TA1182AA	10	512
ESC_TA1181AA	10	512
ESC_TA1180AA	11553	512
ESC_TA1179AA	11549	512
ESC_TA1178AA	11548	512
ESC_TA1176AA	10	512
ESC_TA1174AA	10	512
ESC_TA1173AA	10	512
ESC_TA1172AA	10	512
ESC_TA1171AA	10	512
ESC_TA1170AA	10	512
ESC_TA1169AA	10	512
ESC_TA1168AA	10	512
ESC_TA1167AA	10	512
ESC_TA1166AA	10	512
ESC_TA1165AA	10	512
ESC_TA1164AA	10	512
ESC_TA1163AA	10	512
ESC_TA1162AA	10	512
ESC_TA1161AA	10	512
ESC_TA1160AA	10	512
ESC_TA1159AA	10	512
ESC_TA1158AA	10	512
ESC_TA1157AA	10	512
ESC_TA1156AA	10	512
ESC_TA1155AA	10	512
ESC_TA1154AA	11547	512
ESC_TA1153AA	10	512
ESC_TA1152AA	11564	512

ESC_TA1151AA	10	512
ESC_TA1149AA	10	512
ESC_TA1148AA	10	512
ESC_TA1147AA	10	512
ESC_TA1146AA	10	512
ESC_TA1145AA	10	512
ESC_TA1144AA	10	512
ESC_TA1142AA	10	512
ESC_TA1141AA	11563	512
ESC_TA1140AA	10	512
ESC_TA1139AA	10	512
ESC_TA1138AA	11562	512
ESC_TA1137AA	11561	512
ESC_TA1136AA	10	512
ESC_TA1135AA	10	512
ESC_TA1134AA	10	512
ESC_TA1133AA	10	512
ESC_TA1132AA	10	512
ESC_TA1131AA	10	512
ESC_TA1130AA	10	512
ESC_TA1129AA	10	512
ESC_TA1128AA	10	512
ESC_TA1126AA	10	512
ESC_TA1125AA	10	512
ESC_TA1124AA	10	512
ESC_TA1123AA	10	512
ESC_TA1122AA	10	512
ESC_TA1119AA	11560	512
ESC_TA1117AA	10	512
ESC_TA1116AA	10	512
ESC_TA1115AA	10	512
ESC_TA1114AA	10	512
ESC_TA1113AA	10	512
ESC_TA1112AA	10	512
ESC_TA1111AA	975	512
ESC_TA1110AA	10	512
ESC_TA1109AA	10	512
ESC_TA1108AA	10	512
ESC_TA1107AA	10	512
ESC_TA1106AA	10	512
ESC_TA1105AA	10	512
ESC_TA1104AA	10	512
ESC_TA1103AA	10	512
ESC_TA1102AA	10	512
ESC_TA1101AA	10	512
ESC_TA1100AA	10	512
ESC_TA1099AA	10	512
ESC_TA1097AA	10	512
ESC_TA1096AA	10	512
ESC_TA1095AA	10	512

ESC_TA1094AA	10	512
ESC_TA1093AA	10	512
ESC_TA1092AA	10	512
ESC_TA1091AA	10	512
ESC_TA1090AA	10	512
ESC_TA1089AA	10	512
ESC_TA1088AA	10	512
ESC_TA1087AA	10	512
ESC_TA1086AA	10	512
ESC_TA1085AA	10	512
ESC_TA1084AA	11558	512
ESC_TA1082AA	11559	512
ESC_TA1081AA	10	512
ESC_TA1080AA	10	512
ESC_TA1079AA	11557	512
ESC_TA1078AA	10	512
ESC_TA1076AA	11556	512
ESC_TA1075AA	11555	474
ESC_TA1074AA	10	512
ESC_TA1073AA	10	512
ESC_TA1072AA	10	512
ESC_TA1070AA	11554	512
ESC_TA1069AA	10	512
ESC_TA1068AA	10	512
ESC_TA1067AA	10	512
ESC_TA1066AA	10	512
ESC_TA1064AA	10	512
ESC_TA1063AA	10	512
ESC_TA1062AA	10	512
ESC_TA1061AA	10	512
ESC_TA1060AA	10	512
ESC_TA1059AA	10	512
ESC_TA1058AA	10	512
ESC_TA1057AA	10	512
ESC_TA1056AA	10	512
ESC_TA1055AA	10	512
ESC_TA1054AA	10	512
ESC_TA1053AA	10	512
ESC_TA1052AA	10	512
ESC_TA1051AA	11	936
ESC_TA1050AA	11	936
ESC_TA1049AA	11	936
ESC_TA1048AA	11	936
ESC_TA1047AA	11	936
ESC_TA1046AA	11	936
ESC_TA1045AA	11	936
ESC_TA1044AA	11	936
ESC_TA1043AA	11	936
ESC_TA1042AA	9019	257
ESC_TA1041AA	446	257

ESC_TA1040AA	5978	924
ESC_TA1039AA	5559	257
ESC_TA1038AA	11	936
ESC_TA1037AA	5978	924
ESC_TA1036AA	5978	924
ESC_TA1035AA	5978	924
ESC_TA1034AA	5559	257
ESC_TA1033AA	5978	924
ESC_TA1032AA	5559	257
ESC_TA1031AA	5978	924
ESC_TA1030AA	5978	924
ESC_TA1029AA	5978	924
ESC_TA1028AA	5559	257
ESC_TA1027AA	11	936
ESC_TA1026AA	5978	924
ESC_TA1025AA	5559	257
ESC_TA1024AA	5978	924
ESC_TA1023AA	5978	924
ESC_TA1022AA	5978	924
ESC_TA1021AA	5978	924
ESC_TA1020AA	5978	924
ESC_TA1019AA	5559	257
ESC_TA1018AA	5559	257
ESC_TA1017AA	5978	924
ESC_TA1016AA	11	936
ESC_TA1015AA	5978	924
ESC_TA1014AA	5559	257
ESC_TA1013AA	5978	924
ESC_TA1012AA	5978	924
ESC_TA1011AA	5978	924
ESC_TA1010AA	5978	924
ESC_TA1009AA	5559	257
ESC_TA1008AA	5978	924
ESC_TA1007AA	5978	924
ESC_TA1006AA	5978	924
ESC_TA1005AA	11	936
ESC_TA1004AA	11	936
ESC_TA1003AA	11	936
ESC_TA1002AA	11	936
ESC_TA1001AA	11	936
ESC_TA1000AA	11	936
ESC_TA0999AA	11	936
ESC_TA0998AA	11	936
ESC_TA0997AA	11	936
ESC_TA0996AA	11	936
ESC_TA0995AA	11	936
ESC_TA0994AA	11	936
ESC_TA0993AA	11	936
ESC_TA0992AA	11	936
ESC_TA0991AA	11	936

ESC_TA0990AA	11	936
ESC_TA0989AA	11	936
ESC_TA0988AA	11	936
ESC_TA0987AA	11	936
ESC_TA0986AA	11	936
ESC_TA0985AA	11	936
ESC_TA0984AA	11	936
ESC_TA0983AA	11	936
ESC_TA0982AA	11	936
ESC_TA0981AA	11	936
ESC_TA0980AA	11	936
ESC_TA0979AA	11	936
ESC_TA0978AA	11	936
ESC_TA0977AA	11	936
ESC_TA0976AA	11	936
ESC_TA0975AA	11	936
ESC_TA0974AA	11	936
ESC_TA0973AA	11	936
ESC_TA0972AA	11	936
ESC_TA0971AA	11	936
ESC_TA0970AA	11	936
ESC_TA0969AA	11	936
ESC_TA0968AA	11	936
ESC_TA0967AA	11	936
ESC_TA0966AA	11	936
ESC_TA0965AA	11	936
ESC_TA0964AA	11	936
ESC_TA0963AA	11	936
ESC_TA0962AA	11	936
ESC_TA0961AA	11	936
ESC_TA0960AA	11	936
ESC_TA0959AA	11	936
ESC_TA0958AA	11	936
ESC_TA0957AA	11	936
ESC_TA0956AA	11	936
ESC_TA0955AA	11	936
ESC_TA0954AA	11	936
ESC_TA0953AA	11	936
ESC_TA0952AA	11	936
ESC_TA0951AA	11	936
ESC_TA0950AA	11	936
ESC_TA0949AA	11	936
ESC_TA0948AA	11	936
ESC_TA0947AA	11	936
ESC_TA0946AA	11	936
ESC_TA0945AA	11	936
ESC_TA0944AA	11	936
ESC_TA0943AA	11	936
ESC_TA0942AA	11	936
ESC_TA0941AA	11	936

ESC_TA0940AA	11	936
ESC_TA0939AA	11	936
ESC_TA0938AA	11	936
ESC_TA0937AA	11	936
ESC_TA0936AA	11	936
ESC_TA0935AA	11	936
ESC_TA0934AA	11	936
ESC_TA0933AA	11	936
ESC_TA0932AA	11	936
ESC_TA0931AA	11	936
ESC_TA0930AA	11	936
ESC_TA0929AA	11	936
ESC_TA0928AA	11	936
ESC_TA0927AA	11	936
ESC_TA0926AA	11	936
ESC_TA0925AA	11	936
ESC_TA0924AA	11	936
ESC_TA0923AA	11	936
ESC_TA0922AA	11	936
ESC_TA0921AA	11	936
ESC_TA0920AA	11	936
ESC_TA0919AA	11	936
ESC_TA0918AA	11	936
ESC_TA0917AA	11	936
ESC_TA0916AA	11	936
ESC_TA0915AA	11	936
ESC_TA0914AA	11	936
ESC_TA0913AA	11	936
ESC_TA0912AA	11	936
ESC_TA0407AA	69	387
ESC_TA0406AA	69	387
ESC_TA0405AA	500	71
ESC_TA0404AA	69	387
ESC_TA0403AA	69	387
ESC_TA0402AA	69	387
ESC_TA0401AA	2541	878
ESC_TA0400AA	2541	878
ESC_TA0399AA	2541	878
ESC_TA0398AA	2541	878
ESC_TA0397AA	131	653
ESC_TA0396AA	131	653
ESC_TA0395AA	131	653
ESC_TA0394AA	500	71
ESC_TA0393AA	131	653
ESC_TA0392AA	131	653
ESC_TA0391AA	131	653
ESC_TA0390AA	131	653
ESC_TA0389AA	963	669
ESC_TA0388AA	69	387
ESC_TA0387AA	500	71

ESC_TA0386AA	131	653
ESC_TA0385AA	1193	761
ESC_TA0384AA	131	653
ESC_TA0383AA	500	71
ESC_TA0382AA	131	653
ESC_TA0381AA	38	669
ESC_TA0380AA	963	669
ESC_TA0379AA	69	387
ESC_TA0378AA	131	653
ESC_TA0377AA	500	71
ESC_TA0376AA	500	71
ESC_TA0375AA	500	71
ESC_TA0374AA	500	71
ESC_TA0373AA	500	71
ESC_TA0372AA	500	71
ESC_TA0371AA	131	653
ESC_TA0370AA	131	653
ESC_TA0369AA	131	653
ESC_TA0368AA	500	71
ESC_TA0367AA	131	653
ESC_TA0366AA	131	653
ESC_TA0365AA	131	653
ESC_TA0364AA	38	669
ESC_TA0363AA	38	669
ESC_TA0362AA	38	669
ESC_TA0361AA	38	669
ESC_TA0360AA	538	629
ESC_TA0359AA	4553	408
ESC_TA0358AA	38	669
ESC_TA0357AA	500	71
ESC_TA0356AA	38	669
ESC_TA0355AA	10	512
ESC_TA0354AA	746	789
ESC_TA0353AA	746	789
ESC_TA0352AA	746	789
ESC_TA0351AA	2541	878
ESC_TA0350AA	69	387
ESC_TA0349AA	69	387
ESC_TA0348AA	69	387
ESC_TA0347AA	69	387
ESC_TA0346AA	963	669
ESC_TA0345AA	69	387
ESC_TA0344AA	963	669
ESC_TA0343AA	963	669
ESC_TA0342AA	963	669
ESC_TA0341AA	963	669
ESC_TA0340AA	963	669
ESC_TA0339AA	131	653
ESC_TA0338AA	131	653
ESC_TA0337AA	648	359

ESC_TA0336AA	131	653
ESC_TA0335AA	648	359
ESC_TA0334AA	58	198
ESC_TA0333AA	131	653
ESC_TA0332AA	131	653
ESC_TA0331AA	131	653
ESC_TA0330AA	131	653
ESC_TA0329AA	131	653
ESC_TA0328AA	131	653
ESC_TA0327AA	131	653
ESC_TA0326AA	131	653
ESC_TA0325AA	617	511
ESC_TA0324AA	617	511
ESC_TA0323AA	69	387
ESC_TA0322AA	617	511
ESC_TA0321AA	617	511
ESC_TA0320AA	617	511
ESC_TA0319AA	617	511
ESC_TA0318AA	617	511
ESC_TA0317AA	617	511
ESC_TA0316AA	1193	761
ESC_TA0315AA	1193	761
ESC_TA0314AA	131	653
ESC_TA0313AA	131	653
ESC_TA0312AA	69	387
ESC_TA0311AA	131	653
ESC_TA0310AA	131	653
ESC_TA0309AA	131	653
ESC_TA0308AA	131	653
ESC_TA0307AA	131	653
ESC_TA0306AA	131	653
ESC_TA0305AA	131	653
ESC_TA0304AA	131	653
ESC_TA0303AA	131	653
ESC_TA0302AA	131	653
ESC_TA0301AA	500	71
ESC_TA0300AA	131	653
ESC_TA0299AA	131	653
ESC_TA0298AA	131	653
ESC_TA0297AA	131	653
ESC_TA0296AA	131	653
ESC_TA0295AA	131	653
ESC_TA0294AA	131	653
ESC_TA0293AA	131	653
ESC_TA0292AA	131	653
ESC_TA0291AA	131	653
ESC_TA0290AA	500	71
ESC_TA0289AA	131	653
ESC_TA0288AA	131	653
ESC_TA0287AA	131	653

ESC_TA0286AA	131	653
ESC_TA0284AA	131	653
ESC_TA0283AA	131	653
ESC_TA0272AA	713	422
ESC_TA0271AA	4283	654
ESC_TA0270AA	106	387
ESC_TA0269AA	106	387
ESC_TA0268AA	2352	198
ESC_TA0267AA	129	257
ESC_TA0266AA	716	795
ESC_TA0265AA	8973	670
ESC_TA0264AA	4290	234
ESC_TA0263AA	2354	257
ESC_TA0262AA	4290	234
ESC_TA0261AA	2562	640
ESC_TA0260AA	28	537
ESC_TA0259AA	4283	654
ESC_TA0258AA	949	175
ESC_TA0256AA	11565	359
ESC_TA0255AA	345	175
ESC_TA0254AA	58	198
ESC_TA0253AA	162	924
ESC_TA0252AA	69	387
ESC_TA0251AA	117	592
ESC_TA0250AA	189	504
ESC_TA0249AA	58	198
ESC_TA0248AA	28	537
ESC_TA0247AA	1611	198
ESC_TA0246AA	314	156
ESC_TA0245AA	8611	422
ESC_TA0244AA	83	740
ESC_TA0243AA	1611	198
ESC_TA0242AA	2183	50
ESC_TA0241AA	457	595
ESC_TA0240AA	131	653
ESC_TA0239AA	124	254
ESC_TA0238AA	131	653
ESC_TA0237AA	937	198
ESC_TA0236AA	57	50
ESC_TA0235AA	69	387
ESC_TA0234AA	1718	269
ESC_TA0233AA	40	198
ESC_TA0232AA	1618	736
ESC_TA0231AA	1157	878
ESC_TA0230AA	196	257
ESC_TA0229AA	949	175
ESC_TA0228AA	58	198
ESC_TA0227AA	83	740
ESC_TA0226AA	457	595
ESC_TA0225AA	1640	50

ESC_TA0224AA	57	50
ESC_TA0223AA	5926	50
ESC_TA0222AA	457	595
ESC_TA0221AA	131	653
ESC_TA0220AA	131	653
ESC_TA0219AA	127	738
ESC_TA0218AA	1086	175
ESC_TA0217AA	457	595
ESC_TA0216AA	57	50
ESC_TA0215AA	5926	50
ESC_TA0214AA	457	595
ESC_TA0213AA	457	595
ESC_TA0212AA	69	387
ESC_TA0211AA	58	198
ESC_TA0210AA	127	738
ESC_TA0209AA	141	644
ESC_TA0208AA	457	595
ESC_TA0207AA	10	512
ESC_TA0206AA	58	198
ESC_TA0205AA	101	696
ESC_TA0204AA	297	234
ESC_TA0203AA	537	756
ESC_TA0202AA	131	653
ESC_TA0201AA	998	644
ESC_TA0200AA	117	592
ESC_TA0199AA	327	204
ESC_TA0198AA	88	160
ESC_TA0197AA	83	740
ESC_TA0196AA	83	740
ESC_TA0195AA	457	595
ESC_TA0194AA	73	737
ESC_SA9397AA	414	387
ESC_SA9396AA	414	387
ESC_SA9395AA	73	737
ESC_SA9394AA	73	737
ESC_SA9393AA	1193	761
ESC_SA9392AA	1193	761
ESC_SA9391AA	1193	761
ESC_SA9390AA	1193	761
ESC_SA9389AA	949	175
ESC_SA9388AA	2797	987
ESC_SA9386AA	155	175
ESC_SA9385AA	226	512
ESC_SA9384AA	949	175
ESC_SA9383AA	949	175
ESC_SA9382AA	949	175
ESC_SA9380AA	11543	160
ESC_SA9379AA	1431	257
ESC_SA9378AA	949	175
ESC_SA9377AA	1431	257

ESC_SA9376AA	949	175
ESC_SA9375AA	3246	900
ESC_SA9374AA	949	175
ESC_SA9373AA	949	175
ESC_SA9372AA	949	175
ESC_SA9371AA	949	175
ESC_SA9370AA	949	175
ESC_SA9369AA	1490	935
ESC_SA9361AA	939	198
ESC_SA9360AA	372	424
ESC_SA9359AA	73	737
ESC_SA9326AA	245	261
ESC_SA9324AA	11	936
ESC_SA9323AA	442	257
ESC_SA9322AA	11	936
ESC_SA9321AA	4213	251
ESC_SA9320AA	11	936
ESC_SA9319AA	32	270
ESC_SA9318AA	57	50
ESC_SA9317AA	1170	629
ESC_SA9316AA	20	198
ESC_SA9315AA	453	935
ESC_SA9314AA	1704	257
ESC_SA9313AA	3580	257
ESC_SA9312AA	501	900
ESC_SA9311AA	1236	380
ESC_SA9310AA	10	512
ESC_SA9309AA	278	924
ESC_SA9308AA	126	740
ESC_SA9307AA	93	71
ESC_SA9306AA	3602	234
ESC_SA9305AA	180	234
ESC_SA9304AA	10	512
ESC_SA9303AA	2163	257
ESC_SA9302AA	1301	270
ESC_SA9301AA	898	108
ESC_SA9300AA	164	175
ESC_SA9299AA	898	108
ESC_SA9298AA	189	504
ESC_SA9297AA	2079	198
ESC_SA9296AA	10	512
ESC_SA9295AA	542	824
ESC_SA9294AA	109	257
ESC_SA9293AA	1079	235
ESC_SA9292AA	10	512
ESC_SA9291AA	117	592
ESC_SA9277AA	327	204
ESC_SA9276AA	209	512
ESC_SA9275AA	11541	286
ESC_SA9274AA	1665	601

ESC_SA9273AA	88	160
ESC_SA9272AA	23	156
ESC_SA9271AA	1992	257
ESC_SA9270AA	10	512
ESC_SA9269AA	2001	670
ESC_SA9268AA	10	512
ESC_SA9267AA	336	878
ESC_SA9266AA	58	198
ESC_SA9265AA	10	512
ESC_SA9264AA	906	198
ESC_SA9263AA	58	198
ESC_SA9262AA	278	924
ESC_SA9261AA	295	160
ESC_SA9260AA	336	878
ESC_SA9259AA	398	445
ESC_SA9258AA	1147	198
ESC_SA9257AA	131	653
ESC_SA9256AA	58	198
ESC_SA9255AA	2541	878
ESC_SA9254AA	11542	76
ESC_SA9253AA	3854	76
ESC_SA9251AA	11	936
ESC_SA9250AA	11	936
ESC_SA9249AA	11	936
ESC_SA9248AA	11	936
ESC_SA9247AA	11	936
ESC_SA9246AA	6643	198
ESC_SA9245AA	196	257
ESC_SA9244AA	2536	261
ESC_SA9243AA	6643	198
ESC_SA9242AA	32	270
ESC_SA9241AA	164	175
ESC_SA9240AA	162	924
ESC_SA9239AA	297	234
ESC_SA9238AA	6643	198
ESC_SA9237AA	34	504
ESC_SA9236AA	5971	979
ESC_SA9235AA	2008	257
ESC_SA9234AA	154	257
ESC_SA9233AA	6643	198
ESC_SA9232AA	3246	900
ESC_SA9231AA	101	696
ESC_SA9230AA	446	257
ESC_SA9229AA	8369	696
ESC_SA9228AA	8369	696
ESC_SA9227AA	2387	261
ESC_SA9226AA	442	257
ESC_SA9225AA	2165	257
ESC_SA9214AA	40	198
ESC_SA9213AA	6251	808

ESC_SA9212AA	25	234
ESC_SA9211AA	32	270
ESC_SA9210AA	167	512
ESC_SA9209AA	245	261
ESC_SA9208AA	11	936
ESC_SA9207AA	11	936
ESC_SA9206AA	11	936
ESC_SA9205AA	11	936
ESC_SA9204AA	11	936
ESC_SA9203AA	11	936
ESC_SA9202AA	11	936
ESC_SA9201AA	11	936
ESC_SA9200AA	11	936
ESC_SA9199AA	11	936
ESC_SA9198AA	11	936
ESC_SA9197AA	11	936
ESC_SA9196AA	11	936
ESC_SA9195AA	11	936
ESC_SA9194AA	11	936
ESC_SA9193AA	11	936
ESC_SA9192AA	11	936
ESC_SA9191AA	11	936
ESC_SA9190AA	11	936
ESC_SA9189AA	11	936
ESC_SA9188AA	11	936
ESC_SA9187AA	11	936
ESC_SA9186AA	11	936
ESC_SA9185AA	11	936
ESC_SA9184AA	11	936
ESC_SA9183AA	11	936
ESC_SA9182AA	11	936
ESC_SA9181AA	11	936
ESC_SA9180AA	11	936
ESC_SA9178AA	648	359
ESC_SA9177AA	410	156
ESC_SA9176AA	167	512
ESC_SA9175AA	167	512
ESC_SA9174AA	167	512
ESC_SA9173AA	21	156
ESC_SA9172AA	3	198
ESC_SA9171AA	29	156
ESC_SA9170AA	3	198
ESC_SA9169AA	21	156
ESC_SA9168AA	17	191
ESC_SA9167AA	414	387
ESC_SA9166AA	16	156
ESC_SA9165AA	21	156
ESC_SA9164AA	21	156
ESC_SA9163AA	40	198
ESC_SA9162AA	16	156

ESC_SA9161AA	11	936
ESC_SA9160AA	21	156
ESC_SA9159AA	16	156
ESC_SA9158AA	17	191
ESC_SA9157AA	11	936
ESC_SA9156AA	16	156
ESC_SA9155AA	21	156
ESC_SA9154AA	16	156
ESC_SA9153AA	29	156
ESC_SA9152AA	21	156
ESC_SA9151AA	11	936
ESC_SA9150AA	16	156
ESC_SA9149AA	21	156
ESC_SA9148AA	16	156
ESC_SA9147AA	17	191
ESC_SA9146AA	3	198
ESC_SA9145AA	21	156
ESC_SA9144AA	16	156
ESC_SA9143AA	16	156
ESC_SA9142AA	16	156
ESC_SA9141AA	16	156
ESC_SA9140AA	2967	289
ESC_SA9139AA	135	653
ESC_SA9138AA	442	257
ESC_SA9137AA	10	512
ESC_SA9136AA	43	512
ESC_SA9135AA	88	160
ESC_SA9134AA	17	191
ESC_SA9133AA	2967	289
ESC_SA9132AA	16	156
ESC_SA9131AA	16	156
ESC_SA9130AA	21	156
ESC_SA9129AA	3	198
ESC_SA9128AA	68	386
ESC_SA9127AA	10	512
ESC_SA9126AA	131	653
ESC_SA9125AA	131	653
ESC_SA9124AA	131	653
ESC_SA9123AA	410	156
ESC_SA9122AA	648	359
ESC_SA9121AA	131	653
ESC_SA9120AA	131	653
ESC_SA9119AA	648	359
ESC_SA9118AA	12	626
ESC_SA9117AA	196	257
ESC_SA9116AA	457	595
ESC_SA9115AA	131	653
ESC_SA9114AA	95	738
ESC_SA9113AA	38	669
ESC_SA9112AA	131	653

ESC_SA9111AA	131	653
ESC_SA9110AA	607	355
ESC_SA9109AA	69	387
ESC_SA9108AA	457	595
ESC_SA9107AA	410	156
ESC_SA9106AA	1722	599
ESC_SA9105AA	405	923
ESC_SA9104AA	131	653
ESC_SA9103AA	131	653
ESC_SA9102AA	11539	741
ESC_SA9101AA	38	669
ESC_SA9100AA	648	359
ESC_SA9099AA	11538	669
ESC_SA9098AA	131	653
ESC_SA9097AA	131	653
ESC_SA9096AA	1326	198
ESC_SA9095AA	38	669
ESC_SA9094AA	1079	235
ESC_SA9093AA	167	512
ESC_SA9092AA	11021	512
ESC_SA9091AA	410	156
ESC_SA9090AA	131	653
ESC_SA9089AA	5135	278
ESC_SA9088AA	131	653
ESC_SA9087AA	410	156
ESC_SA9086AA	219	877
ESC_SA9085AA	10	512
ESC_SA9084AA	617	511
ESC_SA9083AA	68	386
ESC_SA9082AA	167	512
ESC_SA9081AA	963	669
ESC_SA9080AA	131	653
ESC_SA9079AA	405	923
ESC_SA9078AA	167	512
ESC_SA9077AA	38	669
ESC_SA9076AA	10	512
ESC_SA9075AA	131	653
ESC_SA9074AA	131	653
ESC_SA9073AA	10064	711
ESC_SA9072AA	131	653
ESC_SA9071AA	68	386
ESC_SA9070AA	38	669
ESC_SA9069AA	405	923
ESC_SA9068AA	410	156
ESC_SA9067AA	410	156
ESC_SA9066AA	624	359
ESC_SA9065AA	69	387
ESC_SA9064AA	10	512
ESC_SA9063AA	10	512
ESC_SA9062AA	10	512

ESC_SA9061AA	10	512
ESC_SA9060AA	10	512
ESC_SA9059AA	10	512
ESC_SA9058AA	10	512
ESC_SA9057AA	10	512
ESC_SA9055AA	10	512
ESC_SA9053AA	10	512
ESC_SA9052AA	10	512
ESC_SA9051AA	10	512
ESC_SA9050AA	10	512
ESC_SA9049AA	10	512
ESC_SA9048AA	10	512
ESC_SA9047AA	10	512
ESC_SA9046AA	10	512
ESC_SA9045AA	10	512
ESC_SA9044AA	10	512
ESC_SA9043AA	10	512
ESC_SA9042AA	10	512
ESC_SA9041AA	10	512
ESC_SA9040AA	10	512
ESC_SA9039AA	10	512
ESC_SA9038AA	10	512
ESC_SA9037AA	10	512
ESC_SA9036AA	10	512
ESC_SA9035AA	10	512
ESC_SA9034AA	10	512
ESC_SA9033AA	10	512
ESC_SA9032AA	10	512
ESC_SA9031AA	10	512
ESC_SA9030AA	10	512
ESC_SA9028AA	10	512
ESC_SA9027AA	10	512
ESC_SA9026AA	10	512
ESC_SA9025AA	10	512
ESC_SA9024AA	10	512
ESC_SA9023AA	10	512
ESC_SA9022AA	10	512
ESC_SA9021AA	10	512
ESC_SA9020AA	10	512
ESC_SA9019AA	10	512
ESC_SA9017AA	10	512
ESC_SA9016AA	10	512
ESC_SA9015AA	10	512
ESC_SA9014AA	10	512
ESC_SA9013AA	10	512
ESC_SA9012AA	10	512
ESC_SA9011AA	10	512
ESC_SA9010AA	10	512
ESC_SA9009AA	10	512
ESC_SA9008AA	10	512

ESC_SA9007AA	10	512
ESC_SA9006AA	10	512
ESC_SA9005AA	10	512
ESC_SA9004AA	10	512
ESC_SA9003AA	10	512
ESC_SA9002AA	10	512
ESC_SA9001AA	10	512
ESC_SA9000AA	10	512
ESC_SA8999AA	10	512
ESC_SA8998AA	10	512
ESC_SA8997AA	10	512
ESC_SA8996AA	10	512
ESC_SA8995AA	10	512
ESC_SA8994AA	10	512
ESC_SA8993AA	10	512
ESC_SA8992AA	10	512
ESC_SA8991AA	10	512
ESC_SA8990AA	10	512
ESC_SA8989AA	10	512
ESC_SA8988AA	10	512
ESC_SA8987AA	10	512
ESC_SA8986AA	10	512
ESC_SA8985AA	10	512
ESC_SA8984AA	10	512
ESC_SA8983AA	10	512
ESC_SA8982AA	10	512
ESC_SA8981AA	10	512
ESC_SA8980AA	10	512
ESC_SA8979AA	10	512
ESC_SA8977AA	10	512
ESC_SA8976AA	10	512
ESC_SA8975AA	10	512
ESC_SA8974AA	10	512
ESC_SA8973AA	10	512
ESC_SA8972AA	10	512
ESC_SA8971AA	10	512
ESC_SA8970AA	10	512
ESC_SA8969AA	10	512
ESC_SA8968AA	10	512
ESC_SA8967AA	10	512
ESC_SA8966AA	10	512
ESC_SA8965AA	10	512
ESC_SA8963AA	10	512
ESC_SA8962AA	10	512
ESC_SA8960AA	10	512
ESC_SA8957AA	10	512
ESC_SA8956AA	10	512
ESC_SA8953AA	10	512
ESC_SA8952AA	10	512
ESC_SA8951AA	10	512

ESC_SA8950AA	10	512
ESC_SA8949AA	10	512
ESC_SA8948AA	10	512
ESC_SA8947AA	10	512
ESC_SA8946AA	10	512
ESC_SA8945AA	10	512
ESC_SA8944AA	10	512
ESC_SA8943AA	10	512
ESC_SA8942AA	10	512
ESC_SA8941AA	10	512
ESC_SA8940AA	10	512
ESC_SA8939AA	10	512
ESC_SA8938AA	10	512
ESC_SA8937AA	10	512
ESC_SA8936AA	10	512
ESC_SA8935AA	10	512
ESC_SA8934AA	10	512
ESC_SA8933AA	10	512
ESC_SA8932AA	10	512
ESC_SA8931AA	10	512
ESC_SA8930AA	10	512
ESC_SA8929AA	10	512
ESC_SA8928AA	10	512
ESC_SA8927AA	10	512
ESC_SA8926AA	10	512
ESC_SA8925AA	10	512
ESC_SA8924AA	10	512
ESC_SA8922AA	10	512
ESC_SA8921AA	10	512
ESC_SA8920AA	10	512
ESC_SA8919AA	10	512
ESC_SA8918AA	10	512
ESC_SA8917AA	10	512
ESC_SA8916AA	10	512
ESC_SA8915AA	10	512
ESC_SA8912AA	10	512
ESC_SA8910AA	10	512
ESC_SA8909AA	10	512
ESC_SA8908AA	10	512
ESC_SA8907AA	10	512
ESC_SA8906AA	10	512
ESC_SA8905AA	10	512
ESC_SA8904AA	10	512
ESC_SA8903AA	10	512
ESC_SA8902AA	10	512
ESC_SA8901AA	10	512
ESC_SA8900AA	10	512
ESC_SA8899AA	10	512
ESC_SA8898AA	10	512
ESC_SA8897AA	10	512

ESC_SA8896AA	10	512
ESC_SA8895AA	10	512
ESC_SA8894AA	10	512
ESC_SA8893AA	10	512
ESC_SA8892AA	10	512
ESC_SA8891AA	10	512
ESC_SA8890AA	10	512
ESC_SA8889AA	10	512
ESC_SA8888AA	10	512
ESC_SA8887AA	10	512
ESC_SA8886AA	10	512
ESC_SA8885AA	10	512
ESC_SA8884AA	10	512
ESC_SA8883AA	10	512
ESC_SA8882AA	10	512
ESC_SA8881AA	10	512
ESC_SA8880AA	10	512
ESC_SA8879AA	617	511
ESC_SA8878AA	167	512
ESC_SA8877AA	10	512
ESC_SA8876AA	410	156
ESC_SA8875AA	1670	438
ESC_SA8874AA	410	156
ESC_SA8873AA	11540	935
ESC_SA8872AA	648	359
ESC_SA8871AA	38	669
ESC_SA8869AA	127	738
ESC_SA8868AA	5912	156
ESC_SA8867AA	75	257
ESC_SA8866AA	1444	768
ESC_SA8865AA	73	737
ESC_SA8864AA	1385	653
ESC_SA8863AA	9172	160
ESC_SA8862AA	372	424
ESC_SA8861AA	297	234
ESC_SA8860AA	961	623
ESC_SA8859AA	131	653
ESC_SA8858AA	1735	737
ESC_SA8857AA	141	644
ESC_SA8856AA	131	653
ESC_SA8855AA	1946	726
ESC_SA8854AA	162	924
ESC_SA8853AA	2015	684
ESC_SA8852AA	2005	175
ESC_SA8851AA	1946	726
ESC_SA8850AA	48	76
ESC_SA8849AA	155	175
ESC_SA8848AA	48	76
ESC_SA8847AA	155	175
ESC_SA8846AA	297	234

ESC_SA8845AA	6540	801
ESC_SA8844AA	48	76
ESC_SA8843AA	1844	198
ESC_SA8842AA	6540	801
ESC_SA8841AA	11516	1023
ESC_SA8840AA	2371	612
ESC_SA8839AA	165	504
ESC_SA8838AA	3231	519
ESC_SA8837AA	1276	241
ESC_SA8836AA	2280	212
ESC_SA8835AA	5573	396
ESC_SA8834AA	2456	71
ESC_SA8833AA	4103	453
ESC_SA8832AA	5573	396
ESC_SA8831AA	453	935
ESC_SA8830AA	9287	732
ESC_SA8829AA	7416	372
ESC_SA8828AA	5573	396
ESC_SA8827AA	6018	52
ESC_SA8826AA	10	512
ESC_SA8825AA	7416	372
ESC_SA8824AA	7059	0
ESC_SA8823AA	10	512
ESC_SA8822AA	2456	71
ESC_SA8821AA	46	447
ESC_SA8820AA	11515	377
ESC_SA8819AA	1204	961
ESC_SA8818AA	5573	396
ESC_SA8817AA	2456	71
ESC_SA8816AA	11513	719
ESC_SA8815AA	2456	71
ESC_SA8814AA	2307	198
ESC_SA8813AA	7059	0
ESC_SA8812AA	2456	71
ESC_SA8811AA	7059	0
ESC_SA8810AA	388	156
ESC_SA8809AA	1112	504
ESC_SA8808AA	6540	801
ESC_SA8807AA	2456	71
ESC_SA8806AA	2936	515
ESC_SA8805AA	11514	199
ESC_SA8804AA	11513	719
ESC_SA8803AA	4103	453
ESC_SA8802AA	5573	396
ESC_SA8801AA	4173	234
ESC_SA8800AA	1638	519
ESC_SA8799AA	5391	373
ESC_SA8798AA	11512	789
ESC_SA8797AA	746	789
ESC_SA8796AA	1844	198

ESC_SA8795AA	10	512
ESC_SA8794AA	48	76
ESC_SA8793AA	10	512
ESC_SA8792AA	9287	732
ESC_SA8791AA	7416	372
ESC_SA8790AA	7059	0
ESC_SA8789AA	11511	373
ESC_SA8788AA	5643	462
ESC_SA8787AA	48	76
ESC_SA8786AA	5573	396
ESC_SA8785AA	710	291
ESC_SA8784AA	1112	504
ESC_SA8783AA	155	175
ESC_SA8782AA	2456	71
ESC_SA8781AA	2280	212
ESC_SA8780AA	46	447
ESC_SA8779AA	1276	241
ESC_SA8778AA	48	76
ESC_SA8777AA	11508	465
ESC_SA8776AA	3568	801
ESC_SA8775AA	11510	372
ESC_SA8774AA	48	76
ESC_SA8773AA	48	76
ESC_SA8772AA	5573	396
ESC_SA8771AA	1112	504
ESC_SA8770AA	48	76
ESC_SA8769AA	1638	519
ESC_SA8768AA	10	512
ESC_SA8767AA	23	156
ESC_SA8766AA	5643	462
ESC_SA8765AA	11513	719
ESC_SA8764AA	1844	198
ESC_SA8763AA	48	76
ESC_SA8762AA	1112	504
ESC_SA8761AA	2598	198
ESC_SA8760AA	48	76
ESC_SA8759AA	58	198
ESC_SA8758AA	685	311
ESC_SA8757AA	48	76
ESC_SA8756AA	155	175
ESC_SA8755AA	10	512
ESC_SA8754AA	11509	1014
ESC_SA8753AA	155	175
ESC_SA8752AA	1638	519
ESC_SA8751AA	10	512
ESC_SA8750AA	11	936
ESC_SA8749AA	11	936
ESC_SA8748AA	11	936
ESC_SA8747AA	11506	261
ESC_SA8745AA	648	359

ESC_SA8744AA	648	359
ESC_SA8740AA	1722	599
ESC_SA8739AA	1722	599
ESC_SA8729AA	5229	696
ESC_SA8719AA	32	270
ESC_SA8718AA	442	257
ESC_SA8717AA	29	156
ESC_SA8716AA	442	257
ESC_SA8715AA	442	257
ESC_SA8714AA	6992	842
ESC_SA8713AA	3570	156
ESC_SA8712AA	723	156
ESC_SA8711AA	5560	929
ESC_SA8710AA	11	936
ESC_SA8709AA	152	257
ESC_SA8708AA	245	261
ESC_SA8707AA	145	256
ESC_SA8706AA	32	270
ESC_SA8705AA	11	936
ESC_SA8704AA	590	156
ESC_SA8703AA	29	156
ESC_SA8702AA	32	270
ESC_SA8701AA	6992	842
ESC_SA8700AA	21	156
ESC_SA8699AA	442	257
ESC_SA8698AA	11	936
ESC_SA8697AA	675	234
ESC_SA8696AA	206	286
ESC_SA8695AA	675	234
ESC_SA8694AA	10698	512
ESC_SA8693AA	11	936
ESC_SA8686AA	10	512
ESC_SA8685AA	57	50
ESC_SA8680AA	11	936
ESC_SA8679AA	541	701
ESC_SA8678AA	11	936
ESC_SA8677AA	8392	512
ESC_SA8676AA	11537	286
ESC_SA8675AA	10	512
ESC_SA8674AA	11	936
ESC_SA8673AA	137	270
ESC_SA8672AA	11	936
ESC_SA8671AA	11	936
ESC_SA8670AA	11	936
ESC_SA8669AA	11	936
ESC_SA8611AA	1193	761
ESC_SA8610AA	1193	761
ESC_SA8609AA	1193	761
ESC_SA8608AA	1193	761
ESC_SA8607AA	1193	761

ESC_SA8606AA	1193	761
ESC_SA8605AA	1193	761
ESC_SA8604AA	1193	761
ESC_SA8603AA	1193	761
ESC_SA8602AA	1193	761
ESC_SA8601AA	90	156
ESC_SA8600AA	90	156
ESC_SA8599AA	90	156
ESC_SA8598AA	90	156
ESC_SA8597AA	90	156
ESC_SA8596AA	90	156
ESC_SA8595AA	641	935
ESC_SA8592AA	11496	71
ESC_SA8591AA	11495	71
ESC_SA8590AA	11494	936
ESC_SA8587AA	3580	257
ESC_SA8586AA	1423	198
ESC_SA8585AA	95	738
ESC_SA8584AA	3580	257
ESC_SA8583AA	48	76
ESC_SA8582AA	1434	512
ESC_SA8581AA	2601	785
ESC_SA8580AA	155	175
ESC_SA8579AA	1968	76
ESC_SA8578AA	34	504
ESC_SA8577AA	10	512
ESC_SA8576AA	135	653
ESC_SA8575AA	2001	670
ESC_SA8574AA	399	198
ESC_SA8573AA	350	50
ESC_SA8572AA	101	696
ESC_SA8571AA	155	175
ESC_SA8570AA	11536	885
ESC_SA8569AA	10	512
ESC_SA8568AA	95	738
ESC_SA8567AA	154	257
ESC_SA8566AA	117	592
ESC_SA8565AA	155	175
ESC_SA8564AA	117	592
ESC_SA8563AA	10	512
ESC_SA8562AA	101	696
ESC_SA8561AA	949	175
ESC_SA8560AA	58	198
ESC_SA8559AA	48	76
ESC_SA8558AA	180	234
ESC_SA8557AA	1564	512
ESC_SA8556AA	720	900
ESC_SA8555AA	541	701
ESC_SA8554AA	1158	387
ESC_SA8553AA	101	696

ESC_SA8552AA	162	924
ESC_SA8551AA	1618	736
ESC_SA8550AA	3580	257
ESC_SA8549AA	2556	630
ESC_SA8548AA	46	447
ESC_SA8547AA	401	142
ESC_SA8546AA	973	968
ESC_SA8545AA	117	592
ESC_SA8544AA	744	512
ESC_SA8543AA	2144	257
ESC_SA8542AA	906	198
ESC_SA8541AA	278	924
ESC_SA8540AA	10770	268
ESC_SA8539AA	11342	653
ESC_SA8538AA	372	424
ESC_SA8537AA	297	234
ESC_SA8536AA	420	741
ESC_SA8535AA	372	424
ESC_SA8534AA	12	626
ESC_SA8533AA	677	257
ESC_SA8532AA	5487	198
ESC_SA8531AA	10	512
ESC_SA8530AA	329	355
ESC_SA8529AA	223	198
ESC_SA8528AA	11528	731
ESC_SA8527AA	23	156
ESC_SA8526AA	16	156
ESC_SA8525AA	349	670
ESC_SA8524AA	1323	405
ESC_SA8523AA	329	355
ESC_SA8522AA	515	1004
ESC_SA8521AA	20	198
ESC_SA8520AA	119	286
ESC_SA8519AA	583	768
ESC_SA8518AA	636	653
ESC_SA8517AA	446	257
ESC_SA8516AA	3721	93
ESC_SA8515AA	68	386
ESC_SA8514AA	583	768
ESC_SA8513AA	722	422
ESC_SA8512AA	21	156
ESC_SA8511AA	69	387
ESC_SA8510AA	8935	101
ESC_SA8509AA	342	286
ESC_SA8508AA	154	257
ESC_SA8507AA	9236	198
ESC_SA8506AA	446	257
ESC_SA8505AA	11527	80
ESC_SA8504AA	16	156
ESC_SA8503AA	11526	254

ESC_SA8502AA	20	198
ESC_SA8501AA	443	257
ESC_SA8500AA	223	198
ESC_SA8499AA	655	926
ESC_SA8498AA	329	355
ESC_SA8497AA	10	512
ESC_SA8496AA	58	198
ESC_SA8495AA	9021	286
ESC_SA8494AA	343	286
ESC_SA8493AA	205	257
ESC_SA8492AA	297	234
ESC_SA8491AA	329	355
ESC_SA8490AA	657	592
ESC_SA8489AA	162	924
ESC_SA8488AA	21	156
ESC_SA8487AA	306	286
ESC_SA8486AA	5419	175
ESC_SA8485AA	21	156
ESC_SA8484AA	446	257
ESC_SA8483AA	655	926
ESC_SA8482AA	641	935
ESC_SA8481AA	20	198
ESC_SA8480AA	20	198
ESC_SA8479AA	33	198
ESC_SA8478AA	8356	198
ESC_SA8477AA	4038	257
ESC_SA8476AA	101	696
ESC_SA8475AA	10	512
ESC_SA8474AA	155	175
ESC_SA8473AA	3744	519
ESC_SA8472AA	56	198
ESC_SA8471AA	342	286
ESC_SA8470AA	443	257
ESC_SA8469AA	515	1004
ESC_SA8468AA	17	191
ESC_SA8467AA	342	286
ESC_SA8466AA	4429	820
ESC_SA8465AA	329	355
ESC_SA8464AA	11525	254
ESC_SA8463AA	443	257
ESC_SA8462AA	187	257
ESC_SA8461AA	21	156
ESC_SA8460AA	11524	924
ESC_SA8459AA	336	878
ESC_SA8458AA	6096	257
ESC_SA8457AA	5082	198
ESC_SA8456AA	11535	1020
ESC_SA8455AA	21	156
ESC_SA8454AA	642	924
ESC_SA8453AA	5487	198

ESC_SA8452AA	442	257
ESC_SA8451AA	342	286
ESC_SA8450AA	675	234
ESC_SA8449AA	675	234
ESC_SA8448AA	20	198
ESC_SA8447AA	5559	257
ESC_SA8446AA	297	234
ESC_SA8445AA	11523	257
ESC_SA8444AA	21	156
ESC_SA8442AA	343	286
ESC_SA8441AA	297	234
ESC_SA8440AA	127	738
ESC_SA8439AA	8355	71
ESC_SA8438AA	647	734
ESC_SA8437AA	300	286
ESC_SA8436AA	56	198
ESC_SA8435AA	657	592
ESC_SA8434AA	11534	900
ESC_SA8433AA	647	734
ESC_SA8432AA	119	286
ESC_SA8431AA	642	924
ESC_SA8430AA	1611	198
ESC_SA8429AA	504	738
ESC_SA8428AA	329	355
ESC_SA8427AA	5419	175
ESC_SA8426AA	16	156
ESC_SA8425AA	11533	198
ESC_SA8424AA	5502	307
ESC_SA8423AA	1125	198
ESC_SA8422AA	1152	850
ESC_SA8421AA	10	512
ESC_SA8420AA	1665	601
ESC_SA8419AA	1662	924
ESC_SA8418AA	10	512
ESC_SA8417AA	446	257
ESC_SA8416AA	7286	447
ESC_SA8415AA	306	286
ESC_SA8414AA	647	734
ESC_SA8413AA	58	198
ESC_SA8412AA	1611	198
ESC_SA8411AA	57	50
ESC_SA8410AA	3018	903
ESC_SA8409AA	20	198
ESC_SA8408AA	937	198
ESC_SA8407AA	420	741
ESC_SA8406AA	11532	173
ESC_SA8405AA	2103	198
ESC_SA8404AA	101	696
ESC_SA8403AA	300	286
ESC_SA8402AA	70	670

ESC_SA8401AA	10	512
ESC_SA8400AA	300	286
ESC_SA8399AA	5343	286
ESC_SA8398AA	300	286
ESC_SA8397AA	1112	504
ESC_SA8396AA	17	191
ESC_SA8395AA	342	286
ESC_SA8394AA	10924	1004
ESC_SA8393AA	325	71
ESC_SA8392AA	515	1004
ESC_SA8391AA	46	447
ESC_SA8390AA	11531	198
ESC_SA8389AA	25	234
ESC_SA8388AA	69	387
ESC_SA8387AA	29	156
ESC_SA8386AA	11530	383
ESC_SA8385AA	5559	257
ESC_SA8384AA	11530	383
ESC_SA8383AA	641	935
ESC_SA8382AA	515	1004
ESC_SA8381AA	10924	1004
ESC_SA8380AA	10924	1004
ESC_SA8379AA	16	156
ESC_SA8378AA	306	286
ESC_SA8377AA	101	696
ESC_SA8376AA	447	198
ESC_SA8375AA	329	355
ESC_SA8374AA	223	198
ESC_SA8373AA	11	936
ESC_SA8372AA	722	422
ESC_SA8371AA	16	156
ESC_SA8370AA	2387	261
ESC_SA8369AA	8929	286
ESC_SA8368AA	329	355
ESC_SA8367AA	677	257
ESC_SA8366AA	2217	62
ESC_SA8365AA	300	286
ESC_SA8364AA	33	198
ESC_SA8363AA	443	257
ESC_SA8362AA	2387	261
ESC_SA8361AA	2064	261
ESC_SA8360AA	5487	198
ESC_SA8359AA	3714	175
ESC_SA8358AA	20	198
ESC_SA8357AA	17	191
ESC_SA8356AA	1125	198
ESC_SA8355AA	11529	253
ESC_SA8354AA	2385	198
ESC_SA8353AA	7083	640
ESC_SA8352AA	7801	198

ESC_SA8351AA	2164	257
ESC_SA8350AA	28	537
ESC_SA8349AA	10	512
ESC_SA8348AA	647	734
ESC_SA8347AA	11	936
ESC_SA8346AA	152	257
ESC_SA8345AA	152	257
ESC_SA8344AA	245	261
ESC_SA8343AA	245	261
ESC_SA8342AA	245	261
ESC_SA8341AA	245	261
ESC_SA8340AA	245	261
ESC_SA8339AA	245	261
ESC_SA8338AA	245	261
ESC_SA8337AA	245	261
ESC_SA8336AA	245	261
ESC_SA8335AA	245	261
ESC_SA8334AA	245	261
ESC_SA8333AA	245	261
ESC_SA8332AA	224	198
ESC_SA8331AA	393	343
ESC_SA8330AA	393	343
ESC_SA8329AA	393	343
ESC_SA8328AA	393	343
ESC_SA8327AA	393	343
ESC_SA8326AA	393	343
ESC_SA8325AA	393	343
ESC_SA8324AA	393	343
ESC_SA8323AA	393	343
ESC_SA8322AA	393	343
ESC_SA8321AA	393	343
ESC_SA8320AA	38	669
ESC_SA8319AA	38	669
ESC_SA8318AA	393	343
ESC_SA8317AA	393	343
ESC_SA8316AA	38	669
ESC_SA8315AA	38	669
ESC_SA8314AA	393	343
ESC_SA8313AA	9586	198
ESC_SA8312AA	38	669
ESC_SA8311AA	131	653
ESC_SA8310AA	38	669
ESC_SA8309AA	131	653
ESC_SA8308AA	131	653
ESC_SA8307AA	73	737
ESC_SA8306AA	38	669
ESC_SA8305AA	393	343
ESC_SA8304AA	1011	198
ESC_SA8303AA	1011	198
ESC_SA8302AA	12	626

ESC_SA8301AA	1011	198
ESC_SA8300AA	1011	198
ESC_SA8299AA	131	653
ESC_SA8298AA	131	653
ESC_SA8297AA	393	343
ESC_SA8296AA	38	669
ESC_SA8295AA	10284	954
ESC_SA8294AA	730	445
ESC_SA8293AA	372	424
ESC_SA8292AA	2808	737
ESC_SA8291AA	372	424
ESC_SA8290AA	127	738
ESC_SA8289AA	1193	761
ESC_SA8288AA	5298	468
ESC_SA8287AA	164	175
ESC_SA8286AA	4682	175
ESC_SA8285AA	5937	257
ESC_SA8283AA	10	512
ESC_SA8282AA	10	512
ESC_SA8281AA	10	512
ESC_SA8280AA	162	924
ESC_SA8279AA	1122	307
ESC_SA8278AA	1122	307
ESC_SA8277AA	162	924
ESC_SA8276AA	127	738
ESC_SA8275AA	1423	198
ESC_SA8274AA	10	512
ESC_SA8273AA	7808	957
ESC_SA8272AA	7808	957
ESC_SA8270AA	1423	198
ESC_SA8269AA	155	175
ESC_SA8268AA	155	175
ESC_SA8267AA	10	512
ESC_SA8266AA	10	512
ESC_SA8265AA	5937	257
ESC_SA8264AA	5937	257
ESC_SA8263AA	1807	160
ESC_SA8262AA	3	198
ESC_SA8261AA	11	936
ESC_SA8260AA	11	936
ESC_SA8259AA	11	936
ESC_SA8258AA	11	936
ESC_SA8257AA	21	156
ESC_SA8256AA	11	936
ESC_SA8255AA	11	936
ESC_SA8254AA	11	936
ESC_SA8253AA	2388	198
ESC_SA8252AA	11	936
ESC_SA8251AA	38	669
ESC_SA8250AA	33	198

ESC_SA8249AA	11	936
ESC_SA8248AA	11	936
ESC_SA8247AA	11	936
ESC_SA8246AA	11	936
ESC_SA8245AA	11	936
ESC_SA8244AA	5292	738
ESC_SA8243AA	4268	757
ESC_SA8242AA	11	936
ESC_SA8241AA	5418	257
ESC_SA8240AA	206	286
ESC_SA8239AA	165	504
ESC_SA8238AA	90	156
ESC_SA8237AA	90	156
ESC_SA8236AA	206	286
ESC_SA8235AA	120	180
ESC_SA8234AA	8523	926
ESC_SA8233AA	90	156
ESC_SA8232AA	206	286
ESC_SA8231AA	90	156
ESC_SA8230AA	90	156
ESC_SA8229AA	910	929
ESC_SA8228AA	10	512
ESC_SA8227AA	10	512
ESC_SA8226AA	410	156
ESC_SA8225AA	90	156
ESC_SA8224AA	90	156
ESC_SA8223AA	206	286
ESC_SA8222AA	10	512
ESC_SA8221AA	206	286
ESC_SA8220AA	90	156
ESC_SA8219AA	10	512
ESC_SA8218AA	542	824
ESC_SA8217AA	910	929
ESC_SA8216AA	90	156
ESC_SA8215AA	90	156
ESC_SA8214AA	90	156
ESC_SA8213AA	910	929
ESC_SA8212AA	910	929
ESC_SA8211AA	764	170
ESC_SA8210AA	165	504
ESC_SA8209AA	90	156
ESC_SA8208AA	165	504
ESC_SA8207AA	165	504
ESC_SA8206AA	90	156
ESC_SA8205AA	90	156
ESC_SA8204AA	8523	926
ESC_SA8203AA	90	156
ESC_SA8202AA	90	156
ESC_SA8201AA	90	156
ESC_SA8200AA	410	156

ESC_SA8199AA	542	824
ESC_SA8198AA	90	156
ESC_SA8197AA	542	824
ESC_SA8196AA	42	257
ESC_SA8195AA	206	286
ESC_SA8194AA	90	156
ESC_SA8193AA	910	929
ESC_SA8192AA	10	512
ESC_SA8191AA	675	234
ESC_SA8190AA	21	156
ESC_SA8189AA	10988	283
ESC_SA8188AA	675	234
ESC_SA8187AA	17	191
ESC_SA8186AA	69	387
ESC_SA8185AA	342	286
ESC_SA8184AA	11	936
ESC_SA8183AA	1804	935
ESC_SA8182AA	2526	257
ESC_SA8181AA	1421	447
ESC_SA8180AA	20	198
ESC_SA8179AA	278	924
ESC_SA8178AA	898	108
ESC_SA8177AA	6745	885
ESC_SA8176AA	155	175
ESC_SA8175AA	1421	447
ESC_SA8174AA	69	387
ESC_SA8173AA	224	198
ESC_SA8172AA	1081	198
ESC_SA8171AA	11518	512
ESC_SA8170AA	3580	257
ESC_SA8169AA	11519	76
ESC_SA8168AA	11517	504
ESC_SA8167AA	542	824
ESC_SA8166AA	206	286
ESC_SA8165AA	206	286
ESC_SA8164AA	192	696
ESC_SA8163AA	5236	257
ESC_SA8162AA	399	198
ESC_SA8161AA	10	512
ESC_SA8160AA	10	512
ESC_SA8159AA	10	512
ESC_SA8158AA	10	512
ESC_SA8157AA	10	512
ESC_SA8156AA	10	512
ESC_SA8155AA	10	512
ESC_SA8154AA	10	512
ESC_SA8153AA	10	512
ESC_SA8152AA	10	512
ESC_SA8151AA	10	512
ESC_SA8150AA	10	512

ESC_SA8149AA	10	512
ESC_SA8148AA	10	512
ESC_SA8147AA	10	512
ESC_SA8146AA	10	512
ESC_SA8145AA	10	512
ESC_SA8144AA	10	512
ESC_SA8143AA	10	512
ESC_SA8142AA	10	512
ESC_SA8141AA	10	512
ESC_SA8140AA	10	512
ESC_SA8139AA	10	512
ESC_SA8138AA	10	512
ESC_SA8137AA	10	512
ESC_SA8136AA	10	512
ESC_SA8135AA	10	512
ESC_SA8134AA	10	512
ESC_SA8133AA	10	512
ESC_SA8132AA	10	512
ESC_SA8131AA	10	512
ESC_SA8130AA	10	512
ESC_SA8129AA	10	512
ESC_SA8128AA	10	512
ESC_SA8127AA	10	512
ESC_SA8126AA	10	512
ESC_SA8125AA	10	512
ESC_SA8124AA	10	512
ESC_SA8123AA	10	512
ESC_SA8122AA	10	512
ESC_SA8121AA	10	512
ESC_SA8120AA	10	512
ESC_SA8119AA	10	512
ESC_SA8118AA	10	512
ESC_SA8117AA	10	512
ESC_SA8116AA	10	512
ESC_SA8115AA	10	512
ESC_SA8114AA	10	512
ESC_SA8113AA	10	512
ESC_SA8112AA	10	512
ESC_SA8111AA	10	512
ESC_SA8110AA	10	512
ESC_SA8109AA	10	512
ESC_SA8107AA	10	512
ESC_SA8106AA	10	512
ESC_SA8105AA	10	512
ESC_SA8104AA	10	512
ESC_SA8103AA	10	512
ESC_SA8102AA	10	512
ESC_SA8101AA	10	512
ESC_SA8100AA	10	512
ESC_SA8096AA	10	512

ESC_SA8095AA	10	512
ESC_SA8094AA	10	512
ESC_SA8093AA	10	512
ESC_SA8092AA	10	512
ESC_SA8091AA	10	512
ESC_SA8090AA	10	512
ESC_SA8089AA	10	512
ESC_SA8088AA	10	512
ESC_SA8087AA	10	512
ESC_SA8086AA	10	512
ESC_SA8084AA	10	512
ESC_SA8083AA	10	512
ESC_SA8082AA	10	512
ESC_SA8081AA	10	512
ESC_SA8080AA	10	512
ESC_SA8078AA	192	696
ESC_SA8077AA	1434	512
ESC_SA8076AA	2531	198
ESC_SA8075AA	746	789
ESC_SA8074AA	442	257
ESC_SA8073AA	32	270
ESC_SA8072AA	295	160
ESC_SA8071AA	829	257
ESC_SA8070AA	33	198
ESC_SA8069AA	32	270
ESC_SA8068AA	2538	900
ESC_SA8067AA	206	286
ESC_SA8066AA	21	156
ESC_SA8065AA	21	156
ESC_SA8064AA	5352	936
ESC_SA8063AA	3246	900
ESC_SA8062AA	131	653
ESC_SA8061AA	603	198
ESC_SA8060AA	58	198
ESC_SA8059AA	2144	257
ESC_SA8058AA	491	626
ESC_SA8057AA	540	286
ESC_SA8056AA	720	900
ESC_SA8055AA	720	900
ESC_SA8054AA	328	924
ESC_SA8053AA	328	924
ESC_SA8052AA	720	900
ESC_SA8051AA	58	198
ESC_SA8050AA	555	653
ESC_SA8049AA	75	257
ESC_SA8048AA	10	512
ESC_SA8047AA	1250	926
ESC_SA8046AA	29	156
ESC_SA8045AA	162	924
ESC_SA8044AA	10	512

ESC_SA8043AA	88	160
ESC_SA8042AA	57	50
ESC_SA8041AA	555	653
ESC_SA8040AA	11522	924
ESC_SA8039AA	3672	649
ESC_SA8038AA	720	900
ESC_SA8037AA	720	900
ESC_SA8036AA	648	359
ESC_SA8035AA	11522	924
ESC_SA8034AA	491	626
ESC_SA8033AA	1844	198
ESC_SA8032AA	7233	814
ESC_SA8031AA	58	198
ESC_SA8030AA	847	257
ESC_SA8029AA	75	257
ESC_SA8028AA	23	156
ESC_SA8027AA	2522	198
ESC_SA8026AA	88	160
ESC_SA8025AA	720	900
ESC_SA8024AA	88	160
ESC_SA8023AA	2619	738
ESC_SA8021AA	120	180
ESC_SA8020AA	131	653
ESC_SA8019AA	57	50
ESC_SA8018AA	56	198
ESC_SA8017AA	23	156
ESC_SA8016AA	117	592
ESC_SA8015AA	101	696
ESC_SA8014AA	8988	737
ESC_SA8013AA	10	512
ESC_SA8012AA	29	156
ESC_SA8011AA	117	592
ESC_SA8010AA	1140	934
ESC_SA8009AA	162	924
ESC_SA8008AA	58	198
ESC_SA8007AA	1307	257
ESC_SA8006AA	13	257
ESC_SA8005AA	1090	320
ESC_SA8004AA	11521	198
ESC_SA8003AA	11521	198
ESC_SA8002AA	10	512
ESC_SA8001AA	2526	257
ESC_SA8000AA	646	738
ESC_SA7999AA	156	175
ESC_SA7998AA	88	160
ESC_SA7997AA	13	257
ESC_SA7996AA	8988	737
ESC_SA7995AA	3695	924
ESC_SA7994AA	4260	737
ESC_SA7993AA	491	626

ESC_SA7992AA	162	924
ESC_SA7991AA	10	512
ESC_SA7990AA	2785	175
ESC_SA7989AA	491	626
ESC_SA7988AA	90	156
ESC_SA7987AA	11520	595
ESC_SA7986AA	3602	234
ESC_SA7985AA	58	198
ESC_SA7984AA	10	512
ESC_SA7983AA	491	626
ESC_SA7982AA	58	198
ESC_SA7981AA	973	968
ESC_SA7980AA	8211	257
ESC_SA7979AA	3293	936
ESC_SA7978AA	10	512
ESC_SA7977AA	69	387
ESC_SA7976AA	69	387
ESC_SA7975AA	69	387
ESC_SA7974AA	372	424
ESC_SA7973AA	162	924
ESC_SA7972AA	73	737
ESC_SA7971AA	38	669
ESC_SA7970AA	1317	740
ESC_SA7969AA	127	738
ESC_SA7968AA	1060	512
ESC_SA7967AA	1060	512
ESC_SA7966AA	1060	512
ESC_SA7965AA	1060	512
ESC_SA7964AA	1060	512
ESC_SA7963AA	10	512
ESC_SA7962AA	10	512
ESC_SA7961AA	10	512
ESC_SA7960AA	1060	512
ESC_SA7959AA	1060	512
ESC_SA7958AA	10	512
ESC_SA7957AA	10	512
ESC_SA7956AA	1060	512
ESC_SA7955AA	1060	512
ESC_SA7954AA	10	512
ESC_SA7953AA	10	512
ESC_SA7952AA	1060	512
ESC_SA7951AA	1060	512
ESC_SA7950AA	963	669
ESC_SA7949AA	720	900
ESC_SA7948AA	127	738
ESC_SA7947AA	372	424
ESC_SA7946AA	38	669
ESC_SA7945AA	1735	737
ESC_SA7944AA	11443	923
ESC_SA7943AA	372	424

ESC_SA7942AA	929	742
ESC_SA7941AA	372	424
ESC_SA7940AA	5559	257
ESC_SA7939AA	11258	653
ESC_SA7938AA	11486	234
ESC_SA7937AA	10675	926
ESC_SA7936AA	301	307
ESC_SA7935AA	301	307
ESC_SA7934AA	301	307
ESC_SA7933AA	301	307
ESC_SA7932AA	245	261
ESC_SA7931AA	152	257
ESC_SA7930AA	245	261
ESC_SA7929AA	245	261
ESC_SA7928AA	245	261
ESC_SA7927AA	245	261
ESC_SA7926AA	152	257
ESC_SA7925AA	152	257
ESC_SA7924AA	152	257
ESC_SA7923AA	21	156
ESC_SA7922AA	21	156
ESC_SA7921AA	11	936
ESC_SA7920AA	11	936
ESC_SA7919AA	3595	822
ESC_SA7918AA	3595	822
ESC_SA7917AA	3595	822
ESC_SA7916AA	3595	822
ESC_SA7915AA	3595	822
ESC_SA7914AA	3595	822
ESC_SA7913AA	152	257
ESC_SA7912AA	4980	445
ESC_SA7911AA	2179	924
ESC_SA7910AA	4980	445
ESC_SA7909AA	2179	924
ESC_SA7908AA	2966	936
ESC_SA7907AA	11457	936
ESC_SA7906AA	11457	936
ESC_SA7905AA	11458	936
ESC_SA7904AA	11457	936
ESC_SA7903AA	11457	936
ESC_SA7902AA	95	738
ESC_SA7901AA	746	789
ESC_SA7900AA	95	738
ESC_SA7899AA	117	592
ESC_SA7898AA	117	592
ESC_SA7897AA	10	512
ESC_SA7896AA	117	592
ESC_SA7895AA	117	592
ESC_SA7894AA	390	738
ESC_SA7893AA	390	738

ESC_SA7892AA	390	738
ESC_SA7891AA	390	738
ESC_SA7890AA	390	738
ESC_SA7888AA	3595	822
ESC_SA7887AA	3595	822
ESC_SA7886AA	3595	822
ESC_SA7885AA	3595	822
ESC_SA7884AA	3595	822
ESC_SA7883AA	3595	822
ESC_SA7882AA	3595	822
ESC_SA7881AA	3595	822
ESC_SA7880AA	3595	822
ESC_SA7879AA	3595	822
ESC_SA7878AA	3595	822
ESC_SA7877AA	58	198
ESC_SA7876AA	3595	822
ESC_SA7875AA	58	198
ESC_SA7874AA	58	198
ESC_SA7873AA	58	198
ESC_SA7872AA	206	286
ESC_SA7871AA	206	286
ESC_SA7870AA	206	286
ESC_SA7869AA	206	286
ESC_SA7868AA	3595	822
ESC_SA7867AA	3595	822
ESC_SA7866AA	3595	822
ESC_SA7865AA	3595	822
ESC_SA7864AA	3595	822
ESC_SA7863AA	3595	822
ESC_SA7862AA	3595	822
ESC_SA7861AA	3595	822
ESC_SA7860AA	3595	822
ESC_SA7859AA	3595	822
ESC_SA7858AA	3595	822
ESC_SA7857AA	3595	822
ESC_SA7856AA	3595	822
ESC_SA7855AA	3595	822
ESC_SA7854AA	3595	822
ESC_SA7853AA	11415	458
ESC_SA7852AA	11416	504
ESC_SA7851AA	4379	878
ESC_SA7850AA	11415	458
ESC_SA7849AA	23	156
ESC_SA7848AA	4379	878
ESC_SA7847AA	4379	878
ESC_SA7846AA	4379	878
ESC_SA7845AA	11415	458
ESC_SA7844AA	10	512
ESC_SA7843AA	10	512
ESC_SA7842AA	4379	878

ESC_SA7841AA	4379	878
ESC_SA7840AA	4379	878
ESC_SA7839AA	3595	822
ESC_SA7838AA	4379	878
ESC_SA7837AA	4379	878
ESC_SA7836AA	4379	878
ESC_SA7835AA	10	512
ESC_SA7834AA	3595	822
ESC_SA7833AA	3595	822
ESC_SA7832AA	3595	822
ESC_SA7831AA	155	175
ESC_SA7830AA	3595	822
ESC_SA7829AA	155	175
ESC_SA7828AA	3595	822
ESC_SA7827AA	58	198
ESC_SA7826AA	58	198
ESC_SA7825AA	1193	761
ESC_SA7824AA	62	574
ESC_SA7823AA	95	738
ESC_SA7822AA	95	738
ESC_SA7821AA	12	626
ESC_SA7820AA	1193	761
ESC_SA7819AA	1193	761
ESC_SA7818AA	1193	761
ESC_SA7817AA	29	156
ESC_SA7816AA	11	936
ESC_SA7815AA	21	156
ESC_SA7814AA	301	307
ESC_SA7813AA	11	936
ESC_SA7812AA	655	926
ESC_SA7811AA	34	504
ESC_SA7810AA	662	470
ESC_SA7809AA	131	653
ESC_SA7808AA	635	141
ESC_SA7807AA	58	198
ESC_SA7806AA	11505	512
ESC_SA7805AA	679	257
ESC_SA7804AA	154	257
ESC_SA7803AA	6096	257
ESC_SA7802AA	641	935
ESC_SA7801AA	906	198
ESC_SA7800AA	155	175
ESC_SA7799AA	5940	555
ESC_SA7798AA	57	50
ESC_SA7797AA	23	156
ESC_SA7796AA	11504	198
ESC_SA7795AA	351	268
ESC_SA7794AA	906	198
ESC_SA7793AA	278	924
ESC_SA7792AA	1244	291

ESC_SA7791AA	906	198
ESC_SA7790AA	58	198
ESC_SA7789AA	2178	924
ESC_SA7788AA	58	198
ESC_SA7768AA	11	936
ESC_SA7767AA	11	936
ESC_SA7766AA	11	936
ESC_SA7765AA	11	936
ESC_SA7764AA	11	936
ESC_SA7763AA	11	936
ESC_SA7762AA	11	936
ESC_SA7761AA	11	936
ESC_SA7760AA	11	936
ESC_SA7759AA	11	936
ESC_SA7758AA	11	936
ESC_SA7757AA	1408	445
ESC_SA7756AA	2536	261
ESC_SA7755AA	155	175
ESC_SA7754AA	141	644
ESC_SA7753AA	3033	737
ESC_SA7752AA	10066	198
ESC_SA7751AA	1385	653
ESC_SA7750AA	117	592
ESC_SA7749AA	162	924
ESC_SA7748AA	224	198
ESC_SA7747AA	1167	257
ESC_SA7746AA	1236	380
ESC_SA7745AA	131	653
ESC_SA7744AA	131	653
ESC_SA7743AA	131	653
ESC_SA7742AA	131	653
ESC_SA7741AA	245	261
ESC_SA7740AA	245	261
ESC_SA7739AA	245	261
ESC_SA7738AA	245	261
ESC_SA7737AA	442	257
ESC_SA7735AA	33	198
ESC_SA7734AA	11237	285
ESC_SA7733AA	635	141
ESC_SA7732AA	58	198
ESC_SA7731AA	973	968
ESC_SA7730AA	21	156
ESC_SA7729AA	442	257
ESC_SA7728AA	811	234
ESC_SA7727AA	119	286
ESC_SA7726AA	1819	808
ESC_SA7725AA	21	156
ESC_SA7724AA	8708	198
ESC_SA7723AA	137	270
ESC_SA7722AA	7931	234

ESC_SA7721AA	442	257
ESC_SA7720AA	752	512
ESC_SA7719AA	33	198
ESC_SA7718AA	1819	808
ESC_SA7717AA	121	537
ESC_SA7716AA	8549	512
ESC_SA7715AA	21	156
ESC_SA7714AA	906	198
ESC_SA7713AA	10	512
ESC_SA7712AA	21	156
ESC_SA7711AA	21	156
ESC_SA7710AA	117	592
ESC_SA7709AA	2025	194
ESC_SA7708AA	681	393
ESC_SA7707AA	7804	369
ESC_SA7705AA	162	924
ESC_SA7704AA	162	924
ESC_SA7703AA	75	257
ESC_SA7702AA	75	257
ESC_SA7701AA	75	257
ESC_SA7700AA	75	257
ESC_SA7699AA	75	257
ESC_SA7698AA	75	257
ESC_SA7697AA	5937	257
ESC_SA7696AA	642	924
ESC_SA7695AA	1642	198
ESC_SA7694AA	1123	257
ESC_SA7693AA	4682	175
ESC_SA7692AA	10	512
ESC_SA7691AA	847	257
ESC_SA7690AA	718	257
ESC_SA7689AA	718	257
ESC_SA7688AA	718	257
ESC_SA7687AA	718	257
ESC_SA7686AA	1252	567
ESC_SA7685AA	1001	257
ESC_SA7684AA	10	512
ESC_SA7683AA	5748	924
ESC_SA7682AA	718	257
ESC_SA7681AA	1246	198
ESC_SA7680AA	1246	198
ESC_SA7679AA	746	789
ESC_SA7678AA	746	789
ESC_SA7677AA	6968	935
ESC_SA7676AA	6968	935
ESC_SA7675AA	297	234
ESC_SA7674AA	1679	160
ESC_SA7673AA	1679	160
ESC_SA7672AA	1679	160
ESC_SA7671AA	1146	198

ESC_SA7670AA	1146	198
ESC_SA7669AA	1146	198
ESC_SA7668AA	685	311
ESC_SA7667AA	58	198
ESC_SA7666AA	10	512
ESC_SA7665AA	10	512
ESC_SA7664AA	10	512
ESC_SA7663AA	2005	175
ESC_SA7662AA	2005	175
ESC_SA7661AA	2005	175
ESC_SA7660AA	58	198
ESC_SA7659AA	58	198
ESC_SA7658AA	58	198
ESC_SA7657AA	602	257
ESC_SA7656AA	88	160
ESC_SA7655AA	88	160
ESC_SA7654AA	88	160
ESC_SA7652AA	109	257
ESC_SA7651AA	10	512
ESC_SA7650AA	1139	447
ESC_SA7649AA	10	512
ESC_SA7648AA	4087	512
ESC_SA7647AA	4087	512
ESC_SA7646AA	4087	512
ESC_SA7645AA	641	935
ESC_SA7644AA	1642	198
ESC_SA7643AA	1642	198
ESC_SA7642AA	1679	160
ESC_SA7641AA	1679	160
ESC_SA7640AA	1679	160
ESC_SA7639AA	1679	160
ESC_SA7638AA	1679	160
ESC_SA7637AA	5565	198
ESC_SA7636AA	10	512
ESC_SA7635AA	10	512
ESC_SA7634AA	11503	141
ESC_SA7633AA	11503	141
ESC_SA7632AA	10	512
ESC_SA7631AA	10	512
ESC_SA7630AA	10	512
ESC_SA7628AA	8907	234
ESC_SA7627AA	11502	512
ESC_SA7626AA	316	257
ESC_SA7625AA	1423	198
ESC_SA7624AA	1252	567
ESC_SA7623AA	1252	567
ESC_SA7622AA	1252	567
ESC_SA7621AA	5937	257
ESC_SA7620AA	10	512
ESC_SA7619AA	10	512

ESC_SA7618AA	718	257
ESC_SA7617AA	8907	234
ESC_SA7616AA	718	257
ESC_SA7615AA	718	257
ESC_SA7614AA	5937	257
ESC_SA7613AA	1001	257
ESC_SA7612AA	2005	175
ESC_SA7611AA	2005	175
ESC_SA7610AA	2005	175
ESC_SA7609AA	2005	175
ESC_SA7608AA	642	924
ESC_SA7607AA	642	924
ESC_SA7606AA	642	924
ESC_SA7605AA	95	738
ESC_SA7604AA	131	653
ESC_SA7603AA	131	653
ESC_SA7602AA	11390	156
ESC_SA7601AA	245	261
ESC_SA7600AA	245	261
ESC_SA7599AA	90	156
ESC_SA7562AA	17	191
ESC_SA7561AA	21	156
ESC_SA7560AA	101	696
ESC_SA7559AA	101	696
ESC_SA7558AA	5728	1004
ESC_SA7557AA	278	924
ESC_SA7556AA	154	257
ESC_SA7555AA	2521	257
ESC_SA7554AA	329	355
ESC_SA7553AA	328	924
ESC_SA7552AA	1147	198
ESC_SA7551AA	10	512
ESC_SA7550AA	11500	512
ESC_SA7549AA	949	175
ESC_SA7548AA	540	286
ESC_SA7547AA	772	76
ESC_SA7546AA	155	175
ESC_SA7545AA	11499	286
ESC_SA7544AA	657	592
ESC_SA7543AA	23	156
ESC_SA7542AA	7450	512
ESC_SA7541AA	1721	311
ESC_SA7540AA	10	512
ESC_SA7539AA	11	936
ESC_SA7538AA	11	936
ESC_SA7537AA	10	512
ESC_SA7536AA	722	422
ESC_SA7535AA	11	936
ESC_SA7534AA	11	936
ESC_SA7533AA	11	936

ESC_SA7532AA	11501	286
ESC_SA7531AA	11389	261
ESC_SA7530AA	245	261
ESC_SA7529AA	6200	261
ESC_SA7528AA	245	261
ESC_SA7527AA	245	261
ESC_SA7526AA	152	257
ESC_SA7525AA	410	156
ESC_SA7524AA	88	160
ESC_SA7523AA	4087	512
ESC_SA7522AA	1136	108
ESC_SA7520AA	32	270
ESC_SA7519AA	10	512
ESC_SA7518AA	29	156
ESC_SA7517AA	28	537
ESC_SA7516AA	11	936
ESC_SA7515AA	11	936
ESC_SA7514AA	29	156
ESC_SA7513AA	32	270
ESC_SA7512AA	722	422
ESC_SA7511AA	17	191
ESC_SA7510AA	11	936
ESC_SA7509AA	11	936
ESC_SA7508AA	32	270
ESC_SA7507AA	11	936
ESC_SA7506AA	21	156
ESC_SA7505AA	410	156
ESC_SA7504AA	152	257
ESC_SA7502AA	245	261
ESC_SA7501AA	245	261
ESC_SA7500AA	10	512
ESC_SA7499AA	10	512
ESC_SA7498AA	641	935
ESC_SA7497AA	131	653
ESC_SA7496AA	127	738
ESC_SA7495AA	12	626
ESC_SA7494AA	144	740
ESC_SA7493AA	69	387
ESC_SA7492AA	1967	191
ESC_SA7490AA	162	924
ESC_SA7487AA	21	156
ESC_SA7486AA	11	936
ESC_SA7485AA	306	286
ESC_SA7484AA	301	307
ESC_SA7483AA	301	307
ESC_SA7482AA	301	307
ESC_SA7480AA	21	156
ESC_SA7479AA	21	156
ESC_SA7478AA	21	156
ESC_SA7477AA	11488	1022

ESC_SA7476AA	101	696
ESC_SA7475AA	345	175
ESC_SA7474AA	29	156
ESC_SA7473AA	11490	136
ESC_SA7472AA	297	234
ESC_SA7471AA	362	234
ESC_SA7470AA	162	924
ESC_SA7469AA	11489	175
ESC_SA7468AA	345	175
ESC_SA7467AA	10	512
ESC_SA7466AA	10	512
ESC_SA7465AA	58	198
ESC_SA7464AA	4151	971
ESC_SA7463AA	616	156
ESC_SA7462AA	2792	886
ESC_SA7461AA	3714	175
ESC_SA7460AA	515	1004
ESC_SA7459AA	398	445
ESC_SA7458AA	10	512
ESC_SA7457AA	88	160
ESC_SA7455AA	33	198
ESC_SA7454AA	10	512
ESC_SA7453AA	165	504
ESC_SA7452AA	21	156
ESC_SA7451AA	21	156
ESC_SA7450AA	11488	1022
ESC_SA7449AA	21	156
ESC_SA7448AA	21	156
ESC_SA7447AA	21	156
ESC_SA7446AA	21	156
ESC_SA7445AA	21	156
ESC_SA7444AA	21	156
ESC_SA7443AA	2601	785
ESC_SA7442AA	9956	878
ESC_SA7441AA	1679	160
ESC_SA7440AA	602	257
ESC_SA7439AA	3580	257
ESC_SA7438AA	1251	71
ESC_SA7437AA	38	669
ESC_SA7436AA	10	512
ESC_SA7435AA	243	197
ESC_SA7434AA	906	198
ESC_SA7433AA	457	595
ESC_SA7432AA	155	175
ESC_SA7431AA	399	198
ESC_SA7430AA	11485	257
ESC_SA7429AA	131	653
ESC_SA7428AA	2473	257
ESC_SA7427AA	10	512
ESC_SA7426AA	8608	512

ESC_SA7425AA	2144	257
ESC_SA7424AA	2175	212
ESC_SA7423AA	635	141
ESC_SA7422AA	2722	234
ESC_SA7421AA	58	198
ESC_SA7420AA	1434	512
ESC_SA7419AA	501	900
ESC_SA7418AA	898	108
ESC_SA7417AA	428	654
ESC_SA7416AA	1434	512
ESC_SA7415AA	11484	898
ESC_SA7414AA	641	935
ESC_SA7407AA	38	669
ESC_SA7406AA	1193	761
ESC_SA7405AA	131	653
ESC_SA7404AA	131	653
ESC_SA7403AA	131	653
ESC_SA7402AA	3580	257
ESC_SA7401AA	38	669
ESC_SA7400AA	6745	885
ESC_SA7399AA	6745	885
ESC_SA7398AA	38	669
ESC_SA7397AA	38	669
ESC_SA7396AA	38	669
ESC_SA7395AA	38	669
ESC_SA7394AA	1193	761
ESC_SA7393AA	1193	761
ESC_SA7392AA	38	669
ESC_SA7391AA	3580	257
ESC_SA7390AA	38	669
ESC_SA7389AA	131	653
ESC_SA7388AA	38	669
ESC_SA7387AA	38	669
ESC_SA7386AA	38	669
ESC_SA7385AA	38	669
ESC_SA7384AA	131	653
ESC_SA7383AA	131	653
ESC_SA7382AA	38	669
ESC_SA7381AA	131	653
ESC_SA7380AA	131	653
ESC_SA7379AA	3580	257
ESC_SA7378AA	131	653
ESC_SA7377AA	131	653
ESC_SA7376AA	131	653
ESC_SA7375AA	1193	761
ESC_SA7374AA	38	669
ESC_SA7373AA	131	653
ESC_SA7372AA	1193	761
ESC_SA7371AA	131	653
ESC_SA7370AA	131	653

ESC_SA7369AA	38	669
ESC_SA7368AA	131	653
ESC_SA7367AA	38	669
ESC_SA7366AA	131	653
ESC_SA7365AA	131	653
ESC_SA7364AA	131	653
ESC_SA7363AA	141	644
ESC_SA7362AA	141	644
ESC_SA7361AA	141	644
ESC_SA7360AA	141	644
ESC_SA7359AA	141	644
ESC_SA7358AA	141	644
ESC_SA7357AA	141	644
ESC_SA7356AA	141	644
ESC_SA7355AA	141	644
ESC_SA7354AA	141	644
ESC_SA7353AA	141	644
ESC_SA7352AA	141	644
ESC_SA7351AA	141	644
ESC_SA7350AA	141	644
ESC_SA7349AA	141	644
ESC_SA7348AA	141	644
ESC_SA7347AA	141	644
ESC_SA7330AA	152	257
ESC_SA7329AA	3101	160
ESC_SA7328AA	33	198
ESC_SA7327AA	442	257
ESC_SA7326AA	25	234
ESC_SA7325AA	11	936
ESC_SA7324AA	398	445
ESC_SA7323AA	23	156
ESC_SA7322AA	3101	160
ESC_SA7321AA	335	936
ESC_SA7320AA	589	650
ESC_SA7319AA	526	745
ESC_SA7318AA	10	512
ESC_SA7317AA	536	141
ESC_SA7316AA	345	175
ESC_SA7315AA	349	670
ESC_SA7314AA	21	156
ESC_SA7313AA	165	504
ESC_SA7311AA	245	261
ESC_SA7310AA	245	261
ESC_SA7309AA	152	257
ESC_SA7308AA	152	257
ESC_SA7307AA	152	257
ESC_SA7306AA	245	261
ESC_SA7305AA	152	257
ESC_SA7304AA	245	261
ESC_SA7303AA	152	257

ESC_SA7302AA	1503	257
ESC_SA7301AA	152	257
ESC_SA7300AA	245	261
ESC_SA7299AA	245	261
ESC_SA7298AA	631	204
ESC_SA7297AA	152	257
ESC_SA7296AA	152	257
ESC_SA7295AA	152	257
ESC_SA7294AA	245	261
ESC_SA7293AA	245	261
ESC_SA7292AA	245	261
ESC_SA7291AA	152	257
ESC_SA7290AA	245	261
ESC_SA7289AA	152	257
ESC_SA7288AA	11474	198
ESC_SA7287AA	152	257
ESC_SA7286AA	152	257
ESC_SA7285AA	152	257
ESC_SA7284AA	245	261
ESC_SA7283AA	245	261
ESC_SA7282AA	245	261
ESC_SA7281AA	245	261
ESC_SA7280AA	245	261
ESC_SA7279AA	152	257
ESC_SA7278AA	245	261
ESC_SA7277AA	245	261
ESC_SA7276AA	245	261
ESC_SA7275AA	152	257
ESC_SA7274AA	245	261
ESC_SA7273AA	631	204
ESC_SA7272AA	152	257
ESC_SA7271AA	245	261
ESC_SA7270AA	152	257
ESC_SA7269AA	245	261
ESC_SA7268AA	152	257
ESC_SA7267AA	152	257
ESC_SA7266AA	152	257
ESC_SA7265AA	245	261
ESC_SA7264AA	152	257
ESC_SA7263AA	152	257
ESC_SA7262AA	152	257
ESC_SA7261AA	6200	261
ESC_SA7260AA	245	261
ESC_SA7259AA	245	261
ESC_SA7257AA	152	257
ESC_SA7256AA	245	261
ESC_SA7255AA	245	261
ESC_SA7254AA	152	257
ESC_SA7253AA	245	261
ESC_SA7252AA	11	936

ESC_SA7251AA	227	504
ESC_SA7250AA	21	156
ESC_SA7249AA	4	76
ESC_SA7248AA	182	246
ESC_SA7247AA	6044	768
ESC_SA7246AA	21	156
ESC_SA7245AA	722	422
ESC_SA7244AA	10848	512
ESC_SA7243AA	278	924
ESC_SA7242AA	11	936
ESC_SA7241AA	583	768
ESC_SA7240AA	40	198
ESC_SA7239AA	3762	83
ESC_SA7238AA	11471	121
ESC_SA7237AA	38	669
ESC_SA7236AA	2707	924
ESC_SA7235AA	4767	512
ESC_SA7234AA	28	537
ESC_SA7233AA	678	257
ESC_SA7232AA	11473	85
ESC_SA7231AA	11471	121
ESC_SA7230AA	1839	84
ESC_SA7229AA	10	512
ESC_SA7228AA	5399	87
ESC_SA7227AA	335	936
ESC_SA7226AA	706	422
ESC_SA7225AA	39	156
ESC_SA7224AA	10559	773
ESC_SA7223AA	10	512
ESC_SA7222AA	11	936
ESC_SA7221AA	182	246
ESC_SA7220AA	4	76
ESC_SA7219AA	5748	924
ESC_SA7218AA	381	156
ESC_SA7217AA	10	512
ESC_SA7216AA	722	422
ESC_SA7215AA	21	156
ESC_SA7214AA	1491	76
ESC_SA7213AA	6259	257
ESC_SA7212AA	752	512
ESC_SA7211AA	11471	121
ESC_SA7210AA	11471	121
ESC_SA7209AA	21	156
ESC_SA7208AA	11	936
ESC_SA7207AA	788	659
ESC_SA7206AA	655	926
ESC_SA7205AA	206	286
ESC_SA7204AA	11	936
ESC_SA7203AA	11472	1010
ESC_SA7202AA	589	650

ESC_SA7201AA	5399	87
ESC_SA7200AA	793	286
ESC_SA7199AA	10	512
ESC_SA7198AA	11470	276
ESC_SA7197AA	11	936
ESC_SA7196AA	3304	645
ESC_SA7195AA	678	257
ESC_SA7194AA	34	504
ESC_SA7193AA	1	667
ESC_SA7192AA	381	156
ESC_SA7191AA	6259	257
ESC_SA7190AA	583	768
ESC_SA7189AA	378	504
ESC_SA7188AA	4213	251
ESC_SA7187AA	11471	121
ESC_SA7186AA	348	175
ESC_SA7185AA	5540	373
ESC_SA7184AA	11470	276
ESC_SA7183AA	1819	808
ESC_SA7182AA	11	936
ESC_SA7181AA	17	191
ESC_SA7180AA	11469	87
ESC_SA7179AA	11	936
ESC_SA7178AA	21	156
ESC_SA7177AA	1839	84
ESC_SA7176AA	3101	160
ESC_SA7175AA	69	387
ESC_SA7174AA	423	204
ESC_SA7173AA	34	504
ESC_SA7172AA	17	191
ESC_SA7171AA	11479	251
ESC_SA7170AA	17	191
ESC_SA7169AA	11471	121
ESC_SA7168AA	11	936
ESC_SA7167AA	11476	156
ESC_SA7166AA	11	936
ESC_SA7165AA	681	393
ESC_SA7164AA	5399	87
ESC_SA7163AA	11	936
ESC_SA7162AA	517	924
ESC_SA7161AA	316	257
ESC_SA7160AA	1406	174
ESC_SA7159AA	11	936
ESC_SA7158AA	11478	85
ESC_SA7157AA	2346	435
ESC_SA7156AA	2356	379
ESC_SA7155AA	1033	537
ESC_SA7154AA	137	270
ESC_SA7153AA	11478	85
ESC_SA7152AA	11	936

ESC_SA7151AA	11	936
ESC_SA7150AA	642	924
ESC_SA7149AA	10	512
ESC_SA7148AA	602	257
ESC_SA7146AA	655	926
ESC_SA7145AA	4058	198
ESC_SA7144AA	8237	504
ESC_SA7143AA	301	307
ESC_SA7142AA	5784	448
ESC_SA7141AA	101	696
ESC_SA7140AA	11	936
ESC_SA7139AA	11	936
ESC_SA7138AA	10	512
ESC_SA7137AA	569	738
ESC_SA7136AA	394	400
ESC_SA7135AA	753	842
ESC_SA7134AA	589	650
ESC_SA7133AA	583	768
ESC_SA7132AA	11	936
ESC_SA7131AA	2179	924
ESC_SA7130AA	1491	76
ESC_SA7129AA	1817	842
ESC_SA7128AA	20	198
ESC_SA7127AA	11478	85
ESC_SA7126AA	3910	672
ESC_SA7125AA	182	246
ESC_SA7124AA	1136	108
ESC_SA7123AA	33	198
ESC_SA7122AA	6700	512
ESC_SA7121AA	5967	87
ESC_SA7120AA	21	156
ESC_SA7119AA	34	504
ESC_SA7118AA	11	936
ESC_SA7117AA	2700	118
ESC_SA7116AA	11	936
ESC_SA7115AA	48	76
ESC_SA7114AA	200	198
ESC_SA7113AA	11477	1012
ESC_SA7112AA	602	257
ESC_SA7111AA	11471	121
ESC_SA7110AA	1877	773
ESC_SA7109AA	11	936
ESC_SA7108AA	32	270
ESC_SA7107AA	11	936
ESC_SA7106AA	583	768
ESC_SA7105AA	11	936
ESC_SA7104AA	301	307
ESC_SA7102AA	11	936
ESC_SA7101AA	517	924
ESC_SA7100AA	10	512

ESC_SA7099AA	11476	156
ESC_SA7098AA	327	204
ESC_SA7097AA	378	504
ESC_SA7096AA	10	512
ESC_SA7095AA	4	76
ESC_SA7094AA	11	936
ESC_SA7093AA	678	257
ESC_SA7092AA	1839	84
ESC_SA7091AA	33	198
ESC_SA7090AA	28	537
ESC_SA7089AA	11471	121
ESC_SA7088AA	5342	765
ESC_SA7087AA	29	156
ESC_SA7086AA	11	936
ESC_SA7085AA	127	738
ESC_SA7084AA	6048	87
ESC_SA7083AA	167	512
ESC_SA7082AA	11475	122
ESC_SA7081AA	11	936
ESC_SA7080AA	29	156
ESC_SA7079AA	21	156
ESC_SA7078AA	38	669
ESC_SA7077AA	33	198
ESC_SA7076AA	4	76
ESC_SA7075AA	11	936
ESC_SA7074AA	11	936
ESC_SA7073AA	11471	121
ESC_SA7072AA	678	257
ESC_SA7071AA	5371	716
ESC_SA7070AA	10	512
ESC_SA7069AA	11471	121
ESC_SA7068AA	1892	846
ESC_SA7067AA	1049	175
ESC_SA7066AA	200	198
ESC_SA7065AA	491	626
ESC_SA7064AA	4722	537
ESC_SA7063AA	11	936
ESC_SA7062AA	34	504
ESC_SA7061AA	21	156
ESC_SA7060AA	9998	117
ESC_SA7059AA	29	156
ESC_SA7058AA	655	926
ESC_SA7057AA	8825	304
ESC_SA7056AA	6506	914
ESC_SA7055AA	21	156
ESC_SA7054AA	1839	84
ESC_SA7053AA	17	191
ESC_SA7052AA	121	537
ESC_SA7051AA	10698	512
ESC_SA7050AA	182	246

ESC_SA7049AA	342	286
ESC_SA7048AA	752	512
ESC_SA7047AA	301	307
ESC_SA7046AA	29	156
ESC_SA7045AA	1260	512
ESC_SA7044AA	10002	89
ESC_SA7043AA	443	257
ESC_SA7042AA	11	936
ESC_SA7041AA	442	257
ESC_SA7040AA	11471	121
ESC_SA7039AA	11	936
ESC_SA7038AA	21	156
ESC_SA7037AA	11	936
ESC_SA7036AA	4980	445
ESC_SA7035AA	5399	87
ESC_SA7034AA	5415	924
ESC_SA7033AA	11	936
ESC_SA7032AA	11483	373
ESC_SA7031AA	11	936
ESC_SA7030AA	201	257
ESC_SA7029AA	405	923
ESC_SA7028AA	11	936
ESC_SA7027AA	5399	87
ESC_SA7026AA	11482	118
ESC_SA7025AA	4	76
ESC_SA7024AA	11481	1011
ESC_SA7023AA	5390	83
ESC_SA7022AA	11	936
ESC_SA7021AA	526	745
ESC_SA7020AA	16	156
ESC_SA7019AA	335	936
ESC_SA7018AA	1839	84
ESC_SA7017AA	2179	924
ESC_SA7016AA	29	156
ESC_SA7015AA	11	936
ESC_SA7014AA	137	270
ESC_SA7013AA	189	504
ESC_SA7012AA	4981	473
ESC_SA7011AA	327	204
ESC_SA7010AA	2682	87
ESC_SA7009AA	11	936
ESC_SA7008AA	25	234
ESC_SA7007AA	301	307
ESC_SA7006AA	4980	445
ESC_SA7005AA	381	156
ESC_SA7004AA	21	156
ESC_SA7003AA	327	204
ESC_SA7002AA	201	257
ESC_SA7001AA	4642	156
ESC_SA7000AA	746	789

ESC_SA6999AA	2178	924
ESC_SA6998AA	1312	76
ESC_SA6997AA	38	669
ESC_SA6996AA	975	512
ESC_SA6995AA	5547	622
ESC_SA6994AA	11	936
ESC_SA6993AA	11480	286
ESC_SA6992AA	21	156
ESC_SA6991AA	10	512
ESC_SA6990AA	11	936
ESC_SA6989AA	10	512
ESC_SA6988AA	11471	121
ESC_SA6987AA	20	198
ESC_SA6986AA	10	512
ESC_SA6985AA	11	936
ESC_SA6984AA	1839	84
ESC_SA6983AA	8371	898
ESC_SA6982AA	1611	198
ESC_SA6981AA	152	257
ESC_SA6980AA	829	257
ESC_SA6979AA	245	261
ESC_SA6978AA	245	261
ESC_SA6977AA	145	256
ESC_SA6976AA	152	257
ESC_SA6975AA	152	257
ESC_SA6974AA	245	261
ESC_SA6973AA	245	261
ESC_SA6972AA	152	257
ESC_SA6971AA	152	257
ESC_SA6970AA	315	669
ESC_SA6969AA	59	217
ESC_SA6968AA	245	261
ESC_SA6967AA	245	261
ESC_SA6966AA	145	256
ESC_SA6965AA	245	261
ESC_SA6964AA	718	257
ESC_SA6963AA	21	156
ESC_SA6962AA	442	257
ESC_SA6961AA	17	191
ESC_SA6960AA	297	234
ESC_SA6959AA	33	198
ESC_SA6958AA	442	257
ESC_SA6957AA	328	924
ESC_SA6956AA	131	653
ESC_SA6955AA	21	156
ESC_SA6954AA	245	261
ESC_SA6953AA	245	261
ESC_SA6952AA	373	71
ESC_SA6951AA	101	696
ESC_SA6948AA	3057	672

ESC_SA6946AA	117	592
ESC_SA6945AA	48	76
ESC_SA6943AA	11	936
ESC_SA6942AA	2521	257
ESC_SA6941AA	137	270
ESC_SA6940AA	404	761
ESC_SA6939AA	442	257
ESC_SA6938AA	342	286
ESC_SA6937AA	11	936
ESC_SA6936AA	8296	198
ESC_SA6935AA	137	270
ESC_SA6934AA	11	936
ESC_SA6933AA	738	598
ESC_SA6932AA	11	936
ESC_SA6931AA	765	156
ESC_SA6930AA	11	936
ESC_SA6929AA	1041	765
ESC_SA6928AA	11468	142
ESC_SA6927AA	11	936
ESC_SA6926AA	589	650
ESC_SA6925AA	442	257
ESC_SA6924AA	32	270
ESC_SA6923AA	589	650
ESC_SA6922AA	300	286
ESC_SA6921AA	5418	257
ESC_SA6919AA	7562	546
ESC_SA6918AA	11	936
ESC_SA6917AA	34	504
ESC_SA6916AA	11	936
ESC_SA6915AA	642	924
ESC_SA6914AA	12	626
ESC_SA6913AA	6992	842
ESC_SA6912AA	21	156
ESC_SA6911AA	11	936
ESC_SA6910AA	58	198
ESC_SA6909AA	345	175
ESC_SA6908AA	216	512
ESC_SA6907AA	607	355
ESC_SA6906AA	399	198
ESC_SA6905AA	11498	935
ESC_SA6904AA	206	286
ESC_SA6889AA	8881	362
ESC_SA6888AA	963	669
ESC_SA6887AA	131	653
ESC_SA6886AA	131	653
ESC_SA6885AA	131	653
ESC_SA6884AA	58	198
ESC_SA6883AA	38	669
ESC_SA6882AA	131	653
ESC_SA6881AA	38	669

ESC_SA6880AA	38	669
ESC_SA6879AA	131	653
ESC_SA6878AA	131	653
ESC_SA6877AA	131	653
ESC_SA6876AA	410	156
ESC_SA6875AA	131	653
ESC_SA6874AA	131	653
ESC_SA6873AA	372	424
ESC_SA6872AA	906	198
ESC_SA6871AA	648	359
ESC_SA6870AA	648	359
ESC_SA6869AA	131	653
ESC_SA6868AA	131	653
ESC_SA6867AA	131	653
ESC_SA6866AA	131	653
ESC_SA6865AA	963	669
ESC_SA6864AA	8492	234
ESC_SA6863AA	73	737
ESC_SA6862AA	131	653
ESC_SA6861AA	131	653
ESC_SA6860AA	131	653
ESC_SA6859AA	131	653
ESC_SA6858AA	617	511
ESC_SA6857AA	73	737
ESC_SA6856AA	131	653
ESC_SA6855AA	11493	512
ESC_SA6854AA	131	653
ESC_SA6853AA	405	923
ESC_SA6852AA	648	359
ESC_SA6851AA	131	653
ESC_SA6850AA	131	653
ESC_SA6849AA	131	653
ESC_SA6848AA	131	653
ESC_SA6847AA	963	669
ESC_SA6846AA	34	504
ESC_SA6845AA	131	653
ESC_SA6844AA	131	653
ESC_SA6843AA	648	359
ESC_SA6842AA	131	653
ESC_SA6841AA	131	653
ESC_SA6840AA	544	626
ESC_SA6839AA	617	511
ESC_SA6838AA	131	653
ESC_SA6837AA	131	653
ESC_SA6836AA	11492	654
ESC_SA6835AA	38	669
ESC_SA6834AA	131	653
ESC_SA6833AA	95	738
ESC_SA6832AA	131	653
ESC_SA6831AA	770	587

ESC_SA6830AA	636	653
ESC_SA6829AA	11491	234
ESC_SA6828AA	8347	653
ESC_SA6827AA	1193	761
ESC_SA6826AA	12	626
ESC_SA6825AA	131	653
ESC_SA6824AA	131	653
ESC_SA6823AA	131	653
ESC_SA6822AA	131	653
ESC_SA6821AA	131	653
ESC_SA6820AA	127	738
ESC_SA6819AA	131	653
ESC_SA6818AA	38	669
ESC_SA6817AA	648	359
ESC_SA6816AA	6395	504
ESC_SA6815AA	245	261
ESC_SA6814AA	245	261
ESC_SA6813AA	245	261
ESC_SA6812AA	245	261
ESC_SA6811AA	442	257
ESC_SA6810AA	11	936
ESC_SA6809AA	21	156
ESC_SA6808AA	6251	808
ESC_SA6807AA	32	270
ESC_SA6806AA	5627	435
ESC_SA6805AA	657	592
ESC_SA6804AA	2101	108
ESC_SA6803AA	28	537
ESC_SA6802AA	2248	257
ESC_SA6801AA	21	156
ESC_SA6800AA	33	198
ESC_SA6799AA	21	156
ESC_SA6798AA	152	257
ESC_SA6797AA	112	738
ESC_SA6796AA	11465	168
ESC_SA6795AA	537	756
ESC_SA6794AA	1868	764
ESC_SA6793AA	537	756
ESC_SA6792AA	11446	1016
ESC_SA6791AA	4957	911
ESC_SA6790AA	8888	764
ESC_SA6789AA	11441	880
ESC_SA6788AA	1868	764
ESC_SA6787AA	11452	1015
ESC_SA6786AA	491	626
ESC_SA6785AA	11446	1016
ESC_SA6784AA	11446	1016
ESC_SA6783AA	537	756
ESC_SA6782AA	1868	764
ESC_SA6781AA	141	644

ESC_SA6780AA	11451	626
ESC_SA6779AA	11446	1016
ESC_SA6778AA	537	756
ESC_SA6777AA	998	644
ESC_SA6776AA	11450	654
ESC_SA6775AA	1868	764
ESC_SA6774AA	11449	764
ESC_SA6773AA	4957	911
ESC_SA6772AA	363	738
ESC_SA6771AA	11449	764
ESC_SA6770AA	11448	626
ESC_SA6769AA	131	653
ESC_SA6768AA	2556	630
ESC_SA6767AA	1727	257
ESC_SA6766AA	906	198
ESC_SA6765AA	1868	764
ESC_SA6764AA	11448	626
ESC_SA6763AA	11441	880
ESC_SA6762AA	2808	737
ESC_SA6761AA	11441	880
ESC_SA6760AA	11449	764
ESC_SA6759AA	11448	626
ESC_SA6758AA	11441	880
ESC_SA6757AA	537	756
ESC_SA6756AA	58	198
ESC_SA6755AA	999	654
ESC_SA6754AA	537	756
ESC_SA6753AA	491	626
ESC_SA6752AA	11447	772
ESC_SA6751AA	537	756
ESC_SA6750AA	224	198
ESC_SA6749AA	11446	1016
ESC_SA6748AA	4957	911
ESC_SA6747AA	1880	653
ESC_SA6746AA	3489	962
ESC_SA6745AA	1880	653
ESC_SA6744AA	4225	924
ESC_SA6743AA	1880	653
ESC_SA6742AA	4957	911
ESC_SA6741AA	141	644
ESC_SA6740AA	2005	175
ESC_SA6739AA	58	198
ESC_SA6738AA	11445	653
ESC_SA6737AA	11107	373
ESC_SA6736AA	3856	789
ESC_SA6735AA	10	512
ESC_SA6734AA	10	512
ESC_SA6733AA	448	257
ESC_SA6732AA	6211	198
ESC_SA6731AA	2005	175

ESC_SA6730AA	641	935
ESC_SA6729AA	641	935
ESC_SA6728AA	93	71
ESC_SA6727AA	11441	880
ESC_SA6726AA	942	198
ESC_SA6725AA	11441	880
ESC_SA6724AA	155	175
ESC_SA6723AA	10	512
ESC_SA6722AA	11443	923
ESC_SA6721AA	11441	880
ESC_SA6720AA	11441	880
ESC_SA6719AA	162	924
ESC_SA6718AA	196	257
ESC_SA6717AA	11443	923
ESC_SA6716AA	11444	1003
ESC_SA6715AA	8888	764
ESC_SA6714AA	69	387
ESC_SA6713AA	11442	868
ESC_SA6712AA	11435	331
ESC_SA6711AA	11441	880
ESC_SA6710AA	321	741
ESC_SA6709AA	38	669
ESC_SA6708AA	5965	765
ESC_SA6707AA	7908	810
ESC_SA6706AA	68	386
ESC_SA6705AA	11436	1025
ESC_SA6703AA	10	512
ESC_SA6702AA	501	900
ESC_SA6701AA	5965	765
ESC_SA6700AA	6495	336
ESC_SA6699AA	11436	1025
ESC_SA6698AA	423	204
ESC_SA6697AA	11435	331
ESC_SA6696AA	2678	737
ESC_SA6695AA	11435	331
ESC_SA6694AA	11437	653
ESC_SA6693AA	363	738
ESC_SA6692AA	155	175
ESC_SA6691AA	11435	331
ESC_SA6690AA	11440	173
ESC_SA6689AA	68	386
ESC_SA6688AA	129	257
ESC_SA6687AA	11439	257
ESC_SA6686AA	8823	257
ESC_SA6685AA	11438	1024
ESC_SA6684AA	7892	951
ESC_SA6683AA	569	738
ESC_SA6682AA	48	76
ESC_SA6681AA	11437	653
ESC_SA6680AA	5155	238

ESC_SA6679AA	11436	1025
ESC_SA6678AA	8823	257
ESC_SA6677AA	4353	387
ESC_SA6676AA	11107	373
ESC_SA6675AA	11434	373
ESC_SA6674AA	11435	331
ESC_SA6673AA	409	504
ESC_SA6672AA	11445	653
ESC_SA6670AA	88	160
ESC_SA6669AA	8841	377
ESC_SA6668AA	1148	257
ESC_SA6667AA	155	175
ESC_SA6666AA	127	738
ESC_SA6665AA	141	644
ESC_SA6664AA	12	626
ESC_SA6663AA	607	355
ESC_SA6662AA	69	387
ESC_SA6661AA	357	654
ESC_SA6660AA	11462	512
ESC_SA6659AA	38	669
ESC_SA6658AA	131	653
ESC_SA6657AA	11464	900
ESC_SA6656AA	10	512
ESC_SA6655AA	683	175
ESC_SA6654AA	191	198
ESC_SA6653AA	6018	52
ESC_SA6652AA	1177	669
ESC_SA6651AA	6018	52
ESC_SA6650AA	11455	738
ESC_SA6649AA	141	644
ESC_SA6648AA	2160	243
ESC_SA6647AA	38	669
ESC_SA6646AA	4623	257
ESC_SA6645AA	11455	738
ESC_SA6644AA	101	696
ESC_SA6643AA	6018	52
ESC_SA6642AA	141	644
ESC_SA6641AA	11463	868
ESC_SA6640AA	297	234
ESC_SA6639AA	141	644
ESC_SA6638AA	11455	738
ESC_SA6637AA	6021	234
ESC_SA6636AA	683	175
ESC_SA6635AA	10	512
ESC_SA6634AA	162	924
ESC_SA6633AA	131	653
ESC_SA6632AA	607	355
ESC_SA6631AA	3168	261
ESC_SA6630AA	11462	512
ESC_SA6629AA	10982	900

ESC_SA6628AA	141	644
ESC_SA6627AA	157	387
ESC_SA6626AA	162	924
ESC_SA6625AA	10982	900
ESC_SA6624AA	141	644
ESC_SA6623AA	141	644
ESC_SA6622AA	357	654
ESC_SA6621AA	607	355
ESC_SA6620AA	226	512
ESC_SA6619AA	95	738
ESC_SA6618AA	58	198
ESC_SA6617AA	141	644
ESC_SA6616AA	448	257
ESC_SA6615AA	141	644
ESC_SA6614AA	589	650
ESC_SA6613AA	401	142
ESC_SA6612AA	357	654
ESC_SA6611AA	3188	257
ESC_SA6610AA	226	512
ESC_SA6609AA	131	653
ESC_SA6608AA	457	595
ESC_SA6607AA	95	738
ESC_SA6606AA	11455	738
ESC_SA6605AA	11456	654
ESC_SA6604AA	550	756
ESC_SA6603AA	337	257
ESC_SA6602AA	2280	212
ESC_SA6601AA	88	160
ESC_SA6600AA	1125	198
ESC_SA6599AA	11453	175
ESC_SA6598AA	141	644
ESC_SA6597AA	11455	738
ESC_SA6596AA	683	175
ESC_SA6595AA	6272	862
ESC_SA6594AA	357	654
ESC_SA6593AA	420	741
ESC_SA6592AA	131	653
ESC_SA6591AA	998	644
ESC_SA6590AA	607	355
ESC_SA6589AA	348	175
ESC_SA6588AA	155	175
ESC_SA6587AA	2079	198
ESC_SA6586AA	2313	257
ESC_SA6585AA	11453	175
ESC_SA6584AA	127	738
ESC_SA6583AA	73	737
ESC_SA6582AA	95	738
ESC_SA6581AA	95	738
ESC_SA6580AA	91	629
ESC_SA6579AA	12	626

ESC_SA6578AA	1421	447
ESC_SA6577AA	69	387
ESC_SA6576AA	538	629
ESC_SA6575AA	93	71
ESC_SA6574AA	8184	653
ESC_SA6573AA	998	644
ESC_SA6572AA	409	504
ESC_SA6571AA	420	741
ESC_SA6570AA	998	644
ESC_SA6569AA	5409	175
ESC_SA6568AA	345	175
ESC_SA6567AA	70	670
ESC_SA6566AA	5409	175
ESC_SA6565AA	409	504
ESC_SA6564AA	607	355
ESC_SA6563AA	7902	400
ESC_SA6562AA	131	653
ESC_SA6561AA	127	738
ESC_SA6560AA	1421	447
ESC_SA6559AA	131	653
ESC_SA6558AA	538	629
ESC_SA6557AA	70	670
ESC_SA6556AA	135	653
ESC_SA6555AA	57	50
ESC_SA6554AA	46	447
ESC_SA6553AA	10	512
ESC_SA6552AA	448	257
ESC_SA6551AA	135	653
ESC_SA6550AA	73	737
ESC_SA6549AA	1421	447
ESC_SA6548AA	1170	629
ESC_SA6547AA	1421	447
ESC_SA6546AA	448	257
ESC_SA6545AA	2521	257
ESC_SA6544AA	847	257
ESC_SA6543AA	70	670
ESC_SA6542AA	70	670
ESC_SA6541AA	131	653
ESC_SA6540AA	420	741
ESC_SA6539AA	6422	198
ESC_SA6538AA	155	175
ESC_SA6537AA	5708	605
ESC_SA6536AA	7285	175
ESC_SA6535AA	607	355
ESC_SA6534AA	4572	261
ESC_SA6533AA	11453	175
ESC_SA6532AA	95	738
ESC_SA6531AA	101	696
ESC_SA6530AA	362	234
ESC_SA6529AA	1056	257

ESC_SA6528AA	38	669
ESC_SA6527AA	127	738
ESC_SA6526AA	95	738
ESC_SA6525AA	11453	175
ESC_SA6524AA	11454	175
ESC_SA6523AA	224	198
ESC_SA6522AA	69	387
ESC_SA6521AA	607	355
ESC_SA6520AA	4456	654
ESC_SA6519AA	6630	599
ESC_SA6518AA	11453	175
ESC_SA6517AA	538	629
ESC_SA6516AA	939	198
ESC_SA6515AA	998	644
ESC_SA6514AA	11	936
ESC_SA6513AA	11	936
ESC_SA6512AA	11	936
ESC_SA6511AA	11	936
ESC_SA6510AA	11	936
ESC_SA6509AA	11	936
ESC_SA6508AA	11	936
ESC_SA6507AA	11	936
ESC_SA6506AA	11	936
ESC_SA6505AA	38	669
ESC_SA6504AA	540	286
ESC_SA6503AA	847	257
ESC_SA6502AA	6539	198
ESC_SA6501AA	3345	445
ESC_SA6500AA	515	1004
ESC_SA6499AA	88	160
ESC_SA6498AA	361	512
ESC_SA6497AA	117	592
ESC_SA6496AA	189	504
ESC_SA6495AA	3580	257
ESC_SA6494AA	93	71
ESC_SA6493AA	11426	76
ESC_SA6492AA	316	257
ESC_SA6491AA	3525	504
ESC_SA6490AA	10	512
ESC_SA6489AA	278	924
ESC_SA6488AA	11425	935
ESC_SA6487AA	58	198
ESC_SA6486AA	877	935
ESC_SA6485AA	88	160
ESC_SA6484AA	69	387
ESC_SA6483AA	58	198
ESC_SA6482AA	162	924
ESC_SA6479AA	245	261
ESC_SA6477AA	152	257
ESC_SA6476AA	152	257

ESC_SA6475AA	152	257
ESC_SA6474AA	152	257
ESC_SA6473AA	152	257
ESC_SA6472AA	152	257
ESC_SA6471AA	152	257
ESC_SA6470AA	152	257
ESC_SA6469AA	152	257
ESC_SA6468AA	152	257
ESC_SA6467AA	152	257
ESC_SA6466AA	152	257
ESC_SA6465AA	152	257
ESC_SA6464AA	152	257
ESC_SA6463AA	152	257
ESC_SA6462AA	152	257
ESC_SA6461AA	152	257
ESC_SA6460AA	152	257
ESC_SA6459AA	152	257
ESC_SA6458AA	152	257
ESC_SA6457AA	152	257
ESC_SA6456AA	152	257
ESC_SA6455AA	152	257
ESC_SA6454AA	152	257
ESC_SA6453AA	152	257
ESC_SA6452AA	152	257
ESC_SA6451AA	152	257
ESC_SA6450AA	152	257
ESC_SA6449AA	152	257
ESC_SA6448AA	152	257
ESC_SA6447AA	152	257
ESC_SA6446AA	152	257
ESC_SA6445AA	152	257
ESC_SA6444AA	152	257
ESC_SA6443AA	152	257
ESC_SA6442AA	152	257
ESC_SA6441AA	152	257
ESC_SA6440AA	152	257
ESC_SA6439AA	152	257
ESC_SA6438AA	152	257
ESC_SA6437AA	152	257
ESC_SA6436AA	152	257
ESC_SA6435AA	152	257
ESC_SA6434AA	152	257
ESC_SA6433AA	152	257
ESC_SA6432AA	152	257
ESC_SA6431AA	152	257
ESC_SA6426AA	10	512
ESC_SA6414AA	48	76
ESC_SA6413AA	515	1004
ESC_SA6412AA	131	653
ESC_SA6411AA	131	653

ESC_SA6410AA	607	355
ESC_SA6409AA	131	653
ESC_SA6408AA	131	653
ESC_SA6407AA	131	653
ESC_SA6406AA	131	653
ESC_SA6405AA	131	653
ESC_SA6404AA	131	653
ESC_SA6403AA	648	359
ESC_SA6402AA	131	653
ESC_SA6401AA	69	387
ESC_SA6400AA	131	653
ESC_SA6397AA	10	512
ESC_SA6396AA	10	512
ESC_SA6395AA	93	71
ESC_SA6394AA	10	512
ESC_SA6393AA	93	71
ESC_SA6392AA	10	512
ESC_SA6391AA	10	512
ESC_SA6390AA	93	71
ESC_SA6389AA	93	71
ESC_SA6387AA	93	71
ESC_SA6386AA	93	71
ESC_SA6385AA	93	71
ESC_SA6384AA	93	71
ESC_SA6383AA	93	71
ESC_SA6382AA	93	71
ESC_SA6381AA	93	71
ESC_SA6380AA	10	512
ESC_SA6379AA	93	71
ESC_SA6378AA	10	512
ESC_SA6377AA	10	512
ESC_SA6376AA	10	512
ESC_SA6375AA	93	71
ESC_SA6374AA	93	71
ESC_SA6373AA	10	512
ESC_SA6372AA	93	71
ESC_SA6371AA	10	512
ESC_SA6370AA	10	512
ESC_SA6369AA	93	71
ESC_SA6368AA	93	71
ESC_SA6367AA	93	71
ESC_SA6366AA	93	71
ESC_SA6365AA	10	512
ESC_SA6364AA	3021	447
ESC_SA6363AA	10	512
ESC_SA6362AA	3021	447
ESC_SA6361AA	3021	447
ESC_SA6360AA	3021	447
ESC_SA6359AA	10	512
ESC_SA6358AA	10	512

ESC_SA6357AA	10	512
ESC_SA6356AA	3021	447
ESC_SA6355AA	10	512
ESC_SA6354AA	7290	512
ESC_SA6352AA	162	924
ESC_SA6325AA	761	512
ESC_SA6324AA	101	696
ESC_SA6323AA	10	512
ESC_SA6322AA	101	696
ESC_SA6321AA	48	76
ESC_SA6320AA	11397	251
ESC_SA6319AA	48	76
ESC_SA6318AA	95	738
ESC_SA6317AA	117	592
ESC_SA6314AA	5926	50
ESC_SA6313AA	162	924
ESC_SA6312AA	48	76
ESC_SA6311AA	5926	50
ESC_SA6309AA	5926	50
ESC_SA6308AA	5926	50
ESC_SA6306AA	117	592
ESC_SA6305AA	390	738
ESC_SA6304AA	2272	160
ESC_SA6303AA	371	50
ESC_SA6302AA	117	592
ESC_SA6301AA	101	696
ESC_SA6300AA	349	670
ESC_SA6299AA	10	512
ESC_SA6298AA	117	592
ESC_SA6297AA	95	738
ESC_SA6296AA	2179	924
ESC_SA6295AA	648	359
ESC_SA6294AA	5925	126
ESC_SA6292AA	23	156
ESC_SA6291AA	23	156
ESC_SA6289AA	6286	592
ESC_SA6288AA	95	738
ESC_SA6287AA	117	592
ESC_SA6286AA	140	738
ESC_SA6285AA	93	71
ESC_SA6284AA	117	592
ESC_SA6283AA	23	156
ESC_SA6282AA	95	738
ESC_SA6281AA	95	738
ESC_SA6280AA	357	654
ESC_SA6279AA	140	738
ESC_SA6278AA	117	592
ESC_SA6277AA	23	156
ESC_SA6271AA	23	156
ESC_SA6269AA	141	644

ESC_SA6268AA	95	738
ESC_SA6266AA	69	387
ESC_SA6257AA	117	592
ESC_SA6256AA	23	156
ESC_SA6253AA	602	257
ESC_SA6252AA	23	156
ESC_SA6250AA	38	669
ESC_SA6249AA	23	156
ESC_SA6246AA	117	592
ESC_SA6245AA	1638	519
ESC_SA6244AA	1638	519
ESC_SA6243AA	1638	519
ESC_SA6242AA	95	738
ESC_SA6241AA	117	592
ESC_SA6240AA	117	592
ESC_SA6239AA	135	653
ESC_SA6238AA	1638	519
ESC_SA6237AA	140	738
ESC_SA6236AA	95	738
ESC_SA6235AA	140	738
ESC_SA6234AA	117	592
ESC_SA6233AA	95	738
ESC_SA6232AA	23	156
ESC_SA6231AA	95	738
ESC_SA6230AA	388	156
ESC_SA6229AA	429	654
ESC_SA6228AA	23	156
ESC_SA6227AA	23	156
ESC_SA6226AA	648	359
ESC_SA6225AA	140	738
ESC_SA6224AA	140	738
ESC_SA6223AA	95	738
ESC_SA6222AA	770	587
ESC_SA6221AA	156	175
ESC_SA6220AA	101	696
ESC_SA6219AA	117	592
ESC_SA6218AA	140	738
ESC_SA6217AA	155	175
ESC_SA6216AA	140	738
ESC_SA6215AA	117	592
ESC_SA6214AA	117	592
ESC_SA6213AA	95	738
ESC_SA6212AA	569	738
ESC_SA6211AA	95	738
ESC_SA6210AA	23	156
ESC_SA6209AA	95	738
ESC_SA6208AA	115	669
ESC_SA6206AA	95	738
ESC_SA6203AA	93	71
ESC_SA6202AA	141	644

ESC_SA6201AA	428	654
ESC_SA6200AA	10	512
ESC_SA6199AA	93	71
ESC_SA6198AA	23	156
ESC_SA6197AA	95	738
ESC_SA6195AA	117	592
ESC_SA6189AA	101	696
ESC_SA6188AA	95	738
ESC_SA6187AA	4045	592
ESC_SA6185AA	1852	654
ESC_SA6184AA	23	156
ESC_SA6183AA	752	512
ESC_SA6182AA	1101	842
ESC_SA6181AA	10774	512
ESC_SA6180AA	752	512
ESC_SA6179AA	69	387
ESC_SA6178AA	752	512
ESC_SA6177AA	11396	359
ESC_SA6176AA	135	653
ESC_SA6175AA	88	160
ESC_SA6174AA	752	512
ESC_SA6173AA	428	654
ESC_SA6172AA	117	592
ESC_SA6170AA	135	653
ESC_SA6169AA	117	592
ESC_SA6168AA	2230	126
ESC_SA6167AA	70	670
ESC_SA6166AA	93	71
ESC_SA6165AA	429	654
ESC_SA6164AA	117	592
ESC_SA6163AA	140	738
ESC_SA6162AA	1431	257
ESC_SA6161AA	95	738
ESC_SA6160AA	429	654
ESC_SA6159AA	5766	198
ESC_SA6156AA	117	592
ESC_SA6155AA	117	592
ESC_SA6151AA	95	738
ESC_SA6150AA	140	738
ESC_SA6149AA	23	156
ESC_SA6143AA	357	654
ESC_SA6142AA	602	257
ESC_SA6141AA	117	592
ESC_SA6139AA	23	156
ESC_SA6137AA	429	654
ESC_SA6136AA	117	592
ESC_SA6133AA	101	696
ESC_SA6132AA	95	738
ESC_SA6129AA	117	592
ESC_SA6128AA	140	738

ESC_SA6127AA	429	654
ESC_SA6126AA	212	198
ESC_SA6125AA	95	738
ESC_SA6124AA	2705	504
ESC_SA6123AA	357	654
ESC_SA6121AA	371	50
ESC_SA6119AA	5925	126
ESC_SA6117AA	1828	738
ESC_SA6116AA	11398	76
ESC_SA6115AA	6286	592
ESC_SA6114AA	457	595
ESC_SA6113AA	429	654
ESC_SA6112AA	10	512
ESC_SA6111AA	1146	198
ESC_SA6110AA	23	156
ESC_SA6109AA	297	234
ESC_SA6108AA	117	592
ESC_SA6107AA	602	257
ESC_SA6106AA	355	764
ESC_SA6105AA	101	696
ESC_SA6104AA	117	592
ESC_SA6103AA	429	654
ESC_SA6102AA	101	696
ESC_SA6101AA	5224	76
ESC_SA6100AA	117	592
ESC_SA6099AA	117	592
ESC_SA6098AA	117	592
ESC_SA6097AA	23	156
ESC_SA6096AA	23	156
ESC_SA6095AA	357	654
ESC_SA6094AA	141	644
ESC_SA6093AA	155	175
ESC_SA6092AA	88	160
ESC_SA6091AA	141	644
ESC_SA6090AA	141	644
ESC_SA6089AA	11400	512
ESC_SA6088AA	23	156
ESC_SA6087AA	88	160
ESC_SA6086AA	95	738
ESC_SA6082AA	1304	257
ESC_SA6081AA	2272	160
ESC_SA6080AA	48	76
ESC_SA6078AA	1618	736
ESC_SA6077AA	135	653
ESC_SA6074AA	101	696
ESC_SA6073AA	95	738
ESC_SA6072AA	93	71
ESC_SA6071AA	23	156
ESC_SA6070AA	345	175
ESC_SA6069AA	88	160

ESC_SA6068AA	95	738
ESC_SA6067AA	117	592
ESC_SA6066AA	345	175
ESC_SA6065AA	23	156
ESC_SA6064AA	93	71
ESC_SA6063AA	10	512
ESC_SA6061AA	428	654
ESC_SA6059AA	88	160
ESC_SA6058AA	101	696
ESC_SA6057AA	93	71
ESC_SA6055AA	88	160
ESC_SA6054AA	1638	519
ESC_SA6053AA	10	512
ESC_SA6052AA	10	512
ESC_SA6051AA	373	71
ESC_SA6050AA	10	512
ESC_SA6049AA	3721	93
ESC_SA6048AA	10	512
ESC_SA6047AA	373	71
ESC_SA6045AA	23	156
ESC_SA6044AA	131	653
ESC_SA6043AA	1618	736
ESC_SA6042AA	117	592
ESC_SA6040AA	95	738
ESC_SA6039AA	140	738
ESC_SA6038AA	429	654
ESC_SA6037AA	1638	519
ESC_SA6036AA	8573	867
ESC_SA6035AA	1618	736
ESC_SA6034AA	428	654
ESC_SA6033AA	162	924
ESC_SA6032AA	117	592
ESC_SA6031AA	349	670
ESC_SA6030AA	140	738
ESC_SA6029AA	117	592
ESC_SA6028AA	11399	263
ESC_SA6027AA	131	653
ESC_SA6026AA	117	592
ESC_SA6025AA	429	654
ESC_SA6024AA	10	512
ESC_SA6023AA	117	592
ESC_SA6022AA	429	654
ESC_SA6020AA	23	156
ESC_SA6019AA	95	738
ESC_SA6018AA	88	160
ESC_SA6017AA	117	592
ESC_SA6015AA	117	592
ESC_SA6014AA	420	741
ESC_SA6013AA	5926	50
ESC_SA6012AA	117	592

ESC_SA6010AA	95	738
ESC_SA6009AA	355	764
ESC_SA6008AA	1056	257
ESC_SA6007AA	117	592
ESC_SA6006AA	23	156
ESC_SA6005AA	23	156
ESC_SA6004AA	95	738
ESC_SA6002AA	4162	180
ESC_SA6001AA	162	924
ESC_SA6000AA	101	696
ESC_SA5999AA	429	654
ESC_SA5998AA	569	738
ESC_SA5997AA	101	696
ESC_SA5996AA	117	592
ESC_SA5995AA	101	696
ESC_SA5994AA	95	738
ESC_SA5993AA	101	696
ESC_SA5992AA	1638	519
ESC_SA5991AA	117	592
ESC_SA5990AA	117	592
ESC_SA5989AA	95	738
ESC_SA5987AA	648	359
ESC_SA5985AA	117	592
ESC_SA5984AA	101	696
ESC_SA5983AA	117	592
ESC_SA5982AA	1618	736
ESC_SA5981AA	95	738
ESC_SA5980AA	297	234
ESC_SA5979AA	10	512
ESC_SA5978AA	140	738
ESC_SA5977AA	1618	736
ESC_SA5976AA	101	696
ESC_SA5975AA	371	50
ESC_SA5974AA	428	654
ESC_SA5973AA	457	595
ESC_SA5972AA	11402	156
ESC_SA5971AA	95	738
ESC_SA5970AA	101	696
ESC_SA5969AA	1618	736
ESC_SA5968AA	371	50
ESC_SA5967AA	1618	736
ESC_SA5966AA	937	198
ESC_SA5963AA	88	160
ESC_SA5962AA	48	76
ESC_SA5961AA	88	160
ESC_SA5960AA	616	156
ESC_SA5959AA	140	738
ESC_SA5958AA	95	738
ESC_SA5957AA	117	592
ESC_SA5956AA	93	71

ESC_SA5955AA	140	738
ESC_SA5954AA	117	592
ESC_SA5953AA	11401	741
ESC_SA5952AA	117	592
ESC_SA5951AA	95	738
ESC_SA5950AA	95	738
ESC_SA5949AA	117	592
ESC_SA5948AA	117	592
ESC_SA5946AA	117	592
ESC_SA5945AA	140	738
ESC_SA5944AA	371	50
ESC_SA5943AA	3721	93
ESC_SA5942AA	117	592
ESC_SA5941AA	357	654
ESC_SA5940AA	23	156
ESC_SA5939AA	95	738
ESC_SA5937AA	140	738
ESC_SA5936AA	429	654
ESC_SA5935AA	10	512
ESC_SA5934AA	602	257
ESC_SA5933AA	141	644
ESC_SA5932AA	93	71
ESC_SA5931AA	140	738
ESC_SA5930AA	140	738
ESC_SA5927AA	156	175
ESC_SA5926AA	23	156
ESC_SA5922AA	23	156
ESC_SA5919AA	95	738
ESC_SA5918AA	95	738
ESC_SA5915AA	746	789
ESC_SA5914AA	95	738
ESC_SA5913AA	1638	519
ESC_SA5912AA	101	696
ESC_SA5907AA	1170	629
ESC_SA5906AA	117	592
ESC_SA5905AA	9616	272
ESC_SA5904AA	355	764
ESC_SA5903AA	2309	669
ESC_SA5902AA	10	512
ESC_SA5901AA	117	592
ESC_SA5900AA	373	71
ESC_SA5899AA	3995	198
ESC_SA5898AA	155	175
ESC_SA5897AA	919	421
ESC_SA5896AA	23	156
ESC_SA5895AA	23	156
ESC_SA5894AA	23	156
ESC_SA5893AA	140	738
ESC_SA5892AA	23	156
ESC_SA5891AA	155	175

ESC_SA5890AA	23	156
ESC_SA5889AA	23	156
ESC_SA5888AA	11414	1
ESC_SA5887AA	23	156
ESC_SA5886AA	11402	156
ESC_SA5885AA	140	738
ESC_SA5884AA	410	156
ESC_SA5883AA	117	592
ESC_SA5882AA	95	738
ESC_SA5881AA	23	156
ESC_SA5880AA	117	592
ESC_SA5879AA	428	654
ESC_SA5878AA	11413	784
ESC_SA5866AA	73	737
ESC_SA5864AA	131	653
ESC_SA5863AA	131	653
ESC_SA5862AA	95	738
ESC_SA5861AA	8137	198
ESC_SA5860AA	131	653
ESC_SA5859AA	117	592
ESC_SA5858AA	131	653
ESC_SA5857AA	73	737
ESC_SA5856AA	452	743
ESC_SA5855AA	69	387
ESC_SA5854AA	641	935
ESC_SA5853AA	162	924
ESC_SA5852AA	10	512
ESC_SA5851AA	10	512
ESC_SA5850AA	648	359
ESC_SA5849AA	73	737
ESC_SA5848AA	4702	626
ESC_SA5847AA	11409	761
ESC_SA5846AA	354	15
ESC_SA5845AA	424	234
ESC_SA5844AA	131	653
ESC_SA5843AA	131	653
ESC_SA5842AA	648	359
ESC_SA5841AA	648	359
ESC_SA5840AA	11408	627
ESC_SA5839AA	5584	640
ESC_SA5838AA	2179	924
ESC_SA5837AA	648	359
ESC_SA5836AA	1193	761
ESC_SA5835AA	1721	311
ESC_SA5834AA	354	15
ESC_SA5833AA	1193	761
ESC_SA5832AA	46	447
ESC_SA5831AA	453	935
ESC_SA5830AA	69	387
ESC_SA5829AA	10	512

ESC_SA5828AA	2003	669
ESC_SA5826AA	405	923
ESC_SA5825AA	11407	512
ESC_SA5824AA	648	359
ESC_SA5823AA	95	738
ESC_SA5822AA	1193	761
ESC_SA5821AA	11406	20
ESC_SA5820AA	648	359
ESC_SA5819AA	131	653
ESC_SA5818AA	410	156
ESC_SA5817AA	648	359
ESC_SA5816AA	11406	20
ESC_SA5814AA	69	387
ESC_SA5813AA	345	175
ESC_SA5812AA	1434	512
ESC_SA5811AA	73	737
ESC_SA5810AA	131	653
ESC_SA5809AA	648	359
ESC_SA5808AA	1485	408
ESC_SA5807AA	648	359
ESC_SA5806AA	648	359
ESC_SA5805AA	140	738
ESC_SA5804AA	1485	408
ESC_SA5803AA	1485	408
ESC_SA5802AA	93	71
ESC_SA5801AA	853	512
ESC_SA5800AA	354	15
ESC_SA5799AA	101	696
ESC_SA5798AA	117	592
ESC_SA5797AA	648	359
ESC_SA5796AA	141	644
ESC_SA5795AA	648	359
ESC_SA5794AA	1485	408
ESC_SA5793AA	648	359
ESC_SA5792AA	648	359
ESC_SA5791AA	1485	408
ESC_SA5790AA	1011	198
ESC_SA5789AA	648	359
ESC_SA5788AA	88	160
ESC_SA5787AA	648	359
ESC_SA5786AA	522	701
ESC_SA5785AA	88	160
ESC_SA5784AA	11405	512
ESC_SA5783AA	10	512
ESC_SA5782AA	69	387
ESC_SA5780AA	95	738
ESC_SA5779AA	1485	408
ESC_SA5778AA	1485	408
ESC_SA5777AA	131	653
ESC_SA5776AA	1485	408

ESC_SA5775AA	10	512
ESC_SA5774AA	117	592
ESC_SA5773AA	155	175
ESC_SA5772AA	744	512
ESC_SA5771AA	1485	408
ESC_SA5770AA	212	198
ESC_SA5769AA	131	653
ESC_SA5768AA	752	512
ESC_SA5767AA	131	653
ESC_SA5766AA	1485	408
ESC_SA5765AA	131	653
ESC_SA5764AA	1485	408
ESC_SA5763AA	1485	408
ESC_SA5762AA	428	654
ESC_SA5761AA	1485	408
ESC_SA5760AA	95	738
ESC_SA5739AA	48	76
ESC_SA5738AA	11404	286
ESC_SA5737AA	23	156
ESC_SA5736AA	2847	212
ESC_SA5735AA	10011	221
ESC_SA5734AA	69	387
ESC_SA5733AA	23	156
ESC_SA5732AA	11403	286
ESC_SA5730AA	117	592
ESC_SA5729AA	117	592
ESC_SA5728AA	10011	221
ESC_SA5727AA	10011	221
ESC_SA5726AA	117	592
ESC_SA5724AA	602	257
ESC_SA5722AA	10011	221
ESC_SA5721AA	1727	257
ESC_SA5717AA	5704	71
ESC_SA5716AA	1011	198
ESC_SA5711AA	117	592
ESC_SA5710AA	10011	221
ESC_SA5708AA	23	156
ESC_SA5706AA	69	387
ESC_SA5704AA	162	924
ESC_SA5703AA	10	512
ESC_SA5701AA	10011	221
ESC_SA5700AA	162	924
ESC_SA5699AA	101	696
ESC_SA5698AA	10011	221
ESC_SA5697AA	10011	221
ESC_SA5692AA	117	592
ESC_SA5691AA	350	50
ESC_SA5688AA	11412	286
ESC_SA5687AA	11412	286
ESC_SA5686AA	11412	286

ESC_SA5684AA	117	592
ESC_SA5683AA	117	592
ESC_SA5682AA	117	592
ESC_SA5679AA	117	592
ESC_SA5676AA	10011	221
ESC_SA5675AA	10011	221
ESC_SA5673AA	10011	221
ESC_SA5672AA	11412	286
ESC_SA5671AA	11412	286
ESC_SA5670AA	23	156
ESC_SA5666AA	10011	221
ESC_SA5648AA	1794	204
ESC_SA5647AA	306	286
ESC_SA5646AA	21	156
ESC_SA5645AA	677	257
ESC_SA5644AA	306	286
ESC_SA5643AA	11	936
ESC_SA5642AA	1705	156
ESC_SA5641AA	11	936
ESC_SA5640AA	21	156
ESC_SA5639AA	5266	198
ESC_SA5638AA	300	286
ESC_SA5637AA	327	204
ESC_SA5636AA	658	684
ESC_SA5635AA	21	156
ESC_SA5634AA	119	286
ESC_SA5633AA	119	286
ESC_SA5632AA	119	286
ESC_SA5631AA	300	286
ESC_SA5630AA	11	936
ESC_SA5629AA	688	284
ESC_SA5628AA	11	936
ESC_SA5627AA	660	286
ESC_SA5626AA	5472	191
ESC_SA5625AA	332	257
ESC_SA5624AA	1079	235
ESC_SA5623AA	1079	235
ESC_SA5621AA	58	198
ESC_SA5620AA	540	286
ESC_SA5619AA	457	595
ESC_SA5618AA	58	198
ESC_SA5617AA	34	504
ESC_SA5616AA	744	512
ESC_SA5615AA	744	512
ESC_SA5614AA	93	71
ESC_SA5613AA	155	175
ESC_SA5612AA	2253	71
ESC_SA5611AA	2705	504
ESC_SA5610AA	1589	156
ESC_SA5609AA	1589	156

ESC_SA5608AA	799	852
ESC_SA5607AA	1629	924
ESC_SA5606AA	685	311
ESC_SA5605AA	10	512
ESC_SA5604AA	773	71
ESC_SA5603AA	117	592
ESC_SA5602AA	7056	672
ESC_SA5601AA	5912	156
ESC_SA5600AA	7315	817
ESC_SA5599AA	10	512
ESC_SA5598AA	11411	447
ESC_SA5597AA	11424	286
ESC_SA5596AA	354	15
ESC_SA5595AA	93	71
ESC_SA5594AA	10	512
ESC_SA5593AA	11410	496
ESC_SA5592AA	10	512
ESC_SA5591AA	48	76
ESC_SA5590AA	196	257
ESC_SA5589AA	1716	512
ESC_SA5588AA	1716	512
ESC_SA5587AA	1716	512
ESC_SA5586AA	48	76
ESC_SA5585AA	4429	820
ESC_SA5584AA	4429	820
ESC_SA5583AA	10	512
ESC_SA5582AA	46	447
ESC_SA5581AA	101	696
ESC_SA5580AA	165	504
ESC_SA5579AA	1716	512
ESC_SA5578AA	2035	113
ESC_SA5577AA	2614	975
ESC_SA5576AA	101	696
ESC_SA5575AA	5951	406
ESC_SA5574AA	189	504
ESC_SA5573AA	10	512
ESC_SA5572AA	4429	820
ESC_SA5571AA	11423	175
ESC_SA5570AA	209	512
ESC_SA5569AA	167	512
ESC_SA5568AA	226	512
ESC_SA5567AA	744	512
ESC_SA5566AA	48	76
ESC_SA5565AA	1421	447
ESC_SA5564AA	4204	511
ESC_SA5563AA	744	512
ESC_SA5562AA	744	512
ESC_SA5561AA	224	198
ESC_SA5560AA	165	504
ESC_SA5559AA	4429	820

ESC_SA5558AA	9378	519
ESC_SA5557AA	354	15
ESC_SA5556AA	48	76
ESC_SA5555AA	2345	795
ESC_SA5554AA	3107	512
ESC_SA5553AA	871	447
ESC_SA5552AA	6438	65
ESC_SA5551AA	10	512
ESC_SA5550AA	6438	65
ESC_SA5549AA	871	447
ESC_SA5548AA	69	387
ESC_SA5547AA	206	286
ESC_SA5546AA	3941	71
ESC_SA5545AA	744	512
ESC_SA5544AA	10	512
ESC_SA5543AA	165	504
ESC_SA5542AA	1249	257
ESC_SA5541AA	2973	234
ESC_SA5540AA	2973	234
ESC_SA5539AA	46	447
ESC_SA5538AA	10	512
ESC_SA5537AA	46	447
ESC_SA5536AA	48	76
ESC_SA5535AA	542	824
ESC_SA5534AA	10	512
ESC_SA5533AA	10	512
ESC_SA5532AA	11422	286
ESC_SA5531AA	602	257
ESC_SA5530AA	170	475
ESC_SA5529AA	11421	601
ESC_SA5528AA	906	198
ESC_SA5527AA	11421	601
ESC_SA5526AA	23	156
ESC_SA5525AA	155	175
ESC_SA5524AA	93	71
ESC_SA5523AA	11420	512
ESC_SA5522AA	1716	512
ESC_SA5521AA	10	512
ESC_SA5520AA	1716	512
ESC_SA5519AA	1716	512
ESC_SA5518AA	4429	820
ESC_SA5517AA	10	512
ESC_SA5516AA	4429	820
ESC_SA5515AA	1716	512
ESC_SA5514AA	1716	512
ESC_SA5513AA	1716	512
ESC_SA5512AA	1716	512
ESC_SA5511AA	707	257
ESC_SA5510AA	11419	504
ESC_SA5509AA	101	696

ESC_SA5508AA	4429	820
ESC_SA5507AA	1716	512
ESC_SA5506AA	101	696
ESC_SA5505AA	1716	512
ESC_SA5504AA	117	592
ESC_SA5503AA	11419	504
ESC_SA5502AA	101	696
ESC_SA5501AA	165	504
ESC_SA5500AA	7140	789
ESC_SA5499AA	11418	311
ESC_SA5498AA	7450	512
ESC_SA5497AA	5748	924
ESC_SA5496AA	1716	512
ESC_SA5495AA	1716	512
ESC_SA5494AA	3274	512
ESC_SA5493AA	1716	512
ESC_SA5492AA	11417	696
ESC_SA5468AA	131	653
ESC_SA5467AA	10	512
ESC_SA5466AA	44	512
ESC_SA5465AA	131	653
ESC_SA5464AA	131	653
ESC_SA5463AA	131	653
ESC_SA5462AA	131	653
ESC_SA5461AA	450	71
ESC_SA5460AA	131	653
ESC_SA5459AA	131	653
ESC_SA5458AA	1193	761
ESC_SA5457AA	38	669
ESC_SA5456AA	1193	761
ESC_SA5455AA	131	653
ESC_SA5454AA	1193	761
ESC_SA5453AA	95	738
ESC_SA5452AA	48	76
ESC_SA5451AA	131	653
ESC_SA5450AA	410	156
ESC_SA5449AA	131	653
ESC_SA5448AA	131	653
ESC_SA5447AA	131	653
ESC_SA5446AA	1193	761
ESC_SA5445AA	131	653
ESC_SA5444AA	12	626
ESC_SA5443AA	131	653
ESC_SA5442AA	10	512
ESC_SA5441AA	131	653
ESC_SA5440AA	12	626
ESC_SA5439AA	131	653
ESC_SA5438AA	131	653
ESC_SA5437AA	131	653
ESC_SA5436AA	206	286

ESC_SA5435AA	206	286
ESC_SA5433AA	206	286
ESC_SA5432AA	206	286
ESC_SA5431AA	206	286
ESC_SA5430AA	206	286
ESC_SA5429AA	206	286
ESC_SA5428AA	206	286
ESC_SA5427AA	206	286
ESC_SA5426AA	206	286
ESC_SA5425AA	206	286
ESC_SA5424AA	206	286
ESC_SA5423AA	206	286
ESC_SA5422AA	206	286
ESC_SA5421AA	206	286
ESC_SA5420AA	206	286
ESC_SA5419AA	206	286
ESC_SA5418AA	206	286
ESC_SA5417AA	206	286
ESC_SA5416AA	206	286
ESC_SA5415AA	206	286
ESC_SA5414AA	206	286
ESC_SA5413AA	206	286
ESC_SA5412AA	206	286
ESC_SA5411AA	206	286
ESC_SA5410AA	206	286
ESC_SA5409AA	206	286
ESC_SA5408AA	206	286
ESC_SA5407AA	206	286
ESC_SA5406AA	206	286
ESC_SA5405AA	206	286
ESC_SA5404AA	206	286
ESC_SA5403AA	206	286
ESC_SA5401AA	10	512
ESC_SA5399AA	155	175
ESC_SA5398AA	226	512
ESC_SA5397AA	155	175
ESC_SA5396AA	155	175
ESC_SA5395AA	10842	286
ESC_SA5394AA	3580	257
ESC_SA5393AA	3580	257
ESC_SA5392AA	3580	257
ESC_SA5391AA	10	512
ESC_SA5390AA	10	512
ESC_SA5389AA	10	512
ESC_SA5388AA	3580	257
ESC_SA5387AA	10	512
ESC_SA5386AA	4450	844
ESC_SA5376AA	11	936
ESC_SA5375AA	11	936
ESC_SA5374AA	11	936

ESC_SA5373AA	11	936
ESC_SA5372AA	11	936
ESC_SA5371AA	11	936
ESC_SA5370AA	223	198
ESC_SA5221AA	10	512
ESC_SA5220AA	10	512
ESC_SA5218AA	10	512
ESC_SA5217AA	10	512
ESC_SA5216AA	10	512
ESC_SA5215AA	10	512
ESC_SA5214AA	10	512
ESC_SA5212AA	10	512
ESC_SA5210AA	10	512
ESC_SA5209AA	101	696
ESC_SA5208AA	101	696
ESC_SA5207AA	101	696
ESC_SA5206AA	101	696
ESC_SA5205AA	101	696
ESC_SA5204AA	101	696
ESC_SA5203AA	101	696
ESC_SA5195AA	88	160
ESC_SA5194AA	73	737
ESC_SA5193AA	131	653
ESC_SA5192AA	95	738
ESC_SA5191AA	491	626
ESC_SA5190AA	88	160
ESC_SA5189AA	73	737
ESC_SA5188AA	11427	935
ESC_SA5187AA	550	756
ESC_SA5186AA	607	355
ESC_SA5185AA	58	198
ESC_SA5184AA	10	512
ESC_SA5183AA	127	738
ESC_SA5182AA	127	738
ESC_SA5181AA	967	212
ESC_SA5180AA	2248	257
ESC_SA5179AA	73	737
ESC_SA5178AA	131	653
ESC_SA5177AA	131	653
ESC_SA5176AA	10	512
ESC_SA5175AA	38	669
ESC_SA5174AA	131	653
ESC_SA5173AA	349	670
ESC_SA5172AA	131	653
ESC_SA5171AA	550	756
ESC_SA5170AA	2173	431
ESC_SA5169AA	2020	387
ESC_SA5168AA	69	387
ESC_SA5167AA	69	387
ESC_SA5166AA	1480	160

ESC_SA5165AA	73	737
ESC_SA5164AA	69	387
ESC_SA5163AA	2622	537
ESC_SA5162AA	95	738
ESC_SA5161AA	95	738
ESC_SA5160AA	450	71
ESC_SA5159AA	69	387
ESC_SA5158AA	95	738
ESC_SA5157AA	131	653
ESC_SA5156AA	925	387
ESC_SA5155AA	69	387
ESC_SA5154AA	372	424
ESC_SA5153AA	69	387
ESC_SA5152AA	1808	744
ESC_SA5151AA	73	737
ESC_SA5150AA	88	160
ESC_SA5149AA	141	644
ESC_SA5148AA	69	387
ESC_SA5147AA	88	160
ESC_SA5146AA	540	286
ESC_SA5145AA	998	644
ESC_SA5144AA	14	761
ESC_SA5143AA	10	512
ESC_SA5142AA	117	592
ESC_SA5141AA	1136	108
ESC_SA5140AA	453	935
ESC_SA5139AA	131	653
ESC_SA5138AA	155	175
ESC_SA5137AA	88	160
ESC_SA5136AA	23	156
ESC_SA5135AA	162	924
ESC_SA5134AA	361	512
ESC_SA5133AA	131	653
ESC_SA5132AA	131	653
ESC_SA5131AA	357	654
ESC_SA5130AA	443	257
ESC_SA5129AA	73	737
ESC_SA5128AA	73	737
ESC_SA5127AA	1193	761
ESC_SA5126AA	950	50
ESC_SA5125AA	95	738
ESC_SA5124AA	654	257
ESC_SA5123AA	131	653
ESC_SA5122AA	73	737
ESC_SA5121AA	95	738
ESC_SA5120AA	14	761
ESC_SA5119AA	73	737
ESC_SA5118AA	73	737
ESC_SA5117AA	73	737
ESC_SA5116AA	404	761

ESC_SA5115AA	73	737
ESC_SA5114AA	73	737
ESC_SA5113AA	131	653
ESC_SA5112AA	46	447
ESC_SA5111AA	141	644
ESC_SA5110AA	131	653
ESC_SA5109AA	117	592
ESC_SA5108AA	131	653
ESC_SA5107AA	362	234
ESC_SA5106AA	131	653
ESC_SA5105AA	73	737
ESC_SA5104AA	635	141
ESC_SA5103AA	405	923
ESC_SA5102AA	131	653
ESC_SA5101AA	131	653
ESC_SA5100AA	131	653
ESC_SA5099AA	131	653
ESC_SA5098AA	69	387
ESC_SA5097AA	12	626
ESC_SA5096AA	93	71
ESC_SA5095AA	131	653
ESC_SA5094AA	69	387
ESC_SA5093AA	91	629
ESC_SA5092AA	73	737
ESC_SA5091AA	998	644
ESC_SA5090AA	69	387
ESC_SA5089AA	10	512
ESC_SA5088AA	73	737
ESC_SA5087AA	73	737
ESC_SA5086AA	12	626
ESC_SA5085AA	12	626
ESC_SA5084AA	73	737
ESC_SA5083AA	404	761
ESC_SA5082AA	95	738
ESC_SA5081AA	95	738
ESC_SA5080AA	73	737
ESC_SA5079AA	14	761
ESC_SA5078AA	550	756
ESC_SA5077AA	69	387
ESC_SA5076AA	11429	217
ESC_SA5075AA	12	626
ESC_SA5074AA	73	737
ESC_SA5073AA	73	737
ESC_SA5072AA	73	737
ESC_SA5071AA	11428	738
ESC_SA5070AA	144	740
ESC_SA5069AA	10	512
ESC_SA5068AA	95	738
ESC_SA5067AA	73	737
ESC_SA5066AA	393	343

ESC_SA5065AA	10	512
ESC_SA5064AA	405	923
ESC_SA5063AA	404	761
ESC_SA5062AA	127	738
ESC_SA5061AA	69	387
ESC_SA5060AA	73	737
ESC_SA5059AA	62	574
ESC_SA5058AA	131	653
ESC_SA5057AA	73	737
ESC_SA5056AA	131	653
ESC_SA5055AA	73	737
ESC_SA5054AA	144	740
ESC_SA5053AA	144	740
ESC_SA5052AA	144	740
ESC_SA5051AA	12	626
ESC_SA5050AA	14	761
ESC_SA5049AA	95	738
ESC_SA5048AA	95	738
ESC_SA5047AA	127	738
ESC_SA5046AA	95	738
ESC_SA5045AA	405	923
ESC_SA5044AA	127	738
ESC_SA5043AA	1303	791
ESC_SA5042AA	1896	380
ESC_SA5041AA	131	653
ESC_SA5040AA	95	738
ESC_SA5039AA	131	653
ESC_SA5038AA	1308	257
ESC_SA5037AA	73	737
ESC_SA5036AA	117	592
ESC_SA5035AA	2590	529
ESC_SA5034AA	1670	438
ESC_SA5033AA	131	653
ESC_SA5032AA	131	653
ESC_SA5031AA	405	923
ESC_SA5030AA	297	234
ESC_SA5029AA	453	935
ESC_SA5028AA	349	670
ESC_SA5027AA	131	653
ESC_SA5026AA	131	653
ESC_SA5025AA	93	71
ESC_SA5024AA	93	71
ESC_SA5023AA	93	71
ESC_SA5022AA	93	71
ESC_SA5021AA	93	71
ESC_SA5020AA	93	71
ESC_SA5019AA	93	71
ESC_SA5018AA	93	71
ESC_SA5017AA	93	71
ESC_SA5016AA	93	71

ESC_SA5015AA	93	71
ESC_SA5014AA	93	71
ESC_SA5013AA	93	71
ESC_SA5012AA	93	71
ESC_SA5011AA	93	71
ESC_SA5010AA	93	71
ESC_SA5009AA	93	71
ESC_SA5008AA	93	71
ESC_SA5007AA	93	71
ESC_SA5006AA	93	71
ESC_SA5005AA	93	71
ESC_SA5004AA	93	71
ESC_SA5003AA	93	71
ESC_SA5002AA	93	71
ESC_SA5001AA	93	71
ESC_SA5000AA	93	71
ESC_SA4999AA	93	71
ESC_SA4998AA	93	71
ESC_SA4997AA	93	71
ESC_SA4996AA	93	71
ESC_SA4995AA	93	71
ESC_SA4994AA	93	71
ESC_SA4993AA	93	71
ESC_SA4992AA	93	71
ESC_SA4991AA	93	71
ESC_SA4990AA	205	257
ESC_SA4989AA	131	653
ESC_SA4988AA	224	198
ESC_SA4987AA	101	696
ESC_SA4986AA	683	175
ESC_SA4985AA	683	175
ESC_SA4984AA	10	512
ESC_SA4983AA	117	592
ESC_SA4982AA	3258	592
ESC_SA4981AA	3258	592
ESC_SA4980AA	117	592
ESC_SA4979AA	115	669
ESC_SA4978AA	117	592
ESC_SA4977AA	58	198
ESC_SA4976AA	58	198
ESC_SA4975AA	10	512
ESC_SA4974AA	10	512
ESC_SA4973AA	10	512
ESC_SA4972AA	227	504
ESC_SA4971AA	4981	473
ESC_SA4970AA	206	286
ESC_SA4964AA	38	669
ESC_SA4963AA	38	669
ESC_SA4962AA	38	669
ESC_SA4961AA	38	669

ESC_SA4960AA	38	669
ESC_SA4959AA	38	669
ESC_SA4958AA	38	669
ESC_SA4957AA	38	669
ESC_SA4956AA	38	669
ESC_SA4955AA	38	669
ESC_SA4954AA	38	669
ESC_SA4953AA	38	669
ESC_SA4952AA	38	669
ESC_SA4951AA	38	669
ESC_SA4950AA	38	669
ESC_SA4949AA	38	669
ESC_SA4948AA	38	669
ESC_SA4912AA	212	198
ESC_SA4911AA	167	512
ESC_SA4910AA	58	198
ESC_SA4898AA	11391	153
ESC_SA4897AA	11391	153
ESC_SA4896AA	21	156
ESC_SA4895AA	21	156
ESC_SA4894AA	21	156
ESC_SA4893AA	21	156
ESC_SA4892AA	21	156
ESC_SA4891AA	21	156
ESC_SA4890AA	11391	153
ESC_SA4889AA	11391	153
ESC_SA4888AA	21	156
ESC_SA4887AA	21	156
ESC_SA4886AA	21	156
ESC_SA4885AA	21	156
ESC_SA4884AA	21	156
ESC_SA4883AA	21	156
ESC_SA4882AA	131	653
ESC_SA4881AA	131	653
ESC_SA4880AA	224	198
ESC_SA4879AA	405	923
ESC_SA4878AA	410	156
ESC_SA4877AA	167	512
ESC_SA4876AA	131	653
ESC_SA4875AA	131	653
ESC_SA4874AA	744	512
ESC_SA4873AA	744	512
ESC_SA4872AA	10	512
ESC_SA4871AA	10	512
ESC_SA4870AA	8954	198
ESC_SA4869AA	448	257
ESC_SA4868AA	131	653
ESC_SA4867AA	131	653
ESC_SA4866AA	453	935
ESC_SA4865AA	7468	935

ESC_SA4864AA	69	387
ESC_SA4862AA	69	387
ESC_SA4861AA	46	447
ESC_SA4860AA	46	447
ESC_SA4859AA	131	653
ESC_SA4858AA	617	511
ESC_SA4857AA	167	512
ESC_SA4856AA	361	512
ESC_SA4855AA	224	198
ESC_SA4854AA	453	935
ESC_SA4853AA	1284	604
ESC_SA4852AA	354	15
ESC_SA4851AA	48	76
ESC_SA4850AA	11394	1030
ESC_SA4849AA	448	257
ESC_SA4848AA	10	512
ESC_SA4847AA	393	343
ESC_SA4846AA	224	198
ESC_SA4845AA	58	198
ESC_SA4844AA	68	386
ESC_SA4843AA	410	156
ESC_SA4842AA	90	156
ESC_SA4841AA	167	512
ESC_SA4840AA	410	156
ESC_SA4839AA	88	160
ESC_SA4838AA	500	71
ESC_SA4837AA	80	731
ESC_SA4836AA	2671	257
ESC_SA4835AA	744	512
ESC_SA4834AA	2671	257
ESC_SA4833AA	68	386
ESC_SA4832AA	10	512
ESC_SA4831AA	1480	160
ESC_SA4830AA	6332	153
ESC_SA4829AA	10	512
ESC_SA4828AA	617	511
ESC_SA4827AA	345	175
ESC_SA4826AA	641	935
ESC_SA4825AA	410	156
ESC_SA4824AA	10	512
ESC_SA4823AA	361	512
ESC_SA4822AA	410	156
ESC_SA4821AA	131	653
ESC_SA4820AA	11393	359
ESC_SA4819AA	131	653
ESC_SA4818AA	405	923
ESC_SA4817AA	131	653
ESC_SA4816AA	405	923
ESC_SA4815AA	443	257
ESC_SA4814AA	88	160

ESC_SA4813AA	131	653
ESC_SA4812AA	44	512
ESC_SA4811AA	88	160
ESC_SA4810AA	44	512
ESC_SA4809AA	23	156
ESC_SA4808AA	648	359
ESC_SA4807AA	7470	50
ESC_SA4806AA	1480	160
ESC_SA4804AA	2141	597
ESC_SA4803AA	162	924
ESC_SA4802AA	10	512
ESC_SA4801AA	131	653
ESC_SA4799AA	131	653
ESC_SA4797AA	131	653
ESC_SA4796AA	131	653
ESC_SA4795AA	10	512
ESC_SA4794AA	131	653
ESC_SA4793AA	2035	113
ESC_SA4792AA	533	198
ESC_SA4791AA	448	257
ESC_SA4790AA	224	198
ESC_SA4789AA	6332	153
ESC_SA4788AA	635	141
ESC_SA4787AA	1291	512
ESC_SA4786AA	1291	512
ESC_SA4785AA	372	424
ESC_SA4784AA	10	512
ESC_SA4783AA	131	653
ESC_SA4782AA	410	156
ESC_SA4779AA	405	923
ESC_SA4778AA	131	653
ESC_SA4777AA	3024	696
ESC_SA4776AA	131	653
ESC_SA4775AA	1177	669
ESC_SA4774AA	410	156
ESC_SA4773AA	602	257
ESC_SA4772AA	131	653
ESC_SA4771AA	10	512
ESC_SA4770AA	10	512
ESC_SA4769AA	131	653
ESC_SA4767AA	56	198
ESC_SA4765AA	46	447
ESC_SA4764AA	69	387
ESC_SA4763AA	131	653
ESC_SA4762AA	46	447
ESC_SA4761AA	131	653
ESC_SA4760AA	7469	180
ESC_SA4758AA	10	512
ESC_SA4757AA	90	156
ESC_SA4756AA	224	198

ESC_SA4755AA	155	175
ESC_SA4754AA	744	512
ESC_SA4753AA	361	512
ESC_SA4752AA	88	160
ESC_SA4751AA	10	512
ESC_SA4750AA	131	653
ESC_SA4749AA	683	175
ESC_SA4748AA	648	359
ESC_SA4747AA	162	924
ESC_SA4746AA	131	653
ESC_SA4745AA	155	175
ESC_SA4744AA	224	198
ESC_SA4743AA	131	653
ESC_SA4742AA	131	653
ESC_SA4741AA	131	653
ESC_SA4740AA	3564	160
ESC_SA4739AA	131	653
ESC_SA4738AA	648	359
ESC_SA4737AA	131	653
ESC_SA4736AA	162	924
ESC_SA4735AA	167	512
ESC_SA4734AA	1284	604
ESC_SA4733AA	131	653
ESC_SA4732AA	10	512
ESC_SA4731AA	131	653
ESC_SA4730AA	59	217
ESC_SA4729AA	59	217
ESC_SA4728AA	224	198
ESC_SA4727AA	131	653
ESC_SA4726AA	131	653
ESC_SA4725AA	361	512
ESC_SA4724AA	10	512
ESC_SA4722AA	10	512
ESC_SA4721AA	648	359
ESC_SA4720AA	167	512
ESC_SA4719AA	131	653
ESC_SA4718AA	131	653
ESC_SA4717AA	131	653
ESC_SA4716AA	227	504
ESC_SA4715AA	44	512
ESC_SA4714AA	131	653
ESC_SA4713AA	1284	604
ESC_SA4712AA	131	653
ESC_SA4711AA	167	512
ESC_SA4710AA	23	156
ESC_SA4709AA	88	160
ESC_SA4708AA	405	923
ESC_SA4707AA	131	653
ESC_SA4706AA	38	669
ESC_SA4705AA	131	653

ESC_SA4704AA	88	160
ESC_SA4703AA	131	653
ESC_SA4702AA	131	653
ESC_SA4701AA	10	512
ESC_SA4700AA	56	198
ESC_SA4699AA	9028	696
ESC_SA4697AA	131	653
ESC_SA4696AA	354	15
ESC_SA4695AA	131	653
ESC_SA4694AA	58	198
ESC_SA4693AA	131	653
ESC_SA4692AA	131	653
ESC_SA4691AA	131	653
ESC_SA4690AA	23	156
ESC_SA4689AA	2003	669
ESC_SA4688AA	381	156
ESC_SA4687AA	7358	923
ESC_SA4686AA	131	653
ESC_SA4685AA	131	653
ESC_SA4684AA	58	198
ESC_SA4681AA	744	512
ESC_SA4680AA	224	198
ESC_SA4679AA	131	653
ESC_SA4678AA	58	198
ESC_SA4677AA	88	160
ESC_SA4676AA	10	512
ESC_SA4675AA	131	653
ESC_SA4674AA	648	359
ESC_SA4672AA	73	737
ESC_SA4671AA	2496	789
ESC_SA4670AA	131	653
ESC_SA4669AA	11395	511
ESC_SA4668AA	357	654
ESC_SA4667AA	10	512
ESC_SA4666AA	2025	194
ESC_SA4665AA	10	512
ESC_SA4664AA	9030	175
ESC_SA4663AA	617	511
ESC_SA4662AA	131	653
ESC_SA4661AA	131	653
ESC_SA4660AA	3873	175
ESC_SA4659AA	131	653
ESC_SA4658AA	1421	447
ESC_SA4657AA	58	198
ESC_SA4656AA	224	198
ESC_SA4655AA	227	504
ESC_SA4654AA	88	160
ESC_SA4652AA	58	198
ESC_SA4651AA	7470	50
ESC_SA4650AA	131	653

ESC_SA4648AA	135	653
ESC_SA4647AA	354	15
ESC_SA4646AA	58	198
ESC_SA4645AA	135	653
ESC_SA4644AA	69	387
ESC_SA4643AA	354	15
ESC_SA4642AA	410	156
ESC_SA4641AA	141	644
ESC_SA4640AA	73	737
ESC_SA4639AA	648	359
ESC_SA4638AA	131	653
ESC_SA4637AA	73	737
ESC_SA4636AA	48	76
ESC_SA4635AA	58	198
ESC_SA4634AA	2509	257
ESC_SA4633AA	69	387
ESC_SA4632AA	162	924
ESC_SA4631AA	48	76
ESC_SA4630AA	648	359
ESC_SA4629AA	48	76
ESC_SA4628AA	9029	156
ESC_SA4627AA	3873	175
ESC_SA4626AA	410	156
ESC_SA4625AA	448	257
ESC_SA4624AA	744	512
ESC_SA4623AA	10	512
ESC_SA4622AA	453	935
ESC_SA4621AA	131	653
ESC_SA4620AA	167	512
ESC_SA4619AA	1056	257
ESC_SA4618AA	362	234
ESC_SA4617AA	56	198
ESC_SA4615AA	361	512
ESC_SA4614AA	641	935
ESC_SA4613AA	131	653
ESC_SA4612AA	93	71
ESC_SA4611AA	453	935
ESC_SA4610AA	131	653
ESC_SA4609AA	44	512
ESC_SA4608AA	224	198
ESC_SA4606AA	11430	928
ESC_SA4605AA	1851	380
ESC_SA4604AA	744	512
ESC_SA4603AA	542	824
ESC_SA4602AA	10	512
ESC_SA4601AA	617	511
ESC_SA4600AA	617	511
ESC_SA4599AA	4119	537
ESC_SA4598AA	617	511
ESC_SA4597AA	38	669

ESC_SA4596AA	617	511
ESC_SA4595AA	617	511
ESC_SA4594AA	131	653
ESC_SA4593AA	453	935
ESC_SA4592AA	877	935
ESC_SA4591AA	69	387
ESC_SA4590AA	131	653
ESC_SA4589AA	533	198
ESC_SA4588AA	131	653
ESC_SA4587AA	131	653
ESC_SA4586AA	746	789
ESC_SA4585AA	744	512
ESC_SA4584AA	131	653
ESC_SA4583AA	131	653
ESC_SA4582AA	93	71
ESC_SA4581AA	6241	900
ESC_SA4580AA	117	592
ESC_SA4579AA	1434	512
ESC_SA4578AA	410	156
ESC_SA4577AA	410	156
ESC_SA4575AA	648	359
ESC_SA4574AA	410	156
ESC_SA4573AA	410	156
ESC_SA4572AA	131	653
ESC_SA4571AA	38	669
ESC_SA4570AA	1723	900
ESC_SA4569AA	1723	900
ESC_SA4568AA	131	653
ESC_SA4567AA	131	653
ESC_SA4566AA	10	512
ESC_SA4565AA	744	512
ESC_SA4564AA	10	512
ESC_SA4563AA	224	198
ESC_SA4562AA	90	156
ESC_SA4561AA	88	160
ESC_SA4560AA	69	387
ESC_SA4559AA	410	156
ESC_SA4557AA	410	156
ESC_SA4556AA	410	156
ESC_SA4555AA	410	156
ESC_SA4554AA	410	156
ESC_SA4553AA	410	156
ESC_SA4552AA	167	512
ESC_SA4551AA	410	156
ESC_SA4550AA	410	156
ESC_SA4549AA	131	653
ESC_SA4548AA	410	156
ESC_SA4547AA	131	653
ESC_SA4546AA	410	156
ESC_SA4545AA	410	156

ESC_SA4544AA	69	387
ESC_SA4543AA	58	198
ESC_SA4542AA	69	387
ESC_SA4541AA	648	359
ESC_SA4540AA	69	387
ESC_SA4539AA	542	824
ESC_SA4538AA	871	447
ESC_SA4537AA	871	447
ESC_SA4536AA	131	653
ESC_SA4535AA	131	653
ESC_SA4534AA	393	343
ESC_SA4533AA	393	343
ESC_SA4532AA	648	359
ESC_SA4531AA	131	653
ESC_SA4530AA	361	512
ESC_SA4529AA	453	935
ESC_SA4528AA	117	592
ESC_SA4527AA	44	512
ESC_SA4525AA	131	653
ESC_SA4524AA	410	156
ESC_SA4523AA	410	156
ESC_SA4522AA	410	156
ESC_SA4521AA	410	156
ESC_SA4520AA	410	156
ESC_SA4519AA	1177	669
ESC_SA4518AA	410	156
ESC_SA4517AA	410	156
ESC_SA4516AA	410	156
ESC_SA4515AA	410	156
ESC_SA4514AA	88	160
ESC_SA4513AA	405	923
ESC_SA4512AA	410	156
ESC_SA4511AA	73	737
ESC_SA4510AA	10	512
ESC_SA4509AA	131	653
ESC_SA4508AA	131	653
ESC_SA4507AA	131	653
ESC_SA4506AA	101	696
ESC_SA4505AA	641	935
ESC_SA4504AA	131	653
ESC_SA4503AA	349	670
ESC_SA4502AA	10	512
ESC_SA4501AA	7036	445
ESC_SA4500AA	156	175
ESC_SA4499AA	491	626
ESC_SA4498AA	410	156
ESC_SA4497AA	10	512
ESC_SA4496AA	354	15
ESC_SA4495AA	10	512
ESC_SA4494AA	6589	848

ESC_SA4493AA	11433	512
ESC_SA4492AA	10	512
ESC_SA4491AA	10	512
ESC_SA4490AA	73	737
ESC_SA4489AA	11432	512
ESC_SA4411AA	301	307
ESC_SA4410AA	58	198
ESC_SA4409AA	11431	198
ESC_SA4408AA	56	198
ESC_SA4407AA	5727	257
ESC_SA4406AA	5727	257
ESC_SA4405AA	10	512
ESC_SA4404AA	2325	447
ESC_SA4403AA	398	445
ESC_SA4402AA	683	175
ESC_SA4401AA	515	1004
ESC_SA4400AA	2325	447
ESC_SA4399AA	5727	257
ESC_SA4398AA	10	512
ESC_SA4397AA	69	387
ESC_SA4396AA	10	512
ESC_SA4395AA	7588	447
ESC_SA4394AA	7588	447
ESC_SA4393AA	301	307
ESC_SA4392AA	46	447
ESC_SA4391AA	68	386
ESC_SA4390AA	68	386
ESC_SA4389AA	453	935
ESC_SA4388AA	453	935
ESC_SA4387AA	10	512
ESC_SA4386AA	10	512
ESC_SA4385AA	355	764
ESC_SA4384AA	68	386
ESC_SA4383AA	399	198
ESC_SA4382AA	156	175
ESC_SA4381AA	398	445
ESC_SA4380AA	744	512
ESC_SA4379AA	744	512
ESC_SA4378AA	23	156
ESC_SA4377AA	137	270
ESC_SA4376AA	453	935
ESC_SA4375AA	212	198
ESC_SA4374AA	2600	445
ESC_SA4373AA	7474	198
ESC_SA4372AA	99	198
ESC_SA4371AA	99	198
ESC_SA4370AA	607	355
ESC_SA4369AA	10	512
ESC_SA4368AA	10	512
ESC_SA4367AA	10	512

ESC_SA4366AA	10	512
ESC_SA4365AA	10	512
ESC_SA4363AA	2522	198
ESC_SA4362AA	10	512
ESC_SA4361AA	10	512
ESC_SA4360AA	10	512
ESC_SA4359AA	10	512
ESC_SA4358AA	6083	323
ESC_SA4357AA	354	15
ESC_SA4356AA	354	15
ESC_SA4355AA	44	512
ESC_SA4354AA	137	270
ESC_SA4353AA	354	15
ESC_SA4352AA	23	156
ESC_SA4351AA	1196	257
ESC_SA4350AA	88	160
ESC_SA4349AA	1722	599
ESC_SA4348AA	549	387
ESC_SA4347AA	1406	174
ESC_SA4346AA	453	935
ESC_SA4345AA	549	387
ESC_SA4344AA	131	653
ESC_SA4343AA	155	175
ESC_SA4342AA	3024	696
ESC_SA4341AA	10	512
ESC_SA4340AA	453	935
ESC_SA4339AA	393	343
ESC_SA4338AA	131	653
ESC_SA4337AA	131	653
ESC_SA4336AA	1674	595
ESC_SA4335AA	1011	198
ESC_SA4334AA	46	447
ESC_SA4333AA	388	156
ESC_SA4332AA	388	156
ESC_SA4331AA	457	595
ESC_SA4330AA	23	156
ESC_SA4329AA	46	447
ESC_SA4328AA	453	935
ESC_SA4327AA	99	198
ESC_SA4326AA	2600	445
ESC_SA4325AA	131	653
ESC_SA4324AA	131	653
ESC_SA4323AA	131	653
ESC_SA4322AA	2639	653
ESC_SA4321AA	131	653
ESC_SA4320AA	131	653
ESC_SA4319AA	131	653
ESC_SA4318AA	131	653
ESC_SA4317AA	131	653
ESC_SA4316AA	131	653

ESC_SA4315AA	131	653
ESC_SA4314AA	131	653
ESC_SA4313AA	131	653
ESC_SA4312AA	131	653
ESC_SA4311AA	131	653
ESC_SA4310AA	131	653
ESC_SA4309AA	131	653
ESC_SA4308AA	131	653
ESC_SA4307AA	131	653
ESC_SA4306AA	131	653
ESC_SA4305AA	58	198
ESC_SA4304AA	372	424
ESC_SA4303AA	1725	175
ESC_SA4302AA	11081	1004
ESC_SA4301AA	306	286
ESC_SA4300AA	306	286
ESC_SA4299AA	17	191
ESC_SA4298AA	11460	286
ESC_SA4297AA	8828	669
ESC_SA4296AA	2536	261
ESC_SA4295AA	58	198
ESC_SA4293AA	405	923
ESC_SA4292AA	354	15
ESC_SA4291AA	69	387
ESC_SA4290AA	583	768
ESC_SA4289AA	11459	151
ESC_SA4288AA	655	926
ESC_SA4287AA	738	598
ESC_SA4286AA	300	286
ESC_SA4285AA	48	76
ESC_SA4284AA	278	924
ESC_SA4283AA	6	445
ESC_SA4281AA	20	198
ESC_SA4280AA	678	257
ESC_SA4279AA	3249	156
ESC_SA4278AA	101	696
ESC_SA4277AA	101	696
ESC_SA4276AA	657	592
ESC_SA4275AA	21	156
ESC_SA4274AA	21	156
ESC_SA4273AA	6130	268
ESC_SA4272AA	17	191
ESC_SA4271AA	11	936
ESC_SA4270AA	16	156
ESC_SA4269AA	21	156
ESC_SA4268AA	5560	929
ESC_SA4267AA	5725	156
ESC_SA4266AA	11	936
ESC_SA4265AA	386	191
ESC_SA4264AA	21	156

ESC_SA4263AA	32	270
ESC_SA4262AA	16	156
ESC_SA4261AA	17	191
ESC_SA4260AA	11	936
ESC_SA4259AA	29	156
ESC_SA4257AA	11	936
ESC_SA4256AA	21	156
ESC_SA4255AA	32	270
ESC_SA4254AA	21	156
ESC_SA4253AA	445	156
ESC_SA4252AA	11	936
ESC_SA4249AA	11	936
ESC_SA4248AA	11	936
ESC_SA4246AA	11	936
ESC_SA4244AA	11	936
ESC_SA4243AA	11	936
ESC_SA4242AA	11	936
ESC_SA4241AA	11	936
ESC_SA4239AA	11	936
ESC_SA4238AA	11	936
ESC_SA4237AA	11	936
ESC_SA4236AA	11	936
ESC_SA4235AA	11	936
ESC_SA4234AA	11	936
ESC_SA4233AA	11	936
ESC_SA4232AA	11	936
ESC_SA4231AA	11	936
ESC_SA4230AA	11	936
ESC_SA4229AA	11	936
ESC_SA4228AA	11	936
ESC_SA4227AA	11	936
ESC_SA4226AA	11	936
ESC_SA4225AA	11	936
ESC_SA4224AA	11	936
ESC_SA4223AA	11	936
ESC_SA4222AA	11	936
ESC_SA4221AA	11	936
ESC_SA4220AA	11	936
ESC_SA4219AA	11	936
ESC_SA4218AA	11	936
ESC_SA4217AA	11	936
ESC_SA4216AA	11	936
ESC_SA4215AA	11	936
ESC_SA4214AA	11	936
ESC_SA4212AA	11	936
ESC_SA4211AA	11	936
ESC_SA4210AA	11	936
ESC_SA4209AA	11	936
ESC_SA4208AA	11	936
ESC_SA4207AA	11	936

ESC_SA4206AA	11	936
ESC_SA4205AA	11	936
ESC_SA4204AA	11	936
ESC_SA4203AA	11	936
ESC_SA4201AA	11	936
ESC_SA4199AA	21	156
ESC_SA4196AA	17	191
ESC_SA4193AA	16	156
ESC_SA4192AA	11	936
ESC_SA4190AA	11	936
ESC_SA4189AA	11	936
ESC_SA4188AA	11	936
ESC_SA4187AA	11	936
ESC_SA4186AA	11	936
ESC_SA4185AA	11	936
ESC_SA4184AA	11	936
ESC_SA4183AA	11	936
ESC_SA4182AA	11	936
ESC_SA4181AA	11	936
ESC_SA4178AA	69	387
ESC_SA4177AA	11	936
ESC_SA4176AA	11	936
ESC_SA4174AA	11	936
ESC_SA4173AA	11	936
ESC_SA4172AA	11	936
ESC_SA4171AA	11	936
ESC_SA4170AA	11	936
ESC_SA4169AA	11	936
ESC_SA4168AA	11	936
ESC_SA4167AA	11	936
ESC_SA4166AA	11	936
ESC_SA4165AA	11	936
ESC_SA4163AA	156	175
ESC_SA4162AA	447	198
ESC_SA4161AA	10	512
ESC_SA4160AA	11461	234
ESC_SA4159AA	33	198
ESC_SA4158AA	300	286
ESC_SA4157AA	767	198
ESC_SA4156AA	342	286
ESC_SA4155AA	583	768
ESC_SA4154AA	442	257
ESC_SA4151AA	1967	191
ESC_SA4150AA	1967	191
ESC_SA4149AA	21	156
ESC_SA4148AA	21	156
ESC_SA4147AA	21	156
ESC_SA4146AA	21	156
ESC_SA4145AA	1967	191
ESC_SA4144AA	11	936

ESC_SA4143AA	5937	257
ESC_SA4142AA	718	257
ESC_SA4141AA	718	257
ESC_SA4139AA	1967	191
ESC_SA4138AA	718	257
ESC_SA4137AA	718	257
ESC_SA4136AA	718	257
ESC_SA4135AA	718	257
ESC_SA4134AA	718	257
ESC_SA4133AA	2387	261
ESC_SA4132AA	162	924
ESC_SA4131AA	5559	257
ESC_SA4130AA	5559	257
ESC_SA4129AA	5559	257
ESC_SA4128AA	1967	191
ESC_SA4127AA	5559	257
ESC_SA4126AA	5559	257
ESC_SA4124AA	5559	257
ESC_SA4123AA	5559	257
ESC_SA4122AA	5559	257
ESC_SA4121AA	5559	257
ESC_SA4120AA	5559	257
ESC_SA4119AA	5559	257
ESC_SA4118AA	5559	257
ESC_SA4117AA	21	156
ESC_SA4116AA	5559	257
ESC_SA4115AA	5559	257
ESC_SA4114AA	5559	257
ESC_SA4113AA	5559	257
ESC_SA4112AA	5559	257
ESC_SA4111AA	5559	257
ESC_SA4110AA	5559	257
ESC_SA4109AA	5559	257
ESC_SA4108AA	5559	257
ESC_SA4107AA	5559	257
ESC_SA4106AA	21	156
ESC_SA4105AA	5559	257
ESC_SA4104AA	5559	257
ESC_SA4103AA	5559	257
ESC_SA4102AA	5559	257
ESC_SA4101AA	5559	257
ESC_SA4100AA	5559	257
ESC_SA4099AA	5559	257
ESC_SA4098AA	5559	257
ESC_SA4097AA	5559	257
ESC_SA4096AA	5559	257
ESC_SA4095AA	21	156
ESC_SA4094AA	5559	257
ESC_SA4093AA	5559	257
ESC_SA4092AA	5559	257

ESC_SA4091AA	21	156
ESC_SA4090AA	21	156
ESC_SA4089AA	21	156
ESC_SA4088AA	361	512
ESC_SA4087AA	167	512
ESC_SA4086AA	617	511
ESC_SA4085AA	218	519
ESC_SA4084AA	73	737
ESC_SA4083AA	2279	657
ESC_SA4082AA	73	737
ESC_SA4081AA	394	400
ESC_SA4080AA	216	512
ESC_SA4079AA	131	653
ESC_SA4078AA	1193	761
ESC_SA4077AA	2178	924
ESC_SA4076AA	491	626
ESC_SA4075AA	491	626
ESC_SA4074AA	75	257
ESC_SA4072AA	182	246
ESC_SA4071AA	182	246
ESC_SA4070AA	182	246
ESC_SA4069AA	182	246
ESC_SA4068AA	182	246
ESC_SA4067AA	182	246
ESC_SA4066AA	182	246
ESC_SA4065AA	182	246
ESC_SA4064AA	182	246
ESC_SA4063AA	182	246
ESC_SA4062AA	182	246
ESC_SA4061AA	182	246
ESC_SA4060AA	182	246
ESC_SA4059AA	182	246
ESC_SA4058AA	182	246
ESC_SA4057AA	182	246
ESC_SA4056AA	182	246
ESC_SA4055AA	182	246
ESC_SA4054AA	182	246
ESC_SA4053AA	182	246
ESC_SA4052AA	182	246
ESC_SA4051AA	182	246
ESC_SA4050AA	182	246
ESC_SA4049AA	182	246
ESC_SA4048AA	182	246
ESC_SA4047AA	182	246
ESC_SA4046AA	182	246
ESC_SA4045AA	182	246
ESC_SA4044AA	182	246
ESC_SA4043AA	182	246
ESC_SA4042AA	182	246
ESC_SA4041AA	182	246

ESC_SA4040AA	182	246
ESC_SA4039AA	182	246
ESC_SA4038AA	182	246
ESC_SA4037AA	182	246
ESC_SA4036AA	182	246
ESC_SA4035AA	182	246
ESC_SA4034AA	182	246
ESC_SA4033AA	182	246
ESC_SA4032AA	182	246
ESC_SA4031AA	182	246
ESC_SA4030AA	182	246
ESC_SA4029AA	182	246
ESC_SA4028AA	182	246
ESC_SA4027AA	182	246
ESC_SA4018AA	10	512
ESC_SA4017AA	10	512
ESC_SA4016AA	10	512
ESC_SA4015AA	11466	512
ESC_SA4014AA	11467	512
ESC_SA4013AA	10	512
ESC_SA4011AA	10	512
ESC_SA4010AA	10	512
ESC_SA4009AA	10	512
ESC_SA4007AA	10	512
ESC_SA4006AA	10	512
ESC_SA4005AA	6495	336
ESC_SA4004AA	599	626
ESC_SA4003AA	131	653
ESC_SA4002AA	372	424
ESC_SA4001AA	648	359
ESC_SA4000AA	90	156
ESC_SA3999AA	131	653
ESC_SA3998AA	69	387
ESC_SA3997AA	648	359
ESC_SA3996AA	10	512
ESC_SA3995AA	95	738
ESC_SA3994AA	131	653
ESC_SA3993AA	38	669
ESC_SA3992AA	648	359
ESC_SA3991AA	131	653
ESC_SA3990AA	405	923
ESC_SA3989AA	131	653
ESC_SA3988AA	10	512
ESC_SA3987AA	6217	504
ESC_SA3986AA	10	512
ESC_SA3985AA	665	79
ESC_SA3984AA	3863	257
ESC_SA3983AA	453	935
ESC_SA3982AA	131	653
ESC_SA3981AA	131	653

ESC_SA3980AA	10	512
ESC_SA3979AA	10	512
ESC_SA3975AA	10	512
ESC_SA3974AA	10	512
ESC_SA3972AA	10	512
ESC_SA3971AA	10	512
ESC_SA3969AA	10	512
ESC_SA3968AA	10	512
ESC_SA3967AA	10	512
ESC_SA3966AA	10	512
ESC_SA3965AA	10	512
ESC_SA3963AA	10	512
ESC_SA3962AA	10	512
ESC_SA3961AA	10	512
ESC_SA3960AA	10	512
ESC_SA3959AA	10	512
ESC_SA3958AA	10	512
ESC_SA3957AA	11	936
ESC_SA3956AA	11	936
ESC_SA3955AA	11	936
ESC_SA3954AA	11	936
ESC_SA3953AA	11	936
ESC_SA3952AA	11	936
ESC_SA3951AA	11	936
ESC_SA3950AA	11	936
ESC_SA3949AA	11	936
ESC_SA3948AA	11	936
ESC_SA3947AA	11	936
ESC_SA3946AA	11	936
ESC_SA3945AA	11	936
ESC_SA3944AA	11	936
ESC_SA3943AA	11	936
ESC_SA3942AA	11	936
ESC_SA3941AA	11	936
ESC_SA3940AA	11	936
ESC_SA3939AA	11	936
ESC_SA3938AA	11	936
ESC_SA3937AA	11	936
ESC_SA3936AA	11	936
ESC_SA3935AA	11	936
ESC_SA3934AA	11	936
ESC_SA3933AA	11	936
ESC_SA3932AA	11	936
ESC_SA3931AA	11	936
ESC_SA3930AA	11	936
ESC_SA3929AA	11	936
ESC_SA3928AA	11	936
ESC_SA3927AA	11	936
ESC_SA3926AA	11	936
ESC_SA3925AA	11	936

ESC_SA3924AA	11	936
ESC_SA3923AA	11	936
ESC_SA3922AA	11	936
ESC_SA3921AA	11	936
ESC_SA3920AA	11	936
ESC_SA3919AA	648	359
ESC_SA3918AA	90	156
ESC_SA3917AA	362	234
ESC_SA3916AA	1060	512
ESC_SA3914AA	10	512
ESC_SA3913AA	10	512
ESC_SA3912AA	472	601
ESC_SA3911AA	10	512
ESC_SA3910AA	10	512
ESC_SA3909AA	10	512
ESC_SA3908AA	131	653
ESC_SA3903AA	38	669
ESC_SA3902AA	2569	107
ESC_SA3901AA	3777	50
ESC_SA3900AA	155	175
ESC_SA3899AA	155	175
ESC_SA3898AA	155	175
ESC_SA3897AA	155	175
ESC_SA3895AA	410	156
ESC_SA3892AA	11368	653
ESC_SA3891AA	11367	935
ESC_SA3890AA	245	261
ESC_SA3889AA	245	261
ESC_SA3888AA	152	257
ESC_SA3887AA	245	261
ESC_SA3886AA	245	261
ESC_SA3885AA	10	512
ESC_SA3883AA	245	261
ESC_SA3882AA	245	261
ESC_SA3881AA	245	261
ESC_SA3880AA	245	261
ESC_SA3879AA	245	261
ESC_SA3878AA	245	261
ESC_SA3877AA	245	261
ESC_SA3876AA	152	257
ESC_SA3875AA	152	257
ESC_SA3874AA	245	261
ESC_SA3873AA	152	257
ESC_SA3872AA	245	261
ESC_SA3871AA	770	587
ESC_SA3868AA	38	669
ESC_SA3867AA	4243	914
ESC_SA3866AA	2253	71
ESC_SA3865AA	155	175
ESC_SA3864AA	3580	257

ESC_SA3863AA	23	156
ESC_SA3862AA	131	653
ESC_SA3861AA	23	156
ESC_SA3860AA	1618	736
ESC_SA3859AA	9956	878
ESC_SA3858AA	10519	175
ESC_SA3857AA	155	175
ESC_SA3856AA	101	696
ESC_SA3855AA	23	156
ESC_SA3854AA	8582	912
ESC_SA3853AA	3580	257
ESC_SA3852AA	38	669
ESC_SA3851AA	3580	257
ESC_SA3850AA	69	387
ESC_SA3849AA	117	592
ESC_SA3848AA	212	198
ESC_SA3847AA	2040	198
ESC_SA3846AA	1684	504
ESC_SA3845AA	3580	257
ESC_SA3844AA	978	740
ESC_SA3843AA	297	234
ESC_SA3842AA	9316	861
ESC_SA3841AA	681	393
ESC_SA3840AA	155	175
ESC_SA3839AA	1167	257
ESC_SA3838AA	542	824
ESC_SA3837AA	428	654
ESC_SA3836AA	1434	512
ESC_SA3828AA	7611	512
ESC_SA3826AA	401	142
ESC_SA3817AA	355	764
ESC_SA3806AA	11377	257
ESC_SA3802AA	10	512
ESC_SA3801AA	10	512
ESC_SA3800AA	162	924
ESC_SA3799AA	3525	504
ESC_SA3798AA	1485	408
ESC_SA3797AA	5765	234
ESC_SA3796AA	23	156
ESC_SA3795AA	6488	198
ESC_SA3794AA	162	924
ESC_SA3793AA	57	50
ESC_SA3792AA	2223	76
ESC_SA3791AA	58	198
ESC_SA3790AA	1727	257
ESC_SA3789AA	10	512
ESC_SA3788AA	602	257
ESC_SA3787AA	154	257
ESC_SA3786AA	11376	76
ESC_SA3785AA	355	764

ESC_SA3784AA	164	175
ESC_SA3783AA	162	924
ESC_SA3782AA	164	175
ESC_SA3781AA	10	512
ESC_SA3780AA	770	587
ESC_SA3779AA	538	629
ESC_SA3778AA	10	512
ESC_SA3777AA	744	512
ESC_SA3776AA	1316	471
ESC_SA3775AA	136	392
ESC_SA3774AA	1510	4
ESC_SA3773AA	4995	198
ESC_SA3772AA	2175	212
ESC_SA3771AA	10	512
ESC_SA3770AA	117	592
ESC_SA3769AA	88	160
ESC_SA3768AA	23	156
ESC_SA3767AA	428	654
ESC_SA3766AA	1844	198
ESC_SA3765AA	38	669
ESC_SA3764AA	117	592
ESC_SA3763AA	457	595
ESC_SA3762AA	70	670
ESC_SA3761AA	1429	198
ESC_SA3760AA	58	198
ESC_SA3759AA	5853	868
ESC_SA3758AA	1611	198
ESC_SA3757AA	117	592
ESC_SA3756AA	58	198
ESC_SA3755AA	6189	175
ESC_SA3754AA	607	355
ESC_SA3753AA	1611	198
ESC_SA3752AA	399	198
ESC_SA3751AA	101	696
ESC_SA3750AA	117	592
ESC_SA3749AA	11375	512
ESC_SA3748AA	1844	198
ESC_SA3747AA	355	764
ESC_SA3746AA	117	592
ESC_SA3745AA	1080	198
ESC_SA3744AA	69	387
ESC_SA3743AA	56	198
ESC_SA3742AA	93	71
ESC_SA3741AA	2973	234
ESC_SA3740AA	9588	258
ESC_SA3739AA	58	198
ESC_SA3738AA	1236	380
ESC_SA3737AA	69	387
ESC_SA3736AA	155	175
ESC_SA3735AA	398	445

ESC_SA3734AA	101	696
ESC_SA3733AA	871	447
ESC_SA3732AA	10	512
ESC_SA3731AA	154	257
ESC_SA3730AA	11374	808
ESC_SA3729AA	345	175
ESC_SA3728AA	11373	1028
ESC_SA3727AA	536	141
ESC_SA3726AA	10	512
ESC_SA3725AA	5853	868
ESC_SA3724AA	11372	166
ESC_SA3723AA	212	198
ESC_SA3722AA	58	198
ESC_SA3721AA	58	198
ESC_SA3720AA	681	393
ESC_SA3719AA	11371	587
ESC_SA3718AA	401	142
ESC_SA3717AA	355	764
ESC_SA3716AA	345	175
ESC_SA3715AA	48	76
ESC_SA3714AA	58	198
ESC_SA3713AA	399	198
ESC_SA3712AA	101	696
ESC_SA3711AA	1139	447
ESC_SA3710AA	877	935
ESC_SA3709AA	117	592
ESC_SA3708AA	11370	118
ESC_SA3707AA	8161	76
ESC_SA3706AA	93	71
ESC_SA3705AA	1844	198
ESC_SA3704AA	5493	198
ESC_SA3703AA	388	156
ESC_SA3702AA	607	355
ESC_SA3701AA	1665	601
ESC_SA3700AA	11369	175
ESC_SA3699AA	93	71
ESC_SA3698AA	69	387
ESC_SA3697AA	57	50
ESC_SA3696AA	88	160
ESC_SA3695AA	2556	630
ESC_SA3694AA	8618	512
ESC_SA3693AA	86	935
ESC_SA3692AA	1147	198
ESC_SA3691AA	5340	826
ESC_SA3690AA	453	935
ESC_SA3689AA	23	156
ESC_SA3688AA	101	696
ESC_SA3687AA	355	764
ESC_SA3686AA	3580	257
ESC_SA3685AA	58	198

ESC_SA3684AA	3580	257
ESC_SA3683AA	9956	878
ESC_SA3682AA	5440	160
ESC_SA3681AA	10	512
ESC_SA3680AA	115	669
ESC_SA3679AA	124	254
ESC_SA3678AA	399	198
ESC_SA3677AA	1434	512
ESC_SA3676AA	399	198
ESC_SA3675AA	11216	886
ESC_SA3674AA	770	587
ESC_SA3673AA	1434	512
ESC_SA3672AA	11378	234
ESC_SA3671AA	216	512
ESC_SA3670AA	2307	198
ESC_SA3669AA	23	156
ESC_SA3668AA	5891	878
ESC_SA3667AA	117	592
ESC_SA3666AA	48	76
ESC_SA3665AA	2531	198
ESC_SA3664AA	1308	257
ESC_SA3663AA	1618	736
ESC_SA3662AA	2079	198
ESC_SA3661AA	399	198
ESC_SA3660AA	13	257
ESC_SA3659AA	457	595
ESC_SA3658AA	38	669
ESC_SA3652AA	58	198
ESC_SA3651AA	10	512
ESC_SA3650AA	5940	555
ESC_SA3648AA	617	511
ESC_SA3647AA	1312	76
ESC_SA3646AA	131	653
ESC_SA3645AA	131	653
ESC_SA3643AA	11016	512
ESC_SA3642AA	542	824
ESC_SA3641AA	48	76
ESC_SA3640AA	70	670
ESC_SA3639AA	43	512
ESC_SA3638AA	602	257
ESC_SA3637AA	227	504
ESC_SA3636AA	205	257
ESC_SA3635AA	131	653
ESC_SA3634AA	167	512
ESC_SA3633AA	648	359
ESC_SA3632AA	2141	597
ESC_SA3631AA	744	512
ESC_SA3630AA	11317	447
ESC_SA3629AA	1114	504
ESC_SA3628AA	3075	701

ESC_SA3627AA	38	669
ESC_SA3626AA	349	670
ESC_SA3625AA	349	670
ESC_SA3624AA	443	257
ESC_SA3623AA	449	387
ESC_SA3622AA	382	481
ESC_SA3621AA	38	669
ESC_SA3620AA	10	512
ESC_SA3619AA	10292	512
ESC_SA3618AA	517	924
ESC_SA3617AA	393	343
ESC_SA3616AA	46	447
ESC_SA3615AA	3910	672
ESC_SA3614AA	1434	512
ESC_SA3613AA	38	669
ESC_SA3612AA	159	789
ESC_SA3611AA	609	447
ESC_SA3610AA	38	669
ESC_SA3609AA	3177	362
ESC_SA3608AA	38	669
ESC_SA3607AA	744	512
ESC_SA3606AA	2346	435
ESC_SA3605AA	617	511
ESC_SA3604AA	617	511
ESC_SA3603AA	38	669
ESC_SA3602AA	69	387
ESC_SA3601AA	69	387
ESC_SA3600AA	162	924
ESC_SA3599AA	772	76
ESC_SA3598AA	648	359
ESC_SA3597AA	131	653
ESC_SA3596AA	90	156
ESC_SA3595AA	11379	701
ESC_SA3594AA	155	175
ESC_SA3593AA	38	669
ESC_SA3592AA	226	512
ESC_SA3591AA	38	669
ESC_SA3590AA	1193	761
ESC_SA3589AA	3171	515
ESC_SA3588AA	609	447
ESC_SA3587AA	131	653
ESC_SA3586AA	7174	512
ESC_SA3585AA	7122	519
ESC_SA3584AA	665	79
ESC_SA3583AA	48	76
ESC_SA3582AA	381	156
ESC_SA3581AA	3171	515
ESC_SA3580AA	58	198
ESC_SA3579AA	38	669
ESC_SA3578AA	393	343

ESC_SA3577AA	398	445
ESC_SA3576AA	227	504
ESC_SA3575AA	485	587
ESC_SA3574AA	10	512
ESC_SA3573AA	38	669
ESC_SA3572AA	38	669
ESC_SA3571AA	34	504
ESC_SA3570AA	38	669
ESC_SA3569AA	3171	515
ESC_SA3568AA	88	160
ESC_SA3567AA	6488	198
ESC_SA3566AA	155	175
ESC_SA3565AA	5899	592
ESC_SA3564AA	405	923
ESC_SA3563AA	405	923
ESC_SA3562AA	10	512
ESC_SA3561AA	11381	669
ESC_SA3560AA	1193	761
ESC_SA3559AA	38	669
ESC_SA3558AA	4450	844
ESC_SA3557AA	656	789
ESC_SA3556AA	70	670
ESC_SA3555AA	226	512
ESC_SA3554AA	226	512
ESC_SA3553AA	10	512
ESC_SA3552AA	405	923
ESC_SA3551AA	131	653
ESC_SA3550AA	38	669
ESC_SA3549AA	131	653
ESC_SA3548AA	2141	597
ESC_SA3547AA	69	387
ESC_SA3546AA	38	669
ESC_SA3545AA	393	343
ESC_SA3544AA	38	669
ESC_SA3543AA	38	669
ESC_SA3542AA	449	387
ESC_SA3541AA	38	669
ESC_SA3540AA	69	387
ESC_SA3539AA	69	387
ESC_SA3538AA	69	387
ESC_SA3537AA	69	387
ESC_SA3536AA	131	653
ESC_SA3535AA	38	669
ESC_SA3534AA	11379	701
ESC_SA3533AA	38	669
ESC_SA3532AA	131	653
ESC_SA3531AA	405	923
ESC_SA3530AA	131	653
ESC_SA3529AA	38	669
ESC_SA3528AA	393	343

ESC_SA3527AA	405	923
ESC_SA3526AA	648	359
ESC_SA3525AA	485	587
ESC_SA3524AA	38	669
ESC_SA3523AA	405	923
ESC_SA3522AA	38	669
ESC_SA3521AA	501	900
ESC_SA3520AA	617	511
ESC_SA3519AA	617	511
ESC_SA3518AA	10	512
ESC_SA3517AA	10	512
ESC_SA3516AA	10	512
ESC_SA3515AA	10	512
ESC_SA3514AA	46	447
ESC_SA3513AA	10	512
ESC_SA3512AA	410	156
ESC_SA3511AA	1114	504
ESC_SA3510AA	38	669
ESC_SA3509AA	1114	504
ESC_SA3508AA	394	400
ESC_SA3507AA	394	400
ESC_SA3506AA	1312	76
ESC_SA3505AA	394	400
ESC_SA3504AA	617	511
ESC_SA3503AA	617	511
ESC_SA3502AA	617	511
ESC_SA3501AA	617	511
ESC_SA3500AA	617	511
ESC_SA3499AA	617	511
ESC_SA3498AA	617	511
ESC_SA3497AA	405	923
ESC_SA3496AA	405	923
ESC_SA3495AA	405	923
ESC_SA3494AA	405	923
ESC_SA3493AA	10	512
ESC_SA3492AA	10	512
ESC_SA3491AA	10	512
ESC_SA3490AA	10	512
ESC_SA3489AA	69	387
ESC_SA3488AA	69	387
ESC_SA3487AA	69	387
ESC_SA3486AA	69	387
ESC_SA3485AA	69	387
ESC_SA3484AA	69	387
ESC_SA3483AA	69	387
ESC_SA3482AA	131	653
ESC_SA3481AA	131	653
ESC_SA3480AA	131	653
ESC_SA3479AA	617	511
ESC_SA3478AA	410	156

ESC_SA3477AA	11382	653
ESC_SA3476AA	394	400
ESC_SA3475AA	648	359
ESC_SA3474AA	617	511
ESC_SA3473AA	405	923
ESC_SA3472AA	657	592
ESC_SA3471AA	69	387
ESC_SA3470AA	131	653
ESC_SA3469AA	6109	198
ESC_SA3468AA	642	924
ESC_SA3467AA	10	512
ESC_SA3466AA	6109	198
ESC_SA3465AA	6109	198
ESC_SA3464AA	6109	198
ESC_SA3463AA	602	257
ESC_SA3462AA	6968	935
ESC_SA3461AA	6968	935
ESC_SA3459AA	602	257
ESC_SA3458AA	602	257
ESC_SA3457AA	642	924
ESC_SA3456AA	602	257
ESC_SA3455AA	6968	935
ESC_SA3454AA	6968	935
ESC_SA3453AA	6968	935
ESC_SA3452AA	300	286
ESC_SA3451AA	4087	512
ESC_SA3450AA	642	924
ESC_SA3448AA	300	286
ESC_SA3447AA	10	512
ESC_SA3446AA	10	512
ESC_SA3445AA	428	654
ESC_SA3444AA	141	644
ESC_SA3443AA	1236	380
ESC_SA3442AA	549	387
ESC_SA3441AA	117	592
ESC_SA3440AA	3889	291
ESC_SA3439AA	9293	257
ESC_SA3438AA	648	359
ESC_SA3437AA	648	359
ESC_SA3436AA	38	669
ESC_SA3435AA	1049	175
ESC_SA3434AA	442	257
ESC_SA3433AA	33	198
ESC_SA3432AA	738	598
ESC_SA3431AA	28	537
ESC_SA3430AA	442	257
ESC_SA3429AA	442	257
ESC_SA3428AA	6274	257
ESC_SA3427AA	4151	971
ESC_SA3426AA	165	504

ESC_SA3425AA	398	445
ESC_SA3424AA	616	156
ESC_SA3423AA	10	512
ESC_SA3422AA	515	1004
ESC_SA3421AA	10	512
ESC_SA3420AA	10	512
ESC_SA3419AA	34	504
ESC_SA3418AA	224	198
ESC_SA3417AA	744	512
ESC_SA3416AA	224	198
ESC_SA3415AA	398	445
ESC_SA3414AA	10	512
ESC_SA3412AA	224	198
ESC_SA3411AA	398	445
ESC_SA3410AA	1585	512
ESC_SA3409AA	398	445
ESC_SA3408AA	2536	261
ESC_SA3407AA	10	512
ESC_SA3406AA	398	445
ESC_SA3405AA	10	512
ESC_SA3404AA	10	512
ESC_SA3403AA	11	936
ESC_SA3402AA	10	512
ESC_SA3401AA	10	512
ESC_SA3400AA	10	512
ESC_SA3399AA	10	512
ESC_SA3398AA	10	512
ESC_SA3397AA	10	512
ESC_SA3396AA	2520	878
ESC_SA3395AA	2385	198
ESC_SA3394AA	8354	268
ESC_SA3393AA	515	1004
ESC_SA3392AA	5937	257
ESC_SA3391AA	770	587
ESC_SA3390AA	441	198
ESC_SA3389AA	7873	198
ESC_SA3388AA	295	160
ESC_SA3387AA	5973	92
ESC_SA3386AA	5973	92
ESC_SA3385AA	205	257
ESC_SA3384AA	295	160
ESC_SA3383AA	2178	924
ESC_SA3381AA	58	198
ESC_SA3380AA	58	198
ESC_SA3375AA	11	936
ESC_SA3374AA	11	936
ESC_SA3373AA	11	936
ESC_SA3371AA	11	936
ESC_SA3370AA	21	156
ESC_SA3369AA	11	936

ESC_SA3367AA	11	936
ESC_SA3366AA	224	198
ESC_SA3365AA	224	198
ESC_SA3364AA	10	512
ESC_SA3363AA	224	198
ESC_SA3362AA	224	198
ESC_SA3361AA	10	512
ESC_SA3360AA	58	198
ESC_SA3359AA	744	512
ESC_SA3358AA	6206	257
ESC_SA3357AA	224	198
ESC_SA3343AA	1967	191
ESC_SA3342AA	26	257
ESC_SA3341AA	1819	808
ESC_SA3340AA	10	512
ESC_SA3339AA	34	504
ESC_SA3338AA	21	156
ESC_SA3337AA	33	198
ESC_SA3336AA	34	504
ESC_SA3335AA	21	156
ESC_SA3334AA	32	270
ESC_SA3333AA	404	761
ESC_SA3332AA	21	156
ESC_SA3331AA	5627	435
ESC_SA3330AA	5584	640
ESC_SA3329AA	11386	160
ESC_SA3328AA	117	592
ESC_SA3327AA	278	924
ESC_SA3326AA	877	935
ESC_SA3325AA	278	924
ESC_SA3324AA	4198	878
ESC_SA3323AA	206	286
ESC_SA3322AA	327	204
ESC_SA3321AA	206	286
ESC_SA3320AA	11392	261
ESC_SA3319AA	752	512
ESC_SA3318AA	278	924
ESC_SA3317AA	1844	198
ESC_SA3316AA	10	512
ESC_SA3315AA	9439	512
ESC_SA3314AA	4458	325
ESC_SA3313AA	752	512
ESC_SA3312AA	48	76
ESC_SA3311AA	101	696
ESC_SA3310AA	1679	160
ESC_SA3309AA	155	175
ESC_SA3308AA	1434	512
ESC_SA3307AA	23	156
ESC_SA3306AA	297	234
ESC_SA3305AA	540	286

ESC_SA3304AA	1665	601
ESC_SA3303AA	11339	198
ESC_SA3302AA	10	512
ESC_SA3301AA	1056	257
ESC_SA3300AA	1303	791
ESC_SA3299AA	58	198
ESC_SA3298AA	1423	198
ESC_SA3297AA	156	175
ESC_SA3296AA	10	512
ESC_SA3295AA	648	359
ESC_SA3294AA	278	924
ESC_SA3293AA	515	1004
ESC_SA3292AA	4537	76
ESC_SA3291AA	58	198
ESC_SA3290AA	58	198
ESC_SA3289AA	1244	291
ESC_SA3288AA	10	512
ESC_SA3287AA	3107	512
ESC_SA3286AA	69	387
ESC_SA3285AA	641	935
ESC_SA3284AA	898	108
ESC_SA3283AA	388	156
ESC_SA3282AA	803	739
ESC_SA3281AA	4537	76
ESC_SA3280AA	75	257
ESC_SA3278AA	2307	198
ESC_SA3277AA	10	512
ESC_SA3276AA	8959	842
ESC_SA3275AA	711	935
ESC_SA3274AA	224	198
ESC_SA3273AA	10	512
ESC_SA3272AA	4768	820
ESC_SA3271AA	73	737
ESC_SA3270AA	555	653
ESC_SA3269AA	372	424
ESC_SA3268AA	131	653
ESC_SA3267AA	131	653
ESC_SA3266AA	131	653
ESC_SA3265AA	5891	878
ESC_SA3264AA	131	653
ESC_SA3263AA	998	644
ESC_SA3262AA	1861	726
ESC_SA3261AA	491	626
ESC_SA3260AA	372	424
ESC_SA3259AA	1861	726
ESC_SA3258AA	11	936
ESC_SA3257AA	43	512
ESC_SA3256AA	117	592
ESC_SA3255AA	101	696
ESC_SA3254AA	48	76

ESC_SA3253AA	720	900
ESC_SA3252AA	196	257
ESC_SA3251AA	7624	175
ESC_SA3250AA	1665	601
ESC_SA3249AA	399	198
ESC_SA3248AA	162	924
ESC_SA3247AA	3090	212
ESC_SA3246AA	10	512
ESC_SA3245AA	1246	198
ESC_SA3244AA	155	175
ESC_SA3243AA	362	234
ESC_SA3242AA	162	924
ESC_SA3241AA	429	654
ESC_SA3240AA	3190	221
ESC_SA3239AA	1818	71
ESC_SA3238AA	164	175
ESC_SA3236AA	162	924
ESC_SA3235AA	58	198
ESC_SA3234AA	135	653
ESC_SA3233AA	58	198
ESC_SA3232AA	1115	263
ESC_SA3231AA	73	737
ESC_SA3230AA	127	738
ESC_SA3229AA	538	629
ESC_SA3228AA	372	424
ESC_SA3227AA	1643	780
ESC_SA3226AA	372	424
ESC_SA3225AA	372	424
ESC_SA3224AA	1385	653
ESC_SA3223AA	141	644
ESC_SA3222AA	372	424
ESC_SA3221AA	372	424
ESC_SA3220AA	73	737
ESC_SA3219AA	5940	555
ESC_SA3218AA	372	424
ESC_SA3217AA	1193	761
ESC_SA3216AA	131	653
ESC_SA3215AA	555	653
ESC_SA3214AA	372	424
ESC_SA3213AA	73	737
ESC_SA3212AA	127	738
ESC_SA3211AA	73	737
ESC_SA3210AA	372	424
ESC_SA3209AA	646	738
ESC_SA3208AA	906	198
ESC_SA3207AA	961	623
ESC_SA3206AA	80	731
ESC_SA3205AA	372	424
ESC_SA3204AA	297	234
ESC_SA3203AA	646	738

ESC_SA3202AA	127	738
ESC_SA3201AA	372	424
ESC_SA3200AA	5600	373
ESC_SA3199AA	372	424
ESC_SA3198AA	372	424
ESC_SA3197AA	58	198
ESC_SA3196AA	127	738
ESC_SA3195AA	131	653
ESC_SA3194AA	372	424
ESC_SA3193AA	12	626
ESC_SA3192AA	83	740
ESC_SA3191AA	95	738
ESC_SA3190AA	10	512
ESC_SA3189AA	372	424
ESC_SA3188AA	372	424
ESC_SA3187AA	95	738
ESC_SA3186AA	12	626
ESC_SA3185AA	12	626
ESC_SA3184AA	372	424
ESC_SA3183AA	73	737
ESC_SA3182AA	141	644
ESC_SA3181AA	372	424
ESC_SA3180AA	38	669
ESC_SA3179AA	372	424
ESC_SA3178AA	2604	626
ESC_SA3177AA	12	626
ESC_SA3176AA	58	198
ESC_SA3175AA	73	737
ESC_SA3174AA	345	175
ESC_SA3173AA	83	740
ESC_SA3172AA	372	424
ESC_SA3171AA	372	424
ESC_SA3170AA	224	198
ESC_SA3169AA	301	307
ESC_SA3168AA	11366	257
ESC_SA3167AA	152	257
ESC_SA3166AA	245	261
ESC_SA3165AA	29	156
ESC_SA3164AA	2230	126
ESC_SA3163AA	394	400
ESC_SA3162AA	80	731
ESC_SA3161AA	59	217
ESC_SA3160AA	131	653
ESC_SA3159AA	38	669
ESC_SA3158AA	95	738
ESC_SA3157AA	95	738
ESC_SA3156AA	73	737
ESC_SA3155AA	95	738
ESC_SA3154AA	95	738
ESC_SA3153AA	162	924

ESC_SA3152AA	58	198
ESC_SA3151AA	95	738
ESC_SA3150AA	69	387
ESC_SA3149AA	144	740
ESC_SA3148AA	12	626
ESC_SA3147AA	80	731
ESC_SA3146AA	21	156
ESC_SA3145AA	21	156
ESC_SA3144AA	21	156
ESC_SA3143AA	29	156
ESC_SA3142AA	617	511
ESC_SA3141AA	648	359
ESC_SA3140AA	14	761
ESC_SA3139AA	73	737
ESC_SA3138AA	131	653
ESC_SA3137AA	38	669
ESC_SA3136AA	69	387
ESC_SA3135AA	69	387
ESC_SA3134AA	12	626
ESC_SA3133AA	73	737
ESC_SA3132AA	69	387
ESC_SA3131AA	11365	198
ESC_SA3130AA	11364	900
ESC_SA3129AA	11363	1029
ESC_SA3128AA	11362	380
ESC_SA3127AA	245	261
ESC_SA3126AA	245	261
ESC_SA3125AA	152	257
ESC_SA3124AA	152	257
ESC_SA3123AA	152	257
ESC_SA3122AA	152	257
ESC_SA3121AA	152	257
ESC_SA3120AA	152	257
ESC_SA3118AA	245	261
ESC_SA3117AA	301	307
ESC_SA3116AA	11359	598
ESC_SA3115AA	152	257
ESC_SA3114AA	245	261
ESC_SA3113AA	83	740
ESC_SA3112AA	127	738
ESC_SA3111AA	625	756
ESC_SA3110AA	2604	626
ESC_SA3109AA	6210	766
ESC_SA3108AA	12	626
ESC_SA3107AA	127	738
ESC_SA3106AA	12	626
ESC_SA3105AA	12	626
ESC_SA3104AA	127	738
ESC_SA3103AA	127	738
ESC_SA3102AA	2015	684

ESC_SA3101AA	127	738
ESC_SA3100AA	11358	653
ESC_SA3099AA	301	307
ESC_SA3098AA	301	307
ESC_SA3097AA	21	156
ESC_SA3096AA	21	156
ESC_SA3095AA	21	156
ESC_SA3094AA	29	156
ESC_SA3093AA	11	936
ESC_SA3092AA	744	512
ESC_SA3091AA	7581	996
ESC_SA3090AA	744	512
ESC_SA3089AA	457	595
ESC_SA3088AA	12	626
ESC_SA3087AA	3580	257
ESC_SA3086AA	127	738
ESC_SA3085AA	12	626
ESC_SA3084AA	9066	878
ESC_SA3083AA	2604	626
ESC_SA3082AA	11263	424
ESC_SA3081AA	162	924
ESC_SA3080AA	372	424
ESC_SA3079AA	12	626
ESC_SA3078AA	135	653
ESC_SA3077AA	135	653
ESC_SA3076AA	10	512
ESC_SA3075AA	127	738
ESC_SA3074AA	73	737
ESC_SA3073AA	929	742
ESC_SA3072AA	998	644
ESC_SA3071AA	12	626
ESC_SA3070AA	1735	737
ESC_SA3069AA	372	424
ESC_SA3068AA	127	738
ESC_SA3067AA	80	731
ESC_SA3066AA	201	257
ESC_SA3065AA	1193	761
ESC_SA3064AA	73	737
ESC_SA3063AA	410	156
ESC_SA3062AA	136	392
ESC_SA3061AA	73	737
ESC_SA3060AA	141	644
ESC_SA3059AA	1967	191
ESC_SA2962AA	1431	257
ESC_SA2961AA	1286	474
ESC_SA2960AA	69	387
ESC_SA2958AA	8877	842
ESC_SA2957AA	665	79
ESC_SA2956AA	359	696
ESC_SA2955AA	7104	234

ESC_SA2954AA	624	359
ESC_SA2921AA	8573	867
ESC_SA2920AA	8560	234
ESC_SA2919AA	1730	234
ESC_SA2918AA	752	512
ESC_SA2917AA	58	198
ESC_SA2916AA	442	257
ESC_SA2915AA	197	601
ESC_SA2914AA	607	355
ESC_SA2913AA	2307	198
ESC_SA2912AA	336	878
ESC_SA2911AA	10	512
ESC_SA2910AA	1081	198
ESC_SA2909AA	155	175
ESC_SA2908AA	99	198
ESC_SA2907AA	278	924
ESC_SA2906AA	11361	1018
ESC_SA2905AA	86	935
ESC_SA2904AA	9587	387
ESC_SA2903AA	2005	175
ESC_SA2902AA	1201	512
ESC_SA2901AA	1286	474
ESC_SA2900AA	154	257
ESC_SA2899AA	58	198
ESC_SA2898AA	164	175
ESC_SA2897AA	69	387
ESC_SA2892AA	245	261
ESC_SA2891AA	245	261
ESC_SA2890AA	245	261
ESC_SA2889AA	245	261
ESC_SA2888AA	11360	257
ESC_SA2887AA	21	156
ESC_SA2886AA	300	286
ESC_SA2885AA	278	924
ESC_SA2884AA	32	270
ESC_SA2883AA	201	257
ESC_SA2882AA	582	420
ESC_SA2881AA	6274	257
ESC_SA2880AA	11	936
ESC_SA2879AA	381	156
ESC_SA2878AA	33	198
ESC_SA2877AA	10988	283
ESC_SA2876AA	34	504
ESC_SA2875AA	582	420
ESC_SA2874AA	33	198
ESC_SA2873AA	405	923
ESC_SA2872AA	17	191
ESC_SA2871AA	3695	924
ESC_SA2870AA	33	198
ESC_SA2869AA	442	257

ESC_SA2868AA	1819	808
ESC_SA2867AA	33	198
ESC_SA2866AA	11357	878
ESC_SA2864AA	11356	285
ESC_SA2863AA	11355	156
ESC_SA2862AA	1892	846
ESC_SA2861AA	11	936
ESC_SA2860AA	752	512
ESC_SA2859AA	34	504
ESC_SA2858AA	765	156
ESC_SA2857AA	1041	765
ESC_SA2856AA	34	504
ESC_SA2855AA	152	257
ESC_SA2854AA	245	261
ESC_SA2853AA	245	261
ESC_SA2852AA	245	261
ESC_SA2851AA	152	257
ESC_SA2850AA	245	261
ESC_SA2849AA	245	261
ESC_SA2848AA	11	936
ESC_SA2847AA	11	936
ESC_SA2846AA	17	191
ESC_SA2845AA	11	936
ESC_SA2844AA	34	504
ESC_SA2843AA	11	936
ESC_SA2842AA	6144	512
ESC_SA2841AA	655	926
ESC_SA2840AA	5295	355
ESC_SA2839AA	11	936
ESC_SA2838AA	11	936
ESC_SA2837AA	11	936
ESC_SA2836AA	11	936
ESC_SA2835AA	17	191
ESC_SA2834AA	11	936
ESC_SA2833AA	11	936
ESC_SA2832AA	11	936
ESC_SA2831AA	11	936
ESC_SA2830AA	21	156
ESC_SA2829AA	11	936
ESC_SA2828AA	245	261
ESC_SA2827AA	245	261
ESC_SA2826AA	245	261
ESC_SA2825AA	245	261
ESC_SA2824AA	245	261
ESC_SA2823AA	442	257
ESC_SA2822AA	28	537
ESC_SA2821AA	5627	435
ESC_SA2820AA	8708	198
ESC_SA2819AA	21	156
ESC_SA2818AA	300	286

ESC_SA2817AA	442	257
ESC_SA2816AA	11	936
ESC_SA2815AA	11	936
ESC_SA2814AA	11	936
ESC_SA2813AA	11	936
ESC_SA2812AA	11	936
ESC_SA2811AA	11	936
ESC_SA2810AA	11	936
ESC_SA2809AA	11	936
ESC_SA2808AA	11	936
ESC_SA2807AA	11	936
ESC_SA2806AA	372	424
ESC_SA2803AA	6506	914
ESC_SA2802AA	666	257
ESC_SA2801AA	3672	649
ESC_SA2800AA	7456	512
ESC_SA2791AA	1286	474
ESC_SA2790AA	224	198
ESC_SA2789AA	10	512
ESC_SA2788AA	206	286
ESC_SA2787AA	641	935
ESC_SA2786AA	117	592
ESC_SA2785AA	392	234
ESC_SA2784AA	10	512
ESC_SA2783AA	218	519
ESC_SA2782AA	278	924
ESC_SA2781AA	752	512
ESC_SA2780AA	1914	956
ESC_SA2779AA	278	924
ESC_SA2778AA	58	198
ESC_SA2777AA	10	512
ESC_SA2776AA	164	175
ESC_SA2775AA	602	257
ESC_SA2774AA	155	175
ESC_SA2773AA	399	198
ESC_SA2772AA	1844	198
ESC_SA2771AA	641	935
ESC_SA2770AA	714	318
ESC_SA2769AA	607	355
ESC_SA2768AA	399	198
ESC_SA2767AA	11	936
ESC_SA2766AA	245	261
ESC_SA2765AA	245	261
ESC_SA2764AA	245	261
ESC_SA2763AA	245	261
ESC_SA2761AA	245	261
ESC_SA2760AA	145	256
ESC_SA2759AA	152	257
ESC_SA2758AA	245	261
ESC_SA2757AA	245	261

ESC_SA2756AA	245	261
ESC_SA2755AA	245	261
ESC_SA2754AA	245	261
ESC_SA2753AA	152	257
ESC_SA2752AA	245	261
ESC_SA2751AA	245	261
ESC_SA2750AA	21	156
ESC_SA2749AA	11	936
ESC_SA2748AA	11	936
ESC_SA2747AA	21	156
ESC_SA2746AA	11	936
ESC_SA2745AA	11	936
ESC_SA2744AA	80	731
ESC_SA2743AA	11	936
ESC_SA2742AA	11	936
ESC_SA2741AA	11	936
ESC_SA2740AA	93	71
ESC_SA2739AA	401	142
ESC_SA2738AA	491	626
ESC_SA2737AA	11	936
ESC_SA2736AA	11	936
ESC_SA2735AA	11	936
ESC_SA2734AA	11	936
ESC_SA2733AA	11	936
ESC_SA2732AA	21	156
ESC_SA2731AA	10	512
ESC_SA2730AA	206	286
ESC_SA2729AA	40	198
ESC_SA2728AA	11	936
ESC_SA2727AA	11354	842
ESC_SA2726AA	17	191
ESC_SA2725AA	33	198
ESC_SA2724AA	752	512
ESC_SA2723AA	11	936
ESC_SA2722AA	164	175
ESC_SA2721AA	11	936
ESC_SA2720AA	11	936
ESC_SA2719AA	11	936
ESC_SA2718AA	6992	842
ESC_SA2717AA	1056	257
ESC_SA2716AA	442	257
ESC_SA2715AA	386	191
ESC_SA2714AA	11	936
ESC_SA2713AA	69	387
ESC_SA2712AA	11	936
ESC_SA2711AA	11	936
ESC_SA2710AA	11	936
ESC_SA2709AA	11	936
ESC_SA2708AA	11347	285
ESC_SA2707AA	442	257

ESC_SA2706AA	11060	198
ESC_SA2705AA	11	936
ESC_SA2704AA	11	936
ESC_SA2703AA	21	156
ESC_SA2702AA	11	936
ESC_SA2701AA	399	198
ESC_SA2700AA	10	512
ESC_SA2699AA	752	512
ESC_SA2698AA	11	936
ESC_SA2697AA	2570	355
ESC_SA2696AA	11	936
ESC_SA2695AA	32	270
ESC_SA2693AA	100	504
ESC_SA2684AA	48	76
ESC_SA2683AA	48	76
ESC_SA2681AA	100	504
ESC_SA2680AA	100	504
ESC_SA2676AA	11353	307
ESC_SA2675AA	100	504
ESC_SA2669AA	100	504
ESC_SA2668AA	48	76
ESC_SA2663AA	100	504
ESC_SA2662AA	48	76
ESC_SA2660AA	100	504
ESC_SA2659AA	100	504
ESC_SA2658AA	48	76
ESC_SA2655AA	100	504
ESC_SA2654AA	11352	504
ESC_SA2653AA	100	504
ESC_SA2649AA	48	76
ESC_SA2646AA	100	504
ESC_SA2645AA	100	504
ESC_SA2641AA	100	504
ESC_SA2640AA	90	156
ESC_SA2637AA	100	504
ESC_SA2632AA	48	76
ESC_SA2630AA	23	156
ESC_SA2629AA	23	156
ESC_SA2628AA	23	156
ESC_SA2627AA	23	156
ESC_SA2626AA	23	156
ESC_SA2625AA	23	156
ESC_SA2624AA	42	257
ESC_SA2623AA	42	257
ESC_SA2622AA	42	257
ESC_SA2621AA	42	257
ESC_SA2620AA	42	257
ESC_SA2619AA	10	512
ESC_SA2618AA	10	512
ESC_SA2617AA	10	512

ESC_SA2616AA	10	512
ESC_SA2615AA	10	512
ESC_SA2614AA	10	512
ESC_SA2612AA	3524	935
ESC_SA2611AA	90	156
ESC_SA2604AA	11	936
ESC_SA2603AA	11	936
ESC_SA2602AA	10	512
ESC_SA2601AA	11	936
ESC_SA2600AA	32	270
ESC_SA2599AA	11	936
ESC_SA2598AA	11	936
ESC_SA2597AA	11	936
ESC_SA2596AA	11	936
ESC_SA2595AA	11	936
ESC_SA2594AA	11	936
ESC_SA2593AA	11	936
ESC_SA2592AA	59	217
ESC_SA2591AA	11	936
ESC_SA2590AA	11	936
ESC_SA2589AA	11	936
ESC_SA2588AA	11	936
ESC_SA2587AA	2570	355
ESC_SA2586AA	80	731
ESC_SA2585AA	10	512
ESC_SA2584AA	165	504
ESC_SA2583AA	13	257
ESC_SA2582AA	21	156
ESC_SA2581AA	442	257
ESC_SA2580AA	442	257
ESC_SA2579AA	442	257
ESC_SA2578AA	11	936
ESC_SA2577AA	10	512
ESC_SA2576AA	88	160
ESC_SA2575AA	17	191
ESC_SA2574AA	11	936
ESC_SA2573AA	11	936
ESC_SA2572AA	29	156
ESC_SA2571AA	11	936
ESC_SA2570AA	1819	808
ESC_SA2569AA	442	257
ESC_SA2568AA	11	936
ESC_SA2567AA	32	270
ESC_SA2566AA	11	936
ESC_SA2565AA	442	257
ESC_SA2564AA	442	257
ESC_SA2563AA	300	286
ESC_SA2562AA	33	198
ESC_SA2561AA	21	156
ESC_SA2560AA	11	936

ESC_SA2559AA	442	257
ESC_SA2558AA	21	156
ESC_SA2557AA	11	936
ESC_SA2556AA	11	936
ESC_SA2555AA	11	936
ESC_SA2554AA	25	234
ESC_SA2553AA	788	659
ESC_SA2552AA	301	307
ESC_SA2551AA	34	504
ESC_SA2550AA	11	936
ESC_SA2549AA	11	936
ESC_SA2548AA	34	504
ESC_SA2547AA	442	257
ESC_SA2546AA	21	156
ESC_SA2545AA	29	156
ESC_SA2543AA	646	738
ESC_SA2542AA	929	742
ESC_SA2541AA	929	742
ESC_SA2540AA	929	742
ESC_SA2539AA	10	512
ESC_SA2538AA	2001	670
ESC_SA2537AA	8582	912
ESC_SA2536AA	4623	257
ESC_SA2535AA	9529	1002
ESC_SA2534AA	2001	670
ESC_SA2533AA	1611	198
ESC_SA2532AA	9422	868
ESC_SA2531AA	73	737
ESC_SA2530AA	117	592
ESC_SA2529AA	937	198
ESC_SA2528AA	1611	198
ESC_SA2527AA	2248	257
ESC_SA2526AA	174	660
ESC_SA2525AA	937	198
ESC_SA2524AA	1629	924
ESC_SA2523AA	24	277
ESC_SA2522AA	1718	269
ESC_SA2521AA	1718	269
ESC_SA2520AA	40	198
ESC_SA2519AA	38	669
ESC_SA2518AA	38	669
ESC_SA2517AA	4219	630
ESC_SA2516AA	1079	235
ESC_SA2515AA	1629	924
ESC_SA2514AA	2248	257
ESC_SA2513AA	2144	257
ESC_SA2512AA	2144	257
ESC_SA2511AA	1079	235
ESC_SA2510AA	4219	630
ESC_SA2509AA	297	234

ESC_SA2508AA	297	234
ESC_SA2507AA	9293	257
ESC_SA2506AA	583	768
ESC_SA2505AA	811	234
ESC_SA2503AA	58	198
ESC_SA2502AA	278	924
ESC_SA2501AA	5130	512
ESC_SA2500AA	117	592
ESC_SA2499AA	162	924
ESC_SA2498AA	278	924
ESC_SA2497AA	101	696
ESC_SA2496AA	10	512
ESC_SA2495AA	1201	512
ESC_SA2494AA	154	257
ESC_SA2493AA	48	76
ESC_SA2492AA	11351	842
ESC_SA2491AA	131	653
ESC_SA2490AA	11350	669
ESC_SA2488AA	152	257
ESC_SA2487AA	11349	234
ESC_SA2486AA	245	261
ESC_SA2485AA	245	261
ESC_SA2484AA	6200	261
ESC_SA2483AA	11348	234
ESC_SA2482AA	152	257
ESC_SA2481AA	245	261
ESC_SA2480AA	152	257
ESC_SA2479AA	245	261
ESC_SA2478AA	1502	257
ESC_SA2477AA	152	257
ESC_SA2476AA	152	257
ESC_SA2475AA	152	257
ESC_SA2474AA	245	261
ESC_SA2473AA	152	257
ESC_SA2472AA	152	257
ESC_SA2471AA	152	257
ESC_SA2470AA	11	936
ESC_SA2469AA	11	936
ESC_SA2468AA	11	936
ESC_SA2467AA	11	936
ESC_SA2466AA	11	936
ESC_SA2465AA	11	936
ESC_SA2464AA	11	936
ESC_SA2463AA	11	936
ESC_SA2462AA	11	936
ESC_SA2461AA	21	156
ESC_SA2460AA	11	936
ESC_SA2459AA	11	936
ESC_SA2458AA	11	936
ESC_SA2457AA	404	761

ESC_SA2456AA	301	307
ESC_SA2455AA	301	307
ESC_SA2454AA	301	307
ESC_SA2453AA	301	307
ESC_SA2452AA	29	156
ESC_SA2451AA	29	156
ESC_SA2450AA	29	156
ESC_SA2449AA	29	156
ESC_SA2448AA	21	156
ESC_SA2447AA	21	156
ESC_SA2445AA	361	512
ESC_SA2444AA	297	234
ESC_SA2443AA	361	512
ESC_SA2442AA	297	234
ESC_SA2441AA	11345	160
ESC_SA2440AA	11340	696
ESC_SA2439AA	11339	198
ESC_SA2438AA	906	198
ESC_SA2437AA	152	257
ESC_SA2436AA	245	261
ESC_SA2435AA	245	261
ESC_SA2434AA	152	257
ESC_SA2433AA	145	256
ESC_SA2432AA	245	261
ESC_SA2430AA	245	261
ESC_SA2429AA	245	261
ESC_SA2428AA	6200	261
ESC_SA2427AA	245	261
ESC_SA2426AA	635	141
ESC_SA2425AA	404	761
ESC_SA2424AA	10909	743
ESC_SA2423AA	10	512
ESC_SA2422AA	21	156
ESC_SA2421AA	2531	198
ESC_SA2420AA	176	601
ESC_SA2419AA	10818	257
ESC_SA2418AA	11346	738
ESC_SA2417AA	399	198
ESC_SA2416AA	442	257
ESC_SA2415AA	21	156
ESC_SA2414AA	69	387
ESC_SA2413AA	155	175
ESC_SA2412AA	34	504
ESC_SA2411AA	3101	160
ESC_SA2410AA	409	504
ESC_SA2409AA	11347	285
ESC_SA2408AA	442	257
ESC_SA2401AA	152	257
ESC_SA2400AA	245	261
ESC_SA2399AA	6200	261

ESC_SA2398AA	11	936
ESC_SA2397AA	5627	435
ESC_SA2396AA	11	936
ESC_SA2395AA	11	936
ESC_SA2394AA	21	156
ESC_SA2393AA	21	156
ESC_SA2392AA	25	234
ESC_SA2391AA	11	936
ESC_SA2390AA	11	936
ESC_SA2389AA	21	156
ESC_SA2388AA	11	936
ESC_SA2387AA	442	257
ESC_SA2386AA	21	156
ESC_SA2385AA	11	936
ESC_SA2384AA	106	387
ESC_SA2383AA	131	653
ESC_SA2382AA	847	257
ESC_SA2381AA	164	175
ESC_SA2380AA	336	878
ESC_SA2379AA	10	512
ESC_SA2378AA	5743	257
ESC_SA2377AA	165	504
ESC_SA2376AA	1125	198
ESC_SA2375AA	23	156
ESC_SA2374AA	109	257
ESC_SA2373AA	9598	76
ESC_SA2372AA	278	924
ESC_SA2371AA	11344	519
ESC_SA2370AA	10	512
ESC_SA2369AA	10	512
ESC_SA2368AA	69	387
ESC_SA2367AA	295	160
ESC_SA2366AA	540	286
ESC_SA2365AA	58	198
ESC_SA2364AA	164	175
ESC_SA2363AA	3519	101
ESC_SA2362AA	399	198
ESC_SA2361AA	155	175
ESC_SA2360AA	1400	666
ESC_SA2359AA	278	924
ESC_SA2358AA	399	198
ESC_SA2357AA	2602	257
ESC_SA2356AA	69	387
ESC_SA2355AA	399	198
ESC_SA2354AA	7416	372
ESC_SA2353AA	635	141
ESC_SA2352AA	1304	257
ESC_SA2351AA	1830	512
ESC_SA2350AA	442	257
ESC_SA2349AA	1463	263

ESC_SA2348AA	3519	101
ESC_SA2347AA	7040	156
ESC_SA2346AA	3779	926
ESC_SA2345AA	442	257
ESC_SA2344AA	1665	601
ESC_SA2343AA	141	644
ESC_SA2342AA	216	512
ESC_SA2341AA	399	198
ESC_SA2340AA	906	198
ESC_SA2339AA	5552	261
ESC_SA2338AA	11	936
ESC_SA2337AA	11	936
ESC_SA2336AA	11	936
ESC_SA2335AA	11	936
ESC_SA2334AA	11	936
ESC_SA2333AA	11	936
ESC_SA2332AA	11	936
ESC_SA2331AA	11	936
ESC_SA2330AA	1723	900
ESC_SA2329AA	141	644
ESC_SA2328AA	4481	257
ESC_SA2327AA	174	660
ESC_SA2326AA	73	737
ESC_SA2325AA	127	738
ESC_SA2324AA	569	738
ESC_SA2323AA	9422	868
ESC_SA2322AA	136	392
ESC_SA2321AA	589	650
ESC_SA2320AA	162	924
ESC_SA2319AA	17	191
ESC_SA2318AA	11	936
ESC_SA2317AA	11	936
ESC_SA2316AA	17	191
ESC_SA2315AA	442	257
ESC_SA2314AA	11	936
ESC_SA2313AA	117	592
ESC_SA2312AA	73	737
ESC_SA2311AA	117	592
ESC_SA2310AA	967	212
ESC_SA2309AA	131	653
ESC_SA2308AA	11343	198
ESC_SA2307AA	162	924
ESC_SA2306AA	1170	629
ESC_SA2305AA	144	740
ESC_SA2304AA	73	737
ESC_SA2303AA	372	424
ESC_SA2302AA	132	924
ESC_SA2301AA	162	924
ESC_SA2300AA	73	737
ESC_SA2299AA	162	924

ESC_SA2298AA	327	204
ESC_SA2297AA	12	626
ESC_SA2296AA	11342	653
ESC_SA2295AA	3018	903
ESC_SA2294AA	73	737
ESC_SA2293AA	12	626
ESC_SA2292AA	967	212
ESC_SA2291AA	258	190
ESC_SA2290AA	1512	197
ESC_SA2289AA	11338	936
ESC_SA2288AA	17	191
ESC_SA2287AA	21	156
ESC_SA2286AA	11	936
ESC_SA2285AA	11	936
ESC_SA2284AA	17	191
ESC_SA2283AA	11337	191
ESC_SA2282AA	17	191
ESC_SA2281AA	300	286
ESC_SA2280AA	442	257
ESC_SA2279AA	752	512
ESC_SA2278AA	752	512
ESC_SA2277AA	33	198
ESC_SA2276AA	6274	257
ESC_SA2275AA	11	936
ESC_SA2273AA	1084	180
ESC_SA2272AA	73	737
ESC_SA2271AA	537	756
ESC_SA2270AA	127	738
ESC_SA2269AA	127	738
ESC_SA2268AA	5407	738
ESC_SA2267AA	404	761
ESC_SA2266AA	404	761
ESC_SA2264AA	80	731
ESC_SA2263AA	73	737
ESC_SA2262AA	73	737
ESC_SA2261AA	127	738
ESC_SA2260AA	95	738
ESC_SA2259AA	73	737
ESC_SA2258AA	127	738
ESC_SA2257AA	1441	740
ESC_SA2256AA	1122	307
ESC_SA2255AA	95	738
ESC_SA2254AA	10	512
ESC_SA2253AA	10	512
ESC_SA2252AA	706	422
ESC_SA2251AA	5423	76
ESC_SA2250AA	88	160
ESC_SA2249AA	537	756
ESC_SA2248AA	127	738
ESC_SA2247AA	127	738

ESC_SA2246AA	5407	738
ESC_SA2245AA	5423	76
ESC_SA2244AA	127	738
ESC_SA2243AA	80	731
ESC_SA2242AA	73	737
ESC_SA2241AA	73	737
ESC_SA2240AA	12	626
ESC_SA2239AA	73	737
ESC_SA2238AA	127	738
ESC_SA2236AA	1128	924
ESC_SA2231AA	73	737
ESC_SA2230AA	12	626
ESC_SA2229AA	95	738
ESC_SA2228AA	73	737
ESC_SA2227AA	219	877
ESC_SA2226AA	4198	878
ESC_SA2225AA	73	737
ESC_SA2223AA	62	574
ESC_SA2222AA	12	626
ESC_SA2220AA	73	737
ESC_SA2219AA	11341	156
ESC_SA2218AA	5429	156
ESC_SA2217AA	11	936
ESC_SA2216AA	11	936
ESC_SA2215AA	11	936
ESC_SA2214AA	11	936
ESC_SA2213AA	11	936
ESC_SA2212AA	11	936
ESC_SA2211AA	11	936
ESC_SA2210AA	11	936
ESC_SA2209AA	11	936
ESC_SA2208AA	11	936
ESC_SA2207AA	11	936
ESC_SA2206AA	537	756
ESC_SA2205AA	245	261
ESC_SA2204AA	206	286
ESC_SA2203AA	69	387
ESC_SA2202AA	21	156
ESC_SA2201AA	21	156
ESC_SA2200AA	34	504
ESC_SA2199AA	21	156
ESC_SA2198AA	34	504
ESC_SA2197AA	10	512
ESC_SA2196AA	21	156
ESC_SA2195AA	10	512
ESC_SA2192AA	11	936
ESC_SA2191AA	11	936
ESC_SA2190AA	738	598
ESC_SA2189AA	11336	198
ESC_SA2188AA	655	926

ESC_SA2187AA	11	936
ESC_SA2168AA	73	737
ESC_SA2167AA	4995	198
ESC_SA2166AA	4995	198
ESC_SA2165AA	963	669
ESC_SA2164AA	963	669
ESC_SA2157AA	1967	191
ESC_SA2156AA	11	936
ESC_SA2155AA	5560	929
ESC_SA2154AA	5560	929
ESC_SA2153AA	11335	286
ESC_SA2152AA	4685	87
ESC_SA2151AA	11	936
ESC_SA2150AA	11	936
ESC_SA2149AA	11	936
ESC_SA2148AA	11	936
ESC_SA2147AA	10	512
ESC_SA2146AA	10	512
ESC_SA2145AA	10	512
ESC_SA2144AA	10	512
ESC_SA2143AA	10	512
ESC_SA2142AA	10	512
ESC_SA2141AA	10	512
ESC_SA2140AA	10	512
ESC_SA2139AA	10	512
ESC_SA2138AA	11	936
ESC_SA2137AA	11333	257
ESC_SA2136AA	11334	512
ESC_SA2135AA	152	257
ESC_SA2134AA	5592	830
ESC_SA2133AA	245	261
ESC_SA2132AA	245	261
ESC_SA2131AA	152	257
ESC_SA2130AA	145	256
ESC_SA2129AA	245	261
ESC_SA2128AA	8878	261
ESC_SA2127AA	11	936
ESC_SA2126AA	11	936
ESC_SA2125AA	301	307
ESC_SA2124AA	21	156
ESC_SA2123AA	5560	929
ESC_SA2122AA	5560	929
ESC_SA2121AA	5560	929
ESC_SA2120AA	5560	929
ESC_SA2119AA	5560	929
ESC_SA2118AA	5560	929
ESC_SA2117AA	5560	929
ESC_SA2116AA	5560	929
ESC_SA2115AA	5560	929
ESC_SA2114AA	5560	929

ESC_SA2113AA	5560	929
ESC_SA2112AA	5560	929
ESC_SA2111AA	5560	929
ESC_SA2110AA	5560	929
ESC_SA2109AA	5560	929
ESC_SA2108AA	5560	929
ESC_SA2107AA	5560	929
ESC_SA2106AA	5560	929
ESC_SA2105AA	5560	929
ESC_SA2104AA	5560	929
ESC_SA2103AA	5560	929
ESC_SA2102AA	5560	929
ESC_SA2101AA	5560	929
ESC_SA2100AA	5560	929
ESC_SA2099AA	48	76
ESC_SA2098AA	245	261
ESC_SA2097AA	152	257
ESC_SA2096AA	95	738
ESC_SA2095AA	517	924
ESC_SA2094AA	95	738
ESC_SA2093AA	300	286
ESC_SA2092AA	295	160
ESC_SA2091AA	21	156
ESC_SA2090AA	73	737
ESC_SA2089AA	11497	261
ESC_SA2088AA	73	737
ESC_SA2087AA	200	198
ESC_SA2086AA	6	445
ESC_SA2085AA	398	445
ESC_SA2084AA	131	653
ESC_SA2083AA	17	191
ESC_SA2082AA	11	936
ESC_SA2081AA	1316	471
ESC_SA2080AA	1819	808
ESC_SA2079AA	10	512
ESC_SA2078AA	10	512
ESC_SA2077AA	10	512
ESC_SA2076AA	155	175
ESC_SA2075AA	155	175
ESC_SA2074AA	245	261
ESC_SA2073AA	245	261
ESC_SA2072AA	245	261
ESC_SA2071AA	245	261
ESC_SA2070AA	245	261
ESC_SA2069AA	245	261
ESC_SA2068AA	245	261
ESC_SA2067AA	245	261
ESC_SA2066AA	152	257
ESC_SA2065AA	245	261
ESC_SA2064AA	9277	743

ESC_SA2063AA	9277	743
ESC_SA2062AA	747	640
ESC_SA2061AA	38	669
ESC_SA2060AA	9281	156
ESC_SA2059AA	9274	504
ESC_SA2058AA	38	669
ESC_SA2057AA	58	198
ESC_SA2056AA	9274	504
ESC_SA2055AA	1204	961
ESC_SA2054AA	58	198
ESC_SA2053AA	540	286
ESC_SA2052AA	223	198
ESC_SA2051AA	316	257
ESC_SA2050AA	316	257
ESC_SA2049AA	316	257
ESC_SA2048AA	316	257
ESC_SA2047AA	181	311
ESC_SA2046AA	181	311
ESC_SA2045AA	9274	504
ESC_SA2044AA	181	311
ESC_SA2043AA	337	257
ESC_SA2042AA	181	311
ESC_SA2041AA	641	935
ESC_SA2040AA	10	512
ESC_SA2039AA	2741	696
ESC_SA2038AA	398	445
ESC_SA2037AA	469	257
ESC_SA2036AA	469	257
ESC_SA2035AA	469	257
ESC_SA2034AA	9274	504
ESC_SA2033AA	469	257
ESC_SA2032AA	452	743
ESC_SA2031AA	452	743
ESC_SA2030AA	155	175
ESC_SA2029AA	43	512
ESC_SA2028AA	9283	198
ESC_SA2027AA	43	512
ESC_SA2026AA	43	512
ESC_SA2025AA	43	512
ESC_SA2024AA	485	587
ESC_SA2023AA	485	587
ESC_SA2022AA	2705	504
ESC_SA2021AA	485	587
ESC_SA2020AA	485	587
ESC_SA2019AA	410	156
ESC_SA2018AA	515	1004
ESC_SA2017AA	410	156
ESC_SA2016AA	410	156
ESC_SA2015AA	410	156
ESC_SA2014AA	9279	311

ESC_SA2013AA	29	156
ESC_SA2012AA	9274	504
ESC_SA2011AA	9274	504
ESC_SA2010AA	9277	743
ESC_SA2009AA	747	640
ESC_SA2008AA	101	696
ESC_SA2007AA	2175	212
ESC_SA2006AA	101	696
ESC_SA2005AA	101	696
ESC_SA2004AA	10	512
ESC_SA2003AA	603	198
ESC_SA2002AA	10	512
ESC_SA2001AA	603	198
ESC_SA2000AA	1727	257
ESC_SA1999AA	2705	504
ESC_SA1998AA	603	198
ESC_SA1997AA	226	512
ESC_SA1996AA	59	217
ESC_SA1995AA	59	217
ESC_SA1994AA	59	217
ESC_SA1993AA	9278	72
ESC_SA1992AA	59	217
ESC_SA1991AA	5148	963
ESC_SA1990AA	5148	963
ESC_SA1989AA	5148	963
ESC_SA1988AA	2705	504
ESC_SA1987AA	11324	969
ESC_SA1986AA	5148	963
ESC_SA1985AA	394	400
ESC_SA1984AA	58	198
ESC_SA1983AA	394	400
ESC_SA1982AA	394	400
ESC_SA1981AA	394	400
ESC_SA1980AA	452	743
ESC_SA1979AA	452	743
ESC_SA1978AA	452	743
ESC_SA1977AA	2914	670
ESC_SA1976AA	11	936
ESC_SA1975AA	569	738
ESC_SA1974AA	131	653
ESC_SA1973AA	34	504
ESC_SA1972AA	8630	257
ESC_SA1965AA	11	936
ESC_SA1964AA	21	156
ESC_SA1963AA	11	936
ESC_SA1962AA	635	141
ESC_SA1961AA	21	156
ESC_SA1960AA	17	191
ESC_SA1959AA	2356	379
ESC_SA1958AA	40	198

ESC_SA1957AA	675	234
ESC_SA1956AA	6049	87
ESC_SA1955AA	5254	765
ESC_SA1954AA	25	234
ESC_SA1953AA	635	141
ESC_SA1952AA	21	156
ESC_SA1951AA	11	936
ESC_SA1950AA	11	936
ESC_SA1949AA	11	936
ESC_SA1948AA	21	156
ESC_SA1947AA	659	286
ESC_SA1946AA	32	270
ESC_SA1945AA	17	191
ESC_SA1944AA	2531	198
ESC_SA1936AA	131	653
ESC_SA1921AA	540	286
ESC_SA1920AA	21	156
ESC_SA1919AA	58	198
ESC_SA1918AA	11	936
ESC_SA1917AA	11	936
ESC_SA1916AA	2531	198
ESC_SA1915AA	11	936
ESC_SA1914AA	442	257
ESC_SA1913AA	21	156
ESC_SA1912AA	11	936
ESC_SA1911AA	11323	257
ESC_SA1910AA	11	936
ESC_SA1909AA	301	307
ESC_SA1908AA	300	286
ESC_SA1907AA	11	936
ESC_SA1906AA	504	738
ESC_SA1905AA	33	198
ESC_SA1904AA	21	156
ESC_SA1903AA	40	198
ESC_SA1902AA	11	936
ESC_SA1901AA	6	445
ESC_SA1900AA	11	936
ESC_SA1899AA	11	936
ESC_SA1898AA	155	175
ESC_SA1897AA	11	936
ESC_SA1896AA	11	936
ESC_SA1895AA	11	936
ESC_SA1894AA	409	504
ESC_SA1893AA	191	198
ESC_SA1892AA	11	936
ESC_SA1891AA	11	936
ESC_SA1890AA	11	936
ESC_SA1889AA	442	257
ESC_SA1888AA	4496	924
ESC_SA1887AA	10	512

ESC_SA1886AA	5292	738
ESC_SA1885AA	517	924
ESC_SA1884AA	342	286
ESC_SA1883AA	6880	726
ESC_SA1882AA	11	936
ESC_SA1881AA	90	156
ESC_SA1880AA	10	512
ESC_SA1879AA	90	156
ESC_SA1878AA	10	512
ESC_SA1877AA	90	156
ESC_SA1876AA	11322	156
ESC_SA1875AA	11322	156
ESC_SA1874AA	910	929
ESC_SA1873AA	42	257
ESC_SA1872AA	90	156
ESC_SA1871AA	11321	877
ESC_SA1870AA	227	504
ESC_SA1869AA	90	156
ESC_SA1868AA	90	156
ESC_SA1867AA	90	156
ESC_SA1866AA	90	156
ESC_SA1865AA	90	156
ESC_SA1864AA	42	257
ESC_SA1863AA	10	512
ESC_SA1862AA	90	156
ESC_SA1861AA	100	504
ESC_SA1860AA	88	160
ESC_SA1859AA	11332	935
ESC_SA1858AA	10	512
ESC_SA1857AA	4576	987
ESC_SA1856AA	4576	987
ESC_SA1855AA	5759	109
ESC_SA1854AA	42	257
ESC_SA1853AA	42	257
ESC_SA1852AA	42	257
ESC_SA1851AA	641	935
ESC_SA1850AA	410	156
ESC_SA1848AA	90	156
ESC_SA1847AA	88	160
ESC_SA1846AA	10	512
ESC_SA1845AA	23	156
ESC_SA1844AA	6354	447
ESC_SA1843AA	23	156
ESC_SA1842AA	90	156
ESC_SA1841AA	90	156
ESC_SA1840AA	90	156
ESC_SA1839AA	10	512
ESC_SA1838AA	10	512
ESC_SA1837AA	90	156
ESC_SA1836AA	10	512

ESC_SA1822AA	73	737
ESC_SA1821AA	73	737
ESC_SA1820AA	73	737
ESC_SA1819AA	73	737
ESC_SA1818AA	73	737
ESC_SA1817AA	73	737
ESC_SA1816AA	73	737
ESC_SA1815AA	73	737
ESC_SA1814AA	73	737
ESC_SA1813AA	73	737
ESC_SA1811AA	73	737
ESC_SA1810AA	73	737
ESC_SA1809AA	73	737
ESC_SA1808AA	73	737
ESC_SA1801AA	10	512
ESC_SA1799AA	21	156
ESC_SA1798AA	343	286
ESC_SA1797AA	17	191
ESC_SA1796AA	6048	87
ESC_SA1795AA	744	512
ESC_SA1794AA	963	669
ESC_SA1793AA	963	669
ESC_SA1792AA	69	387
ESC_SA1791AA	131	653
ESC_SA1790AA	131	653
ESC_SA1789AA	648	359
ESC_SA1788AA	216	512
ESC_SA1787AA	131	653
ESC_SA1786AA	648	359
ESC_SA1785AA	167	512
ESC_SA1784AA	131	653
ESC_SA1783AA	131	653
ESC_SA1782AA	131	653
ESC_SA1781AA	648	359
ESC_SA1780AA	131	653
ESC_SA1779AA	131	653
ESC_SA1778AA	73	737
ESC_SA1777AA	2554	653
ESC_SA1776AA	1193	761
ESC_SA1775AA	131	653
ESC_SA1774AA	131	653
ESC_SA1773AA	131	653
ESC_SA1772AA	131	653
ESC_SA1771AA	131	653
ESC_SA1770AA	405	923
ESC_SA1769AA	131	653
ESC_SA1768AA	540	286
ESC_SA1767AA	69	387
ESC_SA1766AA	10387	175
ESC_SA1765AA	127	738

ESC_SA1764AA	131	653
ESC_SA1763AA	131	653
ESC_SA1762AA	131	653
ESC_SA1761AA	648	359
ESC_SA1760AA	131	653
ESC_SA1759AA	59	217
ESC_SA1758AA	1193	761
ESC_SA1757AA	12	626
ESC_SA1756AA	69	387
ESC_SA1755AA	131	653
ESC_SA1754AA	602	257
ESC_SA1753AA	359	696
ESC_SA1752AA	167	512
ESC_SA1751AA	131	653
ESC_SA1750AA	998	644
ESC_SA1749AA	361	512
ESC_SA1748AA	38	669
ESC_SA1747AA	405	923
ESC_SA1746AA	131	653
ESC_SA1745AA	131	653
ESC_SA1744AA	131	653
ESC_SA1743AA	227	504
ESC_SA1742AA	131	653
ESC_SA1741AA	648	359
ESC_SA1740AA	131	653
ESC_SA1739AA	131	653
ESC_SA1738AA	648	359
ESC_SA1737AA	405	923
ESC_SA1736AA	131	653
ESC_SA1735AA	131	653
ESC_SA1734AA	131	653
ESC_SA1733AA	131	653
ESC_SA1732AA	131	653
ESC_SA1731AA	11328	734
ESC_SA1730AA	420	741
ESC_SA1729AA	372	424
ESC_SA1728AA	3572	512
ESC_SA1727AA	167	512
ESC_SA1726AA	131	653
ESC_SA1725AA	93	71
ESC_SA1724AA	38	669
ESC_SA1723AA	131	653
ESC_SA1722AA	5451	912
ESC_SA1721AA	69	387
ESC_SA1720AA	10	512
ESC_SA1719AA	131	653
ESC_SA1718AA	131	653
ESC_SA1717AA	131	653
ESC_SA1716AA	131	653
ESC_SA1715AA	131	653

ESC_SA1714AA	131	653
ESC_SA1713AA	131	653
ESC_SA1712AA	69	387
ESC_SA1711AA	59	217
ESC_SA1710AA	1316	471
ESC_SA1709AA	48	76
ESC_SA1708AA	617	511
ESC_SA1707AA	405	923
ESC_SA1706AA	131	653
ESC_SA1705AA	131	653
ESC_SA1704AA	1193	761
ESC_SA1703AA	131	653
ESC_SA1702AA	131	653
ESC_SA1701AA	131	653
ESC_SA1700AA	131	653
ESC_SA1699AA	12	626
ESC_SA1698AA	405	923
ESC_SA1697AA	131	653
ESC_SA1696AA	131	653
ESC_SA1695AA	131	653
ESC_SA1694AA	405	923
ESC_SA1693AA	131	653
ESC_SA1692AA	648	359
ESC_SA1691AA	131	653
ESC_SA1690AA	131	653
ESC_SA1689AA	405	923
ESC_SA1688AA	131	653
ESC_SA1687AA	131	653
ESC_SA1686AA	70	670
ESC_SA1685AA	405	923
ESC_SA1684AA	405	923
ESC_SA1683AA	361	512
ESC_SA1682AA	131	653
ESC_SA1681AA	1193	761
ESC_SA1680AA	405	923
ESC_SA1679AA	12	626
ESC_SA1678AA	940	257
ESC_SA1677AA	131	653
ESC_SA1676AA	131	653
ESC_SA1675AA	131	653
ESC_SA1674AA	131	653
ESC_SA1673AA	1967	191
ESC_SA1672AA	131	653
ESC_SA1671AA	131	653
ESC_SA1670AA	10102	738
ESC_SA1669AA	9239	256
ESC_SA1668AA	131	653
ESC_SA1667AA	328	924
ESC_SA1666AA	131	653
ESC_SA1665AA	410	156

ESC_SA1664AA	354	15
ESC_SA1663AA	2197	511
ESC_SA1662AA	38	669
ESC_SA1661AA	43	512
ESC_SA1660AA	44	512
ESC_SA1659AA	131	653
ESC_SA1658AA	117	592
ESC_SA1657AA	131	653
ESC_SA1656AA	162	924
ESC_SA1655AA	1284	604
ESC_SA1654AA	131	653
ESC_SA1653AA	155	175
ESC_SA1652AA	38	669
ESC_SA1651AA	453	935
ESC_SA1650AA	131	653
ESC_SA1649AA	1304	257
ESC_SA1648AA	617	511
ESC_SA1647AA	131	653
ESC_SA1646AA	537	756
ESC_SA1645AA	537	756
ESC_SA1644AA	537	756
ESC_SA1643AA	537	756
ESC_SA1642AA	537	756
ESC_SA1641AA	537	756
ESC_SA1640AA	537	756
ESC_SA1639AA	537	756
ESC_SA1638AA	537	756
ESC_SA1637AA	537	756
ESC_SA1636AA	537	756
ESC_SA1635AA	537	756
ESC_SA1634AA	537	756
ESC_SA1633AA	537	756
ESC_SA1632AA	537	756
ESC_SA1631AA	537	756
ESC_SA1630AA	537	756
ESC_SA1629AA	537	756
ESC_SA1628AA	537	756
ESC_SA1627AA	537	756
ESC_SA1626AA	537	756
ESC_SA1625AA	537	756
ESC_SA1624AA	354	15
ESC_SA1623AA	405	923
ESC_SA1622AA	2172	447
ESC_SA1621AA	410	156
ESC_SA1620AA	617	511
ESC_SA1619AA	457	595
ESC_SA1618AA	2309	669
ESC_SA1617AA	354	15
ESC_SA1614AA	48	76
ESC_SA1612AA	11327	815

ESC_SA1610AA	9401	198
ESC_SA1608AA	95	738
ESC_SA1607AA	115	669
ESC_SA1606AA	2599	257
ESC_SA1605AA	410	156
ESC_SA1601AA	11326	519
ESC_SA1597AA	657	592
ESC_SA1595AA	10	512
ESC_SA1593AA	405	923
ESC_SA1590AA	354	15
ESC_SA1588AA	405	923
ESC_SA1586AA	206	286
ESC_SA1585AA	205	257
ESC_SA1584AA	131	653
ESC_SA1583AA	224	198
ESC_SA1581AA	877	935
ESC_SA1579AA	8346	234
ESC_SA1577AA	410	156
ESC_SA1575AA	48	76
ESC_SA1574AA	46	447
ESC_SA1572AA	648	359
ESC_SA1571AA	167	512
ESC_SA1570AA	405	923
ESC_SA1566AA	453	935
ESC_SA1564AA	46	447
ESC_SA1563AA	8262	234
ESC_SA1562AA	942	198
ESC_SA1560AA	48	76
ESC_SA1559AA	167	512
ESC_SA1556AA	448	257
ESC_SA1555AA	542	824
ESC_SA1552AA	542	824
ESC_SA1550AA	10	512
ESC_SA1549AA	131	653
ESC_SA1547AA	48	76
ESC_SA1545AA	167	512
ESC_SA1544AA	410	156
ESC_SA1543AA	11325	672
ESC_SA1542AA	141	644
ESC_SA1540AA	11	936
ESC_SA1539AA	11	936
ESC_SA1538AA	11	936
ESC_SA1537AA	11	936
ESC_SA1536AA	11	936
ESC_SA1535AA	11	936
ESC_SA1534AA	11	936
ESC_SA1533AA	278	924
ESC_SA1532AA	11	936
ESC_SA1531AA	11	936
ESC_SA1530AA	11	936

ESC_SA1529AA	11	936
ESC_SA1528AA	21	156
ESC_SA1527AA	11	936
ESC_SA1526AA	11	936
ESC_SA1525AA	1303	791
ESC_SA1524AA	11	936
ESC_SA1523AA	11	936
ESC_SA1522AA	11	936
ESC_SA1521AA	542	824
ESC_SA1520AA	770	587
ESC_SA1519AA	69	387
ESC_SA1518AA	10939	198
ESC_SA1517AA	155	175
ESC_SA1516AA	569	738
ESC_SA1515AA	11331	670
ESC_SA1514AA	11330	257
ESC_SA1513AA	34	504
ESC_SA1512AA	164	175
ESC_SA1511AA	58	198
ESC_SA1510AA	11	936
ESC_SA1509AA	361	512
ESC_SA1508AA	1193	761
ESC_SA1507AA	68	386
ESC_SA1506AA	1079	235
ESC_SA1505AA	641	935
ESC_SA1504AA	38	669
ESC_SA1503AA	68	386
ESC_SA1502AA	131	653
ESC_SA1501AA	58	198
ESC_SA1500AA	4981	473
ESC_SA1499AA	11329	182
ESC_SA1498AA	10	512
ESC_SA1497AA	10	512
ESC_SA1496AA	10	512
ESC_SA1495AA	10	512
ESC_SA1494AA	10	512
ESC_SA1476AA	11319	198
ESC_SA1475AA	11318	257
ESC_SA1474AA	10893	198
ESC_SA1473AA	11320	900
ESC_SA1472AA	11317	447
ESC_SA1471AA	152	257
ESC_SA1470AA	152	257
ESC_SA1469AA	245	261
ESC_SA1468AA	99	198
ESC_SA1467AA	152	257
ESC_SA1466AA	245	261
ESC_SA1465AA	245	261
ESC_SA1464AA	245	261
ESC_SA1463AA	636	653

ESC_SA1462AA	69	387
ESC_SA1461AA	5484	738
ESC_SA1459AA	38	669
ESC_SA1458AA	131	653
ESC_SA1457AA	127	738
ESC_SA1456AA	624	359
ESC_SA1455AA	1193	761
ESC_SA1454AA	28	537
ESC_SA1453AA	167	512
ESC_SA1452AA	410	156
ESC_SA1451AA	95	738
ESC_SA1449AA	1819	808
ESC_SA1448AA	21	156
ESC_SA1447AA	11	936
ESC_SA1446AA	11	936
ESC_SA1445AA	11	936
ESC_SA1444AA	8630	257
ESC_SA1443AA	11	936
ESC_SA1442AA	17	191
ESC_SA1441AA	11	936
ESC_SA1440AA	442	257
ESC_SA1439AA	21	156
ESC_SA1438AA	442	257
ESC_SA1437AA	1819	808
ESC_SA1436AA	245	261
ESC_SA1435AA	8677	483
ESC_SA1434AA	162	924
ESC_SA1433AA	155	175
ESC_SA1432AA	457	595
ESC_SA1431AA	11316	645
ESC_SA1430AA	10	512
ESC_SA1429AA	10	512
ESC_SA1428AA	410	156
ESC_SA1427AA	410	156
ESC_SA1426AA	410	156
ESC_SA1424AA	515	1004
ESC_SA1423AA	10	512
ESC_SA1422AA	410	156
ESC_SA1421AA	38	669
ESC_SA1420AA	11	936
ESC_SA1419AA	11	936
ESC_SA1418AA	21	156
ESC_SA1417AA	21	156
ESC_SA1416AA	21	156
ESC_SA1415AA	29	156
ESC_SA1414AA	21	156
ESC_SA1413AA	8878	261
ESC_SA1412AA	1611	198
ESC_SA1411AA	11315	257
ESC_SA1409AA	11314	257

ESC_SA1408AA	245	261
ESC_SA1407AA	152	257
ESC_SA1406AA	245	261
ESC_SA1405AA	245	261
ESC_SA1404AA	7384	257
ESC_SA1403AA	245	261
ESC_SA1402AA	245	261
ESC_SA1400AA	245	261
ESC_SA1399AA	245	261
ESC_SA1398AA	245	261
ESC_SA1397AA	245	261
ESC_SA1396AA	33	198
ESC_SA1395AA	301	307
ESC_SA1394AA	8630	257
ESC_SA1393AA	10	512
ESC_SA1392AA	21	156
ESC_SA1391AA	11301	94
ESC_SA1389AA	11	936
ESC_SA1388AA	405	923
ESC_SA1387AA	11	936
ESC_SA1386AA	11	936
ESC_SA1385AA	11	936
ESC_SA1384AA	11	936
ESC_SA1383AA	11	936
ESC_SA1382AA	152	257
ESC_SA1381AA	152	257
ESC_SA1380AA	245	261
ESC_SA1379AA	245	261
ESC_SA1378AA	245	261
ESC_SA1377AA	245	261
ESC_SA1376AA	245	261
ESC_SA1375AA	442	257
ESC_SA1374AA	675	234
ESC_SA1373AA	1804	935
ESC_SA1372AA	11	936
ESC_SA1371AA	11	936
ESC_SA1370AA	11	936
ESC_SA1369AA	11	936
ESC_SA1368AA	11	936
ESC_SA1367AA	11	936
ESC_SA1365AA	648	359
ESC_SA1364AA	405	923
ESC_SA1363AA	10	512
ESC_SA1362AA	38	669
ESC_SA1361AA	38	669
ESC_SA1360AA	69	387
ESC_SA1359AA	2307	198
ESC_SA1358AA	2307	198
ESC_SA1357AA	11	936
ESC_SA1356AA	11	936

ESC_SA1355AA	11	936
ESC_SA1354AA	11	936
ESC_SA1353AA	11	936
ESC_SA1352AA	11	936
ESC_SA1351AA	11	936
ESC_SA1350AA	11	936
ESC_SA1349AA	11	936
ESC_SA1348AA	11	936
ESC_SA1347AA	11	936
ESC_SA1346AA	11	936
ESC_SA1345AA	11	936
ESC_SA1344AA	11	936
ESC_SA1343AA	11	936
ESC_SA1342AA	11	936
ESC_SA1341AA	11	936
ESC_SA1340AA	11	936
ESC_SA1339AA	11	936
ESC_SA1338AA	11	936
ESC_SA1337AA	11	936
ESC_SA1336AA	11	936
ESC_SA1335AA	11	936
ESC_SA1334AA	11	936
ESC_SA1333AA	9700	198
ESC_SA1332AA	295	160
ESC_SA1331AA	164	175
ESC_SA1330AA	58	198
ESC_SA1329AA	218	519
ESC_SA1328AA	10	512
ESC_SA1327AA	877	935
ESC_SA1326AA	1582	198
ESC_SA1325AA	117	592
ESC_SA1324AA	2526	257
ESC_SA1323AA	567	740
ESC_SA1322AA	189	504
ESC_SA1321AA	846	915
ESC_SA1320AA	224	198
ESC_SA1319AA	154	257
ESC_SA1318AA	206	286
ESC_SA1317AA	10	512
ESC_SA1316AA	316	257
ESC_SA1315AA	11	936
ESC_SA1314AA	11308	924
ESC_SA1313AA	11309	653
ESC_SA1312AA	11312	626
ESC_SA1311AA	11311	900
ESC_SA1310AA	11310	393
ESC_SA1309AA	245	261
ESC_SA1308AA	245	261
ESC_SA1307AA	245	261
ESC_SA1306AA	5475	256

ESC_SA1305AA	245	261
ESC_SA1304AA	33	198
ESC_SA1303AA	11	936
ESC_SA1302AA	3101	160
ESC_SA1301AA	752	512
ESC_SA1300AA	1819	808
ESC_SA1299AA	738	598
ESC_SA1298AA	12	626
ESC_SA1297AA	5254	765
ESC_SA1296AA	675	234
ESC_SA1295AA	11	936
ESC_SA1294AA	738	598
ESC_SA1293AA	2617	217
ESC_SA1292AA	21	156
ESC_SA1291AA	21	156
ESC_SA1290AA	11	936
ESC_SA1289AA	21	156
ESC_SA1288AA	410	156
ESC_SA1287AA	1236	380
ESC_SA1286AA	10	512
ESC_SA1285AA	602	257
ESC_SA1284AA	10	512
ESC_SA1283AA	10	512
ESC_SA1282AA	10	512
ESC_SA1281AA	10	512
ESC_SA1280AA	10	512
ESC_SA1279AA	10	512
ESC_SA1278AA	11313	512
ESC_SA1277AA	10	512
ESC_SA1276AA	10	512
ESC_SA1275AA	10	512
ESC_SA1274AA	10	512
ESC_SA1273AA	10	512
ESC_SA1272AA	10	512
ESC_SA1271AA	10	512
ESC_SA1270AA	10	512
ESC_SA1269AA	10	512
ESC_SA1268AA	10	512
ESC_SA1267AA	10	512
ESC_SA1266AA	10	512
ESC_SA1265AA	10	512
ESC_SA1264AA	10	512
ESC_SA1263AA	10	512
ESC_SA1262AA	10	512
ESC_SA1261AA	10	512
ESC_SA1260AA	10	512
ESC_SA1259AA	10	512
ESC_SA1258AA	10	512
ESC_SA1257AA	10	512
ESC_SA1256AA	10	512

ESC_SA1255AA	10	512
ESC_SA1254AA	10	512
ESC_SA1253AA	10	512
ESC_SA1252AA	10	512
ESC_SA1251AA	10	512
ESC_SA1250AA	10	512
ESC_SA1249AA	10	512
ESC_SA1248AA	10	512
ESC_SA1247AA	10	512
ESC_SA1246AA	10	512
ESC_SA1245AA	10	512
ESC_SA1244AA	10	512
ESC_SA1243AA	10	512
ESC_SA1242AA	10	512
ESC_SA1241AA	10	512
ESC_SA1240AA	10	512
ESC_SA1239AA	10	512
ESC_SA1238AA	10	512
ESC_SA1237AA	10	512
ESC_SA1236AA	10	512
ESC_SA1235AA	10	512
ESC_SA1234AA	10	512
ESC_SA1233AA	10	512
ESC_SA1232AA	10	512
ESC_SA1231AA	10	512
ESC_SA1230AA	10	512
ESC_SA1229AA	10	512
ESC_SA1228AA	10	512
ESC_SA1227AA	10	512
ESC_SA1226AA	10	512
ESC_SA1225AA	10	512
ESC_SA1224AA	10	512
ESC_SA1223AA	10	512
ESC_SA1222AA	10	512
ESC_SA1221AA	10	512
ESC_SA1220AA	10	512
ESC_SA1219AA	10	512
ESC_SA1218AA	10	512
ESC_SA1217AA	10	512
ESC_SA1216AA	10	512
ESC_SA1215AA	10	512
ESC_SA1214AA	10	512
ESC_SA1213AA	10	512
ESC_SA1212AA	10	512
ESC_SA1211AA	10	512
ESC_SA1210AA	10	512
ESC_SA1209AA	10	512
ESC_SA1208AA	10	512
ESC_SA1207AA	10	512
ESC_SA1206AA	10	512

ESC_SA1205AA	10	512
ESC_SA1204AA	10	512
ESC_SA1203AA	10	512
ESC_SA1202AA	10	512
ESC_SA1201AA	10	512
ESC_SA1200AA	10	512
ESC_SA1199AA	10	512
ESC_SA1198AA	10	512
ESC_SA1197AA	10	512
ESC_SA1196AA	10	512
ESC_SA1195AA	10	512
ESC_SA1194AA	10	512
ESC_SA1193AA	10	512
ESC_SA1192AA	10	512
ESC_SA1191AA	10	512
ESC_SA1190AA	10	512
ESC_SA1189AA	10	512
ESC_SA1188AA	10	512
ESC_SA1187AA	10	512
ESC_SA1186AA	10	512
ESC_SA1185AA	10	512
ESC_SA1184AA	10	512
ESC_SA1183AA	10	512
ESC_SA1182AA	10	512
ESC_SA1181AA	10	512
ESC_SA1180AA	10	512
ESC_SA1179AA	10	512
ESC_SA1178AA	10	512
ESC_SA1177AA	10	512
ESC_SA1176AA	10	512
ESC_SA1175AA	10	512
ESC_SA1174AA	10	512
ESC_SA1173AA	10	512
ESC_SA1172AA	10	512
ESC_SA1171AA	10	512
ESC_SA1170AA	10	512
ESC_SA1169AA	10	512
ESC_SA1168AA	10	512
ESC_SA1167AA	10	512
ESC_SA1166AA	10	512
ESC_SA1165AA	10	512
ESC_SA1164AA	10	512
ESC_SA1163AA	10	512
ESC_SA1162AA	10	512
ESC_SA1161AA	10	512
ESC_SA1160AA	10	512
ESC_SA1159AA	10	512
ESC_SA1158AA	10	512
ESC_SA1157AA	10	512
ESC_SA1156AA	10	512

ESC_SA1155AA	10	512
ESC_SA1154AA	10	512
ESC_SA1153AA	10	512
ESC_SA1152AA	10	512
ESC_SA1151AA	10	512
ESC_SA1150AA	10	512
ESC_SA1149AA	10	512
ESC_SA1148AA	10	512
ESC_SA1147AA	10	512
ESC_SA1146AA	10	512
ESC_SA1145AA	10	512
ESC_SA1144AA	10	512
ESC_SA1143AA	10	512
ESC_SA1142AA	10	512
ESC_SA1141AA	10	512
ESC_SA1140AA	10	512
ESC_SA1139AA	10	512
ESC_SA1138AA	10	512
ESC_SA1137AA	10	512
ESC_SA1136AA	10	512
ESC_SA1135AA	10	512
ESC_SA1134AA	10	512
ESC_SA1133AA	10	512
ESC_SA1132AA	10	512
ESC_SA1131AA	10	512
ESC_SA1130AA	10	512
ESC_SA1129AA	10	512
ESC_SA1128AA	10	512
ESC_SA1127AA	10	512
ESC_SA1126AA	10	512
ESC_SA1125AA	10	512
ESC_SA1124AA	10	512
ESC_SA1123AA	10	512
ESC_SA1122AA	10	512
ESC_SA1121AA	10	512
ESC_SA1120AA	10	512
ESC_SA1119AA	10	512
ESC_SA1118AA	10	512
ESC_SA1117AA	10	512
ESC_SA1116AA	10	512
ESC_SA1115AA	10	512
ESC_SA1114AA	10	512
ESC_SA1113AA	10	512
ESC_SA1112AA	10	512
ESC_SA1111AA	10	512
ESC_SA1110AA	10	512
ESC_SA1109AA	11	936
ESC_SA1108AA	11	936
ESC_SA1107AA	11	936
ESC_SA1106AA	11	936

ESC_SA1105AA	11	936
ESC_SA1104AA	11	936
ESC_SA1103AA	11	936
ESC_SA1102AA	11	936
ESC_SA1101AA	11	936
ESC_SA1100AA	1284	604
ESC_SA1099AA	1284	604
ESC_SA1098AA	167	512
ESC_SA1097AA	167	512
ESC_SA1096AA	152	257
ESC_SA1095AA	152	257
ESC_SA1094AA	245	261
ESC_SA1093AA	152	257
ESC_SA1092AA	152	257
ESC_SA1091AA	245	261
ESC_SA1090AA	245	261
ESC_SA1089AA	243	197
ESC_SA1088AA	11306	257
ESC_SA1087AA	11305	234
ESC_SA1086AA	11304	234
ESC_SA1085AA	11	936
ESC_SA1084AA	11	936
ESC_SA1083AA	301	307
ESC_SA1082AA	21	156
ESC_SA1081AA	21	156
ESC_SA1080AA	21	156
ESC_SA1079AA	21	156
ESC_SA1078AA	11303	355
ESC_SA1077AA	11303	355
ESC_SA1076AA	11303	355
ESC_SA1075AA	8123	738
ESC_SA1074AA	9023	888
ESC_SA1073AA	10	512
ESC_SA1072AA	10	512
ESC_SA1071AA	10	512
ESC_SA1068AA	48	76
ESC_SA1067AA	658	684
ESC_SA1066AA	301	307
ESC_SA1065AA	642	924
ESC_SA1064AA	448	257
ESC_SA1059AA	245	261
ESC_SA1058AA	245	261
ESC_SA1057AA	245	261
ESC_SA1056AA	245	261
ESC_SA1055AA	245	261
ESC_SA1054AA	21	156
ESC_SA1053AA	10	512
ESC_SA1052AA	11	936
ESC_SA1051AA	21	156
ESC_SA1050AA	152	257

ESC_SA1049AA	11302	261
ESC_SA1048AA	245	261
ESC_SA1047AA	17	191
ESC_SA1046AA	5133	504
ESC_SA1045AA	3570	156
ESC_SA1044AA	5133	504
ESC_SA1043AA	155	175
ESC_SA1042AA	3570	156
ESC_SA1041AA	7019	936
ESC_SA1040AA	11	936
ESC_SA1039AA	11	936
ESC_SA1038AA	11	936
ESC_SA1037AA	11	936
ESC_SA1036AA	11	936
ESC_SA1035AA	11	936
ESC_SA1032AA	301	307
ESC_SA1031AA	301	307
ESC_SA1030AA	21	156
ESC_SA1029AA	11296	504
ESC_SA1028AA	11297	445
ESC_SA1027AA	11294	504
ESC_SA1026AA	11300	790
ESC_SA1025AA	11299	936
ESC_SA1024AA	11301	94
ESC_SA1023AA	11292	789
ESC_SA1022AA	11298	512
ESC_SA1021AA	11295	696
ESC_SA1020AA	11291	94
ESC_SA1019AA	11290	512
ESC_SA1018AA	11293	204
ESC_SA1014AA	162	924
ESC_SA1010AA	11287	970
ESC_SA1009AA	11289	512
ESC_SA1008AA	11285	924
ESC_SA1007AA	11288	868
ESC_SA1006AA	11284	76
ESC_SA1005AA	11282	447
ESC_SA1004AA	11286	878
ESC_SA1003AA	2178	924
ESC_SA1002AA	48	76
ESC_SA1001AA	11283	315
ESC_SA1000AA	165	504
ESC_SA0999AA	11279	504
ESC_SA0998AA	48	76
ESC_SA0997AA	11281	519
ESC_SA0996AA	11280	311
ESC_SA0995AA	11278	512
ESC_SA0994AA	11277	447
ESC_SA0958AA	3933	318
ESC_SA0957AA	245	261

ESC_SA0956AA	547	653
ESC_SA0955AA	675	234
ESC_SA0954AA	28	537
ESC_SA0953AA	25	234
ESC_SA0952AA	162	924
ESC_SA0951AA	34	504
ESC_SA0950AA	11276	257
ESC_SA0949AA	11	936
ESC_SA0947AA	372	424
ESC_SA0946AA	2025	194
ESC_SA0945AA	372	424
ESC_SA0944AA	127	738
ESC_SA0943AA	12	626
ESC_SA0942AA	1065	250
ESC_SA0941AA	83	740
ESC_SA0940AA	372	424
ESC_SA0939AA	1170	629
ESC_SA0938AA	1718	269
ESC_SA0937AA	10752	370
ESC_SA0936AA	58	198
ESC_SA0935AA	11275	764
ESC_SA0934AA	145	256
ESC_SA0932AA	243	197
ESC_SA0931AA	245	261
ESC_SA0930AA	152	257
ESC_SA0929AA	6200	261
ESC_SA0928AA	245	261
ESC_SA0927AA	245	261
ESC_SA0918AA	21	156
ESC_SA0917AA	21	156
ESC_SA0916AA	33	198
ESC_SA0915AA	342	286
ESC_SA0914AA	21	156
ESC_SA0896AA	393	343
ESC_SA0895AA	11	936
ESC_SA0894AA	11	936
ESC_SA0892AA	11	936
ESC_SA0891AA	11	936
ESC_SA0890AA	11	936
ESC_SA0889AA	2325	447
ESC_SA0888AA	2325	447
ESC_SA0887AA	2325	447
ESC_SA0886AA	2325	447
ESC_SA0885AA	152	257
ESC_SA0884AA	152	257
ESC_SA0883AA	2325	447
ESC_SA0882AA	2325	447
ESC_SA0881AA	152	257
ESC_SA0880AA	152	257
ESC_SA0879AA	245	261

ESC_SA0878AA	245	261
ESC_SA0877AA	245	261
ESC_SA0876AA	152	257
ESC_SA0875AA	245	261
ESC_SA0874AA	245	261
ESC_SA0873AA	152	257
ESC_SA0872AA	152	257
ESC_SA0871AA	152	257
ESC_SA0870AA	245	261
ESC_SA0869AA	152	257
ESC_SA0868AA	245	261
ESC_SA0867AA	152	257
ESC_SA0866AA	245	261
ESC_SA0865AA	245	261
ESC_SA0864AA	2325	447
ESC_SA0863AA	2325	447
ESC_SA0862AA	2325	447
ESC_SA0861AA	2325	447
ESC_SA0859AA	93	71
ESC_SA0857AA	93	71
ESC_SA0856AA	11	936
ESC_SA0855AA	11	936
ESC_SA0854AA	11	936
ESC_SA0853AA	152	257
ESC_SA0852AA	152	257
ESC_SA0851AA	206	286
ESC_SA0850AA	647	734
ESC_SA0849AA	11268	198
ESC_SA0848AA	441	198
ESC_SA0843AA	647	734
ESC_SA0842AA	2217	62
ESC_SA0841AA	206	286
ESC_SA0840AA	8354	268
ESC_SA0839AA	718	257
ESC_SA0838AA	206	286
ESC_SA0837AA	718	257
ESC_SA0836AA	718	257
ESC_SA0835AA	718	257
ESC_SA0834AA	718	257
ESC_SA0833AA	1817	842
ESC_SA0832AA	332	257
ESC_SA0831AA	332	257
ESC_SA0830AA	206	286
ESC_SA0829AA	206	286
ESC_SA0828AA	718	257
ESC_SA0827AA	718	257
ESC_SA0826AA	718	257
ESC_SA0825AA	441	198
ESC_SA0824AA	8405	696
ESC_SA0823AA	206	286

ESC_SA0822AA	8405	696
ESC_SA0821AA	718	257
ESC_SA0820AA	8405	696
ESC_SA0819AA	1817	842
ESC_SA0818AA	1817	842
ESC_SA0817AA	8405	696
ESC_SA0816AA	647	734
ESC_SA0815AA	333	286
ESC_SA0814AA	718	257
ESC_SA0813AA	441	198
ESC_SA0812AA	441	198
ESC_SA0811AA	718	257
ESC_SA0810AA	441	198
ESC_SA0809AA	441	198
ESC_SA0808AA	718	257
ESC_SA0807AA	206	286
ESC_SA0806AA	718	257
ESC_SA0805AA	647	734
ESC_SA0804AA	647	734
ESC_SA0803AA	718	257
ESC_SA0802AA	8354	268
ESC_SA0801AA	206	286
ESC_SA0800AA	2715	588
ESC_SA0799AA	206	286
ESC_SA0798AA	206	286
ESC_SA0797AA	441	198
ESC_SA0796AA	718	257
ESC_SA0795AA	441	198
ESC_SA0794AA	718	257
ESC_SA0793AA	718	257
ESC_SA0792AA	441	198
ESC_SA0791AA	718	257
ESC_SA0790AA	8405	696
ESC_SA0789AA	8354	268
ESC_SA0788AA	306	286
ESC_SA0787AA	441	198
ESC_SA0786AA	206	286
ESC_SA0785AA	718	257
ESC_SA0784AA	206	286
ESC_SA0783AA	718	257
ESC_SA0782AA	718	257
ESC_SA0780AA	206	286
ESC_SA0779AA	441	198
ESC_SA0778AA	2715	588
ESC_SA0777AA	2715	588
ESC_SA0776AA	2715	588
ESC_SA0774AA	2715	588
ESC_SA0773AA	8354	268
ESC_SA0772AA	8354	268
ESC_SA0771AA	718	257

ESC_SA0770AA	8354	268
ESC_SA0769AA	8354	268
ESC_SA0768AA	7646	553
ESC_SA0767AA	2715	588
ESC_SA0766AA	718	257
ESC_SA0765AA	2715	588
ESC_SA0764AA	2385	198
ESC_SA0762AA	2715	588
ESC_SA0761AA	1817	842
ESC_SA0760AA	1817	842
ESC_SA0759AA	441	198
ESC_SA0758AA	718	257
ESC_SA0757AA	206	286
ESC_SA0756AA	718	257
ESC_SA0755AA	718	257
ESC_SA0754AA	718	257
ESC_SA0753AA	206	286
ESC_SA0752AA	718	257
ESC_SA0751AA	2715	588
ESC_SA0750AA	718	257
ESC_SA0749AA	8354	268
ESC_SA0748AA	8354	268
ESC_SA0747AA	718	257
ESC_SA0746AA	718	257
ESC_SA0745AA	647	734
ESC_SA0744AA	2715	588
ESC_SA0743AA	441	198
ESC_SA0742AA	2715	588
ESC_SA0741AA	441	198
ESC_SA0740AA	2715	588
ESC_SA0739AA	718	257
ESC_SA0738AA	333	286
ESC_SA0737AA	2715	588
ESC_SA0736AA	443	257
ESC_SA0735AA	718	257
ESC_SA0734AA	718	257
ESC_SA0733AA	206	286
ESC_SA0732AA	718	257
ESC_SA0731AA	8405	696
ESC_SA0730AA	718	257
ESC_SA0729AA	8405	696
ESC_SA0728AA	441	198
ESC_SA0727AA	718	257
ESC_SA0726AA	8405	696
ESC_SA0725AA	8405	696
ESC_SA0724AA	8405	696
ESC_SA0720AA	718	257
ESC_SA0719AA	441	198
ESC_SA0718AA	718	257
ESC_SA0717AA	206	286

ESC_SA0716AA	8354	268
ESC_SA0712AA	206	286
ESC_SA0711AA	2217	62
ESC_SA0710AA	206	286
ESC_SA0709AA	8405	696
ESC_SA0705AA	333	286
ESC_SA0702AA	441	198
ESC_SA0701AA	206	286
ESC_SA0699AA	2385	198
ESC_SA0695AA	718	257
ESC_SA0694AA	8354	268
ESC_SA0693AA	332	257
ESC_SA0692AA	8354	268
ESC_SA0691AA	1817	842
ESC_SA0690AA	1817	842
ESC_SA0689AA	1817	842
ESC_SA0688AA	647	734
ESC_SA0687AA	1817	842
ESC_SA0686AA	1817	842
ESC_SA0685AA	1817	842
ESC_SA0684AA	2715	588
ESC_SA0683AA	8354	268
ESC_SA0682AA	2715	588
ESC_SA0681AA	206	286
ESC_SA0680AA	206	286
ESC_SA0679AA	206	286
ESC_SA0678AA	8354	268
ESC_SA0677AA	718	257
ESC_SA0676AA	718	257
ESC_SA0675AA	329	355
ESC_SA0674AA	1817	842
ESC_SA0673AA	11269	257
ESC_SA0672AA	8354	268
ESC_SA0671AA	8354	268
ESC_SA0670AA	718	257
ESC_SA0669AA	441	198
ESC_SA0668AA	718	257
ESC_SA0667AA	333	286
ESC_SA0666AA	718	257
ESC_SA0665AA	718	257
ESC_SA0664AA	718	257
ESC_SA0663AA	333	286
ESC_SA0662AA	10	512
ESC_SA0661AA	206	286
ESC_SA0660AA	718	257
ESC_SA0659AA	192	696
ESC_SA0658AA	206	286
ESC_SA0657AA	718	257
ESC_SA0655AA	718	257
ESC_SA0653AA	333	286

ESC_SA0652AA	1794	204
ESC_SA0651AA	443	257
ESC_SA0650AA	8354	268
ESC_SA0649AA	206	286
ESC_SA0648AA	718	257
ESC_SA0647AA	718	257
ESC_SA0646AA	2715	588
ESC_SA0645AA	8354	268
ESC_SA0644AA	332	257
ESC_SA0643AA	8354	268
ESC_SA0642AA	718	257
ESC_SA0641AA	8354	268
ESC_SA0640AA	718	257
ESC_SA0639AA	718	257
ESC_SA0638AA	647	734
ESC_SA0637AA	8354	268
ESC_SA0636AA	2715	588
ESC_SA0635AA	8354	268
ESC_SA0634AA	333	286
ESC_SA0633AA	718	257
ESC_SA0632AA	8354	268
ESC_SA0631AA	718	257
ESC_SA0630AA	1817	842
ESC_SA0629AA	718	257
ESC_SA0628AA	206	286
ESC_SA0627AA	206	286
ESC_SA0626AA	1817	842
ESC_SA0625AA	718	257
ESC_SA0624AA	332	257
ESC_SA0623AA	718	257
ESC_SA0622AA	718	257
ESC_SA0621AA	3692	614
ESC_SA0620AA	8354	268
ESC_SA0619AA	718	257
ESC_SA0618AA	718	257
ESC_SA0617AA	718	257
ESC_SA0616AA	333	286
ESC_SA0615AA	206	286
ESC_SA0614AA	718	257
ESC_SA0613AA	332	257
ESC_SA0612AA	718	257
ESC_SA0611AA	332	257
ESC_SA0610AA	2715	588
ESC_SA0609AA	206	286
ESC_SA0608AA	206	286
ESC_SA0607AA	162	924
ESC_SA0606AA	718	257
ESC_SA0605AA	206	286
ESC_SA0604AA	718	257
ESC_SA0603AA	297	234

ESC_SA0602AA	718	257
ESC_SA0601AA	206	286
ESC_SA0600AA	332	257
ESC_SA0599AA	441	198
ESC_SA0598AA	718	257
ESC_SA0597AA	8354	268
ESC_SA0596AA	206	286
ESC_SA0595AA	206	286
ESC_SA0594AA	206	286
ESC_SA0593AA	8354	268
ESC_SA0592AA	8354	268
ESC_SA0591AA	333	286
ESC_SA0590AA	8354	268
ESC_SA0589AA	718	257
ESC_SA0588AA	8354	268
ESC_SA0587AA	21	156
ESC_SA0586AA	718	257
ESC_SA0585AA	718	257
ESC_SA0584AA	8354	268
ESC_SA0583AA	10	512
ESC_SA0582AA	718	257
ESC_SA0581AA	718	257
ESC_SA0580AA	718	257
ESC_SA0579AA	718	257
ESC_SA0578AA	718	257
ESC_SA0577AA	441	198
ESC_SA0576AA	441	198
ESC_SA0575AA	441	198
ESC_SA0574AA	441	198
ESC_SA0573AA	206	286
ESC_SA0572AA	206	286
ESC_SA0571AA	441	198
ESC_SA0570AA	441	198
ESC_SA0569AA	718	257
ESC_SA0568AA	718	257
ESC_SA0567AA	245	261
ESC_SA0565AA	245	261
ESC_SA0564AA	245	261
ESC_SA0563AA	245	261
ESC_SA0562AA	245	261
ESC_SA0561AA	469	257
ESC_SA0560AA	32	270
ESC_SA0559AA	765	156
ESC_SA0558AA	21	156
ESC_SA0557AA	442	257
ESC_SA0556AA	2346	435
ESC_SA0555AA	21	156
ESC_SA0554AA	25	234
ESC_SA0553AA	1819	808
ESC_SA0552AA	442	257

ESC_SA0551AA	21	156
ESC_SA0550AA	6274	257
ESC_SA0549AA	34	504
ESC_SA0548AA	25	234
ESC_SA0547AA	11	936
ESC_SA0545AA	354	15
ESC_SA0543AA	224	198
ESC_SA0540AA	155	175
ESC_SA0536AA	744	512
ESC_SA0533AA	162	924
ESC_SA0531AA	101	696
ESC_SA0530AA	162	924
ESC_SA0527AA	744	512
ESC_SA0526AA	23	156
ESC_SA0525AA	162	924
ESC_SA0524AA	1485	408
ESC_SA0523AA	744	512
ESC_SA0521AA	156	175
ESC_SA0519AA	90	156
ESC_SA0518AA	224	198
ESC_SA0517AA	5259	817
ESC_SA0514AA	359	696
ESC_SA0512AA	1196	257
ESC_SA0511AA	117	592
ESC_SA0509AA	156	175
ESC_SA0507AA	155	175
ESC_SA0504AA	602	257
ESC_SA0502AA	2847	212
ESC_SA0494AA	90	156
ESC_SA0493AA	1196	257
ESC_SA0492AA	706	422
ESC_SA0491AA	131	653
ESC_SA0490AA	224	198
ESC_SA0489AA	12	626
ESC_SA0488AA	297	234
ESC_SA0487AA	491	626
ESC_SA0486AA	131	653
ESC_SA0485AA	998	644
ESC_SA0484AA	9040	936
ESC_SA0483AA	9040	936
ESC_SA0482AA	11274	936
ESC_SA0481AA	7019	936
ESC_SA0480AA	7019	936
ESC_SA0479AA	11	936
ESC_SA0478AA	11	936
ESC_SA0477AA	11	936
ESC_SA0476AA	11	936
ESC_SA0475AA	11	936
ESC_SA0474AA	7019	936
ESC_SA0473AA	7019	936

ESC_SA0472AA	11	936
ESC_SA0471AA	11	936
ESC_SA0470AA	7019	936
ESC_SA0469AA	7019	936
ESC_SA0468AA	7019	936
ESC_SA0467AA	11	936
ESC_SA0466AA	1308	257
ESC_SA0465AA	1308	257
ESC_SA0464AA	1148	257
ESC_SA0463AA	1727	257
ESC_SA0462AA	1308	257
ESC_SA0461AA	56	198
ESC_SA0460AA	10	512
ESC_SA0459AA	154	257
ESC_SA0458AA	101	696
ESC_SA0457AA	108	257
ESC_SA0456AA	58	198
ESC_SA0455AA	11267	512
ESC_SA0454AA	11	936
ESC_SA0453AA	11	936
ESC_SA0452AA	11	936
ESC_SA0451AA	11	936
ESC_SA0450AA	1973	291
ESC_SA0449AA	1973	291
ESC_SA0447AA	1973	291
ESC_SA0446AA	1973	291
ESC_SA0445AA	973	968
ESC_SA0444AA	648	359
ESC_SA0443AA	648	359
ESC_SA0442AA	372	424
ESC_SA0441AA	646	738
ESC_SA0440AA	372	424
ESC_SA0439AA	2541	878
ESC_SA0437AA	245	261
ESC_SA0436AA	10	512
ESC_SA0435AA	301	307
ESC_SA0434AA	11266	307
ESC_SA0433AA	301	307
ESC_SA0432AA	301	307
ESC_SA0431AA	301	307
ESC_SA0430AA	301	307
ESC_SA0429AA	6570	425
ESC_SA0428AA	1946	726
ESC_SA0427AA	73	737
ESC_SA0426AA	372	424
ESC_SA0425AA	12	626
ESC_SA0424AA	11273	257
ESC_SA0423AA	127	738
ESC_SA0422AA	73	737
ESC_SA0421AA	95	738

ESC_SA0420AA	372	424
ESC_SA0419AA	5940	555
ESC_SA0418AA	11272	50
ESC_SA0417AA	117	592
ESC_SA0416AA	3476	512
ESC_SA0415AA	10	512
ESC_SA0414AA	345	175
ESC_SA0413AA	399	198
ESC_SA0412AA	6711	71
ESC_SA0411AA	569	738
ESC_SA0410AA	115	669
ESC_SA0409AA	636	653
ESC_SA0408AA	648	359
ESC_SA0407AA	372	424
ESC_SA0406AA	617	511
ESC_SA0405AA	362	234
ESC_SA0404AA	11271	695
ESC_SA0403AA	1946	726
ESC_SA0402AA	68	386
ESC_SA0401AA	1170	629
ESC_SA0400AA	131	653
ESC_SA0399AA	372	424
ESC_SA0398AA	4086	842
ESC_SA0397AA	6630	599
ESC_SA0396AA	362	234
ESC_SA0395AA	11270	141
ESC_SA0394AA	131	653
ESC_SA0393AA	357	654
ESC_SA0392AA	11264	738
ESC_SA0391AA	973	968
ESC_SA0390AA	12	626
ESC_SA0389AA	11265	740
ESC_SA0388AA	95	738
ESC_SA0387AA	1278	797
ESC_SA0386AA	131	653
ESC_SA0385AA	135	653
ESC_SA0384AA	963	669
ESC_SA0383AA	131	653
ESC_SA0382AA	95	738
ESC_SA0381AA	101	696
ESC_SA0380AA	1994	944
ESC_SA0379AA	2952	926
ESC_SA0378AA	131	653
ESC_SA0377AA	127	738
ESC_SA0376AA	69	387
ESC_SA0375AA	131	653
ESC_SA0374AA	131	653
ESC_SA0373AA	10	512
ESC_SA0372AA	95	738
ESC_SA0371AA	517	924

ESC_SA0370AA	12	626
ESC_SA0369AA	38	669
ESC_SA0368AA	131	653
ESC_SA0367AA	10	512
ESC_SA0366AA	131	653
ESC_SA0365AA	131	653
ESC_SA0364AA	12	626
ESC_SA0363AA	131	653
ESC_SA0362AA	69	387
ESC_SA0360AA	73	737
ESC_SA0359AA	69	387
ESC_SA0358AA	131	653
ESC_SA0357AA	127	738
ESC_SA0356AA	357	654
ESC_SA0355AA	131	653
ESC_SA0354AA	973	968
ESC_SA0353AA	73	737
ESC_SA0352AA	297	234
ESC_SA0351AA	131	653
ESC_SA0350AA	4260	737
ESC_SA0349AA	131	653
ESC_SA0348AA	421	744
ESC_SA0347AA	847	257
ESC_SA0346AA	5602	900
ESC_SA0345AA	95	738
ESC_SA0344AA	372	424
ESC_SA0343AA	714	318
ESC_SA0342AA	2015	684
ESC_SA0341AA	12	626
ESC_SA0340AA	4118	924
ESC_SA0339AA	457	595
ESC_SA0338AA	372	424
ESC_SA0337AA	6009	847
ESC_SA0336AA	372	424
ESC_SA0335AA	646	738
ESC_SA0334AA	491	626
ESC_SA0333AA	2622	537
ESC_SA0332AA	963	669
ESC_SA0331AA	38	669
ESC_SA0329AA	73	737
ESC_SA0328AA	11262	560
ESC_SA0327AA	95	738
ESC_SA0326AA	11263	424
ESC_SA0325AA	11261	738
ESC_SA0324AA	646	738
ESC_SA0323AA	372	424
ESC_SA0322AA	372	424
ESC_SA0321AA	372	424
ESC_SA0320AA	189	504
ESC_SA0319AA	189	504

ESC_SA0318AA	189	504
ESC_SA0317AA	165	504
ESC_SA0316AA	11260	359
ESC_SA0315AA	301	307
ESC_SA0314AA	301	307
ESC_SA0313AA	301	307
ESC_SA0312AA	11	936
ESC_SA0311AA	29	156
ESC_SA0310AA	11	936
ESC_SA0309AA	10	512
ESC_SA0308AA	11	936
ESC_SA0307AA	11	936
ESC_SA0306AA	655	926
ESC_SA0305AA	33	198
ESC_SA0304AA	11	936
ESC_SA0303AA	21	156
ESC_SA0301AA	399	198
ESC_SA0300AA	399	198
ESC_SA0299AA	399	198
ESC_SA0298AA	399	198
ESC_SA0297AA	399	198
ESC_SA0296AA	399	198
ESC_SA0295AA	399	198
ESC_SA0294AA	399	198
ESC_SA0293AA	399	198
ESC_SA0292AA	399	198
ESC_SA0291AA	399	198
ESC_SA0290AA	399	198
ESC_SA0289AA	399	198
ESC_SA0288AA	404	761
ESC_SA0287AA	69	387
ESC_SA0286AA	399	198
ESC_SA0285AA	10238	257
ESC_SA0284AA	399	198
ESC_SA0283AA	399	198
ESC_SA0282AA	399	198
ESC_SA0281AA	399	198
ESC_SA0280AA	399	198
ESC_SA0279AA	399	198
ESC_SA0278AA	399	198
ESC_SA0277AA	3168	261
ESC_SA0276AA	399	198
ESC_SA0275AA	399	198
ESC_SA0274AA	399	198
ESC_SA0273AA	399	198
ESC_SA0272AA	349	670
ESC_SA0271AA	399	198
ESC_SA0270AA	399	198
ESC_SA0269AA	10	512
ESC_SA0268AA	399	198

ESC_SA0267AA	11	936
ESC_SA0266AA	11258	653
ESC_SA0264AA	11257	175
ESC_SA0263AA	11259	642
ESC_SA0262AA	372	424
ESC_SA0261AA	372	424
ESC_SA0260AA	372	424
ESC_SA0259AA	372	424
ESC_SA0258AA	12	626
ESC_SA0257AA	1844	198
ESC_SA0256AA	219	877
ESC_SA0255AA	8492	234
ESC_SA0254AA	372	424
ESC_SA0253AA	68	386
ESC_SA0252AA	372	424
ESC_SA0251AA	998	644
ESC_SA0250AA	12	626
ESC_SA0249AA	2622	537
ESC_SA0248AA	4392	234
ESC_SA0247AA	135	653
ESC_SA0246AA	131	653
ESC_SA0245AA	11256	424
ESC_SA0244AA	1735	737
ESC_SA0243AA	2622	537
ESC_SA0242AA	372	424
ESC_SA0241AA	372	424
ESC_SA0240AA	681	393
ESC_SA0239AA	141	644
ESC_SA0238AA	245	261
ESC_SA0237AA	152	257
ESC_SA0236AA	152	257
ESC_SA0235AA	152	257
ESC_SA0234AA	152	257
ESC_SA0233AA	152	257
ESC_SA0232AA	8001	465
ESC_SA0231AA	152	257
ESC_SA0230AA	152	257
ESC_SA0229AA	1431	257
ESC_SA0228AA	152	257
ESC_SA0226AA	583	768
ESC_SA0225AA	491	626
ESC_SA0224AA	152	257
ESC_SA0223AA	8878	261
ESC_SA0222AA	152	257
ESC_SA0221AA	21	156
ESC_SA0220AA	21	156
ESC_SA0219AA	21	156
ESC_SA0218AA	21	156
ESC_SA0217AA	43	512
ESC_SA0216AA	301	307

ESC_SA0215AA	21	156
ESC_SA0214AA	131	653
ESC_SA0213AA	515	1004
ESC_SA0211AA	5940	555
ESC_SA0210AA	1286	474
ESC_SA0209AA	10	512
ESC_SA0208AA	10	512
ESC_SA0207AA	10	512
ESC_SA0206AA	10	512
ESC_SA0205AA	2280	212
ESC_SA0204AA	1851	380
ESC_SA0203AA	711	935
ESC_SA0202AA	154	257
ESC_SA0201AA	155	175
ESC_SA0200AA	191	198
ESC_SA0199AA	11348	234
ESC_SA0198AA	2520	878
ESC_SA0197AA	11383	336
ESC_SA0196AA	1159	381
ESC_SA0195AA	68	386
ESC_SA0194AA	1611	198
ESC_SA0193AA	141	644
ESC_SA0192AA	10	512
ESC_SA0191AA	86	935
ESC_SA0190AA	372	424
ESC_SA0189AA	167	512
ESC_SA0188AA	410	156
ESC_SA0187AA	410	156
ESC_SA0186AA	410	156
ESC_SA0185AA	9919	942
ESC_SA0184AA	83	740
ESC_SA0183AA	152	257
ESC_SA0182AA	245	261
ESC_SA0181AA	245	261
ESC_SA0180AA	152	257
ESC_SA0179AA	630	261
ESC_SA0178AA	152	257
ESC_SA0177AA	245	261
ESC_SA0176AA	11236	261
ESC_SA0175AA	245	261
ESC_SA0174AA	245	261
ESC_SA0173AA	245	261
ESC_SA0172AA	245	261
ESC_SA0171AA	152	257
ESC_SA0170AA	10	512
ESC_SA0169AA	11	936
ESC_SA0168AA	11	936
ESC_SA0167AA	328	924
ESC_SA0166AA	11	936
ESC_SA0165AA	73	737

ESC_SA0164AA	11	936
ESC_SA0163AA	11	936
ESC_SA0162AA	11	936
ESC_SA0161AA	11	936
ESC_SA0160AA	2144	257
ESC_SA0159AA	11	936
ESC_SA0158AA	10334	935
ESC_SA0157AA	25	234
ESC_SA0156AA	738	598
ESC_SA0155AA	11	936
ESC_SA0154AA	11	936
ESC_SA0153AA	11	936
ESC_SA0152AA	11	936
ESC_SA0151AA	11	936
ESC_SA0150AA	11	936
ESC_SA0149AA	10	512
ESC_SA0148AA	442	257
ESC_SA0147AA	11	936
ESC_SA0146AA	11	936
ESC_SA0145AA	1817	842
ESC_SA0144AA	11	936
ESC_SA0143AA	11	936
ESC_SA0142AA	5292	738
ESC_SA0141AA	16	156
ESC_SA0140AA	16	156
ESC_SA0139AA	297	234
ESC_SA0138AA	297	234
ESC_SA0137AA	297	234
ESC_SA0136AA	297	234
ESC_SA0135AA	297	234
ESC_SA0134AA	297	234
ESC_SA0133AA	297	234
ESC_SA0132AA	297	234
ESC_SA0131AA	297	234
ESC_SA0130AA	297	234
ESC_SA0129AA	16	156
ESC_SA0128AA	224	198
ESC_SA0127AA	162	924
ESC_SA0126AA	162	924
ESC_SA0125AA	297	234
ESC_SA0124AA	16	156
ESC_SA0123AA	16	156
ESC_SA0122AA	297	234
ESC_SA0121AA	297	234
ESC_SA0120AA	297	234
ESC_SA0119AA	297	234
ESC_SA0118AA	297	234
ESC_SA0117AA	1722	599
ESC_SA0116AA	167	512
ESC_SA0115AA	69	387

ESC_SA0114AA	617	511
ESC_SA0113AA	410	156
ESC_SA0112AA	43	512
ESC_SA0111AA	156	175
ESC_SA0110AA	95	738
ESC_SA0109AA	410	156
ESC_SA0108AA	131	653
ESC_SA0107AA	448	257
ESC_SA0106AA	448	257
ESC_SA0105AA	448	257
ESC_SA0104AA	448	257
ESC_SA0103AA	448	257
ESC_SA0102AA	245	261
ESC_SA0101AA	245	261
ESC_SA0100AA	342	286
ESC_SA0099AA	11	936
ESC_SA0098AA	16	156
ESC_SA0097AA	723	156
ESC_SA0094AA	11	936
ESC_SA0093AA	746	789
ESC_SA0092AA	93	71
ESC_SA0091AA	93	71
ESC_SA0090AA	93	71
ESC_SA0089AA	93	71
ESC_SA0088AA	93	71
ESC_SA0087AA	93	71
ESC_SA0086AA	93	71
ESC_SA0085AA	93	71
ESC_SA0084AA	93	71
ESC_SA0083AA	93	71
ESC_SA0082AA	93	71
ESC_SA0081AA	93	71
ESC_SA0080AA	93	71
ESC_SA0079AA	93	71
ESC_SA0078AA	93	71
ESC_SA0077AA	93	71
ESC_SA0076AA	93	71
ESC_SA0075AA	93	71
ESC_SA0074AA	93	71
ESC_SA0073AA	93	71
ESC_SA0072AA	93	71
ESC_SA0071AA	93	71
ESC_SA0070AA	93	71
ESC_SA0069AA	93	71
ESC_SA0068AA	93	71
ESC_SA0067AA	93	71
ESC_SA0066AA	93	71
ESC_SA0065AA	93	71
ESC_SA0064AA	93	71
ESC_SA0063AA	93	71

ESC_SA0062AA	93	71
ESC_SA0061AA	93	71
ESC_SA0060AA	93	71
ESC_SA0059AA	93	71
ESC_SA0058AA	93	71
ESC_SA0057AA	93	71
ESC_SA0056AA	93	71
ESC_SA0055AA	93	71
ESC_SA0054AA	93	71
ESC_SA0053AA	93	71
ESC_SA0052AA	93	71
ESC_SA0051AA	93	71
ESC_SA0050AA	93	71
ESC_SA0049AA	93	71
ESC_SA0048AA	93	71
ESC_SA0047AA	93	71
ESC_SA0046AA	93	71
ESC_SA0045AA	93	71
ESC_SA0044AA	93	71
ESC_SA0043AA	93	71
ESC_SA0042AA	93	71
ESC_SA0041AA	93	71
ESC_SA0040AA	93	71
ESC_SA0039AA	93	71
ESC_SA0038AA	93	71
ESC_SA0037AA	93	71
ESC_SA0036AA	93	71
ESC_SA0035AA	93	71
ESC_SA0034AA	93	71
ESC_SA0033AA	93	71
ESC_SA0032AA	93	71
ESC_SA0031AA	93	71
ESC_SA0030AA	93	71
ESC_SA0029AA	93	71
ESC_SA0028AA	10115	481
ESC_SA0027AA	10115	481
ESC_SA0026AA	11255	506
ESC_SA0025AA	716	795
ESC_SA0024AA	11171	504
ESC_SA0023AA	11254	198
ESC_SA0022AA	290	662
ESC_SA0021AA	7364	191
ESC_SA0020AA	243	197
ESC_SA0019AA	273	102
ESC_SA0018AA	88	160
ESC_SA0017AA	10	512
ESC_SA0016AA	131	653
ESC_SA0015AA	10	512
ESC_SA0014AA	131	653
ESC_SA0013AA	88	160

ESC_SA0012AA	101	696
ESC_SA0011AA	131	653
ESC_SA0010AA	131	653
ESC_SA0009AA	131	653
ESC_SA0008AA	2599	257
ESC_SA0007AA	131	653
ESC_SA0006AA	131	653
ESC_SA0005AA	10	512
ESC_SA0004AA	131	653
ESC_SA0003AA	131	653
ESC_SA0002AA	131	653
ESC_SA0001AA	131	653
ESC_SA0000AA	131	653
ESC_RA9999AA	410	156
ESC_RA9998AA	131	653
ESC_RA9997AA	131	653
ESC_RA9996AA	1585	512
ESC_RA9995AA	117	592
ESC_RA9994AA	1585	512
ESC_RA9993AA	3171	515
ESC_RA9992AA	131	653
ESC_RA9991AA	1585	512
ESC_RA9990AA	1193	761
ESC_RA9989AA	131	653
ESC_RA9988AA	131	653
ESC_RA9987AA	131	653
ESC_RA9986AA	69	387
ESC_RA9985AA	131	653
ESC_RA9984AA	131	653
ESC_RA9983AA	131	653
ESC_RA9982AA	1193	761
ESC_RA9981AA	131	653
ESC_RA9980AA	131	653
ESC_RA9979AA	11253	653
ESC_RA9978AA	131	653
ESC_RA9977AA	443	257
ESC_RA9976AA	38	669
ESC_RA9975AA	131	653
ESC_RA9974AA	131	653
ESC_RA9973AA	75	257
ESC_RA9972AA	131	653
ESC_RA9971AA	648	359
ESC_RA9970AA	131	653
ESC_RA9969AA	131	653
ESC_RA9968AA	131	653
ESC_RA9967AA	162	924
ESC_RA9966AA	131	653
ESC_RA9965AA	131	653
ESC_RA9964AA	131	653
ESC_RA9963AA	131	653

ESC_RA9962AA	131	653
ESC_RA9961AA	131	653
ESC_RA9960AA	131	653
ESC_RA9959AA	131	653
ESC_RA9958AA	410	156
ESC_RA9957AA	131	653
ESC_RA9956AA	131	653
ESC_RA9955AA	131	653
ESC_RA9954AA	131	653
ESC_RA9953AA	131	653
ESC_RA9952AA	131	653
ESC_RA9951AA	131	653
ESC_RA9950AA	131	653
ESC_RA9949AA	354	15
ESC_RA9948AA	131	653
ESC_RA9947AA	131	653
ESC_RA9946AA	1642	198
ESC_RA9945AA	131	653
ESC_RA9944AA	88	160
ESC_RA9943AA	744	512
ESC_RA9942AA	131	653
ESC_RA9941AA	88	160
ESC_RA9940AA	131	653
ESC_RA9939AA	131	653
ESC_RA9938AA	648	359
ESC_RA9937AA	131	653
ESC_RA9936AA	4981	473
ESC_RA9935AA	131	653
ESC_RA9934AA	10	512
ESC_RA9933AA	648	359
ESC_RA9932AA	131	653
ESC_RA9931AA	405	923
ESC_RA9930AA	131	653
ESC_RA9929AA	131	653
ESC_RA9928AA	10	512
ESC_RA9927AA	131	653
ESC_RA9926AA	131	653
ESC_RA9925AA	131	653
ESC_RA9924AA	131	653
ESC_RA9923AA	131	653
ESC_RA9922AA	131	653
ESC_RA9921AA	648	359
ESC_RA9920AA	131	653
ESC_RA9919AA	131	653
ESC_RA9918AA	131	653
ESC_RA9917AA	10405	653
ESC_RA9916AA	3171	515
ESC_RA9915AA	131	653
ESC_RA9914AA	1193	761
ESC_RA9913AA	354	15

ESC_RA9912AA	131	653
ESC_RA9911AA	131	653
ESC_RA9910AA	405	923
ESC_RA9909AA	131	653
ESC_RA9908AA	131	653
ESC_RA9907AA	131	653
ESC_RA9906AA	131	653
ESC_RA9905AA	131	653
ESC_RA9904AA	428	654
ESC_RA9902AA	131	653
ESC_RA9901AA	131	653
ESC_RA9900AA	131	653
ESC_RA9899AA	131	653
ESC_RA9898AA	10	512
ESC_RA9897AA	88	160
ESC_RA9896AA	354	15
ESC_RA9895AA	3052	669
ESC_RA9894AA	131	653
ESC_RA9893AA	131	653
ESC_RA9892AA	131	653
ESC_RA9891AA	744	512
ESC_RA9890AA	131	653
ESC_RA9889AA	131	653
ESC_RA9888AA	224	198
ESC_RA9887AA	1642	198
ESC_RA9886AA	131	653
ESC_RA9885AA	131	653
ESC_RA9884AA	131	653
ESC_RA9883AA	131	653
ESC_RA9882AA	1193	761
ESC_RA9881AA	131	653
ESC_RA9880AA	131	653
ESC_RA9879AA	131	653
ESC_RA9878AA	949	175
ESC_RA9877AA	131	653
ESC_RA9876AA	1642	198
ESC_RA9875AA	131	653
ESC_RA9874AA	131	653
ESC_RA9873AA	617	511
ESC_RA9872AA	131	653
ESC_RA9871AA	1193	761
ESC_RA9870AA	410	156
ESC_RA9869AA	131	653
ESC_RA9868AA	131	653
ESC_RA9867AA	131	653
ESC_RA9866AA	131	653
ESC_RA9865AA	656	789
ESC_RA9864AA	648	359
ESC_RA9863AA	38	669
ESC_RA9862AA	10	512

ESC_RA9861AA	131	653
ESC_RA9860AA	141	644
ESC_RA9859AA	131	653
ESC_RA9858AA	1193	761
ESC_RA9857AA	131	653
ESC_RA9856AA	152	257
ESC_RA9855AA	10769	257
ESC_RA9854AA	152	257
ESC_RA9853AA	245	261
ESC_RA9852AA	131	653
ESC_RA9851AA	7384	257
ESC_RA9850AA	630	261
ESC_RA9849AA	245	261
ESC_RA9848AA	152	257
ESC_RA9847AA	131	653
ESC_RA9846AA	245	261
ESC_RA9845AA	1504	254
ESC_RA9844AA	1598	447
ESC_RA9843AA	245	261
ESC_RA9842AA	131	653
ESC_RA9841AA	152	257
ESC_RA9840AA	245	261
ESC_RA9839AA	245	261
ESC_RA9838AA	1193	761
ESC_RA9837AA	131	653
ESC_RA9836AA	131	653
ESC_RA9835AA	1193	761
ESC_RA9834AA	224	198
ESC_RA9833AA	131	653
ESC_RA9832AA	131	653
ESC_RA9831AA	88	160
ESC_RA9830AA	131	653
ESC_RA9829AA	131	653
ESC_RA9828AA	69	387
ESC_RA9827AA	131	653
ESC_RA9826AA	88	160
ESC_RA9825AA	744	512
ESC_RA9824AA	744	512
ESC_RA9823AA	162	924
ESC_RA9822AA	131	653
ESC_RA9821AA	131	653
ESC_RA9820AA	131	653
ESC_RA9819AA	1193	761
ESC_RA9818AA	69	387
ESC_RA9817AA	131	653
ESC_RA9816AA	405	923
ESC_RA9815AA	131	653
ESC_RA9814AA	131	653
ESC_RA9813AA	405	923
ESC_RA9812AA	69	387

ESC_RA9811AA	69	387
ESC_RA9810AA	648	359
ESC_RA9809AA	648	359
ESC_RA9808AA	131	653
ESC_RA9807AA	131	653
ESC_RA9806AA	1193	761
ESC_RA9805AA	131	653
ESC_RA9804AA	88	160
ESC_RA9803AA	636	653
ESC_RA9802AA	131	653
ESC_RA9801AA	2599	257
ESC_RA9800AA	131	653
ESC_RA9799AA	131	653
ESC_RA9798AA	131	653
ESC_RA9797AA	2599	257
ESC_RA9796AA	648	359
ESC_RA9795AA	744	512
ESC_RA9794AA	224	198
ESC_RA9793AA	88	160
ESC_RA9792AA	131	653
ESC_RA9791AA	131	653
ESC_RA9790AA	131	653
ESC_RA9789AA	131	653
ESC_RA9788AA	131	653
ESC_RA9787AA	131	653
ESC_RA9786AA	1193	761
ESC_RA9785AA	131	653
ESC_RA9784AA	10	512
ESC_RA9783AA	131	653
ESC_RA9782AA	131	653
ESC_RA9781AA	131	653
ESC_RA9780AA	131	653
ESC_RA9779AA	167	512
ESC_RA9778AA	131	653
ESC_RA9777AA	131	653
ESC_RA9776AA	1193	761
ESC_RA9775AA	131	653
ESC_RA9774AA	131	653
ESC_RA9773AA	131	653
ESC_RA9772AA	131	653
ESC_RA9771AA	410	156
ESC_RA9770AA	131	653
ESC_RA9769AA	11	936
ESC_RA9768AA	29	156
ESC_RA9767AA	21	156
ESC_RA9766AA	152	257
ESC_RA9765AA	245	261
ESC_RA9764AA	245	261
ESC_RA9762AA	11	936
ESC_RA9761AA	1817	842

ESC_RA9760AA	29	156
ESC_RA9759AA	343	286
ESC_RA9758AA	119	286
ESC_RA9757AA	29	156
ESC_RA9756AA	25	234
ESC_RA9755AA	12	626
ESC_RA9754AA	300	286
ESC_RA9753AA	29	156
ESC_RA9752AA	306	286
ESC_RA9751AA	11	936
ESC_RA9750AA	11	936
ESC_RA9749AA	11	936
ESC_RA9748AA	11	936
ESC_RA9747AA	11	936
ESC_RA9746AA	11	936
ESC_RA9745AA	11	936
ESC_RA9744AA	11	936
ESC_RA9743AA	11	936
ESC_RA9742AA	11	936
ESC_RA9741AA	162	924
ESC_RA9740AA	11	936
ESC_RA9739AA	11	936
ESC_RA9738AA	245	261
ESC_RA9737AA	11	936
ESC_RA9736AA	11	936
ESC_RA9735AA	947	257
ESC_RA9734AA	17	191
ESC_RA9733AA	354	15
ESC_RA9732AA	162	924
ESC_RA9731AA	1196	257
ESC_RA9730AA	457	595
ESC_RA9729AA	457	595
ESC_RA9171AA	354	15
ESC_RA9169AA	29	156
ESC_RA9168AA	21	156
ESC_RA9167AA	21	156
ESC_RA9166AA	21	156
ESC_RA9165AA	11249	653
ESC_RA9164AA	11248	142
ESC_RA9163AA	1967	191
ESC_RA9162AA	21	156
ESC_RA9161AA	602	257
ESC_RA9160AA	602	257
ESC_RA9159AA	602	257
ESC_RA9158AA	602	257
ESC_RA9157AA	3032	912
ESC_RA9156AA	457	595
ESC_RA9155AA	38	669
ESC_RA9154AA	533	198
ESC_RA9153AA	10	512

ESC_RA9152AA	10	512
ESC_RA9151AA	394	400
ESC_RA9150AA	648	359
ESC_RA9149AA	38	669
ESC_RA9148AA	1193	761
ESC_RA9147AA	1193	761
ESC_RA9146AA	131	653
ESC_RA9145AA	11252	669
ESC_RA9144AA	131	653
ESC_RA9143AA	38	669
ESC_RA9142AA	744	512
ESC_RA9141AA	131	653
ESC_RA9140AA	1148	257
ESC_RA9139AA	131	653
ESC_RA9138AA	2003	669
ESC_RA9137AA	11251	673
ESC_RA9136AA	131	653
ESC_RA9135AA	405	923
ESC_RA9134AA	70	670
ESC_RA9133AA	3941	71
ESC_RA9132AA	648	359
ESC_RA9131AA	162	924
ESC_RA9130AA	131	653
ESC_RA9129AA	1049	175
ESC_RA9128AA	131	653
ESC_RA9127AA	10	512
ESC_RA9126AA	58	198
ESC_RA9125AA	131	653
ESC_RA9124AA	44	512
ESC_RA9123AA	2025	194
ESC_RA9122AA	38	669
ESC_RA9121AA	3177	362
ESC_RA9120AA	131	653
ESC_RA9119AA	10	512
ESC_RA9118AA	155	175
ESC_RA9117AA	648	359
ESC_RA9116AA	131	653
ESC_RA9115AA	69	387
ESC_RA9114AA	10	512
ESC_RA9113AA	405	923
ESC_RA9112AA	405	923
ESC_RA9111AA	131	653
ESC_RA9110AA	641	935
ESC_RA9109AA	38	669
ESC_RA9108AA	69	387
ESC_RA9107AA	542	824
ESC_RA9106AA	542	824
ESC_RA9105AA	2858	400
ESC_RA9103AA	38	669
ESC_RA9102AA	38	669

ESC_RA9101AA	38	669
ESC_RA9100AA	602	257
ESC_RA9099AA	69	387
ESC_RA9098AA	38	669
ESC_RA9097AA	38	669
ESC_RA9096AA	130	387
ESC_RA9095AA	130	387
ESC_RA9094AA	847	257
ESC_RA9093AA	205	257
ESC_RA9092AA	130	387
ESC_RA9091AA	93	71
ESC_RA9090AA	1967	191
ESC_RA9089AA	58	198
ESC_RA9088AA	8608	512
ESC_RA9068AA	676	737
ESC_RA9067AA	602	257
ESC_RA9066AA	48	76
ESC_RA9065AA	11240	738
ESC_RA9064AA	11239	76
ESC_RA9051AA	2659	679
ESC_RA9050AA	167	512
ESC_RA9049AA	10	512
ESC_RA9048AA	2079	198
ESC_RA9047AA	1722	599
ESC_RA9046AA	998	644
ESC_RA9045AA	961	623
ESC_RA9044AA	141	644
ESC_RA9043AA	961	623
ESC_RA9042AA	372	424
ESC_RA9041AA	11250	740
ESC_RA9040AA	141	644
ESC_RA9039AA	11	936
ESC_RA9038AA	617	511
ESC_RA9037AA	2659	679
ESC_RA9034AA	675	234
ESC_RA9030AA	3695	924
ESC_RA9029AA	3695	924
ESC_RA9028AA	297	234
ESC_RA9027AA	33	198
ESC_RA9026AA	675	234
ESC_RA9024AA	675	234
ESC_RA9023AA	675	234
ESC_RA9022AA	3695	924
ESC_RA9021AA	3695	924
ESC_RA9017AA	657	592
ESC_RA9015AA	11238	592
ESC_RA9014AA	1125	198
ESC_RA9013AA	1125	198
ESC_RA9012AA	657	592
ESC_RA9006AA	443	257

ESC_RA9004AA	446	257
ESC_RA9002AA	297	234
ESC_RA8995AA	297	234
ESC_RA8985AA	1248	156
ESC_RA8984AA	448	257
ESC_RA8983AA	448	257
ESC_RA8982AA	332	257
ESC_RA8981AA	448	257
ESC_RA8979AA	33	198
ESC_RA8977AA	33	198
ESC_RA8976AA	692	234
ESC_RA8974AA	332	257
ESC_RA8972AA	1125	198
ESC_RA8966AA	245	261
ESC_RA8965AA	245	261
ESC_RA8964AA	145	256
ESC_RA8963AA	152	257
ESC_RA8962AA	152	257
ESC_RA8961AA	152	257
ESC_RA8960AA	152	257
ESC_RA8959AA	245	261
ESC_RA8958AA	245	261
ESC_RA8957AA	152	257
ESC_RA8956AA	8305	235
ESC_RA8955AA	648	359
ESC_RA8954AA	38	669
ESC_RA8953AA	152	257
ESC_RA8952AA	906	198
ESC_RA8951AA	88	160
ESC_RA8950AA	1092	739
ESC_RA8949AA	11	936
ESC_RA8948AA	17	191
ESC_RA8947AA	29	156
ESC_RA8946AA	21	156
ESC_RA8945AA	21	156
ESC_RA8944AA	442	257
ESC_RA8943AA	25	234
ESC_RA8942AA	29	156
ESC_RA8941AA	1092	739
ESC_RA8940AA	11	936
ESC_RA8939AA	155	175
ESC_RA8938AA	21	156
ESC_RA8937AA	11	936
ESC_RA8936AA	11	936
ESC_RA8935AA	1193	761
ESC_RA8934AA	442	257
ESC_RA8933AA	21	156
ESC_RA8932AA	718	257
ESC_RA8931AA	29	156
ESC_RA8930AA	21	156

ESC_RA8929AA	245	261
ESC_RA8928AA	245	261
ESC_RA8927AA	152	257
ESC_RA8926AA	152	257
ESC_RA8925AA	245	261
ESC_RA8924AA	245	261
ESC_RA8923AA	152	257
ESC_RA8922AA	216	512
ESC_RA8921AA	216	512
ESC_RA8920AA	401	142
ESC_RA8919AA	95	738
ESC_RA8918AA	11	936
ESC_RA8917AA	11	936
ESC_RA8916AA	11	936
ESC_RA8915AA	216	512
ESC_RA8914AA	88	160
ESC_RA8913AA	38	669
ESC_RA8911AA	38	669
ESC_RA8910AA	648	359
ESC_RA8909AA	10334	935
ESC_RA8908AA	11	936
ESC_RA8907AA	10334	935
ESC_RA8906AA	11	936
ESC_RA8905AA	11	936
ESC_RA8904AA	10	512
ESC_RA8903AA	10	512
ESC_RA8902AA	10	512
ESC_RA8901AA	10	512
ESC_RA8900AA	10	512
ESC_RA8899AA	10	512
ESC_RA8898AA	10	512
ESC_RA8897AA	10	512
ESC_RA8896AA	10	512
ESC_RA8895AA	10	512
ESC_RA8894AA	10	512
ESC_RA8893AA	10	512
ESC_RA8892AA	10	512
ESC_RA8891AA	131	653
ESC_RA8890AA	131	653
ESC_RA8889AA	131	653
ESC_RA8888AA	131	653
ESC_RA8887AA	131	653
ESC_RA8886AA	131	653
ESC_RA8885AA	131	653
ESC_RA8884AA	131	653
ESC_RA8883AA	131	653
ESC_RA8882AA	131	653
ESC_RA8881AA	131	653
ESC_RA8880AA	131	653
ESC_RA8879AA	405	923

ESC_RA8878AA	405	923
ESC_RA8877AA	6726	493
ESC_RA8876AA	998	644
ESC_RA8875AA	127	738
ESC_RA8874AA	48	76
ESC_RA8873AA	1193	761
ESC_RA8872AA	410	156
ESC_RA8871AA	95	738
ESC_RA8870AA	1193	761
ESC_RA8869AA	9748	653
ESC_RA8868AA	95	738
ESC_RA8867AA	1193	761
ESC_RA8866AA	744	512
ESC_RA8865AA	1193	761
ESC_RA8864AA	648	359
ESC_RA8863AA	648	359
ESC_RA8862AA	648	359
ESC_RA8861AA	127	738
ESC_RA8859AA	1193	761
ESC_RA8858AA	1193	761
ESC_RA8857AA	410	156
ESC_RA8856AA	69	387
ESC_RA8855AA	1193	761
ESC_RA8854AA	131	653
ESC_RA8853AA	131	653
ESC_RA8831AA	245	261
ESC_RA8830AA	245	261
ESC_RA8829AA	245	261
ESC_RA8828AA	152	257
ESC_RA8827AA	152	257
ESC_RA8826AA	11230	512
ESC_RA8825AA	11173	71
ESC_RA8824AA	11173	71
ESC_RA8823AA	405	923
ESC_RA8822AA	746	789
ESC_RA8821AA	410	156
ESC_RA8820AA	44	512
ESC_RA8819AA	44	512
ESC_RA8818AA	44	512
ESC_RA8817AA	11236	261
ESC_RA8816AA	245	261
ESC_RA8815AA	245	261
ESC_RA8814AA	245	261
ESC_RA8813AA	245	261
ESC_RA8812AA	152	257
ESC_RA8811AA	152	257
ESC_RA8810AA	11	936
ESC_RA8809AA	11	936
ESC_RA8808AA	10	512
ESC_RA8807AA	6274	257

ESC_RA8806AA	3021	447
ESC_RA8805AA	6880	726
ESC_RA8804AA	7326	156
ESC_RA8803AA	11237	285
ESC_RA8802AA	25	234
ESC_RA8801AA	33	198
ESC_RA8800AA	21	156
ESC_RA8799AA	8422	233
ESC_RA8798AA	11	936
ESC_RA8797AA	11	936
ESC_RA8796AA	11	936
ESC_RA8795AA	617	511
ESC_RA8794AA	940	257
ESC_RA8793AA	940	257
ESC_RA8792AA	405	923
ESC_RA8791AA	167	512
ESC_RA8790AA	940	257
ESC_RA8789AA	167	512
ESC_RA8788AA	167	512
ESC_RA8787AA	940	257
ESC_RA8786AA	405	923
ESC_RA8785AA	167	512
ESC_RA8784AA	167	512
ESC_RA8783AA	405	923
ESC_RA8782AA	648	359
ESC_RA8781AA	940	257
ESC_RA8780AA	410	156
ESC_RA8778AA	205	257
ESC_RA8777AA	410	156
ESC_RA8776AA	156	175
ESC_RA8775AA	1284	604
ESC_RA8774AA	2659	679
ESC_RA8773AA	405	923
ESC_RA8771AA	648	359
ESC_RA8770AA	648	359
ESC_RA8769AA	167	512
ESC_RA8768AA	405	923
ESC_RA8767AA	940	257
ESC_RA8766AA	405	923
ESC_RA8765AA	361	512
ESC_RA8764AA	167	512
ESC_RA8763AA	167	512
ESC_RA8762AA	167	512
ESC_RA8761AA	648	359
ESC_RA8760AA	405	923
ESC_RA8759AA	405	923
ESC_RA8758AA	405	923
ESC_RA8757AA	2851	156
ESC_RA8756AA	940	257
ESC_RA8755AA	940	257

ESC_RA8754AA	940	257
ESC_RA8753AA	617	511
ESC_RA8752AA	167	512
ESC_RA8751AA	167	512
ESC_RA8750AA	2659	679
ESC_RA8749AA	167	512
ESC_RA8748AA	443	257
ESC_RA8747AA	11242	410
ESC_RA8746AA	617	511
ESC_RA8745AA	648	359
ESC_RA8744AA	10886	447
ESC_RA8743AA	69	387
ESC_RA8742AA	5954	923
ESC_RA8741AA	405	923
ESC_RA8740AA	205	257
ESC_RA8739AA	2659	679
ESC_RA8738AA	617	511
ESC_RA8737AA	2659	679
ESC_RA8736AA	2659	679
ESC_RA8735AA	648	359
ESC_RA8734AA	3541	842
ESC_RA8733AA	167	512
ESC_RA8732AA	167	512
ESC_RA8731AA	2450	447
ESC_RA8730AA	167	512
ESC_RA8729AA	617	511
ESC_RA8728AA	167	512
ESC_RA8727AA	155	175
ESC_RA8726AA	4537	76
ESC_RA8725AA	504	738
ESC_RA8724AA	10	512
ESC_RA8723AA	10	512
ESC_RA8722AA	10	512
ESC_RA8721AA	3478	512
ESC_RA8720AA	414	387
ESC_RA8719AA	69	387
ESC_RA8718AA	10909	743
ESC_RA8717AA	10909	743
ESC_RA8716AA	10	512
ESC_RA8715AA	10	512
ESC_RA8714AA	6806	935
ESC_RA8713AA	155	175
ESC_RA8712AA	10	512
ESC_RA8711AA	11241	512
ESC_RA8710AA	11241	512
ESC_RA8709AA	540	286
ESC_RA8708AA	34	504
ESC_RA8707AA	394	400
ESC_RA8706AA	10	512
ESC_RA8705AA	10	512

ESC_RA8704AA	127	738
ESC_RA8703AA	127	738
ESC_RA8702AA	10	512
ESC_RA8701AA	8803	765
ESC_RA8700AA	10	512
ESC_RA8699AA	452	743
ESC_RA8698AA	1136	108
ESC_RA8697AA	2787	198
ESC_RA8696AA	2371	612
ESC_RA8695AA	10	512
ESC_RA8694AA	210	108
ESC_RA8693AA	1163	579
ESC_RA8692AA	48	76
ESC_RA8691AA	10	512
ESC_RA8690AA	10	512
ESC_RA8689AA	641	935
ESC_RA8688AA	48	76
ESC_RA8687AA	101	696
ESC_RA8686AA	10	512
ESC_RA8685AA	2036	512
ESC_RA8684AA	6775	1004
ESC_RA8683AA	8802	682
ESC_RA8682AA	8802	682
ESC_RA8681AA	31	387
ESC_RA8680AA	10	512
ESC_RA8679AA	10	512
ESC_RA8678AA	10	512
ESC_RA8677AA	11245	512
ESC_RA8676AA	607	355
ESC_RA8675AA	449	387
ESC_RA8674AA	616	156
ESC_RA8673AA	295	160
ESC_RA8672AA	11244	142
ESC_RA8671AA	10955	234
ESC_RA8670AA	10	512
ESC_RA8669AA	10	512
ESC_RA8668AA	452	743
ESC_RA8667AA	392	234
ESC_RA8666AA	210	108
ESC_RA8665AA	48	76
ESC_RA8664AA	2722	234
ESC_RA8663AA	607	355
ESC_RA8662AA	43	512
ESC_RA8661AA	11243	474
ESC_RA8660AA	155	175
ESC_RA8659AA	6836	544
ESC_RA8658AA	515	1004
ESC_RA8657AA	155	175
ESC_RA8656AA	9529	1002
ESC_RA8655AA	1112	504

ESC_RA8654AA	48	76
ESC_RA8653AA	648	359
ESC_RA8652AA	770	587
ESC_RA8651AA	2334	701
ESC_RA8650AA	1196	257
ESC_RA8649AA	155	175
ESC_RA8648AA	48	76
ESC_RA8647AA	2197	511
ESC_RA8646AA	354	15
ESC_RA8645AA	366	198
ESC_RA8644AA	2005	175
ESC_RA8643AA	3896	791
ESC_RA8642AA	744	512
ESC_RA8641AA	394	400
ESC_RA8640AA	1567	234
ESC_RA8639AA	1567	234
ESC_RA8638AA	1308	257
ESC_RA8637AA	155	175
ESC_RA8636AA	4138	198
ESC_RA8635AA	155	175
ESC_RA8634AA	155	175
ESC_RA8633AA	9858	453
ESC_RA8632AA	1567	234
ESC_RA8631AA	8800	76
ESC_RA8630AA	2739	76
ESC_RA8629AA	2739	76
ESC_RA8628AA	155	175
ESC_RA8627AA	349	670
ESC_RA8626AA	347	286
ESC_RA8625AA	347	286
ESC_RA8624AA	349	670
ESC_RA8623AA	349	670
ESC_RA8622AA	3580	257
ESC_RA8621AA	155	175
ESC_RA8620AA	23	156
ESC_RA8619AA	349	670
ESC_RA8618AA	423	204
ESC_RA8617AA	155	175
ESC_RA8616AA	1567	234
ESC_RA8615AA	10	512
ESC_RA8614AA	10	512
ESC_RA8613AA	648	359
ESC_RA8612AA	452	743
ESC_RA8611AA	2076	400
ESC_RA8610AA	93	71
ESC_RA8609AA	21	156
ESC_RA8594AA	21	156
ESC_RA8593AA	399	198
ESC_RA8592AA	11246	87
ESC_RA8591AA	17	191

ESC_RA8589AA	515	1004
ESC_RA8588AA	6786	824
ESC_RA8587AA	5229	696
ESC_RA8585AA	48	76
ESC_RA8584AA	906	198
ESC_RA8583AA	515	1004
ESC_RA8582AA	2144	257
ESC_RA8581AA	69	387
ESC_RA8580AA	278	924
ESC_RA8579AA	3014	410
ESC_RA8578AA	48	76
ESC_RA8577AA	278	924
ESC_RA8576AA	1193	761
ESC_RA8575AA	746	789
ESC_RA8574AA	3937	76
ESC_RA8573AA	38	669
ESC_RA8572AA	10	512
ESC_RA8571AA	224	198
ESC_RA8570AA	7122	519
ESC_RA8569AA	48	76
ESC_RA8568AA	38	669
ESC_RA8566AA	398	445
ESC_RA8565AA	5855	519
ESC_RA8564AA	206	286
ESC_RA8563AA	746	789
ESC_RA8562AA	542	824
ESC_RA8561AA	906	198
ESC_RA8560AA	906	198
ESC_RA8559AA	746	789
ESC_RA8558AA	746	789
ESC_RA8557AA	410	156
ESC_RA8556AA	354	15
ESC_RA8553AA	716	795
ESC_RA8552AA	744	512
ESC_RA8551AA	1244	291
ESC_RA8550AA	398	445
ESC_RA8549AA	1193	761
ESC_RA8548AA	38	669
ESC_RA8547AA	8680	76
ESC_RA8546AA	794	924
ESC_RA8545AA	101	696
ESC_RA8544AA	1193	761
ESC_RA8543AA	48	76
ESC_RA8542AA	165	504
ESC_RA8541AA	196	257
ESC_RA8540AA	3337	512
ESC_RA8539AA	10564	512
ESC_RA8538AA	10	512
ESC_RA8537AA	1193	761
ESC_RA8536AA	10565	257

ESC_RA8535AA	6747	701
ESC_RA8534AA	1684	504
ESC_RA8533AA	1684	504
ESC_RA8532AA	6747	701
ESC_RA8531AA	10562	699
ESC_RA8530AA	515	1004
ESC_RA8529AA	906	198
ESC_RA8528AA	226	512
ESC_RA8527AA	69	387
ESC_RA8526AA	542	824
ESC_RA8525AA	10	512
ESC_RA8524AA	216	512
ESC_RA8523AA	10	512
ESC_RA8522AA	6449	504
ESC_RA8521AA	6449	504
ESC_RA8520AA	144	740
ESC_RA8519AA	10	512
ESC_RA8518AA	6449	504
ESC_RA8517AA	6836	544
ESC_RA8516AA	2732	234
ESC_RA8515AA	6836	544
ESC_RA8514AA	6449	504
ESC_RA8513AA	6449	504
ESC_RA8512AA	6449	504
ESC_RA8511AA	8262	234
ESC_RA8510AA	1485	408
ESC_RA8509AA	6786	824
ESC_RA8508AA	6786	824
ESC_RA8507AA	10	512
ESC_RA8506AA	457	595
ESC_RA8505AA	216	512
ESC_RA8504AA	216	512
ESC_RA8503AA	1193	761
ESC_RA8502AA	216	512
ESC_RA8501AA	515	1004
ESC_RA8500AA	58	198
ESC_RA8499AA	195	76
ESC_RA8498AA	767	198
ESC_RA8497AA	515	1004
ESC_RA8496AA	117	592
ESC_RA8495AA	58	198
ESC_RA8494AA	23	156
ESC_RA8493AA	8466	257
ESC_RA8492AA	195	76
ESC_RA8491AA	515	1004
ESC_RA8490AA	3018	903
ESC_RA8489AA	38	669
ESC_RA8488AA	1081	198
ESC_RA8487AA	10	512
ESC_RA8486AA	38	669

ESC_RA8485AA	569	738
ESC_RA8484AA	2144	257
ESC_RA8483AA	34	504
ESC_RA8482AA	7626	960
ESC_RA8481AA	1193	761
ESC_RA8480AA	10	512
ESC_RA8479AA	410	156
ESC_RA8478AA	195	76
ESC_RA8477AA	2067	257
ESC_RA8476AA	195	76
ESC_RA8475AA	6786	824
ESC_RA8474AA	515	1004
ESC_RA8473AA	10	512
ESC_RA8472AA	10	512
ESC_RA8471AA	515	1004
ESC_RA8470AA	398	445
ESC_RA8469AA	48	76
ESC_RA8468AA	648	359
ESC_RA8466AA	10	512
ESC_RA8465AA	648	359
ESC_RA8464AA	3076	175
ESC_RA8463AA	1308	257
ESC_RA8462AA	10	512
ESC_RA8461AA	3944	512
ESC_RA8460AA	3714	175
ESC_RA8459AA	48	76
ESC_RA8458AA	457	595
ESC_RA8457AA	5229	696
ESC_RA8456AA	354	15
ESC_RA8455AA	101	696
ESC_RA8454AA	354	15
ESC_RA8453AA	10	512
ESC_RA8452AA	88	160
ESC_RA8451AA	453	935
ESC_RA8450AA	453	935
ESC_RA8449AA	101	696
ESC_RA8448AA	4086	842
ESC_RA8446AA	3014	410
ESC_RA8445AA	3014	410
ESC_RA8444AA	155	175
ESC_RA8443AA	206	286
ESC_RA8442AA	58	198
ESC_RA8441AA	58	198
ESC_RA8440AA	354	15
ESC_RA8439AA	6021	234
ESC_RA8438AA	641	935
ESC_RA8437AA	10	512
ESC_RA8436AA	10	512
ESC_RA8435AA	3171	515
ESC_RA8434AA	48	76

ESC_RA8433AA	6860	817
ESC_RA8432AA	2380	198
ESC_RA8431AA	410	156
ESC_RA8430AA	155	175
ESC_RA8429AA	155	175
ESC_RA8427AA	542	824
ESC_RA8425AA	48	76
ESC_RA8424AA	206	286
ESC_RA8423AA	1114	504
ESC_RA8422AA	1193	761
ESC_RA8420AA	3631	71
ESC_RA8419AA	717	256
ESC_RA8418AA	2175	212
ESC_RA8417AA	394	400
ESC_RA8416AA	442	257
ESC_RA8415AA	6786	824
ESC_RA8414AA	773	71
ESC_RA8413AA	569	738
ESC_RA8412AA	569	738
ESC_RA8411AA	773	71
ESC_RA8410AA	617	511
ESC_RA8409AA	1638	519
ESC_RA8408AA	2614	975
ESC_RA8407AA	3014	410
ESC_RA8406AA	877	935
ESC_RA8405AA	6786	824
ESC_RA8404AA	10	512
ESC_RA8402AA	4429	820
ESC_RA8401AA	1727	257
ESC_RA8400AA	1727	257
ESC_RA8399AA	48	76
ESC_RA8398AA	6449	504
ESC_RA8397AA	10586	504
ESC_RA8395AA	6449	504
ESC_RA8394AA	6786	824
ESC_RA8393AA	761	512
ESC_RA8392AA	226	512
ESC_RA8391AA	515	1004
ESC_RA8390AA	69	387
ESC_RA8389AA	6449	504
ESC_RA8388AA	10575	175
ESC_RA8387AA	6449	504
ESC_RA8386AA	1800	924
ESC_RA8385AA	971	198
ESC_RA8384AA	34	504
ESC_RA8383AA	442	257
ESC_RA8382AA	131	653
ESC_RA8381AA	617	511
ESC_RA8380AA	13	257
ESC_RA8379AA	48	76

ESC_RA8378AA	542	824
ESC_RA8377AA	218	519
ESC_RA8375AA	6449	504
ESC_RA8374AA	10	512
ESC_RA8373AA	542	824
ESC_RA8372AA	38	669
ESC_RA8371AA	6449	504
ESC_RA8370AA	8466	257
ESC_RA8369AA	3631	71
ESC_RA8368AA	48	76
ESC_RA8366AA	131	653
ESC_RA8365AA	6449	504
ESC_RA8364AA	101	696
ESC_RA8363AA	6786	824
ESC_RA8362AA	48	76
ESC_RA8361AA	515	1004
ESC_RA8360AA	6786	824
ESC_RA8359AA	58	198
ESC_RA8358AA	69	387
ESC_RA8357AA	515	1004
ESC_RA8356AA	6449	504
ESC_RA8355AA	515	1004
ESC_RA8354AA	10570	504
ESC_RA8353AA	131	653
ESC_RA8352AA	542	824
ESC_RA8351AA	515	1004
ESC_RA8350AA	6449	504
ESC_RA8349AA	201	257
ESC_RA8348AA	8262	234
ESC_RA8347AA	206	286
ESC_RA8346AA	10592	789
ESC_RA8345AA	206	286
ESC_RA8344AA	69	387
ESC_RA8343AA	10	512
ESC_RA8342AA	206	286
ESC_RA8341AA	6805	286
ESC_RA8340AA	10568	462
ESC_RA8339AA	10	512
ESC_RA8338AA	155	175
ESC_RA8337AA	10588	76
ESC_RA8335AA	3572	512
ESC_RA8334AA	602	257
ESC_RA8333AA	3014	410
ESC_RA8332AA	34	504
ESC_RA8331AA	38	669
ESC_RA8330AA	9378	519
ESC_RA8329AA	38	669
ESC_RA8328AA	3057	672
ESC_RA8327AA	10567	817
ESC_RA8326AA	542	824

ESC_RA8325AA	542	824
ESC_RA8323AA	48	76
ESC_RA8322AA	542	824
ESC_RA8321AA	10577	286
ESC_RA8320AA	10577	286
ESC_RA8319AA	1716	512
ESC_RA8318AA	515	1004
ESC_RA8317AA	10	512
ESC_RA8316AA	5229	696
ESC_RA8315AA	542	824
ESC_RA8313AA	101	696
ESC_RA8312AA	3171	515
ESC_RA8311AA	542	824
ESC_RA8310AA	602	257
ESC_RA8309AA	1121	198
ESC_RA8308AA	6786	824
ESC_RA8307AA	744	512
ESC_RA8306AA	101	696
ESC_RA8305AA	10572	972
ESC_RA8304AA	746	789
ESC_RA8303AA	77	286
ESC_RA8302AA	10576	463
ESC_RA8301AA	10	512
ESC_RA8300AA	542	824
ESC_RA8299AA	6730	481
ESC_RA8298AA	3856	789
ESC_RA8297AA	7297	842
ESC_RA8296AA	10	512
ESC_RA8295AA	48	76
ESC_RA8294AA	155	175
ESC_RA8293AA	656	789
ESC_RA8292AA	1193	761
ESC_RA8291AA	190	504
ESC_RA8290AA	48	76
ESC_RA8289AA	500	71
ESC_RA8286AA	602	257
ESC_RA8285AA	131	653
ESC_RA8284AA	195	76
ESC_RA8283AA	10	512
ESC_RA8282AA	2170	234
ESC_RA8281AA	10	512
ESC_RA8280AA	10	512
ESC_RA8279AA	3052	669
ESC_RA8278AA	1196	257
ESC_RA8277AA	763	878
ESC_RA8276AA	10	512
ESC_RA8275AA	1112	504
ESC_RA8274AA	48	76
ESC_RA8273AA	190	504
ESC_RA8272AA	361	512

ESC_RA8271AA	48	76
ESC_RA8270AA	7051	32
ESC_RA8269AA	10594	75
ESC_RA8268AA	1286	474
ESC_RA8267AA	48	76
ESC_RA8266AA	1286	474
ESC_RA8265AA	1286	474
ESC_RA8264AA	1286	474
ESC_RA8263AA	10562	699
ESC_RA8262AA	2012	257
ESC_RA8261AA	48	76
ESC_RA8260AA	10578	824
ESC_RA8259AA	1193	761
ESC_RA8258AA	206	286
ESC_RA8256AA	6775	1004
ESC_RA8254AA	1788	785
ESC_RA8253AA	1421	447
ESC_RA8252AA	1421	447
ESC_RA8251AA	6308	765
ESC_RA8250AA	10595	814
ESC_RA8249AA	683	175
ESC_RA8248AA	165	504
ESC_RA8247AA	3171	515
ESC_RA8246AA	48	76
ESC_RA8245AA	10562	699
ESC_RA8244AA	195	76
ESC_RA8243AA	206	286
ESC_RA8242AA	48	76
ESC_RA8241AA	10	512
ESC_RA8240AA	1638	519
ESC_RA8239AA	5951	406
ESC_RA8238AA	410	156
ESC_RA8237AA	34	504
ESC_RA8235AA	345	175
ESC_RA8234AA	10586	504
ESC_RA8233AA	5204	311
ESC_RA8232AA	101	696
ESC_RA8231AA	10	512
ESC_RA8230AA	224	198
ESC_RA8229AA	48	76
ESC_RA8228AA	10	512
ESC_RA8227AA	9749	512
ESC_RA8226AA	10	512
ESC_RA8225AA	155	175
ESC_RA8224AA	542	824
ESC_RA8223AA	10560	504
ESC_RA8222AA	10560	504
ESC_RA8221AA	10	512
ESC_RA8220AA	48	76
ESC_RA8219AA	10	512

ESC_RA8218AA	457	595
ESC_RA8217AA	10	512
ESC_RA8216AA	542	824
ESC_RA8215AA	131	653
ESC_RA8214AA	131	653
ESC_RA8213AA	196	257
ESC_RA8212AA	457	595
ESC_RA8211AA	457	595
ESC_RA8210AA	181	311
ESC_RA8209AA	515	1004
ESC_RA8208AA	354	15
ESC_RA8207AA	457	595
ESC_RA8206AA	9402	789
ESC_RA8205AA	206	286
ESC_RA8204AA	351	268
ESC_RA8203AA	515	1004
ESC_RA8202AA	101	696
ESC_RA8201AA	515	1004
ESC_RA8200AA	6449	504
ESC_RA8199AA	6449	504
ESC_RA8198AA	7176	1004
ESC_RA8197AA	155	175
ESC_RA8196AA	10	512
ESC_RA8195AA	906	198
ESC_RA8194AA	38	669
ESC_RA8193AA	746	789
ESC_RA8192AA	7122	519
ESC_RA8191AA	155	175
ESC_RA8190AA	542	824
ESC_RA8189AA	7059	0
ESC_RA8188AA	7176	1004
ESC_RA8187AA	48	76
ESC_RA8186AA	156	175
ESC_RA8185AA	398	445
ESC_RA8184AA	226	512
ESC_RA8183AA	165	504
ESC_RA8182AA	711	935
ESC_RA8181AA	746	789
ESC_RA8180AA	763	878
ESC_RA8179AA	101	696
ESC_RA8178AA	1140	934
ESC_RA8177AA	6449	504
ESC_RA8176AA	6449	504
ESC_RA8175AA	871	447
ESC_RA8174AA	189	504
ESC_RA8173AA	6730	481
ESC_RA8172AA	2064	261
ESC_RA8171AA	1286	474
ESC_RA8170AA	278	924
ESC_RA8169AA	1788	785

ESC_RA8168AA	8466	257
ESC_RA8167AA	7224	630
ESC_RA8166AA	131	653
ESC_RA8165AA	7176	1004
ESC_RA8164AA	101	696
ESC_RA8163AA	1193	761
ESC_RA8162AA	48	76
ESC_RA8161AA	685	311
ESC_RA8160AA	336	878
ESC_RA8159AA	394	400
ESC_RA8158AA	8677	483
ESC_RA8157AA	155	175
ESC_RA8156AA	77	286
ESC_RA8154AA	48	76
ESC_RA8153AA	772	76
ESC_RA8151AA	206	286
ESC_RA8150AA	3580	257
ESC_RA8149AA	3274	512
ESC_RA8148AA	10	512
ESC_RA8147AA	155	175
ESC_RA8146AA	10	512
ESC_RA8145AA	542	824
ESC_RA8144AA	10	512
ESC_RA8143AA	542	824
ESC_RA8142AA	58	198
ESC_RA8141AA	189	504
ESC_RA8139AA	6730	481
ESC_RA8138AA	4429	820
ESC_RA8137AA	4429	820
ESC_RA8136AA	542	824
ESC_RA8135AA	58	198
ESC_RA8133AA	6732	1004
ESC_RA8132AA	7176	1004
ESC_RA8131AA	77	286
ESC_RA8130AA	10590	1007
ESC_RA8129AA	1638	519
ESC_RA8128AA	654	257
ESC_RA8127AA	13	257
ESC_RA8126AA	1244	291
ESC_RA8125AA	515	1004
ESC_RA8124AA	515	1004
ESC_RA8123AA	195	76
ESC_RA8121AA	4015	512
ESC_RA8120AA	398	445
ESC_RA8119AA	515	1004
ESC_RA8118AA	515	1004
ESC_RA8117AA	131	653
ESC_RA8116AA	29	156
ESC_RA8115AA	29	156
ESC_RA8114AA	1193	761

ESC_RA8113AA	3054	198
ESC_RA8112AA	48	76
ESC_RA8111AA	10562	699
ESC_RA8110AA	10562	699
ESC_RA8109AA	6449	504
ESC_RA8108AA	10562	699
ESC_RA8107AA	10562	699
ESC_RA8106AA	6786	824
ESC_RA8105AA	398	445
ESC_RA8104AA	10591	512
ESC_RA8103AA	48	76
ESC_RA8102AA	69	387
ESC_RA8101AA	549	387
ESC_RA8100AA	48	76
ESC_RA8099AA	10	512
ESC_RA8098AA	871	447
ESC_RA8097AA	33	198
ESC_RA8096AA	131	653
ESC_RA8095AA	11247	935
ESC_RA8094AA	847	257
ESC_RA8093AA	131	653
ESC_RA8092AA	2768	198
ESC_RA8091AA	131	653
ESC_RA8090AA	131	653
ESC_RA8089AA	131	653
ESC_RA8088AA	10	512
ESC_RA8087AA	354	15
ESC_RA8086AA	131	653
ESC_RA8085AA	131	653
ESC_RA8084AA	131	653
ESC_RA8083AA	131	653
ESC_RA8082AA	131	653
ESC_RA8081AA	131	653
ESC_RA8080AA	131	653
ESC_RA8079AA	131	653
ESC_RA8078AA	131	653
ESC_RA8077AA	131	653
ESC_RA8076AA	131	653
ESC_RA8075AA	131	653
ESC_RA8074AA	1011	198
ESC_RA8073AA	11226	447
ESC_RA8071AA	11229	71
ESC_RA8070AA	11228	198
ESC_RA8069AA	11227	198
ESC_RA8068AA	11226	447
ESC_RA8067AA	11227	198
ESC_RA8066AA	11226	447
ESC_RA8065AA	11226	447
ESC_RA8064AA	11226	447
ESC_RA8063AA	11226	447

ESC_RA8062AA	8804	71
ESC_RA8061AA	11226	447
ESC_RA8060AA	11226	447
ESC_RA8059AA	11226	447
ESC_RA8054AA	10011	221
ESC_RA8053AA	101	696
ESC_RA8052AA	10011	221
ESC_RA8051AA	10011	221
ESC_RA8050AA	10009	670
ESC_RA8048AA	21	156
ESC_RA8047AA	21	156
ESC_RA8044AA	337	257
ESC_RA8043AA	11225	512
ESC_RA8041AA	152	257
ESC_RA8040AA	6870	603
ESC_RA8037AA	405	923
ESC_RA8036AA	652	204
ESC_RA8035AA	167	512
ESC_RA8033AA	453	935
ESC_RA8032AA	2171	164
ESC_RA8031AA	156	175
ESC_RA8030AA	155	175
ESC_RA8029AA	1258	257
ESC_RA8028AA	1258	257
ESC_RA8027AA	11223	198
ESC_RA8026AA	11224	156
ESC_RA8025AA	223	198
ESC_RA8024AA	155	175
ESC_RA8023AA	155	175
ESC_RA8022AA	155	175
ESC_RA8021AA	306	286
ESC_RA8019AA	90	156
ESC_RA8018AA	90	156
ESC_RA8017AA	90	156
ESC_RA8016AA	101	696
ESC_RA8015AA	152	257
ESC_RA8014AA	714	318
ESC_RA8013AA	109	257
ESC_RA8012AA	446	257
ESC_RA8011AA	8977	198
ESC_RA8010AA	11222	311
ESC_RA8009AA	11221	286
ESC_RA8008AA	245	261
ESC_RA8006AA	245	261
ESC_RA8005AA	245	261
ESC_RA8004AA	245	261
ESC_RA8003AA	245	261
ESC_RA8002AA	245	261
ESC_RA8001AA	245	261
ESC_RA8000AA	245	261

ESC_RA7999AA	245	261
ESC_RA7998AA	245	261
ESC_RA7997AA	17	191
ESC_RA7995AA	216	512
ESC_RA7994AA	906	198
ESC_RA7993AA	17	191
ESC_RA7992AA	88	160
ESC_RA7991AA	25	234
ESC_RA7990AA	10	512
ESC_RA7989AA	29	156
ESC_RA7988AA	342	286
ESC_RA7987AA	738	598
ESC_RA7986AA	25	234
ESC_RA7985AA	21	156
ESC_RA7984AA	21	156
ESC_RA7983AA	11	936
ESC_RA7982AA	11	936
ESC_RA7979AA	11219	234
ESC_RA7958AA	10	512
ESC_RA7957AA	10	512
ESC_RA7956AA	10	512
ESC_RA7955AA	10	512
ESC_RA7954AA	10	512
ESC_RA7953AA	10	512
ESC_RA7952AA	10	512
ESC_RA7951AA	10	512
ESC_RA7949AA	10	512
ESC_RA7948AA	16	156
ESC_RA7947AA	5073	900
ESC_RA7946AA	131	653
ESC_RA7945AA	86	935
ESC_RA7944AA	58	198
ESC_RA7943AA	69	387
ESC_RA7942AA	3580	257
ESC_RA7941AA	2253	71
ESC_RA7940AA	58	198
ESC_RA7939AA	1944	934
ESC_RA7938AA	29	156
ESC_RA7937AA	58	198
ESC_RA7936AA	58	198
ESC_RA7935AA	16	156
ESC_RA7934AA	58	198
ESC_RA7933AA	58	198
ESC_RA7932AA	11220	286
ESC_RA7931AA	58	198
ESC_RA7930AA	16	156
ESC_RA7929AA	58	198
ESC_RA7928AA	16	156
ESC_RA7927AA	23	156
ESC_RA7926AA	58	198

ESC_RA7925AA	2522	198
ESC_RA7924AA	16	156
ESC_RA7923AA	16	156
ESC_RA7922AA	2522	198
ESC_RA7921AA	29	156
ESC_RA7920AA	16	156
ESC_RA7919AA	101	696
ESC_RA7918AA	58	198
ESC_RA7917AA	342	286
ESC_RA7916AA	16	156
ESC_RA7915AA	58	198
ESC_RA7914AA	16	156
ESC_RA7913AA	16	156
ESC_RA7912AA	16	156
ESC_RA7911AA	29	156
ESC_RA7910AA	2522	198
ESC_RA7909AA	58	198
ESC_RA7908AA	16	156
ESC_RA7906AA	16	156
ESC_RA7905AA	16	156
ESC_RA7904AA	16	156
ESC_RA7903AA	16	156
ESC_RA7902AA	16	156
ESC_RA7901AA	16	156
ESC_RA7900AA	16	156
ESC_RA7899AA	16	156
ESC_RA7898AA	58	198
ESC_RA7897AA	16	156
ESC_RA7896AA	16	156
ESC_RA7895AA	16	156
ESC_RA7894AA	16	156
ESC_RA7893AA	155	175
ESC_RA7891AA	11218	445
ESC_RA7890AA	245	261
ESC_RA7889AA	245	261
ESC_RA7888AA	245	261
ESC_RA7887AA	245	261
ESC_RA7886AA	245	261
ESC_RA7885AA	131	653
ESC_RA7884AA	315	669
ESC_RA7882AA	11	936
ESC_RA7881AA	11	936
ESC_RA7880AA	11	936
ESC_RA7879AA	11	936
ESC_RA7878AA	11	936
ESC_RA7877AA	11	936
ESC_RA7876AA	245	261
ESC_RA7875AA	6200	261
ESC_RA7874AA	152	257
ESC_RA7873AA	152	257

ESC_RA7872AA	11	936
ESC_RA7871AA	8392	512
ESC_RA7870AA	361	512
ESC_RA7869AA	2083	257
ESC_RA7866AA	11214	512
ESC_RA7865AA	21	156
ESC_RA7864AA	10	512
ESC_RA7863AA	156	175
ESC_RA7862AA	223	198
ESC_RA7861AA	10	512
ESC_RA7860AA	718	257
ESC_RA7859AA	306	286
ESC_RA7858AA	10	512
ESC_RA7857AA	10	512
ESC_RA7856AA	515	1004
ESC_RA7855AA	10	512
ESC_RA7854AA	330	504
ESC_RA7853AA	306	286
ESC_RA7852AA	245	261
ESC_RA7851AA	245	261
ESC_RA7850AA	245	261
ESC_RA7849AA	245	261
ESC_RA7848AA	245	261
ESC_RA7847AA	152	257
ESC_RA7846AA	152	257
ESC_RA7845AA	245	261
ESC_RA7844AA	17	191
ESC_RA7843AA	10	512
ESC_RA7842AA	69	387
ESC_RA7841AA	117	592
ESC_RA7840AA	394	400
ESC_RA7839AA	58	198
ESC_RA7838AA	38	669
ESC_RA7837AA	131	653
ESC_RA7836AA	131	653
ESC_RA7835AA	69	387
ESC_RA7834AA	2450	447
ESC_RA7833AA	156	175
ESC_RA7832AA	410	156
ESC_RA7831AA	23	156
ESC_RA7830AA	23	156
ESC_RA7829AA	131	653
ESC_RA7828AA	131	653
ESC_RA7827AA	131	653
ESC_RA7826AA	11217	301
ESC_RA7825AA	16	156
ESC_RA7824AA	17	191
ESC_RA7823AA	753	842
ESC_RA7822AA	753	842
ESC_RA7821AA	17	191

ESC_RA7820AA	720	900
ESC_RA7819AA	16	156
ESC_RA7818AA	16	156
ESC_RA7817AA	21	156
ESC_RA7816AA	25	234
ESC_RA7815AA	8549	512
ESC_RA7814AA	21	156
ESC_RA7813AA	16	156
ESC_RA7812AA	1794	204
ESC_RA7811AA	17	191
ESC_RA7810AA	33	198
ESC_RA7809AA	675	234
ESC_RA7808AA	675	234
ESC_RA7807AA	11	936
ESC_RA7805AA	811	234
ESC_RA7804AA	21	156
ESC_RA7803AA	21	156
ESC_RA7802AA	753	842
ESC_RA7801AA	21	156
ESC_RA7800AA	69	387
ESC_RA7799AA	21	156
ESC_RA7798AA	17	191
ESC_RA7797AA	21	156
ESC_RA7796AA	10	512
ESC_RA7795AA	21	156
ESC_RA7794AA	21	156
ESC_RA7793AA	21	156
ESC_RA7792AA	738	598
ESC_RA7791AA	29	156
ESC_RA7790AA	33	198
ESC_RA7789AA	21	156
ESC_RA7788AA	21	156
ESC_RA7787AA	300	286
ESC_RA7786AA	21	156
ESC_RA7785AA	21	156
ESC_RA7784AA	21	156
ESC_RA7783AA	21	156
ESC_RA7782AA	21	156
ESC_RA7781AA	21	156
ESC_RA7780AA	21	156
ESC_RA7779AA	21	156
ESC_RA7778AA	21	156
ESC_RA7777AA	8621	251
ESC_RA7776AA	21	156
ESC_RA7775AA	442	257
ESC_RA7774AA	442	257
ESC_RA7773AA	1415	504
ESC_RA7772AA	1415	504
ESC_RA7771AA	453	935
ESC_RA7770AA	131	653

ESC_RA7769AA	131	653
ESC_RA7768AA	405	923
ESC_RA7767AA	73	737
ESC_RA7766AA	131	653
ESC_RA7765AA	10	512
ESC_RA7764AA	21	156
ESC_RA7763AA	21	156
ESC_RA7762AA	301	307
ESC_RA7761AA	11	936
ESC_RA7760AA	11	936
ESC_RA7759AA	6495	336
ESC_RA7758AA	245	261
ESC_RA7757AA	152	257
ESC_RA7756AA	152	257
ESC_RA7755AA	245	261
ESC_RA7754AA	245	261
ESC_RA7753AA	245	261
ESC_RA7752AA	245	261
ESC_RA7751AA	152	257
ESC_RA7750AA	152	257
ESC_RA7749AA	245	261
ESC_RA7748AA	245	261
ESC_RA7747AA	17	191
ESC_RA7742AA	117	592
ESC_RA7741AA	906	198
ESC_RA7739AA	3321	935
ESC_RA7738AA	906	198
ESC_RA7737AA	10	512
ESC_RA7736AA	906	198
ESC_RA7735AA	88	160
ESC_RA7734AA	1564	512
ESC_RA7733AA	10334	935
ESC_RA7732AA	442	257
ESC_RA7731AA	11	936
ESC_RA7730AA	33	198
ESC_RA7729AA	10334	935
ESC_RA7728AA	6274	257
ESC_RA7727AA	1041	765
ESC_RA7726AA	1041	765
ESC_RA7725AA	21	156
ESC_RA7724AA	29	156
ESC_RA7722AA	7505	696
ESC_RA7721AA	1122	307
ESC_RA7720AA	135	653
ESC_RA7719AA	2040	198
ESC_RA7718AA	11216	886
ESC_RA7717AA	86	935
ESC_RA7716AA	101	696
ESC_RA7715AA	1611	198
ESC_RA7714AA	355	764

ESC_RA7713AA	752	512
ESC_RA7712AA	428	654
ESC_RA7711AA	5853	868
ESC_RA7710AA	1684	504
ESC_RA7709AA	1684	504
ESC_RA7708AA	351	268
ESC_RA7707AA	752	512
ESC_RA7706AA	11215	408
ESC_RA7705AA	3580	257
ESC_RA7704AA	352	744
ESC_RA7703AA	115	669
ESC_RA7702AA	2280	212
ESC_RA7701AA	9956	878
ESC_RA7700AA	86	935
ESC_RA7699AA	144	740
ESC_RA7698AA	13	257
ESC_RA7697AA	381	156
ESC_RA7696AA	8629	935
ESC_RA7695AA	11	936
ESC_RA7694AA	16	156
ESC_RA7693AA	11	936
ESC_RA7692AA	1629	924
ESC_RA7691AA	300	286
ESC_RA7690AA	10	512
ESC_RA7689AA	180	234
ESC_RA7688AA	58	198
ESC_RA7687AA	69	387
ESC_RA7686AA	88	160
ESC_RA7685AA	410	156
ESC_RA7684AA	162	924
ESC_RA7683AA	410	156
ESC_RA7682AA	162	924
ESC_RA7681AA	1844	198
ESC_RA7680AA	155	175
ESC_RA7679AA	2964	912
ESC_RA7678AA	394	400
ESC_RA7677AA	162	924
ESC_RA7676AA	131	653
ESC_RA7675AA	162	924
ESC_RA7674AA	394	400
ESC_RA7673AA	155	175
ESC_RA7672AA	155	175
ESC_RA7671AA	90	156
ESC_RA7670AA	29	156
ESC_RA7669AA	540	286
ESC_RA7668AA	57	50
ESC_RA7667AA	88	160
ESC_RA7666AA	57	50
ESC_RA7665AA	410	156
ESC_RA7664AA	88	160

ESC_RA7663AA	10	512
ESC_RA7662AA	1607	844
ESC_RA7661AA	10	512
ESC_RA7660AA	540	286
ESC_RA7659AA	398	445
ESC_RA7658AA	58	198
ESC_RA7657AA	48	76
ESC_RA7656AA	1244	291
ESC_RA7655AA	10	512
ESC_RA7654AA	641	935
ESC_RA7653AA	641	935
ESC_RA7505AA	73	737
ESC_RA7504AA	216	512
ESC_RA7503AA	216	512
ESC_RA7502AA	216	512
ESC_RA7501AA	69	387
ESC_RA7500AA	127	738
ESC_RA7499AA	372	424
ESC_RA7498AA	393	343
ESC_RA7497AA	131	653
ESC_RA7496AA	131	653
ESC_RA7495AA	131	653
ESC_RA7494AA	131	653
ESC_RA7493AA	73	737
ESC_RA7492AA	95	738
ESC_RA7491AA	131	653
ESC_RA7490AA	131	653
ESC_RA7489AA	10	512
ESC_RA7488AA	127	738
ESC_RA7487AA	393	343
ESC_RA7486AA	117	592
ESC_RA7485AA	405	923
ESC_RA7484AA	131	653
ESC_RA7483AA	69	387
ESC_RA7482AA	69	387
ESC_RA7481AA	550	756
ESC_RA7480AA	95	738
ESC_RA7479AA	73	737
ESC_RA7478AA	38	669
ESC_RA7477AA	405	923
ESC_RA7476AA	393	343
ESC_RA7475AA	58	198
ESC_RA7474AA	1193	761
ESC_RA7473AA	131	653
ESC_RA7472AA	200	198
ESC_RA7471AA	448	257
ESC_RA7470AA	131	653
ESC_RA7469AA	773	71
ESC_RA7468AA	131	653
ESC_RA7467AA	131	653

ESC_RA7466AA	131	653
ESC_RA7465AA	95	738
ESC_RA7464AA	38	669
ESC_RA7463AA	648	359
ESC_RA7462AA	167	512
ESC_RA7461AA	405	923
ESC_RA7460AA	69	387
ESC_RA7459AA	131	653
ESC_RA7458AA	131	653
ESC_RA7457AA	73	737
ESC_RA7456AA	961	623
ESC_RA7455AA	131	653
ESC_RA7454AA	144	740
ESC_RA7453AA	1193	761
ESC_RA7452AA	131	653
ESC_RA7451AA	131	653
ESC_RA7450AA	38	669
ESC_RA7449AA	95	738
ESC_RA7448AA	131	653
ESC_RA7447AA	73	737
ESC_RA7446AA	144	740
ESC_RA7445AA	141	644
ESC_RA7444AA	69	387
ESC_RA7443AA	10	512
ESC_RA7442AA	10	512
ESC_RA7441AA	131	653
ESC_RA7440AA	38	669
ESC_RA7439AA	117	592
ESC_RA7438AA	245	261
ESC_RA7437AA	245	261
ESC_RA7436AA	245	261
ESC_RA7435AA	1502	257
ESC_RA7434AA	245	261
ESC_RA7433AA	245	261
ESC_RA7432AA	68	386
ESC_RA7431AA	660	286
ESC_RA7430AA	21	156
ESC_RA7429AA	152	257
ESC_RA7428AA	11	936
ESC_RA7427AA	21	156
ESC_RA7426AA	21	156
ESC_RA7425AA	101	696
ESC_RA7424AA	58	198
ESC_RA7423AA	1665	601
ESC_RA7422AA	155	175
ESC_RA7421AA	5082	198
ESC_RA7420AA	162	924
ESC_RA7419AA	8927	387
ESC_RA7418AA	11039	257
ESC_RA7417AA	73	737

ESC_RA7416AA	23	156
ESC_RA7415AA	7473	789
ESC_RA7414AA	642	924
ESC_RA7413AA	5995	504
ESC_RA7412AA	5573	396
ESC_RA7411AA	906	198
ESC_RA7410AA	328	924
ESC_RA7409AA	156	175
ESC_RA7408AA	10	512
ESC_RA7407AA	140	738
ESC_RA7406AA	155	175
ESC_RA7405AA	603	198
ESC_RA7404AA	155	175
ESC_RA7403AA	11202	447
ESC_RA7402AA	100	504
ESC_RA7401AA	10	512
ESC_RA7400AA	58	198
ESC_RA7399AA	10	512
ESC_RA7398AA	2223	76
ESC_RA7397AA	1125	198
ESC_RA7396AA	88	160
ESC_RA7395AA	1385	653
ESC_RA7394AA	10	512
ESC_RA7393AA	555	653
ESC_RA7392AA	746	789
ESC_RA7391AA	5396	924
ESC_RA7390AA	57	50
ESC_RA7389AA	90	156
ESC_RA7388AA	20	198
ESC_RA7387AA	29	156
ESC_RA7386AA	1463	263
ESC_RA7385AA	410	156
ESC_RA7384AA	9971	924
ESC_RA7383AA	1638	519
ESC_RA7382AA	1721	311
ESC_RA7381AA	10	512
ESC_RA7380AA	327	204
ESC_RA7379AA	10	512
ESC_RA7378AA	10	512
ESC_RA7377AA	11203	175
ESC_RA7376AA	542	824
ESC_RA7375AA	34	504
ESC_RA7374AA	706	422
ESC_RA7373AA	1992	257
ESC_RA7372AA	429	654
ESC_RA7371AA	297	234
ESC_RA7370AA	93	71
ESC_RA7369AA	1434	512
ESC_RA7368AA	602	257
ESC_RA7367AA	405	923

ESC_RA7366AA	1611	198
ESC_RA7365AA	5309	286
ESC_RA7364AA	955	670
ESC_RA7363AA	58	198
ESC_RA7362AA	4559	878
ESC_RA7361AA	88	160
ESC_RA7360AA	56	198
ESC_RA7359AA	10	512
ESC_RA7358AA	155	175
ESC_RA7357AA	6594	257
ESC_RA7356AA	17	191
ESC_RA7355AA	101	696
ESC_RA7354AA	10	512
ESC_RA7353AA	9637	504
ESC_RA7352AA	216	512
ESC_RA7351AA	1146	198
ESC_RA7350AA	20	198
ESC_RA7349AA	11204	764
ESC_RA7348AA	542	824
ESC_RA7347AA	1245	270
ESC_RA7346AA	20	198
ESC_RA7345AA	11204	764
ESC_RA7344AA	1102	830
ESC_RA7343AA	201	257
ESC_RA7342AA	20	198
ESC_RA7341AA	135	653
ESC_RA7340AA	1161	764
ESC_RA7339AA	2567	649
ESC_RA7338AA	349	670
ESC_RA7337AA	10966	824
ESC_RA7336AA	1408	445
ESC_RA7335AA	101	696
ESC_RA7334AA	3714	175
ESC_RA7333AA	10	512
ESC_RA7332AA	8120	950
ESC_RA7331AA	1178	512
ESC_RA7330AA	58	198
ESC_RA7329AA	11205	235
ESC_RA7328AA	5873	76
ESC_RA7327AA	542	824
ESC_RA7326AA	11206	425
ESC_RA7325AA	10	512
ESC_RA7324AA	101	696
ESC_RA7323AA	3381	268
ESC_RA7322AA	5663	670
ESC_RA7321AA	90	156
ESC_RA7320AA	5265	512
ESC_RA7319AA	11207	204
ESC_RA7318AA	5044	1004
ESC_RA7317AA	6344	254

ESC_RA7316AA	93	71
ESC_RA7315AA	500	71
ESC_RA7314AA	117	592
ESC_RA7313AA	746	789
ESC_RA7312AA	10	512
ESC_RA7311AA	295	160
ESC_RA7310AA	188	257
ESC_RA7309AA	165	504
ESC_RA7308AA	109	257
ESC_RA7307AA	302	850
ESC_RA7306AA	448	257
ESC_RA7305AA	62	574
ESC_RA7304AA	94	257
ESC_RA7303AA	6466	311
ESC_RA7302AA	405	923
ESC_RA7301AA	11208	447
ESC_RA7300AA	95	738
ESC_RA7299AA	641	935
ESC_RA7298AA	162	924
ESC_RA7297AA	555	653
ESC_RA7296AA	1434	512
ESC_RA7295AA	348	175
ESC_RA7294AA	1403	924
ESC_RA7293AA	8556	257
ESC_RA7292AA	4121	504
ESC_RA7291AA	118	985
ESC_RA7290AA	155	175
ESC_RA7289AA	641	935
ESC_RA7288AA	10	512
ESC_RA7287AA	1091	512
ESC_RA7286AA	1091	512
ESC_RA7285AA	101	696
ESC_RA7284AA	10	512
ESC_RA7283AA	10983	626
ESC_RA7282AA	6517	71
ESC_RA7281AA	2079	198
ESC_RA7280AA	6517	71
ESC_RA7279AA	641	935
ESC_RA7278AA	10	512
ESC_RA7277AA	201	257
ESC_RA7276AA	10	512
ESC_RA7275AA	154	257
ESC_RA7274AA	11	936
ESC_RA7273AA	28	537
ESC_RA7272AA	16	156
ESC_RA7271AA	16	156
ESC_RA7270AA	16	156
ESC_RA7269AA	16	156
ESC_RA7268AA	16	156
ESC_RA7267AA	16	156

ESC_RA7266AA	16	156
ESC_RA7265AA	11209	257
ESC_RA7264AA	342	286
ESC_RA7263AA	382	481
ESC_RA7262AA	17	191
ESC_RA7261AA	6175	257
ESC_RA7260AA	11	936
ESC_RA7259AA	17	191
ESC_RA7258AA	21	156
ESC_RA7257AA	16	156
ESC_RA7256AA	678	257
ESC_RA7255AA	16	156
ESC_RA7254AA	16	156
ESC_RA7253AA	32	270
ESC_RA7252AA	32	270
ESC_RA7251AA	32	270
ESC_RA7250AA	306	286
ESC_RA7249AA	32	270
ESC_RA7248AA	32	270
ESC_RA7247AA	333	286
ESC_RA7246AA	206	286
ESC_RA7245AA	11	936
ESC_RA7244AA	5559	257
ESC_RA7243AA	5559	257
ESC_RA7242AA	5559	257
ESC_RA7241AA	5559	257
ESC_RA7240AA	5559	257
ESC_RA7239AA	5559	257
ESC_RA7238AA	5559	257
ESC_RA7237AA	5559	257
ESC_RA7236AA	1967	191
ESC_RA7235AA	718	257
ESC_RA7234AA	21	156
ESC_RA7233AA	21	156
ESC_RA7232AA	21	156
ESC_RA7231AA	11	936
ESC_RA7230AA	5559	257
ESC_RA7229AA	5559	257
ESC_RA7228AA	5559	257
ESC_RA7227AA	5559	257
ESC_RA7226AA	11	936
ESC_RA7225AA	11	936
ESC_RA7224AA	641	935
ESC_RA7223AA	77	286
ESC_RA7222AA	11	936
ESC_RA7221AA	10	512
ESC_RA7220AA	2473	257
ESC_RA7219AA	11201	257
ESC_RA7218AA	8777	447
ESC_RA7217AA	6160	198

ESC_RA7216AA	21	156
ESC_RA7215AA	5392	156
ESC_RA7214AA	119	286
ESC_RA7213AA	11	936
ESC_RA7212AA	446	257
ESC_RA7211AA	119	286
ESC_RA7210AA	21	156
ESC_RA7209AA	16	156
ESC_RA7208AA	48	76
ESC_RA7207AA	2002	778
ESC_RA7206AA	641	935
ESC_RA7205AA	10	512
ESC_RA7204AA	335	936
ESC_RA7203AA	48	76
ESC_RA7202AA	1721	311
ESC_RA7201AA	1582	198
ESC_RA7200AA	381	156
ESC_RA7199AA	95	738
ESC_RA7198AA	58	198
ESC_RA7197AA	3703	766
ESC_RA7196AA	16	156
ESC_RA7195AA	21	156
ESC_RA7194AA	17	191
ESC_RA7193AA	1283	766
ESC_RA7192AA	113	156
ESC_RA7191AA	16	156
ESC_RA7190AA	1304	257
ESC_RA7189AA	223	198
ESC_RA7188AA	7254	781
ESC_RA7187AA	788	659
ESC_RA7186AA	1717	212
ESC_RA7185AA	1664	204
ESC_RA7184AA	86	935
ESC_RA7183AA	11211	765
ESC_RA7182AA	316	257
ESC_RA7181AA	2346	435
ESC_RA7180AA	1380	914
ESC_RA7179AA	11	936
ESC_RA7178AA	343	286
ESC_RA7177AA	655	926
ESC_RA7176AA	17	191
ESC_RA7175AA	6175	257
ESC_RA7174AA	677	257
ESC_RA7173AA	16	156
ESC_RA7172AA	343	286
ESC_RA7171AA	655	926
ESC_RA7170AA	655	926
ESC_RA7169AA	17	191
ESC_RA7168AA	16	156
ESC_RA7167AA	32	270

ESC_RA7166AA	21	156
ESC_RA7165AA	32	270
ESC_RA7164AA	32	270
ESC_RA7163AA	32	270
ESC_RA7162AA	718	257
ESC_RA7161AA	32	270
ESC_RA7160AA	306	286
ESC_RA7159AA	718	257
ESC_RA7158AA	21	156
ESC_RA7157AA	765	156
ESC_RA7156AA	1967	191
ESC_RA7155AA	5559	257
ESC_RA7154AA	1967	191
ESC_RA7153AA	1967	191
ESC_RA7152AA	21	156
ESC_RA7151AA	5559	257
ESC_RA7150AA	5559	257
ESC_RA7149AA	718	257
ESC_RA7148AA	718	257
ESC_RA7147AA	718	257
ESC_RA7146AA	17	191
ESC_RA7145AA	21	156
ESC_RA7144AA	442	257
ESC_RA7143AA	4684	257
ESC_RA7142AA	342	286
ESC_RA7141AA	4496	924
ESC_RA7140AA	223	198
ESC_RA7139AA	947	257
ESC_RA7138AA	206	286
ESC_RA7137AA	590	156
ESC_RA7136AA	381	156
ESC_RA7135AA	21	156
ESC_RA7134AA	328	924
ESC_RA7133AA	21	156
ESC_RA7132AA	21	156
ESC_RA7131AA	300	286
ESC_RA7130AA	17	191
ESC_RA7129AA	2388	198
ESC_RA7128AA	28	537
ESC_RA7127AA	21	156
ESC_RA7126AA	3	198
ESC_RA7125AA	11212	766
ESC_RA7124AA	949	175
ESC_RA7123AA	394	400
ESC_RA7122AA	11213	776
ESC_RA7121AA	48	76
ESC_RA7120AA	2201	650
ESC_RA7119AA	101	696
ESC_RA7118AA	11212	766
ESC_RA7117AA	11	936

ESC_RA7116AA	11	936
ESC_RA7115AA	21	156
ESC_RA7114AA	11	936
ESC_RA7113AA	17	191
ESC_RA7112AA	1060	512
ESC_RA7111AA	10	512
ESC_RA7110AA	17	191
ESC_RA7109AA	6175	257
ESC_RA7108AA	32	270
ESC_RA7107AA	5559	257
ESC_RA7106AA	17	191
ESC_RA7105AA	5559	257
ESC_RA7104AA	32	270
ESC_RA7103AA	718	257
ESC_RA7102AA	5559	257
ESC_RA7101AA	718	257
ESC_RA7100AA	5559	257
ESC_RA7099AA	5559	257
ESC_RA7098AA	5559	257
ESC_RA7097AA	5559	257
ESC_RA7096AA	2813	756
ESC_RA7095AA	5559	257
ESC_RA7094AA	21	156
ESC_RA7093AA	21	156
ESC_RA7092AA	683	175
ESC_RA7091AA	69	387
ESC_RA7090AA	336	878
ESC_RA7089AA	4496	924
ESC_RA7088AA	336	878
ESC_RA7087AA	1431	257
ESC_RA7086AA	162	924
ESC_RA7085AA	4	76
ESC_RA7084AA	90	156
ESC_RA7083AA	10	512
ESC_RA7082AA	48	76
ESC_RA7081AA	280	142
ESC_RA7080AA	280	142
ESC_RA7079AA	10	512
ESC_RA7078AA	77	286
ESC_RA7077AA	3580	257
ESC_RA7076AA	280	142
ESC_RA7075AA	1408	445
ESC_RA7074AA	270	204
ESC_RA7073AA	533	198
ESC_RA7072AA	533	198
ESC_RA7071AA	155	175
ESC_RA7070AA	1408	445
ESC_RA7069AA	155	175
ESC_RA7068AA	155	175
ESC_RA7067AA	11201	257

ESC_RA7066AA	155	175
ESC_RA7065AA	8777	447
ESC_RA7064AA	1011	198
ESC_RA7063AA	1011	198
ESC_RA7062AA	297	234
ESC_RA7061AA	345	175
ESC_RA7060AA	297	234
ESC_RA7059AA	6186	868
ESC_RA7058AA	6186	868
ESC_RA7057AA	6186	868
ESC_RA7056AA	500	71
ESC_RA7055AA	15	766
ESC_RA7054AA	19	774
ESC_RA7053AA	95	738
ESC_RA7052AA	1312	76
ESC_RA7051AA	21	156
ESC_RA7050AA	95	738
ESC_RA7049AA	16	156
ESC_RA7048AA	2388	198
ESC_RA7047AA	716	795
ESC_RA7046AA	725	765
ESC_RA7045AA	1139	447
ESC_RA7044AA	618	166
ESC_RA7043AA	5502	307
ESC_RA7042AA	11	936
ESC_RA7041AA	73	737
ESC_RA7040AA	2350	235
ESC_RA7039AA	12	626
ESC_RA7038AA	200	198
ESC_RA7037AA	69	387
ESC_RA7036AA	136	392
ESC_RA7035AA	28	537
ESC_RA7034AA	847	257
ESC_RA7033AA	3337	512
ESC_RA7032AA	2352	198
ESC_RA7031AA	2338	257
ESC_RA7030AA	3090	212
ESC_RA7029AA	335	936
ESC_RA7028AA	106	387
ESC_RA7027AA	423	204
ESC_RA7026AA	69	387
ESC_RA7025AA	2337	670
ESC_RA7024AA	11	936
ESC_RA7023AA	11	936
ESC_RA7022AA	714	318
ESC_RA7021AA	19	774
ESC_RA7020AA	11	936
ESC_RA7019AA	349	670
ESC_RA7018AA	155	175
ESC_RA7017AA	12	626

ESC_RA7016AA	1952	880
ESC_RA7015AA	2230	126
ESC_RA7014AA	245	261
ESC_RA7013AA	152	257
ESC_RA7012AA	245	261
ESC_RA7011AA	152	257
ESC_RA7010AA	152	257
ESC_RA7009AA	245	261
ESC_RA7008AA	245	261
ESC_RA7007AA	245	261
ESC_RA7006AA	93	71
ESC_RA7005AA	10	512
ESC_RA7004AA	1508	261
ESC_RA7003AA	86	935
ESC_RA7002AA	1408	445
ESC_RA7001AA	10	512
ESC_RA7000AA	10	512
ESC_RA6999AA	10	512
ESC_RA6998AA	10	512
ESC_RA6997AA	10	512
ESC_RA6996AA	10	512
ESC_RA6995AA	10	512
ESC_RA6994AA	10	512
ESC_RA6992AA	4104	373
ESC_RA6991AA	2371	612
ESC_RA6990AA	10187	464
ESC_RA6989AA	10431	227
ESC_RA6988AA	8825	304
ESC_RA6987AA	372	424
ESC_RA6986AA	3616	532
ESC_RA6985AA	7966	460
ESC_RA6984AA	747	640
ESC_RA6983AA	1121	198
ESC_RA6982AA	167	512
ESC_RA6981AA	457	595
ESC_RA6980AA	131	653
ESC_RA6979AA	372	424
ESC_RA6978AA	657	592
ESC_RA6977AA	73	737
ESC_RA6976AA	3018	903
ESC_RA6975AA	648	359
ESC_RA6974AA	2141	597
ESC_RA6957AA	99	198
ESC_RA6956AA	783	204
ESC_RA6955AA	10	512
ESC_RA6954AA	8157	924
ESC_RA6942AA	583	768
ESC_RA6941AA	3233	936
ESC_RA6940AA	131	653
ESC_RA6939AA	131	653

ESC_RA6938AA	131	653
ESC_RA6937AA	131	653
ESC_RA6936AA	131	653
ESC_RA6935AA	95	738
ESC_RA6934AA	95	738
ESC_RA6933AA	2279	657
ESC_RA6932AA	2279	657
ESC_RA6931AA	10	512
ESC_RA6930AA	390	738
ESC_RA6929AA	21	156
ESC_RA6928AA	95	738
ESC_RA6927AA	95	738
ESC_RA6926AA	73	737
ESC_RA6924AA	95	738
ESC_RA6923AA	95	738
ESC_RA6922AA	131	653
ESC_RA6921AA	73	737
ESC_RA6920AA	95	738
ESC_RA6919AA	131	653
ESC_RA6918AA	73	737
ESC_RA6917AA	73	737
ESC_RA6916AA	131	653
ESC_RA6915AA	73	737
ESC_RA6914AA	73	737
ESC_RA6913AA	95	738
ESC_RA6912AA	131	653
ESC_RA6911AA	95	738
ESC_RA6910AA	95	738
ESC_RA6909AA	95	738
ESC_RA6908AA	95	738
ESC_RA6907AA	73	737
ESC_RA6906AA	95	738
ESC_RA6905AA	95	738
ESC_RA6904AA	131	653
ESC_RA6903AA	555	653
ESC_RA6902AA	69	387
ESC_RA6901AA	245	261
ESC_RA6900AA	245	261
ESC_RA6899AA	245	261
ESC_RA6898AA	245	261
ESC_RA6897AA	152	257
ESC_RA6896AA	152	257
ESC_RA6895AA	152	257
ESC_RA6894AA	152	257
ESC_RA6893AA	152	257
ESC_RA6892AA	152	257
ESC_RA6891AA	152	257
ESC_RA6890AA	152	257
ESC_RA6889AA	152	257
ESC_RA6888AA	152	257

ESC_RA6887AA	152	257
ESC_RA6886AA	152	257
ESC_RA6885AA	152	257
ESC_RA6884AA	152	257
ESC_RA6883AA	152	257
ESC_RA6882AA	152	257
ESC_RA6881AA	152	257
ESC_RA6880AA	152	257
ESC_RA6879AA	152	257
ESC_RA6878AA	152	257
ESC_RA6877AA	152	257
ESC_RA6876AA	152	257
ESC_RA6875AA	152	257
ESC_RA6874AA	152	257
ESC_RA6873AA	152	257
ESC_RA6872AA	152	257
ESC_RA6871AA	152	257
ESC_RA6870AA	152	257
ESC_RA6869AA	152	257
ESC_RA6868AA	152	257
ESC_RA6867AA	17	191
ESC_RA6865AA	11	936
ESC_RA6864AA	11	936
ESC_RA6863AA	11	936
ESC_RA6862AA	11	936
ESC_RA6861AA	11	936
ESC_RA6860AA	442	257
ESC_RA6859AA	11	936
ESC_RA6858AA	442	257
ESC_RA6857AA	442	257
ESC_RA6856AA	301	307
ESC_RA6855AA	11	936
ESC_RA6854AA	11	936
ESC_RA6853AA	11	936
ESC_RA6852AA	11	936
ESC_RA6851AA	335	936
ESC_RA6850AA	7010	198
ESC_RA6849AA	11	936
ESC_RA6848AA	17	191
ESC_RA6847AA	32	270
ESC_RA6846AA	442	257
ESC_RA6845AA	335	936
ESC_RA6844AA	335	936
ESC_RA6841AA	196	257
ESC_RA6840AA	388	156
ESC_RA6839AA	2766	198
ESC_RA6838AA	641	935
ESC_RA6837AA	491	626
ESC_RA6836AA	11	936
ESC_RA6835AA	11	936

ESC_RA6834AA	11	936
ESC_RA6833AA	11	936
ESC_RA6832AA	11	936
ESC_RA6831AA	17	191
ESC_RA6830AA	17	191
ESC_RA6829AA	11	936
ESC_RA6828AA	11	936
ESC_RA6827AA	11	936
ESC_RA6826AA	11	936
ESC_RA6825AA	11	936
ESC_RA6824AA	11	936
ESC_RA6823AA	11	936
ESC_RA6822AA	167	512
ESC_RA6821AA	405	923
ESC_RA6820AA	69	387
ESC_RA6819AA	167	512
ESC_RA6818AA	648	359
ESC_RA6817AA	940	257
ESC_RA6816AA	10	512
ESC_RA6815AA	167	512
ESC_RA6814AA	405	923
ESC_RA6813AA	648	359
ESC_RA6812AA	405	923
ESC_RA6807AA	7384	257
ESC_RA6806AA	152	257
ESC_RA6805AA	245	261
ESC_RA6804AA	245	261
ESC_RA6803AA	152	257
ESC_RA6802AA	245	261
ESC_RA6801AA	245	261
ESC_RA6800AA	245	261
ESC_RA6799AA	11194	935
ESC_RA6798AA	301	307
ESC_RA6797AA	21	156
ESC_RA6795AA	21	156
ESC_RA6794AA	21	156
ESC_RA6792AA	21	156
ESC_RA6791AA	11	936
ESC_RA6788AA	6745	885
ESC_RA6787AA	1684	504
ESC_RA6786AA	11	936
ESC_RA6785AA	11	936
ESC_RA6784AA	772	76
ESC_RA6782AA	11	936
ESC_RA6781AA	9421	198
ESC_RA6780AA	11075	257
ESC_RA6779AA	245	261
ESC_RA6778AA	11195	85
ESC_RA6777AA	131	653
ESC_RA6776AA	10	512

ESC_RA6775AA	101	696
ESC_RA6774AA	583	768
ESC_RA6773AA	10	512
ESC_RA6772AA	583	768
ESC_RA6771AA	11	936
ESC_RA6770AA	25	234
ESC_RA6769AA	245	261
ESC_RA6767AA	152	257
ESC_RA6766AA	245	261
ESC_RA6765AA	245	261
ESC_RA6764AA	245	261
ESC_RA6763AA	152	257
ESC_RA6762AA	152	257
ESC_RA6761AA	152	257
ESC_RA6760AA	245	261
ESC_RA6759AA	152	257
ESC_RA6758AA	152	257
ESC_RA6757AA	152	257
ESC_RA6756AA	245	261
ESC_RA6755AA	245	261
ESC_RA6754AA	245	261
ESC_RA6753AA	152	257
ESC_RA6752AA	245	261
ESC_RA6751AA	152	257
ESC_RA6750AA	152	257
ESC_RA6749AA	245	261
ESC_RA6748AA	152	257
ESC_RA6747AA	152	257
ESC_RA6746AA	152	257
ESC_RA6745AA	245	261
ESC_RA6744AA	152	257
ESC_RA6743AA	152	257
ESC_RA6742AA	152	257
ESC_RA6741AA	152	257
ESC_RA6740AA	152	257
ESC_RA6739AA	152	257
ESC_RA6738AA	245	261
ESC_RA6737AA	152	257
ESC_RA6736AA	152	257
ESC_RA6735AA	152	257
ESC_RA6734AA	245	261
ESC_RA6733AA	152	257
ESC_RA6732AA	152	257
ESC_RA6731AA	245	261
ESC_RA6730AA	245	261
ESC_RA6729AA	1503	257
ESC_RA6728AA	152	257
ESC_RA6727AA	245	261
ESC_RA6726AA	245	261
ESC_RA6725AA	152	257

ESC_RA6724AA	245	261
ESC_RA6723AA	245	261
ESC_RA6722AA	152	257
ESC_RA6721AA	152	257
ESC_RA6720AA	245	261
ESC_RA6719AA	245	261
ESC_RA6718AA	152	257
ESC_RA6717AA	152	257
ESC_RA6716AA	245	261
ESC_RA6715AA	245	261
ESC_RA6714AA	152	257
ESC_RA6713AA	152	257
ESC_RA6712AA	245	261
ESC_RA6711AA	152	257
ESC_RA6710AA	152	257
ESC_RA6709AA	245	261
ESC_RA6708AA	245	261
ESC_RA6707AA	245	261
ESC_RA6706AA	245	261
ESC_RA6705AA	245	261
ESC_RA6704AA	245	261
ESC_RA6703AA	280	142
ESC_RA6702AA	281	355
ESC_RA6701AA	7444	937
ESC_RA6700AA	88	160
ESC_RA6699AA	155	175
ESC_RA6698AA	101	696
ESC_RA6697AA	877	935
ESC_RA6696AA	58	198
ESC_RA6695AA	11	936
ESC_RA6694AA	11	936
ESC_RA6693AA	11	936
ESC_RA6692AA	11	936
ESC_RA6691AA	11	936
ESC_RA6690AA	11	936
ESC_RA6689AA	11	936
ESC_RA6688AA	11	936
ESC_RA6687AA	11	936
ESC_RA6686AA	11	936
ESC_RA6685AA	11	936
ESC_RA6684AA	11	936
ESC_RA6683AA	11	936
ESC_RA6682AA	1967	191
ESC_RA6681AA	11	936
ESC_RA6680AA	11	936
ESC_RA6679AA	11	936
ESC_RA6678AA	11	936
ESC_RA6677AA	11	936
ESC_RA6676AA	11	936
ESC_RA6675AA	11	936

ESC_RA6674AA	11	936
ESC_RA6673AA	11	936
ESC_RA6672AA	4627	204
ESC_RA6671AA	10	512
ESC_RA6670AA	279	142
ESC_RA6669AA	1101	842
ESC_RA6668AA	5288	261
ESC_RA6667AA	5288	261
ESC_RA6666AA	5288	261
ESC_RA6665AA	10334	935
ESC_RA6664AA	11	936
ESC_RA6663AA	11	936
ESC_RA6662AA	11	936
ESC_RA6661AA	11	936
ESC_RA6660AA	11	936
ESC_RA6659AA	11	936
ESC_RA6658AA	11	936
ESC_RA6657AA	343	286
ESC_RA6656AA	162	924
ESC_RA6655AA	1611	198
ESC_RA6654AA	7249	257
ESC_RA6653AA	1611	198
ESC_RA6652AA	95	738
ESC_RA6651AA	95	738
ESC_RA6650AA	95	738
ESC_RA6649AA	95	738
ESC_RA6648AA	95	738
ESC_RA6647AA	73	737
ESC_RA6646AA	95	738
ESC_RA6645AA	95	738
ESC_RA6644AA	73	737
ESC_RA6643AA	95	738
ESC_RA6642AA	95	738
ESC_RA6641AA	73	737
ESC_RA6640AA	95	738
ESC_RA6639AA	95	738
ESC_RA6638AA	95	738
ESC_RA6637AA	95	738
ESC_RA6636AA	95	738
ESC_RA6635AA	95	738
ESC_RA6634AA	5288	261
ESC_RA6633AA	5288	261
ESC_RA6632AA	5288	261
ESC_RA6631AA	5288	261
ESC_RA6630AA	5288	261
ESC_RA6629AA	355	764
ESC_RA6628AA	155	175
ESC_RA6627AA	117	592
ESC_RA6626AA	58	198
ESC_RA6625AA	224	198

ESC_RA6623AA	21	156
ESC_RA6622AA	21	156
ESC_RA6621AA	21	156
ESC_RA6620AA	11193	180
ESC_RA6619AA	101	696
ESC_RA6618AA	8463	198
ESC_RA6617AA	58	198
ESC_RA6616AA	56	198
ESC_RA6615AA	3042	663
ESC_RA6614AA	10	512
ESC_RA6613AA	3042	663
ESC_RA6612AA	392	234
ESC_RA6611AA	117	592
ESC_RA6610AA	56	198
ESC_RA6609AA	3057	672
ESC_RA6606AA	56	198
ESC_RA6605AA	10	512
ESC_RA6604AA	10	512
ESC_RA6603AA	5177	175
ESC_RA6602AA	657	592
ESC_RA6600AA	58	198
ESC_RA6599AA	58	198
ESC_RA6598AA	448	257
ESC_RA6596AA	11192	198
ESC_RA6595AA	10	512
ESC_RA6594AA	1807	160
ESC_RA6593AA	117	592
ESC_RA6591AA	10	512
ESC_RA6588AA	448	257
ESC_RA6587AA	5177	175
ESC_RA6586AA	4035	257
ESC_RA6585AA	392	234
ESC_RA6584AA	3057	672
ESC_RA6583AA	56	198
ESC_RA6582AA	616	156
ESC_RA6581AA	5177	175
ESC_RA6580AA	1087	235
ESC_RA6579AA	56	198
ESC_RA6578AA	117	592
ESC_RA6577AA	3042	663
ESC_RA6576AA	10	512
ESC_RA6575AA	641	935
ESC_RA6574AA	500	71
ESC_RA6573AA	10	512
ESC_RA6572AA	86	935
ESC_RA6571AA	1642	198
ESC_RA6570AA	1727	257
ESC_RA6569AA	99	198
ESC_RA6568AA	88	160
ESC_RA6567AA	165	504

ESC_RA6566AA	398	445
ESC_RA6565AA	164	175
ESC_RA6564AA	117	592
ESC_RA6563AA	11198	256
ESC_RA6562AA	162	924
ESC_RA6561AA	5759	109
ESC_RA6560AA	155	175
ESC_RA6559AA	109	257
ESC_RA6558AA	295	160
ESC_RA6557AA	681	393
ESC_RA6556AA	58	198
ESC_RA6555AA	9046	175
ESC_RA6554AA	10	512
ESC_RA6553AA	218	519
ESC_RA6552AA	4198	878
ESC_RA6551AA	88	160
ESC_RA6550AA	1718	269
ESC_RA6549AA	278	924
ESC_RA6548AA	339	0
ESC_RA6547AA	10	512
ESC_RA6546AA	10	512
ESC_RA6545AA	336	878
ESC_RA6544AA	10	512
ESC_RA6543AA	58	198
ESC_RA6542AA	117	592
ESC_RA6541AA	164	175
ESC_RA6540AA	101	696
ESC_RA6539AA	898	108
ESC_RA6538AA	189	504
ESC_RA6537AA	189	504
ESC_RA6536AA	11197	65
ESC_RA6535AA	58	198
ESC_RA6534AA	4358	71
ESC_RA6533AA	3107	512
ESC_RA6532AA	515	1004
ESC_RA6531AA	746	789
ESC_RA6530AA	1684	504
ESC_RA6529AA	1665	601
ESC_RA6528AA	154	257
ESC_RA6527AA	752	512
ESC_RA6526AA	58	198
ESC_RA6525AA	58	198
ESC_RA6524AA	101	696
ESC_RA6523AA	10	512
ESC_RA6522AA	1139	447
ESC_RA6521AA	1081	198
ESC_RA6520AA	5759	109
ESC_RA6519AA	361	512
ESC_RA6518AA	10	512
ESC_RA6517AA	1304	257

ESC_RA6516AA	278	924
ESC_RA6515AA	6506	914
ESC_RA6514AA	58	198
ESC_RA6513AA	58	198
ESC_RA6512AA	1684	504
ESC_RA6511AA	58	198
ESC_RA6510AA	155	175
ESC_RA6509AA	8712	512
ESC_RA6508AA	164	175
ESC_RA6507AA	1423	198
ESC_RA6506AA	108	257
ESC_RA6505AA	906	198
ESC_RA6504AA	2773	257
ESC_RA6503AA	297	234
ESC_RA6502AA	34	504
ESC_RA6501AA	227	504
ESC_RA6500AA	9263	198
ESC_RA6499AA	708	924
ESC_RA6498AA	101	696
ESC_RA6497AA	1147	198
ESC_RA6496AA	155	175
ESC_RA6495AA	10	512
ESC_RA6494AA	218	519
ESC_RA6493AA	69	387
ESC_RA6492AA	327	204
ESC_RA6491AA	29	156
ESC_RA6490AA	1121	198
ESC_RA6489AA	345	175
ESC_RA6488AA	1079	235
ESC_RA6487AA	607	355
ESC_RA6486AA	398	445
ESC_RA6485AA	372	424
ESC_RA6484AA	681	393
ESC_RA6483AA	218	519
ESC_RA6482AA	131	653
ESC_RA6481AA	906	198
ESC_RA6480AA	372	424
ESC_RA6479AA	297	234
ESC_RA6478AA	11196	311
ESC_RA6477AA	154	257
ESC_RA6476AA	155	175
ESC_RA6475AA	3525	504
ESC_RA6474AA	973	968
ESC_RA6473AA	117	592
ESC_RA6472AA	345	175
ESC_RA6471AA	2531	198
ESC_RA6470AA	3721	93
ESC_RA6469AA	602	257
ESC_RA6468AA	1850	269
ESC_RA6467AA	1946	726

ESC_RA6466AA	1564	512
ESC_RA6464AA	245	261
ESC_RA6463AA	245	261
ESC_RA6462AA	245	261
ESC_RA6461AA	245	261
ESC_RA6460AA	245	261
ESC_RA6459AA	718	257
ESC_RA6458AA	17	191
ESC_RA6457AA	21	156
ESC_RA6456AA	491	626
ESC_RA6455AA	442	257
ESC_RA6454AA	660	286
ESC_RA6453AA	17	191
ESC_RA6452AA	442	257
ESC_RA6451AA	11	936
ESC_RA6450AA	442	257
ESC_RA6449AA	7083	640
ESC_RA6448AA	442	257
ESC_RA6447AA	152	257
ESC_RA6446AA	152	257
ESC_RA6444AA	245	261
ESC_RA6443AA	245	261
ESC_RA6442AA	245	261
ESC_RA6441AA	245	261
ESC_RA6440AA	245	261
ESC_RA6439AA	245	261
ESC_RA6438AA	245	261
ESC_RA6437AA	245	261
ESC_RA6436AA	152	257
ESC_RA6435AA	1316	471
ESC_RA6434AA	152	257
ESC_RA6433AA	131	653
ESC_RA6432AA	648	359
ESC_RA6431AA	410	156
ESC_RA6430AA	160	257
ESC_RA6429AA	410	156
ESC_RA6428AA	46	447
ESC_RA6427AA	69	387
ESC_RA6426AA	405	923
ESC_RA6425AA	405	923
ESC_RA6424AA	361	512
ESC_RA6423AA	167	512
ESC_RA6422AA	167	512
ESC_RA6421AA	38	669
ESC_RA6420AA	227	504
ESC_RA6419AA	2083	257
ESC_RA6418AA	38	669
ESC_RA6417AA	167	512
ESC_RA6416AA	11	936
ESC_RA6415AA	11	936

ESC_RA6414AA	11	936
ESC_RA6413AA	245	261
ESC_RA6412AA	245	261
ESC_RA6411AA	245	261
ESC_RA6410AA	245	261
ESC_RA6409AA	245	261
ESC_RA6408AA	200	198
ESC_RA6407AA	2387	261
ESC_RA6406AA	10	512
ESC_RA6404AA	1485	408
ESC_RA6403AA	16	156
ESC_RA6402AA	17	191
ESC_RA6397AA	10	512
ESC_RA6396AA	405	923
ESC_RA6395AA	131	653
ESC_RA6394AA	11179	175
ESC_RA6393AA	23	156
ESC_RA6392AA	648	359
ESC_RA6389AA	10331	76
ESC_RA6388AA	744	512
ESC_RA6384AA	11	936
ESC_RA6383AA	11	936
ESC_RA6382AA	11	936
ESC_RA6381AA	11	936
ESC_RA6380AA	11	936
ESC_RA6379AA	11	936
ESC_RA6373AA	1316	471
ESC_RA6372AA	162	924
ESC_RA6369AA	16	156
ESC_RA6368AA	16	156
ESC_RA6367AA	16	156
ESC_RA6366AA	16	156
ESC_RA6365AA	16	156
ESC_RA6364AA	16	156
ESC_RA6363AA	16	156
ESC_RA6362AA	16	156
ESC_RA6360AA	16	156
ESC_RA6359AA	16	156
ESC_RA6358AA	16	156
ESC_RA6357AA	16	156
ESC_RA6356AA	16	156
ESC_RA6355AA	16	156
ESC_RA6354AA	16	156
ESC_RA6353AA	16	156
ESC_RA6352AA	16	156
ESC_RA6351AA	58	198
ESC_RA6350AA	16	156
ESC_RA6349AA	16	156
ESC_RA6348AA	394	400
ESC_RA6347AA	73	737

ESC_RA6346AA	1421	447
ESC_RA6345AA	131	653
ESC_RA6344AA	69	387
ESC_RA6343AA	69	387
ESC_RA6342AA	10	512
ESC_RA6341AA	10	512
ESC_RA6340AA	1193	761
ESC_RA6339AA	10	512
ESC_RA6338AA	38	669
ESC_RA6337AA	940	257
ESC_RA6336AA	69	387
ESC_RA6335AA	38	669
ESC_RA6334AA	131	653
ESC_RA6333AA	38	669
ESC_RA6332AA	131	653
ESC_RA6331AA	69	387
ESC_RA6330AA	761	512
ESC_RA6329AA	69	387
ESC_RA6328AA	58	198
ESC_RA6327AA	131	653
ESC_RA6326AA	120	180
ESC_RA6325AA	131	653
ESC_RA6324AA	120	180
ESC_RA6323AA	131	653
ESC_RA6322AA	12	626
ESC_RA6321AA	10	512
ESC_RA6320AA	131	653
ESC_RA6319AA	38	669
ESC_RA6318AA	1652	670
ESC_RA6317AA	10	512
ESC_RA6316AA	11183	314
ESC_RA6315AA	12	626
ESC_RA6314AA	3877	512
ESC_RA6313AA	648	359
ESC_RA6312AA	131	653
ESC_RA6311AA	12	626
ESC_RA6310AA	10	512
ESC_RA6309AA	372	424
ESC_RA6308AA	10	512
ESC_RA6307AA	10	512
ESC_RA6306AA	131	653
ESC_RA6305AA	120	180
ESC_RA6304AA	393	343
ESC_RA6303AA	44	512
ESC_RA6302AA	12	626
ESC_RA6301AA	93	71
ESC_RA6300AA	131	653
ESC_RA6299AA	131	653
ESC_RA6298AA	12	626
ESC_RA6297AA	131	653

ESC_RA6296AA	12	626
ESC_RA6295AA	131	653
ESC_RA6294AA	131	653
ESC_RA6293AA	394	400
ESC_RA6292AA	93	71
ESC_RA6291AA	69	387
ESC_RA6290AA	69	387
ESC_RA6289AA	38	669
ESC_RA6288AA	10	512
ESC_RA6287AA	1193	761
ESC_RA6286AA	10	512
ESC_RA6285AA	69	387
ESC_RA6284AA	12	626
ESC_RA6283AA	131	653
ESC_RA6282AA	131	653
ESC_RA6281AA	69	387
ESC_RA6280AA	998	644
ESC_RA6279AA	453	935
ESC_RA6278AA	131	653
ESC_RA6277AA	141	644
ESC_RA6276AA	131	653
ESC_RA6275AA	3877	512
ESC_RA6274AA	120	180
ESC_RA6273AA	10	512
ESC_RA6272AA	10	512
ESC_RA6271AA	11182	387
ESC_RA6270AA	38	669
ESC_RA6269AA	58	198
ESC_RA6268AA	38	669
ESC_RA6267AA	38	669
ESC_RA6266AA	131	653
ESC_RA6265AA	131	653
ESC_RA6264AA	131	653
ESC_RA6263AA	405	923
ESC_RA6262AA	617	511
ESC_RA6261AA	73	737
ESC_RA6260AA	95	738
ESC_RA6259AA	62	574
ESC_RA6258AA	10	512
ESC_RA6257AA	767	198
ESC_RA6256AA	73	737
ESC_RA6255AA	131	653
ESC_RA6254AA	131	653
ESC_RA6253AA	93	71
ESC_RA6252AA	3873	175
ESC_RA6251AA	10	512
ESC_RA6250AA	648	359
ESC_RA6249AA	1193	761
ESC_RA6248AA	10	512
ESC_RA6247AA	174	660

ESC_RA6246AA	10	512
ESC_RA6245AA	10	512
ESC_RA6244AA	10	512
ESC_RA6243AA	226	512
ESC_RA6242AA	746	789
ESC_RA6241AA	38	669
ESC_RA6240AA	93	71
ESC_RA6239AA	131	653
ESC_RA6238AA	10	512
ESC_RA6237AA	14	761
ESC_RA6235AA	617	511
ESC_RA6230AA	1193	761
ESC_RA6228AA	245	261
ESC_RA6227AA	28	537
ESC_RA6226AA	21	156
ESC_RA6225AA	11178	234
ESC_RA6224AA	21	156
ESC_RA6223AA	21	156
ESC_RA6222AA	21	156
ESC_RA6221AA	3944	512
ESC_RA6220AA	10	512
ESC_RA6219AA	1642	198
ESC_RA6218AA	302	850
ESC_RA6217AA	106	387
ESC_RA6216AA	278	924
ESC_RA6215AA	351	268
ESC_RA6214AA	399	198
ESC_RA6213AA	117	592
ESC_RA6212AA	5919	699
ESC_RA6211AA	106	387
ESC_RA6210AA	58	198
ESC_RA6209AA	117	592
ESC_RA6208AA	683	175
ESC_RA6207AA	69	387
ESC_RA6206AA	446	257
ESC_RA6205AA	4623	257
ESC_RA6204AA	58	198
ESC_RA6203AA	453	935
ESC_RA6202AA	446	257
ESC_RA6201AA	58	198
ESC_RA6200AA	1967	191
ESC_RA6198AA	23	156
ESC_RA6193AA	648	359
ESC_RA6190AA	1725	175
ESC_RA6188AA	11177	198
ESC_RA6184AA	205	257
ESC_RA6182AA	410	156
ESC_RA6181AA	542	824
ESC_RA6180AA	245	261
ESC_RA6179AA	32	270

ESC_RA6178AA	32	270
ESC_RA6177AA	21	156
ESC_RA6176AA	33	198
ESC_RA6175AA	25	234
ESC_RA6174AA	21	156
ESC_RA6173AA	4663	257
ESC_RA6172AA	1114	504
ESC_RA6171AA	1114	504
ESC_RA6169AA	4704	504
ESC_RA6168AA	1286	474
ESC_RA6165AA	1286	474
ESC_RA6164AA	1286	474
ESC_RA6162AA	167	512
ESC_RA6157AA	11176	512
ESC_RA6156AA	6448	175
ESC_RA6154AA	617	511
ESC_RA6153AA	617	511
ESC_RA6152AA	617	511
ESC_RA6151AA	617	511
ESC_RA6150AA	617	511
ESC_RA6149AA	617	511
ESC_RA6148AA	617	511
ESC_RA6147AA	617	511
ESC_RA6146AA	617	511
ESC_RA6145AA	617	511
ESC_RA6144AA	226	512
ESC_RA6143AA	226	512
ESC_RA6142AA	9461	504
ESC_RA6141AA	9461	504
ESC_RA6140AA	9461	504
ESC_RA6139AA	9461	504
ESC_RA6138AA	9461	504
ESC_RA6137AA	165	504
ESC_RA6136AA	9461	504
ESC_RA6135AA	9497	291
ESC_RA6134AA	542	824
ESC_RA6133AA	542	824
ESC_RA6132AA	93	71
ESC_RA6131AA	12	626
ESC_RA6130AA	58	198
ESC_RA6129AA	10	512
ESC_RA6128AA	88	160
ESC_RA6127AA	4421	257
ESC_RA6126AA	12	626
ESC_RA6125AA	1485	408
ESC_RA6124AA	683	175
ESC_RA6123AA	1170	629
ESC_RA6122AA	10	512
ESC_RA6121AA	117	592
ESC_RA6120AA	23	156

ESC_RA6119AA	2015	684
ESC_RA6118AA	8453	512
ESC_RA6116AA	115	669
ESC_RA6115AA	95	738
ESC_RA6114AA	95	738
ESC_RA6113AA	14	761
ESC_RA6112AA	14	761
ESC_RA6111AA	569	738
ESC_RA6110AA	569	738
ESC_RA6109AA	131	653
ESC_RA6108AA	1193	761
ESC_RA6107AA	1193	761
ESC_RA6106AA	569	738
ESC_RA6105AA	2619	738
ESC_RA6104AA	569	738
ESC_RA6103AA	80	731
ESC_RA6102AA	80	731
ESC_RA6101AA	80	731
ESC_RA6100AA	80	731
ESC_RA6099AA	372	424
ESC_RA6098AA	372	424
ESC_RA6097AA	131	653
ESC_RA6096AA	2619	738
ESC_RA6094AA	80	731
ESC_RA6093AA	372	424
ESC_RA6092AA	80	731
ESC_RA6091AA	95	738
ESC_RA6090AA	131	653
ESC_RA6089AA	14	761
ESC_RA6088AA	95	738
ESC_RA6087AA	131	653
ESC_RA6086AA	491	626
ESC_RA6085AA	491	626
ESC_RA6084AA	372	424
ESC_RA6083AA	95	738
ESC_RA6082AA	95	738
ESC_RA6081AA	90	156
ESC_RA6080AA	90	156
ESC_RA6078AA	90	156
ESC_RA6077AA	90	156
ESC_RA6076AA	90	156
ESC_RA6075AA	90	156
ESC_RA6074AA	90	156
ESC_RA6073AA	90	156
ESC_RA6072AA	10	512
ESC_RA6071AA	90	156
ESC_RA6070AA	11186	936
ESC_RA6069AA	11	936
ESC_RA6068AA	1804	935
ESC_RA6067AA	11	936

ESC_RA6066AA	11185	936
ESC_RA6063AA	11	936
ESC_RA6062AA	1804	935
ESC_RA6061AA	11	936
ESC_RA6060AA	617	511
ESC_RA6059AA	11184	257
ESC_RA6058AA	906	198
ESC_RA6057AA	101	696
ESC_RA6056AA	2792	886
ESC_RA6055AA	160	257
ESC_RA6054AA	2485	212
ESC_RA6053AA	2164	257
ESC_RA6052AA	75	257
ESC_RA6051AA	1423	198
ESC_RA6050AA	130	387
ESC_RA6049AA	10788	515
ESC_RA6048AA	1020	924
ESC_RA6047AA	73	737
ESC_RA6046AA	2144	257
ESC_RA6045AA	10	512
ESC_RA6044AA	155	175
ESC_RA6043AA	5442	512
ESC_RA6042AA	617	511
ESC_RA6041AA	617	511
ESC_RA6040AA	617	511
ESC_RA6039AA	617	511
ESC_RA6038AA	48	76
ESC_RA6037AA	1485	408
ESC_RA6035AA	245	261
ESC_RA6034AA	152	257
ESC_RA6033AA	245	261
ESC_RA6032AA	245	261
ESC_RA6031AA	245	261
ESC_RA6030AA	245	261
ESC_RA6029AA	38	669
ESC_RA6028AA	38	669
ESC_RA6027AA	410	156
ESC_RA6026AA	10	512
ESC_RA6025AA	8453	512
ESC_RA6024AA	48	76
ESC_RA6022AA	11	936
ESC_RA6021AA	11	936
ESC_RA6020AA	11	936
ESC_RA6019AA	11	936
ESC_RA6018AA	11	936
ESC_RA6017AA	11	936
ESC_RA6016AA	11	936
ESC_RA6015AA	11	936
ESC_RA6014AA	11	936
ESC_RA6013AA	11	936

ESC_RA6012AA	11	936
ESC_RA6011AA	11	936
ESC_RA6010AA	11	936
ESC_RA6009AA	11	936
ESC_RA6008AA	11	936
ESC_RA6007AA	11	936
ESC_RA6006AA	11	936
ESC_RA6005AA	11	936
ESC_RA6004AA	11	936
ESC_RA6003AA	32	270
ESC_RA6002AA	11	936
ESC_RA6001AA	11	936
ESC_RA6000AA	11	936
ESC_RA5999AA	11	936
ESC_RA5998AA	11	936
ESC_RA5997AA	11	936
ESC_RA5996AA	11	936
ESC_RA5995AA	28	537
ESC_RA5994AA	11	936
ESC_RA5993AA	11	936
ESC_RA5992AA	11	936
ESC_RA5991AA	442	257
ESC_RA5990AA	11	936
ESC_RA5989AA	11	936
ESC_RA5988AA	11	936
ESC_RA5987AA	297	234
ESC_RA5986AA	162	924
ESC_RA5985AA	164	175
ESC_RA5984AA	201	257
ESC_RA5983AA	1629	924
ESC_RA5982AA	117	592
ESC_RA5981AA	218	519
ESC_RA5980AA	4038	257
ESC_RA5979AA	69	387
ESC_RA5978AA	11190	924
ESC_RA5977AA	10	512
ESC_RA5976AA	106	387
ESC_RA5975AA	167	512
ESC_RA5974AA	17	191
ESC_RA5973AA	17	191
ESC_RA5972AA	655	926
ESC_RA5971AA	8265	153
ESC_RA5970AA	655	926
ESC_RA5969AA	165	504
ESC_RA5968AA	655	926
ESC_RA5967AA	11	936
ESC_RA5966AA	21	156
ESC_RA5965AA	11	936
ESC_RA5964AA	11	936
ESC_RA5963AA	11	936

ESC_RA5962AA	101	696
ESC_RA5961AA	101	696
ESC_RA5960AA	9406	512
ESC_RA5959AA	9378	519
ESC_RA5958AA	453	935
ESC_RA5957AA	10	512
ESC_RA5956AA	10	512
ESC_RA5955AA	1695	504
ESC_RA5954AA	101	696
ESC_RA5953AA	48	76
ESC_RA5952AA	10	512
ESC_RA5951AA	10	512
ESC_RA5950AA	641	935
ESC_RA5949AA	1258	257
ESC_RA5948AA	34	504
ESC_RA5947AA	6449	504
ESC_RA5946AA	641	935
ESC_RA5945AA	10	512
ESC_RA5944AA	542	824
ESC_RA5943AA	48	76
ESC_RA5942AA	641	935
ESC_RA5940AA	48	76
ESC_RA5939AA	131	653
ESC_RA5938AA	131	653
ESC_RA5937AA	46	447
ESC_RA5936AA	457	595
ESC_RA5935AA	11189	175
ESC_RA5934AA	131	653
ESC_RA5933AA	963	669
ESC_RA5932AA	7110	446
ESC_RA5931AA	131	653
ESC_RA5930AA	131	653
ESC_RA5929AA	940	257
ESC_RA5928AA	131	653
ESC_RA5927AA	131	653
ESC_RA5926AA	405	923
ESC_RA5925AA	131	653
ESC_RA5924AA	2003	669
ESC_RA5923AA	38	669
ESC_RA5922AA	131	653
ESC_RA5921AA	2083	257
ESC_RA5920AA	6784	701
ESC_RA5919AA	405	923
ESC_RA5918AA	617	511
ESC_RA5917AA	10	512
ESC_RA5916AA	90	156
ESC_RA5915AA	131	653
ESC_RA5914AA	38	669
ESC_RA5913AA	9525	519
ESC_RA5912AA	1722	599

ESC_RA5911AA	38	669
ESC_RA5910AA	131	653
ESC_RA5909AA	131	653
ESC_RA5908AA	38	669
ESC_RA5907AA	131	653
ESC_RA5906AA	131	653
ESC_RA5905AA	540	286
ESC_RA5904AA	38	669
ESC_RA5903AA	69	387
ESC_RA5902AA	38	669
ESC_RA5901AA	38	669
ESC_RA5900AA	131	653
ESC_RA5899AA	5584	640
ESC_RA5898AA	131	653
ESC_RA5897AA	131	653
ESC_RA5896AA	10	512
ESC_RA5895AA	1193	761
ESC_RA5894AA	11188	387
ESC_RA5892AA	131	653
ESC_RA5891AA	167	512
ESC_RA5890AA	5584	640
ESC_RA5889AA	131	653
ESC_RA5888AA	10	512
ESC_RA5887AA	1193	761
ESC_RA5886AA	10	512
ESC_RA5885AA	131	653
ESC_RA5884AA	648	359
ESC_RA5883AA	2614	975
ESC_RA5882AA	38	669
ESC_RA5881AA	131	653
ESC_RA5880AA	38	669
ESC_RA5879AA	131	653
ESC_RA5878AA	131	653
ESC_RA5877AA	131	653
ESC_RA5876AA	3018	903
ESC_RA5875AA	5455	512
ESC_RA5874AA	382	481
ESC_RA5873AA	940	257
ESC_RA5871AA	11	936
ESC_RA5870AA	11187	257
ESC_RA5869AA	1340	595
ESC_RA5868AA	88	160
ESC_RA5867AA	1340	595
ESC_RA5866AA	533	198
ESC_RA5865AA	1340	595
ESC_RA5864AA	1642	198
ESC_RA5863AA	11	936
ESC_RA5862AA	1967	191
ESC_RA5861AA	569	738
ESC_RA5860AA	95	738

ESC_RA5859AA	80	731
ESC_RA5858AA	550	756
ESC_RA5857AA	95	738
ESC_RA5856AA	550	756
ESC_RA5855AA	569	738
ESC_RA5854AA	95	738
ESC_RA5853AA	80	731
ESC_RA5852AA	1924	626
ESC_RA5851AA	11175	743
ESC_RA5850AA	1876	629
ESC_RA5849AA	95	738
ESC_RA5848AA	131	653
ESC_RA5847AA	131	653
ESC_RA5846AA	2682	87
ESC_RA5845AA	141	644
ESC_RA5844AA	4736	87
ESC_RA5843AA	2144	257
ESC_RA5842AA	73	737
ESC_RA5841AA	636	653
ESC_RA5840AA	73	737
ESC_RA5839AA	131	653
ESC_RA5838AA	131	653
ESC_RA5837AA	636	653
ESC_RA5836AA	95	738
ESC_RA5835AA	95	738
ESC_RA5834AA	95	738
ESC_RA5833AA	95	738
ESC_RA5832AA	11174	757
ESC_RA5831AA	11174	757
ESC_RA5830AA	91	629
ESC_RA5829AA	91	629
ESC_RA5828AA	131	653
ESC_RA5827AA	131	653
ESC_RA5826AA	131	653
ESC_RA5825AA	131	653
ESC_RA5824AA	73	737
ESC_RA5823AA	95	738
ESC_RA5822AA	131	653
ESC_RA5821AA	131	653
ESC_RA5820AA	73	737
ESC_RA5819AA	73	737
ESC_RA5818AA	131	653
ESC_RA5817AA	131	653
ESC_RA5816AA	95	738
ESC_RA5815AA	95	738
ESC_RA5814AA	95	738
ESC_RA5813AA	73	737
ESC_RA5812AA	131	653
ESC_RA5811AA	245	261
ESC_RA5810AA	245	261

ESC_RA5807AA	95	738
ESC_RA5806AA	11171	504
ESC_RA5805AA	11165	885
ESC_RA5804AA	10227	141
ESC_RA5803AA	11172	160
ESC_RA5802AA	11173	71
ESC_RA5800AA	245	261
ESC_RA5799AA	21	156
ESC_RA5798AA	21	156
ESC_RA5797AA	21	156
ESC_RA5795AA	38	669
ESC_RA5794AA	38	669
ESC_RA5793AA	38	669
ESC_RA5792AA	11	936
ESC_RA5791AA	11170	936
ESC_RA5790AA	11	936
ESC_RA5789AA	11	936
ESC_RA5788AA	11	936
ESC_RA5787AA	11	936
ESC_RA5786AA	11	936
ESC_RA5785AA	11	936
ESC_RA5784AA	877	935
ESC_RA5783AA	683	175
ESC_RA5782AA	1679	160
ESC_RA5781AA	23	156
ESC_RA5780AA	58	198
ESC_RA5779AA	11161	71
ESC_RA5778AA	10	512
ESC_RA5777AA	10	512
ESC_RA5776AA	898	108
ESC_RA5775AA	906	198
ESC_RA5774AA	457	595
ESC_RA5773AA	10	512
ESC_RA5772AA	295	160
ESC_RA5771AA	398	445
ESC_RA5770AA	963	669
ESC_RA5769AA	58	198
ESC_RA5768AA	609	447
ESC_RA5767AA	10	512
ESC_RA5766AA	10	512
ESC_RA5765AA	117	592
ESC_RA5764AA	2035	113
ESC_RA5763AA	68	386
ESC_RA5762AA	189	504
ESC_RA5761AA	11160	234
ESC_RA5759AA	393	343
ESC_RA5758AA	11151	512
ESC_RA5757AA	11	936
ESC_RA5756AA	11	936
ESC_RA5755AA	11	936

ESC_RA5754AA	11	936
ESC_RA5753AA	11	936
ESC_RA5752AA	11	936
ESC_RA5751AA	11	936
ESC_RA5750AA	11	936
ESC_RA5749AA	11	936
ESC_RA5748AA	11	936
ESC_RA5747AA	11	936
ESC_RA5746AA	11	936
ESC_RA5745AA	11	936
ESC_RA5744AA	11	936
ESC_RA5743AA	11	936
ESC_RA5742AA	11	936
ESC_RA5741AA	11	936
ESC_RA5740AA	11	936
ESC_RA5739AA	11	936
ESC_RA5738AA	11	936
ESC_RA5737AA	11	936
ESC_RA5736AA	11	936
ESC_RA5735AA	11	936
ESC_RA5734AA	11	936
ESC_RA5733AA	11	936
ESC_RA5732AA	11	936
ESC_RA5731AA	11	936
ESC_RA5730AA	11	936
ESC_RA5729AA	11	936
ESC_RA5728AA	11	936
ESC_RA5727AA	11	936
ESC_RA5726AA	11	936
ESC_RA5725AA	11	936
ESC_RA5723AA	88	160
ESC_RA5722AA	744	512
ESC_RA5721AA	69	387
ESC_RA5720AA	7652	512
ESC_RA5719AA	117	592
ESC_RA5718AA	607	355
ESC_RA5717AA	4198	878
ESC_RA5716AA	56	198
ESC_RA5715AA	58	198
ESC_RA5714AA	399	198
ESC_RA5713AA	10	512
ESC_RA5712AA	131	653
ESC_RA5711AA	131	653
ESC_RA5710AA	131	653
ESC_RA5709AA	131	653
ESC_RA5708AA	131	653
ESC_RA5707AA	1642	198
ESC_RA5706AA	973	968
ESC_RA5705AA	131	653
ESC_RA5704AA	393	343

ESC_RA5703AA	131	653
ESC_RA5702AA	1193	761
ESC_RA5701AA	38	669
ESC_RA5700AA	131	653
ESC_RA5699AA	69	387
ESC_RA5698AA	58	198
ESC_RA5697AA	131	653
ESC_RA5696AA	131	653
ESC_RA5695AA	90	156
ESC_RA5694AA	131	653
ESC_RA5693AA	131	653
ESC_RA5692AA	131	653
ESC_RA5691AA	131	653
ESC_RA5690AA	648	359
ESC_RA5689AA	23	156
ESC_RA5688AA	744	512
ESC_RA5687AA	69	387
ESC_RA5686AA	69	387
ESC_RA5685AA	131	653
ESC_RA5684AA	58	198
ESC_RA5683AA	1011	198
ESC_RA5682AA	73	737
ESC_RA5681AA	58	198
ESC_RA5680AA	1193	761
ESC_RA5679AA	131	653
ESC_RA5678AA	131	653
ESC_RA5677AA	10	512
ESC_RA5676AA	131	653
ESC_RA5675AA	538	629
ESC_RA5674AA	131	653
ESC_RA5673AA	11155	550
ESC_RA5672AA	2936	515
ESC_RA5671AA	131	653
ESC_RA5670AA	131	653
ESC_RA5669AA	131	653
ESC_RA5668AA	1480	160
ESC_RA5667AA	405	923
ESC_RA5666AA	131	653
ESC_RA5665AA	405	923
ESC_RA5664AA	10	512
ESC_RA5663AA	131	653
ESC_RA5662AA	131	653
ESC_RA5661AA	69	387
ESC_RA5660AA	11154	592
ESC_RA5659AA	648	359
ESC_RA5658AA	131	653
ESC_RA5657AA	648	359
ESC_RA5655AA	6332	153
ESC_RA5654AA	9748	653
ESC_RA5653AA	1722	599

ESC_RA5652AA	744	512
ESC_RA5651AA	88	160
ESC_RA5650AA	48	76
ESC_RA5649AA	11153	79
ESC_RA5648AA	10	512
ESC_RA5647AA	10	512
ESC_RA5646AA	69	387
ESC_RA5645AA	1080	198
ESC_RA5644AA	7601	108
ESC_RA5643AA	2230	126
ESC_RA5642AA	38	669
ESC_RA5641AA	278	924
ESC_RA5640AA	906	198
ESC_RA5639AA	10	512
ESC_RA5638AA	88	160
ESC_RA5637AA	617	511
ESC_RA5636AA	1122	307
ESC_RA5635AA	127	738
ESC_RA5634AA	1434	512
ESC_RA5633AA	162	924
ESC_RA5632AA	58	198
ESC_RA5631AA	10	512
ESC_RA5630AA	5229	696
ESC_RA5629AA	162	924
ESC_RA5628AA	6404	257
ESC_RA5627AA	2521	257
ESC_RA5626AA	155	175
ESC_RA5625AA	607	355
ESC_RA5624AA	399	198
ESC_RA5623AA	101	696
ESC_RA5622AA	345	175
ESC_RA5621AA	3714	175
ESC_RA5620AA	278	924
ESC_RA5619AA	11152	512
ESC_RA5618AA	3580	257
ESC_RA5617AA	117	592
ESC_RA5616AA	10	512
ESC_RA5615AA	223	198
ESC_RA5614AA	16	156
ESC_RA5613AA	152	257
ESC_RA5612AA	245	261
ESC_RA5611AA	245	261
ESC_RA5610AA	201	257
ESC_RA5609AA	1494	180
ESC_RA5608AA	3519	101
ESC_RA5607AA	11	936
ESC_RA5606AA	301	307
ESC_RA5605AA	301	307
ESC_RA5604AA	21	156
ESC_RA5603AA	21	156

ESC_RA5602AA	21	156
ESC_RA5601AA	21	156
ESC_RA5600AA	2622	537
ESC_RA5599AA	12	626
ESC_RA5598AA	162	924
ESC_RA5597AA	372	424
ESC_RA5596AA	372	424
ESC_RA5595AA	7724	175
ESC_RA5594AA	372	424
ESC_RA5593AA	162	924
ESC_RA5592AA	5597	72
ESC_RA5591AA	372	424
ESC_RA5590AA	127	738
ESC_RA5589AA	73	737
ESC_RA5588AA	372	424
ESC_RA5587AA	372	424
ESC_RA5586AA	73	737
ESC_RA5585AA	646	738
ESC_RA5584AA	2015	684
ESC_RA5583AA	372	424
ESC_RA5582AA	131	653
ESC_RA5581AA	1643	780
ESC_RA5580AA	372	424
ESC_RA5579AA	12	626
ESC_RA5578AA	12	626
ESC_RA5577AA	127	738
ESC_RA5576AA	73	737
ESC_RA5575AA	12	626
ESC_RA5574AA	372	424
ESC_RA5573AA	372	424
ESC_RA5572AA	17	191
ESC_RA5571AA	998	644
ESC_RA5570AA	11159	267
ESC_RA5569AA	6520	653
ESC_RA5568AA	11	936
ESC_RA5567AA	3594	924
ESC_RA5566AA	126	740
ESC_RA5565AA	2556	630
ESC_RA5564AA	117	592
ESC_RA5563AA	101	696
ESC_RA5562AA	401	142
ESC_RA5561AA	10061	512
ESC_RA5560AA	48	76
ESC_RA5559AA	10	512
ESC_RA5558AA	48	76
ESC_RA5557AA	8576	212
ESC_RA5556AA	457	595
ESC_RA5555AA	410	156
ESC_RA5554AA	126	740
ESC_RA5553AA	2175	212

ESC_RA5552AA	48	76
ESC_RA5551AA	162	924
ESC_RA5550AA	3580	257
ESC_RA5549AA	58	198
ESC_RA5548AA	11158	235
ESC_RA5547AA	9587	387
ESC_RA5545AA	131	653
ESC_RA5544AA	11150	601
ESC_RA5543AA	245	261
ESC_RA5542AA	245	261
ESC_RA5541AA	152	257
ESC_RA5540AA	245	261
ESC_RA5539AA	152	257
ESC_RA5538AA	245	261
ESC_RA5537AA	245	261
ESC_RA5536AA	5968	519
ESC_RA5535AA	216	512
ESC_RA5534AA	11157	924
ESC_RA5533AA	2705	504
ESC_RA5532AA	4038	257
ESC_RA5531AA	2954	908
ESC_RA5530AA	10	512
ESC_RA5529AA	708	924
ESC_RA5528AA	212	198
ESC_RA5527AA	11156	787
ESC_RA5526AA	155	175
ESC_RA5525AA	297	234
ESC_RA5524AA	155	175
ESC_RA5523AA	5273	682
ESC_RA5522AA	297	234
ESC_RA5521AA	191	198
ESC_RA5520AA	5273	682
ESC_RA5519AA	11	936
ESC_RA5518AA	11	936
ESC_RA5517AA	11	936
ESC_RA5516AA	1804	935
ESC_RA5515AA	11	936
ESC_RA5514AA	1804	935
ESC_RA5513AA	11	936
ESC_RA5512AA	11	936
ESC_RA5511AA	11	936
ESC_RA5510AA	11	936
ESC_RA5509AA	11	936
ESC_RA5508AA	11	936
ESC_RA5507AA	5855	519
ESC_RA5506AA	4956	198
ESC_RA5505AA	5870	504
ESC_RA5504AA	877	935
ESC_RA5503AA	10	512
ESC_RA5502AA	10	512

ESC_RA5501AA	6775	1004
ESC_RA5500AA	165	504
ESC_RA5499AA	515	1004
ESC_RA5498AA	515	1004
ESC_RA5497AA	6214	512
ESC_RA5496AA	515	1004
ESC_RA5495AA	877	935
ESC_RA5494AA	6018	52
ESC_RA5493AA	48	76
ESC_RA5492AA	2040	198
ESC_RA5491AA	162	924
ESC_RA5490AA	7153	263
ESC_RA5488AA	10645	519
ESC_RA5485AA	515	1004
ESC_RA5484AA	165	504
ESC_RA5483AA	2170	234
ESC_RA5481AA	2705	504
ESC_RA5480AA	1147	198
ESC_RA5479AA	69	387
ESC_RA5478AA	5044	1004
ESC_RA5477AA	1163	579
ESC_RA5476AA	196	257
ESC_RA5475AA	68	386
ESC_RA5474AA	351	268
ESC_RA5473AA	167	512
ESC_RA5472AA	10603	512
ESC_RA5471AA	1722	599
ESC_RA5470AA	1196	257
ESC_RA5469AA	278	924
ESC_RA5468AA	744	512
ESC_RA5467AA	1011	198
ESC_RA5466AA	1121	198
ESC_RA5465AA	410	156
ESC_RA5464AA	10	512
ESC_RA5463AA	1141	512
ESC_RA5462AA	394	400
ESC_RA5461AA	345	175
ESC_RA5460AA	10	512
ESC_RA5459AA	29	156
ESC_RA5458AA	5229	696
ESC_RA5457AA	156	175
ESC_RA5456AA	1286	474
ESC_RA5455AA	2170	234
ESC_RA5454AA	10	512
ESC_RA5453AA	2935	512
ESC_RA5452AA	226	512
ESC_RA5451AA	641	935
ESC_RA5450AA	101	696
ESC_RA5449AA	4956	198
ESC_RA5448AA	515	1004

ESC_RA5447AA	1121	198
ESC_RA5446AA	4238	512
ESC_RA5445AA	877	935
ESC_RA5444AA	34	504
ESC_RA5443AA	484	71
ESC_RA5442AA	34	504
ESC_RA5441AA	1722	599
ESC_RA5440AA	10	512
ESC_RA5439AA	10602	198
ESC_RA5438AA	192	696
ESC_RA5437AA	38	669
ESC_RA5436AA	38	669
ESC_RA5435AA	2170	234
ESC_RA5434AA	10499	387
ESC_RA5433AA	665	79
ESC_RA5432AA	665	79
ESC_RA5431AA	665	79
ESC_RA5430AA	665	79
ESC_RA5429AA	665	79
ESC_RA5428AA	665	79
ESC_RA5427AA	86	935
ESC_RA5426AA	86	935
ESC_RA5425AA	6856	76
ESC_RA5424AA	131	653
ESC_RA5423AA	131	653
ESC_RA5422AA	973	968
ESC_RA5421AA	973	968
ESC_RA5420AA	156	175
ESC_RA5417AA	11140	76
ESC_RA5416AA	48	76
ESC_RA5414AA	88	160
ESC_RA5413AA	93	71
ESC_RA5412AA	410	156
ESC_RA5411AA	410	156
ESC_RA5410AA	410	156
ESC_RA5409AA	410	156
ESC_RA5408AA	410	156
ESC_RA5407AA	410	156
ESC_RA5406AA	410	156
ESC_RA5405AA	410	156
ESC_RA5404AA	410	156
ESC_RA5403AA	410	156
ESC_RA5402AA	410	156
ESC_RA5401AA	410	156
ESC_RA5317AA	12	626
ESC_RA5316AA	12	626
ESC_RA5315AA	429	654
ESC_RA5314AA	8190	175
ESC_RA5313AA	12	626
ESC_RA5312AA	641	935

ESC_RA5311AA	357	654
ESC_RA5310AA	355	764
ESC_RA5309AA	453	935
ESC_RA5308AA	99	198
ESC_RA5307AA	1674	595
ESC_RA5306AA	131	653
ESC_RA5305AA	73	737
ESC_RA5303AA	1140	934
ESC_RA5302AA	405	923
ESC_RA5301AA	636	653
ESC_RA5300AA	1485	408
ESC_RA5299AA	617	511
ESC_RA5298AA	88	160
ESC_RA5297AA	131	653
ESC_RA5296AA	354	15
ESC_RA5295AA	778	638
ESC_RA5294AA	73	737
ESC_RA5293AA	69	387
ESC_RA5292AA	1485	408
ESC_RA5291AA	73	737
ESC_RA5290AA	10	512
ESC_RA5289AA	131	653
ESC_RA5288AA	405	923
ESC_RA5287AA	8371	898
ESC_RA5286AA	88	160
ESC_RA5285AA	429	654
ESC_RA5284AA	117	592
ESC_RA5283AA	429	654
ESC_RA5282AA	1196	257
ESC_RA5281AA	38	669
ESC_RA5280AA	88	160
ESC_RA5279AA	38	669
ESC_RA5278AA	357	654
ESC_RA5277AA	38	669
ESC_RA5276AA	131	653
ESC_RA5275AA	1196	257
ESC_RA5274AA	224	198
ESC_RA5273AA	11164	198
ESC_RA5272AA	372	424
ESC_RA5271AA	744	512
ESC_RA5270AA	429	654
ESC_RA5269AA	131	653
ESC_RA5268AA	23	156
ESC_RA5267AA	73	737
ESC_RA5266AA	11169	738
ESC_RA5265AA	648	359
ESC_RA5264AA	131	653
ESC_RA5263AA	2178	924
ESC_RA5262AA	2509	257
ESC_RA5261AA	345	175

ESC_RA5260AA	219	877
ESC_RA5259AA	963	669
ESC_RA5258AA	405	923
ESC_RA5257AA	410	156
ESC_RA5256AA	88	160
ESC_RA5255AA	69	387
ESC_RA5254AA	11168	15
ESC_RA5253AA	2165	257
ESC_RA5252AA	349	670
ESC_RA5251AA	404	761
ESC_RA5250AA	69	387
ESC_RA5249AA	93	71
ESC_RA5248AA	12	626
ESC_RA5247AA	73	737
ESC_RA5246AA	7937	198
ESC_RA5245AA	429	654
ESC_RA5243AA	8977	198
ESC_RA5242AA	38	669
ESC_RA5241AA	1485	408
ESC_RA5240AA	429	654
ESC_RA5239AA	101	696
ESC_RA5238AA	38	669
ESC_RA5237AA	711	935
ESC_RA5236AA	6836	544
ESC_RA5235AA	429	654
ESC_RA5234AA	118	985
ESC_RA5233AA	12	626
ESC_RA5232AA	101	696
ESC_RA5231AA	394	400
ESC_RA5230AA	5150	661
ESC_RA5229AA	131	653
ESC_RA5228AA	69	387
ESC_RA5227AA	38	669
ESC_RA5226AA	95	738
ESC_RA5225AA	648	359
ESC_RA5224AA	973	968
ESC_RA5223AA	38	669
ESC_RA5222AA	69	387
ESC_RA5221AA	34	504
ESC_RA5220AA	69	387
ESC_RA5219AA	200	198
ESC_RA5218AA	11167	626
ESC_RA5217AA	135	653
ESC_RA5216AA	11166	900
ESC_RA5215AA	5358	453
ESC_RA5214AA	73	737
ESC_RA5213AA	11163	738
ESC_RA5212AA	131	653
ESC_RA5211AA	38	669
ESC_RA5210AA	73	737

ESC_RA5209AA	963	669
ESC_RA5208AA	1193	761
ESC_RA5207AA	1236	380
ESC_RA5206AA	69	387
ESC_RA5205AA	429	654
ESC_RA5204AA	1126	198
ESC_RA5203AA	93	71
ESC_RA5202AA	131	653
ESC_RA5201AA	69	387
ESC_RA5200AA	359	696
ESC_RA5199AA	38	669
ESC_RA5198AA	429	654
ESC_RA5197AA	1594	71
ESC_RA5196AA	1137	512
ESC_RA5195AA	117	592
ESC_RA5194AA	38	669
ESC_RA5193AA	69	387
ESC_RA5192AA	448	257
ESC_RA5191AA	131	653
ESC_RA5190AA	131	653
ESC_RA5189AA	131	653
ESC_RA5188AA	69	387
ESC_RA5187AA	963	669
ESC_RA5186AA	38	669
ESC_RA5185AA	206	286
ESC_RA5184AA	58	198
ESC_RA5183AA	767	198
ESC_RA5182AA	1431	257
ESC_RA5181AA	57	50
ESC_RA5180AA	429	654
ESC_RA5179AA	38	669
ESC_RA5178AA	707	257
ESC_RA5177AA	1423	198
ESC_RA5176AA	2015	684
ESC_RA5174AA	167	512
ESC_RA5173AA	345	175
ESC_RA5172AA	88	160
ESC_RA5171AA	11162	32
ESC_RA5170AA	95	738
ESC_RA5169AA	141	644
ESC_RA5168AA	746	789
ESC_RA5167AA	141	644
ESC_RA5166AA	88	160
ESC_RA5165AA	73	737
ESC_RA5164AA	88	160
ESC_RA5163AA	12	626
ESC_RA5162AA	131	653
ESC_RA5161AA	95	738
ESC_RA5160AA	131	653
ESC_RA5159AA	73	737

ESC_RA5158AA	1057	761
ESC_RA5157AA	1589	156
ESC_RA5156AA	2328	257
ESC_RA5155AA	73	737
ESC_RA5154AA	394	400
ESC_RA5153AA	95	738
ESC_RA5152AA	973	968
ESC_RA5151AA	968	737
ESC_RA5150AA	73	737
ESC_RA5149AA	349	670
ESC_RA5148AA	390	738
ESC_RA5147AA	73	737
ESC_RA5146AA	245	261
ESC_RA5145AA	245	261
ESC_RA5144AA	245	261
ESC_RA5143AA	607	355
ESC_RA5142AA	327	204
ESC_RA5141AA	10	512
ESC_RA5140AA	1071	175
ESC_RA5139AA	8147	850
ESC_RA5138AA	746	789
ESC_RA5137AA	11149	76
ESC_RA5136AA	11147	789
ESC_RA5135AA	11148	71
ESC_RA5134AA	11146	291
ESC_RA5133AA	1159	381
ESC_RA5131AA	1159	381
ESC_RA5130AA	1159	381
ESC_RA5129AA	1159	381
ESC_RA5128AA	1159	381
ESC_RA5127AA	1159	381
ESC_RA5126AA	1159	381
ESC_RA5125AA	1159	381
ESC_RA5124AA	1159	381
ESC_RA5123AA	1159	381
ESC_RA5122AA	1159	381
ESC_RA5121AA	1159	381
ESC_RA5120AA	1159	381
ESC_RA5119AA	1159	381
ESC_RA5118AA	1159	381
ESC_RA5117AA	1159	381
ESC_RA5116AA	1159	381
ESC_RA5115AA	1159	381
ESC_RA5113AA	1159	381
ESC_RA5112AA	1159	381
ESC_RA5111AA	1159	381
ESC_RA5110AA	1159	381
ESC_RA5109AA	1159	381
ESC_RA5108AA	1159	381
ESC_RA5107AA	1159	381

ESC_RA5106AA	1159	381
ESC_RA5105AA	1159	381
ESC_RA5104AA	1159	381
ESC_RA5103AA	1159	381
ESC_RA5102AA	1159	381
ESC_RA5101AA	1159	381
ESC_RA5100AA	1159	381
ESC_RA5099AA	1159	381
ESC_RA5098AA	2179	924
ESC_RA5097AA	38	669
ESC_RA5096AA	4684	257
ESC_RA5095AA	101	696
ESC_RA5094AA	38	669
ESC_RA5093AA	1771	868
ESC_RA5092AA	38	669
ESC_RA5090AA	43	512
ESC_RA5089AA	1159	381
ESC_RA5088AA	711	935
ESC_RA5087AA	711	935
ESC_RA5086AA	69	387
ESC_RA5085AA	1159	381
ESC_RA5084AA	1159	381
ESC_RA5083AA	11143	670
ESC_RA5082AA	11142	387
ESC_RA5081AA	11141	387
ESC_RA5080AA	2350	235
ESC_RA5079AA	141	644
ESC_RA5078AA	11145	71
ESC_RA5077AA	648	359
ESC_RA5076AA	648	359
ESC_RA5074AA	648	359
ESC_RA5073AA	648	359
ESC_RA5072AA	648	359
ESC_RA5071AA	648	359
ESC_RA5070AA	1421	447
ESC_RA5069AA	11144	670
ESC_RA5068AA	135	653
ESC_RA5067AA	744	512
ESC_RA5066AA	660	286
ESC_RA5065AA	660	286
ESC_RA5064AA	457	595
ESC_RA5063AA	457	595
ESC_RA5062AA	457	595
ESC_RA5061AA	457	595
ESC_RA5060AA	457	595
ESC_RA5059AA	457	595
ESC_RA5058AA	457	595
ESC_RA5057AA	457	595
ESC_RA5056AA	457	595
ESC_RA5055AA	457	595

ESC_RA5054AA	457	595
ESC_RA5053AA	457	595
ESC_RA5052AA	457	595
ESC_RA5051AA	457	595
ESC_RA5050AA	457	595
ESC_RA5049AA	457	595
ESC_RA5048AA	457	595
ESC_RA5047AA	457	595
ESC_RA5046AA	457	595
ESC_RA5045AA	457	595
ESC_RA5044AA	457	595
ESC_RA5043AA	457	595
ESC_RA5042AA	457	595
ESC_RA5041AA	457	595
ESC_RA5040AA	457	595
ESC_RA5039AA	457	595
ESC_RA5038AA	457	595
ESC_RA5037AA	457	595
ESC_RA5036AA	457	595
ESC_RA5035AA	457	595
ESC_RA5034AA	457	595
ESC_RA5033AA	457	595
ESC_RA5032AA	457	595
ESC_RA5031AA	457	595
ESC_RA5030AA	457	595
ESC_RA5029AA	457	595
ESC_RA5028AA	457	595
ESC_RA5027AA	457	595
ESC_RA5026AA	457	595
ESC_RA5025AA	457	595
ESC_RA5024AA	457	595
ESC_RA5023AA	457	595
ESC_RA5022AA	457	595
ESC_RA5021AA	457	595
ESC_RA5020AA	457	595
ESC_RA5019AA	457	595
ESC_RA5018AA	457	595
ESC_RA5017AA	457	595
ESC_RA5016AA	457	595
ESC_RA5015AA	457	595
ESC_RA5014AA	457	595
ESC_RA5013AA	457	595
ESC_RA5012AA	457	595
ESC_RA5011AA	457	595
ESC_RA5010AA	457	595
ESC_RA5009AA	145	256
ESC_RA5008AA	152	257
ESC_RA5007AA	245	261
ESC_RA5006AA	152	257
ESC_RA5005AA	152	257

ESC_RA5004AA	245	261
ESC_RA5003AA	7384	257
ESC_RA5002AA	152	257
ESC_RA5001AA	245	261
ESC_RA5000AA	152	257
ESC_RA4999AA	297	234
ESC_RA4998AA	245	261
ESC_RA4997AA	5236	257
ESC_RA4996AA	11139	160
ESC_RA4995AA	58	198
ESC_RA4994AA	10	512
ESC_RA4993AA	21	156
ESC_RA4992AA	11138	198
ESC_RA4991AA	109	257
ESC_RA4990AA	641	935
ESC_RA4989AA	218	519
ESC_RA4988AA	68	386
ESC_RA4987AA	442	257
ESC_RA4986AA	738	598
ESC_RA4985AA	11137	286
ESC_RA4984AA	13	257
ESC_RA4983AA	88	160
ESC_RA4982AA	5419	175
ESC_RA4981AA	10	512
ESC_RA4980AA	245	261
ESC_RA4979AA	245	261
ESC_RA4978AA	245	261
ESC_RA4977AA	245	261
ESC_RA4976AA	21	156
ESC_RA4975AA	11	936
ESC_RA4974AA	14	761
ESC_RA4973AA	306	286
ESC_RA4972AA	11	936
ESC_RA4971AA	33	198
ESC_RA4970AA	738	598
ESC_RA4968AA	167	512
ESC_RA4967AA	88	160
ESC_RA4966AA	2329	924
ESC_RA4965AA	58	198
ESC_RA4964AA	11136	869
ESC_RA4963AA	8431	160
ESC_RA4962AA	2035	113
ESC_RA4961AA	10613	160
ESC_RA4960AA	1125	198
ESC_RA4959AA	58	198
ESC_RA4958AA	6024	175
ESC_RA4957AA	1087	235
ESC_RA4956AA	155	175
ESC_RA4955AA	117	592
ESC_RA4954AA	442	257

ESC_RA4953AA	88	160
ESC_RA4952AA	1303	791
ESC_RA4951AA	744	512
ESC_RA4950AA	3889	291
ESC_RA4949AA	4379	878
ESC_RA4948AA	2165	257
ESC_RA4947AA	10	512
ESC_RA4946AA	117	592
ESC_RA4945AA	11135	447
ESC_RA4944AA	10	512
ESC_RA4943AA	617	511
ESC_RA4942AA	636	653
ESC_RA4941AA	11135	447
ESC_RA4940AA	11133	268
ESC_RA4939AA	11134	855
ESC_RA4938AA	11132	595
ESC_RA4937AA	11	936
ESC_RA4936AA	11	936
ESC_RA4935AA	11	936
ESC_RA4934AA	11	936
ESC_RA4933AA	11	936
ESC_RA4932AA	11	936
ESC_RA4931AA	11	936
ESC_RA4930AA	11	936
ESC_RA4929AA	11	936
ESC_RA4928AA	11	936
ESC_RA4927AA	11	936
ESC_RA4926AA	11	936
ESC_RA4925AA	11	936
ESC_RA4924AA	11	936
ESC_RA4923AA	11	936
ESC_RA4922AA	11	936
ESC_RA4921AA	11	936
ESC_RA4920AA	11	936
ESC_RA4919AA	11	936
ESC_RA4918AA	11	936
ESC_RA4917AA	11	936
ESC_RA4916AA	11	936
ESC_RA4915AA	11	936
ESC_RA4914AA	11	936
ESC_RA4913AA	11	936
ESC_RA4912AA	11	936
ESC_RA4911AA	11	936
ESC_RA4910AA	11	936
ESC_RA4909AA	11	936
ESC_RA4908AA	11	936
ESC_RA4907AA	11	936
ESC_RA4906AA	11	936
ESC_RA4905AA	11	936
ESC_RA4904AA	11	936

ESC_RA4903AA	11	936
ESC_RA4902AA	11	936
ESC_RA4901AA	11	936
ESC_RA4900AA	11	936
ESC_RA4899AA	11	936
ESC_RA4898AA	11	936
ESC_RA4897AA	11	936
ESC_RA4896AA	11	936
ESC_RA4895AA	11	936
ESC_RA4894AA	11	936
ESC_RA4893AA	11	936
ESC_RA4892AA	11	936
ESC_RA4891AA	11	936
ESC_RA4890AA	11	936
ESC_RA4889AA	10326	175
ESC_RA4888AA	683	175
ESC_RA4886AA	245	261
ESC_RA4884AA	11131	406
ESC_RA4883AA	38	669
ESC_RA4882AA	46	447
ESC_RA4881AA	14	761
ESC_RA4880AA	58	198
ESC_RA4879AA	131	653
ESC_RA4878AA	1196	257
ESC_RA4877AA	69	387
ESC_RA4876AA	1858	757
ESC_RA4875AA	1858	757
ESC_RA4874AA	11130	924
ESC_RA4873AA	718	257
ESC_RA4872AA	993	789
ESC_RA4871AA	10	512
ESC_RA4870AA	58	198
ESC_RA4869AA	10	512
ESC_RA4868AA	223	198
ESC_RA4867AA	58	198
ESC_RA4866AA	295	160
ESC_RA4865AA	58	198
ESC_RA4864AA	10897	175
ESC_RA4863AA	442	257
ESC_RA4862AA	515	1004
ESC_RA4861AA	155	175
ESC_RA4860AA	11129	257
ESC_RA4859AA	1071	175
ESC_RA4857AA	56	198
ESC_RA4856AA	88	160
ESC_RA4855AA	332	257
ESC_RA4852AA	11128	520
ESC_RA4849AA	2253	71
ESC_RA4848AA	641	935
ESC_RA4847AA	88	160

ESC_RA4846AA	131	653
ESC_RA4845AA	34	504
ESC_RA4844AA	137	270
ESC_RA4843AA	131	653
ESC_RA4842AA	137	270
ESC_RA4841AA	345	175
ESC_RA4840AA	34	504
ESC_RA4839AA	10	512
ESC_RA4838AA	10923	504
ESC_RA4837AA	744	512
ESC_RA4836AA	17	191
ESC_RA4835AA	745	234
ESC_RA4834AA	297	234
ESC_RA4833AA	297	234
ESC_RA4832AA	152	257
ESC_RA4831AA	245	261
ESC_RA4830AA	328	924
ESC_RA4829AA	33	198
ESC_RA4828AA	21	156
ESC_RA4827AA	937	198
ESC_RA4826AA	829	257
ESC_RA4825AA	29	156
ESC_RA4824AA	25	234
ESC_RA4823AA	155	175
ESC_RA4822AA	101	696
ESC_RA4821AA	410	156
ESC_RA4820AA	410	156
ESC_RA4819AA	410	156
ESC_RA4818AA	9048	835
ESC_RA4816AA	1720	935
ESC_RA4815AA	11124	512
ESC_RA4814AA	11124	512
ESC_RA4813AA	410	156
ESC_RA4812AA	10	512
ESC_RA4811AA	5082	198
ESC_RA4810AA	48	76
ESC_RA4809AA	2521	257
ESC_RA4808AA	2179	924
ESC_RA4807AA	1431	257
ESC_RA4806AA	602	257
ESC_RA4805AA	58	198
ESC_RA4804AA	11126	160
ESC_RA4803AA	155	175
ESC_RA4802AA	58	198
ESC_RA4801AA	58	198
ESC_RA4800AA	648	359
ESC_RA4799AA	648	359
ESC_RA4798AA	58	198
ESC_RA4797AA	345	175
ESC_RA4796AA	155	175

ESC_RA4795AA	34	504
ESC_RA4794AA	11125	447
ESC_RA4793AA	847	257
ESC_RA4792AA	847	257
ESC_RA4791AA	106	387
ESC_RA4790AA	88	160
ESC_RA4789AA	410	156
ESC_RA4788AA	1431	257
ESC_RA4787AA	1431	257
ESC_RA4786AA	1431	257
ESC_RA4785AA	69	387
ESC_RA4784AA	69	387
ESC_RA4783AA	58	198
ESC_RA4782AA	224	198
ESC_RA4781AA	2521	257
ESC_RA4780AA	58	198
ESC_RA4779AA	109	257
ESC_RA4778AA	129	257
ESC_RA4777AA	2522	198
ESC_RA4776AA	2144	257
ESC_RA4775AA	10	512
ESC_RA4774AA	448	257
ESC_RA4773AA	10	512
ESC_RA4772AA	5082	198
ESC_RA4771AA	5082	198
ESC_RA4770AA	48	76
ESC_RA4769AA	442	257
ESC_RA4768AA	2179	924
ESC_RA4767AA	162	924
ESC_RA4766AA	11127	251
ESC_RA4765AA	542	824
ESC_RA4764AA	410	156
ESC_RA4763AA	2522	198
ESC_RA4762AA	10	512
ESC_RA4761AA	10	512
ESC_RA4760AA	212	198
ESC_RA4759AA	212	198
ESC_RA4758AA	361	512
ESC_RA4757AA	361	512
ESC_RA4756AA	56	198
ESC_RA4755AA	361	512
ESC_RA4754AA	410	156
ESC_RA4753AA	533	198
ESC_RA4752AA	533	198
ESC_RA4751AA	4553	408
ESC_RA4750AA	3231	519
ESC_RA4749AA	3231	519
ESC_RA4748AA	34	504
ESC_RA4747AA	11122	926
ESC_RA4746AA	11121	924

ESC_RA4745AA	11123	926
ESC_RA4744AA	11013	198
ESC_RA4743AA	589	650
ESC_RA4742AA	589	650
ESC_RA4741AA	589	650
ESC_RA4740AA	589	650
ESC_RA4739AA	107	527
ESC_RA4738AA	941	537
ESC_RA4696AA	6858	94
ESC_RA4695AA	589	650
ESC_RA4694AA	582	420
ESC_RA4693AA	589	650
ESC_RA4692AA	589	650
ESC_RA4691AA	582	420
ESC_RA4690AA	10	512
ESC_RA4689AA	731	285
ESC_RA4688AA	11	936
ESC_RA4687AA	11	936
ESC_RA4686AA	11	936
ESC_RA4685AA	11	936
ESC_RA4684AA	11	936
ESC_RA4683AA	11	936
ESC_RA4682AA	11	936
ESC_RA4681AA	11	936
ESC_RA4680AA	11	936
ESC_RA4679AA	11	936
ESC_RA4678AA	11	936
ESC_RA4677AA	11	936
ESC_RA4676AA	11	936
ESC_RA4675AA	11	936
ESC_RA4674AA	11	936
ESC_RA4673AA	11	936
ESC_RA4672AA	11013	198
ESC_RA4671AA	589	650
ESC_RA4670AA	11118	455
ESC_RA4669AA	11118	455
ESC_RA4668AA	11118	455
ESC_RA4667AA	583	768
ESC_RA4666AA	589	650
ESC_RA4665AA	152	257
ESC_RA4664AA	152	257
ESC_RA4663AA	145	256
ESC_RA4662AA	165	504
ESC_RA4661AA	2280	212
ESC_RA4660AA	58	198
ESC_RA4659AA	245	261
ESC_RA4656AA	131	653
ESC_RA4655AA	405	923
ESC_RA4654AA	131	653
ESC_RA4653AA	1193	761

ESC_RA4652AA	38	669
ESC_RA4651AA	44	512
ESC_RA4650AA	131	653
ESC_RA4649AA	131	653
ESC_RA4648AA	4419	463
ESC_RA4647AA	131	653
ESC_RA4646AA	131	653
ESC_RA4645AA	131	653
ESC_RA4644AA	11120	174
ESC_RA4643AA	131	653
ESC_RA4642AA	131	653
ESC_RA4641AA	540	286
ESC_RA4640AA	48	76
ESC_RA4639AA	906	198
ESC_RA4638AA	297	234
ESC_RA4637AA	155	175
ESC_RA4636AA	11115	935
ESC_RA4635AA	101	696
ESC_RA4634AA	58	198
ESC_RA4633AA	227	504
ESC_RA4632AA	1429	198
ESC_RA4631AA	23	156
ESC_RA4630AA	162	924
ESC_RA4629AA	48	76
ESC_RA4628AA	58	198
ESC_RA4627AA	58	198
ESC_RA4626AA	607	355
ESC_RA4625AA	398	445
ESC_RA4624AA	10	512
ESC_RA4623AA	227	504
ESC_RA4622AA	6519	592
ESC_RA4621AA	23	156
ESC_RA4620AA	117	592
ESC_RA4619AA	167	512
ESC_RA4618AA	12	626
ESC_RA4617AA	11114	626
ESC_RA4616AA	117	592
ESC_RA4615AA	681	393
ESC_RA4614AA	746	789
ESC_RA4613AA	3249	156
ESC_RA4612AA	1670	438
ESC_RA4611AA	23	156
ESC_RA4610AA	117	592
ESC_RA4609AA	38	669
ESC_RA4608AA	38	669
ESC_RA4607AA	117	592
ESC_RA4606AA	681	393
ESC_RA4605AA	141	644
ESC_RA4604AA	38	669
ESC_RA4603AA	131	653

ESC_RA4602AA	131	653
ESC_RA4601AA	131	653
ESC_RA4600AA	131	653
ESC_RA4599AA	131	653
ESC_RA4598AA	131	653
ESC_RA4597AA	636	653
ESC_RA4596AA	38	669
ESC_RA4595AA	450	71
ESC_RA4594AA	10	512
ESC_RA4593AA	10	512
ESC_RA4592AA	10	512
ESC_RA4591AA	10	512
ESC_RA4590AA	10	512
ESC_RA4589AA	10	512
ESC_RA4587AA	10	512
ESC_RA4586AA	10	512
ESC_RA4585AA	10	512
ESC_RA4584AA	10	512
ESC_RA4583AA	10	512
ESC_RA4582AA	10	512
ESC_RA4581AA	10	512
ESC_RA4580AA	10	512
ESC_RA4579AA	10	512
ESC_RA4578AA	10	512
ESC_RA4577AA	10	512
ESC_RA4576AA	10	512
ESC_RA4575AA	10	512
ESC_RA4574AA	10	512
ESC_RA4573AA	10	512
ESC_RA4572AA	10	512
ESC_RA4571AA	10	512
ESC_RA4570AA	10	512
ESC_RA4569AA	10	512
ESC_RA4568AA	10	512
ESC_RA4567AA	10	512
ESC_RA4566AA	10	512
ESC_RA4565AA	10	512
ESC_RA4564AA	10	512
ESC_RA4563AA	10	512
ESC_RA4562AA	10	512
ESC_RA4561AA	10	512
ESC_RA4560AA	10	512
ESC_RA4559AA	10	512
ESC_RA4558AA	10	512
ESC_RA4557AA	10	512
ESC_RA4556AA	10	512
ESC_RA4555AA	10	512
ESC_RA4554AA	10	512
ESC_RA4553AA	10	512
ESC_RA4550AA	10	512

ESC_RA4549AA	10	512
ESC_RA4548AA	10	512
ESC_RA4547AA	10	512
ESC_RA4546AA	10	512
ESC_RA4545AA	10	512
ESC_RA4544AA	10	512
ESC_RA4543AA	10	512
ESC_RA4542AA	10	512
ESC_RA4541AA	10	512
ESC_RA4540AA	10	512
ESC_RA4539AA	10	512
ESC_RA4538AA	131	653
ESC_RA4537AA	131	653
ESC_RA4536AA	70	670
ESC_RA4535AA	131	653
ESC_RA4534AA	131	653
ESC_RA4533AA	38	669
ESC_RA4532AA	131	653
ESC_RA4531AA	2279	657
ESC_RA4530AA	131	653
ESC_RA4529AA	131	653
ESC_RA4528AA	131	653
ESC_RA4527AA	8671	653
ESC_RA4526AA	62	574
ESC_RA4525AA	131	653
ESC_RA4524AA	1725	175
ESC_RA4523AA	973	968
ESC_RA4522AA	1723	900
ESC_RA4521AA	372	424
ESC_RA4520AA	372	424
ESC_RA4519AA	127	738
ESC_RA4518AA	978	740
ESC_RA4517AA	10	512
ESC_RA4516AA	2008	257
ESC_RA4515AA	2008	257
ESC_RA4514AA	38	669
ESC_RA4513AA	11119	257
ESC_RA4512AA	73	737
ESC_RA4511AA	2008	257
ESC_RA4510AA	86	935
ESC_RA4509AA	2474	542
ESC_RA4508AA	141	644
ESC_RA4507AA	156	175
ESC_RA4506AA	9528	820
ESC_RA4505AA	86	935
ESC_RA4504AA	28	537
ESC_RA4503AA	372	424
ESC_RA4502AA	127	738
ESC_RA4501AA	100	504
ESC_RA4500AA	100	504

ESC_RA4499AA	10	512
ESC_RA4498AA	90	156
ESC_RA4497AA	90	156
ESC_RA4496AA	9963	318
ESC_RA4495AA	101	696
ESC_RA4494AA	156	175
ESC_RA4492AA	156	175
ESC_RA4491AA	224	198
ESC_RA4490AA	101	696
ESC_RA4489AA	602	257
ESC_RA4487AA	162	924
ESC_RA4486AA	11117	257
ESC_RA4483AA	156	175
ESC_RA4481AA	155	175
ESC_RA4480AA	11116	198
ESC_RA4477AA	57	50
ESC_RA4476AA	1101	842
ESC_RA4474AA	11	936
ESC_RA4473AA	11	936
ESC_RA4472AA	11	936
ESC_RA4471AA	11	936
ESC_RA4470AA	11	936
ESC_RA4469AA	11	936
ESC_RA4468AA	11	936
ESC_RA4467AA	11	936
ESC_RA4466AA	11	936
ESC_RA4465AA	11	936
ESC_RA4464AA	11	936
ESC_RA4463AA	11	936
ESC_RA4462AA	11	936
ESC_RA4461AA	11	936
ESC_RA4460AA	11	936
ESC_RA4459AA	11	936
ESC_RA4458AA	11	936
ESC_RA4457AA	11	936
ESC_RA4456AA	11	936
ESC_RA4455AA	11	936
ESC_RA4454AA	11	936
ESC_RA4453AA	121	537
ESC_RA4452AA	11	936
ESC_RA4451AA	11	936
ESC_RA4450AA	90	156
ESC_RA4449AA	1431	257
ESC_RA4448AA	131	653
ESC_RA4447AA	131	653
ESC_RA4446AA	38	669
ESC_RA4445AA	212	198
ESC_RA4444AA	131	653
ESC_RA4443AA	744	512
ESC_RA4442AA	602	257

ESC_RA4441AA	10	512
ESC_RA4440AA	10	512
ESC_RA4439AA	10	512
ESC_RA4438AA	10	512
ESC_RA4437AA	10	512
ESC_RA4436AA	10	512
ESC_RA4435AA	2141	597
ESC_RA4434AA	131	653
ESC_RA4433AA	131	653
ESC_RA4432AA	131	653
ESC_RA4431AA	405	923
ESC_RA4430AA	450	71
ESC_RA4417AA	10	512
ESC_RA4412AA	58	198
ESC_RA4411AA	11	936
ESC_RA4410AA	156	175
ESC_RA4409AA	155	175
ESC_RA4408AA	16	156
ESC_RA4407AA	1884	234
ESC_RA4406AA	21	156
ESC_RA4405AA	1252	567
ESC_RA4404AA	1421	447
ESC_RA4403AA	58	198
ESC_RA4402AA	69	387
ESC_RA4401AA	4198	878
ESC_RA4400AA	58	198
ESC_RA4399AA	29	156
ESC_RA4398AA	156	175
ESC_RA4397AA	1262	737
ESC_RA4396AA	127	738
ESC_RA4395AA	95	738
ESC_RA4394AA	69	387
ESC_RA4393AA	3695	924
ESC_RA4392AA	420	741
ESC_RA4391AA	245	261
ESC_RA4390AA	245	261
ESC_RA4389AA	245	261
ESC_RA4388AA	11	936
ESC_RA4387AA	11111	605
ESC_RA4386AA	1193	761
ESC_RA4385AA	12	626
ESC_RA4384AA	93	71
ESC_RA4383AA	9699	198
ESC_RA4382AA	9699	198
ESC_RA4381AA	11105	257
ESC_RA4380AA	11104	257
ESC_RA4379AA	9699	198
ESC_RA4378AA	11106	175
ESC_RA4376AA	34	504
ESC_RA4375AA	100	504

ESC_RA4374AA	16	156
ESC_RA4372AA	11090	76
ESC_RA4371AA	11088	76
ESC_RA4370AA	11087	261
ESC_RA4369AA	29	156
ESC_RA4368AA	152	257
ESC_RA4367AA	152	257
ESC_RA4366AA	131	653
ESC_RA4365AA	68	386
ESC_RA4364AA	131	653
ESC_RA4363AA	131	653
ESC_RA4362AA	131	653
ESC_RA4361AA	131	653
ESC_RA4360AA	131	653
ESC_RA4359AA	998	644
ESC_RA4358AA	131	653
ESC_RA4357AA	131	653
ESC_RA4356AA	405	923
ESC_RA4355AA	131	653
ESC_RA4354AA	131	653
ESC_RA4351AA	201	257
ESC_RA4350AA	69	387
ESC_RA4349AA	6496	198
ESC_RA4348AA	752	512
ESC_RA4347AA	69	387
ESC_RA4346AA	1434	512
ESC_RA4345AA	5374	156
ESC_RA4344AA	10	512
ESC_RA4343AA	607	355
ESC_RA4342AA	69	387
ESC_RA4341AA	3014	410
ESC_RA4340AA	58	198
ESC_RA4339AA	410	156
ESC_RA4338AA	29	156
ESC_RA4337AA	5869	257
ESC_RA4336AA	847	257
ESC_RA4335AA	10	512
ESC_RA4334AA	131	653
ESC_RA4333AA	131	653
ESC_RA4332AA	131	653
ESC_RA4331AA	648	359
ESC_RA4330AA	69	387
ESC_RA4329AA	131	653
ESC_RA4328AA	405	923
ESC_RA4327AA	38	669
ESC_RA4326AA	90	156
ESC_RA4325AA	90	156
ESC_RA4324AA	90	156
ESC_RA4323AA	90	156
ESC_RA4322AA	90	156

ESC_RA4321AA	120	180
ESC_RA4320AA	90	156
ESC_RA4319AA	90	156
ESC_RA4318AA	90	156
ESC_RA4317AA	90	156
ESC_RA4316AA	90	156
ESC_RA4315AA	120	180
ESC_RA4314AA	90	156
ESC_RA4313AA	90	156
ESC_RA4312AA	90	156
ESC_RA4311AA	38	669
ESC_RA4310AA	46	447
ESC_RA4309AA	131	653
ESC_RA4308AA	131	653
ESC_RA4307AA	131	653
ESC_RA4306AA	131	653
ESC_RA4305AA	131	653
ESC_RA4304AA	131	653
ESC_RA4303AA	131	653
ESC_RA4302AA	2279	657
ESC_RA4301AA	131	653
ESC_RA4300AA	131	653
ESC_RA4299AA	131	653
ESC_RA4298AA	8671	653
ESC_RA4297AA	131	653
ESC_RA4296AA	131	653
ESC_RA4295AA	11	936
ESC_RA4294AA	11	936
ESC_RA4293AA	11	936
ESC_RA4292AA	11	936
ESC_RA4291AA	11	936
ESC_RA4290AA	11	936
ESC_RA4289AA	11	936
ESC_RA4288AA	11	936
ESC_RA4287AA	11	936
ESC_RA4286AA	11	936
ESC_RA4285AA	21	156
ESC_RA4284AA	11	936
ESC_RA4283AA	11	936
ESC_RA4282AA	11	936
ESC_RA4281AA	11	936
ESC_RA4280AA	11	936
ESC_RA4279AA	11	936
ESC_RA4278AA	11	936
ESC_RA4277AA	11	936
ESC_RA4276AA	90	156
ESC_RA4275AA	10	512
ESC_RA4274AA	90	156
ESC_RA4273AA	11	936
ESC_RA4272AA	405	923

ESC_RA4271AA	131	653
ESC_RA4270AA	131	653
ESC_RA4269AA	1193	761
ESC_RA4268AA	131	653
ESC_RA4267AA	131	653
ESC_RA4266AA	8671	653
ESC_RA4265AA	8671	653
ESC_RA4264AA	131	653
ESC_RA4263AA	1060	512
ESC_RA4262AA	1060	512
ESC_RA4261AA	1060	512
ESC_RA4260AA	152	257
ESC_RA4259AA	245	261
ESC_RA4258AA	245	261
ESC_RA4257AA	148	191
ESC_RA4256AA	99	198
ESC_RA4254AA	448	257
ESC_RA4253AA	641	935
ESC_RA4252AA	517	924
ESC_RA4251AA	11001	924
ESC_RA4249AA	1312	76
ESC_RA4248AA	6272	862
ESC_RA4246AA	752	512
ESC_RA4245AA	752	512
ESC_RA4244AA	34	504
ESC_RA4242AA	189	504
ESC_RA4241AA	517	924
ESC_RA4240AA	517	924
ESC_RA4239AA	517	924
ESC_RA4238AA	442	257
ESC_RA4236AA	20	198
ESC_RA4235AA	1380	914
ESC_RA4234AA	3570	156
ESC_RA4233AA	4	76
ESC_RA4232AA	800	926
ESC_RA4231AA	4702	626
ESC_RA4230AA	34	504
ESC_RA4229AA	34	504
ESC_RA4228AA	1490	935
ESC_RA4227AA	337	257
ESC_RA4226AA	189	504
ESC_RA4225AA	1380	914
ESC_RA4224AA	443	257
ESC_RA4223AA	517	924
ESC_RA4222AA	1312	76
ESC_RA4221AA	10	512
ESC_RA4220AA	2086	435
ESC_RA4219AA	517	924
ESC_RA4218AA	1380	914
ESC_RA4217AA	517	924

ESC_RA4216AA	6272	862
ESC_RA4215AA	752	512
ESC_RA4214AA	4119	537
ESC_RA4213AA	28	537
ESC_RA4212AA	131	653
ESC_RA4211AA	752	512
ESC_RA4209AA	34	504
ESC_RA4208AA	189	504
ESC_RA4207AA	517	924
ESC_RA4206AA	799	852
ESC_RA4205AA	189	504
ESC_RA4204AA	40	198
ESC_RA4203AA	752	512
ESC_RA4202AA	10	512
ESC_RA4201AA	31	387
ESC_RA4200AA	189	504
ESC_RA4199AA	2178	924
ESC_RA4198AA	443	257
ESC_RA4197AA	382	481
ESC_RA4196AA	1312	76
ESC_RA4195AA	752	512
ESC_RA4194AA	70	670
ESC_RA4193AA	752	512
ESC_RA4192AA	1491	76
ESC_RA4191AA	752	512
ESC_RA4190AA	940	257
ESC_RA4189AA	1295	198
ESC_RA4188AA	2178	924
ESC_RA4187AA	517	924
ESC_RA4186AA	48	76
ESC_RA4185AA	1491	76
ESC_RA4184AA	40	198
ESC_RA4183AA	1201	512
ESC_RA4182AA	414	387
ESC_RA4181AA	88	160
ESC_RA4180AA	4633	118
ESC_RA4179AA	4119	537
ESC_RA4178AA	4	76
ESC_RA4177AA	4	76
ESC_RA4176AA	1491	76
ESC_RA4175AA	381	156
ESC_RA4174AA	189	504
ESC_RA4173AA	10	512
ESC_RA4172AA	752	512
ESC_RA4171AA	1491	76
ESC_RA4170AA	616	156
ESC_RA4169AA	414	387
ESC_RA4168AA	38	669
ESC_RA4167AA	4060	744
ESC_RA4166AA	1491	76

ESC_RA4165AA	443	257
ESC_RA4164AA	616	156
ESC_RA4163AA	28	537
ESC_RA4162AA	2040	198
ESC_RA4161AA	616	156
ESC_RA4160AA	1491	76
ESC_RA4159AA	642	924
ESC_RA4158AA	1491	76
ESC_RA4157AA	4213	251
ESC_RA4156AA	450	71
ESC_RA4155AA	616	156
ESC_RA4154AA	6272	862
ESC_RA4153AA	1125	198
ESC_RA4152AA	752	512
ESC_RA4151AA	337	257
ESC_RA4150AA	328	924
ESC_RA4149AA	517	924
ESC_RA4148AA	3933	318
ESC_RA4147AA	1312	76
ESC_RA4146AA	218	519
ESC_RA4145AA	1849	601
ESC_RA4144AA	10	512
ESC_RA4143AA	517	924
ESC_RA4142AA	517	924
ESC_RA4141AA	517	924
ESC_RA4140AA	517	924
ESC_RA4139AA	517	924
ESC_RA4138AA	34	504
ESC_RA4137AA	517	924
ESC_RA4136AA	4	76
ESC_RA4135AA	31	387
ESC_RA4134AA	382	481
ESC_RA4133AA	2178	924
ESC_RA4132AA	1491	76
ESC_RA4131AA	155	175
ESC_RA4130AA	131	653
ESC_RA4129AA	20	198
ESC_RA4128AA	32	270
ESC_RA4127AA	4	76
ESC_RA4126AA	1491	76
ESC_RA4125AA	6272	862
ESC_RA4124AA	6272	862
ESC_RA4123AA	31	387
ESC_RA4122AA	1380	914
ESC_RA4121AA	34	504
ESC_RA4120AA	10	512
ESC_RA4119AA	1136	108
ESC_RA4118AA	10	512
ESC_RA4116AA	2178	924
ESC_RA4115AA	1312	76

ESC_RA4114AA	10	512
ESC_RA4113AA	2332	601
ESC_RA4112AA	295	160
ESC_RA4111AA	11004	537
ESC_RA4110AA	517	924
ESC_RA4109AA	6272	862
ESC_RA4108AA	10	512
ESC_RA4107AA	803	739
ESC_RA4106AA	31	387
ESC_RA4105AA	11003	924
ESC_RA4104AA	641	935
ESC_RA4103AA	752	512
ESC_RA4102AA	10	512
ESC_RA4101AA	34	504
ESC_RA4100AA	34	504
ESC_RA4099AA	34	504
ESC_RA4098AA	3570	156
ESC_RA4097AA	752	512
ESC_RA4096AA	34	504
ESC_RA4095AA	40	198
ESC_RA4094AA	6272	862
ESC_RA4093AA	218	519
ESC_RA4092AA	686	670
ESC_RA4091AA	48	76
ESC_RA4090AA	2040	198
ESC_RA4089AA	11005	77
ESC_RA4088AA	2178	924
ESC_RA4087AA	226	512
ESC_RA4086AA	2178	924
ESC_RA4085AA	752	512
ESC_RA4084AA	327	204
ESC_RA4083AA	1312	76
ESC_RA4082AA	752	512
ESC_RA4081AA	328	924
ESC_RA4080AA	752	512
ESC_RA4079AA	335	936
ESC_RA4077AA	752	512
ESC_RA4076AA	517	924
ESC_RA4075AA	2346	435
ESC_RA4074AA	2356	379
ESC_RA4073AA	69	387
ESC_RA4072AA	4	76
ESC_RA4071AA	803	739
ESC_RA4070AA	1193	761
ESC_RA4069AA	6272	862
ESC_RA4068AA	590	156
ESC_RA4067AA	173	254
ESC_RA4066AA	382	481
ESC_RA4065AA	6955	76
ESC_RA4064AA	156	175

ESC_RA4063AA	1125	198
ESC_RA4062AA	5276	77
ESC_RA4061AA	443	257
ESC_RA4060AA	6955	76
ESC_RA4059AA	443	257
ESC_RA4058AA	1312	76
ESC_RA4057AA	7937	198
ESC_RA4055AA	443	257
ESC_RA4054AA	7937	198
ESC_RA4053AA	40	198
ESC_RA4052AA	6272	862
ESC_RA4051AA	7937	198
ESC_RA4050AA	517	924
ESC_RA4049AA	10	512
ESC_RA4048AA	642	924
ESC_RA4047AA	3303	537
ESC_RA4046AA	616	156
ESC_RA4045AA	793	286
ESC_RA4044AA	6303	387
ESC_RA4043AA	4	76
ESC_RA4042AA	2088	768
ESC_RA4041AA	4	76
ESC_RA4040AA	6303	387
ESC_RA4039AA	443	257
ESC_RA4038AA	795	924
ESC_RA4037AA	224	198
ESC_RA4036AA	793	286
ESC_RA4035AA	1491	76
ESC_RA4034AA	93	71
ESC_RA4033AA	752	512
ESC_RA4032AA	2040	198
ESC_RA4031AA	603	198
ESC_RA4030AA	10	512
ESC_RA4029AA	8164	795
ESC_RA4028AA	69	387
ESC_RA4027AA	29	156
ESC_RA4026AA	226	512
ESC_RA4025AA	361	512
ESC_RA4024AA	8168	1004
ESC_RA4023AA	361	512
ESC_RA4022AA	10	512
ESC_RA4021AA	34	504
ESC_RA4020AA	69	387
ESC_RA4019AA	29	156
ESC_RA4018AA	48	76
ESC_RA4017AA	345	175
ESC_RA4016AA	101	696
ESC_RA4015AA	48	76
ESC_RA4014AA	10	512
ESC_RA4013AA	48	76

ESC_RA4012AA	361	512
ESC_RA4011AA	48	76
ESC_RA4010AA	3889	291
ESC_RA4009AA	3889	291
ESC_RA4008AA	7939	1004
ESC_RA4007AA	10	512
ESC_RA4006AA	361	512
ESC_RA4005AA	77	286
ESC_RA4004AA	34	504
ESC_RA4003AA	398	445
ESC_RA4002AA	69	387
ESC_RA4001AA	877	935
ESC_RA4000AA	345	175
ESC_RA3999AA	117	592
ESC_RA3998AA	77	286
ESC_RA3997AA	10	512
ESC_RA3996AA	101	696
ESC_RA3995AA	58	198
ESC_RA3994AA	8170	76
ESC_RA3993AA	10	512
ESC_RA3992AA	29	156
ESC_RA3991AA	20	198
ESC_RA3990AA	7939	1004
ESC_RA3989AA	398	445
ESC_RA3988AA	4429	820
ESC_RA3987AA	10	512
ESC_RA3986AA	20	198
ESC_RA3983AA	542	824
ESC_RA3982AA	10	512
ESC_RA3981AA	20	198
ESC_RA3980AA	48	76
ESC_RA3979AA	2614	975
ESC_RA3978AA	398	445
ESC_RA3977AA	202	311
ESC_RA3976AA	165	504
ESC_RA3975AA	101	696
ESC_RA3974AA	70	670
ESC_RA3973AA	5841	55
ESC_RA3972AA	3889	291
ESC_RA3971AA	165	504
ESC_RA3970AA	40	198
ESC_RA3969AA	11081	1004
ESC_RA3968AA	165	504
ESC_RA3967AA	154	257
ESC_RA3966AA	877	935
ESC_RA3965AA	165	504
ESC_RA3964AA	11080	519
ESC_RA3963AA	58	198
ESC_RA3962AA	746	789
ESC_RA3961AA	3889	291

ESC_RA3960AA	609	447
ESC_RA3959AA	48	76
ESC_RA3958AA	34	504
ESC_RA3957AA	70	670
ESC_RA3956AA	2936	515
ESC_RA3955AA	4628	76
ESC_RA3954AA	10	512
ESC_RA3953AA	10	512
ESC_RA3952AA	216	512
ESC_RA3951AA	8868	936
ESC_RA3950AA	361	512
ESC_RA3949AA	20	198
ESC_RA3948AA	761	512
ESC_RA3947AA	744	512
ESC_RA3946AA	761	512
ESC_RA3945AA	398	445
ESC_RA3944AA	11081	1004
ESC_RA3943AA	29	156
ESC_RA3942AA	20	198
ESC_RA3941AA	69	387
ESC_RA3940AA	20	198
ESC_RA3939AA	641	935
ESC_RA3938AA	48	76
ESC_RA3937AA	10	512
ESC_RA3936AA	20	198
ESC_RA3935AA	746	789
ESC_RA3934AA	20	198
ESC_RA3933AA	398	445
ESC_RA3932AA	8169	504
ESC_RA3931AA	8165	111
ESC_RA3930AA	877	935
ESC_RA3929AA	609	447
ESC_RA3928AA	361	512
ESC_RA3927AA	34	504
ESC_RA3926AA	8167	71
ESC_RA3925AA	372	424
ESC_RA3924AA	345	175
ESC_RA3923AA	34	504
ESC_RA3922AA	345	175
ESC_RA3921AA	877	935
ESC_RA3920AA	29	156
ESC_RA3919AA	8166	789
ESC_RA3918AA	6615	307
ESC_RA3917AA	641	935
ESC_RA3916AA	226	512
ESC_RA3915AA	8166	789
ESC_RA3914AA	744	512
ESC_RA3913AA	354	15
ESC_RA3912AA	770	587
ESC_RA3911AA	131	653

ESC_RA3910AA	131	653
ESC_RA3909AA	131	653
ESC_RA3908AA	131	653
ESC_RA3907AA	131	653
ESC_RA3906AA	131	653
ESC_RA3905AA	648	359
ESC_RA3904AA	69	387
ESC_RA3903AA	405	923
ESC_RA3902AA	131	653
ESC_RA3901AA	361	512
ESC_RA3900AA	131	653
ESC_RA3899AA	131	653
ESC_RA3898AA	131	653
ESC_RA3897AA	131	653
ESC_RA3896AA	131	653
ESC_RA3884AA	10	512
ESC_RA3883AA	93	71
ESC_RA3882AA	10	512
ESC_RA3881AA	11	936
ESC_RA3880AA	38	669
ESC_RA3879AA	2279	657
ESC_RA3878AA	131	653
ESC_RA3877AA	131	653
ESC_RA3876AA	131	653
ESC_RA3875AA	648	359
ESC_RA3874AA	69	387
ESC_RA3873AA	131	653
ESC_RA3872AA	131	653
ESC_RA3871AA	131	653
ESC_RA3870AA	131	653
ESC_RA3869AA	44	512
ESC_RA3868AA	410	156
ESC_RA3867AA	131	653
ESC_RA3866AA	405	923
ESC_RA3865AA	38	669
ESC_RA3864AA	131	653
ESC_RA3863AA	58	198
ESC_RA3862AA	131	653
ESC_RA3861AA	131	653
ESC_RA3860AA	8671	653
ESC_RA3859AA	131	653
ESC_RA3858AA	131	653
ESC_RA3857AA	648	359
ESC_RA3856AA	131	653
ESC_RA3855AA	131	653
ESC_RA3854AA	2178	924
ESC_RA3853AA	131	653
ESC_RA3852AA	69	387
ESC_RA3851AA	131	653
ESC_RA3850AA	131	653

ESC_RA3849AA	131	653
ESC_RA3848AA	131	653
ESC_RA3847AA	131	653
ESC_RA3846AA	131	653
ESC_RA3845AA	131	653
ESC_RA3844AA	12	626
ESC_RA3843AA	131	653
ESC_RA3839AA	354	15
ESC_RA3838AA	354	15
ESC_RA3837AA	69	387
ESC_RA3836AA	410	156
ESC_RA3835AA	131	653
ESC_RA3834AA	131	653
ESC_RA3833AA	410	156
ESC_RA3832AA	131	653
ESC_RA3831AA	131	653
ESC_RA3830AA	131	653
ESC_RA3829AA	131	653
ESC_RA3828AA	410	156
ESC_RA3827AA	131	653
ESC_RA3826AA	1193	761
ESC_RA3824AA	131	653
ESC_RA3823AA	410	156
ESC_RA3822AA	131	653
ESC_RA3821AA	10	512
ESC_RA3820AA	131	653
ESC_RA3819AA	131	653
ESC_RA3818AA	648	359
ESC_RA3817AA	11060	198
ESC_RA3816AA	2847	212
ESC_RA3815AA	95	738
ESC_RA3813AA	1114	504
ESC_RA3812AA	10	512
ESC_RA3811AA	10	512
ESC_RA3809AA	617	511
ESC_RA3808AA	448	257
ESC_RA3807AA	131	653
ESC_RA3806AA	131	653
ESC_RA3805AA	131	653
ESC_RA3804AA	131	653
ESC_RA3803AA	10	512
ESC_RA3802AA	131	653
ESC_RA3801AA	10	512
ESC_RA3800AA	410	156
ESC_RA3799AA	393	343
ESC_RA3798AA	635	141
ESC_RA3797AA	7095	142
ESC_RA3796AA	141	644
ESC_RA3795AA	38	669
ESC_RA3794AA	10	512

ESC_RA3793AA	583	768
ESC_RA3792AA	542	824
ESC_RA3791AA	515	1004
ESC_RA3790AA	1196	257
ESC_RA3789AA	167	512
ESC_RA3788AA	410	156
ESC_RA3787AA	410	156
ESC_RA3786AA	11066	512
ESC_RA3785AA	11066	512
ESC_RA3784AA	10	512
ESC_RA3783AA	131	653
ESC_RA3782AA	410	156
ESC_RA3781AA	8655	359
ESC_RA3780AA	11	936
ESC_RA3779AA	648	359
ESC_RA3778AA	10754	785
ESC_RA3777AA	11067	592
ESC_RA3776AA	11	936
ESC_RA3775AA	69	387
ESC_RA3774AA	95	738
ESC_RA3773AA	131	653
ESC_RA3772AA	963	669
ESC_RA3771AA	95	738
ESC_RA3770AA	11068	737
ESC_RA3769AA	131	653
ESC_RA3768AA	131	653
ESC_RA3767AA	131	653
ESC_RA3766AA	144	740
ESC_RA3765AA	131	653
ESC_RA3764AA	131	653
ESC_RA3763AA	405	923
ESC_RA3762AA	131	653
ESC_RA3761AA	131	653
ESC_RA3760AA	3057	672
ESC_RA3759AA	131	653
ESC_RA3758AA	2599	257
ESC_RA3757AA	95	738
ESC_RA3756AA	95	738
ESC_RA3755AA	10522	626
ESC_RA3754AA	162	924
ESC_RA3753AA	11069	391
ESC_RA3752AA	11072	445
ESC_RA3751AA	11073	319
ESC_RA3750AA	11071	870
ESC_RA3749AA	11074	175
ESC_RA3748AA	11070	512
ESC_RA3747AA	44	512
ESC_RA3746AA	11075	257
ESC_RA3745AA	10187	464
ESC_RA3744AA	4419	463

ESC_RA3743AA	11077	198
ESC_RA3742AA	442	257
ESC_RA3741AA	11	936
ESC_RA3740AA	11078	936
ESC_RA3739AA	11	936
ESC_RA3738AA	11	936
ESC_RA3737AA	167	512
ESC_RA3736AA	10	512
ESC_RA3735AA	10	512
ESC_RA3734AA	10	512
ESC_RA3733AA	223	198
ESC_RA3732AA	40	198
ESC_RA3731AA	48	76
ESC_RA3730AA	11	936
ESC_RA3729AA	167	512
ESC_RA3728AA	167	512
ESC_RA3727AA	95	738
ESC_RA3726AA	38	669
ESC_RA3725AA	191	198
ESC_RA3724AA	410	156
ESC_RA3723AA	223	198
ESC_RA3722AA	10	512
ESC_RA3721AA	761	512
ESC_RA3720AA	761	512
ESC_RA3719AA	38	669
ESC_RA3718AA	10	512
ESC_RA3717AA	10	512
ESC_RA3716AA	10	512
ESC_RA3715AA	10	512
ESC_RA3714AA	354	15
ESC_RA3713AA	32	270
ESC_RA3711AA	10	512
ESC_RA3710AA	10	512
ESC_RA3709AA	131	653
ESC_RA3708AA	11	936
ESC_RA3707AA	1060	512
ESC_RA3706AA	1079	235
ESC_RA3705AA	11084	235
ESC_RA3704AA	641	935
ESC_RA3703AA	761	512
ESC_RA3702AA	8165	111
ESC_RA3701AA	7131	512
ESC_RA3700AA	5415	924
ESC_RA3699AA	10	512
ESC_RA3698AA	446	257
ESC_RA3697AA	206	286
ESC_RA3696AA	410	156
ESC_RA3695AA	195	76
ESC_RA3694AA	48	76
ESC_RA3693AA	10	512

ESC_RA3692AA	7369	512
ESC_RA3691AA	10	512
ESC_RA3690AA	10	512
ESC_RA3689AA	10	512
ESC_RA3688AA	10	512
ESC_RA3687AA	10	512
ESC_RA3686AA	10	512
ESC_RA3685AA	10	512
ESC_RA3683AA	11085	850
ESC_RA3682AA	10	512
ESC_RA3681AA	167	512
ESC_RA3680AA	10	512
ESC_RA3679AA	58	198
ESC_RA3678AA	11086	929
ESC_RA3677AA	10	512
ESC_RA3676AA	16	156
ESC_RA3674AA	10	512
ESC_RA3673AA	2325	447
ESC_RA3672AA	3714	175
ESC_RA3671AA	7511	198
ESC_RA3670AA	95	738
ESC_RA3669AA	95	738
ESC_RA3668AA	3714	175
ESC_RA3667AA	2705	504
ESC_RA3666AA	3042	663
ESC_RA3665AA	4038	257
ESC_RA3664AA	446	257
ESC_RA3663AA	993	789
ESC_RA3662AA	3630	71
ESC_RA3661AA	1611	198
ESC_RA3660AA	1611	198
ESC_RA3659AA	1611	198
ESC_RA3658AA	155	175
ESC_RA3657AA	162	924
ESC_RA3656AA	156	175
ESC_RA3655AA	278	924
ESC_RA3654AA	10	512
ESC_RA3653AA	10	512
ESC_RA3652AA	10	512
ESC_RA3651AA	10	512
ESC_RA3650AA	315	669
ESC_RA3649AA	1721	311
ESC_RA3648AA	10	512
ESC_RA3647AA	429	654
ESC_RA3646AA	405	923
ESC_RA3645AA	405	923
ESC_RA3644AA	405	923
ESC_RA3643AA	10170	512
ESC_RA3642AA	10170	512
ESC_RA3641AA	10	512

ESC_RA3640AA	708	924
ESC_RA3639AA	156	175
ESC_RA3638AA	224	198
ESC_RA3637AA	58	198
ESC_RA3636AA	10	512
ESC_RA3635AA	201	257
ESC_RA3634AA	206	286
ESC_RA3633AA	206	286
ESC_RA3632AA	189	504
ESC_RA3631AA	165	504
ESC_RA3630AA	10641	286
ESC_RA3629AA	10	512
ESC_RA3628AA	7366	824
ESC_RA3627AA	48	76
ESC_RA3626AA	381	156
ESC_RA3625AA	761	512
ESC_RA3624AA	10	512
ESC_RA3623AA	167	512
ESC_RA3622AA	29	156
ESC_RA3621AA	1193	761
ESC_RA3620AA	761	512
ESC_RA3619AA	533	198
ESC_RA3618AA	93	71
ESC_RA3617AA	4417	234
ESC_RA3616AA	2217	62
ESC_RA3615AA	2217	62
ESC_RA3614AA	2217	62
ESC_RA3613AA	17	191
ESC_RA3612AA	973	968
ESC_RA3611AA	394	400
ESC_RA3610AA	95	738
ESC_RA3609AA	963	669
ESC_RA3608AA	491	626
ESC_RA3607AA	73	737
ESC_RA3606AA	95	738
ESC_RA3605AA	10	512
ESC_RA3604AA	95	738
ESC_RA3603AA	95	738
ESC_RA3602AA	152	257
ESC_RA3601AA	357	654
ESC_RA3600AA	245	261
ESC_RA3599AA	144	740
ESC_RA3598AA	152	257
ESC_RA3597AA	54	256
ESC_RA3596AA	152	257
ESC_RA3595AA	967	212
ESC_RA3594AA	152	257
ESC_RA3593AA	69	387
ESC_RA3592AA	243	197
ESC_RA3591AA	11089	761

ESC_RA3590AA	106	387
ESC_RA3589AA	14	761
ESC_RA3588AA	245	261
ESC_RA3587AA	998	644
ESC_RA3586AA	38	669
ESC_RA3585AA	998	644
ESC_RA3584AA	245	261
ESC_RA3583AA	270	204
ESC_RA3582AA	245	261
ESC_RA3580AA	6	445
ESC_RA3579AA	245	261
ESC_RA3578AA	245	261
ESC_RA3577AA	245	261
ESC_RA3576AA	152	257
ESC_RA3575AA	11	936
ESC_RA3574AA	11	936
ESC_RA3573AA	11	936
ESC_RA3572AA	11	936
ESC_RA3571AA	11	936
ESC_RA3567AA	152	257
ESC_RA3566AA	152	257
ESC_RA3565AA	152	257
ESC_RA3564AA	245	261
ESC_RA3563AA	152	257
ESC_RA3562AA	152	257
ESC_RA3561AA	245	261
ESC_RA3560AA	245	261
ESC_RA3559AA	245	261
ESC_RA3558AA	245	261
ESC_RA3557AA	152	257
ESC_RA3556AA	2064	261
ESC_RA3555AA	243	197
ESC_RA3554AA	245	261
ESC_RA3553AA	245	261
ESC_RA3552AA	245	261
ESC_RA3551AA	245	261
ESC_RA3550AA	245	261
ESC_RA3547AA	10	512
ESC_RA3546AA	1079	235
ESC_RA3545AA	10	512
ESC_RA3544AA	4164	880
ESC_RA3543AA	826	926
ESC_RA3542AA	1250	926
ESC_RA3541AA	11033	926
ESC_RA3540AA	826	926
ESC_RA3539AA	11034	926
ESC_RA3538AA	826	926
ESC_RA3537AA	1709	936
ESC_RA3536AA	10	512
ESC_RA3535AA	4164	880

ESC_RA3534AA	218	519
ESC_RA3533AA	1250	926
ESC_RA3532AA	1250	926
ESC_RA3531AA	1250	926
ESC_RA3530AA	11033	926
ESC_RA3529AA	1250	926
ESC_RA3528AA	1730	234
ESC_RA3527AA	641	935
ESC_RA3526AA	641	935
ESC_RA3525AA	1250	926
ESC_RA3524AA	11032	198
ESC_RA3523AA	141	644
ESC_RA3522AA	10	512
ESC_RA3521AA	6445	878
ESC_RA3520AA	3593	153
ESC_RA3519AA	716	795
ESC_RA3518AA	1176	343
ESC_RA3517AA	738	598
ESC_RA3516AA	11	936
ESC_RA3515AA	21	156
ESC_RA3514AA	11	936
ESC_RA3513AA	59	217
ESC_RA3512AA	306	286
ESC_RA3511AA	127	738
ESC_RA3506AA	744	512
ESC_RA3505AA	7250	919
ESC_RA3504AA	362	234
ESC_RA3503AA	1246	198
ESC_RA3502AA	160	257
ESC_RA3501AA	69	387
ESC_RA3500AA	1880	653
ESC_RA3499AA	2521	257
ESC_RA3498AA	10	512
ESC_RA3497AA	117	592
ESC_RA3496AA	2164	257
ESC_RA3495AA	131	653
ESC_RA3494AA	772	76
ESC_RA3493AA	345	175
ESC_RA3492AA	11031	257
ESC_RA3491AA	10752	370
ESC_RA3489AA	90	156
ESC_RA3488AA	955	670
ESC_RA3487AA	10	512
ESC_RA3486AA	90	156
ESC_RA3485AA	90	156
ESC_RA3484AA	90	156
ESC_RA3483AA	100	504
ESC_RA3482AA	11	936
ESC_RA3481AA	156	175
ESC_RA3480AA	88	160

ESC_RA3479AA	165	504
ESC_RA3478AA	48	76
ESC_RA3477AA	442	257
ESC_RA3476AA	442	257
ESC_RA3475AA	442	257
ESC_RA3474AA	156	175
ESC_RA3473AA	156	175
ESC_RA3472AA	4	76
ESC_RA3471AA	1193	761
ESC_RA3470AA	69	387
ESC_RA3469AA	131	653
ESC_RA3468AA	88	160
ESC_RA3467AA	62	574
ESC_RA3466AA	131	653
ESC_RA3465AA	10	512
ESC_RA3464AA	131	653
ESC_RA3463AA	1312	76
ESC_RA3462AA	11038	286
ESC_RA3461AA	131	653
ESC_RA3460AA	58	198
ESC_RA3459AA	38	669
ESC_RA3458AA	140	738
ESC_RA3457AA	140	738
ESC_RA3456AA	11	936
ESC_RA3455AA	140	738
ESC_RA3454AA	21	156
ESC_RA3453AA	11	936
ESC_RA3452AA	5245	118
ESC_RA3451AA	4638	123
ESC_RA3450AA	4638	123
ESC_RA3449AA	11037	0
ESC_RA3448AA	11036	83
ESC_RA3447AA	11025	257
ESC_RA3445AA	167	512
ESC_RA3443AA	48	76
ESC_RA3442AA	5759	109
ESC_RA3441AA	10	512
ESC_RA3440AA	11027	696
ESC_RA3439AA	10	512
ESC_RA3438AA	11030	512
ESC_RA3437AA	48	76
ESC_RA3433AA	38	669
ESC_RA3432AA	2003	669
ESC_RA3431AA	648	359
ESC_RA3430AA	131	653
ESC_RA3429AA	131	653
ESC_RA3428AA	88	160
ESC_RA3426AA	388	156
ESC_RA3415AA	167	512
ESC_RA3414AA	1818	71

ESC_RA3413AA	117	592
ESC_RA3412AA	117	592
ESC_RA3411AA	11026	512
ESC_RA3410AA	1818	71
ESC_RA3409AA	1818	71
ESC_RA3408AA	1818	71
ESC_RA3407AA	1818	71
ESC_RA3406AA	1818	71
ESC_RA3405AA	117	592
ESC_RA3404AA	95	738
ESC_RA3403AA	1914	956
ESC_RA3402AA	117	592
ESC_RA3401AA	95	738
ESC_RA3400AA	93	71
ESC_RA3399AA	69	387
ESC_RA3397AA	69	387
ESC_RA3396AA	69	387
ESC_RA3394AA	4710	71
ESC_RA3393AA	69	387
ESC_RA3392AA	57	50
ESC_RA3391AA	117	592
ESC_RA3390AA	95	738
ESC_RA3389AA	95	738
ESC_RA3388AA	95	738
ESC_RA3387AA	69	387
ESC_RA3386AA	69	387
ESC_RA3384AA	95	738
ESC_RA3383AA	95	738
ESC_RA3382AA	95	738
ESC_RA3381AA	355	764
ESC_RA3380AA	355	764
ESC_RA3379AA	95	738
ESC_RA3378AA	95	738
ESC_RA3377AA	117	592
ESC_RA3376AA	117	592
ESC_RA3375AA	95	738
ESC_RA3374AA	355	764
ESC_RA3373AA	117	592
ESC_RA3372AA	95	738
ESC_RA3371AA	117	592
ESC_RA3370AA	95	738
ESC_RA3369AA	95	738
ESC_RA3368AA	95	738
ESC_RA3367AA	155	175
ESC_RA3366AA	752	512
ESC_RA3365AA	155	175
ESC_RA3364AA	69	387
ESC_RA3363AA	69	387
ESC_RA3361AA	95	738
ESC_RA3360AA	155	175

ESC_RA3359AA	69	387
ESC_RA3358AA	69	387
ESC_RA3357AA	93	71
ESC_RA3355AA	95	738
ESC_RA3354AA	117	592
ESC_RA3353AA	752	512
ESC_RA3352AA	11029	212
ESC_RA3351AA	117	592
ESC_RA3350AA	95	738
ESC_RA3349AA	95	738
ESC_RA3348AA	69	387
ESC_RA3347AA	69	387
ESC_RA3346AA	69	387
ESC_RA3345AA	69	387
ESC_RA3344AA	541	701
ESC_RA3343AA	10	512
ESC_RA3339AA	3998	198
ESC_RA3338AA	7576	903
ESC_RA3334AA	10	512
ESC_RA3332AA	162	924
ESC_RA3326AA	8097	212
ESC_RA3325AA	5285	108
ESC_RA3324AA	58	198
ESC_RA3322AA	69	387
ESC_RA3321AA	362	234
ESC_RA3319AA	155	175
ESC_RA3318AA	2521	257
ESC_RA3317AA	12	626
ESC_RA3316AA	182	246
ESC_RA3315AA	182	246
ESC_RA3314AA	58	198
ESC_RA3313AA	182	246
ESC_RA3312AA	11028	198
ESC_RA3311AA	11028	198
ESC_RA3310AA	11028	198
ESC_RA3309AA	11028	198
ESC_RA3308AA	11028	198
ESC_RA3307AA	11028	198
ESC_RA3306AA	11028	198
ESC_RA3304AA	1196	257
ESC_RA3303AA	607	355
ESC_RA3302AA	200	198
ESC_RA3301AA	117	592
ESC_RA3299AA	58	198
ESC_RA3298AA	362	234
ESC_RA3297AA	1431	257
ESC_RA3296AA	69	387
ESC_RA3295AA	38	669
ESC_RA3294AA	11035	453
ESC_RA3293AA	162	924

ESC_RA3292AA	3640	257
ESC_RA3291AA	925	387
ESC_RA3290AA	10	512
ESC_RA3289AA	69	387
ESC_RA3288AA	131	653
ESC_RA3287AA	361	512
ESC_RA3286AA	69	387
ESC_RA3285AA	44	512
ESC_RA3284AA	10	512
ESC_RA3283AA	69	387
ESC_RA3282AA	10	512
ESC_RA3281AA	216	512
ESC_RA3280AA	131	653
ESC_RA3279AA	10	512
ESC_RA3278AA	131	653
ESC_RA3277AA	405	923
ESC_RA3276AA	783	204
ESC_RA3275AA	973	968
ESC_RA3274AA	543	842
ESC_RA3273AA	11041	512
ESC_RA3272AA	11040	114
ESC_RA3271AA	3638	737
ESC_RA3270AA	2017	768
ESC_RA3269AA	11039	257
ESC_RA3268AA	8583	307
ESC_RA3267AA	10	512
ESC_RA3266AA	133	373
ESC_RA3265AA	133	373
ESC_RA3264AA	4038	257
ESC_RA3263AA	714	318
ESC_RA3261AA	1170	629
ESC_RA3260AA	182	246
ESC_RA3259AA	10	512
ESC_RA3258AA	224	198
ESC_RA3257AA	182	246
ESC_RA3256AA	182	246
ESC_RA3255AA	453	935
ESC_RA3254AA	182	246
ESC_RA3252AA	117	592
ESC_RA3251AA	44	512
ESC_RA3250AA	537	756
ESC_RA3249AA	942	198
ESC_RA3248AA	1170	629
ESC_RA3247AA	357	654
ESC_RA3246AA	58	198
ESC_RA3244AA	3998	198
ESC_RA3243AA	155	175
ESC_RA3241AA	563	445
ESC_RA3240AA	6623	257
ESC_RA3237AA	69	387

ESC_RA3235AA	372	424
ESC_RA3233AA	8611	422
ESC_RA3232AA	135	653
ESC_RA3231AA	129	257
ESC_RA3229AA	69	387
ESC_RA3228AA	95	738
ESC_RA3227AA	182	246
ESC_RA3226AA	182	246
ESC_RA3221AA	11028	198
ESC_RA3220AA	69	387
ESC_RA3219AA	11028	198
ESC_RA3218AA	11028	198
ESC_RA3217AA	11028	198
ESC_RA3216AA	11028	198
ESC_RA3215AA	11028	198
ESC_RA3214AA	11028	198
ESC_RA3213AA	11028	198
ESC_RA3212AA	1011	198
ESC_RA3211AA	1485	408
ESC_RA3210AA	34	504
ESC_RA3209AA	155	175
ESC_RA3208AA	88	160
ESC_RA3207AA	3640	257
ESC_RA3206AA	58	198
ESC_RA3205AA	10	512
ESC_RA3204AA	925	387
ESC_RA3203AA	361	512
ESC_RA3202AA	69	387
ESC_RA3201AA	182	246
ESC_RA3200AA	10	512
ESC_RA3199AA	130	387
ESC_RA3198AA	48	76
ESC_RA3197AA	182	246
ESC_RA3196AA	224	198
ESC_RA3195AA	11110	968
ESC_RA3194AA	165	504
ESC_RA3193AA	744	512
ESC_RA3192AA	744	512
ESC_RA3191AA	182	246
ESC_RA3190AA	607	355
ESC_RA3189AA	182	246
ESC_RA3188AA	182	246
ESC_RA3187AA	405	923
ESC_RA3186AA	182	246
ESC_RA3185AA	182	246
ESC_RA3184AA	182	246
ESC_RA3183AA	189	504
ESC_RA3181AA	2076	400
ESC_RA3180AA	3998	198
ESC_RA3179AA	3998	198

ESC_RA3178AA	720	900
ESC_RA3177AA	58	198
ESC_RA3176AA	162	924
ESC_RA3174AA	108	257
ESC_RA3173AA	196	257
ESC_RA3172AA	4213	251
ESC_RA3171AA	10	512
ESC_RA3170AA	744	512
ESC_RA3169AA	69	387
ESC_RA3168AA	1914	956
ESC_RA3167AA	88	160
ESC_RA3166AA	117	592
ESC_RA3165AA	746	789
ESC_RA3164AA	10	512
ESC_RA3163AA	648	359
ESC_RA3162AA	332	257
ESC_RA3161AA	457	595
ESC_RA3160AA	332	257
ESC_RA3158AA	58	198
ESC_RA3157AA	38	669
ESC_RA3155AA	3247	778
ESC_RA3154AA	11028	198
ESC_RA3153AA	11028	198
ESC_RA3149AA	399	198
ESC_RA3148AA	399	198
ESC_RA3147AA	10	512
ESC_RA3146AA	10	512
ESC_RA3145AA	410	156
ESC_RA3144AA	130	387
ESC_RA3143AA	130	387
ESC_RA3141AA	182	246
ESC_RA3140AA	3075	701
ESC_RA3139AA	38	669
ESC_RA3138AA	48	76
ESC_RA3137AA	448	257
ESC_RA3136AA	448	257
ESC_RA3135AA	448	257
ESC_RA3134AA	448	257
ESC_RA3133AA	155	175
ESC_RA3132AA	1485	408
ESC_RA3131AA	88	160
ESC_RA3130AA	361	512
ESC_RA3129AA	297	234
ESC_RA3128AA	1485	408
ESC_RA3127AA	536	141
ESC_RA3126AA	11028	198
ESC_RA3125AA	11028	198
ESC_RA3124AA	11028	198
ESC_RA3123AA	11028	198
ESC_RA3122AA	101	696

ESC_RA3121AA	11028	198
ESC_RA3119AA	536	141
ESC_RA3118AA	11028	198
ESC_RA3117AA	1140	934
ESC_RA3116AA	940	257
ESC_RA3113AA	744	512
ESC_RA3111AA	332	257
ESC_RA3110AA	23	156
ESC_RA3109AA	1429	198
ESC_RA3108AA	372	424
ESC_RA3107AA	5566	373
ESC_RA3106AA	5566	373
ESC_RA3104AA	69	387
ESC_RA3101AA	10	512
ESC_RA3096AA	348	175
ESC_RA3095AA	117	592
ESC_RA3094AA	162	924
ESC_RA3092AA	2599	257
ESC_RA3091AA	131	653
ESC_RA3088AA	3171	515
ESC_RA3087AA	5748	924
ESC_RA3084AA	58	198
ESC_RA3083AA	1170	629
ESC_RA3082AA	117	592
ESC_RA3081AA	357	654
ESC_RA3080AA	448	257
ESC_RA3079AA	1485	408
ESC_RA3078AA	744	512
ESC_RA3077AA	448	257
ESC_RA3076AA	69	387
ESC_RA3075AA	10	512
ESC_RA3074AA	182	246
ESC_RA3073AA	182	246
ESC_RA3072AA	410	156
ESC_RA3071AA	182	246
ESC_RA3069AA	117	592
ESC_RA3068AA	182	246
ESC_RA3067AA	1585	512
ESC_RA3066AA	182	246
ESC_RA3065AA	224	198
ESC_RA3064AA	182	246
ESC_RA3063AA	182	246
ESC_RA3062AA	182	246
ESC_RA3061AA	182	246
ESC_RA3060AA	182	246
ESC_RA3059AA	182	246
ESC_RA3058AA	182	246
ESC_RA3057AA	410	156
ESC_RA3056AA	182	246
ESC_RA3055AA	182	246

ESC_RA3054AA	44	512
ESC_RA3053AA	131	653
ESC_RA3052AA	117	592
ESC_RA3051AA	540	286
ESC_RA3050AA	1196	257
ESC_RA3049AA	457	595
ESC_RA3048AA	1585	512
ESC_RA3047AA	131	653
ESC_RA3046AA	201	257
ESC_RA3045AA	93	71
ESC_RA3044AA	720	900
ESC_RA3043AA	69	387
ESC_RA3042AA	1485	408
ESC_RA3041AA	1170	629
ESC_RA3040AA	216	512
ESC_RA3039AA	744	512
ESC_RA3038AA	11045	447
ESC_RA3037AA	11044	512
ESC_RA3036AA	1485	408
ESC_RA3034AA	453	935
ESC_RA3033AA	2178	924
ESC_RA3032AA	167	512
ESC_RA3031AA	332	257
ESC_RA3030AA	131	653
ESC_RA3029AA	332	257
ESC_RA3028AA	720	900
ESC_RA3027AA	23	156
ESC_RA3026AA	58	198
ESC_RA3025AA	4038	257
ESC_RA3024AA	428	654
ESC_RA3023AA	2014	406
ESC_RA3022AA	345	175
ESC_RA3020AA	227	504
ESC_RA3019AA	362	234
ESC_RA3018AA	409	504
ESC_RA3017AA	182	246
ESC_RA3016AA	182	246
ESC_RA3015AA	182	246
ESC_RA3014AA	182	246
ESC_RA3013AA	617	511
ESC_RA3012AA	182	246
ESC_RA3011AA	182	246
ESC_RA3010AA	295	160
ESC_RA3009AA	182	246
ESC_RA3008AA	182	246
ESC_RA3007AA	11028	198
ESC_RA3006AA	409	504
ESC_RA3005AA	536	141
ESC_RA3004AA	536	141
ESC_RA3003AA	635	141

ESC_RA3002AA	69	387
ESC_RA3001AA	69	387
ESC_RA3000AA	607	355
ESC_RA2999AA	11028	198
ESC_RA2998AA	11028	198
ESC_RA2996AA	58	198
ESC_RA2995AA	38	669
ESC_RA2994AA	48	76
ESC_RA2993AA	414	387
ESC_RA2992AA	1955	900
ESC_RA2991AA	156	175
ESC_RA2990AA	95	738
ESC_RA2989AA	56	198
ESC_RA2988AA	648	359
ESC_RA2984AA	88	160
ESC_RA2979AA	58	198
ESC_RA2978AA	2074	858
ESC_RA2977AA	607	355
ESC_RA2976AA	10	512
ESC_RA2975AA	648	359
ESC_RA2974AA	90	156
ESC_RA2973AA	58	198
ESC_RA2972AA	648	359
ESC_RA2971AA	2705	504
ESC_RA2970AA	388	156
ESC_RA2969AA	357	654
ESC_RA2968AA	189	504
ESC_RA2967AA	677	257
ESC_RA2965AA	69	387
ESC_RA2964AA	11043	207
ESC_RA2963AA	602	257
ESC_RA2962AA	295	160
ESC_RA2961AA	11042	565
ESC_RA2960AA	372	424
ESC_RA2959AA	1079	235
ESC_RA2957AA	46	447
ESC_RA2956AA	5566	373
ESC_RA2955AA	8611	422
ESC_RA2954AA	135	653
ESC_RA2953AA	372	424
ESC_RA2952AA	563	445
ESC_RA2951AA	7347	373
ESC_RA2950AA	133	373
ESC_RA2949AA	5566	373
ESC_RA2948AA	11109	373
ESC_RA2947AA	23	156
ESC_RA2946AA	23	156
ESC_RA2945AA	5566	373
ESC_RA2944AA	11109	373
ESC_RA2943AA	5566	373

ESC_RA2942AA	5566	373
ESC_RA2941AA	297	234
ESC_RA2940AA	182	246
ESC_RA2939AA	182	246
ESC_RA2937AA	182	246
ESC_RA2936AA	48	76
ESC_RA2935AA	48	76
ESC_RA2934AA	182	246
ESC_RA2933AA	182	246
ESC_RA2932AA	165	504
ESC_RA2931AA	182	246
ESC_RA2930AA	744	512
ESC_RA2929AA	206	286
ESC_RA2928AA	1434	512
ESC_RA2927AA	10777	824
ESC_RA2926AA	10	512
ESC_RA2925AA	165	504
ESC_RA2924AA	657	592
ESC_RA2923AA	10	512
ESC_RA2922AA	543	842
ESC_RA2921AA	11108	71
ESC_RA2920AA	95	738
ESC_RA2919AA	206	286
ESC_RA2918AA	10	512
ESC_RA2917AA	10	512
ESC_RA2916AA	100	504
ESC_RA2915AA	20	198
ESC_RA2914AA	100	504
ESC_RA2913AA	5759	109
ESC_RA2912AA	3744	519
ESC_RA2911AA	641	935
ESC_RA2910AA	10	512
ESC_RA2909AA	88	160
ESC_RA2908AA	6223	512
ESC_RA2907AA	11107	373
ESC_RA2906AA	59	217
ESC_RA2905AA	69	387
ESC_RA2904AA	973	968
ESC_RA2903AA	10	512
ESC_RA2902AA	392	234
ESC_RA2901AA	1125	198
ESC_RA2900AA	297	234
ESC_RA2899AA	2328	257
ESC_RA2898AA	216	512
ESC_RA2897AA	731	285
ESC_RA2896AA	10	512
ESC_RA2895AA	11103	789
ESC_RA2894AA	48	76
ESC_RA2893AA	539	257
ESC_RA2892AA	56	198

ESC_RA2891AA	847	257
ESC_RA2890AA	847	257
ESC_RA2889AA	11102	387
ESC_RA2888AA	446	257
ESC_RA2887AA	68	386
ESC_RA2886AA	410	156
ESC_RA2885AA	101	696
ESC_RA2884AA	34	504
ESC_RA2883AA	69	387
ESC_RA2882AA	2768	198
ESC_RA2881AA	9566	900
ESC_RA2880AA	9008	627
ESC_RA2878AA	3910	672
ESC_RA2877AA	73	737
ESC_RA2876AA	12	626
ESC_RA2875AA	783	204
ESC_RA2874AA	95	738
ESC_RA2872AA	680	367
ESC_RA2871AA	11101	268
ESC_RA2870AA	11100	924
ESC_RA2869AA	40	198
ESC_RA2868AA	141	644
ESC_RA2867AA	5296	257
ESC_RA2866AA	7630	373
ESC_RA2865AA	201	257
ESC_RA2864AA	2614	975
ESC_RA2863AA	11099	422
ESC_RA2862AA	155	175
ESC_RA2861AA	155	175
ESC_RA2860AA	58	198
ESC_RA2859AA	1303	791
ESC_RA2858AA	278	924
ESC_RA2857AA	1510	4
ESC_RA2856AA	69	387
ESC_RA2855AA	155	175
ESC_RA2854AA	278	924
ESC_RA2853AA	1091	512
ESC_RA2852AA	10	512
ESC_RA2850AA	11098	924
ESC_RA2849AA	372	424
ESC_RA2847AA	73	737
ESC_RA2846AA	929	742
ESC_RA2845AA	73	737
ESC_RA2844AA	537	756
ESC_RA2843AA	155	175
ESC_RA2842AA	1790	198
ESC_RA2841AA	6188	257
ESC_RA2840AA	111	175
ESC_RA2839AA	2521	257
ESC_RA2838AA	442	257

ESC_RA2837AA	3576	175
ESC_RA2836AA	8927	387
ESC_RA2835AA	11097	256
ESC_RA2834AA	1510	4
ESC_RA2833AA	681	393
ESC_RA2832AA	4481	257
ESC_RA2831AA	398	445
ESC_RA2830AA	11050	387
ESC_RA2829AA	2073	198
ESC_RA2828AA	34	504
ESC_RA2827AA	399	198
ESC_RA2826AA	399	198
ESC_RA2825AA	540	286
ESC_RA2824AA	446	257
ESC_RA2823AA	88	160
ESC_RA2822AA	216	512
ESC_RA2821AA	635	141
ESC_RA2820AA	216	512
ESC_RA2819AA	5913	935
ESC_RA2818AA	10	512
ESC_RA2816AA	23	156
ESC_RA2815AA	69	387
ESC_RA2814AA	117	592
ESC_RA2813AA	2614	975
ESC_RA2812AA	1727	257
ESC_RA2811AA	491	626
ESC_RA2810AA	162	924
ESC_RA2809AA	11096	512
ESC_RA2808AA	155	175
ESC_RA2807AA	23	156
ESC_RA2806AA	744	512
ESC_RA2805AA	57	50
ESC_RA2804AA	23	156
ESC_RA2803AA	48	76
ESC_RA2802AA	1819	808
ESC_RA2801AA	2522	198
ESC_RA2800AA	11052	257
ESC_RA2799AA	117	592
ESC_RA2798AA	11051	373
ESC_RA2797AA	162	924
ESC_RA2796AA	2280	212
ESC_RA2795AA	11050	387
ESC_RA2794AA	1508	261
ESC_RA2793AA	708	924
ESC_RA2792AA	5597	72
ESC_RA2790AA	40	198
ESC_RA2789AA	602	257
ESC_RA2788AA	3553	512
ESC_RA2787AA	13	257
ESC_RA2786AA	69	387

ESC_RA2785AA	1431	257
ESC_RA2784AA	1137	512
ESC_RA2783AA	1137	512
ESC_RA2782AA	635	141
ESC_RA2781AA	1140	934
ESC_RA2780AA	212	198
ESC_RA2779AA	216	512
ESC_RA2778AA	216	512
ESC_RA2777AA	132	924
ESC_RA2776AA	1170	629
ESC_RA2775AA	1408	445
ESC_RA2774AA	973	968
ESC_RA2773AA	7900	430
ESC_RA2772AA	5597	72
ESC_RA2771AA	20	198
ESC_RA2770AA	129	257
ESC_RA2769AA	155	175
ESC_RA2768AA	11049	52
ESC_RA2767AA	533	198
ESC_RA2766AA	351	268
ESC_RA2765AA	501	900
ESC_RA2764AA	224	198
ESC_RA2763AA	117	592
ESC_RA2762AA	69	387
ESC_RA2761AA	93	71
ESC_RA2760AA	297	234
ESC_RA2759AA	10	512
ESC_RA2758AA	117	592
ESC_RA2757AA	117	592
ESC_RA2756AA	1286	474
ESC_RA2755AA	5825	924
ESC_RA2754AA	155	175
ESC_RA2753AA	4541	676
ESC_RA2752AA	8923	987
ESC_RA2751AA	392	234
ESC_RA2750AA	10	512
ESC_RA2749AA	847	257
ESC_RA2748AA	4198	878
ESC_RA2747AA	196	257
ESC_RA2746AA	2521	257
ESC_RA2745AA	20	198
ESC_RA2744AA	1406	174
ESC_RA2743AA	405	923
ESC_RA2742AA	10	512
ESC_RA2741AA	533	198
ESC_RA2740AA	297	234
ESC_RA2739AA	1611	198
ESC_RA2738AA	20	198
ESC_RA2737AA	40	198
ESC_RA2736AA	1642	198

ESC_RA2735AA	20	198
ESC_RA2734AA	40	198
ESC_RA2733AA	20	198
ESC_RA2732AA	40	198
ESC_RA2731AA	10	512
ESC_RA2730AA	88	160
ESC_RA2729AA	7093	868
ESC_RA2728AA	410	156
ESC_RA2727AA	410	156
ESC_RA2726AA	10	512
ESC_RA2725AA	871	447
ESC_RA2724AA	641	935
ESC_RA2723AA	3574	257
ESC_RA2722AA	10	512
ESC_RA2721AA	101	696
ESC_RA2720AA	136	392
ESC_RA2719AA	2354	257
ESC_RA2718AA	4054	198
ESC_RA2717AA	216	512
ESC_RA2716AA	2522	198
ESC_RA2715AA	23	156
ESC_RA2714AA	2602	257
ESC_RA2713AA	6096	257
ESC_RA2712AA	11048	738
ESC_RA2711AA	10	512
ESC_RA2710AA	10955	234
ESC_RA2709AA	11095	175
ESC_RA2708AA	11047	512
ESC_RA2707AA	398	445
ESC_RA2706AA	345	175
ESC_RA2705AA	348	175
ESC_RA2704AA	4088	198
ESC_RA2703AA	11046	924
ESC_RA2702AA	661	92
ESC_RA2701AA	164	175
ESC_RA2700AA	3601	653
ESC_RA2699AA	906	198
ESC_RA2698AA	69	387
ESC_RA2697AA	457	595
ESC_RA2696AA	540	286
ESC_RA2695AA	4388	198
ESC_RA2694AA	602	257
ESC_RA2693AA	59	217
ESC_RA2692AA	23	156
ESC_RA2691AA	23	156
ESC_RA2690AA	399	198
ESC_RA2689AA	359	696
ESC_RA2688AA	101	696
ESC_RA2687AA	795	924
ESC_RA2686AA	101	696

ESC_RA2685AA	1434	512
ESC_RA2684AA	10	512
ESC_RA2683AA	191	198
ESC_RA2682AA	13	257
ESC_RA2681AA	117	592
ESC_RA2680AA	131	653
ESC_RA2679AA	2005	175
ESC_RA2678AA	2280	212
ESC_RA2677AA	187	257
ESC_RA2676AA	1665	601
ESC_RA2675AA	58	198
ESC_RA2674AA	1665	601
ESC_RA2673AA	154	257
ESC_RA2672AA	101	696
ESC_RA2671AA	1463	263
ESC_RA2670AA	58	198
ESC_RA2669AA	1665	601
ESC_RA2668AA	10	512
ESC_RA2666AA	10	512
ESC_RA2665AA	1510	4
ESC_RA2664AA	583	768
ESC_RA2663AA	942	198
ESC_RA2662AA	11094	76
ESC_RA2661AA	9721	234
ESC_RA2660AA	59	217
ESC_RA2659AA	2161	198
ESC_RA2658AA	69	387
ESC_RA2657AA	11053	630
ESC_RA2656AA	48	76
ESC_RA2655AA	398	445
ESC_RA2654AA	20	198
ESC_RA2653AA	59	217
ESC_RA2652AA	155	175
ESC_RA2651AA	1072	355
ESC_RA2650AA	95	738
ESC_RA2649AA	327	204
ESC_RA2648AA	80	731
ESC_RA2647AA	372	424
ESC_RA2646AA	2521	257
ESC_RA2645AA	297	234
ESC_RA2644AA	212	198
ESC_RA2643AA	410	156
ESC_RA2642AA	1629	924
ESC_RA2641AA	58	198
ESC_RA2640AA	297	234
ESC_RA2639AA	648	359
ESC_RA2638AA	847	257
ESC_RA2637AA	847	257
ESC_RA2636AA	2178	924
ESC_RA2635AA	847	257

ESC_RA2634AA	6345	199
ESC_RA2633AA	847	257
ESC_RA2632AA	847	257
ESC_RA2631AA	847	257
ESC_RA2630AA	155	175
ESC_RA2629AA	906	198
ESC_RA2628AA	906	198
ESC_RA2627AA	34	504
ESC_RA2625AA	10	512
ESC_RA2624AA	11684	311
ESC_RA2623AA	906	198
ESC_RA2622AA	2178	924
ESC_RA2621AA	5759	109
ESC_RA2620AA	6998	629
ESC_RA2619AA	223	198
ESC_RA2618AA	2087	118
ESC_RA2617AA	567	740
ESC_RA2616AA	11027	696
ESC_RA2615AA	2087	118
ESC_RA2614AA	5759	109
ESC_RA2613AA	2087	118
ESC_RA2612AA	5759	109
ESC_RA2611AA	5759	109
ESC_RA2610AA	5759	109
ESC_RA2608AA	410	156
ESC_RA2607AA	405	923
ESC_RA2606AA	410	156
ESC_RA2605AA	410	156
ESC_RA2604AA	410	156
ESC_RA2603AA	410	156
ESC_RA2602AA	410	156
ESC_RA2601AA	410	156
ESC_RA2600AA	410	156
ESC_RA2599AA	410	156
ESC_RA2598AA	410	156
ESC_RA2597AA	410	156
ESC_RA2596AA	410	156
ESC_RA2595AA	410	156
ESC_RA2594AA	410	156
ESC_RA2593AA	410	156
ESC_RA2592AA	410	156
ESC_RA2591AA	410	156
ESC_RA2590AA	410	156
ESC_RA2589AA	410	156
ESC_RA2588AA	410	156
ESC_RA2587AA	410	156
ESC_RA2586AA	410	156
ESC_RA2585AA	410	156
ESC_RA2584AA	410	156
ESC_RA2583AA	410	156

ESC_RA2582AA	410	156
ESC_RA2581AA	410	156
ESC_RA2580AA	410	156
ESC_RA2579AA	410	156
ESC_RA2578AA	410	156
ESC_RA2577AA	410	156
ESC_RA2576AA	410	156
ESC_RA2575AA	410	156
ESC_RA2574AA	410	156
ESC_RA2573AA	410	156
ESC_RA2572AA	410	156
ESC_RA2571AA	410	156
ESC_RA2570AA	410	156
ESC_RA2569AA	410	156
ESC_RA2568AA	410	156
ESC_RA2567AA	410	156
ESC_RA2566AA	410	156
ESC_RA2565AA	410	156
ESC_RA2564AA	410	156
ESC_RA2563AA	410	156
ESC_RA2562AA	410	156
ESC_RA2561AA	410	156
ESC_RA2560AA	410	156
ESC_RA2559AA	410	156
ESC_RA2558AA	410	156
ESC_RA2557AA	410	156
ESC_RA2556AA	410	156
ESC_RA2555AA	410	156
ESC_RA2554AA	410	156
ESC_RA2553AA	410	156
ESC_RA2552AA	410	156
ESC_RA2551AA	410	156
ESC_RA2550AA	372	424
ESC_RA2549AA	38	669
ESC_RA2548AA	80	731
ESC_RA2547AA	625	756
ESC_RA2546AA	457	595
ESC_RA2545AA	167	512
ESC_RA2544AA	131	653
ESC_RA2543AA	73	737
ESC_RA2542AA	131	653
ESC_RA2541AA	224	198
ESC_RA2540AA	38	669
ESC_RA2539AA	405	923
ESC_RA2538AA	38	669
ESC_RA2537AA	973	968
ESC_RA2536AA	354	15
ESC_RA2535AA	224	198
ESC_RA2534AA	224	198
ESC_RA2533AA	410	156

ESC_RA2532AA	88	160
ESC_RA2531AA	11093	380
ESC_RA2530AA	224	198
ESC_RA2529AA	539	257
ESC_RA2528AA	23	156
ESC_RA2527AA	448	257
ESC_RA2526AA	624	359
ESC_RA2525AA	155	175
ESC_RA2524AA	354	15
ESC_RA2523AA	362	234
ESC_RA2522AA	131	653
ESC_RA2521AA	131	653
ESC_RA2520AA	428	654
ESC_RA2519AA	1284	604
ESC_RA2518AA	12	626
ESC_RA2517AA	617	511
ESC_RA2516AA	73	737
ESC_RA2515AA	372	424
ESC_RA2514AA	539	257
ESC_RA2513AA	131	653
ESC_RA2512AA	88	160
ESC_RA2511AA	744	512
ESC_RA2510AA	744	512
ESC_RA2509AA	362	234
ESC_RA2508AA	131	653
ESC_RA2507AA	372	424
ESC_RA2491AA	52	71
ESC_RA2489AA	53	863
ESC_RA2488AA	53	863
ESC_RA2487AA	54	256
ESC_RA2485AA	56	198
ESC_RA2482AA	73	737
ESC_RA2479AA	59	217
ESC_RA2478AA	59	217
ESC_RA2477AA	61	936
ESC_RA2475AA	62	574
ESC_RA2471AA	64	978
ESC_RA2465AA	70	670
ESC_RA2464AA	405	923
ESC_RA2463AA	72	923
ESC_RA2461AA	345	175
ESC_RA2453AA	79	744
ESC_RA2452AA	12	626
ESC_RA2444AA	84	175
ESC_RA2443AA	85	234
ESC_RA2442AA	86	935
ESC_RA2441AA	88	160
ESC_RA2438AA	23	156
ESC_RA2431AA	131	653
ESC_RA2430AA	131	653

ESC_RA2429AA	73	737
ESC_RA2428AA	73	737
ESC_RA2427AA	73	737
ESC_RA2426AA	73	737
ESC_RA2425AA	73	737
ESC_RA2424AA	73	737
ESC_RA2423AA	73	737
ESC_RA2422AA	73	737
ESC_RA2421AA	1193	761
ESC_RA2420AA	1193	761
ESC_RA2419AA	131	653
ESC_RA2418AA	1193	761
ESC_RA2417AA	1193	761
ESC_RA2416AA	131	653
ESC_RA2415AA	131	653
ESC_RA2414AA	131	653
ESC_RA2413AA	131	653
ESC_RA2412AA	131	653
ESC_RA2411AA	131	653
ESC_RA2410AA	131	653
ESC_RA2409AA	131	653
ESC_RA2408AA	131	653
ESC_RA2407AA	131	653
ESC_RA2406AA	131	653
ESC_RA2405AA	131	653
ESC_RA2404AA	73	737
ESC_RA2403AA	73	737
ESC_RA2402AA	73	737
ESC_RA2401AA	131	653
ESC_RA2400AA	131	653
ESC_RA2399AA	131	653
ESC_RA2398AA	73	737
ESC_RA2397AA	73	737
ESC_RA2314AA	349	670
ESC_RA2313AA	10	512
ESC_RA2312AA	10	512
ESC_RA2311AA	10	512
ESC_RA2310AA	10	512
ESC_RA2309AA	10	512
ESC_RA2308AA	10	512
ESC_RA2307AA	10	512
ESC_RA2306AA	10	512
ESC_RA2305AA	10	512
ESC_RA2301AA	10	512
ESC_RA2290AA	10	512
ESC_RA2279AA	10	512
ESC_RA2268AA	10	512
ESC_RA2260AA	10	512
ESC_RA2259AA	10	512
ESC_RA2258AA	10	512

ESC_RA2257AA	10	512
ESC_RA2256AA	446	257
ESC_RA2255AA	342	286
ESC_RA2254AA	17	191
ESC_RA2253AA	21	156
ESC_RA2252AA	17	191
ESC_RA2251AA	17	191
ESC_RA2250AA	655	926
ESC_RA2249AA	17	191
ESC_RA2248AA	17	191
ESC_RA2247AA	16	156
ESC_RA2246AA	306	286
ESC_RA2245AA	675	234
ESC_RA2244AA	117	592
ESC_RA2243AA	17	191
ESC_RA2242AA	16	156
ESC_RA2241AA	16	156
ESC_RA2240AA	21	156
ESC_RA2239AA	17	191
ESC_RA2238AA	21	156
ESC_RA2237AA	3057	672
ESC_RA2236AA	17	191
ESC_RA2235AA	16	156
ESC_RA2234AA	21	156
ESC_RA2232AA	32	270
ESC_RA2231AA	17	191
ESC_RA2230AA	21	156
ESC_RA2229AA	21	156
ESC_RA2228AA	659	286
ESC_RA2227AA	17	191
ESC_RA2226AA	17	191
ESC_RA2225AA	17	191
ESC_RA2224AA	16	156
ESC_RA2223AA	17	191
ESC_RA2222AA	17	191
ESC_RA2221AA	17	191
ESC_RA2220AA	16	156
ESC_RA2219AA	17	191
ESC_RA2218AA	17	191
ESC_RA2217AA	16	156
ESC_RA2216AA	16	156
ESC_RA2215AA	32	270
ESC_RA2214AA	16	156
ESC_RA2213AA	21	156
ESC_RA2212AA	21	156
ESC_RA2211AA	17	191
ESC_RA2210AA	12	626
ESC_RA2209AA	17	191
ESC_RA2208AA	11055	191
ESC_RA2207AA	17	191

ESC_RA2206AA	16	156
ESC_RA2205AA	21	156
ESC_RA2204AA	21	156
ESC_RA2203AA	21	156
ESC_RA2201AA	16	156
ESC_RA2200AA	16	156
ESC_RA2199AA	642	924
ESC_RA2198AA	21	156
ESC_RA2197AA	16	156
ESC_RA2196AA	16	156
ESC_RA2195AA	21	156
ESC_RA2194AA	1967	191
ESC_RA2193AA	11054	198
ESC_RA2192AA	21	156
ESC_RA2191AA	21	156
ESC_RA2190AA	656	789
ESC_RA2189AA	348	175
ESC_RA2188AA	21	156
ESC_RA2187AA	642	924
ESC_RA2186AA	17	191
ESC_RA2185AA	21	156
ESC_RA2184AA	8264	586
ESC_RA2183AA	16	156
ESC_RA2182AA	17	191
ESC_RA2181AA	17	191
ESC_RA2180AA	17	191
ESC_RA2179AA	17	191
ESC_RA2178AA	348	175
ESC_RA2177AA	17	191
ESC_RA2176AA	17	191
ESC_RA2175AA	32	270
ESC_RA2174AA	17	191
ESC_RA2173AA	17	191
ESC_RA2172AA	70	670
ESC_RA2171AA	16	156
ESC_RA2170AA	5426	156
ESC_RA2169AA	17	191
ESC_RA2168AA	17	191
ESC_RA2167AA	675	234
ESC_RA2166AA	21	156
ESC_RA2165AA	17	191
ESC_RA2164AA	17	191
ESC_RA2163AA	16	156
ESC_RA2161AA	33	198
ESC_RA2160AA	17	191
ESC_RA2159AA	679	257
ESC_RA2158AA	21	156
ESC_RA2157AA	17	191
ESC_RA2156AA	17	191
ESC_RA2155AA	342	286

ESC_RA2154AA	7093	868
ESC_RA2153AA	16	156
ESC_RA2152AA	17	191
ESC_RA2151AA	17	191
ESC_RA2150AA	17	191
ESC_RA2149AA	677	257
ESC_RA2148AA	17	191
ESC_RA2147AA	723	156
ESC_RA2146AA	32	270
ESC_RA2145AA	17	191
ESC_RA2144AA	154	257
ESC_RA2143AA	17	191
ESC_RA2142AA	17	191
ESC_RA2141AA	21	156
ESC_RA2140AA	17	191
ESC_RA2139AA	17	191
ESC_RA2138AA	17	191
ESC_RA2137AA	17	191
ESC_RA2136AA	17	191
ESC_RA2135AA	21	156
ESC_RA2133AA	342	286
ESC_RA2132AA	17	191
ESC_RA2131AA	6175	257
ESC_RA2130AA	6175	257
ESC_RA2129AA	21	156
ESC_RA2128AA	1792	156
ESC_RA2127AA	6175	257
ESC_RA2125AA	21	156
ESC_RA2124AA	17	191
ESC_RA2123AA	17	191
ESC_RA2122AA	17	191
ESC_RA2121AA	32	270
ESC_RA2120AA	16	156
ESC_RA2119AA	655	926
ESC_RA2118AA	17	191
ESC_RA2117AA	16	156
ESC_RA2116AA	11058	156
ESC_RA2115AA	21	156
ESC_RA2114AA	3695	924
ESC_RA2113AA	1967	191
ESC_RA2112AA	16	156
ESC_RA2111AA	32	270
ESC_RA2110AA	21	156
ESC_RA2109AA	343	286
ESC_RA2108AA	16	156
ESC_RA2107AA	16	156
ESC_RA2106AA	16	156
ESC_RA2105AA	17	191
ESC_RA2104AA	655	926
ESC_RA2103AA	16	156

ESC_RA2102AA	21	156
ESC_RA2101AA	40	198
ESC_RA2100AA	127	738
ESC_RA2099AA	38	669
ESC_RA2098AA	405	923
ESC_RA2097AA	38	669
ESC_RA2096AA	69	387
ESC_RA2095AA	131	653
ESC_RA2094AA	69	387
ESC_RA2093AA	405	923
ESC_RA2092AA	131	653
ESC_RA2091AA	349	670
ESC_RA2090AA	38	669
ESC_RA2089AA	69	387
ESC_RA2088AA	131	653
ESC_RA2087AA	131	653
ESC_RA2086AA	167	512
ESC_RA2085AA	11057	576
ESC_RA2084AA	58	198
ESC_RA2083AA	131	653
ESC_RA2082AA	405	923
ESC_RA2081AA	3033	737
ESC_RA2080AA	11056	761
ESC_RA2078AA	4214	824
ESC_RA2077AA	4214	824
ESC_RA2076AA	20	198
ESC_RA2075AA	10	512
ESC_RA2074AA	93	71
ESC_RA2073AA	93	71
ESC_RA2072AA	93	71
ESC_RA2071AA	93	71
ESC_RA2070AA	93	71
ESC_RA2069AA	93	71
ESC_RA2067AA	93	71
ESC_RA2066AA	93	71
ESC_RA2065AA	93	71
ESC_RA2064AA	93	71
ESC_RA2063AA	93	71
ESC_RA2062AA	93	71
ESC_RA2061AA	93	71
ESC_RA2060AA	93	71
ESC_RA2058AA	93	71
ESC_RA2057AA	93	71
ESC_RA2054AA	93	71
ESC_RA2053AA	93	71
ESC_RA2052AA	93	71
ESC_RA2051AA	93	71
ESC_RA2050AA	93	71
ESC_RA2049AA	93	71
ESC_RA2046AA	93	71

ESC_RA2045AA	93	71
ESC_RA2044AA	93	71
ESC_RA2043AA	93	71
ESC_RA2040AA	93	71
ESC_RA2039AA	93	71
ESC_RA2037AA	93	71
ESC_RA2035AA	93	71
ESC_RA2034AA	93	71
ESC_RA2033AA	93	71
ESC_RA2032AA	93	71
ESC_RA2031AA	93	71
ESC_RA2029AA	93	71
ESC_RA2027AA	93	71
ESC_RA2026AA	93	71
ESC_RA2024AA	93	71
ESC_RA2022AA	93	71
ESC_RA2021AA	93	71
ESC_RA2019AA	93	71
ESC_RA2018AA	93	71
ESC_RA2017AA	93	71
ESC_RA2016AA	93	71
ESC_RA2015AA	93	71
ESC_RA2013AA	93	71
ESC_RA2012AA	93	71
ESC_RA2011AA	93	71
ESC_RA2008AA	93	71
ESC_RA2007AA	93	71
ESC_RA2005AA	93	71
ESC_RA2004AA	93	71
ESC_RA2001AA	93	71
ESC_RA2000AA	93	71
ESC_RA1999AA	93	71
ESC_RA1998AA	93	71
ESC_RA1997AA	93	71
ESC_RA1992AA	93	71
ESC_RA1991AA	93	71
ESC_RA1989AA	93	71
ESC_RA1988AA	93	71
ESC_RA1987AA	93	71
ESC_RA1984AA	93	71
ESC_RA1980AA	93	71
ESC_RA1979AA	93	71
ESC_RA1978AA	93	71
ESC_RA1977AA	93	71
ESC_RA1976AA	93	71
ESC_RA1975AA	93	71
ESC_RA1974AA	93	71
ESC_RA1973AA	93	71
ESC_RA1971AA	93	71
ESC_RA1970AA	93	71

ESC_RA1969AA	93	71
ESC_RA1968AA	93	71
ESC_RA1966AA	93	71
ESC_RA1963AA	93	71
ESC_RA1962AA	93	71
ESC_RA1961AA	93	71
ESC_RA1960AA	93	71
ESC_RA1959AA	93	71
ESC_RA1958AA	648	359
ESC_RA1957AA	73	737
ESC_RA1956AA	131	653
ESC_RA1955AA	404	761
ESC_RA1954AA	12	626
ESC_RA1953AA	95	738
ESC_RA1952AA	131	653
ESC_RA1951AA	38	669
ESC_RA1950AA	73	737
ESC_RA1949AA	88	160
ESC_RA1948AA	131	653
ESC_RA1947AA	131	653
ESC_RA1946AA	69	387
ESC_RA1945AA	131	653
ESC_RA1944AA	131	653
ESC_RA1943AA	127	738
ESC_RA1942AA	131	653
ESC_RA1941AA	131	653
ESC_RA1940AA	95	738
ESC_RA1939AA	131	653
ESC_RA1938AA	131	653
ESC_RA1937AA	12	626
ESC_RA1936AA	73	737
ESC_RA1935AA	73	737
ESC_RA1934AA	73	737
ESC_RA1933AA	131	653
ESC_RA1932AA	73	737
ESC_RA1931AA	131	653
ESC_RA1930AA	127	738
ESC_RA1929AA	5903	180
ESC_RA1928AA	73	737
ESC_RA1927AA	127	738
ESC_RA1926AA	399	198
ESC_RA1925AA	219	877
ESC_RA1924AA	69	387
ESC_RA1923AA	744	512
ESC_RA1922AA	127	738
ESC_RA1921AA	69	387
ESC_RA1920AA	117	592
ESC_RA1919AA	636	653
ESC_RA1918AA	131	653
ESC_RA1917AA	11091	738

ESC_RA1916AA	127	738
ESC_RA1915AA	73	737
ESC_RA1914AA	73	737
ESC_RA1913AA	1193	761
ESC_RA1912AA	73	737
ESC_RA1911AA	73	737
ESC_RA1880AA	155	175
ESC_RA1879AA	69	387
ESC_RA1878AA	131	653
ESC_RA1877AA	73	737
ESC_RA1876AA	11113	923
ESC_RA1875AA	131	653
ESC_RA1874AA	73	737
ESC_RA1873AA	73	737
ESC_RA1872AA	405	923
ESC_RA1871AA	162	924
ESC_RA1870AA	1057	761
ESC_RA1869AA	131	653
ESC_RA1868AA	73	737
ESC_RA1867AA	144	740
ESC_RA1866AA	12	626
ESC_RA1865AA	73	737
ESC_RA1864AA	88	160
ESC_RA1863AA	73	737
ESC_RA1862AA	349	670
ESC_RA1861AA	404	761
ESC_RA1860AA	12	626
ESC_RA1859AA	144	740
ESC_RA1858AA	635	141
ESC_RA1857AA	635	141
ESC_RA1856AA	635	141
ESC_RA1855AA	635	141
ESC_RA1854AA	11112	141
ESC_RA1853AA	635	141
ESC_RA1852AA	401	142
ESC_RA1851AA	635	141
ESC_RA1850AA	2795	355
ESC_RA1849AA	1485	408
ESC_RA1848AA	635	141
ESC_RA1847AA	69	387
ESC_RA1846AA	635	141
ESC_RA1845AA	635	141
ESC_RA1844AA	399	198
ESC_RA1843AA	635	141
ESC_RA1842AA	401	142
ESC_RA1841AA	635	141
ESC_RA1840AA	131	653
ESC_RA1839AA	401	142
ESC_RA1838AA	635	141
ESC_RA1837AA	635	141

ESC_RA1836AA	635	141
ESC_RA1835AA	401	142
ESC_RA1834AA	635	141
ESC_RA1833AA	409	504
ESC_RA1832AA	401	142
ESC_RA1831AA	131	653
ESC_RA1830AA	635	141
ESC_RA1829AA	635	141
ESC_RA1828AA	635	141
ESC_RA1827AA	73	737
ESC_RA1826AA	635	141
ESC_RA1825AA	399	198
ESC_RA1824AA	472	601
ESC_RA1823AA	635	141
ESC_RA1822AA	635	141
ESC_RA1821AA	635	141
ESC_RA1819AA	11063	141
ESC_RA1818AA	635	141
ESC_RA1817AA	635	141
ESC_RA1816AA	635	141
ESC_RA1815AA	472	601
ESC_RA1814AA	472	601
ESC_RA1813AA	472	601
ESC_RA1812AA	472	601
ESC_RA1811AA	635	141
ESC_RA1810AA	635	141
ESC_RA1809AA	472	601
ESC_RA1808AA	635	141
ESC_RA1807AA	401	142
ESC_RA1806AA	635	141
ESC_RA1805AA	635	141
ESC_RA1804AA	635	141
ESC_RA1803AA	635	141
ESC_RA1802AA	635	141
ESC_RA1801AA	635	141
ESC_RA1800AA	472	601
ESC_RA1799AA	635	141
ESC_RA1798AA	635	141
ESC_RA1797AA	401	142
ESC_RA1796AA	635	141
ESC_RA1795AA	635	141
ESC_RA1794AA	635	141
ESC_RA1793AA	635	141
ESC_RA1792AA	635	141
ESC_RA1791AA	635	141
ESC_RA1790AA	635	141
ESC_RA1789AA	635	141
ESC_RA1788AA	635	141
ESC_RA1787AA	59	217
ESC_RA1786AA	635	141

ESC_RA1785AA	635	141
ESC_RA1784AA	635	141
ESC_RA1783AA	635	141
ESC_RA1782AA	399	198
ESC_RA1781AA	635	141
ESC_RA1780AA	401	142
ESC_RA1779AA	399	198
ESC_RA1778AA	472	601
ESC_RA1777AA	635	141
ESC_RA1776AA	635	141
ESC_RA1775AA	635	141
ESC_RA1774AA	635	141
ESC_RA1773AA	11063	141
ESC_RA1772AA	401	142
ESC_RA1771AA	635	141
ESC_RA1770AA	1722	599
ESC_RA1769AA	472	601
ESC_RA1768AA	401	142
ESC_RA1767AA	399	198
ESC_RA1766AA	635	141
ESC_RA1765AA	6188	257
ESC_RA1764AA	635	141
ESC_RA1763AA	635	141
ESC_RA1762AA	635	141
ESC_RA1761AA	635	141
ESC_RA1760AA	635	141
ESC_RA1759AA	635	141
ESC_RA1758AA	401	142
ESC_RA1757AA	635	141
ESC_RA1756AA	127	738
ESC_RA1755AA	635	141
ESC_RA1754AA	635	141
ESC_RA1753AA	635	141
ESC_RA1752AA	472	601
ESC_RA1751AA	401	142
ESC_RA1750AA	635	141
ESC_RA1749AA	635	141
ESC_RA1748AA	401	142
ESC_RA1747AA	635	141
ESC_RA1746AA	399	198
ESC_RA1745AA	635	141
ESC_RA1744AA	401	142
ESC_RA1743AA	401	142
ESC_RA1742AA	472	601
ESC_RA1741AA	550	756
ESC_RA1740AA	73	737
ESC_RA1739AA	635	141
ESC_RA1738AA	635	141
ESC_RA1737AA	409	504
ESC_RA1736AA	69	387

ESC_RA1735AA	6188	257
ESC_RA1734AA	635	141
ESC_RA1733AA	635	141
ESC_RA1732AA	635	141
ESC_RA1731AA	472	601
ESC_RA1730AA	635	141
ESC_RA1729AA	11061	142
ESC_RA1728AA	635	141
ESC_RA1727AA	80	731
ESC_RA1726AA	401	142
ESC_RA1725AA	635	141
ESC_RA1724AA	635	141
ESC_RA1723AA	399	198
ESC_RA1722AA	635	141
ESC_RA1721AA	176	601
ESC_RA1720AA	401	142
ESC_RA1719AA	635	141
ESC_RA1718AA	635	141
ESC_RA1717AA	11064	737
ESC_RA1716AA	635	141
ESC_RA1715AA	635	141
ESC_RA1714AA	635	141
ESC_RA1713AA	399	198
ESC_RA1712AA	11063	141
ESC_RA1711AA	401	142
ESC_RA1710AA	635	141
ESC_RA1709AA	635	141
ESC_RA1708AA	635	141
ESC_RA1707AA	399	198
ESC_RA1706AA	401	142
ESC_RA1705AA	95	738
ESC_RA1704AA	401	142
ESC_RA1703AA	635	141
ESC_RA1702AA	635	141
ESC_RA1701AA	635	141
ESC_RA1700AA	635	141
ESC_RA1699AA	401	142
ESC_RA1698AA	472	601
ESC_RA1697AA	635	141
ESC_RA1696AA	401	142
ESC_RA1695AA	472	601
ESC_RA1694AA	635	141
ESC_RA1693AA	401	142
ESC_RA1692AA	635	141
ESC_RA1691AA	401	142
ESC_RA1690AA	2795	355
ESC_RA1689AA	11062	142
ESC_RA1688AA	399	198
ESC_RA1687AA	131	653
ESC_RA1686AA	635	141

ESC_RA1685AA	472	601
ESC_RA1684AA	635	141
ESC_RA1683AA	11061	142
ESC_RA1682AA	635	141
ESC_RA1681AA	409	504
ESC_RA1680AA	472	601
ESC_RA1679AA	176	601
ESC_RA1678AA	69	387
ESC_RA1677AA	11061	142
ESC_RA1676AA	131	653
ESC_RA1675AA	635	141
ESC_RA1674AA	472	601
ESC_RA1673AA	635	141
ESC_RA1672AA	401	142
ESC_RA1671AA	472	601
ESC_RA1670AA	635	141
ESC_RA1669AA	635	141
ESC_RA1668AA	635	141
ESC_RA1667AA	635	141
ESC_RA1666AA	635	141
ESC_RA1665AA	127	738
ESC_RA1664AA	6188	257
ESC_RA1663AA	635	141
ESC_RA1662AA	399	198
ESC_RA1661AA	635	141
ESC_RA1660AA	635	141
ESC_RA1659AA	10	512
ESC_RA1658AA	1673	257
ESC_RA1657AA	10	512
ESC_RA1656AA	1850	269
ESC_RA1655AA	48	76
ESC_RA1654AA	394	400
ESC_RA1653AA	131	653
ESC_RA1652AA	88	160
ESC_RA1651AA	398	445
ESC_RA1650AA	155	175
ESC_RA1649AA	73	737
ESC_RA1648AA	7187	76
ESC_RA1647AA	117	592
ESC_RA1628AA	1193	761
ESC_RA1627AA	295	160
ESC_RA1626AA	1193	761
ESC_RA1611AA	11024	256
ESC_RA1610AA	11023	512
ESC_RA1608AA	100	504
ESC_RA1607AA	86	935
ESC_RA1606AA	410	156
ESC_RA1605AA	4247	936
ESC_RA1604AA	10	512
ESC_RA1603AA	90	156

ESC_RA1602AA	10	512
ESC_RA1601AA	11014	50
ESC_RA1600AA	11013	198
ESC_RA1599AA	131	653
ESC_RA1598AA	131	653
ESC_RA1597AA	131	653
ESC_RA1596AA	131	653
ESC_RA1595AA	131	653
ESC_RA1594AA	152	257
ESC_RA1593AA	245	261
ESC_RA1592AA	245	261
ESC_RA1591AA	245	261
ESC_RA1590AA	245	261
ESC_RA1589AA	152	257
ESC_RA1588AA	152	257
ESC_RA1587AA	245	261
ESC_RA1586AA	245	261
ESC_RA1585AA	152	257
ESC_RA1581AA	717	256
ESC_RA1580AA	243	197
ESC_RA1534AA	12	626
ESC_RA1533AA	5415	924
ESC_RA1532AA	372	424
ESC_RA1531AA	155	175
ESC_RA1530AA	131	653
ESC_RA1529AA	23	156
ESC_RA1528AA	11022	512
ESC_RA1527AA	196	257
ESC_RA1526AA	11012	76
ESC_RA1525AA	162	924
ESC_RA1524AA	602	257
ESC_RA1523AA	6745	885
ESC_RA1522AA	1147	198
ESC_RA1521AA	58	198
ESC_RA1520AA	6957	221
ESC_RA1519AA	8131	900
ESC_RA1518AA	25	234
ESC_RA1517AA	355	764
ESC_RA1516AA	355	764
ESC_RA1515AA	349	670
ESC_RA1514AA	349	670
ESC_RA1513AA	355	764
ESC_RA1512AA	355	764
ESC_RA1511AA	355	764
ESC_RA1510AA	117	592
ESC_RA1509AA	155	175
ESC_RA1508AA	349	670
ESC_RA1507AA	349	670
ESC_RA1506AA	602	257
ESC_RA1505AA	4994	584

ESC_RA1504AA	349	670
ESC_RA1503AA	349	670
ESC_RA1502AA	355	764
ESC_RA1501AA	355	764
ESC_RA1500AA	191	198
ESC_RA1499AA	355	764
ESC_RA1498AA	131	653
ESC_RA1497AA	355	764
ESC_RA1496AA	1286	474
ESC_RA1495AA	10	512
ESC_RA1494AA	4537	76
ESC_RA1493AA	355	764
ESC_RA1492AA	6726	493
ESC_RA1491AA	355	764
ESC_RA1490AA	756	257
ESC_RA1489AA	1251	71
ESC_RA1488AA	191	198
ESC_RA1487AA	69	387
ESC_RA1486AA	48	76
ESC_RA1485AA	1286	474
ESC_RA1484AA	349	670
ESC_RA1483AA	10	512
ESC_RA1482AA	5375	639
ESC_RA1481AA	2040	198
ESC_RA1480AA	5825	924
ESC_RA1479AA	1594	71
ESC_RA1478AA	115	669
ESC_RA1477AA	131	653
ESC_RA1476AA	5825	924
ESC_RA1475AA	5825	924
ESC_RA1474AA	189	504
ESC_RA1473AA	1056	257
ESC_RA1472AA	2178	924
ESC_RA1471AA	5825	924
ESC_RA1470AA	5825	924
ESC_RA1469AA	5375	639
ESC_RA1468AA	9427	506
ESC_RA1467AA	9424	512
ESC_RA1466AA	10	512
ESC_RA1465AA	131	653
ESC_RA1464AA	5825	924
ESC_RA1463AA	10	512
ESC_RA1462AA	10	512
ESC_RA1461AA	5825	924
ESC_RA1460AA	10	512
ESC_RA1459AA	10	512
ESC_RA1458AA	1266	210
ESC_RA1457AA	442	257
ESC_RA1456AA	3107	512
ESC_RA1455AA	10	512

ESC_RA1454AA	10	512
ESC_RA1453AA	355	764
ESC_RA1452AA	752	512
ESC_RA1451AA	10	512
ESC_RA1450AA	10	512
ESC_RA1449AA	602	257
ESC_RA1448AA	5825	924
ESC_RA1447AA	10	512
ESC_RA1446AA	10	512
ESC_RA1445AA	328	924
ESC_RA1444AA	10	512
ESC_RA1443AA	752	512
ESC_RA1442AA	1730	234
ESC_RA1441AA	355	764
ESC_RA1440AA	10	512
ESC_RA1439AA	131	653
ESC_RA1438AA	5825	924
ESC_RA1437AA	648	359
ESC_RA1436AA	1266	210
ESC_RA1435AA	10	512
ESC_RA1434AA	5825	924
ESC_RA1433AA	10	512
ESC_RA1432AA	1266	210
ESC_RA1431AA	189	504
ESC_RA1430AA	10	512
ESC_RA1429AA	10	512
ESC_RA1428AA	1286	474
ESC_RA1427AA	355	764
ESC_RA1426AA	10	512
ESC_RA1425AA	9425	71
ESC_RA1424AA	117	592
ESC_RA1423AA	9426	75
ESC_RA1421AA	155	175
ESC_RA1420AA	154	257
ESC_RA1419AA	3549	924
ESC_RA1418AA	1642	198
ESC_RA1417AA	1829	964
ESC_RA1416AA	1850	269
ESC_RA1415AA	212	198
ESC_RA1414AA	3747	373
ESC_RA1413AA	11016	512
ESC_RA1412AA	11016	512
ESC_RA1411AA	11016	512
ESC_RA1410AA	11015	737
ESC_RA1409AA	1128	924
ESC_RA1408AA	536	141
ESC_RA1407AA	2139	789
ESC_RA1406AA	130	387
ESC_RA1405AA	44	512
ESC_RA1404AA	3268	669

ESC_RA1403AA	2852	257
ESC_RA1402AA	48	76
ESC_RA1401AA	38	669
ESC_RA1400AA	758	447
ESC_RA1399AA	2067	257
ESC_RA1398AA	165	504
ESC_RA1397AA	4040	696
ESC_RA1396AA	48	76
ESC_RA1395AA	361	512
ESC_RA1394AA	11017	462
ESC_RA1393AA	58	198
ESC_RA1392AA	58	198
ESC_RA1391AA	414	387
ESC_RA1390AA	405	923
ESC_RA1389AA	3052	669
ESC_RA1388AA	10	512
ESC_RA1387AA	44	512
ESC_RA1386AA	5947	447
ESC_RA1385AA	11020	682
ESC_RA1384AA	162	924
ESC_RA1383AA	38	669
ESC_RA1382AA	38	669
ESC_RA1381AA	131	653
ESC_RA1380AA	4040	696
ESC_RA1379AA	38	669
ESC_RA1378AA	226	512
ESC_RA1377AA	361	512
ESC_RA1376AA	2083	257
ESC_RA1375AA	457	595
ESC_RA1374AA	410	156
ESC_RA1373AA	224	198
ESC_RA1372AA	224	198
ESC_RA1371AA	617	511
ESC_RA1370AA	10	512
ESC_RA1369AA	2705	504
ESC_RA1368AA	226	512
ESC_RA1367AA	6778	198
ESC_RA1366AA	3075	701
ESC_RA1365AA	515	1004
ESC_RA1364AA	131	653
ESC_RA1363AA	10	512
ESC_RA1362AA	354	15
ESC_RA1361AA	354	15
ESC_RA1360AA	746	789
ESC_RA1359AA	973	968
ESC_RA1358AA	48	76
ESC_RA1357AA	131	653
ESC_RA1356AA	38	669
ESC_RA1355AA	11019	592
ESC_RA1354AA	11018	460

ESC_RA1353AA	68	386
ESC_RA1352AA	1788	785
ESC_RA1351AA	1788	785
ESC_RA1350AA	648	359
ESC_RA1349AA	2914	670
ESC_RA1348AA	10	512
ESC_RA1347AA	12	626
ESC_RA1346AA	12	626
ESC_RA1345AA	3268	669
ESC_RA1344AA	5846	512
ESC_RA1343AA	38	669
ESC_RA1342AA	38	669
ESC_RA1341AA	7160	647
ESC_RA1340AA	189	504
ESC_RA1339AA	5869	257
ESC_RA1338AA	141	644
ESC_RA1337AA	2689	519
ESC_RA1336AA	453	935
ESC_RA1335AA	315	669
ESC_RA1334AA	5643	462
ESC_RA1333AA	117	592
ESC_RA1332AA	504	738
ESC_RA1331AA	40	198
ESC_RA1330AA	40	198
ESC_RA1329AA	40	198
ESC_RA1326AA	31	387
ESC_RA1324AA	40	198
ESC_RA1323AA	40	198
ESC_RA1322AA	31	387
ESC_RA1321AA	31	387
ESC_RA1318AA	40	198
ESC_RA1317AA	40	198
ESC_RA1316AA	40	198
ESC_RA1315AA	200	198
ESC_RA1314AA	31	387
ESC_RA1313AA	10	512
ESC_RA1312AA	31	387
ESC_RA1311AA	10	512
ESC_RA1310AA	31	387
ESC_RA1309AA	10	512
ESC_RA1308AA	34	504
ESC_RA1307AA	31	387
ESC_RA1306AA	31	387
ESC_RA1305AA	31	387
ESC_RA1304AA	31	387
ESC_RA1303AA	10	512
ESC_RA1302AA	34	504
ESC_RA1301AA	31	387
ESC_RA1300AA	31	387
ESC_RA1299AA	31	387

ESC_RA1298AA	515	1004
ESC_RA1297AA	40	198
ESC_RA1296AA	1380	914
ESC_RA1295AA	31	387
ESC_RA1294AA	31	387
ESC_RA1293AA	40	198
ESC_RA1292AA	34	504
ESC_RA1291AA	31	387
ESC_RA1290AA	31	387
ESC_RA1289AA	10	512
ESC_RA1288AA	34	504
ESC_RA1287AA	10	512
ESC_RA1286AA	40	198
ESC_RA1285AA	31	387
ESC_RA1284AA	31	387
ESC_RA1283AA	31	387
ESC_RA1282AA	117	592
ESC_RA1281AA	140	738
ESC_RA1280AA	117	592
ESC_RA1279AA	7080	504
ESC_RA1278AA	1618	736
ESC_RA1277AA	359	696
ESC_RA1276AA	43	512
ESC_RA1275AA	746	789
ESC_RA1274AA	117	592
ESC_RA1273AA	9862	156
ESC_RA1272AA	9862	156
ESC_RA1271AA	9862	156
ESC_RA1270AA	9862	156
ESC_RA1269AA	7080	504
ESC_RA1268AA	48	76
ESC_RA1267AA	1196	257
ESC_RA1266AA	155	175
ESC_RA1265AA	43	512
ESC_RA1264AA	1196	257
ESC_RA1263AA	117	592
ESC_RA1262AA	295	160
ESC_RA1261AA	117	592
ESC_RA1260AA	10	512
ESC_RA1258AA	1485	408
ESC_RA1257AA	295	160
ESC_RA1256AA	295	160
ESC_RA1255AA	2040	198
ESC_RA1254AA	997	915
ESC_RA1253AA	295	160
ESC_RA1252AA	295	160
ESC_RA1250AA	11	936
ESC_RA1249AA	32	270
ESC_RA1248AA	11	936
ESC_RA1247AA	4419	463

ESC_RA1246AA	21	156
ESC_RA1245AA	52	71
ESC_RA1244AA	306	286
ESC_RA1243AA	306	286
ESC_RA1242AA	795	924
ESC_RA1241AA	795	924
ESC_RA1238AA	306	286
ESC_RA1237AA	306	286
ESC_RA1236AA	306	286
ESC_RA1235AA	306	286
ESC_RA1233AA	306	286
ESC_RA1232AA	11	936
ESC_RA1231AA	306	286
ESC_RA1230AA	306	286
ESC_RA1227AA	306	286
ESC_RA1226AA	306	286
ESC_RA1225AA	306	286
ESC_RA1224AA	1485	408
ESC_RA1223AA	1485	408
ESC_RA1221AA	306	286
ESC_RA1217AA	20	198
ESC_RA1216AA	5614	257
ESC_RA1215AA	306	286
ESC_RA1214AA	306	286
ESC_RA1213AA	659	286
ESC_RA1212AA	306	286
ESC_RA1211AA	306	286
ESC_RA1210AA	306	286
ESC_RA1209AA	617	511
ESC_RA1208AA	131	653
ESC_RA1207AA	297	234
ESC_RA1206AA	131	653
ESC_RA1205AA	224	198
ESC_RA1204AA	1193	761
ESC_RA1203AA	540	286
ESC_RA1202AA	127	738
ESC_RA1201AA	405	923
ESC_RA1200AA	410	156
ESC_RA1199AA	405	923
ESC_RA1198AA	73	737
ESC_RA1197AA	1284	604
ESC_RA1196AA	224	198
ESC_RA1195AA	11021	512
ESC_RA1194AA	540	286
ESC_RA1193AA	17	191
ESC_RA1192AA	745	234
ESC_RA1191AA	1029	291
ESC_RA1190AA	745	234
ESC_RA1189AA	58	198
ESC_RA1188AA	189	504

ESC_RA1187AA	744	512
ESC_RA1186AA	10	512
ESC_RA1185AA	302	850
ESC_RA1184AA	10	512
ESC_RA1183AA	744	512
ESC_RA1182AA	10	512
ESC_RA1181AA	10	512
ESC_RA1180AA	58	198
ESC_RA1179AA	877	935
ESC_RA1178AA	744	512
ESC_RA1177AA	744	512
ESC_RA1176AA	10	512
ESC_RA1174AA	10591	512
ESC_RA1173AA	744	512
ESC_RA1172AA	93	71
ESC_RA1171AA	744	512
ESC_RA1170AA	56	198
ESC_RA1169AA	744	512
ESC_RA1168AA	10	512
ESC_RA1167AA	58	198
ESC_RA1165AA	88	160
ESC_RA1164AA	342	286
ESC_RA1163AA	744	512
ESC_RA1162AA	131	653
ESC_RA1161AA	10	512
ESC_RA1160AA	744	512
ESC_RA1159AA	342	286
ESC_RA1158AA	5579	175
ESC_RA1157AA	58	198
ESC_RA1156AA	88	160
ESC_RA1155AA	10	512
ESC_RA1154AA	88	160
ESC_RA1153AA	10	512
ESC_RA1151AA	88	160
ESC_RA1150AA	10	512
ESC_RA1149AA	2496	789
ESC_RA1148AA	88	160
ESC_RA1147AA	744	512
ESC_RA1146AA	342	286
ESC_RA1145AA	744	512
ESC_RA1144AA	34	504
ESC_RA1143AA	58	198
ESC_RA1142AA	88	160
ESC_RA1141AA	152	257
ESC_RA1140AA	152	257
ESC_RA1139AA	152	257
ESC_RA1138AA	245	261
ESC_RA1137AA	245	261
ESC_RA1136AA	152	257
ESC_RA1135AA	152	257

ESC_RA1134AA	11011	373
ESC_RA1133AA	11011	373
ESC_RA1132AA	1953	373
ESC_RA1131AA	372	424
ESC_RA1130AA	372	424
ESC_RA1129AA	372	424
ESC_RA1128AA	372	424
ESC_RA1127AA	372	424
ESC_RA1126AA	372	424
ESC_RA1125AA	372	424
ESC_RA1124AA	372	424
ESC_RA1123AA	372	424
ESC_RA1122AA	372	424
ESC_RA1121AA	372	424
ESC_RA1120AA	372	424
ESC_RA1119AA	372	424
ESC_RA1118AA	372	424
ESC_RA1117AA	372	424
ESC_RA1116AA	372	424
ESC_RA1115AA	372	424
ESC_RA1114AA	372	424
ESC_RA1113AA	372	424
ESC_RA1112AA	372	424
ESC_RA1111AA	372	424
ESC_RA1110AA	372	424
ESC_RA1109AA	372	424
ESC_RA1108AA	11010	512
ESC_RA1107AA	58	198
ESC_RA1106AA	11	936
ESC_RA1104AA	11009	632
ESC_RA1102AA	21	156
ESC_RA1101AA	21	156
ESC_RA1100AA	21	156
ESC_RA1099AA	152	257
ESC_RA1098AA	148	191
ESC_RA1097AA	152	257
ESC_RA1096AA	245	261
ESC_RA1095AA	630	261
ESC_RA1094AA	245	261
ESC_RA1093AA	152	257
ESC_RA1092AA	245	261
ESC_RA1090AA	2064	261
ESC_RA1089AA	152	257
ESC_RA1088AA	288	102
ESC_RA1087AA	25	234
ESC_RA1086AA	6957	221
ESC_RA1085AA	21	156
ESC_RA1084AA	11	936
ESC_RA1083AA	8131	900
ESC_RA1082AA	11	936

ESC_RA1081AA	216	512
ESC_RA1080AA	216	512
ESC_RA1079AA	10	512
ESC_RA1078AA	971	198
ESC_RA1077AA	75	257
ESC_RA1076AA	675	234
ESC_RA1075AA	11008	108
ESC_RA1073AA	11008	108
ESC_RA1072AA	11008	108
ESC_RA1071AA	25	234
ESC_RA1070AA	40	198
ESC_RA1069AA	971	198
ESC_RA1068AA	40	198
ESC_RA1067AA	10	512
ESC_RA1066AA	447	198
ESC_RA1065AA	11007	924
ESC_RA1064AA	675	234
ESC_RA1063AA	675	234
ESC_RA1062AA	447	198
ESC_RA1061AA	447	198
ESC_RA1059AA	155	175
ESC_RA1058AA	40	198
ESC_RA1057AA	10	512
ESC_RA1056AA	8607	257
ESC_RA1055AA	154	257
ESC_RA1054AA	43	512
ESC_RA1053AA	10	512
ESC_RA1052AA	10	512
ESC_RA1051AA	10	512
ESC_RA1050AA	10	512
ESC_RA1049AA	58	198
ESC_RA1048AA	8607	257
ESC_RA1047AA	10	512
ESC_RA1046AA	101	696
ESC_RA1045AA	43	512
ESC_RA1044AA	10	512
ESC_RA1042AA	10	512
ESC_RA1041AA	3249	156
ESC_RA1039AA	3249	156
ESC_RA1038AA	3249	156
ESC_RA1037AA	155	175
ESC_RA1035AA	692	234
ESC_RA1034AA	40	198
ESC_RA1033AA	1423	198
ESC_RA1032AA	443	257
ESC_RA1031AA	58	198
ESC_RA1030AA	88	160
ESC_RA1029AA	3249	156
ESC_RA1028AA	154	257
ESC_RA1027AA	447	198

ESC_RA1026AA	2283	817
ESC_RA1025AA	10	512
ESC_RA1024AA	675	234
ESC_RA1019AA	11006	991
ESC_RA1018AA	4702	626
ESC_RA1016AA	11002	669
ESC_RA1015AA	11000	257
ESC_RA1013AA	90	156
ESC_RA1012AA	90	156
ESC_RA1011AA	90	156
ESC_RA1010AA	90	156
ESC_RA1009AA	42	257
ESC_RA1008AA	90	156
ESC_RA1007AA	6354	447
ESC_RA1006AA	42	257
ESC_RA1005AA	90	156
ESC_RA1004AA	90	156
ESC_RA1003AA	100	504
ESC_RA1002AA	90	156
ESC_RA1001AA	100	504
ESC_RA1000AA	10999	198
ESC_RA0998AA	216	512
ESC_RA0997AA	216	512
ESC_RA0996AA	216	512
ESC_RA0995AA	216	512
ESC_RA0994AA	10643	665
ESC_RA0993AA	8881	362
ESC_RA0992AA	10998	670
ESC_RA0991AA	10997	473
ESC_RA0990AA	10994	789
ESC_RA0989AA	8881	362
ESC_RA0987AA	8881	362
ESC_RA0986AA	4981	473
ESC_RA0985AA	10997	473
ESC_RA0984AA	394	400
ESC_RA0983AA	10996	76
ESC_RA0982AA	10995	257
ESC_RA0981AA	10	512
ESC_RA0980AA	10994	789
ESC_RA0979AA	8881	362
ESC_RA0978AA	8881	362
ESC_RA0977AA	10993	738
ESC_RA0976AA	10992	76
ESC_RA0975AA	216	512
ESC_RA0974AA	9269	198
ESC_RA0973AA	33	198
ESC_RA0972AA	11	936
ESC_RA0971AA	33	198
ESC_RA0967AA	6603	168
ESC_RA0966AA	2602	257

ESC_RA0965AA	10817	235
ESC_RA0964AA	919	421
ESC_RA0963AA	10	512
ESC_RA0962AA	1955	900
ESC_RA0961AA	1276	241
ESC_RA0960AA	10991	76
ESC_RA0959AA	10990	76
ESC_RA0958AA	11	936
ESC_RA0957AA	442	257
ESC_RA0956AA	10988	283
ESC_RA0955AA	10989	204
ESC_RA0954AA	10987	204
ESC_RA0953AA	10987	204
ESC_RA0952AA	131	653
ESC_RA0951AA	131	653
ESC_RA0950AA	449	387
ESC_RA0948AA	224	198
ESC_RA0947AA	350	50
ESC_RA0946AA	131	653
ESC_RA0945AA	648	359
ESC_RA0944AA	58	198
ESC_RA0943AA	4553	408
ESC_RA0942AA	21	156
ESC_RA0941AA	21	156
ESC_RA0940AA	21	156
ESC_RA0939AA	131	653
ESC_RA0938AA	10986	630
ESC_RA0937AA	676	737
ESC_RA0936AA	131	653
ESC_RA0935AA	131	653
ESC_RA0934AA	131	653
ESC_RA0933AA	21	156
ESC_RA0932AA	1236	380
ESC_RA0931AA	10985	653
ESC_RA0930AA	1236	380
ESC_RA0929AA	83	740
ESC_RA0928AA	131	653
ESC_RA0927AA	5640	653
ESC_RA0926AA	131	653
ESC_RA0925AA	10984	654
ESC_RA0924AA	5640	653
ESC_RA0923AA	21	156
ESC_RA0922AA	29	156
ESC_RA0921AA	21	156
ESC_RA0920AA	21	156
ESC_RA0919AA	57	50
ESC_RA0918AA	369	156
ESC_RA0917AA	602	257
ESC_RA0916AA	5881	555
ESC_RA0915AA	134	738

ESC_RA0914AA	131	653
ESC_RA0913AA	351	268
ESC_RA0912AA	162	924
ESC_RA0911AA	428	654
ESC_RA0910AA	6069	344
ESC_RA0909AA	1594	71
ESC_RA0908AA	1429	198
ESC_RA0907AA	10	512
ESC_RA0906AA	21	156
ESC_RA0905AA	10	512
ESC_RA0904AA	117	592
ESC_RA0903AA	6069	344
ESC_RA0902AA	1775	51
ESC_RA0901AA	6069	344
ESC_RA0900AA	117	592
ESC_RA0899AA	937	198
ESC_RA0898AA	93	71
ESC_RA0897AA	6069	344
ESC_RA0896AA	6069	344
ESC_RA0895AA	1611	198
ESC_RA0894AA	117	592
ESC_RA0893AA	6069	344
ESC_RA0892AA	117	592
ESC_RA0891AA	6069	344
ESC_RA0890AA	399	198
ESC_RA0889AA	6069	344
ESC_RA0888AA	6069	344
ESC_RA0887AA	635	141
ESC_RA0886AA	6069	344
ESC_RA0885AA	6069	344
ESC_RA0884AA	349	670
ESC_RA0883AA	134	738
ESC_RA0882AA	6069	344
ESC_RA0881AA	6069	344
ESC_RA0880AA	6069	344
ESC_RA0879AA	131	653
ESC_RA0878AA	131	653
ESC_RA0877AA	2040	198
ESC_RA0876AA	6069	344
ESC_RA0875AA	6069	344
ESC_RA0874AA	58	198
ESC_RA0873AA	6069	344
ESC_RA0872AA	602	257
ESC_RA0871AA	602	257
ESC_RA0870AA	2171	164
ESC_RA0869AA	212	198
ESC_RA0868AA	117	592
ESC_RA0867AA	95	738
ESC_RA0866AA	6069	344
ESC_RA0865AA	58	198

ESC_RA0864AA	602	257
ESC_RA0863AA	369	156
ESC_RA0862AA	297	234
ESC_RA0861AA	5940	555
ESC_RA0860AA	428	654
ESC_RA0859AA	68	386
ESC_RA0858AA	602	257
ESC_RA0857AA	6707	445
ESC_RA0856AA	602	257
ESC_RA0855AA	5940	555
ESC_RA0854AA	117	592
ESC_RA0853AA	117	592
ESC_RA0852AA	117	592
ESC_RA0851AA	501	900
ESC_RA0850AA	155	175
ESC_RA0849AA	117	592
ESC_RA0848AA	602	257
ESC_RA0847AA	1914	956
ESC_RA0846AA	6069	344
ESC_RA0845AA	117	592
ESC_RA0844AA	101	696
ESC_RA0843AA	10	512
ESC_RA0842AA	117	592
ESC_RA0841AA	117	592
ESC_RA0840AA	162	924
ESC_RA0839AA	8249	738
ESC_RA0838AA	4162	180
ESC_RA0837AA	1914	956
ESC_RA0836AA	2040	198
ESC_RA0835AA	501	900
ESC_RA0834AA	117	592
ESC_RA0833AA	69	387
ESC_RA0832AA	117	592
ESC_RA0831AA	101	696
ESC_RA0830AA	2040	198
ESC_RA0829AA	2040	198
ESC_RA0828AA	602	257
ESC_RA0827AA	57	50
ESC_RA0826AA	93	71
ESC_RA0825AA	398	445
ESC_RA0824AA	5881	555
ESC_RA0823AA	752	512
ESC_RA0822AA	162	924
ESC_RA0821AA	5794	50
ESC_RA0820AA	117	592
ESC_RA0819AA	2171	164
ESC_RA0818AA	23	156
ESC_RA0817AA	6069	344
ESC_RA0816AA	117	592
ESC_RA0815AA	501	900

ESC_RA0814AA	6069	344
ESC_RA0813AA	398	445
ESC_RA0812AA	6096	257
ESC_RA0811AA	1112	504
ESC_RA0810AA	134	738
ESC_RA0809AA	155	175
ESC_RA0808AA	57	50
ESC_RA0807AA	13	257
ESC_RA0806AA	117	592
ESC_RA0805AA	117	592
ESC_RA0804AA	399	198
ESC_RA0803AA	6069	344
ESC_RA0802AA	57	50
ESC_RA0801AA	117	592
ESC_RA0800AA	117	592
ESC_RA0799AA	117	592
ESC_RA0798AA	117	592
ESC_RA0797AA	117	592
ESC_RA0796AA	117	592
ESC_RA0794AA	1286	474
ESC_RA0793AA	117	592
ESC_RA0792AA	131	653
ESC_RA0791AA	6069	344
ESC_RA0790AA	131	653
ESC_RA0789AA	131	653
ESC_RA0788AA	131	653
ESC_RA0787AA	131	653
ESC_RA0786AA	131	653
ESC_RA0785AA	131	653
ESC_RA0784AA	131	653
ESC_RA0783AA	131	653
ESC_RA0782AA	131	653
ESC_RA0781AA	131	653
ESC_RA0780AA	131	653
ESC_RA0779AA	131	653
ESC_RA0778AA	131	653
ESC_RA0777AA	131	653
ESC_RA0776AA	131	653
ESC_RA0775AA	131	653
ESC_RA0774AA	21	156
ESC_RA0773AA	131	653
ESC_RA0772AA	131	653
ESC_RA0771AA	131	653
ESC_RA0770AA	131	653
ESC_RA0769AA	131	653
ESC_RA0768AA	676	737
ESC_RA0767AA	131	653
ESC_RA0766AA	131	653
ESC_RA0765AA	1236	380
ESC_RA0764AA	345	175

ESC_RA0763AA	2973	234
ESC_RA0762AA	744	512
ESC_RA0761AA	2973	234
ESC_RA0760AA	95	738
ESC_RA0759AA	1302	817
ESC_RA0758AA	3638	737
ESC_RA0757AA	88	160
ESC_RA0715AA	145	256
ESC_RA0714AA	245	261
ESC_RA0713AA	131	653
ESC_RA0712AA	131	653
ESC_RA0711AA	405	923
ESC_RA0710AA	131	653
ESC_RA0709AA	131	653
ESC_RA0708AA	131	653
ESC_RA0707AA	131	653
ESC_RA0706AA	131	653
ESC_RA0705AA	131	653
ESC_RA0704AA	131	653
ESC_RA0703AA	131	653
ESC_RA0702AA	131	653
ESC_RA0701AA	131	653
ESC_RA0700AA	131	653
ESC_RA0699AA	131	653
ESC_RA0698AA	131	653
ESC_RA0697AA	131	653
ESC_RA0696AA	131	653
ESC_RA0695AA	131	653
ESC_RA0694AA	131	653
ESC_RA0693AA	131	653
ESC_RA0692AA	131	653
ESC_RA0691AA	131	653
ESC_RA0690AA	131	653
ESC_RA0689AA	131	653
ESC_RA0688AA	131	653
ESC_RA0684AA	131	653
ESC_RA0683AA	131	653
ESC_RA0682AA	131	653
ESC_RA0681AA	131	653
ESC_RA0680AA	405	923
ESC_RA0679AA	131	653
ESC_RA0678AA	131	653
ESC_RA0677AA	131	653
ESC_RA0676AA	131	653
ESC_RA0675AA	1172	234
ESC_RA0674AA	152	257
ESC_RA0673AA	152	257
ESC_RA0672AA	152	257
ESC_RA0671AA	152	257
ESC_RA0670AA	152	257

ESC_RA0669AA	152	257
ESC_RA0668AA	152	257
ESC_RA0667AA	152	257
ESC_RA0666AA	152	257
ESC_RA0665AA	152	257
ESC_RA0664AA	152	257
ESC_RA0663AA	152	257
ESC_RA0662AA	152	257
ESC_RA0661AA	152	257
ESC_RA0660AA	152	257
ESC_RA0659AA	152	257
ESC_RA0658AA	152	257
ESC_RA0657AA	152	257
ESC_RA0656AA	152	257
ESC_RA0655AA	152	257
ESC_RA0654AA	152	257
ESC_RA0653AA	152	257
ESC_RA0652AA	245	261
ESC_RA0651AA	245	261
ESC_RA0650AA	517	924
ESC_RA0649AA	152	257
ESC_RA0648AA	245	261
ESC_RA0647AA	10942	175
ESC_RA0646AA	10941	842
ESC_RA0645AA	10940	669
ESC_RA0644AA	1844	198
ESC_RA0643AA	152	257
ESC_RA0642AA	152	257
ESC_RA0641AA	131	653
ESC_RA0640AA	131	653
ESC_RA0639AA	131	653
ESC_RA0638AA	131	653
ESC_RA0637AA	131	653
ESC_RA0636AA	131	653
ESC_RA0635AA	131	653
ESC_RA0634AA	504	738
ESC_RA0633AA	10	512
ESC_RA0632AA	1611	198
ESC_RA0631AA	301	307
ESC_RA0630AA	11	936
ESC_RA0629AA	21	156
ESC_RA0628AA	21	156
ESC_RA0627AA	245	261
ESC_RA0624AA	131	653
ESC_RA0623AA	131	653
ESC_RA0622AA	131	653
ESC_RA0621AA	131	653
ESC_RA0620AA	131	653
ESC_RA0619AA	131	653
ESC_RA0618AA	405	923

ESC_RA0617AA	131	653
ESC_RA0616AA	131	653
ESC_RA0615AA	131	653
ESC_RA0614AA	131	653
ESC_RA0613AA	131	653
ESC_RA0612AA	131	653
ESC_RA0611AA	131	653
ESC_RA0610AA	131	653
ESC_RA0609AA	131	653
ESC_RA0608AA	131	653
ESC_RA0607AA	131	653
ESC_RA0606AA	131	653
ESC_RA0605AA	131	653
ESC_RA0604AA	131	653
ESC_RA0603AA	131	653
ESC_RA0602AA	131	653
ESC_RA0601AA	131	653
ESC_RA0600AA	131	653
ESC_RA0599AA	131	653
ESC_RA0598AA	131	653
ESC_RA0597AA	131	653
ESC_RA0596AA	131	653
ESC_RA0595AA	131	653
ESC_RA0594AA	131	653
ESC_RA0593AA	131	653
ESC_RA0592AA	131	653
ESC_RA0591AA	131	653
ESC_RA0590AA	131	653
ESC_RA0589AA	131	653
ESC_RA0588AA	131	653
ESC_RA0587AA	131	653
ESC_RA0586AA	131	653
ESC_RA0585AA	131	653
ESC_RA0584AA	131	653
ESC_RA0583AA	131	653
ESC_RA0582AA	131	653
ESC_RA0581AA	131	653
ESC_RA0580AA	2040	198
ESC_RA0579AA	2040	198
ESC_RA0578AA	10951	406
ESC_RA0577AA	10951	406
ESC_RA0576AA	131	653
ESC_RA0575AA	131	653
ESC_RA0574AA	131	653
ESC_RA0573AA	131	653
ESC_RA0572AA	131	653
ESC_RA0571AA	131	653
ESC_RA0570AA	131	653
ESC_RA0569AA	131	653
ESC_RA0568AA	131	653

ESC_RA0567AA	131	653
ESC_RA0566AA	131	653
ESC_RA0565AA	131	653
ESC_RA0564AA	131	653
ESC_RA0563AA	131	653
ESC_RA0562AA	131	653
ESC_RA0561AA	998	644
ESC_RA0560AA	998	644
ESC_RA0559AA	131	653
ESC_RA0558AA	131	653
ESC_RA0557AA	12	626
ESC_RA0556AA	131	653
ESC_RA0555AA	131	653
ESC_RA0554AA	131	653
ESC_RA0553AA	131	653
ESC_RA0552AA	131	653
ESC_RA0551AA	131	653
ESC_RA0550AA	12	626
ESC_RA0549AA	131	653
ESC_RA0548AA	88	160
ESC_RA0547AA	88	160
ESC_RA0546AA	131	653
ESC_RA0545AA	131	653
ESC_RA0544AA	131	653
ESC_RA0543AA	131	653
ESC_RA0542AA	131	653
ESC_RA0541AA	131	653
ESC_RA0540AA	131	653
ESC_RA0539AA	131	653
ESC_RA0538AA	131	653
ESC_RA0537AA	131	653
ESC_RA0536AA	131	653
ESC_RA0535AA	131	653
ESC_RA0534AA	131	653
ESC_RA0533AA	131	653
ESC_RA0532AA	131	653
ESC_RA0531AA	131	653
ESC_RA0530AA	131	653
ESC_RA0529AA	131	653
ESC_RA0528AA	131	653
ESC_RA0527AA	131	653
ESC_RA0526AA	131	653
ESC_RA0525AA	131	653
ESC_RA0524AA	131	653
ESC_RA0523AA	131	653
ESC_RA0522AA	131	653
ESC_RA0521AA	131	653
ESC_RA0520AA	131	653
ESC_RA0519AA	131	653
ESC_RA0518AA	131	653

ESC_RA0517AA	131	653
ESC_RA0516AA	131	653
ESC_RA0515AA	131	653
ESC_RA0514AA	12	626
ESC_RA0513AA	12	626
ESC_RA0512AA	131	653
ESC_RA0511AA	131	653
ESC_RA0510AA	131	653
ESC_RA0509AA	131	653
ESC_RA0508AA	224	198
ESC_RA0507AA	224	198
ESC_RA0506AA	131	653
ESC_RA0505AA	131	653
ESC_RA0504AA	131	653
ESC_RA0503AA	131	653
ESC_RA0502AA	131	653
ESC_RA0501AA	131	653
ESC_RA0500AA	131	653
ESC_RA0499AA	69	387
ESC_RA0498AA	69	387
ESC_RA0497AA	131	653
ESC_RA0496AA	131	653
ESC_RA0495AA	131	653
ESC_RA0494AA	131	653
ESC_RA0493AA	131	653
ESC_RA0492AA	131	653
ESC_RA0491AA	1119	361
ESC_RA0490AA	1119	361
ESC_RA0489AA	131	653
ESC_RA0488AA	131	653
ESC_RA0487AA	224	198
ESC_RA0486AA	224	198
ESC_RA0485AA	405	923
ESC_RA0484AA	131	653
ESC_RA0483AA	131	653
ESC_RA0482AA	131	653
ESC_RA0481AA	131	653
ESC_RA0480AA	131	653
ESC_RA0479AA	131	653
ESC_RA0478AA	152	257
ESC_RA0477AA	152	257
ESC_RA0476AA	152	257
ESC_RA0475AA	245	261
ESC_RA0474AA	245	261
ESC_RA0473AA	145	256
ESC_RA0472AA	288	102
ESC_RA0471AA	245	261
ESC_RA0470AA	152	257
ESC_RA0469AA	152	257
ESC_RA0468AA	152	257

ESC_RA0467AA	152	257
ESC_RA0466AA	245	261
ESC_RA0465AA	245	261
ESC_RA0464AA	152	257
ESC_RA0463AA	145	256
ESC_RA0462AA	245	261
ESC_RA0461AA	245	261
ESC_RA0460AA	152	257
ESC_RA0459AA	152	257
ESC_RA0458AA	245	261
ESC_RA0457AA	245	261
ESC_RA0456AA	245	261
ESC_RA0455AA	245	261
ESC_RA0454AA	245	261
ESC_RA0453AA	245	261
ESC_RA0452AA	1025	261
ESC_RA0445AA	95	738
ESC_RA0444AA	95	738
ESC_RA0443AA	95	738
ESC_RA0442AA	95	738
ESC_RA0441AA	95	738
ESC_RA0440AA	429	654
ESC_RA0439AA	95	738
ESC_RA0438AA	648	359
ESC_RA0437AA	95	738
ESC_RA0436AA	117	592
ESC_RA0435AA	2040	198
ESC_RA0434AA	429	654
ESC_RA0433AA	117	592
ESC_RA0432AA	648	359
ESC_RA0431AA	117	592
ESC_RA0430AA	429	654
ESC_RA0429AA	155	175
ESC_RA0428AA	117	592
ESC_RA0427AA	117	592
ESC_RA0426AA	428	654
ESC_RA0425AA	95	738
ESC_RA0424AA	95	738
ESC_RA0423AA	95	738
ESC_RA0422AA	10	512
ESC_RA0421AA	48	76
ESC_RA0420AA	48	76
ESC_RA0419AA	101	696
ESC_RA0418AA	101	696
ESC_RA0417AA	117	592
ESC_RA0416AA	616	156
ESC_RA0415AA	10	512
ESC_RA0413AA	648	359
ESC_RA0412AA	1638	519
ESC_RA0411AA	10	512

ESC_RA0409AA	95	738
ESC_RA0408AA	117	592
ESC_RA0407AA	117	592
ESC_RA0406AA	117	592
ESC_RA0405AA	101	696
ESC_RA0404AA	101	696
ESC_RA0403AA	8716	49
ESC_RA0402AA	38	669
ESC_RA0401AA	117	592
ESC_RA0400AA	352	744
ESC_RA0399AA	162	924
ESC_RA0398AA	162	924
ESC_RA0397AA	648	359
ESC_RA0396AA	162	924
ESC_RA0395AA	162	924
ESC_RA0394AA	117	592
ESC_RA0392AA	101	696
ESC_RA0391AA	803	739
ESC_RA0390AA	10	512
ESC_RA0389AA	162	924
ESC_RA0388AA	38	669
ESC_RA0387AA	155	175
ESC_RA0386AA	371	50
ESC_RA0385AA	429	654
ESC_RA0384AA	162	924
ESC_RA0383AA	101	696
ESC_RA0382AA	101	696
ESC_RA0381AA	117	592
ESC_RA0380AA	95	738
ESC_RA0379AA	428	654
ESC_RA0378AA	155	175
ESC_RA0377AA	155	175
ESC_RA0376AA	155	175
ESC_RA0375AA	155	175
ESC_RA0374AA	919	421
ESC_RA0373AA	919	421
ESC_RA0372AA	919	421
ESC_RA0371AA	919	421
ESC_RA0370AA	919	421
ESC_RA0368AA	117	592
ESC_RA0367AA	919	421
ESC_RA0366AA	95	738
ESC_RA0365AA	117	592
ESC_RA0364AA	57	50
ESC_RA0363AA	57	50
ESC_RA0362AA	57	50
ESC_RA0361AA	429	654
ESC_RA0360AA	117	592
ESC_RA0359AA	117	592
ESC_RA0358AA	69	387

ESC_RA0357AA	38	669
ESC_RA0356AA	542	824
ESC_RA0355AA	602	257
ESC_RA0354AA	10	512
ESC_RA0353AA	95	738
ESC_RA0352AA	140	738
ESC_RA0351AA	355	764
ESC_RA0350AA	155	175
ESC_RA0349AA	95	738
ESC_RA0348AA	101	696
ESC_RA0347AA	117	592
ESC_RA0346AA	8716	49
ESC_RA0345AA	429	654
ESC_RA0344AA	117	592
ESC_RA0343AA	117	592
ESC_RA0342AA	140	738
ESC_RA0341AA	117	592
ESC_RA0340AA	117	592
ESC_RA0338AA	117	592
ESC_RA0337AA	117	592
ESC_RA0336AA	117	592
ESC_RA0335AA	117	592
ESC_RA0334AA	117	592
ESC_RA0333AA	117	592
ESC_RA0332AA	117	592
ESC_RA0331AA	117	592
ESC_RA0330AA	101	696
ESC_RA0329AA	117	592
ESC_RA0328AA	117	592
ESC_RA0327AA	1638	519
ESC_RA0326AA	101	696
ESC_RA0325AA	117	592
ESC_RA0324AA	140	738
ESC_RA0323AA	95	738
ESC_RA0322AA	117	592
ESC_RA0321AA	117	592
ESC_RA0320AA	117	592
ESC_RA0319AA	117	592
ESC_RA0318AA	10944	592
ESC_RA0317AA	69	387
ESC_RA0316AA	1638	519
ESC_RA0315AA	117	592
ESC_RA0314AA	117	592
ESC_RA0313AA	10981	598
ESC_RA0312AA	117	592
ESC_RA0311AA	117	592
ESC_RA0310AA	95	738
ESC_RA0309AA	101	696
ESC_RA0308AA	117	592
ESC_RA0307AA	117	592

ESC_RA0305AA	1638	519
ESC_RA0304AA	10943	592
ESC_RA0303AA	117	592
ESC_RA0302AA	117	592
ESC_RA0301AA	101	696
ESC_RA0300AA	131	653
ESC_RA0299AA	371	50
ESC_RA0298AA	4045	592
ESC_RA0297AA	355	764
ESC_RA0296AA	117	592
ESC_RA0295AA	1158	387
ESC_RA0294AA	1638	519
ESC_RA0293AA	117	592
ESC_RA0292AA	117	592
ESC_RA0291AA	95	738
ESC_RA0290AA	95	738
ESC_RA0289AA	428	654
ESC_RA0288AA	38	669
ESC_RA0287AA	429	654
ESC_RA0286AA	38	669
ESC_RA0285AA	429	654
ESC_RA0284AA	429	654
ESC_RA0283AA	131	653
ESC_RA0282AA	429	654
ESC_RA0281AA	2040	198
ESC_RA0280AA	2040	198
ESC_RA0279AA	429	654
ESC_RA0278AA	949	175
ESC_RA0277AA	2040	198
ESC_RA0276AA	7963	664
ESC_RA0275AA	2040	198
ESC_RA0274AA	2040	198
ESC_RA0273AA	2040	198
ESC_RA0272AA	131	653
ESC_RA0271AA	429	654
ESC_RA0270AA	2040	198
ESC_RA0269AA	429	654
ESC_RA0268AA	2040	198
ESC_RA0267AA	2040	198
ESC_RA0266AA	131	653
ESC_RA0265AA	131	653
ESC_RA0264AA	69	387
ESC_RA0263AA	10	512
ESC_RA0262AA	10	512
ESC_RA0261AA	405	923
ESC_RA0260AA	405	923
ESC_RA0259AA	17	191
ESC_RA0258AA	1081	198
ESC_RA0257AA	10	512
ESC_RA0256AA	100	504

ESC_RA0255AA	641	935
ESC_RA0254AA	100	504
ESC_RA0253AA	12	626
ESC_RA0252AA	100	504
ESC_RA0251AA	10945	257
ESC_RA0249AA	10	512
ESC_RA0248AA	100	504
ESC_RA0247AA	73	737
ESC_RA0246AA	10	512
ESC_RA0245AA	641	935
ESC_RA0244AA	100	504
ESC_RA0243AA	100	504
ESC_RA0241AA	100	504
ESC_RA0240AA	100	504
ESC_RA0239AA	772	76
ESC_RA0238AA	100	504
ESC_RA0237AA	100	504
ESC_RA0236AA	58	198
ESC_RA0235AA	1670	438
ESC_RA0234AA	100	504
ESC_RA0233AA	100	504
ESC_RA0232AA	100	504
ESC_RA0231AA	100	504
ESC_RA0230AA	1	667
ESC_RA0229AA	10	512
ESC_RA0228AA	100	504
ESC_RA0227AA	100	504
ESC_RA0226AA	100	504
ESC_RA0225AA	9217	935
ESC_RA0223AA	10	512
ESC_RA0222AA	448	257
ESC_RA0221AA	117	592
ESC_RA0220AA	11	936
ESC_RA0219AA	659	286
ESC_RA0218AA	200	198
ESC_RA0217AA	10	512
ESC_RA0216AA	4599	504
ESC_RA0215AA	99	198
ESC_RA0214AA	2353	76
ESC_RA0213AA	3101	160
ESC_RA0212AA	442	257
ESC_RA0211AA	10	512
ESC_RA0210AA	10948	156
ESC_RA0209AA	58	198
ESC_RA0208AA	355	764
ESC_RA0207AA	17	191
ESC_RA0206AA	744	512
ESC_RA0205AA	1914	956
ESC_RA0204AA	675	234
ESC_RA0203AA	95	738

ESC_RA0202AA	5706	160
ESC_RA0201AA	410	156
ESC_RA0200AA	131	653
ESC_RA0199AA	88	160
ESC_RA0198AA	625	756
ESC_RA0197AA	90	156
ESC_RA0196AA	162	924
ESC_RA0195AA	491	626
ESC_RA0194AA	2473	257
ESC_RA0193AA	711	935
ESC_RA0192AA	442	257
ESC_RA0191AA	162	924
ESC_RA0190AA	95	738
ESC_RA0189AA	12	626
ESC_RA0188AA	6134	738
ESC_RA0187AA	607	355
ESC_RA0186AA	457	595
ESC_RA0185AA	10	512
ESC_RA0184AA	2217	62
ESC_RA0183AA	1992	257
ESC_RA0182AA	1112	504
ESC_RA0181AA	2368	406
ESC_RA0180AA	10946	540
ESC_RA0179AA	335	936
ESC_RA0178AA	57	50
ESC_RA0177AA	1463	263
ESC_RA0176AA	23	156
ESC_RA0175AA	10947	14
ESC_RA0174AA	127	738
ESC_RA0173AA	1844	198
ESC_RA0172AA	10971	393
ESC_RA0171AA	423	204
ESC_RA0170AA	295	160
ESC_RA0169AA	2280	212
ESC_RA0168AA	1771	868
ESC_RA0167AA	1844	198
ESC_RA0166AA	23	156
ESC_RA0165AA	9857	512
ESC_RA0164AA	555	653
ESC_RA0163AA	1131	936
ESC_RA0162AA	500	71
ESC_RA0161AA	410	156
ESC_RA0160AA	2619	738
ESC_RA0159AA	2064	261
ESC_RA0158AA	57	50
ESC_RA0157AA	410	156
ESC_RA0156AA	925	387
ESC_RA0155AA	4576	987
ESC_RA0154AA	953	447
ESC_RA0153AA	6526	737

ESC_RA0152AA	1695	504
ESC_RA0151AA	5236	257
ESC_RA0150AA	2079	198
ESC_RA0149AA	1252	567
ESC_RA0148AA	10979	257
ESC_RA0147AA	10	512
ESC_RA0146AA	2491	512
ESC_RA0145AA	746	789
ESC_RA0144AA	10	512
ESC_RA0143AA	162	924
ESC_RA0142AA	10978	740
ESC_RA0141AA	10949	512
ESC_RA0140AA	206	286
ESC_RA0139AA	1889	285
ESC_RA0138AA	1889	285
ESC_RA0137AA	555	653
ESC_RA0136AA	388	156
ESC_RA0135AA	101	696
ESC_RA0134AA	10	512
ESC_RA0133AA	11	936
ESC_RA0132AA	2607	198
ESC_RA0131AA	156	175
ESC_RA0130AA	21	156
ESC_RA0129AA	212	198
ESC_RA0128AA	56	198
ESC_RA0127AA	1403	924
ESC_RA0126AA	6505	376
ESC_RA0125AA	2520	878
ESC_RA0124AA	7250	919
ESC_RA0123AA	69	387
ESC_RA0122AA	58	198
ESC_RA0121AA	162	924
ESC_RA0120AA	131	653
ESC_RA0119AA	11	936
ESC_RA0118AA	607	355
ESC_RA0117AA	80	731
ESC_RA0116AA	8259	750
ESC_RA0115AA	372	424
ESC_RA0114AA	5600	373
ESC_RA0113AA	6505	376
ESC_RA0112AA	963	669
ESC_RA0111AA	1724	863
ESC_RA0110AA	2076	400
ESC_RA0109AA	1858	757
ESC_RA0108AA	140	738
ESC_RA0107AA	127	738
ESC_RA0106AA	131	653
ESC_RA0105AA	648	359
ESC_RA0104AA	555	653
ESC_RA0103AA	6505	376

ESC_RA0102AA	2721	373
ESC_RA0101AA	10950	623
ESC_RA0100AA	10982	900
ESC_RA0099AA	1844	198
ESC_RA0098AA	12	626
ESC_RA0097AA	127	738
ESC_RA0096AA	155	175
ESC_RA0095AA	642	924
ESC_RA0094AA	6506	914
ESC_RA0093AA	10952	737
ESC_RA0092AA	6076	817
ESC_RA0091AA	162	924
ESC_RA0090AA	6076	817
ESC_RA0089AA	3576	175
ESC_RA0088AA	224	198
ESC_RA0087AA	405	923
ESC_RA0086AA	2785	175
ESC_RA0085AA	101	696
ESC_RA0084AA	101	696
ESC_RA0083AA	104	737
ESC_RA0082AA	95	738
ESC_RA0081AA	2521	257
ESC_RA0080AA	1704	257
ESC_RA0079AA	5260	331
ESC_RA0078AA	388	156
ESC_RA0077AA	2721	373
ESC_RA0076AA	9574	427
ESC_RA0075AA	2678	737
ESC_RA0074AA	1726	257
ESC_RA0073AA	1155	740
ESC_RA0072AA	372	424
ESC_RA0071AA	155	175
ESC_RA0070AA	6497	221
ESC_RA0069AA	38	669
ESC_RA0068AA	10953	789
ESC_RA0067AA	362	234
ESC_RA0066AA	8820	234
ESC_RA0065AA	6498	766
ESC_RA0064AA	155	175
ESC_RA0063AA	7581	996
ESC_RA0062AA	906	198
ESC_RA0061AA	12	626
ESC_RA0060AA	135	653
ESC_RA0059AA	6577	763
ESC_RA0058AA	1159	381
ESC_RA0057AA	1858	757
ESC_RA0056AA	2602	257
ESC_RA0055AA	12	626
ESC_RA0054AA	206	286
ESC_RA0053AA	101	696

ESC_RA0052AA	4198	878
ESC_RA0051AA	206	286
ESC_RA0050AA	6529	198
ESC_RA0049AA	10955	234
ESC_RA0048AA	216	512
ESC_RA0047AA	6447	512
ESC_RA0046AA	906	198
ESC_RA0045AA	648	359
ESC_RA0044AA	533	198
ESC_RA0043AA	10954	525
ESC_RA0042AA	10980	789
ESC_RA0041AA	1167	257
ESC_RA0040AA	5443	373
ESC_RA0039AA	73	737
ESC_RA0038AA	7605	910
ESC_RA0037AA	127	738
ESC_RA0036AA	31	387
ESC_RA0035AA	925	387
ESC_RA0034AA	345	175
ESC_RA0033AA	58	198
ESC_RA0032AA	6404	257
ESC_RA0031AA	48	76
ESC_RA0030AA	429	654
ESC_RA0029AA	1294	512
ESC_RA0028AA	329	355
ESC_RA0027AA	86	935
ESC_RA0026AA	101	696
ESC_RA0025AA	216	512
ESC_RA0024AA	4559	878
ESC_RA0023AA	602	257
ESC_RA0022AA	1889	285
ESC_RA0021AA	1434	512
ESC_RA0020AA	1415	504
ESC_RA0019AA	2325	447
ESC_RA0018AA	224	198
ESC_RA0017AA	73	737
ESC_RA0016AA	3014	410
ESC_RA0015AA	10	512
ESC_RA0014AA	500	71
ESC_RA0013AA	648	359
ESC_RA0012AA	1308	257
ESC_RA0011AA	23	156
ESC_RA0010AA	10956	234
ESC_RA0009AA	2217	62
ESC_RA0008AA	57	50
ESC_RA0007AA	410	156
ESC_RA0006AA	127	738
ESC_RA0005AA	10	512
ESC_RA0004AA	925	387
ESC_RA0003AA	23	156

ESC_RA0002AA	48	76
ESC_RA0001AA	925	387
ESC_RA0000AA	336	878
ESC_QA9999AA	2491	512
ESC_QA9998AA	2491	512
ESC_QA9997AA	5579	175
ESC_QA9996AA	88	160
ESC_QA9995AA	57	50
ESC_QA9994AA	7605	910
ESC_QA9993AA	88	160
ESC_QA9992AA	23	156
ESC_QA9991AA	6522	439
ESC_QA9990AA	6522	439
ESC_QA9989AA	2329	924
ESC_QA9988AA	2354	257
ESC_QA9987AA	68	386
ESC_QA9986AA	57	50
ESC_QA9985AA	21	156
ESC_QA9984AA	29	156
ESC_QA9983AA	73	737
ESC_QA9982AA	2946	818
ESC_QA9981AA	1079	235
ESC_QA9980AA	88	160
ESC_QA9979AA	1081	198
ESC_QA9978AA	1081	198
ESC_QA9977AA	10	512
ESC_QA9976AA	625	756
ESC_QA9975AA	336	878
ESC_QA9974AA	10	512
ESC_QA9973AA	73	737
ESC_QA9972AA	1308	257
ESC_QA9971AA	2079	198
ESC_QA9970AA	998	644
ESC_QA9969AA	961	623
ESC_QA9968AA	10959	766
ESC_QA9967AA	127	738
ESC_QA9966AA	12	626
ESC_QA9965AA	2604	626
ESC_QA9964AA	349	670
ESC_QA9963AA	10958	217
ESC_QA9962AA	5260	331
ESC_QA9961AA	540	286
ESC_QA9960AA	206	286
ESC_QA9959AA	8009	731
ESC_QA9958AA	6565	453
ESC_QA9957AA	6503	257
ESC_QA9956AA	117	592
ESC_QA9955AA	624	359
ESC_QA9954AA	1079	235
ESC_QA9953AA	131	653

ESC_QA9952AA	4179	772
ESC_QA9951AA	10957	903
ESC_QA9950AA	5547	622
ESC_QA9949AA	1642	198
ESC_QA9948AA	35	745
ESC_QA9947AA	117	592
ESC_QA9946AA	155	175
ESC_QA9945AA	10	512
ESC_QA9944AA	10	512
ESC_QA9943AA	12	626
ESC_QA9942AA	118	985
ESC_QA9941AA	10983	626
ESC_QA9940AA	1081	198
ESC_QA9939AA	388	156
ESC_QA9938AA	200	198
ESC_QA9937AA	162	924
ESC_QA9936AA	224	198
ESC_QA9935AA	10962	672
ESC_QA9934AA	625	756
ESC_QA9933AA	8934	844
ESC_QA9932AA	43	512
ESC_QA9931AA	95	738
ESC_QA9930AA	43	512
ESC_QA9929AA	10	512
ESC_QA9928AA	6189	175
ESC_QA9927AA	10	512
ESC_QA9926AA	189	504
ESC_QA9925AA	906	198
ESC_QA9924AA	10961	198
ESC_QA9923AA	1145	512
ESC_QA9922AA	12	626
ESC_QA9921AA	351	268
ESC_QA9920AA	117	592
ESC_QA9919AA	117	592
ESC_QA9918AA	10	512
ESC_QA9917AA	1114	504
ESC_QA9916AA	973	968
ESC_QA9915AA	155	175
ESC_QA9914AA	155	175
ESC_QA9913AA	10960	440
ESC_QA9912AA	23	156
ESC_QA9911AA	90	156
ESC_QA9910AA	141	644
ESC_QA9909AA	1946	726
ESC_QA9908AA	95	738
ESC_QA9907AA	127	738
ESC_QA9906AA	127	738
ESC_QA9905AA	165	504
ESC_QA9904AA	4576	987
ESC_QA9903AA	95	738

ESC_QA9902AA	23	156
ESC_QA9901AA	95	738
ESC_QA9900AA	58	198
ESC_QA9899AA	555	653
ESC_QA9898AA	10	512
ESC_QA9897AA	56	198
ESC_QA9896AA	2520	878
ESC_QA9895AA	10964	286
ESC_QA9894AA	388	156
ESC_QA9893AA	93	71
ESC_QA9892AA	23	156
ESC_QA9891AA	350	50
ESC_QA9890AA	10963	850
ESC_QA9889AA	23	156
ESC_QA9888AA	6529	198
ESC_QA9887AA	95	738
ESC_QA9886AA	88	160
ESC_QA9885AA	86	935
ESC_QA9884AA	90	156
ESC_QA9883AA	641	935
ESC_QA9882AA	1007	268
ESC_QA9881AA	223	198
ESC_QA9880AA	95	738
ESC_QA9879AA	602	257
ESC_QA9878AA	8009	731
ESC_QA9877AA	93	71
ESC_QA9876AA	5681	286
ESC_QA9875AA	10	512
ESC_QA9874AA	38	669
ESC_QA9873AA	8099	191
ESC_QA9872AA	2614	975
ESC_QA9871AA	1844	198
ESC_QA9870AA	73	737
ESC_QA9869AA	88	160
ESC_QA9868AA	12	626
ESC_QA9867AA	2721	373
ESC_QA9866AA	127	738
ESC_QA9865AA	640	768
ESC_QA9864AA	95	738
ESC_QA9863AA	328	924
ESC_QA9862AA	335	936
ESC_QA9861AA	20	198
ESC_QA9860AA	201	257
ESC_QA9859AA	23	156
ESC_QA9858AA	10966	824
ESC_QA9857AA	5841	55
ESC_QA9856AA	973	968
ESC_QA9855AA	5663	670
ESC_QA9854AA	130	387
ESC_QA9853AA	1161	764

ESC_QA9852AA	2567	649
ESC_QA9851AA	10965	101
ESC_QA9850AA	1408	445
ESC_QA9849AA	135	653
ESC_QA9848AA	646	738
ESC_QA9847AA	641	935
ESC_QA9846AA	162	924
ESC_QA9845AA	117	592
ESC_QA9844AA	5663	670
ESC_QA9843AA	10	512
ESC_QA9842AA	998	644
ESC_QA9841AA	442	257
ESC_QA9840AA	410	156
ESC_QA9839AA	603	198
ESC_QA9838AA	10967	198
ESC_QA9837AA	95	738
ESC_QA9836AA	10	512
ESC_QA9835AA	127	738
ESC_QA9834AA	998	644
ESC_QA9833AA	131	653
ESC_QA9832AA	21	156
ESC_QA9831AA	6526	737
ESC_QA9830AA	34	504
ESC_QA9829AA	227	504
ESC_QA9828AA	127	738
ESC_QA9827AA	301	307
ESC_QA9826AA	1122	307
ESC_QA9825AA	405	923
ESC_QA9824AA	405	923
ESC_QA9823AA	405	923
ESC_QA9822AA	58	198
ESC_QA9821AA	5765	234
ESC_QA9820AA	8084	801
ESC_QA9819AA	648	359
ESC_QA9818AA	442	257
ESC_QA9817AA	10968	900
ESC_QA9816AA	2350	235
ESC_QA9815AA	115	669
ESC_QA9814AA	2559	373
ESC_QA9813AA	8084	801
ESC_QA9812AA	135	653
ESC_QA9811AA	156	175
ESC_QA9810AA	6467	452
ESC_QA9809AA	2521	257
ESC_QA9808AA	753	842
ESC_QA9807AA	1724	863
ESC_QA9806AA	906	198
ESC_QA9805AA	4049	234
ESC_QA9804AA	12	626
ESC_QA9803AA	10970	626

ESC_QA9802AA	95	738
ESC_QA9801AA	95	738
ESC_QA9800AA	95	738
ESC_QA9799AA	73	737
ESC_QA9798AA	602	257
ESC_QA9797AA	625	756
ESC_QA9796AA	73	737
ESC_QA9795AA	12	626
ESC_QA9794AA	453	935
ESC_QA9793AA	12	626
ESC_QA9792AA	2557	737
ESC_QA9791AA	73	737
ESC_QA9790AA	6343	504
ESC_QA9789AA	10	512
ESC_QA9788AA	80	731
ESC_QA9787AA	1994	944
ESC_QA9786AA	141	644
ESC_QA9785AA	73	737
ESC_QA9784AA	7706	525
ESC_QA9783AA	906	198
ESC_QA9782AA	1141	512
ESC_QA9781AA	1727	257
ESC_QA9780AA	10975	560
ESC_QA9779AA	127	738
ESC_QA9778AA	127	738
ESC_QA9777AA	681	393
ESC_QA9776AA	8157	924
ESC_QA9775AA	906	198
ESC_QA9774AA	355	764
ESC_QA9773AA	345	175
ESC_QA9772AA	162	924
ESC_QA9771AA	372	424
ESC_QA9770AA	10969	512
ESC_QA9769AA	83	740
ESC_QA9768AA	12	626
ESC_QA9767AA	2539	257
ESC_QA9766AA	83	740
ESC_QA9765AA	83	740
ESC_QA9764AA	929	742
ESC_QA9763AA	10	512
ESC_QA9762AA	1735	737
ESC_QA9761AA	555	653
ESC_QA9760AA	1735	737
ESC_QA9759AA	998	644
ESC_QA9758AA	10974	420
ESC_QA9757AA	127	738
ESC_QA9756AA	998	644
ESC_QA9755AA	998	644
ESC_QA9754AA	127	738
ESC_QA9753AA	95	738

ESC_QA9752AA	23	156
ESC_QA9751AA	998	644
ESC_QA9749AA	8009	731
ESC_QA9748AA	2354	257
ESC_QA9747AA	2354	257
ESC_QA9745AA	91	629
ESC_QA9744AA	155	175
ESC_QA9743AA	13	257
ESC_QA9742AA	8009	731
ESC_QA9741AA	641	935
ESC_QA9740AA	1114	504
ESC_QA9739AA	336	878
ESC_QA9738AA	442	257
ESC_QA9737AA	10972	198
ESC_QA9736AA	1721	311
ESC_QA9735AA	12	626
ESC_QA9734AA	542	824
ESC_QA9733AA	10973	127
ESC_QA9732AA	10	512
ESC_QA9731AA	675	234
ESC_QA9730AA	155	175
ESC_QA9729AA	154	257
ESC_QA9728AA	56	198
ESC_QA9727AA	127	738
ESC_QA9726AA	336	878
ESC_QA9725AA	23	156
ESC_QA9724AA	4576	987
ESC_QA9723AA	302	850
ESC_QA9722AA	95	738
ESC_QA9721AA	10977	156
ESC_QA9720AA	127	738
ESC_QA9719AA	361	512
ESC_QA9718AA	350	50
ESC_QA9717AA	88	160
ESC_QA9716AA	998	644
ESC_QA9715AA	23	156
ESC_QA9714AA	1308	257
ESC_QA9713AA	90	156
ESC_QA9712AA	120	180
ESC_QA9711AA	23	156
ESC_QA9710AA	929	742
ESC_QA9709AA	6531	878
ESC_QA9708AA	167	512
ESC_QA9707AA	167	512
ESC_QA9706AA	167	512
ESC_QA9705AA	617	511
ESC_QA9704AA	1196	257
ESC_QA9703AA	2006	198
ESC_QA9702AA	167	512
ESC_QA9701AA	167	512

ESC_QA9700AA	167	512
ESC_QA9699AA	167	512
ESC_QA9698AA	1735	737
ESC_QA9697AA	12	626
ESC_QA9696AA	127	738
ESC_QA9695AA	12	626
ESC_QA9694AA	372	424
ESC_QA9693AA	372	424
ESC_QA9692AA	95	738
ESC_QA9691AA	297	234
ESC_QA9690AA	127	738
ESC_QA9689AA	372	424
ESC_QA9688AA	162	924
ESC_QA9687AA	12	626
ESC_QA9686AA	9021	286
ESC_QA9685AA	11	936
ESC_QA9684AA	10	512
ESC_QA9683AA	10	512
ESC_QA9682AA	95	738
ESC_QA9681AA	11	936
ESC_QA9680AA	33	198
ESC_QA9679AA	589	650
ESC_QA9678AA	29	156
ESC_QA9677AA	10976	738
ESC_QA9676AA	73	737
ESC_QA9675AA	6196	223
ESC_QA9674AA	16	156
ESC_QA9673AA	10769	257
ESC_QA9669AA	10939	198
ESC_QA9668AA	10938	868
ESC_QA9667AA	10163	924
ESC_QA9664AA	10937	387
ESC_QA9663AA	10935	644
ESC_QA9661AA	10934	387
ESC_QA9660AA	10933	637
ESC_QA9659AA	10516	738
ESC_QA9656AA	10936	386
ESC_QA9654AA	10931	400
ESC_QA9653AA	10932	737
ESC_QA9652AA	10927	738
ESC_QA9651AA	10926	198
ESC_QA9650AA	10925	519
ESC_QA9649AA	10928	175
ESC_QA9647AA	10929	512
ESC_QA9646AA	10930	338
ESC_QA9645AA	10924	1004
ESC_QA9644AA	152	257
ESC_QA9643AA	245	261
ESC_QA9642AA	10	512
ESC_QA9641AA	223	198

ESC_QA9640AA	657	592
ESC_QA9639AA	58	198
ESC_QA9638AA	2514	504
ESC_QA9637AA	515	1004
ESC_QA9636AA	515	1004
ESC_QA9635AA	718	257
ESC_QA9634AA	223	198
ESC_QA9633AA	58	198
ESC_QA9632AA	56	198
ESC_QA9631AA	2387	261
ESC_QA9630AA	9973	198
ESC_QA9629AA	297	234
ESC_QA9628AA	101	696
ESC_QA9627AA	677	257
ESC_QA9626AA	648	359
ESC_QA9625AA	297	234
ESC_QA9624AA	7083	640
ESC_QA9623AA	1248	156
ESC_QA9622AA	29	156
ESC_QA9621AA	542	824
ESC_QA9620AA	345	175
ESC_QA9619AA	10	512
ESC_QA9618AA	137	270
ESC_QA9617AA	542	824
ESC_QA9616AA	10	512
ESC_QA9614AA	10	512
ESC_QA9613AA	131	653
ESC_QA9612AA	10	512
ESC_QA9611AA	342	286
ESC_QA9609AA	10	512
ESC_QA9608AA	3274	512
ESC_QA9607AA	10	512
ESC_QA9606AA	10	512
ESC_QA9604AA	641	935
ESC_QA9603AA	744	512
ESC_QA9602AA	342	286
ESC_QA9601AA	10923	504
ESC_QA9600AA	1790	198
ESC_QA9599AA	4379	878
ESC_QA9598AA	744	512
ESC_QA9597AA	10923	504
ESC_QA9596AA	34	504
ESC_QA9595AA	744	512
ESC_QA9594AA	58	198
ESC_QA9593AA	342	286
ESC_QA9592AA	10	512
ESC_QA9591AA	4253	512
ESC_QA9590AA	88	160
ESC_QA9588AA	10	512
ESC_QA9587AA	10	512

ESC_QA9586AA	10	512
ESC_QA9585AA	58	198
ESC_QA9584AA	641	935
ESC_QA9583AA	10	512
ESC_QA9582AA	88	160
ESC_QA9581AA	88	160
ESC_QA9580AA	744	512
ESC_QA9579AA	10	512
ESC_QA9578AA	10	512
ESC_QA9577AA	23	156
ESC_QA9576AA	34	504
ESC_QA9575AA	744	512
ESC_QA9574AA	744	512
ESC_QA9573AA	10	512
ESC_QA9572AA	10	512
ESC_QA9571AA	10	512
ESC_QA9569AA	744	512
ESC_QA9568AA	10	512
ESC_QA9567AA	10	512
ESC_QA9566AA	13	257
ESC_QA9565AA	744	512
ESC_QA9564AA	10	512
ESC_QA9563AA	744	512
ESC_QA9562AA	10	512
ESC_QA9561AA	1112	504
ESC_QA9560AA	152	257
ESC_QA9559AA	152	257
ESC_QA9558AA	245	261
ESC_QA9557AA	245	261
ESC_QA9556AA	152	257
ESC_QA9555AA	245	261
ESC_QA9554AA	245	261
ESC_QA9553AA	245	261
ESC_QA9552AA	152	257
ESC_QA9551AA	245	261
ESC_QA9550AA	145	256
ESC_QA9549AA	245	261
ESC_QA9548AA	152	257
ESC_QA9547AA	245	261
ESC_QA9546AA	245	261
ESC_QA9545AA	152	257
ESC_QA9544AA	245	261
ESC_QA9543AA	245	261
ESC_QA9542AA	245	261
ESC_QA9541AA	245	261
ESC_QA9540AA	10	512
ESC_QA9539AA	69	387
ESC_QA9538AA	38	669
ESC_QA9537AA	38	669
ESC_QA9536AA	69	387

ESC_QA9535AA	38	669
ESC_QA9534AA	648	359
ESC_QA9533AA	69	387
ESC_QA9532AA	361	512
ESC_QA9531AA	38	669
ESC_QA9530AA	38	669
ESC_QA9529AA	38	669
ESC_QA9528AA	38	669
ESC_QA9527AA	224	198
ESC_QA9526AA	38	669
ESC_QA9525AA	38	669
ESC_QA9524AA	38	669
ESC_QA9523AA	38	669
ESC_QA9522AA	38	669
ESC_QA9521AA	38	669
ESC_QA9520AA	38	669
ESC_QA9519AA	38	669
ESC_QA9518AA	38	669
ESC_QA9517AA	648	359
ESC_QA9516AA	617	511
ESC_QA9515AA	48	76
ESC_QA9514AA	38	669
ESC_QA9513AA	38	669
ESC_QA9512AA	38	669
ESC_QA9511AA	38	669
ESC_QA9510AA	38	669
ESC_QA9509AA	38	669
ESC_QA9508AA	38	669
ESC_QA9507AA	38	669
ESC_QA9506AA	38	669
ESC_QA9505AA	349	670
ESC_QA9504AA	3268	669
ESC_QA9503AA	38	669
ESC_QA9502AA	38	669
ESC_QA9501AA	38	669
ESC_QA9500AA	38	669
ESC_QA9499AA	38	669
ESC_QA9498AA	38	669
ESC_QA9497AA	38	669
ESC_QA9496AA	38	669
ESC_QA9495AA	38	669
ESC_QA9494AA	38	669
ESC_QA9493AA	38	669
ESC_QA9492AA	38	669
ESC_QA9491AA	38	669
ESC_QA9490AA	38	669
ESC_QA9489AA	38	669
ESC_QA9488AA	38	669
ESC_QA9486AA	224	198
ESC_QA9485AA	58	198

ESC_QA9484AA	38	669
ESC_QA9483AA	38	669
ESC_QA9482AA	38	669
ESC_QA9480AA	38	669
ESC_QA9479AA	10	512
ESC_QA9478AA	38	669
ESC_QA9477AA	38	669
ESC_QA9476AA	3268	669
ESC_QA9475AA	38	669
ESC_QA9474AA	38	669
ESC_QA9473AA	69	387
ESC_QA9472AA	38	669
ESC_QA9471AA	38	669
ESC_QA9470AA	3268	669
ESC_QA9469AA	69	387
ESC_QA9468AA	38	669
ESC_QA9467AA	38	669
ESC_QA9466AA	38	669
ESC_QA9465AA	38	669
ESC_QA9464AA	38	669
ESC_QA9463AA	38	669
ESC_QA9462AA	69	387
ESC_QA9461AA	361	512
ESC_QA9460AA	10921	672
ESC_QA9459AA	10922	645
ESC_QA9457AA	10918	626
ESC_QA9456AA	10919	258
ESC_QA9454AA	10920	343
ESC_QA9453AA	12	626
ESC_QA9452AA	10915	76
ESC_QA9451AA	10916	896
ESC_QA9450AA	10917	387
ESC_QA9449AA	10914	217
ESC_QA9448AA	10912	512
ESC_QA9447AA	10913	420
ESC_QA9446AA	10909	743
ESC_QA9445AA	10907	261
ESC_QA9444AA	10905	644
ESC_QA9443AA	10906	737
ESC_QA9442AA	10911	669
ESC_QA9441AA	10902	512
ESC_QA9440AA	10903	924
ESC_QA9439AA	10901	924
ESC_QA9438AA	10904	191
ESC_QA9437AA	10910	447
ESC_QA9436AA	10908	387
ESC_QA9435AA	10899	512
ESC_QA9434AA	10900	257
ESC_QA9433AA	10898	453
ESC_QA9432AA	10	512

ESC_QA9431AA	152	257
ESC_QA9430AA	145	256
ESC_QA9429AA	152	257
ESC_QA9403AA	7593	198
ESC_QA9401AA	906	198
ESC_QA9400AA	457	595
ESC_QA9399AA	428	654
ESC_QA9398AA	635	141
ESC_QA9397AA	388	156
ESC_QA9396AA	23	156
ESC_QA9395AA	388	156
ESC_QA9394AA	23	156
ESC_QA9393AA	428	654
ESC_QA9392AA	329	355
ESC_QA9391AA	329	355
ESC_QA9390AA	329	355
ESC_QA9389AA	329	355
ESC_QA9388AA	329	355
ESC_QA9387AA	329	355
ESC_QA9386AA	329	355
ESC_QA9385AA	100	504
ESC_QA9384AA	90	156
ESC_QA9383AA	329	355
ESC_QA9382AA	10	512
ESC_QA9381AA	5771	950
ESC_QA9380AA	725	765
ESC_QA9379AA	329	355
ESC_QA9378AA	329	355
ESC_QA9377AA	329	355
ESC_QA9376AA	329	355
ESC_QA9375AA	329	355
ESC_QA9374AA	329	355
ESC_QA9373AA	329	355
ESC_QA9372AA	111	175
ESC_QA9371AA	329	355
ESC_QA9370AA	38	669
ESC_QA9369AA	329	355
ESC_QA9368AA	329	355
ESC_QA9367AA	111	175
ESC_QA9366AA	111	175
ESC_QA9365AA	329	355
ESC_QA9364AA	329	355
ESC_QA9363AA	11	936
ESC_QA9362AA	329	355
ESC_QA9361AA	270	204
ESC_QA9360AA	270	204
ESC_QA9359AA	224	198
ESC_QA9357AA	38	669
ESC_QA9355AA	224	198
ESC_QA9354AA	224	198

ESC_QA9353AA	224	198
ESC_QA9352AA	100	504
ESC_QA9351AA	10	512
ESC_QA9350AA	90	156
ESC_QA9349AA	165	504
ESC_QA9348AA	90	156
ESC_QA9347AA	910	929
ESC_QA9346AA	90	156
ESC_QA9345AA	10	512
ESC_QA9344AA	542	824
ESC_QA9343AA	90	156
ESC_QA9342AA	1771	868
ESC_QA9341AA	245	261
ESC_QA9340AA	1025	261
ESC_QA9339AA	1025	261
ESC_QA9338AA	6367	261
ESC_QA9337AA	245	261
ESC_QA9336AA	152	257
ESC_QA9335AA	1025	261
ESC_QA9334AA	245	261
ESC_QA9333AA	245	261
ESC_QA9332AA	1025	261
ESC_QA9331AA	152	257
ESC_QA9330AA	7616	257
ESC_QA9329AA	155	175
ESC_QA9328AA	746	789
ESC_QA9327AA	58	198
ESC_QA9326AA	155	175
ESC_QA9325AA	155	175
ESC_QA9324AA	155	175
ESC_QA9323AA	155	175
ESC_QA9322AA	155	175
ESC_QA9321AA	9284	847
ESC_QA9320AA	9284	847
ESC_QA9319AA	155	175
ESC_QA9318AA	155	175
ESC_QA9317AA	2461	76
ESC_QA9316AA	155	175
ESC_QA9315AA	602	257
ESC_QA9314AA	155	175
ESC_QA9313AA	5826	198
ESC_QA9312AA	2772	257
ESC_QA9311AA	6186	868
ESC_QA9310AA	2165	257
ESC_QA9309AA	2772	257
ESC_QA9308AA	540	286
ESC_QA9307AA	540	286
ESC_QA9306AA	542	824
ESC_QA9305AA	540	286
ESC_QA9304AA	540	286

ESC_QA9303AA	540	286
ESC_QA9302AA	155	175
ESC_QA9301AA	155	175
ESC_QA9300AA	540	286
ESC_QA9299AA	155	175
ESC_QA9298AA	155	175
ESC_QA9297AA	540	286
ESC_QA9296AA	443	257
ESC_QA9295AA	48	76
ESC_QA9294AA	6025	233
ESC_QA9293AA	540	286
ESC_QA9292AA	155	175
ESC_QA9291AA	155	175
ESC_QA9290AA	9286	857
ESC_QA9289AA	155	175
ESC_QA9288AA	155	175
ESC_QA9287AA	155	175
ESC_QA9286AA	4392	234
ESC_QA9285AA	155	175
ESC_QA9284AA	155	175
ESC_QA9283AA	86	935
ESC_QA9282AA	942	198
ESC_QA9281AA	540	286
ESC_QA9280AA	2067	257
ESC_QA9279AA	2067	257
ESC_QA9278AA	2067	257
ESC_QA9277AA	2067	257
ESC_QA9276AA	212	198
ESC_QA9275AA	155	175
ESC_QA9274AA	212	198
ESC_QA9273AA	1423	198
ESC_QA9272AA	2614	975
ESC_QA9271AA	295	160
ESC_QA9270AA	2614	975
ESC_QA9269AA	2614	975
ESC_QA9268AA	196	257
ESC_QA9267AA	10654	246
ESC_QA9266AA	212	198
ESC_QA9265AA	212	198
ESC_QA9264AA	337	257
ESC_QA9263AA	9285	1008
ESC_QA9262AA	540	286
ESC_QA9261AA	152	257
ESC_QA9260AA	10896	261
ESC_QA9259AA	152	257
ESC_QA9258AA	243	197
ESC_QA9257AA	152	257
ESC_QA9256AA	152	257
ESC_QA9255AA	152	257
ESC_QA9254AA	152	257

ESC_QA9253AA	152	257
ESC_QA9252AA	1503	257
ESC_QA9251AA	152	257
ESC_QA9250AA	245	261
ESC_QA9249AA	152	257
ESC_QA9248AA	152	257
ESC_QA9247AA	243	197
ESC_QA9246AA	243	197
ESC_QA9245AA	152	257
ESC_QA9244AA	245	261
ESC_QA9243AA	1502	257
ESC_QA9242AA	152	257
ESC_QA9241AA	152	257
ESC_QA9240AA	152	257
ESC_QA9239AA	152	257
ESC_QA9238AA	152	257
ESC_QA9237AA	145	256
ESC_QA9236AA	152	257
ESC_QA9235AA	152	257
ESC_QA9234AA	152	257
ESC_QA9233AA	152	257
ESC_QA9232AA	148	191
ESC_QA9231AA	152	257
ESC_QA9211AA	152	257
ESC_QA9210AA	152	257
ESC_QA9209AA	152	257
ESC_QA9208AA	152	257
ESC_QA9207AA	152	257
ESC_QA9206AA	1503	257
ESC_QA9205AA	152	257
ESC_QA9204AA	152	257
ESC_QA9203AA	152	257
ESC_QA9202AA	152	257
ESC_QA9201AA	152	257
ESC_QA9200AA	152	257
ESC_QA9199AA	152	257
ESC_QA9198AA	152	257
ESC_QA9197AA	152	257
ESC_QA9196AA	152	257
ESC_QA9195AA	245	261
ESC_QA9194AA	630	261
ESC_QA9193AA	152	257
ESC_QA9192AA	152	257
ESC_QA9191AA	152	257
ESC_QA9190AA	152	257
ESC_QA9189AA	152	257
ESC_QA9188AA	152	257
ESC_QA9187AA	152	257
ESC_QA9186AA	152	257
ESC_QA9185AA	152	257

ESC_QA9184AA	152	257
ESC_QA9183AA	245	261
ESC_QA9182AA	152	257
ESC_QA9181AA	245	261
ESC_QA9180AA	152	257
ESC_QA9179AA	152	257
ESC_QA9178AA	152	257
ESC_QA9177AA	152	257
ESC_QA9176AA	152	257
ESC_QA9175AA	245	261
ESC_QA9174AA	245	261
ESC_QA9173AA	145	256
ESC_QA9172AA	152	257
ESC_QA9171AA	152	257
ESC_QA9170AA	152	257
ESC_QA9169AA	152	257
ESC_QA9168AA	152	257
ESC_QA9167AA	152	257
ESC_QA9166AA	1122	307
ESC_QA9165AA	3234	257
ESC_QA9164AA	8583	307
ESC_QA9163AA	345	175
ESC_QA9162AA	8618	512
ESC_QA9161AA	1079	235
ESC_QA9160AA	898	108
ESC_QA9159AA	10	512
ESC_QA9158AA	117	592
ESC_QA9157AA	83	740
ESC_QA9156AA	38	669
ESC_QA9155AA	80	731
ESC_QA9154AA	372	424
ESC_QA9153AA	38	669
ESC_QA9152AA	17	191
ESC_QA9151AA	3107	512
ESC_QA9150AA	58	198
ESC_QA9149AA	10894	268
ESC_QA9148AA	569	738
ESC_QA9147AA	196	257
ESC_QA9146AA	68	386
ESC_QA9145AA	38	669
ESC_QA9144AA	372	424
ESC_QA9143AA	372	424
ESC_QA9142AA	491	626
ESC_QA9141AA	162	924
ESC_QA9140AA	7841	924
ESC_QA9139AA	40	198
ESC_QA9138AA	141	644
ESC_QA9137AA	12	626
ESC_QA9136AA	127	738
ESC_QA9135AA	137	270

ESC_QA9134AA	10	512
ESC_QA9133AA	8233	512
ESC_QA9132AA	1049	175
ESC_QA9131AA	10890	160
ESC_QA9130AA	88	160
ESC_QA9129AA	46	447
ESC_QA9128AA	88	160
ESC_QA9127AA	88	160
ESC_QA9126AA	347	286
ESC_QA9125AA	359	696
ESC_QA9124AA	48	76
ESC_QA9123AA	32	270
ESC_QA9122AA	32	270
ESC_QA9121AA	137	270
ESC_QA9120AA	10850	512
ESC_QA9119AA	10889	160
ESC_QA9118AA	10888	824
ESC_QA9117AA	10	512
ESC_QA9116AA	3011	791
ESC_QA9115AA	48	76
ESC_QA9114AA	746	789
ESC_QA9113AA	524	270
ESC_QA9112AA	88	160
ESC_QA9111AA	156	175
ESC_QA9110AA	152	257
ESC_QA9109AA	245	261
ESC_QA9108AA	245	261
ESC_QA9107AA	1759	102
ESC_QA9106AA	152	257
ESC_QA9105AA	152	257
ESC_QA9104AA	245	261
ESC_QA9103AA	245	261
ESC_QA9102AA	245	261
ESC_QA9101AA	245	261
ESC_QA9100AA	245	261
ESC_QA9099AA	245	261
ESC_QA9098AA	245	261
ESC_QA9097AA	245	261
ESC_QA9096AA	152	257
ESC_QA9094AA	69	387
ESC_QA9093AA	504	738
ESC_QA9092AA	21	156
ESC_QA9091AA	4305	512
ESC_QA9090AA	5292	738
ESC_QA9089AA	278	924
ESC_QA9088AA	21	156
ESC_QA9087AA	2353	76
ESC_QA9086AA	33	198
ESC_QA9085AA	33	198
ESC_QA9084AA	442	257

ESC_QA9083AA	34	504
ESC_QA9082AA	33	198
ESC_QA9081AA	1877	773
ESC_QA9080AA	32	270
ESC_QA9079AA	278	924
ESC_QA9078AA	21	156
ESC_QA9077AA	677	257
ESC_QA9076AA	25	234
ESC_QA9075AA	33	198
ESC_QA9074AA	8381	234
ESC_QA9073AA	10	512
ESC_QA9072AA	33	198
ESC_QA9071AA	5989	156
ESC_QA9070AA	6314	56
ESC_QA9069AA	21	156
ESC_QA9068AA	131	653
ESC_QA9067AA	131	653
ESC_QA9066AA	131	653
ESC_QA9065AA	131	653
ESC_QA9064AA	12	626
ESC_QA9063AA	648	359
ESC_QA9062AA	38	669
ESC_QA9061AA	131	653
ESC_QA9060AA	38	669
ESC_QA9059AA	131	653
ESC_QA9058AA	131	653
ESC_QA9057AA	648	359
ESC_QA9056AA	131	653
ESC_QA9055AA	131	653
ESC_QA9054AA	648	359
ESC_QA9053AA	80	731
ESC_QA9052AA	127	738
ESC_QA9051AA	1312	76
ESC_QA9050AA	69	387
ESC_QA9049AA	131	653
ESC_QA9048AA	131	653
ESC_QA9047AA	648	359
ESC_QA9046AA	131	653
ESC_QA9045AA	131	653
ESC_QA9044AA	602	257
ESC_QA9043AA	224	198
ESC_QA9042AA	515	1004
ESC_QA9041AA	46	447
ESC_QA9040AA	10	512
ESC_QA9039AA	224	198
ESC_QA9038AA	46	447
ESC_QA9037AA	46	447
ESC_QA9036AA	69	387
ESC_QA9035AA	69	387
ESC_QA9034AA	616	156

ESC_QA9033AA	224	198
ESC_QA9032AA	10	512
ESC_QA9031AA	515	1004
ESC_QA9030AA	10	512
ESC_QA9029AA	69	387
ESC_QA9028AA	46	447
ESC_QA9027AA	48	76
ESC_QA9026AA	224	198
ESC_QA9025AA	278	924
ESC_QA9024AA	20	198
ESC_QA9023AA	10	512
ESC_QA9022AA	224	198
ESC_QA9021AA	10	512
ESC_QA9020AA	23	156
ESC_QA9019AA	224	198
ESC_QA9018AA	69	387
ESC_QA9017AA	224	198
ESC_QA9016AA	224	198
ESC_QA9015AA	1137	512
ESC_QA9014AA	10	512
ESC_QA9013AA	224	198
ESC_QA9012AA	10	512
ESC_QA9011AA	10	512
ESC_QA9010AA	69	387
ESC_QA9009AA	224	198
ESC_QA9008AA	224	198
ESC_QA9007AA	38	669
ESC_QA9006AA	501	900
ESC_QA9005AA	38	669
ESC_QA9004AA	10891	670
ESC_QA9003AA	349	670
ESC_QA9002AA	205	257
ESC_QA9001AA	1421	447
ESC_QA9000AA	131	653
ESC_QA8999AA	457	595
ESC_QA8998AA	1196	257
ESC_QA8997AA	7358	923
ESC_QA8996AA	1193	761
ESC_QA8995AA	131	653
ESC_QA8994AA	448	257
ESC_QA8993AA	131	653
ESC_QA8992AA	131	653
ESC_QA8991AA	44	512
ESC_QA8990AA	131	653
ESC_QA8989AA	2659	679
ESC_QA8988AA	2349	71
ESC_QA8987AA	1193	761
ESC_QA8986AA	44	512
ESC_QA8985AA	131	653
ESC_QA8984AA	410	156

ESC_QA8983AA	131	653
ESC_QA8982AA	405	923
ESC_QA8981AA	405	923
ESC_QA8980AA	131	653
ESC_QA8979AA	1163	579
ESC_QA8978AA	4385	934
ESC_QA8977AA	224	198
ESC_QA8976AA	224	198
ESC_QA8975AA	10	512
ESC_QA8974AA	10	512
ESC_QA8973AA	224	198
ESC_QA8972AA	10	512
ESC_QA8971AA	224	198
ESC_QA8970AA	392	234
ESC_QA8969AA	224	198
ESC_QA8968AA	10	512
ESC_QA8967AA	540	286
ESC_QA8966AA	10	512
ESC_QA8965AA	86	935
ESC_QA8964AA	540	286
ESC_QA8963AA	648	359
ESC_QA8962AA	683	175
ESC_QA8961AA	10892	824
ESC_QA8960AA	398	445
ESC_QA8959AA	20	198
ESC_QA8958AA	10893	198
ESC_QA8957AA	540	286
ESC_QA8956AA	10892	824
ESC_QA8955AA	2008	257
ESC_QA8954AA	10	512
ESC_QA8953AA	44	512
ESC_QA8952AA	20	198
ESC_QA8951AA	20	198
ESC_QA8950AA	20	198
ESC_QA8949AA	20	198
ESC_QA8948AA	155	175
ESC_QA8947AA	155	175
ESC_QA8946AA	58	198
ESC_QA8945AA	155	175
ESC_QA8944AA	10	512
ESC_QA8943AA	7618	925
ESC_QA8942AA	515	1004
ESC_QA8941AA	101	696
ESC_QA8940AA	135	653
ESC_QA8939AA	278	924
ESC_QA8938AA	2473	257
ESC_QA8937AA	10895	924
ESC_QA8936AA	10	512
ESC_QA8935AA	515	1004
ESC_QA8934AA	7618	925

ESC_QA8933AA	603	198
ESC_QA8932AA	130	387
ESC_QA8931AA	109	257
ESC_QA8930AA	155	175
ESC_QA8929AA	224	198
ESC_QA8928AA	2766	198
ESC_QA8927AA	515	1004
ESC_QA8926AA	515	1004
ESC_QA8925AA	10886	447
ESC_QA8920AA	152	257
ESC_QA8919AA	152	257
ESC_QA8918AA	245	261
ESC_QA8917AA	245	261
ESC_QA8916AA	152	257
ESC_QA8915AA	145	256
ESC_QA8913AA	15	766
ESC_QA8912AA	216	512
ESC_QA8911AA	216	512
ESC_QA8910AA	216	512
ESC_QA8909AA	152	257
ESC_QA8908AA	148	191
ESC_QA8907AA	245	261
ESC_QA8906AA	152	257
ESC_QA8905AA	152	257
ESC_QA8904AA	245	261
ESC_QA8903AA	152	257
ESC_QA8902AA	117	592
ESC_QA8901AA	131	653
ESC_QA8900AA	1421	447
ESC_QA8899AA	117	592
ESC_QA8898AA	131	653
ESC_QA8897AA	569	738
ESC_QA8896AA	162	924
ESC_QA8895AA	1955	900
ESC_QA8894AA	345	175
ESC_QA8893AA	101	696
ESC_QA8892AA	8588	257
ESC_QA8891AA	369	156
ESC_QA8890AA	3580	257
ESC_QA8889AA	1771	868
ESC_QA8888AA	8220	447
ESC_QA8887AA	2606	198
ESC_QA8886AA	1611	198
ESC_QA8885AA	21	156
ESC_QA8884AA	10	512
ESC_QA8883AA	1705	156
ESC_QA8882AA	442	257
ESC_QA8881AA	21	156
ESC_QA8880AA	21	156
ESC_QA8879AA	469	257

ESC_QA8878AA	3864	204
ESC_QA8877AA	2103	198
ESC_QA8876AA	200	198
ESC_QA8875AA	6263	122
ESC_QA8874AA	1490	935
ESC_QA8873AA	33	198
ESC_QA8872AA	33	198
ESC_QA8871AA	1763	120
ESC_QA8870AA	3947	337
ESC_QA8869AA	1858	757
ESC_QA8868AA	131	653
ESC_QA8867AA	998	644
ESC_QA8866AA	543	842
ESC_QA8865AA	6431	512
ESC_QA8864AA	393	343
ESC_QA8863AA	73	737
ESC_QA8861AA	141	644
ESC_QA8860AA	452	743
ESC_QA8859AA	10	512
ESC_QA8858AA	29	156
ESC_QA8857AA	398	445
ESC_QA8856AA	2952	926
ESC_QA8855AA	4247	936
ESC_QA8854AA	906	198
ESC_QA8853AA	10	512
ESC_QA8852AA	10	512
ESC_QA8851AA	10	512
ESC_QA8850AA	90	156
ESC_QA8849AA	90	156
ESC_QA8848AA	90	156
ESC_QA8847AA	227	504
ESC_QA8846AA	6256	76
ESC_QA8845AA	23	156
ESC_QA8843AA	877	935
ESC_QA8842AA	6354	447
ESC_QA8841AA	3889	291
ESC_QA8840AA	5876	847
ESC_QA8839AA	206	286
ESC_QA8838AA	10883	337
ESC_QA8837AA	10	512
ESC_QA8836AA	48	76
ESC_QA8835AA	8863	261
ESC_QA8834AA	1081	198
ESC_QA8833AA	10885	156
ESC_QA8831AA	5614	257
ESC_QA8830AA	1146	198
ESC_QA8829AA	10	512
ESC_QA8828AA	90	156
ESC_QA8827AA	90	156
ESC_QA8826AA	6584	609

ESC_QA8825AA	6584	609
ESC_QA8824AA	9963	318
ESC_QA8823AA	10	512
ESC_QA8822AA	9963	318
ESC_QA8821AA	6584	609
ESC_QA8820AA	410	156
ESC_QA8819AA	641	935
ESC_QA8818AA	410	156
ESC_QA8817AA	910	929
ESC_QA8816AA	910	929
ESC_QA8815AA	910	929
ESC_QA8814AA	910	929
ESC_QA8813AA	46	447
ESC_QA8812AA	10	512
ESC_QA8811AA	910	929
ESC_QA8810AA	910	929
ESC_QA8809AA	910	929
ESC_QA8808AA	10884	926
ESC_QA8807AA	10884	926
ESC_QA8806AA	10884	926
ESC_QA8805AA	1486	71
ESC_QA8804AA	10	512
ESC_QA8803AA	10	512
ESC_QA8802AA	910	929
ESC_QA8801AA	206	286
ESC_QA8800AA	90	156
ESC_QA8799AA	100	504
ESC_QA8797AA	1086	175
ESC_QA8796AA	641	935
ESC_QA8795AA	216	512
ESC_QA8794AA	216	512
ESC_QA8793AA	216	512
ESC_QA8792AA	216	512
ESC_QA8791AA	216	512
ESC_QA8790AA	216	512
ESC_QA8789AA	216	512
ESC_QA8788AA	216	512
ESC_QA8786AA	216	512
ESC_QA8785AA	216	512
ESC_QA8784AA	216	512
ESC_QA8782AA	216	512
ESC_QA8781AA	152	257
ESC_QA8780AA	7187	76
ESC_QA8779AA	88	160
ESC_QA8778AA	398	445
ESC_QA8777AA	131	653
ESC_QA8776AA	1673	257
ESC_QA8775AA	10	512
ESC_QA8774AA	117	592
ESC_QA8773AA	394	400

ESC_QA8772AA	10	512
ESC_QA8771AA	48	76
ESC_QA8770AA	1850	269
ESC_QA8769AA	117	592
ESC_QA8721AA	155	175
ESC_QA8720AA	3630	71
ESC_QA8710AA	152	257
ESC_QA8709AA	152	257
ESC_QA8708AA	152	257
ESC_QA8707AA	152	257
ESC_QA8706AA	245	261
ESC_QA8705AA	152	257
ESC_QA8702AA	3052	669
ESC_QA8700AA	191	198
ESC_QA8699AA	10	512
ESC_QA8698AA	10	512
ESC_QA8697AA	38	669
ESC_QA8695AA	69	387
ESC_QA8694AA	2599	257
ESC_QA8693AA	38	669
ESC_QA8692AA	5601	789
ESC_QA8691AA	450	71
ESC_QA8690AA	349	670
ESC_QA8689AA	349	670
ESC_QA8688AA	10	512
ESC_QA8687AA	10857	987
ESC_QA8686AA	191	198
ESC_QA8684AA	770	587
ESC_QA8683AA	10	512
ESC_QA8681AA	152	257
ESC_QA8680AA	152	257
ESC_QA8679AA	10853	249
ESC_QA8678AA	127	738
ESC_QA8677AA	3006	512
ESC_QA8676AA	3006	512
ESC_QA8675AA	57	50
ESC_QA8674AA	43	512
ESC_QA8673AA	10	512
ESC_QA8672AA	349	670
ESC_QA8671AA	349	670
ESC_QA8670AA	5964	355
ESC_QA8669AA	5964	355
ESC_QA8668AA	515	1004
ESC_QA8667AA	4512	71
ESC_QA8666AA	10	512
ESC_QA8665AA	93	71
ESC_QA8664AA	9721	234
ESC_QA8663AA	93	71
ESC_QA8662AA	93	71
ESC_QA8661AA	48	76

ESC_QA8660AA	352	744
ESC_QA8659AA	117	592
ESC_QA8658AA	93	71
ESC_QA8657AA	117	592
ESC_QA8656AA	117	592
ESC_QA8655AA	117	592
ESC_QA8654AA	95	738
ESC_QA8653AA	57	50
ESC_QA8652AA	9721	234
ESC_QA8651AA	9721	234
ESC_QA8650AA	43	512
ESC_QA8649AA	43	512
ESC_QA8648AA	10	512
ESC_QA8647AA	10	512
ESC_QA8646AA	43	512
ESC_QA8645AA	43	512
ESC_QA8644AA	4512	71
ESC_QA8643AA	4512	71
ESC_QA8642AA	206	286
ESC_QA8641AA	352	744
ESC_QA8640AA	10	512
ESC_QA8639AA	1049	175
ESC_QA8638AA	69	387
ESC_QA8637AA	10	512
ESC_QA8636AA	4512	71
ESC_QA8635AA	2184	50
ESC_QA8634AA	5962	923
ESC_QA8633AA	5931	670
ESC_QA8632AA	10	512
ESC_QA8631AA	10	512
ESC_QA8630AA	10855	76
ESC_QA8629AA	69	387
ESC_QA8628AA	4512	71
ESC_QA8627AA	10	512
ESC_QA8626AA	10	512
ESC_QA8625AA	10	512
ESC_QA8624AA	616	156
ESC_QA8623AA	5963	198
ESC_QA8622AA	10	512
ESC_QA8621AA	10	512
ESC_QA8620AA	10	512
ESC_QA8619AA	10	512
ESC_QA8618AA	10	512
ESC_QA8617AA	10	512
ESC_QA8616AA	10095	602
ESC_QA8615AA	210	108
ESC_QA8614AA	1850	269
ESC_QA8613AA	10	512
ESC_QA8612AA	6015	234
ESC_QA8611AA	10	512

ESC_QA8610AA	181	311
ESC_QA8609AA	5784	448
ESC_QA8608AA	388	156
ESC_QA8607AA	10	512
ESC_QA8606AA	131	653
ESC_QA8605AA	1072	355
ESC_QA8604AA	162	924
ESC_QA8603AA	127	738
ESC_QA8602AA	127	738
ESC_QA8601AA	666	257
ESC_QA8600AA	95	738
ESC_QA8599AA	117	592
ESC_QA8598AA	657	592
ESC_QA8597AA	10854	738
ESC_QA8596AA	453	935
ESC_QA8595AA	5628	156
ESC_QA8594AA	127	738
ESC_QA8593AA	206	286
ESC_QA8592AA	10856	500
ESC_QA8591AA	10	512
ESC_QA8590AA	127	738
ESC_QA8589AA	155	175
ESC_QA8588AA	58	198
ESC_QA8587AA	11	936
ESC_QA8586AA	11	936
ESC_QA8585AA	4496	924
ESC_QA8583AA	58	198
ESC_QA8582AA	11	936
ESC_QA8581AA	410	156
ESC_QA8580AA	399	198
ESC_QA8579AA	11	936
ESC_QA8578AA	131	653
ESC_QA8577AA	656	789
ESC_QA8576AA	297	234
ESC_QA8575AA	11	936
ESC_QA8574AA	13	257
ESC_QA8573AA	8825	304
ESC_QA8572AA	6	445
ESC_QA8571AA	11	936
ESC_QA8570AA	5295	355
ESC_QA8569AA	152	257
ESC_QA8568AA	152	257
ESC_QA8567AA	245	261
ESC_QA8566AA	245	261
ESC_QA8565AA	152	257
ESC_QA8564AA	245	261
ESC_QA8563AA	245	261
ESC_QA8562AA	245	261
ESC_QA8561AA	10851	261
ESC_QA8560AA	10850	512

ESC_QA8559AA	1193	761
ESC_QA8558AA	16	156
ESC_QA8557AA	722	422
ESC_QA8556AA	21	156
ESC_QA8555AA	29	156
ESC_QA8554AA	21	156
ESC_QA8553AA	4058	198
ESC_QA8552AA	21	156
ESC_QA8551AA	11	936
ESC_QA8550AA	1817	842
ESC_QA8549AA	1892	846
ESC_QA8548AA	678	257
ESC_QA8547AA	21	156
ESC_QA8546AA	21	156
ESC_QA8545AA	21	156
ESC_QA8544AA	21	156
ESC_QA8543AA	753	842
ESC_QA8542AA	301	307
ESC_QA8541AA	21	156
ESC_QA8540AA	442	257
ESC_QA8539AA	21	156
ESC_QA8538AA	399	198
ESC_QA8537AA	135	653
ESC_QA8536AA	9862	156
ESC_QA8535AA	602	257
ESC_QA8534AA	10852	936
ESC_QA8533AA	11	936
ESC_QA8532AA	11	936
ESC_QA8531AA	245	261
ESC_QA8530AA	245	261
ESC_QA8529AA	1759	102
ESC_QA8528AA	152	257
ESC_QA8527AA	152	257
ESC_QA8526AA	245	261
ESC_QA8525AA	245	261
ESC_QA8524AA	245	261
ESC_QA8523AA	245	261
ESC_QA8522AA	245	261
ESC_QA8521AA	245	261
ESC_QA8520AA	152	257
ESC_QA8519AA	442	257
ESC_QA8518AA	6	445
ESC_QA8517AA	10	512
ESC_QA8515AA	442	257
ESC_QA8514AA	33	198
ESC_QA8513AA	675	234
ESC_QA8512AA	677	257
ESC_QA8511AA	442	257
ESC_QA8510AA	442	257
ESC_QA8509AA	442	257

ESC_QA8508AA	11	936
ESC_QA8507AA	10849	669
ESC_QA8506AA	11	936
ESC_QA8505AA	3570	156
ESC_QA8504AA	11	936
ESC_QA8503AA	155	175
ESC_QA8502AA	11	936
ESC_QA8501AA	5292	738
ESC_QA8500AA	3101	160
ESC_QA8499AA	6	445
ESC_QA8498AA	21	156
ESC_QA8497AA	11	936
ESC_QA8496AA	11	936
ESC_QA8495AA	442	257
ESC_QA8494AA	11	936
ESC_QA8493AA	58	198
ESC_QA8492AA	1146	198
ESC_QA8491AA	58	198
ESC_QA8490AA	152	257
ESC_QA8489AA	21	156
ESC_QA8488AA	345	175
ESC_QA8487AA	448	257
ESC_QA8486AA	245	261
ESC_QA8485AA	245	261
ESC_QA8484AA	245	261
ESC_QA8483AA	245	261
ESC_QA8482AA	152	257
ESC_QA8481AA	245	261
ESC_QA8480AA	245	261
ESC_QA8479AA	245	261
ESC_QA8478AA	145	256
ESC_QA8477AA	152	257
ESC_QA8476AA	243	197
ESC_QA8475AA	245	261
ESC_QA8474AA	245	261
ESC_QA8473AA	245	261
ESC_QA8472AA	152	257
ESC_QA8471AA	152	257
ESC_QA8470AA	245	261
ESC_QA8469AA	245	261
ESC_QA8468AA	152	257
ESC_QA8467AA	245	261
ESC_QA8466AA	145	256
ESC_QA8465AA	145	256
ESC_QA8464AA	245	261
ESC_QA8463AA	152	257
ESC_QA8462AA	152	257
ESC_QA8461AA	245	261
ESC_QA8460AA	245	261
ESC_QA8459AA	245	261

ESC_QA8458AA	245	261
ESC_QA8457AA	245	261
ESC_QA8456AA	245	261
ESC_QA8455AA	152	257
ESC_QA8454AA	152	257
ESC_QA8453AA	152	257
ESC_QA8452AA	152	257
ESC_QA8451AA	152	257
ESC_QA8450AA	145	256
ESC_QA8449AA	152	257
ESC_QA8448AA	245	261
ESC_QA8447AA	245	261
ESC_QA8446AA	148	191
ESC_QA8445AA	245	261
ESC_QA8444AA	245	261
ESC_QA8443AA	152	257
ESC_QA8442AA	245	261
ESC_QA8441AA	245	261
ESC_QA8440AA	152	257
ESC_QA8439AA	152	257
ESC_QA8438AA	1021	261
ESC_QA8437AA	145	256
ESC_QA8436AA	152	257
ESC_QA8435AA	152	257
ESC_QA8434AA	8356	198
ESC_QA8433AA	131	653
ESC_QA8432AA	6	445
ESC_QA8431AA	442	257
ESC_QA8430AA	11	936
ESC_QA8429AA	517	924
ESC_QA8428AA	21	156
ESC_QA8427AA	11	936
ESC_QA8426AA	28	537
ESC_QA8425AA	603	198
ESC_QA8424AA	6	445
ESC_QA8423AA	504	738
ESC_QA8422AA	11	936
ESC_QA8421AA	33	198
ESC_QA8420AA	443	257
ESC_QA8419AA	661	92
ESC_QA8418AA	329	355
ESC_QA8417AA	11	936
ESC_QA8416AA	10	512
ESC_QA8415AA	7006	936
ESC_QA8414AA	11	936
ESC_QA8413AA	99	198
ESC_QA8412AA	11	936
ESC_QA8411AA	504	738
ESC_QA8410AA	5292	738
ESC_QA8409AA	2067	257

ESC_QA8408AA	811	234
ESC_QA8407AA	738	598
ESC_QA8406AA	3593	153
ESC_QA8405AA	10202	257
ESC_QA8404AA	6880	726
ESC_QA8403AA	21	156
ESC_QA8402AA	306	286
ESC_QA8401AA	6	445
ESC_QA8400AA	752	512
ESC_QA8399AA	200	198
ESC_QA8398AA	29	156
ESC_QA8397AA	542	824
ESC_QA8396AA	11	936
ESC_QA8395AA	17	191
ESC_QA8394AA	21	156
ESC_QA8393AA	32	270
ESC_QA8392AA	306	286
ESC_QA8391AA	301	307
ESC_QA8390AA	10	512
ESC_QA8389AA	4	76
ESC_QA8388AA	11	936
ESC_QA8387AA	46	447
ESC_QA8386AA	11	936
ESC_QA8385AA	583	768
ESC_QA8384AA	34	504
ESC_QA8383AA	21	156
ESC_QA8382AA	17	191
ESC_QA8381AA	10	512
ESC_QA8380AA	131	653
ESC_QA8379AA	442	257
ESC_QA8378AA	11	936
ESC_QA8377AA	300	286
ESC_QA8376AA	2966	936
ESC_QA8375AA	442	257
ESC_QA8374AA	17	191
ESC_QA8373AA	34	504
ESC_QA8372AA	10	512
ESC_QA8371AA	11	936
ESC_QA8370AA	442	257
ESC_QA8369AA	33	198
ESC_QA8368AA	648	359
ESC_QA8367AA	11	936
ESC_QA8366AA	3075	701
ESC_QA8365AA	677	257
ESC_QA8364AA	43	512
ESC_QA8363AA	295	160
ESC_QA8362AA	11	936
ESC_QA8361AA	5277	101
ESC_QA8360AA	11	936
ESC_QA8359AA	17	191

ESC_QA8358AA	661	92
ESC_QA8357AA	16	156
ESC_QA8356AA	11	936
ESC_QA8355AA	442	257
ESC_QA8354AA	442	257
ESC_QA8353AA	10	512
ESC_QA8352AA	33	198
ESC_QA8351AA	5989	156
ESC_QA8350AA	349	670
ESC_QA8349AA	278	924
ESC_QA8348AA	11	936
ESC_QA8347AA	11	936
ESC_QA8346AA	11	936
ESC_QA8345AA	2028	257
ESC_QA8344AA	32	270
ESC_QA8343AA	381	156
ESC_QA8342AA	21	156
ESC_QA8341AA	659	286
ESC_QA8340AA	25	234
ESC_QA8339AA	301	307
ESC_QA8338AA	10847	217
ESC_QA8337AA	10848	512
ESC_QA8336AA	33	198
ESC_QA8335AA	38	669
ESC_QA8334AA	11	936
ESC_QA8333AA	11	936
ESC_QA8332AA	11	936
ESC_QA8331AA	11	936
ESC_QA8330AA	6671	297
ESC_QA8329AA	11	936
ESC_QA8328AA	11	936
ESC_QA8327AA	11	936
ESC_QA8326AA	11	936
ESC_QA8325AA	6671	297
ESC_QA8324AA	58	198
ESC_QA8323AA	223	198
ESC_QA8321AA	345	175
ESC_QA8320AA	34	504
ESC_QA8319AA	58	198
ESC_QA8318AA	93	71
ESC_QA8317AA	399	198
ESC_QA8316AA	1084	180
ESC_QA8315AA	278	924
ESC_QA8314AA	131	653
ESC_QA8313AA	131	653
ESC_QA8312AA	131	653
ESC_QA8311AA	131	653
ESC_QA8310AA	131	653
ESC_QA8309AA	131	653
ESC_QA8308AA	131	653

ESC_QA8307AA	131	653
ESC_QA8306AA	1193	761
ESC_QA8305AA	69	387
ESC_QA8304AA	69	387
ESC_QA8303AA	69	387
ESC_QA8302AA	69	387
ESC_QA8301AA	69	387
ESC_QA8300AA	69	387
ESC_QA8299AA	405	923
ESC_QA8298AA	405	923
ESC_QA8297AA	405	923
ESC_QA8296AA	405	923
ESC_QA8295AA	1193	761
ESC_QA8294AA	10	512
ESC_QA8293AA	209	512
ESC_QA8292AA	44	512
ESC_QA8291AA	10	512
ESC_QA8290AA	48	76
ESC_QA8289AA	48	76
ESC_QA8288AA	8125	512
ESC_QA8287AA	131	653
ESC_QA8286AA	131	653
ESC_QA8285AA	131	653
ESC_QA8284AA	1193	761
ESC_QA8283AA	131	653
ESC_QA8282AA	10	512
ESC_QA8281AA	405	923
ESC_QA8280AA	156	175
ESC_QA8279AA	224	198
ESC_QA8278AA	1193	761
ESC_QA8277AA	1193	761
ESC_QA8276AA	38	669
ESC_QA8275AA	38	669
ESC_QA8274AA	38	669
ESC_QA8273AA	1177	669
ESC_QA8272AA	38	669
ESC_QA8271AA	95	738
ESC_QA8270AA	95	738
ESC_QA8269AA	95	738
ESC_QA8268AA	648	359
ESC_QA8267AA	1193	761
ESC_QA8266AA	648	359
ESC_QA8265AA	648	359
ESC_QA8264AA	12	626
ESC_QA8263AA	12	626
ESC_QA8262AA	12	626
ESC_QA8261AA	127	738
ESC_QA8260AA	73	737
ESC_QA8259AA	450	71
ESC_QA8258AA	155	175

ESC_QA8257AA	10	512
ESC_QA8256AA	1193	761
ESC_QA8255AA	648	359
ESC_QA8254AA	405	923
ESC_QA8253AA	1266	210
ESC_QA8252AA	155	175
ESC_QA8251AA	393	343
ESC_QA8250AA	1196	257
ESC_QA8249AA	10	512
ESC_QA8248AA	410	156
ESC_QA8247AA	62	574
ESC_QA8246AA	174	660
ESC_QA8245AA	1193	761
ESC_QA8244AA	1193	761
ESC_QA8243AA	1193	761
ESC_QA8242AA	1193	761
ESC_QA8241AA	131	653
ESC_QA8240AA	10844	375
ESC_QA8239AA	243	197
ESC_QA8238AA	152	257
ESC_QA8237AA	152	257
ESC_QA8236AA	245	261
ESC_QA8235AA	152	257
ESC_QA8234AA	245	261
ESC_QA8233AA	152	257
ESC_QA8232AA	245	261
ESC_QA8231AA	6200	261
ESC_QA8230AA	245	261
ESC_QA8229AA	152	257
ESC_QA8228AA	245	261
ESC_QA8227AA	245	261
ESC_QA8226AA	245	261
ESC_QA8225AA	345	175
ESC_QA8224AA	6263	122
ESC_QA8223AA	152	257
ESC_QA8222AA	11	936
ESC_QA8221AA	11	936
ESC_QA8220AA	11	936
ESC_QA8219AA	11	936
ESC_QA8218AA	11	936
ESC_QA8217AA	1432	257
ESC_QA8216AA	10	512
ESC_QA8215AA	80	731
ESC_QA8214AA	372	424
ESC_QA8213AA	127	738
ESC_QA8212AA	372	424
ESC_QA8211AA	3018	903
ESC_QA8210AA	420	741
ESC_QA8209AA	1193	761
ESC_QA8208AA	69	387

ESC_QA8207AA	73	737
ESC_QA8206AA	1723	900
ESC_QA8205AA	998	644
ESC_QA8204AA	127	738
ESC_QA8203AA	2015	684
ESC_QA8202AA	372	424
ESC_QA8201AA	73	737
ESC_QA8200AA	10845	737
ESC_QA8199AA	372	424
ESC_QA8198AA	10765	870
ESC_QA8197AA	372	424
ESC_QA8196AA	297	234
ESC_QA8195AA	770	587
ESC_QA8194AA	10	512
ESC_QA8193AA	540	286
ESC_QA8192AA	4417	234
ESC_QA8191AA	10	512
ESC_QA8190AA	641	935
ESC_QA8189AA	11	936
ESC_QA8188AA	11	936
ESC_QA8187AA	11	936
ESC_QA8186AA	11	936
ESC_QA8185AA	11	936
ESC_QA8184AA	11	936
ESC_QA8183AA	11	936
ESC_QA8182AA	11	936
ESC_QA8181AA	11	936
ESC_QA8180AA	11	936
ESC_QA8179AA	11	936
ESC_QA8178AA	11	936
ESC_QA8177AA	11	936
ESC_QA8176AA	11	936
ESC_QA8175AA	11	936
ESC_QA8174AA	11	936
ESC_QA8173AA	11	936
ESC_QA8172AA	11	936
ESC_QA8171AA	11	936
ESC_QA8170AA	11	936
ESC_QA8169AA	11	936
ESC_QA8168AA	11	936
ESC_QA8167AA	11	936
ESC_QA8166AA	11	936
ESC_QA8165AA	11	936
ESC_QA8164AA	11	936
ESC_QA8163AA	11	936
ESC_QA8162AA	11	936
ESC_QA8161AA	11	936
ESC_QA8160AA	11	936
ESC_QA8159AA	11	936
ESC_QA8158AA	11	936

ESC_QA8157AA	11	936
ESC_QA8156AA	11	936
ESC_QA8155AA	11	936
ESC_QA8154AA	11	936
ESC_QA8153AA	11	936
ESC_QA8152AA	11	936
ESC_QA8151AA	11	936
ESC_QA8150AA	11	936
ESC_QA8149AA	11	936
ESC_QA8148AA	11	936
ESC_QA8147AA	11	936
ESC_QA8146AA	11	936
ESC_QA8145AA	11	936
ESC_QA8144AA	11	936
ESC_QA8143AA	11	936
ESC_QA8142AA	11	936
ESC_QA8141AA	11	936
ESC_QA8140AA	11	936
ESC_QA8139AA	11	936
ESC_QA8138AA	11	936
ESC_QA8137AA	11	936
ESC_QA8136AA	11	936
ESC_QA8135AA	11	936
ESC_QA8134AA	11	936
ESC_QA8133AA	11	936
ESC_QA8132AA	11	936
ESC_QA8131AA	11	936
ESC_QA8130AA	11	936
ESC_QA8129AA	11	936
ESC_QA8128AA	11	936
ESC_QA8127AA	1598	447
ESC_QA8126AA	1598	447
ESC_QA8125AA	10	512
ESC_QA8124AA	354	15
ESC_QA8123AA	48	76
ESC_QA8122AA	450	71
ESC_QA8121AA	131	653
ESC_QA8120AA	69	387
ESC_QA8119AA	93	71
ESC_QA8118AA	695	519
ESC_QA8117AA	450	71
ESC_QA8115AA	450	71
ESC_QA8114AA	695	519
ESC_QA8113AA	131	653
ESC_QA8112AA	2006	198
ESC_QA8111AA	58	198
ESC_QA8109AA	131	653
ESC_QA8108AA	69	387
ESC_QA8107AA	1148	257
ESC_QA8106AA	46	447

ESC_QA8104AA	131	653
ESC_QA8103AA	963	669
ESC_QA8102AA	617	511
ESC_QA8101AA	127	738
ESC_QA8100AA	8855	175
ESC_QA8098AA	744	512
ESC_QA8097AA	46	447
ESC_QA8096AA	69	387
ESC_QA8095AA	10	512
ESC_QA8094AA	162	924
ESC_QA8093AA	695	519
ESC_QA8092AA	167	512
ESC_QA8091AA	648	359
ESC_QA8090AA	224	198
ESC_QA8089AA	10	512
ESC_QA8088AA	3897	789
ESC_QA8087AA	648	359
ESC_QA8086AA	205	257
ESC_QA8084AA	457	595
ESC_QA8083AA	8869	885
ESC_QA8082AA	3944	512
ESC_QA8079AA	10	512
ESC_QA8077AA	43	512
ESC_QA8076AA	93	71
ESC_QA8075AA	131	653
ESC_QA8074AA	38	669
ESC_QA8073AA	1288	512
ESC_QA8072AA	648	359
ESC_QA8070AA	69	387
ESC_QA8068AA	617	511
ESC_QA8067AA	746	789
ESC_QA8066AA	744	512
ESC_QA8065AA	540	286
ESC_QA8064AA	58	198
ESC_QA8062AA	2541	878
ESC_QA8061AA	12	626
ESC_QA8060AA	95	738
ESC_QA8059AA	156	175
ESC_QA8057AA	131	653
ESC_QA8056AA	616	156
ESC_QA8055AA	973	968
ESC_QA8054AA	398	445
ESC_QA8053AA	278	924
ESC_QA8052AA	6416	1004
ESC_QA8051AA	398	445
ESC_QA8050AA	2536	261
ESC_QA8049AA	101	696
ESC_QA8048AA	394	400
ESC_QA8047AA	278	924
ESC_QA8046AA	2536	261

ESC_QA8045AA	10	512
ESC_QA8044AA	10	512
ESC_QA8043AA	2325	447
ESC_QA8042AA	10846	291
ESC_QA8041AA	58	198
ESC_QA8040AA	2325	447
ESC_QA8039AA	162	924
ESC_QA8038AA	10	512
ESC_QA8037AA	428	654
ESC_QA8036AA	23	156
ESC_QA8035AA	6006	238
ESC_QA8034AA	127	738
ESC_QA8033AA	404	761
ESC_QA8032AA	2179	924
ESC_QA8031AA	95	738
ESC_QA8030AA	703	743
ESC_QA8029AA	357	654
ESC_QA8028AA	355	764
ESC_QA8027AA	95	738
ESC_QA8026AA	404	761
ESC_QA8025AA	10843	737
ESC_QA8024AA	141	644
ESC_QA8023AA	69	387
ESC_QA8022AA	131	653
ESC_QA8021AA	141	644
ESC_QA8020AA	73	737
ESC_QA8019AA	59	217
ESC_QA8018AA	73	737
ESC_QA8017AA	131	653
ESC_QA8016AA	131	653
ESC_QA8015AA	58	198
ESC_QA8014AA	69	387
ESC_QA8013AA	569	738
ESC_QA8012AA	88	160
ESC_QA8011AA	95	738
ESC_QA8010AA	2556	630
ESC_QA8009AA	127	738
ESC_QA8008AA	95	738
ESC_QA8007AA	404	761
ESC_QA8006AA	95	738
ESC_QA8005AA	295	160
ESC_QA8004AA	636	653
ESC_QA8003AA	127	738
ESC_QA8002AA	404	761
ESC_QA8001AA	73	737
ESC_QA8000AA	73	737
ESC_QA7999AA	404	761
ESC_QA7998AA	141	644
ESC_QA7997AA	69	387
ESC_QA7996AA	2076	400

ESC_QA7995AA	95	738
ESC_QA7994AA	131	653
ESC_QA7993AA	648	359
ESC_QA7992AA	80	731
ESC_QA7991AA	73	737
ESC_QA7990AA	73	737
ESC_QA7989AA	62	574
ESC_QA7987AA	131	653
ESC_QA7986AA	10842	286
ESC_QA7985AA	69	387
ESC_QA7984AA	131	653
ESC_QA7983AA	12	626
ESC_QA7982AA	69	387
ESC_QA7981AA	141	644
ESC_QA7980AA	421	744
ESC_QA7979AA	95	738
ESC_QA7978AA	95	738
ESC_QA7977AA	69	387
ESC_QA7976AA	12	626
ESC_QA7975AA	141	644
ESC_QA7974AA	131	653
ESC_QA7973AA	681	393
ESC_QA7972AA	162	924
ESC_QA7971AA	95	738
ESC_QA7970AA	95	738
ESC_QA7969AA	69	387
ESC_QA7968AA	73	737
ESC_QA7967AA	141	644
ESC_QA7966AA	2015	684
ESC_QA7965AA	968	737
ESC_QA7964AA	10	512
ESC_QA7963AA	95	738
ESC_QA7962AA	127	738
ESC_QA7961AA	10	512
ESC_QA7960AA	62	574
ESC_QA7959AA	69	387
ESC_QA7958AA	95	738
ESC_QA7957AA	421	744
ESC_QA7956AA	131	653
ESC_QA7955AA	569	738
ESC_QA7954AA	10	512
ESC_QA7953AA	537	756
ESC_QA7952AA	404	761
ESC_QA7951AA	38	669
ESC_QA7950AA	58	198
ESC_QA7949AA	95	738
ESC_QA7948AA	141	644
ESC_QA7947AA	453	935
ESC_QA7946AA	95	738
ESC_QA7945AA	69	387

ESC_QA7944AA	404	761
ESC_QA7943AA	131	653
ESC_QA7942AA	405	923
ESC_QA7941AA	404	761
ESC_QA7940AA	69	387
ESC_QA7939AA	144	740
ESC_QA7938AA	141	644
ESC_QA7937AA	95	738
ESC_QA7936AA	95	738
ESC_QA7935AA	73	737
ESC_QA7934AA	131	653
ESC_QA7933AA	457	595
ESC_QA7932AA	131	653
ESC_QA7931AA	10	512
ESC_QA7930AA	69	387
ESC_QA7929AA	131	653
ESC_QA7928AA	73	737
ESC_QA7927AA	404	761
ESC_QA7926AA	1310	891
ESC_QA7925AA	73	737
ESC_QA7924AA	131	653
ESC_QA7923AA	10801	160
ESC_QA7922AA	73	737
ESC_QA7921AA	95	738
ESC_QA7920AA	404	761
ESC_QA7919AA	131	653
ESC_QA7918AA	38	669
ESC_QA7917AA	73	737
ESC_QA7916AA	95	738
ESC_QA7915AA	95	738
ESC_QA7914AA	73	737
ESC_QA7913AA	131	653
ESC_QA7912AA	12	626
ESC_QA7911AA	95	738
ESC_QA7910AA	1057	761
ESC_QA7909AA	23	156
ESC_QA7908AA	131	653
ESC_QA7907AA	69	387
ESC_QA7906AA	73	737
ESC_QA7905AA	127	738
ESC_QA7904AA	10	512
ESC_QA7903AA	95	738
ESC_QA7902AA	131	653
ESC_QA7901AA	59	217
ESC_QA7900AA	73	737
ESC_QA7899AA	10841	761
ESC_QA7898AA	69	387
ESC_QA7897AA	69	387
ESC_QA7896AA	12	626
ESC_QA7895AA	141	644

ESC_QA7894AA	372	424
ESC_QA7893AA	69	387
ESC_QA7892AA	12	626
ESC_QA7891AA	141	644
ESC_QA7890AA	141	644
ESC_QA7889AA	117	592
ESC_QA7888AA	141	644
ESC_QA7887AA	141	644
ESC_QA7886AA	141	644
ESC_QA7885AA	69	387
ESC_QA7884AA	95	738
ESC_QA7883AA	69	387
ESC_QA7882AA	1126	198
ESC_QA7881AA	38	669
ESC_QA7880AA	421	744
ESC_QA7879AA	73	737
ESC_QA7878AA	410	156
ESC_QA7877AA	73	737
ESC_QA7876AA	38	669
ESC_QA7875AA	69	387
ESC_QA7874AA	131	653
ESC_QA7873AA	73	737
ESC_QA7872AA	69	387
ESC_QA7871AA	73	737
ESC_QA7870AA	69	387
ESC_QA7869AA	38	669
ESC_QA7868AA	93	71
ESC_QA7867AA	69	387
ESC_QA7866AA	12	626
ESC_QA7865AA	141	644
ESC_QA7864AA	10	512
ESC_QA7863AA	131	653
ESC_QA7862AA	404	761
ESC_QA7861AA	88	160
ESC_QA7860AA	10	512
ESC_QA7859AA	95	738
ESC_QA7858AA	131	653
ESC_QA7857AA	73	737
ESC_QA7856AA	73	737
ESC_QA7855AA	69	387
ESC_QA7854AA	95	738
ESC_QA7853AA	404	761
ESC_QA7852AA	10	512
ESC_QA7851AA	1064	756
ESC_QA7850AA	421	744
ESC_QA7849AA	1193	761
ESC_QA7848AA	69	387
ESC_QA7847AA	1193	761
ESC_QA7846AA	538	629
ESC_QA7845AA	998	644

ESC_QA7844AA	104	737
ESC_QA7843AA	80	731
ESC_QA7842AA	95	738
ESC_QA7841AA	10	512
ESC_QA7840AA	404	761
ESC_QA7839AA	73	737
ESC_QA7838AA	131	653
ESC_QA7837AA	88	160
ESC_QA7836AA	131	653
ESC_QA7835AA	73	737
ESC_QA7834AA	127	738
ESC_QA7833AA	73	737
ESC_QA7832AA	131	653
ESC_QA7831AA	10840	737
ESC_QA7830AA	95	738
ESC_QA7829AA	681	393
ESC_QA7828AA	73	737
ESC_QA7827AA	62	574
ESC_QA7826AA	14	761
ESC_QA7825AA	95	738
ESC_QA7824AA	537	756
ESC_QA7823AA	69	387
ESC_QA7822AA	2558	782
ESC_QA7821AA	1663	737
ESC_QA7820AA	73	737
ESC_QA7819AA	117	592
ESC_QA7818AA	69	387
ESC_QA7817AA	73	737
ESC_QA7816AA	141	644
ESC_QA7815AA	144	740
ESC_QA7814AA	14	761
ESC_QA7813AA	410	156
ESC_QA7812AA	88	160
ESC_QA7811AA	1083	203
ESC_QA7810AA	69	387
ESC_QA7809AA	362	234
ESC_QA7808AA	10839	234
ESC_QA7806AA	6559	234
ESC_QA7805AA	9192	595
ESC_QA7804AA	56	198
ESC_QA7803AA	278	924
ESC_QA7802AA	95	738
ESC_QA7801AA	4481	257
ESC_QA7800AA	2163	257
ESC_QA7799AA	4175	270
ESC_QA7798AA	446	257
ESC_QA7797AA	1844	198
ESC_QA7796AA	155	175
ESC_QA7795AA	155	175
ESC_QA7794AA	8619	924

ESC_QA7793AA	10	512
ESC_QA7792AA	442	257
ESC_QA7791AA	57	50
ESC_QA7790AA	329	355
ESC_QA7789AA	937	198
ESC_QA7788AA	973	968
ESC_QA7787AA	95	738
ESC_QA7786AA	56	198
ESC_QA7785AA	8935	101
ESC_QA7784AA	56	198
ESC_QA7783AA	4260	737
ESC_QA7782AA	56	198
ESC_QA7781AA	6189	175
ESC_QA7780AA	1125	198
ESC_QA7779AA	201	257
ESC_QA7778AA	657	592
ESC_QA7777AA	75	257
ESC_QA7776AA	937	198
ESC_QA7775AA	4086	842
ESC_QA7774AA	2163	257
ESC_QA7773AA	10	512
ESC_QA7772AA	9190	156
ESC_QA7771AA	56	198
ESC_QA7770AA	10	512
ESC_QA7769AA	58	198
ESC_QA7768AA	8651	908
ESC_QA7767AA	336	878
ESC_QA7766AA	58	198
ESC_QA7765AA	4481	257
ESC_QA7764AA	278	924
ESC_QA7763AA	297	234
ESC_QA7762AA	118	985
ESC_QA7761AA	603	198
ESC_QA7760AA	101	696
ESC_QA7759AA	58	198
ESC_QA7758AA	1001	257
ESC_QA7757AA	1432	257
ESC_QA7756AA	1081	198
ESC_QA7755AA	4151	971
ESC_QA7754AA	329	355
ESC_QA7753AA	10	512
ESC_QA7752AA	8860	175
ESC_QA7751AA	7812	257
ESC_QA7750AA	206	286
ESC_QA7749AA	5973	92
ESC_QA7748AA	58	198
ESC_QA7747AA	187	257
ESC_QA7746AA	10	512
ESC_QA7745AA	2521	257
ESC_QA7744AA	155	175

ESC_QA7743AA	10838	114
ESC_QA7742AA	118	985
ESC_QA7741AA	101	696
ESC_QA7740AA	224	198
ESC_QA7739AA	10837	898
ESC_QA7738AA	398	445
ESC_QA7737AA	5597	72
ESC_QA7736AA	392	234
ESC_QA7735AA	1246	198
ESC_QA7734AA	300	286
ESC_QA7733AA	29	156
ESC_QA7732AA	1308	257
ESC_QA7731AA	38	669
ESC_QA7730AA	1246	198
ESC_QA7729AA	10836	962
ESC_QA7728AA	10836	962
ESC_QA7727AA	10836	962
ESC_QA7726AA	10836	962
ESC_QA7723AA	245	261
ESC_QA7722AA	152	257
ESC_QA7720AA	245	261
ESC_QA7719AA	245	261
ESC_QA7718AA	245	261
ESC_QA7717AA	245	261
ESC_QA7716AA	152	257
ESC_QA7715AA	152	257
ESC_QA7714AA	245	261
ESC_QA7713AA	245	261
ESC_QA7712AA	152	257
ESC_QA7711AA	245	261
ESC_QA7710AA	145	256
ESC_QA7709AA	152	257
ESC_QA7708AA	245	261
ESC_QA7707AA	245	261
ESC_QA7706AA	245	261
ESC_QA7705AA	245	261
ESC_QA7704AA	245	261
ESC_QA7703AA	245	261
ESC_QA7702AA	245	261
ESC_QA7701AA	630	261
ESC_QA7700AA	245	261
ESC_QA7699AA	245	261
ESC_QA7698AA	148	191
ESC_QA7697AA	5517	257
ESC_QA7696AA	7384	257
ESC_QA7695AA	660	286
ESC_QA7694AA	117	592
ESC_QA7693AA	11	936
ESC_QA7692AA	156	175
ESC_QA7690AA	56	198

ESC_QA7689AA	56	198
ESC_QA7684AA	56	198
ESC_QA7683AA	56	198
ESC_QA7682AA	56	198
ESC_QA7681AA	2165	257
ESC_QA7680AA	11	936
ESC_QA7678AA	56	198
ESC_QA7677AA	11	936
ESC_QA7676AA	156	175
ESC_QA7673AA	11	936
ESC_QA7672AA	56	198
ESC_QA7670AA	10835	387
ESC_QA7669AA	56	198
ESC_QA7668AA	5487	198
ESC_QA7667AA	11	936
ESC_QA7665AA	11	936
ESC_QA7664AA	11	936
ESC_QA7663AA	11	936
ESC_QA7662AA	5487	198
ESC_QA7661AA	11	936
ESC_QA7660AA	11	936
ESC_QA7659AA	10	512
ESC_QA7658AA	10	512
ESC_QA7657AA	10	512
ESC_QA7656AA	10	512
ESC_QA7655AA	10	512
ESC_QA7654AA	10	512
ESC_QA7653AA	10	512
ESC_QA7652AA	10	512
ESC_QA7651AA	10	512
ESC_QA7650AA	10	512
ESC_QA7649AA	10	512
ESC_QA7648AA	10	512
ESC_QA7647AA	10	512
ESC_QA7646AA	10	512
ESC_QA7645AA	10	512
ESC_QA7644AA	10	512
ESC_QA7643AA	10	512
ESC_QA7642AA	10	512
ESC_QA7641AA	10	512
ESC_QA7640AA	10	512
ESC_QA7639AA	10	512
ESC_QA7638AA	10	512
ESC_QA7637AA	10	512
ESC_QA7636AA	10	512
ESC_QA7635AA	10	512
ESC_QA7634AA	10	512
ESC_QA7633AA	10	512
ESC_QA7632AA	10	512
ESC_QA7631AA	10	512

ESC_QA7630AA	10	512
ESC_QA7629AA	10	512
ESC_QA7628AA	10	512
ESC_QA7627AA	10	512
ESC_QA7626AA	10	512
ESC_QA7625AA	10	512
ESC_QA7624AA	10	512
ESC_QA7623AA	17	191
ESC_QA7622AA	7804	369
ESC_QA7621AA	48	76
ESC_QA7620AA	714	318
ESC_QA7619AA	48	76
ESC_QA7618AA	522	701
ESC_QA7617AA	711	935
ESC_QA7616AA	1992	257
ESC_QA7615AA	4624	387
ESC_QA7614AA	58	198
ESC_QA7613AA	10	512
ESC_QA7612AA	847	257
ESC_QA7611AA	2521	257
ESC_QA7610AA	1204	961
ESC_QA7609AA	711	935
ESC_QA7608AA	1087	235
ESC_QA7607AA	10	512
ESC_QA7606AA	937	198
ESC_QA7605AA	95	738
ESC_QA7604AA	75	257
ESC_QA7603AA	23	156
ESC_QA7602AA	32	270
ESC_QA7601AA	278	924
ESC_QA7600AA	336	878
ESC_QA7599AA	155	175
ESC_QA7598AA	711	935
ESC_QA7597AA	1611	198
ESC_QA7596AA	6599	951
ESC_QA7595AA	137	270
ESC_QA7594AA	327	204
ESC_QA7593AA	847	257
ESC_QA7592AA	657	592
ESC_QA7591AA	442	257
ESC_QA7590AA	1727	257
ESC_QA7589AA	1280	359
ESC_QA7588AA	164	175
ESC_QA7587AA	6189	175
ESC_QA7586AA	540	286
ESC_QA7585AA	940	257
ESC_QA7584AA	603	198
ESC_QA7583AA	164	175
ESC_QA7582AA	765	156
ESC_QA7581AA	2280	212

ESC_QA7580AA	548	512
ESC_QA7579AA	58	198
ESC_QA7578AA	1131	936
ESC_QA7577AA	2539	257
ESC_QA7576AA	58	198
ESC_QA7575AA	216	512
ESC_QA7574AA	4086	842
ESC_QA7573AA	540	286
ESC_QA7572AA	297	234
ESC_QA7571AA	297	234
ESC_QA7570AA	457	595
ESC_QA7568AA	5340	826
ESC_QA7567AA	5424	257
ESC_QA7566AA	641	935
ESC_QA7564AA	940	257
ESC_QA7563AA	940	257
ESC_QA7562AA	32	270
ESC_QA7561AA	58	198
ESC_QA7560AA	10	512
ESC_QA7559AA	10831	936
ESC_QA7558AA	10834	598
ESC_QA7557AA	10830	789
ESC_QA7556AA	10832	204
ESC_QA7555AA	10833	198
ESC_QA7554AA	10828	51
ESC_QA7553AA	10828	51
ESC_QA7552AA	10828	51
ESC_QA7551AA	2715	588
ESC_QA7550AA	10829	516
ESC_QA7549AA	10828	51
ESC_QA7548AA	10827	198
ESC_QA7547AA	344	355
ESC_QA7546AA	329	355
ESC_QA7544AA	48	76
ESC_QA7543AA	101	696
ESC_QA7542AA	88	160
ESC_QA7541AA	117	592
ESC_QA7540AA	515	1004
ESC_QA7539AA	245	261
ESC_QA7519AA	428	654
ESC_QA7518AA	11	936
ESC_QA7517AA	11	936
ESC_QA7516AA	11	936
ESC_QA7515AA	11	936
ESC_QA7514AA	11	936
ESC_QA7513AA	5430	756
ESC_QA7512AA	69	387
ESC_QA7511AA	12	626
ESC_QA7508AA	127	738
ESC_QA7506AA	4219	630

ESC_QA7504AA	73	737
ESC_QA7503AA	131	653
ESC_QA7502AA	73	737
ESC_QA7500AA	73	737
ESC_QA7498AA	95	738
ESC_QA7497AA	80	731
ESC_QA7496AA	127	738
ESC_QA7493AA	420	741
ESC_QA7492AA	343	286
ESC_QA7491AA	21	156
ESC_QA7490AA	1196	257
ESC_QA7489AA	1196	257
ESC_QA7486AA	58	198
ESC_QA7462AA	130	387
ESC_QA7461AA	602	257
ESC_QA7460AA	6707	445
ESC_QA7459AA	86	935
ESC_QA7458AA	420	741
ESC_QA7457AA	10	512
ESC_QA7456AA	155	175
ESC_QA7455AA	1011	198
ESC_QA7454AA	58	198
ESC_QA7453AA	224	198
ESC_QA7452AA	156	175
ESC_QA7451AA	156	175
ESC_QA7450AA	48	76
ESC_QA7449AA	10825	512
ESC_QA7448AA	48	76
ESC_QA7447AA	1011	198
ESC_QA7446AA	226	512
ESC_QA7445AA	224	198
ESC_QA7444AA	226	512
ESC_QA7443AA	10	512
ESC_QA7442AA	43	512
ESC_QA7441AA	617	511
ESC_QA7440AA	167	512
ESC_QA7439AA	48	76
ESC_QA7438AA	131	653
ESC_QA7437AA	48	76
ESC_QA7436AA	38	669
ESC_QA7435AA	167	512
ESC_QA7434AA	457	595
ESC_QA7433AA	997	915
ESC_QA7432AA	4553	408
ESC_QA7431AA	648	359
ESC_QA7430AA	648	359
ESC_QA7429AA	6	445
ESC_QA7428AA	12	626
ESC_QA7427AA	127	738
ESC_QA7426AA	155	175

ESC_QA7425AA	90	156
ESC_QA7424AA	7892	951
ESC_QA7423AA	2178	924
ESC_QA7422AA	744	512
ESC_QA7421AA	12	626
ESC_QA7420AA	372	424
ESC_QA7419AA	10	512
ESC_QA7418AA	165	504
ESC_QA7417AA	90	156
ESC_QA7416AA	108	257
ESC_QA7415AA	10	512
ESC_QA7414AA	48	76
ESC_QA7413AA	38	669
ESC_QA7412AA	38	669
ESC_QA7411AA	111	175
ESC_QA7410AA	206	286
ESC_QA7409AA	398	445
ESC_QA7408AA	23	156
ESC_QA7407AA	662	470
ESC_QA7406AA	58	198
ESC_QA7405AA	662	470
ESC_QA7404AA	540	286
ESC_QA7403AA	10	512
ESC_QA7402AA	58	198
ESC_QA7401AA	327	204
ESC_QA7400AA	135	653
ESC_QA7399AA	10	512
ESC_QA7398AA	685	311
ESC_QA7397AA	548	512
ESC_QA7396AA	10	512
ESC_QA7395AA	58	198
ESC_QA7392AA	152	257
ESC_QA7390AA	245	261
ESC_QA7388AA	152	257
ESC_QA7387AA	245	261
ESC_QA7386AA	245	261
ESC_QA7385AA	152	257
ESC_QA7383AA	152	257
ESC_QA7382AA	152	257
ESC_QA7381AA	245	261
ESC_QA7380AA	245	261
ESC_QA7379AA	10824	235
ESC_QA7378AA	101	696
ESC_QA7377AA	131	653
ESC_QA7376AA	10	512
ESC_QA7375AA	10	512
ESC_QA7374AA	58	198
ESC_QA7373AA	8076	504
ESC_QA7372AA	10	512
ESC_QA7371AA	1611	198

ESC_QA7370AA	10	512
ESC_QA7369AA	1121	198
ESC_QA7368AA	1121	198
ESC_QA7367AA	548	512
ESC_QA7366AA	88	160
ESC_QA7365AA	10	512
ESC_QA7364AA	295	160
ESC_QA7363AA	1091	512
ESC_QA7362AA	1307	257
ESC_QA7361AA	69	387
ESC_QA7360AA	2325	447
ESC_QA7359AA	69	387
ESC_QA7358AA	69	387
ESC_QA7357AA	69	387
ESC_QA7356AA	69	387
ESC_QA7355AA	69	387
ESC_QA7354AA	602	257
ESC_QA7352AA	10822	286
ESC_QA7346AA	10820	844
ESC_QA7341AA	10820	844
ESC_QA7337AA	10821	141
ESC_QA7334AA	206	286
ESC_QA7333AA	1159	381
ESC_QA7332AA	1304	257
ESC_QA7331AA	155	175
ESC_QA7330AA	1112	504
ESC_QA7329AA	215	512
ESC_QA7328AA	998	644
ESC_QA7327AA	372	424
ESC_QA7326AA	679	257
ESC_QA7325AA	1248	156
ESC_QA7324AA	1421	447
ESC_QA7323AA	10818	257
ESC_QA7322AA	141	644
ESC_QA7321AA	10817	235
ESC_QA7320AA	446	257
ESC_QA7319AA	196	257
ESC_QA7318AA	10819	900
ESC_QA7317AA	297	234
ESC_QA7316AA	69	387
ESC_QA7315AA	10	512
ESC_QA7311AA	69	387
ESC_QA7310AA	69	387
ESC_QA7309AA	69	387
ESC_QA7308AA	69	387
ESC_QA7307AA	69	387
ESC_QA7306AA	69	387
ESC_QA7305AA	69	387
ESC_QA7304AA	69	387
ESC_QA7303AA	69	387

ESC_QA7302AA	69	387
ESC_QA7301AA	10816	424
ESC_QA7300AA	73	737
ESC_QA7299AA	73	737
ESC_QA7298AA	131	653
ESC_QA7297AA	58	198
ESC_QA7296AA	131	653
ESC_QA7295AA	131	653
ESC_QA7294AA	95	738
ESC_QA7293AA	73	737
ESC_QA7292AA	301	307
ESC_QA7291AA	10815	652
ESC_QA7290AA	58	198
ESC_QA7289AA	59	217
ESC_QA7288AA	517	924
ESC_QA7287AA	95	738
ESC_QA7286AA	2040	198
ESC_QA7285AA	58	198
ESC_QA7284AA	23	156
ESC_QA7283AA	69	387
ESC_QA7282AA	678	257
ESC_QA7281AA	131	653
ESC_QA7280AA	1193	761
ESC_QA7279AA	69	387
ESC_QA7278AA	93	71
ESC_QA7277AA	1193	761
ESC_QA7276AA	131	653
ESC_QA7275AA	1722	599
ESC_QA7274AA	14	761
ESC_QA7272AA	345	175
ESC_QA7271AA	152	257
ESC_QA7270AA	152	257
ESC_QA7269AA	245	261
ESC_QA7268AA	152	257
ESC_QA7267AA	152	257
ESC_QA7266AA	152	257
ESC_QA7265AA	245	261
ESC_QA7264AA	898	108
ESC_QA7263AA	201	257
ESC_QA7262AA	88	160
ESC_QA7261AA	135	653
ESC_QA7260AA	34	504
ESC_QA7259AA	10	512
ESC_QA7258AA	2602	257
ESC_QA7257AA	21	156
ESC_QA7256AA	29	156
ESC_QA7255AA	428	654
ESC_QA7254AA	57	50
ESC_QA7253AA	10	512
ESC_QA7252AA	101	696

ESC_QA7247AA	10806	764
ESC_QA7246AA	10805	198
ESC_QA7244AA	10810	406
ESC_QA7196AA	10805	198
ESC_QA7195AA	21	156
ESC_QA7194AA	29	156
ESC_QA7193AA	301	307
ESC_QA7192AA	301	307
ESC_QA7191AA	301	307
ESC_QA7190AA	224	198
ESC_QA7189AA	515	1004
ESC_QA7188AA	744	512
ESC_QA7187AA	10811	924
ESC_QA7186AA	38	669
ESC_QA7185AA	68	386
ESC_QA7184AA	665	79
ESC_QA7183AA	101	696
ESC_QA7182AA	117	592
ESC_QA7181AA	117	592
ESC_QA7180AA	131	653
ESC_QA7179AA	117	592
ESC_QA7178AA	665	79
ESC_QA7177AA	1594	71
ESC_QA7176AA	117	592
ESC_QA7175AA	23	156
ESC_QA7174AA	23	156
ESC_QA7173AA	533	198
ESC_QA7172AA	131	653
ESC_QA7171AA	3406	108
ESC_QA7170AA	10807	512
ESC_QA7169AA	162	924
ESC_QA7168AA	354	15
ESC_QA7167AA	117	592
ESC_QA7166AA	155	175
ESC_QA7165AA	10	512
ESC_QA7164AA	1137	512
ESC_QA7163AA	665	79
ESC_QA7162AA	38	669
ESC_QA7161AA	88	160
ESC_QA7160AA	4980	445
ESC_QA7159AA	56	198
ESC_QA7158AA	10813	512
ESC_QA7157AA	1594	71
ESC_QA7156AA	156	175
ESC_QA7155AA	23	156
ESC_QA7154AA	10811	924
ESC_QA7153AA	135	653
ESC_QA7152AA	38	669
ESC_QA7151AA	617	511
ESC_QA7150AA	345	175

ESC_QA7149AA	1730	234
ESC_QA7148AA	10808	160
ESC_QA7147AA	101	696
ESC_QA7146AA	10812	257
ESC_QA7145AA	665	79
ESC_QA7144AA	997	915
ESC_QA7143AA	770	587
ESC_QA7142AA	770	587
ESC_QA7141AA	189	504
ESC_QA7140AA	156	175
ESC_QA7139AA	23	156
ESC_QA7138AA	1246	198
ESC_QA7137AA	7322	175
ESC_QA7136AA	354	15
ESC_QA7135AA	10	512
ESC_QA7134AA	10	512
ESC_QA7133AA	48	76
ESC_QA7132AA	752	512
ESC_QA7131AA	4980	445
ESC_QA7130AA	752	512
ESC_QA7129AA	398	445
ESC_QA7128AA	752	512
ESC_QA7127AA	665	79
ESC_QA7126AA	10809	256
ESC_QA7125AA	10814	406
ESC_QA7124AA	7104	234
ESC_QA7123AA	371	50
ESC_QA7122AA	101	696
ESC_QA7121AA	93	71
ESC_QA7120AA	10	512
ESC_QA7119AA	1551	40
ESC_QA7118AA	4243	914
ESC_QA7117AA	354	15
ESC_QA7116AA	997	915
ESC_QA7115AA	38	669
ESC_QA7114AA	453	935
ESC_QA7113AA	48	76
ESC_QA7112AA	1158	387
ESC_QA7111AA	4980	445
ESC_QA7110AA	1621	815
ESC_QA7109AA	155	175
ESC_QA7108AA	770	587
ESC_QA7107AA	155	175
ESC_QA7106AA	154	257
ESC_QA7105AA	3994	512
ESC_QA7103AA	648	359
ESC_QA7102AA	10	512
ESC_QA7100AA	1304	257
ESC_QA7099AA	117	592
ESC_QA7098AA	744	512

ESC_QA7097AA	398	445
ESC_QA7096AA	453	935
ESC_QA7095AA	88	160
ESC_QA7094AA	58	198
ESC_QA7093AA	2197	511
ESC_QA7092AA	1147	198
ESC_QA7091AA	127	738
ESC_QA7069AA	200	198
ESC_QA7068AA	10	512
ESC_QA7067AA	152	257
ESC_QA7066AA	1025	261
ESC_QA7065AA	152	257
ESC_QA7064AA	245	261
ESC_QA7063AA	152	257
ESC_QA7062AA	152	257
ESC_QA7061AA	243	197
ESC_QA7060AA	145	256
ESC_QA7059AA	152	257
ESC_QA7058AA	148	191
ESC_QA7057AA	245	261
ESC_QA7056AA	152	257
ESC_QA7055AA	152	257
ESC_QA7054AA	152	257
ESC_QA7053AA	245	261
ESC_QA7052AA	245	261
ESC_QA7051AA	145	256
ESC_QA7050AA	245	261
ESC_QA7049AA	1751	102
ESC_QA7048AA	245	261
ESC_QA7047AA	245	261
ESC_QA7046AA	288	102
ESC_QA7045AA	152	257
ESC_QA7044AA	288	102
ESC_QA7043AA	245	261
ESC_QA7042AA	152	257
ESC_QA7041AA	243	197
ESC_QA7040AA	152	257
ESC_QA7039AA	245	261
ESC_QA7038AA	245	261
ESC_QA7037AA	152	257
ESC_QA7036AA	442	257
ESC_QA7035AA	661	92
ESC_QA7034AA	5822	696
ESC_QA7033AA	11	936
ESC_QA7032AA	17	191
ESC_QA7031AA	6130	268
ESC_QA7030AA	10804	738
ESC_QA7029AA	300	286
ESC_QA7028AA	11	936
ESC_QA7027AA	442	257

ESC_QA7026AA	21	156
ESC_QA7025AA	11	936
ESC_QA7024AA	17	191
ESC_QA7023AA	949	175
ESC_QA7022AA	678	257
ESC_QA7021AA	17	191
ESC_QA7020AA	131	653
ESC_QA7019AA	10803	234
ESC_QA7018AA	442	257
ESC_QA7017AA	21	156
ESC_QA7016AA	69	387
ESC_QA7015AA	6	445
ESC_QA7014AA	738	598
ESC_QA7013AA	25	234
ESC_QA7012AA	7641	738
ESC_QA7011AA	21	156
ESC_QA7010AA	25	234
ESC_QA7009AA	4213	251
ESC_QA7008AA	10802	257
ESC_QA7007AA	21	156
ESC_QA7006AA	144	740
ESC_QA7005AA	442	257
ESC_QA7004AA	29	156
ESC_QA7003AA	11	936
ESC_QA7002AA	5292	738
ESC_QA7001AA	33	198
ESC_QA7000AA	5419	175
ESC_QA6797AA	155	175
ESC_QA6796AA	8590	212
ESC_QA6795AA	744	512
ESC_QA6794AA	1718	269
ESC_QA6793AA	2522	198
ESC_QA6792AA	154	257
ESC_QA6791AA	1056	257
ESC_QA6790AA	5037	198
ESC_QA6789AA	2524	789
ESC_QA6788AA	224	198
ESC_QA6787AA	16	156
ESC_QA6786AA	152	257
ESC_QA6785AA	243	197
ESC_QA6784AA	245	261
ESC_QA6782AA	245	261
ESC_QA6781AA	243	197
ESC_QA6780AA	152	257
ESC_QA6779AA	245	261
ESC_QA6778AA	7384	257
ESC_QA6777AA	152	257
ESC_QA6776AA	152	257
ESC_QA6775AA	7384	257
ESC_QA6774AA	152	257

ESC_QA6773AA	152	257
ESC_QA6772AA	29	156
ESC_QA6771AA	2170	234
ESC_QA6770AA	1141	512
ESC_QA6768AA	131	653
ESC_QA6767AA	42	257
ESC_QA6766AA	567	740
ESC_QA6765AA	5706	160
ESC_QA6764AA	955	670
ESC_QA6763AA	1081	198
ESC_QA6762AA	10800	307
ESC_QA6761AA	351	268
ESC_QA6760AA	974	160
ESC_QA6759AA	38	669
ESC_QA6758AA	2607	198
ESC_QA6757AA	115	669
ESC_QA6756AA	399	198
ESC_QA6755AA	2531	198
ESC_QA6754AA	10	512
ESC_QA6753AA	16	156
ESC_QA6752AA	245	261
ESC_QA6751AA	152	257
ESC_QA6750AA	245	261
ESC_QA6749AA	152	257
ESC_QA6748AA	152	257
ESC_QA6747AA	245	261
ESC_QA6746AA	245	261
ESC_QA6745AA	152	257
ESC_QA6744AA	152	257
ESC_QA6743AA	152	257
ESC_QA6742AA	245	261
ESC_QA6741AA	152	257
ESC_QA6740AA	245	261
ESC_QA6739AA	152	257
ESC_QA6738AA	152	257
ESC_QA6737AA	152	257
ESC_QA6736AA	1025	261
ESC_QA6735AA	9148	288
ESC_QA6734AA	152	257
ESC_QA6733AA	245	261
ESC_QA6732AA	245	261
ESC_QA6731AA	145	256
ESC_QA6730AA	245	261
ESC_QA6729AA	311	204
ESC_QA6728AA	245	261
ESC_QA6727AA	245	261
ESC_QA6726AA	245	261
ESC_QA6725AA	245	261
ESC_QA6724AA	152	257
ESC_QA6723AA	152	257

ESC_QA6722AA	7375	197
ESC_QA6721AA	152	257
ESC_QA6720AA	148	191
ESC_QA6719AA	152	257
ESC_QA6717AA	245	261
ESC_QA6716AA	245	261
ESC_QA6702AA	43	512
ESC_QA6701AA	11	936
ESC_QA6700AA	152	257
ESC_QA6699AA	245	261
ESC_QA6698AA	152	257
ESC_QA6697AA	245	261
ESC_QA6696AA	245	261
ESC_QA6695AA	245	261
ESC_QA6694AA	245	261
ESC_QA6693AA	245	261
ESC_QA6692AA	245	261
ESC_QA6691AA	29	156
ESC_QA6690AA	11	936
ESC_QA6689AA	34	504
ESC_QA6688AA	29	156
ESC_QA6687AA	504	738
ESC_QA6686AA	152	257
ESC_QA6685AA	152	257
ESC_QA6684AA	152	257
ESC_QA6683AA	145	256
ESC_QA6682AA	152	257
ESC_QA6681AA	152	257
ESC_QA6680AA	152	257
ESC_QA6679AA	152	257
ESC_QA6678AA	152	257
ESC_QA6677AA	152	257
ESC_QA6676AA	152	257
ESC_QA6675AA	152	257
ESC_QA6674AA	152	257
ESC_QA6673AA	5517	257
ESC_QA6672AA	152	257
ESC_QA6671AA	152	257
ESC_QA6670AA	152	257
ESC_QA6669AA	152	257
ESC_QA6668AA	152	257
ESC_QA6667AA	152	257
ESC_QA6666AA	152	257
ESC_QA6665AA	152	257
ESC_QA6664AA	152	257
ESC_QA6663AA	152	257
ESC_QA6662AA	152	257
ESC_QA6661AA	152	257
ESC_QA6660AA	152	257
ESC_QA6659AA	152	257

ESC_QA6658AA	152	257
ESC_QA6657AA	152	257
ESC_QA6656AA	152	257
ESC_QA6655AA	152	257
ESC_QA6654AA	152	257
ESC_QA6653AA	152	257
ESC_QA6652AA	152	257
ESC_QA6651AA	148	191
ESC_QA6650AA	152	257
ESC_QA6649AA	152	257
ESC_QA6648AA	245	261
ESC_QA6647AA	131	653
ESC_QA6646AA	68	386
ESC_QA6645AA	68	386
ESC_QA6644AA	38	669
ESC_QA6643AA	90	156
ESC_QA6642AA	48	76
ESC_QA6641AA	2496	789
ESC_QA6640AA	11	936
ESC_QA6639AA	8820	234
ESC_QA6638AA	10797	198
ESC_QA6637AA	8927	387
ESC_QA6636AA	54	256
ESC_QA6635AA	6188	257
ESC_QA6634AA	372	424
ESC_QA6633AA	1276	241
ESC_QA6632AA	349	670
ESC_QA6631AA	101	696
ESC_QA6630AA	681	393
ESC_QA6629AA	245	261
ESC_QA6628AA	152	257
ESC_QA6627AA	152	257
ESC_QA6626AA	148	191
ESC_QA6625AA	152	257
ESC_QA6624AA	148	191
ESC_QA6620AA	152	257
ESC_QA6619AA	152	257
ESC_QA6618AA	152	257
ESC_QA6617AA	152	257
ESC_QA6616AA	152	257
ESC_QA6613AA	152	257
ESC_QA6612AA	10792	261
ESC_QA6611AA	10663	261
ESC_QA6610AA	152	257
ESC_QA6609AA	152	257
ESC_QA6608AA	1502	257
ESC_QA6607AA	10790	261
ESC_QA6606AA	152	257
ESC_QA6605AA	243	197
ESC_QA6604AA	152	257

ESC_QA6603AA	152	257
ESC_QA6602AA	145	256
ESC_QA6601AA	8878	261
ESC_QA6600AA	152	257
ESC_QA6599AA	152	257
ESC_QA6598AA	152	257
ESC_QA6597AA	6918	260
ESC_QA6596AA	10789	257
ESC_QA6595AA	152	257
ESC_QA6594AA	245	261
ESC_QA6593AA	2426	261
ESC_QA6592AA	152	257
ESC_QA6591AA	245	261
ESC_QA6590AA	152	257
ESC_QA6589AA	152	257
ESC_QA6588AA	152	257
ESC_QA6587AA	152	257
ESC_QA6586AA	8292	256
ESC_QA6585AA	152	257
ESC_QA6584AA	152	257
ESC_QA6583AA	152	257
ESC_QA6582AA	152	257
ESC_QA6581AA	245	261
ESC_QA6580AA	245	261
ESC_QA6579AA	245	261
ESC_QA6578AA	152	257
ESC_QA6577AA	1025	261
ESC_QA6576AA	152	257
ESC_QA6575AA	1025	261
ESC_QA6574AA	152	257
ESC_QA6573AA	152	257
ESC_QA6572AA	1025	261
ESC_QA6571AA	152	257
ESC_QA6570AA	152	257
ESC_QA6569AA	152	257
ESC_QA6568AA	245	261
ESC_QA6567AA	152	257
ESC_QA6566AA	245	261
ESC_QA6565AA	245	261
ESC_QA6564AA	245	261
ESC_QA6563AA	152	257
ESC_QA6562AA	152	257
ESC_QA6561AA	152	257
ESC_QA6560AA	245	261
ESC_QA6559AA	152	257
ESC_QA6558AA	245	261
ESC_QA6557AA	245	261
ESC_QA6556AA	245	261
ESC_QA6555AA	152	257
ESC_QA6554AA	245	261

ESC_QA6553AA	152	257
ESC_QA6552AA	245	261
ESC_QA6551AA	152	257
ESC_QA6550AA	245	261
ESC_QA6549AA	245	261
ESC_QA6548AA	152	257
ESC_QA6547AA	152	257
ESC_QA6546AA	245	261
ESC_QA6545AA	245	261
ESC_QA6544AA	152	257
ESC_QA6543AA	245	261
ESC_QA6542AA	152	257
ESC_QA6541AA	1025	261
ESC_QA6540AA	245	261
ESC_QA6539AA	152	257
ESC_QA6538AA	1025	261
ESC_QA6537AA	152	257
ESC_QA6536AA	1025	261
ESC_QA6535AA	245	261
ESC_QA6534AA	245	261
ESC_QA6533AA	1025	261
ESC_QA6532AA	152	257
ESC_QA6531AA	152	257
ESC_QA6530AA	245	261
ESC_QA6529AA	1025	261
ESC_QA6528AA	245	261
ESC_QA6527AA	245	261
ESC_QA6526AA	1025	261
ESC_QA6525AA	245	261
ESC_QA6524AA	245	261
ESC_QA6523AA	245	261
ESC_QA6522AA	1025	261
ESC_QA6521AA	245	261
ESC_QA6520AA	245	261
ESC_QA6519AA	1025	261
ESC_QA6518AA	152	257
ESC_QA6517AA	245	261
ESC_QA6516AA	245	261
ESC_QA6515AA	245	261
ESC_QA6514AA	152	257
ESC_QA6513AA	245	261
ESC_QA6512AA	1025	261
ESC_QA6511AA	245	261
ESC_QA6510AA	245	261
ESC_QA6509AA	245	261
ESC_QA6508AA	245	261
ESC_QA6507AA	245	261
ESC_QA6506AA	245	261
ESC_QA6505AA	245	261
ESC_QA6504AA	6	445

ESC_QA6503AA	270	204
ESC_QA6501AA	245	261
ESC_QA6500AA	152	257
ESC_QA6499AA	152	257
ESC_QA6498AA	152	257
ESC_QA6497AA	152	257
ESC_QA6496AA	243	197
ESC_QA6495AA	152	257
ESC_QA6494AA	152	257
ESC_QA6493AA	152	257
ESC_QA6492AA	152	257
ESC_QA6491AA	152	257
ESC_QA6490AA	152	257
ESC_QA6489AA	152	257
ESC_QA6488AA	152	257
ESC_QA6487AA	245	261
ESC_QA6486AA	245	261
ESC_QA6485AA	245	261
ESC_QA6484AA	245	261
ESC_QA6483AA	245	261
ESC_QA6482AA	245	261
ESC_QA6481AA	243	197
ESC_QA6480AA	1025	261
ESC_QA6479AA	1513	256
ESC_QA6478AA	245	261
ESC_QA6477AA	245	261
ESC_QA6476AA	245	261
ESC_QA6475AA	245	261
ESC_QA6474AA	152	257
ESC_QA6473AA	152	257
ESC_QA6472AA	152	257
ESC_QA6471AA	152	257
ESC_QA6470AA	152	257
ESC_QA6469AA	152	257
ESC_QA6468AA	152	257
ESC_QA6467AA	152	257
ESC_QA6466AA	152	257
ESC_QA6465AA	152	257
ESC_QA6464AA	152	257
ESC_QA6463AA	152	257
ESC_QA6462AA	1502	257
ESC_QA6461AA	152	257
ESC_QA6460AA	152	257
ESC_QA6459AA	152	257
ESC_QA6458AA	152	257
ESC_QA6457AA	152	257
ESC_QA6456AA	152	257
ESC_QA6455AA	152	257
ESC_QA6454AA	152	257
ESC_QA6453AA	152	257

ESC_QA6452AA	152	257
ESC_QA6451AA	152	257
ESC_QA6450AA	152	257
ESC_QA6449AA	152	257
ESC_QA6448AA	152	257
ESC_QA6447AA	152	257
ESC_QA6446AA	152	257
ESC_QA6445AA	152	257
ESC_QA6444AA	152	257
ESC_QA6443AA	152	257
ESC_QA6442AA	245	261
ESC_QA6441AA	152	257
ESC_QA6440AA	243	197
ESC_QA6439AA	243	197
ESC_QA6438AA	152	257
ESC_QA6437AA	152	257
ESC_QA6436AA	152	257
ESC_QA6435AA	245	261
ESC_QA6434AA	152	257
ESC_QA6433AA	152	257
ESC_QA6432AA	152	257
ESC_QA6431AA	152	257
ESC_QA6430AA	152	257
ESC_QA6429AA	152	257
ESC_QA6428AA	152	257
ESC_QA6427AA	152	257
ESC_QA6426AA	152	257
ESC_QA6425AA	127	738
ESC_QA6424AA	10	512
ESC_QA6423AA	69	387
ESC_QA6422AA	744	512
ESC_QA6421AA	744	512
ESC_QA6420AA	91	629
ESC_QA6419AA	48	76
ESC_QA6418AA	48	76
ESC_QA6416AA	131	653
ESC_QA6415AA	131	653
ESC_QA6414AA	2448	234
ESC_QA6411AA	131	653
ESC_QA6410AA	12	626
ESC_QA6409AA	12	626
ESC_QA6408AA	38	669
ESC_QA6406AA	156	175
ESC_QA6405AA	80	731
ESC_QA6404AA	11	936
ESC_QA6403AA	131	653
ESC_QA6402AA	131	653
ESC_QA6401AA	131	653
ESC_QA6400AA	131	653
ESC_QA6399AA	131	653

ESC_QA6355AA	218	519
ESC_QA6354AA	23	156
ESC_QA6353AA	117	592
ESC_QA6352AA	665	79
ESC_QA6351AA	206	286
ESC_QA6350AA	88	160
ESC_QA6349AA	206	286
ESC_QA6348AA	2345	795
ESC_QA6347AA	48	76
ESC_QA6346AA	215	512
ESC_QA6345AA	1998	11
ESC_QA6344AA	1800	924
ESC_QA6343AA	1998	11
ESC_QA6342AA	1998	11
ESC_QA6341AA	48	76
ESC_QA6340AA	48	76
ESC_QA6339AA	48	76
ESC_QA6338AA	1800	924
ESC_QA6337AA	10430	71
ESC_QA6336AA	48	76
ESC_QA6335AA	1998	11
ESC_QA6334AA	155	175
ESC_QA6333AA	361	512
ESC_QA6332AA	155	175
ESC_QA6331AA	710	291
ESC_QA6330AA	607	355
ESC_QA6329AA	929	742
ESC_QA6328AA	48	76
ESC_QA6327AA	88	160
ESC_QA6326AA	58	198
ESC_QA6325AA	131	653
ESC_QA6324AA	131	653
ESC_QA6323AA	131	653
ESC_QA6322AA	131	653
ESC_QA6321AA	131	653
ESC_QA6320AA	131	653
ESC_QA6319AA	131	653
ESC_QA6318AA	131	653
ESC_QA6317AA	10787	198
ESC_QA6316AA	131	653
ESC_QA6315AA	86	935
ESC_QA6314AA	1250	926
ESC_QA6313AA	3168	261
ESC_QA6312AA	1434	512
ESC_QA6311AA	1079	235
ESC_QA6310AA	10786	875
ESC_QA6309AA	536	141
ESC_QA6308AA	152	257
ESC_QA6306AA	9084	198
ESC_QA6305AA	10	512

ESC_QA6304AA	10	512
ESC_QA6303AA	953	447
ESC_QA6302AA	93	71
ESC_QA6301AA	23	156
ESC_QA6300AA	90	156
ESC_QA6299AA	42	257
ESC_QA6298AA	69	387
ESC_QA6297AA	6257	156
ESC_QA6296AA	100	504
ESC_QA6295AA	100	504
ESC_QA6294AA	42	257
ESC_QA6293AA	10	512
ESC_QA6292AA	10793	512
ESC_QA6291AA	90	156
ESC_QA6290AA	90	156
ESC_QA6289AA	6234	257
ESC_QA6288AA	641	935
ESC_QA6287AA	5873	76
ESC_QA6286AA	998	644
ESC_QA6285AA	641	935
ESC_QA6284AA	10	512
ESC_QA6283AA	90	156
ESC_QA6282AA	23	156
ESC_QA6281AA	90	156
ESC_QA6280AA	23	156
ESC_QA6279AA	10	512
ESC_QA6278AA	398	445
ESC_QA6277AA	100	504
ESC_QA6276AA	6162	337
ESC_QA6275AA	5765	234
ESC_QA6274AA	2496	789
ESC_QA6273AA	4682	175
ESC_QA6272AA	641	935
ESC_QA6271AA	42	257
ESC_QA6270AA	6584	609
ESC_QA6269AA	9967	257
ESC_QA6268AA	165	504
ESC_QA6267AA	1114	504
ESC_QA6266AA	23	156
ESC_QA6265AA	88	160
ESC_QA6264AA	3524	935
ESC_QA6263AA	10	512
ESC_QA6262AA	10	512
ESC_QA6261AA	38	669
ESC_QA6260AA	3580	257
ESC_QA6259AA	90	156
ESC_QA6258AA	10	512
ESC_QA6257AA	131	653
ESC_QA6256AA	131	653
ESC_QA6255AA	131	653

ESC_QA6254AA	131	653
ESC_QA6253AA	131	653
ESC_QA6252AA	131	653
ESC_QA6251AA	131	653
ESC_QA6250AA	131	653
ESC_QA6249AA	131	653
ESC_QA6248AA	10	512
ESC_QA6247AA	10	512
ESC_QA6246AA	10	512
ESC_QA6245AA	131	653
ESC_QA6244AA	131	653
ESC_QA6243AA	131	653
ESC_QA6242AA	93	71
ESC_QA6241AA	6448	175
ESC_QA6240AA	2536	261
ESC_QA6239AA	90	156
ESC_QA6238AA	2522	198
ESC_QA6237AA	162	924
ESC_QA6236AA	10	512
ESC_QA6235AA	744	512
ESC_QA6234AA	361	512
ESC_QA6233AA	5727	257
ESC_QA6232AA	361	512
ESC_QA6230AA	361	512
ESC_QA6229AA	224	198
ESC_QA6228AA	398	445
ESC_QA6227AA	5727	257
ESC_QA6223AA	131	653
ESC_QA6222AA	131	653
ESC_QA6221AA	131	653
ESC_QA6220AA	131	653
ESC_QA6219AA	131	653
ESC_QA6218AA	152	257
ESC_QA6217AA	152	257
ESC_QA6216AA	152	257
ESC_QA6215AA	7384	257
ESC_QA6214AA	6200	261
ESC_QA6213AA	152	257
ESC_QA6212AA	245	261
ESC_QA6211AA	245	261
ESC_QA6210AA	245	261
ESC_QA6209AA	152	257
ESC_QA6208AA	245	261
ESC_QA6207AA	152	257
ESC_QA6206AA	245	261
ESC_QA6205AA	10769	257
ESC_QA6204AA	152	257
ESC_QA6203AA	245	261
ESC_QA6202AA	245	261
ESC_QA6201AA	152	257

ESC_QA6200AA	152	257
ESC_QA6199AA	152	257
ESC_QA6198AA	245	261
ESC_QA6197AA	152	257
ESC_QA6196AA	152	257
ESC_QA6195AA	148	191
ESC_QA6194AA	152	257
ESC_QA6193AA	152	257
ESC_QA6192AA	152	257
ESC_QA6191AA	245	261
ESC_QA6190AA	245	261
ESC_QA6189AA	152	257
ESC_QA6188AA	245	261
ESC_QA6187AA	11	936
ESC_QA6186AA	12	626
ESC_QA6185AA	11	936
ESC_QA6184AA	398	445
ESC_QA6183AA	1585	512
ESC_QA6182AA	1152	850
ESC_QA6181AA	398	445
ESC_QA6180AA	361	512
ESC_QA6178AA	398	445
ESC_QA6177AA	1585	512
ESC_QA6176AA	398	445
ESC_QA6175AA	1585	512
ESC_QA6174AA	10	512
ESC_QA6173AA	398	445
ESC_QA6172AA	398	445
ESC_QA6171AA	165	504
ESC_QA6170AA	361	512
ESC_QA6169AA	1585	512
ESC_QA6168AA	1585	512
ESC_QA6167AA	5416	445
ESC_QA6166AA	2325	447
ESC_QA6165AA	155	175
ESC_QA6164AA	2325	447
ESC_QA6163AA	361	512
ESC_QA6162AA	361	512
ESC_QA6161AA	73	737
ESC_QA6160AA	10	512
ESC_QA6159AA	569	738
ESC_QA6158AA	3232	198
ESC_QA6157AA	355	764
ESC_QA6156AA	10	512
ESC_QA6155AA	162	924
ESC_QA6154AA	589	650
ESC_QA6153AA	589	650
ESC_QA6152AA	115	669
ESC_QA6151AA	648	359
ESC_QA6150AA	569	738

ESC_QA6149AA	1084	180
ESC_QA6148AA	131	653
ESC_QA6147AA	224	198
ESC_QA6146AA	646	738
ESC_QA6145AA	1115	263
ESC_QA6144AA	73	737
ESC_QA6143AA	10	512
ESC_QA6142AA	10	512
ESC_QA6141AA	73	737
ESC_QA6140AA	33	198
ESC_QA6139AA	17	191
ESC_QA6138AA	33	198
ESC_QA6137AA	21	156
ESC_QA6136AA	10785	758
ESC_QA6135AA	4748	234
ESC_QA6134AA	131	653
ESC_QA6133AA	1819	808
ESC_QA6132AA	4942	36
ESC_QA6131AA	348	175
ESC_QA6130AA	442	257
ESC_QA6086AA	245	261
ESC_QA6085AA	245	261
ESC_QA6084AA	8271	928
ESC_QA6083AA	1079	235
ESC_QA6082AA	1850	269
ESC_QA6081AA	212	198
ESC_QA6080AA	10	512
ESC_QA6079AA	26	257
ESC_QA6078AA	2521	257
ESC_QA6077AA	2354	257
ESC_QA6076AA	428	654
ESC_QA6075AA	542	824
ESC_QA6074AA	3601	653
ESC_QA6073AA	69	387
ESC_QA6072AA	135	653
ESC_QA6071AA	1148	257
ESC_QA6070AA	2144	257
ESC_QA6069AA	10781	537
ESC_QA6068AA	1049	175
ESC_QA6067AA	589	650
ESC_QA6066AA	1083	203
ESC_QA6065AA	1246	198
ESC_QA6064AA	5073	900
ESC_QA6063AA	720	900
ESC_QA6062AA	8501	108
ESC_QA6061AA	2541	878
ESC_QA6060AA	46	447
ESC_QA6059AA	720	900
ESC_QA6058AA	10788	515
ESC_QA6057AA	69	387

ESC_QA6056AA	2079	198
ESC_QA6055AA	117	592
ESC_QA6054AA	1079	235
ESC_QA6053AA	13	257
ESC_QA6052AA	136	392
ESC_QA6051AA	130	387
ESC_QA6050AA	10782	748
ESC_QA6049AA	80	731
ESC_QA6048AA	10780	452
ESC_QA6047AA	423	204
ESC_QA6046AA	10431	227
ESC_QA6045AA	711	935
ESC_QA6044AA	91	629
ESC_QA6043AA	162	924
ESC_QA6042AA	10	512
ESC_QA6041AA	1656	175
ESC_QA6040AA	737	175
ESC_QA6039AA	58	198
ESC_QA6038AA	942	198
ESC_QA6037AA	4038	257
ESC_QA6036AA	40	198
ESC_QA6035AA	540	286
ESC_QA6034AA	297	234
ESC_QA6033AA	3580	257
ESC_QA6032AA	3580	257
ESC_QA6031AA	69	387
ESC_QA6030AA	10778	198
ESC_QA6029AA	1147	198
ESC_QA6028AA	1236	380
ESC_QA6027AA	399	198
ESC_QA6026AA	345	175
ESC_QA6025AA	154	257
ESC_QA6024AA	1716	512
ESC_QA6023AA	101	696
ESC_QA6022AA	117	592
ESC_QA6021AA	1308	257
ESC_QA6020AA	11	936
ESC_QA6019AA	11	936
ESC_QA6018AA	11	936
ESC_QA6017AA	11	936
ESC_QA6016AA	11	936
ESC_QA6015AA	11	936
ESC_QA6014AA	11	936
ESC_QA6013AA	11	936
ESC_QA6012AA	11	936
ESC_QA6011AA	11	936
ESC_QA6010AA	11	936
ESC_QA6009AA	11	936
ESC_QA6008AA	11	936
ESC_QA6007AA	11	936

ESC_QA6006AA	11	936
ESC_QA6005AA	10	512
ESC_QA6004AA	1196	257
ESC_QA6003AA	10779	496
ESC_QA6002AA	58	198
ESC_QA6001AA	5876	847
ESC_QA5999AA	10738	50
ESC_QA5998AA	10740	799
ESC_QA5997AA	10702	410
ESC_QA5996AA	10738	50
ESC_QA5995AA	9862	156
ESC_QA5994AA	10729	654
ESC_QA5993AA	10739	50
ESC_QA5992AA	10721	696
ESC_QA5991AA	10729	654
ESC_QA5990AA	10721	696
ESC_QA5989AA	10733	653
ESC_QA5987AA	9862	156
ESC_QA5986AA	10735	257
ESC_QA5985AA	10736	198
ESC_QA5984AA	10734	956
ESC_QA5983AA	10732	592
ESC_QA5982AA	10730	175
ESC_QA5981AA	10731	198
ESC_QA5979AA	10728	126
ESC_QA5978AA	10727	385
ESC_QA5977AA	10729	654
ESC_QA5976AA	10726	234
ESC_QA5975AA	10721	696
ESC_QA5974AA	10725	935
ESC_QA5971AA	10723	198
ESC_QA5970AA	10722	900
ESC_QA5969AA	10721	696
ESC_QA5968AA	10724	630
ESC_QA5967AA	10721	696
ESC_QA5965AA	10720	653
ESC_QA5964AA	11	936
ESC_QA5963AA	11	936
ESC_QA5962AA	8825	304
ESC_QA5961AA	11	936
ESC_QA5960AA	11	936
ESC_QA5959AA	11	936
ESC_QA5958AA	11	936
ESC_QA5957AA	152	257
ESC_QA5956AA	11	936
ESC_QA5955AA	11	936
ESC_QA5954AA	11	936
ESC_QA5953AA	11	936
ESC_QA5952AA	17	191
ESC_QA5951AA	29	156

ESC_QA5950AA	29	156
ESC_QA5949AA	11	936
ESC_QA5948AA	137	270
ESC_QA5947AA	16	156
ESC_QA5946AA	11	936
ESC_QA5945AA	11	936
ESC_QA5944AA	11	936
ESC_QA5943AA	359	696
ESC_QA5942AA	58	198
ESC_QA5941AA	1495	311
ESC_QA5940AA	1495	311
ESC_QA5939AA	90	156
ESC_QA5938AA	131	653
ESC_QA5935AA	57	50
ESC_QA5934AA	1011	198
ESC_QA5933AA	69	387
ESC_QA5932AA	607	355
ESC_QA5931AA	10292	512
ESC_QA5930AA	10	512
ESC_QA5929AA	10774	512
ESC_QA5928AA	1140	934
ESC_QA5927AA	162	924
ESC_QA5925AA	10	512
ESC_QA5924AA	2936	515
ESC_QA5922AA	1638	519
ESC_QA5918AA	6856	76
ESC_QA5917AA	155	175
ESC_QA5916AA	7458	824
ESC_QA5914AA	1140	934
ESC_QA5913AA	1589	156
ESC_QA5912AA	48	76
ESC_QA5911AA	101	696
ESC_QA5908AA	602	257
ESC_QA5907AA	10773	291
ESC_QA5906AA	10773	291
ESC_QA5905AA	93	71
ESC_QA5904AA	540	286
ESC_QA5903AA	10772	204
ESC_QA5900AA	10	512
ESC_QA5899AA	1788	785
ESC_QA5898AA	871	447
ESC_QA5897AA	1638	519
ESC_QA5896AA	48	76
ESC_QA5895AA	1638	519
ESC_QA5894AA	1638	519
ESC_QA5893AA	641	935
ESC_QA5892AA	10776	935
ESC_QA5891AA	641	935
ESC_QA5890AA	195	76
ESC_QA5889AA	10	512

ESC_QA5888AA	641	935
ESC_QA5887AA	48	76
ESC_QA5886AA	10775	198
ESC_QA5885AA	515	1004
ESC_QA5884AA	48	76
ESC_QA5883AA	10	512
ESC_QA5882AA	10	512
ESC_QA5881AA	48	76
ESC_QA5880AA	1638	519
ESC_QA5879AA	10671	52
ESC_QA5878AA	10777	824
ESC_QA5877AA	10777	824
ESC_QA5876AA	10777	824
ESC_QA5875AA	877	935
ESC_QA5874AA	115	669
ESC_QA5873AA	48	76
ESC_QA5872AA	1944	934
ESC_QA5871AA	1286	474
ESC_QA5870AA	355	764
ESC_QA5869AA	2280	212
ESC_QA5868AA	21	156
ESC_QA5867AA	58	198
ESC_QA5866AA	372	424
ESC_QA5865AA	73	737
ESC_QA5864AA	998	644
ESC_QA5863AA	2722	234
ESC_QA5862AA	162	924
ESC_QA5861AA	405	923
ESC_QA5860AA	73	737
ESC_QA5859AA	131	653
ESC_QA5858AA	295	160
ESC_QA5857AA	10	512
ESC_QA5856AA	2015	684
ESC_QA5855AA	2137	268
ESC_QA5854AA	372	424
ESC_QA5853AA	372	424
ESC_QA5852AA	34	504
ESC_QA5851AA	127	738
ESC_QA5850AA	2604	626
ESC_QA5849AA	117	592
ESC_QA5848AA	141	644
ESC_QA5847AA	73	737
ESC_QA5846AA	155	175
ESC_QA5845AA	372	424
ESC_QA5844AA	7900	430
ESC_QA5843AA	10771	257
ESC_QA5842AA	56	198
ESC_QA5841AA	616	156
ESC_QA5838AA	602	257
ESC_QA5837AA	8590	212

ESC_QA5835AA	10	512
ESC_QA5834AA	10770	268
ESC_QA5833AA	162	924
ESC_QA5832AA	4937	519
ESC_QA5831AA	2556	630
ESC_QA5830AA	349	670
ESC_QA5829AA	70	670
ESC_QA5828AA	12	626
ESC_QA5827AA	744	512
ESC_QA5826AA	372	424
ESC_QA5825AA	135	653
ESC_QA5824AA	372	424
ESC_QA5823AA	73	737
ESC_QA5822AA	7581	996
ESC_QA5821AA	372	424
ESC_QA5820AA	73	737
ESC_QA5819AA	162	924
ESC_QA5818AA	12	626
ESC_QA5817AA	73	737
ESC_QA5816AA	641	935
ESC_QA5815AA	162	924
ESC_QA5814AA	457	595
ESC_QA5813AA	5903	180
ESC_QA5812AA	4260	737
ESC_QA5811AA	648	359
ESC_QA5810AA	117	592
ESC_QA5809AA	69	387
ESC_QA5808AA	351	268
ESC_QA5807AA	10	512
ESC_QA5805AA	11	936
ESC_QA5804AA	32	270
ESC_QA5803AA	722	422
ESC_QA5802AA	145	256
ESC_QA5801AA	152	257
ESC_QA5800AA	245	261
ESC_QA5799AA	152	257
ESC_QA5798AA	273	102
ESC_QA5797AA	152	257
ESC_QA5796AA	152	257
ESC_QA5795AA	245	261
ESC_QA5794AA	152	257
ESC_QA5793AA	245	261
ESC_QA5792AA	243	197
ESC_QA5791AA	152	257
ESC_QA5790AA	245	261
ESC_QA5789AA	152	257
ESC_QA5788AA	245	261
ESC_QA5787AA	245	261
ESC_QA5786AA	245	261
ESC_QA5785AA	152	257

ESC_QA5784AA	152	257
ESC_QA5783AA	245	261
ESC_QA5782AA	245	261
ESC_QA5781AA	245	261
ESC_QA5780AA	152	257
ESC_QA5779AA	130	387
ESC_QA5778AA	4267	257
ESC_QA5777AA	10	512
ESC_QA5776AA	847	257
ESC_QA5775AA	1804	935
ESC_QA5774AA	28	537
ESC_QA5773AA	69	387
ESC_QA5772AA	11	936
ESC_QA5771AA	11	936
ESC_QA5770AA	21	156
ESC_QA5769AA	11	936
ESC_QA5768AA	11	936
ESC_QA5767AA	11	936
ESC_QA5766AA	2758	512
ESC_QA5765AA	7024	886
ESC_QA5764AA	21	156
ESC_QA5763AA	6	445
ESC_QA5762AA	34	504
ESC_QA5761AA	59	217
ESC_QA5760AA	11	936
ESC_QA5759AA	1312	76
ESC_QA5758AA	38	669
ESC_QA5756AA	362	234
ESC_QA5754AA	457	595
ESC_QA5753AA	6321	541
ESC_QA5752AA	354	15
ESC_QA5751AA	131	653
ESC_QA5750AA	648	359
ESC_QA5749AA	131	653
ESC_QA5748AA	69	387
ESC_QA5747AA	131	653
ESC_QA5746AA	73	737
ESC_QA5745AA	1193	761
ESC_QA5744AA	38	669
ESC_QA5743AA	636	653
ESC_QA5742AA	409	504
ESC_QA5741AA	73	737
ESC_QA5740AA	10	512
ESC_QA5739AA	973	968
ESC_QA5738AA	1444	768
ESC_QA5737AA	457	595
ESC_QA5736AA	405	923
ESC_QA5735AA	10767	653
ESC_QA5734AA	44	512
ESC_QA5733AA	10767	653

ESC_QA5732AA	131	653
ESC_QA5731AA	1914	956
ESC_QA5730AA	131	653
ESC_QA5729AA	2003	669
ESC_QA5728AA	38	669
ESC_QA5727AA	2015	684
ESC_QA5726AA	540	286
ESC_QA5725AA	131	653
ESC_QA5724AA	131	653
ESC_QA5723AA	162	924
ESC_QA5722AA	34	504
ESC_QA5721AA	7366	824
ESC_QA5720AA	624	359
ESC_QA5719AA	1868	764
ESC_QA5718AA	1193	761
ESC_QA5717AA	648	359
ESC_QA5715AA	354	15
ESC_QA5713AA	963	669
ESC_QA5712AA	12	626
ESC_QA5711AA	10795	504
ESC_QA5710AA	38	669
ESC_QA5709AA	349	670
ESC_QA5708AA	38	669
ESC_QA5707AA	1674	595
ESC_QA5706AA	38	669
ESC_QA5705AA	38	669
ESC_QA5704AA	69	387
ESC_QA5703AA	1193	761
ESC_QA5702AA	405	923
ESC_QA5701AA	963	669
ESC_QA5700AA	648	359
ESC_QA5699AA	95	738
ESC_QA5698AA	1431	257
ESC_QA5697AA	405	923
ESC_QA5696AA	457	595
ESC_QA5695AA	681	393
ESC_QA5694AA	38	669
ESC_QA5693AA	155	175
ESC_QA5692AA	38	669
ESC_QA5691AA	1722	599
ESC_QA5690AA	457	595
ESC_QA5689AA	354	15
ESC_QA5687AA	963	669
ESC_QA5686AA	69	387
ESC_QA5685AA	349	670
ESC_QA5684AA	127	738
ESC_QA5683AA	131	653
ESC_QA5682AA	91	629
ESC_QA5681AA	10768	359
ESC_QA5680AA	245	261

ESC_QA5679AA	10703	923
ESC_QA5678AA	152	257
ESC_QA5677AA	152	257
ESC_QA5676AA	152	257
ESC_QA5675AA	152	257
ESC_QA5674AA	152	257
ESC_QA5673AA	152	257
ESC_QA5672AA	245	261
ESC_QA5671AA	1753	261
ESC_QA5670AA	152	257
ESC_QA5669AA	8878	261
ESC_QA5668AA	152	257
ESC_QA5667AA	152	257
ESC_QA5666AA	152	257
ESC_QA5665AA	152	257
ESC_QA5664AA	152	257
ESC_QA5663AA	245	261
ESC_QA5662AA	245	261
ESC_QA5661AA	152	257
ESC_QA5659AA	152	257
ESC_QA5658AA	152	257
ESC_QA5657AA	245	261
ESC_QA5656AA	245	261
ESC_QA5655AA	10718	108
ESC_QA5654AA	10	512
ESC_QA5653AA	746	789
ESC_QA5652AA	131	653
ESC_QA5651AA	10	512
ESC_QA5650AA	10	512
ESC_QA5649AA	2772	257
ESC_QA5648AA	227	504
ESC_QA5647AA	1162	394
ESC_QA5646AA	10717	257
ESC_QA5645AA	919	421
ESC_QA5644AA	130	387
ESC_QA5643AA	2063	198
ESC_QA5642AA	919	421
ESC_QA5641AA	58	198
ESC_QA5640AA	227	504
ESC_QA5639AA	906	198
ESC_QA5638AA	631	204
ESC_QA5592AA	10	512
ESC_QA5591AA	10	512
ESC_QA5590AA	10	512
ESC_QA5589AA	10	512
ESC_QA5588AA	10	512
ESC_QA5587AA	10	512
ESC_QA5586AA	10	512
ESC_QA5585AA	10	512
ESC_QA5584AA	10	512

ESC_QA5583AA	10	512
ESC_QA5544AA	16	156
ESC_QA5543AA	1792	156
ESC_QA5542AA	342	286
ESC_QA5541AA	655	926
ESC_QA5539AA	10	512
ESC_QA5538AA	10	512
ESC_QA5537AA	10	512
ESC_QA5536AA	10	512
ESC_QA5535AA	10	512
ESC_QA5534AA	10	512
ESC_QA5533AA	10	512
ESC_QA5527AA	131	653
ESC_QA5526AA	131	653
ESC_QA5525AA	131	653
ESC_QA5524AA	131	653
ESC_QA5523AA	11	936
ESC_QA5522AA	8211	257
ESC_QA5521AA	342	286
ESC_QA5520AA	95	738
ESC_QA5519AA	144	740
ESC_QA5518AA	101	696
ESC_QA5517AA	847	257
ESC_QA5516AA	131	653
ESC_QA5515AA	17	191
ESC_QA5514AA	131	653
ESC_QA5513AA	343	286
ESC_QA5512AA	635	141
ESC_QA5511AA	10711	50
ESC_QA5510AA	95	738
ESC_QA5509AA	2836	221
ESC_QA5508AA	607	355
ESC_QA5507AA	21	156
ESC_QA5502AA	109	257
ESC_QA5501AA	501	900
ESC_QA5500AA	1706	156
ESC_QA5499AA	2325	447
ESC_QA5498AA	4532	234
ESC_QA5497AA	88	160
ESC_QA5496AA	162	924
ESC_QA5495AA	162	924
ESC_QA5494AA	2111	184
ESC_QA5493AA	155	175
ESC_QA5492AA	164	175
ESC_QA5491AA	10	512
ESC_QA5490AA	10	512
ESC_QA5489AA	4080	300
ESC_QA5488AA	1423	198
ESC_QA5487AA	162	924
ESC_QA5486AA	2111	184

ESC_QA5485AA	155	175
ESC_QA5484AA	164	175
ESC_QA5483AA	10	512
ESC_QA5482AA	10	512
ESC_QA5481AA	4080	300
ESC_QA5480AA	1423	198
ESC_QA5479AA	3188	257
ESC_QA5478AA	58	198
ESC_QA5477AA	398	445
ESC_QA5476AA	10713	961
ESC_QA5475AA	38	669
ESC_QA5474AA	1431	257
ESC_QA5473AA	10	512
ESC_QA5472AA	10	512
ESC_QA5471AA	9439	512
ESC_QA5470AA	10712	198
ESC_QA5469AA	1286	474
ESC_QA5468AA	6631	234
ESC_QA5467AA	4151	971
ESC_QA5466AA	1423	198
ESC_QA5465AA	1167	257
ESC_QA5464AA	101	696
ESC_QA5463AA	173	254
ESC_QA5462AA	58	198
ESC_QA5461AA	109	257
ESC_QA5460AA	772	76
ESC_QA5459AA	1992	257
ESC_QA5458AA	1007	268
ESC_QA5457AA	1011	198
ESC_QA5456AA	1167	257
ESC_QA5455AA	1167	257
ESC_QA5454AA	641	935
ESC_QA5453AA	772	76
ESC_QA5452AA	1992	257
ESC_QA5451AA	1007	268
ESC_QA5450AA	1011	198
ESC_QA5449AA	1167	257
ESC_QA5448AA	1167	257
ESC_QA5447AA	1125	198
ESC_QA5446AA	1642	198
ESC_QA5445AA	10715	512
ESC_QA5444AA	6422	198
ESC_QA5443AA	898	108
ESC_QA5442AA	10	512
ESC_QA5441AA	58	198
ESC_QA5440AA	4038	257
ESC_QA5439AA	6617	519
ESC_QA5438AA	10	512
ESC_QA5437AA	10	512
ESC_QA5436AA	10714	257

ESC_QA5435AA	162	924
ESC_QA5434AA	56	198
ESC_QA5433AA	3042	663
ESC_QA5432AA	56	198
ESC_QA5431AA	10	512
ESC_QA5430AA	772	76
ESC_QA5429AA	155	175
ESC_QA5428AA	10	512
ESC_QA5427AA	155	175
ESC_QA5426AA	1718	269
ESC_QA5425AA	57	50
ESC_QA5424AA	4080	300
ESC_QA5379AA	4995	198
ESC_QA5377AA	10	512
ESC_QA5376AA	542	824
ESC_QA5375AA	10	512
ESC_QA5373AA	4995	198
ESC_QA5372AA	3021	447
ESC_QA5371AA	95	738
ESC_QA5369AA	10	512
ESC_QA5368AA	10	512
ESC_QA5367AA	10	512
ESC_QA5366AA	1079	235
ESC_QA5365AA	127	738
ESC_QA5364AA	10	512
ESC_QA5363AA	127	738
ESC_QA5362AA	10	512
ESC_QA5361AA	10	512
ESC_QA5360AA	4995	198
ESC_QA5359AA	4995	198
ESC_QA5358AA	10	512
ESC_QA5357AA	4995	198
ESC_QA5356AA	127	738
ESC_QA5355AA	452	743
ESC_QA5354AA	10737	493
ESC_QA5353AA	10	512
ESC_QA5352AA	10	512
ESC_QA5351AA	10	512
ESC_QA5350AA	10	512
ESC_QA5349AA	10	512
ESC_QA5348AA	10	512
ESC_QA5347AA	10	512
ESC_QA5346AA	1079	235
ESC_QA5344AA	10	512
ESC_QA5343AA	452	743
ESC_QA5342AA	10	512
ESC_QA5341AA	1721	311
ESC_QA5340AA	1079	235
ESC_QA5339AA	8592	307
ESC_QA5338AA	127	738

ESC_QA5337AA	925	387
ESC_QA5336AA	10	512
ESC_QA5335AA	10	512
ESC_QA5333AA	73	737
ESC_QA5332AA	73	737
ESC_QA5331AA	73	737
ESC_QA5330AA	73	737
ESC_QA5329AA	73	737
ESC_QA5328AA	73	737
ESC_QA5327AA	73	737
ESC_QA5326AA	73	737
ESC_QA5325AA	73	737
ESC_QA5324AA	73	737
ESC_QA5323AA	73	737
ESC_QA5322AA	73	737
ESC_QA5321AA	73	737
ESC_QA5320AA	73	737
ESC_QA5319AA	73	737
ESC_QA5318AA	73	737
ESC_QA5317AA	73	737
ESC_QA5316AA	73	737
ESC_QA5315AA	73	737
ESC_QA5314AA	73	737
ESC_QA5313AA	73	737
ESC_QA5312AA	73	737
ESC_QA5311AA	73	737
ESC_QA5310AA	73	737
ESC_QA5309AA	73	737
ESC_QA5308AA	73	737
ESC_QA5307AA	73	737
ESC_QA5306AA	73	737
ESC_QA5305AA	73	737
ESC_QA5304AA	73	737
ESC_QA5303AA	73	737
ESC_QA5302AA	73	737
ESC_QA5301AA	73	737
ESC_QA5300AA	73	737
ESC_QA5299AA	10	512
ESC_QA5298AA	73	737
ESC_QA5295AA	10	512
ESC_QA5294AA	73	737
ESC_QA5289AA	131	653
ESC_QA5288AA	93	71
ESC_QA5287AA	93	71
ESC_QA5286AA	93	71
ESC_QA5285AA	93	71
ESC_QA5284AA	93	71
ESC_QA5283AA	93	71
ESC_QA5282AA	93	71
ESC_QA5281AA	93	71

ESC_QA5280AA	93	71
ESC_QA5279AA	93	71
ESC_QA5278AA	93	71
ESC_QA5277AA	93	71
ESC_QA5276AA	93	71
ESC_QA5275AA	93	71
ESC_QA5274AA	93	71
ESC_QA5273AA	93	71
ESC_QA5272AA	93	71
ESC_QA5271AA	93	71
ESC_QA5270AA	93	71
ESC_QA5268AA	93	71
ESC_QA5267AA	93	71
ESC_QA5266AA	93	71
ESC_QA5265AA	93	71
ESC_QA5264AA	93	71
ESC_QA5263AA	93	71
ESC_QA5262AA	93	71
ESC_QA5261AA	93	71
ESC_QA5260AA	10	512
ESC_QA5259AA	10	512
ESC_QA5258AA	10	512
ESC_QA5255AA	10	512
ESC_QA5252AA	10	512
ESC_QA5250AA	10	512
ESC_QA5249AA	10	512
ESC_QA5248AA	10	512
ESC_QA5247AA	10741	512
ESC_QA5246AA	10	512
ESC_QA5245AA	10	512
ESC_QA5244AA	10	512
ESC_QA5243AA	10	512
ESC_QA5242AA	10	512
ESC_QA5241AA	10	512
ESC_QA5240AA	10	512
ESC_QA5239AA	10	512
ESC_QA5238AA	10	512
ESC_QA5237AA	10	512
ESC_QA5236AA	10	512
ESC_QA5235AA	10	512
ESC_QA5234AA	10	512
ESC_QA5233AA	10	512
ESC_QA5232AA	10	512
ESC_QA5231AA	10	512
ESC_QA5230AA	10	512
ESC_QA5229AA	10	512
ESC_QA5228AA	10	512
ESC_QA5227AA	10	512
ESC_QA5226AA	10	512
ESC_QA5225AA	10	512

ESC_QA5224AA	10	512
ESC_QA5222AA	10	512
ESC_QA5221AA	10	512
ESC_QA5220AA	10	512
ESC_QA5219AA	10	512
ESC_QA5218AA	10	512
ESC_QA5217AA	10	512
ESC_QA5216AA	10	512
ESC_QA5215AA	10	512
ESC_QA5214AA	10	512
ESC_QA5213AA	10	512
ESC_QA5212AA	10	512
ESC_QA5211AA	10	512
ESC_QA5210AA	10	512
ESC_QA5209AA	10	512
ESC_QA5208AA	10	512
ESC_QA5207AA	10	512
ESC_QA5206AA	10	512
ESC_QA5205AA	10	512
ESC_QA5204AA	10	512
ESC_QA5203AA	10	512
ESC_QA5202AA	10	512
ESC_QA5201AA	10	512
ESC_QA5200AA	10	512
ESC_QA5199AA	10	512
ESC_QA5198AA	10	512
ESC_QA5196AA	10	512
ESC_QA5195AA	10	512
ESC_QA5193AA	10	512
ESC_QA5192AA	10	512
ESC_QA5191AA	10	512
ESC_QA5190AA	10	512
ESC_QA5189AA	10	512
ESC_QA5188AA	10	512
ESC_QA5187AA	10	512
ESC_QA5186AA	10	512
ESC_QA5185AA	10	512
ESC_QA5183AA	10	512
ESC_QA5181AA	10	512
ESC_QA5180AA	10	512
ESC_QA5179AA	10	512
ESC_QA5178AA	10	512
ESC_QA5177AA	10	512
ESC_QA5176AA	10	512
ESC_QA5175AA	10	512
ESC_QA5174AA	10	512
ESC_QA5173AA	10	512
ESC_QA5172AA	10	512
ESC_QA5170AA	10	512
ESC_QA5169AA	10	512

ESC_QA5168AA	10	512
ESC_QA5167AA	10	512
ESC_QA5166AA	10	512
ESC_QA5165AA	10	512
ESC_QA5164AA	10	512
ESC_QA5163AA	10	512
ESC_QA5162AA	10	512
ESC_QA5161AA	10	512
ESC_QA5160AA	10	512
ESC_QA5158AA	10	512
ESC_QA5157AA	10	512
ESC_QA5156AA	10	512
ESC_QA5155AA	10	512
ESC_QA5154AA	10	512
ESC_QA5153AA	10	512
ESC_QA5152AA	10	512
ESC_QA5150AA	10	512
ESC_QA5149AA	10	512
ESC_QA5147AA	10	512
ESC_QA5146AA	10	512
ESC_QA5145AA	10	512
ESC_QA5144AA	10	512
ESC_QA5143AA	10	512
ESC_QA5142AA	10	512
ESC_QA5141AA	10	512
ESC_QA5140AA	10	512
ESC_QA5139AA	10	512
ESC_QA5138AA	10	512
ESC_QA5137AA	10	512
ESC_QA5136AA	10	512
ESC_QA5135AA	10	512
ESC_QA5134AA	10	512
ESC_QA5133AA	10	512
ESC_QA5132AA	10	512
ESC_QA5131AA	10	512
ESC_QA5130AA	10	512
ESC_QA5129AA	10	512
ESC_QA5128AA	10	512
ESC_QA5127AA	10	512
ESC_QA5126AA	10	512
ESC_QA5125AA	10	512
ESC_QA5124AA	10	512
ESC_QA5123AA	10	512
ESC_QA5122AA	10	512
ESC_QA5121AA	10	512
ESC_QA5120AA	10	512
ESC_QA5119AA	10	512
ESC_QA5118AA	10	512
ESC_QA5117AA	10	512
ESC_QA5116AA	10	512

ESC_QA5115AA	10	512
ESC_QA5114AA	10	512
ESC_QA5113AA	10	512
ESC_QA5112AA	10	512
ESC_QA5111AA	10	512
ESC_QA5110AA	10	512
ESC_QA5109AA	10	512
ESC_QA5108AA	10	512
ESC_QA5107AA	10	512
ESC_QA5106AA	10	512
ESC_QA5105AA	10	512
ESC_QA5104AA	10	512
ESC_QA5103AA	10	512
ESC_QA5102AA	10	512
ESC_QA5101AA	10	512
ESC_QA5100AA	10	512
ESC_QA5099AA	10	512
ESC_QA5098AA	10	512
ESC_QA5097AA	10	512
ESC_QA5096AA	10	512
ESC_QA5095AA	10	512
ESC_QA5094AA	10	512
ESC_QA5093AA	10	512
ESC_QA5092AA	10	512
ESC_QA5091AA	10	512
ESC_QA5090AA	10742	524
ESC_QA5088AA	10	512
ESC_QA5086AA	10	512
ESC_QA5083AA	10	512
ESC_QA5082AA	10	512
ESC_QA5081AA	10	512
ESC_QA5080AA	10	512
ESC_QA5079AA	10	512
ESC_QA5078AA	10	512
ESC_QA5077AA	10	512
ESC_QA5076AA	10	512
ESC_QA5075AA	10	512
ESC_QA5074AA	10	512
ESC_QA5073AA	10	512
ESC_QA5071AA	10	512
ESC_QA5070AA	10	512
ESC_QA5069AA	10	512
ESC_QA5068AA	10	512
ESC_QA5067AA	10	512
ESC_QA5066AA	10	512
ESC_QA5065AA	10	512
ESC_QA5063AA	10	512
ESC_QA5062AA	10	512
ESC_QA5061AA	10	512
ESC_QA5058AA	10	512

ESC_QA5057AA	10	512
ESC_QA5056AA	10	512
ESC_QA5055AA	10	512
ESC_QA5054AA	10	512
ESC_QA5053AA	10	512
ESC_QA5052AA	10	512
ESC_QA5051AA	10	512
ESC_QA5050AA	10	512
ESC_QA5049AA	10	512
ESC_QA5048AA	10	512
ESC_QA5046AA	10	512
ESC_QA5045AA	10	512
ESC_QA5044AA	10	512
ESC_QA5043AA	10	512
ESC_QA5042AA	10	512
ESC_QA5040AA	10	512
ESC_QA5038AA	10	512
ESC_QA5037AA	10	512
ESC_QA5036AA	10	512
ESC_QA5035AA	10	512
ESC_QA5033AA	10	512
ESC_QA5031AA	10	512
ESC_QA5028AA	10	512
ESC_QA5027AA	10	512
ESC_QA5026AA	10	512
ESC_QA5023AA	10	512
ESC_QA5021AA	10	512
ESC_QA5020AA	10	512
ESC_QA5019AA	10	512
ESC_QA5018AA	10	512
ESC_QA5017AA	10	512
ESC_QA5016AA	10	512
ESC_QA5015AA	10	512
ESC_QA5014AA	10	512
ESC_QA5013AA	10	512
ESC_QA5008AA	11	936
ESC_QA5007AA	11	936
ESC_QA5006AA	678	257
ESC_QA5005AA	21	156
ESC_QA5004AA	73	737
ESC_QA5003AA	73	737
ESC_QA4927AA	919	421
ESC_QA4918AA	131	653
ESC_QA4917AA	131	653
ESC_QA4915AA	11	936
ESC_QA4914AA	131	653
ESC_QA4913AA	131	653
ESC_QA4912AA	354	15
ESC_QA4911AA	10	512
ESC_QA4889AA	10	512

ESC_QA4888AA	10	512
ESC_QA4887AA	10	512
ESC_QA4886AA	10	512
ESC_QA4884AA	10	512
ESC_QA4883AA	10	512
ESC_QA4882AA	10	512
ESC_QA4881AA	10	512
ESC_QA4880AA	10	512
ESC_QA4879AA	10	512
ESC_QA4878AA	10	512
ESC_QA4877AA	10	512
ESC_QA4876AA	1060	512
ESC_QA4867AA	11	936
ESC_QA4866AA	11	936
ESC_QA4865AA	11	936
ESC_QA4863AA	73	737
ESC_QA4862AA	73	737
ESC_QA4861AA	73	737
ESC_QA4860AA	131	653
ESC_QA4859AA	131	653
ESC_QA4858AA	131	653
ESC_QA4857AA	131	653
ESC_QA4856AA	131	653
ESC_QA4855AA	131	653
ESC_QA4854AA	131	653
ESC_QA4853AA	131	653
ESC_QA4852AA	131	653
ESC_QA4851AA	131	653
ESC_QA4850AA	131	653
ESC_QA4849AA	131	653
ESC_QA4848AA	131	653
ESC_QA4847AA	131	653
ESC_QA4845AA	131	653
ESC_QA4844AA	131	653
ESC_QA4843AA	131	653
ESC_QA4842AA	131	653
ESC_QA4841AA	131	653
ESC_QA4840AA	131	653
ESC_QA4839AA	131	653
ESC_QA4838AA	131	653
ESC_QA4837AA	131	653
ESC_QA4836AA	131	653
ESC_QA4835AA	131	653
ESC_QA4834AA	131	653
ESC_QA4833AA	131	653
ESC_QA4832AA	131	653
ESC_QA4831AA	131	653
ESC_QA4830AA	131	653
ESC_QA4829AA	131	653
ESC_QA4828AA	131	653

ESC_QA4827AA	131	653
ESC_QA4826AA	131	653
ESC_QA4825AA	131	653
ESC_QA4824AA	131	653
ESC_QA4823AA	131	653
ESC_QA4822AA	131	653
ESC_QA4821AA	131	653
ESC_QA4820AA	131	653
ESC_QA4818AA	131	653
ESC_QA4817AA	131	653
ESC_QA4816AA	131	653
ESC_QA4806AA	11	936
ESC_QA4805AA	11	936
ESC_QA4804AA	11	936
ESC_QA4803AA	11	936
ESC_QA4802AA	11	936
ESC_QA4781AA	998	644
ESC_QA4780AA	420	741
ESC_QA4779AA	95	738
ESC_QA4778AA	127	738
ESC_QA4777AA	88	160
ESC_QA4776AA	968	737
ESC_QA4775AA	69	387
ESC_QA4774AA	127	738
ESC_QA4773AA	2619	738
ESC_QA4772AA	127	738
ESC_QA4771AA	12	626
ESC_QA4770AA	131	653
ESC_QA4769AA	10743	574
ESC_QA4768AA	62	574
ESC_QA4767AA	73	737
ESC_QA4766AA	95	738
ESC_QA4765AA	73	737
ESC_QA4764AA	80	731
ESC_QA4763AA	2178	924
ESC_QA4762AA	3856	789
ESC_QA4761AA	1597	629
ESC_QA4760AA	117	592
ESC_QA4759AA	1193	761
ESC_QA4758AA	8582	912
ESC_QA4757AA	62	574
ESC_QA4756AA	4713	653
ESC_QA4755AA	95	738
ESC_QA4754AA	349	670
ESC_QA4753AA	131	653
ESC_QA4752AA	453	935
ESC_QA4751AA	73	737
ESC_QA4750AA	127	738
ESC_QA4749AA	1193	761
ESC_QA4748AA	8433	512

ESC_QA4747AA	127	738
ESC_QA4746AA	73	737
ESC_QA4745AA	141	644
ESC_QA4744AA	131	653
ESC_QA4743AA	10745	817
ESC_QA4742AA	131	653
ESC_QA4741AA	1117	234
ESC_QA4740AA	2713	878
ESC_QA4739AA	998	644
ESC_QA4738AA	127	738
ESC_QA4737AA	73	737
ESC_QA4736AA	127	738
ESC_QA4735AA	939	198
ESC_QA4734AA	1278	797
ESC_QA4733AA	5409	175
ESC_QA4732AA	1193	761
ESC_QA4731AA	154	257
ESC_QA4730AA	10	512
ESC_QA4729AA	95	738
ESC_QA4728AA	95	738
ESC_QA4727AA	7870	198
ESC_QA4726AA	10744	737
ESC_QA4725AA	127	738
ESC_QA4724AA	131	653
ESC_QA4723AA	12	626
ESC_QA4722AA	131	653
ESC_QA4721AA	95	738
ESC_QA4720AA	73	737
ESC_QA4719AA	69	387
ESC_QA4718AA	69	387
ESC_QA4717AA	131	653
ESC_QA4716AA	131	653
ESC_QA4715AA	38	669
ESC_QA4714AA	69	387
ESC_QA4713AA	1056	257
ESC_QA4712AA	12	626
ESC_QA4711AA	69	387
ESC_QA4710AA	95	738
ESC_QA4709AA	73	737
ESC_QA4708AA	95	738
ESC_QA4707AA	12	626
ESC_QA4706AA	457	595
ESC_QA4705AA	1323	405
ESC_QA4704AA	127	738
ESC_QA4703AA	95	738
ESC_QA4702AA	420	741
ESC_QA4701AA	1231	738
ESC_QA4700AA	80	731
ESC_QA4699AA	69	387
ESC_QA4698AA	95	738

ESC_QA4697AA	69	387
ESC_QA4696AA	127	738
ESC_QA4695AA	127	738
ESC_QA4694AA	10746	649
ESC_QA4693AA	80	731
ESC_QA4692AA	95	738
ESC_QA4691AA	131	653
ESC_QA4690AA	69	387
ESC_QA4689AA	80	731
ESC_QA4688AA	127	738
ESC_QA4687AA	420	741
ESC_QA4686AA	73	737
ESC_QA4685AA	73	737
ESC_QA4684AA	961	623
ESC_QA4683AA	12	626
ESC_QA4682AA	95	738
ESC_QA4681AA	127	738
ESC_QA4680AA	93	71
ESC_QA4679AA	393	343
ESC_QA4678AA	95	738
ESC_QA4677AA	73	737
ESC_QA4676AA	95	738
ESC_QA4675AA	5891	878
ESC_QA4674AA	38	669
ESC_QA4673AA	73	737
ESC_QA4672AA	95	738
ESC_QA4671AA	127	738
ESC_QA4670AA	69	387
ESC_QA4669AA	73	737
ESC_QA4668AA	95	738
ESC_QA4667AA	131	653
ESC_QA4666AA	3672	649
ESC_QA4665AA	1326	198
ESC_QA4664AA	420	741
ESC_QA4663AA	73	737
ESC_QA4662AA	10747	737
ESC_QA4661AA	297	234
ESC_QA4660AA	131	653
ESC_QA4659AA	73	737
ESC_QA4658AA	127	738
ESC_QA4657AA	127	738
ESC_QA4656AA	14	761
ESC_QA4655AA	95	738
ESC_QA4654AA	10	512
ESC_QA4653AA	420	741
ESC_QA4652AA	420	741
ESC_QA4651AA	95	738
ESC_QA4650AA	297	234
ESC_QA4649AA	141	644
ESC_QA4648AA	73	737

ESC_QA4647AA	336	878
ESC_QA4646AA	69	387
ESC_QA4645AA	2811	233
ESC_QA4644AA	978	740
ESC_QA4643AA	978	740
ESC_QA4641AA	2248	257
ESC_QA4640AA	141	644
ESC_QA4639AA	73	737
ESC_QA4638AA	23	156
ESC_QA4637AA	1161	764
ESC_QA4636AA	131	653
ESC_QA4635AA	69	387
ESC_QA4634AA	420	741
ESC_QA4633AA	73	737
ESC_QA4632AA	127	738
ESC_QA4631AA	297	234
ESC_QA4630AA	10748	644
ESC_QA4629AA	73	737
ESC_QA4628AA	131	653
ESC_QA4627AA	73	737
ESC_QA4626AA	404	761
ESC_QA4625AA	95	738
ESC_QA4624AA	95	738
ESC_QA4623AA	144	740
ESC_QA4622AA	95	738
ESC_QA4621AA	68	386
ESC_QA4620AA	10	512
ESC_QA4619AA	131	653
ESC_QA4618AA	95	738
ESC_QA4617AA	131	653
ESC_QA4616AA	127	738
ESC_QA4615AA	349	670
ESC_QA4614AA	95	738
ESC_QA4613AA	95	738
ESC_QA4612AA	95	738
ESC_QA4611AA	131	653
ESC_QA4610AA	95	738
ESC_QA4609AA	95	738
ESC_QA4608AA	491	626
ESC_QA4607AA	1859	653
ESC_QA4606AA	59	217
ESC_QA4605AA	491	626
ESC_QA4604AA	58	198
ESC_QA4603AA	3018	903
ESC_QA4602AA	1193	761
ESC_QA4601AA	219	877
ESC_QA4600AA	73	737
ESC_QA4599AA	95	738
ESC_QA4598AA	127	738
ESC_QA4597AA	69	387

ESC_QA4596AA	127	738
ESC_QA4595AA	131	653
ESC_QA4594AA	410	156
ESC_QA4593AA	95	738
ESC_QA4592AA	127	738
ESC_QA4591AA	10	512
ESC_QA4590AA	127	738
ESC_QA4589AA	73	737
ESC_QA4588AA	3764	71
ESC_QA4586AA	95	738
ESC_QA4585AA	1859	653
ESC_QA4584AA	2565	768
ESC_QA4583AA	345	175
ESC_QA4582AA	73	737
ESC_QA4581AA	1193	761
ESC_QA4580AA	95	738
ESC_QA4579AA	404	761
ESC_QA4578AA	73	737
ESC_QA4577AA	1385	653
ESC_QA4576AA	127	738
ESC_QA4575AA	91	629
ESC_QA4574AA	127	738
ESC_QA4573AA	1193	761
ESC_QA4572AA	372	424
ESC_QA4571AA	420	741
ESC_QA4570AA	95	738
ESC_QA4569AA	95	738
ESC_QA4568AA	538	629
ESC_QA4567AA	95	738
ESC_QA4566AA	12	626
ESC_QA4565AA	95	738
ESC_QA4564AA	10	512
ESC_QA4563AA	349	670
ESC_QA4562AA	69	387
ESC_QA4561AA	5026	387
ESC_QA4560AA	420	741
ESC_QA4559AA	95	738
ESC_QA4558AA	127	738
ESC_QA4557AA	73	737
ESC_QA4556AA	127	738
ESC_QA4555AA	12	626
ESC_QA4554AA	362	234
ESC_QA4553AA	80	731
ESC_QA4552AA	144	740
ESC_QA4551AA	69	387
ESC_QA4550AA	95	738
ESC_QA4549AA	131	653
ESC_QA4548AA	70	670
ESC_QA4547AA	6998	629
ESC_QA4546AA	4713	653

ESC_QA4545AA	12	626
ESC_QA4544AA	95	738
ESC_QA4543AA	73	737
ESC_QA4542AA	58	198
ESC_QA4541AA	357	654
ESC_QA4540AA	12	626
ESC_QA4539AA	127	738
ESC_QA4538AA	10	512
ESC_QA4537AA	95	738
ESC_QA4536AA	69	387
ESC_QA4535AA	131	653
ESC_QA4534AA	538	629
ESC_QA4533AA	69	387
ESC_QA4532AA	95	738
ESC_QA4531AA	357	654
ESC_QA4530AA	95	738
ESC_QA4529AA	12	626
ESC_QA4528AA	127	738
ESC_QA4527AA	127	738
ESC_QA4526AA	95	738
ESC_QA4525AA	131	653
ESC_QA4524AA	69	387
ESC_QA4523AA	961	623
ESC_QA4522AA	69	387
ESC_QA4521AA	73	737
ESC_QA4520AA	127	738
ESC_QA4519AA	12	626
ESC_QA4518AA	38	669
ESC_QA4517AA	8371	898
ESC_QA4516AA	95	738
ESC_QA4515AA	1193	761
ESC_QA4514AA	998	644
ESC_QA4513AA	73	737
ESC_QA4512AA	127	738
ESC_QA4511AA	95	738
ESC_QA4510AA	38	669
ESC_QA4509AA	95	738
ESC_QA4508AA	1656	175
ESC_QA4507AA	404	761
ESC_QA4506AA	95	738
ESC_QA4505AA	1304	257
ESC_QA4504AA	80	731
ESC_QA4503AA	127	738
ESC_QA4502AA	95	738
ESC_QA4501AA	80	731
ESC_QA4500AA	10750	653
ESC_QA4499AA	9135	257
ESC_QA4498AA	10749	738
ESC_QA4497AA	131	653
ESC_QA4496AA	73	737

ESC_QA4495AA	1081	198
ESC_QA4494AA	69	387
ESC_QA4493AA	131	653
ESC_QA4492AA	95	738
ESC_QA4491AA	162	924
ESC_QA4490AA	5064	198
ESC_QA4489AA	547	653
ESC_QA4488AA	69	387
ESC_QA4487AA	95	738
ESC_QA4486AA	372	424
ESC_QA4485AA	95	738
ESC_QA4484AA	6998	629
ESC_QA4483AA	73	737
ESC_QA4482AA	69	387
ESC_QA4481AA	681	393
ESC_QA4480AA	1193	761
ESC_QA4479AA	73	737
ESC_QA4478AA	69	387
ESC_QA4477AA	69	387
ESC_QA4476AA	131	653
ESC_QA4475AA	131	653
ESC_QA4474AA	2	900
ESC_QA4473AA	95	738
ESC_QA4472AA	95	738
ESC_QA4471AA	59	217
ESC_QA4470AA	131	653
ESC_QA4469AA	131	653
ESC_QA4468AA	95	738
ESC_QA4467AA	69	387
ESC_QA4466AA	681	393
ESC_QA4465AA	95	738
ESC_QA4464AA	69	387
ESC_QA4463AA	6250	737
ESC_QA4462AA	131	653
ESC_QA4461AA	131	653
ESC_QA4460AA	73	737
ESC_QA4459AA	95	738
ESC_QA4458AA	95	738
ESC_QA4457AA	362	234
ESC_QA4456AA	80	731
ESC_QA4455AA	95	738
ESC_QA4454AA	998	644
ESC_QA4453AA	404	761
ESC_QA4452AA	73	737
ESC_QA4451AA	73	737
ESC_QA4450AA	69	387
ESC_QA4449AA	38	669
ESC_QA4448AA	95	738
ESC_QA4447AA	6690	504
ESC_QA4446AA	127	738

ESC_QA4445AA	62	574
ESC_QA4444AA	10	512
ESC_QA4443AA	73	737
ESC_QA4442AA	131	653
ESC_QA4441AA	38	669
ESC_QA4440AA	80	731
ESC_QA4439AA	73	737
ESC_QA4438AA	636	653
ESC_QA4437AA	10	512
ESC_QA4436AA	491	626
ESC_QA4435AA	2622	537
ESC_QA4434AA	636	653
ESC_QA4433AA	95	738
ESC_QA4432AA	73	737
ESC_QA4431AA	1193	761
ESC_QA4430AA	1735	737
ESC_QA4429AA	95	738
ESC_QA4428AA	95	738
ESC_QA4427AA	73	737
ESC_QA4426AA	69	387
ESC_QA4425AA	101	696
ESC_QA4424AA	141	644
ESC_QA4423AA	95	738
ESC_QA4422AA	73	737
ESC_QA4421AA	95	738
ESC_QA4420AA	73	737
ESC_QA4419AA	390	738
ESC_QA4418AA	95	738
ESC_QA4417AA	10	512
ESC_QA4416AA	127	738
ESC_QA4415AA	95	738
ESC_QA4414AA	12	626
ESC_QA4413AA	131	653
ESC_QA4412AA	569	738
ESC_QA4411AA	357	654
ESC_QA4410AA	95	738
ESC_QA4409AA	95	738
ESC_QA4408AA	1859	653
ESC_QA4407AA	569	738
ESC_QA4406AA	173	254
ESC_QA4405AA	131	653
ESC_QA4404AA	127	738
ESC_QA4403AA	95	738
ESC_QA4401AA	69	387
ESC_QA4400AA	131	653
ESC_QA4399AA	95	738
ESC_QA4398AA	69	387
ESC_QA4397AA	131	653
ESC_QA4396AA	95	738
ESC_QA4395AA	393	343

ESC_QA4394AA	131	653
ESC_QA4383AA	10	512
ESC_QA4382AA	10	512
ESC_QA4381AA	10	512
ESC_QA4380AA	10	512
ESC_QA4379AA	10	512
ESC_QA4378AA	10	512
ESC_QA4377AA	10	512
ESC_QA4376AA	10	512
ESC_QA4356AA	131	653
ESC_QA4355AA	131	653
ESC_QA4353AA	131	653
ESC_QA4352AA	131	653
ESC_QA4351AA	131	653
ESC_QA4350AA	131	653
ESC_QA4349AA	131	653
ESC_QA4348AA	131	653
ESC_QA4347AA	131	653
ESC_QA4336AA	131	653
ESC_QA4335AA	131	653
ESC_QA4334AA	131	653
ESC_QA4332AA	1122	307
ESC_QA4331AA	1122	307
ESC_QA4330AA	394	400
ESC_QA4329AA	131	653
ESC_QA4328AA	131	653
ESC_QA4327AA	131	653
ESC_QA4326AA	131	653
ESC_QA4325AA	131	653
ESC_QA4324AA	131	653
ESC_QA4323AA	131	653
ESC_QA4322AA	131	653
ESC_QA4321AA	394	400
ESC_QA4320AA	131	653
ESC_QA4319AA	10	512
ESC_QA4318AA	1122	307
ESC_QA4279AA	131	653
ESC_QA4276AA	90	156
ESC_QA4273AA	5869	257
ESC_QA4272AA	10	512
ESC_QA3295AA	131	653
ESC_QA3294AA	1193	761
ESC_QA3293AA	420	741
ESC_QA3292AA	10707	761
ESC_QA3291AA	131	653
ESC_QA3290AA	10708	198
ESC_QA3289AA	95	738
ESC_QA3288AA	131	653
ESC_QA3287AA	131	653
ESC_QA3286AA	131	653

ESC_QA3284AA	540	286
ESC_QA3283AA	73	737
ESC_QA3281AA	10	512
ESC_QA3280AA	8098	257
ESC_QA3279AA	1434	512
ESC_QA3278AA	68	386
ESC_QA3277AA	10704	71
ESC_QA3276AA	2522	198
ESC_QA3275AA	1727	257
ESC_QA3274AA	12	626
ESC_QA3273AA	152	257
ESC_QA3272AA	152	257
ESC_QA3271AA	152	257
ESC_QA3270AA	152	257
ESC_QA3269AA	152	257
ESC_QA3268AA	152	257
ESC_QA3267AA	152	257
ESC_QA3266AA	152	257
ESC_QA3265AA	152	257
ESC_QA3264AA	152	257
ESC_QA3263AA	152	257
ESC_QA3262AA	152	257
ESC_QA3261AA	152	257
ESC_QA3260AA	152	257
ESC_QA3259AA	152	257
ESC_QA3258AA	152	257
ESC_QA3257AA	152	257
ESC_QA3256AA	152	257
ESC_QA3255AA	152	257
ESC_QA3254AA	152	257
ESC_QA3253AA	152	257
ESC_QA3252AA	152	257
ESC_QA3251AA	152	257
ESC_QA3250AA	152	257
ESC_QA3249AA	152	257
ESC_QA3248AA	152	257
ESC_QA3247AA	152	257
ESC_QA3246AA	152	257
ESC_QA3245AA	152	257
ESC_QA3244AA	152	257
ESC_QA3243AA	152	257
ESC_QA3242AA	152	257
ESC_QA3241AA	152	257
ESC_QA3240AA	152	257
ESC_QA3239AA	152	257
ESC_QA3238AA	152	257
ESC_QA3237AA	152	257
ESC_QA3236AA	152	257
ESC_QA3235AA	152	257
ESC_QA3234AA	152	257

ESC_QA3233AA	152	257
ESC_QA3232AA	152	257
ESC_QA3231AA	152	257
ESC_QA3230AA	152	257
ESC_QA3229AA	152	257
ESC_QA3228AA	152	257
ESC_QA3227AA	152	257
ESC_QA3226AA	152	257
ESC_QA3225AA	152	257
ESC_QA3224AA	152	257
ESC_QA3223AA	152	257
ESC_QA3222AA	152	257
ESC_QA3221AA	58	198
ESC_QA3220AA	10	512
ESC_QA3219AA	10	512
ESC_QA3218AA	10	512
ESC_QA3217AA	10	512
ESC_QA3216AA	10	512
ESC_QA3215AA	10	512
ESC_QA3214AA	10	512
ESC_QA3213AA	10	512
ESC_QA3212AA	10	512
ESC_QA3211AA	10	512
ESC_QA3210AA	10	512
ESC_QA3209AA	10	512
ESC_QA3208AA	10	512
ESC_QA3207AA	10	512
ESC_QA3206AA	10	512
ESC_QA3205AA	10	512
ESC_QA3204AA	617	511
ESC_QA3203AA	93	71
ESC_QA3202AA	93	71
ESC_QA3201AA	93	71
ESC_QA3200AA	93	71
ESC_QA3199AA	93	71
ESC_QA3198AA	93	71
ESC_QA3197AA	93	71
ESC_QA3196AA	93	71
ESC_QA3195AA	93	71
ESC_QA3194AA	93	71
ESC_QA3193AA	93	71
ESC_QA3192AA	93	71
ESC_QA3191AA	93	71
ESC_QA3190AA	93	71
ESC_QA3189AA	93	71
ESC_QA3188AA	93	71
ESC_QA3187AA	93	71
ESC_QA3186AA	93	71
ESC_QA3185AA	93	71
ESC_QA3184AA	93	71

ESC_QA3183AA	93	71
ESC_QA3182AA	93	71
ESC_QA3181AA	93	71
ESC_QA3180AA	93	71
ESC_QA3179AA	93	71
ESC_QA3178AA	93	71
ESC_QA3177AA	93	71
ESC_QA3176AA	93	71
ESC_QA3175AA	93	71
ESC_QA3174AA	93	71
ESC_QA3173AA	93	71
ESC_QA3168AA	306	286
ESC_QA3167AA	10698	512
ESC_QA3166AA	616	156
ESC_QA3165AA	301	307
ESC_QA3164AA	442	257
ESC_QA3163AA	442	257
ESC_QA3162AA	306	286
ESC_QA3161AA	504	738
ESC_QA3160AA	442	257
ESC_QA3159AA	17	191
ESC_QA3158AA	300	286
ESC_QA3157AA	162	924
ESC_QA3156AA	17	191
ESC_QA3155AA	3101	160
ESC_QA3154AA	25	234
ESC_QA3153AA	442	257
ESC_QA3152AA	442	257
ESC_QA3151AA	10	512
ESC_QA3150AA	1722	599
ESC_QA3149AA	131	653
ESC_QA3148AA	95	738
ESC_QA3147AA	131	653
ESC_QA3146AA	131	653
ESC_QA3145AA	410	156
ESC_QA3144AA	44	512
ESC_QA3143AA	405	923
ESC_QA3142AA	167	512
ESC_QA3141AA	38	669
ESC_QA3140AA	410	156
ESC_QA3139AA	38	669
ESC_QA3138AA	38	669
ESC_QA3137AA	448	257
ESC_QA3136AA	69	387
ESC_QA3134AA	648	359
ESC_QA3133AA	44	512
ESC_QA3132AA	648	359
ESC_QA3131AA	352	744
ESC_QA3130AA	7479	669
ESC_QA3129AA	69	387

ESC_QA3128AA	127	738
ESC_QA3127AA	7092	740
ESC_QA3126AA	167	512
ESC_QA3125AA	361	512
ESC_QA3124AA	38	669
ESC_QA3123AA	95	738
ESC_QA3122AA	38	669
ESC_QA3121AA	361	512
ESC_QA3120AA	38	669
ESC_QA3119AA	361	512
ESC_QA3118AA	716	795
ESC_QA3117AA	38	669
ESC_QA3116AA	405	923
ESC_QA3115AA	38	669
ESC_QA3114AA	354	15
ESC_QA3113AA	127	738
ESC_QA3112AA	38	669
ESC_QA3111AA	38	669
ESC_QA3110AA	156	175
ESC_QA3109AA	744	512
ESC_QA3108AA	405	923
ESC_QA3107AA	10	512
ESC_QA3106AA	6468	512
ESC_QA3105AA	7476	1004
ESC_QA3104AA	1611	198
ESC_QA3103AA	205	257
ESC_QA3102AA	3288	286
ESC_QA3101AA	540	286
ESC_QA3100AA	6732	1004
ESC_QA3099AA	2599	257
ESC_QA3098AA	69	387
ESC_QA3097AA	398	445
ESC_QA3096AA	156	175
ESC_QA3095AA	410	156
ESC_QA3094AA	167	512
ESC_QA3093AA	38	669
ESC_QA3092AA	167	512
ESC_QA3091AA	155	175
ESC_QA3090AA	38	669
ESC_QA3089AA	405	923
ESC_QA3088AA	167	512
ESC_QA3087AA	155	175
ESC_QA3086AA	167	512
ESC_QA3085AA	361	512
ESC_QA3084AA	167	512
ESC_QA3083AA	156	175
ESC_QA3082AA	398	445
ESC_QA3081AA	73	737
ESC_QA3080AA	410	156
ESC_QA3079AA	940	257

ESC_QA3078AA	167	512
ESC_QA3077AA	1722	599
ESC_QA3076AA	156	175
ESC_QA3075AA	101	696
ESC_QA3074AA	410	156
ESC_QA3073AA	38	669
ESC_QA3072AA	2346	435
ESC_QA3071AA	410	156
ESC_QA3070AA	88	160
ESC_QA3069AA	38	669
ESC_QA3068AA	7478	669
ESC_QA3067AA	617	511
ESC_QA3066AA	457	595
ESC_QA3065AA	648	359
ESC_QA3064AA	607	355
ESC_QA3063AA	38	669
ESC_QA3062AA	405	923
ESC_QA3061AA	38	669
ESC_QA3060AA	940	257
ESC_QA3059AA	38	669
ESC_QA3058AA	448	257
ESC_QA3057AA	648	359
ESC_QA3056AA	405	923
ESC_QA3055AA	390	738
ESC_QA3054AA	354	15
ESC_QA3053AA	405	923
ESC_QA3052AA	38	669
ESC_QA3051AA	940	257
ESC_QA3050AA	297	234
ESC_QA3049AA	167	512
ESC_QA3048AA	38	669
ESC_QA3047AA	648	359
ESC_QA3046AA	405	923
ESC_QA3045AA	646	738
ESC_QA3044AA	10	512
ESC_QA3043AA	3076	175
ESC_QA3042AA	410	156
ESC_QA3041AA	38	669
ESC_QA3040AA	131	653
ESC_QA3039AA	448	257
ESC_QA3038AA	1702	512
ESC_QA3037AA	1588	100
ESC_QA3036AA	940	257
ESC_QA3035AA	3541	842
ESC_QA3034AA	361	512
ESC_QA3033AA	410	156
ESC_QA3032AA	131	653
ESC_QA3031AA	131	653
ESC_QA3030AA	131	653
ESC_QA3029AA	131	653

ESC_QA3028AA	131	653
ESC_QA3027AA	131	653
ESC_QA3026AA	167	512
ESC_QA3025AA	533	198
ESC_QA3024AA	38	669
ESC_QA3023AA	127	738
ESC_QA3022AA	69	387
ESC_QA3021AA	7480	234
ESC_QA3020AA	38	669
ESC_QA3019AA	38	669
ESC_QA3018AA	648	359
ESC_QA3016AA	167	512
ESC_QA3015AA	131	653
ESC_QA3014AA	361	512
ESC_QA3013AA	191	198
ESC_QA3012AA	10	512
ESC_QA3011AA	10	512
ESC_QA3010AA	617	511
ESC_QA3009AA	10	512
ESC_QA3008AA	410	156
ESC_QA3007AA	167	512
ESC_QA3006AA	1737	243
ESC_QA3005AA	10	512
ESC_QA3004AA	167	512
ESC_QA3003AA	38	669
ESC_QA3002AA	540	286
ESC_QA3001AA	69	387
ESC_QA3000AA	10	512
ESC_QA2999AA	167	512
ESC_QA2998AA	3672	649
ESC_QA2997AA	625	756
ESC_QA2996AA	940	257
ESC_QA2995AA	443	257
ESC_QA2994AA	1015	175
ESC_QA2993AA	38	669
ESC_QA2992AA	167	512
ESC_QA2991AA	69	387
ESC_QA2990AA	38	669
ESC_QA2989AA	167	512
ESC_QA2988AA	405	923
ESC_QA2987AA	46	447
ESC_QA2986AA	38	669
ESC_QA2985AA	410	156
ESC_QA2984AA	131	653
ESC_QA2983AA	69	387
ESC_QA2982AA	120	180
ESC_QA2981AA	38	669
ESC_QA2980AA	394	400
ESC_QA2979AA	405	923
ESC_QA2978AA	167	512

ESC_QA2977AA	8072	286
ESC_QA2976AA	224	198
ESC_QA2975AA	3489	962
ESC_QA2974AA	1193	761
ESC_QA2973AA	349	670
ESC_QA2972AA	3018	903
ESC_QA2971AA	167	512
ESC_QA2970AA	131	653
ESC_QA2969AA	167	512
ESC_QA2968AA	38	669
ESC_QA2967AA	410	156
ESC_QA2966AA	38	669
ESC_QA2965AA	10	512
ESC_QA2964AA	38	669
ESC_QA2963AA	10	512
ESC_QA2962AA	405	923
ESC_QA2961AA	617	511
ESC_QA2960AA	405	923
ESC_QA2959AA	205	257
ESC_QA2958AA	617	511
ESC_QA2957AA	38	669
ESC_QA2956AA	38	669
ESC_QA2955AA	8282	76
ESC_QA2954AA	2014	406
ESC_QA2953AA	1284	604
ESC_QA2952AA	410	156
ESC_QA2951AA	1011	198
ESC_QA2950AA	3580	257
ESC_QA2949AA	1284	604
ESC_QA2948AA	410	156
ESC_QA2947AA	127	738
ESC_QA2946AA	10	512
ESC_QA2945AA	38	669
ESC_QA2944AA	162	924
ESC_QA2943AA	405	923
ESC_QA2942AA	48	76
ESC_QA2941AA	10	512
ESC_QA2940AA	8383	512
ESC_QA2939AA	405	923
ESC_QA2938AA	10	512
ESC_QA2937AA	38	669
ESC_QA2936AA	167	512
ESC_QA2935AA	4450	844
ESC_QA2934AA	38	669
ESC_QA2933AA	69	387
ESC_QA2932AA	127	738
ESC_QA2931AA	410	156
ESC_QA2930AA	10	512
ESC_QA2929AA	46	447
ESC_QA2928AA	38	669

ESC_QA2927AA	38	669
ESC_QA2926AA	2083	257
ESC_QA2925AA	205	257
ESC_QA2924AA	167	512
ESC_QA2923AA	192	696
ESC_QA2922AA	963	669
ESC_QA2921AA	224	198
ESC_QA2920AA	410	156
ESC_QA2919AA	940	257
ESC_QA2918AA	38	669
ESC_QA2917AA	46	447
ESC_QA2916AA	1642	198
ESC_QA2915AA	648	359
ESC_QA2914AA	636	653
ESC_QA2913AA	38	669
ESC_QA2912AA	38	669
ESC_QA2911AA	38	669
ESC_QA2910AA	38	669
ESC_QA2909AA	1284	604
ESC_QA2908AA	940	257
ESC_QA2907AA	38	669
ESC_QA2906AA	38	669
ESC_QA2905AA	127	738
ESC_QA2904AA	1415	504
ESC_QA2903AA	2851	156
ESC_QA2902AA	2851	156
ESC_QA2901AA	227	504
ESC_QA2900AA	167	512
ESC_QA2899AA	8346	234
ESC_QA2898AA	38	669
ESC_QA2897AA	354	15
ESC_QA2896AA	617	511
ESC_QA2895AA	405	923
ESC_QA2894AA	10	512
ESC_QA2893AA	405	923
ESC_QA2892AA	2035	113
ESC_QA2891AA	1598	447
ESC_QA2890AA	2952	926
ESC_QA2889AA	8796	955
ESC_QA2888AA	656	789
ESC_QA2887AA	744	512
ESC_QA2886AA	744	512
ESC_QA2885AA	1598	447
ESC_QA2884AA	354	15
ESC_QA2883AA	410	156
ESC_QA2882AA	11	936
ESC_QA2881AA	12	626
ESC_QA2880AA	46	447
ESC_QA2879AA	7233	814
ESC_QA2878AA	10701	257

ESC_QA2877AA	189	504
ESC_QA2876AA	877	935
ESC_QA2875AA	906	198
ESC_QA2874AA	1992	257
ESC_QA2873AA	540	286
ESC_QA2872AA	4198	878
ESC_QA2871AA	10	512
ESC_QA2870AA	10	512
ESC_QA2869AA	58	198
ESC_QA2868AA	33	198
ESC_QA2867AA	306	286
ESC_QA2866AA	17	191
ESC_QA2865AA	21	156
ESC_QA2864AA	33	198
ESC_QA2863AA	21	156
ESC_QA2862AA	21	156
ESC_QA2861AA	3032	912
ESC_QA2860AA	6130	268
ESC_QA2858AA	6688	156
ESC_QA2857AA	90	156
ESC_QA2856AA	504	738
ESC_QA2855AA	200	198
ESC_QA2854AA	154	257
ESC_QA2853AA	154	257
ESC_QA2852AA	154	257
ESC_QA2851AA	154	257
ESC_QA2850AA	1286	474
ESC_QA2849AA	23	156
ESC_QA2848AA	8211	257
ESC_QA2847AA	401	142
ESC_QA2846AA	162	924
ESC_QA2845AA	162	924
ESC_QA2844AA	5796	592
ESC_QA2843AA	295	160
ESC_QA2842AA	10	512
ESC_QA2841AA	6880	726
ESC_QA2840AA	10700	504
ESC_QA2839AA	115	669
ESC_QA2838AA	101	696
ESC_QA2837AA	2175	212
ESC_QA2836AA	162	924
ESC_QA2835AA	716	795
ESC_QA2834AA	10	512
ESC_QA2833AA	642	924
ESC_QA2832AA	398	445
ESC_QA2831AA	58	198
ESC_QA2830AA	8444	970
ESC_QA2829AA	10702	410
ESC_QA2828AA	131	653
ESC_QA2827AA	58	198

ESC_QA2826AA	3550	924
ESC_QA2825AA	34	504
ESC_QA2824AA	10	512
ESC_QA2823AA	162	924
ESC_QA2822AA	648	359
ESC_QA2821AA	367	156
ESC_QA2820AA	398	445
ESC_QA2819AA	1888	900
ESC_QA2818AA	131	653
ESC_QA2817AA	1727	257
ESC_QA2816AA	2144	257
ESC_QA2815AA	164	175
ESC_QA2814AA	131	653
ESC_QA2813AA	216	512
ESC_QA2764AA	16	156
ESC_QA2763AA	101	696
ESC_QA2762AA	3672	649
ESC_QA2761AA	141	644
ESC_QA2760AA	101	696
ESC_QA2759AA	2531	198
ESC_QA2758AA	428	654
ESC_QA2757AA	1079	235
ESC_QA2756AA	399	198
ESC_QA2755AA	401	142
ESC_QA2754AA	1434	512
ESC_QA2753AA	23	156
ESC_QA2752AA	3476	512
ESC_QA2742AA	297	234
ESC_QA2741AA	616	156
ESC_QA2699AA	4977	71
ESC_QA2698AA	156	175
ESC_QA2696AA	10705	936
ESC_QA2695AA	11	936
ESC_QA2694AA	11	936
ESC_QA2693AA	11	936
ESC_QA2690AA	10	512
ESC_QA2689AA	10	512
ESC_QA2688AA	453	935
ESC_QA2687AA	34	504
ESC_QA2686AA	21	156
ESC_QA2685AA	3101	160
ESC_QA2684AA	648	359
ESC_QA2683AA	11	936
ESC_QA2682AA	10	512
ESC_QA2681AA	11	936
ESC_QA2680AA	122	768
ESC_QA2679AA	11	936
ESC_QA2678AA	517	924
ESC_QA2677AA	11	936
ESC_QA2676AA	5292	738

ESC_QA2675AA	3101	160
ESC_QA2674AA	29	156
ESC_QA2673AA	504	738
ESC_QA2672AA	21	156
ESC_QA2671AA	200	198
ESC_QA2670AA	21	156
ESC_QA2669AA	11	936
ESC_QA2668AA	504	738
ESC_QA2667AA	11	936
ESC_QA2666AA	3519	101
ESC_QA2665AA	11	936
ESC_QA2664AA	636	653
ESC_QA2663AA	636	653
ESC_QA2662AA	167	512
ESC_QA2661AA	167	512
ESC_QA2660AA	167	512
ESC_QA2659AA	167	512
ESC_QA2658AA	167	512
ESC_QA2657AA	636	653
ESC_QA2656AA	636	653
ESC_QA2655AA	156	175
ESC_QA2654AA	156	175
ESC_QA2653AA	10706	217
ESC_QA2652AA	738	598
ESC_QA2650AA	90	156
ESC_QA2649AA	90	156
ESC_QA2648AA	100	504
ESC_QA2647AA	73	737
ESC_QA2646AA	10530	156
ESC_QA2645AA	646	738
ESC_QA2644AA	2144	257
ESC_QA2643AA	2144	257
ESC_QA2642AA	69	387
ESC_QA2641AA	80	731
ESC_QA2640AA	2521	257
ESC_QA2639AA	17	191
ESC_QA2638AA	723	156
ESC_QA2637AA	46	447
ESC_QA2636AA	1300	844
ESC_QA2635AA	17	191
ESC_QA2634AA	20	198
ESC_QA2633AA	21	156
ESC_QA2632AA	69	387
ESC_QA2631AA	32	270
ESC_QA2630AA	5082	198
ESC_QA2629AA	69	387
ESC_QA2628AA	11	936
ESC_QA2627AA	21	156
ESC_QA2626AA	540	286
ESC_QA2625AA	187	257

ESC_QA2624AA	17	191
ESC_QA2623AA	8076	504
ESC_QA2622AA	11	936
ESC_QA2621AA	1610	198
ESC_QA2620AA	372	424
ESC_QA2619AA	12	626
ESC_QA2618AA	93	71
ESC_QA2617AA	100	504
ESC_QA2616AA	100	504
ESC_QA2615AA	10	512
ESC_QA2614AA	10	512
ESC_QA2613AA	90	156
ESC_QA2612AA	10	512
ESC_QA2611AA	131	653
ESC_QA2610AA	641	935
ESC_QA2609AA	73	737
ESC_QA2608AA	131	653
ESC_QA2607AA	372	424
ESC_QA2606AA	1260	512
ESC_QA2605AA	10	512
ESC_QA2604AA	3524	935
ESC_QA2603AA	127	738
ESC_QA2602AA	42	257
ESC_QA2601AA	90	156
ESC_QA2600AA	10530	156
ESC_QA2599AA	2520	878
ESC_QA2598AA	10	512
ESC_QA2597AA	6242	424
ESC_QA2596AA	12	626
ESC_QA2595AA	372	424
ESC_QA2594AA	10	512
ESC_QA2593AA	10	512
ESC_QA2592AA	69	387
ESC_QA2591AA	10	512
ESC_QA2590AA	95	738
ESC_QA2589AA	93	71
ESC_QA2588AA	2682	87
ESC_QA2587AA	10710	118
ESC_QA2586AA	10709	135
ESC_QA2585AA	2682	87
ESC_QA2584AA	973	968
ESC_QA2583AA	100	504
ESC_QA2582AA	1723	900
ESC_QA2581AA	372	424
ESC_QA2580AA	929	742
ESC_QA2579AA	10	512
ESC_QA2578AA	7259	672
ESC_QA2577AA	93	71
ESC_QA2576AA	90	156
ESC_QA2575AA	90	156

ESC_QA2574AA	196	257
ESC_QA2573AA	196	257
ESC_QA2572AA	6187	234
ESC_QA2571AA	8612	198
ESC_QA2570AA	6096	257
ESC_QA2569AA	28	537
ESC_QA2568AA	10	512
ESC_QA2567AA	937	198
ESC_QA2566AA	48	76
ESC_QA2565AA	10	512
ESC_QA2564AA	40	198
ESC_QA2563AA	131	653
ESC_QA2562AA	131	653
ESC_QA2561AA	131	653
ESC_QA2560AA	131	653
ESC_QA2559AA	10752	370
ESC_QA2558AA	1704	257
ESC_QA2557AA	10751	175
ESC_QA2556AA	372	424
ESC_QA2555AA	1170	629
ESC_QA2554AA	11	936
ESC_QA2553AA	11	936
ESC_QA2552AA	11	936
ESC_QA2551AA	11	936
ESC_QA2550AA	11	936
ESC_QA2549AA	11	936
ESC_QA2548AA	11	936
ESC_QA2547AA	11	936
ESC_QA2546AA	11	936
ESC_QA2545AA	11	936
ESC_QA2544AA	11	936
ESC_QA2543AA	11	936
ESC_QA2542AA	11	936
ESC_QA2541AA	11	936
ESC_QA2540AA	11	936
ESC_QA2539AA	11	936
ESC_QA2538AA	11	936
ESC_QA2537AA	11	936
ESC_QA2536AA	11	936
ESC_QA2535AA	11	936
ESC_QA2534AA	11	936
ESC_QA2533AA	11	936
ESC_QA2532AA	11	936
ESC_QA2531AA	11	936
ESC_QA2530AA	11	936
ESC_QA2529AA	11	936
ESC_QA2528AA	11	936
ESC_QA2527AA	8716	49
ESC_QA2526AA	117	592
ESC_QA2525AA	68	386

ESC_QA2524AA	68	386
ESC_QA2523AA	10	512
ESC_QA2522AA	602	257
ESC_QA2521AA	602	257
ESC_QA2520AA	10	512
ESC_QA2519AA	43	512
ESC_QA2518AA	10	512
ESC_QA2517AA	10	512
ESC_QA2516AA	10	512
ESC_QA2515AA	43	512
ESC_QA2514AA	1079	235
ESC_QA2513AA	162	924
ESC_QA2512AA	349	670
ESC_QA2511AA	162	924
ESC_QA2510AA	162	924
ESC_QA2509AA	1140	934
ESC_QA2508AA	349	670
ESC_QA2507AA	155	175
ESC_QA2506AA	355	764
ESC_QA2505AA	641	935
ESC_QA2504AA	48	76
ESC_QA2503AA	10	512
ESC_QA2502AA	394	400
ESC_QA2501AA	394	400
ESC_QA2500AA	95	738
ESC_QA2499AA	117	592
ESC_QA2498AA	70	670
ESC_QA2497AA	137	270
ESC_QA2496AA	1421	447
ESC_QA2495AA	8714	160
ESC_QA2494AA	58	198
ESC_QA2493AA	88	160
ESC_QA2492AA	7529	198
ESC_QA2491AA	58	198
ESC_QA2490AA	23	156
ESC_QA2489AA	753	842
ESC_QA2488AA	23	156
ESC_QA2487AA	278	924
ESC_QA2486AA	8719	160
ESC_QA2485AA	101	696
ESC_QA2484AA	10	512
ESC_QA2483AA	93	71
ESC_QA2482AA	8721	268
ESC_QA2481AA	88	160
ESC_QA2480AA	10	512
ESC_QA2479AA	57	50
ESC_QA2478AA	8712	512
ESC_QA2477AA	117	592
ESC_QA2476AA	394	400
ESC_QA2475AA	6031	108

ESC_QA2474AA	117	592
ESC_QA2473AA	93	71
ESC_QA2472AA	155	175
ESC_QA2471AA	155	175
ESC_QA2470AA	10	512
ESC_QA2469AA	10	512
ESC_QA2468AA	3744	519
ESC_QA2467AA	117	592
ESC_QA2466AA	117	592
ESC_QA2465AA	10	512
ESC_QA2464AA	117	592
ESC_QA2463AA	117	592
ESC_QA2462AA	10	512
ESC_QA2461AA	215	512
ESC_QA2460AA	101	696
ESC_QA2459AA	10	512
ESC_QA2458AA	88	160
ESC_QA2457AA	10	512
ESC_QA2456AA	101	696
ESC_QA2455AA	3595	822
ESC_QA2454AA	3595	822
ESC_QA2453AA	3595	822
ESC_QA2452AA	3595	822
ESC_QA2451AA	641	935
ESC_QA2450AA	752	512
ESC_QA2449AA	744	512
ESC_QA2448AA	58	198
ESC_QA2447AA	58	198
ESC_QA2446AA	362	234
ESC_QA2445AA	2496	789
ESC_QA2444AA	7036	445
ESC_QA2443AA	8718	1004
ESC_QA2442AA	5451	912
ESC_QA2441AA	69	387
ESC_QA2440AA	685	311
ESC_QA2439AA	10	512
ESC_QA2438AA	10	512
ESC_QA2437AA	5708	605
ESC_QA2436AA	167	512
ESC_QA2435AA	155	175
ESC_QA2434AA	540	286
ESC_QA2433AA	540	286
ESC_QA2432AA	744	512
ESC_QA2431AA	58	198
ESC_QA2430AA	8715	965
ESC_QA2429AA	58	198
ESC_QA2428AA	540	286
ESC_QA2427AA	162	924
ESC_QA2426AA	54	256
ESC_QA2425AA	167	512

ESC_QA2424AA	744	512
ESC_QA2423AA	540	286
ESC_QA2422AA	155	175
ESC_QA2421AA	162	924
ESC_QA2420AA	162	924
ESC_QA2419AA	540	286
ESC_QA2418AA	58	198
ESC_QA2417AA	58	198
ESC_QA2416AA	58	198
ESC_QA2415AA	58	198
ESC_QA2414AA	10	512
ESC_QA2413AA	683	175
ESC_QA2412AA	162	924
ESC_QA2411AA	1851	380
ESC_QA2410AA	23	156
ESC_QA2409AA	23	156
ESC_QA2408AA	191	198
ESC_QA2407AA	162	924
ESC_QA2406AA	162	924
ESC_QA2405AA	1011	198
ESC_QA2404AA	8717	204
ESC_QA2403AA	162	924
ESC_QA2402AA	155	175
ESC_QA2401AA	58	198
ESC_QA2400AA	694	512
ESC_QA2399AA	4985	254
ESC_QA2398AA	4985	254
ESC_QA2397AA	141	644
ESC_QA2396AA	2068	653
ESC_QA2395AA	2068	653
ESC_QA2394AA	4213	251
ESC_QA2393AA	95	738
ESC_QA2392AA	155	175
ESC_QA2391AA	69	387
ESC_QA2390AA	48	76
ESC_QA2389AA	131	653
ESC_QA2388AA	2496	789
ESC_QA2387AA	2496	789
ESC_QA2386AA	1431	257
ESC_QA2385AA	1431	257
ESC_QA2384AA	162	924
ESC_QA2383AA	162	924
ESC_QA2382AA	141	644
ESC_QA2381AA	2705	504
ESC_QA2380AA	88	160
ESC_QA2379AA	8713	924
ESC_QA2378AA	7593	198
ESC_QA2377AA	131	653
ESC_QA2376AA	131	653
ESC_QA2375AA	48	76

ESC_QA2374AA	355	764
ESC_QA2373AA	38	669
ESC_QA2372AA	1011	198
ESC_QA2371AA	752	512
ESC_QA2370AA	58	198
ESC_QA2369AA	48	76
ESC_QA2368AA	10	512
ESC_QA2367AA	162	924
ESC_QA2366AA	7804	369
ESC_QA2365AA	1148	257
ESC_QA2364AA	349	670
ESC_QA2363AA	155	175
ESC_QA2362AA	155	175
ESC_QA2361AA	131	653
ESC_QA2360AA	23	156
ESC_QA2359AA	58	198
ESC_QA2358AA	648	359
ESC_QA2357AA	355	764
ESC_QA2356AA	162	924
ESC_QA2355AA	162	924
ESC_QA2354AA	48	76
ESC_QA2353AA	162	924
ESC_QA2352AA	48	76
ESC_QA2351AA	69	387
ESC_QA2350AA	43	512
ESC_QA2349AA	43	512
ESC_QA2348AA	752	512
ESC_QA2347AA	58	198
ESC_QA2346AA	10	512
ESC_QA2345AA	10	512
ESC_QA2344AA	10	512
ESC_QA2343AA	10	512
ESC_QA2342AA	602	257
ESC_QA2341AA	58	198
ESC_QA2340AA	744	512
ESC_QA2339AA	206	286
ESC_QA2338AA	2165	257
ESC_QA2337AA	162	924
ESC_QA2336AA	58	198
ESC_QA2332AA	101	696
ESC_QA2331AA	117	592
ESC_QA2330AA	10	512
ESC_QA2329AA	10	512
ESC_QA2328AA	602	257
ESC_QA2327AA	10	512
ESC_QA2326AA	43	512
ESC_QA2325AA	602	257
ESC_QA2324AA	2079	198
ESC_QA2323AA	8720	592
ESC_QA2322AA	1056	257

ESC_QA2321AA	162	924
ESC_QA2320AA	162	924
ESC_QA2319AA	4994	584
ESC_QA2318AA	58	198
ESC_QA2316AA	58	198
ESC_QA2315AA	10	512
ESC_QA2314AA	162	924
ESC_QA2313AA	1011	198
ESC_QA2312AA	1246	198
ESC_QA2311AA	118	985
ESC_QA2310AA	10	512
ESC_QA2309AA	3190	221
ESC_QA2308AA	155	175
ESC_QA2307AA	155	175
ESC_QA2306AA	355	764
ESC_QA2305AA	115	669
ESC_QA2304AA	3107	512
ESC_QA2303AA	162	924
ESC_QA2302AA	162	924
ESC_QA2301AA	453	935
ESC_QA2300AA	10	512
ESC_QA2299AA	752	512
ESC_QA2298AA	162	924
ESC_QA2297AA	453	935
ESC_QA2296AA	453	935
ESC_QA2295AA	191	198
ESC_QA2294AA	355	764
ESC_QA2293AA	155	175
ESC_QA2292AA	10	512
ESC_QA2291AA	453	935
ESC_QA2290AA	453	935
ESC_QA2289AA	355	764
ESC_QA2288AA	10	512
ESC_QA2287AA	117	592
ESC_QA2286AA	10	512
ESC_QA2285AA	10	512
ESC_QA2284AA	10	512
ESC_QA2283AA	10	512
ESC_QA2282AA	10	512
ESC_QA2281AA	10	512
ESC_QA2280AA	10	512
ESC_QA2279AA	10	512
ESC_QA2278AA	10	512
ESC_QA2277AA	10	512
ESC_QA2276AA	10	512
ESC_QA2275AA	10	512
ESC_QA2274AA	10	512
ESC_QA2273AA	10	512
ESC_QA2272AA	10	512
ESC_QA2271AA	10	512

ESC_QA2270AA	10	512
ESC_QA2269AA	10	512
ESC_QA2268AA	10	512
ESC_QA2267AA	10	512
ESC_QA2266AA	10	512
ESC_QA2265AA	10	512
ESC_QA2264AA	10	512
ESC_QA2263AA	10	512
ESC_QA2262AA	10	512
ESC_QA2261AA	10	512
ESC_QA2260AA	10	512
ESC_QA2259AA	10	512
ESC_QA2258AA	10	512
ESC_QA2257AA	10	512
ESC_QA2256AA	10	512
ESC_QA2255AA	10	512
ESC_QA2254AA	10	512
ESC_QA2253AA	10	512
ESC_QA2252AA	10	512
ESC_QA2251AA	10	512
ESC_QA2250AA	10	512
ESC_QA2249AA	10	512
ESC_QA2248AA	10	512
ESC_QA2247AA	10	512
ESC_QA2246AA	10	512
ESC_QA2245AA	648	359
ESC_QA2244AA	648	359
ESC_QA2243AA	648	359
ESC_QA2242AA	648	359
ESC_QA2241AA	648	359
ESC_QA2240AA	648	359
ESC_QA2239AA	12	626
ESC_QA2238AA	12	626
ESC_QA2237AA	12	626
ESC_QA2236AA	12	626
ESC_QA2235AA	12	626
ESC_QA2234AA	12	626
ESC_QA2231AA	752	512
ESC_QA2229AA	93	71
ESC_QA2228AA	349	670
ESC_QA2227AA	3107	512
ESC_QA2226AA	355	764
ESC_QA2225AA	10	512
ESC_QA2224AA	752	512
ESC_QA2223AA	602	257
ESC_QA2222AA	10	512
ESC_QA2221AA	10	512
ESC_QA2220AA	355	764
ESC_QA2219AA	162	924
ESC_QA2218AA	115	669

ESC_QA2217AA	117	592
ESC_QA2216AA	349	670
ESC_QA2215AA	349	670
ESC_QA2214AA	355	764
ESC_QA2213AA	355	764
ESC_QA2212AA	355	764
ESC_QA2211AA	355	764
ESC_QA2210AA	355	764
ESC_QA2209AA	355	764
ESC_QA2208AA	355	764
ESC_QA2207AA	641	935
ESC_QA2206AA	10	512
ESC_QA2205AA	34	504
ESC_QA2204AA	155	175
ESC_QA2203AA	58	198
ESC_QA2202AA	117	592
ESC_QA2201AA	117	592
ESC_QA2200AA	744	512
ESC_QA2199AA	48	76
ESC_QA2198AA	48	76
ESC_QA2197AA	10	512
ESC_QA2196AA	10	512
ESC_QA2195AA	10	512
ESC_QA2194AA	10	512
ESC_QA2193AA	685	311
ESC_QA2192AA	744	512
ESC_QA2191AA	58	198
ESC_QA2190AA	744	512
ESC_QA2189AA	100	504
ESC_QA2188AA	744	512
ESC_QA2187AA	10	512
ESC_QA2185AA	11	936
ESC_QA2184AA	109	257
ESC_QA2183AA	392	234
ESC_QA2182AA	515	1004
ESC_QA2181AA	32	270
ESC_QA2180AA	343	286
ESC_QA2179AA	1122	307
ESC_QA2178AA	10	512
ESC_QA2177AA	10693	785
ESC_QA2176AA	1248	156
ESC_QA2175AA	9964	1004
ESC_QA2174AA	336	878
ESC_QA2173AA	10	512
ESC_QA2172AA	32	270
ESC_QA2171AA	755	261
ESC_QA2170AA	10	512
ESC_QA2169AA	1406	174
ESC_QA2168AA	154	257
ESC_QA2167AA	10	512

ESC_QA2166AA	446	257
ESC_QA2165AA	32	270
ESC_QA2164AA	1113	221
ESC_QA2163AA	8935	101
ESC_QA2162AA	1415	504
ESC_QA2161AA	5082	198
ESC_QA2160AA	10697	935
ESC_QA2159AA	13	257
ESC_QA2158AA	10	512
ESC_QA2157AA	109	257
ESC_QA2156AA	10696	311
ESC_QA2155AA	101	696
ESC_QA2154AA	155	175
ESC_QA2153AA	10066	198
ESC_QA2152AA	155	175
ESC_QA2151AA	3090	212
ESC_QA2150AA	6521	842
ESC_QA2149AA	6998	629
ESC_QA2148AA	17	191
ESC_QA2147AA	300	286
ESC_QA2146AA	10695	805
ESC_QA2145AA	442	257
ESC_QA2144AA	17	191
ESC_QA2143AA	29	156
ESC_QA2142AA	21	156
ESC_QA2141AA	10	512
ESC_QA2140AA	297	234
ESC_QA2139AA	602	257
ESC_QA2138AA	58	198
ESC_QA2137AA	998	644
ESC_QA2136AA	5308	261
ESC_QA2135AA	164	175
ESC_QA2134AA	10	512
ESC_QA2133AA	399	198
ESC_QA2132AA	10694	141
ESC_QA2130AA	10066	198
ESC_QA2129AA	1735	737
ESC_QA2128AA	4151	971
ESC_QA2127AA	73	737
ESC_QA2126AA	372	424
ESC_QA2125AA	961	623
ESC_QA2124AA	355	764
ESC_QA2123AA	4550	150
ESC_QA2122AA	70	670
ESC_QA2121AA	4138	198
ESC_QA2120AA	155	175
ESC_QA2119AA	1060	512
ESC_QA2118AA	1079	235
ESC_QA2117AA	1079	235
ESC_QA2116AA	1060	512

ESC_QA2115AA	10	512
ESC_QA2114AA	1060	512
ESC_QA2113AA	1060	512
ESC_QA2112AA	1060	512
ESC_QA2111AA	2035	113
ESC_QA2110AA	7440	198
ESC_QA2109AA	10753	629
ESC_QA2108AA	10	512
ESC_QA2107AA	1060	512
ESC_QA2106AA	1060	512
ESC_QA2105AA	1060	512
ESC_QA2104AA	4527	880
ESC_QA2103AA	1060	512
ESC_QA2102AA	10	512
ESC_QA2101AA	1277	42
ESC_QA2100AA	10	512
ESC_QA2099AA	10	512
ESC_QA2098AA	1250	926
ESC_QA2097AA	10	512
ESC_QA2096AA	58	198
ESC_QA2095AA	34	504
ESC_QA2094AA	7440	198
ESC_QA2093AA	1245	270
ESC_QA2092AA	1060	512
ESC_QA2091AA	7440	198
ESC_QA2090AA	10	512
ESC_QA2089AA	10	512
ESC_QA2088AA	10	512
ESC_QA2087AA	10	512
ESC_QA2086AA	10	512
ESC_QA2085AA	1250	926
ESC_QA2084AA	10	512
ESC_QA2083AA	10	512
ESC_QA2082AA	2040	198
ESC_QA2081AA	1060	512
ESC_QA2080AA	641	935
ESC_QA2079AA	10	512
ESC_QA2078AA	10	512
ESC_QA2077AA	641	935
ESC_QA2076AA	1250	926
ESC_QA2075AA	1060	512
ESC_QA2074AA	10	512
ESC_QA2073AA	10	512
ESC_QA2072AA	1245	270
ESC_QA2071AA	11	936
ESC_QA2070AA	69	387
ESC_QA2069AA	69	387
ESC_QA2068AA	69	387
ESC_QA2067AA	404	761
ESC_QA2066AA	117	592

ESC_QA2065AA	963	669
ESC_QA2064AA	131	653
ESC_QA2063AA	10754	785
ESC_QA2062AA	155	175
ESC_QA2061AA	38	669
ESC_QA2060AA	38	669
ESC_QA2059AA	57	50
ESC_QA2058AA	38	669
ESC_QA2057AA	95	738
ESC_QA2056AA	155	175
ESC_QA2055AA	155	175
ESC_QA2054AA	38	669
ESC_QA2052AA	155	175
ESC_QA2051AA	1640	50
ESC_QA2050AA	101	696
ESC_QA2049AA	38	669
ESC_QA2048AA	155	175
ESC_QA2047AA	155	175
ESC_QA2046AA	2852	257
ESC_QA2044AA	155	175
ESC_QA2043AA	155	175
ESC_QA2042AA	155	175
ESC_QA2041AA	362	234
ESC_QA2040AA	362	234
ESC_QA2039AA	155	175
ESC_QA2038AA	155	175
ESC_QA2036AA	350	50
ESC_QA2034AA	38	669
ESC_QA2033AA	155	175
ESC_QA2032AA	155	175
ESC_QA2031AA	38	669
ESC_QA2030AA	155	175
ESC_QA2029AA	38	669
ESC_QA2027AA	38	669
ESC_QA2024AA	154	257
ESC_QA2023AA	38	669
ESC_QA2022AA	38	669
ESC_QA2021AA	38	669
ESC_QA2020AA	38	669
ESC_QA2019AA	155	175
ESC_QA2018AA	155	175
ESC_QA2017AA	38	669
ESC_QA2016AA	38	669
ESC_QA2015AA	38	669
ESC_QA2014AA	117	592
ESC_QA2013AA	117	592
ESC_QA2012AA	141	644
ESC_QA2011AA	117	592
ESC_QA2010AA	155	175
ESC_QA2009AA	155	175

ESC_QA2008AA	155	175
ESC_QA2005AA	155	175
ESC_QA2003AA	2473	257
ESC_QA2002AA	348	175
ESC_QA2001AA	297	234
ESC_QA2000AA	2774	257
ESC_QA1999AA	10	512
ESC_QA1998AA	10755	80
ESC_QA1997AA	906	198
ESC_QA1996AA	10	512
ESC_QA1995AA	10	512
ESC_QA1994AA	10	512
ESC_QA1993AA	10	512
ESC_QA1992AA	10	512
ESC_QA1991AA	6234	257
ESC_QA1990AA	10	512
ESC_QA1989AA	42	257
ESC_QA1988AA	10	512
ESC_QA1987AA	10757	257
ESC_QA1986AA	10	512
ESC_QA1984AA	10	512
ESC_QA1983AA	10756	731
ESC_QA1982AA	17	191
ESC_QA1981AA	21	156
ESC_QA1980AA	8825	304
ESC_QA1979AA	394	400
ESC_QA1978AA	738	598
ESC_QA1977AA	17	191
ESC_QA1976AA	21	156
ESC_QA1975AA	641	935
ESC_QA1974AA	21	156
ESC_QA1973AA	2772	257
ESC_QA1972AA	17	191
ESC_QA1971AA	442	257
ESC_QA1970AA	8825	304
ESC_QA1969AA	349	670
ESC_QA1968AA	21	156
ESC_QA1967AA	738	598
ESC_QA1966AA	21	156
ESC_QA1965AA	34	504
ESC_QA1964AA	8825	304
ESC_QA1963AA	5133	504
ESC_QA1962AA	21	156
ESC_QA1960AA	381	156
ESC_QA1959AA	11	936
ESC_QA1958AA	11	936
ESC_QA1957AA	95	738
ESC_QA1956AA	101	696
ESC_QA1953AA	765	156
ESC_QA1952AA	398	445

ESC_QA1951AA	101	696
ESC_QA1950AA	2328	257
ESC_QA1949AA	906	198
ESC_QA1948AA	7715	257
ESC_QA1947AA	8217	257
ESC_QA1946AA	8217	257
ESC_QA1945AA	10598	257
ESC_QA1944AA	10760	596
ESC_QA1943AA	4623	257
ESC_QA1942AA	10758	504
ESC_QA1941AA	2280	212
ESC_QA1940AA	17	191
ESC_QA1939AA	38	669
ESC_QA1938AA	155	175
ESC_QA1937AA	2485	212
ESC_QA1936AA	5765	234
ESC_QA1935AA	10783	198
ESC_QA1934AA	10598	257
ESC_QA1933AA	10759	257
ESC_QA1932AA	765	156
ESC_QA1931AA	38	669
ESC_QA1930AA	58	198
ESC_QA1929AA	4392	234
ESC_QA1928AA	5768	569
ESC_QA1927AA	2325	447
ESC_QA1926AA	10761	993
ESC_QA1925AA	11	936
ESC_QA1924AA	10	512
ESC_QA1923AA	906	198
ESC_QA1922AA	5309	286
ESC_QA1921AA	10	512
ESC_QA1920AA	998	644
ESC_QA1919AA	100	504
ESC_QA1918AA	10	512
ESC_QA1917AA	1721	311
ESC_QA1916AA	942	198
ESC_QA1915AA	100	504
ESC_QA1914AA	100	504
ESC_QA1913AA	6475	637
ESC_QA1912AA	90	156
ESC_QA1911AA	100	504
ESC_QA1910AA	2144	257
ESC_QA1909AA	56	198
ESC_QA1908AA	16	156
ESC_QA1907AA	224	198
ESC_QA1906AA	3580	257
ESC_QA1905AA	8264	586
ESC_QA1904AA	100	504
ESC_QA1903AA	8217	257
ESC_QA1902AA	100	504

ESC_QA1901AA	443	257
ESC_QA1900AA	4576	987
ESC_QA1899AA	410	156
ESC_QA1898AA	3580	257
ESC_QA1897AA	2485	212
ESC_QA1896AA	11	936
ESC_QA1895AA	2728	198
ESC_QA1894AA	100	504
ESC_QA1893AA	6234	257
ESC_QA1892AA	10	512
ESC_QA1891AA	10614	926
ESC_QA1890AA	10	512
ESC_QA1889AA	10	512
ESC_QA1888AA	8496	382
ESC_QA1887AA	90	156
ESC_QA1886AA	677	257
ESC_QA1885AA	10	512
ESC_QA1884AA	12	626
ESC_QA1883AA	8217	257
ESC_QA1882AA	2715	588
ESC_QA1881AA	42	257
ESC_QA1876AA	6234	257
ESC_QA1875AA	11	936
ESC_QA1874AA	10	512
ESC_QA1873AA	297	234
ESC_QA1872AA	372	424
ESC_QA1871AA	372	424
ESC_QA1870AA	12	626
ESC_QA1869AA	1196	257
ESC_QA1868AA	744	512
ESC_QA1867AA	410	156
ESC_QA1866AA	372	424
ESC_QA1865AA	224	198
ESC_QA1864AA	6688	156
ESC_QA1863AA	200	198
ESC_QA1862AA	404	761
ESC_QA1861AA	25	234
ESC_QA1860AA	295	160
ESC_QA1859AA	300	286
ESC_QA1858AA	295	160
ESC_QA1857AA	33	198
ESC_QA1856AA	6688	156
ESC_QA1855AA	33	198
ESC_QA1854AA	10	512
ESC_QA1853AA	661	92
ESC_QA1850AA	32	270
ESC_QA1849AA	3695	924
ESC_QA1845AA	10	512
ESC_QA1844AA	10	512
ESC_QA1843AA	131	653

ESC_QA1842AA	131	653
ESC_QA1841AA	10762	175
ESC_QA1840AA	131	653
ESC_QA1839AA	131	653
ESC_QA1838AA	131	653
ESC_QA1837AA	131	653
ESC_QA1836AA	10762	175
ESC_QA1835AA	131	653
ESC_QA1834AA	38	669
ESC_QA1833AA	131	653
ESC_QA1832AA	131	653
ESC_QA1831AA	131	653
ESC_QA1830AA	131	653
ESC_QA1829AA	131	653
ESC_QA1828AA	131	653
ESC_QA1827AA	131	653
ESC_QA1826AA	131	653
ESC_QA1825AA	131	653
ESC_QA1824AA	131	653
ESC_QA1823AA	131	653
ESC_QA1822AA	131	653
ESC_QA1820AA	5292	738
ESC_QA1819AA	300	286
ESC_QA1818AA	2952	926
ESC_QA1817AA	12	626
ESC_QA1816AA	8526	696
ESC_QA1815AA	8550	72
ESC_QA1814AA	681	393
ESC_QA1813AA	73	737
ESC_QA1812AA	681	393
ESC_QA1811AA	349	670
ESC_QA1810AA	677	257
ESC_QA1809AA	677	257
ESC_QA1807AA	677	257
ESC_QA1806AA	677	257
ESC_QA1805AA	8527	737
ESC_QA1804AA	8527	737
ESC_QA1802AA	3306	630
ESC_QA1800AA	73	737
ESC_QA1799AA	73	737
ESC_QA1797AA	73	737
ESC_QA1796AA	73	737
ESC_QA1795AA	73	737
ESC_QA1794AA	73	737
ESC_QA1793AA	73	737
ESC_QA1790AA	1873	769
ESC_QA1789AA	1873	769
ESC_QA1788AA	10784	299
ESC_QA1787AA	196	257
ESC_QA1786AA	196	257

ESC_QA1784AA	2076	400
ESC_QA1781AA	681	393
ESC_QA1779AA	226	512
ESC_QA1778AA	226	512
ESC_QA1777AA	5073	900
ESC_QA1776AA	226	512
ESC_QA1775AA	126	740
ESC_QA1773AA	442	257
ESC_QA1770AA	362	234
ESC_QA1766AA	5780	476
ESC_QA1765AA	1727	257
ESC_QA1764AA	5780	476
ESC_QA1763AA	8532	471
ESC_QA1762AA	212	198
ESC_QA1761AA	212	198
ESC_QA1759AA	681	393
ESC_QA1758AA	212	198
ESC_QA1757AA	8533	739
ESC_QA1755AA	8533	739
ESC_QA1754AA	38	669
ESC_QA1753AA	8533	739
ESC_QA1751AA	676	737
ESC_QA1750AA	676	737
ESC_QA1749AA	676	737
ESC_QA1748AA	681	393
ESC_QA1747AA	8535	257
ESC_QA1746AA	8535	257
ESC_QA1745AA	336	878
ESC_QA1743AA	336	878
ESC_QA1741AA	336	878
ESC_QA1739AA	1204	961
ESC_QA1738AA	8826	926
ESC_QA1736AA	1204	961
ESC_QA1735AA	1431	257
ESC_QA1733AA	6316	257
ESC_QA1732AA	1431	257
ESC_QA1731AA	4080	300
ESC_QA1730AA	2521	257
ESC_QA1729AA	8827	198
ESC_QA1728AA	1204	961
ESC_QA1727AA	8525	917
ESC_QA1726AA	681	393
ESC_QA1725AA	8825	304
ESC_QA1724AA	127	738
ESC_QA1723AA	127	738
ESC_QA1721AA	127	738
ESC_QA1720AA	127	738
ESC_QA1719AA	73	737
ESC_QA1718AA	73	737
ESC_QA1715AA	32	270

ESC_QA1714AA	648	359
ESC_QA1713AA	200	198
ESC_QA1712AA	101	696
ESC_QA1711AA	58	198
ESC_QA1710AA	5876	847
ESC_QA1709AA	349	670
ESC_QA1708AA	101	696
ESC_QA1707AA	1896	380
ESC_QA1706AA	10764	444
ESC_QA1705AA	155	175
ESC_QA1704AA	1704	257
ESC_QA1700AA	131	653
ESC_QA1699AA	372	424
ESC_QA1698AA	457	595
ESC_QA1697AA	1336	424
ESC_QA1696AA	127	738
ESC_QA1695AA	4279	900
ESC_QA1694AA	1735	737
ESC_QA1693AA	28	537
ESC_QA1692AA	372	424
ESC_QA1691AA	372	424
ESC_QA1690AA	372	424
ESC_QA1689AA	73	737
ESC_QA1688AA	10763	737
ESC_QA1687AA	127	738
ESC_QA1686AA	73	737
ESC_QA1685AA	1284	604
ESC_QA1684AA	940	257
ESC_QA1683AA	167	512
ESC_QA1682AA	410	156
ESC_QA1681AA	940	257
ESC_QA1680AA	656	789
ESC_QA1679AA	648	359
ESC_QA1678AA	410	156
ESC_QA1677AA	205	257
ESC_QA1676AA	167	512
ESC_QA1675AA	40	198
ESC_QA1674AA	4657	175
ESC_QA1673AA	17	191
ESC_QA1653AA	8296	198
ESC_QA1652AA	4213	251
ESC_QA1651AA	504	738
ESC_QA1650AA	841	204
ESC_QA1649AA	32	270
ESC_QA1648AA	10	512
ESC_QA1647AA	278	924
ESC_QA1646AA	21	156
ESC_QA1645AA	21	156
ESC_QA1644AA	29	156
ESC_QA1643AA	16	156

ESC_QA1642AA	32	270
ESC_QA1641AA	25	234
ESC_QA1640AA	21	156
ESC_QA1639AA	137	270
ESC_QA1638AA	23	156
ESC_QA1637AA	56	198
ESC_QA1636AA	372	424
ESC_QA1635AA	372	424
ESC_QA1634AA	90	156
ESC_QA1633AA	90	156
ESC_QA1632AA	8755	257
ESC_QA1631AA	1491	76
ESC_QA1630AA	1491	76
ESC_QA1629AA	1491	76
ESC_QA1628AA	414	387
ESC_QA1627AA	1491	76
ESC_QA1626AA	1491	76
ESC_QA1621AA	414	387
ESC_QA1620AA	1491	76
ESC_QA1618AA	442	257
ESC_QA1617AA	17	191
ESC_QA1616AA	447	198
ESC_QA1615AA	6	445
ESC_QA1614AA	1819	808
ESC_QA1613AA	32	270
ESC_QA1612AA	1064	756
ESC_QA1611AA	25	234
ESC_QA1610AA	447	198
ESC_QA1609AA	32	270
ESC_QA1608AA	17	191
ESC_QA1607AA	73	737
ESC_QA1606AA	38	669
ESC_QA1605AA	73	737
ESC_QA1604AA	38	669
ESC_QA1603AA	73	737
ESC_QA1602AA	38	669
ESC_QA1601AA	73	737
ESC_QA1600AA	38	669
ESC_QA1599AA	6316	257
ESC_QA1598AA	7250	919
ESC_QA1597AA	131	653
ESC_QA1596AA	10	512
ESC_QA1595AA	162	924
ESC_QA1594AA	2521	257
ESC_QA1593AA	1880	653
ESC_QA1592AA	10	512
ESC_QA1591AA	10	512
ESC_QA1590AA	86	935
ESC_QA1589AA	58	198
ESC_QA1588AA	165	504

ESC_QA1587AA	5784	448
ESC_QA1586AA	602	257
ESC_QA1585AA	4162	180
ESC_QA1584AA	130	387
ESC_QA1583AA	4162	180
ESC_QA1582AA	967	212
ESC_QA1581AA	1946	726
ESC_QA1580AA	967	212
ESC_QA1579AA	2562	640
ESC_QA1578AA	1079	235
ESC_QA1577AA	744	512
ESC_QA1576AA	10	512
ESC_QA1575AA	744	512
ESC_QA1574AA	196	257
ESC_QA1573AA	162	924
ESC_QA1572AA	2607	198
ESC_QA1571AA	2144	257
ESC_QA1570AA	10752	370
ESC_QA1569AA	101	696
ESC_QA1568AA	21	156
ESC_QA1567AA	5390	83
ESC_QA1566AA	25	234
ESC_QA1565AA	738	598
ESC_QA1564AA	21	156
ESC_QA1563AA	33	198
ESC_QA1562AA	394	400
ESC_QA1561AA	21	156
ESC_QA1560AA	21	156
ESC_QA1559AA	442	257
ESC_QA1558AA	8825	304
ESC_QA1557AA	21	156
ESC_QA1556AA	32	270
ESC_QA1555AA	8825	304
ESC_QA1554AA	297	234
ESC_QA1553AA	278	924
ESC_QA1552AA	8825	304
ESC_QA1532AA	10	512
ESC_QA1531AA	10	512
ESC_QA1530AA	10	512
ESC_QA1529AA	10	512
ESC_QA1528AA	10	512
ESC_QA1527AA	10	512
ESC_QA1526AA	10	512
ESC_QA1525AA	10	512
ESC_QA1524AA	10	512
ESC_QA1523AA	10	512
ESC_QA1522AA	10	512
ESC_QA1521AA	10	512
ESC_QA1520AA	10	512
ESC_QA1519AA	10	512

ESC_QA1517AA	10	512
ESC_QA1516AA	10	512
ESC_QA1515AA	10	512
ESC_QA1514AA	10	512
ESC_QA1513AA	10	512
ESC_QA1512AA	10	512
ESC_QA1511AA	10	512
ESC_QA1510AA	10	512
ESC_QA1509AA	10	512
ESC_QA1508AA	10	512
ESC_QA1507AA	10	512
ESC_QA1506AA	10	512
ESC_QA1505AA	10	512
ESC_QA1504AA	10	512
ESC_QA1503AA	10	512
ESC_QA1502AA	10	512
ESC_QA1501AA	10	512
ESC_QA1500AA	10	512
ESC_QA1499AA	10	512
ESC_QA1498AA	10	512
ESC_QA1497AA	10	512
ESC_QA1494AA	306	286
ESC_QA1493AA	127	738
ESC_QA1492AA	372	424
ESC_QA1491AA	372	424
ESC_QA1490AA	73	737
ESC_QA1489AA	372	424
ESC_QA1488AA	3601	653
ESC_QA1487AA	10766	419
ESC_QA1486AA	127	738
ESC_QA1485AA	2650	738
ESC_QA1484AA	12	626
ESC_QA1483AA	155	175
ESC_QA1482AA	1431	257
ESC_QA1481AA	372	424
ESC_QA1480AA	10765	870
ESC_QA1479AA	5683	286
ESC_QA1478AA	372	424
ESC_QA1477AA	141	644
ESC_QA1472AA	1753	261
ESC_QA1471AA	245	261
ESC_QA1469AA	245	261
ESC_QA1468AA	245	261
ESC_QA1467AA	245	261
ESC_QA1466AA	245	261
ESC_QA1465AA	152	257
ESC_QA1464AA	10692	102
ESC_QA1463AA	245	261
ESC_QA1462AA	152	257
ESC_QA1461AA	152	257

ESC_QA1460AA	245	261
ESC_QA1459AA	5475	256
ESC_QA1458AA	152	257
ESC_QA1457AA	152	257
ESC_QA1456AA	145	256
ESC_QA1455AA	152	257
ESC_QA1454AA	152	257
ESC_QA1453AA	245	261
ESC_QA1452AA	145	256
ESC_QA1451AA	152	257
ESC_QA1450AA	152	257
ESC_QA1449AA	245	261
ESC_QA1448AA	245	261
ESC_QA1447AA	245	261
ESC_QA1446AA	245	261
ESC_QA1445AA	245	261
ESC_QA1444AA	145	256
ESC_QA1443AA	152	257
ESC_QA1442AA	152	257
ESC_QA1441AA	145	256
ESC_QA1440AA	245	261
ESC_QA1439AA	152	257
ESC_QA1438AA	152	257
ESC_QA1437AA	152	257
ESC_QA1436AA	252	190
ESC_QA1435AA	245	261
ESC_QA1434AA	152	257
ESC_QA1433AA	245	261
ESC_QA1432AA	245	261
ESC_QA1431AA	152	257
ESC_QA1430AA	152	257
ESC_QA1429AA	245	261
ESC_QA1428AA	245	261
ESC_QA1427AA	152	257
ESC_QA1426AA	152	257
ESC_QA1425AA	152	257
ESC_QA1424AA	152	257
ESC_QA1423AA	245	261
ESC_QA1422AA	245	261
ESC_QA1421AA	152	257
ESC_QA1420AA	152	257
ESC_QA1419AA	245	261
ESC_QA1418AA	152	257
ESC_QA1417AA	288	102
ESC_QA1416AA	152	257
ESC_QA1415AA	152	257
ESC_QA1414AA	245	261
ESC_QA1413AA	152	257
ESC_QA1412AA	11	936
ESC_QA1411AA	11	936

ESC_QA1410AA	301	307
ESC_QA1409AA	152	257
ESC_QA1408AA	145	256
ESC_QA1407AA	245	261
ESC_QA1406AA	152	257
ESC_QA1405AA	152	257
ESC_QA1404AA	245	261
ESC_QA1403AA	152	257
ESC_QA1402AA	152	257
ESC_QA1401AA	152	257
ESC_QA1400AA	152	257
ESC_QA1399AA	245	261
ESC_QA1398AA	245	261
ESC_QA1397AA	152	257
ESC_QA1396AA	152	257
ESC_QA1395AA	145	256
ESC_QA1394AA	145	256
ESC_QA1393AA	245	261
ESC_QA1392AA	152	257
ESC_QA1391AA	152	257
ESC_QA1390AA	145	256
ESC_QA1389AA	152	257
ESC_QA1388AA	145	256
ESC_QA1387AA	152	257
ESC_QA1386AA	152	257
ESC_QA1385AA	152	257
ESC_QA1384AA	152	257
ESC_QA1383AA	152	257
ESC_QA1382AA	245	261
ESC_QA1381AA	152	257
ESC_QA1380AA	152	257
ESC_QA1379AA	152	257
ESC_QA1378AA	152	257
ESC_QA1377AA	152	257
ESC_QA1376AA	245	261
ESC_QA1375AA	245	261
ESC_QA1374AA	245	261
ESC_QA1373AA	245	261
ESC_QA1372AA	152	257
ESC_QA1371AA	152	257
ESC_QA1370AA	245	261
ESC_QA1369AA	152	257
ESC_QA1368AA	152	257
ESC_QA1367AA	152	257
ESC_QA1366AA	152	257
ESC_QA1365AA	630	261
ESC_QA1364AA	245	261
ESC_QA1363AA	245	261
ESC_QA1362AA	152	257
ESC_QA1361AA	152	257

ESC_QA1360AA	152	257
ESC_QA1359AA	245	261
ESC_QA1358AA	245	261
ESC_QA1357AA	152	257
ESC_QA1356AA	152	257
ESC_QA1355AA	152	257
ESC_QA1354AA	152	257
ESC_QA1353AA	152	257
ESC_QA1352AA	245	261
ESC_QA1351AA	628	261
ESC_QA1350AA	152	257
ESC_QA1349AA	152	257
ESC_QA1348AA	245	261
ESC_QA1347AA	245	261
ESC_QA1346AA	245	261
ESC_QA1345AA	245	261
ESC_QA1344AA	152	257
ESC_QA1343AA	152	257
ESC_QA1342AA	245	261
ESC_QA1341AA	6200	261
ESC_QA1340AA	152	257
ESC_QA1339AA	152	257
ESC_QA1338AA	152	257
ESC_QA1337AA	152	257
ESC_QA1336AA	152	257
ESC_QA1335AA	152	257
ESC_QA1334AA	152	257
ESC_QA1333AA	152	257
ESC_QA1332AA	152	257
ESC_QA1331AA	152	257
ESC_QA1330AA	152	257
ESC_QA1329AA	152	257
ESC_QA1328AA	152	257
ESC_QA1327AA	152	257
ESC_QA1326AA	152	257
ESC_QA1325AA	152	257
ESC_QA1324AA	152	257
ESC_QA1323AA	152	257
ESC_QA1322AA	245	261
ESC_QA1321AA	152	257
ESC_QA1320AA	245	261
ESC_QA1319AA	245	261
ESC_QA1318AA	245	261
ESC_QA1317AA	152	257
ESC_QA1316AA	152	257
ESC_QA1315AA	152	257
ESC_QA1314AA	152	257
ESC_QA1313AA	245	261
ESC_QA1312AA	152	257
ESC_QA1311AA	152	257

ESC_QA1310AA	152	257
ESC_QA1309AA	152	257
ESC_QA1308AA	152	257
ESC_QA1307AA	152	257
ESC_QA1306AA	245	261
ESC_QA1305AA	152	257
ESC_QA1304AA	152	257
ESC_QA1303AA	152	257
ESC_QA1302AA	243	197
ESC_QA1301AA	145	256
ESC_QA1300AA	245	261
ESC_QA1299AA	152	257
ESC_QA1298AA	152	257
ESC_QA1297AA	152	257
ESC_QA1296AA	152	257
ESC_QA1295AA	152	257
ESC_QA1294AA	145	256
ESC_QA1293AA	152	257
ESC_QA1292AA	152	257
ESC_QA1291AA	152	257
ESC_QA1290AA	152	257
ESC_QA1289AA	152	257
ESC_QA1288AA	152	257
ESC_QA1287AA	152	257
ESC_QA1286AA	152	257
ESC_QA1285AA	152	257
ESC_QA1284AA	152	257
ESC_QA1283AA	152	257
ESC_QA1282AA	152	257
ESC_QA1281AA	152	257
ESC_QA1280AA	152	257
ESC_QA1279AA	152	257
ESC_QA1278AA	152	257
ESC_QA1277AA	152	257
ESC_QA1276AA	152	257
ESC_QA1275AA	152	257
ESC_QA1274AA	152	257
ESC_QA1273AA	152	257
ESC_QA1272AA	152	257
ESC_QA1271AA	152	257
ESC_QA1270AA	152	257
ESC_QA1269AA	152	257
ESC_QA1268AA	152	257
ESC_QA1267AA	152	257
ESC_QA1266AA	152	257
ESC_QA1265AA	145	256
ESC_QA1264AA	245	261
ESC_QA1263AA	152	257
ESC_QA1262AA	152	257
ESC_QA1261AA	152	257

ESC_QA1260AA	152	257
ESC_QA1259AA	152	257
ESC_QA1258AA	5517	257
ESC_QA1257AA	152	257
ESC_QA1256AA	152	257
ESC_QA1255AA	145	256
ESC_QA1254AA	145	256
ESC_QA1253AA	152	257
ESC_QA1252AA	152	257
ESC_QA1251AA	152	257
ESC_QA1250AA	152	257
ESC_QA1249AA	245	261
ESC_QA1248AA	152	257
ESC_QA1247AA	152	257
ESC_QA1246AA	152	257
ESC_QA1245AA	152	257
ESC_QA1244AA	1025	261
ESC_QA1243AA	152	257
ESC_QA1242AA	152	257
ESC_QA1241AA	152	257
ESC_QA1240AA	152	257
ESC_QA1239AA	152	257
ESC_QA1238AA	245	261
ESC_QA1237AA	152	257
ESC_QA1236AA	152	257
ESC_QA1235AA	1025	261
ESC_QA1234AA	152	257
ESC_QA1233AA	245	261
ESC_QA1232AA	245	261
ESC_QA1231AA	6200	261
ESC_QA1230AA	245	261
ESC_QA1229AA	152	257
ESC_QA1228AA	245	261
ESC_QA1227AA	145	256
ESC_QA1226AA	245	261
ESC_QA1225AA	245	261
ESC_QA1224AA	152	257
ESC_QA1223AA	152	257
ESC_QA1222AA	152	257
ESC_QA1221AA	152	257
ESC_QA1220AA	245	261
ESC_QA1219AA	152	257
ESC_QA1218AA	152	257
ESC_QA1217AA	152	257
ESC_QA1216AA	152	257
ESC_QA1215AA	152	257
ESC_QA1214AA	152	257
ESC_QA1213AA	245	261
ESC_QA1212AA	152	257
ESC_QA1211AA	152	257

ESC_QA1210AA	145	256
ESC_QA1209AA	152	257
ESC_QA1208AA	152	257
ESC_QA1207AA	148	191
ESC_QA1206AA	145	256
ESC_QA1205AA	152	257
ESC_QA1204AA	152	257
ESC_QA1203AA	152	257
ESC_QA1202AA	245	261
ESC_QA1201AA	245	261
ESC_QA1200AA	147	102
ESC_QA1199AA	152	257
ESC_QA1198AA	152	257
ESC_QA1197AA	145	256
ESC_QA1196AA	152	257
ESC_QA1195AA	152	257
ESC_QA1194AA	245	261
ESC_QA1193AA	152	257
ESC_QA1192AA	245	261
ESC_QA1191AA	245	261
ESC_QA1190AA	152	257
ESC_QA1189AA	245	261
ESC_QA1188AA	245	261
ESC_QA1187AA	152	257
ESC_QA1186AA	152	257
ESC_QA1185AA	152	257
ESC_QA1184AA	152	257
ESC_QA1183AA	152	257
ESC_QA1182AA	245	261
ESC_QA1181AA	152	257
ESC_QA1180AA	245	261
ESC_QA1179AA	349	670
ESC_QA1178AA	145	256
ESC_QA1177AA	5517	257
ESC_QA1176AA	152	257
ESC_QA1175AA	152	257
ESC_QA1174AA	1503	257
ESC_QA1173AA	152	257
ESC_QA1172AA	152	257
ESC_QA1171AA	152	257
ESC_QA1170AA	152	257
ESC_QA1169AA	152	257
ESC_QA1168AA	152	257
ESC_QA1167AA	152	257
ESC_QA1166AA	152	257
ESC_QA1165AA	152	257
ESC_QA1164AA	152	257
ESC_QA1163AA	245	261
ESC_QA1162AA	245	261
ESC_QA1161AA	152	257

ESC_QA1160AA	152	257
ESC_QA1159AA	152	257
ESC_QA1158AA	152	257
ESC_QA1157AA	245	261
ESC_QA1156AA	245	261
ESC_QA1155AA	152	257
ESC_QA1154AA	152	257
ESC_QA1153AA	245	261
ESC_QA1152AA	152	257
ESC_QA1151AA	152	257
ESC_QA1150AA	152	257
ESC_QA1149AA	152	257
ESC_QA1148AA	245	261
ESC_QA1147AA	152	257
ESC_QA1146AA	152	257
ESC_QA1145AA	152	257
ESC_QA1144AA	152	257
ESC_QA1143AA	149	102
ESC_QA1142AA	152	257
ESC_QA1141AA	152	257
ESC_QA1140AA	152	257
ESC_QA1139AA	152	257
ESC_QA1138AA	145	256
ESC_QA1137AA	245	261
ESC_QA1136AA	152	257
ESC_QA1135AA	245	261
ESC_QA1134AA	152	257
ESC_QA1133AA	245	261
ESC_QA1132AA	152	257
ESC_QA1131AA	152	257
ESC_QA1130AA	152	257
ESC_QA1129AA	245	261
ESC_QA1128AA	152	257
ESC_QA1127AA	152	257
ESC_QA1126AA	152	257
ESC_QA1125AA	152	257
ESC_QA1124AA	245	261
ESC_QA1123AA	152	257
ESC_QA1122AA	152	257
ESC_QA1121AA	152	257
ESC_QA1120AA	245	261
ESC_QA1119AA	152	257
ESC_QA1118AA	152	257
ESC_QA1117AA	145	256
ESC_QA1116AA	245	261
ESC_QA1115AA	152	257
ESC_QA1114AA	152	257
ESC_QA1113AA	152	257
ESC_QA1112AA	5517	257
ESC_QA1111AA	152	257

ESC_QA1110AA	145	256
ESC_QA1109AA	245	261
ESC_QA1108AA	152	257
ESC_QA1107AA	152	257
ESC_QA1106AA	245	261
ESC_QA1105AA	152	257
ESC_QA1104AA	152	257
ESC_QA1103AA	152	257
ESC_QA1102AA	243	197
ESC_QA1101AA	245	261
ESC_QA1100AA	152	257
ESC_QA1099AA	245	261
ESC_QA1098AA	152	257
ESC_QA1097AA	245	261
ESC_QA1096AA	152	257
ESC_QA1095AA	152	257
ESC_QA1094AA	152	257
ESC_QA1093AA	245	261
ESC_QA1092AA	152	257
ESC_QA1091AA	152	257
ESC_QA1090AA	152	257
ESC_QA1089AA	245	261
ESC_QA1088AA	152	257
ESC_QA1087AA	152	257
ESC_QA1086AA	152	257
ESC_QA1085AA	152	257
ESC_QA1084AA	152	257
ESC_QA1083AA	245	261
ESC_QA1082AA	152	257
ESC_QA1081AA	245	261
ESC_QA1080AA	245	261
ESC_QA1079AA	152	257
ESC_QA1078AA	245	261
ESC_QA1077AA	152	257
ESC_QA1076AA	245	261
ESC_QA1075AA	245	261
ESC_QA1074AA	245	261
ESC_QA1073AA	630	261
ESC_QA1072AA	152	257
ESC_QA1071AA	245	261
ESC_QA1070AA	245	261
ESC_QA1069AA	152	257
ESC_QA1068AA	152	257
ESC_QA1067AA	152	257
ESC_QA1066AA	152	257
ESC_QA1065AA	245	261
ESC_QA1064AA	152	257
ESC_QA1063AA	245	261
ESC_QA1062AA	245	261
ESC_QA1061AA	152	257

ESC_QA1060AA	152	257
ESC_QA1059AA	152	257
ESC_QA1058AA	245	261
ESC_QA1057AA	245	261
ESC_QA1056AA	152	257
ESC_QA1055AA	152	257
ESC_QA1054AA	152	257
ESC_QA1053AA	152	257
ESC_QA1052AA	152	257
ESC_QA1051AA	152	257
ESC_QA1050AA	152	257
ESC_QA1049AA	152	257
ESC_QA1048AA	152	257
ESC_QA1047AA	152	257
ESC_QA1046AA	152	257
ESC_QA1045AA	152	257
ESC_QA1044AA	152	257
ESC_QA1043AA	152	257
ESC_QA1042AA	152	257
ESC_QA1040AA	152	257
ESC_QA1039AA	152	257
ESC_QA1038AA	152	257
ESC_QA1037AA	152	257
ESC_QA1036AA	5517	257
ESC_QA1035AA	152	257
ESC_QA1034AA	152	257
ESC_QA1033AA	152	257
ESC_QA1032AA	152	257
ESC_QA1031AA	152	257
ESC_QA1030AA	152	257
ESC_QA1029AA	152	257
ESC_QA1028AA	152	257
ESC_QA1027AA	152	257
ESC_QA1026AA	152	257
ESC_QA1025AA	152	257
ESC_QA1024AA	152	257
ESC_QA1023AA	152	257
ESC_QA1022AA	152	257
ESC_QA1021AA	152	257
ESC_QA1019AA	152	257
ESC_QA1017AA	152	257
ESC_QA1016AA	152	257
ESC_QA1015AA	152	257
ESC_QA1014AA	243	197
ESC_QA1013AA	152	257
ESC_QA1012AA	152	257
ESC_QA1011AA	152	257
ESC_QA1010AA	152	257
ESC_QA1009AA	152	257
ESC_QA1008AA	1503	257

ESC_QA1007AA	152	257
ESC_QA1005AA	152	257
ESC_QA1004AA	1503	257
ESC_QA1002AA	148	191
ESC_QA1001AA	152	257
ESC_QA1000AA	152	257
ESC_QA0999AA	145	256
ESC_QA0998AA	245	261
ESC_QA0997AA	10676	286
ESC_QA0996AA	10675	926
ESC_QA0995AA	10676	286
ESC_QA0994AA	152	257
ESC_QA0993AA	245	261
ESC_QA0992AA	152	257
ESC_QA0991AA	245	261
ESC_QA0990AA	245	261
ESC_QA0989AA	245	261
ESC_QA0988AA	1024	261
ESC_QA0987AA	717	256
ESC_QA0986AA	152	257
ESC_QA0985AA	152	257
ESC_QA0984AA	152	257
ESC_QA0983AA	10674	261
ESC_QA0982AA	152	257
ESC_QA0981AA	152	257
ESC_QA0980AA	152	257
ESC_QA0979AA	152	257
ESC_QA0978AA	152	257
ESC_QA0977AA	243	197
ESC_QA0976AA	152	257
ESC_QA0975AA	1759	102
ESC_QA0974AA	152	257
ESC_QA0973AA	152	257
ESC_QA0972AA	152	257
ESC_QA0971AA	152	257
ESC_QA0970AA	152	257
ESC_QA0969AA	152	257
ESC_QA0968AA	152	257
ESC_QA0967AA	152	257
ESC_QA0966AA	152	257
ESC_QA0965AA	152	257
ESC_QA0964AA	152	257
ESC_QA0963AA	152	257
ESC_QA0962AA	152	257
ESC_QA0961AA	152	257
ESC_QA0960AA	152	257
ESC_QA0959AA	152	257
ESC_QA0958AA	152	257
ESC_QA0957AA	152	257
ESC_QA0956AA	152	257

ESC_QA0955AA	152	257
ESC_QA0954AA	152	257
ESC_QA0953AA	152	257
ESC_QA0952AA	152	257
ESC_QA0951AA	152	257
ESC_QA0950AA	152	257
ESC_QA0949AA	152	257
ESC_QA0948AA	152	257
ESC_QA0947AA	152	257
ESC_QA0946AA	152	257
ESC_QA0945AA	152	257
ESC_QA0944AA	152	257
ESC_QA0943AA	152	257
ESC_QA0942AA	152	257
ESC_QA0941AA	152	257
ESC_QA0940AA	152	257
ESC_QA0939AA	152	257
ESC_QA0938AA	152	257
ESC_QA0937AA	152	257
ESC_QA0936AA	152	257
ESC_QA0935AA	152	257
ESC_QA0934AA	152	257
ESC_QA0933AA	152	257
ESC_QA0932AA	152	257
ESC_QA0931AA	152	257
ESC_QA0930AA	152	257
ESC_QA0929AA	145	256
ESC_QA0927AA	152	257
ESC_QA0926AA	1273	256
ESC_QA0925AA	152	257
ESC_QA0924AA	152	257
ESC_QA0923AA	245	261
ESC_QA0922AA	245	261
ESC_QA0921AA	152	257
ESC_QA0920AA	152	257
ESC_QA0919AA	245	261
ESC_QA0918AA	5400	261
ESC_QA0917AA	245	261
ESC_QA0916AA	152	257
ESC_QA0915AA	6200	261
ESC_QA0914AA	152	257
ESC_QA0913AA	152	257
ESC_QA0912AA	152	257
ESC_QA0911AA	245	261
ESC_QA0910AA	9148	288
ESC_QA0909AA	152	257
ESC_QA0908AA	245	261
ESC_QA0907AA	245	261
ESC_QA0906AA	152	257
ESC_QA0905AA	245	261

ESC_QA0904AA	245	261
ESC_QA0903AA	245	261
ESC_QA0902AA	152	257
ESC_QA0901AA	152	257
ESC_QA0900AA	152	257
ESC_QA0899AA	152	257
ESC_QA0898AA	152	257
ESC_QA0897AA	152	257
ESC_QA0896AA	152	257
ESC_QA0895AA	6918	260
ESC_QA0894AA	152	257
ESC_QA0893AA	10673	261
ESC_QA0892AA	10672	598
ESC_QA0891AA	152	257
ESC_QA0890AA	152	257
ESC_QA0889AA	152	257
ESC_QA0888AA	245	261
ESC_QA0887AA	245	261
ESC_QA0886AA	245	261
ESC_QA0885AA	152	257
ESC_QA0884AA	245	261
ESC_QA0883AA	152	257
ESC_QA0882AA	152	257
ESC_QA0881AA	245	261
ESC_QA0880AA	245	261
ESC_QA0879AA	245	261
ESC_QA0878AA	245	261
ESC_QA0877AA	245	261
ESC_QA0876AA	152	257
ESC_QA0875AA	152	257
ESC_QA0874AA	245	261
ESC_QA0873AA	152	257
ESC_QA0872AA	145	256
ESC_QA0871AA	152	257
ESC_QA0870AA	152	257
ESC_QA0869AA	152	257
ESC_QA0868AA	152	257
ESC_QA0867AA	152	257
ESC_QA0866AA	145	256
ESC_QA0863AA	152	257
ESC_QA0862AA	152	257
ESC_QA0861AA	152	257
ESC_QA0859AA	152	257
ESC_QA0857AA	152	257
ESC_QA0856AA	152	257
ESC_QA0855AA	152	257
ESC_QA0854AA	152	257
ESC_QA0853AA	148	191
ESC_QA0851AA	152	257
ESC_QA0850AA	152	257

ESC_QA0847AA	7375	197
ESC_QA0846AA	152	257
ESC_QA0845AA	152	257
ESC_QA0844AA	152	257
ESC_QA0843AA	152	257
ESC_QA0842AA	152	257
ESC_QA0841AA	152	257
ESC_QA0840AA	152	257
ESC_QA0837AA	148	191
ESC_QA0835AA	152	257
ESC_QA0833AA	152	257
ESC_QA0830AA	152	257
ESC_QA0829AA	152	257
ESC_QA0828AA	145	256
ESC_QA0825AA	152	257
ESC_QA0824AA	152	257
ESC_QA0823AA	152	257
ESC_QA0822AA	152	257
ESC_QA0821AA	152	257
ESC_QA0820AA	152	257
ESC_QA0819AA	152	257
ESC_QA0818AA	152	257
ESC_QA0817AA	152	257
ESC_QA0816AA	152	257
ESC_QA0815AA	152	257
ESC_QA0814AA	148	191
ESC_QA0813AA	152	257
ESC_QA0812AA	152	257
ESC_QA0811AA	152	257
ESC_QA0810AA	152	257
ESC_QA0809AA	152	257
ESC_QA0808AA	152	257
ESC_QA0807AA	152	257
ESC_QA0806AA	152	257
ESC_QA0805AA	152	257
ESC_QA0804AA	152	257
ESC_QA0803AA	152	257
ESC_QA0802AA	152	257
ESC_QA0801AA	152	257
ESC_QA0800AA	152	257
ESC_QA0799AA	152	257
ESC_QA0798AA	152	257
ESC_QA0797AA	152	257
ESC_QA0796AA	152	257
ESC_QA0795AA	152	257
ESC_QA0794AA	152	257
ESC_QA0793AA	152	257
ESC_QA0792AA	152	257
ESC_QA0791AA	152	257
ESC_QA0790AA	152	257

ESC_QA0789AA	152	257
ESC_QA0788AA	152	257
ESC_QA0787AA	152	257
ESC_QA0786AA	152	257
ESC_QA0785AA	152	257
ESC_QA0784AA	1502	257
ESC_QA0783AA	152	257
ESC_QA0782AA	145	256
ESC_QA0781AA	152	257
ESC_QA0780AA	152	257
ESC_QA0779AA	152	257
ESC_QA0778AA	152	257
ESC_QA0777AA	5517	257
ESC_QA0776AA	152	257
ESC_QA0775AA	152	257
ESC_QA0774AA	152	257
ESC_QA0773AA	152	257
ESC_QA0772AA	152	257
ESC_QA0771AA	152	257
ESC_QA0770AA	152	257
ESC_QA0769AA	152	257
ESC_QA0768AA	152	257
ESC_QA0767AA	152	257
ESC_QA0766AA	152	257
ESC_QA0765AA	152	257
ESC_QA0764AA	152	257
ESC_QA0763AA	152	257
ESC_QA0762AA	152	257
ESC_QA0761AA	152	257
ESC_QA0760AA	152	257
ESC_QA0759AA	152	257
ESC_QA0758AA	152	257
ESC_QA0757AA	152	257
ESC_QA0756AA	152	257
ESC_QA0755AA	152	257
ESC_QA0754AA	152	257
ESC_QA0753AA	152	257
ESC_QA0752AA	152	257
ESC_QA0751AA	152	257
ESC_QA0750AA	152	257
ESC_QA0749AA	152	257
ESC_QA0747AA	152	257
ESC_QA0746AA	152	257
ESC_QA0745AA	152	257
ESC_QA0744AA	152	257
ESC_QA0743AA	5475	256
ESC_QA0742AA	152	257
ESC_QA0741AA	145	256
ESC_QA0740AA	152	257
ESC_QA0739AA	152	257

ESC_QA0738AA	152	257
ESC_QA0737AA	1502	257
ESC_QA0736AA	5517	257
ESC_QA0735AA	152	257
ESC_QA0734AA	5475	256
ESC_QA0733AA	152	257
ESC_QA0732AA	152	257
ESC_QA0731AA	273	102
ESC_QA0730AA	152	257
ESC_QA0729AA	152	257
ESC_QA0728AA	152	257
ESC_QA0727AA	152	257
ESC_QA0726AA	152	257
ESC_QA0725AA	152	257
ESC_QA0724AA	152	257
ESC_QA0723AA	152	257
ESC_QA0722AA	152	257
ESC_QA0721AA	152	257
ESC_QA0720AA	152	257
ESC_QA0719AA	152	257
ESC_QA0718AA	152	257
ESC_QA0717AA	152	257
ESC_QA0716AA	152	257
ESC_QA0715AA	152	257
ESC_QA0714AA	152	257
ESC_QA0713AA	152	257
ESC_QA0712AA	152	257
ESC_QA0711AA	5592	830
ESC_QA0710AA	99	198
ESC_QA0709AA	152	257
ESC_QA0708AA	101	696
ESC_QA0707AA	10	512
ESC_QA0706AA	88	160
ESC_QA0705AA	1434	512
ESC_QA0704AA	1434	512
ESC_QA0703AA	5334	512
ESC_QA0702AA	1623	891
ESC_QA0701AA	1830	512
ESC_QA0700AA	399	198
ESC_QA0699AA	10671	52
ESC_QA0698AA	152	257
ESC_QA0697AA	243	197
ESC_QA0696AA	152	257
ESC_QA0695AA	152	257
ESC_QA0694AA	152	257
ESC_QA0693AA	152	257
ESC_QA0692AA	152	257
ESC_QA0691AA	152	257
ESC_QA0690AA	152	257
ESC_QA0689AA	145	256

ESC_QA0688AA	145	256
ESC_QA0687AA	152	257
ESC_QA0686AA	152	257
ESC_QA0685AA	152	257
ESC_QA0684AA	152	257
ESC_QA0683AA	152	257
ESC_QA0682AA	145	256
ESC_QA0681AA	145	256
ESC_QA0680AA	152	257
ESC_QA0679AA	152	257
ESC_QA0678AA	152	257
ESC_QA0677AA	152	257
ESC_QA0676AA	152	257
ESC_QA0675AA	152	257
ESC_QA0674AA	152	257
ESC_QA0673AA	152	257
ESC_QA0672AA	152	257
ESC_QA0671AA	152	257
ESC_QA0670AA	245	261
ESC_QA0669AA	152	257
ESC_QA0668AA	245	261
ESC_QA0667AA	152	257
ESC_QA0666AA	152	257
ESC_QA0665AA	245	261
ESC_QA0664AA	152	257
ESC_QA0663AA	152	257
ESC_QA0662AA	152	257
ESC_QA0661AA	152	257
ESC_QA0660AA	152	257
ESC_QA0659AA	152	257
ESC_QA0658AA	152	257
ESC_QA0657AA	152	257
ESC_QA0656AA	152	257
ESC_QA0655AA	152	257
ESC_QA0654AA	152	257
ESC_QA0653AA	152	257
ESC_QA0652AA	152	257
ESC_QA0651AA	152	257
ESC_QA0650AA	152	257
ESC_QA0649AA	152	257
ESC_QA0648AA	148	191
ESC_QA0647AA	245	261
ESC_QA0646AA	152	257
ESC_QA0645AA	245	261
ESC_QA0644AA	152	257
ESC_QA0643AA	152	257
ESC_QA0642AA	245	261
ESC_QA0641AA	152	257
ESC_QA0640AA	152	257
ESC_QA0639AA	152	257

ESC_QA0638AA	152	257
ESC_QA0637AA	245	261
ESC_QA0636AA	7384	257
ESC_QA0635AA	152	257
ESC_QA0634AA	7384	257
ESC_QA0633AA	7384	257
ESC_QA0632AA	7384	257
ESC_QA0631AA	7384	257
ESC_QA0630AA	245	261
ESC_QA0629AA	152	257
ESC_QA0628AA	245	261
ESC_QA0627AA	245	261
ESC_QA0626AA	7384	257
ESC_QA0625AA	245	261
ESC_QA0624AA	245	261
ESC_QA0623AA	245	261
ESC_QA0622AA	152	257
ESC_QA0621AA	152	257
ESC_QA0620AA	245	261
ESC_QA0619AA	245	261
ESC_QA0618AA	245	261
ESC_QA0617AA	152	257
ESC_QA0616AA	152	257
ESC_QA0615AA	152	257
ESC_QA0614AA	630	261
ESC_QA0613AA	152	257
ESC_QA0612AA	245	261
ESC_QA0611AA	628	261
ESC_QA0610AA	245	261
ESC_QA0609AA	245	261
ESC_QA0608AA	152	257
ESC_QA0607AA	245	261
ESC_QA0606AA	152	257
ESC_QA0605AA	152	257
ESC_QA0604AA	152	257
ESC_QA0603AA	152	257
ESC_QA0602AA	152	257
ESC_QA0601AA	152	257
ESC_QA0600AA	152	257
ESC_QA0599AA	152	257
ESC_QA0598AA	152	257
ESC_QA0597AA	152	257
ESC_QA0596AA	152	257
ESC_QA0595AA	245	261
ESC_QA0594AA	145	256
ESC_QA0593AA	152	257
ESC_QA0592AA	152	257
ESC_QA0591AA	152	257
ESC_QA0590AA	145	256
ESC_QA0589AA	152	257

ESC_QA0588AA	245	261
ESC_QA0587AA	152	257
ESC_QA0586AA	152	257
ESC_QA0585AA	245	261
ESC_QA0584AA	245	261
ESC_QA0583AA	152	257
ESC_QA0582AA	245	261
ESC_QA0581AA	245	261
ESC_QA0580AA	152	257
ESC_QA0579AA	152	257
ESC_QA0578AA	152	257
ESC_QA0577AA	152	257
ESC_QA0576AA	152	257
ESC_QA0575AA	152	257
ESC_QA0574AA	152	257
ESC_QA0573AA	152	257
ESC_QA0572AA	152	257
ESC_QA0571AA	152	257
ESC_QA0570AA	152	257
ESC_QA0569AA	245	261
ESC_QA0568AA	152	257
ESC_QA0567AA	630	261
ESC_QA0566AA	152	257
ESC_QA0565AA	152	257
ESC_QA0564AA	245	261
ESC_QA0563AA	152	257
ESC_QA0562AA	245	261
ESC_QA0561AA	152	257
ESC_QA0560AA	152	257
ESC_QA0559AA	152	257
ESC_QA0558AA	152	257
ESC_QA0557AA	245	261
ESC_QA0556AA	245	261
ESC_QA0555AA	243	197
ESC_QA0554AA	245	261
ESC_QA0553AA	245	261
ESC_QA0552AA	152	257
ESC_QA0551AA	152	257
ESC_QA0549AA	152	257
ESC_QA0548AA	152	257
ESC_QA0547AA	152	257
ESC_QA0546AA	152	257
ESC_QA0545AA	152	257
ESC_QA0544AA	152	257
ESC_QA0543AA	245	261
ESC_QA0542AA	152	257
ESC_QA0541AA	152	257
ESC_QA0540AA	245	261
ESC_QA0539AA	152	257
ESC_QA0538AA	245	261

ESC_QA0537AA	273	102
ESC_QA0536AA	152	257
ESC_QA0535AA	245	261
ESC_QA0534AA	6200	261
ESC_QA0533AA	245	261
ESC_QA0532AA	152	257
ESC_QA0531AA	152	257
ESC_QA0530AA	245	261
ESC_QA0529AA	245	261
ESC_QA0528AA	152	257
ESC_QA0527AA	245	261
ESC_QA0526AA	152	257
ESC_QA0525AA	152	257
ESC_QA0524AA	245	261
ESC_QA0523AA	152	257
ESC_QA0522AA	152	257
ESC_QA0521AA	245	261
ESC_QA0520AA	245	261
ESC_QA0519AA	152	257
ESC_QA0518AA	152	257
ESC_QA0517AA	245	261
ESC_QA0516AA	245	261
ESC_QA0515AA	630	261
ESC_QA0514AA	152	257
ESC_QA0513AA	245	261
ESC_QA0512AA	152	257
ESC_QA0511AA	245	261
ESC_QA0510AA	145	256
ESC_QA0509AA	152	257
ESC_QA0508AA	152	257
ESC_QA0507AA	152	257
ESC_QA0506AA	152	257
ESC_QA0505AA	152	257
ESC_QA0504AA	245	261
ESC_QA0503AA	152	257
ESC_QA0502AA	152	257
ESC_QA0501AA	245	261
ESC_QA0500AA	152	257
ESC_QA0499AA	152	257
ESC_QA0498AA	245	261
ESC_QA0497AA	152	257
ESC_QA0496AA	245	261
ESC_QA0495AA	152	257
ESC_QA0494AA	152	257
ESC_QA0493AA	145	256
ESC_QA0492AA	152	257
ESC_QA0491AA	152	257
ESC_QA0490AA	245	261
ESC_QA0489AA	7384	257
ESC_QA0488AA	152	257

ESC_QA0487AA	145	256
ESC_QA0486AA	152	257
ESC_QA0485AA	152	257
ESC_QA0484AA	152	257
ESC_QA0483AA	152	257
ESC_QA0482AA	152	257
ESC_QA0481AA	152	257
ESC_QA0480AA	152	257
ESC_QA0479AA	152	257
ESC_QA0478AA	152	257
ESC_QA0477AA	152	257
ESC_QA0476AA	152	257
ESC_QA0475AA	152	257
ESC_QA0474AA	152	257
ESC_QA0473AA	152	257
ESC_QA0472AA	152	257
ESC_QA0471AA	152	257
ESC_QA0470AA	152	257
ESC_QA0469AA	152	257
ESC_QA0468AA	152	257
ESC_QA0467AA	152	257
ESC_QA0466AA	152	257
ESC_QA0465AA	152	257
ESC_QA0464AA	152	257
ESC_QA0463AA	152	257
ESC_QA0462AA	5475	256
ESC_QA0461AA	152	257
ESC_QA0460AA	152	257
ESC_QA0459AA	152	257
ESC_QA0458AA	152	257
ESC_QA0457AA	152	257
ESC_QA0456AA	152	257
ESC_QA0455AA	152	257
ESC_QA0454AA	152	257
ESC_QA0453AA	148	191
ESC_QA0452AA	148	191
ESC_QA0451AA	152	257
ESC_QA0450AA	152	257
ESC_QA0449AA	152	257
ESC_QA0448AA	145	256
ESC_QA0447AA	152	257
ESC_QA0446AA	152	257
ESC_QA0445AA	152	257
ESC_QA0444AA	10670	257
ESC_QA0443AA	152	257
ESC_QA0442AA	152	257
ESC_QA0441AA	152	257
ESC_QA0440AA	147	102
ESC_QA0439AA	152	257
ESC_QA0438AA	152	257

ESC_QA0437AA	152	257
ESC_QA0436AA	152	257
ESC_QA0435AA	152	257
ESC_QA0434AA	152	257
ESC_QA0433AA	152	257
ESC_QA0432AA	152	257
ESC_QA0431AA	152	257
ESC_QA0430AA	152	257
ESC_QA0429AA	152	257
ESC_QA0428AA	152	257
ESC_QA0427AA	152	257
ESC_QA0426AA	152	257
ESC_QA0425AA	1503	257
ESC_QA0424AA	152	257
ESC_QA0423AA	152	257
ESC_QA0422AA	152	257
ESC_QA0421AA	152	257
ESC_QA0420AA	152	257
ESC_QA0419AA	152	257
ESC_QA0418AA	152	257
ESC_QA0417AA	152	257
ESC_QA0416AA	152	257
ESC_QA0415AA	152	257
ESC_QA0414AA	152	257
ESC_QA0413AA	152	257
ESC_QA0412AA	152	257
ESC_QA0411AA	5475	256
ESC_QA0410AA	152	257
ESC_QA0409AA	152	257
ESC_QA0408AA	152	257
ESC_QA0407AA	152	257
ESC_QA0406AA	152	257
ESC_QA0405AA	152	257
ESC_QA0404AA	152	257
ESC_QA0403AA	152	257
ESC_QA0402AA	145	256
ESC_QA0401AA	152	257
ESC_QA0400AA	152	257
ESC_QA0399AA	152	257
ESC_QA0398AA	152	257
ESC_QA0397AA	152	257
ESC_QA0396AA	152	257
ESC_QA0395AA	1503	257
ESC_QA0394AA	152	257
ESC_QA0393AA	152	257
ESC_QA0392AA	152	257
ESC_QA0391AA	152	257
ESC_QA0390AA	152	257
ESC_QA0389AA	152	257
ESC_QA0388AA	152	257

ESC_QA0387AA	152	257
ESC_QA0386AA	152	257
ESC_QA0385AA	152	257
ESC_QA0384AA	152	257
ESC_QA0383AA	152	257
ESC_QA0382AA	245	261
ESC_QA0381AA	152	257
ESC_QA0380AA	152	257
ESC_QA0379AA	152	257
ESC_QA0378AA	152	257
ESC_QA0377AA	152	257
ESC_QA0376AA	152	257
ESC_QA0375AA	152	257
ESC_QA0374AA	152	257
ESC_QA0373AA	245	261
ESC_QA0372AA	152	257
ESC_QA0371AA	245	261
ESC_QA0370AA	245	261
ESC_QA0369AA	273	102
ESC_QA0368AA	245	261
ESC_QA0367AA	245	261
ESC_QA0366AA	245	261
ESC_QA0365AA	270	204
ESC_QA0364AA	245	261
ESC_QA0363AA	152	257
ESC_QA0362AA	245	261
ESC_QA0361AA	152	257
ESC_QA0360AA	243	197
ESC_QA0359AA	152	257
ESC_QA0358AA	152	257
ESC_QA0357AA	5475	256
ESC_QA0356AA	152	257
ESC_QA0355AA	152	257
ESC_QA0354AA	145	256
ESC_QA0353AA	152	257
ESC_QA0352AA	152	257
ESC_QA0351AA	152	257
ESC_QA0350AA	152	257
ESC_QA0349AA	1502	257
ESC_QA0348AA	152	257
ESC_QA0347AA	152	257
ESC_QA0346AA	152	257
ESC_QA0345AA	152	257
ESC_QA0344AA	152	257
ESC_QA0343AA	152	257
ESC_QA0342AA	152	257
ESC_QA0341AA	152	257
ESC_QA0340AA	152	257
ESC_QA0339AA	152	257
ESC_QA0338AA	152	257

ESC_QA0337AA	152	257
ESC_QA0336AA	252	190
ESC_QA0335AA	152	257
ESC_QA0334AA	152	257
ESC_QA0333AA	152	257
ESC_QA0332AA	152	257
ESC_QA0331AA	152	257
ESC_QA0330AA	288	102
ESC_QA0329AA	1273	256
ESC_QA0328AA	148	191
ESC_QA0327AA	152	257
ESC_QA0326AA	152	257
ESC_QA0325AA	152	257
ESC_QA0324AA	152	257
ESC_QA0323AA	152	257
ESC_QA0322AA	152	257
ESC_QA0321AA	5475	256
ESC_QA0320AA	152	257
ESC_QA0319AA	152	257
ESC_QA0318AA	152	257
ESC_QA0317AA	152	257
ESC_QA0316AA	152	257
ESC_QA0315AA	152	257
ESC_QA0314AA	152	257
ESC_QA0313AA	152	257
ESC_QA0312AA	152	257
ESC_QA0311AA	152	257
ESC_QA0310AA	152	257
ESC_QA0309AA	5475	256
ESC_QA0308AA	152	257
ESC_QA0307AA	152	257
ESC_QA0306AA	152	257
ESC_QA0305AA	152	257
ESC_QA0304AA	152	257
ESC_QA0303AA	152	257
ESC_QA0302AA	152	257
ESC_QA0301AA	10669	257
ESC_QA0300AA	152	257
ESC_QA0299AA	152	257
ESC_QA0298AA	152	257
ESC_QA0297AA	152	257
ESC_QA0296AA	152	257
ESC_QA0295AA	152	257
ESC_QA0294AA	152	257
ESC_QA0293AA	152	257
ESC_QA0292AA	152	257
ESC_QA0291AA	152	257
ESC_QA0290AA	152	257
ESC_QA0289AA	152	257
ESC_QA0288AA	152	257

ESC_QA0287AA	152	257
ESC_QA0286AA	152	257
ESC_QA0285AA	152	257
ESC_QA0284AA	152	257
ESC_QA0283AA	152	257
ESC_QA0282AA	152	257
ESC_QA0281AA	152	257
ESC_QA0280AA	152	257
ESC_QA0279AA	152	257
ESC_QA0278AA	152	257
ESC_QA0277AA	152	257
ESC_QA0276AA	152	257
ESC_QA0275AA	152	257
ESC_QA0274AA	152	257
ESC_QA0273AA	152	257
ESC_QA0272AA	152	257
ESC_QA0271AA	152	257
ESC_QA0270AA	152	257
ESC_QA0269AA	152	257
ESC_QA0268AA	5475	256
ESC_QA0267AA	152	257
ESC_QA0266AA	152	257
ESC_QA0265AA	152	257
ESC_QA0264AA	152	257
ESC_QA0263AA	152	257
ESC_QA0262AA	1502	257
ESC_QA0261AA	152	257
ESC_QA0260AA	152	257
ESC_QA0259AA	152	257
ESC_QA0258AA	152	257
ESC_QA0257AA	152	257
ESC_QA0256AA	152	257
ESC_QA0255AA	152	257
ESC_QA0254AA	152	257
ESC_QA0253AA	152	257
ESC_QA0252AA	152	257
ESC_QA0251AA	152	257
ESC_QA0250AA	152	257
ESC_QA0249AA	152	257
ESC_QA0248AA	152	257
ESC_QA0247AA	152	257
ESC_QA0246AA	152	257
ESC_QA0245AA	152	257
ESC_QA0244AA	152	257
ESC_QA0243AA	152	257
ESC_QA0242AA	152	257
ESC_QA0241AA	152	257
ESC_QA0240AA	152	257
ESC_QA0239AA	1273	256
ESC_QA0238AA	152	257

ESC_QA0237AA	152	257
ESC_QA0236AA	145	256
ESC_QA0235AA	152	257
ESC_QA0234AA	152	257
ESC_QA0233AA	1751	102
ESC_QA0232AA	152	257
ESC_QA0231AA	152	257
ESC_QA0230AA	152	257
ESC_QA0229AA	152	257
ESC_QA0228AA	152	257
ESC_QA0227AA	152	257
ESC_QA0226AA	245	261
ESC_QA0225AA	245	261
ESC_QA0224AA	245	261
ESC_QA0223AA	245	261
ESC_QA0222AA	152	257
ESC_QA0221AA	152	257
ESC_QA0220AA	243	197
ESC_QA0219AA	152	257
ESC_QA0218AA	630	261
ESC_QA0217AA	245	261
ESC_QA0216AA	148	191
ESC_QA0215AA	245	261
ESC_QA0213AA	152	257
ESC_QA0212AA	243	197
ESC_QA0211AA	245	261
ESC_QA0210AA	245	261
ESC_QA0209AA	5619	102
ESC_QA0208AA	245	261
ESC_QA0207AA	288	102
ESC_QA0206AA	152	257
ESC_QA0205AA	245	261
ESC_QA0204AA	148	191
ESC_QA0203AA	152	257
ESC_QA0202AA	245	261
ESC_QA0201AA	152	257
ESC_QA0200AA	6200	261
ESC_QA0199AA	152	257
ESC_QA0198AA	245	261
ESC_QA0197AA	152	257
ESC_QA0196AA	245	261
ESC_QA0195AA	245	261
ESC_QA0194AA	245	261
ESC_QA0193AA	152	257
ESC_QA0192AA	152	257
ESC_QA0191AA	245	261
ESC_QA0190AA	152	257
ESC_QA0189AA	152	257
ESC_QA0188AA	152	257
ESC_QA0187AA	152	257

ESC_QA0186AA	164	175
ESC_QA0185AA	10668	566
ESC_QA0184AA	441	198
ESC_QA0183AA	295	160
ESC_QA0182AA	399	198
ESC_QA0181AA	635	141
ESC_QA0180AA	536	141
ESC_QA0179AA	2354	257
ESC_QA0178AA	10	512
ESC_QA0177AA	164	175
ESC_QA0176AA	278	924
ESC_QA0174AA	155	175
ESC_QA0173AA	4663	257
ESC_QA0172AA	10	512
ESC_QA0171AA	162	924
ESC_QA0170AA	302	850
ESC_QA0169AA	453	935
ESC_QA0168AA	131	653
ESC_QA0167AA	949	175
ESC_QA0166AA	23	156
ESC_QA0165AA	949	175
ESC_QA0164AA	2722	234
ESC_QA0163AA	446	257
ESC_QA0162AA	13	257
ESC_QA0161AA	1304	257
ESC_QA0160AA	13	257
ESC_QA0159AA	683	175
ESC_QA0158AA	641	935
ESC_QA0157AA	515	1004
ESC_QA0155AA	38	669
ESC_QA0154AA	4197	856
ESC_QA0153AA	10667	256
ESC_QA0152AA	245	261
ESC_QA0151AA	152	257
ESC_QA0150AA	152	257
ESC_QA0149AA	5400	261
ESC_QA0148AA	10666	261
ESC_QA0147AA	152	257
ESC_QA0146AA	152	257
ESC_QA0145AA	245	261
ESC_QA0144AA	152	257
ESC_QA0143AA	245	261
ESC_QA0142AA	152	257
ESC_QA0141AA	245	261
ESC_QA0140AA	245	261
ESC_QA0139AA	152	257
ESC_QA0138AA	152	257
ESC_QA0137AA	152	257
ESC_QA0136AA	152	257
ESC_QA0135AA	152	257

ESC_QA0134AA	245	261
ESC_QA0133AA	6200	261
ESC_QA0132AA	152	257
ESC_QA0131AA	152	257
ESC_QA0130AA	245	261
ESC_QA0129AA	6200	261
ESC_QA0128AA	152	257
ESC_QA0127AA	280	142
ESC_QA0126AA	245	261
ESC_QA0125AA	1751	102
ESC_QA0124AA	10665	512
ESC_QA0122AA	152	257
ESC_QA0121AA	10664	817
ESC_QA0120AA	11	936
ESC_QA0119AA	11	936
ESC_QA0118AA	301	307
ESC_QA0117AA	301	307
ESC_QA0116AA	301	307
ESC_QA0115AA	29	156
ESC_QA0114AA	29	156
ESC_QA0108AA	245	261
ESC_QA0107AA	1025	261
ESC_QA0106AA	245	261
ESC_QA0105AA	245	261
ESC_QA0104AA	628	261
ESC_QA0103AA	145	256
ESC_QA0102AA	152	257
ESC_QA0101AA	245	261
ESC_QA0100AA	245	261
ESC_QA0099AA	152	257
ESC_QA0098AA	152	257
ESC_QA0097AA	245	261
ESC_QA0096AA	1503	257
ESC_QA0095AA	245	261
ESC_QA0094AA	152	257
ESC_QA0093AA	245	261
ESC_QA0092AA	245	261
ESC_QA0091AA	245	261
ESC_QA0090AA	152	257
ESC_QA0089AA	245	261
ESC_QA0088AA	245	261
ESC_QA0087AA	245	261
ESC_QA0086AA	5517	257
ESC_QA0085AA	1025	261
ESC_QA0084AA	152	257
ESC_QA0083AA	152	257
ESC_QA0082AA	245	261
ESC_QA0081AA	245	261
ESC_QA0080AA	152	257
ESC_QA0079AA	152	257

ESC_QA0078AA	152	257
ESC_QA0077AA	152	257
ESC_QA0076AA	152	257
ESC_QA0075AA	152	257
ESC_QA0074AA	152	257
ESC_QA0073AA	152	257
ESC_QA0072AA	152	257
ESC_QA0071AA	152	257
ESC_QA0070AA	152	257
ESC_QA0069AA	152	257
ESC_QA0068AA	152	257
ESC_QA0067AA	152	257
ESC_QA0066AA	152	257
ESC_QA0065AA	152	257
ESC_QA0064AA	152	257
ESC_QA0063AA	152	257
ESC_QA0062AA	152	257
ESC_QA0061AA	152	257
ESC_QA0060AA	152	257
ESC_QA0059AA	152	257
ESC_QA0058AA	152	257
ESC_QA0057AA	152	257
ESC_QA0056AA	152	257
ESC_QA0055AA	152	257
ESC_QA0054AA	152	257
ESC_QA0053AA	152	257
ESC_QA0052AA	152	257
ESC_QA0051AA	152	257
ESC_QA0050AA	152	257
ESC_QA0049AA	152	257
ESC_QA0048AA	152	257
ESC_QA0047AA	152	257
ESC_QA0046AA	152	257
ESC_QA0045AA	152	257
ESC_QA0044AA	7375	197
ESC_QA0043AA	152	257
ESC_QA0042AA	152	257
ESC_QA0041AA	152	257
ESC_QA0040AA	152	257
ESC_QA0039AA	245	261
ESC_QA0038AA	152	257
ESC_QA0037AA	245	261
ESC_QA0036AA	245	261
ESC_QA0035AA	245	261
ESC_QA0034AA	245	261
ESC_QA0033AA	245	261
ESC_QA0032AA	245	261
ESC_QA0031AA	152	257
ESC_QA0030AA	152	257
ESC_QA0029AA	152	257

ESC_QA0028AA	152	257
ESC_QA0027AA	152	257
ESC_QA0026AA	152	257
ESC_QA0024AA	152	257
ESC_QA0023AA	152	257
ESC_QA0022AA	152	257
ESC_QA0021AA	152	257
ESC_QA0020AA	152	257
ESC_QA0019AA	152	257
ESC_QA0018AA	152	257
ESC_QA0017AA	152	257
ESC_QA0016AA	152	257
ESC_QA0015AA	152	257
ESC_QA0014AA	152	257
ESC_QA0013AA	152	257
ESC_QA0012AA	152	257
ESC_QA0011AA	152	257
ESC_QA0010AA	273	102
ESC_QA0009AA	152	257
ESC_QA0008AA	148	191
ESC_QA0007AA	152	257
ESC_QA0006AA	152	257
ESC_QA0005AA	152	257
ESC_QA0004AA	152	257
ESC_QA0003AA	152	257
ESC_QA0002AA	152	257
ESC_QA0001AA	152	257
ESC_QA0000AA	152	257
ESC_PA9999AA	152	257
ESC_PA9998AA	152	257
ESC_PA9997AA	152	257
ESC_PA9996AA	152	257
ESC_PA9995AA	152	257
ESC_PA9994AA	152	257
ESC_PA9993AA	152	257
ESC_PA9992AA	152	257
ESC_PA9991AA	152	257
ESC_PA9990AA	152	257
ESC_PA9989AA	152	257
ESC_PA9988AA	152	257
ESC_PA9987AA	152	257
ESC_PA9986AA	152	257
ESC_PA9985AA	152	257
ESC_PA9984AA	152	257
ESC_PA9983AA	152	257
ESC_PA9982AA	152	257
ESC_PA9981AA	245	261
ESC_PA9980AA	1273	256
ESC_PA9979AA	152	257
ESC_PA9978AA	152	257

ESC_PA9977AA	152	257
ESC_PA9976AA	152	257
ESC_PA9975AA	152	257
ESC_PA9974AA	152	257
ESC_PA9973AA	245	261
ESC_PA9972AA	245	261
ESC_PA9971AA	245	261
ESC_PA9970AA	152	257
ESC_PA9969AA	245	261
ESC_PA9968AA	152	257
ESC_PA9967AA	245	261
ESC_PA9966AA	245	261
ESC_PA9965AA	245	261
ESC_PA9964AA	10663	261
ESC_PA9963AA	10663	261
ESC_PA9962AA	245	261
ESC_PA9961AA	245	261
ESC_PA9960AA	245	261
ESC_PA9959AA	245	261
ESC_PA9958AA	245	261
ESC_PA9957AA	152	257
ESC_PA9956AA	152	257
ESC_PA9955AA	152	257
ESC_PA9954AA	152	257
ESC_PA9953AA	245	261
ESC_PA9952AA	152	257
ESC_PA9951AA	152	257
ESC_PA9950AA	152	257
ESC_PA9949AA	152	257
ESC_PA9948AA	152	257
ESC_PA9947AA	273	102
ESC_PA9946AA	5475	256
ESC_PA9945AA	273	102
ESC_PA9944AA	245	261
ESC_PA9943AA	245	261
ESC_PA9942AA	245	261
ESC_PA9941AA	152	257
ESC_PA9940AA	152	257
ESC_PA9939AA	245	261
ESC_PA9938AA	245	261
ESC_PA9937AA	5658	257
ESC_PA9936AA	1025	261
ESC_PA9935AA	152	257
ESC_PA9934AA	152	257
ESC_PA9933AA	152	257
ESC_PA9932AA	152	257
ESC_PA9931AA	245	261
ESC_PA9930AA	245	261
ESC_PA9929AA	245	261
ESC_PA9928AA	152	257

ESC_PA9927AA	245	261
ESC_PA9926AA	152	257
ESC_PA9925AA	245	261
ESC_PA9924AA	152	257
ESC_PA9923AA	152	257
ESC_PA9922AA	152	257
ESC_PA9921AA	152	257
ESC_PA9920AA	152	257
ESC_PA9919AA	152	257
ESC_PA9918AA	152	257
ESC_PA9917AA	245	261
ESC_PA9916AA	145	256
ESC_PA9915AA	152	257
ESC_PA9914AA	152	257
ESC_PA9913AA	152	257
ESC_PA9912AA	152	257
ESC_PA9911AA	152	257
ESC_PA9910AA	152	257
ESC_PA9909AA	152	257
ESC_PA9908AA	152	257
ESC_PA9907AA	245	261
ESC_PA9906AA	152	257
ESC_PA9905AA	152	257
ESC_PA9904AA	152	257
ESC_PA9903AA	152	257
ESC_PA9902AA	152	257
ESC_PA9901AA	245	261
ESC_PA9900AA	152	257
ESC_PA9897AA	152	257
ESC_PA9896AA	152	257
ESC_PA9895AA	152	257
ESC_PA9894AA	152	257
ESC_PA9893AA	245	261
ESC_PA9892AA	245	261
ESC_PA9891AA	152	257
ESC_PA9890AA	152	257
ESC_PA9889AA	152	257
ESC_PA9888AA	152	257
ESC_PA9887AA	152	257
ESC_PA9886AA	152	257
ESC_PA9885AA	152	257
ESC_PA9884AA	245	261
ESC_PA9883AA	245	261
ESC_PA9882AA	152	257
ESC_PA9881AA	152	257
ESC_PA9880AA	245	261
ESC_PA9879AA	152	257
ESC_PA9878AA	245	261
ESC_PA9877AA	245	261
ESC_PA9876AA	245	261

ESC_PA9875AA	245	261
ESC_PA9874AA	152	257
ESC_PA9873AA	152	257
ESC_PA9872AA	152	257
ESC_PA9871AA	152	257
ESC_PA9870AA	6988	261
ESC_PA9869AA	152	257
ESC_PA9868AA	245	261
ESC_PA9867AA	152	257
ESC_PA9866AA	152	257
ESC_PA9865AA	245	261
ESC_PA9864AA	152	257
ESC_PA9863AA	152	257
ESC_PA9862AA	152	257
ESC_PA9861AA	152	257
ESC_PA9860AA	152	257
ESC_PA9859AA	152	257
ESC_PA9858AA	152	257
ESC_PA9857AA	152	257
ESC_PA9856AA	152	257
ESC_PA9855AA	152	257
ESC_PA9854AA	152	257
ESC_PA9853AA	245	261
ESC_PA9852AA	152	257
ESC_PA9851AA	245	261
ESC_PA9850AA	152	257
ESC_PA9849AA	152	257
ESC_PA9848AA	245	261
ESC_PA9847AA	152	257
ESC_PA9846AA	152	257
ESC_PA9845AA	152	257
ESC_PA9844AA	152	257
ESC_PA9843AA	152	257
ESC_PA9842AA	145	256
ESC_PA9841AA	152	257
ESC_PA9840AA	245	261
ESC_PA9839AA	245	261
ESC_PA9838AA	245	261
ESC_PA9837AA	245	261
ESC_PA9836AA	245	261
ESC_PA9835AA	152	257
ESC_PA9834AA	152	257
ESC_PA9833AA	245	261
ESC_PA9832AA	245	261
ESC_PA9831AA	245	261
ESC_PA9830AA	152	257
ESC_PA9829AA	152	257
ESC_PA9828AA	152	257
ESC_PA9827AA	245	261
ESC_PA9826AA	245	261

ESC_PA9825AA	245	261
ESC_PA9824AA	152	257
ESC_PA9823AA	245	261
ESC_PA9822AA	152	257
ESC_PA9821AA	152	257
ESC_PA9820AA	245	261
ESC_PA9819AA	152	257
ESC_PA9817AA	5517	257
ESC_PA9816AA	152	257
ESC_PA9815AA	245	261
ESC_PA9814AA	152	257
ESC_PA9813AA	152	257
ESC_PA9812AA	245	261
ESC_PA9811AA	152	257
ESC_PA9810AA	152	257
ESC_PA9809AA	152	257
ESC_PA9808AA	152	257
ESC_PA9807AA	152	257
ESC_PA9806AA	152	257
ESC_PA9805AA	152	257
ESC_PA9804AA	152	257
ESC_PA9803AA	152	257
ESC_PA9802AA	245	261
ESC_PA9801AA	152	257
ESC_PA9800AA	152	257
ESC_PA9799AA	152	257
ESC_PA9798AA	152	257
ESC_PA9797AA	152	257
ESC_PA9796AA	245	261
ESC_PA9795AA	245	261
ESC_PA9789AA	245	261
ESC_PA9788AA	152	257
ESC_PA9787AA	152	257
ESC_PA9786AA	152	257
ESC_PA9785AA	245	261
ESC_PA9784AA	152	257
ESC_PA9783AA	152	257
ESC_PA9782AA	152	257
ESC_PA9781AA	152	257
ESC_PA9780AA	152	257
ESC_PA9779AA	152	257
ESC_PA9778AA	152	257
ESC_PA9777AA	245	261
ESC_PA9776AA	152	257
ESC_PA9775AA	152	257
ESC_PA9774AA	152	257
ESC_PA9773AA	10662	261
ESC_PA9772AA	152	257
ESC_PA9771AA	152	257
ESC_PA9770AA	10661	924

ESC_PA9767AA	10660	108
ESC_PA9766AA	10660	108
ESC_PA9765AA	10660	108
ESC_PA9764AA	10660	108
ESC_PA9763AA	10659	234
ESC_PA9762AA	10658	257
ESC_PA9761AA	8630	257
ESC_PA9760AA	10657	234
ESC_PA9759AA	8630	257
ESC_PA9758AA	10656	808
ESC_PA9757AA	10655	257
ESC_PA9756AA	245	261
ESC_PA9755AA	152	257
ESC_PA9754AA	152	257
ESC_PA9753AA	245	261
ESC_PA9752AA	152	257
ESC_PA9751AA	245	261
ESC_PA9750AA	152	257
ESC_PA9749AA	245	261
ESC_PA9748AA	152	257
ESC_PA9747AA	152	257
ESC_PA9746AA	245	261
ESC_PA9745AA	245	261
ESC_PA9744AA	245	261
ESC_PA9743AA	1024	261
ESC_PA9742AA	152	257
ESC_PA9741AA	245	261
ESC_PA9740AA	152	257
ESC_PA9739AA	10654	246
ESC_PA9738AA	152	257
ESC_PA9737AA	245	261
ESC_PA9736AA	245	261
ESC_PA9735AA	152	257
ESC_PA9734AA	145	256
ESC_PA9733AA	245	261
ESC_PA9732AA	245	261
ESC_PA9731AA	152	257
ESC_PA9730AA	243	197
ESC_PA9729AA	152	257
ESC_PA9728AA	245	261
ESC_PA9727AA	245	261
ESC_PA9726AA	245	261
ESC_PA9725AA	148	191
ESC_PA9724AA	152	257
ESC_PA9723AA	245	261
ESC_PA9722AA	245	261
ESC_PA9721AA	152	257
ESC_PA9720AA	152	257
ESC_PA9719AA	152	257
ESC_PA9718AA	152	257

ESC_PA9717AA	152	257
ESC_PA9716AA	152	257
ESC_PA9715AA	152	257
ESC_PA9714AA	245	261
ESC_PA9713AA	145	256
ESC_PA9712AA	245	261
ESC_PA9711AA	152	257
ESC_PA9710AA	243	197
ESC_PA9709AA	152	257
ESC_PA9708AA	152	257
ESC_PA9707AA	1024	261
ESC_PA9705AA	8878	261
ESC_PA9704AA	152	257
ESC_PA9703AA	145	256
ESC_PA9702AA	7375	197
ESC_PA9701AA	152	257
ESC_PA9700AA	152	257
ESC_PA9699AA	152	257
ESC_PA9698AA	245	261
ESC_PA9697AA	245	261
ESC_PA9696AA	152	257
ESC_PA9695AA	245	261
ESC_PA9694AA	152	257
ESC_PA9693AA	245	261
ESC_PA9692AA	152	257
ESC_PA9691AA	7384	257
ESC_PA9690AA	245	261
ESC_PA9689AA	152	257
ESC_PA9688AA	152	257
ESC_PA9687AA	152	257
ESC_PA9686AA	152	257
ESC_PA9685AA	152	257
ESC_PA9684AA	245	261
ESC_PA9683AA	152	257
ESC_PA9682AA	152	257
ESC_PA9680AA	152	257
ESC_PA9679AA	245	261
ESC_PA9678AA	152	257
ESC_PA9677AA	152	257
ESC_PA9676AA	152	257
ESC_PA9675AA	152	257
ESC_PA9674AA	152	257
ESC_PA9673AA	152	257
ESC_PA9672AA	152	257
ESC_PA9671AA	152	257
ESC_PA9670AA	152	257
ESC_PA9669AA	152	257
ESC_PA9668AA	152	257
ESC_PA9667AA	152	257
ESC_PA9666AA	245	261

ESC_PA9665AA	245	261
ESC_PA9664AA	152	257
ESC_PA9663AA	361	512
ESC_PA9662AA	245	261
ESC_PA9661AA	152	257
ESC_PA9660AA	152	257
ESC_PA9659AA	152	257
ESC_PA9658AA	152	257
ESC_PA9657AA	145	256
ESC_PA9656AA	245	261
ESC_PA9655AA	152	257
ESC_PA9654AA	1503	257
ESC_PA9653AA	152	257
ESC_PA9652AA	152	257
ESC_PA9651AA	145	256
ESC_PA9650AA	152	257
ESC_PA9649AA	245	261
ESC_PA9648AA	245	261
ESC_PA9647AA	245	261
ESC_PA9646AA	152	257
ESC_PA9645AA	152	257
ESC_PA9644AA	152	257
ESC_PA9643AA	152	257
ESC_PA9642AA	152	257
ESC_PA9641AA	245	261
ESC_PA9640AA	152	257
ESC_PA9639AA	245	261
ESC_PA9638AA	245	261
ESC_PA9637AA	245	261
ESC_PA9636AA	1504	254
ESC_PA9635AA	245	261
ESC_PA9634AA	245	261
ESC_PA9633AA	245	261
ESC_PA9632AA	152	257
ESC_PA9631AA	245	261
ESC_PA9630AA	245	261
ESC_PA9629AA	245	261
ESC_PA9628AA	245	261
ESC_PA9627AA	245	261
ESC_PA9626AA	152	257
ESC_PA9625AA	152	257
ESC_PA9624AA	152	257
ESC_PA9623AA	243	197
ESC_PA9622AA	245	261
ESC_PA9621AA	152	257
ESC_PA9620AA	152	257
ESC_PA9619AA	245	261
ESC_PA9618AA	152	257
ESC_PA9617AA	152	257
ESC_PA9616AA	152	257

ESC_PA9615AA	152	257
ESC_PA9614AA	245	261
ESC_PA9613AA	152	257
ESC_PA9612AA	245	261
ESC_PA9611AA	152	257
ESC_PA9610AA	152	257
ESC_PA9609AA	7384	257
ESC_PA9608AA	7384	257
ESC_PA9607AA	152	257
ESC_PA9606AA	7384	257
ESC_PA9605AA	245	261
ESC_PA9604AA	7384	257
ESC_PA9603AA	152	257
ESC_PA9602AA	152	257
ESC_PA9601AA	152	257
ESC_PA9600AA	152	257
ESC_PA9599AA	245	261
ESC_PA9598AA	245	261
ESC_PA9597AA	152	257
ESC_PA9596AA	152	257
ESC_PA9595AA	145	256
ESC_PA9594AA	152	257
ESC_PA9593AA	245	261
ESC_PA9592AA	152	257
ESC_PA9591AA	152	257
ESC_PA9590AA	145	256
ESC_PA9589AA	152	257
ESC_PA9588AA	245	261
ESC_PA9587AA	152	257
ESC_PA9586AA	152	257
ESC_PA9585AA	152	257
ESC_PA9584AA	245	261
ESC_PA9583AA	152	257
ESC_PA9582AA	152	257
ESC_PA9581AA	152	257
ESC_PA9580AA	152	257
ESC_PA9579AA	245	261
ESC_PA9578AA	145	256
ESC_PA9577AA	152	257
ESC_PA9576AA	245	261
ESC_PA9575AA	152	257
ESC_PA9574AA	152	257
ESC_PA9573AA	152	257
ESC_PA9572AA	152	257
ESC_PA9571AA	145	256
ESC_PA9570AA	152	257
ESC_PA9569AA	152	257
ESC_PA9568AA	152	257
ESC_PA9567AA	245	261
ESC_PA9566AA	152	257

ESC_PA9565AA	152	257
ESC_PA9564AA	152	257
ESC_PA9563AA	152	257
ESC_PA9562AA	152	257
ESC_PA9561AA	152	257
ESC_PA9560AA	152	257
ESC_PA9559AA	152	257
ESC_PA9558AA	148	191
ESC_PA9557AA	145	256
ESC_PA9556AA	152	257
ESC_PA9555AA	152	257
ESC_PA9554AA	152	257
ESC_PA9553AA	245	261
ESC_PA9552AA	245	261
ESC_PA9551AA	245	261
ESC_PA9550AA	152	257
ESC_PA9549AA	245	261
ESC_PA9548AA	152	257
ESC_PA9547AA	152	257
ESC_PA9546AA	145	256
ESC_PA9545AA	152	257
ESC_PA9544AA	152	257
ESC_PA9543AA	145	256
ESC_PA9542AA	245	261
ESC_PA9541AA	152	257
ESC_PA9540AA	245	261
ESC_PA9539AA	152	257
ESC_PA9538AA	245	261
ESC_PA9537AA	152	257
ESC_PA9536AA	152	257
ESC_PA9535AA	152	257
ESC_PA9534AA	1502	257
ESC_PA9533AA	152	257
ESC_PA9532AA	245	261
ESC_PA9531AA	152	257
ESC_PA9530AA	152	257
ESC_PA9529AA	145	256
ESC_PA9528AA	152	257
ESC_PA9527AA	245	261
ESC_PA9526AA	245	261
ESC_PA9525AA	245	261
ESC_PA9524AA	152	257
ESC_PA9523AA	152	257
ESC_PA9522AA	245	261
ESC_PA9521AA	152	257
ESC_PA9520AA	152	257
ESC_PA9519AA	152	257
ESC_PA9518AA	245	261
ESC_PA9517AA	245	261
ESC_PA9516AA	245	261

ESC_PA9515AA	152	257
ESC_PA9514AA	152	257
ESC_PA9513AA	152	257
ESC_PA9512AA	245	261
ESC_PA9511AA	152	257
ESC_PA9510AA	152	257
ESC_PA9509AA	152	257
ESC_PA9508AA	152	257
ESC_PA9507AA	245	261
ESC_PA9506AA	152	257
ESC_PA9505AA	152	257
ESC_PA9504AA	628	261
ESC_PA9503AA	152	257
ESC_PA9502AA	152	257
ESC_PA9501AA	152	257
ESC_PA9499AA	245	261
ESC_PA9498AA	152	257
ESC_PA9497AA	245	261
ESC_PA9496AA	152	257
ESC_PA9494AA	7384	257
ESC_PA9493AA	152	257
ESC_PA9492AA	7384	257
ESC_PA9491AA	7384	257
ESC_PA9490AA	152	257
ESC_PA9489AA	245	261
ESC_PA9488AA	7384	257
ESC_PA9487AA	6370	256
ESC_PA9486AA	152	257
ESC_PA9485AA	145	256
ESC_PA9484AA	7384	257
ESC_PA9483AA	7384	257
ESC_PA9482AA	7384	257
ESC_PA9481AA	145	256
ESC_PA9480AA	7384	257
ESC_PA9479AA	7384	257
ESC_PA9478AA	7384	257
ESC_PA9477AA	152	257
ESC_PA9476AA	152	257
ESC_PA9475AA	245	261
ESC_PA9474AA	7384	257
ESC_PA9473AA	7384	257
ESC_PA9472AA	7384	257
ESC_PA9471AA	152	257
ESC_PA9470AA	7384	257
ESC_PA9469AA	7384	257
ESC_PA9468AA	7384	257
ESC_PA9467AA	152	257
ESC_PA9466AA	245	261
ESC_PA9465AA	245	261
ESC_PA9464AA	245	261

ESC_PA9463AA	245	261
ESC_PA9462AA	7384	257
ESC_PA9461AA	152	257
ESC_PA9460AA	145	256
ESC_PA9459AA	152	257
ESC_PA9458AA	152	257
ESC_PA9457AA	245	261
ESC_PA9456AA	7384	257
ESC_PA9455AA	152	257
ESC_PA9454AA	152	257
ESC_PA9453AA	413	121
ESC_PA9452AA	152	257
ESC_PA9451AA	152	257
ESC_PA9450AA	152	257
ESC_PA9449AA	152	257
ESC_PA9448AA	245	261
ESC_PA9447AA	152	257
ESC_PA9446AA	152	257
ESC_PA9445AA	152	257
ESC_PA9444AA	152	257
ESC_PA9443AA	245	261
ESC_PA9442AA	152	257
ESC_PA9441AA	245	261
ESC_PA9440AA	245	261
ESC_PA9439AA	152	257
ESC_PA9438AA	245	261
ESC_PA9437AA	245	261
ESC_PA9436AA	245	261
ESC_PA9435AA	152	257
ESC_PA9434AA	152	257
ESC_PA9430AA	152	257
ESC_PA9429AA	152	257
ESC_PA9428AA	152	257
ESC_PA9427AA	152	257
ESC_PA9424AA	152	257
ESC_PA9421AA	152	257
ESC_PA9420AA	152	257
ESC_PA9419AA	152	257
ESC_PA9418AA	152	257
ESC_PA9417AA	152	257
ESC_PA9416AA	152	257
ESC_PA9415AA	152	257
ESC_PA9414AA	152	257
ESC_PA9413AA	152	257
ESC_PA9412AA	152	257
ESC_PA9411AA	152	257
ESC_PA9410AA	152	257
ESC_PA9409AA	152	257
ESC_PA9408AA	152	257
ESC_PA9407AA	152	257

ESC_PA9406AA	152	257
ESC_PA9405AA	152	257
ESC_PA9404AA	152	257
ESC_PA9403AA	152	257
ESC_PA9402AA	152	257
ESC_PA9400AA	152	257
ESC_PA9399AA	152	257
ESC_PA9398AA	152	257
ESC_PA9397AA	152	257
ESC_PA9396AA	152	257
ESC_PA9394AA	152	257
ESC_PA9393AA	152	257
ESC_PA9392AA	152	257
ESC_PA9390AA	152	257
ESC_PA9389AA	152	257
ESC_PA9388AA	245	261
ESC_PA9387AA	152	257
ESC_PA9386AA	152	257
ESC_PA9385AA	5240	261
ESC_PA9384AA	152	257
ESC_PA9383AA	152	257
ESC_PA9382AA	152	257
ESC_PA9381AA	147	102
ESC_PA9380AA	245	261
ESC_PA9379AA	245	261
ESC_PA9378AA	245	261
ESC_PA9377AA	145	256
ESC_PA9376AA	245	261
ESC_PA9375AA	152	257
ESC_PA9374AA	152	257
ESC_PA9373AA	152	257
ESC_PA9372AA	152	257
ESC_PA9371AA	152	257
ESC_PA9370AA	152	257
ESC_PA9369AA	152	257
ESC_PA9368AA	152	257
ESC_PA9367AA	152	257
ESC_PA9366AA	145	256
ESC_PA9365AA	152	257
ESC_PA9364AA	270	204
ESC_PA9363AA	152	257
ESC_PA9362AA	152	257
ESC_PA9361AA	245	261
ESC_PA9360AA	152	257
ESC_PA9359AA	152	257
ESC_PA9358AA	245	261
ESC_PA9357AA	152	257
ESC_PA9356AA	152	257
ESC_PA9355AA	245	261
ESC_PA9354AA	152	257

ESC_PA9353AA	152	257
ESC_PA9352AA	245	261
ESC_PA9351AA	152	257
ESC_PA9350AA	152	257
ESC_PA9349AA	152	257
ESC_PA9348AA	1502	257
ESC_PA9347AA	145	256
ESC_PA9346AA	152	257
ESC_PA9345AA	152	257
ESC_PA9344AA	152	257
ESC_PA9343AA	152	257
ESC_PA9342AA	152	257
ESC_PA9341AA	152	257
ESC_PA9340AA	152	257
ESC_PA9339AA	152	257
ESC_PA9338AA	152	257
ESC_PA9337AA	245	261
ESC_PA9336AA	145	256
ESC_PA9335AA	152	257
ESC_PA9334AA	152	257
ESC_PA9333AA	145	256
ESC_PA9332AA	152	257
ESC_PA9331AA	8734	102
ESC_PA9330AA	4267	257
ESC_PA9329AA	245	261
ESC_PA9328AA	152	257
ESC_PA9327AA	245	261
ESC_PA9326AA	152	257
ESC_PA9325AA	152	257
ESC_PA9324AA	152	257
ESC_PA9323AA	152	257
ESC_PA9322AA	152	257
ESC_PA9321AA	152	257
ESC_PA9320AA	152	257
ESC_PA9319AA	152	257
ESC_PA9318AA	152	257
ESC_PA9317AA	152	257
ESC_PA9316AA	245	261
ESC_PA9315AA	7384	257
ESC_PA9314AA	145	256
ESC_PA9313AA	152	257
ESC_PA9312AA	152	257
ESC_PA9311AA	152	257
ESC_PA9310AA	152	257
ESC_PA9309AA	245	261
ESC_PA9308AA	145	256
ESC_PA9307AA	152	257
ESC_PA9306AA	245	261
ESC_PA9305AA	152	257
ESC_PA9304AA	152	257

ESC_PA9303AA	152	257
ESC_PA9302AA	152	257
ESC_PA9301AA	245	261
ESC_PA9300AA	245	261
ESC_PA9299AA	245	261
ESC_PA9298AA	245	261
ESC_PA9297AA	6200	261
ESC_PA9296AA	152	257
ESC_PA9295AA	245	261
ESC_PA9294AA	152	257
ESC_PA9293AA	245	261
ESC_PA9292AA	145	256
ESC_PA9291AA	148	191
ESC_PA9290AA	145	256
ESC_PA9289AA	226	512
ESC_PA9287AA	6200	261
ESC_PA9286AA	152	257
ESC_PA9285AA	152	257
ESC_PA9284AA	152	257
ESC_PA9283AA	152	257
ESC_PA9282AA	152	257
ESC_PA9281AA	245	261
ESC_PA9280AA	245	261
ESC_PA9279AA	245	261
ESC_PA9278AA	245	261
ESC_PA9277AA	245	261
ESC_PA9276AA	6200	261
ESC_PA9275AA	245	261
ESC_PA9274AA	245	261
ESC_PA9273AA	245	261
ESC_PA9272AA	152	257
ESC_PA9271AA	152	257
ESC_PA9270AA	152	257
ESC_PA9269AA	152	257
ESC_PA9268AA	245	261
ESC_PA9267AA	152	257
ESC_PA9266AA	152	257
ESC_PA9265AA	145	256
ESC_PA9264AA	152	257
ESC_PA9263AA	245	261
ESC_PA9262AA	245	261
ESC_PA9261AA	152	257
ESC_PA9260AA	152	257
ESC_PA9259AA	152	257
ESC_PA9258AA	152	257
ESC_PA9257AA	1273	256
ESC_PA9256AA	152	257
ESC_PA9255AA	152	257
ESC_PA9254AA	152	257
ESC_PA9253AA	145	256

ESC_PA9252AA	152	257
ESC_PA9251AA	152	257
ESC_PA9250AA	245	261
ESC_PA9249AA	245	261
ESC_PA9248AA	245	261
ESC_PA9247AA	245	261
ESC_PA9246AA	152	257
ESC_PA9245AA	152	257
ESC_PA9244AA	245	261
ESC_PA9243AA	152	257
ESC_PA9242AA	152	257
ESC_PA9241AA	152	257
ESC_PA9240AA	152	257
ESC_PA9239AA	152	257
ESC_PA9238AA	152	257
ESC_PA9237AA	152	257
ESC_PA9236AA	152	257
ESC_PA9235AA	152	257
ESC_PA9234AA	152	257
ESC_PA9233AA	152	257
ESC_PA9232AA	245	261
ESC_PA9231AA	152	257
ESC_PA9230AA	1503	257
ESC_PA9229AA	152	257
ESC_PA9228AA	1503	257
ESC_PA9227AA	245	261
ESC_PA9226AA	245	261
ESC_PA9225AA	245	261
ESC_PA9224AA	152	257
ESC_PA9223AA	245	261
ESC_PA9222AA	145	256
ESC_PA9221AA	245	261
ESC_PA9220AA	245	261
ESC_PA9219AA	145	256
ESC_PA9218AA	245	261
ESC_PA9217AA	145	256
ESC_PA9216AA	152	257
ESC_PA9215AA	245	261
ESC_PA9214AA	245	261
ESC_PA9213AA	152	257
ESC_PA9212AA	152	257
ESC_PA9211AA	152	257
ESC_PA9210AA	152	257
ESC_PA9209AA	152	257
ESC_PA9208AA	152	257
ESC_PA9207AA	148	191
ESC_PA9205AA	152	257
ESC_PA9204AA	152	257
ESC_PA9203AA	152	257
ESC_PA9202AA	152	257

ESC_PA9201AA	145	256
ESC_PA9200AA	152	257
ESC_PA9199AA	152	257
ESC_PA9198AA	152	257
ESC_PA9197AA	245	261
ESC_PA9196AA	152	257
ESC_PA9195AA	152	257
ESC_PA9194AA	152	257
ESC_PA9193AA	152	257
ESC_PA9192AA	152	257
ESC_PA9191AA	152	257
ESC_PA9190AA	152	257
ESC_PA9189AA	245	261
ESC_PA9188AA	152	257
ESC_PA9187AA	245	261
ESC_PA9186AA	245	261
ESC_PA9185AA	152	257
ESC_PA9184AA	245	261
ESC_PA9183AA	152	257
ESC_PA9182AA	245	261
ESC_PA9181AA	152	257
ESC_PA9179AA	148	191
ESC_PA9178AA	1025	261
ESC_PA9177AA	243	197
ESC_PA9176AA	152	257
ESC_PA9175AA	245	261
ESC_PA9174AA	152	257
ESC_PA9173AA	152	257
ESC_PA9172AA	245	261
ESC_PA9171AA	152	257
ESC_PA9170AA	245	261
ESC_PA9169AA	152	257
ESC_PA9168AA	152	257
ESC_PA9167AA	245	261
ESC_PA9166AA	245	261
ESC_PA9165AA	152	257
ESC_PA9164AA	245	261
ESC_PA9163AA	152	257
ESC_PA9162AA	152	257
ESC_PA9160AA	245	261
ESC_PA9159AA	152	257
ESC_PA9158AA	152	257
ESC_PA9157AA	245	261
ESC_PA9156AA	245	261
ESC_PA9155AA	245	261
ESC_PA9154AA	152	257
ESC_PA9153AA	152	257
ESC_PA9152AA	152	257
ESC_PA9151AA	152	257
ESC_PA9150AA	245	261

ESC_PA9149AA	152	257
ESC_PA9148AA	152	257
ESC_PA9147AA	152	257
ESC_PA9146AA	152	257
ESC_PA9145AA	152	257
ESC_PA9144AA	245	261
ESC_PA9143AA	245	261
ESC_PA9142AA	245	261
ESC_PA9141AA	245	261
ESC_PA9140AA	152	257
ESC_PA9139AA	6200	261
ESC_PA9138AA	152	257
ESC_PA9137AA	245	261
ESC_PA9136AA	145	256
ESC_PA9135AA	152	257
ESC_PA9134AA	152	257
ESC_PA9133AA	245	261
ESC_PA9132AA	245	261
ESC_PA9131AA	245	261
ESC_PA9130AA	152	257
ESC_PA9129AA	245	261
ESC_PA9128AA	145	256
ESC_PA9127AA	152	257
ESC_PA9126AA	245	261
ESC_PA9125AA	152	257
ESC_PA9124AA	152	257
ESC_PA9123AA	245	261
ESC_PA9122AA	152	257
ESC_PA9121AA	145	256
ESC_PA9120AA	145	256
ESC_PA9119AA	152	257
ESC_PA9118AA	245	261
ESC_PA9117AA	245	261
ESC_PA9116AA	152	257
ESC_PA9115AA	245	261
ESC_PA9114AA	245	261
ESC_PA9113AA	152	257
ESC_PA9112AA	145	256
ESC_PA9111AA	6200	261
ESC_PA9110AA	6200	261
ESC_PA9109AA	5475	256
ESC_PA9108AA	145	256
ESC_PA9107AA	145	256
ESC_PA9106AA	145	256
ESC_PA9105AA	245	261
ESC_PA9104AA	245	261
ESC_PA9103AA	152	257
ESC_PA9102AA	152	257
ESC_PA9101AA	152	257
ESC_PA9100AA	245	261

ESC_PA9099AA	152	257
ESC_PA9098AA	152	257
ESC_PA9097AA	145	256
ESC_PA9096AA	152	257
ESC_PA9095AA	145	256
ESC_PA9094AA	243	197
ESC_PA9093AA	152	257
ESC_PA9092AA	245	261
ESC_PA9091AA	152	257
ESC_PA9089AA	245	261
ESC_PA9088AA	152	257
ESC_PA9087AA	152	257
ESC_PA9086AA	245	261
ESC_PA9085AA	245	261
ESC_PA9084AA	245	261
ESC_PA9083AA	10653	234
ESC_PA9081AA	152	257
ESC_PA9080AA	152	257
ESC_PA9079AA	243	197
ESC_PA9078AA	152	257
ESC_PA9077AA	152	257
ESC_PA9076AA	152	257
ESC_PA9075AA	152	257
ESC_PA9074AA	152	257
ESC_PA9073AA	152	257
ESC_PA9072AA	152	257
ESC_PA9071AA	245	261
ESC_PA9070AA	145	256
ESC_PA9069AA	152	257
ESC_PA9068AA	152	257
ESC_PA9067AA	245	261
ESC_PA9066AA	245	261
ESC_PA9065AA	6200	261
ESC_PA9064AA	245	261
ESC_PA9063AA	6200	261
ESC_PA9062AA	148	191
ESC_PA9061AA	245	261
ESC_PA9060AA	152	257
ESC_PA9059AA	152	257
ESC_PA9058AA	245	261
ESC_PA9057AA	245	261
ESC_PA9056AA	245	261
ESC_PA9055AA	245	261
ESC_PA9054AA	245	261
ESC_PA9053AA	245	261
ESC_PA9052AA	152	257
ESC_PA9051AA	152	257
ESC_PA9050AA	245	261
ESC_PA9049AA	245	261
ESC_PA9048AA	245	261

ESC_PA9047AA	152	257
ESC_PA9046AA	245	261
ESC_PA9045AA	245	261
ESC_PA9044AA	152	257
ESC_PA9043AA	245	261
ESC_PA9042AA	245	261
ESC_PA9041AA	245	261
ESC_PA9040AA	245	261
ESC_PA9039AA	245	261
ESC_PA9038AA	152	257
ESC_PA9037AA	152	257
ESC_PA9036AA	628	261
ESC_PA9035AA	7039	260
ESC_PA9034AA	152	257
ESC_PA9033AA	152	257
ESC_PA9032AA	152	257
ESC_PA9031AA	152	257
ESC_PA9030AA	152	257
ESC_PA9029AA	152	257
ESC_PA9028AA	152	257
ESC_PA9027AA	6200	261
ESC_PA9026AA	245	261
ESC_PA9025AA	245	261
ESC_PA9024AA	245	261
ESC_PA9023AA	245	261
ESC_PA9022AA	152	257
ESC_PA9021AA	148	191
ESC_PA9020AA	245	261
ESC_PA9019AA	1503	257
ESC_PA9018AA	245	261
ESC_PA9017AA	152	257
ESC_PA9016AA	152	257
ESC_PA9015AA	245	261
ESC_PA9014AA	245	261
ESC_PA9013AA	152	257
ESC_PA9011AA	152	257
ESC_PA9010AA	245	261
ESC_PA9009AA	4267	257
ESC_PA9008AA	152	257
ESC_PA9007AA	152	257
ESC_PA9006AA	245	261
ESC_PA9005AA	152	257
ESC_PA9004AA	245	261
ESC_PA9003AA	245	261
ESC_PA9002AA	152	257
ESC_PA9001AA	152	257
ESC_PA9000AA	245	261
ESC_PA8999AA	152	257
ESC_PA8998AA	245	261
ESC_PA8997AA	152	257

ESC_PA8996AA	245	261
ESC_PA8995AA	1503	257
ESC_PA8994AA	152	257
ESC_PA8993AA	245	261
ESC_PA8992AA	245	261
ESC_PA8991AA	152	257
ESC_PA8990AA	245	261
ESC_PA8989AA	152	257
ESC_PA8988AA	152	257
ESC_PA8987AA	245	261
ESC_PA8986AA	152	257
ESC_PA8985AA	245	261
ESC_PA8984AA	243	197
ESC_PA8983AA	245	261
ESC_PA8982AA	245	261
ESC_PA8981AA	152	257
ESC_PA8980AA	152	257
ESC_PA8979AA	152	257
ESC_PA8978AA	245	261
ESC_PA8977AA	245	261
ESC_PA8976AA	152	257
ESC_PA8975AA	152	257
ESC_PA8974AA	243	197
ESC_PA8973AA	152	257
ESC_PA8972AA	245	261
ESC_PA8971AA	245	261
ESC_PA8970AA	5517	257
ESC_PA8969AA	152	257
ESC_PA8968AA	152	257
ESC_PA8967AA	245	261
ESC_PA8966AA	245	261
ESC_PA8965AA	245	261
ESC_PA8964AA	152	257
ESC_PA8963AA	152	257
ESC_PA8962AA	152	257
ESC_PA8961AA	145	256
ESC_PA8960AA	152	257
ESC_PA8959AA	152	257
ESC_PA8958AA	245	261
ESC_PA8957AA	152	257
ESC_PA8956AA	152	257
ESC_PA8955AA	152	257
ESC_PA8954AA	152	257
ESC_PA8953AA	152	257
ESC_PA8952AA	152	257
ESC_PA8951AA	152	257
ESC_PA8950AA	152	257
ESC_PA8949AA	152	257
ESC_PA8948AA	152	257
ESC_PA8947AA	152	257

ESC_PA8946AA	628	261
ESC_PA8945AA	7384	257
ESC_PA8944AA	152	257
ESC_PA8943AA	6035	257
ESC_PA8942AA	245	261
ESC_PA8941AA	152	257
ESC_PA8940AA	152	257
ESC_PA8939AA	152	257
ESC_PA8938AA	152	257
ESC_PA8937AA	152	257
ESC_PA8936AA	152	257
ESC_PA8935AA	245	261
ESC_PA8934AA	152	257
ESC_PA8933AA	152	257
ESC_PA8932AA	152	257
ESC_PA8931AA	245	261
ESC_PA8930AA	152	257
ESC_PA8929AA	245	261
ESC_PA8928AA	152	257
ESC_PA8927AA	152	257
ESC_PA8926AA	152	257
ESC_PA8925AA	245	261
ESC_PA8924AA	6200	261
ESC_PA8923AA	245	261
ESC_PA8922AA	152	257
ESC_PA8921AA	147	102
ESC_PA8920AA	245	261
ESC_PA8919AA	152	257
ESC_PA8918AA	245	261
ESC_PA8917AA	6200	261
ESC_PA8916AA	245	261
ESC_PA8915AA	152	257
ESC_PA8914AA	152	257
ESC_PA8913AA	245	261
ESC_PA8912AA	245	261
ESC_PA8911AA	245	261
ESC_PA8910AA	152	257
ESC_PA8909AA	245	261
ESC_PA8908AA	152	257
ESC_PA8907AA	152	257
ESC_PA8906AA	152	257
ESC_PA8905AA	145	256
ESC_PA8904AA	245	261
ESC_PA8903AA	245	261
ESC_PA8902AA	152	257
ESC_PA8901AA	152	257
ESC_PA8900AA	152	257
ESC_PA8899AA	245	261
ESC_PA8898AA	152	257
ESC_PA8897AA	5240	261

ESC_PA8896AA	152	257
ESC_PA8895AA	245	261
ESC_PA8894AA	145	256
ESC_PA8893AA	152	257
ESC_PA8892AA	152	257
ESC_PA8891AA	152	257
ESC_PA8890AA	145	256
ESC_PA8889AA	152	257
ESC_PA8888AA	7039	260
ESC_PA8887AA	245	261
ESC_PA8886AA	6200	261
ESC_PA8885AA	245	261
ESC_PA8884AA	152	257
ESC_PA8883AA	245	261
ESC_PA8882AA	245	261
ESC_PA8881AA	152	257
ESC_PA8880AA	245	261
ESC_PA8879AA	245	261
ESC_PA8878AA	245	261
ESC_PA8877AA	245	261
ESC_PA8876AA	152	257
ESC_PA8875AA	152	257
ESC_PA8874AA	152	257
ESC_PA8873AA	152	257
ESC_PA8872AA	245	261
ESC_PA8871AA	145	256
ESC_PA8870AA	152	257
ESC_PA8869AA	152	257
ESC_PA8868AA	152	257
ESC_PA8867AA	152	257
ESC_PA8866AA	152	257
ESC_PA8865AA	6200	261
ESC_PA8864AA	245	261
ESC_PA8863AA	245	261
ESC_PA8862AA	152	257
ESC_PA8861AA	152	257
ESC_PA8860AA	6200	261
ESC_PA8859AA	152	257
ESC_PA8858AA	152	257
ESC_PA8857AA	245	261
ESC_PA8856AA	152	257
ESC_PA8855AA	145	256
ESC_PA8854AA	152	257
ESC_PA8853AA	152	257
ESC_PA8852AA	245	261
ESC_PA8851AA	145	256
ESC_PA8850AA	243	197
ESC_PA8849AA	152	257
ESC_PA8848AA	152	257
ESC_PA8847AA	152	257

ESC_PA8846AA	152	257
ESC_PA8845AA	245	261
ESC_PA8844AA	152	257
ESC_PA8843AA	152	257
ESC_PA8842AA	152	257
ESC_PA8841AA	152	257
ESC_PA8840AA	145	256
ESC_PA8839AA	152	257
ESC_PA8838AA	152	257
ESC_PA8837AA	152	257
ESC_PA8836AA	152	257
ESC_PA8835AA	152	257
ESC_PA8834AA	152	257
ESC_PA8833AA	245	261
ESC_PA8832AA	152	257
ESC_PA8831AA	152	257
ESC_PA8830AA	152	257
ESC_PA8829AA	145	256
ESC_PA8828AA	152	257
ESC_PA8827AA	245	261
ESC_PA8826AA	152	257
ESC_PA8825AA	245	261
ESC_PA8824AA	245	261
ESC_PA8823AA	152	257
ESC_PA8822AA	152	257
ESC_PA8821AA	152	257
ESC_PA8820AA	152	257
ESC_PA8819AA	245	261
ESC_PA8818AA	245	261
ESC_PA8817AA	152	257
ESC_PA8816AA	6200	261
ESC_PA8815AA	245	261
ESC_PA8814AA	6200	261
ESC_PA8813AA	152	257
ESC_PA8812AA	152	257
ESC_PA8811AA	152	257
ESC_PA8810AA	152	257
ESC_PA8809AA	245	261
ESC_PA8808AA	152	257
ESC_PA8807AA	152	257
ESC_PA8806AA	152	257
ESC_PA8805AA	152	257
ESC_PA8804AA	152	257
ESC_PA8803AA	152	257
ESC_PA8802AA	152	257
ESC_PA8801AA	245	261
ESC_PA8800AA	245	261
ESC_PA8799AA	245	261
ESC_PA8798AA	245	261
ESC_PA8797AA	245	261

ESC_PA8796AA	152	257
ESC_PA8795AA	273	102
ESC_PA8794AA	245	261
ESC_PA8793AA	152	257
ESC_PA8792AA	152	257
ESC_PA8791AA	245	261
ESC_PA8790AA	152	257
ESC_PA8789AA	245	261
ESC_PA8788AA	152	257
ESC_PA8787AA	245	261
ESC_PA8786AA	245	261
ESC_PA8785AA	152	257
ESC_PA8784AA	245	261
ESC_PA8783AA	245	261
ESC_PA8782AA	245	261
ESC_PA8781AA	6200	261
ESC_PA8780AA	245	261
ESC_PA8779AA	245	261
ESC_PA8778AA	145	256
ESC_PA8777AA	152	257
ESC_PA8776AA	152	257
ESC_PA8775AA	152	257
ESC_PA8774AA	152	257
ESC_PA8773AA	152	257
ESC_PA8772AA	152	257
ESC_PA8771AA	152	257
ESC_PA8770AA	152	257
ESC_PA8769AA	5517	257
ESC_PA8768AA	245	261
ESC_PA8767AA	152	257
ESC_PA8766AA	245	261
ESC_PA8765AA	245	261
ESC_PA8764AA	245	261
ESC_PA8763AA	152	257
ESC_PA8762AA	6200	261
ESC_PA8761AA	245	261
ESC_PA8760AA	152	257
ESC_PA8759AA	152	257
ESC_PA8758AA	245	261
ESC_PA8757AA	152	257
ESC_PA8756AA	152	257
ESC_PA8755AA	245	261
ESC_PA8754AA	245	261
ESC_PA8753AA	245	261
ESC_PA8752AA	152	257
ESC_PA8751AA	245	261
ESC_PA8750AA	152	257
ESC_PA8749AA	152	257
ESC_PA8748AA	152	257
ESC_PA8747AA	152	257

ESC_PA8746AA	245	261
ESC_PA8745AA	628	261
ESC_PA8744AA	245	261
ESC_PA8743AA	148	191
ESC_PA8742AA	245	261
ESC_PA8741AA	245	261
ESC_PA8740AA	245	261
ESC_PA8739AA	145	256
ESC_PA8738AA	152	257
ESC_PA8737AA	152	257
ESC_PA8736AA	245	261
ESC_PA8735AA	152	257
ESC_PA8734AA	152	257
ESC_PA8733AA	245	261
ESC_PA8732AA	152	257
ESC_PA8730AA	152	257
ESC_PA8729AA	243	197
ESC_PA8728AA	152	257
ESC_PA8727AA	245	261
ESC_PA8726AA	245	261
ESC_PA8725AA	152	257
ESC_PA8724AA	152	257
ESC_PA8723AA	245	261
ESC_PA8722AA	152	257
ESC_PA8721AA	145	256
ESC_PA8720AA	152	257
ESC_PA8719AA	152	257
ESC_PA8718AA	245	261
ESC_PA8717AA	245	261
ESC_PA8716AA	245	261
ESC_PA8715AA	152	257
ESC_PA8714AA	152	257
ESC_PA8713AA	245	261
ESC_PA8712AA	245	261
ESC_PA8711AA	152	257
ESC_PA8710AA	245	261
ESC_PA8709AA	245	261
ESC_PA8708AA	245	261
ESC_PA8707AA	245	261
ESC_PA8706AA	152	257
ESC_PA8705AA	152	257
ESC_PA8704AA	245	261
ESC_PA8703AA	245	261
ESC_PA8702AA	152	257
ESC_PA8700AA	152	257
ESC_PA8699AA	245	261
ESC_PA8698AA	152	257
ESC_PA8696AA	152	257
ESC_PA8695AA	1503	257
ESC_PA8694AA	245	261

ESC_PA8693AA	145	256
ESC_PA8692AA	245	261
ESC_PA8691AA	245	261
ESC_PA8690AA	152	257
ESC_PA8689AA	273	102
ESC_PA8688AA	245	261
ESC_PA8687AA	152	257
ESC_PA8686AA	245	261
ESC_PA8685AA	152	257
ESC_PA8684AA	245	261
ESC_PA8683AA	152	257
ESC_PA8682AA	152	257
ESC_PA8681AA	152	257
ESC_PA8680AA	245	261
ESC_PA8679AA	10652	198
ESC_PA8678AA	10651	257
ESC_PA8677AA	152	257
ESC_PA8676AA	152	257
ESC_PA8675AA	245	261
ESC_PA8674AA	152	257
ESC_PA8673AA	7039	260
ESC_PA8672AA	152	257
ESC_PA8671AA	152	257
ESC_PA8670AA	152	257
ESC_PA8669AA	245	261
ESC_PA8668AA	245	261
ESC_PA8667AA	152	257
ESC_PA8666AA	152	257
ESC_PA8665AA	243	197
ESC_PA8664AA	152	257
ESC_PA8663AA	152	257
ESC_PA8662AA	628	261
ESC_PA8661AA	152	257
ESC_PA8660AA	152	257
ESC_PA8659AA	1502	257
ESC_PA8658AA	152	257
ESC_PA8657AA	245	261
ESC_PA8656AA	152	257
ESC_PA8655AA	152	257
ESC_PA8654AA	152	257
ESC_PA8653AA	152	257
ESC_PA8652AA	152	257
ESC_PA8651AA	152	257
ESC_PA8650AA	152	257
ESC_PA8649AA	152	257
ESC_PA8648AA	152	257
ESC_PA8647AA	1502	257
ESC_PA8646AA	1502	257
ESC_PA8645AA	152	257
ESC_PA8644AA	152	257

ESC_PA8643AA	245	261
ESC_PA8642AA	152	257
ESC_PA8641AA	145	256
ESC_PA8640AA	152	257
ESC_PA8639AA	152	257
ESC_PA8638AA	152	257
ESC_PA8637AA	152	257
ESC_PA8636AA	628	261
ESC_PA8635AA	152	257
ESC_PA8634AA	245	261
ESC_PA8633AA	152	257
ESC_PA8632AA	152	257
ESC_PA8631AA	152	257
ESC_PA8630AA	245	261
ESC_PA8629AA	152	257
ESC_PA8628AA	152	257
ESC_PA8627AA	152	257
ESC_PA8626AA	152	257
ESC_PA8625AA	152	257
ESC_PA8624AA	152	257
ESC_PA8623AA	152	257
ESC_PA8622AA	152	257
ESC_PA8621AA	152	257
ESC_PA8620AA	152	257
ESC_PA8619AA	152	257
ESC_PA8618AA	152	257
ESC_PA8617AA	152	257
ESC_PA8616AA	152	257
ESC_PA8615AA	152	257
ESC_PA8614AA	152	257
ESC_PA8613AA	245	261
ESC_PA8612AA	7384	257
ESC_PA8611AA	152	257
ESC_PA8610AA	7384	257
ESC_PA8609AA	245	261
ESC_PA8608AA	152	257
ESC_PA8607AA	152	257
ESC_PA8606AA	245	261
ESC_PA8605AA	152	257
ESC_PA8604AA	152	257
ESC_PA8603AA	245	261
ESC_PA8602AA	245	261
ESC_PA8601AA	152	257
ESC_PA8600AA	152	257
ESC_PA8599AA	152	257
ESC_PA8598AA	152	257
ESC_PA8597AA	152	257
ESC_PA8596AA	243	197
ESC_PA8595AA	152	257
ESC_PA8594AA	152	257

ESC_PA8593AA	148	191
ESC_PA8592AA	145	256
ESC_PA8591AA	245	261
ESC_PA8590AA	152	257
ESC_PA8589AA	152	257
ESC_PA8588AA	152	257
ESC_PA8587AA	152	257
ESC_PA8586AA	152	257
ESC_PA8585AA	152	257
ESC_PA8584AA	152	257
ESC_PA8583AA	7384	257
ESC_PA8582AA	152	257
ESC_PA8581AA	7384	257
ESC_PA8580AA	152	257
ESC_PA8579AA	7384	257
ESC_PA8578AA	145	256
ESC_PA8577AA	152	257
ESC_PA8576AA	245	261
ESC_PA8575AA	152	257
ESC_PA8574AA	152	257
ESC_PA8573AA	145	256
ESC_PA8572AA	152	257
ESC_PA8571AA	152	257
ESC_PA8570AA	152	257
ESC_PA8569AA	152	257
ESC_PA8568AA	245	261
ESC_PA8567AA	245	261
ESC_PA8566AA	145	256
ESC_PA8565AA	245	261
ESC_PA8564AA	152	257
ESC_PA8563AA	145	256
ESC_PA8562AA	152	257
ESC_PA8561AA	152	257
ESC_PA8560AA	152	257
ESC_PA8559AA	152	257
ESC_PA8558AA	152	257
ESC_PA8557AA	152	257
ESC_PA8556AA	152	257
ESC_PA8555AA	152	257
ESC_PA8554AA	152	257
ESC_PA8553AA	152	257
ESC_PA8552AA	152	257
ESC_PA8551AA	145	256
ESC_PA8550AA	152	257
ESC_PA8549AA	145	256
ESC_PA8548AA	152	257
ESC_PA8547AA	152	257
ESC_PA8546AA	152	257
ESC_PA8545AA	243	197
ESC_PA8544AA	245	261

ESC_PA8543AA	152	257
ESC_PA8541AA	152	257
ESC_PA8540AA	145	256
ESC_PA8539AA	145	256
ESC_PA8538AA	145	256
ESC_PA8537AA	243	197
ESC_PA8536AA	245	261
ESC_PA8535AA	245	261
ESC_PA8534AA	152	257
ESC_PA8533AA	152	257
ESC_PA8532AA	152	257
ESC_PA8531AA	145	256
ESC_PA8530AA	152	257
ESC_PA8529AA	152	257
ESC_PA8528AA	245	261
ESC_PA8527AA	152	257
ESC_PA8526AA	145	256
ESC_PA8525AA	245	261
ESC_PA8524AA	152	257
ESC_PA8523AA	152	257
ESC_PA8522AA	145	256
ESC_PA8521AA	245	261
ESC_PA8520AA	152	257
ESC_PA8519AA	245	261
ESC_PA8518AA	245	261
ESC_PA8517AA	152	257
ESC_PA8516AA	145	256
ESC_PA8515AA	152	257
ESC_PA8514AA	145	256
ESC_PA8513AA	5240	261
ESC_PA8512AA	152	257
ESC_PA8511AA	152	257
ESC_PA8510AA	152	257
ESC_PA8509AA	152	257
ESC_PA8508AA	152	257
ESC_PA8507AA	245	261
ESC_PA8506AA	145	256
ESC_PA8505AA	245	261
ESC_PA8504AA	152	257
ESC_PA8503AA	245	261
ESC_PA8502AA	152	257
ESC_PA8501AA	152	257
ESC_PA8500AA	152	257
ESC_PA8499AA	152	257
ESC_PA8498AA	152	257
ESC_PA8497AA	152	257
ESC_PA8496AA	245	261
ESC_PA8495AA	152	257
ESC_PA8494AA	145	256
ESC_PA8493AA	152	257

ESC_PA8492AA	245	261
ESC_PA8491AA	152	257
ESC_PA8490AA	152	257
ESC_PA8489AA	152	257
ESC_PA8488AA	152	257
ESC_PA8486AA	58	198
ESC_PA8484AA	58	198
ESC_PA8483AA	58	198
ESC_PA8482AA	58	198
ESC_PA8481AA	58	198
ESC_PA8480AA	58	198
ESC_PA8479AA	58	198
ESC_PA8478AA	58	198
ESC_PA8477AA	58	198
ESC_PA8476AA	58	198
ESC_PA8475AA	58	198
ESC_PA8474AA	58	198
ESC_PA8473AA	58	198
ESC_PA8472AA	58	198
ESC_PA8471AA	58	198
ESC_PA8470AA	58	198
ESC_PA8469AA	58	198
ESC_PA8468AA	58	198
ESC_PA8467AA	58	198
ESC_PA8466AA	58	198
ESC_PA8465AA	58	198
ESC_PA8464AA	58	198
ESC_PA8463AA	58	198
ESC_PA8462AA	58	198
ESC_PA8461AA	58	198
ESC_PA8460AA	58	198
ESC_PA8459AA	58	198
ESC_PA8458AA	58	198
ESC_PA8457AA	58	198
ESC_PA8456AA	58	198
ESC_PA8455AA	58	198
ESC_PA8454AA	58	198
ESC_PA8453AA	58	198
ESC_PA8452AA	245	261
ESC_PA8451AA	152	257
ESC_PA8450AA	58	198
ESC_PA8449AA	58	198
ESC_PA8448AA	58	198
ESC_PA8447AA	58	198
ESC_PA8446AA	58	198
ESC_PA8445AA	58	198
ESC_PA8444AA	58	198
ESC_PA8443AA	58	198
ESC_PA8442AA	58	198
ESC_PA8441AA	58	198

ESC_PA8440AA	58	198
ESC_PA8438AA	58	198
ESC_PA8437AA	58	198
ESC_PA8436AA	58	198
ESC_PA8435AA	58	198
ESC_PA8434AA	58	198
ESC_PA8433AA	58	198
ESC_PA8432AA	58	198
ESC_PA8431AA	58	198
ESC_PA8430AA	58	198
ESC_PA8429AA	58	198
ESC_PA8428AA	58	198
ESC_PA8427AA	58	198
ESC_PA8426AA	58	198
ESC_PA8425AA	58	198
ESC_PA8424AA	58	198
ESC_PA8423AA	58	198
ESC_PA8422AA	58	198
ESC_PA8421AA	58	198
ESC_PA8420AA	58	198
ESC_PA8419AA	58	198
ESC_PA8418AA	58	198
ESC_PA8417AA	58	198
ESC_PA8416AA	58	198
ESC_PA8415AA	58	198
ESC_PA8414AA	58	198
ESC_PA8413AA	58	198
ESC_PA8412AA	58	198
ESC_PA8411AA	58	198
ESC_PA8410AA	58	198
ESC_PA8409AA	58	198
ESC_PA8408AA	58	198
ESC_PA8407AA	58	198
ESC_PA8406AA	58	198
ESC_PA8405AA	58	198
ESC_PA8404AA	58	198
ESC_PA8403AA	58	198
ESC_PA8402AA	58	198
ESC_PA8401AA	58	198
ESC_PA8400AA	58	198
ESC_PA8399AA	58	198
ESC_PA8398AA	58	198
ESC_PA8397AA	58	198
ESC_PA8396AA	58	198
ESC_PA8395AA	58	198
ESC_PA8394AA	58	198
ESC_PA8392AA	58	198
ESC_PA8391AA	58	198
ESC_PA8390AA	58	198
ESC_PA8389AA	58	198

ESC_PA8388AA	58	198
ESC_PA8387AA	58	198
ESC_PA8386AA	58	198
ESC_PA8385AA	58	198
ESC_PA8384AA	58	198
ESC_PA8383AA	58	198
ESC_PA8381AA	58	198
ESC_PA8380AA	58	198
ESC_PA8379AA	58	198
ESC_PA8378AA	58	198
ESC_PA8376AA	58	198
ESC_PA8375AA	152	257
ESC_PA8374AA	245	261
ESC_PA8373AA	152	257
ESC_PA8372AA	152	257
ESC_PA8371AA	152	257
ESC_PA8370AA	152	257
ESC_PA8369AA	152	257
ESC_PA8368AA	152	257
ESC_PA8367AA	152	257
ESC_PA8366AA	152	257
ESC_PA8364AA	152	257
ESC_PA8362AA	245	261
ESC_PA8361AA	145	256
ESC_PA8360AA	245	261
ESC_PA8359AA	245	261
ESC_PA8358AA	152	257
ESC_PA8357AA	245	261
ESC_PA8356AA	6035	257
ESC_PA8355AA	6035	257
ESC_PA8354AA	152	257
ESC_PA8353AA	152	257
ESC_PA8352AA	245	261
ESC_PA8351AA	152	257
ESC_PA8350AA	245	261
ESC_PA8349AA	6200	261
ESC_PA8348AA	152	257
ESC_PA8347AA	245	261
ESC_PA8346AA	245	261
ESC_PA8345AA	152	257
ESC_PA8344AA	152	257
ESC_PA8343AA	152	257
ESC_PA8342AA	245	261
ESC_PA8341AA	245	261
ESC_PA8340AA	152	257
ESC_PA8339AA	145	256
ESC_PA8338AA	145	256
ESC_PA8337AA	145	256
ESC_PA8336AA	152	257
ESC_PA8335AA	152	257

ESC_PA8334AA	245	261
ESC_PA8333AA	5592	830
ESC_PA8332AA	245	261
ESC_PA8331AA	152	257
ESC_PA8330AA	152	257
ESC_PA8329AA	152	257
ESC_PA8328AA	245	261
ESC_PA8327AA	152	257
ESC_PA8326AA	245	261
ESC_PA8325AA	152	257
ESC_PA8324AA	152	257
ESC_PA8323AA	152	257
ESC_PA8322AA	152	257
ESC_PA8321AA	152	257
ESC_PA8320AA	152	257
ESC_PA8319AA	152	257
ESC_PA8318AA	152	257
ESC_PA8317AA	152	257
ESC_PA8316AA	145	256
ESC_PA8315AA	152	257
ESC_PA8314AA	152	257
ESC_PA8313AA	245	261
ESC_PA8312AA	152	257
ESC_PA8311AA	152	257
ESC_PA8310AA	152	257
ESC_PA8309AA	245	261
ESC_PA8308AA	152	257
ESC_PA8307AA	245	261
ESC_PA8306AA	145	256
ESC_PA8305AA	245	261
ESC_PA8304AA	145	256
ESC_PA8303AA	245	261
ESC_PA8302AA	152	257
ESC_PA8301AA	152	257
ESC_PA8300AA	152	257
ESC_PA8299AA	245	261
ESC_PA8298AA	152	257
ESC_PA8297AA	152	257
ESC_PA8296AA	152	257
ESC_PA8295AA	152	257
ESC_PA8294AA	152	257
ESC_PA8293AA	152	257
ESC_PA8292AA	152	257
ESC_PA8291AA	145	256
ESC_PA8290AA	245	261
ESC_PA8289AA	152	257
ESC_PA8288AA	152	257
ESC_PA8287AA	152	257
ESC_PA8286AA	152	257
ESC_PA8285AA	152	257

ESC_PA8284AA	245	261
ESC_PA8283AA	245	261
ESC_PA8282AA	152	257
ESC_PA8281AA	152	257
ESC_PA8280AA	245	261
ESC_PA8279AA	58	198
ESC_PA8278AA	58	198
ESC_PA8277AA	58	198
ESC_PA8276AA	58	198
ESC_PA8275AA	58	198
ESC_PA8274AA	167	512
ESC_PA8273AA	58	198
ESC_PA8272AA	58	198
ESC_PA8271AA	58	198
ESC_PA8270AA	58	198
ESC_PA8269AA	58	198
ESC_PA8268AA	58	198
ESC_PA8267AA	58	198
ESC_PA8266AA	58	198
ESC_PA8265AA	58	198
ESC_PA8264AA	58	198
ESC_PA8263AA	58	198
ESC_PA8262AA	58	198
ESC_PA8261AA	58	198
ESC_PA8260AA	58	198
ESC_PA8258AA	58	198
ESC_PA8257AA	58	198
ESC_PA8256AA	58	198
ESC_PA8255AA	58	198
ESC_PA8253AA	58	198
ESC_PA8252AA	58	198
ESC_PA8251AA	58	198
ESC_PA8250AA	58	198
ESC_PA8249AA	58	198
ESC_PA8248AA	58	198
ESC_PA8247AA	58	198
ESC_PA8246AA	58	198
ESC_PA8245AA	58	198
ESC_PA8244AA	58	198
ESC_PA8243AA	58	198
ESC_PA8242AA	58	198
ESC_PA8241AA	58	198
ESC_PA8240AA	58	198
ESC_PA8239AA	58	198
ESC_PA8238AA	58	198
ESC_PA8237AA	58	198
ESC_PA8236AA	58	198
ESC_PA8235AA	58	198
ESC_PA8233AA	58	198
ESC_PA8232AA	58	198

ESC_PA8231AA	58	198
ESC_PA8230AA	58	198
ESC_PA8229AA	58	198
ESC_PA8228AA	58	198
ESC_PA8227AA	58	198
ESC_PA8226AA	58	198
ESC_PA8225AA	58	198
ESC_PA8223AA	58	198
ESC_PA8222AA	58	198
ESC_PA8221AA	1586	198
ESC_PA8220AA	58	198
ESC_PA8217AA	58	198
ESC_PA8216AA	58	198
ESC_PA8215AA	58	198
ESC_PA8214AA	58	198
ESC_PA8213AA	58	198
ESC_PA8212AA	58	198
ESC_PA8211AA	58	198
ESC_PA8210AA	58	198
ESC_PA8208AA	58	198
ESC_PA8207AA	58	198
ESC_PA8205AA	58	198
ESC_PA8204AA	58	198
ESC_PA8203AA	58	198
ESC_PA8202AA	58	198
ESC_PA8201AA	58	198
ESC_PA8200AA	245	261
ESC_PA8199AA	152	257
ESC_PA8198AA	152	257
ESC_PA8197AA	152	257
ESC_PA8196AA	245	261
ESC_PA8195AA	245	261
ESC_PA8194AA	152	257
ESC_PA8193AA	145	256
ESC_PA8192AA	152	257
ESC_PA8191AA	155	175
ESC_PA8185AA	245	261
ESC_PA8184AA	148	191
ESC_PA8183AA	152	257
ESC_PA8182AA	152	257
ESC_PA8181AA	152	257
ESC_PA8180AA	152	257
ESC_PA8179AA	152	257
ESC_PA8178AA	152	257
ESC_PA8177AA	152	257
ESC_PA8176AA	152	257
ESC_PA8175AA	152	257
ESC_PA8174AA	152	257
ESC_PA8173AA	152	257
ESC_PA8172AA	152	257

ESC_PA8171AA	152	257
ESC_PA8170AA	152	257
ESC_PA8169AA	245	261
ESC_PA8168AA	152	257
ESC_PA8167AA	245	261
ESC_PA8166AA	152	257
ESC_PA8165AA	152	257
ESC_PA8164AA	245	261
ESC_PA8163AA	301	307
ESC_PA8162AA	301	307
ESC_PA8161AA	301	307
ESC_PA8160AA	301	307
ESC_PA8159AA	155	175
ESC_PA8158AA	58	198
ESC_PA8157AA	58	198
ESC_PA8156AA	152	257
ESC_PA8155AA	1025	261
ESC_PA8154AA	245	261
ESC_PA8153AA	152	257
ESC_PA8152AA	245	261
ESC_PA8151AA	245	261
ESC_PA8150AA	152	257
ESC_PA8149AA	245	261
ESC_PA8148AA	5475	256
ESC_PA8147AA	245	261
ESC_PA8146AA	245	261
ESC_PA8145AA	245	261
ESC_PA8144AA	245	261
ESC_PA8143AA	152	257
ESC_PA8142AA	152	257
ESC_PA8141AA	152	257
ESC_PA8140AA	648	359
ESC_PA8139AA	93	71
ESC_PA8138AA	6856	76
ESC_PA8137AA	962	198
ESC_PA8136AA	457	595
ESC_PA8135AA	38	669
ESC_PA8134AA	10648	299
ESC_PA8133AA	117	592
ESC_PA8132AA	69	387
ESC_PA8131AA	2522	198
ESC_PA8130AA	10614	926
ESC_PA8128AA	10614	926
ESC_PA8127AA	1771	868
ESC_PA8126AA	718	257
ESC_PA8125AA	90	156
ESC_PA8050AA	10	512
ESC_PA8049AA	101	696
ESC_PA8048AA	44	512
ESC_PA8047AA	354	15

ESC_PA8046AA	10650	843
ESC_PA8045AA	10	512
ESC_PA8044AA	10	512
ESC_PA8043AA	3288	286
ESC_PA8042AA	10649	511
ESC_PA8041AA	1001	257
ESC_PA8040AA	744	512
ESC_PA8039AA	10	512
ESC_PA8038AA	542	824
ESC_PA8037AA	6913	512
ESC_PA8036AA	215	512
ESC_PA8035AA	8128	234
ESC_PA8034AA	117	592
ESC_PA8033AA	1286	474
ESC_PA8032AA	58	198
ESC_PA8031AA	2076	400
ESC_PA8030AA	328	924
ESC_PA8029AA	877	935
ESC_PA8028AA	394	400
ESC_PA8027AA	2003	669
ESC_PA8026AA	3171	515
ESC_PA8025AA	394	400
ESC_PA8024AA	10	512
ESC_PA8023AA	1163	579
ESC_PA8022AA	155	175
ESC_PA8021AA	648	359
ESC_PA8020AA	6778	198
ESC_PA8019AA	38	669
ESC_PA8018AA	156	175
ESC_PA8017AA	349	670
ESC_PA8016AA	394	400
ESC_PA8015AA	394	400
ESC_PA8014AA	189	504
ESC_PA8013AA	43	512
ESC_PA8012AA	226	512
ESC_PA8011AA	224	198
ESC_PA8010AA	10	512
ESC_PA8009AA	10	512
ESC_PA8008AA	10	512
ESC_PA8007AA	10	512
ESC_PA8006AA	10	512
ESC_PA8005AA	10	512
ESC_PA8004AA	10	512
ESC_PA8003AA	10	512
ESC_PA8002AA	10	512
ESC_PA8001AA	10	512
ESC_PA8000AA	10	512
ESC_PA7999AA	10	512
ESC_PA7998AA	10	512
ESC_PA7997AA	10	512

ESC_PA7996AA	10	512
ESC_PA7995AA	10	512
ESC_PA7994AA	10	512
ESC_PA7993AA	10	512
ESC_PA7992AA	10	512
ESC_PA7991AA	10	512
ESC_PA7990AA	10	512
ESC_PA7989AA	10	512
ESC_PA7988AA	10	512
ESC_PA7987AA	10	512
ESC_PA7986AA	10	512
ESC_PA7985AA	10	512
ESC_PA7984AA	10	512
ESC_PA7983AA	10	512
ESC_PA7982AA	10	512
ESC_PA7981AA	10	512
ESC_PA7980AA	10	512
ESC_PA7979AA	10	512
ESC_PA7978AA	10	512
ESC_PA7977AA	10	512
ESC_PA7976AA	10	512
ESC_PA7975AA	10	512
ESC_PA7974AA	10	512
ESC_PA7973AA	10	512
ESC_PA7972AA	10	512
ESC_PA7959AA	648	359
ESC_PA7958AA	648	359
ESC_PA7957AA	648	359
ESC_PA7956AA	648	359
ESC_PA7955AA	648	359
ESC_PA7954AA	648	359
ESC_PA7953AA	12	626
ESC_PA7952AA	12	626
ESC_PA7951AA	12	626
ESC_PA7950AA	12	626
ESC_PA7949AA	12	626
ESC_PA7948AA	12	626
ESC_PA7897AA	10	512
ESC_PA7896AA	90	156
ESC_PA7895AA	372	424
ESC_PA7894AA	336	878
ESC_PA7893AA	1025	261
ESC_PA7892AA	245	261
ESC_PA7891AA	245	261
ESC_PA7890AA	245	261
ESC_PA7889AA	245	261
ESC_PA7888AA	245	261
ESC_PA7887AA	628	261
ESC_PA7886AA	245	261
ESC_PA7885AA	245	261

ESC_PA7884AA	245	261
ESC_PA7883AA	245	261
ESC_PA7882AA	245	261
ESC_PA7881AA	630	261
ESC_PA7880AA	245	261
ESC_PA7879AA	245	261
ESC_PA7878AA	245	261
ESC_PA7877AA	245	261
ESC_PA7876AA	245	261
ESC_PA7875AA	245	261
ESC_PA7874AA	245	261
ESC_PA7873AA	245	261
ESC_PA7872AA	245	261
ESC_PA7871AA	245	261
ESC_PA7870AA	245	261
ESC_PA7869AA	245	261
ESC_PA7868AA	245	261
ESC_PA7867AA	245	261
ESC_PA7866AA	245	261
ESC_PA7865AA	245	261
ESC_PA7864AA	630	261
ESC_PA7863AA	7384	257
ESC_PA7862AA	7384	257
ESC_PA7861AA	145	256
ESC_PA7860AA	245	261
ESC_PA7859AA	245	261
ESC_PA7858AA	7384	257
ESC_PA7857AA	245	261
ESC_PA7856AA	245	261
ESC_PA7855AA	245	261
ESC_PA7854AA	245	261
ESC_PA7853AA	245	261
ESC_PA7852AA	245	261
ESC_PA7851AA	245	261
ESC_PA7850AA	245	261
ESC_PA7849AA	245	261
ESC_PA7848AA	245	261
ESC_PA7847AA	245	261
ESC_PA7846AA	245	261
ESC_PA7845AA	245	261
ESC_PA7844AA	245	261
ESC_PA7843AA	245	261
ESC_PA7842AA	245	261
ESC_PA7841AA	145	256
ESC_PA7840AA	245	261
ESC_PA7839AA	245	261
ESC_PA7838AA	145	256
ESC_PA7837AA	245	261
ESC_PA7836AA	245	261
ESC_PA7835AA	245	261

ESC_PA7834AA	245	261
ESC_PA7833AA	245	261
ESC_PA7832AA	245	261
ESC_PA7831AA	245	261
ESC_PA7830AA	245	261
ESC_PA7829AA	145	256
ESC_PA7828AA	245	261
ESC_PA7827AA	145	256
ESC_PA7826AA	245	261
ESC_PA7825AA	245	261
ESC_PA7824AA	245	261
ESC_PA7823AA	245	261
ESC_PA7822AA	145	256
ESC_PA7821AA	245	261
ESC_PA7820AA	245	261
ESC_PA7819AA	245	261
ESC_PA7818AA	245	261
ESC_PA7817AA	245	261
ESC_PA7816AA	245	261
ESC_PA7815AA	245	261
ESC_PA7814AA	245	261
ESC_PA7813AA	245	261
ESC_PA7812AA	245	261
ESC_PA7811AA	245	261
ESC_PA7810AA	245	261
ESC_PA7809AA	245	261
ESC_PA7808AA	245	261
ESC_PA7807AA	7384	257
ESC_PA7806AA	245	261
ESC_PA7805AA	630	261
ESC_PA7804AA	630	261
ESC_PA7803AA	245	261
ESC_PA7802AA	245	261
ESC_PA7801AA	7384	257
ESC_PA7800AA	245	261
ESC_PA7799AA	245	261
ESC_PA7798AA	245	261
ESC_PA7797AA	245	261
ESC_PA7796AA	245	261
ESC_PA7795AA	145	256
ESC_PA7794AA	245	261
ESC_PA7793AA	245	261
ESC_PA7792AA	245	261
ESC_PA7791AA	145	256
ESC_PA7790AA	245	261
ESC_PA7789AA	245	261
ESC_PA7788AA	145	256
ESC_PA7787AA	245	261
ESC_PA7786AA	245	261
ESC_PA7785AA	245	261

ESC_PA7784AA	245	261
ESC_PA7783AA	245	261
ESC_PA7782AA	245	261
ESC_PA7780AA	152	257
ESC_PA7779AA	245	261
ESC_PA7778AA	152	257
ESC_PA7777AA	1025	261
ESC_PA7776AA	152	257
ESC_PA7775AA	245	261
ESC_PA7774AA	245	261
ESC_PA7773AA	245	261
ESC_PA7772AA	152	257
ESC_PA7771AA	245	261
ESC_PA7769AA	10	512
ESC_PA7768AA	100	504
ESC_PA7767AA	100	504
ESC_PA7766AA	90	156
ESC_PA7765AA	362	234
ESC_PA7764AA	10647	257
ESC_PA7763AA	10	512
ESC_PA7761AA	90	156
ESC_PA7760AA	100	504
ESC_PA7759AA	90	156
ESC_PA7758AA	90	156
ESC_PA7757AA	10	512
ESC_PA7756AA	90	156
ESC_PA7755AA	3580	257
ESC_PA7754AA	10	512
ESC_PA7753AA	100	504
ESC_PA7752AA	100	504
ESC_PA7751AA	953	447
ESC_PA7750AA	90	156
ESC_PA7749AA	90	156
ESC_PA7748AA	8496	382
ESC_PA7747AA	602	257
ESC_PA7746AA	2951	587
ESC_PA7745AA	155	175
ESC_PA7744AA	5296	257
ESC_PA7743AA	737	175
ESC_PA7742AA	336	878
ESC_PA7741AA	5780	476
ESC_PA7740AA	135	653
ESC_PA7739AA	245	261
ESC_PA7738AA	245	261
ESC_PA7737AA	245	261
ESC_PA7736AA	245	261
ESC_PA7735AA	145	256
ESC_PA7734AA	245	261
ESC_PA7733AA	245	261
ESC_PA7732AA	245	261

ESC_PA7731AA	245	261
ESC_PA7730AA	245	261
ESC_PA7729AA	145	256
ESC_PA7728AA	245	261
ESC_PA7727AA	7384	257
ESC_PA7726AA	245	261
ESC_PA7725AA	145	256
ESC_PA7724AA	245	261
ESC_PA7723AA	245	261
ESC_PA7722AA	245	261
ESC_PA7721AA	245	261
ESC_PA7720AA	245	261
ESC_PA7719AA	245	261
ESC_PA7718AA	245	261
ESC_PA7717AA	145	256
ESC_PA7716AA	245	261
ESC_PA7715AA	245	261
ESC_PA7714AA	245	261
ESC_PA7713AA	245	261
ESC_PA7712AA	245	261
ESC_PA7711AA	245	261
ESC_PA7710AA	245	261
ESC_PA7709AA	245	261
ESC_PA7708AA	245	261
ESC_PA7707AA	245	261
ESC_PA7706AA	245	261
ESC_PA7705AA	245	261
ESC_PA7704AA	245	261
ESC_PA7703AA	245	261
ESC_PA7702AA	245	261
ESC_PA7701AA	245	261
ESC_PA7700AA	245	261
ESC_PA7699AA	245	261
ESC_PA7698AA	245	261
ESC_PA7697AA	245	261
ESC_PA7696AA	245	261
ESC_PA7695AA	245	261
ESC_PA7694AA	245	261
ESC_PA7693AA	145	256
ESC_PA7692AA	245	261
ESC_PA7691AA	145	256
ESC_PA7690AA	630	261
ESC_PA7689AA	245	261
ESC_PA7688AA	245	261
ESC_PA7687AA	245	261
ESC_PA7686AA	245	261
ESC_PA7685AA	245	261
ESC_PA7684AA	145	256
ESC_PA7683AA	245	261
ESC_PA7682AA	245	261

ESC_PA7681AA	245	261
ESC_PA7680AA	245	261
ESC_PA7679AA	7384	257
ESC_PA7678AA	7384	257
ESC_PA7677AA	7384	257
ESC_PA7676AA	7384	257
ESC_PA7675AA	245	261
ESC_PA7674AA	245	261
ESC_PA7673AA	245	261
ESC_PA7672AA	245	261
ESC_PA7671AA	7384	257
ESC_PA7670AA	7384	257
ESC_PA7669AA	245	261
ESC_PA7668AA	145	256
ESC_PA7667AA	7384	257
ESC_PA7666AA	145	256
ESC_PA7665AA	245	261
ESC_PA7664AA	245	261
ESC_PA7663AA	245	261
ESC_PA7662AA	245	261
ESC_PA7661AA	245	261
ESC_PA7660AA	245	261
ESC_PA7659AA	245	261
ESC_PA7658AA	6200	261
ESC_PA7657AA	245	261
ESC_PA7656AA	245	261
ESC_PA7655AA	245	261
ESC_PA7654AA	245	261
ESC_PA7653AA	245	261
ESC_PA7652AA	245	261
ESC_PA7651AA	145	256
ESC_PA7650AA	245	261
ESC_PA7649AA	245	261
ESC_PA7648AA	245	261
ESC_PA7647AA	245	261
ESC_PA7646AA	145	256
ESC_PA7645AA	245	261
ESC_PA7644AA	245	261
ESC_PA7643AA	245	261
ESC_PA7642AA	245	261
ESC_PA7641AA	245	261
ESC_PA7640AA	245	261
ESC_PA7639AA	245	261
ESC_PA7638AA	245	261
ESC_PA7637AA	245	261
ESC_PA7636AA	245	261
ESC_PA7635AA	6200	261
ESC_PA7634AA	6369	261
ESC_PA7633AA	245	261
ESC_PA7632AA	245	261

ESC_PA7631AA	245	261
ESC_PA7630AA	245	261
ESC_PA7629AA	245	261
ESC_PA7628AA	245	261
ESC_PA7627AA	245	261
ESC_PA7626AA	245	261
ESC_PA7625AA	245	261
ESC_PA7624AA	245	261
ESC_PA7623AA	245	261
ESC_PA7622AA	245	261
ESC_PA7621AA	245	261
ESC_PA7620AA	245	261
ESC_PA7619AA	245	261
ESC_PA7618AA	245	261
ESC_PA7617AA	245	261
ESC_PA7616AA	245	261
ESC_PA7615AA	7039	260
ESC_PA7614AA	245	261
ESC_PA7613AA	245	261
ESC_PA7612AA	245	261
ESC_PA7611AA	245	261
ESC_PA7610AA	245	261
ESC_PA7609AA	245	261
ESC_PA7608AA	245	261
ESC_PA7607AA	145	256
ESC_PA7606AA	245	261
ESC_PA7605AA	245	261
ESC_PA7604AA	245	261
ESC_PA7603AA	245	261
ESC_PA7602AA	245	261
ESC_PA7601AA	245	261
ESC_PA7600AA	245	261
ESC_PA7599AA	245	261
ESC_PA7598AA	145	256
ESC_PA7597AA	245	261
ESC_PA7596AA	245	261
ESC_PA7595AA	245	261
ESC_PA7594AA	245	261
ESC_PA7593AA	245	261
ESC_PA7592AA	245	261
ESC_PA7591AA	245	261
ESC_PA7590AA	245	261
ESC_PA7589AA	245	261
ESC_PA7588AA	245	261
ESC_PA7587AA	145	256
ESC_PA7586AA	245	261
ESC_PA7585AA	145	256
ESC_PA7584AA	145	256
ESC_PA7583AA	7384	257
ESC_PA7582AA	245	261

ESC_PA7581AA	7384	257
ESC_PA7580AA	7384	257
ESC_PA7579AA	245	261
ESC_PA7578AA	7384	257
ESC_PA7577AA	7384	257
ESC_PA7576AA	245	261
ESC_PA7575AA	7384	257
ESC_PA7574AA	245	261
ESC_PA7573AA	145	256
ESC_PA7572AA	145	256
ESC_PA7571AA	245	261
ESC_PA7570AA	245	261
ESC_PA7569AA	245	261
ESC_PA7568AA	245	261
ESC_PA7567AA	245	261
ESC_PA7566AA	245	261
ESC_PA7565AA	245	261
ESC_PA7564AA	245	261
ESC_PA7563AA	245	261
ESC_PA7562AA	245	261
ESC_PA7561AA	245	261
ESC_PA7560AA	145	256
ESC_PA7559AA	245	261
ESC_PA7558AA	245	261
ESC_PA7557AA	245	261
ESC_PA7556AA	245	261
ESC_PA7555AA	245	261
ESC_PA7554AA	245	261
ESC_PA7553AA	245	261
ESC_PA7552AA	245	261
ESC_PA7551AA	245	261
ESC_PA7550AA	7384	257
ESC_PA7549AA	628	261
ESC_PA7548AA	245	261
ESC_PA7547AA	245	261
ESC_PA7546AA	245	261
ESC_PA7545AA	245	261
ESC_PA7544AA	245	261
ESC_PA7543AA	6200	261
ESC_PA7542AA	245	261
ESC_PA7541AA	245	261
ESC_PA7540AA	145	256
ESC_PA7539AA	245	261
ESC_PA7538AA	245	261
ESC_PA7537AA	245	261
ESC_PA7536AA	245	261
ESC_PA7535AA	245	261
ESC_PA7534AA	245	261
ESC_PA7533AA	245	261
ESC_PA7532AA	245	261

ESC_PA7531AA	245	261
ESC_PA7530AA	245	261
ESC_PA7529AA	245	261
ESC_PA7528AA	245	261
ESC_PA7527AA	245	261
ESC_PA7526AA	245	261
ESC_PA7525AA	245	261
ESC_PA7524AA	145	256
ESC_PA7523AA	245	261
ESC_PA7522AA	245	261
ESC_PA7521AA	245	261
ESC_PA7520AA	245	261
ESC_PA7519AA	245	261
ESC_PA7518AA	245	261
ESC_PA7517AA	6370	256
ESC_PA7516AA	145	256
ESC_PA7515AA	245	261
ESC_PA7514AA	245	261
ESC_PA7513AA	245	261
ESC_PA7512AA	245	261
ESC_PA7511AA	245	261
ESC_PA7510AA	245	261
ESC_PA7509AA	145	256
ESC_PA7508AA	245	261
ESC_PA7507AA	245	261
ESC_PA7506AA	245	261
ESC_PA7505AA	245	261
ESC_PA7504AA	145	256
ESC_PA7503AA	245	261
ESC_PA7502AA	145	256
ESC_PA7501AA	245	261
ESC_PA7500AA	145	256
ESC_PA7499AA	245	261
ESC_PA7498AA	245	261
ESC_PA7497AA	245	261
ESC_PA7496AA	245	261
ESC_PA7495AA	245	261
ESC_PA7494AA	245	261
ESC_PA7493AA	245	261
ESC_PA7492AA	245	261
ESC_PA7491AA	245	261
ESC_PA7490AA	245	261
ESC_PA7489AA	245	261
ESC_PA7488AA	245	261
ESC_PA7487AA	1025	261
ESC_PA7486AA	1025	261
ESC_PA7485AA	245	261
ESC_PA7484AA	245	261
ESC_PA7483AA	245	261
ESC_PA7482AA	145	256

ESC_PA7481AA	245	261
ESC_PA7480AA	245	261
ESC_PA7479AA	245	261
ESC_PA7478AA	245	261
ESC_PA7477AA	145	256
ESC_PA7476AA	245	261
ESC_PA7475AA	245	261
ESC_PA7474AA	245	261
ESC_PA7473AA	245	261
ESC_PA7472AA	1025	261
ESC_PA7471AA	245	261
ESC_PA7470AA	245	261
ESC_PA7469AA	7384	257
ESC_PA7468AA	245	261
ESC_PA7467AA	245	261
ESC_PA7466AA	245	261
ESC_PA7465AA	245	261
ESC_PA7464AA	245	261
ESC_PA7463AA	245	261
ESC_PA7462AA	245	261
ESC_PA7461AA	245	261
ESC_PA7460AA	245	261
ESC_PA7459AA	245	261
ESC_PA7458AA	145	256
ESC_PA7457AA	245	261
ESC_PA7456AA	7384	257
ESC_PA7455AA	245	261
ESC_PA7454AA	5312	235
ESC_PA7453AA	245	261
ESC_PA7452AA	245	261
ESC_PA7451AA	245	261
ESC_PA7450AA	6200	261
ESC_PA7449AA	245	261
ESC_PA7448AA	245	261
ESC_PA7447AA	245	261
ESC_PA7446AA	245	261
ESC_PA7445AA	245	261
ESC_PA7444AA	245	261
ESC_PA7443AA	245	261
ESC_PA7442AA	6200	261
ESC_PA7441AA	245	261
ESC_PA7440AA	245	261
ESC_PA7439AA	245	261
ESC_PA7438AA	245	261
ESC_PA7437AA	245	261
ESC_PA7436AA	245	261
ESC_PA7435AA	145	256
ESC_PA7434AA	245	261
ESC_PA7433AA	245	261
ESC_PA7432AA	245	261

ESC_PA7431AA	245	261
ESC_PA7430AA	245	261
ESC_PA7429AA	245	261
ESC_PA7428AA	245	261
ESC_PA7427AA	6200	261
ESC_PA7426AA	245	261
ESC_PA7425AA	245	261
ESC_PA7424AA	245	261
ESC_PA7423AA	245	261
ESC_PA7422AA	245	261
ESC_PA7421AA	7384	257
ESC_PA7420AA	245	261
ESC_PA7419AA	7384	257
ESC_PA7418AA	245	261
ESC_PA7417AA	245	261
ESC_PA7416AA	245	261
ESC_PA7415AA	245	261
ESC_PA7414AA	245	261
ESC_PA7413AA	245	261
ESC_PA7412AA	145	256
ESC_PA7411AA	245	261
ESC_PA7410AA	245	261
ESC_PA7409AA	245	261
ESC_PA7408AA	245	261
ESC_PA7407AA	245	261
ESC_PA7406AA	245	261
ESC_PA7405AA	245	261
ESC_PA7404AA	245	261
ESC_PA7403AA	245	261
ESC_PA7402AA	245	261
ESC_PA7401AA	245	261
ESC_PA7400AA	7384	257
ESC_PA7399AA	245	261
ESC_PA7398AA	245	261
ESC_PA7397AA	245	261
ESC_PA7396AA	245	261
ESC_PA7395AA	245	261
ESC_PA7394AA	6200	261
ESC_PA7393AA	245	261
ESC_PA7392AA	245	261
ESC_PA7391AA	245	261
ESC_PA7390AA	245	261
ESC_PA7389AA	73	737
ESC_PA7388AA	1973	291
ESC_PA7387AA	10499	387
ESC_PA7386AA	877	935
ESC_PA7385AA	10	512
ESC_PA7384AA	38	669
ESC_PA7383AA	515	1004
ESC_PA7382AA	4956	198

ESC_PA7381AA	641	935
ESC_PA7380AA	10	512
ESC_PA7379AA	226	512
ESC_PA7378AA	1286	474
ESC_PA7377AA	156	175
ESC_PA7376AA	5229	696
ESC_PA7375AA	10	512
ESC_PA7374AA	410	156
ESC_PA7373AA	1121	198
ESC_PA7372AA	10645	519
ESC_PA7371AA	744	512
ESC_PA7370AA	2170	234
ESC_PA7369AA	2705	504
ESC_PA7368AA	162	924
ESC_PA7367AA	877	935
ESC_PA7366AA	515	1004
ESC_PA7365AA	515	1004
ESC_PA7364AA	6214	512
ESC_PA7363AA	6775	1004
ESC_PA7362AA	515	1004
ESC_PA7361AA	336	878
ESC_PA7360AA	336	878
ESC_PA7358AA	10644	424
ESC_PA7357AA	10643	665
ESC_PA7356AA	152	257
ESC_PA7355AA	152	257
ESC_PA7354AA	152	257
ESC_PA7353AA	6200	261
ESC_PA7352AA	152	257
ESC_PA7351AA	245	261
ESC_PA7350AA	152	257
ESC_PA7349AA	152	257
ESC_PA7348AA	152	257
ESC_PA7347AA	245	261
ESC_PA7346AA	152	257
ESC_PA7345AA	93	71
ESC_PA7344AA	93	71
ESC_PA7343AA	11	936
ESC_PA7342AA	11	936
ESC_PA7341AA	11	936
ESC_PA7340AA	11	936
ESC_PA7339AA	11	936
ESC_PA7338AA	1494	180
ESC_PA7335AA	118	985
ESC_PA7334AA	372	424
ESC_PA7333AA	11	936
ESC_PA7332AA	877	935
ESC_PA7331AA	401	142
ESC_PA7330AA	540	286
ESC_PA7328AA	10	512

ESC_PA7327AA	155	175
ESC_PA7326AA	9616	272
ESC_PA7325AA	4197	856
ESC_PA7324AA	162	924
ESC_PA7323AA	398	445
ESC_PA7322AA	1152	850
ESC_PA7320AA	11	936
ESC_PA7319AA	1415	504
ESC_PA7318AA	349	670
ESC_PA7316AA	88	160
ESC_PA7315AA	73	737
ESC_PA7314AA	58	198
ESC_PA7313AA	919	421
ESC_PA7312AA	2425	257
ESC_PA7311AA	8591	592
ESC_PA7310AA	5442	512
ESC_PA7307AA	69	387
ESC_PA7305AA	4197	856
ESC_PA7304AA	206	286
ESC_PA7303AA	4197	856
ESC_PA7302AA	354	15
ESC_PA7301AA	10	512
ESC_PA7300AA	10	512
ESC_PA7299AA	90	156
ESC_PA7298AA	10	512
ESC_PA7297AA	3057	672
ESC_PA7296AA	10640	504
ESC_PA7295AA	1	667
ESC_PA7294AA	641	935
ESC_PA7293AA	48	76
ESC_PA7292AA	90	156
ESC_PA7291AA	761	512
ESC_PA7290AA	3234	257
ESC_PA7289AA	1914	956
ESC_PA7288AA	540	286
ESC_PA7287AA	681	393
ESC_PA7286AA	428	654
ESC_PA7285AA	327	204
ESC_PA7284AA	706	422
ESC_PA7283AA	10	512
ESC_PA7282AA	372	424
ESC_PA7281AA	3889	291
ESC_PA7280AA	10	512
ESC_PA7279AA	10618	592
ESC_PA7278AA	6345	199
ESC_PA7277AA	8817	512
ESC_PA7276AA	10617	76
ESC_PA7275AA	345	175
ESC_PA7274AA	10616	447
ESC_PA7273AA	10615	512

ESC_PA7272AA	744	512
ESC_PA7271AA	1721	311
ESC_PA7270AA	744	512
ESC_PA7269AA	448	257
ESC_PA7268AA	10	512
ESC_PA7267AA	1564	512
ESC_PA7266AA	10	512
ESC_PA7265AA	744	512
ESC_PA7264AA	224	198
ESC_PA7263AA	540	286
ESC_PA7262AA	540	286
ESC_PA7261AA	1114	504
ESC_PA7260AA	2522	198
ESC_PA7259AA	993	789
ESC_PA7258AA	88	160
ESC_PA7257AA	117	592
ESC_PA7256AA	10	512
ESC_PA7255AA	457	595
ESC_PA7254AA	2436	179
ESC_PA7253AA	10	512
ESC_PA7252AA	2325	447
ESC_PA7251AA	167	512
ESC_PA7250AA	744	512
ESC_PA7249AA	617	511
ESC_PA7248AA	448	257
ESC_PA7247AA	10	512
ESC_PA7246AA	744	512
ESC_PA7245AA	44	512
ESC_PA7244AA	744	512
ESC_PA7243AA	44	512
ESC_PA7242AA	410	156
ESC_PA7241AA	69	387
ESC_PA7240AA	23	156
ESC_PA7239AA	219	877
ESC_PA7238AA	410	156
ESC_PA7237AA	58	198
ESC_PA7236AA	88	160
ESC_PA7235AA	10642	592
ESC_PA7234AA	10613	160
ESC_PA7233AA	10639	156
ESC_PA7232AA	761	512
ESC_PA7231AA	23	156
ESC_PA7230AA	1968	76
ESC_PA7229AA	245	261
ESC_PA7228AA	145	256
ESC_PA7227AA	152	257
ESC_PA7226AA	152	257
ESC_PA7225AA	152	257
ESC_PA7224AA	152	257
ESC_PA7223AA	152	257

ESC_PA7222AA	152	257
ESC_PA7221AA	152	257
ESC_PA7220AA	152	257
ESC_PA7219AA	152	257
ESC_PA7218AA	152	257
ESC_PA7217AA	152	257
ESC_PA7216AA	152	257
ESC_PA7215AA	152	257
ESC_PA7214AA	152	257
ESC_PA7213AA	152	257
ESC_PA7212AA	152	257
ESC_PA7211AA	152	257
ESC_PA7210AA	152	257
ESC_PA7209AA	152	257
ESC_PA7208AA	152	257
ESC_PA7207AA	152	257
ESC_PA7206AA	152	257
ESC_PA7205AA	152	257
ESC_PA7204AA	152	257
ESC_PA7203AA	152	257
ESC_PA7202AA	152	257
ESC_PA7201AA	152	257
ESC_PA7200AA	152	257
ESC_PA7199AA	152	257
ESC_PA7198AA	152	257
ESC_PA7197AA	152	257
ESC_PA7196AA	152	257
ESC_PA7195AA	152	257
ESC_PA7194AA	152	257
ESC_PA7193AA	152	257
ESC_PA7192AA	152	257
ESC_PA7191AA	152	257
ESC_PA7190AA	152	257
ESC_PA7189AA	152	257
ESC_PA7188AA	152	257
ESC_PA7187AA	152	257
ESC_PA7186AA	152	257
ESC_PA7185AA	152	257
ESC_PA7184AA	152	257
ESC_PA7183AA	152	257
ESC_PA7182AA	152	257
ESC_PA7181AA	152	257
ESC_PA7180AA	152	257
ESC_PA7179AA	152	257
ESC_PA7178AA	152	257
ESC_PA7177AA	152	257
ESC_PA7176AA	152	257
ESC_PA7175AA	152	257
ESC_PA7174AA	152	257
ESC_PA7173AA	152	257

ESC_PA7172AA	152	257
ESC_PA7171AA	152	257
ESC_PA7170AA	152	257
ESC_PA7169AA	152	257
ESC_PA7168AA	152	257
ESC_PA7167AA	152	257
ESC_PA7166AA	152	257
ESC_PA7165AA	152	257
ESC_PA7164AA	152	257
ESC_PA7163AA	152	257
ESC_PA7162AA	152	257
ESC_PA7161AA	152	257
ESC_PA7160AA	152	257
ESC_PA7159AA	152	257
ESC_PA7158AA	152	257
ESC_PA7157AA	152	257
ESC_PA7156AA	152	257
ESC_PA7155AA	152	257
ESC_PA7154AA	152	257
ESC_PA7153AA	152	257
ESC_PA7152AA	152	257
ESC_PA7151AA	152	257
ESC_PA7150AA	152	257
ESC_PA7149AA	152	257
ESC_PA7148AA	152	257
ESC_PA7147AA	152	257
ESC_PA7146AA	152	257
ESC_PA7145AA	152	257
ESC_PA7144AA	152	257
ESC_PA7143AA	152	257
ESC_PA7142AA	152	257
ESC_PA7141AA	152	257
ESC_PA7140AA	152	257
ESC_PA7139AA	152	257
ESC_PA7138AA	152	257
ESC_PA7137AA	152	257
ESC_PA7136AA	152	257
ESC_PA7135AA	152	257
ESC_PA7134AA	152	257
ESC_PA7133AA	152	257
ESC_PA7132AA	152	257
ESC_PA7131AA	152	257
ESC_PA7130AA	152	257
ESC_PA7129AA	152	257
ESC_PA7128AA	152	257
ESC_PA7127AA	152	257
ESC_PA7126AA	152	257
ESC_PA7125AA	152	257
ESC_PA7124AA	152	257
ESC_PA7123AA	152	257

ESC_PA7122AA	152	257
ESC_PA7121AA	152	257
ESC_PA7120AA	152	257
ESC_PA7119AA	152	257
ESC_PA7118AA	152	257
ESC_PA7117AA	152	257
ESC_PA7116AA	152	257
ESC_PA7115AA	152	257
ESC_PA7114AA	152	257
ESC_PA7113AA	152	257
ESC_PA7112AA	152	257
ESC_PA7111AA	152	257
ESC_PA7110AA	152	257
ESC_PA7109AA	152	257
ESC_PA7108AA	152	257
ESC_PA7107AA	152	257
ESC_PA7106AA	152	257
ESC_PA7105AA	152	257
ESC_PA7104AA	152	257
ESC_PA7103AA	152	257
ESC_PA7102AA	152	257
ESC_PA7101AA	152	257
ESC_PA7100AA	152	257
ESC_PA7099AA	152	257
ESC_PA7098AA	152	257
ESC_PA7097AA	152	257
ESC_PA7096AA	152	257
ESC_PA7095AA	152	257
ESC_PA7094AA	152	257
ESC_PA7093AA	152	257
ESC_PA7092AA	152	257
ESC_PA7091AA	152	257
ESC_PA7090AA	152	257
ESC_PA7089AA	152	257
ESC_PA7088AA	152	257
ESC_PA7087AA	152	257
ESC_PA7086AA	152	257
ESC_PA7085AA	152	257
ESC_PA7084AA	152	257
ESC_PA7083AA	152	257
ESC_PA7082AA	152	257
ESC_PA7081AA	152	257
ESC_PA7080AA	152	257
ESC_PA7079AA	152	257
ESC_PA7078AA	152	257
ESC_PA7077AA	152	257
ESC_PA7076AA	152	257
ESC_PA7075AA	152	257
ESC_PA7074AA	152	257
ESC_PA7073AA	152	257

ESC_PA7072AA	152	257
ESC_PA7071AA	152	257
ESC_PA7070AA	152	257
ESC_PA7069AA	152	257
ESC_PA7068AA	152	257
ESC_PA7067AA	152	257
ESC_PA7066AA	152	257
ESC_PA7065AA	152	257
ESC_PA7064AA	152	257
ESC_PA7063AA	152	257
ESC_PA7062AA	152	257
ESC_PA7061AA	152	257
ESC_PA7060AA	152	257
ESC_PA7059AA	152	257
ESC_PA7058AA	152	257
ESC_PA7057AA	152	257
ESC_PA7056AA	152	257
ESC_PA7055AA	152	257
ESC_PA7054AA	152	257
ESC_PA7053AA	152	257
ESC_PA7052AA	152	257
ESC_PA7051AA	152	257
ESC_PA7050AA	152	257
ESC_PA7049AA	152	257
ESC_PA7048AA	152	257
ESC_PA7047AA	152	257
ESC_PA7046AA	152	257
ESC_PA7045AA	152	257
ESC_PA7044AA	152	257
ESC_PA7043AA	152	257
ESC_PA7042AA	152	257
ESC_PA7041AA	152	257
ESC_PA7040AA	152	257
ESC_PA7039AA	152	257
ESC_PA7038AA	152	257
ESC_PA7037AA	152	257
ESC_PA7036AA	152	257
ESC_PA7035AA	152	257
ESC_PA7034AA	152	257
ESC_PA7033AA	152	257
ESC_PA7032AA	152	257
ESC_PA7031AA	152	257
ESC_PA7030AA	152	257
ESC_PA7029AA	152	257
ESC_PA7028AA	152	257
ESC_PA7027AA	152	257
ESC_PA7026AA	152	257
ESC_PA7025AA	152	257
ESC_PA7024AA	152	257
ESC_PA7023AA	152	257

ESC_PA7022AA	152	257
ESC_PA7021AA	152	257
ESC_PA7020AA	152	257
ESC_PA7019AA	152	257
ESC_PA7018AA	152	257
ESC_PA7017AA	152	257
ESC_PA7016AA	152	257
ESC_PA7015AA	152	257
ESC_PA7014AA	152	257
ESC_PA7013AA	152	257
ESC_PA7012AA	152	257
ESC_PA7011AA	152	257
ESC_PA7010AA	152	257
ESC_PA7009AA	152	257
ESC_PA7008AA	152	257
ESC_PA7007AA	152	257
ESC_PA7006AA	152	257
ESC_PA7005AA	152	257
ESC_PA7004AA	152	257
ESC_PA7003AA	152	257
ESC_PA7002AA	152	257
ESC_PA7001AA	152	257
ESC_PA7000AA	152	257
ESC_PA6999AA	152	257
ESC_PA6998AA	152	257
ESC_PA6997AA	152	257
ESC_PA6996AA	152	257
ESC_PA6995AA	152	257
ESC_PA6994AA	152	257
ESC_PA6993AA	152	257
ESC_PA6992AA	152	257
ESC_PA6991AA	152	257
ESC_PA6990AA	152	257
ESC_PA6989AA	152	257
ESC_PA6988AA	152	257
ESC_PA6987AA	152	257
ESC_PA6986AA	152	257
ESC_PA6985AA	152	257
ESC_PA6984AA	152	257
ESC_PA6983AA	152	257
ESC_PA6982AA	152	257
ESC_PA6981AA	152	257
ESC_PA6980AA	152	257
ESC_PA6979AA	152	257
ESC_PA6978AA	152	257
ESC_PA6977AA	152	257
ESC_PA6976AA	152	257
ESC_PA6975AA	152	257
ESC_PA6974AA	152	257
ESC_PA6973AA	152	257

ESC_PA6972AA	152	257
ESC_PA6971AA	152	257
ESC_PA6970AA	152	257
ESC_PA6969AA	152	257
ESC_PA6968AA	152	257
ESC_PA6967AA	152	257
ESC_PA6966AA	152	257
ESC_PA6965AA	152	257
ESC_PA6964AA	152	257
ESC_PA6963AA	152	257
ESC_PA6962AA	152	257
ESC_PA6961AA	152	257
ESC_PA6960AA	152	257
ESC_PA6959AA	152	257
ESC_PA6958AA	152	257
ESC_PA6957AA	152	257
ESC_PA6956AA	152	257
ESC_PA6955AA	152	257
ESC_PA6954AA	152	257
ESC_PA6953AA	152	257
ESC_PA6952AA	152	257
ESC_PA6951AA	152	257
ESC_PA6950AA	152	257
ESC_PA6949AA	152	257
ESC_PA6948AA	152	257
ESC_PA6947AA	1415	504
ESC_PA6945AA	5260	331
ESC_PA6938AA	10	512
ESC_PA6936AA	10	512
ESC_PA6934AA	10	512
ESC_PA6933AA	10	512
ESC_PA6932AA	10	512
ESC_PA6931AA	10	512
ESC_PA6930AA	10	512
ESC_PA6929AA	10	512
ESC_PA6928AA	10	512
ESC_PA6927AA	10	512
ESC_PA6926AA	10	512
ESC_PA6925AA	10	512
ESC_PA6924AA	10	512
ESC_PA6923AA	10	512
ESC_PA6922AA	10	512
ESC_PA6921AA	10	512
ESC_PA6920AA	10	512
ESC_PA6919AA	10	512
ESC_PA6918AA	10	512
ESC_PA6916AA	10	512
ESC_PA6915AA	10	512
ESC_PA6914AA	10	512
ESC_PA6913AA	10	512

ESC_PA6912AA	10	512
ESC_PA6911AA	10	512
ESC_PA6910AA	10	512
ESC_PA6909AA	10	512
ESC_PA6908AA	10	512
ESC_PA6906AA	10	512
ESC_PA6905AA	10	512
ESC_PA6904AA	10	512
ESC_PA6903AA	10	512
ESC_PA6902AA	10	512
ESC_PA6901AA	10	512
ESC_PA6899AA	10	512
ESC_PA6898AA	10	512
ESC_PA6896AA	10	512
ESC_PA6895AA	10	512
ESC_PA6893AA	10	512
ESC_PA6892AA	10	512
ESC_PA6891AA	10	512
ESC_PA6890AA	10	512
ESC_PA6889AA	10	512
ESC_PA6888AA	10	512
ESC_PA6887AA	10	512
ESC_PA6886AA	10	512
ESC_PA6885AA	10	512
ESC_PA6883AA	10	512
ESC_PA6882AA	10	512
ESC_PA6881AA	10	512
ESC_PA6880AA	10	512
ESC_PA6877AA	10	512
ESC_PA6875AA	10	512
ESC_PA6873AA	10	512
ESC_PA6872AA	10	512
ESC_PA6871AA	10	512
ESC_PA6868AA	405	923
ESC_PA6849AA	73	737
ESC_PA6848AA	73	737
ESC_PA6847AA	73	737
ESC_PA6846AA	73	737
ESC_PA6845AA	73	737
ESC_PA6844AA	10	512
ESC_PA6843AA	1463	263
ESC_PA6842AA	1140	934
ESC_PA6841AA	93	71
ESC_PA6840AA	602	257
ESC_PA6838AA	69	387
ESC_PA6837AA	115	669
ESC_PA6836AA	540	286
ESC_PA6835AA	69	387
ESC_PA6832AA	1140	934
ESC_PA6831AA	1914	956

ESC_PA6830AA	93	71
ESC_PA6829AA	345	175
ESC_PA6828AA	162	924
ESC_PA6826AA	10	512
ESC_PA6825AA	295	160
ESC_PA6824AA	295	160
ESC_PA6823AA	359	696
ESC_PA6822AA	295	160
ESC_PA6821AA	295	160
ESC_PA6820AA	1140	934
ESC_PA6818AA	10	512
ESC_PA6817AA	10619	198
ESC_PA6816AA	295	160
ESC_PA6815AA	1434	512
ESC_PA6814AA	295	160
ESC_PA6811AA	295	160
ESC_PA6810AA	88	160
ESC_PA6808AA	23	156
ESC_PA6807AA	602	257
ESC_PA6805AA	295	160
ESC_PA6804AA	453	935
ESC_PA6803AA	1594	71
ESC_PA6802AA	1140	934
ESC_PA6801AA	533	198
ESC_PA6796AA	1914	956
ESC_PA6795AA	3858	935
ESC_PA6794AA	1594	71
ESC_PA6793AA	189	504
ESC_PA6792AA	2171	164
ESC_PA6791AA	3580	257
ESC_PA6790AA	156	175
ESC_PA6789AA	69	387
ESC_PA6788AA	69	387
ESC_PA6787AA	744	512
ESC_PA6785AA	189	504
ESC_PA6784AA	295	160
ESC_PA6783AA	57	50
ESC_PA6782AA	10	512
ESC_PA6781AA	10	512
ESC_PA6780AA	295	160
ESC_PA6779AA	398	445
ESC_PA6777AA	156	175
ESC_PA6776AA	453	935
ESC_PA6775AA	295	160
ESC_PA6774AA	189	504
ESC_PA6773AA	295	160
ESC_PA6772AA	398	445
ESC_PA6771AA	398	445
ESC_PA6769AA	23	156
ESC_PA6768AA	744	512

ESC_PA6766AA	115	669
ESC_PA6765AA	115	669
ESC_PA6764AA	295	160
ESC_PA6763AA	398	445
ESC_PA6762AA	58	198
ESC_PA6761AA	295	160
ESC_PA6760AA	359	696
ESC_PA6759AA	10621	286
ESC_PA6758AA	10	512
ESC_PA6757AA	295	160
ESC_PA6756AA	2171	164
ESC_PA6755AA	58	198
ESC_PA6754AA	48	76
ESC_PA6753AA	10	512
ESC_PA6752AA	641	935
ESC_PA6751AA	206	286
ESC_PA6750AA	602	257
ESC_PA6749AA	295	160
ESC_PA6748AA	88	160
ESC_PA6747AA	10	512
ESC_PA6746AA	1011	198
ESC_PA6745AA	453	935
ESC_PA6744AA	1140	934
ESC_PA6743AA	1140	934
ESC_PA6742AA	1011	198
ESC_PA6741AA	3580	257
ESC_PA6740AA	10	512
ESC_PA6739AA	10620	62
ESC_PA6738AA	1914	956
ESC_PA6737AA	1914	956
ESC_PA6736AA	162	924
ESC_PA6735AA	88	160
ESC_PA6734AA	1140	934
ESC_PA6733AA	3270	76
ESC_PA6712AA	69	387
ESC_PA6711AA	155	175
ESC_PA6710AA	195	76
ESC_PA6709AA	770	587
ESC_PA6708AA	409	504
ESC_PA6707AA	720	900
ESC_PA6706AA	1656	175
ESC_PA6705AA	602	257
ESC_PA6704AA	117	592
ESC_PA6703AA	8591	592
ESC_PA6702AA	345	175
ESC_PA6701AA	1147	198
ESC_PA6700AA	388	156
ESC_PA6699AA	34	504
ESC_PA6698AA	10638	544
ESC_PA6697AA	5765	234

ESC_PA6696AA	117	592
ESC_PA6695AA	117	592
ESC_PA6694AA	69	387
ESC_PA6693AA	48	76
ESC_PA6692AA	136	392
ESC_PA6691AA	3202	71
ESC_PA6690AA	67	257
ESC_PA6689AA	1607	844
ESC_PA6688AA	8147	850
ESC_PA6687AA	609	447
ESC_PA6686AA	706	422
ESC_PA6685AA	681	393
ESC_PA6684AA	401	142
ESC_PA6683AA	669	76
ESC_PA6682AA	101	696
ESC_PA6681AA	602	257
ESC_PA6680AA	1611	198
ESC_PA6679AA	752	512
ESC_PA6678AA	428	654
ESC_PA6677AA	101	696
ESC_PA6676AA	117	592
ESC_PA6675AA	10	512
ESC_PA6674AA	58	198
ESC_PA6673AA	973	968
ESC_PA6672AA	58	198
ESC_PA6671AA	10622	355
ESC_PA6670AA	443	257
ESC_PA6669AA	1665	601
ESC_PA6668AA	88	160
ESC_PA6667AA	10	512
ESC_PA6666AA	278	924
ESC_PA6665AA	399	198
ESC_PA6664AA	196	257
ESC_PA6663AA	162	924
ESC_PA6662AA	34	504
ESC_PA6661AA	154	257
ESC_PA6660AA	652	204
ESC_PA6659AA	3580	257
ESC_PA6658AA	648	359
ESC_PA6657AA	369	156
ESC_PA6656AA	9293	257
ESC_PA6655AA	58	198
ESC_PA6654AA	10	512
ESC_PA6653AA	1307	257
ESC_PA6652AA	2491	512
ESC_PA6651AA	216	512
ESC_PA6650AA	10632	935
ESC_PA6649AA	1434	512
ESC_PA6648AA	491	626
ESC_PA6647AA	10	512

ESC_PA6646AA	95	738
ESC_PA6645AA	297	234
ESC_PA6644AA	124	254
ESC_PA6643AA	399	198
ESC_PA6642AA	88	160
ESC_PA6641AA	541	701
ESC_PA6640AA	602	257
ESC_PA6639AA	117	592
ESC_PA6638AA	117	592
ESC_PA6637AA	345	175
ESC_PA6636AA	10	512
ESC_PA6635AA	10624	386
ESC_PA6634AA	117	592
ESC_PA6633AA	23	156
ESC_PA6632AA	349	670
ESC_PA6631AA	48	76
ESC_PA6630AA	10623	878
ESC_PA6629AA	336	878
ESC_PA6628AA	109	257
ESC_PA6627AA	88	160
ESC_PA6626AA	695	519
ESC_PA6625AA	155	175
ESC_PA6624AA	58	198
ESC_PA6623AA	7324	257
ESC_PA6622AA	23	156
ESC_PA6621AA	681	393
ESC_PA6620AA	69	387
ESC_PA6619AA	642	924
ESC_PA6618AA	348	175
ESC_PA6617AA	155	175
ESC_PA6616AA	101	696
ESC_PA6615AA	540	286
ESC_PA6614AA	196	257
ESC_PA6613AA	4663	257
ESC_PA6612AA	648	359
ESC_PA6611AA	23	156
ESC_PA6610AA	3580	257
ESC_PA6609AA	2040	198
ESC_PA6608AA	428	654
ESC_PA6607AA	101	696
ESC_PA6606AA	131	653
ESC_PA6605AA	117	592
ESC_PA6604AA	201	257
ESC_PA6603AA	117	592
ESC_PA6602AA	906	198
ESC_PA6601AA	8556	257
ESC_PA6600AA	295	160
ESC_PA6599AA	201	257
ESC_PA6598AA	345	175
ESC_PA6597AA	648	359

ESC_PA6596AA	1137	512
ESC_PA6595AA	5784	448
ESC_PA6594AA	58	198
ESC_PA6593AA	10626	512
ESC_PA6592AA	8302	52
ESC_PA6591AA	12	626
ESC_PA6590AA	362	234
ESC_PA6589AA	457	595
ESC_PA6588AA	10625	160
ESC_PA6587AA	7233	814
ESC_PA6586AA	1594	71
ESC_PA6585AA	278	924
ESC_PA6584AA	58	198
ESC_PA6583AA	2079	198
ESC_PA6582AA	2387	261
ESC_PA6581AA	607	355
ESC_PA6580AA	453	935
ESC_PA6579AA	101	696
ESC_PA6578AA	117	592
ESC_PA6577AA	602	257
ESC_PA6576AA	88	160
ESC_PA6575AA	109	257
ESC_PA6574AA	10	512
ESC_PA6573AA	10	512
ESC_PA6572AA	399	198
ESC_PA6571AA	401	142
ESC_PA6570AA	399	198
ESC_PA6569AA	2077	198
ESC_PA6568AA	101	696
ESC_PA6567AA	278	924
ESC_PA6566AA	429	654
ESC_PA6565AA	162	924
ESC_PA6564AA	69	387
ESC_PA6563AA	117	592
ESC_PA6562AA	10	512
ESC_PA6561AA	23	156
ESC_PA6560AA	428	654
ESC_PA6559AA	3580	257
ESC_PA6558AA	131	653
ESC_PA6557AA	117	592
ESC_PA6556AA	401	142
ESC_PA6555AA	428	654
ESC_PA6554AA	501	900
ESC_PA6553AA	58	198
ESC_PA6552AA	5956	71
ESC_PA6551AA	607	355
ESC_PA6550AA	34	504
ESC_PA6549AA	101	696
ESC_PA6548AA	10	512
ESC_PA6547AA	906	198

ESC_PA6546AA	1611	198
ESC_PA6545AA	10627	519
ESC_PA6544AA	58	198
ESC_PA6543AA	448	257
ESC_PA6542AA	398	445
ESC_PA6541AA	131	653
ESC_PA6540AA	373	71
ESC_PA6539AA	355	764
ESC_PA6538AA	1316	471
ESC_PA6537AA	58	198
ESC_PA6536AA	8140	878
ESC_PA6535AA	117	592
ESC_PA6534AA	3107	512
ESC_PA6533AA	155	175
ESC_PA6532AA	9584	257
ESC_PA6531AA	155	175
ESC_PA6530AA	1851	380
ESC_PA6529AA	69	387
ESC_PA6528AA	10628	474
ESC_PA6527AA	616	156
ESC_PA6526AA	2040	198
ESC_PA6525AA	973	968
ESC_PA6524AA	978	740
ESC_PA6523AA	58	198
ESC_PA6522AA	1236	380
ESC_PA6521AA	1326	198
ESC_PA6520AA	1434	512
ESC_PA6519AA	155	175
ESC_PA6518AA	6707	445
ESC_PA6517AA	6707	445
ESC_PA6516AA	3580	257
ESC_PA6515AA	8951	943
ESC_PA6514AA	978	740
ESC_PA6513AA	2073	198
ESC_PA6512AA	46	447
ESC_PA6511AA	602	257
ESC_PA6510AA	1594	71
ESC_PA6509AA	58	198
ESC_PA6508AA	278	924
ESC_PA6507AA	657	592
ESC_PA6506AA	3580	257
ESC_PA6505AA	10	512
ESC_PA6504AA	278	924
ESC_PA6503AA	197	601
ESC_PA6502AA	224	198
ESC_PA6501AA	101	696
ESC_PA6500AA	2065	512
ESC_PA6499AA	6617	519
ESC_PA6498AA	117	592
ESC_PA6497AA	1508	261

ESC_PA6496AA	115	669
ESC_PA6495AA	3580	257
ESC_PA6494AA	6949	765
ESC_PA6493AA	58	198
ESC_PA6492AA	2556	630
ESC_PA6491AA	23	156
ESC_PA6490AA	117	592
ESC_PA6489AA	1727	257
ESC_PA6488AA	1158	387
ESC_PA6487AA	2556	630
ESC_PA6486AA	1611	198
ESC_PA6485AA	69	387
ESC_PA6484AA	4293	212
ESC_PA6483AA	216	512
ESC_PA6482AA	399	198
ESC_PA6481AA	10631	592
ESC_PA6480AA	93	71
ESC_PA6479AA	355	764
ESC_PA6478AA	10	512
ESC_PA6477AA	58	198
ESC_PA6476AA	10	512
ESC_PA6475AA	101	696
ESC_PA6474AA	1429	198
ESC_PA6473AA	8602	567
ESC_PA6472AA	216	512
ESC_PA6471AA	216	512
ESC_PA6470AA	10	512
ESC_PA6469AA	10	512
ESC_PA6468AA	117	592
ESC_PA6467AA	10630	198
ESC_PA6466AA	7804	369
ESC_PA6465AA	1433	601
ESC_PA6464AA	6002	198
ESC_PA6462AA	8588	257
ESC_PA6461AA	1434	512
ESC_PA6460AA	10629	175
ESC_PA6459AA	1704	257
ESC_PA6458AA	3580	257
ESC_PA6457AA	349	670
ESC_PA6456AA	23	156
ESC_PA6455AA	155	175
ESC_PA6454AA	399	198
ESC_PA6453AA	607	355
ESC_PA6452AA	2171	164
ESC_PA6451AA	69	387
ESC_PA6450AA	187	257
ESC_PA6449AA	10	512
ESC_PA6448AA	57	50
ESC_PA6447AA	362	234
ESC_PA6446AA	10633	587

ESC_PA6445AA	155	175
ESC_PA6444AA	351	268
ESC_PA6443AA	131	653
ESC_PA6442AA	5451	912
ESC_PA6441AA	154	257
ESC_PA6440AA	354	15
ESC_PA6439AA	8591	592
ESC_PA6438AA	117	592
ESC_PA6437AA	2175	212
ESC_PA6436AA	58	198
ESC_PA6435AA	1072	355
ESC_PA6434AA	369	156
ESC_PA6433AA	10	512
ESC_PA6432AA	10	512
ESC_PA6431AA	86	935
ESC_PA6430AA	154	257
ESC_PA6429AA	10	512
ESC_PA6428AA	973	968
ESC_PA6427AA	616	156
ESC_PA6426AA	1844	198
ESC_PA6425AA	131	653
ESC_PA6424AA	10634	257
ESC_PA6423AA	443	257
ESC_PA6422AA	1665	601
ESC_PA6421AA	117	592
ESC_PA6420AA	1125	198
ESC_PA6419AA	429	654
ESC_PA6418AA	69	387
ESC_PA6417AA	428	654
ESC_PA6416AA	162	924
ESC_PA6415AA	457	595
ESC_PA6414AA	10637	50
ESC_PA6413AA	101	696
ESC_PA6412AA	3258	592
ESC_PA6411AA	58	198
ESC_PA6410AA	351	268
ESC_PA6409AA	540	286
ESC_PA6408AA	10636	198
ESC_PA6407AA	117	592
ESC_PA6406AA	46	447
ESC_PA6405AA	10	512
ESC_PA6404AA	1684	504
ESC_PA6403AA	124	254
ESC_PA6402AA	10635	653
ESC_PA6401AA	3594	924
ESC_PA6400AA	10	512
ESC_PA6399AA	90	156
ESC_PA6398AA	90	156
ESC_PA6397AA	90	156
ESC_PA6396AA	10	512

ESC_PA6395AA	90	156
ESC_PA6394AA	453	935
ESC_PA6393AA	90	156
ESC_PA6392AA	90	156
ESC_PA6391AA	90	156
ESC_PA6390AA	410	156
ESC_PA6389AA	3524	935
ESC_PA6388AA	100	504
ESC_PA6387AA	2521	257
ESC_PA6386AA	90	156
ESC_PA6385AA	453	935
ESC_PA6384AA	29	156
ESC_PA6383AA	131	653
ESC_PA6381AA	20	198
ESC_PA6380AA	141	644
ESC_PA6379AA	7815	257
ESC_PA6378AA	129	257
ESC_PA6377AA	355	764
ESC_PA6376AA	11	936
ESC_PA6375AA	297	234
ESC_PA6374AA	12	626
ESC_PA6373AA	410	156
ESC_PA6372AA	11	936
ESC_PA6371AA	278	924
ESC_PA6370AA	129	257
ESC_PA6369AA	95	738
ESC_PA6368AA	770	587
ESC_PA6367AA	11	936
ESC_PA6366AA	127	738
ESC_PA6365AA	961	623
ESC_PA6361AA	6711	71
ESC_PA6360AA	90	156
ESC_PA6359AA	8496	382
ESC_PA6358AA	10	512
ESC_PA6357AA	10614	926
ESC_PA6356AA	100	504
ESC_PA6355AA	56	198
ESC_PA6354AA	9857	512
ESC_PA6353AA	10	512
ESC_PA6352AA	100	504
ESC_PA6351AA	6711	71
ESC_PA6350AA	100	504
ESC_PA6349AA	100	504
ESC_PA6348AA	90	156
ESC_PA6347AA	12	626
ESC_PA6346AA	929	742
ESC_PA6345AA	12	626
ESC_PA6344AA	58	198
ESC_PA6343AA	127	738
ESC_PA6342AA	245	261

ESC_PA6341AA	245	261
ESC_PA6340AA	245	261
ESC_PA6339AA	245	261
ESC_PA6338AA	152	257
ESC_PA6337AA	152	257
ESC_PA6336AA	152	257
ESC_PA6335AA	245	261
ESC_PA6334AA	245	261
ESC_PA6333AA	630	261
ESC_PA6332AA	152	257
ESC_PA6331AA	245	261
ESC_PA6330AA	152	257
ESC_PA6329AA	1765	102
ESC_PA6328AA	295	160
ESC_PA6327AA	295	160
ESC_PA6326AA	189	504
ESC_PA6325AA	8560	234
ESC_PA6324AA	1178	512
ESC_PA6323AA	155	175
ESC_PA6322AA	10425	263
ESC_PA6321AA	752	512
ESC_PA6320AA	8680	76
ESC_PA6319AA	38	669
ESC_PA6318AA	101	696
ESC_PA6317AA	398	445
ESC_PA6316AA	58	198
ESC_PA6315AA	1704	257
ESC_PA6314AA	10	512
ESC_PA6313AA	154	257
ESC_PA6312AA	189	504
ESC_PA6311AA	398	445
ESC_PA6310AA	4050	512
ESC_PA6309AA	101	696
ESC_PA6308AA	6113	257
ESC_PA6307AA	10	512
ESC_PA6306AA	58	198
ESC_PA6305AA	11	936
ESC_PA6304AA	11	936
ESC_PA6303AA	11	936
ESC_PA6302AA	442	257
ESC_PA6301AA	11	936
ESC_PA6300AA	11	936
ESC_PA6299AA	583	768
ESC_PA6298AA	11	936
ESC_PA6297AA	25	234
ESC_PA6296AA	11	936
ESC_PA6295AA	381	156
ESC_PA6294AA	90	156
ESC_PA6293AA	33	198
ESC_PA6292AA	5601	789

ESC_PA6291AA	648	359
ESC_PA6290AA	11	936
ESC_PA6289AA	738	598
ESC_PA6288AA	206	286
ESC_PA6287AA	10	512
ESC_PA6286AA	11	936
ESC_PA6285AA	6	445
ESC_PA6284AA	300	286
ESC_PA6283AA	10	512
ESC_PA6282AA	11	936
ESC_PA6281AA	11	936
ESC_PA6280AA	6918	260
ESC_PA6279AA	145	256
ESC_PA6278AA	245	261
ESC_PA6277AA	245	261
ESC_PA6276AA	152	257
ESC_PA6275AA	245	261
ESC_PA6274AA	152	257
ESC_PA6273AA	152	257
ESC_PA6272AA	152	257
ESC_PA6271AA	288	102
ESC_PA6270AA	33	198
ESC_PA6269AA	2795	355
ESC_PA6268AA	442	257
ESC_PA6267AA	10	512
ESC_PA6266AA	657	592
ESC_PA6265AA	5601	789
ESC_PA6264AA	7024	886
ESC_PA6263AA	25	234
ESC_PA6262AA	1819	808
ESC_PA6261AA	2836	221
ESC_PA6260AA	2353	76
ESC_PA6259AA	10	512
ESC_PA6258AA	6	445
ESC_PA6257AA	21	156
ESC_PA6256AA	381	156
ESC_PA6255AA	182	246
ESC_PA6254AA	10	512
ESC_PA6253AA	2089	276
ESC_PA6252AA	675	234
ESC_PA6251AA	10612	355
ESC_PA6250AA	270	204
ESC_PA6249AA	270	204
ESC_PA6248AA	270	204
ESC_PA6247AA	270	204
ESC_PA6246AA	6	445
ESC_PA6245AA	6	445
ESC_PA6244AA	6	445
ESC_PA6243AA	6	445
ESC_PA6242AA	6	445

ESC_PA6241AA	6	445
ESC_PA6240AA	280	142
ESC_PA6239AA	280	142
ESC_PA6238AA	280	142
ESC_PA6237AA	280	142
ESC_PA6236AA	280	142
ESC_PA6235AA	280	142
ESC_PA6234AA	270	204
ESC_PA6233AA	963	669
ESC_PA6232AA	2522	198
ESC_PA6231AA	69	387
ESC_PA6230AA	69	387
ESC_PA6229AA	117	592
ESC_PA6228AA	10	512
ESC_PA6227AA	34	504
ESC_PA6226AA	34	504
ESC_PA6225AA	57	50
ESC_PA6224AA	648	359
ESC_PA6223AA	10611	504
ESC_PA6222AA	648	359
ESC_PA6221AA	69	387
ESC_PA6220AA	10610	512
ESC_PA6219AA	648	359
ESC_PA6218AA	617	511
ESC_PA6217AA	617	511
ESC_PA6216AA	399	198
ESC_PA6215AA	10	512
ESC_PA6214AA	8828	669
ESC_PA6213AA	942	198
ESC_PA6211AA	5745	156
ESC_PA6210AA	278	924
ESC_PA6209AA	472	601
ESC_PA6208AA	187	257
ESC_PA6207AA	4628	76
ESC_PA6206AA	327	204
ESC_PA6205AA	410	156
ESC_PA6203AA	399	198
ESC_PA6202AA	10609	311
ESC_PA6201AA	10608	512
ESC_PA6200AA	205	257
ESC_PA6199AA	131	653
ESC_PA6198AA	131	653
ESC_PA6197AA	131	653
ESC_PA6196AA	457	595
ESC_PA6195AA	10607	15
ESC_PA6194AA	131	653
ESC_PA6193AA	131	653
ESC_PA6192AA	131	653
ESC_PA6191AA	131	653
ESC_PA6190AA	131	653

ESC_PA6189AA	131	653
ESC_PA6188AA	131	653
ESC_PA6187AA	131	653
ESC_PA6186AA	131	653
ESC_PA6185AA	131	653
ESC_PA6184AA	416	738
ESC_PA6183AA	10606	653
ESC_PA6182AA	131	653
ESC_PA6181AA	10605	653
ESC_PA6180AA	131	653
ESC_PA6179AA	131	653
ESC_PA6178AA	131	653
ESC_PA6177AA	131	653
ESC_PA6176AA	131	653
ESC_PA6175AA	131	653
ESC_PA6169AA	154	257
ESC_PA6168AA	34	504
ESC_PA6166AA	1081	198
ESC_PA6165AA	154	257
ESC_PA6164AA	1611	198
ESC_PA6162AA	448	257
ESC_PA6161AA	448	257
ESC_PA6160AA	1079	235
ESC_PA6159AA	10604	935
ESC_PA6158AA	1800	924
ESC_PA6157AA	3234	257
ESC_PA6156AA	6949	765
ESC_PA6155AA	10604	935
ESC_PA6154AA	224	198
ESC_PA6153AA	515	1004
ESC_PA6152AA	48	76
ESC_PA6151AA	10	512
ESC_PA6150AA	1121	198
ESC_PA6149AA	10603	512
ESC_PA6148AA	5044	1004
ESC_PA6147AA	69	387
ESC_PA6146AA	1147	198
ESC_PA6145AA	1163	579
ESC_PA6144AA	2170	234
ESC_PA6143AA	1196	257
ESC_PA6142AA	2935	512
ESC_PA6141AA	34	504
ESC_PA6140AA	1722	599
ESC_PA6139AA	278	924
ESC_PA6138AA	38	669
ESC_PA6137AA	167	512
ESC_PA6136AA	351	268
ESC_PA6135AA	345	175
ESC_PA6134AA	7153	263
ESC_PA6133AA	1011	198

ESC_PA6131AA	10602	198
ESC_PA6130AA	484	71
ESC_PA6129AA	4238	512
ESC_PA6128AA	196	257
ESC_PA6127AA	34	504
ESC_PA6126AA	101	696
ESC_PA6125AA	68	386
ESC_PA6124AA	394	400
ESC_PA6123AA	192	696
ESC_PA6119AA	1722	599
ESC_PA6118AA	2040	198
ESC_PA6117AA	152	257
ESC_PA6116AA	10	512
ESC_PA6115AA	9857	512
ESC_PA6114AA	2040	198
ESC_PA6113AA	5949	935
ESC_PA6112AA	974	160
ESC_PA6111AA	165	504
ESC_PA6110AA	6018	52
ESC_PA6109AA	10	512
ESC_PA6108AA	10	512
ESC_PA6107AA	877	935
ESC_PA6106AA	5870	504
ESC_PA6105AA	4956	198
ESC_PA6104AA	5855	519
ESC_PA6068AA	243	197
ESC_PA6067AA	243	197
ESC_PA6066AA	152	257
ESC_PA6065AA	152	257
ESC_PA6064AA	245	261
ESC_PA6063AA	245	261
ESC_PA6062AA	152	257
ESC_PA6061AA	245	261
ESC_PA6060AA	245	261
ESC_PA6059AA	245	261
ESC_PA6058AA	245	261
ESC_PA6057AA	152	257
ESC_PA6056AA	245	261
ESC_PA6055AA	245	261
ESC_PA6054AA	630	261
ESC_PA6053AA	152	257
ESC_PA6052AA	245	261
ESC_PA6051AA	3012	900
ESC_PA6050AA	117	592
ESC_PA6049AA	10601	257
ESC_PA6048AA	216	512
ESC_PA6047AA	10600	311
ESC_PA6046AA	182	246
ESC_PA6045AA	40	198
ESC_PA6044AA	4424	351

ESC_PA6043AA	6274	257
ESC_PA6042AA	11	936
ESC_PA6041AA	11	936
ESC_PA6040AA	17	191
ESC_PA6039AA	327	204
ESC_PA6038AA	152	257
ESC_PA6037AA	245	261
ESC_PA6036AA	245	261
ESC_PA6035AA	152	257
ESC_PA6034AA	152	257
ESC_PA6033AA	245	261
ESC_PA6032AA	245	261
ESC_PA6031AA	152	257
ESC_PA6030AA	152	257
ESC_PA6029AA	152	257
ESC_PA6028AA	245	261
ESC_PA6027AA	152	257
ESC_PA6026AA	245	261
ESC_PA6025AA	245	261
ESC_PA6024AA	245	261
ESC_PA6023AA	245	261
ESC_PA6022AA	152	257
ESC_PA6021AA	245	261
ESC_PA6020AA	245	261
ESC_PA6019AA	245	261
ESC_PA6018AA	278	924
ESC_PA6017AA	165	504
ESC_PA6016AA	154	257
ESC_PA6015AA	302	850
ESC_PA6014AA	6338	664
ESC_PA6013AA	155	175
ESC_PA6012AA	86	935
ESC_PA6011AA	17	191
ESC_PA6010AA	10	512
ESC_PA6009AA	10	512
ESC_PA6008AA	155	175
ESC_PA6007AA	2217	62
ESC_PA6006AA	189	504
ESC_PA6005AA	1308	257
ESC_PA6004AA	10	512
ESC_PA6003AA	1125	198
ESC_PA6002AA	898	108
ESC_PA6001AA	10598	257
ESC_PA6000AA	772	76
ESC_PA5999AA	11	936
ESC_PA5998AA	11	936
ESC_PA5997AA	11	936
ESC_PA5996AA	11	936
ESC_PA5995AA	11	936
ESC_PA5994AA	162	924

ESC_PA5993AA	131	653
ESC_PA5992AA	131	653
ESC_PA5991AA	38	669
ESC_PA5990AA	131	653
ESC_PA5989AA	131	653
ESC_PA5988AA	131	653
ESC_PA5987AA	131	653
ESC_PA5986AA	131	653
ESC_PA5985AA	38	669
ESC_PA5984AA	405	923
ESC_PA5983AA	131	653
ESC_PA5982AA	131	653
ESC_PA5981AA	131	653
ESC_PA5980AA	131	653
ESC_PA5979AA	131	653
ESC_PA5978AA	131	653
ESC_PA5977AA	38	669
ESC_PA5976AA	131	653
ESC_PA5975AA	131	653
ESC_PA5974AA	131	653
ESC_PA5973AA	131	653
ESC_PA5972AA	442	257
ESC_PA5971AA	21	156
ESC_PA5970AA	5292	738
ESC_PA5969AA	6130	268
ESC_PA5968AA	442	257
ESC_PA5967AA	4748	234
ESC_PA5966AA	11	936
ESC_PA5965AA	10	512
ESC_PA5964AA	11	936
ESC_PA5963AA	131	653
ESC_PA5962AA	10	512
ESC_PA5961AA	11	936
ESC_PA5960AA	6	445
ESC_PA5959AA	26	257
ESC_PA5958AA	11	936
ESC_PA5956AA	10	512
ESC_PA5955AA	10	512
ESC_PA5954AA	34	504
ESC_PA5953AA	10559	773
ESC_PA5952AA	25	234
ESC_PA5951AA	33	198
ESC_PA5950AA	34	504
ESC_PA5949AA	301	307
ESC_PA5948AA	10599	738
ESC_PA5947AA	21	156
ESC_PA5946AA	33	198
ESC_PA5945AA	10	512
ESC_PA5944AA	6274	257
ESC_PA5943AA	504	738

ESC_PA5942AA	223	198
ESC_PA5941AA	167	512
ESC_PA5940AA	167	512
ESC_PA5939AA	167	512
ESC_PA5938AA	167	512
ESC_PA5937AA	11	936
ESC_PA5936AA	617	511
ESC_PA5935AA	167	512
ESC_PA5934AA	617	511
ESC_PA5933AA	617	511
ESC_PA5932AA	10	512
ESC_PA5931AA	11	936
ESC_PA5930AA	11	936
ESC_PA5929AA	11	936
ESC_PA5928AA	11	936
ESC_PA5927AA	11	936
ESC_PA5926AA	11	936
ESC_PA5925AA	11	936
ESC_PA5924AA	11	936
ESC_PA5923AA	301	307
ESC_PA5922AA	29	156
ESC_PA5921AA	21	156
ESC_PA5920AA	21	156
ESC_PA5919AA	152	257
ESC_PA5918AA	2179	924
ESC_PA5917AA	156	175
ESC_PA5916AA	48	76
ESC_PA5915AA	5454	286
ESC_PA5914AA	48	76
ESC_PA5913AA	156	175
ESC_PA5912AA	156	175
ESC_PA5911AA	156	175
ESC_PA5910AA	156	175
ESC_PA5909AA	156	175
ESC_PA5908AA	156	175
ESC_PA5907AA	1403	924
ESC_PA5906AA	448	257
ESC_PA5905AA	156	175
ESC_PA5904AA	410	156
ESC_PA5903AA	156	175
ESC_PA5902AA	10	512
ESC_PA5901AA	602	257
ESC_PA5900AA	156	175
ESC_PA5899AA	10596	311
ESC_PA5898AA	137	270
ESC_PA5897AA	25	234
ESC_PA5896AA	655	926
ESC_PA5895AA	655	926
ESC_PA5894AA	335	936
ESC_PA5893AA	33	198

ESC_PA5892AA	655	926
ESC_PA5890AA	10587	63
ESC_PA5889AA	10594	75
ESC_PA5888AA	10573	71
ESC_PA5886AA	10589	198
ESC_PA5883AA	10585	309
ESC_PA5882AA	10582	670
ESC_PA5881AA	10584	1004
ESC_PA5880AA	10583	504
ESC_PA5879AA	10580	71
ESC_PA5878AA	10581	257
ESC_PA5873AA	10571	1004
ESC_PA5872AA	10573	71
ESC_PA5868AA	10569	311
ESC_PA5865AA	10579	789
ESC_PA5864AA	10587	63
ESC_PA5862AA	10566	824
ESC_PA5861AA	10574	481
ESC_PA5858AA	10562	699
ESC_PA5857AA	10561	483
ESC_PA5856AA	10563	71
ESC_PA5853AA	152	257
ESC_PA5852AA	152	257
ESC_PA5851AA	152	257
ESC_PA5850AA	1665	601
ESC_PA5849AA	939	198
ESC_PA5848AA	40	198
ESC_PA5847AA	152	257
ESC_PA5846AA	152	257
ESC_PA5845AA	152	257
ESC_PA5844AA	152	257
ESC_PA5843AA	152	257
ESC_PA5836AA	655	926
ESC_PA5835AA	335	936
ESC_PA5834AA	655	926
ESC_PA5833AA	655	926
ESC_PA5832AA	137	270
ESC_PA5831AA	33	198
ESC_PA5830AA	25	234
ESC_PA5829AA	10552	254
ESC_PA5828AA	90	156
ESC_PA5827AA	10	512
ESC_PA5826AA	100	504
ESC_PA5825AA	761	512
ESC_PA5824AA	10	512
ESC_PA5823AA	90	156
ESC_PA5822AA	8496	382
ESC_PA5821AA	29	156
ESC_PA5820AA	90	156
ESC_PA5819AA	90	156

ESC_PA5818AA	6957	221
ESC_PA5817AA	20	198
ESC_PA5816AA	10559	773
ESC_PA5815AA	381	156
ESC_PA5814AA	34	504
ESC_PA5813AA	21	156
ESC_PA5812AA	17	191
ESC_PA5811AA	746	789
ESC_PA5810AA	10593	156
ESC_PA5809AA	504	738
ESC_PA5808AA	152	257
ESC_PA5807AA	152	257
ESC_PA5806AA	245	261
ESC_PA5805AA	245	261
ESC_PA5804AA	245	261
ESC_PA5803AA	152	257
ESC_PA5802AA	245	261
ESC_PA5801AA	152	257
ESC_PA5800AA	152	257
ESC_PA5798AA	10551	190
ESC_PA5797AA	245	261
ESC_PA5796AA	630	261
ESC_PA5795AA	152	257
ESC_PA5794AA	1	667
ESC_PA5793AA	141	644
ESC_PA5792AA	10	512
ESC_PA5791AA	90	156
ESC_PA5790AA	90	156
ESC_PA5789AA	90	156
ESC_PA5788AA	90	156
ESC_PA5787AA	657	592
ESC_PA5786AA	657	592
ESC_PA5785AA	10548	928
ESC_PA5784AA	200	198
ESC_PA5783AA	300	286
ESC_PA5782AA	738	598
ESC_PA5781AA	442	257
ESC_PA5780AA	21	156
ESC_PA5779AA	40	198
ESC_PA5778AA	11	936
ESC_PA5777AA	17	191
ESC_PA5776AA	10	512
ESC_PA5775AA	11	936
ESC_PA5774AA	10	512
ESC_PA5773AA	677	257
ESC_PA5772AA	381	156
ESC_PA5771AA	11	936
ESC_PA5770AA	5292	738
ESC_PA5769AA	841	204
ESC_PA5768AA	10550	257

ESC_PA5767AA	442	257
ESC_PA5766AA	517	924
ESC_PA5765AA	442	257
ESC_PA5764AA	25	234
ESC_PA5763AA	34	504
ESC_PA5762AA	11	936
ESC_PA5761AA	6060	41
ESC_PA5760AA	3570	156
ESC_PA5759AA	10	512
ESC_PA5758AA	11	936
ESC_PA5757AA	11	936
ESC_PA5756AA	11	936
ESC_PA5755AA	11	936
ESC_PA5754AA	11	936
ESC_PA5753AA	11	936
ESC_PA5747AA	145	256
ESC_PA5746AA	152	257
ESC_PA5745AA	152	257
ESC_PA5744AA	152	257
ESC_PA5743AA	245	261
ESC_PA5742AA	245	261
ESC_PA5741AA	245	261
ESC_PA5740AA	245	261
ESC_PA5739AA	1753	261
ESC_PA5738AA	148	191
ESC_PA5737AA	152	257
ESC_PA5736AA	152	257
ESC_PA5735AA	152	257
ESC_PA5734AA	245	261
ESC_PA5733AA	10545	175
ESC_PA5732AA	278	924
ESC_PA5731AA	2040	198
ESC_PA5730AA	154	257
ESC_PA5729AA	4379	878
ESC_PA5728AA	398	445
ESC_PA5727AA	1463	263
ESC_PA5726AA	10	512
ESC_PA5725AA	10547	257
ESC_PA5724AA	218	519
ESC_PA5723AA	9853	261
ESC_PA5722AA	101	696
ESC_PA5721AA	10546	79
ESC_PA5720AA	2485	212
ESC_PA5719AA	278	924
ESC_PA5718AA	115	669
ESC_PA5717AA	345	175
ESC_PA5716AA	975	512
ESC_PA5715AA	152	257
ESC_PA5714AA	152	257
ESC_PA5713AA	95	738

ESC_PA5712AA	62	574
ESC_PA5711AA	357	654
ESC_PA5710AA	405	923
ESC_PA5709AA	404	761
ESC_PA5708AA	3877	512
ESC_PA5707AA	59	217
ESC_PA5706AA	1193	761
ESC_PA5705AA	10544	574
ESC_PA5704AA	69	387
ESC_PA5703AA	7882	740
ESC_PA5702AA	23	156
ESC_PA5701AA	416	738
ESC_PA5700AA	11	936
ESC_PA5699AA	301	307
ESC_PA5698AA	301	307
ESC_PA5697AA	21	156
ESC_PA5696AA	117	592
ESC_PA5691AA	93	71
ESC_PA5687AA	10328	175
ESC_PA5681AA	354	15
ESC_PA5678AA	10543	0
ESC_PA5670AA	152	257
ESC_PA5669AA	152	257
ESC_PA5668AA	145	256
ESC_PA5667AA	145	256
ESC_PA5666AA	145	256
ESC_PA5665AA	145	256
ESC_PA5664AA	10541	670
ESC_PA5663AA	10542	234
ESC_PA5662AA	10540	880
ESC_PA5661AA	245	261
ESC_PA5660AA	152	257
ESC_PA5659AA	152	257
ESC_PA5658AA	152	257
ESC_PA5657AA	152	257
ESC_PA5656AA	288	102
ESC_PA5655AA	245	261
ESC_PA5654AA	152	257
ESC_PA5653AA	152	257
ESC_PA5652AA	1753	261
ESC_PA5651AA	245	261
ESC_PA5650AA	245	261
ESC_PA5649AA	245	261
ESC_PA5648AA	300	286
ESC_PA5647AA	301	307
ESC_PA5646AA	21	156
ESC_PA5642AA	152	257
ESC_PA5641AA	2040	198
ESC_PA5640AA	167	512
ESC_PA5639AA	167	512

ESC_PA5638AA	38	669
ESC_PA5637AA	167	512
ESC_PA5636AA	167	512
ESC_PA5635AA	2253	71
ESC_PA5634AA	167	512
ESC_PA5633AA	167	512
ESC_PA5632AA	167	512
ESC_PA5631AA	617	511
ESC_PA5630AA	155	175
ESC_PA5629AA	617	511
ESC_PA5628AA	167	512
ESC_PA5627AA	167	512
ESC_PA5626AA	167	512
ESC_PA5625AA	167	512
ESC_PA5624AA	167	512
ESC_PA5623AA	162	924
ESC_PA5622AA	16	156
ESC_PA5534AA	10539	508
ESC_PA5469AA	10	512
ESC_PA5468AA	10	512
ESC_PA5467AA	10	512
ESC_PA5429AA	10329	15
ESC_PA5427AA	131	653
ESC_PA5421AA	131	653
ESC_PA5420AA	58	198
ESC_PA5419AA	540	286
ESC_PA5418AA	38	669
ESC_PA5417AA	155	175
ESC_PA5416AA	34	504
ESC_PA5415AA	1114	504
ESC_PA5414AA	641	935
ESC_PA5413AA	58	198
ESC_PA5412AA	58	198
ESC_PA5411AA	38	669
ESC_PA5410AA	187	257
ESC_PA5409AA	58	198
ESC_PA5408AA	4138	198
ESC_PA5407AA	101	696
ESC_PA5406AA	10	512
ESC_PA5405AA	641	935
ESC_PA5404AA	641	935
ESC_PA5403AA	542	824
ESC_PA5402AA	58	198
ESC_PA5401AA	8610	257
ESC_PA5400AA	16	156
ESC_PA5399AA	11	936
ESC_PA5398AA	3574	257
ESC_PA5396AA	38	669
ESC_PA5391AA	38	669
ESC_PA5384AA	148	191

ESC_PA5383AA	148	191
ESC_PA5382AA	148	191
ESC_PA5381AA	200	198
ESC_PA5380AA	11	936
ESC_PA5379AA	2966	936
ESC_PA5378AA	21	156
ESC_PA5377AA	11	936
ESC_PA5376AA	11	936
ESC_PA5375AA	11	936
ESC_PA5374AA	29	156
ESC_PA5373AA	101	696
ESC_PA5372AA	5903	180
ESC_PA5371AA	4550	150
ESC_PA5370AA	1252	567
ESC_PA5369AA	641	935
ESC_PA5368AA	58	198
ESC_PA5367AA	1115	263
ESC_PA5366AA	38	669
ESC_PA5365AA	58	198
ESC_PA5364AA	6732	1004
ESC_PA5363AA	1201	512
ESC_PA5362AA	1079	235
ESC_PA5361AA	162	924
ESC_PA5360AA	10	512
ESC_PA5359AA	752	512
ESC_PA5358AA	58	198
ESC_PA5357AA	164	175
ESC_PA5356AA	58	198
ESC_PA5355AA	1611	198
ESC_PA5354AA	9237	938
ESC_PA5353AA	70	670
ESC_PA5352AA	10537	842
ESC_PA5351AA	10536	415
ESC_PA5350AA	720	900
ESC_PA5349AA	428	654
ESC_PA5348AA	603	198
ESC_PA5347AA	7900	430
ESC_PA5346AA	919	421
ESC_PA5345AA	919	421
ESC_PA5344AA	1248	156
ESC_PA5343AA	2178	924
ESC_PA5342AA	10538	900
ESC_PA5341AA	443	257
ESC_PA5340AA	442	257
ESC_PA5339AA	10337	448
ESC_PA5338AA	10064	711
ESC_PA5337AA	10064	711
ESC_PA5335AA	1193	761
ESC_PA5334AA	131	653
ESC_PA5333AA	720	900

ESC_PA5332AA	131	653
ESC_PA5331AA	131	653
ESC_PA5330AA	7508	198
ESC_PA5329AA	405	923
ESC_PA5328AA	148	191
ESC_PA5327AA	148	191
ESC_PA5326AA	148	191
ESC_PA5325AA	148	191
ESC_PA5324AA	245	261
ESC_PA5323AA	245	261
ESC_PA5322AA	152	257
ESC_PA5321AA	10524	261
ESC_PA5320AA	245	261
ESC_PA5319AA	10523	261
ESC_PA5318AA	245	261
ESC_PA5317AA	152	257
ESC_PA5316AA	245	261
ESC_PA5315AA	245	261
ESC_PA5314AA	245	261
ESC_PA5313AA	1025	261
ESC_PA5312AA	152	257
ESC_PA5311AA	152	257
ESC_PA5310AA	152	257
ESC_PA5309AA	245	261
ESC_PA5308AA	152	257
ESC_PA5307AA	245	261
ESC_PA5306AA	152	257
ESC_PA5305AA	245	261
ESC_PA5304AA	245	261
ESC_PA5303AA	245	261
ESC_PA5302AA	152	257
ESC_PA5301AA	152	257
ESC_PA5300AA	245	261
ESC_PA5299AA	245	261
ESC_PA5298AA	245	261
ESC_PA5296AA	245	261
ESC_PA5295AA	148	191
ESC_PA5294AA	245	261
ESC_PA5293AA	245	261
ESC_PA5292AA	311	204
ESC_PA5290AA	245	261
ESC_PA5289AA	288	102
ESC_PA5288AA	245	261
ESC_PA5287AA	148	191
ESC_PA5286AA	152	257
ESC_PA5285AA	245	261
ESC_PA5284AA	152	257
ESC_PA5283AA	245	261
ESC_PA5282AA	245	261
ESC_PA5281AA	245	261

ESC_PA5280AA	245	261
ESC_PA5279AA	152	257
ESC_PA5277AA	148	191
ESC_PA5276AA	152	257
ESC_PA5275AA	1273	256
ESC_PA5274AA	2079	198
ESC_PA5273AA	90	156
ESC_PA5272AA	155	175
ESC_PA5271AA	428	654
ESC_PA5270AA	155	175
ESC_PA5269AA	10	512
ESC_PA5268AA	540	286
ESC_PA5267AA	681	393
ESC_PA5266AA	209	512
ESC_PA5265AA	4537	76
ESC_PA5264AA	58	198
ESC_PA5263AA	162	924
ESC_PA5262AA	144	740
ESC_PA5261AA	1434	512
ESC_PA5260AA	1434	512
ESC_PA5259AA	7906	198
ESC_PA5258AA	86	935
ESC_PA5257AA	101	696
ESC_PA5256AA	10	512
ESC_PA5255AA	720	900
ESC_PA5254AA	88	160
ESC_PA5253AA	8951	943
ESC_PA5252AA	10	512
ESC_PA5251AA	10	512
ESC_PA5250AA	10525	789
ESC_PA5249AA	744	512
ESC_PA5248AA	1434	512
ESC_PA5247AA	1434	512
ESC_PA5246AA	10526	76
ESC_PA5245AA	58	198
ESC_PA5244AA	2175	212
ESC_PA5243AA	10	512
ESC_PA5242AA	847	257
ESC_PA5241AA	3580	257
ESC_PA5240AA	2766	198
ESC_PA5239AA	11	936
ESC_PA5238AA	135	653
ESC_PA5237AA	567	740
ESC_PA5236AA	355	764
ESC_PA5235AA	540	286
ESC_PA5234AA	540	286
ESC_PA5233AA	348	175
ESC_PA5232AA	1011	198
ESC_PA5231AA	453	935
ESC_PA5230AA	58	198

ESC_PA5229AA	1079	235
ESC_PA5228AA	155	175
ESC_PA5227AA	429	654
ESC_PA5226AA	8588	257
ESC_PA5225AA	58	198
ESC_PA5224AA	4138	198
ESC_PA5223AA	10	512
ESC_PA5222AA	3580	257
ESC_PA5221AA	23	156
ESC_PA5220AA	117	592
ESC_PA5219AA	1081	198
ESC_PA5218AA	101	696
ESC_PA5217AA	602	257
ESC_PA5216AA	69	387
ESC_PA5215AA	12	626
ESC_PA5214AA	131	653
ESC_PA5213AA	131	653
ESC_PA5212AA	429	654
ESC_PA5211AA	117	592
ESC_PA5210AA	538	629
ESC_PA5209AA	101	696
ESC_PA5208AA	399	198
ESC_PA5207AA	38	669
ESC_PA5206AA	8619	924
ESC_PA5205AA	212	198
ESC_PA5204AA	1844	198
ESC_PA5203AA	681	393
ESC_PA5202AA	196	257
ESC_PA5201AA	10	512
ESC_PA5199AA	10527	72
ESC_PA5198AA	10	512
ESC_PA5197AA	2171	164
ESC_PA5196AA	117	592
ESC_PA5195AA	1236	380
ESC_PA5194AA	2792	886
ESC_PA5193AA	602	257
ESC_PA5192AA	1594	71
ESC_PA5191AA	95	738
ESC_PA5190AA	2271	929
ESC_PA5189AA	58	198
ESC_PA5188AA	58	198
ESC_PA5187AA	635	141
ESC_PA5186AA	297	234
ESC_PA5185AA	409	504
ESC_PA5184AA	616	156
ESC_PA5183AA	4980	445
ESC_PA5182AA	648	359
ESC_PA5181AA	428	654
ESC_PA5180AA	6032	257
ESC_PA5179AA	7618	925

ESC_PA5178AA	278	924
ESC_PA5177AA	3202	71
ESC_PA5176AA	218	519
ESC_PA5175AA	607	355
ESC_PA5174AA	355	764
ESC_PA5173AA	1914	956
ESC_PA5172AA	48	76
ESC_PA5171AA	355	764
ESC_PA5170AA	607	355
ESC_PA5169AA	23	156
ESC_PA5168AA	21	156
ESC_PA5165AA	4981	473
ESC_PA5164AA	10	512
ESC_PA5163AA	7616	257
ESC_PA5162AA	10	512
ESC_PA5161AA	10	512
ESC_PA5160AA	90	156
ESC_PA5159AA	90	156
ESC_PA5158AA	90	156
ESC_PA5157AA	641	935
ESC_PA5156AA	118	985
ESC_PA5155AA	100	504
ESC_PA5154AA	10	512
ESC_PA5153AA	1	667
ESC_PA5152AA	6321	541
ESC_PA5149AA	90	156
ESC_PA5148AA	10	512
ESC_PA5147AA	10528	175
ESC_PA5145AA	10	512
ESC_PA5144AA	10	512
ESC_PA5142AA	10	512
ESC_PA5141AA	10	512
ESC_PA5140AA	100	504
ESC_PA5139AA	10	512
ESC_PA5138AA	10	512
ESC_PA5137AA	542	824
ESC_PA5136AA	100	504
ESC_PA5135AA	10	512
ESC_PA5134AA	9956	878
ESC_PA5133AA	540	286
ESC_PA5132AA	10	512
ESC_PA5130AA	10	512
ESC_PA5129AA	10531	198
ESC_PA5128AA	4247	936
ESC_PA5126AA	132	924
ESC_PA5125AA	100	504
ESC_PA5124AA	10530	156
ESC_PA5123AA	10	512
ESC_PA5122AA	10	512
ESC_PA5121AA	156	175

ESC_PA5120AA	162	924
ESC_PA5119AA	11	936
ESC_PA5118AA	11	936
ESC_PA5117AA	11	936
ESC_PA5116AA	12	626
ESC_PA5115AA	88	160
ESC_PA5114AA	38	669
ESC_PA5113AA	12	626
ESC_PA5112AA	10	512
ESC_PA5111AA	224	198
ESC_PA5110AA	10529	234
ESC_PA5109AA	517	924
ESC_PA5108AA	372	424
ESC_PA5107AA	372	424
ESC_PA5106AA	372	424
ESC_PA5105AA	10	512
ESC_PA5104AA	10	512
ESC_PA5103AA	155	175
ESC_PA5102AA	11	936
ESC_PA5101AA	11	936
ESC_PA5100AA	11	936
ESC_PA5099AA	11	936
ESC_PA5098AA	11	936
ESC_PA5097AA	11	936
ESC_PA5096AA	655	926
ESC_PA5095AA	295	160
ESC_PA5094AA	11	936
ESC_PA5093AA	11	936
ESC_PA5092AA	11	936
ESC_PA5091AA	11	936
ESC_PA5090AA	11	936
ESC_PA5089AA	11	936
ESC_PA5088AA	11	936
ESC_PA5087AA	11	936
ESC_PA5086AA	11	936
ESC_PA5085AA	11	936
ESC_PA5084AA	11	936
ESC_PA5083AA	11	936
ESC_PA5082AA	11	936
ESC_PA5081AA	11	936
ESC_PA5080AA	517	924
ESC_PA5079AA	11	936
ESC_PA5078AA	54	256
ESC_PA5077AA	6998	629
ESC_PA5076AA	9237	938
ESC_PA5075AA	6096	257
ESC_PA5074AA	6476	854
ESC_PA5073AA	11	936
ESC_PA5072AA	121	537
ESC_PA5071AA	33	198

ESC_PA5070AA	33	198
ESC_PA5069AA	11	936
ESC_PA5068AA	442	257
ESC_PA5067AA	33	198
ESC_PA5066AA	10	512
ESC_PA5065AA	6957	221
ESC_PA5064AA	10535	198
ESC_PA5063AA	10534	156
ESC_PA5062AA	10533	735
ESC_PA5061AA	675	234
ESC_PA5060AA	10532	406
ESC_PA5059AA	33	198
ESC_PA5058AA	11	936
ESC_PA5057AA	33	198
ESC_PA5056AA	206	286
ESC_PA5055AA	11	936
ESC_PA5054AA	738	598
ESC_PA5053AA	33	198
ESC_PA5052AA	442	257
ESC_PA5051AA	29	156
ESC_PA5050AA	442	257
ESC_PA5049AA	11	936
ESC_PA5048AA	4305	512
ESC_PA5047AA	655	926
ESC_PA5046AA	11	936
ESC_PA5045AA	21	156
ESC_PA5044AA	43	512
ESC_PA5043AA	17	191
ESC_PA5042AA	173	254
ESC_PA5041AA	29	156
ESC_PA5040AA	1819	808
ESC_PA5039AA	33	198
ESC_PA5038AA	21	156
ESC_PA5037AA	25	234
ESC_PA5036AA	11	936
ESC_PA5034AA	148	191
ESC_PA5033AA	148	191
ESC_PA5032AA	95	738
ESC_PA5027AA	152	257
ESC_PA5025AA	152	257
ESC_PA5018AA	10	512
ESC_PA5017AA	46	447
ESC_PA5016AA	10512	175
ESC_PA5015AA	278	924
ESC_PA5014AA	1141	512
ESC_PA5013AA	10	512
ESC_PA5012AA	1115	263
ESC_PA5011AA	205	257
ESC_PA5010AA	101	696
ESC_PA5009AA	355	764

ESC_PA5008AA	33	198
ESC_PA5007AA	2079	198
ESC_PA5006AA	164	175
ESC_PA5005AA	949	175
ESC_PA5004AA	10520	234
ESC_PA5003AA	90	156
ESC_PA5002AA	953	447
ESC_PA5001AA	90	156
ESC_PA5000AA	373	71
ESC_PA4999AA	10	512
ESC_PA4998AA	764	170
ESC_PA4997AA	42	257
ESC_PA4996AA	131	653
ESC_PA4995AA	131	653
ESC_PA4994AA	73	737
ESC_PA4993AA	59	217
ESC_PA4992AA	162	924
ESC_PA4991AA	6901	960
ESC_PA4990AA	5268	86
ESC_PA4989AA	617	511
ESC_PA4988AA	131	653
ESC_PA4987AA	10	512
ESC_PA4986AA	127	738
ESC_PA4985AA	10187	464
ESC_PA4984AA	10	512
ESC_PA4983AA	10510	261
ESC_PA4982AA	152	257
ESC_PA4981AA	245	261
ESC_PA4980AA	245	261
ESC_PA4979AA	245	261
ESC_PA4978AA	245	261
ESC_PA4977AA	655	926
ESC_PA4976AA	21	156
ESC_PA4975AA	10	512
ESC_PA4974AA	21	156
ESC_PA4973AA	442	257
ESC_PA4972AA	25	234
ESC_PA4971AA	137	270
ESC_PA4970AA	121	537
ESC_PA4969AA	442	257
ESC_PA4968AA	6229	156
ESC_PA4967AA	442	257
ESC_PA4966AA	16	156
ESC_PA4965AA	3101	160
ESC_PA4964AA	655	926
ESC_PA4963AA	16	156
ESC_PA4962AA	69	387
ESC_PA4961AA	2563	257
ESC_PA4960AA	69	387
ESC_PA4954AA	10508	738

ESC_PA4952AA	131	653
ESC_PA4951AA	95	738
ESC_PA4949AA	1193	761
ESC_PA4947AA	599	626
ESC_PA4946AA	12	626
ESC_PA4945AA	59	217
ESC_PA4943AA	2546	914
ESC_PA4936AA	69	387
ESC_PA4934AA	2520	878
ESC_PA4933AA	446	257
ESC_PA4930AA	6096	257
ESC_PA4929AA	6241	900
ESC_PA4928AA	162	924
ESC_PA4927AA	69	387
ESC_PA4926AA	906	198
ESC_PA4918AA	11	936
ESC_PA4916AA	11	936
ESC_PA4915AA	301	307
ESC_PA4914AA	301	307
ESC_PA4913AA	21	156
ESC_PA4912AA	29	156
ESC_PA4911AA	491	626
ESC_PA4910AA	737	175
ESC_PA4909AA	738	598
ESC_PA4908AA	11	936
ESC_PA4907AA	26	257
ESC_PA4906AA	11	936
ESC_PA4905AA	11	936
ESC_PA4903AA	3101	160
ESC_PA4902AA	245	261
ESC_PA4901AA	245	261
ESC_PA4900AA	245	261
ESC_PA4899AA	245	261
ESC_PA4898AA	245	261
ESC_PA4897AA	6200	261
ESC_PA4896AA	152	257
ESC_PA4895AA	245	261
ESC_PA4894AA	245	261
ESC_PA4892AA	149	102
ESC_PA4891AA	245	261
ESC_PA4890AA	152	257
ESC_PA4889AA	245	261
ESC_PA4888AA	245	261
ESC_PA4887AA	152	257
ESC_PA4886AA	245	261
ESC_PA4885AA	245	261
ESC_PA4884AA	152	257
ESC_PA4883AA	152	257
ESC_PA4882AA	245	261
ESC_PA4881AA	245	261

ESC_PA4880AA	152	257
ESC_PA4879AA	1503	257
ESC_PA4878AA	630	261
ESC_PA4877AA	152	257
ESC_PA4876AA	245	261
ESC_PA4875AA	245	261
ESC_PA4874AA	152	257
ESC_PA4873AA	152	257
ESC_PA4872AA	152	257
ESC_PA4871AA	152	257
ESC_PA4870AA	245	261
ESC_PA4869AA	152	257
ESC_PA4868AA	8878	261
ESC_PA4867AA	245	261
ESC_PA4866AA	73	737
ESC_PA4856AA	117	592
ESC_PA4855AA	1060	512
ESC_PA4854AA	1060	512
ESC_PA4853AA	388	156
ESC_PA4851AA	11	936
ESC_PA4850AA	10	512
ESC_PA4849AA	10	512
ESC_PA4848AA	10	512
ESC_PA4847AA	4545	808
ESC_PA4846AA	11	936
ESC_PA4845AA	11	936
ESC_PA4844AA	11	936
ESC_PA4843AA	11	936
ESC_PA4842AA	11	936
ESC_PA4841AA	10	512
ESC_PA4840AA	10	512
ESC_PA4839AA	1147	198
ESC_PA4838AA	131	653
ESC_PA4837AA	164	175
ESC_PA4836AA	155	175
ESC_PA4835AA	278	924
ESC_PA4834AA	3580	257
ESC_PA4833AA	58	198
ESC_PA4832AA	10	512
ESC_PA4831AA	11	936
ESC_PA4830AA	442	257
ESC_PA4829AA	11	936
ESC_PA4828AA	11	936
ESC_PA4827AA	200	198
ESC_PA4826AA	11	936
ESC_PA4825AA	6880	726
ESC_PA4824AA	34	504
ESC_PA4823AA	5285	108
ESC_PA4822AA	607	355
ESC_PA4821AA	164	175

ESC_PA4820AA	10519	175
ESC_PA4819AA	10	512
ESC_PA4818AA	115	669
ESC_PA4817AA	10	512
ESC_PA4816AA	10	512
ESC_PA4815AA	10	512
ESC_PA4814AA	10	512
ESC_PA4813AA	10	512
ESC_PA4812AA	10	512
ESC_PA4811AA	10	512
ESC_PA4810AA	10	512
ESC_PA4809AA	10	512
ESC_PA4808AA	10	512
ESC_PA4807AA	10	512
ESC_PA4805AA	10	512
ESC_PA4804AA	10	512
ESC_PA4803AA	10	512
ESC_PA4802AA	10	512
ESC_PA4801AA	10	512
ESC_PA4800AA	10	512
ESC_PA4799AA	10	512
ESC_PA4798AA	10	512
ESC_PA4797AA	10	512
ESC_PA4796AA	10	512
ESC_PA4795AA	10	512
ESC_PA4794AA	10	512
ESC_PA4793AA	10	512
ESC_PA4792AA	10	512
ESC_PA4791AA	10	512
ESC_PA4790AA	10	512
ESC_PA4789AA	10	512
ESC_PA4788AA	10	512
ESC_PA4787AA	10	512
ESC_PA4786AA	10	512
ESC_PA4785AA	10	512
ESC_PA4784AA	10	512
ESC_PA4783AA	10	512
ESC_PA4781AA	10	512
ESC_PA4780AA	10	512
ESC_PA4779AA	10	512
ESC_PA4778AA	10	512
ESC_PA4777AA	10	512
ESC_PA4776AA	10	512
ESC_PA4775AA	10	512
ESC_PA4773AA	10	512
ESC_PA4772AA	10	512
ESC_PA4771AA	10	512
ESC_PA4770AA	10	512
ESC_PA4769AA	10	512
ESC_PA4768AA	10	512

ESC_PA4766AA	10	512
ESC_PA4765AA	10	512
ESC_PA4764AA	10	512
ESC_PA4763AA	10	512
ESC_PA4762AA	10	512
ESC_PA4761AA	10	512
ESC_PA4760AA	10	512
ESC_PA4759AA	10	512
ESC_PA4758AA	10	512
ESC_PA4757AA	9857	512
ESC_PA4756AA	10	512
ESC_PA4755AA	10	512
ESC_PA4754AA	42	257
ESC_PA4753AA	1	667
ESC_PA4752AA	10	512
ESC_PA4751AA	42	257
ESC_PA4750AA	761	512
ESC_PA4747AA	117	592
ESC_PA4746AA	38	669
ESC_PA4745AA	131	653
ESC_PA4744AA	131	653
ESC_PA4743AA	131	653
ESC_PA4742AA	40	198
ESC_PA4741AA	152	257
ESC_PA4740AA	245	261
ESC_PA4739AA	245	261
ESC_PA4738AA	152	257
ESC_PA4737AA	245	261
ESC_PA4736AA	245	261
ESC_PA4735AA	245	261
ESC_PA4734AA	245	261
ESC_PA4733AA	245	261
ESC_PA4732AA	245	261
ESC_PA4731AA	1753	261
ESC_PA4730AA	152	257
ESC_PA4729AA	245	261
ESC_PA4728AA	145	256
ESC_PA4727AA	152	257
ESC_PA4726AA	152	257
ESC_PA4725AA	245	261
ESC_PA4724AA	630	261
ESC_PA4723AA	152	257
ESC_PA4722AA	152	257
ESC_PA3908AA	73	737
ESC_PA3907AA	405	923
ESC_PA3906AA	678	257
ESC_PA3905AA	131	653
ESC_PA3904AA	681	393
ESC_PA3903AA	746	789
ESC_PA3902AA	10518	198

ESC_PA3901AA	328	924
ESC_PA3899AA	372	424
ESC_PA3898AA	2035	113
ESC_PA3896AA	101	696
ESC_PA3895AA	10517	252
ESC_PA3894AA	224	198
ESC_PA3893AA	962	198
ESC_PA3891AA	152	257
ESC_PA3890AA	152	257
ESC_PA3889AA	270	204
ESC_PA3888AA	152	257
ESC_PA3887AA	245	261
ESC_PA3886AA	10509	261
ESC_PA3885AA	145	256
ESC_PA3884AA	145	256
ESC_PA3883AA	152	257
ESC_PA3882AA	152	257
ESC_PA3881AA	245	261
ESC_PA3880AA	245	261
ESC_PA3879AA	152	257
ESC_PA3878AA	245	261
ESC_PA3877AA	245	261
ESC_PA3876AA	152	257
ESC_PA3875AA	152	257
ESC_PA3874AA	245	261
ESC_PA3873AA	152	257
ESC_PA3872AA	152	257
ESC_PA3871AA	245	261
ESC_PA3870AA	152	257
ESC_PA3869AA	152	257
ESC_PA3868AA	152	257
ESC_PA3867AA	152	257
ESC_PA3866AA	152	257
ESC_PA3865AA	152	257
ESC_PA3864AA	152	257
ESC_PA3863AA	245	261
ESC_PA3862AA	152	257
ESC_PA3861AA	245	261
ESC_PA3860AA	152	257
ESC_PA3859AA	245	261
ESC_PA3858AA	152	257
ESC_PA3857AA	245	261
ESC_PA3856AA	245	261
ESC_PA3855AA	152	257
ESC_PA3854AA	245	261
ESC_PA3853AA	245	261
ESC_PA3852AA	145	256
ESC_PA3851AA	245	261
ESC_PA3850AA	245	261
ESC_PA3849AA	152	257

ESC_PA3848AA	245	261
ESC_PA3847AA	245	261
ESC_PA3846AA	5400	261
ESC_PA3845AA	5658	257
ESC_PA3844AA	152	257
ESC_PA3843AA	152	257
ESC_PA3842AA	245	261
ESC_PA3841AA	152	257
ESC_PA3840AA	245	261
ESC_PA3839AA	152	257
ESC_PA3838AA	152	257
ESC_PA3837AA	152	257
ESC_PA3836AA	131	653
ESC_PA3835AA	1385	653
ESC_PA3834AA	131	653
ESC_PA3832AA	399	198
ESC_PA3831AA	58	198
ESC_PA3830AA	641	935
ESC_PA3829AA	399	198
ESC_PA3828AA	949	175
ESC_PA3827AA	48	76
ESC_PA3826AA	117	592
ESC_PA3825AA	1638	519
ESC_PA3824AA	372	424
ESC_PA3823AA	361	512
ESC_PA3822AA	1844	198
ESC_PA3821AA	155	175
ESC_PA3820AA	5853	868
ESC_PA3819AA	58	198
ESC_PA3818AA	10	512
ESC_PA3817AA	155	175
ESC_PA3816AA	115	669
ESC_PA3815AA	973	968
ESC_PA3814AA	2175	212
ESC_PA3813AA	127	738
ESC_PA3812AA	7804	369
ESC_PA3811AA	10511	355
ESC_PA3810AA	441	198
ESC_PA3809AA	58	198
ESC_PA3808AA	189	504
ESC_PA3807AA	162	924
ESC_PA3806AA	2536	261
ESC_PA3805AA	10	512
ESC_PA3804AA	5451	912
ESC_PA3803AA	5897	912
ESC_PA3802AA	162	924
ESC_PA3801AA	327	204
ESC_PA3800AA	5536	926
ESC_PA3799AA	2521	257
ESC_PA3798AA	2035	113

ESC_PA3797AA	58	198
ESC_PA3796AA	3188	257
ESC_PA3795AA	48	76
ESC_PA3794AA	162	924
ESC_PA3793AA	109	257
ESC_PA3792AA	1684	504
ESC_PA3791AA	278	924
ESC_PA3790AA	898	108
ESC_PA3789AA	278	924
ESC_PA3788AA	336	878
ESC_PA3787AA	4398	924
ESC_PA3786AA	342	286
ESC_PA3785AA	131	653
ESC_PA3784AA	131	653
ESC_PA3783AA	69	387
ESC_PA3782AA	95	738
ESC_PA3781AA	38	669
ESC_PA3780AA	1193	761
ESC_PA3779AA	131	653
ESC_PA3778AA	131	653
ESC_PA3777AA	131	653
ESC_PA3776AA	131	653
ESC_PA3775AA	131	653
ESC_PA3774AA	38	669
ESC_PA3773AA	648	359
ESC_PA3772AA	410	156
ESC_PA3771AA	38	669
ESC_PA3770AA	428	654
ESC_PA3769AA	428	654
ESC_PA3768AA	428	654
ESC_PA3767AA	428	654
ESC_PA3766AA	428	654
ESC_PA3765AA	428	654
ESC_PA3764AA	428	654
ESC_PA3763AA	428	654
ESC_PA3762AA	428	654
ESC_PA3761AA	428	654
ESC_PA3760AA	95	738
ESC_PA3759AA	95	738
ESC_PA3758AA	95	738
ESC_PA3757AA	95	738
ESC_PA3756AA	95	738
ESC_PA3755AA	95	738
ESC_PA3754AA	95	738
ESC_PA3753AA	95	738
ESC_PA3752AA	95	738
ESC_PA3751AA	95	738
ESC_PA3750AA	567	740
ESC_PA3749AA	567	740
ESC_PA3748AA	567	740

ESC_PA3747AA	567	740
ESC_PA3746AA	567	740
ESC_PA3745AA	567	740
ESC_PA3744AA	567	740
ESC_PA3743AA	567	740
ESC_PA3742AA	567	740
ESC_PA3741AA	567	740
ESC_PA3740AA	73	737
ESC_PA3739AA	73	737
ESC_PA3738AA	73	737
ESC_PA3737AA	73	737
ESC_PA3736AA	73	737
ESC_PA3735AA	73	737
ESC_PA3734AA	73	737
ESC_PA3733AA	73	737
ESC_PA3732AA	73	737
ESC_PA3731AA	73	737
ESC_PA3730AA	11	936
ESC_PA3729AA	11	936
ESC_PA3728AA	11	936
ESC_PA3727AA	1193	761
ESC_PA3726AA	1722	599
ESC_PA3725AA	131	653
ESC_PA3724AA	648	359
ESC_PA3723AA	744	512
ESC_PA3722AA	405	923
ESC_PA3721AA	131	653
ESC_PA3720AA	648	359
ESC_PA3719AA	131	653
ESC_PA3718AA	7706	525
ESC_PA3717AA	5442	512
ESC_PA3716AA	616	156
ESC_PA3715AA	349	670
ESC_PA3714AA	108	257
ESC_PA3713AA	2557	737
ESC_PA3712AA	1308	257
ESC_PA3711AA	6096	257
ESC_PA3710AA	1706	156
ESC_PA3709AA	5082	198
ESC_PA3708AA	1148	257
ESC_PA3707AA	10513	341
ESC_PA3706AA	617	511
ESC_PA3705AA	167	512
ESC_PA3704AA	167	512
ESC_PA3703AA	617	511
ESC_PA3702AA	973	968
ESC_PA3701AA	617	511
ESC_PA3700AA	617	511
ESC_PA3699AA	167	512
ESC_PA3698AA	167	512

ESC_PA3697AA	191	198
ESC_PA3696AA	617	511
ESC_PA3695AA	11	936
ESC_PA3694AA	11	936
ESC_PA3693AA	617	511
ESC_PA3692AA	11	936
ESC_PA3691AA	973	968
ESC_PA3690AA	617	511
ESC_PA3689AA	617	511
ESC_PA3688AA	167	512
ESC_PA3687AA	131	653
ESC_PA3686AA	648	359
ESC_PA3685AA	131	653
ESC_PA3684AA	131	653
ESC_PA3683AA	44	512
ESC_PA3682AA	131	653
ESC_PA3681AA	11	936
ESC_PA3680AA	11	936
ESC_PA3679AA	11	936
ESC_PA3678AA	725	765
ESC_PA3677AA	517	924
ESC_PA3676AA	8559	886
ESC_PA3675AA	442	257
ESC_PA3674AA	7019	936
ESC_PA3672AA	6700	512
ESC_PA3671AA	11	936
ESC_PA3670AA	11	936
ESC_PA3669AA	2028	257
ESC_PA3668AA	11	936
ESC_PA3667AA	17	191
ESC_PA3666AA	11	936
ESC_PA3665AA	442	257
ESC_PA3664AA	7032	212
ESC_PA3663AA	441	198
ESC_PA3662AA	366	198
ESC_PA3661AA	141	644
ESC_PA3660AA	10514	257
ESC_PA3659AA	10	512
ESC_PA3658AA	10	512
ESC_PA3657AA	10	512
ESC_PA3656AA	10	512
ESC_PA3655AA	10	512
ESC_PA3654AA	8559	886
ESC_PA3653AA	46	447
ESC_PA3652AA	33	198
ESC_PA3651AA	583	768
ESC_PA3650AA	6265	233
ESC_PA3649AA	6274	257
ESC_PA3648AA	941	537
ESC_PA3647AA	182	246

ESC_PA3646AA	10	512
ESC_PA3645AA	131	653
ESC_PA3644AA	442	257
ESC_PA3643AA	11	936
ESC_PA3642AA	683	175
ESC_PA3641AA	11	936
ESC_PA3640AA	11	936
ESC_PA3639AA	95	738
ESC_PA3638AA	11	936
ESC_PA3637AA	11	936
ESC_PA3636AA	11	936
ESC_PA3635AA	11	936
ESC_PA3634AA	11	936
ESC_PA3633AA	131	653
ESC_PA3632AA	167	512
ESC_PA3630AA	131	653
ESC_PA3629AA	131	653
ESC_PA3628AA	405	923
ESC_PA3627AA	131	653
ESC_PA3626AA	648	359
ESC_PA3625AA	1193	761
ESC_PA3624AA	131	653
ESC_PA3623AA	602	257
ESC_PA3622AA	10515	772
ESC_PA3621AA	21	156
ESC_PA3620AA	10	512
ESC_PA3619AA	21	156
ESC_PA3618AA	34	504
ESC_PA3617AA	34	504
ESC_PA3616AA	6506	914
ESC_PA3615AA	442	257
ESC_PA3614AA	4	76
ESC_PA3613AA	7326	156
ESC_PA3612AA	675	234
ESC_PA3611AA	182	246
ESC_PA3610AA	4	76
ESC_PA3609AA	11	936
ESC_PA3608AA	11	936
ESC_PA3607AA	11	936
ESC_PA3606AA	11	936
ESC_PA3605AA	11	936
ESC_PA3604AA	11	936
ESC_PA3603AA	11	936
ESC_PA3602AA	11	936
ESC_PA3601AA	11	936
ESC_PA3600AA	11	936
ESC_PA3599AA	11	936
ESC_PA3598AA	11	936
ESC_PA3597AA	131	653
ESC_PA3596AA	131	653

ESC_PA3595AA	131	653
ESC_PA3594AA	131	653
ESC_PA3593AA	131	653
ESC_PA3592AA	648	359
ESC_PA3591AA	648	359
ESC_PA3590AA	131	653
ESC_PA3589AA	127	738
ESC_PA3588AA	1304	257
ESC_PA3587AA	372	424
ESC_PA3586AA	95	738
ESC_PA3585AA	963	669
ESC_PA3584AA	349	670
ESC_PA3583AA	5414	878
ESC_PA3582AA	12	626
ESC_PA3581AA	6669	433
ESC_PA3580AA	538	629
ESC_PA3579AA	10516	738
ESC_PA3578AA	7250	919
ESC_PA3577AA	297	234
ESC_PA3576AA	362	234
ESC_PA3575AA	362	234
ESC_PA3574AA	1257	649
ESC_PA3573AA	967	212
ESC_PA3572AA	117	592
ESC_PA3571AA	362	234
ESC_PA3570AA	58	198
ESC_PA3569AA	108	257
ESC_PA3568AA	9001	660
ESC_PA3567AA	40	198
ESC_PA3566AA	69	387
ESC_PA3565AA	2165	257
ESC_PA3564AA	847	257
ESC_PA3563AA	681	393
ESC_PA3562AA	720	900
ESC_PA3561AA	1850	269
ESC_PA3560AA	6998	629
ESC_PA3559AA	10	512
ESC_PA3558AA	10	512
ESC_PA3557AA	4247	936
ESC_PA3556AA	126	740
ESC_PA3555AA	90	156
ESC_PA3554AA	10	512
ESC_PA3553AA	10	512
ESC_PA3552AA	10	512
ESC_PA3551AA	23	156
ESC_PA3550AA	953	447
ESC_PA3549AA	10	512
ESC_PA3548AA	641	935
ESC_PA3547AA	131	653
ESC_PA3546AA	641	935

ESC_PA3545AA	761	512
ESC_PA3544AA	100	504
ESC_PA3543AA	93	71
ESC_PA3541AA	90	156
ESC_PA3540AA	100	504
ESC_PA3539AA	10	512
ESC_PA3538AA	6691	878
ESC_PA3537AA	761	512
ESC_PA3536AA	206	286
ESC_PA3535AA	206	286
ESC_PA3534AA	373	71
ESC_PA3533AA	10	512
ESC_PA3532AA	90	156
ESC_PA3531AA	100	504
ESC_PA3530AA	9217	935
ESC_PA3529AA	641	935
ESC_PA3528AA	90	156
ESC_PA3527AA	100	504
ESC_PA3526AA	10	512
ESC_PA3525AA	93	71
ESC_PA3524AA	100	504
ESC_PA3523AA	10	512
ESC_PA3522AA	10	512
ESC_PA3521AA	761	512
ESC_PA3520AA	1	667
ESC_PA3519AA	10	512
ESC_PA3518AA	100	504
ESC_PA3517AA	100	504
ESC_PA3516AA	1146	198
ESC_PA3515AA	5378	197
ESC_PA3514AA	1434	512
ESC_PA3513AA	428	654
ESC_PA3512AA	3580	257
ESC_PA3511AA	1510	4
ESC_PA3510AA	86	935
ESC_PA3509AA	4267	257
ESC_PA3508AA	2607	198
ESC_PA3507AA	1079	235
ESC_PA3506AA	4980	445
ESC_PA3505AA	457	595
ESC_PA3504AA	1914	956
ESC_PA3503AA	1079	235
ESC_PA3502AA	93	71
ESC_PA3501AA	117	592
ESC_PA3500AA	10	512
ESC_PA3499AA	746	789
ESC_PA3498AA	746	789
ESC_PA3497AA	93	71
ESC_PA3496AA	212	198
ESC_PA3495AA	8146	234

ESC_PA3494AA	1011	198
ESC_PA3493AA	616	156
ESC_PA3492AA	746	789
ESC_PA3491AA	9721	234
ESC_PA3490AA	2476	198
ESC_PA3489AA	117	592
ESC_PA3488AA	1582	198
ESC_PA3487AA	156	175
ESC_PA3486AA	746	789
ESC_PA3485AA	1844	198
ESC_PA3484AA	2178	924
ESC_PA3483AA	48	76
ESC_PA3482AA	1485	408
ESC_PA3481AA	223	198
ESC_PA3480AA	48	76
ESC_PA3479AA	3107	512
ESC_PA3478AA	2161	198
ESC_PA3477AA	155	175
ESC_PA3476AA	212	198
ESC_PA3475AA	43	512
ESC_PA3474AA	155	175
ESC_PA3473AA	373	71
ESC_PA3472AA	90	156
ESC_PA3471AA	115	669
ESC_PA3470AA	354	15
ESC_PA3469AA	714	318
ESC_PA3468AA	117	592
ESC_PA3467AA	2309	669
ESC_PA3466AA	10	512
ESC_PA3465AA	10	512
ESC_PA3464AA	362	234
ESC_PA3463AA	665	79
ESC_PA3462AA	93	71
ESC_PA3461AA	429	654
ESC_PA3460AA	3057	672
ESC_PA3459AA	23	156
ESC_PA3458AA	1158	387
ESC_PA3457AA	68	386
ESC_PA3456AA	648	359
ESC_PA3455AA	57	50
ESC_PA3454AA	93	71
ESC_PA3453AA	648	359
ESC_PA3452AA	57	50
ESC_PA3451AA	9719	512
ESC_PA3450AA	155	175
ESC_PA3449AA	69	387
ESC_PA3448AA	4243	914
ESC_PA3447AA	117	592
ESC_PA3446AA	9720	156
ESC_PA3445AA	9718	235

ESC_PA3444AA	420	741
ESC_PA3443AA	38	669
ESC_PA3442AA	196	257
ESC_PA3440AA	58	198
ESC_PA3439AA	212	198
ESC_PA3438AA	1630	76
ESC_PA3437AA	2309	669
ESC_PA3436AA	48	76
ESC_PA3435AA	93	71
ESC_PA3434AA	156	175
ESC_PA3433AA	10	512
ESC_PA3432AA	4243	914
ESC_PA3431AA	131	653
ESC_PA3430AA	10	512
ESC_PA3429AA	48	76
ESC_PA3428AA	665	79
ESC_PA3427AA	6731	669
ESC_PA3426AA	328	924
ESC_PA3425AA	746	789
ESC_PA3424AA	300	286
ESC_PA3423AA	11	936
ESC_PA3422AA	517	924
ESC_PA3421AA	21	156
ESC_PA3420AA	582	420
ESC_PA3419AA	655	926
ESC_PA3418AA	811	234
ESC_PA3417AA	11	936
ESC_PA3416AA	6274	257
ESC_PA3415AA	11	936
ESC_PA3414AA	17	191
ESC_PA3412AA	10	512
ESC_PA3411AA	21	156
ESC_PA3410AA	11	936
ESC_PA3409AA	11	936
ESC_PA3408AA	5743	257
ESC_PA3407AA	11	936
ESC_PA3406AA	11	936
ESC_PA3405AA	11	936
ESC_PA2458AA	38	669
ESC_PA2457AA	963	669
ESC_PA2456AA	963	669
ESC_PA2455AA	963	669
ESC_PA2454AA	963	669
ESC_PA2453AA	1508	261
ESC_PA2452AA	423	204
ESC_PA2451AA	1508	261
ESC_PA2450AA	5434	0
ESC_PA2449AA	131	653
ESC_PA2448AA	10436	257
ESC_PA2446AA	10471	936

ESC_PA2445AA	10470	257
ESC_PA2444AA	8660	175
ESC_PA2443AA	8908	156
ESC_PA2442AA	10469	257
ESC_PA2440AA	678	257
ESC_PA2439AA	245	261
ESC_PA2438AA	152	257
ESC_PA2437AA	245	261
ESC_PA2436AA	10451	19
ESC_PA2435AA	21	156
ESC_PA2434AA	29	156
ESC_PA2432AA	38	669
ESC_PA2431AA	46	447
ESC_PA2430AA	10439	257
ESC_PA2429AA	10438	257
ESC_PA2428AA	10437	936
ESC_PA2427AA	10436	257
ESC_PA2426AA	8660	175
ESC_PA2425AA	4138	198
ESC_PA2423AA	410	156
ESC_PA2422AA	58	198
ESC_PA2421AA	155	175
ESC_PA2420AA	224	198
ESC_PA2419AA	155	175
ESC_PA2418AA	10431	227
ESC_PA2416AA	10421	286
ESC_PA2415AA	10420	936
ESC_PA2414AA	10416	50
ESC_PA2413AA	10423	257
ESC_PA2412AA	10418	198
ESC_PA2411AA	10417	232
ESC_PA2410AA	10407	156
ESC_PA2409AA	10406	387
ESC_PA2408AA	10222	738
ESC_PA2407AA	10414	738
ESC_PA2406AA	10415	737
ESC_PA2405AA	10252	626
ESC_PA2404AA	10413	737
ESC_PA2403AA	10405	653
ESC_PA2402AA	10404	512
ESC_PA2401AA	10403	420
ESC_PA2400AA	10237	424
ESC_PA2399AA	10402	737
ESC_PA2398AA	10271	626
ESC_PA2396AA	10401	574
ESC_PA2395AA	10411	738
ESC_PA2394AA	10288	737
ESC_PA2393AA	10410	741
ESC_PA2392AA	10412	626
ESC_PA2391AA	10409	728

ESC_PA2390AA	10408	737
ESC_PA2389AA	131	653
ESC_PA2388AA	372	424
ESC_PA2387AA	167	512
ESC_PA2386AA	131	653
ESC_PA2385AA	998	644
ESC_PA2384AA	998	644
ESC_PA2383AA	998	644
ESC_PA2382AA	1193	761
ESC_PA2381AA	131	653
ESC_PA2380AA	8881	362
ESC_PA2379AA	21	156
ESC_PA2378AA	21	156
ESC_PA2377AA	21	156
ESC_PA2376AA	21	156
ESC_PA2375AA	7006	936
ESC_PA2374AA	152	257
ESC_PA2373AA	152	257
ESC_PA2372AA	152	257
ESC_PA2371AA	1503	257
ESC_PA2370AA	73	737
ESC_PA2369AA	12	626
ESC_PA2368AA	12	626
ESC_PA2367AA	383	123
ESC_PA2366AA	383	123
ESC_PA2365AA	383	123
ESC_PA2364AA	383	123
ESC_PA2363AA	383	123
ESC_PA2362AA	6373	828
ESC_PA2361AA	5578	830
ESC_PA2360AA	413	121
ESC_PA2357AA	10	512
ESC_PA2356AA	10	512
ESC_PA2353AA	10	512
ESC_PA2352AA	10	512
ESC_PA2351AA	10459	512
ESC_PA2350AA	10	512
ESC_PA2349AA	10	512
ESC_PA2347AA	10	512
ESC_PA2346AA	10	512
ESC_PA2343AA	3268	669
ESC_PA2342AA	3268	669
ESC_PA2341AA	189	504
ESC_PA2340AA	10	512
ESC_PA2339AA	48	76
ESC_PA2338AA	155	175
ESC_PA2337AA	10	512
ESC_PA2336AA	156	175
ESC_PA2335AA	7941	504
ESC_PA2334AA	6756	962

ESC_PA2333AA	10	512
ESC_PA2332AA	10	512
ESC_PA2331AA	1011	198
ESC_PA2330AA	1684	504
ESC_PA2329AA	206	286
ESC_PA2328AA	10460	71
ESC_PA2327AA	10	512
ESC_PA2326AA	10	512
ESC_PA2325AA	3941	71
ESC_PA2324AA	3586	406
ESC_PA2323AA	1011	198
ESC_PA2322AA	206	286
ESC_PA2321AA	7454	286
ESC_PA2320AA	6764	504
ESC_PA2319AA	10	512
ESC_PA2317AA	10	512
ESC_PA2316AA	10	512
ESC_PA2314AA	10	512
ESC_PA2313AA	33	198
ESC_PA2312AA	201	257
ESC_PA2311AA	3580	257
ESC_PA2310AA	21	156
ESC_PA2309AA	21	156
ESC_PA2308AA	5133	504
ESC_PA2307AA	2388	198
ESC_PA2306AA	21	156
ESC_PA2305AA	6661	198
ESC_PA2304AA	11	936
ESC_PA2303AA	11	936
ESC_PA2302AA	1122	307
ESC_PA2301AA	11	936
ESC_PA2300AA	11	936
ESC_PA2299AA	11	936
ESC_PA2298AA	11	936
ESC_PA2297AA	11	936
ESC_PA2296AA	21	156
ESC_PA2295AA	11	936
ESC_PA2294AA	21	156
ESC_PA2293AA	2388	198
ESC_PA2292AA	3546	512
ESC_PA2291AA	2388	198
ESC_PA2290AA	11	936
ESC_PA2289AA	11	936
ESC_PA2288AA	11	936
ESC_PA2287AA	11	936
ESC_PA2286AA	16	156
ESC_PA2285AA	2388	198
ESC_PA2284AA	2385	198
ESC_PA2283AA	88	160
ESC_PA2282AA	33	198

ESC_PA2281AA	165	504
ESC_PA2280AA	11	936
ESC_PA2279AA	11	936
ESC_PA2278AA	11	936
ESC_PA2277AA	11	936
ESC_PA2276AA	11	936
ESC_PA2275AA	11	936
ESC_PA2274AA	11	936
ESC_PA2273AA	11	936
ESC_PA2272AA	11	936
ESC_PA2271AA	11	936
ESC_PA2270AA	11	936
ESC_PA2269AA	11	936
ESC_PA2268AA	11	936
ESC_PA2267AA	11	936
ESC_PA2266AA	11	936
ESC_PA2265AA	11	936
ESC_PA2264AA	11	936
ESC_PA2263AA	11	936
ESC_PA2262AA	11	936
ESC_PA2261AA	11	936
ESC_PA2260AA	11	936
ESC_PA2259AA	11	936
ESC_PA2258AA	11	936
ESC_PA2257AA	11	936
ESC_PA2256AA	11	936
ESC_PA2255AA	11	936
ESC_PA2254AA	11	936
ESC_PA2253AA	11	936
ESC_PA2252AA	11	936
ESC_PA2251AA	11	936
ESC_PA2250AA	11	936
ESC_PA2249AA	11	936
ESC_PA2248AA	11	936
ESC_PA2247AA	11	936
ESC_PA2246AA	11	936
ESC_PA2245AA	11	936
ESC_PA2244AA	11	936
ESC_PA2243AA	11	936
ESC_PA2242AA	11	936
ESC_PA2236AA	10	512
ESC_PA2234AA	10	512
ESC_PA2233AA	10	512
ESC_PA2232AA	10	512
ESC_PA2231AA	10	512
ESC_PA2230AA	10	512
ESC_PA2229AA	10	512
ESC_PA2228AA	10	512
ESC_PA2227AA	10	512
ESC_PA2226AA	10	512

ESC_PA2225AA	10	512
ESC_PA2224AA	10	512
ESC_PA2223AA	10	512
ESC_PA2222AA	10	512
ESC_PA2221AA	10	512
ESC_PA2220AA	10	512
ESC_PA2219AA	10	512
ESC_PA2218AA	10	512
ESC_PA2217AA	10	512
ESC_PA2216AA	442	257
ESC_PA2215AA	1125	198
ESC_PA2214AA	446	257
ESC_PA2213AA	442	257
ESC_PA2212AA	442	257
ESC_PA2211AA	442	257
ESC_PA2210AA	446	257
ESC_PA2209AA	442	257
ESC_PA2208AA	1125	198
ESC_PA2207AA	306	286
ESC_PA2206AA	5559	257
ESC_PA2205AA	446	257
ESC_PA2204AA	446	257
ESC_PA2203AA	1125	198
ESC_PA2202AA	1125	198
ESC_PA2201AA	5487	198
ESC_PA2200AA	6118	257
ESC_PA2199AA	5978	924
ESC_PA2198AA	10461	644
ESC_PA2197AA	5559	257
ESC_PA2196AA	1125	198
ESC_PA2195AA	446	257
ESC_PA2194AA	442	257
ESC_PA2193AA	1125	198
ESC_PA2192AA	1125	198
ESC_PA2191AA	446	257
ESC_PA2190AA	446	257
ESC_PA2189AA	446	257
ESC_PA2188AA	442	257
ESC_PA2187AA	1125	198
ESC_PA2186AA	21	156
ESC_PA2185AA	5978	924
ESC_PA2184AA	5559	257
ESC_PA2183AA	7715	257
ESC_PA2182AA	21	156
ESC_PA2181AA	1125	198
ESC_PA2180AA	4038	257
ESC_PA2179AA	7715	257
ESC_PA2178AA	7715	257
ESC_PA2177AA	1125	198
ESC_PA2176AA	7715	257

ESC_PA2175AA	1125	198
ESC_PA2174AA	7715	257
ESC_PA2173AA	5559	257
ESC_PA2172AA	7715	257
ESC_PA2171AA	5559	257
ESC_PA2170AA	5978	924
ESC_PA2169AA	1125	198
ESC_PA2168AA	1125	198
ESC_PA2167AA	5559	257
ESC_PA2166AA	446	257
ESC_PA2165AA	446	257
ESC_PA2164AA	1125	198
ESC_PA2163AA	442	257
ESC_PA2162AA	446	257
ESC_PA2161AA	442	257
ESC_PA2160AA	1125	198
ESC_PA2159AA	442	257
ESC_PA2158AA	446	257
ESC_PA2157AA	446	257
ESC_PA2156AA	7715	257
ESC_PA2155AA	5978	924
ESC_PA2154AA	5978	924
ESC_PA2153AA	5978	924
ESC_PA2152AA	7715	257
ESC_PA2151AA	1125	198
ESC_PA2150AA	300	286
ESC_PA2149AA	7715	257
ESC_PA2148AA	1125	198
ESC_PA2147AA	5487	198
ESC_PA2146AA	446	257
ESC_PA2145AA	1125	198
ESC_PA2144AA	446	257
ESC_PA2143AA	446	257
ESC_PA2142AA	1125	198
ESC_PA2141AA	1125	198
ESC_PA2140AA	1125	198
ESC_PA2139AA	442	257
ESC_PA2138AA	442	257
ESC_PA2137AA	1125	198
ESC_PA2136AA	7715	257
ESC_PA2135AA	1125	198
ESC_PA2134AA	5978	924
ESC_PA2133AA	5559	257
ESC_PA2132AA	446	257
ESC_PA2131AA	7715	257
ESC_PA2130AA	5978	924
ESC_PA2129AA	1125	198
ESC_PA2128AA	5487	198
ESC_PA2127AA	446	257
ESC_PA2126AA	6118	257

ESC_PA2125AA	446	257
ESC_PA2124AA	1125	198
ESC_PA2123AA	5559	257
ESC_PA2122AA	300	286
ESC_PA2121AA	5978	924
ESC_PA2120AA	1125	198
ESC_PA2119AA	446	257
ESC_PA2118AA	7715	257
ESC_PA2117AA	154	257
ESC_PA2116AA	446	257
ESC_PA2115AA	1125	198
ESC_PA2114AA	16	156
ESC_PA2113AA	446	257
ESC_PA2112AA	1125	198
ESC_PA2111AA	7715	257
ESC_PA2110AA	442	257
ESC_PA2109AA	1125	198
ESC_PA2108AA	5978	924
ESC_PA2107AA	297	234
ESC_PA2106AA	5487	198
ESC_PA2105AA	1125	198
ESC_PA2104AA	937	198
ESC_PA2103AA	11	936
ESC_PA2102AA	11	936
ESC_PA2101AA	11	936
ESC_PA2100AA	1125	198
ESC_PA2099AA	329	355
ESC_PA2098AA	329	355
ESC_PA2097AA	7715	257
ESC_PA2096AA	7715	257
ESC_PA2095AA	7715	257
ESC_PA2094AA	5978	924
ESC_PA2093AA	154	257
ESC_PA2092AA	446	257
ESC_PA2091AA	5559	257
ESC_PA2090AA	5978	924
ESC_PA2089AA	446	257
ESC_PA2088AA	7715	257
ESC_PA2087AA	223	198
ESC_PA2086AA	1125	198
ESC_PA2085AA	442	257
ESC_PA2084AA	7715	257
ESC_PA2083AA	5559	257
ESC_PA2082AA	446	257
ESC_PA2081AA	7715	257
ESC_PA2080AA	5978	924
ESC_PA2079AA	5978	924
ESC_PA2078AA	1125	198
ESC_PA2077AA	937	198
ESC_PA2076AA	937	198

ESC_PA2075AA	446	257
ESC_PA2074AA	446	257
ESC_PA2073AA	5419	175
ESC_PA2072AA	442	257
ESC_PA2071AA	1125	198
ESC_PA2070AA	446	257
ESC_PA2069AA	1125	198
ESC_PA2068AA	11	936
ESC_PA2067AA	5978	924
ESC_PA2066AA	223	198
ESC_PA2065AA	21	156
ESC_PA2064AA	6118	257
ESC_PA2063AA	1125	198
ESC_PA2062AA	6118	257
ESC_PA2061AA	5559	257
ESC_PA2060AA	1125	198
ESC_PA2059AA	5559	257
ESC_PA2058AA	5559	257
ESC_PA2057AA	7715	257
ESC_PA2056AA	1125	198
ESC_PA2055AA	1125	198
ESC_PA2054AA	446	257
ESC_PA2053AA	1125	198
ESC_PA2052AA	1125	198
ESC_PA2051AA	442	257
ESC_PA2050AA	5978	924
ESC_PA2049AA	3018	903
ESC_PA2048AA	442	257
ESC_PA2047AA	442	257
ESC_PA2046AA	3018	903
ESC_PA2045AA	442	257
ESC_PA2044AA	446	257
ESC_PA2043AA	1125	198
ESC_PA2042AA	442	257
ESC_PA2041AA	1125	198
ESC_PA2040AA	1125	198
ESC_PA2039AA	56	198
ESC_PA2038AA	442	257
ESC_PA2037AA	131	653
ESC_PA2036AA	2016	243
ESC_PA2035AA	10	512
ESC_PA2034AA	117	592
ESC_PA2033AA	140	738
ESC_PA2032AA	23	156
ESC_PA2031AA	95	738
ESC_PA2030AA	3559	900
ESC_PA2029AA	131	653
ESC_PA2028AA	38	669
ESC_PA2027AA	38	669
ESC_PA2026AA	405	923

ESC_PA2025AA	131	653
ESC_PA2024AA	131	653
ESC_PA2023AA	648	359
ESC_PA2022AA	131	653
ESC_PA2021AA	131	653
ESC_PA2020AA	131	653
ESC_PA2019AA	131	653
ESC_PA2018AA	69	387
ESC_PA2017AA	131	653
ESC_PA2016AA	38	669
ESC_PA2015AA	131	653
ESC_PA2014AA	38	669
ESC_PA2013AA	8347	653
ESC_PA2012AA	38	669
ESC_PA2011AA	131	653
ESC_PA2010AA	131	653
ESC_PA2009AA	141	644
ESC_PA2008AA	131	653
ESC_PA2007AA	131	653
ESC_PA2006AA	131	653
ESC_PA2005AA	131	653
ESC_PA2004AA	131	653
ESC_PA2003AA	131	653
ESC_PA2002AA	131	653
ESC_PA2001AA	38	669
ESC_PA2000AA	405	923
ESC_PA1999AA	131	653
ESC_PA1998AA	131	653
ESC_PA1997AA	131	653
ESC_PA1996AA	131	653
ESC_PA1995AA	372	424
ESC_PA1994AA	131	653
ESC_PA1993AA	131	653
ESC_PA1992AA	131	653
ESC_PA1991AA	131	653
ESC_PA1990AA	131	653
ESC_PA1989AA	141	644
ESC_PA1988AA	1722	599
ESC_PA1987AA	1193	761
ESC_PA1986AA	1193	761
ESC_PA1985AA	648	359
ESC_PA1984AA	69	387
ESC_PA1983AA	617	511
ESC_PA1982AA	131	653
ESC_PA1981AA	393	343
ESC_PA1980AA	8347	653
ESC_PA1979AA	131	653
ESC_PA1978AA	617	511
ESC_PA1977AA	38	669
ESC_PA1976AA	38	669

ESC_PA1975AA	131	653
ESC_PA1974AA	131	653
ESC_PA1973AA	69	387
ESC_PA1972AA	131	653
ESC_PA1971AA	131	653
ESC_PA1970AA	131	653
ESC_PA1969AA	648	359
ESC_PA1968AA	10462	923
ESC_PA1967AA	131	653
ESC_PA1966AA	8347	653
ESC_PA1965AA	131	653
ESC_PA1964AA	38	669
ESC_PA1963AA	131	653
ESC_PA1962AA	131	653
ESC_PA1961AA	348	175
ESC_PA1960AA	69	387
ESC_PA1959AA	131	653
ESC_PA1958AA	648	359
ESC_PA1957AA	162	924
ESC_PA1956AA	131	653
ESC_PA1955AA	131	653
ESC_PA1954AA	131	653
ESC_PA1953AA	405	923
ESC_PA1952AA	38	669
ESC_PA1951AA	131	653
ESC_PA1950AA	131	653
ESC_PA1949AA	131	653
ESC_PA1948AA	38	669
ESC_PA1947AA	38	669
ESC_PA1946AA	46	447
ESC_PA1945AA	38	669
ESC_PA1944AA	131	653
ESC_PA1943AA	131	653
ESC_PA1942AA	38	669
ESC_PA1939AA	394	400
ESC_PA1938AA	349	670
ESC_PA1937AA	38	669
ESC_PA1936AA	10507	501
ESC_PA1935AA	349	670
ESC_PA1934AA	10464	111
ESC_PA1933AA	48	76
ESC_PA1932AA	1193	761
ESC_PA1931AA	38	669
ESC_PA1930AA	1163	579
ESC_PA1929AA	442	257
ESC_PA1928AA	2732	234
ESC_PA1927AA	773	71
ESC_PA1926AA	10	512
ESC_PA1925AA	3580	257
ESC_PA1924AA	10463	355

ESC_PA1923AA	648	359
ESC_PA1922AA	38	669
ESC_PA1921AA	95	738
ESC_PA1920AA	10	512
ESC_PA1919AA	131	653
ESC_PA1918AA	1193	761
ESC_PA1917AA	405	923
ESC_PA1916AA	69	387
ESC_PA1914AA	48	76
ESC_PA1913AA	48	76
ESC_PA1912AA	131	653
ESC_PA1911AA	28	537
ESC_PA1910AA	603	198
ESC_PA1909AA	648	359
ESC_PA1908AA	394	400
ESC_PA1907AA	1193	761
ESC_PA1906AA	38	669
ESC_PA1905AA	117	592
ESC_PA1904AA	131	653
ESC_PA1903AA	657	592
ESC_PA1902AA	131	653
ESC_PA1901AA	93	71
ESC_PA1900AA	93	71
ESC_PA1899AA	617	511
ESC_PA1898AA	101	696
ESC_PA1897AA	3045	670
ESC_PA1896AA	23	156
ESC_PA1895AA	38	669
ESC_PA1894AA	744	512
ESC_PA1893AA	998	644
ESC_PA1892AA	10	512
ESC_PA1891AA	38	669
ESC_PA1890AA	131	653
ESC_PA1889AA	196	257
ESC_PA1888AA	23	156
ESC_PA1887AA	10	512
ESC_PA1886AA	1193	761
ESC_PA1885AA	131	653
ESC_PA1884AA	69	387
ESC_PA1883AA	4040	696
ESC_PA1882AA	38	669
ESC_PA1881AA	443	257
ESC_PA1880AA	2179	924
ESC_PA1879AA	131	653
ESC_PA1878AA	2179	924
ESC_PA1877AA	131	653
ESC_PA1876AA	7394	257
ESC_PA1875AA	38	669
ESC_PA1874AA	10506	37
ESC_PA1873AA	450	71

ESC_PA1872AA	131	653
ESC_PA1871AA	131	653
ESC_PA1870AA	1193	761
ESC_PA1869AA	131	653
ESC_PA1854AA	3021	447
ESC_PA1851AA	3021	447
ESC_PA1850AA	3021	447
ESC_PA1848AA	3021	447
ESC_PA1845AA	3021	447
ESC_PA1844AA	3021	447
ESC_PA1808AA	8365	123
ESC_PA1807AA	8367	764
ESC_PA1806AA	8373	764
ESC_PA1804AA	8368	286
ESC_PA1803AA	8366	282
ESC_PA1802AA	2087	118
ESC_PA1801AA	2087	118
ESC_PA1800AA	11	936
ESC_PA1799AA	11	936
ESC_PA1798AA	11	936
ESC_PA1797AA	11	936
ESC_PA1796AA	11	936
ESC_PA1795AA	11	936
ESC_PA1794AA	11	936
ESC_PA1793AA	11	936
ESC_PA1792AA	11	936
ESC_PA1791AA	11	936
ESC_PA1790AA	11	936
ESC_PA1788AA	131	653
ESC_PA1786AA	131	653
ESC_PA1783AA	11	936
ESC_PA1782AA	11	936
ESC_PA1781AA	11	936
ESC_PA1780AA	11	936
ESC_PA1779AA	11	936
ESC_PA1778AA	11	936
ESC_PA1777AA	11	936
ESC_PA1776AA	11	936
ESC_PA1775AA	11	936
ESC_PA1774AA	11	936
ESC_PA1773AA	11	936
ESC_PA1772AA	11	936
ESC_PA1771AA	11	936
ESC_PA1770AA	11	936
ESC_PA1769AA	11	936
ESC_PA1768AA	11	936
ESC_PA1767AA	11	936
ESC_PA1766AA	11	936
ESC_PA1765AA	11	936
ESC_PA1764AA	11	936

ESC_PA1763AA	11	936
ESC_PA1762AA	11	936
ESC_PA1761AA	11	936
ESC_PA1760AA	11	936
ESC_PA1759AA	11	936
ESC_PA1758AA	11	936
ESC_PA1757AA	11	936
ESC_PA1756AA	11	936
ESC_PA1755AA	11	936
ESC_PA1754AA	11	936
ESC_PA1753AA	11	936
ESC_PA1752AA	11	936
ESC_PA1751AA	11	936
ESC_PA1750AA	11	936
ESC_PA1749AA	11	936
ESC_PA1748AA	11	936
ESC_PA1747AA	11	936
ESC_PA1746AA	11	936
ESC_PA1745AA	11	936
ESC_PA1744AA	11	936
ESC_PA1743AA	11	936
ESC_PA1742AA	11	936
ESC_PA1741AA	11	936
ESC_PA1740AA	11	936
ESC_PA1739AA	11	936
ESC_PA1738AA	11	936
ESC_PA1737AA	11	936
ESC_PA1736AA	11	936
ESC_PA1735AA	11	936
ESC_PA1734AA	11	936
ESC_PA1733AA	11	936
ESC_PA1732AA	11	936
ESC_PA1731AA	11	936
ESC_PA1730AA	11	936
ESC_PA1729AA	10466	936
ESC_PA1728AA	11	936
ESC_PA1727AA	11	936
ESC_PA1726AA	11	936
ESC_PA1725AA	11	936
ESC_PA1724AA	11	936
ESC_PA1723AA	11	936
ESC_PA1722AA	11	936
ESC_PA1721AA	11	936
ESC_PA1720AA	11	936
ESC_PA1719AA	11	936
ESC_PA1718AA	11	936
ESC_PA1717AA	11	936
ESC_PA1716AA	11	936
ESC_PA1715AA	11	936
ESC_PA1714AA	11	936

ESC_PA1713AA	11	936
ESC_PA1712AA	10465	936
ESC_PA1711AA	11	936
ESC_PA1710AA	11	936
ESC_PA1709AA	11	936
ESC_PA1708AA	11	936
ESC_PA1707AA	11	936
ESC_PA1706AA	11	936
ESC_PA1705AA	11	936
ESC_PA1704AA	11	936
ESC_PA1703AA	11	936
ESC_PA1702AA	11	936
ESC_PA1701AA	11	936
ESC_PA1700AA	11	936
ESC_PA1699AA	11	936
ESC_PA1698AA	11	936
ESC_PA1697AA	11	936
ESC_PA1696AA	11	936
ESC_PA1695AA	11	936
ESC_PA1694AA	11	936
ESC_PA1693AA	11	936
ESC_PA1692AA	11	936
ESC_PA1691AA	11	936
ESC_PA1690AA	11	936
ESC_PA1689AA	11	936
ESC_PA1688AA	11	936
ESC_PA1687AA	10468	936
ESC_PA1686AA	11	936
ESC_PA1685AA	11	936
ESC_PA1684AA	11	936
ESC_PA1683AA	11	936
ESC_PA1682AA	10467	936
ESC_PA1681AA	11	936
ESC_PA1680AA	11	936
ESC_PA1679AA	11	936
ESC_PA1678AA	11	936
ESC_PA1677AA	11	936
ESC_PA1676AA	11	936
ESC_PA1675AA	11	936
ESC_PA1674AA	11	936
ESC_PA1673AA	11	936
ESC_PA1672AA	11	936
ESC_PA1671AA	11	936
ESC_PA1670AA	11	936
ESC_PA1669AA	11	936
ESC_PA1668AA	11	936
ESC_PA1667AA	11	936
ESC_PA1666AA	11	936
ESC_PA1665AA	11	936
ESC_PA1664AA	11	936

ESC_PA1663AA	11	936
ESC_PA1662AA	11	936
ESC_PA1661AA	11	936
ESC_PA1660AA	11	936
ESC_PA1659AA	11	936
ESC_PA1658AA	11	936
ESC_PA1657AA	11	936
ESC_PA1656AA	11	936
ESC_PA1655AA	11	936
ESC_PA1654AA	11	936
ESC_PA1653AA	11	936
ESC_PA1652AA	11	936
ESC_PA1651AA	11	936
ESC_PA1650AA	11	936
ESC_PA1649AA	11	936
ESC_PA1648AA	11	936
ESC_PA1647AA	11	936
ESC_PA1646AA	11	936
ESC_PA1645AA	11	936
ESC_PA1644AA	11	936
ESC_PA1642AA	11	936
ESC_PA1641AA	11	936
ESC_PA1640AA	11	936
ESC_PA1616AA	11	936
ESC_PA1615AA	11	936
ESC_PA1614AA	11	936
ESC_PA1613AA	11	936
ESC_PA1612AA	11	936
ESC_PA1611AA	11	936
ESC_PA1610AA	11	936
ESC_PA1609AA	11	936
ESC_PA1608AA	11	936
ESC_PA1607AA	11	936
ESC_PA1606AA	11	936
ESC_PA1605AA	11	936
ESC_PA1604AA	11	936
ESC_PA1603AA	11	936
ESC_PA1602AA	11	936
ESC_PA1601AA	11	936
ESC_PA1599AA	11	936
ESC_PA1598AA	11	936
ESC_PA1597AA	80	731
ESC_PA1596AA	998	644
ESC_PA1595AA	998	644
ESC_PA1594AA	998	644
ESC_PA1593AA	80	731
ESC_PA1592AA	80	731
ESC_PA1591AA	998	644
ESC_PA1590AA	998	644
ESC_PA1589AA	998	644

ESC_PA1588AA	998	644
ESC_PA1587AA	998	644
ESC_PA1586AA	998	644
ESC_PA1585AA	998	644
ESC_PA1584AA	998	644
ESC_PA1583AA	998	644
ESC_PA1582AA	80	731
ESC_PA1581AA	998	644
ESC_PA1580AA	998	644
ESC_PA1579AA	80	731
ESC_PA1578AA	80	731
ESC_PA1577AA	80	731
ESC_PA1576AA	80	731
ESC_PA1511AA	3021	447
ESC_PA1510AA	10	512
ESC_PA1509AA	73	737
ESC_PA1508AA	131	653
ESC_PA1507AA	73	737
ESC_PA1506AA	131	653
ESC_PA1505AA	95	738
ESC_PA1504AA	70	670
ESC_PA1503AA	351	268
ESC_PA1502AA	1421	447
ESC_PA1501AA	205	257
ESC_PA1500AA	127	738
ESC_PA1498AA	73	737
ESC_PA1497AA	73	737
ESC_PA1496AA	10	512
ESC_PA1495AA	4088	198
ESC_PA1494AA	69	387
ESC_PA1493AA	648	359
ESC_PA1492AA	117	592
ESC_PA1491AA	131	653
ESC_PA1490AA	131	653
ESC_PA1489AA	95	738
ESC_PA1488AA	155	175
ESC_PA1487AA	95	738
ESC_PA1486AA	131	653
ESC_PA1485AA	3640	257
ESC_PA1484AA	95	738
ESC_PA1483AA	131	653
ESC_PA1482AA	38	669
ESC_PA1481AA	131	653
ESC_PA1480AA	131	653
ESC_PA1479AA	131	653
ESC_PA1478AA	224	198
ESC_PA1477AA	95	738
ESC_PA1476AA	617	511
ESC_PA1475AA	542	824
ESC_PA1474AA	95	738

ESC_PA1473AA	226	512
ESC_PA1472AA	349	670
ESC_PA1471AA	95	738
ESC_PA1470AA	131	653
ESC_PA1469AA	3033	737
ESC_PA1468AA	69	387
ESC_PA1467AA	167	512
ESC_PA1466AA	357	654
ESC_PA1465AA	95	738
ESC_PA1464AA	131	653
ESC_PA1463AA	453	935
ESC_PA1462AA	1670	438
ESC_PA1461AA	10	512
ESC_PA1460AA	131	653
ESC_PA1459AA	69	387
ESC_PA1458AA	12	626
ESC_PA1457AA	131	653
ESC_PA1456AA	117	592
ESC_PA1455AA	8442	76
ESC_PA1454AA	46	447
ESC_PA1453AA	38	669
ESC_PA1452AA	95	738
ESC_PA1451AA	95	738
ESC_PA1450AA	69	387
ESC_PA1449AA	131	653
ESC_PA1448AA	457	595
ESC_PA1447AA	224	198
ESC_PA1446AA	95	738
ESC_PA1445AA	1193	761
ESC_PA1444AA	38	669
ESC_PA1443AA	10	512
ESC_PA1442AA	1193	761
ESC_PA1441AA	127	738
ESC_PA1440AA	131	653
ESC_PA1439AA	69	387
ESC_PA1438AA	929	742
ESC_PA1437AA	69	387
ESC_PA1436AA	1193	761
ESC_PA1435AA	131	653
ESC_PA1434AA	10	512
ESC_PA1433AA	117	592
ESC_PA1432AA	131	653
ESC_PA1431AA	95	738
ESC_PA1430AA	648	359
ESC_PA1429AA	95	738
ESC_PA1428AA	59	217
ESC_PA1427AA	131	653
ESC_PA1424AA	8450	764
ESC_PA1423AA	59	217
ESC_PA1422AA	59	217

ESC_PA1421AA	131	653
ESC_PA1419AA	73	737
ESC_PA1418AA	73	737
ESC_PA1415AA	127	738
ESC_PA1414AA	939	198
ESC_PA1413AA	131	653
ESC_PA1412AA	155	175
ESC_PA1411AA	131	653
ESC_PA1410AA	131	653
ESC_PA1409AA	131	653
ESC_PA1408AA	69	387
ESC_PA1407AA	131	653
ESC_PA1406AA	95	738
ESC_PA1405AA	676	737
ESC_PA1404AA	131	653
ESC_PA1403AA	1193	761
ESC_PA1402AA	69	387
ESC_PA1401AA	131	653
ESC_PA1400AA	162	924
ESC_PA1399AA	538	629
ESC_PA1398AA	1491	76
ESC_PA1397AA	683	175
ESC_PA1396AA	12	626
ESC_PA1395AA	38	669
ESC_PA1394AA	131	653
ESC_PA1393AA	131	653
ESC_PA1392AA	131	653
ESC_PA1391AA	38	669
ESC_PA1390AA	95	738
ESC_PA1389AA	23	156
ESC_PA1388AA	95	738
ESC_PA1387AA	2768	198
ESC_PA1386AA	1257	649
ESC_PA1385AA	131	653
ESC_PA1384AA	73	737
ESC_PA1383AA	1193	761
ESC_PA1382AA	69	387
ESC_PA1381AA	127	738
ESC_PA1380AA	38	669
ESC_PA1379AA	69	387
ESC_PA1378AA	131	653
ESC_PA1377AA	8441	47
ESC_PA1376AA	69	387
ESC_PA1375AA	73	737
ESC_PA1374AA	1193	761
ESC_PA1373AA	405	923
ESC_PA1372AA	95	738
ESC_PA1371AA	1193	761
ESC_PA1370AA	131	653
ESC_PA1369AA	1193	761

ESC_PA1368AA	95	738
ESC_PA1367AA	95	738
ESC_PA1366AA	555	653
ESC_PA1365AA	155	175
ESC_PA1364AA	95	738
ESC_PA1363AA	38	669
ESC_PA1362AA	167	512
ESC_PA1361AA	95	738
ESC_PA1360AA	4450	844
ESC_PA1359AA	69	387
ESC_PA1358AA	1193	761
ESC_PA1357AA	8449	475
ESC_PA1356AA	131	653
ESC_PA1355AA	8448	257
ESC_PA1353AA	131	653
ESC_PA1352AA	131	653
ESC_PA1351AA	4358	71
ESC_PA1350AA	69	387
ESC_PA1349AA	95	738
ESC_PA1348AA	38	669
ESC_PA1347AA	95	738
ESC_PA1346AA	69	387
ESC_PA1345AA	8451	669
ESC_PA1344AA	131	653
ESC_PA1343AA	69	387
ESC_PA1342AA	1193	761
ESC_PA1341AA	609	447
ESC_PA1340AA	5308	261
ESC_PA1339AA	127	738
ESC_PA1338AA	38	669
ESC_PA1337AA	69	387
ESC_PA1336AA	62	574
ESC_PA1333AA	131	653
ESC_PA1332AA	131	653
ESC_PA1331AA	117	592
ESC_PA1330AA	73	737
ESC_PA1327AA	388	156
ESC_PA1326AA	12	626
ESC_PA1325AA	38	669
ESC_PA1324AA	69	387
ESC_PA1323AA	69	387
ESC_PA1321AA	69	387
ESC_PA1319AA	73	737
ESC_PA1318AA	95	738
ESC_PA1316AA	12	626
ESC_PA1315AA	134	738
ESC_PA1312AA	683	175
ESC_PA1311AA	580	445
ESC_PA1310AA	297	234
ESC_PA1309AA	127	738

ESC_PA1308AA	69	387
ESC_PA1306AA	2005	175
ESC_PA1304AA	69	387
ESC_PA1302AA	38	669
ESC_PA1301AA	69	387
ESC_PA1300AA	144	740
ESC_PA1299AA	117	592
ESC_PA1298AA	57	50
ESC_PA1296AA	117	592
ESC_PA1295AA	212	198
ESC_PA1294AA	117	592
ESC_PA1293AA	10	512
ESC_PA1290AA	457	595
ESC_PA1289AA	1564	512
ESC_PA1288AA	10	512
ESC_PA1287AA	2079	198
ESC_PA1286AA	48	76
ESC_PA1285AA	4045	592
ESC_PA1284AA	540	286
ESC_PA1283AA	401	142
ESC_PA1282AA	4978	936
ESC_PA1280AA	115	669
ESC_PA1277AA	2040	198
ESC_PA1276AA	69	387
ESC_PA1275AA	4045	592
ESC_PA1274AA	297	234
ESC_PA1273AA	351	268
ESC_PA1272AA	5840	234
ESC_PA1271AA	162	924
ESC_PA1270AA	69	387
ESC_PA1269AA	69	387
ESC_PA1268AA	2025	194
ESC_PA1267AA	57	50
ESC_PA1266AA	155	175
ESC_PA1265AA	355	764
ESC_PA1264AA	38	669
ESC_PA1263AA	117	592
ESC_PA1262AA	3572	512
ESC_PA1261AA	131	653
ESC_PA1260AA	57	50
ESC_PA1259AA	58	198
ESC_PA1258AA	58	198
ESC_PA1255AA	117	592
ESC_PA1254AA	117	592
ESC_PA1253AA	23	156
ESC_PA1252AA	93	71
ESC_PA1251AA	706	422
ESC_PA1246AA	95	738
ESC_PA1245AA	7235	738
ESC_PA1244AA	38	669

ESC_PA1243AA	2556	630
ESC_PA1242AA	38	669
ESC_PA1241AA	95	738
ESC_PA1240AA	95	738
ESC_PA1239AA	1278	797
ESC_PA1236AA	7234	744
ESC_PA1235AA	101	696
ESC_PA1233AA	131	653
ESC_PA1231AA	69	387
ESC_PA1229AA	1193	761
ESC_PA1228AA	1193	761
ESC_PA1227AA	1193	761
ESC_PA1226AA	1193	761
ESC_PA1225AA	1193	761
ESC_PA1224AA	1193	761
ESC_PA1223AA	1193	761
ESC_PA1222AA	1193	761
ESC_PA1221AA	1193	761
ESC_PA1220AA	1193	761
ESC_PA1219AA	14	761
ESC_PA1218AA	1193	761
ESC_PA1217AA	1193	761
ESC_PA1216AA	1193	761
ESC_PA1215AA	1193	761
ESC_PA1214AA	1193	761
ESC_PA1213AA	1193	761
ESC_PA1212AA	1193	761
ESC_PA1211AA	1193	761
ESC_PA1210AA	1193	761
ESC_PA1209AA	1193	761
ESC_PA1208AA	1193	761
ESC_PA1207AA	1193	761
ESC_PA1206AA	1193	761
ESC_PA1205AA	1193	761
ESC_PA1204AA	1193	761
ESC_PA1203AA	1193	761
ESC_PA1202AA	1193	761
ESC_PA1201AA	1193	761
ESC_PA1200AA	1193	761
ESC_PA1199AA	1193	761
ESC_PA1198AA	1193	761
ESC_PA1197AA	404	761
ESC_PA1196AA	1193	761
ESC_PA1195AA	1193	761
ESC_PA1194AA	1193	761
ESC_PA1193AA	1193	761
ESC_PA1192AA	1193	761
ESC_PA1191AA	1193	761
ESC_PA1190AA	1193	761
ESC_PA1189AA	1193	761

ESC_PA1188AA	1193	761
ESC_PA1187AA	1193	761
ESC_PA1186AA	1193	761
ESC_PA1185AA	1193	761
ESC_PA1184AA	1193	761
ESC_PA1183AA	1193	761
ESC_PA1182AA	1193	761
ESC_PA1181AA	1193	761
ESC_PA1180AA	1193	761
ESC_PA1179AA	1193	761
ESC_PA1178AA	550	756
ESC_PA1177AA	1193	761
ESC_PA1176AA	14	761
ESC_PA1175AA	404	761
ESC_PA1174AA	1193	761
ESC_PA1173AA	1193	761
ESC_PA1172AA	14	761
ESC_PA1171AA	1193	761
ESC_PA1170AA	1193	761
ESC_PA1169AA	1193	761
ESC_PA1168AA	404	761
ESC_PA1056AA	540	286
ESC_PA1055AA	117	592
ESC_PA1054AA	73	737
ESC_PA1053AA	73	737
ESC_PA1052AA	73	737
ESC_PA1051AA	73	737
ESC_PA1050AA	5242	743
ESC_PA1049AA	73	737
ESC_PA1048AA	1193	761
ESC_PA1047AA	95	738
ESC_PA1046AA	127	738
ESC_PA1045AA	69	387
ESC_PA1044AA	95	738
ESC_PA1043AA	12	626
ESC_PA1042AA	88	160
ESC_PA1041AA	131	653
ESC_PA1040AA	131	653
ESC_PA1039AA	8197	740
ESC_PA1038AA	95	738
ESC_PA1037AA	95	738
ESC_PA1036AA	131	653
ESC_PA1035AA	95	738
ESC_PA1034AA	73	737
ESC_PA1032AA	349	670
ESC_PA1031AA	91	629
ESC_PA1030AA	95	738
ESC_PA1029AA	73	737
ESC_PA1028AA	73	737
ESC_PA1027AA	62	574

ESC_PA1026AA	69	387
ESC_PA1025AA	131	653
ESC_PA1024AA	73	737
ESC_PA1023AA	372	424
ESC_PA1022AA	8196	653
ESC_PA1021AA	8196	653
ESC_PA1020AA	73	737
ESC_PA1019AA	2003	669
ESC_PA1018AA	38	669
ESC_PA1017AA	73	737
ESC_PA1016AA	62	574
ESC_PA1015AA	38	669
ESC_PA1014AA	1434	512
ESC_PA1013AA	73	737
ESC_PA1012AA	127	738
ESC_PA1011AA	73	737
ESC_PA1010AA	144	740
ESC_PA1009AA	906	198
ESC_PA1008AA	131	653
ESC_PA1006AA	648	359
ESC_PA1005AA	69	387
ESC_PA1004AA	681	393
ESC_PA1003AA	73	737
ESC_PA1002AA	549	387
ESC_PA1001AA	131	653
ESC_PA1000AA	349	670
ESC_PA0999AA	131	653
ESC_PA0998AA	127	738
ESC_PA0997AA	69	387
ESC_PA0996AA	73	737
ESC_PA0995AA	127	738
ESC_PA0994AA	164	175
ESC_PA0993AA	95	738
ESC_PA0992AA	393	343
ESC_PA0991AA	73	737
ESC_PA0990AA	10	512
ESC_PA0989AA	131	653
ESC_PA0988AA	131	653
ESC_PA0987AA	144	740
ESC_PA0986AA	57	50
ESC_PA0985AA	131	653
ESC_PA0984AA	38	669
ESC_PA0983AA	95	738
ESC_PA0982AA	69	387
ESC_PA0981AA	131	653
ESC_PA0980AA	4702	626
ESC_PA0979AA	131	653
ESC_PA0978AA	73	737
ESC_PA0977AA	131	653
ESC_PA0976AA	131	653

ESC_PA0975AA	131	653
ESC_PA0973AA	11	936
ESC_PA0969AA	295	160
ESC_PA0968AA	410	156
ESC_PA0967AA	167	512
ESC_PA0966AA	11	936
ESC_PA0965AA	11	936
ESC_PA0964AA	11	936
ESC_PA0963AA	11	936
ESC_PA0962AA	11	936
ESC_PA0961AA	11	936
ESC_PA0960AA	11	936
ESC_PA0959AA	11	936
ESC_PA0958AA	11	936
ESC_PA0957AA	11	936
ESC_PA0956AA	48	76
ESC_PA0955AA	1244	291
ESC_PA0954AA	10	512
ESC_PA0953AA	57	50
ESC_PA0952AA	10	512
ESC_PA0951AA	8565	512
ESC_PA0950AA	100	504
ESC_PA0949AA	763	878
ESC_PA0948AA	48	76
ESC_PA0947AA	542	824
ESC_PA0946AA	20	198
ESC_PA0945AA	48	76
ESC_PA0944AA	155	175
ESC_PA0943AA	69	387
ESC_PA0942AA	7483	234
ESC_PA0941AA	155	175
ESC_PA0939AA	1727	257
ESC_PA0938AA	44	512
ESC_PA0937AA	540	286
ESC_PA0936AA	469	257
ESC_PA0935AA	6646	917
ESC_PA0934AA	7473	789
ESC_PA0933AA	540	286
ESC_PA0932AA	155	175
ESC_PA0931AA	297	234
ESC_PA0930AA	6	445
ESC_PA0929AA	6	445
ESC_PA0928AA	117	592
ESC_PA0927AA	117	592
ESC_PA0926AA	280	142
ESC_PA0925AA	131	653
ESC_PA0924AA	131	653
ESC_PA0923AA	131	653
ESC_PA0922AA	131	653
ESC_PA0921AA	131	653

ESC_PA0920AA	131	653
ESC_PA0919AA	131	653
ESC_PA0918AA	10472	653
ESC_PA0917AA	131	653
ESC_PA0916AA	131	653
ESC_PA0915AA	517	924
ESC_PA0914AA	131	653
ESC_PA0913AA	131	653
ESC_PA0912AA	131	653
ESC_PA0911AA	131	653
ESC_PA0910AA	38	669
ESC_PA0893AA	155	175
ESC_PA0892AA	11	936
ESC_PA0891AA	11	936
ESC_PA0890AA	11	936
ESC_PA0889AA	11	936
ESC_PA0888AA	11	936
ESC_PA0887AA	11	936
ESC_PA0886AA	11	936
ESC_PA0885AA	11	936
ESC_PA0884AA	11	936
ESC_PA0883AA	11	936
ESC_PA0882AA	11	936
ESC_PA0881AA	11	936
ESC_PA0880AA	11	936
ESC_PA0879AA	11	936
ESC_PA0878AA	11	936
ESC_PA0877AA	11	936
ESC_PA0876AA	11	936
ESC_PA0875AA	11	936
ESC_PA0874AA	11	936
ESC_PA0873AA	11	936
ESC_PA0872AA	11	936
ESC_PA0871AA	11	936
ESC_PA0870AA	11	936
ESC_PA0869AA	11	936
ESC_PA0868AA	11	936
ESC_PA0867AA	11	936
ESC_PA0866AA	11	936
ESC_PA0865AA	410	156
ESC_PA0864AA	6332	153
ESC_PA0863AA	48	76
ESC_PA0862AA	48	76
ESC_PA0861AA	48	76
ESC_PA0860AA	48	76
ESC_PA0859AA	46	447
ESC_PA0858AA	46	447
ESC_PA0857AA	38	669
ESC_PA0856AA	38	669
ESC_PA0855AA	131	653

ESC_PA0854AA	131	653
ESC_PA0853AA	10	512
ESC_PA0852AA	10	512
ESC_PA0851AA	6332	153
ESC_PA0850AA	165	504
ESC_PA0849AA	1284	604
ESC_PA0848AA	1284	604
ESC_PA0847AA	410	156
ESC_PA0846AA	410	156
ESC_PA0845AA	1284	604
ESC_PA0844AA	1284	604
ESC_PA0843AA	38	669
ESC_PA0842AA	38	669
ESC_PA0841AA	38	669
ESC_PA0840AA	38	669
ESC_PA0839AA	940	257
ESC_PA0838AA	940	257
ESC_PA0837AA	361	512
ESC_PA0836AA	361	512
ESC_PA0835AA	617	511
ESC_PA0834AA	617	511
ESC_PA0833AA	6359	473
ESC_PA0832AA	6359	473
ESC_PA0831AA	38	669
ESC_PA0830AA	38	669
ESC_PA0829AA	617	511
ESC_PA0828AA	617	511
ESC_PA0827AA	167	512
ESC_PA0826AA	167	512
ESC_PA0825AA	410	156
ESC_PA0824AA	6332	153
ESC_PA0823AA	602	257
ESC_PA0822AA	165	504
ESC_PA0821AA	410	156
ESC_PA0820AA	602	257
ESC_PA0819AA	38	669
ESC_PA0818AA	38	669
ESC_PA0817AA	10479	447
ESC_PA0816AA	10479	447
ESC_PA0815AA	38	669
ESC_PA0814AA	38	669
ESC_PA0813AA	10	512
ESC_PA0812AA	710	291
ESC_PA0811AA	21	156
ESC_PA0810AA	410	156
ESC_PA0809AA	410	156
ESC_PA0808AA	38	669
ESC_PA0807AA	38	669
ESC_PA0806AA	38	669
ESC_PA0805AA	38	669

ESC_PA0804AA	410	156
ESC_PA0800AA	1060	512
ESC_PA0799AA	1060	512
ESC_PA0798AA	1060	512
ESC_PA0797AA	1060	512
ESC_PA0796AA	155	175
ESC_PA0795AA	155	175
ESC_PA0794AA	155	175
ESC_PA0793AA	155	175
ESC_PA0792AA	155	175
ESC_PA0790AA	2966	936
ESC_PA0789AA	11	936
ESC_PA0788AA	11	936
ESC_PA0787AA	11	936
ESC_PA0786AA	10478	257
ESC_PA0785AA	11	936
ESC_PA0784AA	11	936
ESC_PA0783AA	21	156
ESC_PA0782AA	10477	113
ESC_PA0781AA	5934	257
ESC_PA0780AA	17	191
ESC_PA0779AA	21	156
ESC_PA0778AA	11	936
ESC_PA0777AA	21	156
ESC_PA0776AA	11	936
ESC_PA0775AA	7316	286
ESC_PA0774AA	11	936
ESC_PA0773AA	21	156
ESC_PA0772AA	278	924
ESC_PA0771AA	11	936
ESC_PA0770AA	11	936
ESC_PA0769AA	7019	936
ESC_PA0768AA	21	156
ESC_PA0767AA	11	936
ESC_PA0766AA	7019	936
ESC_PA0763AA	11	936
ESC_PA0762AA	11	936
ESC_PA0761AA	11	936
ESC_PA0760AA	11	936
ESC_PA0759AA	11	936
ESC_PA0758AA	7019	936
ESC_PA0757AA	11	936
ESC_PA0756AA	11	936
ESC_PA0754AA	11	936
ESC_PA0753AA	11	936
ESC_PA0752AA	25	234
ESC_PA0751AA	504	738
ESC_PA0750AA	747	640
ESC_PA0749AA	7019	936
ESC_PA0748AA	11	936

ESC_PA0747AA	11	936
ESC_PA0746AA	11	936
ESC_PA0745AA	11	936
ESC_PA0744AA	21	156
ESC_PA0743AA	29	156
ESC_PA0742AA	21	156
ESC_PA0741AA	21	156
ESC_PA0740AA	11	936
ESC_PA0739AA	11	936
ESC_PA0738AA	10481	909
ESC_PA0737AA	11	936
ESC_PA0736AA	21	156
ESC_PA0735AA	21	156
ESC_PA0734AA	11	936
ESC_PA0733AA	21	156
ESC_PA0732AA	11	936
ESC_PA0731AA	11	936
ESC_PA0730AA	11	936
ESC_PA0729AA	11	936
ESC_PA0728AA	21	156
ESC_PA0727AA	21	156
ESC_PA0726AA	11	936
ESC_PA0725AA	21	156
ESC_PA0724AA	11	936
ESC_PA0723AA	11	936
ESC_PA0722AA	11	936
ESC_PA0721AA	11	936
ESC_PA0720AA	11	936
ESC_PA0719AA	11	936
ESC_PA0718AA	7019	936
ESC_PA0717AA	11	936
ESC_PA0716AA	11	936
ESC_PA0715AA	11	936
ESC_PA0714AA	11	936
ESC_PA0713AA	11	936
ESC_PA0712AA	11	936
ESC_PA0711AA	11	936
ESC_PA0710AA	11	936
ESC_PA0709AA	11	936
ESC_PA0708AA	7019	936
ESC_PA0707AA	10480	936
ESC_PA0706AA	11	936
ESC_PA0705AA	11	936
ESC_PA0704AA	11	936
ESC_PA0703AA	21	156
ESC_PA0702AA	21	156
ESC_PA0701AA	25	234
ESC_PA0700AA	6639	257
ESC_PA0699AA	6639	257
ESC_PA0698AA	278	924

ESC_PA0697AA	388	156
ESC_PA0696AA	6639	257
ESC_PA0695AA	33	198
ESC_PA0694AA	5324	936
ESC_PA0693AA	7019	936
ESC_PA0692AA	11	936
ESC_PA0691AA	21	156
ESC_PA0690AA	21	156
ESC_PA0689AA	11	936
ESC_PA0688AA	11	936
ESC_PA0687AA	11	936
ESC_PA0686AA	5891	878
ESC_PA0685AA	11	936
ESC_PA0684AA	11	936
ESC_PA0683AA	21	156
ESC_PA0682AA	11	936
ESC_PA0681AA	33	198
ESC_PA0680AA	2966	936
ESC_PA0679AA	11	936
ESC_PA0678AA	11	936
ESC_PA0677AA	7019	936
ESC_PA0676AA	11	936
ESC_PA0675AA	11	936
ESC_PA0674AA	11	936
ESC_PA0673AA	11	936
ESC_PA0672AA	11	936
ESC_PA0671AA	11	936
ESC_PA0670AA	11	936
ESC_PA0669AA	11	936
ESC_PA0668AA	21	156
ESC_PA0667AA	11	936
ESC_PA0666AA	11	936
ESC_PA0665AA	11	936
ESC_PA0664AA	7019	936
ESC_PA0663AA	11	936
ESC_PA0662AA	294	156
ESC_PA0661AA	5292	738
ESC_PA0660AA	343	286
ESC_PA0659AA	294	156
ESC_PA0658AA	10484	994
ESC_PA0657AA	10483	899
ESC_PA0656AA	297	234
ESC_PA0655AA	8699	286
ESC_PA0654AA	25	234
ESC_PA0653AA	25	234
ESC_PA0652AA	11	936
ESC_PA0651AA	11	936
ESC_PA0650AA	10485	935
ESC_PA0649AA	11	936
ESC_PA0648AA	11	936

ESC_PA0647AA	11	936
ESC_PA0646AA	7019	936
ESC_PA0645AA	7019	936
ESC_PA0644AA	11	936
ESC_PA0643AA	11	936
ESC_PA0642AA	99	198
ESC_PA0641AA	349	670
ESC_PA0640AA	99	198
ESC_PA0639AA	99	198
ESC_PA0638AA	294	156
ESC_PA0637AA	294	156
ESC_PA0636AA	294	156
ESC_PA0635AA	294	156
ESC_PA0634AA	11	936
ESC_PA0633AA	11	936
ESC_PA0632AA	16	156
ESC_PA0631AA	11	936
ESC_PA0630AA	11	936
ESC_PA0629AA	7019	936
ESC_PA0628AA	11	936
ESC_PA0627AA	11	936
ESC_PA0626AA	7019	936
ESC_PA0625AA	11	936
ESC_PA0624AA	11	936
ESC_PA0623AA	11	936
ESC_PA0622AA	11	936
ESC_PA0621AA	11	936
ESC_PA0620AA	7019	936
ESC_PA0619AA	11	936
ESC_PA0618AA	7019	936
ESC_PA0617AA	11	936
ESC_PA0616AA	504	738
ESC_PA0615AA	25	234
ESC_PA0614AA	223	198
ESC_PA0613AA	25	234
ESC_PA0612AA	25	234
ESC_PA0611AA	25	234
ESC_PA0610AA	25	234
ESC_PA0609AA	25	234
ESC_PA0608AA	25	234
ESC_PA0607AA	25	234
ESC_PA0606AA	25	234
ESC_PA0605AA	306	286
ESC_PA0604AA	11	936
ESC_PA0603AA	25	234
ESC_PA0602AA	29	156
ESC_PA0601AA	33	198
ESC_PA0600AA	504	738
ESC_PA0599AA	7260	257
ESC_PA0598AA	11	936

ESC_PA0597AA	11	936
ESC_PA0596AA	11	936
ESC_PA0595AA	11	936
ESC_PA0594AA	443	257
ESC_PA0593AA	504	738
ESC_PA0592AA	11	936
ESC_PA0591AA	11	936
ESC_PA0590AA	154	257
ESC_PA0589AA	2101	108
ESC_PA0588AA	21	156
ESC_PA0587AA	21	156
ESC_PA0586AA	1723	900
ESC_PA0585AA	8699	286
ESC_PA0584AA	447	198
ESC_PA0583AA	99	198
ESC_PA0582AA	11	936
ESC_PA0581AA	11	936
ESC_PA0580AA	11	936
ESC_PA0579AA	11	936
ESC_PA0578AA	11	936
ESC_PA0577AA	7019	936
ESC_PA0576AA	11	936
ESC_PA0575AA	11	936
ESC_PA0574AA	11	936
ESC_PA0573AA	11	936
ESC_PA0572AA	21	156
ESC_PA0571AA	21	156
ESC_PA0570AA	21	156
ESC_PA0569AA	21	156
ESC_PA0568AA	21	156
ESC_PA0567AA	5429	156
ESC_PA0566AA	21	156
ESC_PA0565AA	21	156
ESC_PA0564AA	10486	156
ESC_PA0563AA	21	156
ESC_PA0562AA	11	936
ESC_PA0561AA	11	936
ESC_PA0560AA	11	936
ESC_PA0559AA	11	936
ESC_PA0558AA	99	198
ESC_PA0557AA	29	156
ESC_PA0556AA	11	936
ESC_PA0555AA	294	156
ESC_PA0554AA	29	156
ESC_PA0553AA	21	156
ESC_PA0552AA	33	198
ESC_PA0551AA	33	198
ESC_PA0550AA	679	257
ESC_PA0549AA	679	257
ESC_PA0548AA	25	234

ESC_PA0547AA	25	234
ESC_PA0546AA	33	198
ESC_PA0545AA	25	234
ESC_PA0544AA	655	926
ESC_PA0543AA	2161	198
ESC_PA0542AA	294	156
ESC_PA0541AA	294	156
ESC_PA0540AA	294	156
ESC_PA0539AA	294	156
ESC_PA0538AA	16	156
ESC_PA0537AA	294	156
ESC_PA0536AA	294	156
ESC_PA0535AA	7019	936
ESC_PA0534AA	294	156
ESC_PA0533AA	294	156
ESC_PA0532AA	11	936
ESC_PA0531AA	11	936
ESC_PA0530AA	11	936
ESC_PA0529AA	11	936
ESC_PA0528AA	11	936
ESC_PA0527AA	11	936
ESC_PA0526AA	11	936
ESC_PA0525AA	11	936
ESC_PA0524AA	11	936
ESC_PA0523AA	11	936
ESC_PA0522AA	11	936
ESC_PA0521AA	11	936
ESC_PA0520AA	11	936
ESC_PA0519AA	11	936
ESC_PA0518AA	11	936
ESC_PA0517AA	11	936
ESC_PA0516AA	11	936
ESC_PA0515AA	11	936
ESC_PA0514AA	297	234
ESC_PA0513AA	11	936
ESC_PA0512AA	21	156
ESC_PA0511AA	342	286
ESC_PA0510AA	11	936
ESC_PA0509AA	11	936
ESC_PA0508AA	11	936
ESC_PA0507AA	11	936
ESC_PA0506AA	7282	270
ESC_PA0505AA	6274	257
ESC_PA0504AA	349	670
ESC_PA0503AA	11	936
ESC_PA0502AA	11	936
ESC_PA0501AA	7019	936
ESC_PA0500AA	11	936
ESC_PA0499AA	223	198
ESC_PA0498AA	11	936

ESC_PA0497AA	33	198
ESC_PA0496AA	294	156
ESC_PA0495AA	294	156
ESC_PA0494AA	294	156
ESC_PA0493AA	11	936
ESC_PA0492AA	11	936
ESC_PA0491AA	11	936
ESC_PA0490AA	11	936
ESC_PA0489AA	11	936
ESC_PA0488AA	11	936
ESC_PA0487AA	294	156
ESC_PA0486AA	294	156
ESC_PA0485AA	294	156
ESC_PA0484AA	294	156
ESC_PA0483AA	294	156
ESC_PA0482AA	16	156
ESC_PA0481AA	294	156
ESC_PA0480AA	294	156
ESC_PA0479AA	11	936
ESC_PA0478AA	11	936
ESC_PA0477AA	11	936
ESC_PA0476AA	11	936
ESC_PA0475AA	11	936
ESC_PA0474AA	11	936
ESC_PA0473AA	11	936
ESC_PA0472AA	11	936
ESC_PA0471AA	11	936
ESC_PA0470AA	11	936
ESC_PA0469AA	11	936
ESC_PA0468AA	11	936
ESC_PA0467AA	11	936
ESC_PA0466AA	11	936
ESC_PA0465AA	11	936
ESC_PA0464AA	11	936
ESC_PA0463AA	11	936
ESC_PA0462AA	11	936
ESC_PA0461AA	11	936
ESC_PA0460AA	11	936
ESC_PA0459AA	11	936
ESC_PA0458AA	11	936
ESC_PA0457AA	11	936
ESC_PA0456AA	527	286
ESC_PA0455AA	11	936
ESC_PA0454AA	11	936
ESC_PA0453AA	11	936
ESC_PA0452AA	11	936
ESC_PA0451AA	11	936
ESC_PA0450AA	11	936
ESC_PA0449AA	11	936
ESC_PA0448AA	11	936

ESC_PA0447AA	11	936
ESC_PA0446AA	11	936
ESC_PA0445AA	11	936
ESC_PA0444AA	11	936
ESC_PA0443AA	11	936
ESC_PA0442AA	11	936
ESC_PA0441AA	223	198
ESC_PA0440AA	10	512
ESC_PA0439AA	7316	286
ESC_PA0438AA	119	286
ESC_PA0437AA	7316	286
ESC_PA0436AA	7316	286
ESC_PA0435AA	21	156
ESC_PA0434AA	21	156
ESC_PA0433AA	21	156
ESC_PA0432AA	21	156
ESC_PA0431AA	11	936
ESC_PA0430AA	11	936
ESC_PA0429AA	11	936
ESC_PA0428AA	11	936
ESC_PA0427AA	11	936
ESC_PA0426AA	11	936
ESC_PA0425AA	11	936
ESC_PA0424AA	11	936
ESC_PA0423AA	11	936
ESC_PA0422AA	11	936
ESC_PA0421AA	11	936
ESC_PA0420AA	11	936
ESC_PA0419AA	11	936
ESC_PA0418AA	11	936
ESC_PA0417AA	11	936
ESC_PA0416AA	11	936
ESC_PA0415AA	11	936
ESC_PA0414AA	11	936
ESC_PA0413AA	11	936
ESC_PA0412AA	11	936
ESC_PA0411AA	11	936
ESC_PA0410AA	11	936
ESC_PA0409AA	11	936
ESC_PA0408AA	11	936
ESC_PA0407AA	7019	936
ESC_PA0406AA	7019	936
ESC_PA0405AA	2966	936
ESC_PA0404AA	7019	936
ESC_PA0403AA	11	936
ESC_PA0402AA	11	936
ESC_PA0401AA	11	936
ESC_PA0400AA	7019	936
ESC_PA0399AA	11	936
ESC_PA0398AA	10488	936

ESC_PA0397AA	11	936
ESC_PA0396AA	11	936
ESC_PA0395AA	11	936
ESC_PA0394AA	2966	936
ESC_PA0393AA	11	936
ESC_PA0392AA	11	936
ESC_PA0391AA	11	936
ESC_PA0390AA	10478	257
ESC_PA0389AA	10478	257
ESC_PA0388AA	2389	160
ESC_PA0387AA	10487	160
ESC_PA0386AA	9860	234
ESC_PA0385AA	297	234
ESC_PA0384AA	297	234
ESC_PA0383AA	297	234
ESC_PA0382AA	7019	936
ESC_PA0381AA	11	936
ESC_PA0380AA	11	936
ESC_PA0379AA	11	936
ESC_PA0378AA	11	936
ESC_PA0377AA	2966	936
ESC_PA0376AA	675	234
ESC_PA0375AA	675	234
ESC_PA0374AA	21	156
ESC_PA0373AA	2966	936
ESC_PA0372AA	11	936
ESC_PA0371AA	21	156
ESC_PA0370AA	21	156
ESC_PA0369AA	21	156
ESC_PA0368AA	659	286
ESC_PA0367AA	21	156
ESC_PA0366AA	21	156
ESC_PA0365AA	29	156
ESC_PA0364AA	29	156
ESC_PA0363AA	504	738
ESC_PA0362AA	504	738
ESC_PA0361AA	504	738
ESC_PA0360AA	10	512
ESC_PA0359AA	659	286
ESC_PA0358AA	659	286
ESC_PA0357AA	504	738
ESC_PA0356AA	504	738
ESC_PA0355AA	11	936
ESC_PA0354AA	7019	936
ESC_PA0353AA	3759	175
ESC_PA0352AA	33	198
ESC_PA0351AA	173	254
ESC_PA0350AA	2914	670
ESC_PA0349AA	7032	212
ESC_PA0348AA	69	387

ESC_PA0347AA	58	198
ESC_PA0346AA	33	198
ESC_PA0345AA	33	198
ESC_PA0344AA	11	936
ESC_PA0343AA	11	936
ESC_PA0342AA	11	936
ESC_PA0341AA	11	936
ESC_PA0340AA	10490	234
ESC_PA0339AA	25	234
ESC_PA0338AA	111	175
ESC_PA0337AA	10489	234
ESC_PA0336AA	11	936
ESC_PA0335AA	11	936
ESC_PA0334AA	11	936
ESC_PA0333AA	11	936
ESC_PA0332AA	11	936
ESC_PA0331AA	11	936
ESC_PA0330AA	442	257
ESC_PA0329AA	25	234
ESC_PA0328AA	388	156
ESC_PA0327AA	388	156
ESC_PA0326AA	543	842
ESC_PA0325AA	4532	234
ESC_PA0324AA	335	936
ESC_PA0323AA	504	738
ESC_PA0322AA	675	234
ESC_PA0321AA	335	936
ESC_PA0320AA	11	936
ESC_PA0319AA	11	936
ESC_PA0318AA	11	936
ESC_PA0317AA	11	936
ESC_PA0316AA	11	936
ESC_PA0315AA	7019	936
ESC_PA0314AA	11	936
ESC_PA0313AA	11	936
ESC_PA0312AA	11	936
ESC_PA0311AA	11	936
ESC_PA0310AA	11	936
ESC_PA0309AA	11	936
ESC_PA0308AA	11	936
ESC_PA0307AA	11	936
ESC_PA0306AA	16	156
ESC_PA0305AA	212	198
ESC_PA0302AA	173	254
ESC_PA0293AA	23	156
ESC_PA0292AA	940	257
ESC_PA0291AA	10502	512
ESC_PA0290AA	2353	76
ESC_PA0289AA	5305	404
ESC_PA0288AA	10503	111

ESC_PA0287AA	4	76
ESC_PA0286AA	10501	458
ESC_PA0285AA	5587	257
ESC_PA0284AA	58	198
ESC_PA0279AA	2332	601
ESC_PA0278AA	5392	156
ESC_PA0277AA	4	76
ESC_PA0276AA	23	156
ESC_PA0275AA	46	447
ESC_PA0274AA	2332	601
ESC_PA0273AA	2353	76
ESC_PA0272AA	23	156
ESC_PA0271AA	23	156
ESC_PA0270AA	3864	204
ESC_PA0269AA	2332	601
ESC_PA0268AA	1564	512
ESC_PA0267AA	5305	404
ESC_PA0266AA	1564	512
ESC_PA0265AA	10504	156
ESC_PA0264AA	2332	601
ESC_PA0262AA	5305	404
ESC_PA0261AA	3075	701
ESC_PA0260AA	4	76
ESC_PA0259AA	4	76
ESC_PA0258AA	173	254
ESC_PA0257AA	4	76
ESC_PA0256AA	155	175
ESC_PA0255AA	23	156
ESC_PA0254AA	23	156
ESC_PA0253AA	23	156
ESC_PA0252AA	3864	204
ESC_PA0251AA	23	156
ESC_PA0250AA	443	257
ESC_PA0249AA	10505	257
ESC_PA0248AA	10	512
ESC_PA0247AA	2332	601
ESC_PA0246AA	4	76
ESC_PA0244AA	998	644
ESC_PA0243AA	38	669
ESC_PA0242AA	501	900
ESC_PA0241AA	131	653
ESC_PA0240AA	998	644
ESC_PA0239AA	73	737
ESC_PA0238AA	131	653
ESC_PA0237AA	131	653
ESC_PA0236AA	69	387
ESC_PA0235AA	69	387
ESC_PA0234AA	73	737
ESC_PA0233AA	131	653
ESC_PA0232AA	2279	657

ESC_PA0231AA	3601	653
ESC_PA0210AA	10	512
ESC_PA0209AA	10	512
ESC_PA0208AA	10	512
ESC_PA0204AA	10	512
ESC_PA0168AA	10491	923
ESC_PA0167AA	131	653
ESC_PA0166AA	131	653
ESC_PA0165AA	131	653
ESC_PA0164AA	131	653
ESC_PA0163AA	131	653
ESC_PA0162AA	131	653
ESC_PA0161AA	131	653
ESC_PA0160AA	405	923
ESC_PA0159AA	405	923
ESC_PA0158AA	405	923
ESC_PA0156AA	12	626
ESC_PA0155AA	405	923
ESC_PA0154AA	12	626
ESC_PA0153AA	131	653
ESC_PA0152AA	131	653
ESC_PA0151AA	131	653
ESC_PA0150AA	131	653
ESC_PA0149AA	131	653
ESC_PA0148AA	69	387
ESC_PA0147AA	131	653
ESC_PA0146AA	131	653
ESC_PA0145AA	131	653
ESC_PA0144AA	131	653
ESC_PA0143AA	131	653
ESC_PA0142AA	131	653
ESC_PA0141AA	131	653
ESC_PA0140AA	131	653
ESC_PA0139AA	131	653
ESC_PA0138AA	131	653
ESC_PA0137AA	131	653
ESC_PA0136AA	131	653
ESC_PA0135AA	131	653
ESC_PA0134AA	131	653
ESC_PA0133AA	131	653
ESC_PA0132AA	131	653
ESC_PA0131AA	131	653
ESC_PA0130AA	131	653
ESC_PA0129AA	131	653
ESC_PA0128AA	131	653
ESC_PA0127AA	131	653
ESC_PA0126AA	405	923
ESC_PA0125AA	131	653
ESC_PA0124AA	131	653
ESC_PA0123AA	131	653

ESC_PA0122AA	131	653
ESC_PA0121AA	131	653
ESC_PA0120AA	131	653
ESC_PA0119AA	131	653
ESC_PA0118AA	131	653
ESC_PA0117AA	131	653
ESC_PA0116AA	131	653
ESC_PA0115AA	131	653
ESC_PA0114AA	131	653
ESC_PA0113AA	131	653
ESC_PA0112AA	131	653
ESC_PA0111AA	131	653
ESC_PA0109AA	131	653
ESC_PA0108AA	131	653
ESC_PA0106AA	131	653
ESC_PA0105AA	131	653
ESC_PA0104AA	131	653
ESC_PA0103AA	131	653
ESC_PA0102AA	131	653
ESC_PA0101AA	131	653
ESC_PA0100AA	131	653
ESC_PA0099AA	131	653
ESC_PA0098AA	131	653
ESC_PA0097AA	131	653
ESC_PA0096AA	131	653
ESC_PA0095AA	131	653
ESC_PA0094AA	131	653
ESC_PA0093AA	131	653
ESC_PA0092AA	131	653
ESC_PA0091AA	131	653
ESC_PA0090AA	131	653
ESC_PA0089AA	131	653
ESC_PA0088AA	131	653
ESC_PA0087AA	131	653
ESC_PA0086AA	131	653
ESC_PA0085AA	131	653
ESC_PA0084AA	131	653
ESC_PA0083AA	131	653
ESC_PA0082AA	131	653
ESC_PA0081AA	131	653
ESC_PA0080AA	131	653
ESC_PA0079AA	131	653
ESC_PA0078AA	131	653
ESC_PA0077AA	131	653
ESC_PA0076AA	131	653
ESC_PA0075AA	131	653
ESC_PA0074AA	131	653
ESC_PA0073AA	131	653
ESC_PA0072AA	457	595
ESC_PA0071AA	410	156

ESC_PA0070AA	648	359
ESC_PA0069AA	617	511
ESC_PA0068AA	410	156
ESC_PA0067AA	131	653
ESC_PA0064AA	7100	924
ESC_PA0063AA	58	198
ESC_PA0062AA	2476	198
ESC_PA0061AA	58	198
ESC_PA0060AA	648	359
ESC_PA0059AA	10	512
ESC_PA0058AA	10496	291
ESC_PA0057AA	154	257
ESC_PA0056AA	10	512
ESC_PA0055AA	1079	235
ESC_PA0054AA	2111	184
ESC_PA0053AA	10495	512
ESC_PA0052AA	10	512
ESC_PA0051AA	847	257
ESC_PA0050AA	540	286
ESC_PA0049AA	117	592
ESC_PA0048AA	58	198
ESC_PA0047AA	10	512
ESC_PA0046AA	1423	198
ESC_PA0045AA	7233	814
ESC_PA0044AA	56	198
ESC_PA0043AA	164	175
ESC_PA0042AA	1286	474
ESC_PA0041AA	58	198
ESC_PA0040AA	58	198
ESC_PA0039AA	131	653
ESC_PA0038AA	48	76
ESC_PA0037AA	10	512
ESC_PA0036AA	10	512
ESC_PA0035AA	88	160
ESC_PA0034AA	906	198
ESC_PA0033AA	10	512
ESC_PA0032AA	17	191
ESC_PA0031AA	17	191
ESC_PA0030AA	10492	783
ESC_PA0029AA	10	512
ESC_PA0028AA	399	198
ESC_PA0027AA	73	737
ESC_PA0026AA	349	670
ESC_PA0025AA	349	670
ESC_PA0024AA	349	670
ESC_PA0023AA	349	670
ESC_PA0022AA	349	670
ESC_PA0021AA	349	670
ESC_PA0020AA	349	670
ESC_PA0019AA	349	670

ESC_PA0018AA	29	156
ESC_PA0017AA	100	504
ESC_PA0016AA	93	71
ESC_PA0015AA	398	445
ESC_PA0014AA	10	512
ESC_PA0013AA	5786	513
ESC_PA0012AA	10	512
ESC_PA0011AA	156	175
ESC_PA0010AA	7323	513
ESC_PA0009AA	7323	513
ESC_PA0008AA	10	512
ESC_PA0007AA	4247	936
ESC_PA0006AA	100	504
ESC_PA0005AA	1	667
ESC_PA0004AA	10493	257
ESC_PA0001AA	10	512
ESC_PA0000AA	10	512
ESC_OA9999AA	10	512
ESC_OA9998AA	10	512
ESC_OA9997AA	10	512
ESC_OA9996AA	10	512
ESC_OA9995AA	10	512
ESC_OA9994AA	10	512
ESC_OA9993AA	10	512
ESC_OA9992AA	10	512
ESC_OA9991AA	10	512
ESC_OA9990AA	10	512
ESC_OA9989AA	42	257
ESC_OA9988AA	42	257
ESC_OA9987AA	1	667
ESC_OA9986AA	10	512
ESC_OA9985AA	10	512
ESC_OA9984AA	10	512
ESC_OA9983AA	10	512
ESC_OA9980AA	10	512
ESC_OA9979AA	95	738
ESC_OA9978AA	351	268
ESC_OA9977AA	11	936
ESC_OA9975AA	11	936
ESC_OA9973AA	388	156
ESC_OA9972AA	723	156
ESC_OA9970AA	641	935
ESC_OA9969AA	11	936
ESC_OA9968AA	11	936
ESC_OA9966AA	1712	512
ESC_OA9965AA	10	512
ESC_OA9964AA	10	512
ESC_OA9963AA	10	512
ESC_OA9962AA	10	512
ESC_OA9961AA	10	512

ESC_OA9960AA	10	512
ESC_OA9959AA	10	512
ESC_OA9957AA	10	512
ESC_OA9956AA	10	512
ESC_OA9955AA	10	512
ESC_OA9953AA	10	512
ESC_OA9952AA	10	512
ESC_OA9950AA	10	512
ESC_OA9949AA	10	512
ESC_OA9948AA	10	512
ESC_OA9947AA	10	512
ESC_OA9946AA	10	512
ESC_OA9945AA	10	512
ESC_OA9943AA	16	156
ESC_OA9939AA	21	156
ESC_OA9938AA	17	191
ESC_OA9937AA	442	257
ESC_OA9936AA	1819	808
ESC_OA9935AA	21	156
ESC_OA9934AA	11	936
ESC_OA9933AA	21	156
ESC_OA9932AA	11	936
ESC_OA9931AA	33	198
ESC_OA9930AA	278	924
ESC_OA9929AA	410	156
ESC_OA9927AA	10	512
ESC_OA9922AA	10	512
ESC_OA9921AA	602	257
ESC_OA9920AA	10	512
ESC_OA9919AA	10	512
ESC_OA9918AA	10	512
ESC_OA9917AA	10	512
ESC_OA9916AA	10	512
ESC_OA9915AA	10	512
ESC_OA9914AA	10	512
ESC_OA9909AA	152	257
ESC_OA9908AA	152	257
ESC_OA9907AA	152	257
ESC_OA9906AA	7006	936
ESC_OA9905AA	278	924
ESC_OA9904AA	941	537
ESC_OA9903AA	25	234
ESC_OA9902AA	17	191
ESC_OA9901AA	342	286
ESC_OA9900AA	25	234
ESC_OA9899AA	21	156
ESC_OA9898AA	442	257
ESC_OA9897AA	442	257
ESC_OA9896AA	10	512
ESC_OA9895AA	32	270

ESC_OA9894AA	300	286
ESC_OA9893AA	10458	257
ESC_OA9892AA	738	598
ESC_OA9891AA	442	257
ESC_OA9890AA	21	156
ESC_OA9889AA	33	198
ESC_OA9888AA	48	76
ESC_OA9886AA	6872	601
ESC_OA9885AA	11	936
ESC_OA9884AA	11	936
ESC_OA9883AA	7445	512
ESC_OA9882AA	88	160
ESC_OA9881AA	9699	198
ESC_OA9880AA	101	696
ESC_OA9879AA	10	512
ESC_OA9878AA	1615	156
ESC_OA9877AA	10	512
ESC_OA9876AA	540	286
ESC_OA9875AA	75	257
ESC_OA9874AA	10	512
ESC_OA9873AA	9562	198
ESC_OA9872AA	10	512
ESC_OA9871AA	58	198
ESC_OA9870AA	410	156
ESC_OA9869AA	457	595
ESC_OA9868AA	1193	761
ESC_OA9867AA	501	900
ESC_OA9866AA	73	737
ESC_OA9865AA	95	738
ESC_OA9864AA	131	653
ESC_OA9863AA	131	653
ESC_OA9862AA	8183	654
ESC_OA9861AA	38	669
ESC_OA9860AA	410	156
ESC_OA9859AA	295	160
ESC_OA9858AA	131	653
ESC_OA9857AA	131	653
ESC_OA9856AA	450	71
ESC_OA9855AA	141	644
ESC_OA9854AA	131	653
ESC_OA9853AA	131	653
ESC_OA9852AA	131	653
ESC_OA9851AA	131	653
ESC_OA9850AA	58	198
ESC_OA9849AA	405	923
ESC_OA9848AA	457	595
ESC_OA9847AA	69	387
ESC_OA9846AA	38	669
ESC_OA9845AA	38	669
ESC_OA9844AA	95	738

ESC_OA9843AA	131	653
ESC_OA9842AA	1494	180
ESC_OA9841AA	141	644
ESC_OA9840AA	131	653
ESC_OA9839AA	1193	761
ESC_OA9838AA	131	653
ESC_OA9837AA	131	653
ESC_OA9836AA	131	653
ESC_OA9835AA	34	504
ESC_OA9834AA	405	923
ESC_OA9833AA	38	669
ESC_OA9832AA	131	653
ESC_OA9831AA	1431	257
ESC_OA9830AA	636	653
ESC_OA9829AA	117	592
ESC_OA9828AA	131	653
ESC_OA9827AA	131	653
ESC_OA9826AA	1193	761
ESC_OA9825AA	1193	761
ESC_OA9824AA	404	761
ESC_OA9823AA	8188	653
ESC_OA9822AA	10	512
ESC_OA9821AA	131	653
ESC_OA9820AA	131	653
ESC_OA9819AA	131	653
ESC_OA9818AA	38	669
ESC_OA9817AA	8187	669
ESC_OA9816AA	1193	761
ESC_OA9815AA	131	653
ESC_OA9814AA	95	738
ESC_OA9813AA	1196	257
ESC_OA9812AA	127	738
ESC_OA9811AA	1147	198
ESC_OA9810AA	1193	761
ESC_OA9809AA	131	653
ESC_OA9808AA	131	653
ESC_OA9807AA	131	653
ESC_OA9806AA	131	653
ESC_OA9805AA	2197	511
ESC_OA9804AA	131	653
ESC_OA9803AA	131	653
ESC_OA9802AA	38	669
ESC_OA9801AA	38	669
ESC_OA9800AA	131	653
ESC_OA9799AA	131	653
ESC_OA9798AA	167	512
ESC_OA9797AA	58	198
ESC_OA9796AA	442	257
ESC_OA9795AA	131	653
ESC_OA9794AA	8189	653

ESC_OA9793AA	131	653
ESC_OA9792AA	405	923
ESC_OA9791AA	131	653
ESC_OA9790AA	131	653
ESC_OA9789AA	38	669
ESC_OA9788AA	1193	761
ESC_OA9787AA	1193	761
ESC_OA9786AA	131	653
ESC_OA9785AA	1193	761
ESC_OA9784AA	90	156
ESC_OA9783AA	1193	761
ESC_OA9782AA	10	512
ESC_OA9781AA	1193	761
ESC_OA9780AA	131	653
ESC_OA9779AA	38	669
ESC_OA9778AA	224	198
ESC_OA9777AA	5420	71
ESC_OA9776AA	131	653
ESC_OA9775AA	131	653
ESC_OA9774AA	38	669
ESC_OA9773AA	131	653
ESC_OA9772AA	69	387
ESC_OA9771AA	12	626
ESC_OA9770AA	131	653
ESC_OA9769AA	10	512
ESC_OA9768AA	58	198
ESC_OA9767AA	2301	512
ESC_OA9766AA	69	387
ESC_OA9765AA	131	653
ESC_OA9764AA	1057	761
ESC_OA9763AA	69	387
ESC_OA9762AA	648	359
ESC_OA9761AA	131	653
ESC_OA9760AA	131	653
ESC_OA9759AA	131	653
ESC_OA9758AA	131	653
ESC_OA9757AA	131	653
ESC_OA9756AA	131	653
ESC_OA9755AA	131	653
ESC_OA9754AA	131	653
ESC_OA9753AA	131	653
ESC_OA9752AA	131	653
ESC_OA9751AA	131	653
ESC_OA9750AA	131	653
ESC_OA9749AA	2279	657
ESC_OA9748AA	131	653
ESC_OA9747AA	38	669
ESC_OA9746AA	224	198
ESC_OA9745AA	135	653
ESC_OA9744AA	38	669

ESC_OA9743AA	500	71
ESC_OA9742AA	10	512
ESC_OA9741AA	372	424
ESC_OA9740AA	1193	761
ESC_OA9739AA	410	156
ESC_OA9738AA	131	653
ESC_OA9737AA	38	669
ESC_OA9736AA	34	504
ESC_OA9735AA	95	738
ESC_OA9734AA	38	669
ESC_OA9733AA	155	175
ESC_OA9732AA	1193	761
ESC_OA9731AA	131	653
ESC_OA9730AA	131	653
ESC_OA9729AA	744	512
ESC_OA9728AA	648	359
ESC_OA9727AA	648	359
ESC_OA9726AA	131	653
ESC_OA9725AA	131	653
ESC_OA9724AA	7973	286
ESC_OA9723AA	93	71
ESC_OA9722AA	10	512
ESC_OA9721AA	7973	286
ESC_OA9720AA	10	512
ESC_OA9719AA	162	924
ESC_OA9718AA	162	924
ESC_OA9717AA	93	71
ESC_OA9716AA	224	198
ESC_OA9715AA	1589	156
ESC_OA9714AA	1140	934
ESC_OA9713AA	756	257
ESC_OA9712AA	10	512
ESC_OA9711AA	1385	653
ESC_OA9710AA	1167	257
ESC_OA9709AA	744	512
ESC_OA9708AA	410	156
ESC_OA9707AA	93	71
ESC_OA9706AA	746	789
ESC_OA9705AA	744	512
ESC_OA9704AA	93	71
ESC_OA9703AA	744	512
ESC_OA9702AA	410	156
ESC_OA9701AA	1196	257
ESC_OA9700AA	354	15
ESC_OA9699AA	58	198
ESC_OA9698AA	1081	198
ESC_OA9697AA	69	387
ESC_OA9696AA	5956	71
ESC_OA9695AA	453	935
ESC_OA9694AA	162	924

ESC_OA9693AA	744	512
ESC_OA9692AA	10	512
ESC_OA9691AA	10	512
ESC_OA9690AA	1011	198
ESC_OA9689AA	1011	198
ESC_OA9688AA	58	198
ESC_OA9687AA	58	198
ESC_OA9686AA	1011	198
ESC_OA9685AA	86	935
ESC_OA9684AA	156	175
ESC_OA9683AA	1079	235
ESC_OA9682AA	7233	814
ESC_OA9681AA	354	15
ESC_OA9680AA	156	175
ESC_OA9679AA	349	670
ESC_OA9678AA	7233	814
ESC_OA9676AA	1463	263
ESC_OA9675AA	2179	924
ESC_OA9674AA	1582	198
ESC_OA9673AA	1589	156
ESC_OA9672AA	707	257
ESC_OA9671AA	1196	257
ESC_OA9670AA	131	653
ESC_OA9669AA	28	537
ESC_OA9668AA	131	653
ESC_OA9667AA	73	737
ESC_OA9666AA	393	343
ESC_OA9665AA	2020	387
ESC_OA9664AA	38	669
ESC_OA9663AA	38	669
ESC_OA9662AA	162	924
ESC_OA9661AA	12	626
ESC_OA9660AA	12	626
ESC_OA9659AA	410	156
ESC_OA9658AA	224	198
ESC_OA9657AA	127	738
ESC_OA9656AA	59	217
ESC_OA9655AA	73	737
ESC_OA9654AA	131	653
ESC_OA9653AA	127	738
ESC_OA9652AA	394	400
ESC_OA9651AA	537	756
ESC_OA9650AA	95	738
ESC_OA9649AA	131	653
ESC_OA9648AA	1248	156
ESC_OA9647AA	131	653
ESC_OA9646AA	73	737
ESC_OA9645AA	1993	646
ESC_OA9644AA	73	737
ESC_OA9643AA	428	654

ESC_OA9642AA	73	737
ESC_OA9641AA	73	737
ESC_OA9640AA	131	653
ESC_OA9639AA	80	731
ESC_OA9638AA	127	738
ESC_OA9637AA	131	653
ESC_OA9635AA	95	738
ESC_OA9634AA	108	257
ESC_OA9633AA	453	935
ESC_OA9632AA	69	387
ESC_OA9631AA	127	738
ESC_OA9630AA	4702	626
ESC_OA9629AA	131	653
ESC_OA9628AA	963	669
ESC_OA9627AA	73	737
ESC_OA9626AA	131	653
ESC_OA9625AA	131	653
ESC_OA9624AA	681	393
ESC_OA9623AA	73	737
ESC_OA9622AA	69	387
ESC_OA9621AA	1615	156
ESC_OA9620AA	10	512
ESC_OA9619AA	38	669
ESC_OA9618AA	38	669
ESC_OA9617AA	1057	761
ESC_OA9616AA	69	387
ESC_OA9615AA	8184	653
ESC_OA9614AA	38	669
ESC_OA9613AA	131	653
ESC_OA9612AA	1091	512
ESC_OA9611AA	69	387
ESC_OA9610AA	131	653
ESC_OA9609AA	141	644
ESC_OA9608AA	404	761
ESC_OA9607AA	69	387
ESC_OA9606AA	131	653
ESC_OA9605AA	69	387
ESC_OA9604AA	410	156
ESC_OA9603AA	3631	71
ESC_OA9602AA	131	653
ESC_OA9601AA	410	156
ESC_OA9600AA	131	653
ESC_OA9599AA	357	654
ESC_OA9598AA	131	653
ESC_OA9597AA	131	653
ESC_OA9596AA	162	924
ESC_OA9595AA	131	653
ESC_OA9594AA	127	738
ESC_OA9593AA	131	653
ESC_OA9592AA	648	359

ESC_OA9591AA	38	669
ESC_OA9590AA	131	653
ESC_OA9589AA	209	512
ESC_OA9588AA	501	900
ESC_OA9587AA	131	653
ESC_OA9586AA	38	669
ESC_OA9585AA	43	512
ESC_OA9584AA	131	653
ESC_OA9583AA	162	924
ESC_OA9582AA	744	512
ESC_OA9581AA	394	400
ESC_OA9580AA	131	653
ESC_OA9579AA	1279	160
ESC_OA9578AA	405	923
ESC_OA9577AA	10497	669
ESC_OA9576AA	2003	669
ESC_OA9575AA	131	653
ESC_OA9574AA	744	512
ESC_OA9573AA	636	653
ESC_OA9572AA	131	653
ESC_OA9571AA	34	504
ESC_OA9570AA	95	738
ESC_OA9569AA	131	653
ESC_OA9568AA	131	653
ESC_OA9567AA	648	359
ESC_OA9566AA	8181	175
ESC_OA9565AA	6215	962
ESC_OA9564AA	38	669
ESC_OA9563AA	10	512
ESC_OA9562AA	131	653
ESC_OA9561AA	38	669
ESC_OA9560AA	405	923
ESC_OA9559AA	38	669
ESC_OA9558AA	1193	761
ESC_OA9557AA	131	653
ESC_OA9556AA	131	653
ESC_OA9555AA	10	512
ESC_OA9554AA	6355	625
ESC_OA9553AA	648	359
ESC_OA9552AA	58	198
ESC_OA9551AA	88	160
ESC_OA9550AA	88	160
ESC_OA9549AA	131	653
ESC_OA9548AA	131	653
ESC_OA9547AA	1193	761
ESC_OA9544AA	243	197
ESC_OA9543AA	1753	261
ESC_OA9540AA	243	197
ESC_OA9539AA	1753	261
ESC_OA9537AA	243	197

ESC_OA9536AA	145	256
ESC_OA9535AA	145	256
ESC_OA9534AA	243	197
ESC_OA9533AA	145	256
ESC_OA9532AA	243	197
ESC_OA9531AA	243	197
ESC_OA9530AA	145	256
ESC_OA9529AA	145	256
ESC_OA9526AA	145	256
ESC_OA9524AA	145	256
ESC_OA9523AA	245	261
ESC_OA9522AA	1753	261
ESC_OA9521AA	7364	191
ESC_OA9519AA	145	256
ESC_OA9518AA	152	257
ESC_OA9517AA	10	512
ESC_OA9516AA	152	257
ESC_OA9515AA	144	740
ESC_OA9514AA	7027	817
ESC_OA9513AA	73	737
ESC_OA9512AA	131	653
ESC_OA9511AA	73	737
ESC_OA9510AA	73	737
ESC_OA9509AA	413	121
ESC_OA9508AA	148	191
ESC_OA9507AA	648	359
ESC_OA9506AA	59	217
ESC_OA9505AA	73	737
ESC_OA9504AA	59	217
ESC_OA9503AA	6199	217
ESC_OA9502AA	504	738
ESC_OA9501AA	4419	463
ESC_OA9500AA	5592	830
ESC_OA9499AA	2067	257
ESC_OA9498AA	7368	511
ESC_OA9497AA	1819	808
ESC_OA9496AA	6317	743
ESC_OA9495AA	59	217
ESC_OA9494AA	10	512
ESC_OA9493AA	10448	377
ESC_OA9491AA	4419	463
ESC_OA9490AA	152	257
ESC_OA9489AA	152	257
ESC_OA9488AA	152	257
ESC_OA9487AA	152	257
ESC_OA9486AA	152	257
ESC_OA9485AA	152	257
ESC_OA9484AA	152	257
ESC_OA9483AA	152	257
ESC_OA9482AA	152	257

ESC_OA9481AA	152	257
ESC_OA9480AA	152	257
ESC_OA9479AA	152	257
ESC_OA9478AA	152	257
ESC_OA9477AA	152	257
ESC_OA9476AA	152	257
ESC_OA9475AA	152	257
ESC_OA9474AA	152	257
ESC_OA9473AA	152	257
ESC_OA9472AA	152	257
ESC_OA9471AA	152	257
ESC_OA9470AA	152	257
ESC_OA9469AA	152	257
ESC_OA9468AA	152	257
ESC_OA9467AA	152	257
ESC_OA9466AA	152	257
ESC_OA9465AA	152	257
ESC_OA9464AA	152	257
ESC_OA9463AA	152	257
ESC_OA9462AA	152	257
ESC_OA9461AA	152	257
ESC_OA9460AA	152	257
ESC_OA9459AA	152	257
ESC_OA9458AA	152	257
ESC_OA9457AA	152	257
ESC_OA9456AA	152	257
ESC_OA9455AA	152	257
ESC_OA9454AA	152	257
ESC_OA9453AA	152	257
ESC_OA9452AA	152	257
ESC_OA9451AA	152	257
ESC_OA9450AA	152	257
ESC_OA9449AA	152	257
ESC_OA9448AA	152	257
ESC_OA9447AA	152	257
ESC_OA9446AA	152	257
ESC_OA9445AA	152	257
ESC_OA9444AA	152	257
ESC_OA9443AA	152	257
ESC_OA9442AA	152	257
ESC_OA9441AA	152	257
ESC_OA9440AA	152	257
ESC_OA9439AA	152	257
ESC_OA9438AA	152	257
ESC_OA9437AA	152	257
ESC_OA9436AA	152	257
ESC_OA9435AA	152	257
ESC_OA9434AA	152	257
ESC_OA9433AA	152	257
ESC_OA9432AA	152	257

ESC_OA9431AA	152	257
ESC_OA9430AA	152	257
ESC_OA9429AA	152	257
ESC_OA9428AA	152	257
ESC_OA9427AA	152	257
ESC_OA9426AA	152	257
ESC_OA9425AA	152	257
ESC_OA9424AA	152	257
ESC_OA9423AA	152	257
ESC_OA9422AA	152	257
ESC_OA9421AA	152	257
ESC_OA9420AA	152	257
ESC_OA9419AA	152	257
ESC_OA9418AA	152	257
ESC_OA9417AA	152	257
ESC_OA9416AA	152	257
ESC_OA9415AA	152	257
ESC_OA9414AA	152	257
ESC_OA9413AA	152	257
ESC_OA9412AA	152	257
ESC_OA9411AA	152	257
ESC_OA9410AA	152	257
ESC_OA9409AA	152	257
ESC_OA9408AA	152	257
ESC_OA9407AA	152	257
ESC_OA9406AA	152	257
ESC_OA9405AA	152	257
ESC_OA9404AA	152	257
ESC_OA9403AA	152	257
ESC_OA9402AA	152	257
ESC_OA9401AA	152	257
ESC_OA9400AA	152	257
ESC_OA9399AA	152	257
ESC_OA9398AA	152	257
ESC_OA9397AA	152	257
ESC_OA9396AA	152	257
ESC_OA9395AA	152	257
ESC_OA9394AA	152	257
ESC_OA9393AA	152	257
ESC_OA9392AA	152	257
ESC_OA9391AA	152	257
ESC_OA9390AA	152	257
ESC_OA9389AA	152	257
ESC_OA9388AA	152	257
ESC_OA9387AA	152	257
ESC_OA9386AA	152	257
ESC_OA9385AA	152	257
ESC_OA9384AA	152	257
ESC_OA9383AA	152	257
ESC_OA9382AA	152	257

ESC_OA9381AA	152	257
ESC_OA9380AA	152	257
ESC_OA9379AA	152	257
ESC_OA9378AA	152	257
ESC_OA9377AA	152	257
ESC_OA9376AA	152	257
ESC_OA9375AA	152	257
ESC_OA9374AA	152	257
ESC_OA9373AA	152	257
ESC_OA9372AA	152	257
ESC_OA9371AA	152	257
ESC_OA9370AA	152	257
ESC_OA9369AA	152	257
ESC_OA9368AA	152	257
ESC_OA9367AA	152	257
ESC_OA9366AA	152	257
ESC_OA9365AA	152	257
ESC_OA9364AA	152	257
ESC_OA9363AA	152	257
ESC_OA9362AA	152	257
ESC_OA9361AA	152	257
ESC_OA9360AA	152	257
ESC_OA9359AA	152	257
ESC_OA9358AA	152	257
ESC_OA9357AA	152	257
ESC_OA9356AA	152	257
ESC_OA9355AA	152	257
ESC_OA9354AA	152	257
ESC_OA9353AA	152	257
ESC_OA9352AA	152	257
ESC_OA9351AA	152	257
ESC_OA9350AA	152	257
ESC_OA9349AA	152	257
ESC_OA9348AA	152	257
ESC_OA9347AA	152	257
ESC_OA9346AA	152	257
ESC_OA9345AA	152	257
ESC_OA9344AA	152	257
ESC_OA9343AA	152	257
ESC_OA9342AA	152	257
ESC_OA9341AA	152	257
ESC_OA9340AA	152	257
ESC_OA9339AA	152	257
ESC_OA9338AA	152	257
ESC_OA9337AA	152	257
ESC_OA9336AA	152	257
ESC_OA9335AA	152	257
ESC_OA9334AA	152	257
ESC_OA9333AA	152	257
ESC_OA9332AA	152	257

ESC_OA9331AA	152	257
ESC_OA9330AA	152	257
ESC_OA9329AA	152	257
ESC_OA9328AA	152	257
ESC_OA9327AA	152	257
ESC_OA9326AA	152	257
ESC_OA9325AA	152	257
ESC_OA9324AA	152	257
ESC_OA9323AA	152	257
ESC_OA9322AA	152	257
ESC_OA9321AA	152	257
ESC_OA9320AA	152	257
ESC_OA9319AA	152	257
ESC_OA9318AA	152	257
ESC_OA9317AA	152	257
ESC_OA9316AA	152	257
ESC_OA9315AA	152	257
ESC_OA9314AA	152	257
ESC_OA9313AA	152	257
ESC_OA9312AA	152	257
ESC_OA9311AA	152	257
ESC_OA9310AA	152	257
ESC_OA9309AA	152	257
ESC_OA9308AA	152	257
ESC_OA9307AA	152	257
ESC_OA9306AA	152	257
ESC_OA9305AA	152	257
ESC_OA9304AA	152	257
ESC_OA9303AA	152	257
ESC_OA9302AA	152	257
ESC_OA9301AA	152	257
ESC_OA9300AA	152	257
ESC_OA9299AA	152	257
ESC_OA9298AA	152	257
ESC_OA9297AA	152	257
ESC_OA9296AA	152	257
ESC_OA9295AA	152	257
ESC_OA9294AA	152	257
ESC_OA9293AA	152	257
ESC_OA9292AA	152	257
ESC_OA9291AA	152	257
ESC_OA9290AA	152	257
ESC_OA9289AA	152	257
ESC_OA9288AA	1750	102
ESC_OA9287AA	148	191
ESC_OA9286AA	7379	190
ESC_OA9285AA	145	256
ESC_OA9284AA	152	257
ESC_OA9283AA	245	261
ESC_OA9282AA	152	257

ESC_OA9281AA	245	261
ESC_OA9280AA	147	102
ESC_OA9279AA	152	257
ESC_OA9278AA	245	261
ESC_OA9277AA	152	257
ESC_OA9276AA	152	257
ESC_OA9275AA	245	261
ESC_OA9274AA	152	257
ESC_OA9273AA	152	257
ESC_OA9272AA	152	257
ESC_OA9271AA	245	261
ESC_OA9270AA	289	924
ESC_OA9269AA	257	102
ESC_OA9268AA	628	261
ESC_OA9267AA	245	261
ESC_OA9266AA	152	257
ESC_OA9265AA	152	257
ESC_OA9264AA	152	257
ESC_OA9263AA	152	257
ESC_OA9262AA	152	257
ESC_OA9261AA	152	257
ESC_OA9260AA	152	257
ESC_OA9259AA	1503	257
ESC_OA9258AA	152	257
ESC_OA9257AA	152	257
ESC_OA9256AA	152	257
ESC_OA9255AA	152	257
ESC_OA9254AA	152	257
ESC_OA9253AA	152	257
ESC_OA9252AA	152	257
ESC_OA9251AA	152	257
ESC_OA9250AA	152	257
ESC_OA9249AA	152	257
ESC_OA9248AA	1503	257
ESC_OA9247AA	152	257
ESC_OA9246AA	152	257
ESC_OA9245AA	152	257
ESC_OA9244AA	152	257
ESC_OA9243AA	152	257
ESC_OA9242AA	152	257
ESC_OA9241AA	152	257
ESC_OA9240AA	152	257
ESC_OA9239AA	152	257
ESC_OA9238AA	152	257
ESC_OA9237AA	152	257
ESC_OA9236AA	152	257
ESC_OA9235AA	152	257
ESC_OA9234AA	152	257
ESC_OA9233AA	152	257
ESC_OA9232AA	152	257

ESC_OA9231AA	152	257
ESC_OA9230AA	152	257
ESC_OA9229AA	152	257
ESC_OA9228AA	152	257
ESC_OA9227AA	152	257
ESC_OA9226AA	245	261
ESC_OA9225AA	245	261
ESC_OA9224AA	245	261
ESC_OA9223AA	245	261
ESC_OA9222AA	245	261
ESC_OA9221AA	245	261
ESC_OA9220AA	245	261
ESC_OA9219AA	245	261
ESC_OA9218AA	145	256
ESC_OA9217AA	245	261
ESC_OA9216AA	152	257
ESC_OA9215AA	152	257
ESC_OA9214AA	152	257
ESC_OA9213AA	152	257
ESC_OA9212AA	152	257
ESC_OA9211AA	152	257
ESC_OA9210AA	152	257
ESC_OA9209AA	152	257
ESC_OA9208AA	152	257
ESC_OA9207AA	152	257
ESC_OA9206AA	245	261
ESC_OA9205AA	245	261
ESC_OA9204AA	245	261
ESC_OA9203AA	245	261
ESC_OA9202AA	245	261
ESC_OA9201AA	245	261
ESC_OA9200AA	245	261
ESC_OA9199AA	245	261
ESC_OA9198AA	245	261
ESC_OA9197AA	245	261
ESC_OA9196AA	1504	254
ESC_OA9195AA	1504	254
ESC_OA9194AA	152	257
ESC_OA9193AA	152	257
ESC_OA9192AA	152	257
ESC_OA9191AA	152	257
ESC_OA9190AA	152	257
ESC_OA9189AA	152	257
ESC_OA9188AA	152	257
ESC_OA9187AA	152	257
ESC_OA9186AA	152	257
ESC_OA9185AA	152	257
ESC_OA9184AA	152	257
ESC_OA9183AA	152	257
ESC_OA9182AA	152	257

ESC_OA9181AA	152	257
ESC_OA9180AA	152	257
ESC_OA9179AA	152	257
ESC_OA9178AA	152	257
ESC_OA9177AA	152	257
ESC_OA9176AA	152	257
ESC_OA9175AA	152	257
ESC_OA9174AA	152	257
ESC_OA9173AA	152	257
ESC_OA9172AA	152	257
ESC_OA9171AA	152	257
ESC_OA9170AA	152	257
ESC_OA9169AA	152	257
ESC_OA9168AA	152	257
ESC_OA9167AA	152	257
ESC_OA9166AA	152	257
ESC_OA9165AA	152	257
ESC_OA9164AA	152	257
ESC_OA9163AA	152	257
ESC_OA9162AA	1503	257
ESC_OA9161AA	152	257
ESC_OA9160AA	152	257
ESC_OA9159AA	152	257
ESC_OA9158AA	152	257
ESC_OA9157AA	152	257
ESC_OA9156AA	152	257
ESC_OA9155AA	152	257
ESC_OA9154AA	152	257
ESC_OA9153AA	152	257
ESC_OA9152AA	10449	257
ESC_OA9151AA	152	257
ESC_OA9150AA	152	257
ESC_OA9149AA	152	257
ESC_OA9148AA	152	257
ESC_OA9147AA	152	257
ESC_OA9146AA	152	257
ESC_OA9145AA	152	257
ESC_OA9144AA	152	257
ESC_OA9143AA	152	257
ESC_OA9142AA	152	257
ESC_OA9141AA	152	257
ESC_OA9140AA	1025	261
ESC_OA9139AA	245	261
ESC_OA9138AA	245	261
ESC_OA9137AA	1025	261
ESC_OA9136AA	245	261
ESC_OA9135AA	245	261
ESC_OA9134AA	245	261
ESC_OA9133AA	245	261
ESC_OA9132AA	245	261

ESC_OA9131AA	245	261
ESC_OA9130AA	245	261
ESC_OA9129AA	245	261
ESC_OA9128AA	245	261
ESC_OA9127AA	145	256
ESC_OA9126AA	245	261
ESC_OA9125AA	245	261
ESC_OA9124AA	245	261
ESC_OA9123AA	245	261
ESC_OA9122AA	245	261
ESC_OA9120AA	245	261
ESC_OA9119AA	152	257
ESC_OA9118AA	152	257
ESC_OA9117AA	152	257
ESC_OA9116AA	152	257
ESC_OA9115AA	152	257
ESC_OA9114AA	152	257
ESC_OA9113AA	152	257
ESC_OA9112AA	152	257
ESC_OA9111AA	152	257
ESC_OA9110AA	152	257
ESC_OA9109AA	152	257
ESC_OA9108AA	152	257
ESC_OA9107AA	152	257
ESC_OA9106AA	152	257
ESC_OA9105AA	152	257
ESC_OA9104AA	152	257
ESC_OA9103AA	1503	257
ESC_OA9102AA	152	257
ESC_OA9101AA	1503	257
ESC_OA9100AA	152	257
ESC_OA9099AA	152	257
ESC_OA9098AA	152	257
ESC_OA9097AA	152	257
ESC_OA9096AA	152	257
ESC_OA9095AA	152	257
ESC_OA9094AA	152	257
ESC_OA9093AA	152	257
ESC_OA9092AA	152	257
ESC_OA9091AA	152	257
ESC_OA9090AA	152	257
ESC_OA9089AA	152	257
ESC_OA9088AA	152	257
ESC_OA9087AA	152	257
ESC_OA9086AA	152	257
ESC_OA9085AA	1503	257
ESC_OA9084AA	152	257
ESC_OA9083AA	152	257
ESC_OA9082AA	152	257
ESC_OA9081AA	152	257

ESC_OA9080AA	152	257
ESC_OA9079AA	152	257
ESC_OA9078AA	152	257
ESC_OA9077AA	152	257
ESC_OA9076AA	152	257
ESC_OA9075AA	152	257
ESC_OA9074AA	152	257
ESC_OA9073AA	152	257
ESC_OA9072AA	152	257
ESC_OA9071AA	152	257
ESC_OA9070AA	152	257
ESC_OA9069AA	152	257
ESC_OA9068AA	1025	261
ESC_OA9067AA	245	261
ESC_OA9066AA	245	261
ESC_OA9063AA	245	261
ESC_OA9062AA	245	261
ESC_OA9061AA	245	261
ESC_OA9060AA	245	261
ESC_OA9059AA	245	261
ESC_OA9058AA	245	261
ESC_OA9057AA	245	261
ESC_OA9056AA	245	261
ESC_OA9055AA	245	261
ESC_OA9054AA	245	261
ESC_OA9053AA	10450	261
ESC_OA9052AA	245	261
ESC_OA9051AA	630	261
ESC_OA9050AA	245	261
ESC_OA9049AA	245	261
ESC_OA9048AA	245	261
ESC_OA9047AA	245	261
ESC_OA9046AA	245	261
ESC_OA9045AA	245	261
ESC_OA9044AA	245	261
ESC_OA9043AA	152	257
ESC_OA9042AA	1025	261
ESC_OA9041AA	245	261
ESC_OA9040AA	245	261
ESC_OA9039AA	245	261
ESC_OA9038AA	245	261
ESC_OA9037AA	245	261
ESC_OA9035AA	245	261
ESC_OA9034AA	245	261
ESC_OA9033AA	245	261
ESC_OA9031AA	245	261
ESC_OA9029AA	245	261
ESC_OA9028AA	152	257
ESC_OA9027AA	152	257
ESC_OA9026AA	152	257

ESC_OA9025AA	152	257
ESC_OA9024AA	152	257
ESC_OA9023AA	1504	254
ESC_OA9022AA	152	257
ESC_OA9021AA	152	257
ESC_OA9020AA	152	257
ESC_OA9019AA	152	257
ESC_OA9018AA	152	257
ESC_OA9017AA	152	257
ESC_OA9016AA	152	257
ESC_OA9015AA	5517	257
ESC_OA9014AA	152	257
ESC_OA9013AA	152	257
ESC_OA9012AA	152	257
ESC_OA9011AA	152	257
ESC_OA9010AA	152	257
ESC_OA9009AA	152	257
ESC_OA9008AA	152	257
ESC_OA9007AA	152	257
ESC_OA9006AA	152	257
ESC_OA9005AA	152	257
ESC_OA9004AA	152	257
ESC_OA9003AA	152	257
ESC_OA9002AA	152	257
ESC_OA9001AA	152	257
ESC_OA9000AA	152	257
ESC_OA8999AA	152	257
ESC_OA8998AA	152	257
ESC_OA8997AA	152	257
ESC_OA8996AA	152	257
ESC_OA8995AA	152	257
ESC_OA8994AA	152	257
ESC_OA8993AA	152	257
ESC_OA8992AA	152	257
ESC_OA8991AA	152	257
ESC_OA8990AA	152	257
ESC_OA8989AA	152	257
ESC_OA8988AA	152	257
ESC_OA8987AA	152	257
ESC_OA8986AA	152	257
ESC_OA8985AA	152	257
ESC_OA8984AA	152	257
ESC_OA8983AA	152	257
ESC_OA8982AA	152	257
ESC_OA8981AA	145	256
ESC_OA8980AA	1025	261
ESC_OA8979AA	245	261
ESC_OA8978AA	245	261
ESC_OA8973AA	145	256
ESC_OA8972AA	245	261

ESC_OA8971AA	245	261
ESC_OA8970AA	245	261
ESC_OA8969AA	245	261
ESC_OA8968AA	245	261
ESC_OA8967AA	245	261
ESC_OA8966AA	245	261
ESC_OA8965AA	245	261
ESC_OA8964AA	245	261
ESC_OA8963AA	630	261
ESC_OA8962AA	628	261
ESC_OA8961AA	245	261
ESC_OA8960AA	245	261
ESC_OA8959AA	245	261
ESC_OA8958AA	245	261
ESC_OA8957AA	245	261
ESC_OA8956AA	245	261
ESC_OA8955AA	245	261
ESC_OA8954AA	245	261
ESC_OA8953AA	245	261
ESC_OA8951AA	245	261
ESC_OA8950AA	245	261
ESC_OA8949AA	245	261
ESC_OA8948AA	245	261
ESC_OA8947AA	245	261
ESC_OA8946AA	245	261
ESC_OA8945AA	245	261
ESC_OA8944AA	245	261
ESC_OA8943AA	245	261
ESC_OA8942AA	145	256
ESC_OA8941AA	152	257
ESC_OA8940AA	152	257
ESC_OA8939AA	152	257
ESC_OA8938AA	152	257
ESC_OA8937AA	152	257
ESC_OA8936AA	152	257
ESC_OA8935AA	152	257
ESC_OA8934AA	152	257
ESC_OA8933AA	152	257
ESC_OA8932AA	152	257
ESC_OA8931AA	152	257
ESC_OA8930AA	152	257
ESC_OA8929AA	152	257
ESC_OA8928AA	152	257
ESC_OA8927AA	152	257
ESC_OA8926AA	152	257
ESC_OA8925AA	152	257
ESC_OA8924AA	152	257
ESC_OA8923AA	152	257
ESC_OA8922AA	152	257
ESC_OA8921AA	152	257

ESC_OA8920AA	152	257
ESC_OA8919AA	152	257
ESC_OA8918AA	152	257
ESC_OA8917AA	152	257
ESC_OA8916AA	152	257
ESC_OA8915AA	152	257
ESC_OA8914AA	152	257
ESC_OA8913AA	152	257
ESC_OA8912AA	152	257
ESC_OA8911AA	152	257
ESC_OA8910AA	152	257
ESC_OA8909AA	152	257
ESC_OA8908AA	152	257
ESC_OA8907AA	152	257
ESC_OA8906AA	152	257
ESC_OA8905AA	152	257
ESC_OA8904AA	152	257
ESC_OA8903AA	152	257
ESC_OA8902AA	152	257
ESC_OA8901AA	152	257
ESC_OA8900AA	152	257
ESC_OA8899AA	152	257
ESC_OA8898AA	152	257
ESC_OA8897AA	152	257
ESC_OA8896AA	152	257
ESC_OA8895AA	152	257
ESC_OA8894AA	152	257
ESC_OA8893AA	152	257
ESC_OA8892AA	152	257
ESC_OA8891AA	152	257
ESC_OA8890AA	152	257
ESC_OA8889AA	1502	257
ESC_OA8888AA	152	257
ESC_OA8887AA	245	261
ESC_OA8886AA	245	261
ESC_OA8885AA	245	261
ESC_OA8884AA	245	261
ESC_OA8883AA	245	261
ESC_OA8882AA	245	261
ESC_OA8881AA	10452	261
ESC_OA8880AA	245	261
ESC_OA8879AA	245	261
ESC_OA8878AA	245	261
ESC_OA8877AA	245	261
ESC_OA8876AA	245	261
ESC_OA8875AA	245	261
ESC_OA8874AA	1025	261
ESC_OA8873AA	245	261
ESC_OA8872AA	245	261
ESC_OA8871AA	1025	261

ESC_OA8868AA	145	256
ESC_OA8867AA	245	261
ESC_OA8866AA	245	261
ESC_OA8865AA	245	261
ESC_OA8863AA	245	261
ESC_OA8861AA	1025	261
ESC_OA8860AA	245	261
ESC_OA8859AA	245	261
ESC_OA8858AA	245	261
ESC_OA8857AA	152	257
ESC_OA8856AA	152	257
ESC_OA8855AA	152	257
ESC_OA8851AA	152	257
ESC_OA8850AA	152	257
ESC_OA8849AA	152	257
ESC_OA8848AA	152	257
ESC_OA8847AA	152	257
ESC_OA8846AA	152	257
ESC_OA8845AA	152	257
ESC_OA8844AA	152	257
ESC_OA8843AA	152	257
ESC_OA8842AA	152	257
ESC_OA8841AA	152	257
ESC_OA8840AA	152	257
ESC_OA8839AA	152	257
ESC_OA8838AA	1025	261
ESC_OA8837AA	152	257
ESC_OA8836AA	152	257
ESC_OA8835AA	152	257
ESC_OA8834AA	152	257
ESC_OA8833AA	152	257
ESC_OA8832AA	152	257
ESC_OA8831AA	152	257
ESC_OA8829AA	152	257
ESC_OA8828AA	152	257
ESC_OA8827AA	152	257
ESC_OA8826AA	152	257
ESC_OA8825AA	152	257
ESC_OA8824AA	152	257
ESC_OA8823AA	152	257
ESC_OA8822AA	152	257
ESC_OA8821AA	152	257
ESC_OA8820AA	152	257
ESC_OA8819AA	152	257
ESC_OA8818AA	152	257
ESC_OA8817AA	245	261
ESC_OA8815AA	245	261
ESC_OA8813AA	245	261
ESC_OA8812AA	628	261
ESC_OA8811AA	245	261

ESC_OA8809AA	245	261
ESC_OA8807AA	152	257
ESC_OA8806AA	152	257
ESC_OA8805AA	1503	257
ESC_OA8804AA	152	257
ESC_OA8803AA	152	257
ESC_OA8802AA	152	257
ESC_OA8801AA	152	257
ESC_OA8800AA	152	257
ESC_OA8799AA	152	257
ESC_OA8798AA	152	257
ESC_OA8797AA	1021	261
ESC_OA8796AA	245	261
ESC_OA8795AA	245	261
ESC_OA8794AA	245	261
ESC_OA8793AA	245	261
ESC_OA8792AA	245	261
ESC_OA8791AA	630	261
ESC_OA8790AA	145	256
ESC_OA8789AA	145	256
ESC_OA8788AA	245	261
ESC_OA8787AA	152	257
ESC_OA8786AA	152	257
ESC_OA8785AA	152	257
ESC_OA8784AA	152	257
ESC_OA8783AA	152	257
ESC_OA8782AA	1503	257
ESC_OA8781AA	152	257
ESC_OA8780AA	152	257
ESC_OA8779AA	152	257
ESC_OA8778AA	152	257
ESC_OA8777AA	152	257
ESC_OA8776AA	152	257
ESC_OA8775AA	152	257
ESC_OA8774AA	152	257
ESC_OA8773AA	152	257
ESC_OA8772AA	152	257
ESC_OA8771AA	152	257
ESC_OA8770AA	152	257
ESC_OA8769AA	152	257
ESC_OA8768AA	152	257
ESC_OA8767AA	152	257
ESC_OA8766AA	152	257
ESC_OA8765AA	152	257
ESC_OA8764AA	152	257
ESC_OA8763AA	152	257
ESC_OA8762AA	152	257
ESC_OA8761AA	152	257
ESC_OA8760AA	152	257
ESC_OA8759AA	152	257

ESC_OA8758AA	152	257
ESC_OA8757AA	152	257
ESC_OA8756AA	152	257
ESC_OA8755AA	152	257
ESC_OA8754AA	152	257
ESC_OA8753AA	152	257
ESC_OA8752AA	152	257
ESC_OA8751AA	152	257
ESC_OA8750AA	245	261
ESC_OA8748AA	152	257
ESC_OA8747AA	152	257
ESC_OA8746AA	152	257
ESC_OA8745AA	152	257
ESC_OA8744AA	152	257
ESC_OA8743AA	152	257
ESC_OA8742AA	152	257
ESC_OA8741AA	152	257
ESC_OA8740AA	152	257
ESC_OA8739AA	152	257
ESC_OA8738AA	152	257
ESC_OA8737AA	152	257
ESC_OA8736AA	152	257
ESC_OA8735AA	152	257
ESC_OA8732AA	245	261
ESC_OA8731AA	245	261
ESC_OA8730AA	1025	261
ESC_OA8729AA	152	257
ESC_OA8728AA	245	261
ESC_OA8727AA	145	256
ESC_OA8726AA	245	261
ESC_OA8725AA	245	261
ESC_OA8724AA	152	257
ESC_OA8723AA	152	257
ESC_OA8722AA	152	257
ESC_OA8721AA	1025	261
ESC_OA8720AA	243	197
ESC_OA8719AA	152	257
ESC_OA8718AA	1130	31
ESC_OA8717AA	1753	261
ESC_OA8716AA	252	190
ESC_OA8715AA	148	191
ESC_OA8714AA	152	257
ESC_OA8713AA	245	261
ESC_OA8712AA	245	261
ESC_OA8711AA	145	256
ESC_OA8710AA	245	261
ESC_OA8709AA	245	261
ESC_OA8708AA	245	261
ESC_OA8707AA	290	662
ESC_OA8706AA	148	191

ESC_OA8705AA	245	261
ESC_OA8704AA	152	257
ESC_OA8703AA	1273	256
ESC_OA8702AA	273	102
ESC_OA8701AA	148	191
ESC_OA8700AA	145	256
ESC_OA8699AA	1273	256
ESC_OA8698AA	243	197
ESC_OA8697AA	288	102
ESC_OA8696AA	148	191
ESC_OA8694AA	1759	102
ESC_OA8693AA	252	190
ESC_OA8692AA	152	257
ESC_OA8691AA	1025	261
ESC_OA8690AA	243	197
ESC_OA8689AA	243	197
ESC_OA8688AA	152	257
ESC_OA8687AA	148	191
ESC_OA8686AA	245	261
ESC_OA8685AA	245	261
ESC_OA8684AA	630	261
ESC_OA8683AA	147	102
ESC_OA8682AA	1130	31
ESC_OA8681AA	243	197
ESC_OA8680AA	147	102
ESC_OA8679AA	257	102
ESC_OA8678AA	243	197
ESC_OA8677AA	146	897
ESC_OA8676AA	252	190
ESC_OA8675AA	243	197
ESC_OA8674AA	250	102
ESC_OA8673AA	5613	102
ESC_OA8672AA	152	257
ESC_OA8671AA	145	256
ESC_OA8670AA	145	256
ESC_OA8669AA	10454	185
ESC_OA8668AA	243	197
ESC_OA8667AA	245	261
ESC_OA8664AA	245	261
ESC_OA8663AA	1743	197
ESC_OA8662AA	1753	261
ESC_OA8661AA	152	257
ESC_OA8660AA	152	257
ESC_OA8659AA	245	261
ESC_OA8658AA	1767	928
ESC_OA8657AA	250	102
ESC_OA8656AA	152	257
ESC_OA8655AA	152	257
ESC_OA8654AA	243	197
ESC_OA8653AA	245	261

ESC_OA8652AA	289	924
ESC_OA8651AA	243	197
ESC_OA8650AA	145	256
ESC_OA8649AA	10453	197
ESC_OA8648AA	1739	188
ESC_OA8647AA	1766	102
ESC_OA8646AA	152	257
ESC_OA8645AA	243	197
ESC_OA8644AA	147	102
ESC_OA8643AA	145	256
ESC_OA8642AA	243	197
ESC_OA8641AA	152	257
ESC_OA8640AA	145	256
ESC_OA8639AA	245	261
ESC_OA8638AA	148	191
ESC_OA8637AA	145	256
ESC_OA8636AA	245	261
ESC_OA8635AA	7248	31
ESC_OA8634AA	245	261
ESC_OA8633AA	1024	261
ESC_OA8632AA	245	261
ESC_OA8631AA	145	256
ESC_OA8630AA	245	261
ESC_OA8629AA	630	261
ESC_OA8628AA	1753	261
ESC_OA8627AA	149	102
ESC_OA8626AA	148	191
ESC_OA8625AA	243	197
ESC_OA8624AA	10453	197
ESC_OA8623AA	243	197
ESC_OA8622AA	1767	928
ESC_OA8621AA	1765	102
ESC_OA8620AA	250	102
ESC_OA8619AA	1273	256
ESC_OA8618AA	273	102
ESC_OA8617AA	243	197
ESC_OA8616AA	628	261
ESC_OA8615AA	152	257
ESC_OA8614AA	1273	256
ESC_OA8613AA	145	256
ESC_OA8612AA	245	261
ESC_OA8611AA	243	197
ESC_OA8610AA	7364	191
ESC_OA8609AA	145	256
ESC_OA8608AA	152	257
ESC_OA8607AA	243	197
ESC_OA8606AA	273	102
ESC_OA8605AA	148	191
ESC_OA8604AA	243	197
ESC_OA8603AA	289	924

ESC_OA8602AA	10457	257
ESC_OA8601AA	289	924
ESC_OA8600AA	152	257
ESC_OA8599AA	152	257
ESC_OA8598AA	243	197
ESC_OA8597AA	1749	95
ESC_OA8596AA	148	191
ESC_OA8595AA	252	190
ESC_OA8594AA	260	865
ESC_OA8593AA	273	102
ESC_OA8592AA	10455	190
ESC_OA8591AA	10456	190
ESC_OA8590AA	252	190
ESC_OA8589AA	243	197
ESC_OA8588AA	253	190
ESC_OA8587AA	148	191
ESC_OA8586AA	243	197
ESC_OA8585AA	5619	102
ESC_OA8584AA	243	197
ESC_OA8583AA	152	257
ESC_OA8582AA	245	261
ESC_OA8581AA	245	261
ESC_OA8580AA	258	190
ESC_OA8579AA	10455	190
ESC_OA8578AA	253	190
ESC_OA8577AA	145	256
ESC_OA8576AA	243	197
ESC_OA8575AA	147	102
ESC_OA8574AA	146	897
ESC_OA8573AA	245	261
ESC_OA8572AA	9853	261
ESC_OA8571AA	34	504
ESC_OA8570AA	398	445
ESC_OA8569AA	21	156
ESC_OA8568AA	491	626
ESC_OA8567AA	245	261
ESC_OA8566AA	245	261
ESC_OA8565AA	245	261
ESC_OA8564AA	152	257
ESC_OA8563AA	152	257
ESC_OA8562AA	152	257
ESC_OA8561AA	245	261
ESC_OA8560AA	152	257
ESC_OA8559AA	252	190
ESC_OA8558AA	1503	257
ESC_OA8557AA	152	257
ESC_OA8556AA	1722	599
ESC_OA8555AA	10445	175
ESC_OA8552AA	101	696
ESC_OA8551AA	38	669

ESC_OA8550AA	1294	512
ESC_OA8549AA	1312	76
ESC_OA8548AA	131	653
ESC_OA8547AA	38	669
ESC_OA8546AA	131	653
ESC_OA8544AA	131	653
ESC_OA8543AA	131	653
ESC_OA8542AA	131	653
ESC_OA8541AA	86	935
ESC_OA8540AA	38	669
ESC_OA8539AA	636	653
ESC_OA8538AA	10445	175
ESC_OA8537AA	131	653
ESC_OA8532AA	711	935
ESC_OA8530AA	10444	653
ESC_OA8528AA	38	669
ESC_OA8527AA	69	387
ESC_OA8526AA	38	669
ESC_OA8525AA	131	653
ESC_OA8524AA	73	737
ESC_OA8523AA	4985	254
ESC_OA8522AA	34	504
ESC_OA8521AA	38	669
ESC_OA8520AA	38	669
ESC_OA8519AA	131	653
ESC_OA8518AA	949	175
ESC_OA8517AA	1722	599
ESC_OA8514AA	5792	328
ESC_OA8513AA	46	447
ESC_OA8512AA	38	669
ESC_OA8511AA	12	626
ESC_OA8510AA	711	935
ESC_OA8509AA	38	669
ESC_OA8508AA	101	696
ESC_OA8507AA	69	387
ESC_OA8505AA	58	198
ESC_OA8504AA	6361	261
ESC_OA8501AA	38	669
ESC_OA8500AA	711	935
ESC_OA8499AA	46	447
ESC_OA8497AA	101	696
ESC_OA8494AA	10	512
ESC_OA8493AA	162	924
ESC_OA8492AA	394	400
ESC_OA8491AA	101	696
ESC_OA8490AA	131	653
ESC_OA8489AA	405	923
ESC_OA8488AA	131	653
ESC_OA8487AA	2785	175
ESC_OA8486AA	88	160

ESC_OA8484AA	617	511
ESC_OA8483AA	69	387
ESC_OA8482AA	949	175
ESC_OA8481AA	131	653
ESC_OA8480AA	159	789
ESC_OA8479AA	131	653
ESC_OA8478AA	949	175
ESC_OA8477AA	38	669
ESC_OA8476AA	131	653
ESC_OA8475AA	131	653
ESC_OA8474AA	131	653
ESC_OA8473AA	141	644
ESC_OA8472AA	131	653
ESC_OA8471AA	131	653
ESC_OA8470AA	648	359
ESC_OA8469AA	38	669
ESC_OA8468AA	131	653
ESC_OA8467AA	617	511
ESC_OA8466AA	131	653
ESC_OA8465AA	131	653
ESC_OA8464AA	949	175
ESC_OA8463AA	1491	76
ESC_OA8462AA	38	669
ESC_OA8461AA	1491	76
ESC_OA8460AA	636	653
ESC_OA8459AA	617	511
ESC_OA8458AA	2211	512
ESC_OA8457AA	949	175
ESC_OA8456AA	131	653
ESC_OA8455AA	34	504
ESC_OA8454AA	131	653
ESC_OA8453AA	131	653
ESC_OA8452AA	1312	76
ESC_OA8451AA	216	512
ESC_OA8450AA	394	400
ESC_OA8449AA	131	653
ESC_OA8448AA	38	669
ESC_OA8447AA	192	696
ESC_OA8446AA	73	737
ESC_OA8445AA	23	156
ESC_OA8444AA	648	359
ESC_OA8443AA	2450	447
ESC_OA8442AA	1280	359
ESC_OA8441AA	648	359
ESC_OA8440AA	44	512
ESC_OA8438AA	648	359
ESC_OA8436AA	501	900
ESC_OA8435AA	38	669
ESC_OA8434AA	131	653
ESC_OA8433AA	131	653

ESC_OA8432AA	1312	76
ESC_OA8431AA	998	644
ESC_OA8430AA	453	935
ESC_OA8429AA	1722	599
ESC_OA8428AA	457	595
ESC_OA8426AA	457	595
ESC_OA8424AA	457	595
ESC_OA8423AA	457	595
ESC_OA8422AA	457	595
ESC_OA8421AA	69	387
ESC_OA8420AA	1722	599
ESC_OA8419AA	1722	599
ESC_OA8418AA	1722	599
ESC_OA8417AA	1722	599
ESC_OA8416AA	744	512
ESC_OA8415AA	38	669
ESC_OA8414AA	9547	76
ESC_OA8413AA	48	76
ESC_OA8412AA	2067	257
ESC_OA8411AA	48	76
ESC_OA8410AA	5870	504
ESC_OA8408AA	48	76
ESC_OA8407AA	69	387
ESC_OA8406AA	2067	257
ESC_OA8405AA	5848	701
ESC_OA8404AA	93	71
ESC_OA8403AA	744	512
ESC_OA8402AA	93	71
ESC_OA8401AA	93	71
ESC_OA8400AA	93	71
ESC_OA8399AA	101	696
ESC_OA8398AA	101	696
ESC_OA8397AA	38	669
ESC_OA8396AA	167	512
ESC_OA8395AA	167	512
ESC_OA8393AA	167	512
ESC_OA8391AA	9402	789
ESC_OA8388AA	38	669
ESC_OA8387AA	1722	599
ESC_OA8381AA	34	504
ESC_OA8380AA	744	512
ESC_OA8379AA	34	504
ESC_OA8378AA	34	504
ESC_OA8376AA	34	504
ESC_OA8375AA	34	504
ESC_OA8374AA	9402	789
ESC_OA8372AA	1722	599
ESC_OA8371AA	1722	599
ESC_OA8370AA	1722	599
ESC_OA8369AA	1722	599

ESC_OA8368AA	1722	599
ESC_OA8366AA	101	696
ESC_OA8365AA	101	696
ESC_OA8364AA	515	1004
ESC_OA8363AA	48	76
ESC_OA8362AA	1638	519
ESC_OA8361AA	515	1004
ESC_OA8360AA	617	511
ESC_OA8359AA	167	512
ESC_OA8358AA	167	512
ESC_OA8357AA	617	511
ESC_OA8356AA	167	512
ESC_OA8355AA	167	512
ESC_OA8353AA	9402	789
ESC_OA8352AA	10498	512
ESC_OA8351AA	167	512
ESC_OA8350AA	394	400
ESC_OA8349AA	394	400
ESC_OA8348AA	746	789
ESC_OA8346AA	101	696
ESC_OA8343AA	9402	789
ESC_OA8341AA	515	1004
ESC_OA8340AA	515	1004
ESC_OA8339AA	10499	387
ESC_OA8338AA	10499	387
ESC_OA8337AA	1081	198
ESC_OA8336AA	515	1004
ESC_OA8335AA	69	387
ESC_OA8334AA	10499	387
ESC_OA8333AA	224	198
ESC_OA8332AA	6984	156
ESC_OA8331AA	515	1004
ESC_OA8330AA	410	156
ESC_OA8329AA	542	824
ESC_OA8327AA	101	696
ESC_OA8326AA	48	76
ESC_OA8325AA	48	76
ESC_OA8323AA	34	504
ESC_OA8322AA	195	76
ESC_OA8321AA	398	445
ESC_OA8319AA	69	387
ESC_OA8318AA	101	696
ESC_OA8317AA	515	1004
ESC_OA8316AA	3285	257
ESC_OA8315AA	101	696
ESC_OA8313AA	457	595
ESC_OA8312AA	457	595
ESC_OA8311AA	457	595
ESC_OA8310AA	224	198
ESC_OA8309AA	457	595

ESC_OA8308AA	131	653
ESC_OA8306AA	48	76
ESC_OA8305AA	34	504
ESC_OA8304AA	34	504
ESC_OA8303AA	101	696
ESC_OA8302AA	34	504
ESC_OA8301AA	34	504
ESC_OA8300AA	6799	198
ESC_OA8299AA	34	504
ESC_OA8297AA	10499	387
ESC_OA8296AA	9402	789
ESC_OA8295AA	101	696
ESC_OA8294AA	101	696
ESC_OA8292AA	542	824
ESC_OA8291AA	48	76
ESC_OA8290AA	1722	599
ESC_OA8289AA	6856	76
ESC_OA8288AA	38	669
ESC_OA8287AA	1722	599
ESC_OA8286AA	542	824
ESC_OA8285AA	515	1004
ESC_OA8284AA	746	789
ESC_OA8283AA	101	696
ESC_OA8281AA	101	696
ESC_OA8279AA	1722	599
ESC_OA8278AA	1722	599
ESC_OA8277AA	1722	599
ESC_OA8276AA	1722	599
ESC_OA8275AA	1722	599
ESC_OA8274AA	201	257
ESC_OA8273AA	69	387
ESC_OA8272AA	101	696
ESC_OA8271AA	2067	257
ESC_OA8269AA	1072	355
ESC_OA8268AA	10500	76
ESC_OA8267AA	665	79
ESC_OA8266AA	4682	175
ESC_OA8265AA	6984	156
ESC_OA8264AA	69	387
ESC_OA8263AA	3285	257
ESC_OA8262AA	10	512
ESC_OA8261AA	101	696
ESC_OA8260AA	3285	257
ESC_OA8259AA	10	512
ESC_OA8258AA	10	512
ESC_OA8257AA	38	669
ESC_OA8256AA	38	669
ESC_OA8255AA	9402	789
ESC_OA8254AA	206	286
ESC_OA8253AA	10499	387

ESC_OA8252AA	38	669
ESC_OA8251AA	38	669
ESC_OA8250AA	40	198
ESC_OA8249AA	2067	257
ESC_OA8247AA	69	387
ESC_OA8246AA	69	387
ESC_OA8245AA	69	387
ESC_OA8243AA	9402	789
ESC_OA8242AA	69	387
ESC_OA8240AA	69	387
ESC_OA8239AA	69	387
ESC_OA8238AA	1722	599
ESC_OA8237AA	1722	599
ESC_OA8236AA	1722	599
ESC_OA8232AA	1722	599
ESC_OA8231AA	1722	599
ESC_OA8229AA	5895	653
ESC_OA8227AA	5895	653
ESC_OA8226AA	5895	653
ESC_OA8224AA	542	824
ESC_OA8223AA	457	595
ESC_OA8222AA	457	595
ESC_OA8219AA	1722	599
ESC_OA8218AA	2067	257
ESC_OA8217AA	2067	257
ESC_OA8216AA	2067	257
ESC_OA8215AA	2067	257
ESC_OA8214AA	2067	257
ESC_OA8212AA	2067	257
ESC_OA8211AA	2067	257
ESC_OA8210AA	2067	257
ESC_OA8209AA	10440	653
ESC_OA8208AA	648	359
ESC_OA8207AA	131	653
ESC_OA8206AA	131	653
ESC_OA8205AA	38	669
ESC_OA8204AA	131	653
ESC_OA8203AA	6009	847
ESC_OA8202AA	1193	761
ESC_OA8201AA	131	653
ESC_OA8200AA	69	387
ESC_OA8199AA	131	653
ESC_OA8198AA	963	669
ESC_OA8197AA	167	512
ESC_OA8196AA	10442	519
ESC_OA8195AA	617	511
ESC_OA8194AA	167	512
ESC_OA8193AA	155	175
ESC_OA8192AA	617	511
ESC_OA8191AA	10441	453

ESC_OA8190AA	167	512
ESC_OA8189AA	617	511
ESC_OA8188AA	617	511
ESC_OA8187AA	617	511
ESC_OA8186AA	10443	286
ESC_OA8185AA	191	198
ESC_OA8184AA	372	424
ESC_OA8183AA	167	512
ESC_OA8182AA	167	512
ESC_OA8181AA	167	512
ESC_OA8180AA	540	286
ESC_OA8179AA	10	512
ESC_OA8178AA	46	447
ESC_OA8177AA	206	286
ESC_OA8176AA	167	512
ESC_OA8175AA	1638	519
ESC_OA8173AA	167	512
ESC_OA8172AA	617	511
ESC_OA8171AA	165	504
ESC_OA8170AA	683	175
ESC_OA8169AA	167	512
ESC_OA8168AA	6852	512
ESC_OA8167AA	744	512
ESC_OA8166AA	1286	474
ESC_OA8165AA	1148	257
ESC_OA8164AA	617	511
ESC_OA8163AA	226	512
ESC_OA8162AA	10	512
ESC_OA8161AA	398	445
ESC_OA8160AA	167	512
ESC_OA8159AA	10	512
ESC_OA8158AA	38	669
ESC_OA8157AA	162	924
ESC_OA8156AA	744	512
ESC_OA8155AA	2065	512
ESC_OA8150AA	906	198
ESC_OA8147AA	10435	76
ESC_OA8146AA	1463	263
ESC_OA8145AA	428	654
ESC_OA8144AA	1463	263
ESC_OA8143AA	7006	936
ESC_OA8142AA	11	936
ESC_OA8141AA	7006	936
ESC_OA8140AA	11	936
ESC_OA8139AA	7006	936
ESC_OA8138AA	11	936
ESC_OA8137AA	11	936
ESC_OA8136AA	599	626
ESC_OA8135AA	392	234
ESC_OA8134AA	245	261

ESC_OA8133AA	145	256
ESC_OA8132AA	245	261
ESC_OA8131AA	152	257
ESC_OA8130AA	10433	198
ESC_OA8129AA	88	160
ESC_OA8128AA	6189	175
ESC_OA8127AA	6189	175
ESC_OA8126AA	189	504
ESC_OA8125AA	10432	175
ESC_OA8124AA	10	512
ESC_OA8123AA	101	696
ESC_OA8122AA	1128	924
ESC_OA8121AA	515	1004
ESC_OA8120AA	201	257
ESC_OA8119AA	1125	198
ESC_OA8118AA	93	71
ESC_OA8117AA	10	512
ESC_OA8116AA	58	198
ESC_OA8115AA	1079	235
ESC_OA8114AA	11	936
ESC_OA8113AA	2521	257
ESC_OA8112AA	278	924
ESC_OA8111AA	328	924
ESC_OA8110AA	1679	160
ESC_OA8109AA	154	257
ESC_OA8108AA	187	257
ESC_OA8107AA	1730	234
ESC_OA8106AA	1967	191
ESC_OA8105AA	135	653
ESC_OA8104AA	355	764
ESC_OA8103AA	2792	886
ESC_OA8102AA	209	512
ESC_OA8101AA	56	198
ESC_OA8100AA	1139	447
ESC_OA8099AA	1147	198
ESC_OA8098AA	1079	235
ESC_OA8097AA	58	198
ESC_OA8096AA	1236	380
ESC_OA8095AA	635	141
ESC_OA8094AA	635	141
ESC_OA8093AA	67	257
ESC_OA8092AA	1141	512
ESC_OA8091AA	3580	257
ESC_OA8090AA	10	512
ESC_OA8089AA	38	669
ESC_OA8088AA	641	935
ESC_OA8087AA	10434	175
ESC_OA8086AA	2520	878
ESC_OA8085AA	48	76
ESC_OA8084AA	38	669

ESC_OA8083AA	152	257
ESC_OA8082AA	252	190
ESC_OA8081AA	245	261
ESC_OA8080AA	152	257
ESC_OA8079AA	152	257
ESC_OA8078AA	152	257
ESC_OA8077AA	152	257
ESC_OA8076AA	245	261
ESC_OA8075AA	148	191
ESC_OA8074AA	245	261
ESC_OA8073AA	245	261
ESC_OA8072AA	131	653
ESC_OA8071AA	131	653
ESC_OA8070AA	131	653
ESC_OA8069AA	457	595
ESC_OA8068AA	1193	761
ESC_OA8067AA	95	738
ESC_OA8066AA	131	653
ESC_OA8065AA	131	653
ESC_OA8064AA	131	653
ESC_OA8063AA	38	669
ESC_OA8062AA	655	926
ESC_OA8061AA	349	670
ESC_OA8060AA	131	653
ESC_OA8059AA	453	935
ESC_OA8058AA	648	359
ESC_OA8057AA	131	653
ESC_OA8056AA	141	644
ESC_OA8055AA	131	653
ESC_OA8054AA	131	653
ESC_OA8053AA	131	653
ESC_OA8052AA	131	653
ESC_OA8051AA	152	257
ESC_OA8050AA	245	261
ESC_OA8049AA	152	257
ESC_OA8048AA	245	261
ESC_OA8047AA	245	261
ESC_OA8046AA	152	257
ESC_OA8045AA	245	261
ESC_OA8044AA	245	261
ESC_OA8043AA	245	261
ESC_OA8042AA	245	261
ESC_OA8041AA	245	261
ESC_OA8040AA	152	257
ESC_OA8039AA	245	261
ESC_OA8038AA	245	261
ESC_OA8037AA	245	261
ESC_OA8035AA	1024	261
ESC_OA8034AA	11	936
ESC_OA8032AA	6323	527

ESC_OA8031AA	17	191
ESC_OA8030AA	182	246
ESC_OA8029AA	182	246
ESC_OA8028AA	38	669
ESC_OA8027AA	2178	924
ESC_OA8026AA	443	257
ESC_OA8025AA	300	286
ESC_OA8024AA	1380	914
ESC_OA8023AA	5584	640
ESC_OA8022AA	381	156
ESC_OA8021AA	69	387
ESC_OA8020AA	1583	532
ESC_OA8019AA	17	191
ESC_OA8018AA	29	156
ESC_OA8017AA	584	414
ESC_OA8016AA	10	512
ESC_OA8015AA	34	504
ESC_OA8014AA	589	650
ESC_OA8013AA	278	924
ESC_OA8012AA	1380	914
ESC_OA8011AA	200	198
ESC_OA8010AA	1491	76
ESC_OA8009AA	10	512
ESC_OA8006AA	4063	156
ESC_OA8005AA	6	445
ESC_OA8004AA	200	198
ESC_OA8003AA	206	286
ESC_OA8002AA	29	156
ESC_OA8001AA	29	156
ESC_OA8000AA	10400	286
ESC_OA7999AA	328	924
ESC_OA7998AA	975	512
ESC_OA7997AA	29	156
ESC_OA7996AA	88	160
ESC_OA7995AA	29	156
ESC_OA7994AA	300	286
ESC_OA7993AA	270	204
ESC_OA7992AA	328	924
ESC_OA7991AA	10	512
ESC_OA7990AA	206	286
ESC_OA7989AA	28	537
ESC_OA7988AA	28	537
ESC_OA7987AA	40	198
ESC_OA7986AA	16	156
ESC_OA7985AA	29	156
ESC_OA7984AA	2353	76
ESC_OA7983AA	20	198
ESC_OA7982AA	795	924
ESC_OA7981AA	2959	914
ESC_OA7980AA	206	286

ESC_OA7979AA	20	198
ESC_OA7978AA	752	512
ESC_OA7977AA	28	537
ESC_OA7976AA	2346	435
ESC_OA7975AA	10419	156
ESC_OA7974AA	8397	355
ESC_OA7973AA	8397	355
ESC_OA7971AA	1380	914
ESC_OA7970AA	40	198
ESC_OA7969AA	38	669
ESC_OA7968AA	2178	924
ESC_OA7967AA	200	198
ESC_OA7966AA	1380	914
ESC_OA7965AA	381	156
ESC_OA7964AA	2	900
ESC_OA7963AA	10	512
ESC_OA7962AA	337	257
ESC_OA7961AA	182	246
ESC_OA7960AA	517	924
ESC_OA7959AA	517	924
ESC_OA7958AA	182	246
ESC_OA7957AA	381	156
ESC_OA7956AA	841	204
ESC_OA7955AA	394	400
ESC_OA7954AA	4213	251
ESC_OA7953AA	34	504
ESC_OA7952AA	34	504
ESC_OA7951AA	34	504
ESC_OA7950AA	517	924
ESC_OA7949AA	17	191
ESC_OA7948AA	34	504
ESC_OA7947AA	182	246
ESC_OA7946AA	5305	404
ESC_OA7945AA	182	246
ESC_OA7944AA	200	198
ESC_OA7943AA	443	257
ESC_OA7942AA	38	669
ESC_OA7941AA	182	246
ESC_OA7940AA	182	246
ESC_OA7939AA	316	257
ESC_OA7938AA	655	926
ESC_OA7937AA	1491	76
ESC_OA7936AA	34	504
ESC_OA7935AA	10446	539
ESC_OA7934AA	130	387
ESC_OA7933AA	1583	532
ESC_OA7932AA	1491	76
ESC_OA7931AA	337	257
ESC_OA7930AA	2178	924
ESC_OA7929AA	4063	156

ESC_OA7928AA	182	246
ESC_OA7927AA	327	204
ESC_OA7926AA	10	512
ESC_OA7925AA	4305	512
ESC_OA7924AA	20	198
ESC_OA7922AA	655	926
ESC_OA7921AA	4213	251
ESC_OA7920AA	34	504
ESC_OA7919AA	4213	251
ESC_OA7918AA	200	198
ESC_OA7917AA	34	504
ESC_OA7916AA	10	512
ESC_OA7915AA	295	160
ESC_OA7914AA	484	71
ESC_OA7913AA	7281	286
ESC_OA7912AA	10	512
ESC_OA7911AA	69	387
ESC_OA7910AA	38	669
ESC_OA7909AA	200	198
ESC_OA7908AA	841	204
ESC_OA7907AA	40	198
ESC_OA7906AA	1380	914
ESC_OA7905AA	10	512
ESC_OA7904AA	200	198
ESC_OA7903AA	10	512
ESC_OA7902AA	414	387
ESC_OA7901AA	381	156
ESC_OA7900AA	5578	830
ESC_OA7899AA	752	512
ESC_OA7898AA	327	204
ESC_OA7897AA	752	512
ESC_OA7896AA	6323	527
ESC_OA7895AA	10422	286
ESC_OA7894AA	517	924
ESC_OA7892AA	655	926
ESC_OA7891AA	4213	251
ESC_OA7890AA	28	537
ESC_OA7889AA	34	504
ESC_OA7888AA	5266	198
ESC_OA7887AA	17	191
ESC_OA7885AA	517	924
ESC_OA7884AA	34	504
ESC_OA7883AA	2959	914
ESC_OA7882AA	34	504
ESC_OA7881AA	29	156
ESC_OA7880AA	328	924
ESC_OA7879AA	589	650
ESC_OA7878AA	10	512
ESC_OA7877AA	159	789
ESC_OA7876AA	4213	251

ESC_OA7875AA	752	512
ESC_OA7874AA	342	286
ESC_OA7873AA	763	878
ESC_OA7872AA	342	286
ESC_OA7871AA	677	257
ESC_OA7870AA	763	878
ESC_OA7869AA	446	257
ESC_OA7868AA	446	257
ESC_OA7867AA	443	257
ESC_OA7866AA	446	257
ESC_OA7865AA	446	257
ESC_OA7864AA	17	191
ESC_OA7863AA	677	257
ESC_OA7862AA	38	669
ESC_OA7861AA	6992	842
ESC_OA7860AA	10	512
ESC_OA7859AA	127	738
ESC_OA7858AA	33	198
ESC_OA7857AA	4748	234
ESC_OA7856AA	300	286
ESC_OA7855AA	349	670
ESC_OA7854AA	763	878
ESC_OA7853AA	73	737
ESC_OA7852AA	1159	381
ESC_OA7851AA	405	923
ESC_OA7850AA	131	653
ESC_OA7849AA	69	387
ESC_OA7848AA	95	738
ESC_OA7847AA	405	923
ESC_OA7846AA	131	653
ESC_OA7845AA	131	653
ESC_OA7844AA	8677	483
ESC_OA7843AA	101	696
ESC_OA7842AA	156	175
ESC_OA7841AA	2325	447
ESC_OA7840AA	48	76
ESC_OA7837AA	58	198
ESC_OA7835AA	1286	474
ESC_OA7834AA	10	512
ESC_OA7833AA	226	512
ESC_OA7832AA	10328	175
ESC_OA7831AA	1638	519
ESC_OA7830AA	4663	257
ESC_OA7828AA	206	286
ESC_OA7826AA	10426	76
ESC_OA7825AA	6448	175
ESC_OA7824AA	1638	519
ESC_OA7823AA	4598	868
ESC_OA7822AA	117	592
ESC_OA7821AA	218	519

ESC_OA7820AA	5876	847
ESC_OA7819AA	1248	156
ESC_OA7818AA	189	504
ESC_OA7817AA	10425	263
ESC_OA7816AA	2522	198
ESC_OA7815AA	1992	257
ESC_OA7814AA	1141	512
ESC_OA7813AA	937	198
ESC_OA7812AA	109	257
ESC_OA7811AA	3234	257
ESC_OA7810AA	155	175
ESC_OA7809AA	1665	601
ESC_OA7808AA	877	935
ESC_OA7807AA	10	512
ESC_OA7806AA	155	175
ESC_OA7805AA	215	512
ESC_OA7804AA	877	935
ESC_OA7803AA	1128	924
ESC_OA7802AA	154	257
ESC_OA7801AA	69	387
ESC_OA7800AA	131	653
ESC_OA7799AA	410	156
ESC_OA7798AA	131	653
ESC_OA7797AA	10424	653
ESC_OA7796AA	69	387
ESC_OA7795AA	69	387
ESC_OA7794AA	90	156
ESC_OA7793AA	10	512
ESC_OA7792AA	681	393
ESC_OA7791AA	131	653
ESC_OA7790AA	405	923
ESC_OA7789AA	648	359
ESC_OA7788AA	131	653
ESC_OA7787AA	1083	203
ESC_OA7786AA	131	653
ESC_OA7785AA	428	654
ESC_OA7784AA	23	156
ESC_OA7783AA	131	653
ESC_OA7782AA	73	737
ESC_OA7781AA	131	653
ESC_OA7780AA	2453	204
ESC_OA7779AA	93	71
ESC_OA7778AA	8182	601
ESC_OA7777AA	12	626
ESC_OA7776AA	131	653
ESC_OA7775AA	88	160
ESC_OA7774AA	131	653
ESC_OA7773AA	357	654
ESC_OA7772AA	73	737
ESC_OA7771AA	357	654

ESC_OA7770AA	58	198
ESC_OA7769AA	1163	579
ESC_OA7768AA	80	731
ESC_OA7767AA	906	198
ESC_OA7766AA	69	387
ESC_OA7765AA	131	653
ESC_OA7764AA	354	15
ESC_OA7763AA	131	653
ESC_OA7762AA	5204	311
ESC_OA7761AA	144	740
ESC_OA7760AA	127	738
ESC_OA7759AA	501	900
ESC_OA7758AA	131	653
ESC_OA7757AA	405	923
ESC_OA7756AA	12	626
ESC_OA7755AA	783	204
ESC_OA7754AA	155	175
ESC_OA7753AA	117	592
ESC_OA7752AA	69	387
ESC_OA7751AA	131	653
ESC_OA7750AA	95	738
ESC_OA7749AA	73	737
ESC_OA7748AA	88	160
ESC_OA7747AA	131	653
ESC_OA7746AA	73	737
ESC_OA7745AA	73	737
ESC_OA7744AA	73	737
ESC_OA7743AA	23	156
ESC_OA7742AA	131	653
ESC_OA7741AA	141	644
ESC_OA7740AA	783	204
ESC_OA7739AA	88	160
ESC_OA7738AA	131	653
ESC_OA7737AA	144	740
ESC_OA7736AA	7429	359
ESC_OA7735AA	73	737
ESC_OA7734AA	131	653
ESC_OA7733AA	95	738
ESC_OA7732AA	167	512
ESC_OA7731AA	1316	471
ESC_OA7730AA	127	738
ESC_OA7729AA	10	512
ESC_OA7728AA	1193	761
ESC_OA7727AA	69	387
ESC_OA7726AA	3249	156
ESC_OA7725AA	80	731
ESC_OA7724AA	127	738
ESC_OA7723AA	141	644
ESC_OA7722AA	88	160
ESC_OA7721AA	131	653

ESC_OA7720AA	1193	761
ESC_OA7719AA	1193	761
ESC_OA7718AA	2558	782
ESC_OA7717AA	537	756
ESC_OA7716AA	224	198
ESC_OA7715AA	141	644
ESC_OA7714AA	12	626
ESC_OA7713AA	209	512
ESC_OA7712AA	95	738
ESC_OA7711AA	641	935
ESC_OA7710AA	1431	257
ESC_OA7709AA	131	653
ESC_OA7707AA	453	935
ESC_OA7706AA	73	737
ESC_OA7705AA	73	737
ESC_OA7704AA	14	761
ESC_OA7703AA	69	387
ESC_OA7702AA	93	71
ESC_OA7701AA	69	387
ESC_OA7700AA	10427	75
ESC_OA7699AA	420	741
ESC_OA7698AA	95	738
ESC_OA7697AA	8180	653
ESC_OA7696AA	69	387
ESC_OA7695AA	58	198
ESC_OA7694AA	23	156
ESC_OA7693AA	453	935
ESC_OA7692AA	131	653
ESC_OA7691AA	655	926
ESC_OA7690AA	6274	257
ESC_OA7689AA	21	156
ESC_OA7688AA	29	156
ESC_OA7687AA	206	286
ESC_OA7686AA	675	234
ESC_OA7685AA	738	598
ESC_OA7684AA	69	387
ESC_OA7683AA	38	669
ESC_OA7682AA	443	257
ESC_OA7681AA	10	512
ESC_OA7680AA	131	653
ESC_OA7679AA	131	653
ESC_OA7678AA	69	387
ESC_OA7677AA	135	653
ESC_OA7676AA	162	924
ESC_OA7675AA	8185	924
ESC_OA7674AA	998	644
ESC_OA7673AA	155	175
ESC_OA7672AA	155	175
ESC_OA7671AA	131	653
ESC_OA7670AA	95	738

ESC_OA7669AA	501	900
ESC_OA7668AA	73	737
ESC_OA7667AA	1279	160
ESC_OA7666AA	2	900
ESC_OA7665AA	73	737
ESC_OA7664AA	73	737
ESC_OA7663AA	131	653
ESC_OA7662AA	12	626
ESC_OA7661AA	550	756
ESC_OA7660AA	80	731
ESC_OA7659AA	1193	761
ESC_OA7658AA	73	737
ESC_OA7657AA	131	653
ESC_OA7656AA	131	653
ESC_OA7655AA	69	387
ESC_OA7654AA	131	653
ESC_OA7653AA	2517	160
ESC_OA7652AA	131	653
ESC_OA7651AA	998	644
ESC_OA7650AA	131	653
ESC_OA7649AA	95	738
ESC_OA7648AA	540	286
ESC_OA7647AA	12	626
ESC_OA7646AA	74	743
ESC_OA7645AA	1333	738
ESC_OA7644AA	8023	903
ESC_OA7643AA	117	592
ESC_OA7642AA	1167	257
ESC_OA7641AA	131	653
ESC_OA7640AA	354	15
ESC_OA7639AA	73	737
ESC_OA7638AA	131	653
ESC_OA7637AA	349	670
ESC_OA7636AA	73	737
ESC_OA7635AA	393	343
ESC_OA7634AA	718	257
ESC_OA7633AA	131	653
ESC_OA7632AA	131	653
ESC_OA7631AA	315	669
ESC_OA7630AA	69	387
ESC_OA7629AA	38	669
ESC_OA7628AA	73	737
ESC_OA7627AA	421	744
ESC_OA7626AA	69	387
ESC_OA7625AA	1177	669
ESC_OA7624AA	95	738
ESC_OA7623AA	117	592
ESC_OA7622AA	131	653
ESC_OA7621AA	937	198
ESC_OA7620AA	12	626

ESC_OA7619AA	657	592
ESC_OA7618AA	8186	629
ESC_OA7617AA	12	626
ESC_OA7616AA	69	387
ESC_OA7615AA	95	738
ESC_OA7614AA	69	387
ESC_OA7613AA	6826	511
ESC_OA7612AA	69	387
ESC_OA7611AA	12	626
ESC_OA7610AA	767	198
ESC_OA7609AA	8190	175
ESC_OA7608AA	58	198
ESC_OA7607AA	117	592
ESC_OA7606AA	10	512
ESC_OA7605AA	156	175
ESC_OA7604AA	12	626
ESC_OA7603AA	127	738
ESC_OA7602AA	349	670
ESC_OA7601AA	88	160
ESC_OA7600AA	141	644
ESC_OA7599AA	357	654
ESC_OA7598AA	95	738
ESC_OA7597AA	127	738
ESC_OA7596AA	141	644
ESC_OA7595AA	131	653
ESC_OA7594AA	131	653
ESC_OA7593AA	73	737
ESC_OA7592AA	453	935
ESC_OA7591AA	131	653
ESC_OA7590AA	95	738
ESC_OA7589AA	10	512
ESC_OA7588AA	80	731
ESC_OA7587AA	609	447
ESC_OA7586AA	11	936
ESC_OA7585AA	11	936
ESC_OA7584AA	11	936
ESC_OA7583AA	46	447
ESC_OA7582AA	405	923
ESC_OA7581AA	38	669
ESC_OA7580AA	131	653
ESC_OA7579AA	23	156
ESC_OA7578AA	131	653
ESC_OA7577AA	131	653
ESC_OA7576AA	95	738
ESC_OA7575AA	10428	387
ESC_OA7574AA	131	653
ESC_OA7573AA	131	653
ESC_OA7572AA	131	653
ESC_OA7571AA	131	653
ESC_OA7570AA	405	923

ESC_OA7569AA	1193	761
ESC_OA7568AA	131	653
ESC_OA7567AA	131	653
ESC_OA7566AA	131	653
ESC_OA7565AA	131	653
ESC_OA7564AA	648	359
ESC_OA7563AA	131	653
ESC_OA7562AA	963	669
ESC_OA7561AA	405	923
ESC_OA7560AA	38	669
ESC_OA7559AA	10	512
ESC_OA7558AA	10	512
ESC_OA7547AA	10	512
ESC_OA7540AA	10	512
ESC_OA7539AA	10	512
ESC_OA7538AA	10	512
ESC_OA7533AA	131	653
ESC_OA7532AA	131	653
ESC_OA7531AA	131	653
ESC_OA7530AA	131	653
ESC_OA7529AA	131	653
ESC_OA7528AA	131	653
ESC_OA7527AA	131	653
ESC_OA7526AA	131	653
ESC_OA7525AA	131	653
ESC_OA7524AA	1177	669
ESC_OA7523AA	2179	924
ESC_OA7522AA	131	653
ESC_OA7521AA	10	512
ESC_OA7520AA	10	512
ESC_OA7519AA	877	935
ESC_OA7518AA	641	935
ESC_OA7517AA	10	512
ESC_OA7516AA	10	512
ESC_OA7515AA	10	512
ESC_OA7514AA	10	512
ESC_OA7512AA	10	512
ESC_OA7511AA	10	512
ESC_OA7510AA	10	512
ESC_OA7509AA	10	512
ESC_OA7508AA	10	512
ESC_OA7507AA	10	512
ESC_OA7506AA	10	512
ESC_OA7504AA	10	512
ESC_OA7503AA	10	512
ESC_OA7502AA	10	512
ESC_OA7501AA	871	447
ESC_OA7500AA	10	512
ESC_OA7499AA	10	512
ESC_OA7498AA	10429	512

ESC_OA7497AA	154	257
ESC_OA7496AA	10	512
ESC_OA7495AA	10	512
ESC_OA7494AA	10	512
ESC_OA7492AA	871	447
ESC_OA7491AA	10	512
ESC_OA7490AA	10	512
ESC_OA7489AA	10	512
ESC_OA7488AA	10	512
ESC_OA7487AA	877	935
ESC_OA7486AA	10	512
ESC_OA7485AA	10	512
ESC_OA7484AA	1114	504
ESC_OA7483AA	10	512
ESC_OA7482AA	10	512
ESC_OA7481AA	10	512
ESC_OA7480AA	117	592
ESC_OA7479AA	648	359
ESC_OA7478AA	95	738
ESC_OA7477AA	648	359
ESC_OA7476AA	1193	761
ESC_OA7475AA	405	923
ESC_OA7474AA	648	359
ESC_OA7473AA	131	653
ESC_OA7472AA	131	653
ESC_OA7471AA	73	737
ESC_OA7470AA	131	653
ESC_OA7469AA	1177	669
ESC_OA7468AA	38	669
ESC_OA7467AA	152	257
ESC_OA7466AA	10	512
ESC_OA7465AA	10	512
ESC_OA7464AA	10	512
ESC_OA7463AA	11	936
ESC_OA7462AA	11	936
ESC_OA7461AA	11	936
ESC_OA7458AA	10399	504
ESC_OA7457AA	10398	234
ESC_OA7456AA	11	936
ESC_OA7455AA	11	936
ESC_OA7454AA	301	307
ESC_OA7453AA	301	307
ESC_OA7452AA	29	156
ESC_OA7451AA	29	156
ESC_OA7450AA	8540	76
ESC_OA7449AA	10392	444
ESC_OA7448AA	10392	444
ESC_OA7447AA	10392	444
ESC_OA7446AA	10392	444
ESC_OA7445AA	6704	512

ESC_OA7444AA	9693	18
ESC_OA7443AA	10386	198
ESC_OA7442AA	8630	257
ESC_OA7441AA	152	257
ESC_OA7440AA	152	257
ESC_OA7439AA	245	261
ESC_OA7427AA	21	156
ESC_OA7426AA	10	512
ESC_OA7425AA	10	512
ESC_OA7424AA	34	504
ESC_OA7423AA	34	504
ESC_OA7422AA	10	512
ESC_OA7421AA	10	512
ESC_OA7420AA	10	512
ESC_OA7419AA	21	156
ESC_OA7416AA	1308	257
ESC_OA7415AA	34	504
ESC_OA7414AA	4577	257
ESC_OA7413AA	34	504
ESC_OA7412AA	1607	844
ESC_OA7411AA	4577	257
ESC_OA7410AA	10	512
ESC_OA7409AA	23	156
ESC_OA7408AA	23	156
ESC_OA7407AA	4577	257
ESC_OA7406AA	88	160
ESC_OA7405AA	218	519
ESC_OA7404AA	10	512
ESC_OA7403AA	10	512
ESC_OA7401AA	218	519
ESC_OA7400AA	10	512
ESC_OA7399AA	218	519
ESC_OA7398AA	21	156
ESC_OA7397AA	21	156
ESC_OA7396AA	40	198
ESC_OA7395AA	34	504
ESC_OA7394AA	34	504
ESC_OA7393AA	34	504
ESC_OA7392AA	34	504
ESC_OA7391AA	34	504
ESC_OA7390AA	34	504
ESC_OA7389AA	34	504
ESC_OA7388AA	34	504
ESC_OA7387AA	681	393
ESC_OA7386AA	88	160
ESC_OA7384AA	1040	765
ESC_OA7382AA	88	160
ESC_OA7380AA	218	519
ESC_OA7379AA	34	504
ESC_OA7378AA	218	519

ESC_OA7377AA	34	504
ESC_OA7376AA	218	519
ESC_OA7375AA	40	198
ESC_OA7374AA	23	156
ESC_OA7373AA	58	198
ESC_OA7370AA	10	512
ESC_OA7369AA	154	257
ESC_OA7368AA	56	198
ESC_OA7367AA	34	504
ESC_OA7366AA	58	198
ESC_OA7365AA	34	504
ESC_OA7364AA	10	512
ESC_OA7362AA	34	504
ESC_OA7361AA	10	512
ESC_OA7359AA	34	504
ESC_OA7357AA	58	198
ESC_OA7356AA	34	504
ESC_OA7353AA	10	512
ESC_OA7352AA	10	512
ESC_OA7351AA	10	512
ESC_OA7350AA	58	198
ESC_OA7349AA	56	198
ESC_OA7348AA	154	257
ESC_OA7347AA	154	257
ESC_OA7345AA	58	198
ESC_OA7344AA	34	504
ESC_OA7343AA	34	504
ESC_OA7342AA	58	198
ESC_OA7341AA	3057	672
ESC_OA7340AA	34	504
ESC_OA7339AA	34	504
ESC_OA7338AA	3057	672
ESC_OA7337AA	34	504
ESC_OA7336AA	34	504
ESC_OA7335AA	993	789
ESC_OA7334AA	10	512
ESC_OA7333AA	34	504
ESC_OA7332AA	23	156
ESC_OA7331AA	993	789
ESC_OA7330AA	34	504
ESC_OA7329AA	101	696
ESC_OA7328AA	10	512
ESC_OA7327AA	40	198
ESC_OA7326AA	10	512
ESC_OA7325AA	23	156
ESC_OA7324AA	34	504
ESC_OA7323AA	993	789
ESC_OA7322AA	993	789
ESC_OA7321AA	23	156
ESC_OA7320AA	2946	818

ESC_OA7319AA	681	393
ESC_OA7317AA	10	512
ESC_OA7316AA	23	156
ESC_OA7314AA	10	512
ESC_OA7313AA	681	393
ESC_OA7312AA	10	512
ESC_OA7311AA	34	504
ESC_OA7310AA	34	504
ESC_OA7309AA	154	257
ESC_OA7308AA	34	504
ESC_OA7307AA	154	257
ESC_OA7306AA	34	504
ESC_OA7305AA	154	257
ESC_OA7303AA	40	198
ESC_OA7302AA	10	512
ESC_OA7301AA	21	156
ESC_OA7300AA	10	512
ESC_OA7299AA	10	512
ESC_OA7298AA	2946	818
ESC_OA7297AA	10	512
ESC_OA7296AA	10	512
ESC_OA7295AA	10	512
ESC_OA7294AA	10	512
ESC_OA7293AA	3057	672
ESC_OA7291AA	3057	672
ESC_OA7290AA	40	198
ESC_OA7289AA	10	512
ESC_OA7288AA	3057	672
ESC_OA7287AA	154	257
ESC_OA7286AA	34	504
ESC_OA7285AA	10	512
ESC_OA7284AA	34	504
ESC_OA7283AA	4577	257
ESC_OA7281AA	4577	257
ESC_OA7280AA	10	512
ESC_OA7279AA	10	512
ESC_OA7278AA	34	504
ESC_OA7277AA	10	512
ESC_OA7276AA	56	198
ESC_OA7275AA	154	257
ESC_OA7273AA	154	257
ESC_OA7272AA	154	257
ESC_OA7271AA	56	198
ESC_OA7270AA	154	257
ESC_OA7269AA	101	696
ESC_OA7268AA	101	696
ESC_OA7267AA	58	198
ESC_OA7266AA	10	512
ESC_OA7265AA	10	512
ESC_OA7264AA	34	504

ESC_OA7263AA	34	504
ESC_OA7262AA	10	512
ESC_OA7261AA	10	512
ESC_OA7260AA	1308	257
ESC_OA7259AA	1607	844
ESC_OA7258AA	1607	844
ESC_OA7257AA	40	198
ESC_OA7256AA	21	156
ESC_OA7255AA	21	156
ESC_OA7254AA	10	512
ESC_OA7252AA	10	512
ESC_OA7251AA	21	156
ESC_OA7250AA	21	156
ESC_OA7249AA	21	156
ESC_OA7248AA	21	156
ESC_OA7191AA	10	512
ESC_OA7190AA	10	512
ESC_OA7189AA	10	512
ESC_OA7188AA	10	512
ESC_OA7187AA	10	512
ESC_OA7186AA	10	512
ESC_OA7185AA	10	512
ESC_OA7184AA	10	512
ESC_OA7183AA	10	512
ESC_OA7182AA	10	512
ESC_OA7181AA	10	512
ESC_OA7180AA	10	512
ESC_OA7179AA	10	512
ESC_OA7178AA	10	512
ESC_OA7177AA	10	512
ESC_OA7176AA	10	512
ESC_OA7175AA	11	936
ESC_OA7174AA	998	644
ESC_OA7173AA	1721	311
ESC_OA7171AA	167	512
ESC_OA7170AA	11	936
ESC_OA7169AA	11	936
ESC_OA7168AA	11	936
ESC_OA7167AA	11	936
ESC_OA7166AA	11	936
ESC_OA7165AA	21	156
ESC_OA7164AA	517	924
ESC_OA7163AA	73	737
ESC_OA7162AA	17	191
ESC_OA7161AA	21	156
ESC_OA7160AA	131	653
ESC_OA7159AA	21	156
ESC_OA7158AA	658	684
ESC_OA7157AA	21	156
ESC_OA7156AA	17	191

ESC_OA7155AA	335	936
ESC_OA7154AA	21	156
ESC_OA7153AA	14	761
ESC_OA7152AA	21	156
ESC_OA7151AA	1695	504
ESC_OA7150AA	2325	447
ESC_OA7149AA	196	257
ESC_OA7148AA	154	257
ESC_OA7147AA	3580	257
ESC_OA7061AA	6021	234
ESC_OA7060AA	10	512
ESC_OA7059AA	10388	254
ESC_OA7058AA	4684	257
ESC_OA7057AA	8677	483
ESC_OA7056AA	1250	926
ESC_OA7055AA	7576	903
ESC_OA7054AA	7576	903
ESC_OA7053AA	58	198
ESC_OA7052AA	224	198
ESC_OA7050AA	58	198
ESC_OA7048AA	641	935
ESC_OA7045AA	58	198
ESC_OA7044AA	58	198
ESC_OA7043AA	224	198
ESC_OA7039AA	58	198
ESC_OA7038AA	224	198
ESC_OA7037AA	641	935
ESC_OA7036AA	641	935
ESC_OA7035AA	345	175
ESC_OA7034AA	6021	234
ESC_OA7033AA	58	198
ESC_OA7032AA	1704	257
ESC_OA7031AA	10391	825
ESC_OA7030AA	641	935
ESC_OA7029AA	10387	175
ESC_OA7028AA	10390	257
ESC_OA7027AA	641	935
ESC_OA7026AA	641	935
ESC_OA7025AA	2165	257
ESC_OA7024AA	10389	198
ESC_OA7023AA	88	160
ESC_OA7022AA	88	160
ESC_OA7021AA	88	160
ESC_OA7020AA	88	160
ESC_OA7019AA	871	447
ESC_OA7018AA	10387	175
ESC_OA7016AA	6186	868
ESC_OA7015AA	6186	868
ESC_OA7014AA	6186	868
ESC_OA7013AA	6186	868

ESC_OA7012AA	6186	868
ESC_OA7011AA	6186	868
ESC_OA7010AA	6186	868
ESC_OA7009AA	165	504
ESC_OA7008AA	6186	868
ESC_OA7007AA	1727	257
ESC_OA7006AA	6186	868
ESC_OA7005AA	6186	868
ESC_OA7004AA	6186	868
ESC_OA7003AA	165	504
ESC_OA7002AA	6186	868
ESC_OA7001AA	6186	868
ESC_OA7000AA	6186	868
ESC_OA6999AA	165	504
ESC_OA6998AA	165	504
ESC_OA6997AA	6186	868
ESC_OA6996AA	641	935
ESC_OA6995AA	6186	868
ESC_OA6994AA	165	504
ESC_OA6993AA	6186	868
ESC_OA6992AA	6186	868
ESC_OA6991AA	6186	868
ESC_OA6990AA	6186	868
ESC_OA6989AA	6186	868
ESC_OA6988AA	6186	868
ESC_OA6987AA	6186	868
ESC_OA6986AA	6186	868
ESC_OA6985AA	1727	257
ESC_OA6984AA	6186	868
ESC_OA6983AA	6186	868
ESC_OA6982AA	6186	868
ESC_OA6981AA	6186	868
ESC_OA6980AA	6186	868
ESC_OA6979AA	6186	868
ESC_OA6978AA	6186	868
ESC_OA6977AA	6186	868
ESC_OA6976AA	6186	868
ESC_OA6975AA	6186	868
ESC_OA6974AA	88	160
ESC_OA6973AA	6186	868
ESC_OA6972AA	6186	868
ESC_OA6971AA	6186	868
ESC_OA6970AA	6186	868
ESC_OA6969AA	5891	878
ESC_OA6968AA	5891	878
ESC_OA6967AA	5891	878
ESC_OA6966AA	8677	483
ESC_OA6965AA	165	504
ESC_OA6964AA	165	504
ESC_OA6963AA	641	935

ESC_OA6962AA	165	504
ESC_OA6961AA	165	504
ESC_OA6960AA	457	595
ESC_OA6959AA	212	198
ESC_OA6958AA	69	387
ESC_OA6957AA	165	504
ESC_OA6956AA	165	504
ESC_OA6955AA	165	504
ESC_OA6954AA	165	504
ESC_OA6953AA	165	504
ESC_OA6952AA	2165	257
ESC_OA6951AA	165	504
ESC_OA6950AA	8677	483
ESC_OA6949AA	165	504
ESC_OA6948AA	4684	257
ESC_OA6947AA	457	595
ESC_OA6946AA	165	504
ESC_OA6945AA	4684	257
ESC_OA6944AA	4684	257
ESC_OA6943AA	297	234
ESC_OA6942AA	165	504
ESC_OA6927AA	11	936
ESC_OA6926AA	11	936
ESC_OA6925AA	16	156
ESC_OA6924AA	32	270
ESC_OA6923AA	2521	257
ESC_OA6921AA	48	76
ESC_OA6920AA	3601	653
ESC_OA6919AA	3301	164
ESC_OA6918AA	48	76
ESC_OA6917AA	5208	789
ESC_OA6916AA	349	670
ESC_OA6915AA	540	286
ESC_OA6914AA	2541	878
ESC_OA6913AA	10	512
ESC_OA6912AA	101	696
ESC_OA6911AA	10	512
ESC_OA6910AA	847	257
ESC_OA6909AA	212	198
ESC_OA6908AA	1421	447
ESC_OA6907AA	10064	711
ESC_OA6906AA	1426	160
ESC_OA6905AA	847	257
ESC_OA6904AA	345	175
ESC_OA6903AA	48	76
ESC_OA6902AA	6488	198
ESC_OA6901AA	88	160
ESC_OA6900AA	73	737
ESC_OA6899AA	101	696
ESC_OA6898AA	155	175

ESC_OA6897AA	224	198
ESC_OA6896AA	973	968
ESC_OA6895AA	131	653
ESC_OA6894AA	1844	198
ESC_OA6893AA	10	512
ESC_OA6892AA	5340	826
ESC_OA6891AA	963	669
ESC_OA6890AA	540	286
ESC_OA6888AA	57	50
ESC_OA6887AA	57	50
ESC_OA6886AA	88	160
ESC_OA6885AA	297	234
ESC_OA6884AA	2521	257
ESC_OA6883AA	58	198
ESC_OA6882AA	23	156
ESC_OA6881AA	23	156
ESC_OA6880AA	58	198
ESC_OA6879AA	5130	512
ESC_OA6878AA	542	824
ESC_OA6877AA	10	512
ESC_OA6876AA	10	512
ESC_OA6875AA	362	234
ESC_OA6874AA	540	286
ESC_OA6873AA	10	512
ESC_OA6872AA	12	626
ESC_OA6871AA	224	198
ESC_OA6870AA	1308	257
ESC_OA6869AA	156	175
ESC_OA6868AA	95	738
ESC_OA6867AA	12	626
ESC_OA6866AA	405	923
ESC_OA6865AA	540	286
ESC_OA6864AA	405	923
ESC_OA6863AA	10	512
ESC_OA6862AA	162	924
ESC_OA6861AA	162	924
ESC_OA6860AA	10	512
ESC_OA6859AA	457	595
ESC_OA6858AA	5130	512
ESC_OA6857AA	4981	473
ESC_OA6856AA	540	286
ESC_OA6855AA	10	512
ESC_OA6854AA	131	653
ESC_OA6853AA	5155	238
ESC_OA6852AA	457	595
ESC_OA6851AA	155	175
ESC_OA6850AA	10	512
ESC_OA6849AA	10395	817
ESC_OA6848AA	1421	447
ESC_OA6846AA	10	512

ESC_OA6845AA	540	286
ESC_OA6844AA	398	445
ESC_OA6843AA	297	234
ESC_OA6842AA	405	923
ESC_OA6840AA	998	644
ESC_OA6839AA	998	644
ESC_OA6838AA	350	50
ESC_OA6837AA	998	644
ESC_OA6836AA	58	198
ESC_OA6835AA	1722	599
ESC_OA6834AA	10	512
ESC_OA6833AA	58	198
ESC_OA6832AA	998	644
ESC_OA6831AA	1421	447
ESC_OA6830AA	10394	198
ESC_OA6829AA	58	198
ESC_OA6828AA	963	669
ESC_OA6827AA	540	286
ESC_OA6826AA	540	286
ESC_OA6825AA	12	626
ESC_OA6824AA	10	512
ESC_OA6823AA	155	175
ESC_OA6822AA	4981	473
ESC_OA6821AA	354	15
ESC_OA6820AA	10	512
ESC_OA6819AA	1642	198
ESC_OA6818AA	973	968
ESC_OA6817AA	963	669
ESC_OA6816AA	744	512
ESC_OA6815AA	10	512
ESC_OA6814AA	38	669
ESC_OA6813AA	2175	212
ESC_OA6812AA	1304	257
ESC_OA6811AA	10393	198
ESC_OA6810AA	212	198
ESC_OA6809AA	2690	76
ESC_OA6808AA	117	592
ESC_OA6807AA	3519	101
ESC_OA6806AA	88	160
ESC_OA6805AA	9065	233
ESC_OA6804AA	10	512
ESC_OA6803AA	212	198
ESC_OA6802AA	457	595
ESC_OA6801AA	10	512
ESC_OA6800AA	1011	198
ESC_OA6799AA	48	76
ESC_OA6798AA	10	512
ESC_OA6797AA	10396	504
ESC_OA6796AA	155	175
ESC_OA6795AA	1955	900

ESC_OA6794AA	12	626
ESC_OA6793AA	58	198
ESC_OA6792AA	1642	198
ESC_OA6791AA	88	160
ESC_OA6790AA	354	15
ESC_OA6789AA	354	15
ESC_OA6788AA	38	669
ESC_OA6787AA	12	626
ESC_OA6786AA	69	387
ESC_OA6785AA	10	512
ESC_OA6784AA	1884	234
ESC_OA6783AA	963	669
ESC_OA6782AA	2541	878
ESC_OA6781AA	69	387
ESC_OA6780AA	442	257
ESC_OA6779AA	69	387
ESC_OA6778AA	58	198
ESC_OA6777AA	58	198
ESC_OA6776AA	69	387
ESC_OA6775AA	34	504
ESC_OA6774AA	48	76
ESC_OA6773AA	10	512
ESC_OA6772AA	69	387
ESC_OA6771AA	69	387
ESC_OA6770AA	10397	257
ESC_OA6769AA	88	160
ESC_OA6768AA	3232	198
ESC_OA6767AA	58	198
ESC_OA6766AA	58	198
ESC_OA6765AA	46	447
ESC_OA6764AA	297	234
ESC_OA6763AA	540	286
ESC_OA6762AA	12	626
ESC_OA6761AA	345	175
ESC_OA6760AA	345	175
ESC_OA6759AA	11	936
ESC_OA6758AA	11	936
ESC_OA6757AA	200	198
ESC_OA6756AA	630	261
ESC_OA6755AA	630	261
ESC_OA6754AA	630	261
ESC_OA6753AA	245	261
ESC_OA6752AA	245	261
ESC_OA6751AA	630	261
ESC_OA6750AA	630	261
ESC_OA6749AA	630	261
ESC_OA6748AA	245	261
ESC_OA6747AA	630	261
ESC_OA6746AA	630	261
ESC_OA6745AA	630	261

ESC_OA6744AA	630	261
ESC_OA6743AA	245	261
ESC_OA6742AA	245	261
ESC_OA6741AA	630	261
ESC_OA6740AA	630	261
ESC_OA6739AA	630	261
ESC_OA6738AA	630	261
ESC_OA6737AA	245	261
ESC_OA6736AA	630	261
ESC_OA6735AA	245	261
ESC_OA6734AA	630	261
ESC_OA6733AA	245	261
ESC_OA6732AA	245	261
ESC_OA6731AA	245	261
ESC_OA6730AA	245	261
ESC_OA6729AA	630	261
ESC_OA6728AA	245	261
ESC_OA6727AA	245	261
ESC_OA6726AA	245	261
ESC_OA6725AA	152	257
ESC_OA6724AA	152	257
ESC_OA6723AA	152	257
ESC_OA6715AA	11	936
ESC_OA6714AA	17	191
ESC_OA6713AA	90	156
ESC_OA6712AA	765	156
ESC_OA6711AA	2522	198
ESC_OA6710AA	17	191
ESC_OA6709AA	17	191
ESC_OA6708AA	155	175
ESC_OA6707AA	297	234
ESC_OA6706AA	101	696
ESC_OA6705AA	641	935
ESC_OA6704AA	1656	175
ESC_OA6703AA	533	198
ESC_OA6702AA	187	257
ESC_OA6701AA	86	935
ESC_OA6700AA	69	387
ESC_OA6699AA	173	254
ESC_OA6696AA	93	71
ESC_OA6694AA	245	261
ESC_OA6693AA	152	257
ESC_OA6692AA	152	257
ESC_OA6691AA	152	257
ESC_OA6690AA	245	261
ESC_OA6689AA	245	261
ESC_OA6688AA	152	257
ESC_OA6687AA	245	261
ESC_OA6686AA	152	257
ESC_OA6685AA	152	257

ESC_OA6684AA	245	261
ESC_OA6683AA	152	257
ESC_OA6682AA	152	257
ESC_OA6681AA	245	261
ESC_OA6680AA	152	257
ESC_OA6679AA	245	261
ESC_OA6678AA	245	261
ESC_OA6677AA	152	257
ESC_OA6676AA	152	257
ESC_OA6675AA	245	261
ESC_OA6674AA	152	257
ESC_OA6673AA	152	257
ESC_OA6672AA	243	197
ESC_OA6671AA	245	261
ESC_OA6670AA	152	257
ESC_OA6669AA	152	257
ESC_OA6668AA	5475	256
ESC_OA6666AA	101	696
ESC_OA6665AA	11	936
ESC_OA6664AA	17	191
ESC_OA6663AA	11	936
ESC_OA6661AA	2387	261
ESC_OA6660AA	11	936
ESC_OA6659AA	115	669
ESC_OA6658AA	10	512
ESC_OA6657AA	10	512
ESC_OA6656AA	10	512
ESC_OA6655AA	10	512
ESC_OA6654AA	10	512
ESC_OA6653AA	10	512
ESC_OA6652AA	10	512
ESC_OA6651AA	10	512
ESC_OA6650AA	10	512
ESC_OA6649AA	10	512
ESC_OA6648AA	10	512
ESC_OA6647AA	10	512
ESC_OA6646AA	10	512
ESC_OA6645AA	10	512
ESC_OA6644AA	10	512
ESC_OA6643AA	10	512
ESC_OA6642AA	10	512
ESC_OA6641AA	10	512
ESC_OA6640AA	10	512
ESC_OA6639AA	10	512
ESC_OA6638AA	10	512
ESC_OA6637AA	33	198
ESC_OA6636AA	3101	160
ESC_OA6635AA	21	156
ESC_OA6634AA	11	936
ESC_OA6633AA	655	926

ESC_OA6632AA	11	936
ESC_OA6631AA	300	286
ESC_OA6630AA	6880	726
ESC_OA6629AA	11	936
ESC_OA6628AA	7006	936
ESC_OA6627AA	3101	160
ESC_OA6626AA	442	257
ESC_OA6625AA	11	936
ESC_OA6624AA	11	936
ESC_OA6623AA	11	936
ESC_OA6622AA	11	936
ESC_OA6621AA	21	156
ESC_OA6620AA	11	936
ESC_OA6619AA	11	936
ESC_OA6618AA	21	156
ESC_OA6617AA	11	936
ESC_OA6616AA	11	936
ESC_OA6615AA	442	257
ESC_OA6614AA	21	156
ESC_OA6613AA	11	936
ESC_OA6612AA	252	190
ESC_OA6611AA	630	261
ESC_OA6610AA	630	261
ESC_OA6609AA	245	261
ESC_OA6608AA	10385	270
ESC_OA6606AA	10349	512
ESC_OA6605AA	401	142
ESC_OA6604AA	540	286
ESC_OA6603AA	11	936
ESC_OA6602AA	301	307
ESC_OA6601AA	301	307
ESC_OA6600AA	21	156
ESC_OA6599AA	29	156
ESC_OA6598AA	1408	445
ESC_OA6597AA	1421	447
ESC_OA6596AA	10348	445
ESC_OA6595AA	48	76
ESC_OA6594AA	1312	76
ESC_OA6593AA	167	512
ESC_OA6592AA	1290	515
ESC_OA6591AA	617	511
ESC_OA6590AA	602	257
ESC_OA6589AA	245	261
ESC_OA6588AA	152	257
ESC_OA6587AA	152	257
ESC_OA6586AA	245	261
ESC_OA6585AA	245	261
ESC_OA6584AA	48	76
ESC_OA6583AA	21	156
ESC_OA6582AA	442	257

ESC_OA6581AA	21	156
ESC_OA6580AA	33	198
ESC_OA6579AA	10	512
ESC_OA6578AA	675	234
ESC_OA6577AA	33	198
ESC_OA6576AA	655	926
ESC_OA6575AA	301	307
ESC_OA6574AA	442	257
ESC_OA6573AA	167	512
ESC_OA6572AA	10	512
ESC_OA6571AA	3268	669
ESC_OA6570AA	38	669
ESC_OA6569AA	540	286
ESC_OA6568AA	648	359
ESC_OA6567AA	617	511
ESC_OA6566AA	8148	1004
ESC_OA6565AA	46	447
ESC_OA6564AA	1408	445
ESC_OA6563AA	10	512
ESC_OA6562AA	8308	817
ESC_OA6561AA	156	175
ESC_OA6560AA	156	175
ESC_OA6559AA	2450	447
ESC_OA6558AA	131	653
ESC_OA6557AA	131	653
ESC_OA6556AA	131	653
ESC_OA6555AA	131	653
ESC_OA6554AA	131	653
ESC_OA6553AA	648	359
ESC_OA6552AA	405	923
ESC_OA6551AA	131	653
ESC_OA6550AA	131	653
ESC_OA6549AA	1304	257
ESC_OA6548AA	131	653
ESC_OA6547AA	4038	257
ESC_OA6546AA	405	923
ESC_OA6545AA	131	653
ESC_OA6544AA	117	592
ESC_OA6543AA	491	626
ESC_OA6541AA	354	15
ESC_OA6540AA	38	669
ESC_OA6539AA	131	653
ESC_OA6538AA	405	923
ESC_OA6537AA	131	653
ESC_OA6536AA	131	653
ESC_OA6535AA	131	653
ESC_OA6534AA	405	923
ESC_OA6533AA	624	359
ESC_OA6532AA	38	669
ESC_OA6531AA	405	923

ESC_OA6530AA	354	15
ESC_OA6529AA	457	595
ESC_OA6528AA	131	653
ESC_OA6527AA	117	592
ESC_OA6526AA	131	653
ESC_OA6525AA	641	935
ESC_OA6524AA	405	923
ESC_OA6523AA	224	198
ESC_OA6522AA	131	653
ESC_OA6521AA	131	653
ESC_OA6520AA	410	156
ESC_OA6519AA	131	653
ESC_OA6518AA	624	359
ESC_OA6517AA	156	175
ESC_OA6516AA	131	653
ESC_OA6515AA	405	923
ESC_OA6514AA	127	738
ESC_OA6513AA	162	924
ESC_OA6512AA	681	393
ESC_OA6511AA	635	141
ESC_OA6509AA	603	198
ESC_OA6508AA	117	592
ESC_OA6507AA	117	592
ESC_OA6506AA	12	626
ESC_OA6505AA	141	644
ESC_OA6504AA	12	626
ESC_OA6503AA	10347	445
ESC_OA6502AA	73	737
ESC_OA6501AA	131	653
ESC_OA6500AA	141	644
ESC_OA6499AA	372	424
ESC_OA6498AA	118	985
ESC_OA6497AA	723	156
ESC_OA6496AA	372	424
ESC_OA6495AA	131	653
ESC_OA6494AA	16	156
ESC_OA6493AA	9237	938
ESC_OA6492AA	1670	438
ESC_OA6491AA	127	738
ESC_OA6490AA	12	626
ESC_OA6489AA	515	1004
ESC_OA6488AA	127	738
ESC_OA6487AA	1730	234
ESC_OA6486AA	399	198
ESC_OA6485AA	4663	257
ESC_OA6484AA	10	512
ESC_OA6483AA	448	257
ESC_OA6482AA	101	696
ESC_OA6481AA	167	512
ESC_OA6480AA	448	257

ESC_OA6479AA	101	696
ESC_OA6478AA	410	156
ESC_OA6476AA	152	257
ESC_OA6474AA	245	261
ESC_OA6473AA	245	261
ESC_OA6472AA	152	257
ESC_OA6471AA	152	257
ESC_OA6470AA	245	261
ESC_OA6465AA	245	261
ESC_OA6463AA	10	512
ESC_OA6462AA	21	156
ESC_OA6460AA	21	156
ESC_OA6456AA	404	761
ESC_OA6455AA	738	598
ESC_OA6454AA	21	156
ESC_OA6453AA	6	445
ESC_OA6452AA	11	936
ESC_OA6451AA	21	156
ESC_OA6450AA	11	936
ESC_OA6445AA	21	156
ESC_OA6442AA	29	156
ESC_OA6441AA	399	198
ESC_OA6440AA	115	669
ESC_OA6439AA	1844	198
ESC_OA6438AA	88	160
ESC_OA6437AA	162	924
ESC_OA6436AA	504	738
ESC_OA6434AA	33	198
ESC_OA6433AA	245	261
ESC_OA6432AA	587	936
ESC_OA6431AA	11	936
ESC_OA6430AA	99	198
ESC_OA6429AA	11	936
ESC_OA6428AA	11	936
ESC_OA6427AA	11	936
ESC_OA6426AA	706	422
ESC_OA6425AA	5579	175
ESC_OA6424AA	765	156
ESC_OA6423AA	47	307
ESC_OA6422AA	155	175
ESC_OA6421AA	212	198
ESC_OA6420AA	345	175
ESC_OA6419AA	10	512
ESC_OA6418AA	101	696
ESC_OA6417AA	405	923
ESC_OA6416AA	2705	504
ESC_OA6415AA	162	924
ESC_OA6414AA	2556	630
ESC_OA6413AA	38	669
ESC_OA6412AA	2175	212

ESC_OA6411AA	5451	912
ESC_OA6410AA	10	512
ESC_OA6409AA	155	175
ESC_OA6408AA	399	198
ESC_OA6407AA	3602	234
ESC_OA6406AA	1423	198
ESC_OA6405AA	187	257
ESC_OA6404AA	1737	243
ESC_OA6403AA	5014	257
ESC_OA6402AA	648	359
ESC_OA6401AA	8584	286
ESC_OA6400AA	446	257
ESC_OA6399AA	1081	198
ESC_OA6398AA	6188	257
ESC_OA6397AA	2025	194
ESC_OA6396AA	201	257
ESC_OA6395AA	11	936
ESC_OA6394AA	162	924
ESC_OA6393AA	10346	108
ESC_OA6392AA	17	191
ESC_OA6391AA	11	936
ESC_OA6390AA	648	359
ESC_OA6389AA	278	924
ESC_OA6388AA	9528	820
ESC_OA6387AA	58	198
ESC_OA6386AA	164	175
ESC_OA6385AA	5971	979
ESC_OA6384AA	423	204
ESC_OA6383AA	17	191
ESC_OA6382AA	152	257
ESC_OA6380AA	1025	261
ESC_OA6379AA	245	261
ESC_OA6378AA	152	257
ESC_OA6377AA	145	256
ESC_OA6376AA	243	197
ESC_OA6375AA	245	261
ESC_OA6374AA	17	191
ESC_OA6373AA	6060	41
ESC_OA6372AA	25	234
ESC_OA6371AA	33	198
ESC_OA6370AA	442	257
ESC_OA6369AA	21	156
ESC_OA6368AA	10	512
ESC_OA6367AA	21	156
ESC_OA6366AA	21	156
ESC_OA6365AA	29	156
ESC_OA6364AA	21	156
ESC_OA6363AA	5308	261
ESC_OA6362AA	616	156
ESC_OA6361AA	101	696

ESC_OA6360AA	428	654
ESC_OA6359AA	8324	257
ESC_OA6358AA	401	142
ESC_OA6357AA	1079	235
ESC_OA6356AA	278	924
ESC_OA6355AA	10	512
ESC_OA6354AA	1079	235
ESC_OA6353AA	58	198
ESC_OA6352AA	9002	257
ESC_OA6351AA	69	387
ESC_OA6319AA	297	234
ESC_OA6318AA	1431	257
ESC_OA6317AA	1431	257
ESC_OA6316AA	101	696
ESC_OA6315AA	648	359
ESC_OA6314AA	648	359
ESC_OA6313AA	4450	844
ESC_OA6311AA	361	512
ESC_OA6310AA	101	696
ESC_OA6309AA	101	696
ESC_OA6308AA	361	512
ESC_OA6307AA	361	512
ESC_OA6306AA	448	257
ESC_OA6305AA	167	512
ESC_OA6304AA	448	257
ESC_OA6302AA	38	669
ESC_OA6300AA	131	653
ESC_OA6299AA	131	653
ESC_OA6298AA	131	653
ESC_OA6297AA	131	653
ESC_OA6296AA	131	653
ESC_OA6295AA	131	653
ESC_OA6294AA	131	653
ESC_OA6293AA	131	653
ESC_OA6292AA	131	653
ESC_OA6291AA	131	653
ESC_OA6290AA	245	261
ESC_OA6289AA	152	257
ESC_OA6288AA	152	257
ESC_OA6287AA	243	197
ESC_OA6286AA	152	257
ESC_OA6285AA	245	261
ESC_OA6284AA	245	261
ESC_OA6283AA	245	261
ESC_OA6282AA	245	261
ESC_OA6281AA	245	261
ESC_OA6280AA	245	261
ESC_OA6279AA	245	261
ESC_OA6278AA	148	191
ESC_OA6277AA	152	257

ESC_OA6276AA	148	191
ESC_OA6275AA	152	257
ESC_OA6274AA	6	445
ESC_OA6273AA	21	156
ESC_OA6272AA	5431	0
ESC_OA6271AA	583	768
ESC_OA6270AA	675	234
ESC_OA6269AA	17	191
ESC_OA6268AA	182	246
ESC_OA6267AA	11	936
ESC_OA6266AA	21	156
ESC_OA6265AA	40	198
ESC_OA6264AA	583	768
ESC_OA6263AA	11	936
ESC_OA6262AA	11	936
ESC_OA6261AA	1491	76
ESC_OA6260AA	4638	123
ESC_OA6259AA	11	936
ESC_OA6258AA	33	198
ESC_OA6257AA	665	79
ESC_OA6256AA	93	71
ESC_OA6255AA	770	587
ESC_OA6254AA	410	156
ESC_OA6253AA	752	512
ESC_OA6252AA	3778	592
ESC_OA6251AA	8570	76
ESC_OA6250AA	23	156
ESC_OA6249AA	117	592
ESC_OA6248AA	58	198
ESC_OA6247AA	350	50
ESC_OA6246AA	10	512
ESC_OA6245AA	57	50
ESC_OA6244AA	57	50
ESC_OA6243AA	10	512
ESC_OA6242AA	34	504
ESC_OA6241AA	58	198
ESC_OA6240AA	58	198
ESC_OA6239AA	3778	592
ESC_OA6238AA	1818	71
ESC_OA6237AA	602	257
ESC_OA6236AA	359	696
ESC_OA6235AA	3249	156
ESC_OA6234AA	371	50
ESC_OA6233AA	101	696
ESC_OA6232AA	602	257
ESC_OA6231AA	602	257
ESC_OA6230AA	8567	512
ESC_OA6229AA	602	257
ESC_OA6228AA	57	50
ESC_OA6227AA	1158	387

ESC_OA6226AA	117	592
ESC_OA6225AA	117	592
ESC_OA6224AA	1158	387
ESC_OA6223AA	58	198
ESC_OA6222AA	57	50
ESC_OA6221AA	117	592
ESC_OA6220AA	58	198
ESC_OA6219AA	117	592
ESC_OA6218AA	88	160
ESC_OA6217AA	10	512
ESC_OA6216AA	5897	912
ESC_OA6215AA	10	512
ESC_OA6214AA	373	71
ESC_OA6213AA	10	512
ESC_OA6212AA	744	512
ESC_OA6211AA	88	160
ESC_OA6210AA	162	924
ESC_OA6209AA	752	512
ESC_OA6208AA	93	71
ESC_OA6207AA	115	669
ESC_OA6206AA	580	445
ESC_OA6205AA	1163	579
ESC_OA6204AA	8330	461
ESC_OA6203AA	648	359
ESC_OA6201AA	117	592
ESC_OA6200AA	117	592
ESC_OA6199AA	38	669
ESC_OA6198AA	10	512
ESC_OA6197AA	48	76
ESC_OA6196AA	226	512
ESC_OA6195AA	997	915
ESC_OA6194AA	189	504
ESC_OA6193AA	10	512
ESC_OA6192AA	350	50
ESC_OA6191AA	1072	355
ESC_OA6190AA	38	669
ESC_OA6189AA	1564	512
ESC_OA6188AA	10113	50
ESC_OA6187AA	4663	257
ESC_OA6186AA	1800	924
ESC_OA6185AA	57	50
ESC_OA6184AA	10	512
ESC_OA6183AA	752	512
ESC_OA6182AA	23	156
ESC_OA6181AA	117	592
ESC_OA6180AA	117	592
ESC_OA6179AA	117	592
ESC_OA6178AA	88	160
ESC_OA6177AA	117	592
ESC_OA6176AA	117	592

ESC_OA6175AA	4663	257
ESC_OA6174AA	117	592
ESC_OA6173AA	5183	402
ESC_OA6172AA	1072	355
ESC_OA6171AA	162	924
ESC_OA6170AA	117	592
ESC_OA6169AA	69	387
ESC_OA6168AA	69	387
ESC_OA6167AA	189	504
ESC_OA6166AA	5183	402
ESC_OA6165AA	69	387
ESC_OA6164AA	117	592
ESC_OA6163AA	8572	285
ESC_OA6162AA	3778	592
ESC_OA6161AA	117	592
ESC_OA6160AA	1304	257
ESC_OA6159AA	135	653
ESC_OA6158AA	1463	263
ESC_OA6157AA	117	592
ESC_OA6156AA	2607	198
ESC_OA6155AA	1818	71
ESC_OA6154AA	117	592
ESC_OA6153AA	10	512
ESC_OA6152AA	117	592
ESC_OA6151AA	38	669
ESC_OA6150AA	88	160
ESC_OA6149AA	10	512
ESC_OA6148AA	69	387
ESC_OA6147AA	117	592
ESC_OA6146AA	602	257
ESC_OA6145AA	10	512
ESC_OA6144AA	48	76
ESC_OA6143AA	69	387
ESC_OA6142AA	1564	512
ESC_OA6141AA	117	592
ESC_OA6140AA	770	587
ESC_OA6139AA	88	160
ESC_OA6138AA	57	50
ESC_OA6137AA	206	286
ESC_OA6136AA	10	512
ESC_OA6135AA	359	696
ESC_OA6134AA	7204	522
ESC_OA6133AA	665	79
ESC_OA6132AA	1818	71
ESC_OA6131AA	1818	71
ESC_OA6130AA	156	175
ESC_OA6129AA	189	504
ESC_OA6128AA	69	387
ESC_OA6127AA	58	198
ESC_OA6126AA	602	257

ESC_OA6125AA	88	160
ESC_OA6124AA	602	257
ESC_OA6123AA	10	512
ESC_OA6122AA	1670	438
ESC_OA6121AA	602	257
ESC_OA6120AA	602	257
ESC_OA6119AA	1304	257
ESC_OA6118AA	3994	512
ESC_OA6117AA	117	592
ESC_OA6116AA	695	519
ESC_OA6115AA	155	175
ESC_OA6114AA	3778	592
ESC_OA6113AA	88	160
ESC_OA6112AA	5183	402
ESC_OA6111AA	2973	234
ESC_OA6110AA	1158	387
ESC_OA6109AA	602	257
ESC_OA6108AA	93	71
ESC_OA6107AA	101	696
ESC_OA6106AA	602	257
ESC_OA6105AA	57	50
ESC_OA6104AA	2040	198
ESC_OA6103AA	997	915
ESC_OA6102AA	57	50
ESC_OA6101AA	354	15
ESC_OA6100AA	117	592
ESC_OA6099AA	752	512
ESC_OA6098AA	1730	234
ESC_OA6097AA	665	79
ESC_OA6096AA	10	512
ESC_OA6095AA	117	592
ESC_OA6094AA	8571	512
ESC_OA6093AA	1771	868
ESC_OA6092AA	5420	71
ESC_OA6091AA	117	592
ESC_OA6090AA	665	79
ESC_OA6089AA	117	592
ESC_OA6088AA	10	512
ESC_OA6087AA	752	512
ESC_OA6086AA	117	592
ESC_OA6085AA	212	198
ESC_OA6084AA	117	592
ESC_OA6083AA	8573	867
ESC_OA6082AA	115	669
ESC_OA6081AA	155	175
ESC_OA6080AA	117	592
ESC_OA6079AA	88	160
ESC_OA6078AA	10	512
ESC_OA6077AA	88	160
ESC_OA6076AA	602	257

ESC_OA6075AA	43	512
ESC_OA6074AA	212	198
ESC_OA6073AA	1158	387
ESC_OA6072AA	117	592
ESC_OA6071AA	602	257
ESC_OA6070AA	117	592
ESC_OA6069AA	351	268
ESC_OA6068AA	602	257
ESC_OA6067AA	8262	234
ESC_OA6066AA	1640	50
ESC_OA6065AA	6023	71
ESC_OA6064AA	155	175
ESC_OA6063AA	453	935
ESC_OA6062AA	38	669
ESC_OA6061AA	162	924
ESC_OA6060AA	753	842
ESC_OA6059AA	752	512
ESC_OA6058AA	354	15
ESC_OA6057AA	115	669
ESC_OA6056AA	117	592
ESC_OA6055AA	10	512
ESC_OA6054AA	10	512
ESC_OA6053AA	602	257
ESC_OA6052AA	11	936
ESC_OA6051AA	11	936
ESC_OA6050AA	11	936
ESC_OA6049AA	752	512
ESC_OA6048AA	11	936
ESC_OA6047AA	11	936
ESC_OA6046AA	11	936
ESC_OA6045AA	219	877
ESC_OA6044AA	10344	669
ESC_OA6043AA	101	696
ESC_OA6042AA	38	669
ESC_OA6041AA	4681	924
ESC_OA6040AA	10344	669
ESC_OA6039AA	46	447
ESC_OA6038AA	10344	669
ESC_OA6037AA	746	789
ESC_OA6036AA	3052	669
ESC_OA6035AA	6311	512
ESC_OA6034AA	245	261
ESC_OA6033AA	152	257
ESC_OA6032AA	145	256
ESC_OA6031AA	10	512
ESC_OA6030AA	11	936
ESC_OA6029AA	11	936
ESC_OA6028AA	11	936
ESC_OA6027AA	11	936
ESC_OA6026AA	11	936

ESC_OA6025AA	11	936
ESC_OA6024AA	11	936
ESC_OA6023AA	11	936
ESC_OA6022AA	11	936
ESC_OA6021AA	11	936
ESC_OA6020AA	11	936
ESC_OA6019AA	11	936
ESC_OA6018AA	11	936
ESC_OA6017AA	11	936
ESC_OA6016AA	245	261
ESC_OA6015AA	245	261
ESC_OA6014AA	152	257
ESC_OA6013AA	152	257
ESC_OA6012AA	40	198
ESC_OA6011AA	17	191
ESC_OA6010AA	937	198
ESC_OA6009AA	4550	150
ESC_OA6008AA	11	936
ESC_OA6007AA	11	936
ESC_OA6006AA	11	936
ESC_OA6005AA	11	936
ESC_OA6004AA	11	936
ESC_OA6003AA	11	936
ESC_OA6002AA	11	936
ESC_OA6001AA	1486	71
ESC_OA6000AA	117	592
ESC_OA5999AA	10341	76
ESC_OA5998AA	2705	504
ESC_OA5997AA	48	76
ESC_OA5996AA	175	311
ESC_OA5995AA	48	76
ESC_OA5994AA	2473	257
ESC_OA5993AA	155	175
ESC_OA5992AA	7585	286
ESC_OA5991AA	1431	257
ESC_OA5990AA	6616	76
ESC_OA5989AA	6395	504
ESC_OA5988AA	906	198
ESC_OA5987AA	355	764
ESC_OA5986AA	10343	924
ESC_OA5985AA	5584	640
ESC_OA5984AA	5474	198
ESC_OA5983AA	6949	765
ESC_OA5982AA	58	198
ESC_OA5981AA	602	257
ESC_OA5980AA	10	512
ESC_OA5979AA	1642	198
ESC_OA5977AA	1196	257
ESC_OA5976AA	93	71
ESC_OA5975AA	4638	123

ESC_OA5974AA	10	512
ESC_OA5973AA	409	504
ESC_OA5971AA	607	355
ESC_OA5970AA	173	254
ESC_OA5969AA	131	653
ESC_OA5968AA	1291	512
ESC_OA5967AA	720	900
ESC_OA5966AA	3580	257
ESC_OA5965AA	23	156
ESC_OA5964AA	5135	278
ESC_OA5963AA	871	447
ESC_OA5962AA	192	696
ESC_OA5961AA	10	512
ESC_OA5960AA	154	257
ESC_OA5959AA	336	878
ESC_OA5958AA	6416	1004
ESC_OA5957AA	187	257
ESC_OA5956AA	388	156
ESC_OA5955AA	2144	257
ESC_OA5954AA	6162	337
ESC_OA5953AA	48	76
ESC_OA5952AA	1727	257
ESC_OA5951AA	58	198
ESC_OA5950AA	4038	257
ESC_OA5949AA	109	257
ESC_OA5948AA	4080	300
ESC_OA5947AA	392	234
ESC_OA5946AA	23	156
ESC_OA5945AA	398	445
ESC_OA5944AA	10342	512
ESC_OA5943AA	3602	234
ESC_OA5942AA	2562	640
ESC_OA5941AA	10	512
ESC_OA5940AA	2175	212
ESC_OA5939AA	442	257
ESC_OA5938AA	366	198
ESC_OA5871AA	10338	198
ESC_OA5870AA	3107	512
ESC_OA5869AA	10338	198
ESC_OA5868AA	10339	587
ESC_OA5867AA	1818	71
ESC_OA5866AA	189	504
ESC_OA5865AA	43	512
ESC_OA5864AA	48	76
ESC_OA5863AA	3107	512
ESC_OA5862AA	43	512
ESC_OA5861AA	1011	198
ESC_OA5860AA	43	512
ESC_OA5859AA	867	198
ESC_OA5858AA	10340	76

ESC_OA5857AA	10338	198
ESC_OA5856AA	43	512
ESC_OA5855AA	602	257
ESC_OA5854AA	867	198
ESC_OA5853AA	359	696
ESC_OA5852AA	43	512
ESC_OA5851AA	206	286
ESC_OA5850AA	1196	257
ESC_OA5849AA	1818	71
ESC_OA5848AA	2705	504
ESC_OA5847AA	10338	198
ESC_OA5846AA	10338	198
ESC_OA5845AA	155	175
ESC_OA5844AA	1324	156
ESC_OA5843AA	178	605
ESC_OA5842AA	354	15
ESC_OA5841AA	43	512
ESC_OA5840AA	1818	71
ESC_OA5839AA	152	257
ESC_OA5838AA	152	257
ESC_OA5837AA	245	261
ESC_OA5836AA	245	261
ESC_OA5835AA	405	923
ESC_OA5834AA	11	936
ESC_OA5833AA	11	936
ESC_OA5832AA	11	936
ESC_OA5831AA	301	307
ESC_OA5830AA	301	307
ESC_OA5829AA	21	156
ESC_OA5828AA	29	156
ESC_OA5827AA	630	261
ESC_OA5826AA	628	261
ESC_OA5825AA	245	261
ESC_OA5824AA	630	261
ESC_OA5823AA	245	261
ESC_OA5822AA	245	261
ESC_OA5821AA	630	261
ESC_OA5820AA	245	261
ESC_OA5819AA	245	261
ESC_OA5818AA	630	261
ESC_OA5817AA	630	261
ESC_OA5816AA	245	261
ESC_OA5815AA	245	261
ESC_OA5814AA	245	261
ESC_OA5813AA	245	261
ESC_OA5812AA	152	257
ESC_OA5811AA	152	257
ESC_OA5810AA	152	257
ESC_OA5809AA	245	261
ESC_OA5808AA	11	936

ESC_OA5807AA	11	936
ESC_OA5806AA	630	261
ESC_OA5805AA	630	261
ESC_OA5804AA	630	261
ESC_OA5803AA	630	261
ESC_OA5802AA	630	261
ESC_OA5801AA	245	261
ESC_OA5800AA	5517	257
ESC_OA5799AA	152	257
ESC_OA5798AA	33	198
ESC_OA5797AA	21	156
ESC_OA5796AA	442	257
ESC_OA5795AA	21	156
ESC_OA5794AA	32	270
ESC_OA5793AA	21	156
ESC_OA5792AA	327	204
ESC_OA5791AA	11	936
ESC_OA5790AA	33	198
ESC_OA5789AA	1201	512
ESC_OA5788AA	448	257
ESC_OA5787AA	245	261
ESC_OA5786AA	245	261
ESC_OA5785AA	152	257
ESC_OA5784AA	152	257
ESC_OA5783AA	152	257
ESC_OA5782AA	245	261
ESC_OA5781AA	152	257
ESC_OA5780AA	245	261
ESC_OA5779AA	245	261
ESC_OA5778AA	152	257
ESC_OA5777AA	245	261
ESC_OA5776AA	152	257
ESC_OA5775AA	245	261
ESC_OA5774AA	243	197
ESC_OA5773AA	187	257
ESC_OA5772AA	23	156
ESC_OA5771AA	2040	198
ESC_OA5770AA	746	789
ESC_OA5769AA	4138	198
ESC_OA5768AA	101	696
ESC_OA5767AA	11	936
ESC_OA5766AA	11	936
ESC_OA5765AA	11	936
ESC_OA5764AA	11	936
ESC_OA5763AA	17	191
ESC_OA5762AA	11	936
ESC_OA5761AA	11	936
ESC_OA5760AA	11	936
ESC_OA5759AA	11	936
ESC_OA5758AA	11	936

ESC_OA5757AA	11	936
ESC_OA5756AA	11	936
ESC_OA5755AA	28	537
ESC_OA5754AA	2346	435
ESC_OA5753AA	10	512
ESC_OA5752AA	40	198
ESC_OA5751AA	336	878
ESC_OA5750AA	3101	160
ESC_OA5749AA	8930	234
ESC_OA5748AA	21	156
ESC_OA5747AA	21	156
ESC_OA5746AA	6	445
ESC_OA5745AA	311	204
ESC_OA5744AA	11	936
ESC_OA5743AA	301	307
ESC_OA5742AA	301	307
ESC_OA5741AA	21	156
ESC_OA5740AA	29	156
ESC_OA5739AA	145	256
ESC_OA5738AA	152	257
ESC_OA5737AA	152	257
ESC_OA5736AA	245	261
ESC_OA5735AA	245	261
ESC_OA5734AA	152	257
ESC_OA5733AA	152	257
ESC_OA5732AA	245	261
ESC_OA5731AA	245	261
ESC_OA5730AA	245	261
ESC_OA5729AA	152	257
ESC_OA5728AA	245	261
ESC_OA5727AA	10330	257
ESC_OA5726AA	152	257
ESC_OA5725AA	245	261
ESC_OA5724AA	145	256
ESC_OA5723AA	245	261
ESC_OA5722AA	245	261
ESC_OA5721AA	152	257
ESC_OA5720AA	10334	935
ESC_OA5719AA	21	156
ESC_OA5718AA	1804	935
ESC_OA5717AA	10333	512
ESC_OA5716AA	101	696
ESC_OA5715AA	11	936
ESC_OA5714AA	1049	175
ESC_OA5713AA	21	156
ESC_OA5712AA	1193	761
ESC_OA5711AA	1193	761
ESC_OA5710AA	131	653
ESC_OA5709AA	131	653
ESC_OA5708AA	648	359

ESC_OA5707AA	131	653
ESC_OA5706AA	131	653
ESC_OA5705AA	131	653
ESC_OA5704AA	12	626
ESC_OA5703AA	131	653
ESC_OA5702AA	131	653
ESC_OA5701AA	131	653
ESC_OA5700AA	131	653
ESC_OA5699AA	10332	653
ESC_OA5698AA	1193	761
ESC_OA5697AA	38	669
ESC_OA5696AA	8412	696
ESC_OA5695AA	718	257
ESC_OA5694AA	718	257
ESC_OA5692AA	648	359
ESC_OA5691AA	847	257
ESC_OA5690AA	212	198
ESC_OA5687AA	648	359
ESC_OA5686AA	648	359
ESC_OA5685AA	648	359
ESC_OA5684AA	117	592
ESC_OA5682AA	59	217
ESC_OA5681AA	38	669
ESC_OA5680AA	38	669
ESC_OA5679AA	429	654
ESC_OA5677AA	11	936
ESC_OA5676AA	33	198
ESC_OA5675AA	33	198
ESC_OA5674AA	10	512
ESC_OA5673AA	11	936
ESC_OA5672AA	11	936
ESC_OA5671AA	25	234
ESC_OA5670AA	398	445
ESC_OA5669AA	11	936
ESC_OA5668AA	11	936
ESC_OA5667AA	3075	701
ESC_OA5666AA	5973	92
ESC_OA5665AA	21	156
ESC_OA5664AA	11	936
ESC_OA5663AA	11	936
ESC_OA5662AA	11	936
ESC_OA5661AA	11	936
ESC_OA5660AA	101	696
ESC_OA5659AA	2966	936
ESC_OA5658AA	21	156
ESC_OA5657AA	21	156
ESC_OA5656AA	21	156
ESC_OA5655AA	21	156
ESC_OA5654AA	655	926
ESC_OA5652AA	2385	198

ESC_OA5651AA	21	156
ESC_OA5650AA	21	156
ESC_OA5649AA	21	156
ESC_OA5648AA	2836	221
ESC_OA5647AA	21	156
ESC_OA5646AA	21	156
ESC_OA5645AA	17	191
ESC_OA5644AA	655	926
ESC_OA5643AA	21	156
ESC_OA5642AA	11	936
ESC_OA5641AA	223	198
ESC_OA5640AA	17	191
ESC_OA5639AA	17	191
ESC_OA5638AA	10336	254
ESC_OA5637AA	11	936
ESC_OA5636AA	675	234
ESC_OA5635AA	21	156
ESC_OA5634AA	173	254
ESC_OA5633AA	332	257
ESC_OA5632AA	32	270
ESC_OA5631AA	11	936
ESC_OA5630AA	101	696
ESC_OA5629AA	993	789
ESC_OA5628AA	11	936
ESC_OA5627AA	21	156
ESC_OA5626AA	675	234
ESC_OA5624AA	10	512
ESC_OA5623AA	10	512
ESC_OA5622AA	33	198
ESC_OA5621AA	11	936
ESC_OA5620AA	10335	198
ESC_OA5619AA	21	156
ESC_OA5618AA	11	936
ESC_OA5617AA	21	156
ESC_OA5616AA	21	156
ESC_OA5615AA	11	936
ESC_OA5614AA	205	257
ESC_OA5613AA	21	156
ESC_OA5612AA	21	156
ESC_OA5611AA	11	936
ESC_OA5610AA	11	936
ESC_OA5609AA	1705	156
ESC_OA5608AA	11	936
ESC_OA5607AA	21	156
ESC_OA5606AA	11	936
ESC_OA5605AA	11	936
ESC_OA5604AA	11	936
ESC_OA5603AA	11	936
ESC_OA5602AA	88	160
ESC_OA5601AA	11	936

ESC_OA5599AA	155	175
ESC_OA5598AA	57	50
ESC_OA5597AA	131	653
ESC_OA5596AA	117	592
ESC_OA5594AA	10328	175
ESC_OA5593AA	57	50
ESC_OA5592AA	43	512
ESC_OA5590AA	354	15
ESC_OA5589AA	10064	711
ESC_OA5588AA	57	50
ESC_OA5587AA	398	445
ESC_OA5585AA	349	670
ESC_OA5584AA	38	669
ESC_OA5583AA	457	595
ESC_OA5581AA	410	156
ESC_OA5580AA	2001	670
ESC_OA5579AA	38	669
ESC_OA5576AA	10	512
ESC_OA5575AA	410	156
ESC_OA5573AA	38	669
ESC_OA5572AA	10	512
ESC_OA5570AA	131	653
ESC_OA5569AA	38	669
ESC_OA5568AA	1011	198
ESC_OA5567AA	1193	761
ESC_OA5566AA	131	653
ESC_OA5565AA	131	653
ESC_OA5564AA	405	923
ESC_OA5563AA	1725	175
ESC_OA5560AA	393	343
ESC_OA5559AA	93	71
ESC_OA5558AA	131	653
ESC_OA5557AA	648	359
ESC_OA5554AA	117	592
ESC_OA5553AA	1196	257
ESC_OA5552AA	354	15
ESC_OA5551AA	131	653
ESC_OA5549AA	57	50
ESC_OA5547AA	1193	761
ESC_OA5546AA	648	359
ESC_OA5544AA	354	15
ESC_OA5543AA	101	696
ESC_OA5541AA	131	653
ESC_OA5540AA	295	160
ESC_OA5539AA	58	198
ESC_OA5538AA	57	50
ESC_OA5537AA	131	653
ESC_OA5536AA	354	15
ESC_OA5534AA	131	653
ESC_OA5533AA	155	175

ESC_OA5532AA	73	737
ESC_OA5531AA	69	387
ESC_OA5530AA	354	15
ESC_OA5527AA	10328	175
ESC_OA5526AA	38	669
ESC_OA5525AA	156	175
ESC_OA5523AA	1196	257
ESC_OA5518AA	38	669
ESC_OA5517AA	1722	599
ESC_OA5516AA	2067	257
ESC_OA5515AA	2067	257
ESC_OA5513AA	746	789
ESC_OA5512AA	93	71
ESC_OA5511AA	101	696
ESC_OA5510AA	542	824
ESC_OA5509AA	3285	257
ESC_OA5508AA	3285	257
ESC_OA5507AA	10	512
ESC_OA5506AA	2067	257
ESC_OA5505AA	457	595
ESC_OA5503AA	515	1004
ESC_OA5502AA	101	696
ESC_OA5501AA	48	76
ESC_OA5500AA	38	669
ESC_OA5499AA	38	669
ESC_OA5498AA	38	669
ESC_OA5497AA	38	669
ESC_OA5496AA	1722	599
ESC_OA5495AA	2067	257
ESC_OA5494AA	69	387
ESC_OA5493AA	2067	257
ESC_OA5492AA	2067	257
ESC_OA5491AA	2067	257
ESC_OA5490AA	2067	257
ESC_OA5489AA	2067	257
ESC_OA5488AA	2067	257
ESC_OA5487AA	648	359
ESC_OA5486AA	131	653
ESC_OA5485AA	73	737
ESC_OA5484AA	131	653
ESC_OA5483AA	131	653
ESC_OA5482AA	131	653
ESC_OA5481AA	617	511
ESC_OA5480AA	131	653
ESC_OA5479AA	131	653
ESC_OA5478AA	131	653
ESC_OA5475AA	10	512
ESC_OA5473AA	10	512
ESC_OA5472AA	86	935
ESC_OA5471AA	9826	519

ESC_OA5470AA	29	156
ESC_OA5469AA	100	504
ESC_OA5468AA	90	156
ESC_OA5467AA	90	156
ESC_OA5466AA	3101	160
ESC_OA5465AA	11	936
ESC_OA5464AA	300	286
ESC_OA5463AA	11	936
ESC_OA5462AA	11	936
ESC_OA5461AA	11	936
ESC_OA5460AA	11	936
ESC_OA5459AA	33	198
ESC_OA5458AA	442	257
ESC_OA5457AA	11	936
ESC_OA5456AA	442	257
ESC_OA5455AA	6130	268
ESC_OA5454AA	10	512
ESC_OA5453AA	11	936
ESC_OA5452AA	11	936
ESC_OA5451AA	10	512
ESC_OA5450AA	675	234
ESC_OA5449AA	11	936
ESC_OA5448AA	10	512
ESC_OA5447AA	1193	761
ESC_OA5446AA	11	936
ESC_OA5445AA	21	156
ESC_OA5444AA	12	626
ESC_OA5443AA	21	156
ESC_OA5442AA	10	512
ESC_OA5441AA	155	175
ESC_OA5440AA	32	270
ESC_OA5439AA	747	640
ESC_OA5438AA	11	936
ESC_OA5437AA	11	936
ESC_OA5436AA	504	738
ESC_OA5435AA	11	936
ESC_OA5434AA	155	175
ESC_OA5433AA	2073	198
ESC_OA5432AA	1716	512
ESC_OA5431AA	164	175
ESC_OA5430AA	58	198
ESC_OA5429AA	457	595
ESC_OA5428AA	847	257
ESC_OA5427AA	162	924
ESC_OA5426AA	1141	512
ESC_OA5425AA	131	653
ESC_OA5424AA	131	653
ESC_OA5423AA	38	669
ESC_OA5422AA	2449	234
ESC_OA5421AA	131	653

ESC_OA5420AA	648	359
ESC_OA5419AA	131	653
ESC_OA5418AA	410	156
ESC_OA5417AA	69	387
ESC_OA5416AA	297	234
ESC_OA5415AA	32	270
ESC_OA5414AA	32	270
ESC_OA5413AA	32	270
ESC_OA5412AA	32	270
ESC_OA5411AA	32	270
ESC_OA5410AA	32	270
ESC_OA5409AA	32	270
ESC_OA5408AA	32	270
ESC_OA5407AA	32	270
ESC_OA5406AA	32	270
ESC_OA5405AA	32	270
ESC_OA5404AA	32	270
ESC_OA5403AA	32	270
ESC_OA5402AA	32	270
ESC_OA5401AA	32	270
ESC_OA5400AA	32	270
ESC_OA5399AA	32	270
ESC_OA5398AA	32	270
ESC_OA5397AA	32	270
ESC_OA5396AA	32	270
ESC_OA5395AA	32	270
ESC_OA5394AA	32	270
ESC_OA5393AA	32	270
ESC_OA5392AA	32	270
ESC_OA5391AA	32	270
ESC_OA5390AA	32	270
ESC_OA5389AA	32	270
ESC_OA5388AA	32	270
ESC_OA5387AA	32	270
ESC_OA5386AA	32	270
ESC_OA5385AA	32	270
ESC_OA5384AA	32	270
ESC_OA5383AA	32	270
ESC_OA5382AA	32	270
ESC_OA5381AA	32	270
ESC_OA5380AA	32	270
ESC_OA5379AA	32	270
ESC_OA5378AA	32	270
ESC_OA5377AA	32	270
ESC_OA5376AA	32	270
ESC_OA5375AA	32	270
ESC_OA5374AA	32	270
ESC_OA5373AA	32	270
ESC_OA5372AA	32	270
ESC_OA5371AA	32	270

ESC_OA5370AA	32	270
ESC_OA5369AA	32	270
ESC_OA5368AA	32	270
ESC_OA5367AA	32	270
ESC_OA5366AA	32	270
ESC_OA5365AA	32	270
ESC_OA5364AA	32	270
ESC_OA5363AA	32	270
ESC_OA5362AA	32	270
ESC_OA5361AA	32	270
ESC_OA5360AA	32	270
ESC_OA5359AA	32	270
ESC_OA5358AA	32	270
ESC_OA5357AA	32	270
ESC_OA5356AA	32	270
ESC_OA5355AA	32	270
ESC_OA5354AA	722	422
ESC_OA5353AA	32	270
ESC_OA5352AA	32	270
ESC_OA5351AA	32	270
ESC_OA5350AA	32	270
ESC_OA5349AA	32	270
ESC_OA5348AA	32	270
ESC_OA5347AA	32	270
ESC_OA5346AA	32	270
ESC_OA5345AA	32	270
ESC_OA5344AA	32	270
ESC_OA5342AA	32	270
ESC_OA5341AA	32	270
ESC_OA5340AA	32	270
ESC_OA5339AA	32	270
ESC_OA5338AA	32	270
ESC_OA5337AA	32	270
ESC_OA5336AA	32	270
ESC_OA5335AA	32	270
ESC_OA5334AA	32	270
ESC_OA5333AA	32	270
ESC_OA5332AA	32	270
ESC_OA5331AA	32	270
ESC_OA5330AA	32	270
ESC_OA5329AA	32	270
ESC_OA5328AA	32	270
ESC_OA5327AA	32	270
ESC_OA5326AA	32	270
ESC_OA5325AA	32	270
ESC_OA5324AA	32	270
ESC_OA5323AA	32	270
ESC_OA5322AA	32	270
ESC_OA5321AA	32	270
ESC_OA5320AA	32	270

ESC_OA5319AA	32	270
ESC_OA5318AA	32	270
ESC_OA5317AA	4942	36
ESC_OA5316AA	32	270
ESC_OA5315AA	32	270
ESC_OA5314AA	32	270
ESC_OA5313AA	131	653
ESC_OA5312AA	410	156
ESC_OA5311AA	131	653
ESC_OA5310AA	131	653
ESC_OA5309AA	131	653
ESC_OA5308AA	131	653
ESC_OA5307AA	131	653
ESC_OA5306AA	131	653
ESC_OA5305AA	1193	761
ESC_OA5304AA	6467	452
ESC_OA5303AA	1193	761
ESC_OA5302AA	131	653
ESC_OA5301AA	95	738
ESC_OA5300AA	131	653
ESC_OA5299AA	131	653
ESC_OA5298AA	131	653
ESC_OA5297AA	131	653
ESC_OA5296AA	131	653
ESC_OA5295AA	1193	761
ESC_OA5294AA	80	731
ESC_OA5293AA	95	738
ESC_OA5292AA	127	738
ESC_OA5291AA	80	731
ESC_OA5290AA	162	924
ESC_OA5289AA	2973	234
ESC_OA5288AA	10313	899
ESC_OA5287AA	73	737
ESC_OA5286AA	12	626
ESC_OA5285AA	10317	362
ESC_OA5284AA	297	234
ESC_OA5283AA	1735	737
ESC_OA5282AA	410	156
ESC_OA5281AA	127	738
ESC_OA5280AA	1385	653
ESC_OA5279AA	2165	257
ESC_OA5278AA	961	623
ESC_OA5277AA	73	737
ESC_OA5276AA	2351	234
ESC_OA5275AA	73	737
ESC_OA5274AA	32	270
ESC_OA5273AA	372	424
ESC_OA5272AA	9024	726
ESC_OA5271AA	448	257
ESC_OA5270AA	361	512

ESC_OA5269AA	6210	766
ESC_OA5268AA	542	824
ESC_OA5267AA	12	626
ESC_OA5266AA	372	424
ESC_OA5265AA	3695	924
ESC_OA5264AA	542	824
ESC_OA5263AA	398	445
ESC_OA5262AA	127	738
ESC_OA5261AA	744	512
ESC_OA5260AA	372	424
ESC_OA5259AA	155	175
ESC_OA5258AA	4981	473
ESC_OA5257AA	10316	851
ESC_OA5256AA	80	731
ESC_OA5255AA	998	644
ESC_OA5254AA	12	626
ESC_OA5253AA	3594	924
ESC_OA5252AA	136	392
ESC_OA5251AA	10315	644
ESC_OA5250AA	3695	924
ESC_OA5249AA	1068	254
ESC_OA5248AA	361	512
ESC_OA5247AA	101	696
ESC_OA5246AA	871	447
ESC_OA5245AA	2973	234
ESC_OA5244AA	1844	198
ESC_OA5243AA	56	198
ESC_OA5242AA	117	592
ESC_OA5241AA	155	175
ESC_OA5240AA	58	198
ESC_OA5239AA	5308	261
ESC_OA5238AA	10314	257
ESC_OA5237AA	131	653
ESC_OA5236AA	131	653
ESC_OA5235AA	697	512
ESC_OA5234AA	617	511
ESC_OA5233AA	131	653
ESC_OA5232AA	131	653
ESC_OA5231AA	131	653
ESC_OA5230AA	131	653
ESC_OA5229AA	131	653
ESC_OA5227AA	589	650
ESC_OA5226AA	2015	684
ESC_OA5225AA	1441	740
ESC_OA5224AA	35	745
ESC_OA5223AA	80	731
ESC_OA5222AA	567	740
ESC_OA5221AA	80	731
ESC_OA5220AA	144	740
ESC_OA5219AA	803	739

ESC_OA5218AA	428	654
ESC_OA5217AA	3354	378
ESC_OA5216AA	582	420
ESC_OA5215AA	428	654
ESC_OA5214AA	1092	739
ESC_OA5213AA	636	653
ESC_OA5212AA	121	537
ESC_OA5211AA	28	537
ESC_OA5210AA	15	766
ESC_OA5209AA	1907	766
ESC_OA5208AA	15	766
ESC_OA5207AA	589	650
ESC_OA5206AA	131	653
ESC_OA5205AA	131	653
ESC_OA5204AA	23	156
ESC_OA5203AA	131	653
ESC_OA5202AA	617	511
ESC_OA5201AA	1193	761
ESC_OA5200AA	131	653
ESC_OA5199AA	131	653
ESC_OA5198AA	131	653
ESC_OA5197AA	345	175
ESC_OA5196AA	399	198
ESC_OA5195AA	297	234
ESC_OA5194AA	3721	93
ESC_OA5193AA	70	670
ESC_OA5192AA	770	587
ESC_OA5191AA	58	198
ESC_OA5190AA	9936	257
ESC_OA5189AA	6949	765
ESC_OA5188AA	1844	198
ESC_OA5187AA	1406	174
ESC_OA5186AA	8578	611
ESC_OA5185AA	101	696
ESC_OA5184AA	399	198
ESC_OA5183AA	9584	257
ESC_OA5182AA	1304	257
ESC_OA5181AA	11	936
ESC_OA5180AA	11	936
ESC_OA5179AA	11	936
ESC_OA5178AA	11	936
ESC_OA5177AA	11	936
ESC_OA5176AA	38	669
ESC_OA5175AA	131	653
ESC_OA5174AA	131	653
ESC_OA5173AA	131	653
ESC_OA5172AA	131	653
ESC_OA5171AA	131	653
ESC_OA5170AA	131	653
ESC_OA5169AA	23	156

ESC_OA5168AA	131	653
ESC_OA5167AA	69	387
ESC_OA5166AA	410	156
ESC_OA5165AA	69	387
ESC_OA5164AA	1177	669
ESC_OA5163AA	405	923
ESC_OA5162AA	10	512
ESC_OA5161AA	10318	758
ESC_OA5160AA	88	160
ESC_OA5159AA	33	198
ESC_OA5158AA	11	936
ESC_OA5157AA	11	936
ESC_OA5156AA	33	198
ESC_OA5155AA	11	936
ESC_OA5154AA	33	198
ESC_OA5153AA	11	936
ESC_OA5152AA	33	198
ESC_OA5151AA	131	653
ESC_OA5150AA	131	653
ESC_OA5149AA	167	512
ESC_OA5148AA	131	653
ESC_OA5147AA	393	343
ESC_OA5146AA	2172	447
ESC_OA5145AA	354	15
ESC_OA5144AA	131	653
ESC_OA5143AA	405	923
ESC_OA5142AA	405	923
ESC_OA5141AA	16	156
ESC_OA5140AA	11	936
ESC_OA5139AA	16	156
ESC_OA5138AA	21	156
ESC_OA5137AA	11	936
ESC_OA5136AA	8354	268
ESC_OA5135AA	648	359
ESC_OA5134AA	354	15
ESC_OA5133AA	131	653
ESC_OA5132AA	131	653
ESC_OA5131AA	38	669
ESC_OA5130AA	131	653
ESC_OA5129AA	405	923
ESC_OA5128AA	131	653
ESC_OA5127AA	354	15
ESC_OA5126AA	38	669
ESC_OA5125AA	131	653
ESC_OA5124AA	746	789
ESC_OA5123AA	73	737
ESC_OA5122AA	410	156
ESC_OA5121AA	44	512
ESC_OA5119AA	410	156
ESC_OA5118AA	410	156

ESC_OA5117AA	410	156
ESC_OA5115AA	410	156
ESC_OA5114AA	69	387
ESC_OA5113AA	131	653
ESC_OA5112AA	393	343
ESC_OA5111AA	131	653
ESC_OA5110AA	648	359
ESC_OA5109AA	12	626
ESC_OA5108AA	405	923
ESC_OA5107AA	38	669
ESC_OA5106AA	131	653
ESC_OA5105AA	6	445
ESC_OA5104AA	10319	935
ESC_OA5103AA	1193	761
ESC_OA5102AA	131	653
ESC_OA5101AA	167	512
ESC_OA5100AA	101	696
ESC_OA5099AA	131	653
ESC_OA5098AA	131	653
ESC_OA5097AA	131	653
ESC_OA5096AA	131	653
ESC_OA5095AA	10320	941
ESC_OA5094AA	131	653
ESC_OA5093AA	38	669
ESC_OA5092AA	131	653
ESC_OA5091AA	405	923
ESC_OA5090AA	38	669
ESC_OA5089AA	131	653
ESC_OA5088AA	131	653
ESC_OA5087AA	131	653
ESC_OA5086AA	162	924
ESC_OA5085AA	131	653
ESC_OA5084AA	131	653
ESC_OA5083AA	1193	761
ESC_OA5082AA	963	669
ESC_OA5081AA	131	653
ESC_OA5080AA	131	653
ESC_OA5079AA	131	653
ESC_OA5078AA	410	156
ESC_OA5077AA	405	923
ESC_OA5076AA	1193	761
ESC_OA5075AA	539	257
ESC_OA5074AA	59	217
ESC_OA5072AA	131	653
ESC_OA5071AA	1193	761
ESC_OA5070AA	131	653
ESC_OA5069AA	131	653
ESC_OA5068AA	131	653
ESC_OA5067AA	648	359
ESC_OA5066AA	131	653

ESC_OA5065AA	131	653
ESC_OA5064AA	69	387
ESC_OA5063AA	131	653
ESC_OA5062AA	131	653
ESC_OA5061AA	131	653
ESC_OA5060AA	12	626
ESC_OA5059AA	131	653
ESC_OA5058AA	38	669
ESC_OA5057AA	38	669
ESC_OA5056AA	38	669
ESC_OA5055AA	8189	653
ESC_OA5054AA	131	653
ESC_OA5053AA	648	359
ESC_OA5052AA	131	653
ESC_OA5051AA	131	653
ESC_OA5050AA	117	592
ESC_OA5049AA	38	669
ESC_OA5048AA	131	653
ESC_OA5047AA	131	653
ESC_OA5046AA	73	737
ESC_OA5045AA	131	653
ESC_OA5044AA	405	923
ESC_OA5043AA	131	653
ESC_OA5042AA	131	653
ESC_OA5041AA	38	669
ESC_OA5040AA	131	653
ESC_OA5039AA	162	924
ESC_OA5038AA	131	653
ESC_OA5037AA	12	626
ESC_OA5036AA	10	512
ESC_OA5035AA	1193	761
ESC_OA5034AA	131	653
ESC_OA5033AA	131	653
ESC_OA5032AA	131	653
ESC_OA5031AA	131	653
ESC_OA5030AA	131	653
ESC_OA5029AA	38	669
ESC_OA5028AA	131	653
ESC_OA5027AA	69	387
ESC_OA5026AA	1193	761
ESC_OA5025AA	127	738
ESC_OA5024AA	1193	761
ESC_OA5023AA	131	653
ESC_OA5022AA	131	653
ESC_OA5021AA	131	653
ESC_OA5020AA	38	669
ESC_OA5019AA	131	653
ESC_OA5018AA	131	653
ESC_OA5017AA	131	653
ESC_OA5016AA	38	669

ESC_OA5015AA	12	626
ESC_OA5014AA	131	653
ESC_OA5013AA	131	653
ESC_OA5012AA	131	653
ESC_OA5011AA	354	15
ESC_OA5010AA	38	669
ESC_OA5009AA	73	737
ESC_OA5008AA	80	731
ESC_OA5007AA	131	653
ESC_OA5006AA	131	653
ESC_OA5005AA	38	669
ESC_OA5004AA	69	387
ESC_OA5003AA	10324	738
ESC_OA5002AA	131	653
ESC_OA5001AA	1193	761
ESC_OA5000AA	131	653
ESC_OA4999AA	131	653
ESC_OA4998AA	131	653
ESC_OA4997AA	131	653
ESC_OA4996AA	450	71
ESC_OA4995AA	131	653
ESC_OA4994AA	131	653
ESC_OA4992AA	12	626
ESC_OA4991AA	131	653
ESC_OA4990AA	393	343
ESC_OA4989AA	648	359
ESC_OA4988AA	131	653
ESC_OA4987AA	131	653
ESC_OA4986AA	10323	653
ESC_OA4985AA	38	669
ESC_OA4984AA	1193	761
ESC_OA4983AA	12	626
ESC_OA4982AA	69	387
ESC_OA4981AA	131	653
ESC_OA4980AA	131	653
ESC_OA4979AA	131	653
ESC_OA4978AA	131	653
ESC_OA4977AA	131	653
ESC_OA4976AA	131	653
ESC_OA4975AA	38	669
ESC_OA4973AA	131	653
ESC_OA4972AA	131	653
ESC_OA4971AA	131	653
ESC_OA4970AA	131	653
ESC_OA4969AA	91	629
ESC_OA4968AA	131	653
ESC_OA4967AA	69	387
ESC_OA4966AA	131	653
ESC_OA4965AA	131	653
ESC_OA4964AA	95	738

ESC_OA4963AA	131	653
ESC_OA4962AA	131	653
ESC_OA4961AA	131	653
ESC_OA4960AA	131	653
ESC_OA4959AA	10	512
ESC_OA4958AA	10322	511
ESC_OA4957AA	131	653
ESC_OA4956AA	131	653
ESC_OA4955AA	131	653
ESC_OA4954AA	131	653
ESC_OA4953AA	10321	653
ESC_OA4952AA	131	653
ESC_OA4951AA	1193	761
ESC_OA4950AA	1193	761
ESC_OA4949AA	131	653
ESC_OA4948AA	131	653
ESC_OA4947AA	131	653
ESC_OA4946AA	155	175
ESC_OA4945AA	38	669
ESC_OA4944AA	131	653
ESC_OA4943AA	131	653
ESC_OA4942AA	405	923
ESC_OA4941AA	405	923
ESC_OA4940AA	131	653
ESC_OA4939AA	131	653
ESC_OA4938AA	131	653
ESC_OA4937AA	773	71
ESC_OA4936AA	69	387
ESC_OA4935AA	73	737
ESC_OA4934AA	156	175
ESC_OA4933AA	69	387
ESC_OA4932AA	131	653
ESC_OA4931AA	131	653
ESC_OA4930AA	131	653
ESC_OA4929AA	5853	868
ESC_OA4928AA	131	653
ESC_OA4927AA	38	669
ESC_OA4926AA	12	626
ESC_OA4925AA	131	653
ESC_OA4924AA	131	653
ESC_OA4923AA	393	343
ESC_OA4922AA	648	359
ESC_OA4921AA	131	653
ESC_OA4920AA	443	257
ESC_OA4919AA	131	653
ESC_OA4918AA	131	653
ESC_OA4917AA	1193	761
ESC_OA4916AA	38	669
ESC_OA4915AA	131	653
ESC_OA4914AA	131	653

ESC_OA4913AA	131	653
ESC_OA4912AA	131	653
ESC_OA4911AA	95	738
ESC_OA4910AA	95	738
ESC_OA4909AA	131	653
ESC_OA4908AA	131	653
ESC_OA4907AA	354	15
ESC_OA4906AA	131	653
ESC_OA4905AA	12	626
ESC_OA4904AA	167	512
ESC_OA4903AA	131	653
ESC_OA4902AA	12	626
ESC_OA4901AA	131	653
ESC_OA4900AA	162	924
ESC_OA4899AA	131	653
ESC_OA4898AA	963	669
ESC_OA4897AA	131	653
ESC_OA4896AA	131	653
ESC_OA4895AA	69	387
ESC_OA4894AA	131	653
ESC_OA4893AA	405	923
ESC_OA4892AA	131	653
ESC_OA4891AA	131	653
ESC_OA4890AA	131	653
ESC_OA4889AA	1266	210
ESC_OA4888AA	648	359
ESC_OA4887AA	12	626
ESC_OA4886AA	73	737
ESC_OA4885AA	131	653
ESC_OA4884AA	1193	761
ESC_OA4883AA	38	669
ESC_OA4882AA	1193	761
ESC_OA4881AA	949	175
ESC_OA4880AA	131	653
ESC_OA4879AA	131	653
ESC_OA4878AA	131	653
ESC_OA4877AA	131	653
ESC_OA4876AA	95	738
ESC_OA4875AA	38	669
ESC_OA4874AA	131	653
ESC_OA4873AA	131	653
ESC_OA4872AA	131	653
ESC_OA4871AA	131	653
ESC_OA4870AA	131	653
ESC_OA4869AA	131	653
ESC_OA4868AA	131	653
ESC_OA4867AA	131	653
ESC_OA4866AA	131	653
ESC_OA4865AA	131	653
ESC_OA4864AA	131	653

ESC_OA4863AA	131	653
ESC_OA4862AA	131	653
ESC_OA4861AA	131	653
ESC_OA4860AA	131	653
ESC_OA4859AA	131	653
ESC_OA4858AA	131	653
ESC_OA4857AA	38	669
ESC_OA4856AA	69	387
ESC_OA4855AA	131	653
ESC_OA4854AA	4553	408
ESC_OA4853AA	131	653
ESC_OA4852AA	4553	408
ESC_OA4851AA	1193	761
ESC_OA4850AA	315	669
ESC_OA4849AA	131	653
ESC_OA4848AA	1177	669
ESC_OA4847AA	131	653
ESC_OA4846AA	1193	761
ESC_OA4845AA	131	653
ESC_OA4844AA	131	653
ESC_OA4843AA	131	653
ESC_OA4842AA	1193	761
ESC_OA4841AA	131	653
ESC_OA4840AA	69	387
ESC_OA4839AA	131	653
ESC_OA4838AA	405	923
ESC_OA4837AA	457	595
ESC_OA4836AA	131	653
ESC_OA4835AA	131	653
ESC_OA4834AA	131	653
ESC_OA4833AA	131	653
ESC_OA4832AA	131	653
ESC_OA4831AA	131	653
ESC_OA4830AA	131	653
ESC_OA4829AA	131	653
ESC_OA4828AA	43	512
ESC_OA4827AA	410	156
ESC_OA4826AA	131	653
ESC_OA4825AA	227	504
ESC_OA4824AA	131	653
ESC_OA4823AA	131	653
ESC_OA4822AA	131	653
ESC_OA4821AA	131	653
ESC_OA4820AA	131	653
ESC_OA4819AA	351	268
ESC_OA4818AA	501	900
ESC_OA4817AA	10	512
ESC_OA4816AA	11	936
ESC_OA4815AA	11	936
ESC_OA4814AA	11	936

ESC_OA4813AA	10	512
ESC_OA4812AA	1304	257
ESC_OA4811AA	58	198
ESC_OA4810AA	58	198
ESC_OA4808AA	401	142
ESC_OA4807AA	10	512
ESC_OA4806AA	1307	257
ESC_OA4805AA	2040	198
ESC_OA4804AA	7100	924
ESC_OA4803AA	117	592
ESC_OA4802AA	401	142
ESC_OA4801AA	101	696
ESC_OA4799AA	10326	175
ESC_OA4798AA	2279	657
ESC_OA4797AA	1136	108
ESC_OA4796AA	218	519
ESC_OA4795AA	1141	512
ESC_OA4794AA	2179	924
ESC_OA4793AA	73	737
ESC_OA4792AA	372	424
ESC_OA4791AA	10325	426
ESC_OA4790AA	372	424
ESC_OA4789AA	95	738
ESC_OA4788AA	73	737
ESC_OA4787AA	1618	736
ESC_OA4783AA	10327	198
ESC_OA4782AA	33	198
ESC_OA4781AA	73	737
ESC_OA4780AA	73	737
ESC_OA4779AA	10	512
ESC_OA4778AA	117	592
ESC_OA4777AA	38	669
ESC_OA4776AA	5579	175
ESC_OA4775AA	86	935
ESC_OA4774AA	8421	261
ESC_OA4773AA	10	512
ESC_OA4772AA	1726	257
ESC_OA4771AA	245	261
ESC_OA4770AA	245	261
ESC_OA4769AA	152	257
ESC_OA4767AA	245	261
ESC_OA4766AA	245	261
ESC_OA4765AA	152	257
ESC_OA4764AA	152	257
ESC_OA4763AA	245	261
ESC_OA4762AA	152	257
ESC_OA4760AA	11	936
ESC_OA4759AA	583	768
ESC_OA4758AA	11	936
ESC_OA4757AA	11	936

ESC_OA4756AA	21	156
ESC_OA4755AA	16	156
ESC_OA4754AA	410	156
ESC_OA4753AA	245	261
ESC_OA4752AA	10312	261
ESC_OA4751AA	148	191
ESC_OA4750AA	11	936
ESC_OA4749AA	32	270
ESC_OA4748AA	11	936
ESC_OA4747AA	11	936
ESC_OA4746AA	442	257
ESC_OA4745AA	11	936
ESC_OA4744AA	11	936
ESC_OA4743AA	11	936
ESC_OA4742AA	11	936
ESC_OA4741AA	301	307
ESC_OA4740AA	301	307
ESC_OA4739AA	11	936
ESC_OA4738AA	11	936
ESC_OA4737AA	11	936
ESC_OA4736AA	11	936
ESC_OA4735AA	11	936
ESC_OA4734AA	11	936
ESC_OA4733AA	11	936
ESC_OA4732AA	11	936
ESC_OA4731AA	6052	83
ESC_OA4730AA	1804	935
ESC_OA4729AA	11	936
ESC_OA4728AA	11	936
ESC_OA4727AA	11	936
ESC_OA4726AA	11	936
ESC_OA4725AA	21	156
ESC_OA4724AA	11	936
ESC_OA4723AA	11	936
ESC_OA4722AA	152	257
ESC_OA4721AA	1193	761
ESC_OA4720AA	245	261
ESC_OA4719AA	245	261
ESC_OA4718AA	152	257
ESC_OA4717AA	245	261
ESC_OA4716AA	152	257
ESC_OA4715AA	148	191
ESC_OA4714AA	11	936
ESC_OA4713AA	301	307
ESC_OA4712AA	245	261
ESC_OA4711AA	152	257
ESC_OA4710AA	152	257
ESC_OA4709AA	10310	587
ESC_OA4708AA	10311	737
ESC_OA4707AA	152	257

ESC_OA4706AA	152	257
ESC_OA4705AA	152	257
ESC_OA4704AA	245	261
ESC_OA4703AA	245	261
ESC_OA4702AA	148	191
ESC_OA4701AA	148	191
ESC_OA4700AA	245	261
ESC_OA4699AA	29	156
ESC_OA4698AA	21	156
ESC_OA4697AA	2914	670
ESC_OA4696AA	2914	670
ESC_OA4695AA	648	359
ESC_OA4694AA	773	71
ESC_OA4693AA	517	924
ESC_OA4692AA	799	852
ESC_OA4691AA	38	669
ESC_OA4690AA	6	445
ESC_OA4689AA	6	445
ESC_OA4688AA	6	445
ESC_OA4687AA	245	261
ESC_OA4685AA	54	256
ESC_OA4684AA	550	756
ESC_OA4683AA	127	738
ESC_OA4682AA	95	738
ESC_OA4681AA	131	653
ESC_OA4680AA	95	738
ESC_OA4679AA	58	198
ESC_OA4678AA	131	653
ESC_OA4677AA	73	737
ESC_OA4676AA	95	738
ESC_OA4675AA	362	234
ESC_OA4674AA	127	738
ESC_OA4673AA	131	653
ESC_OA4672AA	12	626
ESC_OA4671AA	95	738
ESC_OA4670AA	1882	387
ESC_OA4669AA	10309	626
ESC_OA4668AA	127	738
ESC_OA4667AA	131	653
ESC_OA4666AA	357	654
ESC_OA4665AA	1193	761
ESC_OA4664AA	69	387
ESC_OA4663AA	12	626
ESC_OA4662AA	12	626
ESC_OA4661AA	131	653
ESC_OA4660AA	95	738
ESC_OA4659AA	10	512
ESC_OA4658AA	73	737
ESC_OA4657AA	127	738
ESC_OA4656AA	10308	653

ESC_OA4655AA	73	737
ESC_OA4654AA	1858	757
ESC_OA4653AA	117	592
ESC_OA4652AA	1444	768
ESC_OA4651AA	95	738
ESC_OA4650AA	95	738
ESC_OA4649AA	127	738
ESC_OA4648AA	91	629
ESC_OA4647AA	69	387
ESC_OA4646AA	69	387
ESC_OA4644AA	73	737
ESC_OA4643AA	131	653
ESC_OA4642AA	131	653
ESC_OA4641AA	9241	738
ESC_OA4640AA	968	737
ESC_OA4639AA	10	512
ESC_OA4638AA	127	738
ESC_OA4637AA	127	738
ESC_OA4636AA	421	744
ESC_OA4635AA	69	387
ESC_OA4634AA	349	670
ESC_OA4633AA	73	737
ESC_OA4632AA	23	156
ESC_OA4631AA	38	669
ESC_OA4630AA	10307	737
ESC_OA4629AA	361	512
ESC_OA4628AA	8371	898
ESC_OA4627AA	131	653
ESC_OA4626AA	141	644
ESC_OA4625AA	131	653
ESC_OA4624AA	10305	387
ESC_OA4623AA	12	626
ESC_OA4622AA	998	644
ESC_OA4621AA	62	574
ESC_OA4620AA	95	738
ESC_OA4619AA	968	737
ESC_OA4618AA	978	740
ESC_OA4617AA	73	737
ESC_OA4616AA	404	761
ESC_OA4615AA	131	653
ESC_OA4614AA	127	738
ESC_OA4613AA	998	644
ESC_OA4612AA	2021	738
ESC_OA4611AA	10304	654
ESC_OA4610AA	141	644
ESC_OA4609AA	69	387
ESC_OA4608AA	69	387
ESC_OA4607AA	95	738
ESC_OA4606AA	10	512
ESC_OA4605AA	10	512

ESC_OA4604AA	56	198
ESC_OA4603AA	10303	740
ESC_OA4602AA	8026	165
ESC_OA4601AA	162	924
ESC_OA4600AA	95	738
ESC_OA4599AA	127	738
ESC_OA4598AA	999	654
ESC_OA4597AA	73	737
ESC_OA4596AA	10	512
ESC_OA4595AA	10306	254
ESC_OA4594AA	95	738
ESC_OA4593AA	2531	198
ESC_OA4592AA	141	644
ESC_OA4591AA	420	741
ESC_OA4590AA	537	756
ESC_OA4589AA	73	737
ESC_OA4588AA	131	653
ESC_OA4587AA	641	935
ESC_OA4586AA	73	737
ESC_OA4585AA	10302	629
ESC_OA4584AA	625	756
ESC_OA4583AA	141	644
ESC_OA4582AA	127	738
ESC_OA4581AA	38	669
ESC_OA4580AA	131	653
ESC_OA4579AA	73	737
ESC_OA4578AA	69	387
ESC_OA4577AA	141	644
ESC_OA4576AA	394	400
ESC_OA4575AA	62	574
ESC_OA4574AA	973	968
ESC_OA4573AA	7285	175
ESC_OA4572AA	404	761
ESC_OA4571AA	131	653
ESC_OA4570AA	10	512
ESC_OA4569AA	12	626
ESC_OA4568AA	4623	257
ESC_OA4567AA	95	738
ESC_OA4566AA	543	842
ESC_OA4565AA	3352	626
ESC_OA4564AA	127	738
ESC_OA4563AA	14	761
ESC_OA4562AA	69	387
ESC_OA4561AA	538	629
ESC_OA4560AA	59	217
ESC_OA4559AA	59	217
ESC_OA4558AA	131	653
ESC_OA4557AA	95	738
ESC_OA4556AA	5363	512
ESC_OA4555AA	38	669

ESC_OA4554AA	404	761
ESC_OA4553AA	95	738
ESC_OA4552AA	141	644
ESC_OA4551AA	127	738
ESC_OA4550AA	141	644
ESC_OA4549AA	372	424
ESC_OA4548AA	73	737
ESC_OA4547AA	1193	761
ESC_OA4546AA	95	738
ESC_OA4545AA	2554	653
ESC_OA4544AA	127	738
ESC_OA4543AA	1643	780
ESC_OA4542AA	10	512
ESC_OA4541AA	69	387
ESC_OA4540AA	1914	956
ESC_OA4539AA	224	198
ESC_OA4538AA	69	387
ESC_OA4537AA	73	737
ESC_OA4536AA	91	629
ESC_OA4535AA	59	217
ESC_OA4534AA	95	738
ESC_OA4533AA	73	737
ESC_OA4532AA	127	738
ESC_OA4531AA	131	653
ESC_OA4530AA	126	740
ESC_OA4529AA	58	198
ESC_OA4528AA	127	738
ESC_OA4527AA	131	653
ESC_OA4526AA	58	198
ESC_OA4525AA	80	731
ESC_OA4524AA	141	644
ESC_OA4523AA	421	744
ESC_OA4522AA	95	738
ESC_OA4521AA	73	737
ESC_OA4520AA	127	738
ESC_OA4519AA	538	629
ESC_OA4518AA	127	738
ESC_OA4517AA	394	400
ESC_OA4516AA	95	738
ESC_OA4515AA	10301	726
ESC_OA4514AA	999	654
ESC_OA4513AA	405	923
ESC_OA4512AA	1415	504
ESC_OA4511AA	452	743
ESC_OA4510AA	357	654
ESC_OA4509AA	69	387
ESC_OA4508AA	58	198
ESC_OA4507AA	131	653
ESC_OA4506AA	10	512
ESC_OA4505AA	131	653

ESC_OA4504AA	127	738
ESC_OA4503AA	127	738
ESC_OA4502AA	10	512
ESC_OA4501AA	80	731
ESC_OA4500AA	73	737
ESC_OA4499AA	141	644
ESC_OA4498AA	127	738
ESC_OA4497AA	539	257
ESC_OA4496AA	131	653
ESC_OA4495AA	69	387
ESC_OA4494AA	1193	761
ESC_OA4493AA	131	653
ESC_OA4492AA	648	359
ESC_OA4491AA	69	387
ESC_OA4490AA	640	768
ESC_OA4489AA	363	738
ESC_OA4488AA	685	311
ESC_OA4487AA	144	740
ESC_OA4486AA	127	738
ESC_OA4485AA	453	935
ESC_OA4484AA	4204	511
ESC_OA4483AA	1193	761
ESC_OA4482AA	1193	761
ESC_OA4481AA	2178	924
ESC_OA4480AA	101	696
ESC_OA4478AA	38	669
ESC_OA4477AA	1193	761
ESC_OA4476AA	4429	820
ESC_OA4475AA	450	71
ESC_OA4474AA	1193	761
ESC_OA4473AA	38	669
ESC_OA4472AA	131	653
ESC_OA4471AA	3285	257
ESC_OA4470AA	624	359
ESC_OA4469AA	683	175
ESC_OA4468AA	38	669
ESC_OA4467AA	2758	512
ESC_OA4466AA	226	512
ESC_OA4465AA	38	669
ESC_OA4464AA	484	71
ESC_OA4463AA	2913	286
ESC_OA4462AA	38	669
ESC_OA4461AA	38	669
ESC_OA4460AA	2179	924
ESC_OA4459AA	4	76
ESC_OA4458AA	2178	924
ESC_OA4457AA	10	512
ESC_OA4456AA	38	669
ESC_OA4455AA	1491	76
ESC_OA4454AA	10300	463

ESC_OA4452AA	10297	949
ESC_OA4451AA	10296	160
ESC_OA4450AA	73	737
ESC_OA4449AA	10287	286
ESC_OA4448AA	10286	19
ESC_OA4447AA	10298	653
ESC_OA4446AA	10299	626
ESC_OA4445AA	10285	627
ESC_OA4444AA	10294	754
ESC_OA4443AA	10292	512
ESC_OA4442AA	10291	737
ESC_OA4441AA	10290	387
ESC_OA4440AA	10284	954
ESC_OA4439AA	10277	203
ESC_OA4438AA	10289	512
ESC_OA4437AA	10288	737
ESC_OA4436AA	10281	744
ESC_OA4435AA	10280	55
ESC_OA4434AA	10295	737
ESC_OA4433AA	10293	737
ESC_OA4432AA	1850	269
ESC_OA4431AA	10272	156
ESC_OA4430AA	10274	935
ESC_OA4429AA	10282	610
ESC_OA4428AA	10283	25
ESC_OA4427AA	10278	737
ESC_OA4425AA	141	644
ESC_OA4424AA	10279	740
ESC_OA4423AA	10275	740
ESC_OA4422AA	10273	592
ESC_OA4421AA	10276	923
ESC_OA4420AA	10271	626
ESC_OA4419AA	10270	160
ESC_OA4417AA	10268	168
ESC_OA4416AA	10267	737
ESC_OA4415AA	10266	249
ESC_OA4414AA	10264	737
ESC_OA4413AA	10265	387
ESC_OA4412AA	10262	630
ESC_OA4411AA	10260	738
ESC_OA4410AA	10258	923
ESC_OA4409AA	10261	512
ESC_OA4408AA	10257	175
ESC_OA4407AA	10256	504
ESC_OA4406AA	10253	737
ESC_OA4405AA	10255	359
ESC_OA4404AA	10251	737
ESC_OA4403AA	10252	626
ESC_OA4402AA	10250	737
ESC_OA4401AA	10254	160

ESC_OA4400AA	10248	311
ESC_OA4398AA	73	737
ESC_OA4397AA	10263	393
ESC_OA4396AA	10247	217
ESC_OA4395AA	10259	738
ESC_OA4394AA	10269	924
ESC_OA4393AA	117	592
ESC_OA4392AA	10249	424
ESC_OA4391AA	10245	738
ESC_OA4390AA	131	653
ESC_OA4389AA	10238	257
ESC_OA4388AA	10235	19
ESC_OA4387AA	10244	824
ESC_OA4386AA	10242	108
ESC_OA4385AA	10240	644
ESC_OA4384AA	10239	257
ESC_OA4383AA	372	424
ESC_OA4382AA	10243	160
ESC_OA4381AA	131	653
ESC_OA4380AA	998	644
ESC_OA4379AA	10237	424
ESC_OA4378AA	10227	141
ESC_OA4377AA	10246	738
ESC_OA4376AA	10236	626
ESC_OA4375AA	10241	738
ESC_OA4374AA	10223	990
ESC_OA4373AA	9962	684
ESC_OA4372AA	10234	670
ESC_OA4371AA	10231	632
ESC_OA4370AA	2619	738
ESC_OA4369AA	10232	311
ESC_OA4368AA	10229	653
ESC_OA4367AA	10233	874
ESC_OA4366AA	88	160
ESC_OA4365AA	10230	924
ESC_OA4364AA	10221	198
ESC_OA4363AA	131	653
ESC_OA4362AA	10225	160
ESC_OA4361AA	10222	738
ESC_OA4360AA	10219	198
ESC_OA4359AA	10218	340
ESC_OA4357AA	10216	537
ESC_OA4356AA	95	738
ESC_OA4355AA	59	217
ESC_OA4354AA	10213	737
ESC_OA4353AA	10228	696
ESC_OA4352AA	10220	653
ESC_OA4351AA	10211	654
ESC_OA4350AA	10217	951
ESC_OA4349AA	10212	737

ESC_OA4348AA	10226	217
ESC_OA4347AA	10214	380
ESC_OA4346AA	10215	380
ESC_OA4344AA	10224	738
ESC_OA4343AA	152	257
ESC_OA4342AA	152	257
ESC_OA4341AA	245	261
ESC_OA4340AA	152	257
ESC_OA4339AA	152	257
ESC_OA4338AA	245	261
ESC_OA4337AA	145	256
ESC_OA4336AA	1503	257
ESC_OA4335AA	243	197
ESC_OA4334AA	152	257
ESC_OA4333AA	245	261
ESC_OA4332AA	152	257
ESC_OA4331AA	148	191
ESC_OA4330AA	245	261
ESC_OA4329AA	152	257
ESC_OA4328AA	152	257
ESC_OA4327AA	152	257
ESC_OA4326AA	152	257
ESC_OA4325AA	152	257
ESC_OA4324AA	630	261
ESC_OA4323AA	245	261
ESC_OA4322AA	245	261
ESC_OA4321AA	152	257
ESC_OA4320AA	152	257
ESC_OA4319AA	152	257
ESC_OA4318AA	131	653
ESC_OA4317AA	38	669
ESC_OA4316AA	93	71
ESC_OA4315AA	354	15
ESC_OA4314AA	5150	661
ESC_OA4313AA	226	512
ESC_OA4311AA	21	156
ESC_OA4309AA	152	257
ESC_OA4308AA	2179	924
ESC_OA4307AA	453	935
ESC_OA4306AA	295	160
ESC_OA4305AA	2003	669
ESC_OA4304AA	6732	1004
ESC_OA4303AA	10210	156
ESC_OA4302AA	405	923
ESC_OA4301AA	4674	592
ESC_OA4300AA	38	669
ESC_OA4299AA	93	71
ESC_OA4298AA	10	512
ESC_OA4297AA	131	653
ESC_OA4296AA	131	653

ESC_OA4295AA	43	512
ESC_OA4294AA	69	387
ESC_OA4293AA	7454	286
ESC_OA4292AA	58	198
ESC_OA4291AA	58	198
ESC_OA4290AA	2732	234
ESC_OA4289AA	5150	661
ESC_OA4287AA	349	670
ESC_OA4286AA	101	696
ESC_OA4285AA	93	71
ESC_OA4284AA	206	286
ESC_OA4283AA	73	737
ESC_OA4282AA	718	257
ESC_OA4281AA	442	257
ESC_OA4280AA	162	924
ESC_OA4279AA	73	737
ESC_OA4278AA	1967	191
ESC_OA4277AA	5559	257
ESC_OA4276AA	5559	257
ESC_OA4275AA	58	198
ESC_OA4274AA	5937	257
ESC_OA4273AA	5559	257
ESC_OA4272AA	162	924
ESC_OA4271AA	5559	257
ESC_OA4270AA	442	257
ESC_OA4269AA	442	257
ESC_OA4268AA	2787	198
ESC_OA4267AA	442	257
ESC_OA4266AA	442	257
ESC_OA4265AA	131	653
ESC_OA4264AA	131	653
ESC_OA4263AA	38	669
ESC_OA4262AA	95	738
ESC_OA4261AA	2020	387
ESC_OA4260AA	131	653
ESC_OA4259AA	131	653
ESC_OA4258AA	131	653
ESC_OA4257AA	95	738
ESC_OA4256AA	2659	679
ESC_OA4255AA	2936	515
ESC_OA4254AA	752	512
ESC_OA4253AA	117	592
ESC_OA4252AA	2040	198
ESC_OA4251AA	1177	669
ESC_OA4250AA	57	50
ESC_OA4249AA	167	512
ESC_OA4248AA	414	387
ESC_OA4247AA	410	156
ESC_OA4246AA	69	387
ESC_OA4245AA	69	387

ESC_OA4244AA	189	504
ESC_OA4243AA	648	359
ESC_OA4242AA	1163	579
ESC_OA4241AA	43	512
ESC_OA4240AA	6215	962
ESC_OA4239AA	57	50
ESC_OA4238AA	216	512
ESC_OA4237AA	3052	669
ESC_OA4236AA	155	175
ESC_OA4235AA	1193	761
ESC_OA4234AA	648	359
ESC_OA4233AA	69	387
ESC_OA4232AA	131	653
ESC_OA4231AA	10	512
ESC_OA4230AA	93	71
ESC_OA4229AA	131	653
ESC_OA4228AA	131	653
ESC_OA4227AA	131	653
ESC_OA4226AA	131	653
ESC_OA4225AA	405	923
ESC_OA4224AA	131	653
ESC_OA4223AA	131	653
ESC_OA4222AA	131	653
ESC_OA4221AA	131	653
ESC_OA4220AA	131	653
ESC_OA4219AA	6514	512
ESC_OA4218AA	167	512
ESC_OA4217AA	38	669
ESC_OA4216AA	38	669
ESC_OA4215AA	484	71
ESC_OA4214AA	484	71
ESC_OA4213AA	224	198
ESC_OA4211AA	58	198
ESC_OA4210AA	1025	261
ESC_OA4209AA	245	261
ESC_OA4208AA	245	261
ESC_OA4207AA	152	257
ESC_OA4206AA	152	257
ESC_OA4205AA	152	257
ESC_OA4204AA	5517	257
ESC_OA4203AA	245	261
ESC_OA4202AA	245	261
ESC_OA4201AA	152	257
ESC_OA4200AA	152	257
ESC_OA4199AA	32	270
ESC_OA4198AA	11	936
ESC_OA4197AA	11	936
ESC_OA4196AA	11	936
ESC_OA4195AA	330	504
ESC_OA4194AA	21	156

ESC_OA4193AA	32	270
ESC_OA4192AA	32	270
ESC_OA4191AA	11	936
ESC_OA4190AA	589	650
ESC_OA4189AA	306	286
ESC_OA4188AA	33	198
ESC_OA4187AA	659	286
ESC_OA4186AA	6992	842
ESC_OA4185AA	1819	808
ESC_OA4184AA	10	512
ESC_OA4183AA	33	198
ESC_OA4182AA	405	923
ESC_OA4181AA	131	653
ESC_OA4180AA	131	653
ESC_OA4179AA	648	359
ESC_OA4178AA	648	359
ESC_OA4177AA	131	653
ESC_OA4176AA	131	653
ESC_OA4175AA	131	653
ESC_OA4174AA	131	653
ESC_OA4173AA	131	653
ESC_OA4172AA	131	653
ESC_OA4171AA	405	923
ESC_OA4170AA	101	696
ESC_OA4169AA	10	512
ESC_OA4168AA	10208	233
ESC_OA4167AA	11	936
ESC_OA4166AA	11	936
ESC_OA4165AA	1804	935
ESC_OA4164AA	11	936
ESC_OA4163AA	11	936
ESC_OA4162AA	11	936
ESC_OA4161AA	11	936
ESC_OA4160AA	11	936
ESC_OA4159AA	11	936
ESC_OA4158AA	11	936
ESC_OA4157AA	131	653
ESC_OA4156AA	131	653
ESC_OA4155AA	131	653
ESC_OA4154AA	131	653
ESC_OA4153AA	6332	153
ESC_OA4152AA	131	653
ESC_OA4151AA	131	653
ESC_OA4150AA	131	653
ESC_OA4149AA	131	653
ESC_OA4148AA	131	653
ESC_OA4147AA	12	626
ESC_OA4146AA	131	653
ESC_OA4145AA	405	923
ESC_OA4144AA	131	653

ESC_OA4143AA	167	512
ESC_OA4142AA	131	653
ESC_OA4141AA	131	653
ESC_OA4140AA	131	653
ESC_OA4139AA	131	653
ESC_OA4138AA	131	653
ESC_OA4137AA	131	653
ESC_OA4136AA	131	653
ESC_OA4135AA	131	653
ESC_OA4134AA	131	653
ESC_OA4133AA	131	653
ESC_OA4132AA	131	653
ESC_OA4131AA	405	923
ESC_OA4130AA	131	653
ESC_OA4129AA	131	653
ESC_OA4128AA	131	653
ESC_OA4127AA	131	653
ESC_OA4126AA	131	653
ESC_OA4125AA	131	653
ESC_OA4124AA	8347	653
ESC_OA4123AA	405	923
ESC_OA4122AA	648	359
ESC_OA4121AA	10209	198
ESC_OA4120AA	101	696
ESC_OA4119AA	10	512
ESC_OA4118AA	46	447
ESC_OA4117AA	117	592
ESC_OA4116AA	162	924
ESC_OA4115AA	616	156
ESC_OA4114AA	409	504
ESC_OA4113AA	131	653
ESC_OA4112AA	131	653
ESC_OA4111AA	131	653
ESC_OA4110AA	131	653
ESC_OA4109AA	73	737
ESC_OA4108AA	131	653
ESC_OA4107AA	131	653
ESC_OA4106AA	648	359
ESC_OA4105AA	131	653
ESC_OA4104AA	131	653
ESC_OA4103AA	131	653
ESC_OA4102AA	131	653
ESC_OA4101AA	95	738
ESC_OA4100AA	361	512
ESC_OA4099AA	219	877
ESC_OA4098AA	6052	83
ESC_OA4097AA	21	156
ESC_OA4096AA	11	936
ESC_OA4095AA	11	936
ESC_OA4094AA	11	936

ESC_OA4093AA	152	257
ESC_OA4092AA	245	261
ESC_OA4091AA	245	261
ESC_OA4090AA	245	261
ESC_OA4089AA	152	257
ESC_OA4088AA	148	191
ESC_OA4087AA	245	261
ESC_OA4086AA	152	257
ESC_OA4085AA	152	257
ESC_OA4084AA	245	261
ESC_OA4083AA	8305	235
ESC_OA4082AA	152	257
ESC_OA4081AA	148	191
ESC_OA4080AA	245	261
ESC_OA4079AA	245	261
ESC_OA4078AA	252	190
ESC_OA4077AA	245	261
ESC_OA4069AA	10	512
ESC_OA4068AA	11076	71
ESC_OA4067AA	11	936
ESC_OA4066AA	6071	817
ESC_OA4065AA	1434	512
ESC_OA4064AA	11	936
ESC_OA4063AA	21	156
ESC_OA4062AA	1804	935
ESC_OA4061AA	21	156
ESC_OA4060AA	73	737
ESC_OA4059AA	6	445
ESC_OA4058AA	504	738
ESC_OA4057AA	11	936
ESC_OA4056AA	1804	935
ESC_OA4055AA	11	936
ESC_OA4054AA	21	156
ESC_OA4053AA	11	936
ESC_OA4052AA	11	936
ESC_OA4051AA	11	936
ESC_OA4050AA	17	191
ESC_OA4049AA	162	924
ESC_OA4046AA	602	257
ESC_OA4045AA	1196	257
ESC_OA4044AA	1196	257
ESC_OA4042AA	1485	408
ESC_OA4041AA	131	653
ESC_OA4040AA	131	653
ESC_OA4039AA	131	653
ESC_OA4038AA	10	512
ESC_OA4037AA	131	653
ESC_OA4036AA	131	653
ESC_OA4035AA	131	653
ESC_OA4034AA	131	653

ESC_OA4033AA	131	653
ESC_OA4032AA	410	156
ESC_OA4031AA	131	653
ESC_OA4030AA	131	653
ESC_OA4029AA	131	653
ESC_OA4028AA	131	653
ESC_OA4027AA	131	653
ESC_OA4026AA	10207	653
ESC_OA4025AA	131	653
ESC_OA4024AA	131	653
ESC_OA4023AA	131	653
ESC_OA4022AA	131	653
ESC_OA4021AA	155	175
ESC_OA4020AA	38	669
ESC_OA4019AA	8619	924
ESC_OA4018AA	17	191
ESC_OA4017AA	616	156
ESC_OA4016AA	189	504
ESC_OA4015AA	10206	670
ESC_OA4014AA	10	512
ESC_OA4013AA	117	592
ESC_OA4012AA	189	504
ESC_OA4011AA	10	512
ESC_OA4010AA	1080	198
ESC_OA4009AA	278	924
ESC_OA4008AA	10205	503
ESC_OA4007AA	1280	359
ESC_OA4006AA	109	257
ESC_OA4005AA	131	653
ESC_OA4004AA	131	653
ESC_OA4003AA	131	653
ESC_OA4002AA	131	653
ESC_OA4001AA	131	653
ESC_OA4000AA	59	217
ESC_OA3999AA	131	653
ESC_OA3998AA	617	511
ESC_OA3997AA	8671	653
ESC_OA3996AA	131	653
ESC_OA3995AA	1421	447
ESC_OA3994AA	38	669
ESC_OA3993AA	457	595
ESC_OA3992AA	405	923
ESC_OA3991AA	393	343
ESC_OA3990AA	245	261
ESC_OA3989AA	245	261
ESC_OA3988AA	152	257
ESC_OA3987AA	131	653
ESC_OA3986AA	155	175
ESC_OA3985AA	278	924
ESC_OA3984AA	386	191

ESC_OA3983AA	11	936
ESC_OA3982AA	32	270
ESC_OA3981AA	602	257
ESC_OA3980AA	12	626
ESC_OA3979AA	164	175
ESC_OA3978AA	3580	257
ESC_OA3977AA	399	198
ESC_OA3976AA	2040	198
ESC_OA3975AA	162	924
ESC_OA3974AA	607	355
ESC_OA3973AA	2521	257
ESC_OA3972AA	10204	198
ESC_OA3971AA	1308	257
ESC_OA3970AA	1316	471
ESC_OA3969AA	10	512
ESC_OA3968AA	1618	736
ESC_OA3967AA	607	355
ESC_OA3966AA	428	654
ESC_OA3965AA	1123	257
ESC_OA3964AA	117	592
ESC_OA3963AA	117	592
ESC_OA3962AA	131	653
ESC_OA3961AA	2079	198
ESC_OA3960AA	155	175
ESC_OA3959AA	345	175
ESC_OA3958AA	1307	257
ESC_OA3957AA	1079	235
ESC_OA3956AA	352	744
ESC_OA3955AA	399	198
ESC_OA3954AA	428	654
ESC_OA3953AA	245	261
ESC_OA3952AA	245	261
ESC_OA3951AA	6919	257
ESC_OA3950AA	245	261
ESC_OA3949AA	152	257
ESC_OA3948AA	245	261
ESC_OA3947AA	8878	261
ESC_OA3946AA	270	204
ESC_OA3945AA	245	261
ESC_OA3944AA	148	191
ESC_OA3943AA	152	257
ESC_OA3942AA	152	257
ESC_OA3941AA	245	261
ESC_OA3940AA	152	257
ESC_OA3939AA	245	261
ESC_OA3938AA	245	261
ESC_OA3937AA	152	257
ESC_OA3936AA	152	257
ESC_OA3935AA	152	257
ESC_OA3934AA	628	261

ESC_OA3933AA	152	257
ESC_OA3932AA	245	261
ESC_OA3931AA	245	261
ESC_OA3930AA	145	256
ESC_OA3929AA	148	191
ESC_OA3928AA	245	261
ESC_OA3927AA	245	261
ESC_OA3926AA	630	261
ESC_OA3925AA	245	261
ESC_OA3924AA	152	257
ESC_OA3923AA	152	257
ESC_OA3922AA	152	257
ESC_OA3921AA	152	257
ESC_OA3920AA	152	257
ESC_OA3919AA	245	261
ESC_OA3918AA	245	261
ESC_OA3917AA	245	261
ESC_OA3916AA	245	261
ESC_OA3915AA	152	257
ESC_OA3914AA	630	261
ESC_OA3913AA	245	261
ESC_OA3912AA	145	256
ESC_OA3911AA	152	257
ESC_OA3910AA	245	261
ESC_OA3909AA	152	257
ESC_OA3908AA	152	257
ESC_OA3907AA	11	936
ESC_OA3906AA	11	936
ESC_OA3905AA	11	936
ESC_OA3904AA	21	156
ESC_OA3903AA	11	936
ESC_OA3902AA	659	286
ESC_OA3901AA	11	936
ESC_OA3900AA	11	936
ESC_OA3899AA	642	924
ESC_OA3898AA	11	936
ESC_OA3897AA	57	50
ESC_OA3896AA	11	936
ESC_OA3895AA	11	936
ESC_OA3894AA	33	198
ESC_OA3893AA	10	512
ESC_OA3892AA	11	936
ESC_OA3891AA	11	936
ESC_OA3890AA	677	257
ESC_OA3889AA	6260	156
ESC_OA3888AA	446	257
ESC_OA3887AA	21	156
ESC_OA3886AA	32	270
ESC_OA3885AA	21	156
ESC_OA3884AA	11	936

ESC_OA3883AA	442	257
ESC_OA3882AA	11	936
ESC_OA3881AA	6314	56
ESC_OA3880AA	659	286
ESC_OA3879AA	6	445
ESC_OA3878AA	11	936
ESC_OA3877AA	28	537
ESC_OA3876AA	11	936
ESC_OA3875AA	642	924
ESC_OA3874AA	442	257
ESC_OA3873AA	11	936
ESC_OA3872AA	11	936
ESC_OA3871AA	21	156
ESC_OA3870AA	11	936
ESC_OA3869AA	381	156
ESC_OA3868AA	11	936
ESC_OA3867AA	11	936
ESC_OA3866AA	11	936
ESC_OA3865AA	21	156
ESC_OA3864AA	4	76
ESC_OA3863AA	11	936
ESC_OA3862AA	21	156
ESC_OA3861AA	21	156
ESC_OA3860AA	11	936
ESC_OA3859AA	11	936
ESC_OA3858AA	6	445
ESC_OA3857AA	11	936
ESC_OA3856AA	245	261
ESC_OA3855AA	38	669
ESC_OA3854AA	152	257
ESC_OA3853AA	152	257
ESC_OA3852AA	152	257
ESC_OA3851AA	245	261
ESC_OA3850AA	17	191
ESC_OA3849AA	173	254
ESC_OA3848AA	173	254
ESC_OA3847AA	647	734
ESC_OA3846AA	58	198
ESC_OA3845AA	173	254
ESC_OA3844AA	173	254
ESC_OA3843AA	58	198
ESC_OA3842AA	69	387
ESC_OA3841AA	173	254
ESC_OA3840AA	1656	175
ESC_OA3839AA	392	234
ESC_OA3838AA	154	257
ESC_OA3837AA	1656	175
ESC_OA3836AA	173	254
ESC_OA3835AA	58	198
ESC_OA3834AA	10	512

ESC_OA3833AA	10	512
ESC_OA3832AA	10	512
ESC_OA3831AA	10	512
ESC_OA3830AA	10	512
ESC_OA3828AA	10	512
ESC_OA3827AA	10	512
ESC_OA3826AA	10	512
ESC_OA3825AA	10	512
ESC_OA3824AA	10	512
ESC_OA3823AA	10	512
ESC_OA3822AA	10	512
ESC_OA3821AA	10	512
ESC_OA3820AA	10	512
ESC_OA3819AA	10	512
ESC_OA3818AA	10	512
ESC_OA3817AA	10	512
ESC_OA3816AA	10	512
ESC_OA3813AA	10	512
ESC_OA3812AA	10	512
ESC_OA3811AA	10	512
ESC_OA3810AA	10	512
ESC_OA3809AA	10	512
ESC_OA3808AA	10	512
ESC_OA3807AA	10	512
ESC_OA3806AA	10	512
ESC_OA3805AA	10	512
ESC_OA3804AA	10	512
ESC_OA3803AA	10	512
ESC_OA3802AA	10	512
ESC_OA3801AA	10	512
ESC_OA3800AA	10	512
ESC_OA3799AA	10	512
ESC_OA3798AA	10	512
ESC_OA3797AA	10	512
ESC_OA3796AA	10	512
ESC_OA3795AA	10	512
ESC_OA3794AA	10	512
ESC_OA3793AA	10	512
ESC_OA3792AA	10	512
ESC_OA3791AA	10	512
ESC_OA3790AA	10	512
ESC_OA3789AA	10	512
ESC_OA3788AA	10	512
ESC_OA3787AA	10	512
ESC_OA3652AA	245	261
ESC_OA3651AA	243	197
ESC_OA3650AA	152	257
ESC_OA3649AA	152	257
ESC_OA3648AA	270	204
ESC_OA3647AA	152	257

ESC_OA3646AA	3101	160
ESC_OA3645AA	6260	156
ESC_OA3644AA	33	198
ESC_OA3643AA	691	899
ESC_OA3642AA	34	504
ESC_OA3641AA	11	936
ESC_OA3640AA	33	198
ESC_OA3639AA	101	696
ESC_OA3638AA	21	156
ESC_OA3637AA	937	198
ESC_OA3636AA	38	669
ESC_OA3635AA	38	669
ESC_OA3634AA	773	71
ESC_OA3633AA	48	76
ESC_OA3630AA	351	268
ESC_OA3628AA	117	592
ESC_OA3626AA	57	50
ESC_OA3625AA	69	387
ESC_OA3624AA	10	512
ESC_OA3621AA	1818	71
ESC_OA3613AA	95	738
ESC_OA3611AA	1060	512
ESC_OA3586AA	245	261
ESC_OA3585AA	245	261
ESC_OA3584AA	152	257
ESC_OA3583AA	152	257
ESC_OA3582AA	148	191
ESC_OA3581AA	245	261
ESC_OA3580AA	145	256
ESC_OA3579AA	148	191
ESC_OA3578AA	245	261
ESC_OA3577AA	10203	261
ESC_OA3576AA	245	261
ESC_OA3575AA	245	261
ESC_OA3574AA	152	257
ESC_OA3573AA	245	261
ESC_OA3572AA	152	257
ESC_OA3571AA	245	261
ESC_OA3570AA	145	256
ESC_OA3568AA	245	261
ESC_OA3567AA	245	261
ESC_OA3566AA	152	257
ESC_OA3565AA	152	257
ESC_OA3564AA	245	261
ESC_OA3563AA	245	261
ESC_OA3562AA	152	257
ESC_OA3561AA	245	261
ESC_OA3560AA	245	261
ESC_OA3559AA	152	257
ESC_OA3558AA	152	257

ESC_OA3557AA	152	257
ESC_OA3556AA	152	257
ESC_OA3555AA	11	936
ESC_OA3554AA	11	936
ESC_OA3553AA	21	156
ESC_OA3552AA	205	257
ESC_OA3551AA	2387	261
ESC_OA3550AA	21	156
ESC_OA3549AA	33	198
ESC_OA3548AA	11	936
ESC_OA3547AA	11	936
ESC_OA3545AA	21	156
ESC_OA3544AA	11	936
ESC_OA3543AA	342	286
ESC_OA3542AA	11	936
ESC_OA3541AA	10	512
ESC_OA3540AA	21	156
ESC_OA3539AA	11	936
ESC_OA3538AA	11	936
ESC_OA3537AA	11	936
ESC_OA3536AA	11	936
ESC_OA3535AA	21	156
ESC_OA3534AA	131	653
ESC_OA3533AA	21	156
ESC_OA3532AA	21	156
ESC_OA3531AA	11	936
ESC_OA3530AA	25	234
ESC_OA3529AA	11	936
ESC_OA3528AA	21	156
ESC_OA3527AA	21	156
ESC_OA3526AA	21	156
ESC_OA3525AA	442	257
ESC_OA3524AA	738	598
ESC_OA3523AA	11	936
ESC_OA3522AA	442	257
ESC_OA3521AA	10202	257
ESC_OA3520AA	11	936
ESC_OA3519AA	17	191
ESC_OA3518AA	33	198
ESC_OA3517AA	205	257
ESC_OA3516AA	141	644
ESC_OA3514AA	141	644
ESC_OA3513AA	141	644
ESC_OA3512AA	141	644
ESC_OA3511AA	141	644
ESC_OA3510AA	141	644
ESC_OA3509AA	141	644
ESC_OA3508AA	442	257
ESC_OA3507AA	162	924
ESC_OA3506AA	11	936

ESC_OA3505AA	11	936
ESC_OA3504AA	152	257
ESC_OA3503AA	152	257
ESC_OA3502AA	152	257
ESC_OA3501AA	245	261
ESC_OA3500AA	245	261
ESC_OA3499AA	152	257
ESC_OA3498AA	152	257
ESC_OA3497AA	11	936
ESC_OA3496AA	8211	257
ESC_OA3495AA	206	286
ESC_OA3494AA	11	936
ESC_OA3493AA	11	936
ESC_OA3492AA	5559	257
ESC_OA3491AA	11	936
ESC_OA3490AA	11	936
ESC_OA3489AA	372	424
ESC_OA3488AA	998	644
ESC_OA3487AA	11	936
ESC_OA3486AA	6683	924
ESC_OA3485AA	718	257
ESC_OA3484AA	2520	878
ESC_OA3483AA	11	936
ESC_OA3482AA	73	737
ESC_OA3481AA	5559	257
ESC_OA3480AA	11	936
ESC_OA3479AA	11	936
ESC_OA3478AA	11	936
ESC_OA3477AA	206	286
ESC_OA3476AA	32	270
ESC_OA3475AA	11	936
ESC_OA3474AA	646	738
ESC_OA3473AA	1850	269
ESC_OA3472AA	11	936
ESC_OA3471AA	11	936
ESC_OA3470AA	21	156
ESC_OA3469AA	11	936
ESC_OA3468AA	11	936
ESC_OA3467AA	17	191
ESC_OA3466AA	21	156
ESC_OA3465AA	5559	257
ESC_OA3464AA	6274	257
ESC_OA3463AA	3778	592
ESC_OA3462AA	4520	160
ESC_OA3461AA	6914	153
ESC_OA3460AA	8347	653
ESC_OA3459AA	847	257
ESC_OA3458AA	744	512
ESC_OA3457AA	752	512
ESC_OA3456AA	69	387

ESC_OA3455AA	155	175
ESC_OA3454AA	939	198
ESC_OA3453AA	1266	210
ESC_OA3452AA	10	512
ESC_OA3451AA	29	156
ESC_OA3450AA	29	156
ESC_OA3449AA	10201	737
ESC_OA3448AA	10200	670
ESC_OA3447AA	10196	761
ESC_OA3446AA	10195	512
ESC_OA3445AA	10198	653
ESC_OA3444AA	10199	7
ESC_OA3441AA	10197	212
ESC_OA3440AA	10150	584
ESC_OA3389AA	152	257
ESC_OA3388AA	301	307
ESC_OA3387AA	131	653
ESC_OA3386AA	131	653
ESC_OA3385AA	617	511
ESC_OA3384AA	131	653
ESC_OA3383AA	131	653
ESC_OA3382AA	131	653
ESC_OA3381AA	131	653
ESC_OA3380AA	405	923
ESC_OA3379AA	131	653
ESC_OA3378AA	131	653
ESC_OA3377AA	405	923
ESC_OA3376AA	131	653
ESC_OA3375AA	167	512
ESC_OA3374AA	131	653
ESC_OA3373AA	131	653
ESC_OA3372AA	44	512
ESC_OA3371AA	131	653
ESC_OA3370AA	38	669
ESC_OA3369AA	2659	679
ESC_OA3368AA	5756	156
ESC_OA3367AA	131	653
ESC_OA3366AA	131	653
ESC_OA3365AA	131	653
ESC_OA3364AA	109	257
ESC_OA3363AA	131	653
ESC_OA3362AA	131	653
ESC_OA3361AA	1308	257
ESC_OA3360AA	131	653
ESC_OA3359AA	131	653
ESC_OA3358AA	131	653
ESC_OA3357AA	131	653
ESC_OA3356AA	131	653
ESC_OA3355AA	131	653
ESC_OA3354AA	131	653

ESC_OA3353AA	131	653
ESC_OA3352AA	88	160
ESC_OA3351AA	187	257
ESC_OA3350AA	278	924
ESC_OA3349AA	131	653
ESC_OA3348AA	1508	261
ESC_OA3347AA	1308	257
ESC_OA3346AA	201	257
ESC_OA3345AA	1308	257
ESC_OA3344AA	131	653
ESC_OA3343AA	278	924
ESC_OA3342AA	201	257
ESC_OA3341AA	131	653
ESC_OA3340AA	131	653
ESC_OA3339AA	131	653
ESC_OA3338AA	201	257
ESC_OA3337AA	278	924
ESC_OA3336AA	1400	666
ESC_OA3335AA	164	175
ESC_OA3334AA	2536	261
ESC_OA3333AA	131	653
ESC_OA3332AA	10	512
ESC_OA3331AA	278	924
ESC_OA3330AA	278	924
ESC_OA3329AA	48	76
ESC_OA3328AA	131	653
ESC_OA3327AA	131	653
ESC_OA3326AA	131	653
ESC_OA3325AA	131	653
ESC_OA3324AA	278	924
ESC_OA3323AA	187	257
ESC_OA3322AA	187	257
ESC_OA3321AA	131	653
ESC_OA3320AA	10173	447
ESC_OA3319AA	681	393
ESC_OA3318AA	5756	156
ESC_OA3317AA	8863	261
ESC_OA3316AA	1400	666
ESC_OA3315AA	681	393
ESC_OA3314AA	10173	447
ESC_OA3313AA	164	175
ESC_OA3312AA	10	512
ESC_OA3311AA	10	512
ESC_OA3310AA	1161	764
ESC_OA3309AA	1121	198
ESC_OA3308AA	1161	764
ESC_OA3307AA	1123	257
ESC_OA3306AA	2035	113
ESC_OA3305AA	10194	204
ESC_OA3304AA	164	175

ESC_OA3303AA	2425	257
ESC_OA3302AA	1161	764
ESC_OA3301AA	1161	764
ESC_OA3300AA	1121	198
ESC_OA3299AA	1610	198
ESC_OA3298AA	1122	307
ESC_OA3297AA	164	175
ESC_OA3296AA	10193	175
ESC_OA3295AA	1610	198
ESC_OA3294AA	154	257
ESC_OA3293AA	4054	198
ESC_OA3292AA	423	204
ESC_OA3291AA	164	175
ESC_OA3290AA	1161	764
ESC_OA3289AA	1252	567
ESC_OA3288AA	1161	764
ESC_OA3287AA	12	626
ESC_OA3286AA	10	512
ESC_OA3285AA	2346	435
ESC_OA3284AA	10192	592
ESC_OA3283AA	405	923
ESC_OA3281AA	3076	175
ESC_OA3280AA	131	653
ESC_OA3279AA	1193	761
ESC_OA3278AA	11	936
ESC_OA3277AA	11	936
ESC_OA3276AA	11	936
ESC_OA3275AA	301	307
ESC_OA3274AA	21	156
ESC_OA3273AA	21	156
ESC_OA3272AA	21	156
ESC_OA3271AA	21	156
ESC_OA3270AA	245	261
ESC_OA3269AA	152	257
ESC_OA3268AA	245	261
ESC_OA3267AA	152	257
ESC_OA3266AA	152	257
ESC_OA3265AA	245	261
ESC_OA3264AA	245	261
ESC_OA3263AA	6200	261
ESC_OA3262AA	11	936
ESC_OA3261AA	677	257
ESC_OA3260AA	127	738
ESC_OA3259AA	34	504
ESC_OA3258AA	10	512
ESC_OA3257AA	442	257
ESC_OA3256AA	300	286
ESC_OA3255AA	17	191
ESC_OA3254AA	3076	175
ESC_OA3253AA	131	653

ESC_OA3252AA	1015	175
ESC_OA3251AA	131	653
ESC_OA3250AA	10	512
ESC_OA3249AA	349	670
ESC_OA3248AA	38	669
ESC_OA3247AA	5559	257
ESC_OA3246AA	5559	257
ESC_OA3245AA	17	191
ESC_OA3244AA	21	156
ESC_OA3243AA	5559	257
ESC_OA3242AA	5559	257
ESC_OA3241AA	21	156
ESC_OA3240AA	131	653
ESC_OA3239AA	744	512
ESC_OA3238AA	442	257
ESC_OA3237AA	155	175
ESC_OA3235AA	602	257
ESC_OA3234AA	155	175
ESC_OA3233AA	69	387
ESC_OA3232AA	2175	212
ESC_OA3231AA	973	968
ESC_OA3228AA	11	936
ESC_OA3227AA	11	936
ESC_OA3226AA	11	936
ESC_OA3225AA	11	936
ESC_OA3224AA	11	936
ESC_OA3222AA	11	936
ESC_OA3221AA	11	936
ESC_OA3220AA	11	936
ESC_OA3219AA	11	936
ESC_OA3218AA	11	936
ESC_OA3217AA	11	936
ESC_OA3216AA	11	936
ESC_OA3215AA	11	936
ESC_OA3214AA	11	936
ESC_OA3212AA	11	936
ESC_OA3211AA	11	936
ESC_OA3209AA	11	936
ESC_OA3208AA	11	936
ESC_OA3207AA	11	936
ESC_OA3206AA	16	156
ESC_OA3205AA	58	198
ESC_OA3204AA	349	670
ESC_OA3203AA	108	257
ESC_OA3202AA	641	935
ESC_OA3201AA	69	387
ESC_OA3200AA	88	160
ESC_OA3199AA	131	653
ESC_OA3198AA	648	359
ESC_OA3197AA	38	669

ESC_OA3196AA	162	924
ESC_OA3195AA	131	653
ESC_OA3194AA	648	359
ESC_OA3193AA	795	924
ESC_OA3192AA	17	191
ESC_OA3191AA	16	156
ESC_OA3190AA	21	156
ESC_OA3189AA	3258	592
ESC_OA3188AA	155	175
ESC_OA3187AA	1432	257
ESC_OA3186AA	3580	257
ESC_OA3185AA	10	512
ESC_OA3184AA	155	175
ESC_OA3183AA	21	156
ESC_OA3182AA	21	156
ESC_OA3181AA	7648	191
ESC_OA3180AA	501	900
ESC_OA3179AA	4481	257
ESC_OA3178AA	898	108
ESC_OA3177AA	58	198
ESC_OA3176AA	88	160
ESC_OA3175AA	108	257
ESC_OA3174AA	23	156
ESC_OA3173AA	361	512
ESC_OA3172AA	1080	198
ESC_OA3171AA	38	669
ESC_OA3170AA	212	198
ESC_OA3169AA	10	512
ESC_OA3168AA	2778	221
ESC_OA3167AA	746	789
ESC_OA3166AA	88	160
ESC_OA3165AA	109	257
ESC_OA3164AA	58	198
ESC_OA3163AA	56	198
ESC_OA3162AA	1001	257
ESC_OA3161AA	10	512
ESC_OA3160AA	58	198
ESC_OA3159AA	4087	512
ESC_OA3158AA	1193	761
ESC_OA3157AA	69	387
ESC_OA3156AA	88	160
ESC_OA3155AA	648	359
ESC_OA3154AA	1491	76
ESC_OA3153AA	226	512
ESC_OA3152AA	101	696
ESC_OA3151AA	10191	878
ESC_OA3150AA	7284	386
ESC_OA3149AA	117	592
ESC_OA3148AA	10	512
ESC_OA3147AA	58	198

ESC_OA3146AA	10	512
ESC_OA3145AA	5308	261
ESC_OA3144AA	1079	235
ESC_OA3142AA	31	387
ESC_OA3141AA	1163	579
ESC_OA3140AA	4702	626
ESC_OA3139AA	38	669
ESC_OA3138AA	448	257
ESC_OA3134AA	95	738
ESC_OA3132AA	602	257
ESC_OA3131AA	10190	1004
ESC_OA3130AA	5909	76
ESC_OA3128AA	46	447
ESC_OA3127AA	38	669
ESC_OA3126AA	38	669
ESC_OA3119AA	131	653
ESC_OA3114AA	635	141
ESC_OA3111AA	1312	76
ESC_OA3110AA	648	359
ESC_OA3109AA	38	669
ESC_OA3108AA	38	669
ESC_OA3107AA	131	653
ESC_OA3106AA	38	669
ESC_OA3105AA	38	669
ESC_OA3104AA	131	653
ESC_OA3103AA	38	669
ESC_OA3102AA	38	669
ESC_OA3101AA	38	669
ESC_OA3100AA	38	669
ESC_OA3099AA	38	669
ESC_OA3098AA	38	669
ESC_OA3097AA	38	669
ESC_OA3096AA	38	669
ESC_OA3095AA	38	669
ESC_OA3094AA	227	504
ESC_OA3093AA	131	653
ESC_OA3091AA	38	669
ESC_OA3090AA	38	669
ESC_OA3089AA	38	669
ESC_OA3088AA	38	669
ESC_OA3087AA	38	669
ESC_OA3086AA	38	669
ESC_OA3085AA	38	669
ESC_OA3084AA	38	669
ESC_OA3083AA	38	669
ESC_OA3082AA	69	387
ESC_OA3081AA	648	359
ESC_OA3080AA	38	669
ESC_OA3079AA	5051	669
ESC_OA3078AA	38	669

ESC_OA3077AA	38	669
ESC_OA3076AA	38	669
ESC_OA3075AA	69	387
ESC_OA3074AA	69	387
ESC_OA3072AA	38	669
ESC_OA3071AA	46	447
ESC_OA3070AA	131	653
ESC_OA3069AA	38	669
ESC_OA3068AA	38	669
ESC_OA3067AA	38	669
ESC_OA3066AA	38	669
ESC_OA3065AA	3268	669
ESC_OA3064AA	38	669
ESC_OA3063AA	38	669
ESC_OA3062AA	38	669
ESC_OA3061AA	167	512
ESC_OA3060AA	38	669
ESC_OA3059AA	38	669
ESC_OA3058AA	361	512
ESC_OA3057AA	38	669
ESC_OA3056AA	38	669
ESC_OA3055AA	38	669
ESC_OA3054AA	38	669
ESC_OA3053AA	38	669
ESC_OA3052AA	38	669
ESC_OA3051AA	131	653
ESC_OA3050AA	224	198
ESC_OA3049AA	38	669
ESC_OA3048AA	38	669
ESC_OA3047AA	361	512
ESC_OA3046AA	38	669
ESC_OA3045AA	453	935
ESC_OA3044AA	131	653
ESC_OA3043AA	1485	408
ESC_OA3042AA	38	669
ESC_OA3041AA	38	669
ESC_OA3040AA	38	669
ESC_OA3039AA	38	669
ESC_OA3038AA	131	653
ESC_OA3037AA	457	595
ESC_OA3036AA	131	653
ESC_OA3035AA	5150	661
ESC_OA3034AA	93	71
ESC_OA3033AA	43	512
ESC_OA3032AA	38	669
ESC_OA3031AA	38	669
ESC_OA3030AA	167	512
ESC_OA3029AA	131	653
ESC_OA3028AA	7077	519
ESC_OA3027AA	32	270

ESC_OA3026AA	11	936
ESC_OA3025AA	442	257
ESC_OA3024AA	11	936
ESC_OA3023AA	11	936
ESC_OA3022AA	7077	519
ESC_OA3021AA	335	936
ESC_OA3020AA	648	359
ESC_OA3019AA	38	669
ESC_OA3018AA	38	669
ESC_OA3017AA	10	512
ESC_OA3016AA	10	512
ESC_OA3015AA	10	512
ESC_OA3014AA	648	359
ESC_OA3013AA	10048	286
ESC_OA3012AA	38	669
ESC_OA3010AA	38	669
ESC_OA3009AA	10189	868
ESC_OA3008AA	167	512
ESC_OA3007AA	648	359
ESC_OA3006AA	10	512
ESC_OA3005AA	1193	761
ESC_OA3004AA	38	669
ESC_OA3003AA	38	669
ESC_OA3002AA	69	387
ESC_OA3001AA	38	669
ESC_OA3000AA	1177	669
ESC_OA2999AA	484	71
ESC_OA2998AA	1193	761
ESC_OA2997AA	216	512
ESC_OA2996AA	38	669
ESC_OA2995AA	1177	669
ESC_OA2994AA	95	738
ESC_OA2993AA	1638	519
ESC_OA2992AA	73	737
ESC_OA2991AA	1249	257
ESC_OA2990AA	69	387
ESC_OA2989AA	10188	696
ESC_OA2987AA	101	696
ESC_OA2986AA	38	669
ESC_OA2985AA	10	512
ESC_OA2984AA	69	387
ESC_OA2983AA	69	387
ESC_OA2982AA	10	512
ESC_OA2981AA	95	738
ESC_OA2980AA	131	653
ESC_OA2979AA	6237	291
ESC_OA2978AA	2003	669
ESC_OA2977AA	718	257
ESC_OA2976AA	131	653
ESC_OA2975AA	167	512

ESC_OA2974AA	648	359
ESC_OA2973AA	131	653
ESC_OA2972AA	131	653
ESC_OA2971AA	131	653
ESC_OA2970AA	131	653
ESC_OA2969AA	131	653
ESC_OA2968AA	131	653
ESC_OA2967AA	131	653
ESC_OA2966AA	2171	164
ESC_OA2965AA	345	175
ESC_OA2964AA	38	669
ESC_OA2963AA	131	653
ESC_OA2962AA	38	669
ESC_OA2961AA	38	669
ESC_OA2960AA	5559	257
ESC_OA2959AA	5559	257
ESC_OA2958AA	329	355
ESC_OA2957AA	5559	257
ESC_OA2956AA	206	286
ESC_OA2955AA	5559	257
ESC_OA2954AA	5559	257
ESC_OA2953AA	5559	257
ESC_OA2952AA	167	512
ESC_OA2951AA	302	850
ESC_OA2950AA	10185	71
ESC_OA2949AA	10	512
ESC_OA2948AA	73	737
ESC_OA2947AA	648	359
ESC_OA2946AA	10184	387
ESC_OA2944AA	2253	71
ESC_OA2943AA	88	160
ESC_OA2942AA	131	653
ESC_OA2941AA	131	653
ESC_OA2940AA	131	653
ESC_OA2939AA	744	512
ESC_OA2938AA	131	653
ESC_OA2937AA	131	653
ESC_OA2936AA	131	653
ESC_OA2935AA	405	923
ESC_OA2934AA	131	653
ESC_OA2933AA	131	653
ESC_OA2932AA	131	653
ESC_OA2931AA	131	653
ESC_OA2930AA	10183	198
ESC_OA2929AA	226	512
ESC_OA2928AA	398	445
ESC_OA2927AA	131	653
ESC_OA2926AA	641	935
ESC_OA2925AA	11	936
ESC_OA2924AA	399	198

ESC_OA2923AA	11	936
ESC_OA2922AA	4995	198
ESC_OA2921AA	11	936
ESC_OA2920AA	2531	198
ESC_OA2919AA	1139	447
ESC_OA2918AA	48	76
ESC_OA2917AA	685	311
ESC_OA2916AA	10186	504
ESC_OA2915AA	6852	512
ESC_OA2913AA	21	156
ESC_OA2912AA	450	71
ESC_OA2911AA	1193	761
ESC_OA2910AA	716	795
ESC_OA2909AA	88	160
ESC_OA2908AA	11	936
ESC_OA2814AA	648	359
ESC_OA2813AA	131	653
ESC_OA2812AA	131	653
ESC_OA2811AA	131	653
ESC_OA2810AA	131	653
ESC_OA2809AA	131	653
ESC_OA2808AA	167	512
ESC_OA2807AA	131	653
ESC_OA2806AA	44	512
ESC_OA2805AA	131	653
ESC_OA2804AA	648	359
ESC_OA2802AA	472	601
ESC_OA2801AA	131	653
ESC_OA2800AA	5150	661
ESC_OA2799AA	152	257
ESC_OA2797AA	38	669
ESC_OA2796AA	1177	669
ESC_OA2795AA	38	669
ESC_OA2794AA	301	307
ESC_OA2793AA	101	696
ESC_OA2790AA	131	653
ESC_OA2789AA	4702	626
ESC_OA2788AA	152	257
ESC_OA2787AA	152	257
ESC_OA2786AA	152	257
ESC_OA2785AA	2003	669
ESC_OA2784AA	38	669
ESC_OA2783AA	167	512
ESC_OA2782AA	349	670
ESC_OA2781AA	1290	515
ESC_OA2780AA	23	156
ESC_OA2779AA	3177	362
ESC_OA2778AA	38	669
ESC_OA2777AA	38	669
ESC_OA2776AA	38	669

ESC_OA2775AA	3052	669
ESC_OA2774AA	101	696
ESC_OA2773AA	131	653
ESC_OA2772AA	69	387
ESC_OA2771AA	69	387
ESC_OA2770AA	69	387
ESC_OA2769AA	69	387
ESC_OA2768AA	69	387
ESC_OA2767AA	69	387
ESC_OA2766AA	11	936
ESC_OA2765AA	1727	257
ESC_OA2764AA	681	393
ESC_OA2763AA	164	175
ESC_OA2762AA	4038	257
ESC_OA2761AA	1609	198
ESC_OA2760AA	58	198
ESC_OA2759AA	10	512
ESC_OA2758AA	164	175
ESC_OA2757AA	2035	113
ESC_OA2756AA	906	198
ESC_OA2755AA	2008	257
ESC_OA2754AA	10	512
ESC_OA2753AA	2678	737
ESC_OA2752AA	155	175
ESC_OA2751AA	1161	764
ESC_OA2750AA	4054	198
ESC_OA2749AA	1609	198
ESC_OA2748AA	154	257
ESC_OA2747AA	4054	198
ESC_OA2746AA	919	421
ESC_OA2745AA	164	175
ESC_OA2744AA	2008	257
ESC_OA2743AA	8518	934
ESC_OA2742AA	345	175
ESC_OA2741AA	58	198
ESC_OA2740AA	4038	257
ESC_OA2739AA	58	198
ESC_OA2738AA	1508	261
ESC_OA2737AA	4038	257
ESC_OA2736AA	58	198
ESC_OA2735AA	1508	261
ESC_OA2734AA	1508	261
ESC_OA2733AA	1508	261
ESC_OA2732AA	603	198
ESC_OA2731AA	1508	261
ESC_OA2730AA	1665	601
ESC_OA2729AA	1508	261
ESC_OA2728AA	1508	261
ESC_OA2727AA	58	198
ESC_OA2726AA	58	198

ESC_OA2725AA	58	198
ESC_OA2724AA	1508	261
ESC_OA2723AA	1508	261
ESC_OA2722AA	1508	261
ESC_OA2721AA	345	175
ESC_OA2720AA	58	198
ESC_OA2719AA	1508	261
ESC_OA2718AA	1508	261
ESC_OA2716AA	10173	447
ESC_OA2715AA	2520	878
ESC_OA2714AA	58	198
ESC_OA2713AA	1079	235
ESC_OA2712AA	1080	198
ESC_OA2711AA	410	156
ESC_OA2710AA	1204	961
ESC_OA2709AA	967	212
ESC_OA2708AA	2144	257
ESC_OA2707AA	441	198
ESC_OA2706AA	58	198
ESC_OA2705AA	10	512
ESC_OA2704AA	345	175
ESC_OA2703AA	1172	234
ESC_OA2702AA	4173	234
ESC_OA2701AA	155	175
ESC_OA2700AA	4080	300
ESC_OA2699AA	661	92
ESC_OA2698AA	187	257
ESC_OA2697AA	5968	519
ESC_OA2696AA	641	935
ESC_OA2695AA	17	191
ESC_OA2694AA	1252	567
ESC_OA2693AA	58	198
ESC_OA2692AA	2325	447
ESC_OA2691AA	173	254
ESC_OA2690AA	58	198
ESC_OA2689AA	8576	212
ESC_OA2688AA	88	160
ESC_OA2687AA	5903	180
ESC_OA2686AA	297	234
ESC_OA2685AA	173	254
ESC_OA2684AA	187	257
ESC_OA2683AA	641	935
ESC_OA2682AA	2522	198
ESC_OA2681AA	6559	234
ESC_OA2680AA	906	198
ESC_OA2679AA	173	254
ESC_OA2678AA	10	512
ESC_OA2677AA	10	512
ESC_OA2676AA	88	160
ESC_OA2674AA	795	924

ESC_OA2673AA	1582	198
ESC_OA2672AA	7488	156
ESC_OA2671AA	88	160
ESC_OA2670AA	48	76
ESC_OA2669AA	23	156
ESC_OA2668AA	58	198
ESC_OA2667AA	93	71
ESC_OA2666AA	101	696
ESC_OA2665AA	75	257
ESC_OA2664AA	898	108
ESC_OA2663AA	57	50
ESC_OA2661AA	23	156
ESC_OA2660AA	416	738
ESC_OA2659AA	23	156
ESC_OA2658AA	69	387
ESC_OA2657AA	117	592
ESC_OA2656AA	155	175
ESC_OA2653AA	1952	880
ESC_OA2652AA	1167	257
ESC_OA2651AA	57	50
ESC_OA2650AA	410	156
ESC_OA2649AA	75	257
ESC_OA2648AA	189	504
ESC_OA2647AA	710	291
ESC_OA2646AA	88	160
ESC_OA2645AA	101	696
ESC_OA2644AA	101	696
ESC_OA2642AA	88	160
ESC_OA2641AA	117	592
ESC_OA2640AA	1684	504
ESC_OA2638AA	410	156
ESC_OA2637AA	1684	504
ESC_OA2636AA	155	175
ESC_OA2635AA	88	160
ESC_OA2634AA	641	935
ESC_OA2633AA	1684	504
ESC_OA2632AA	10	512
ESC_OA2631AA	101	696
ESC_OA2630AA	3088	311
ESC_OA2629AA	88	160
ESC_OA2628AA	23	156
ESC_OA2627AA	4674	592
ESC_OA2626AA	57	50
ESC_OA2625AA	69	387
ESC_OA2624AA	88	160
ESC_OA2623AA	345	175
ESC_OA2621AA	117	592
ESC_OA2620AA	58	198
ESC_OA2619AA	410	156
ESC_OA2618AA	88	160

ESC_OA2617AA	101	696
ESC_OA2616AA	10	512
ESC_OA2615AA	117	592
ESC_OA2614AA	57	50
ESC_OA2613AA	1112	504
ESC_OA2612AA	23	156
ESC_OA2610AA	5415	924
ESC_OA2609AA	3776	175
ESC_OA2608AA	410	156
ESC_OA2607AA	1112	504
ESC_OA2606AA	410	156
ESC_OA2605AA	410	156
ESC_OA2604AA	48	76
ESC_OA2603AA	3321	935
ESC_OA2601AA	23	156
ESC_OA2600AA	10160	191
ESC_OA2599AA	75	257
ESC_OA2598AA	88	160
ESC_OA2597AA	457	595
ESC_OA2596AA	69	387
ESC_OA2595AA	2175	212
ESC_OA2594AA	3057	672
ESC_OA2593AA	3776	175
ESC_OA2592AA	101	696
ESC_OA2591AA	2230	126
ESC_OA2590AA	398	445
ESC_OA2589AA	10	512
ESC_OA2588AA	10	512
ESC_OA2587AA	101	696
ESC_OA2586AA	135	653
ESC_OA2585AA	57	50
ESC_OA2584AA	5876	847
ESC_OA2583AA	58	198
ESC_OA2582AA	10161	257
ESC_OA2581AA	88	160
ESC_OA2580AA	88	160
ESC_OA2579AA	410	156
ESC_OA2578AA	23	156
ESC_OA2577AA	69	387
ESC_OA2576AA	69	387
ESC_OA2575AA	88	160
ESC_OA2574AA	3776	175
ESC_OA2573AA	69	387
ESC_OA2572AA	101	696
ESC_OA2571AA	88	160
ESC_OA2570AA	453	935
ESC_OA2569AA	101	696
ESC_OA2566AA	3776	175
ESC_OA2565AA	69	387
ESC_OA2564AA	69	387

ESC_OA2563AA	117	592
ESC_OA2562AA	369	156
ESC_OA2561AA	877	935
ESC_OA2560AA	155	175
ESC_OA2559AA	641	935
ESC_OA2558AA	117	592
ESC_OA2557AA	10	512
ESC_OA2556AA	10	512
ESC_OA2555AA	101	696
ESC_OA2554AA	75	257
ESC_OA2553AA	58	198
ESC_OA2552AA	10164	156
ESC_OA2551AA	58	198
ESC_OA2550AA	641	935
ESC_OA2549AA	1011	198
ESC_OA2548AA	683	175
ESC_OA2547AA	155	175
ESC_OA2546AA	847	257
ESC_OA2545AA	155	175
ESC_OA2544AA	38	669
ESC_OA2543AA	5415	924
ESC_OA2542AA	93	71
ESC_OA2541AA	2064	261
ESC_OA2540AA	10163	924
ESC_OA2539AA	88	160
ESC_OA2538AA	57	50
ESC_OA2537AA	165	504
ESC_OA2535AA	3595	822
ESC_OA2534AA	10	512
ESC_OA2533AA	5876	847
ESC_OA2532AA	20	198
ESC_OA2531AA	23	156
ESC_OA2530AA	88	160
ESC_OA2529AA	877	935
ESC_OA2528AA	88	160
ESC_OA2527AA	117	592
ESC_OA2526AA	88	160
ESC_OA2525AA	101	696
ESC_OA2523AA	10162	111
ESC_OA2521AA	10	512
ESC_OA2520AA	154	257
ESC_OA2519AA	57	50
ESC_OA2518AA	93	71
ESC_OA2517AA	58	198
ESC_OA2516AA	88	160
ESC_OA2515AA	57	50
ESC_OA2514AA	58	198
ESC_OA2513AA	88	160
ESC_OA2512AA	117	592
ESC_OA2511AA	75	257

ESC_OA2510AA	10	512
ESC_OA2509AA	69	387
ESC_OA2508AA	23	156
ESC_OA2507AA	3321	935
ESC_OA2506AA	7632	512
ESC_OA2505AA	369	156
ESC_OA2504AA	793	286
ESC_OA2503AA	641	935
ESC_OA2502AA	88	160
ESC_OA2501AA	154	257
ESC_OA2500AA	44	512
ESC_OA2499AA	34	504
ESC_OA2498AA	898	108
ESC_OA2497AA	641	935
ESC_OA2496AA	10	512
ESC_OA2494AA	5876	847
ESC_OA2493AA	8863	261
ESC_OA2492AA	10	512
ESC_OA2491AA	38	669
ESC_OA2490AA	278	924
ESC_OA2489AA	410	156
ESC_OA2488AA	4373	831
ESC_OA2487AA	44	512
ESC_OA2486AA	617	511
ESC_OA2485AA	10	512
ESC_OA2484AA	10166	298
ESC_OA2482AA	93	71
ESC_OA2481AA	59	217
ESC_OA2480AA	410	156
ESC_OA2479AA	58	198
ESC_OA2478AA	10	512
ESC_OA2477AA	7588	447
ESC_OA2476AA	10	512
ESC_OA2475AA	93	71
ESC_OA2474AA	88	160
ESC_OA2472AA	23	156
ESC_OA2470AA	44	512
ESC_OA2469AA	3776	175
ESC_OA2468AA	5876	847
ESC_OA2467AA	117	592
ESC_OA2466AA	1056	257
ESC_OA2465AA	1139	447
ESC_OA2464AA	3776	175
ESC_OA2463AA	10	512
ESC_OA2462AA	641	935
ESC_OA2461AA	410	156
ESC_OA2460AA	3776	175
ESC_OA2459AA	10165	504
ESC_OA2458AA	162	924
ESC_OA2457AA	131	653

ESC_OA2456AA	2628	878
ESC_OA2455AA	278	924
ESC_OA2454AA	3634	71
ESC_OA2453AA	351	268
ESC_OA2452AA	3014	410
ESC_OA2451AA	93	71
ESC_OA2450AA	23	156
ESC_OA2448AA	515	1004
ESC_OA2447AA	1196	257
ESC_OA2446AA	410	156
ESC_OA2445AA	10	512
ESC_OA2444AA	23	156
ESC_OA2443AA	542	824
ESC_OA2442AA	88	160
ESC_OA2441AA	410	156
ESC_OA2440AA	10169	474
ESC_OA2439AA	10	512
ESC_OA2438AA	410	156
ESC_OA2437AA	101	696
ESC_OA2436AA	58	198
ESC_OA2435AA	23	156
ESC_OA2434AA	847	257
ESC_OA2433AA	410	156
ESC_OA2429AA	155	175
ESC_OA2428AA	57	50
ESC_OA2427AA	683	175
ESC_OA2426AA	23	156
ESC_OA2425AA	3776	175
ESC_OA2424AA	10168	156
ESC_OA2423AA	101	696
ESC_OA2422AA	48	76
ESC_OA2421AA	117	592
ESC_OA2420AA	101	696
ESC_OA2419AA	58	198
ESC_OA2418AA	117	592
ESC_OA2417AA	10	512
ESC_OA2415AA	3776	175
ESC_OA2414AA	189	504
ESC_OA2413AA	101	696
ESC_OA2412AA	744	512
ESC_OA2411AA	10	512
ESC_OA2409AA	5341	924
ESC_OA2408AA	101	696
ESC_OA2407AA	48	76
ESC_OA2406AA	410	156
ESC_OA2405AA	457	595
ESC_OA2404AA	793	286
ESC_OA2403AA	88	160
ESC_OA2402AA	44	512
ESC_OA2401AA	189	504

ESC_OA2400AA	69	387
ESC_OA2399AA	88	160
ESC_OA2398AA	206	286
ESC_OA2397AA	10167	76
ESC_OA2396AA	93	71
ESC_OA2395AA	137	270
ESC_OA2394AA	10162	111
ESC_OA2393AA	3519	101
ESC_OA2391AA	93	71
ESC_OA2390AA	767	198
ESC_OA2389AA	10	512
ESC_OA2388AA	10	512
ESC_OA2387AA	1114	504
ESC_OA2386AA	648	359
ESC_OA2385AA	46	447
ESC_OA2384AA	410	156
ESC_OA2383AA	23	156
ESC_OA2382AA	117	592
ESC_OA2380AA	10	512
ESC_OA2379AA	58	198
ESC_OA2378AA	10172	160
ESC_OA2377AA	2073	198
ESC_OA2376AA	57	50
ESC_OA2375AA	2628	878
ESC_OA2374AA	57	50
ESC_OA2373AA	131	653
ESC_OA2372AA	542	824
ESC_OA2371AA	877	935
ESC_OA2370AA	88	160
ESC_OA2369AA	767	198
ESC_OA2368AA	2230	126
ESC_OA2367AA	1722	599
ESC_OA2366AA	88	160
ESC_OA2365AA	602	257
ESC_OA2364AA	57	50
ESC_OA2363AA	58	198
ESC_OA2362AA	20	198
ESC_OA2361AA	2144	257
ESC_OA2360AA	23	156
ESC_OA2359AA	10	512
ESC_OA2358AA	88	160
ESC_OA2357AA	10175	71
ESC_OA2356AA	10171	512
ESC_OA2355AA	871	447
ESC_OA2354AA	410	156
ESC_OA2353AA	641	935
ESC_OA2352AA	795	924
ESC_OA2351AA	10174	37
ESC_OA2350AA	88	160
ESC_OA2349AA	1914	956

ESC_OA2348AA	7584	156
ESC_OA2347AA	155	175
ESC_OA2346AA	58	198
ESC_OA2345AA	69	387
ESC_OA2344AA	453	935
ESC_OA2343AA	10160	191
ESC_OA2342AA	410	156
ESC_OA2341AA	10	512
ESC_OA2340AA	1632	824
ESC_OA2339AA	10170	512
ESC_OA2338AA	155	175
ESC_OA2337AA	2496	789
ESC_OA2336AA	117	592
ESC_OA2335AA	117	592
ESC_OA2334AA	44	512
ESC_OA2332AA	117	592
ESC_OA2331AA	58	198
ESC_OA2330AA	3205	785
ESC_OA2329AA	3377	268
ESC_OA2327AA	23	156
ESC_OA2326AA	1081	198
ESC_OA2325AA	710	291
ESC_OA2323AA	2496	789
ESC_OA2322AA	117	592
ESC_OA2321AA	156	175
ESC_OA2320AA	6158	935
ESC_OA2318AA	4674	592
ESC_OA2317AA	117	592
ESC_OA2316AA	117	592
ESC_OA2315AA	10	512
ESC_OA2314AA	10	512
ESC_OA2313AA	1139	447
ESC_OA2312AA	3014	410
ESC_OA2310AA	2144	257
ESC_OA2309AA	69	387
ESC_OA2308AA	1279	160
ESC_OA2307AA	101	696
ESC_OA2306AA	10	512
ESC_OA2305AA	410	156
ESC_OA2304AA	101	696
ESC_OA2303AA	3776	175
ESC_OA2302AA	5416	445
ESC_OA2301AA	88	160
ESC_OA2300AA	101	696
ESC_OA2299AA	156	175
ESC_OA2298AA	93	71
ESC_OA2297AA	10177	175
ESC_OA2296AA	167	512
ESC_OA2295AA	410	156
ESC_OA2294AA	23	156

ESC_OA2293AA	10176	257
ESC_OA2292AA	2628	878
ESC_OA2291AA	789	512
ESC_OA2290AA	398	445
ESC_OA2289AA	101	696
ESC_OA2288AA	48	76
ESC_OA2287AA	117	592
ESC_OA2285AA	793	286
ESC_OA2282AA	410	156
ESC_OA2281AA	2230	126
ESC_OA2280AA	410	156
ESC_OA2279AA	6286	592
ESC_OA2278AA	993	789
ESC_OA2277AA	88	160
ESC_OA2276AA	3014	410
ESC_OA2275AA	5945	928
ESC_OA2273AA	1722	599
ESC_OA2272AA	410	156
ESC_OA2271AA	3014	410
ESC_OA2269AA	10	512
ESC_OA2267AA	57	50
ESC_OA2266AA	57	50
ESC_OA2265AA	3595	822
ESC_OA2264AA	23	156
ESC_OA2263AA	1081	198
ESC_OA2262AA	58	198
ESC_OA2261AA	3776	175
ESC_OA2260AA	88	160
ESC_OA2259AA	1167	257
ESC_OA2258AA	5876	847
ESC_OA2257AA	10	512
ESC_OA2255AA	648	359
ESC_OA2254AA	542	824
ESC_OA2253AA	57	50
ESC_OA2252AA	23	156
ESC_OA2251AA	3014	410
ESC_OA2250AA	101	696
ESC_OA2248AA	93	71
ESC_OA2247AA	10181	512
ESC_OA2246AA	126	740
ESC_OA2245AA	2230	126
ESC_OA2244AA	48	76
ESC_OA2242AA	1684	504
ESC_OA2241AA	367	156
ESC_OA2240AA	167	512
ESC_OA2239AA	10174	37
ESC_OA2238AA	88	160
ESC_OA2237AA	10	512
ESC_OA2236AA	117	592
ESC_OA2235AA	3205	785

ESC_OA2234AA	57	50
ESC_OA2233AA	88	160
ESC_OA2232AA	1722	599
ESC_OA2231AA	88	160
ESC_OA2230AA	10	512
ESC_OA2229AA	10	512
ESC_OA2228AA	3205	785
ESC_OA2227AA	75	257
ESC_OA2226AA	23	156
ESC_OA2225AA	126	740
ESC_OA2224AA	1684	504
ESC_OA2223AA	10	512
ESC_OA2222AA	10180	50
ESC_OA2221AA	155	175
ESC_OA2220AA	88	160
ESC_OA2219AA	58	198
ESC_OA2218AA	10179	270
ESC_OA2217AA	23	156
ESC_OA2216AA	3322	824
ESC_OA2214AA	410	156
ESC_OA2212AA	410	156
ESC_OA2211AA	126	740
ESC_OA2210AA	5415	924
ESC_OA2209AA	10	512
ESC_OA2208AA	88	160
ESC_OA2207AA	453	935
ESC_OA2206AA	453	935
ESC_OA2205AA	101	696
ESC_OA2204AA	10178	76
ESC_OA2203AA	117	592
ESC_OA2202AA	410	156
ESC_OA2201AA	5876	847
ESC_OA2200AA	88	160
ESC_OA2199AA	1114	504
ESC_OA2198AA	58	198
ESC_OA2197AA	10178	76
ESC_OA2196AA	2496	789
ESC_OA2194AA	847	257
ESC_OA2193AA	367	156
ESC_OA2192AA	877	935
ESC_OA2191AA	156	175
ESC_OA2190AA	2496	789
ESC_OA2189AA	46	447
ESC_OA2188AA	155	175
ESC_OA2187AA	101	696
ESC_OA2186AA	93	71
ESC_OA2185AA	1114	504
ESC_OA2184AA	56	198
ESC_OA2183AA	2197	511
ESC_OA2182AA	10182	101

ESC_OA2181AA	367	156
ESC_OA2180AA	131	653
ESC_OA2179AA	131	653
ESC_OA2178AA	131	653
ESC_OA2177AA	131	653
ESC_OA2176AA	44	512
ESC_OA2175AA	131	653
ESC_OA2174AA	10	512
ESC_OA2173AA	405	923
ESC_OA2172AA	10	512
ESC_OA2171AA	648	359
ESC_OA2170AA	1148	257
ESC_OA2169AA	101	696
ESC_OA2168AA	155	175
ESC_OA2167AA	58	198
ESC_OA2166AA	23	156
ESC_OA2165AA	10159	198
ESC_OA2164AA	131	653
ESC_OA2163AA	131	653
ESC_OA2162AA	131	653
ESC_OA2161AA	410	156
ESC_OA2160AA	617	511
ESC_OA2159AA	131	653
ESC_OA2158AA	131	653
ESC_OA2157AA	131	653
ESC_OA2156AA	131	653
ESC_OA2155AA	131	653
ESC_OA2154AA	101	696
ESC_OA2152AA	8418	105
ESC_OA2151AA	131	653
ESC_OA2150AA	38	669
ESC_OA2148AA	998	644
ESC_OA2147AA	10	512
ESC_OA2146AA	2461	76
ESC_OA2145AA	648	359
ESC_OA2144AA	7427	233
ESC_OA2143AA	156	175
ESC_OA2142AA	38	669
ESC_OA2141AA	744	512
ESC_OA2140AA	410	156
ESC_OA2139AA	131	653
ESC_OA2138AA	131	653
ESC_OA2137AA	131	653
ESC_OA2136AA	131	653
ESC_OA2135AA	131	653
ESC_OA2134AA	131	653
ESC_OA2133AA	131	653
ESC_OA2132AA	500	71
ESC_OA2131AA	131	653
ESC_OA2120AA	1485	408

ESC_OA2119AA	617	511
ESC_OA2118AA	4380	198
ESC_OA2117AA	10	512
ESC_OA2116AA	152	257
ESC_OA2115AA	245	261
ESC_OA2114AA	245	261
ESC_OA2113AA	152	257
ESC_OA2112AA	152	257
ESC_OA2111AA	1025	261
ESC_OA2110AA	245	261
ESC_OA2109AA	152	257
ESC_OA2108AA	152	257
ESC_OA2107AA	245	261
ESC_OA2106AA	245	261
ESC_OA2105AA	278	924
ESC_OA2104AA	442	257
ESC_OA2103AA	32	270
ESC_OA2102AA	442	257
ESC_OA2101AA	33	198
ESC_OA2100AA	10157	234
ESC_OA2099AA	21	156
ESC_OA2098AA	6260	156
ESC_OA2097AA	25	234
ESC_OA2096AA	442	257
ESC_OA2095AA	33	198
ESC_OA2094AA	378	504
ESC_OA2093AA	656	789
ESC_OA2092AA	405	923
ESC_OA2091AA	661	92
ESC_OA2090AA	33	198
ESC_OA2089AA	131	653
ESC_OA2088AA	131	653
ESC_OA2087AA	131	653
ESC_OA2086AA	405	923
ESC_OA2085AA	131	653
ESC_OA2084AA	131	653
ESC_OA2083AA	405	923
ESC_OA2082AA	10	512
ESC_OA2081AA	131	653
ESC_OA2080AA	405	923
ESC_OA2077AA	131	653
ESC_OA2075AA	131	653
ESC_OA2073AA	131	653
ESC_OA2072AA	131	653
ESC_OA2071AA	131	653
ESC_OA2068AA	131	653
ESC_OA2067AA	131	653
ESC_OA2066AA	131	653
ESC_OA2062AA	131	653
ESC_OA2061AA	131	653

ESC_OA2060AA	131	653
ESC_OA2058AA	131	653
ESC_OA2055AA	131	653
ESC_OA2054AA	131	653
ESC_OA2053AA	131	653
ESC_OA2050AA	131	653
ESC_OA2048AA	131	653
ESC_OA2047AA	131	653
ESC_OA2046AA	131	653
ESC_OA2045AA	131	653
ESC_OA2044AA	131	653
ESC_OA2043AA	131	653
ESC_OA2042AA	131	653
ESC_OA2040AA	11	936
ESC_OA2039AA	11	936
ESC_OA2038AA	11	936
ESC_OA2037AA	11	936
ESC_OA2036AA	11	936
ESC_OA2035AA	11	936
ESC_OA2034AA	11	936
ESC_OA2032AA	11	936
ESC_OA2031AA	11	936
ESC_OA2030AA	11	936
ESC_OA2029AA	11	936
ESC_OA2028AA	11	936
ESC_OA2026AA	21	156
ESC_OA2025AA	11	936
ESC_OA2024AA	10158	257
ESC_OA2023AA	58	198
ESC_OA2022AA	3580	257
ESC_OA2021AA	58	198
ESC_OA2020AA	744	512
ESC_OA2019AA	1001	257
ESC_OA2018AA	5853	868
ESC_OA2017AA	2606	198
ESC_OA2016AA	189	504
ESC_OA2015AA	155	175
ESC_OA2014AA	216	512
ESC_OA2013AA	58	198
ESC_OA2012AA	135	653
ESC_OA2011AA	58	198
ESC_OA2010AA	154	257
ESC_OA2009AA	154	257
ESC_OA2008AA	398	445
ESC_OA2006AA	1485	408
ESC_OA2005AA	10	512
ESC_OA2002AA	23	156
ESC_OA2001AA	21	156
ESC_OA2000AA	101	696
ESC_OA1999AA	21	156

ESC_OA1998AA	101	696
ESC_OA1994AA	11	936
ESC_OA1993AA	162	924
ESC_OA1992AA	1071	175
ESC_OA1991AA	95	738
ESC_OA1990AA	101	696
ESC_OA1989AA	539	257
ESC_OA1988AA	48	76
ESC_OA1987AA	453	935
ESC_OA1986AA	10	512
ESC_OA1953AA	38	669
ESC_OA1952AA	2064	261
ESC_OA1951AA	10155	118
ESC_OA1950AA	131	653
ESC_OA1949AA	131	653
ESC_OA1948AA	167	512
ESC_OA1947AA	69	387
ESC_OA1946AA	38	669
ESC_OA1945AA	117	592
ESC_OA1944AA	1147	198
ESC_OA1943AA	795	924
ESC_OA1942AA	394	400
ESC_OA1941AA	10154	156
ESC_OA1940AA	453	935
ESC_OA1939AA	38	669
ESC_OA1938AA	1193	761
ESC_OA1937AA	648	359
ESC_OA1935AA	58	198
ESC_OA1934AA	44	512
ESC_OA1933AA	10	512
ESC_OA1932AA	44	512
ESC_OA1931AA	88	160
ESC_OA1930AA	88	160
ESC_OA1929AA	226	512
ESC_OA1928AA	44	512
ESC_OA1927AA	9440	508
ESC_OA1923AA	69	387
ESC_OA1922AA	131	653
ESC_OA1921AA	38	669
ESC_OA1920AA	131	653
ESC_OA1919AA	4550	150
ESC_OA1917AA	131	653
ESC_OA1916AA	38	669
ESC_OA1915AA	349	670
ESC_OA1914AA	10153	445
ESC_OA1913AA	155	175
ESC_OA1912AA	10	512
ESC_OA1911AA	1485	408
ESC_OA1910AA	2614	975
ESC_OA1909AA	1193	761

ESC_OA1908AA	117	592
ESC_OA1907AA	10149	198
ESC_OA1906AA	131	653
ESC_OA1905AA	38	669
ESC_OA1904AA	131	653
ESC_OA1903AA	315	669
ESC_OA1902AA	1284	604
ESC_OA1901AA	131	653
ESC_OA1900AA	131	653
ESC_OA1899AA	617	511
ESC_OA1898AA	131	653
ESC_OA1897AA	131	653
ESC_OA1896AA	3580	257
ESC_OA1895AA	10148	355
ESC_OA1894AA	131	653
ESC_OA1893AA	131	653
ESC_OA1892AA	131	653
ESC_OA1891AA	963	669
ESC_OA1890AA	131	653
ESC_OA1889AA	131	653
ESC_OA1888AA	963	669
ESC_OA1887AA	127	738
ESC_OA1886AA	131	653
ESC_OA1885AA	963	669
ESC_OA1884AA	773	71
ESC_OA1883AA	773	71
ESC_OA1882AA	38	669
ESC_OA1881AA	131	653
ESC_OA1880AA	131	653
ESC_OA1879AA	963	669
ESC_OA1878AA	410	156
ESC_OA1877AA	44	512
ESC_OA1876AA	38	669
ESC_OA1875AA	1193	761
ESC_OA1874AA	2659	679
ESC_OA1873AA	131	653
ESC_OA1872AA	131	653
ESC_OA1871AA	131	653
ESC_OA1870AA	963	669
ESC_OA1869AA	131	653
ESC_OA1868AA	131	653
ESC_OA1867AA	963	669
ESC_OA1866AA	69	387
ESC_OA1865AA	998	644
ESC_OA1864AA	410	156
ESC_OA1863AA	450	71
ESC_OA1862AA	73	737
ESC_OA1861AA	131	653
ESC_OA1860AA	410	156
ESC_OA1859AA	648	359

ESC_OA1858AA	131	653
ESC_OA1857AA	131	653
ESC_OA1856AA	10	512
ESC_OA1855AA	38	669
ESC_OA1854AA	38	669
ESC_OA1853AA	393	343
ESC_OA1852AA	131	653
ESC_OA1851AA	131	653
ESC_OA1850AA	1193	761
ESC_OA1849AA	38	669
ESC_OA1848AA	131	653
ESC_OA1847AA	2852	257
ESC_OA1846AA	131	653
ESC_OA1845AA	38	669
ESC_OA1844AA	315	669
ESC_OA1843AA	131	653
ESC_OA1842AA	131	653
ESC_OA1841AA	10	512
ESC_OA1840AA	69	387
ESC_OA1839AA	940	257
ESC_OA1838AA	410	156
ESC_OA1837AA	131	653
ESC_OA1836AA	10	512
ESC_OA1835AA	38	669
ESC_OA1834AA	457	595
ESC_OA1833AA	131	653
ESC_OA1832AA	405	923
ESC_OA1831AA	131	653
ESC_OA1829AA	69	387
ESC_OA1828AA	83	740
ESC_OA1827AA	167	512
ESC_OA1826AA	8189	653
ESC_OA1825AA	69	387
ESC_OA1824AA	10	512
ESC_OA1823AA	180	234
ESC_OA1822AA	10	512
ESC_OA1821AA	1177	669
ESC_OA1820AA	131	653
ESC_OA1819AA	131	653
ESC_OA1818AA	131	653
ESC_OA1817AA	648	359
ESC_OA1816AA	3877	512
ESC_OA1815AA	405	923
ESC_OA1814AA	93	71
ESC_OA1813AA	38	669
ESC_OA1812AA	69	387
ESC_OA1811AA	131	653
ESC_OA1810AA	701	174
ESC_OA1809AA	131	653
ESC_OA1808AA	69	387

ESC_OA1807AA	648	359
ESC_OA1806AA	155	175
ESC_OA1805AA	131	653
ESC_OA1804AA	88	160
ESC_OA1803AA	227	504
ESC_OA1802AA	23	156
ESC_OA1801AA	410	156
ESC_OA1800AA	141	644
ESC_OA1799AA	1193	761
ESC_OA1798AA	131	653
ESC_OA1797AA	443	257
ESC_OA1796AA	38	669
ESC_OA1795AA	10	512
ESC_OA1794AA	405	923
ESC_OA1793AA	3873	175
ESC_OA1792AA	131	653
ESC_OA1791AA	1193	761
ESC_OA1790AA	38	669
ESC_OA1789AA	450	71
ESC_OA1788AA	10	512
ESC_OA1787AA	117	592
ESC_OA1786AA	38	669
ESC_OA1785AA	162	924
ESC_OA1784AA	131	653
ESC_OA1783AA	131	653
ESC_OA1782AA	3268	669
ESC_OA1781AA	131	653
ESC_OA1780AA	131	653
ESC_OA1779AA	354	15
ESC_OA1778AA	405	923
ESC_OA1777AA	10	512
ESC_OA1776AA	155	175
ESC_OA1775AA	648	359
ESC_OA1774AA	38	669
ESC_OA1773AA	131	653
ESC_OA1772AA	73	737
ESC_OA1771AA	38	669
ESC_OA1770AA	131	653
ESC_OA1769AA	131	653
ESC_OA1768AA	131	653
ESC_OA1767AA	131	653
ESC_OA1766AA	131	653
ESC_OA1765AA	131	653
ESC_OA1764AA	131	653
ESC_OA1763AA	131	653
ESC_OA1762AA	963	669
ESC_OA1761AA	73	737
ESC_OA1760AA	131	653
ESC_OA1759AA	372	424
ESC_OA1758AA	127	738

ESC_OA1757AA	372	424
ESC_OA1756AA	131	653
ESC_OA1755AA	131	653
ESC_OA1754AA	131	653
ESC_OA1753AA	131	653
ESC_OA1752AA	131	653
ESC_OA1751AA	1193	761
ESC_OA1750AA	1727	257
ESC_OA1749AA	410	156
ESC_OA1748AA	12	626
ESC_OA1747AA	773	71
ESC_OA1746AA	10	512
ESC_OA1745AA	48	76
ESC_OA1744AA	131	653
ESC_OA1743AA	963	669
ESC_OA1742AA	1727	257
ESC_OA1741AA	405	923
ESC_OA1740AA	349	670
ESC_OA1739AA	69	387
ESC_OA1738AA	393	343
ESC_OA1737AA	10	512
ESC_OA1736AA	131	653
ESC_OA1735AA	1955	900
ESC_OA1734AA	410	156
ESC_OA1733AA	131	653
ESC_OA1732AA	1193	761
ESC_OA1731AA	3177	362
ESC_OA1730AA	131	653
ESC_OA1729AA	131	653
ESC_OA1728AA	963	669
ESC_OA1727AA	131	653
ESC_OA1726AA	46	447
ESC_OA1725AA	127	738
ESC_OA1724AA	73	737
ESC_OA1723AA	131	653
ESC_OA1722AA	131	653
ESC_OA1721AA	69	387
ESC_OA1720AA	963	669
ESC_OA1719AA	1722	599
ESC_OA1718AA	69	387
ESC_OA1717AA	73	737
ESC_OA1716AA	131	653
ESC_OA1715AA	131	653
ESC_OA1714AA	131	653
ESC_OA1713AA	131	653
ESC_OA1712AA	1722	599
ESC_OA1711AA	963	669
ESC_OA1710AA	10152	354
ESC_OA1709AA	38	669
ESC_OA1708AA	405	923

ESC_OA1707AA	131	653
ESC_OA1706AA	131	653
ESC_OA1705AA	73	737
ESC_OA1704AA	744	512
ESC_OA1703AA	2346	435
ESC_OA1702AA	963	669
ESC_OA1701AA	38	669
ESC_OA1700AA	1642	198
ESC_OA1699AA	1193	761
ESC_OA1698AA	95	738
ESC_OA1697AA	648	359
ESC_OA1696AA	1193	761
ESC_OA1695AA	88	160
ESC_OA1694AA	10151	198
ESC_OA1693AA	131	653
ESC_OA1692AA	648	359
ESC_OA1691AA	716	795
ESC_OA1690AA	131	653
ESC_OA1689AA	648	359
ESC_OA1688AA	963	669
ESC_OA1687AA	131	653
ESC_OA1686AA	12	626
ESC_OA1685AA	410	156
ESC_OA1684AA	131	653
ESC_OA1683AA	648	359
ESC_OA1682AA	46	447
ESC_OA1681AA	405	923
ESC_OA1680AA	752	512
ESC_OA1679AA	648	359
ESC_OA1678AA	88	160
ESC_OA1676AA	152	257
ESC_OA1674AA	245	261
ESC_OA1672AA	152	257
ESC_OA1670AA	245	261
ESC_OA1668AA	152	257
ESC_OA1666AA	245	261
ESC_OA1662AA	245	261
ESC_OA1659AA	11	936
ESC_OA1658AA	11	936
ESC_OA1657AA	109	257
ESC_OA1656AA	17	191
ESC_OA1655AA	11	936
ESC_OA1654AA	4748	234
ESC_OA1653AA	4038	257
ESC_OA1652AA	7077	519
ESC_OA1651AA	10146	738
ESC_OA1650AA	56	198
ESC_OA1649AA	583	768
ESC_OA1648AA	602	257
ESC_OA1647AA	10147	592

ESC_OA1646AA	10	512
ESC_OA1645AA	10	512
ESC_OA1644AA	641	935
ESC_OA1643AA	131	653
ESC_OA1642AA	405	923
ESC_OA1641AA	405	923
ESC_OA1640AA	6332	153
ESC_OA1638AA	6332	153
ESC_OA1637AA	6332	153
ESC_OA1636AA	6332	153
ESC_OA1635AA	6332	153
ESC_OA1634AA	6332	153
ESC_OA1633AA	744	512
ESC_OA1632AA	744	512
ESC_OA1631AA	744	512
ESC_OA1630AA	744	512
ESC_OA1629AA	744	512
ESC_OA1628AA	744	512
ESC_OA1627AA	744	512
ESC_OA1626AA	744	512
ESC_OA1625AA	744	512
ESC_OA1624AA	131	653
ESC_OA1623AA	38	669
ESC_OA1622AA	1722	599
ESC_OA1621AA	10144	653
ESC_OA1620AA	38	669
ESC_OA1619AA	10145	669
ESC_OA1618AA	648	359
ESC_OA1617AA	38	669
ESC_OA1616AA	152	257
ESC_OA1615AA	156	175
ESC_OA1614AA	11	936
ESC_OA1613AA	11	936
ESC_OA1612AA	48	76
ESC_OA1611AA	648	359
ESC_OA1610AA	38	669
ESC_OA1609AA	156	175
ESC_OA1608AA	10	512
ESC_OA1607AA	350	50
ESC_OA1606AA	1193	761
ESC_OA1605AA	152	257
ESC_OA1604AA	152	257
ESC_OA1603AA	245	261
ESC_OA1602AA	245	261
ESC_OA1601AA	152	257
ESC_OA1600AA	152	257
ESC_OA1599AA	10143	257
ESC_OA1598AA	152	257
ESC_OA1597AA	152	257
ESC_OA1596AA	245	261

ESC_OA1595AA	245	261
ESC_OA1594AA	152	257
ESC_OA1593AA	245	261
ESC_OA1592AA	32	270
ESC_OA1591AA	504	738
ESC_OA1590AA	16	156
ESC_OA1589AA	21	156
ESC_OA1588AA	21	156
ESC_OA1587AA	21	156
ESC_OA1586AA	442	257
ESC_OA1585AA	342	286
ESC_OA1584AA	34	504
ESC_OA1583AA	738	598
ESC_OA1582AA	17	191
ESC_OA0981AA	38	669
ESC_OA0979AA	414	387
ESC_OA0978AA	3052	669
ESC_OA0977AA	226	512
ESC_OA0976AA	38	669
ESC_OA0975AA	38	669
ESC_OA0974AA	101	696
ESC_OA0973AA	533	198
ESC_OA0972AA	1312	76
ESC_OA0971AA	226	512
ESC_OA0970AA	10	512
ESC_OA0969AA	2521	257
ESC_OA0968AA	617	511
ESC_OA0967AA	1638	519
ESC_OA0966AA	162	924
ESC_OA0964AA	10	512
ESC_OA0962AA	59	217
ESC_OA0961AA	10	512
ESC_OA0960AA	59	217
ESC_OA0959AA	59	217
ESC_OA0958AA	10137	217
ESC_OA0957AA	59	217
ESC_OA0956AA	59	217
ESC_OA0955AA	59	217
ESC_OA0954AA	362	234
ESC_OA0953AA	59	217
ESC_OA0952AA	59	217
ESC_OA0951AA	59	217
ESC_OA0950AA	10	512
ESC_OA0949AA	10	512
ESC_OA0948AA	362	234
ESC_OA0946AA	10	512
ESC_OA0944AA	10	512
ESC_OA0943AA	59	217
ESC_OA0942AA	59	217
ESC_OA0940AA	59	217

ESC_OA0939AA	362	234
ESC_OA0938AA	59	217
ESC_OA0937AA	59	217
ESC_OA0936AA	59	217
ESC_OA0934AA	59	217
ESC_OA0933AA	6113	257
ESC_OA0932AA	59	217
ESC_OA0931AA	6113	257
ESC_OA0930AA	6113	257
ESC_OA0929AA	6113	257
ESC_OA0928AA	6113	257
ESC_OA0927AA	6113	257
ESC_OA0926AA	6113	257
ESC_OA0925AA	69	387
ESC_OA0924AA	10	512
ESC_OA0923AA	6113	257
ESC_OA0922AA	59	217
ESC_OA0920AA	10	512
ESC_OA0919AA	6113	257
ESC_OA0918AA	6113	257
ESC_OA0917AA	6113	257
ESC_OA0916AA	6113	257
ESC_OA0915AA	6113	257
ESC_OA0914AA	59	217
ESC_OA0913AA	59	217
ESC_OA0912AA	59	217
ESC_OA0911AA	59	217
ESC_OA0910AA	59	217
ESC_OA0909AA	59	217
ESC_OA0908AA	59	217
ESC_OA0907AA	59	217
ESC_OA0906AA	59	217
ESC_OA0905AA	59	217
ESC_OA0904AA	59	217
ESC_OA0903AA	362	234
ESC_OA0902AA	59	217
ESC_OA0901AA	394	400
ESC_OA0900AA	394	400
ESC_OA0899AA	394	400
ESC_OA0898AA	394	400
ESC_OA0897AA	394	400
ESC_OA0896AA	394	400
ESC_OA0895AA	394	400
ESC_OA0894AA	394	400
ESC_OA0893AA	59	217
ESC_OA0892AA	59	217
ESC_OA0890AA	69	387
ESC_OA0889AA	10	512
ESC_OA0888AA	62	574
ESC_OA0887AA	62	574

ESC_OA0886AA	62	574
ESC_OA0885AA	62	574
ESC_OA0884AA	62	574
ESC_OA0883AA	62	574
ESC_OA0882AA	62	574
ESC_OA0881AA	62	574
ESC_OA0880AA	62	574
ESC_OA0879AA	62	574
ESC_OA0875AA	59	217
ESC_OA0874AA	362	234
ESC_OA0872AA	362	234
ESC_OA0870AA	131	653
ESC_OA0869AA	362	234
ESC_OA0867AA	362	234
ESC_OA0864AA	69	387
ESC_OA0863AA	10	512
ESC_OA0862AA	216	512
ESC_OA0861AA	62	574
ESC_OA0860AA	10	512
ESC_OA0859AA	10	512
ESC_OA0858AA	10	512
ESC_OA0857AA	10	512
ESC_OA0856AA	10	512
ESC_OA0855AA	10	512
ESC_OA0854AA	10	512
ESC_OA0853AA	10	512
ESC_OA0852AA	69	387
ESC_OA0850AA	10	512
ESC_OA0849AA	10	512
ESC_OA0848AA	10	512
ESC_OA0847AA	10	512
ESC_OA0846AA	10	512
ESC_OA0845AA	58	198
ESC_OA0844AA	10	512
ESC_OA0843AA	10	512
ESC_OA0842AA	10	512
ESC_OA0841AA	10	512
ESC_OA0840AA	10	512
ESC_OA0839AA	10	512
ESC_OA0838AA	10	512
ESC_OA0837AA	10	512
ESC_OA0836AA	6113	257
ESC_OA0835AA	131	653
ESC_OA0834AA	59	217
ESC_OA0833AA	59	217
ESC_OA0832AA	59	217
ESC_OA0831AA	59	217
ESC_OA0830AA	59	217
ESC_OA0829AA	59	217
ESC_OA0828AA	59	217

ESC_OA0827AA	59	217
ESC_OA0826AA	59	217
ESC_OA0825AA	59	217
ESC_OA0824AA	12	626
ESC_OA0823AA	394	400
ESC_OA0821AA	394	400
ESC_OA0820AA	59	217
ESC_OA0819AA	59	217
ESC_OA0818AA	59	217
ESC_OA0817AA	59	217
ESC_OA0815AA	59	217
ESC_OA0814AA	59	217
ESC_OA0813AA	362	234
ESC_OA0812AA	12	626
ESC_OA0811AA	59	217
ESC_OA0810AA	394	400
ESC_OA0809AA	6113	257
ESC_OA0808AA	59	217
ESC_OA0807AA	394	400
ESC_OA0806AA	394	400
ESC_OA0804AA	10	512
ESC_OA0803AA	10	512
ESC_OA0802AA	394	400
ESC_OA0801AA	59	217
ESC_OA0800AA	59	217
ESC_OA0796AA	59	217
ESC_OA0794AA	59	217
ESC_OA0793AA	362	234
ESC_OA0792AA	362	234
ESC_OA0791AA	362	234
ESC_OA0789AA	6113	257
ESC_OA0787AA	59	217
ESC_OA0786AA	362	234
ESC_OA0785AA	362	234
ESC_OA0784AA	362	234
ESC_OA0783AA	59	217
ESC_OA0782AA	10	512
ESC_OA0781AA	59	217
ESC_OA0780AA	59	217
ESC_OA0779AA	69	387
ESC_OA0778AA	59	217
ESC_OA0777AA	10	512
ESC_OA0776AA	394	400
ESC_OA0775AA	394	400
ESC_OA0774AA	394	400
ESC_OA0773AA	394	400
ESC_OA0772AA	394	400
ESC_OA0771AA	394	400
ESC_OA0770AA	394	400
ESC_OA0769AA	394	400

ESC_OA0768AA	394	400
ESC_OA0767AA	394	400
ESC_OA0766AA	394	400
ESC_OA0765AA	394	400
ESC_OA0764AA	394	400
ESC_OA0763AA	394	400
ESC_OA0762AA	394	400
ESC_OA0761AA	362	234
ESC_OA0760AA	362	234
ESC_OA0759AA	362	234
ESC_OA0758AA	69	387
ESC_OA0757AA	69	387
ESC_OA0756AA	69	387
ESC_OA0755AA	59	217
ESC_OA0754AA	69	387
ESC_OA0753AA	10	512
ESC_OA0752AA	59	217
ESC_OA0751AA	362	234
ESC_OA0750AA	362	234
ESC_OA0749AA	362	234
ESC_OA0748AA	362	234
ESC_OA0747AA	362	234
ESC_OA0746AA	131	653
ESC_OA0745AA	131	653
ESC_OA0742AA	131	653
ESC_OA0741AA	394	400
ESC_OA0740AA	131	653
ESC_OA0739AA	131	653
ESC_OA0738AA	131	653
ESC_OA0737AA	131	653
ESC_OA0736AA	10	512
ESC_OA0735AA	59	217
ESC_OA0734AA	10	512
ESC_OA0733AA	10	512
ESC_OA0729AA	62	574
ESC_OA0728AA	62	574
ESC_OA0727AA	62	574
ESC_OA0726AA	62	574
ESC_OA0725AA	62	574
ESC_OA0724AA	59	217
ESC_OA0723AA	216	512
ESC_OA0722AA	62	574
ESC_OA0721AA	62	574
ESC_OA0720AA	62	574
ESC_OA0719AA	69	387
ESC_OA0717AA	59	217
ESC_OA0716AA	69	387
ESC_OA0715AA	69	387
ESC_OA0714AA	69	387
ESC_OA0713AA	69	387

ESC_OA0712AA	69	387
ESC_OA0710AA	69	387
ESC_OA0708AA	69	387
ESC_OA0707AA	69	387
ESC_OA0706AA	59	217
ESC_OA0705AA	59	217
ESC_OA0704AA	59	217
ESC_OA0701AA	59	217
ESC_OA0700AA	59	217
ESC_OA0697AA	10	512
ESC_OA0696AA	59	217
ESC_OA0695AA	59	217
ESC_OA0694AA	10	512
ESC_OA0693AA	10	512
ESC_OA0692AA	59	217
ESC_OA0691AA	10	512
ESC_OA0690AA	394	400
ESC_OA0689AA	6113	257
ESC_OA0688AA	6113	257
ESC_OA0687AA	6113	257
ESC_OA0686AA	6113	257
ESC_OA0685AA	6113	257
ESC_OA0684AA	6113	257
ESC_OA0683AA	6113	257
ESC_OA0682AA	6113	257
ESC_OA0681AA	59	217
ESC_OA0680AA	59	217
ESC_OA0679AA	59	217
ESC_OA0678AA	59	217
ESC_OA0677AA	6113	257
ESC_OA0676AA	394	400
ESC_OA0675AA	59	217
ESC_OA0674AA	59	217
ESC_OA0673AA	10138	386
ESC_OA0671AA	59	217
ESC_OA0670AA	59	217
ESC_OA0669AA	394	400
ESC_OA0668AA	394	400
ESC_OA0667AA	394	400
ESC_OA0666AA	59	217
ESC_OA0665AA	59	217
ESC_OA0664AA	59	217
ESC_OA0663AA	59	217
ESC_OA0662AA	59	217
ESC_OA0661AA	59	217
ESC_OA0660AA	59	217
ESC_OA0658AA	10	512
ESC_OA0657AA	69	387
ESC_OA0655AA	59	217
ESC_OA0652AA	59	217

ESC_OA0651AA	59	217
ESC_OA0650AA	59	217
ESC_OA0649AA	59	217
ESC_OA0648AA	59	217
ESC_OA0647AA	394	400
ESC_OA0646AA	394	400
ESC_OA0645AA	59	217
ESC_OA0644AA	59	217
ESC_OA0642AA	59	217
ESC_OA0640AA	59	217
ESC_OA0639AA	59	217
ESC_OA0638AA	59	217
ESC_OA0637AA	59	217
ESC_OA0636AA	59	217
ESC_OA0635AA	59	217
ESC_OA0634AA	59	217
ESC_OA0633AA	362	234
ESC_OA0632AA	59	217
ESC_OA0631AA	59	217
ESC_OA0630AA	59	217
ESC_OA0629AA	59	217
ESC_OA0628AA	69	387
ESC_OA0627AA	69	387
ESC_OA0626AA	69	387
ESC_OA0625AA	69	387
ESC_OA0624AA	69	387
ESC_OA0623AA	69	387
ESC_OA0622AA	69	387
ESC_OA0621AA	69	387
ESC_OA0620AA	69	387
ESC_OA0619AA	69	387
ESC_OA0618AA	59	217
ESC_OA0617AA	59	217
ESC_OA0615AA	10	512
ESC_OA0614AA	69	387
ESC_OA0613AA	69	387
ESC_OA0612AA	69	387
ESC_OA0611AA	69	387
ESC_OA0610AA	69	387
ESC_OA0609AA	69	387
ESC_OA0608AA	69	387
ESC_OA0607AA	69	387
ESC_OA0606AA	69	387
ESC_OA0605AA	69	387
ESC_OA0603AA	10	512
ESC_OA0602AA	131	653
ESC_OA0601AA	10	512
ESC_OA0600AA	394	400
ESC_OA0599AA	394	400
ESC_OA0597AA	59	217

ESC_OA0596AA	59	217
ESC_OA0595AA	59	217
ESC_OA0591AA	62	574
ESC_OA0590AA	62	574
ESC_OA0589AA	216	512
ESC_OA0587AA	73	737
ESC_OA0586AA	69	387
ESC_OA0583AA	10	512
ESC_OA0582AA	394	400
ESC_OA0581AA	10	512
ESC_OA0580AA	58	198
ESC_OA0579AA	58	198
ESC_OA0578AA	58	198
ESC_OA0576AA	58	198
ESC_OA0575AA	1585	512
ESC_OA0574AA	58	198
ESC_OA0573AA	58	198
ESC_OA0572AA	58	198
ESC_OA0571AA	58	198
ESC_OA0570AA	59	217
ESC_OA0568AA	6113	257
ESC_OA0567AA	6113	257
ESC_OA0566AA	6113	257
ESC_OA0565AA	6113	257
ESC_OA0564AA	6113	257
ESC_OA0562AA	6113	257
ESC_OA0561AA	10	512
ESC_OA0560AA	10	512
ESC_OA0559AA	10	512
ESC_OA0558AA	10	512
ESC_OA0557AA	10	512
ESC_OA0556AA	59	217
ESC_OA0555AA	10	512
ESC_OA0554AA	10	512
ESC_OA0553AA	10	512
ESC_OA0552AA	10	512
ESC_OA0551AA	69	387
ESC_OA0550AA	362	234
ESC_OA0549AA	69	387
ESC_OA0548AA	69	387
ESC_OA0547AA	69	387
ESC_OA0544AA	59	217
ESC_OA0543AA	59	217
ESC_OA0542AA	59	217
ESC_OA0541AA	59	217
ESC_OA0540AA	59	217
ESC_OA0539AA	59	217
ESC_OA0538AA	59	217
ESC_OA0536AA	69	387
ESC_OA0535AA	59	217

ESC_OA0534AA	394	400
ESC_OA0532AA	394	400
ESC_OA0531AA	394	400
ESC_OA0530AA	394	400
ESC_OA0529AA	394	400
ESC_OA0528AA	394	400
ESC_OA0527AA	394	400
ESC_OA0526AA	394	400
ESC_OA0525AA	394	400
ESC_OA0522AA	394	400
ESC_OA0521AA	394	400
ESC_OA0520AA	394	400
ESC_OA0517AA	59	217
ESC_OA0516AA	362	234
ESC_OA0515AA	59	217
ESC_OA0514AA	362	234
ESC_OA0513AA	362	234
ESC_OA0512AA	362	234
ESC_OA0511AA	362	234
ESC_OA0510AA	59	217
ESC_OA0509AA	362	234
ESC_OA0507AA	69	387
ESC_OA0506AA	69	387
ESC_OA0505AA	69	387
ESC_OA0502AA	69	387
ESC_OA0501AA	69	387
ESC_OA0500AA	69	387
ESC_OA0498AA	11	936
ESC_OA0497AA	131	653
ESC_OA0496AA	131	653
ESC_OA0494AA	38	669
ESC_OA0493AA	131	653
ESC_OA0492AA	167	512
ESC_OA0491AA	648	359
ESC_OA0490AA	131	653
ESC_OA0489AA	131	653
ESC_OA0488AA	131	653
ESC_OA0487AA	10	512
ESC_OA0486AA	906	198
ESC_OA0485AA	23	156
ESC_OA0484AA	11	936
ESC_OA0483AA	11	936
ESC_OA0482AA	11	936
ESC_OA0481AA	11	936
ESC_OA0480AA	11	936
ESC_OA0479AA	11	936
ESC_OA0478AA	11	936
ESC_OA0477AA	11	936
ESC_OA0476AA	11	936
ESC_OA0475AA	11	936

ESC_OA0474AA	10	512
ESC_OA0473AA	131	653
ESC_OA0472AA	10	512
ESC_OA0471AA	410	156
ESC_OA0470AA	131	653
ESC_OA0469AA	38	669
ESC_OA0468AA	131	653
ESC_OA0467AA	131	653
ESC_OA0466AA	10136	653
ESC_OA0465AA	131	653
ESC_OA0464AA	405	923
ESC_OA0463AA	131	653
ESC_OA0462AA	131	653
ESC_OA0461AA	131	653
ESC_OA0460AA	131	653
ESC_OA0459AA	131	653
ESC_OA0458AA	131	653
ESC_OA0457AA	10	512
ESC_OA0456AA	131	653
ESC_OA0455AA	131	653
ESC_OA0454AA	131	653
ESC_OA0453AA	131	653
ESC_OA0446AA	131	653
ESC_OA0445AA	131	653
ESC_OA0444AA	131	653
ESC_OA0443AA	131	653
ESC_OA0442AA	131	653
ESC_OA0441AA	131	653
ESC_OA0440AA	131	653
ESC_OA0439AA	131	653
ESC_OA0438AA	131	653
ESC_OA0437AA	131	653
ESC_OA0435AA	131	653
ESC_OA0434AA	131	653
ESC_OA0433AA	131	653
ESC_OA0432AA	131	653
ESC_OA0431AA	131	653
ESC_OA0430AA	131	653
ESC_OA0429AA	131	653
ESC_OA0428AA	131	653
ESC_OA0427AA	131	653
ESC_OA0426AA	131	653
ESC_OA0425AA	131	653
ESC_OA0424AA	131	653
ESC_OA0423AA	131	653
ESC_OA0422AA	131	653
ESC_OA0421AA	131	653
ESC_OA0420AA	131	653
ESC_OA0419AA	131	653
ESC_OA0418AA	131	653

ESC_OA0417AA	131	653
ESC_OA0416AA	131	653
ESC_OA0415AA	131	653
ESC_OA0414AA	131	653
ESC_OA0413AA	131	653
ESC_OA0412AA	131	653
ESC_OA0411AA	131	653
ESC_OA0410AA	131	653
ESC_OA0409AA	131	653
ESC_OA0408AA	131	653
ESC_OA0407AA	17	191
ESC_OA0405AA	21	156
ESC_OA0404AA	21	156
ESC_OA0403AA	21	156
ESC_OA0402AA	21	156
ESC_OA0401AA	10132	156
ESC_OA0400AA	11	936
ESC_OA0399AA	21	156
ESC_OA0398AA	10131	286
ESC_OA0397AA	683	175
ESC_OA0396AA	10	512
ESC_OA0395AA	223	198
ESC_OA0394AA	223	198
ESC_OA0393AA	223	198
ESC_OA0392AA	162	924
ESC_OA0391AA	8574	924
ESC_OA0390AA	8574	924
ESC_OA0389AA	8574	924
ESC_OA0388AA	58	198
ESC_OA0387AA	58	198
ESC_OA0386AA	316	257
ESC_OA0385AA	58	198
ESC_OA0384AA	316	257
ESC_OA0383AA	5737	180
ESC_OA0382AA	7	842
ESC_OA0381AA	5737	180
ESC_OA0380AA	58	198
ESC_OA0379AA	8574	924
ESC_OA0378AA	8574	924
ESC_OA0377AA	9216	175
ESC_OA0376AA	6188	257
ESC_OA0375AA	295	160
ESC_OA0374AA	9214	438
ESC_OA0373AA	223	198
ESC_OA0372AA	10	512
ESC_OA0371AA	10	512
ESC_OA0370AA	939	198
ESC_OA0369AA	58	198
ESC_OA0368AA	58	198
ESC_OA0367AA	58	198

ESC_OA0366AA	58	198
ESC_OA0365AA	58	198
ESC_OA0364AA	6197	235
ESC_OA0363AA	9215	936
ESC_OA0362AA	405	923
ESC_OA0361AA	131	653
ESC_OA0360AA	131	653
ESC_OA0359AA	410	156
ESC_OA0358AA	131	653
ESC_OA0357AA	131	653
ESC_OA0356AA	648	359
ESC_OA0355AA	131	653
ESC_OA0354AA	131	653
ESC_OA0353AA	131	653
ESC_OA0350AA	11	936
ESC_OA0349AA	648	359
ESC_OA0348AA	131	653
ESC_OA0347AA	131	653
ESC_OA0346AA	131	653
ESC_OA0345AA	131	653
ESC_OA0344AA	405	923
ESC_OA0343AA	131	653
ESC_OA0342AA	648	359
ESC_OA0341AA	405	923
ESC_OA0340AA	167	512
ESC_OA0339AA	216	512
ESC_OA0338AA	278	924
ESC_OA0336AA	218	519
ESC_OA0334AA	543	842
ESC_OA0332AA	405	923
ESC_OA0331AA	131	653
ESC_OA0330AA	69	387
ESC_OA0329AA	117	592
ESC_OA0328AA	617	511
ESC_OA0327AA	131	653
ESC_OA0326AA	131	653
ESC_OA0325AA	131	653
ESC_OA0324AA	131	653
ESC_OA0323AA	648	359
ESC_OA0322AA	131	653
ESC_OA0321AA	131	653
ESC_OA0320AA	405	923
ESC_OA0319AA	2003	669
ESC_OA0318AA	38	669
ESC_OA0317AA	7584	156
ESC_OA0316AA	12	626
ESC_OA0315AA	648	359
ESC_OA0314AA	156	175
ESC_OA0313AA	42	257
ESC_OA0312AA	42	257

ESC_OA0311AA	156	175
ESC_OA0310AA	448	257
ESC_OA0309AA	93	71
ESC_OA0308AA	1011	198
ESC_OA0306AA	156	175
ESC_OA0305AA	10	512
ESC_OA0304AA	118	985
ESC_OA0303AA	100	504
ESC_OA0302AA	224	198
ESC_OA0301AA	542	824
ESC_OA0300AA	763	878
ESC_OA0299AA	10	512
ESC_OA0298AA	3205	785
ESC_OA0297AA	10	512
ESC_OA0296AA	351	268
ESC_OA0295AA	10	512
ESC_OA0294AA	297	234
ESC_OA0292AA	155	175
ESC_OA0291AA	58	198
ESC_OA0290AA	155	175
ESC_OA0289AA	127	738
ESC_OA0288AA	428	654
ESC_OA0287AA	155	175
ESC_OA0286AA	4663	257
ESC_OA0285AA	10	512
ESC_OA0283AA	9310	198
ESC_OA0282AA	1304	257
ESC_OA0281AA	58	198
ESC_OA0280AA	215	512
ESC_OA0278AA	245	261
ESC_OA0277AA	10	512
ESC_OA0276AA	10	512
ESC_OA0275AA	1079	235
ESC_OA0274AA	11079	198
ESC_OA0273AA	398	445
ESC_OA0272AA	13	257
ESC_OA0271AA	58	198
ESC_OA0270AA	429	654
ESC_OA0269AA	429	654
ESC_OA0268AA	429	654
ESC_OA0267AA	429	654
ESC_OA0266AA	429	654
ESC_OA0265AA	10120	512
ESC_OA0264AA	17	191
ESC_OA0263AA	17	191
ESC_OA0262AA	17	191
ESC_OA0260AA	17	191
ESC_OA0259AA	17	191
ESC_OA0258AA	17	191
ESC_OA0257AA	17	191

ESC_OA0256AA	17	191
ESC_OA0255AA	1011	198
ESC_OA0254AA	1290	515
ESC_OA0253AA	48	76
ESC_OA0252AA	10119	160
ESC_OA0250AA	354	15
ESC_OA0249AA	46	447
ESC_OA0248AA	48	76
ESC_OA0246AA	10115	481
ESC_OA0245AA	167	512
ESC_OA0244AA	167	512
ESC_OA0243AA	205	257
ESC_OA0242AA	4450	844
ESC_OA0241AA	405	923
ESC_OA0240AA	405	923
ESC_OA0239AA	410	156
ESC_OA0238AA	410	156
ESC_OA0236AA	127	738
ESC_OA0235AA	2604	626
ESC_OA0234AA	1735	737
ESC_OA0233AA	607	355
ESC_OA0232AA	73	737
ESC_OA0231AA	10135	257
ESC_OA0230AA	372	424
ESC_OA0229AA	10134	454
ESC_OA0228AA	372	424
ESC_OA0227AA	73	737
ESC_OA0226AA	906	198
ESC_OA0225AA	10	512
ESC_OA0224AA	12	626
ESC_OA0223AA	1262	737
ESC_OA0222AA	372	424
ESC_OA0221AA	372	424
ESC_OA0220AA	189	504
ESC_OA0219AA	457	595
ESC_OA0218AA	746	789
ESC_OA0217AA	73	737
ESC_OA0215AA	1148	257
ESC_OA0214AA	7063	900
ESC_OA0213AA	131	653
ESC_OA0212AA	533	198
ESC_OA0211AA	9290	602
ESC_OA0210AA	127	738
ESC_OA0209AA	994	924
ESC_OA0208AA	297	234
ESC_OA0207AA	994	924
ESC_OA0206AA	131	653
ESC_OA0205AA	117	592
ESC_OA0204AA	167	512
ESC_OA0202AA	1431	257

ESC_OA0201AA	10113	50
ESC_OA0200AA	73	737
ESC_OA0199AA	1193	761
ESC_OA0198AA	12	626
ESC_OA0197AA	165	504
ESC_OA0196AA	245	261
ESC_OA0195AA	245	261
ESC_OA0194AA	245	261
ESC_OA0193AA	152	257
ESC_OA0192AA	245	261
ESC_OA0191AA	1025	261
ESC_OA0190AA	152	257
ESC_OA0189AA	152	257
ESC_OA0188AA	152	257
ESC_OA0187AA	152	257
ESC_OA0186AA	152	257
ESC_OA0185AA	245	261
ESC_OA0184AA	152	257
ESC_OA0183AA	152	257
ESC_OA0182AA	152	257
ESC_OA0181AA	245	261
ESC_OA0180AA	152	257
ESC_OA0179AA	152	257
ESC_OA0178AA	152	257
ESC_OA0177AA	245	261
ESC_OA0176AA	245	261
ESC_OA0175AA	145	256
ESC_OA0174AA	152	257
ESC_OA0173AA	245	261
ESC_OA0172AA	641	935
ESC_OA0171AA	117	592
ESC_OA0170AA	906	198
ESC_OA0169AA	1844	198
ESC_OA0168AA	6274	257
ESC_OA0166AA	21	156
ESC_OA0165AA	33	198
ESC_OA0164AA	21	156
ESC_OA0163AA	504	738
ESC_OA0162AA	442	257
ESC_OA0161AA	442	257
ESC_OA0158AA	38	669
ESC_OA0157AA	21	156
ESC_OA0156AA	11	936
ESC_OA0155AA	10114	257
ESC_OA0154AA	11	936
ESC_OA0153AA	11	936
ESC_OA0152AA	11	936
ESC_OA0151AA	32	270
ESC_OA0150AA	1833	936
ESC_OA0149AA	17	191

ESC_OA0148AA	11	936
ESC_OA0147AA	21	156
ESC_OA0146AA	11	936
ESC_OA0145AA	127	738
ESC_OA0144AA	101	696
ESC_OA0143AA	648	359
ESC_OA0142AA	44	512
ESC_OA0141AA	131	653
ESC_OA0140AA	1193	761
ESC_OA0138AA	11	936
ESC_OA0137AA	11	936
ESC_OA0136AA	11	936
ESC_OA0135AA	11	936
ESC_OA0134AA	11	936
ESC_OA0133AA	11	936
ESC_OA0132AA	11	936
ESC_OA0131AA	11	936
ESC_OA0130AA	11	936
ESC_OA0129AA	11	936
ESC_OA0128AA	11	936
ESC_OA0127AA	11	936
ESC_OA0126AA	11	936
ESC_OA0125AA	131	653
ESC_OA0124AA	127	738
ESC_OA0123AA	10123	738
ESC_OA0122AA	127	738
ESC_OA0121AA	2811	233
ESC_OA0120AA	10122	386
ESC_OA0056AA	393	343
ESC_OA0055AA	155	175
ESC_OA0054AA	10	512
ESC_OA0053AA	405	923
ESC_OA0052AA	167	512
ESC_OA0051AA	648	359
ESC_OA0050AA	167	512
ESC_OA0049AA	1284	604
ESC_OA0048AA	1284	604
ESC_OA0047AA	46	447
ESC_OA0046AA	167	512
ESC_OA0045AA	648	359
ESC_OA0044AA	405	923
ESC_OA0043AA	2011	362
ESC_OA0042AA	405	923
ESC_OA0041AA	940	257
ESC_OA0040AA	2659	679
ESC_OA0039AA	8346	234
ESC_OA0038AA	410	156
ESC_OA0037AA	206	286
ESC_OA0034AA	57	50
ESC_OA0033AA	410	156

ESC_OA0032AA	361	512
ESC_OA0031AA	167	512
ESC_OA0030AA	167	512
ESC_OA0029AA	617	511
ESC_OA0028AA	1193	761
ESC_OA0027AA	2659	679
ESC_OA0026AA	38	669
ESC_OA0025AA	648	359
ESC_OA0024AA	648	359
ESC_OA0023AA	167	512
ESC_OA0022AA	295	160
ESC_OA0021AA	1890	924
ESC_OA0020AA	11	936
ESC_OA0019AA	4213	251
ESC_OA0018AA	224	198
ESC_OA0017AA	940	257
ESC_OA0016AA	205	257
ESC_OA0015AA	46	447
ESC_OA0014AA	410	156
ESC_OA0013AA	167	512
ESC_OA0012AA	167	512
ESC_OA0011AA	167	512
ESC_OA0010AA	167	512
ESC_OA0009AA	167	512
ESC_OA0008AA	1284	604
ESC_OA0007AA	410	156
ESC_OA0006AA	617	511
ESC_OA0005AA	11	936
ESC_OA0004AA	11	936
ESC_OA0003AA	8279	936
ESC_OA0002AA	11	936
ESC_OA0001AA	11	936
ESC_OA0000AA	11	936
ESC_NA9999AA	11	936
ESC_NA9998AA	11	936
ESC_NA9997AA	11	936
ESC_NA9996AA	9008	627
ESC_NA9995AA	11	936
ESC_NA9994AA	8279	936
ESC_NA9993AA	11	936
ESC_NA9992AA	11	936
ESC_NA9991AA	11	936
ESC_NA9990AA	11	936
ESC_NA9987AA	940	257
ESC_NA9986AA	1702	512
ESC_NA9985AA	156	175
ESC_NA9984AA	167	512
ESC_NA9983AA	46	447
ESC_NA9982AA	156	175
ESC_NA9981AA	156	175

ESC_NA9980AA	167	512
ESC_NA9979AA	152	257
ESC_NA9978AA	1585	512
ESC_NA9977AA	405	923
ESC_NA9976AA	410	156
ESC_NA9975AA	617	511
ESC_NA9974AA	2450	447
ESC_NA9973AA	167	512
ESC_NA9972AA	167	512
ESC_NA9971AA	167	512
ESC_NA9970AA	354	15
ESC_NA9969AA	617	511
ESC_NA9968AA	11	936
ESC_NA9967AA	11	936
ESC_NA9966AA	11	936
ESC_NA9965AA	11	936
ESC_NA9964AA	11	936
ESC_NA9963AA	167	512
ESC_NA9962AA	410	156
ESC_NA9960AA	167	512
ESC_NA9959AA	167	512
ESC_NA9958AA	744	512
ESC_NA9957AA	410	156
ESC_NA9956AA	167	512
ESC_NA9955AA	617	511
ESC_NA9954AA	10	512
ESC_NA9953AA	162	924
ESC_NA9952AA	38	669
ESC_NA9951AA	394	400
ESC_NA9950AA	746	789
ESC_NA9949AA	354	15
ESC_NA9948AA	484	71
ESC_NA9947AA	155	175
ESC_NA9946AA	648	359
ESC_NA9945AA	48	76
ESC_NA9944AA	152	257
ESC_NA9943AA	21	156
ESC_NA9942AA	10	512
ESC_NA9941AA	504	738
ESC_NA9940AA	17	191
ESC_NA9939AA	738	598
ESC_NA9938AA	504	738
ESC_NA9937AA	5821	387
ESC_NA9935AA	5292	738
ESC_NA9934AA	33	198
ESC_NA9933AA	17	191
ESC_NA9932AA	25	234
ESC_NA9931AA	504	738
ESC_NA9930AA	342	286
ESC_NA9929AA	25	234

ESC_NA9928AA	504	738
ESC_NA9927AA	16	156
ESC_NA9926AA	11	936
ESC_NA9925AA	17	191
ESC_NA9924AA	17	191
ESC_NA9923AA	738	598
ESC_NA9922AA	11	936
ESC_NA9921AA	21	156
ESC_NA9920AA	583	768
ESC_NA9919AA	5292	738
ESC_NA9918AA	17	191
ESC_NA9917AA	21	156
ESC_NA9916AA	504	738
ESC_NA9915AA	17	191
ESC_NA9914AA	10	512
ESC_NA9913AA	306	286
ESC_NA9912AA	722	422
ESC_NA9911AA	5292	738
ESC_NA9910AA	767	198
ESC_NA9909AA	20	198
ESC_NA9908AA	21	156
ESC_NA9907AA	25	234
ESC_NA9906AA	33	198
ESC_NA9905AA	200	198
ESC_NA9904AA	753	842
ESC_NA9903AA	10	512
ESC_NA9902AA	5887	234
ESC_NA9901AA	21	156
ESC_NA9900AA	101	696
ESC_NA9899AA	583	768
ESC_NA9898AA	504	738
ESC_NA9897AA	25	234
ESC_NA9896AA	33	198
ESC_NA9895AA	69	387
ESC_NA9894AA	10	512
ESC_NA9893AA	504	738
ESC_NA9892AA	738	598
ESC_NA9891AA	491	626
ESC_NA9890AA	753	842
ESC_NA9889AA	2346	435
ESC_NA9888AA	21	156
ESC_NA9887AA	17	191
ESC_NA9886AA	33	198
ESC_NA9885AA	738	598
ESC_NA9884AA	3101	160
ESC_NA9883AA	13	257
ESC_NA9882AA	738	598
ESC_NA9881AA	11	936
ESC_NA9880AA	25	234
ESC_NA9879AA	33	198

ESC_NA9878AA	442	257
ESC_NA9877AA	11	936
ESC_NA9876AA	33	198
ESC_NA9875AA	17	191
ESC_NA9874AA	11	936
ESC_NA9873AA	11	936
ESC_NA9872AA	17	191
ESC_NA9871AA	753	842
ESC_NA9870AA	583	768
ESC_NA9869AA	21	156
ESC_NA9868AA	32	270
ESC_NA9867AA	17	191
ESC_NA9866AA	11	936
ESC_NA9865AA	32	270
ESC_NA9864AA	16	156
ESC_NA9863AA	32	270
ESC_NA9862AA	40	198
ESC_NA9861AA	1892	846
ESC_NA9860AA	11	936
ESC_NA9859AA	200	198
ESC_NA9858AA	765	156
ESC_NA9857AA	442	257
ESC_NA9856AA	57	50
ESC_NA9855AA	11	936
ESC_NA9854AA	504	738
ESC_NA9853AA	753	842
ESC_NA9852AA	33	198
ESC_NA9851AA	753	842
ESC_NA9850AA	11	936
ESC_NA9849AA	33	198
ESC_NA9848AA	17	191
ESC_NA9844AA	10	512
ESC_NA9843AA	10109	670
ESC_NA9842AA	48	76
ESC_NA9841AA	11	936
ESC_NA9840AA	67	257
ESC_NA9839AA	17	191
ESC_NA9838AA	173	254
ESC_NA9837AA	2522	198
ESC_NA9836AA	2522	198
ESC_NA9835AA	11	936
ESC_NA9834AA	11	936
ESC_NA9833AA	10	512
ESC_NA9832AA	11	936
ESC_NA9831AA	441	198
ESC_NA9830AA	3580	257
ESC_NA9829AA	409	504
ESC_NA9828AA	58	198
ESC_NA9827AA	10	512
ESC_NA9826AA	616	156

ESC_NA9825AA	457	595
ESC_NA9824AA	405	923
ESC_NA9823AA	1722	599
ESC_NA9822AA	405	923
ESC_NA9821AA	38	669
ESC_NA9820AA	405	923
ESC_NA9819AA	167	512
ESC_NA9818AA	410	156
ESC_NA9817AA	617	511
ESC_NA9816AA	410	156
ESC_NA9815AA	410	156
ESC_NA9814AA	192	696
ESC_NA9813AA	361	512
ESC_NA9812AA	2851	156
ESC_NA9811AA	167	512
ESC_NA9810AA	410	156
ESC_NA9809AA	167	512
ESC_NA9808AA	405	923
ESC_NA9807AA	152	257
ESC_NA9806AA	152	257
ESC_NA9805AA	4554	738
ESC_NA9804AA	95	738
ESC_NA9803AA	95	738
ESC_NA9802AA	95	738
ESC_NA9800AA	58	198
ESC_NA9799AA	34	504
ESC_NA9798AA	2522	198
ESC_NA9797AA	173	254
ESC_NA9796AA	765	156
ESC_NA9795AA	3494	257
ESC_NA9794AA	10121	257
ESC_NA9793AA	2522	198
ESC_NA9792AA	2522	198
ESC_NA9791AA	58	198
ESC_NA9790AA	173	254
ESC_NA9789AA	155	175
ESC_NA9788AA	17	191
ESC_NA9787AA	2541	878
ESC_NA9786AA	2541	878
ESC_NA9785AA	3602	234
ESC_NA9784AA	2541	878
ESC_NA9783AA	2541	878
ESC_NA9782AA	906	198
ESC_NA9781AA	2713	878
ESC_NA9780AA	641	935
ESC_NA9779AA	1011	198
ESC_NA9778AA	155	175
ESC_NA9777AA	2522	198
ESC_NA9776AA	2522	198
ESC_NA9775AA	2522	198

ESC_NA9774AA	2325	447
ESC_NA9773AA	34	504
ESC_NA9772AA	131	653
ESC_NA9771AA	38	669
ESC_NA9770AA	354	15
ESC_NA9769AA	131	653
ESC_NA9768AA	17	191
ESC_NA9767AA	101	696
ESC_NA9762AA	117	592
ESC_NA9761AA	117	592
ESC_NA9760AA	117	592
ESC_NA9759AA	117	592
ESC_NA9758AA	429	654
ESC_NA9757AA	2309	669
ESC_NA9756AA	2309	669
ESC_NA9755AA	2309	669
ESC_NA9754AA	2309	669
ESC_NA9753AA	2309	669
ESC_NA9752AA	162	924
ESC_NA9751AA	1304	257
ESC_NA9750AA	2309	669
ESC_NA9749AA	162	924
ESC_NA9748AA	2309	669
ESC_NA9747AA	2309	669
ESC_NA9746AA	1304	257
ESC_NA9745AA	2309	669
ESC_NA9744AA	2309	669
ESC_NA9743AA	2309	669
ESC_NA9742AA	2309	669
ESC_NA9741AA	2309	669
ESC_NA9740AA	2309	669
ESC_NA9739AA	2309	669
ESC_NA9738AA	2309	669
ESC_NA9737AA	2309	669
ESC_NA9736AA	162	924
ESC_NA9735AA	2309	669
ESC_NA9734AA	2309	669
ESC_NA9733AA	1304	257
ESC_NA9732AA	117	592
ESC_NA9731AA	117	592
ESC_NA9730AA	10	512
ESC_NA9729AA	117	592
ESC_NA9728AA	117	592
ESC_NA9727AA	117	592
ESC_NA9726AA	117	592
ESC_NA9725AA	117	592
ESC_NA9724AA	117	592
ESC_NA9723AA	117	592
ESC_NA9722AA	117	592
ESC_NA9721AA	117	592

ESC_NA9720AA	117	592
ESC_NA9719AA	117	592
ESC_NA9718AA	117	592
ESC_NA9717AA	117	592
ESC_NA9716AA	117	592
ESC_NA9715AA	117	592
ESC_NA9714AA	117	592
ESC_NA9713AA	10106	737
ESC_NA9710AA	1397	512
ESC_NA9709AA	1602	817
ESC_NA9708AA	23	156
ESC_NA9707AA	10	512
ESC_NA9706AA	152	257
ESC_NA9705AA	152	257
ESC_NA9704AA	131	653
ESC_NA9703AA	2003	669
ESC_NA9702AA	1193	761
ESC_NA9701AA	11	936
ESC_NA9700AA	11	936
ESC_NA9699AA	152	257
ESC_NA9698AA	152	257
ESC_NA9697AA	9008	627
ESC_NA9696AA	21	156
ESC_NA9695AA	659	286
ESC_NA9694AA	642	924
ESC_NA9693AA	21	156
ESC_NA9692AA	21	156
ESC_NA9691AA	25	234
ESC_NA9690AA	659	286
ESC_NA9689AA	17	191
ESC_NA9688AA	11	936
ESC_NA9687AA	17	191
ESC_NA9686AA	11	936
ESC_NA9505AA	21	156
ESC_NA9504AA	11	936
ESC_NA9503AA	11	936
ESC_NA9502AA	11	936
ESC_NA9501AA	10	512
ESC_NA9500AA	10	512
ESC_NA9499AA	10	512
ESC_NA9498AA	10	512
ESC_NA9497AA	10	512
ESC_NA9496AA	10	512
ESC_NA9495AA	10	512
ESC_NA9494AA	10	512
ESC_NA9493AA	10	512
ESC_NA9492AA	10	512
ESC_NA9491AA	10	512
ESC_NA9490AA	10	512
ESC_NA9489AA	10	512

ESC_NA9488AA	10	512
ESC_NA9487AA	10	512
ESC_NA9486AA	10	512
ESC_NA9485AA	10	512
ESC_NA9484AA	10105	546
ESC_NA9483AA	10105	546
ESC_NA9482AA	10105	546
ESC_NA9481AA	1125	198
ESC_NA9478AA	11	936
ESC_NA9477AA	11	936
ESC_NA9476AA	11	936
ESC_NA9475AA	11	936
ESC_NA9474AA	11	936
ESC_NA9473AA	11	936
ESC_NA9472AA	11	936
ESC_NA9471AA	11	936
ESC_NA9470AA	11	936
ESC_NA9469AA	11	936
ESC_NA9468AA	11	936
ESC_NA9467AA	659	286
ESC_NA9466AA	192	696
ESC_NA9465AA	11	936
ESC_NA9464AA	11	936
ESC_NA9463AA	11	936
ESC_NA9462AA	11	936
ESC_NA9461AA	17	191
ESC_NA9460AA	11	936
ESC_NA9459AA	21	156
ESC_NA9458AA	11	936
ESC_NA9457AA	32	270
ESC_NA9456AA	11	936
ESC_NA9455AA	692	234
ESC_NA9454AA	11	936
ESC_NA9453AA	4942	36
ESC_NA9452AA	32	270
ESC_NA9451AA	17	191
ESC_NA9450AA	10102	738
ESC_NA9449AA	10103	286
ESC_NA9448AA	16	156
ESC_NA9447AA	21	156
ESC_NA9446AA	11	936
ESC_NA9445AA	32	270
ESC_NA9444AA	11	936
ESC_NA9443AA	11	936
ESC_NA9442AA	11	936
ESC_NA9441AA	11	936
ESC_NA9440AA	11	936
ESC_NA9439AA	11	936
ESC_NA9438AA	361	512
ESC_NA9437AA	648	359

ESC_NA9436AA	156	175
ESC_NA9435AA	648	359
ESC_NA9434AA	156	175
ESC_NA9433AA	410	156
ESC_NA9432AA	167	512
ESC_NA9431AA	744	512
ESC_NA9430AA	10101	243
ESC_NA9429AA	167	512
ESC_NA9428AA	167	512
ESC_NA9427AA	617	511
ESC_NA9426AA	6448	175
ESC_NA9425AA	120	180
ESC_NA9423AA	167	512
ESC_NA9422AA	167	512
ESC_NA9421AA	617	511
ESC_NA9420AA	2011	362
ESC_NA9419AA	167	512
ESC_NA9418AA	410	156
ESC_NA9417AA	3541	842
ESC_NA9416AA	167	512
ESC_NA9415AA	167	512
ESC_NA9414AA	361	512
ESC_NA9413AA	46	447
ESC_NA9412AA	167	512
ESC_NA9411AA	245	261
ESC_NA9410AA	245	261
ESC_NA9409AA	245	261
ESC_NA9408AA	405	923
ESC_NA9407AA	10100	307
ESC_NA9406AA	11	936
ESC_NA9405AA	5292	738
ESC_NA9404AA	11	936
ESC_NA9403AA	11	936
ESC_NA9402AA	11	936
ESC_NA9401AA	11	936
ESC_NA9400AA	11	936
ESC_NA9399AA	11	936
ESC_NA9398AA	11	936
ESC_NA9397AA	11	936
ESC_NA9396AA	11	936
ESC_NA9395AA	152	257
ESC_NA9394AA	245	261
ESC_NA9393AA	152	257
ESC_NA9392AA	11	936
ESC_NA9391AA	11	936
ESC_NA9390AA	442	257
ESC_NA9389AA	33	198
ESC_NA9388AA	21	156
ESC_NA9387AA	381	156
ESC_NA9386AA	1819	808

ESC_NA9385AA	21	156
ESC_NA9264AA	167	512
ESC_NA9263AA	410	156
ESC_NA9262AA	205	257
ESC_NA9260AA	405	923
ESC_NA9259AA	12	626
ESC_NA9258AA	12	626
ESC_NA9257AA	12	626
ESC_NA9256AA	12	626
ESC_NA9255AA	12	626
ESC_NA9254AA	12	626
ESC_NA9252AA	12	626
ESC_NA9251AA	12	626
ESC_NA9250AA	12	626
ESC_NA9249AA	10	512
ESC_NA9248AA	11	936
ESC_NA9247AA	11	936
ESC_NA9246AA	11	936
ESC_NA9245AA	301	307
ESC_NA9244AA	301	307
ESC_NA9243AA	38	669
ESC_NA9240AA	21	156
ESC_NA9239AA	21	156
ESC_NA9237AA	21	156
ESC_NA9236AA	152	257
ESC_NA9235AA	152	257
ESC_NA9234AA	152	257
ESC_NA9233AA	152	257
ESC_NA9232AA	152	257
ESC_NA9231AA	245	261
ESC_NA9230AA	243	197
ESC_NA9229AA	630	261
ESC_NA9228AA	152	257
ESC_NA9227AA	11	936
ESC_NA9220AA	17	191
ESC_NA9219AA	17	191
ESC_NA9218AA	21	156
ESC_NA9216AA	11	936
ESC_NA9215AA	58	198
ESC_NA9213AA	11	936
ESC_NA9212AA	11	936
ESC_NA9211AA	10	512
ESC_NA9210AA	11	936
ESC_NA9207AA	11	936
ESC_NA9203AA	25	234
ESC_NA9202AA	11	936
ESC_NA9200AA	3101	160
ESC_NA9199AA	21	156
ESC_NA9197AA	300	286
ESC_NA9196AA	11	936

ESC_NA9195AA	11	936
ESC_NA9194AA	300	286
ESC_NA9193AA	398	445
ESC_NA9192AA	11	936
ESC_NA9191AA	10	512
ESC_NA9190AA	10	512
ESC_NA9189AA	11	936
ESC_NA9188AA	11	936
ESC_NA9187AA	11	936
ESC_NA9186AA	29	156
ESC_NA9185AA	11	936
ESC_NA9184AA	10	512
ESC_NA9183AA	131	653
ESC_NA9182AA	10	512
ESC_NA9181AA	10	512
ESC_NA9180AA	11	936
ESC_NA9179AA	219	877
ESC_NA9178AA	4481	257
ESC_NA9177AA	58	198
ESC_NA9176AA	3519	101
ESC_NA9175AA	4198	878
ESC_NA9174AA	616	156
ESC_NA9173AA	655	926
ESC_NA9172AA	17	191
ESC_NA9171AA	155	175
ESC_NA9170AA	295	160
ESC_NA9169AA	10	512
ESC_NA9168AA	388	156
ESC_NA9167AA	295	160
ESC_NA9166AA	846	915
ESC_NA9165AA	5708	605
ESC_NA9164AA	10097	696
ESC_NA9163AA	154	257
ESC_NA9162AA	11	936
ESC_NA9161AA	5748	924
ESC_NA9160AA	1114	504
ESC_NA9159AA	11	936
ESC_NA9158AA	641	935
ESC_NA9157AA	11	936
ESC_NA9156AA	10	512
ESC_NA9155AA	10	512
ESC_NA9153AA	10	512
ESC_NA9152AA	10098	935
ESC_NA9151AA	10	512
ESC_NA9150AA	10	512
ESC_NA9149AA	1679	160
ESC_NA9147AA	10	512
ESC_NA9146AA	10	512
ESC_NA9144AA	10	512
ESC_NA9140AA	10098	935

ESC_NA9139AA	219	877
ESC_NA9138AA	8359	156
ESC_NA9137AA	442	257
ESC_NA9136AA	10	512
ESC_NA9134AA	10	512
ESC_NA9133AA	1123	257
ESC_NA9130AA	10	512
ESC_NA9129AA	10099	268
ESC_NA9128AA	10098	935
ESC_NA9127AA	10	512
ESC_NA9126AA	641	935
ESC_NA9125AA	342	286
ESC_NA9124AA	11	936
ESC_NA9123AA	11	936
ESC_NA9122AA	11	936
ESC_NA9121AA	11	936
ESC_NA9120AA	11	936
ESC_NA9119AA	11	936
ESC_NA9118AA	11	936
ESC_NA9117AA	342	286
ESC_NA9116AA	11	936
ESC_NA9115AA	11	936
ESC_NA9114AA	11	936
ESC_NA9113AA	11	936
ESC_NA9112AA	11	936
ESC_NA9111AA	11	936
ESC_NA9110AA	343	286
ESC_NA9109AA	647	734
ESC_NA9108AA	647	734
ESC_NA9107AA	58	198
ESC_NA9106AA	342	286
ESC_NA9105AA	58	198
ESC_NA9104AA	245	261
ESC_NA9103AA	245	261
ESC_NA9102AA	245	261
ESC_NA9101AA	152	257
ESC_NA9100AA	152	257
ESC_NA9099AA	245	261
ESC_NA9098AA	10096	261
ESC_NA9097AA	152	257
ESC_NA9096AA	630	261
ESC_NA9095AA	152	257
ESC_NA9094AA	152	257
ESC_NA9093AA	245	261
ESC_NA9092AA	152	257
ESC_NA9091AA	152	257
ESC_NA9090AA	152	257
ESC_NA9089AA	442	257
ESC_NA9088AA	32	270
ESC_NA9087AA	21	156

ESC_NA9086AA	33	198
ESC_NA9085AA	21	156
ESC_NA9084AA	21	156
ESC_NA9083AA	6992	842
ESC_NA9082AA	4748	234
ESC_NA9081AA	17	191
ESC_NA9080AA	11	936
ESC_NA9079AA	21	156
ESC_NA9078AA	11	936
ESC_NA9077AA	11	936
ESC_NA9076AA	11	936
ESC_NA9075AA	33	198
ESC_NA9074AA	131	653
ESC_NA9073AA	10	512
ESC_NA9072AA	378	504
ESC_NA9071AA	73	737
ESC_NA9070AA	327	204
ESC_NA9069AA	327	204
ESC_NA9067AA	12	626
ESC_NA9066AA	12	626
ESC_NA9064AA	12	626
ESC_NA9063AA	12	626
ESC_NA9062AA	12	626
ESC_NA9061AA	12	626
ESC_NA9060AA	12	626
ESC_NA9058AA	12	626
ESC_NA9057AA	152	257
ESC_NA9056AA	10	512
ESC_NA9055AA	3574	257
ESC_NA9054AA	10095	602
ESC_NA9053AA	162	924
ESC_NA9052AA	10	512
ESC_NA9051AA	442	257
ESC_NA9050AA	129	257
ESC_NA9049AA	10	512
ESC_NA9048AA	947	257
ESC_NA9047AA	6841	160
ESC_NA9046AA	155	175
ESC_NA9045AA	10095	602
ESC_NA9044AA	8239	175
ESC_NA9043AA	6416	1004
ESC_NA9042AA	6732	1004
ESC_NA9041AA	2169	198
ESC_NA9040AA	1434	512
ESC_NA9039AA	10094	592
ESC_NA9038AA	906	198
ESC_NA9037AA	428	654
ESC_NA9036AA	11	936
ESC_NA9035AA	11	936
ESC_NA9032AA	11	936

ESC_NA9031AA	11	936
ESC_NA9029AA	11	936
ESC_NA9028AA	95	738
ESC_NA9027AA	95	738
ESC_NA9026AA	95	738
ESC_NA9025AA	95	738
ESC_NA9024AA	95	738
ESC_NA9023AA	10088	355
ESC_NA9022AA	95	738
ESC_NA9021AA	95	738
ESC_NA9020AA	95	738
ESC_NA9019AA	95	738
ESC_NA9018AA	95	738
ESC_NA9017AA	8702	76
ESC_NA9016AA	10087	286
ESC_NA9015AA	11	936
ESC_NA9014AA	7936	519
ESC_NA9013AA	10	512
ESC_NA9012AA	69	387
ESC_NA9011AA	8739	76
ESC_NA9010AA	10083	156
ESC_NA9009AA	152	257
ESC_NA9008AA	152	257
ESC_NA9007AA	152	257
ESC_NA9006AA	11	936
ESC_NA9005AA	937	198
ESC_NA9004AA	11	936
ESC_NA9003AA	11	936
ESC_NA9002AA	11	936
ESC_NA9001AA	46	447
ESC_NA9000AA	11	936
ESC_NA8999AA	1849	601
ESC_NA8998AA	11	936
ESC_NA8997AA	11	936
ESC_NA8996AA	446	257
ESC_NA8995AA	11	936
ESC_NA8994AA	11	936
ESC_NA8993AA	11	936
ESC_NA8992AA	145	256
ESC_NA8991AA	10084	936
ESC_NA8990AA	10085	936
ESC_NA8989AA	10083	156
ESC_NA8988AA	8381	234
ESC_NA8987AA	152	257
ESC_NA8986AA	152	257
ESC_NA8985AA	245	261
ESC_NA8984AA	442	257
ESC_NA8983AA	11	936
ESC_NA8961AA	1205	198
ESC_NA8960AA	11	936

ESC_NA8959AA	11	936
ESC_NA8958AA	11	936
ESC_NA8957AA	11	936
ESC_NA8956AA	11	936
ESC_NA8955AA	11	936
ESC_NA8954AA	11	936
ESC_NA8953AA	295	160
ESC_NA8952AA	4197	856
ESC_NA8951AA	11	936
ESC_NA8950AA	1423	198
ESC_NA8949AA	11	936
ESC_NA8948AA	1508	261
ESC_NA8947AA	48	76
ESC_NA8946AA	2005	175
ESC_NA8945AA	11	936
ESC_NA8944AA	11	936
ESC_NA8943AA	11	936
ESC_NA8942AA	2485	212
ESC_NA8941AA	10	512
ESC_NA8940AA	11	936
ESC_NA8939AA	11	936
ESC_NA8938AA	11	936
ESC_NA8937AA	11	936
ESC_NA8936AA	11	936
ESC_NA8935AA	11	936
ESC_NA8934AA	21	156
ESC_NA8933AA	155	175
ESC_NA8932AA	10	512
ESC_NA8931AA	10	512
ESC_NA8930AA	10	512
ESC_NA8929AA	10	512
ESC_NA8928AA	10	512
ESC_NA8927AA	10	512
ESC_NA8926AA	10	512
ESC_NA8925AA	10	512
ESC_NA8924AA	10	512
ESC_NA8923AA	10082	504
ESC_NA8922AA	10	512
ESC_NA8921AA	10	512
ESC_NA8920AA	10	512
ESC_NA8919AA	141	644
ESC_NA8918AA	10	512
ESC_NA8917AA	10	512
ESC_NA8916AA	10	512
ESC_NA8915AA	10	512
ESC_NA8914AA	11	936
ESC_NA8913AA	245	261
ESC_NA8912AA	152	257
ESC_NA8911AA	744	512
ESC_NA8910AA	10081	175

ESC_NA8909AA	410	156
ESC_NA8908AA	405	923
ESC_NA8907AA	405	923
ESC_NA8906AA	167	512
ESC_NA8905AA	940	257
ESC_NA8904AA	410	156
ESC_NA8903AA	940	257
ESC_NA8902AA	167	512
ESC_NA8901AA	540	286
ESC_NA8900AA	410	156
ESC_NA8899AA	448	257
ESC_NA8898AA	44	512
ESC_NA8897AA	636	653
ESC_NA8896AA	10	512
ESC_NA8895AA	410	156
ESC_NA8894AA	245	261
ESC_NA8893AA	152	257
ESC_NA8892AA	10	512
ESC_NA8891AA	206	286
ESC_NA8890AA	3381	268
ESC_NA8889AA	206	286
ESC_NA8888AA	11	936
ESC_NA8886AA	10	512
ESC_NA8885AA	10	512
ESC_NA8884AA	10	512
ESC_NA8883AA	10	512
ESC_NA8882AA	29	156
ESC_NA8881AA	155	175
ESC_NA8880AA	11	936
ESC_NA8879AA	11	936
ESC_NA8878AA	297	234
ESC_NA8877AA	4429	820
ESC_NA8876AA	11	936
ESC_NA8875AA	11	936
ESC_NA8874AA	10	512
ESC_NA8873AA	11	936
ESC_NA8872AA	297	234
ESC_NA8871AA	189	504
ESC_NA8869AA	33	198
ESC_NA8868AA	11	936
ESC_NA8867AA	2836	221
ESC_NA8866AA	33	198
ESC_NA8865AA	10	512
ESC_NA8864AA	711	935
ESC_NA8863AA	386	191
ESC_NA8862AA	17	191
ESC_NA8861AA	11	936
ESC_NA8860AA	21	156
ESC_NA8859AA	11	936
ESC_NA8858AA	11	936

ESC_NA8857AA	11	936
ESC_NA8856AA	11	936
ESC_NA8855AA	11	936
ESC_NA8854AA	11	936
ESC_NA8853AA	589	650
ESC_NA8850AA	5378	197
ESC_NA8849AA	345	175
ESC_NA8484AA	11	936
ESC_NA8483AA	11	936
ESC_NA8482AA	11	936
ESC_NA8481AA	5783	817
ESC_NA8480AA	10	512
ESC_NA8479AA	6622	519
ESC_NA8478AA	405	923
ESC_NA8477AA	167	512
ESC_NA8476AA	167	512
ESC_NA8475AA	167	512
ESC_NA8474AA	405	923
ESC_NA8473AA	410	156
ESC_NA8472AA	167	512
ESC_NA8471AA	1284	604
ESC_NA8470AA	648	359
ESC_NA8469AA	410	156
ESC_NA8468AA	361	512
ESC_NA8466AA	10042	512
ESC_NA8465AA	167	512
ESC_NA8464AA	46	447
ESC_NA8463AA	167	512
ESC_NA8462AA	410	156
ESC_NA8461AA	167	512
ESC_NA8460AA	617	511
ESC_NA8459AA	405	923
ESC_NA8458AA	410	156
ESC_NA8457AA	167	512
ESC_NA8456AA	410	156
ESC_NA8455AA	405	923
ESC_NA8454AA	410	156
ESC_NA8453AA	744	512
ESC_NA8452AA	361	512
ESC_NA8451AA	10	512
ESC_NA8450AA	167	512
ESC_NA8449AA	405	923
ESC_NA8448AA	1702	512
ESC_NA8447AA	405	923
ESC_NA8446AA	410	156
ESC_NA8445AA	2659	679
ESC_NA8444AA	46	447
ESC_NA8443AA	205	257
ESC_NA8442AA	405	923
ESC_NA8441AA	405	923

ESC_NA8440AA	361	512
ESC_NA8439AA	167	512
ESC_NA8438AA	10	512
ESC_NA8437AA	448	257
ESC_NA8436AA	95	738
ESC_NA8435AA	95	738
ESC_NA8434AA	95	738
ESC_NA8433AA	95	738
ESC_NA8432AA	95	738
ESC_NA8431AA	95	738
ESC_NA8430AA	95	738
ESC_NA8429AA	95	738
ESC_NA8428AA	95	738
ESC_NA8427AA	95	738
ESC_NA8425AA	48	76
ESC_NA8424AA	442	257
ESC_NA8423AA	11	936
ESC_NA8422AA	11	936
ESC_NA8421AA	11	936
ESC_NA8420AA	11	936
ESC_NA8419AA	11	936
ESC_NA8418AA	29	156
ESC_NA8417AA	11	936
ESC_NA8416AA	11	936
ESC_NA8415AA	11	936
ESC_NA8414AA	11	936
ESC_NA8413AA	11	936
ESC_NA8412AA	11	936
ESC_NA8411AA	11	936
ESC_NA8410AA	152	257
ESC_NA8408AA	11	936
ESC_NA8407AA	11	936
ESC_NA8406AA	152	257
ESC_NA8405AA	245	261
ESC_NA8404AA	630	261
ESC_NA8403AA	442	257
ESC_NA8402AA	21	156
ESC_NA8401AA	152	257
ESC_NA8400AA	245	261
ESC_NA8399AA	152	257
ESC_NA8398AA	152	257
ESC_NA8397AA	152	257
ESC_NA8396AA	152	257
ESC_NA8395AA	17	191
ESC_NA8394AA	17	191
ESC_NA8393AA	131	653
ESC_NA8392AA	21	156
ESC_NA8391AA	677	257
ESC_NA8390AA	386	191
ESC_NA8389AA	6	445

ESC_NA8388AA	25	234
ESC_NA8387AA	442	257
ESC_NA8386AA	32	270
ESC_NA8385AA	21	156
ESC_NA8384AA	21	156
ESC_NA8383AA	442	257
ESC_NA8382AA	152	257
ESC_NA8380AA	11	936
ESC_NA8379AA	847	257
ESC_NA8378AA	11	936
ESC_NA8377AA	2338	257
ESC_NA8376AA	73	737
ESC_NA8375AA	11	936
ESC_NA8374AA	297	234
ESC_NA8373AA	372	424
ESC_NA8372AA	5292	738
ESC_NA8371AA	8700	924
ESC_NA8370AA	1249	257
ESC_NA8369AA	657	592
ESC_NA8368AA	329	355
ESC_NA8367AA	301	307
ESC_NA8366AA	1892	846
ESC_NA8365AA	101	696
ESC_NA8364AA	301	307
ESC_NA8363AA	101	696
ESC_NA8362AA	201	257
ESC_NA8361AA	29	156
ESC_NA8360AA	162	924
ESC_NA8359AA	69	387
ESC_NA8358AA	812	649
ESC_NA8357AA	25	234
ESC_NA8356AA	32	270
ESC_NA8355AA	25	234
ESC_NA8354AA	297	234
ESC_NA8353AA	101	696
ESC_NA8352AA	10	512
ESC_NA8351AA	8930	234
ESC_NA8350AA	583	768
ESC_NA8349AA	88	160
ESC_NA8348AA	201	257
ESC_NA8347AA	10	512
ESC_NA8346AA	10	512
ESC_NA8345AA	10	512
ESC_NA8344AA	675	234
ESC_NA8343AA	6274	257
ESC_NA8342AA	173	254
ESC_NA8341AA	33	198
ESC_NA8340AA	10069	503
ESC_NA8339AA	10	512
ESC_NA8338AA	306	286

ESC_NA8337AA	10069	503
ESC_NA8336AA	582	420
ESC_NA8335AA	812	649
ESC_NA8334AA	2514	504
ESC_NA8333AA	753	842
ESC_NA8332AA	10	512
ESC_NA8331AA	278	924
ESC_NA8330AA	10071	820
ESC_NA8329AA	301	307
ESC_NA8328AA	23	156
ESC_NA8327AA	10	512
ESC_NA8326AA	582	420
ESC_NA8325AA	301	307
ESC_NA8324AA	937	198
ESC_NA8323AA	301	307
ESC_NA8322AA	32	270
ESC_NA8321AA	11	936
ESC_NA8320AA	993	789
ESC_NA8319AA	767	198
ESC_NA8318AA	88	160
ESC_NA8317AA	155	175
ESC_NA8316AA	1819	808
ESC_NA8315AA	141	644
ESC_NA8314AA	40	198
ESC_NA8313AA	1819	808
ESC_NA8312AA	10070	592
ESC_NA8311AA	301	307
ESC_NA8310AA	388	156
ESC_NA8309AA	718	257
ESC_NA8308AA	26	257
ESC_NA8307AA	6060	41
ESC_NA8306AA	301	307
ESC_NA8305AA	753	842
ESC_NA8304AA	329	355
ESC_NA8303AA	119	286
ESC_NA8302AA	937	198
ESC_NA8301AA	657	592
ESC_NA8300AA	10072	899
ESC_NA8299AA	388	156
ESC_NA8298AA	10	512
ESC_NA8297AA	11	936
ESC_NA8296AA	29	156
ESC_NA8295AA	1176	343
ESC_NA8294AA	1176	343
ESC_NA8293AA	300	286
ESC_NA8292AA	11	936
ESC_NA8291AA	33	198
ESC_NA8290AA	3695	924
ESC_NA8289AA	672	198
ESC_NA8288AA	1176	343

ESC_NA8287AA	10	512
ESC_NA8286AA	1176	343
ESC_NA8285AA	1176	343
ESC_NA8284AA	17	191
ESC_NA8283AA	17	191
ESC_NA8282AA	11	936
ESC_NA8281AA	32	270
ESC_NA8280AA	1804	935
ESC_NA8279AA	11	936
ESC_NA8278AA	11	936
ESC_NA8277AA	11	936
ESC_NA8276AA	11	936
ESC_NA8275AA	583	768
ESC_NA8274AA	937	198
ESC_NA8273AA	141	644
ESC_NA8272AA	737	175
ESC_NA8271AA	10073	136
ESC_NA8270AA	677	257
ESC_NA8268AA	17	191
ESC_NA8262AA	301	307
ESC_NA8261AA	32	270
ESC_NA8260AA	753	842
ESC_NA8259AA	301	307
ESC_NA8258AA	11	936
ESC_NA8257AA	812	649
ESC_NA8254AA	11	936
ESC_NA8253AA	6274	257
ESC_NA8252AA	659	286
ESC_NA8251AA	11	936
ESC_NA8250AA	11	936
ESC_NA8249AA	11	936
ESC_NA8248AA	11	936
ESC_NA8247AA	21	156
ESC_NA8246AA	11	936
ESC_NA8245AA	659	286
ESC_NA8244AA	11	936
ESC_NA8243AA	17	191
ESC_NA8242AA	672	198
ESC_NA8241AA	587	936
ESC_NA8240AA	11	936
ESC_NA8239AA	11	936
ESC_NA8238AA	11	936
ESC_NA8237AA	11	936
ESC_NA8236AA	11	936
ESC_NA8235AA	11	936
ESC_NA8234AA	335	936
ESC_NA8233AA	17	191
ESC_NA8232AA	11	936
ESC_NA8231AA	11	936
ESC_NA8230AA	11	936

ESC_NA8228AA	2520	878
ESC_NA8227AA	223	198
ESC_NA8226AA	583	768
ESC_NA8225AA	753	842
ESC_NA8224AA	40	198
ESC_NA8223AA	583	768
ESC_NA8222AA	675	234
ESC_NA8221AA	583	768
ESC_NA8220AA	3519	101
ESC_NA8218AA	136	392
ESC_NA8217AA	136	392
ESC_NA8215AA	10076	515
ESC_NA8214AA	301	307
ESC_NA8213AA	70	670
ESC_NA8212AA	753	842
ESC_NA8211AA	26	257
ESC_NA8210AA	10	512
ESC_NA8209AA	10	512
ESC_NA8208AA	753	842
ESC_NA8207AA	675	234
ESC_NA8206AA	675	234
ESC_NA8205AA	937	198
ESC_NA8204AA	657	592
ESC_NA8203AA	10075	842
ESC_NA8202AA	278	924
ESC_NA8200AA	360	156
ESC_NA8199AA	300	286
ESC_NA8198AA	40	198
ESC_NA8197AA	10078	592
ESC_NA8196AA	9300	900
ESC_NA8195AA	753	842
ESC_NA8194AA	11	936
ESC_NA8193AA	10074	936
ESC_NA8192AA	11	936
ESC_NA8191AA	11	936
ESC_NA8190AA	11	936
ESC_NA8189AA	11	936
ESC_NA8188AA	11	936
ESC_NA8187AA	11	936
ESC_NA8186AA	11	936
ESC_NA8185AA	4017	257
ESC_NA8184AA	2101	108
ESC_NA8183AA	8237	504
ESC_NA8182AA	446	257
ESC_NA8181AA	6274	257
ESC_NA8180AA	937	198
ESC_NA8179AA	11	936
ESC_NA8178AA	11	936
ESC_NA8177AA	11	936
ESC_NA8176AA	8700	924

ESC_NA8175AA	993	789
ESC_NA8174AA	69	387
ESC_NA8173AA	4975	924
ESC_NA8172AA	10077	234
ESC_NA8171AA	336	878
ESC_NA8170AA	3101	160
ESC_NA8169AA	13	257
ESC_NA8168AA	8649	160
ESC_NA8167AA	3101	160
ESC_NA8166AA	753	842
ESC_NA8165AA	677	257
ESC_NA8164AA	4942	36
ESC_NA8163AA	16	156
ESC_NA8162AA	398	445
ESC_NA8161AA	937	198
ESC_NA8160AA	8384	738
ESC_NA8159AA	2325	447
ESC_NA8158AA	6069	344
ESC_NA8157AA	295	160
ESC_NA8156AA	69	387
ESC_NA8155AA	6008	447
ESC_NA8154AA	10	512
ESC_NA8153AA	746	789
ESC_NA8152AA	223	198
ESC_NA8151AA	154	257
ESC_NA8150AA	2967	289
ESC_NA8149AA	212	198
ESC_NA8148AA	362	234
ESC_NA8147AA	7616	257
ESC_NA8146AA	1850	269
ESC_NA8145AA	602	257
ESC_NA8144AA	401	142
ESC_NA8143AA	10	512
ESC_NA8142AA	428	654
ESC_NA8141AA	1914	956
ESC_NA8140AA	1785	791
ESC_NA8139AA	38	669
ESC_NA8138AA	2207	474
ESC_NA8137AA	10080	508
ESC_NA8136AA	115	669
ESC_NA8135AA	1992	257
ESC_NA8134AA	3580	257
ESC_NA8133AA	10	512
ESC_NA8132AA	12	626
ESC_NA8131AA	11	936
ESC_NA8130AA	410	156
ESC_NA8129AA	5559	257
ESC_NA8128AA	10	512
ESC_NA8127AA	5559	257
ESC_NA8125AA	10	512

ESC_NA8123AA	21	156
ESC_NA8121AA	10	512
ESC_NA8120AA	10	512
ESC_NA8119AA	718	257
ESC_NA8117AA	300	286
ESC_NA8116AA	9137	900
ESC_NA8115AA	372	424
ESC_NA8114AA	10	512
ESC_NA8113AA	11	936
ESC_NA8112AA	5559	257
ESC_NA8111AA	58	198
ESC_NA8110AA	10	512
ESC_NA8109AA	405	923
ESC_NA8108AA	10	512
ESC_NA8107AA	372	424
ESC_NA8106AA	73	737
ESC_NA8105AA	10079	709
ESC_NA8104AA	410	156
ESC_NA8103AA	448	257
ESC_NA8102AA	127	738
ESC_NA8100AA	5559	257
ESC_NA8099AA	847	257
ESC_NA8098AA	224	198
ESC_NA8096AA	5559	257
ESC_NA8095AA	11	936
ESC_NA8094AA	5559	257
ESC_NA8093AA	141	644
ESC_NA8092AA	5559	257
ESC_NA8091AA	223	198
ESC_NA8090AA	2329	924
ESC_NA8089AA	372	424
ESC_NA8088AA	2520	878
ESC_NA8087AA	38	669
ESC_NA8086AA	1049	175
ESC_NA8085AA	446	257
ESC_NA8084AA	332	257
ESC_NA8083AA	2389	160
ESC_NA8082AA	11	936
ESC_NA8080AA	349	670
ESC_NA8079AA	11	936
ESC_NA8078AA	11	936
ESC_NA8077AA	21	156
ESC_NA8076AA	4496	924
ESC_NA8075AA	300	286
ESC_NA8074AA	663	924
ESC_NA8073AA	4975	924
ESC_NA8072AA	155	175
ESC_NA8071AA	10	512
ESC_NA8070AA	753	842
ESC_NA8069AA	642	924

ESC_NA8068AA	300	286
ESC_NA8067AA	993	789
ESC_NA8066AA	301	307
ESC_NA8065AA	515	1004
ESC_NA8064AA	11	936
ESC_NA8063AA	306	286
ESC_NA8062AA	11	936
ESC_NA8061AA	812	649
ESC_NA8060AA	504	738
ESC_NA8059AA	131	653
ESC_NA8058AA	152	257
ESC_NA8057AA	152	257
ESC_NA8056AA	152	257
ESC_NA8055AA	152	257
ESC_NA8054AA	69	387
ESC_NA8053AA	23	156
ESC_NA8052AA	10045	234
ESC_NA8051AA	10044	235
ESC_NA8050AA	1406	174
ESC_NA8049AA	401	142
ESC_NA8048AA	117	592
ESC_NA8047AA	46	447
ESC_NA8046AA	429	654
ESC_NA8045AA	57	50
ESC_NA8044AA	10043	764
ESC_NA8043AA	1611	198
ESC_NA8042AA	58	198
ESC_NA8041AA	10	512
ESC_NA8040AA	101	696
ESC_NA8039AA	409	504
ESC_NA8038AA	1236	380
ESC_NA8037AA	8027	935
ESC_NA8036AA	69	387
ESC_NA8035AA	399	198
ESC_NA8034AA	117	592
ESC_NA8033AA	2556	630
ESC_NA8032AA	9711	562
ESC_NA8031AA	10	512
ESC_NA8030AA	5308	261
ESC_NA8029AA	1072	355
ESC_NA8028AA	117	592
ESC_NA8027AA	2077	198
ESC_NA8026AA	1316	471
ESC_NA8025AA	69	387
ESC_NA8024AA	117	592
ESC_NA8023AA	399	198
ESC_NA8022AA	1123	257
ESC_NA8021AA	224	198
ESC_NA8020AA	2325	447
ESC_NA8019AA	131	653

ESC_NA8018AA	3519	101
ESC_NA8016AA	652	204
ESC_NA8015AA	93	71
ESC_NA8014AA	10	512
ESC_NA8013AA	117	592
ESC_NA8012AA	641	935
ESC_NA8011AA	117	592
ESC_NA8010AA	1582	198
ESC_NA8009AA	10	512
ESC_NA8008AA	58	198
ESC_NA8007AA	399	198
ESC_NA8006AA	297	234
ESC_NA8005AA	117	592
ESC_NA8004AA	399	198
ESC_NA8003AA	10047	268
ESC_NA8002AA	155	175
ESC_NA8001AA	58	198
ESC_NA8000AA	399	198
ESC_NA7999AA	6096	257
ESC_NA7998AA	58	198
ESC_NA7997AA	6780	824
ESC_NA7996AA	515	1004
ESC_NA7995AA	10046	257
ESC_NA7994AA	10	512
ESC_NA7993AA	1252	567
ESC_NA7992AA	297	234
ESC_NA7991AA	1434	512
ESC_NA7990AA	48	76
ESC_NA7989AA	10	512
ESC_NA7988AA	3580	257
ESC_NA7987AA	162	924
ESC_NA7986AA	154	257
ESC_NA7985AA	1139	447
ESC_NA7984AA	73	737
ESC_NA7983AA	2207	474
ESC_NA7982AA	131	653
ESC_NA7981AA	278	924
ESC_NA7980AA	1611	198
ESC_NA7979AA	69	387
ESC_NA7978AA	1294	512
ESC_NA7977AA	8951	943
ESC_NA7976AA	10	512
ESC_NA7975AA	1326	198
ESC_NA7974AA	616	156
ESC_NA7973AA	1429	198
ESC_NA7972AA	162	924
ESC_NA7971AA	2171	164
ESC_NA7970AA	58	198
ESC_NA7969AA	372	424
ESC_NA7968AA	117	592

ESC_NA7967AA	2077	198
ESC_NA7966AA	399	198
ESC_NA7965AA	4045	592
ESC_NA7964AA	3601	653
ESC_NA7963AA	607	355
ESC_NA7962AA	117	592
ESC_NA7961AA	2002	778
ESC_NA7960AA	388	156
ESC_NA7959AA	10	512
ESC_NA7958AA	355	764
ESC_NA7957AA	86	935
ESC_NA7956AA	165	504
ESC_NA7955AA	2178	924
ESC_NA7954AA	10	512
ESC_NA7953AA	58	198
ESC_NA7952AA	345	175
ESC_NA7951AA	10	512
ESC_NA7950AA	165	504
ESC_NA7949AA	117	592
ESC_NA7948AA	10048	286
ESC_NA7947AA	355	764
ESC_NA7946AA	1246	198
ESC_NA7945AA	1303	791
ESC_NA7944AA	607	355
ESC_NA7943AA	2473	257
ESC_NA7942AA	2253	71
ESC_NA7941AA	10	512
ESC_NA7940AA	4493	257
ESC_NA7939AA	155	175
ESC_NA7938AA	10050	198
ESC_NA7937AA	10	512
ESC_NA7936AA	1434	512
ESC_NA7935AA	2040	198
ESC_NA7934AA	2242	234
ESC_NA7933AA	1172	234
ESC_NA7932AA	10049	512
ESC_NA7931AA	1434	512
ESC_NA7930AA	1722	599
ESC_NA7929AA	93	71
ESC_NA7928AA	607	355
ESC_NA7927AA	58	198
ESC_NA7926AA	2144	257
ESC_NA7925AA	399	198
ESC_NA7924AA	57	50
ESC_NA7923AA	155	175
ESC_NA7922AA	3580	257
ESC_NA7921AA	1434	512
ESC_NA7920AA	2175	212
ESC_NA7919AA	56	198
ESC_NA7918AA	602	257

ESC_NA7917AA	10051	307
ESC_NA7916AA	131	653
ESC_NA7915AA	1727	257
ESC_NA7914AA	48	76
ESC_NA7913AA	442	257
ESC_NA7912AA	3580	257
ESC_NA7911AA	7616	257
ESC_NA7910AA	1236	380
ESC_NA7909AA	1434	512
ESC_NA7908AA	109	257
ESC_NA7907AA	1429	198
ESC_NA7906AA	580	445
ESC_NA7905AA	399	198
ESC_NA7904AA	131	653
ESC_NA7903AA	3580	257
ESC_NA7902AA	3580	257
ESC_NA7901AA	101	696
ESC_NA7900AA	5931	670
ESC_NA7899AA	607	355
ESC_NA7898AA	10052	198
ESC_NA7897AA	3580	257
ESC_NA7896AA	117	592
ESC_NA7895AA	10	512
ESC_NA7894AA	1385	653
ESC_NA7893AA	48	76
ESC_NA7892AA	607	355
ESC_NA7891AA	3580	257
ESC_NA7890AA	131	653
ESC_NA7889AA	58	198
ESC_NA7888AA	1429	198
ESC_NA7886AA	8609	474
ESC_NA7885AA	2485	212
ESC_NA7884AA	10	512
ESC_NA7883AA	1434	512
ESC_NA7882AA	2606	198
ESC_NA7881AA	409	504
ESC_NA7880AA	218	519
ESC_NA7879AA	1385	653
ESC_NA7878AA	2792	886
ESC_NA7877AA	399	198
ESC_NA7876AA	5840	234
ESC_NA7875AA	48	76
ESC_NA7874AA	162	924
ESC_NA7873AA	4360	842
ESC_NA7872AA	1385	653
ESC_NA7871AA	978	740
ESC_NA7870AA	355	764
ESC_NA7869AA	278	924
ESC_NA7868AA	10053	637
ESC_NA7867AA	10053	637

ESC_NA7866AA	1385	653
ESC_NA7865AA	101	696
ESC_NA7864AA	8602	567
ESC_NA7863AA	7040	156
ESC_NA7862AA	1434	512
ESC_NA7861AA	569	738
ESC_NA7860AA	616	156
ESC_NA7859AA	906	198
ESC_NA7858AA	278	924
ESC_NA7857AA	746	789
ESC_NA7856AA	58	198
ESC_NA7855AA	1844	198
ESC_NA7854AA	155	175
ESC_NA7853AA	2538	900
ESC_NA7852AA	117	592
ESC_NA7851AA	278	924
ESC_NA7850AA	10	512
ESC_NA7849AA	38	669
ESC_NA7848AA	357	654
ESC_NA7847AA	155	175
ESC_NA7846AA	117	592
ESC_NA7845AA	349	670
ESC_NA7844AA	58	198
ESC_NA7843AA	38	669
ESC_NA7842AA	10055	696
ESC_NA7841AA	218	519
ESC_NA7840AA	1944	934
ESC_NA7839AA	355	764
ESC_NA7838AA	602	257
ESC_NA7837AA	429	654
ESC_NA7836AA	10054	501
ESC_NA7835AA	2702	900
ESC_NA7834AA	392	234
ESC_NA7833AA	58	198
ESC_NA7832AA	8598	935
ESC_NA7831AA	2606	198
ESC_NA7830AA	388	156
ESC_NA7829AA	10056	593
ESC_NA7828AA	48	76
ESC_NA7827AA	1236	380
ESC_NA7826AA	616	156
ESC_NA7825AA	10058	160
ESC_NA7824AA	5295	355
ESC_NA7823AA	6078	512
ESC_NA7822AA	1385	653
ESC_NA7821AA	1972	406
ESC_NA7820AA	1406	174
ESC_NA7819AA	301	307
ESC_NA7818AA	58	198
ESC_NA7817AA	428	654

ESC_NA7816AA	10057	257
ESC_NA7815AA	38	669
ESC_NA7814AA	399	198
ESC_NA7813AA	1434	512
ESC_NA7812AA	8951	943
ESC_NA7811AA	117	592
ESC_NA7810AA	23	156
ESC_NA7809AA	7616	257
ESC_NA7808AA	101	696
ESC_NA7807AA	641	935
ESC_NA7806AA	410	156
ESC_NA7805AA	3580	257
ESC_NA7804AA	117	592
ESC_NA7803AA	58	198
ESC_NA7802AA	681	393
ESC_NA7801AA	648	359
ESC_NA7800AA	93	71
ESC_NA7799AA	38	669
ESC_NA7798AA	1914	956
ESC_NA7797AA	428	654
ESC_NA7796AA	58	198
ESC_NA7795AA	58	198
ESC_NA7794AA	770	587
ESC_NA7793AA	2079	198
ESC_NA7792AA	57	50
ESC_NA7791AA	101	696
ESC_NA7790AA	10	512
ESC_NA7789AA	58	198
ESC_NA7788AA	278	924
ESC_NA7787AA	10059	512
ESC_NA7786AA	58	198
ESC_NA7785AA	8211	257
ESC_NA7784AA	58	198
ESC_NA7783AA	101	696
ESC_NA7782AA	770	587
ESC_NA7781AA	937	198
ESC_NA7780AA	23	156
ESC_NA7779AA	2967	289
ESC_NA7778AA	1141	512
ESC_NA7777AA	10	512
ESC_NA7776AA	101	696
ESC_NA7775AA	295	160
ESC_NA7774AA	1618	736
ESC_NA7773AA	3580	257
ESC_NA7772AA	86	935
ESC_NA7771AA	3519	101
ESC_NA7770AA	1434	512
ESC_NA7769AA	2520	878
ESC_NA7768AA	5956	71
ESC_NA7767AA	457	595

ESC_NA7766AA	428	654
ESC_NA7765AA	602	257
ESC_NA7764AA	6061	71
ESC_NA7763AA	3580	257
ESC_NA7762AA	117	592
ESC_NA7761AA	3090	212
ESC_NA7760AA	101	696
ESC_NA7759AA	501	900
ESC_NA7758AA	117	592
ESC_NA7757AA	3580	257
ESC_NA7756AA	392	234
ESC_NA7755AA	6694	234
ESC_NA7754AA	10	512
ESC_NA7753AA	10060	447
ESC_NA7752AA	69	387
ESC_NA7751AA	216	512
ESC_NA7750AA	109	257
ESC_NA7749AA	744	512
ESC_NA7748AA	366	198
ESC_NA7747AA	154	257
ESC_NA7746AA	3580	257
ESC_NA7745AA	155	175
ESC_NA7744AA	5308	261
ESC_NA7743AA	1727	257
ESC_NA7742AA	117	592
ESC_NA7741AA	216	512
ESC_NA7740AA	355	764
ESC_NA7739AA	2329	924
ESC_NA7738AA	681	393
ESC_NA7737AA	1429	198
ESC_NA7736AA	5308	261
ESC_NA7735AA	540	286
ESC_NA7734AA	224	198
ESC_NA7733AA	345	175
ESC_NA7732AA	134	738
ESC_NA7731AA	442	257
ESC_NA7730AA	10	512
ESC_NA7729AA	10	512
ESC_NA7728AA	58	198
ESC_NA7727AA	355	764
ESC_NA7726AA	3234	257
ESC_NA7725AA	607	355
ESC_NA7724AA	906	198
ESC_NA7723AA	10	512
ESC_NA7722AA	101	696
ESC_NA7721AA	10	512
ESC_NA7720AA	428	654
ESC_NA7719AA	10	512
ESC_NA7718AA	23	156
ESC_NA7717AA	770	587

ESC_NA7716AA	5378	197
ESC_NA7715AA	1844	198
ESC_NA7714AA	7283	669
ESC_NA7713AA	10	512
ESC_NA7712AA	10061	512
ESC_NA7711AA	399	198
ESC_NA7710AA	1429	198
ESC_NA7709AA	351	268
ESC_NA7708AA	607	355
ESC_NA7707AA	101	696
ESC_NA7706AA	602	257
ESC_NA7705AA	23	156
ESC_NA7704AA	23	156
ESC_NA7703AA	191	198
ESC_NA7702AA	401	142
ESC_NA7701AA	355	764
ESC_NA7700AA	101	696
ESC_NA7699AA	196	257
ESC_NA7698AA	10062	924
ESC_NA7697AA	607	355
ESC_NA7696AA	10	512
ESC_NA7695AA	5765	234
ESC_NA7694AA	731	285
ESC_NA7693AA	58	198
ESC_NA7692AA	20	198
ESC_NA7691AA	3322	824
ESC_NA7690AA	117	592
ESC_NA7689AA	3107	512
ESC_NA7688AA	3580	257
ESC_NA7687AA	10	512
ESC_NA7686AA	501	900
ESC_NA7685AA	2556	630
ESC_NA7684AA	212	198
ESC_NA7683AA	10064	711
ESC_NA7682AA	355	764
ESC_NA7681AA	1244	291
ESC_NA7680AA	10	512
ESC_NA7679AA	10	512
ESC_NA7678AA	196	257
ESC_NA7677AA	155	175
ESC_NA7676AA	1159	381
ESC_NA7675AA	117	592
ESC_NA7674AA	401	142
ESC_NA7673AA	10065	387
ESC_NA7672AA	10063	198
ESC_NA7671AA	162	924
ESC_NA7670AA	2171	164
ESC_NA7669AA	164	175
ESC_NA7668AA	540	286
ESC_NA7667AA	165	504

ESC_NA7666AA	5378	197
ESC_NA7665AA	3322	824
ESC_NA7664AA	135	653
ESC_NA7663AA	328	924
ESC_NA7662AA	117	592
ESC_NA7661AA	355	764
ESC_NA7660AA	1236	380
ESC_NA7659AA	155	175
ESC_NA7658AA	428	654
ESC_NA7657AA	164	175
ESC_NA7656AA	48	76
ESC_NA7655AA	2329	924
ESC_NA7654AA	491	626
ESC_NA7653AA	1286	474
ESC_NA7652AA	8951	943
ESC_NA7651AA	162	924
ESC_NA7650AA	3580	257
ESC_NA7649AA	155	175
ESC_NA7648AA	155	175
ESC_NA7647AA	429	654
ESC_NA7646AA	366	198
ESC_NA7645AA	706	422
ESC_NA7644AA	10066	198
ESC_NA7643AA	2005	175
ESC_NA7642AA	6236	856
ESC_NA7641AA	58	198
ESC_NA7640AA	164	175
ESC_NA7639AA	744	512
ESC_NA7638AA	457	595
ESC_NA7637AA	399	198
ESC_NA7636AA	3580	257
ESC_NA7635AA	2026	198
ESC_NA7634AA	1434	512
ESC_NA7633AA	10	512
ESC_NA7632AA	58	198
ESC_NA7631AA	10	512
ESC_NA7630AA	1721	311
ESC_NA7629AA	428	654
ESC_NA7628AA	117	592
ESC_NA7627AA	453	935
ESC_NA7626AA	5748	924
ESC_NA7625AA	58	198
ESC_NA7624AA	2541	878
ESC_NA7623AA	155	175
ESC_NA7622AA	2541	878
ESC_NA7621AA	542	824
ESC_NA7620AA	58	198
ESC_NA7619AA	2541	878
ESC_NA7618AA	2541	878
ESC_NA7617AA	2541	878

ESC_NA7616AA	542	824
ESC_NA7615AA	58	198
ESC_NA7614AA	10068	234
ESC_NA7613AA	17	191
ESC_NA7612AA	4995	198
ESC_NA7611AA	155	175
ESC_NA7610AA	223	198
ESC_NA7606AA	91	629
ESC_NA7605AA	10	512
ESC_NA7604AA	144	740
ESC_NA7603AA	738	598
ESC_NA7602AA	738	598
ESC_NA7601AA	69	387
ESC_NA7599AA	765	156
ESC_NA7598AA	443	257
ESC_NA7597AA	10067	924
ESC_NA7596AA	11	936
ESC_NA7595AA	327	204
ESC_NA7594AA	641	935
ESC_NA7593AA	10011	221
ESC_NA7592AA	10011	221
ESC_NA7591AA	10010	65
ESC_NA7590AA	10009	670
ESC_NA7589AA	152	257
ESC_NA7588AA	152	257
ESC_NA7587AA	152	257
ESC_NA7586AA	245	261
ESC_NA7585AA	245	261
ESC_NA7584AA	152	257
ESC_NA7583AA	245	261
ESC_NA7582AA	152	257
ESC_NA7581AA	152	257
ESC_NA7580AA	245	261
ESC_NA7579AA	152	257
ESC_NA7578AA	245	261
ESC_NA7577AA	152	257
ESC_NA7576AA	11	936
ESC_NA7575AA	659	286
ESC_NA7574AA	11	936
ESC_NA7573AA	127	738
ESC_NA7572AA	659	286
ESC_NA7571AA	442	257
ESC_NA7570AA	6130	268
ESC_NA7569AA	300	286
ESC_NA7568AA	173	254
ESC_NA7567AA	442	257
ESC_NA7566AA	1819	808
ESC_NA7565AA	21	156
ESC_NA7564AA	93	71
ESC_NA7563AA	11	936

ESC_NA7562AA	11	936
ESC_NA7561AA	11	936
ESC_NA7560AA	11	936
ESC_NA7559AA	398	445
ESC_NA7558AA	361	512
ESC_NA7557AA	21	156
ESC_NA7556AA	767	198
ESC_NA7555AA	10	512
ESC_NA7554AA	58	198
ESC_NA7553AA	10	512
ESC_NA7552AA	164	175
ESC_NA7551AA	10007	71
ESC_NA7550AA	1611	198
ESC_NA7549AA	1079	235
ESC_NA7548AA	164	175
ESC_NA7547AA	1079	235
ESC_NA7546AA	117	592
ESC_NA7545AA	270	204
ESC_NA7544AA	245	261
ESC_NA7543AA	245	261
ESC_NA7542AA	245	261
ESC_NA7541AA	11	936
ESC_NA7540AA	11	936
ESC_NA7539AA	423	204
ESC_NA7538AA	11	936
ESC_NA7537AA	11	936
ESC_NA7536AA	11	936
ESC_NA7535AA	11	936
ESC_NA7534AA	11	936
ESC_NA7533AA	11	936
ESC_NA7532AA	11	936
ESC_NA7531AA	11	936
ESC_NA7530AA	11	936
ESC_NA7529AA	11	936
ESC_NA7528AA	11	936
ESC_NA7526AA	10000	118
ESC_NA7525AA	2682	87
ESC_NA7524AA	10006	135
ESC_NA7523AA	10004	83
ESC_NA7522AA	10005	118
ESC_NA7521AA	10003	88
ESC_NA7520AA	2680	6
ESC_NA7519AA	2686	118
ESC_NA7518AA	10002	89
ESC_NA7517AA	10001	121
ESC_NA7516AA	7832	85
ESC_NA7515AA	2541	878
ESC_NA7514AA	2541	878
ESC_NA7513AA	2541	878
ESC_NA7512AA	58	198

ESC_NA7511AA	542	824
ESC_NA7510AA	2541	878
ESC_NA7509AA	3602	234
ESC_NA7508AA	2541	878
ESC_NA7507AA	392	234
ESC_NA7506AA	2541	878
ESC_NA7505AA	1079	235
ESC_NA7504AA	162	924
ESC_NA7503AA	345	175
ESC_NA7502AA	152	257
ESC_NA7501AA	152	257
ESC_NA7500AA	152	257
ESC_NA7499AA	152	257
ESC_NA7498AA	152	257
ESC_NA7497AA	245	261
ESC_NA7496AA	152	257
ESC_NA7495AA	245	261
ESC_NA7494AA	16	156
ESC_NA7493AA	1114	504
ESC_NA7492AA	9999	512
ESC_NA7491AA	1727	257
ESC_NA7490AA	40	198
ESC_NA7489AA	2964	912
ESC_NA7488AA	9347	170
ESC_NA7487AA	206	286
ESC_NA7486AA	10	512
ESC_NA7485AA	9998	117
ESC_NA7484AA	21	156
ESC_NA7483AA	21	156
ESC_NA7482AA	4198	878
ESC_NA7481AA	58	198
ESC_NA7480AA	1112	504
ESC_NA7479AA	8428	257
ESC_NA7478AA	131	653
ESC_NA7477AA	6643	198
ESC_NA7476AA	189	504
ESC_NA7475AA	1123	257
ESC_NA7474AA	871	447
ESC_NA7473AA	1125	198
ESC_NA7472AA	9997	789
ESC_NA7471AA	5914	257
ESC_NA7470AA	2556	630
ESC_NA7469AA	88	160
ESC_NA7468AA	8140	878
ESC_NA7467AA	540	286
ESC_NA7466AA	10	512
ESC_NA7465AA	3476	512
ESC_NA7464AA	58	198
ESC_NA7463AA	2005	175
ESC_NA7462AA	1730	234

ESC_NA7461AA	652	204
ESC_NA7460AA	10	512
ESC_NA7459AA	1126	198
ESC_NA7458AA	131	653
ESC_NA7457AA	300	286
ESC_NA7456AA	11	936
ESC_NA7455AA	306	286
ESC_NA7454AA	4942	36
ESC_NA7453AA	11	936
ESC_NA7452AA	21	156
ESC_NA7451AA	21	156
ESC_NA7450AA	9996	738
ESC_NA7449AA	95	738
ESC_NA7448AA	95	738
ESC_NA7447AA	95	738
ESC_NA7446AA	95	738
ESC_NA7445AA	95	738
ESC_NA7444AA	95	738
ESC_NA7443AA	95	738
ESC_NA7441AA	95	738
ESC_NA7440AA	95	738
ESC_NA7439AA	5559	257
ESC_NA7438AA	1967	191
ESC_NA7437AA	1967	191
ESC_NA7436AA	5559	257
ESC_NA7435AA	5559	257
ESC_NA7434AA	5559	257
ESC_NA7433AA	7100	924
ESC_NA7432AA	17	191
ESC_NA7431AA	156	175
ESC_NA7430AA	21	156
ESC_NA7429AA	5559	257
ESC_NA7428AA	9995	605
ESC_NA7427AA	29	156
ESC_NA7426AA	29	156
ESC_NA7425AA	29	156
ESC_NA7424AA	9994	595
ESC_NA7422AA	9992	824
ESC_NA7421AA	9406	512
ESC_NA7420AA	301	307
ESC_NA7419AA	11	936
ESC_NA7418AA	11	936
ESC_NA7416AA	21	156
ESC_NA7415AA	21	156
ESC_NA7414AA	21	156
ESC_NA7413AA	21	156
ESC_NA7412AA	21	156
ESC_NA7411AA	57	50
ESC_NA7410AA	744	512
ESC_NA7409AA	57	50

ESC_NA7408AA	57	50
ESC_NA7407AA	57	50
ESC_NA7406AA	744	512
ESC_NA7405AA	57	50
ESC_NA7404AA	744	512
ESC_NA7403AA	744	512
ESC_NA7402AA	744	512
ESC_NA7400AA	10	512
ESC_NA7399AA	11	936
ESC_NA7398AA	21	156
ESC_NA7397AA	5559	257
ESC_NA7396AA	21	156
ESC_NA7395AA	718	257
ESC_NA7394AA	11	936
ESC_NA7392AA	156	175
ESC_NA7391AA	156	175
ESC_NA7390AA	57	50
ESC_NA7389AA	744	512
ESC_NA7388AA	245	261
ESC_NA7387AA	243	197
ESC_NA7386AA	152	257
ESC_NA7385AA	245	261
ESC_NA7384AA	152	257
ESC_NA7383AA	245	261
ESC_NA7382AA	152	257
ESC_NA7381AA	152	257
ESC_NA7380AA	152	257
ESC_NA7379AA	152	257
ESC_NA7378AA	152	257
ESC_NA7377AA	152	257
ESC_NA7376AA	152	257
ESC_NA7375AA	152	257
ESC_NA7374AA	152	257
ESC_NA7373AA	152	257
ESC_NA7372AA	1594	71
ESC_NA7371AA	345	175
ESC_NA7370AA	11	936
ESC_NA7369AA	88	160
ESC_NA7368AA	11	936
ESC_NA7367AA	1276	241
ESC_NA7366AA	5995	504
ESC_NA7365AA	10	512
ESC_NA7364AA	69	387
ESC_NA7363AA	2079	198
ESC_NA7362AA	877	935
ESC_NA7361AA	641	935
ESC_NA7360AA	11	936
ESC_NA7359AA	21	156
ESC_NA7358AA	17	191
ESC_NA7357AA	17	191

ESC_NA7356AA	11	936
ESC_NA7355AA	23	156
ESC_NA7354AA	101	696
ESC_NA7353AA	11	936
ESC_NA7352AA	11	936
ESC_NA7351AA	17	191
ESC_NA7350AA	11	936
ESC_NA7349AA	11	936
ESC_NA7348AA	11	936
ESC_NA7347AA	10	512
ESC_NA7346AA	641	935
ESC_NA7345AA	1114	504
ESC_NA7343AA	1114	504
ESC_NA7342AA	57	50
ESC_NA7340AA	723	156
ESC_NA7337AA	165	504
ESC_NA7335AA	9991	257
ESC_NA7334AA	11	936
ESC_NA7333AA	5390	83
ESC_NA7332AA	11	936
ESC_NA7331AA	10	512
ESC_NA7330AA	10	512
ESC_NA7329AA	10	512
ESC_NA7328AA	10	512
ESC_NA7327AA	10	512
ESC_NA7326AA	10	512
ESC_NA7325AA	10	512
ESC_NA7324AA	10	512
ESC_NA7323AA	10	512
ESC_NA7322AA	10	512
ESC_NA7321AA	10	512
ESC_NA7320AA	10	512
ESC_NA7319AA	10	512
ESC_NA7318AA	10	512
ESC_NA7317AA	10	512
ESC_NA7316AA	10	512
ESC_NA7315AA	10	512
ESC_NA7314AA	10	512
ESC_NA7313AA	10	512
ESC_NA7312AA	10	512
ESC_NA7311AA	10	512
ESC_NA7310AA	10	512
ESC_NA7309AA	10	512
ESC_NA7308AA	10	512
ESC_NA7307AA	10	512
ESC_NA7306AA	10	512
ESC_NA7305AA	10	512
ESC_NA7304AA	10	512
ESC_NA7303AA	10	512
ESC_NA7302AA	10	512

ESC_NA7301AA	10	512
ESC_NA7300AA	10	512
ESC_NA7299AA	10	512
ESC_NA7298AA	10	512
ESC_NA7297AA	9993	512
ESC_NA7296AA	10	512
ESC_NA7295AA	10	512
ESC_NA7294AA	10	512
ESC_NA7293AA	10	512
ESC_NA7292AA	10	512
ESC_NA7291AA	10	512
ESC_NA7290AA	10	512
ESC_NA7289AA	10	512
ESC_NA7288AA	10	512
ESC_NA7287AA	10	512
ESC_NA7286AA	10	512
ESC_NA7285AA	10	512
ESC_NA7284AA	10	512
ESC_NA7283AA	10	512
ESC_NA7282AA	10	512
ESC_NA7281AA	10	512
ESC_NA7280AA	10	512
ESC_NA7279AA	10	512
ESC_NA7278AA	10	512
ESC_NA7277AA	10	512
ESC_NA7276AA	10	512
ESC_NA7275AA	10	512
ESC_NA7274AA	10	512
ESC_NA7273AA	10	512
ESC_NA7272AA	10	512
ESC_NA7271AA	10	512
ESC_NA7270AA	10	512
ESC_NA7269AA	10	512
ESC_NA7268AA	10	512
ESC_NA7266AA	10	512
ESC_NA7265AA	10	512
ESC_NA7264AA	10	512
ESC_NA7263AA	10	512
ESC_NA7262AA	10	512
ESC_NA7261AA	10	512
ESC_NA7260AA	10	512
ESC_NA7259AA	10	512
ESC_NA7258AA	10	512
ESC_NA7257AA	10	512
ESC_NA7256AA	10	512
ESC_NA7255AA	10	512
ESC_NA7254AA	10	512
ESC_NA7253AA	10	512
ESC_NA7252AA	10	512
ESC_NA7251AA	10	512

ESC_NA7250AA	10	512
ESC_NA7249AA	10	512
ESC_NA7248AA	10	512
ESC_NA7247AA	10	512
ESC_NA7246AA	10	512
ESC_NA7245AA	10	512
ESC_NA7244AA	10	512
ESC_NA7243AA	10	512
ESC_NA7242AA	10	512
ESC_NA7241AA	10	512
ESC_NA7240AA	10	512
ESC_NA7239AA	10	512
ESC_NA7238AA	10	512
ESC_NA7237AA	10	512
ESC_NA7236AA	10	512
ESC_NA7235AA	10	512
ESC_NA7234AA	10	512
ESC_NA7233AA	10	512
ESC_NA7232AA	10	512
ESC_NA7231AA	10	512
ESC_NA7230AA	10	512
ESC_NA7229AA	10	512
ESC_NA7228AA	10	512
ESC_NA7227AA	10	512
ESC_NA7226AA	10	512
ESC_NA7225AA	10	512
ESC_NA7224AA	10	512
ESC_NA7223AA	10	512
ESC_NA7222AA	10	512
ESC_NA7221AA	10	512
ESC_NA7220AA	10	512
ESC_NA7219AA	10	512
ESC_NA7218AA	10	512
ESC_NA7217AA	10	512
ESC_NA7216AA	10	512
ESC_NA7215AA	10	512
ESC_NA7214AA	10	512
ESC_NA7213AA	10	512
ESC_NA7212AA	10	512
ESC_NA7211AA	10	512
ESC_NA7210AA	10	512
ESC_NA7209AA	10	512
ESC_NA7208AA	10	512
ESC_NA7207AA	10	512
ESC_NA7206AA	10	512
ESC_NA7205AA	10	512
ESC_NA7204AA	10	512
ESC_NA7203AA	10	512
ESC_NA7202AA	10	512
ESC_NA7201AA	10	512

ESC_NA7200AA	10	512
ESC_NA7199AA	10	512
ESC_NA7198AA	10	512
ESC_NA7197AA	10	512
ESC_NA7196AA	10	512
ESC_NA7195AA	10	512
ESC_NA7194AA	10	512
ESC_NA7193AA	10	512
ESC_NA7192AA	10	512
ESC_NA7191AA	10	512
ESC_NA7190AA	10	512
ESC_NA7189AA	10	512
ESC_NA7188AA	10	512
ESC_NA7187AA	10	512
ESC_NA7186AA	10	512
ESC_NA7185AA	10	512
ESC_NA7184AA	10	512
ESC_NA7183AA	10	512
ESC_NA7182AA	10	512
ESC_NA7181AA	10	512
ESC_NA7180AA	10	512
ESC_NA7179AA	10	512
ESC_NA7178AA	10	512
ESC_NA7177AA	10	512
ESC_NA7176AA	10	512
ESC_NA7175AA	10	512
ESC_NA7174AA	10	512
ESC_NA7173AA	10	512
ESC_NA7172AA	10	512
ESC_NA7171AA	10	512
ESC_NA7170AA	10	512
ESC_NA7169AA	10	512
ESC_NA7168AA	10	512
ESC_NA7167AA	10	512
ESC_NA7166AA	10	512
ESC_NA7165AA	10	512
ESC_NA7164AA	10	512
ESC_NA7163AA	10	512
ESC_NA7162AA	10	512
ESC_NA7161AA	10	512
ESC_NA7160AA	10	512
ESC_NA7159AA	10	512
ESC_NA7158AA	10	512
ESC_NA7157AA	10	512
ESC_NA7156AA	10	512
ESC_NA7155AA	10	512
ESC_NA7154AA	10	512
ESC_NA7153AA	10	512
ESC_NA7152AA	10	512
ESC_NA7151AA	10	512

ESC_NA7150AA	10	512
ESC_NA7149AA	10	512
ESC_NA7148AA	10	512
ESC_NA7147AA	10	512
ESC_NA7146AA	10	512
ESC_NA7145AA	10	512
ESC_NA7144AA	10	512
ESC_NA7143AA	10	512
ESC_NA7142AA	10	512
ESC_NA7141AA	10	512
ESC_NA7140AA	10	512
ESC_NA7139AA	10	512
ESC_NA7138AA	10	512
ESC_NA7137AA	10	512
ESC_NA7136AA	10	512
ESC_NA7135AA	10	512
ESC_NA7134AA	10	512
ESC_NA7133AA	10	512
ESC_NA7132AA	10	512
ESC_NA7131AA	10	512
ESC_NA7130AA	10	512
ESC_NA7129AA	10	512
ESC_NA7128AA	11	936
ESC_NA7127AA	11	936
ESC_NA7126AA	11	936
ESC_NA7125AA	21	156
ESC_NA7124AA	11	936
ESC_NA7123AA	504	738
ESC_NA7122AA	10	512
ESC_NA7121AA	4060	744
ESC_NA7120AA	34	504
ESC_NA7119AA	5826	198
ESC_NA7118AA	16	156
ESC_NA7117AA	677	257
ESC_NA7116AA	301	307
ESC_NA7115AA	21	156
ESC_NA7114AA	33	198
ESC_NA7110AA	245	261
ESC_NA7109AA	1665	601
ESC_NA7108AA	48	76
ESC_NA7107AA	10	512
ESC_NA7106AA	152	257
ESC_NA7105AA	152	257
ESC_NA7104AA	245	261
ESC_NA7103AA	245	261
ESC_NA7102AA	245	261
ESC_NA7101AA	145	256
ESC_NA7100AA	245	261
ESC_NA7099AA	7039	260
ESC_NA7098AA	191	198

ESC_NA7097AA	345	175
ESC_NA7096AA	155	175
ESC_NA7095AA	6607	406
ESC_NA7094AA	224	198
ESC_NA7093AA	101	696
ESC_NA7092AA	657	592
ESC_NA7091AA	9989	406
ESC_NA7090AA	2690	76
ESC_NA7089AA	7507	504
ESC_NA7088AA	9990	447
ESC_NA7087AA	48	76
ESC_NA7086AA	616	156
ESC_NA7085AA	212	198
ESC_NA7084AA	9528	820
ESC_NA6985AA	38	669
ESC_NA6984AA	38	669
ESC_NA6983AA	38	669
ESC_NA6982AA	38	669
ESC_NA6981AA	38	669
ESC_NA6980AA	38	669
ESC_NA6979AA	38	669
ESC_NA6978AA	38	669
ESC_NA6977AA	38	669
ESC_NA6976AA	38	669
ESC_NA6975AA	38	669
ESC_NA6974AA	38	669
ESC_NA6973AA	38	669
ESC_NA6972AA	38	669
ESC_NA6971AA	38	669
ESC_NA6970AA	38	669
ESC_NA6969AA	38	669
ESC_NA6968AA	38	669
ESC_NA6967AA	38	669
ESC_NA6966AA	38	669
ESC_NA6965AA	38	669
ESC_NA6964AA	38	669
ESC_NA6963AA	38	669
ESC_NA6962AA	38	669
ESC_NA6961AA	38	669
ESC_NA6960AA	38	669
ESC_NA6959AA	38	669
ESC_NA6958AA	38	669
ESC_NA6957AA	38	669
ESC_NA6956AA	38	669
ESC_NA6955AA	38	669
ESC_NA6954AA	38	669
ESC_NA6953AA	38	669
ESC_NA6952AA	38	669
ESC_NA6951AA	38	669
ESC_NA6950AA	38	669

ESC_NA6949AA	38	669
ESC_NA6948AA	38	669
ESC_NA6947AA	38	669
ESC_NA6946AA	38	669
ESC_NA6945AA	38	669
ESC_NA6944AA	38	669
ESC_NA6943AA	38	669
ESC_NA6942AA	38	669
ESC_NA6941AA	38	669
ESC_NA6940AA	38	669
ESC_NA6939AA	38	669
ESC_NA6938AA	38	669
ESC_NA6937AA	38	669
ESC_NA6936AA	38	669
ESC_NA6935AA	38	669
ESC_NA6934AA	38	669
ESC_NA6933AA	38	669
ESC_NA6932AA	38	669
ESC_NA6931AA	38	669
ESC_NA6930AA	38	669
ESC_NA6929AA	38	669
ESC_NA6928AA	38	669
ESC_NA6927AA	38	669
ESC_NA6926AA	38	669
ESC_NA6925AA	38	669
ESC_NA6924AA	38	669
ESC_NA6923AA	38	669
ESC_NA6922AA	38	669
ESC_NA6921AA	38	669
ESC_NA6920AA	38	669
ESC_NA6919AA	38	669
ESC_NA6918AA	38	669
ESC_NA6917AA	38	669
ESC_NA6916AA	38	669
ESC_NA6915AA	38	669
ESC_NA6914AA	38	669
ESC_NA6913AA	38	669
ESC_NA6912AA	38	669
ESC_NA6911AA	38	669
ESC_NA6910AA	38	669
ESC_NA6909AA	38	669
ESC_NA6908AA	38	669
ESC_NA6907AA	38	669
ESC_NA6906AA	38	669
ESC_NA6905AA	38	669
ESC_NA6904AA	38	669
ESC_NA6903AA	38	669
ESC_NA6902AA	38	669
ESC_NA6901AA	38	669
ESC_NA6900AA	38	669

ESC_NA6899AA	38	669
ESC_NA6898AA	38	669
ESC_NA6897AA	38	669
ESC_NA6896AA	38	669
ESC_NA6895AA	38	669
ESC_NA6890AA	48	76
ESC_NA6889AA	617	511
ESC_NA6887AA	1114	504
ESC_NA6886AA	155	175
ESC_NA6885AA	9987	935
ESC_NA6884AA	9986	512
ESC_NA6883AA	1196	257
ESC_NA6882AA	297	234
ESC_NA6881AA	245	261
ESC_NA6880AA	152	257
ESC_NA6879AA	245	261
ESC_NA6878AA	145	256
ESC_NA6877AA	11	936
ESC_NA6876AA	11	936
ESC_NA6875AA	11	936
ESC_NA6874AA	11	936
ESC_NA6873AA	17	191
ESC_NA6872AA	11	936
ESC_NA6871AA	9959	307
ESC_NA6870AA	218	519
ESC_NA6869AA	101	696
ESC_NA6868AA	23	156
ESC_NA6867AA	10	512
ESC_NA6866AA	69	387
ESC_NA6865AA	906	198
ESC_NA6864AA	90	156
ESC_NA6863AA	5281	886
ESC_NA6862AA	43	512
ESC_NA6861AA	7588	447
ESC_NA6860AA	10	512
ESC_NA6859AA	10	512
ESC_NA6858AA	101	696
ESC_NA6857AA	3057	672
ESC_NA6856AA	34	504
ESC_NA6855AA	10	512
ESC_NA6854AA	1665	601
ESC_NA6853AA	25	234
ESC_NA6852AA	6602	692
ESC_NA6851AA	58	198
ESC_NA6850AA	1434	512
ESC_NA6849AA	23	156
ESC_NA6848AA	20	198
ESC_NA6847AA	388	156
ESC_NA6846AA	1011	198
ESC_NA6845AA	392	234

ESC_NA6844AA	6603	168
ESC_NA6843AA	661	92
ESC_NA6842AA	95	738
ESC_NA6841AA	58	198
ESC_NA6840AA	602	257
ESC_NA6839AA	95	738
ESC_NA6838AA	542	824
ESC_NA6837AA	10	512
ESC_NA6836AA	9962	684
ESC_NA6835AA	925	387
ESC_NA6834AA	58	198
ESC_NA6833AA	90	156
ESC_NA6832AA	9961	156
ESC_NA6831AA	6580	141
ESC_NA6830AA	2035	113
ESC_NA6829AA	453	935
ESC_NA6828AA	9960	204
ESC_NA6827AA	88	160
ESC_NA6826AA	10	512
ESC_NA6825AA	9826	519
ESC_NA6824AA	117	592
ESC_NA6823AA	501	900
ESC_NA6822AA	10	512
ESC_NA6821AA	763	878
ESC_NA6820AA	23	156
ESC_NA6819AA	17	191
ESC_NA6818AA	12	626
ESC_NA6817AA	12	626
ESC_NA6816AA	73	737
ESC_NA6815AA	1125	198
ESC_NA6814AA	101	696
ESC_NA6813AA	711	935
ESC_NA6812AA	4576	987
ESC_NA6811AA	90	156
ESC_NA6810AA	23	156
ESC_NA6809AA	10	512
ESC_NA6808AA	90	156
ESC_NA6807AA	10	512
ESC_NA6806AA	1079	235
ESC_NA6805AA	429	654
ESC_NA6804AA	1091	512
ESC_NA6803AA	10	512
ESC_NA6802AA	48	76
ESC_NA6801AA	90	156
ESC_NA6800AA	9963	318
ESC_NA6799AA	109	257
ESC_NA6798AA	327	204
ESC_NA6797AA	6542	261
ESC_NA6796AA	9965	445
ESC_NA6795AA	641	935

ESC_NA6794AA	1079	235
ESC_NA6793AA	106	387
ESC_NA6792AA	111	175
ESC_NA6791AA	1276	241
ESC_NA6790AA	58	198
ESC_NA6789AA	11	936
ESC_NA6788AA	131	653
ESC_NA6787AA	491	626
ESC_NA6786AA	2619	738
ESC_NA6785AA	555	653
ESC_NA6784AA	720	900
ESC_NA6783AA	101	696
ESC_NA6782AA	394	400
ESC_NA6781AA	69	387
ESC_NA6780AA	56	198
ESC_NA6779AA	1170	629
ESC_NA6778AA	4774	198
ESC_NA6777AA	2307	198
ESC_NA6776AA	3090	212
ESC_NA6775AA	88	160
ESC_NA6774AA	88	160
ESC_NA6773AA	12	626
ESC_NA6772AA	12	626
ESC_NA6771AA	12	626
ESC_NA6770AA	12	626
ESC_NA6769AA	127	738
ESC_NA6768AA	127	738
ESC_NA6767AA	117	592
ESC_NA6766AA	354	15
ESC_NA6765AA	101	696
ESC_NA6764AA	1081	198
ESC_NA6763AA	88	160
ESC_NA6762AA	1079	235
ESC_NA6761AA	4151	971
ESC_NA6760AA	88	160
ESC_NA6759AA	12	626
ESC_NA6758AA	12	626
ESC_NA6757AA	223	198
ESC_NA6756AA	10	512
ESC_NA6755AA	9964	1004
ESC_NA6754AA	73	737
ESC_NA6752AA	16	156
ESC_NA6751AA	10	512
ESC_NA6750AA	515	1004
ESC_NA6749AA	2	900
ESC_NA6748AA	196	257
ESC_NA6747AA	23	156
ESC_NA6746AA	95	738
ESC_NA6745AA	429	654
ESC_NA6744AA	2522	198

ESC_NA6743AA	88	160
ESC_NA6742AA	1125	198
ESC_NA6741AA	131	653
ESC_NA6740AA	131	653
ESC_NA6739AA	100	504
ESC_NA6738AA	429	654
ESC_NA6737AA	90	156
ESC_NA6736AA	95	738
ESC_NA6735AA	1967	191
ESC_NA6734AA	399	198
ESC_NA6733AA	6234	257
ESC_NA6732AA	224	198
ESC_NA6731AA	58	198
ESC_NA6730AA	140	738
ESC_NA6729AA	131	653
ESC_NA6728AA	718	257
ESC_NA6727AA	1679	160
ESC_NA6726AA	603	198
ESC_NA6725AA	99	198
ESC_NA6724AA	2944	597
ESC_NA6723AA	1286	474
ESC_NA6722AA	641	935
ESC_NA6721AA	5766	198
ESC_NA6720AA	10	512
ESC_NA6719AA	1992	257
ESC_NA6718AA	4656	76
ESC_NA6717AA	10	512
ESC_NA6716AA	5679	156
ESC_NA6715AA	1157	878
ESC_NA6714AA	1079	235
ESC_NA6713AA	20	198
ESC_NA6712AA	38	669
ESC_NA6711AA	2520	878
ESC_NA6710AA	2111	184
ESC_NA6709AA	10	512
ESC_NA6708AA	101	696
ESC_NA6707AA	973	968
ESC_NA6706AA	141	644
ESC_NA6705AA	23	156
ESC_NA6704AA	9967	257
ESC_NA6703AA	362	234
ESC_NA6702AA	602	257
ESC_NA6701AA	88	160
ESC_NA6700AA	329	355
ESC_NA6699AA	763	878
ESC_NA6698AA	90	156
ESC_NA6697AA	100	504
ESC_NA6696AA	23	156
ESC_NA6695AA	75	257
ESC_NA6694AA	2354	257

ESC_NA6693AA	115	669
ESC_NA6692AA	86	935
ESC_NA6691AA	9966	551
ESC_NA6690AA	4252	1004
ESC_NA6689AA	1406	174
ESC_NA6688AA	1771	868
ESC_NA6687AA	345	175
ESC_NA6686AA	58	198
ESC_NA6685AA	109	257
ESC_NA6684AA	10	512
ESC_NA6683AA	10	512
ESC_NA6682AA	10	512
ESC_NA6681AA	4151	971
ESC_NA6680AA	95	738
ESC_NA6679AA	9971	924
ESC_NA6678AA	32	270
ESC_NA6677AA	218	519
ESC_NA6676AA	165	504
ESC_NA6675AA	442	257
ESC_NA6674AA	350	50
ESC_NA6673AA	9970	76
ESC_NA6672AA	1178	512
ESC_NA6671AA	6594	257
ESC_NA6670AA	9969	900
ESC_NA6669AA	2325	447
ESC_NA6668AA	10	512
ESC_NA6667AA	154	257
ESC_NA6666AA	100	504
ESC_NA6665AA	58	198
ESC_NA6664AA	155	175
ESC_NA6663AA	127	738
ESC_NA6662AA	949	175
ESC_NA6661AA	131	653
ESC_NA6660AA	9968	156
ESC_NA6658AA	48	76
ESC_NA6657AA	162	924
ESC_NA6656AA	10	512
ESC_NA6655AA	54	256
ESC_NA6654AA	131	653
ESC_NA6653AA	345	175
ESC_NA6652AA	131	653
ESC_NA6651AA	162	924
ESC_NA6650AA	131	653
ESC_NA6649AA	10	512
ESC_NA6648AA	100	504
ESC_NA6647AA	196	257
ESC_NA6646AA	127	738
ESC_NA6645AA	706	422
ESC_NA6644AA	641	935
ESC_NA6643AA	1079	235

ESC_NA6642AA	453	935
ESC_NA6641AA	4516	445
ESC_NA6640AA	376	191
ESC_NA6639AA	162	924
ESC_NA6638AA	580	445
ESC_NA6637AA	95	738
ESC_NA6636AA	155	175
ESC_NA6635AA	90	156
ESC_NA6634AA	90	156
ESC_NA6633AA	973	968
ESC_NA6632AA	38	669
ESC_NA6631AA	86	935
ESC_NA6630AA	101	696
ESC_NA6629AA	88	160
ESC_NA6628AA	88	160
ESC_NA6627AA	100	504
ESC_NA6626AA	34	504
ESC_NA6625AA	58	198
ESC_NA6624AA	641	935
ESC_NA6623AA	101	696
ESC_NA6622AA	105	738
ESC_NA6621AA	961	623
ESC_NA6620AA	12	626
ESC_NA6619AA	131	653
ESC_NA6618AA	10	512
ESC_NA6617AA	1385	653
ESC_NA6616AA	10	512
ESC_NA6615AA	100	504
ESC_NA6614AA	12	626
ESC_NA6613AA	155	175
ESC_NA6612AA	9973	198
ESC_NA6611AA	394	400
ESC_NA6610AA	86	935
ESC_NA6609AA	1112	504
ESC_NA6608AA	162	924
ESC_NA6607AA	127	738
ESC_NA6606AA	101	696
ESC_NA6605AA	641	935
ESC_NA6604AA	1771	868
ESC_NA6603AA	9975	512
ESC_NA6602AA	9972	234
ESC_NA6601AA	58	198
ESC_NA6600AA	9974	160
ESC_NA6599AA	58	198
ESC_NA6598AA	410	156
ESC_NA6597AA	5236	257
ESC_NA6596AA	29	156
ESC_NA6595AA	2217	62
ESC_NA6594AA	2217	62
ESC_NA6593AA	302	850

ESC_NA6592AA	10	512
ESC_NA6591AA	23	156
ESC_NA6590AA	297	234
ESC_NA6589AA	641	935
ESC_NA6588AA	219	877
ESC_NA6587AA	106	387
ESC_NA6586AA	155	175
ESC_NA6585AA	43	512
ESC_NA6584AA	1081	198
ESC_NA6583AA	641	935
ESC_NA6582AA	1390	156
ESC_NA6581AA	10	512
ESC_NA6580AA	117	592
ESC_NA6579AA	58	198
ESC_NA6578AA	136	392
ESC_NA6577AA	5841	55
ESC_NA6576AA	405	923
ESC_NA6575AA	140	738
ESC_NA6574AA	9984	156
ESC_NA6573AA	297	234
ESC_NA6572AA	11	936
ESC_NA6571AA	2520	878
ESC_NA6570AA	10	512
ESC_NA6569AA	925	387
ESC_NA6568AA	135	653
ESC_NA6567AA	88	160
ESC_NA6566AA	162	924
ESC_NA6565AA	9979	235
ESC_NA6564AA	101	696
ESC_NA6563AA	350	50
ESC_NA6562AA	9983	512
ESC_NA6561AA	9982	198
ESC_NA6560AA	10	512
ESC_NA6559AA	155	175
ESC_NA6558AA	1423	198
ESC_NA6557AA	906	198
ESC_NA6556AA	1406	174
ESC_NA6555AA	446	257
ESC_NA6554AA	58	198
ESC_NA6553AA	99	198
ESC_NA6552AA	1148	257
ESC_NA6551AA	10	512
ESC_NA6550AA	1147	198
ESC_NA6549AA	1123	257
ESC_NA6548AA	1291	512
ESC_NA6547AA	100	504
ESC_NA6546AA	10	512
ESC_NA6545AA	1029	291
ESC_NA6544AA	88	160
ESC_NA6543AA	409	504

ESC_NA6542AA	21	156
ESC_NA6541AA	90	156
ESC_NA6540AA	21	156
ESC_NA6539AA	10	512
ESC_NA6538AA	48	76
ESC_NA6537AA	10	512
ESC_NA6536AA	127	738
ESC_NA6535AA	73	737
ESC_NA6534AA	9978	935
ESC_NA6533AA	795	924
ESC_NA6532AA	58	198
ESC_NA6531AA	998	644
ESC_NA6530AA	580	445
ESC_NA6529AA	641	935
ESC_NA6528AA	641	935
ESC_NA6527AA	155	175
ESC_NA6526AA	48	76
ESC_NA6525AA	43	512
ESC_NA6524AA	442	257
ESC_NA6523AA	162	924
ESC_NA6522AA	393	343
ESC_NA6521AA	141	644
ESC_NA6520AA	58	198
ESC_NA6519AA	1091	512
ESC_NA6518AA	6162	337
ESC_NA6517AA	4576	987
ESC_NA6516AA	1304	257
ESC_NA6515AA	1665	601
ESC_NA6514AA	73	737
ESC_NA6513AA	1083	203
ESC_NA6512AA	115	669
ESC_NA6511AA	56	198
ESC_NA6510AA	641	935
ESC_NA6509AA	83	740
ESC_NA6508AA	88	160
ESC_NA6507AA	9981	198
ESC_NA6506AA	906	198
ESC_NA6505AA	1056	257
ESC_NA6504AA	4541	676
ESC_NA6503AA	679	257
ESC_NA6502AA	1147	198
ESC_NA6501AA	9977	76
ESC_NA6500AA	88	160
ESC_NA6499AA	6542	261
ESC_NA6498AA	747	640
ESC_NA6497AA	731	285
ESC_NA6496AA	10	512
ESC_NA6495AA	227	504
ESC_NA6494AA	7893	198
ESC_NA6493AA	9976	156

ESC_NA6492AA	446	257
ESC_NA6491AA	155	175
ESC_NA6490AA	6580	141
ESC_NA6489AA	101	696
ESC_NA6488AA	2144	257
ESC_NA6487AA	2502	235
ESC_NA6486AA	372	424
ESC_NA6485AA	961	623
ESC_NA6484AA	961	623
ESC_NA6483AA	12	626
ESC_NA6482AA	501	900
ESC_NA6481AA	12	626
ESC_NA6480AA	12	626
ESC_NA6478AA	127	738
ESC_NA6476AA	88	160
ESC_NA6475AA	336	878
ESC_NA6474AA	23	156
ESC_NA6473AA	95	738
ESC_NA6472AA	500	71
ESC_NA6471AA	1431	257
ESC_NA6470AA	12	626
ESC_NA6469AA	12	626
ESC_NA6468AA	10	512
ESC_NA6467AA	129	257
ESC_NA6466AA	10	512
ESC_NA6465AA	484	71
ESC_NA6464AA	2230	126
ESC_NA6463AA	359	696
ESC_NA6462AA	542	824
ESC_NA6461AA	88	160
ESC_NA6460AA	127	738
ESC_NA6459AA	9980	247
ESC_NA6458AA	95	738
ESC_NA6457AA	69	387
ESC_NA6456AA	542	824
ESC_NA6455AA	90	156
ESC_NA6454AA	6365	696
ESC_NA6453AA	88	160
ESC_NA6452AA	88	160
ESC_NA6451AA	88	160
ESC_NA6450AA	448	257
ESC_NA6449AA	10	512
ESC_NA6448AA	4559	878
ESC_NA6447AA	925	387
ESC_NA6446AA	642	924
ESC_NA6445AA	543	842
ESC_NA6444AA	2532	257
ESC_NA6443AA	101	696
ESC_NA6442AA	641	935
ESC_NA6441AA	58	198

ESC_NA6440AA	90	156
ESC_NA6439AA	88	160
ESC_NA6438AA	10	512
ESC_NA6437AA	90	156
ESC_NA6436AA	10	512
ESC_NA6435AA	10	512
ESC_NA6434AA	73	737
ESC_NA6433AA	405	923
ESC_NA6432AA	929	742
ESC_NA6431AA	405	923
ESC_NA6430AA	106	387
ESC_NA6429AA	126	740
ESC_NA6428AA	2163	257
ESC_NA6426AA	388	156
ESC_NA6425AA	155	175
ESC_NA6424AA	6498	766
ESC_NA6423AA	216	512
ESC_NA6422AA	10	512
ESC_NA6421AA	10	512
ESC_NA6420AA	34	504
ESC_NA6419AA	9985	519
ESC_NA6418AA	88	160
ESC_NA6417AA	58	198
ESC_NA6416AA	278	924
ESC_NA6415AA	101	696
ESC_NA6414AA	10	512
ESC_NA6413AA	10	512
ESC_NA6412AA	6343	504
ESC_NA6411AA	10	512
ESC_NA6410AA	9988	512
ESC_NA6409AA	6740	447
ESC_NA6408AA	73	737
ESC_NA6407AA	345	175
ESC_NA6406AA	910	929
ESC_NA6405AA	23	156
ESC_NA6404AA	6603	168
ESC_NA6403AA	925	387
ESC_NA6402AA	95	738
ESC_NA6401AA	1679	160
ESC_NA6400AA	648	359
ESC_NA6399AA	20	198
ESC_NA6398AA	100	504
ESC_NA6397AA	88	160
ESC_NA6396AA	100	504
ESC_NA6395AA	23	156
ESC_NA6394AA	410	156
ESC_NA6393AA	947	257
ESC_NA6392AA	38	669
ESC_NA6391AA	641	935
ESC_NA6390AA	117	592

ESC_NA6389AA	56	198
ESC_NA6388AA	7616	257
ESC_NA6387AA	392	234
ESC_NA6386AA	73	737
ESC_NA6385AA	446	257
ESC_NA6384AA	13	257
ESC_NA6383AA	973	968
ESC_NA6382AA	711	935
ESC_NA6381AA	155	175
ESC_NA6380AA	109	257
ESC_NA6379AA	1483	257
ESC_NA6378AA	5221	198
ESC_NA6377AA	10	512
ESC_NA6376AA	10	512
ESC_NA6375AA	101	696
ESC_NA6374AA	109	257
ESC_NA6373AA	88	160
ESC_NA6372AA	10	512
ESC_NA6371AA	11	936
ESC_NA6370AA	73	737
ESC_NA6368AA	372	424
ESC_NA6367AA	1727	257
ESC_NA6366AA	11	936
ESC_NA6365AA	11	936
ESC_NA6364AA	11	936
ESC_NA6363AA	11	936
ESC_NA6362AA	11	936
ESC_NA6361AA	11	936
ESC_NA6360AA	6	445
ESC_NA6359AA	311	204
ESC_NA6358AA	99	198
ESC_NA6357AA	152	257
ESC_NA6356AA	152	257
ESC_NA6355AA	9933	936
ESC_NA6354AA	11	936
ESC_NA6353AA	11	936
ESC_NA6352AA	182	246
ESC_NA6351AA	152	257
ESC_NA6350AA	152	257
ESC_NA6349AA	301	307
ESC_NA6348AA	21	156
ESC_NA6347AA	21	156
ESC_NA6346AA	21	156
ESC_NA6345AA	738	598
ESC_NA6344AA	11	936
ESC_NA6343AA	11	936
ESC_NA6342AA	245	261
ESC_NA6341AA	152	257
ESC_NA6340AA	152	257
ESC_NA6339AA	245	261

ESC_NA6338AA	152	257
ESC_NA6337AA	152	257
ESC_NA6336AA	1025	261
ESC_NA6335AA	245	261
ESC_NA6334AA	152	257
ESC_NA6333AA	152	257
ESC_NA6332AA	245	261
ESC_NA6331AA	152	257
ESC_NA6330AA	245	261
ESC_NA6329AA	245	261
ESC_NA6328AA	245	261
ESC_NA6327AA	152	257
ESC_NA6326AA	152	257
ESC_NA6325AA	152	257
ESC_NA6324AA	245	261
ESC_NA6323AA	1025	261
ESC_NA6322AA	245	261
ESC_NA6321AA	245	261
ESC_NA6320AA	245	261
ESC_NA6319AA	9956	878
ESC_NA6318AA	5292	738
ESC_NA6317AA	6992	842
ESC_NA6316AA	2353	76
ESC_NA6315AA	10	512
ESC_NA6314AA	998	644
ESC_NA6313AA	9955	198
ESC_NA6312AA	659	286
ESC_NA6311AA	21	156
ESC_NA6310AA	17	191
ESC_NA6309AA	25	234
ESC_NA6308AA	11	936
ESC_NA6307AA	11	936
ESC_NA6306AA	11	936
ESC_NA6305AA	11	936
ESC_NA6304AA	25	234
ESC_NA6303AA	11	936
ESC_NA6302AA	442	257
ESC_NA6301AA	661	92
ESC_NA6300AA	366	198
ESC_NA6299AA	847	257
ESC_NA6298AA	23	156
ESC_NA6297AA	1415	504
ESC_NA6296AA	10	512
ESC_NA6295AA	770	587
ESC_NA6294AA	3891	830
ESC_NA6293AA	685	311
ESC_NA6292AA	328	924
ESC_NA6291AA	10	512
ESC_NA6290AA	9954	257
ESC_NA6289AA	48	76

ESC_NA6288AA	906	198
ESC_NA6287AA	58	198
ESC_NA6286AA	224	198
ESC_NA6285AA	109	257
ESC_NA6284AA	5973	92
ESC_NA6283AA	641	935
ESC_NA6282AA	442	257
ESC_NA6281AA	1510	4
ESC_NA6280AA	5912	156
ESC_NA6279AA	58	198
ESC_NA6278AA	5756	156
ESC_NA6277AA	10	512
ESC_NA6276AA	48	76
ESC_NA6275AA	1252	567
ESC_NA6258AA	11	936
ESC_NA6256AA	16	156
ESC_NA6255AA	1114	504
ESC_NA6254AA	1125	198
ESC_NA6253AA	1114	504
ESC_NA6251AA	1727	257
ESC_NA6250AA	329	355
ESC_NA6248AA	1114	504
ESC_NA6247AA	11	936
ESC_NA6246AA	11	936
ESC_NA6245AA	88	160
ESC_NA6244AA	329	355
ESC_NA6243AA	11	936
ESC_NA6242AA	219	877
ESC_NA6241AA	9957	90
ESC_NA6240AA	1817	842
ESC_NA6239AA	12	626
ESC_NA6238AA	442	257
ESC_NA6237AA	11	936
ESC_NA6236AA	95	738
ESC_NA6235AA	278	924
ESC_NA6234AA	11	936
ESC_NA6233AA	11	936
ESC_NA6232AA	11	936
ESC_NA6231AA	11	936
ESC_NA6230AA	11	936
ESC_NA6229AA	11	936
ESC_NA6228AA	11	936
ESC_NA6227AA	11	936
ESC_NA6226AA	11	936
ESC_NA6225AA	11	936
ESC_NA6224AA	11	936
ESC_NA6223AA	11	936
ESC_NA6222AA	11	936
ESC_NA6221AA	11	936
ESC_NA6220AA	11	936

ESC_NA6219AA	11	936
ESC_NA6218AA	11	936
ESC_NA6217AA	11	936
ESC_NA6216AA	11	936
ESC_NA6215AA	11	936
ESC_NA6214AA	11	936
ESC_NA6213AA	11	936
ESC_NA6212AA	11	936
ESC_NA6211AA	11	936
ESC_NA6210AA	11	936
ESC_NA6209AA	11	936
ESC_NA6208AA	11	936
ESC_NA6207AA	11	936
ESC_NA6206AA	11	936
ESC_NA6205AA	11	936
ESC_NA6204AA	11	936
ESC_NA6203AA	11	936
ESC_NA6202AA	11	936
ESC_NA6201AA	1112	504
ESC_NA6200AA	11	936
ESC_NA6199AA	11	936
ESC_NA6198AA	11	936
ESC_NA6197AA	11	936
ESC_NA6196AA	11	936
ESC_NA6195AA	11	936
ESC_NA6194AA	11	936
ESC_NA6193AA	11	936
ESC_NA6192AA	11	936
ESC_NA6191AA	11	936
ESC_NA6190AA	11	936
ESC_NA6189AA	11	936
ESC_NA6188AA	11	936
ESC_NA6187AA	11	936
ESC_NA6186AA	11	936
ESC_NA6185AA	11	936
ESC_NA6184AA	11	936
ESC_NA6183AA	11	936
ESC_NA6182AA	11	936
ESC_NA6181AA	9953	232
ESC_NA6179AA	58	198
ESC_NA6178AA	131	653
ESC_NA6177AA	131	653
ESC_NA6176AA	9213	653
ESC_NA6175AA	636	653
ESC_NA6174AA	131	653
ESC_NA6173AA	131	653
ESC_NA6172AA	131	653
ESC_NA6171AA	131	653
ESC_NA6170AA	131	653
ESC_NA6169AA	38	669

ESC_NA6167AA	101	696
ESC_NA6166AA	101	696
ESC_NA6165AA	101	696
ESC_NA6164AA	101	696
ESC_NA6163AA	101	696
ESC_NA6162AA	101	696
ESC_NA6161AA	21	156
ESC_NA6160AA	17	191
ESC_NA6159AA	16	156
ESC_NA6158AA	3268	669
ESC_NA6157AA	101	696
ESC_NA6156AA	9943	512
ESC_NA6155AA	9940	592
ESC_NA6154AA	9944	175
ESC_NA6153AA	9945	512
ESC_NA6152AA	9938	592
ESC_NA6151AA	602	257
ESC_NA6150AA	9941	175
ESC_NA6149AA	9942	359
ESC_NA6148AA	9939	640
ESC_NA6147AA	9937	153
ESC_NA6146AA	9937	153
ESC_NA6145AA	131	653
ESC_NA6139AA	152	257
ESC_NA6138AA	245	261
ESC_NA6137AA	245	261
ESC_NA6136AA	152	257
ESC_NA6135AA	152	257
ESC_NA6134AA	152	257
ESC_NA6133AA	9946	512
ESC_NA6132AA	16	156
ESC_NA6131AA	21	156
ESC_NA6130AA	120	180
ESC_NA6129AA	11	936
ESC_NA6128AA	40	198
ESC_NA6127AA	200	198
ESC_NA6126AA	6879	850
ESC_NA6125AA	1380	914
ESC_NA6124AA	659	286
ESC_NA6123AA	10	512
ESC_NA6122AA	1147	198
ESC_NA6121AA	152	257
ESC_NA6120AA	152	257
ESC_NA6119AA	152	257
ESC_NA6118AA	4059	204
ESC_NA6117AA	718	257
ESC_NA6116AA	17	191
ESC_NA6115AA	998	644
ESC_NA6114AA	607	355
ESC_NA6113AA	9935	587

ESC_NA6112AA	355	764
ESC_NA6111AA	355	764
ESC_NA6110AA	906	198
ESC_NA6109AA	48	76
ESC_NA6108AA	9934	924
ESC_NA6107AA	43	512
ESC_NA6106AA	1304	257
ESC_NA6105AA	48	76
ESC_NA6104AA	10	512
ESC_NA6103AA	131	653
ESC_NA6102AA	616	156
ESC_NA6101AA	58	198
ESC_NA6100AA	13	257
ESC_NA6099AA	115	669
ESC_NA6098AA	1679	160
ESC_NA6097AA	1434	512
ESC_NA6096AA	2005	175
ESC_NA6095AA	3594	924
ESC_NA6094AA	58	198
ESC_NA6093AA	58	198
ESC_NA6092AA	352	744
ESC_NA6091AA	392	234
ESC_NA6090AA	10	512
ESC_NA6089AA	23	156
ESC_NA6088AA	206	286
ESC_NA6087AA	1308	257
ESC_NA6086AA	906	198
ESC_NA6085AA	10	512
ESC_NA6084AA	38	669
ESC_NA6083AA	442	257
ESC_NA6082AA	101	696
ESC_NA6081AA	223	198
ESC_NA6080AA	9936	257
ESC_NA6079AA	131	653
ESC_NA6078AA	297	234
ESC_NA6077AA	399	198
ESC_NA6076AA	8608	512
ESC_NA6075AA	770	587
ESC_NA6074AA	117	592
ESC_NA6073AA	117	592
ESC_NA6072AA	441	198
ESC_NA6071AA	906	198
ESC_NA6070AA	355	764
ESC_NA6069AA	117	592
ESC_NA6068AA	1236	380
ESC_NA6067AA	8588	257
ESC_NA6066AA	3580	257
ESC_NA6065AA	155	175
ESC_NA6064AA	428	654
ESC_NA6063AA	5940	555

ESC_NA6062AA	9866	512
ESC_NA6061AA	17	191
ESC_NA6060AA	9598	76
ESC_NA6059AA	187	257
ESC_NA6058AA	6853	71
ESC_NA6057AA	1429	198
ESC_NA6056AA	1081	198
ESC_NA6055AA	929	742
ESC_NA6054AA	101	696
ESC_NA6053AA	1684	504
ESC_NA6052AA	2175	212
ESC_NA6051AA	1079	235
ESC_NA6050AA	2556	630
ESC_NA6049AA	1316	471
ESC_NA6048AA	1434	512
ESC_NA6047AA	117	592
ESC_NA6046AA	1611	198
ESC_NA6045AA	919	421
ESC_NA6044AA	1611	198
ESC_NA6043AA	2144	257
ESC_NA6042AA	10	512
ESC_NA6041AA	117	592
ESC_NA6040AA	345	175
ESC_NA6039AA	9711	562
ESC_NA6038AA	135	653
ESC_NA6037AA	978	740
ESC_NA6036AA	4162	180
ESC_NA6035AA	40	198
ESC_NA6034AA	9933	936
ESC_NA6033AA	11	936
ESC_NA6032AA	1060	512
ESC_NA6031AA	1060	512
ESC_NA6030AA	1060	512
ESC_NA6029AA	1060	512
ESC_NA6028AA	1060	512
ESC_NA6027AA	1060	512
ESC_NA6026AA	1060	512
ESC_NA6025AA	1060	512
ESC_NA6024AA	1060	512
ESC_NA6023AA	1060	512
ESC_NA6022AA	1060	512
ESC_NA6021AA	1060	512
ESC_NA6020AA	1060	512
ESC_NA6019AA	1060	512
ESC_NA6018AA	1060	512
ESC_NA6017AA	1060	512
ESC_NA6016AA	101	696
ESC_NA6015AA	9893	696
ESC_NA6014AA	9894	175
ESC_NA6013AA	38	669

ESC_NA6012AA	9894	175
ESC_NA6011AA	9894	175
ESC_NA6010AA	9893	696
ESC_NA6009AA	9893	696
ESC_NA6008AA	101	696
ESC_NA6007AA	9893	696
ESC_NA6005AA	9894	175
ESC_NA6004AA	9894	175
ESC_NA6003AA	2466	307
ESC_NA6002AA	301	307
ESC_NA6001AA	11	936
ESC_NA6000AA	2466	307
ESC_NA5999AA	152	257
ESC_NA5998AA	152	257
ESC_NA5997AA	152	257
ESC_NA5996AA	152	257
ESC_NA5995AA	152	257
ESC_NA5993AA	145	256
ESC_NA5992AA	152	257
ESC_NA5991AA	5390	83
ESC_NA5990AA	10	512
ESC_NA5989AA	11	936
ESC_NA5988AA	11	936
ESC_NA5987AA	636	653
ESC_NA5986AA	450	71
ESC_NA5985AA	216	512
ESC_NA5984AA	207	512
ESC_NA5983AA	215	512
ESC_NA5982AA	10	512
ESC_NA5981AA	10	512
ESC_NA5980AA	152	257
ESC_NA5979AA	17	191
ESC_NA5978AA	17	191
ESC_NA5977AA	17	191
ESC_NA5976AA	21	156
ESC_NA5975AA	10	512
ESC_NA5974AA	9923	935
ESC_NA5973AA	17	191
ESC_NA5972AA	11	936
ESC_NA5971AA	11	936
ESC_NA5970AA	17	191
ESC_NA5969AA	5736	868
ESC_NA5968AA	119	286
ESC_NA5967AA	17	191
ESC_NA5966AA	16	156
ESC_NA5965AA	223	198
ESC_NA5964AA	21	156
ESC_NA5963AA	21	156
ESC_NA5962AA	167	512
ESC_NA5961AA	410	156

ESC_NA5960AA	131	653
ESC_NA5959AA	405	923
ESC_NA5958AA	405	923
ESC_NA5957AA	405	923
ESC_NA5956AA	354	15
ESC_NA5955AA	4450	844
ESC_NA5954AA	10	512
ESC_NA5953AA	4108	156
ESC_NA5952AA	227	504
ESC_NA5951AA	410	156
ESC_NA5950AA	401	142
ESC_NA5948AA	359	696
ESC_NA5947AA	648	359
ESC_NA5946AA	1702	512
ESC_NA5945AA	167	512
ESC_NA5944AA	354	15
ESC_NA5943AA	410	156
ESC_NA5942AA	448	257
ESC_NA5941AA	156	175
ESC_NA5940AA	38	669
ESC_NA5939AA	963	669
ESC_NA5938AA	410	156
ESC_NA5937AA	224	198
ESC_NA5936AA	69	387
ESC_NA5935AA	940	257
ESC_NA5934AA	648	359
ESC_NA5933AA	354	15
ESC_NA5932AA	156	175
ESC_NA5931AA	405	923
ESC_NA5930AA	405	923
ESC_NA5929AA	155	175
ESC_NA5928AA	354	15
ESC_NA5927AA	448	257
ESC_NA5926AA	457	595
ESC_NA5925AA	410	156
ESC_NA5924AA	38	669
ESC_NA5923AA	410	156
ESC_NA5922AA	8453	512
ESC_NA5921AA	9922	156
ESC_NA5920AA	156	175
ESC_NA5919AA	410	156
ESC_NA5918AA	410	156
ESC_NA5917AA	410	156
ESC_NA5916AA	9921	644
ESC_NA5915AA	38	669
ESC_NA5914AA	95	738
ESC_NA5913AA	73	737
ESC_NA5912AA	410	156
ESC_NA5911AA	10	512
ESC_NA5910AA	354	15

ESC_NA5909AA	224	198
ESC_NA5908AA	405	923
ESC_NA5907AA	394	400
ESC_NA5906AA	410	156
ESC_NA5905AA	617	511
ESC_NA5904AA	349	670
ESC_NA5903AA	58	198
ESC_NA5902AA	410	156
ESC_NA5901AA	648	359
ESC_NA5900AA	189	504
ESC_NA5899AA	205	257
ESC_NA5898AA	963	669
ESC_NA5897AA	410	156
ESC_NA5896AA	10	512
ESC_NA5895AA	69	387
ESC_NA5894AA	648	359
ESC_NA5893AA	448	257
ESC_NA5892AA	405	923
ESC_NA5891AA	648	359
ESC_NA5890AA	154	257
ESC_NA5889AA	361	512
ESC_NA5888AA	1434	512
ESC_NA5887AA	127	738
ESC_NA5886AA	648	359
ESC_NA5885AA	17	191
ESC_NA5884AA	17	191
ESC_NA5883AA	718	257
ESC_NA5882AA	11	936
ESC_NA5881AA	906	198
ESC_NA5880AA	11	936
ESC_NA5879AA	16	156
ESC_NA5878AA	16	156
ESC_NA5877AA	16	156
ESC_NA5876AA	21	156
ESC_NA5875AA	21	156
ESC_NA5874AA	21	156
ESC_NA5873AA	723	156
ESC_NA5872AA	21	156
ESC_NA5871AA	17	191
ESC_NA5870AA	1721	311
ESC_NA5869AA	9317	257
ESC_NA5868AA	9317	257
ESC_NA5867AA	11	936
ESC_NA5866AA	16	156
ESC_NA5865AA	32	270
ESC_NA5864AA	17	191
ESC_NA5863AA	11	936
ESC_NA5862AA	424	234
ESC_NA5861AA	17	191
ESC_NA5860AA	21	156

ESC_NA5859AA	11	936
ESC_NA5858AA	8677	483
ESC_NA5857AA	245	261
ESC_NA5856AA	152	257
ESC_NA5855AA	152	257
ESC_NA5854AA	245	261
ESC_NA5853AA	152	257
ESC_NA5852AA	152	257
ESC_NA5851AA	1819	808
ESC_NA5850AA	5292	738
ESC_NA5849AA	11	936
ESC_NA5848AA	3101	160
ESC_NA5847AA	9920	156
ESC_NA5846AA	11	936
ESC_NA5844AA	442	257
ESC_NA5843AA	33	198
ESC_NA5842AA	25	234
ESC_NA5841AA	17	191
ESC_NA5840AA	5292	738
ESC_NA5839AA	21	156
ESC_NA5838AA	3202	71
ESC_NA5837AA	152	257
ESC_NA5836AA	10	512
ESC_NA5835AA	9287	732
ESC_NA5834AA	1616	924
ESC_NA5833AA	9287	732
ESC_NA5832AA	10	512
ESC_NA5831AA	540	286
ESC_NA5830AA	58	198
ESC_NA5829AA	10	512
ESC_NA5828AA	8763	79
ESC_NA5827AA	11	936
ESC_NA5826AA	342	286
ESC_NA5825AA	17	191
ESC_NA5824AA	17	191
ESC_NA5823AA	5310	156
ESC_NA5822AA	11	936
ESC_NA5821AA	17	191
ESC_NA5820AA	343	286
ESC_NA5819AA	16	156
ESC_NA5818AA	609	447
ESC_NA5817AA	165	504
ESC_NA5816AA	394	400
ESC_NA5815AA	165	504
ESC_NA5814AA	7039	260
ESC_NA5813AA	2456	71
ESC_NA5812AA	9897	286
ESC_NA5811AA	361	512
ESC_NA5810AA	245	261
ESC_NA5809AA	245	261

ESC_NA5808AA	245	261
ESC_NA5807AA	152	257
ESC_NA5806AA	152	257
ESC_NA5805AA	152	257
ESC_NA5804AA	245	261
ESC_NA5803AA	245	261
ESC_NA5802AA	11	936
ESC_NA5801AA	16	156
ESC_NA5800AA	11	936
ESC_NA5799AA	5736	868
ESC_NA5798AA	21	156
ESC_NA5797AA	342	286
ESC_NA5796AA	21	156
ESC_NA5795AA	21	156
ESC_NA5794AA	101	696
ESC_NA5793AA	17	191
ESC_NA5792AA	21	156
ESC_NA5791AA	21	156
ESC_NA5790AA	306	286
ESC_NA5789AA	16	156
ESC_NA5788AA	11	936
ESC_NA5787AA	21	156
ESC_NA5786AA	21	156
ESC_NA5785AA	17	191
ESC_NA5784AA	21	156
ESC_NA5783AA	17	191
ESC_NA5782AA	32	270
ESC_NA5781AA	21	156
ESC_NA5780AA	21	156
ESC_NA5779AA	11	936
ESC_NA5778AA	17	191
ESC_NA5777AA	17	191
ESC_NA5776AA	1967	191
ESC_NA5775AA	723	156
ESC_NA5774AA	16	156
ESC_NA5773AA	655	926
ESC_NA5772AA	21	156
ESC_NA5771AA	11	936
ESC_NA5770AA	11	936
ESC_NA5769AA	11	936
ESC_NA5768AA	21	156
ESC_NA5767AA	32	270
ESC_NA5766AA	747	640
ESC_NA5765AA	1967	191
ESC_NA5764AA	32	270
ESC_NA5763AA	11	936
ESC_NA5762AA	21	156
ESC_NA5761AA	1431	257
ESC_NA5760AA	21	156
ESC_NA5759AA	21	156

ESC_NA5758AA	655	926
ESC_NA5757AA	295	160
ESC_NA5756AA	11	936
ESC_NA5755AA	17	191
ESC_NA5754AA	10	512
ESC_NA5753AA	69	387
ESC_NA5752AA	349	670
ESC_NA5751AA	372	424
ESC_NA5750AA	9919	942
ESC_NA5749AA	83	740
ESC_NA5748AA	141	644
ESC_NA5747AA	457	595
ESC_NA5746AA	681	393
ESC_NA5744AA	9894	175
ESC_NA5742AA	9895	935
ESC_NA5740AA	9894	175
ESC_NA5739AA	9893	696
ESC_NA5738AA	9893	696
ESC_NA5737AA	245	261
ESC_NA5736AA	245	261
ESC_NA5735AA	152	257
ESC_NA5734AA	152	257
ESC_NA5733AA	245	261
ESC_NA5732AA	152	257
ESC_NA5731AA	245	261
ESC_NA5730AA	152	257
ESC_NA5729AA	245	261
ESC_NA5728AA	245	261
ESC_NA5727AA	152	257
ESC_NA5726AA	2064	261
ESC_NA5725AA	245	261
ESC_NA5724AA	245	261
ESC_NA5723AA	245	261
ESC_NA5722AA	245	261
ESC_NA5721AA	152	257
ESC_NA5720AA	245	261
ESC_NA5719AA	152	257
ESC_NA5718AA	152	257
ESC_NA5717AA	245	261
ESC_NA5716AA	11	936
ESC_NA5715AA	11	936
ESC_NA5714AA	21	156
ESC_NA5713AA	182	246
ESC_NA5712AA	9918	653
ESC_NA5711AA	1136	108
ESC_NA5710AA	11	936
ESC_NA5709AA	11	936
ESC_NA5708AA	11	936
ESC_NA5707AA	11	936
ESC_NA5706AA	11	936

ESC_NA5705AA	156	175
ESC_NA5704AA	11	936
ESC_NA5703AA	11	936
ESC_NA5702AA	11	936
ESC_NA5701AA	1573	156
ESC_NA5700AA	11	936
ESC_NA5699AA	56	198
ESC_NA5698AA	88	160
ESC_NA5697AA	46	447
ESC_NA5696AA	1091	512
ESC_NA5695AA	10	512
ESC_NA5694AA	1602	817
ESC_NA5691AA	29	156
ESC_NA5690AA	29	156
ESC_NA5689AA	29	156
ESC_NA5688AA	1196	257
ESC_NA5687AA	245	261
ESC_NA5686AA	152	257
ESC_NA5685AA	245	261
ESC_NA5684AA	245	261
ESC_NA5683AA	10	512
ESC_NA5682AA	2077	198
ESC_NA5681AA	1564	512
ESC_NA5680AA	58	198
ESC_NA5679AA	1771	868
ESC_NA5678AA	9917	307
ESC_NA5677AA	21	156
ESC_NA5676AA	11	936
ESC_NA5675AA	679	257
ESC_NA5674AA	80	731
ESC_NA5673AA	223	198
ESC_NA5672AA	21	156
ESC_NA5671AA	11	936
ESC_NA5670AA	16	156
ESC_NA5669AA	17	191
ESC_NA5668AA	21	156
ESC_NA5667AA	141	644
ESC_NA5666AA	643	738
ESC_NA5665AA	10	512
ESC_NA5664AA	73	737
ESC_NA5663AA	723	156
ESC_NA5662AA	21	156
ESC_NA5661AA	21	156
ESC_NA5660AA	4173	234
ESC_NA5659AA	7961	592
ESC_NA5658AA	21	156
ESC_NA5657AA	21	156
ESC_NA5656AA	21	156
ESC_NA5655AA	16	156
ESC_NA5654AA	32	270

ESC_NA5653AA	21	156
ESC_NA5652AA	5732	156
ESC_NA5651AA	659	286
ESC_NA5650AA	11	936
ESC_NA5649AA	16	156
ESC_NA5648AA	5732	156
ESC_NA5647AA	11	936
ESC_NA5646AA	21	156
ESC_NA5645AA	17	191
ESC_NA5644AA	16	156
ESC_NA5643AA	11	936
ESC_NA5642AA	11	936
ESC_NA5641AA	21	156
ESC_NA5640AA	21	156
ESC_NA5639AA	655	926
ESC_NA5638AA	17	191
ESC_NA5637AA	16	156
ESC_NA5636AA	542	824
ESC_NA5635AA	10	512
ESC_NA5634AA	155	175
ESC_NA5633AA	327	204
ESC_NA5632AA	1308	257
ESC_NA5631AA	746	789
ESC_NA5630AA	58	198
ESC_NA5629AA	278	924
ESC_NA5628AA	58	198
ESC_NA5627AA	152	257
ESC_NA5626AA	32	270
ESC_NA5625AA	11	936
ESC_NA5624AA	7431	936
ESC_NA5623AA	11	936
ESC_NA5622AA	21	156
ESC_NA5621AA	32	270
ESC_NA5620AA	11	936
ESC_NA5619AA	11	936
ESC_NA5618AA	10	512
ESC_NA5617AA	10	512
ESC_NA5616AA	90	156
ESC_NA5615AA	6901	960
ESC_NA5614AA	88	160
ESC_NA5612AA	10	512
ESC_NA5611AA	10	512
ESC_NA5610AA	540	286
ESC_NA5609AA	540	286
ESC_NA5608AA	744	512
ESC_NA5607AA	744	512
ESC_NA5606AA	245	261
ESC_NA5605AA	152	257
ESC_NA5604AA	152	257
ESC_NA5603AA	11	936

ESC_NA5602AA	21	156
ESC_NA5601AA	343	286
ESC_NA5600AA	655	926
ESC_NA5599AA	342	286
ESC_NA5598AA	17	191
ESC_NA5597AA	11	936
ESC_NA5596AA	32	270
ESC_NA5595AA	17	191
ESC_NA5594AA	5681	286
ESC_NA5593AA	11	936
ESC_NA5592AA	16	156
ESC_NA5591AA	11	936
ESC_NA5590AA	17	191
ESC_NA5589AA	11	936
ESC_NA5588AA	10	512
ESC_NA5587AA	11	936
ESC_NA5586AA	21	156
ESC_NA5585AA	306	286
ESC_NA5584AA	11	936
ESC_NA5583AA	1041	765
ESC_NA5582AA	11	936
ESC_NA5581AA	17	191
ESC_NA5580AA	152	257
ESC_NA5579AA	152	257
ESC_NA5578AA	245	261
ESC_NA5577AA	152	257
ESC_NA5576AA	245	261
ESC_NA5575AA	152	257
ESC_NA5574AA	152	257
ESC_NA5573AA	152	257
ESC_NA5572AA	245	261
ESC_NA5571AA	245	261
ESC_NA5570AA	152	257
ESC_NA5569AA	152	257
ESC_NA5568AA	245	261
ESC_NA5567AA	152	257
ESC_NA5566AA	17	191
ESC_NA5565AA	16	156
ESC_NA5564AA	21	156
ESC_NA5563AA	1792	156
ESC_NA5562AA	342	286
ESC_NA5561AA	17	191
ESC_NA5560AA	17	191
ESC_NA5559AA	21	156
ESC_NA5558AA	17	191
ESC_NA5557AA	16	156
ESC_NA5556AA	17	191
ESC_NA5555AA	17	191
ESC_NA5554AA	17	191
ESC_NA5553AA	11	936

ESC_NA5552AA	21	156
ESC_NA5551AA	17	191
ESC_NA5550AA	11	936
ESC_NA5549AA	655	926
ESC_NA5548AA	11	936
ESC_NA5547AA	17	191
ESC_NA5546AA	33	198
ESC_NA5545AA	9892	257
ESC_NA5544AA	9892	257
ESC_NA5543AA	9892	257
ESC_NA5542AA	9892	257
ESC_NA5541AA	655	926
ESC_NA5540AA	655	926
ESC_NA5539AA	9892	257
ESC_NA5538AA	655	926
ESC_NA5537AA	11	936
ESC_NA5536AA	17	191
ESC_NA5535AA	7545	191
ESC_NA5534AA	655	926
ESC_NA5533AA	21	156
ESC_NA5532AA	9892	257
ESC_NA5531AA	446	257
ESC_NA5530AA	655	926
ESC_NA5529AA	6879	850
ESC_NA5528AA	17	191
ESC_NA5527AA	17	191
ESC_NA5526AA	16	156
ESC_NA5525AA	655	926
ESC_NA5524AA	21	156
ESC_NA5523AA	17	191
ESC_NA5522AA	17	191
ESC_NA5521AA	21	156
ESC_NA5520AA	21	156
ESC_NA5519AA	17	191
ESC_NA5518AA	11	936
ESC_NA5517AA	4942	36
ESC_NA5516AA	17	191
ESC_NA5515AA	16	156
ESC_NA5514AA	11	936
ESC_NA5513AA	16	156
ESC_NA5512AA	11	936
ESC_NA5511AA	16	156
ESC_NA5510AA	21	156
ESC_NA5509AA	16	156
ESC_NA5508AA	11	936
ESC_NA5507AA	16	156
ESC_NA5506AA	16	156
ESC_NA5505AA	11	936
ESC_NA5504AA	655	926
ESC_NA5503AA	11	936

ESC_NA5502AA	655	926
ESC_NA5501AA	16	156
ESC_NA5500AA	7237	191
ESC_NA5499AA	11	936
ESC_NA5498AA	10	512
ESC_NA5497AA	533	198
ESC_NA5496AA	4545	808
ESC_NA5495AA	1463	263
ESC_NA5494AA	16	156
ESC_NA5493AA	1437	512
ESC_NA5492AA	10	512
ESC_NA5491AA	4545	808
ESC_NA5490AA	12	626
ESC_NA5489AA	3014	410
ESC_NA5488AA	6365	696
ESC_NA5487AA	10	512
ESC_NA5486AA	10	512
ESC_NA5485AA	3326	886
ESC_NA5484AA	4545	808
ESC_NA5483AA	641	935
ESC_NA5482AA	372	424
ESC_NA5481AA	68	386
ESC_NA5480AA	10	512
ESC_NA5479AA	10	512
ESC_NA5478AA	10	512
ESC_NA5477AA	11	936
ESC_NA5476AA	17	191
ESC_NA5475AA	342	286
ESC_NA5474AA	11	936
ESC_NA5473AA	11	936
ESC_NA5472AA	17	191
ESC_NA5471AA	11	936
ESC_NA5470AA	245	261
ESC_NA5469AA	245	261
ESC_NA5468AA	5312	235
ESC_NA5467AA	245	261
ESC_NA5466AA	245	261
ESC_NA5465AA	245	261
ESC_NA5464AA	145	256
ESC_NA5463AA	245	261
ESC_NA5462AA	152	257
ESC_NA5461AA	152	257
ESC_NA5460AA	152	257
ESC_NA5459AA	245	261
ESC_NA5458AA	145	256
ESC_NA5457AA	245	261
ESC_NA5456AA	152	257
ESC_NA5455AA	152	257
ESC_NA5454AA	152	257
ESC_NA5453AA	245	261

ESC_NA5452AA	152	257
ESC_NA5451AA	152	257
ESC_NA5450AA	152	257
ESC_NA5448AA	152	257
ESC_NA5447AA	245	261
ESC_NA5446AA	152	257
ESC_NA5445AA	152	257
ESC_NA5444AA	245	261
ESC_NA5443AA	152	257
ESC_NA5442AA	245	261
ESC_NA5441AA	11	936
ESC_NA5440AA	1041	765
ESC_NA5439AA	11	936
ESC_NA5438AA	11	936
ESC_NA5437AA	675	234
ESC_NA5436AA	11	936
ESC_NA5434AA	2966	936
ESC_NA5433AA	11	936
ESC_NA5432AA	442	257
ESC_NA5431AA	11	936
ESC_NA5430AA	29	156
ESC_NA5429AA	11	936
ESC_NA5428AA	11	936
ESC_NA5427AA	33	198
ESC_NA5426AA	675	234
ESC_NA5425AA	17	191
ESC_NA5424AA	11	936
ESC_NA5423AA	16	156
ESC_NA5422AA	21	156
ESC_NA5421AA	11	936
ESC_NA5420AA	11	936
ESC_NA5419AA	342	286
ESC_NA5418AA	17	191
ESC_NA5417AA	342	286
ESC_NA5416AA	32	270
ESC_NA5415AA	11	936
ESC_NA5414AA	11	936
ESC_NA5413AA	11	936
ESC_NA5412AA	11	936
ESC_NA5411AA	11	936
ESC_NA5410AA	11	936
ESC_NA5409AA	11	936
ESC_NA5408AA	33	198
ESC_NA5407AA	25	234
ESC_NA5406AA	17	191
ESC_NA5405AA	381	156
ESC_NA5404AA	442	257
ESC_NA5403AA	25	234
ESC_NA5402AA	442	257
ESC_NA5401AA	672	198

ESC_NA5400AA	33	198
ESC_NA5399AA	11	936
ESC_NA5398AA	11	936
ESC_NA5397AA	11	936
ESC_NA5396AA	11	936
ESC_NA5395AA	11	936
ESC_NA5394AA	11	936
ESC_NA5393AA	11	936
ESC_NA5392AA	1844	198
ESC_NA5391AA	602	257
ESC_NA5390AA	3006	512
ESC_NA5350AA	655	926
ESC_NA5349AA	655	926
ESC_NA5348AA	723	156
ESC_NA5347AA	327	204
ESC_NA5346AA	11	936
ESC_NA5345AA	11	936
ESC_NA5344AA	17	191
ESC_NA5343AA	69	387
ESC_NA5342AA	38	669
ESC_NA5341AA	10	512
ESC_NA5340AA	69	387
ESC_NA5339AA	38	669
ESC_NA5338AA	569	738
ESC_NA5337AA	95	738
ESC_NA5336AA	428	654
ESC_NA5335AA	10	512
ESC_NA5334AA	404	761
ESC_NA5333AA	10	512
ESC_NA5332AA	155	175
ESC_NA5331AA	10	512
ESC_NA5330AA	10	512
ESC_NA5329AA	746	789
ESC_NA5328AA	59	217
ESC_NA5327AA	2705	504
ESC_NA5326AA	59	217
ESC_NA5325AA	9698	738
ESC_NA5324AA	120	180
ESC_NA5323AA	120	180
ESC_NA5322AA	569	738
ESC_NA5320AA	152	257
ESC_NA5319AA	152	257
ESC_NA5318AA	245	261
ESC_NA5317AA	245	261
ESC_NA5316AA	245	261
ESC_NA5315AA	245	261
ESC_NA5314AA	9317	257
ESC_NA5313AA	11	936
ESC_NA5312AA	16	156
ESC_NA5311AA	17	191

ESC_NA5310AA	11	936
ESC_NA5309AA	17	191
ESC_NA5308AA	11	936
ESC_NA5307AA	675	234
ESC_NA5306AA	17	191
ESC_NA5305AA	16	156
ESC_NA5304AA	21	156
ESC_NA5303AA	17	191
ESC_NA5302AA	17	191
ESC_NA5301AA	17	191
ESC_NA5300AA	11	936
ESC_NA5299AA	17	191
ESC_NA5298AA	7961	592
ESC_NA5297AA	11	936
ESC_NA5296AA	11	936
ESC_NA5295AA	17	191
ESC_NA5294AA	11	936
ESC_NA5293AA	32	270
ESC_NA5292AA	21	156
ESC_NA5291AA	17	191
ESC_NA5290AA	17	191
ESC_NA5289AA	17	191
ESC_NA5288AA	17	191
ESC_NA5287AA	21	156
ESC_NA5286AA	11	936
ESC_NA5285AA	21	156
ESC_NA5284AA	58	198
ESC_NA5283AA	16	156
ESC_NA5282AA	11	936
ESC_NA5281AA	17	191
ESC_NA5280AA	16	156
ESC_NA5279AA	17	191
ESC_NA5278AA	655	926
ESC_NA5277AA	21	156
ESC_NA5276AA	16	156
ESC_NA5275AA	1967	191
ESC_NA5274AA	21	156
ESC_NA5273AA	11	936
ESC_NA5271AA	16	156
ESC_NA5268AA	21	156
ESC_NA5266AA	43	512
ESC_NA5265AA	11	936
ESC_NA5263AA	32	270
ESC_NA5262AA	17	191
ESC_NA5260AA	40	198
ESC_NA5259AA	17	191
ESC_NA5257AA	11	936
ESC_NA5256AA	11	936
ESC_NA5255AA	58	198
ESC_NA5254AA	9873	41

ESC_NA5253AA	21	156
ESC_NA5251AA	409	504
ESC_NA5250AA	156	175
ESC_NA5249AA	218	519
ESC_NA5246AA	541	701
ESC_NA5245AA	540	286
ESC_NA5244AA	10	512
ESC_NA5243AA	245	261
ESC_NA5242AA	1792	156
ESC_NA5241AA	17	191
ESC_NA5240AA	21	156
ESC_NA5239AA	1967	191
ESC_NA5238AA	11	936
ESC_NA5237AA	17	191
ESC_NA5236AA	11	936
ESC_NA5235AA	5292	738
ESC_NA5234AA	21	156
ESC_NA5233AA	11	936
ESC_NA5232AA	343	286
ESC_NA5231AA	21	156
ESC_NA5230AA	655	926
ESC_NA5229AA	21	156
ESC_NA5228AA	21	156
ESC_NA5227AA	17	191
ESC_NA5226AA	16	156
ESC_NA5225AA	16	156
ESC_NA5224AA	11	936
ESC_NA5223AA	21	156
ESC_NA5222AA	11	936
ESC_NA5221AA	11	936
ESC_NA5220AA	11	936
ESC_NA5219AA	11	936
ESC_NA5218AA	10	512
ESC_NA5217AA	11	936
ESC_NA5216AA	1705	156
ESC_NA5215AA	21	156
ESC_NA5214AA	21	156
ESC_NA5213AA	21	156
ESC_NA5212AA	11	936
ESC_NA5211AA	11	936
ESC_NA5210AA	21	156
ESC_NA5208AA	21	156
ESC_NA5207AA	21	156
ESC_NA5206AA	215	512
ESC_NA5205AA	219	877
ESC_NA5204AA	38	669
ESC_NA5203AA	38	669
ESC_NA5202AA	450	71
ESC_NA5201AA	226	512
ESC_NA5200AA	11	936

ESC_NA5199AA	11	936
ESC_NA5198AA	11	936
ESC_NA5197AA	11	936
ESC_NA5196AA	11	936
ESC_NA5195AA	16	156
ESC_NA5194AA	16	156
ESC_NA5193AA	11	936
ESC_NA5192AA	11	936
ESC_NA5191AA	11	936
ESC_NA5190AA	11	936
ESC_NA5189AA	11	936
ESC_NA5188AA	11	936
ESC_NA5187AA	11	936
ESC_NA5186AA	17	191
ESC_NA5185AA	2485	212
ESC_NA5184AA	491	626
ESC_NA5183AA	8588	257
ESC_NA5182AA	245	261
ESC_NA5141AA	17	191
ESC_NA5140AA	17	191
ESC_NA5139AA	342	286
ESC_NA5138AA	17	191
ESC_NA5137AA	17	191
ESC_NA5136AA	73	737
ESC_NA5135AA	1112	504
ESC_NA5134AA	73	737
ESC_NA5133AA	10	512
ESC_NA5132AA	10	512
ESC_NA5131AA	5375	639
ESC_NA5130AA	10	512
ESC_NA5129AA	4260	737
ESC_NA5128AA	245	261
ESC_NA5126AA	11	936
ESC_NA5125AA	11	936
ESC_NA5124AA	10	512
ESC_NA5123AA	215	512
ESC_NA5122AA	398	445
ESC_NA5121AA	152	257
ESC_NA5120AA	245	261
ESC_NA5118AA	297	234
ESC_NA5117AA	11	936
ESC_NA5116AA	16	156
ESC_NA5115AA	21	156
ESC_NA5114AA	32	270
ESC_NA5113AA	33	198
ESC_NA5112AA	21	156
ESC_NA5111AA	21	156
ESC_NA5110AA	1967	191
ESC_NA5109AA	1433	601
ESC_NA5108AA	398	445

ESC_NA5107AA	2079	198
ESC_NA5106AA	16	156
ESC_NA5096AA	40	198
ESC_NA5095AA	21	156
ESC_NA5094AA	7237	191
ESC_NA5093AA	16	156
ESC_NA5092AA	11	936
ESC_NA5091AA	16	156
ESC_NA5090AA	21	156
ESC_NA5089AA	1967	191
ESC_NA5088AA	16	156
ESC_NA5087AA	70	670
ESC_NA5086AA	993	789
ESC_NA5085AA	675	234
ESC_NA5084AA	17	191
ESC_NA5083AA	17	191
ESC_NA5082AA	655	926
ESC_NA5081AA	9871	936
ESC_NA5080AA	152	257
ESC_NA5079AA	11	936
ESC_NA5078AA	11	936
ESC_NA5077AA	11	936
ESC_NA5076AA	11	936
ESC_NA5075AA	1308	257
ESC_NA5074AA	152	257
ESC_NA5073AA	3601	653
ESC_NA5072AA	21	156
ESC_NA5071AA	17	191
ESC_NA5070AA	1967	191
ESC_NA5069AA	2014	406
ESC_NA5068AA	5309	286
ESC_NA5067AA	9870	741
ESC_NA5066AA	127	738
ESC_NA5065AA	372	424
ESC_NA5064AA	9459	76
ESC_NA5063AA	11	936
ESC_NA5062AA	11	936
ESC_NA5061AA	21	156
ESC_NA5060AA	655	926
ESC_NA5059AA	11	936
ESC_NA5058AA	21	156
ESC_NA5057AA	21	156
ESC_NA5056AA	101	696
ESC_NA5055AA	11	936
ESC_NA5054AA	11	936
ESC_NA5053AA	11	936
ESC_NA5052AA	11	936
ESC_NA5051AA	675	234
ESC_NA5050AA	7673	156
ESC_NA5049AA	33	198

ESC_NA5048AA	11	936
ESC_NA5047AA	16	156
ESC_NA5046AA	16	156
ESC_NA5045AA	17	191
ESC_NA5044AA	21	156
ESC_NA5043AA	17	191
ESC_NA5042AA	16	156
ESC_NA5041AA	655	926
ESC_NA5040AA	655	926
ESC_NA5039AA	11	936
ESC_NA5038AA	21	156
ESC_NA5037AA	11	936
ESC_NA5036AA	21	156
ESC_NA5035AA	342	286
ESC_NA5034AA	11	936
ESC_NA5033AA	16	156
ESC_NA5031AA	119	286
ESC_NA5030AA	11	936
ESC_NA5029AA	11	936
ESC_NA5028AA	16	156
ESC_NA5027AA	420	741
ESC_NA5026AA	152	257
ESC_NA5025AA	17	191
ESC_NA5024AA	1967	191
ESC_NA5023AA	1967	191
ESC_NA5022AA	17	191
ESC_NA5021AA	11	936
ESC_NA5020AA	11	936
ESC_NA5019AA	17	191
ESC_NA5018AA	21	156
ESC_NA5017AA	306	286
ESC_NA5016AA	11	936
ESC_NA5015AA	5681	286
ESC_NA5014AA	655	926
ESC_NA5013AA	11	936
ESC_NA5012AA	58	198
ESC_NA5011AA	152	257
ESC_NA5010AA	21	156
ESC_NA5009AA	21	156
ESC_NA5008AA	17	191
ESC_NA5007AA	11	936
ESC_NA5006AA	442	257
ESC_NA5005AA	11	936
ESC_NA5004AA	6449	504
ESC_NA5003AA	21	156
ESC_NA5002AA	152	257
ESC_NA5001AA	691	899
ESC_NA5000AA	11	936
ESC_NA4999AA	940	257
ESC_NA4998AA	9317	257

ESC_NA4996AA	9317	257
ESC_NA4995AA	152	257
ESC_NA4994AA	9317	257
ESC_NA4992AA	16	156
ESC_NA4991AA	21	156
ESC_NA4990AA	226	512
ESC_NA4989AA	2514	504
ESC_NA4988AA	17	191
ESC_NA4987AA	17	191
ESC_NA4986AA	9869	191
ESC_NA4985AA	21	156
ESC_NA4984AA	21	156
ESC_NA4983AA	17	191
ESC_NA4982AA	16	156
ESC_NA4981AA	152	257
ESC_NA4980AA	145	256
ESC_NA4979AA	152	257
ESC_NA4978AA	245	261
ESC_NA4977AA	152	257
ESC_NA4976AA	145	256
ESC_NA4975AA	152	257
ESC_NA4974AA	245	261
ESC_NA4973AA	245	261
ESC_NA4972AA	245	261
ESC_NA4971AA	245	261
ESC_NA4970AA	152	257
ESC_NA4969AA	243	197
ESC_NA4968AA	152	257
ESC_NA4967AA	245	261
ESC_NA4966AA	245	261
ESC_NA4965AA	10	512
ESC_NA4964AA	675	234
ESC_NA4963AA	4305	512
ESC_NA4962AA	6251	808
ESC_NA4961AA	11	936
ESC_NA4960AA	11	936
ESC_NA4959AA	453	935
ESC_NA4958AA	11	936
ESC_NA4957AA	11	936
ESC_NA4956AA	11	936
ESC_NA4955AA	11	936
ESC_NA4954AA	17	191
ESC_NA4953AA	11	936
ESC_NA4952AA	16	156
ESC_NA4951AA	17	191
ESC_NA4950AA	655	926
ESC_NA4949AA	300	286
ESC_NA4948AA	17	191
ESC_NA4947AA	11	936
ESC_NA4946AA	428	654

ESC_NA4945AA	2079	198
ESC_NA4944AA	155	175
ESC_NA4943AA	205	257
ESC_NA4942AA	16	156
ESC_NA4941AA	675	234
ESC_NA4940AA	300	286
ESC_NA4939AA	21	156
ESC_NA4938AA	21	156
ESC_NA4937AA	17	191
ESC_NA4936AA	21	156
ESC_NA4935AA	21	156
ESC_NA4934AA	21	156
ESC_NA4933AA	11	936
ESC_NA4932AA	152	257
ESC_NA4931AA	152	257
ESC_NA4930AA	11	936
ESC_NA4929AA	11	936
ESC_NA4928AA	11	936
ESC_NA4927AA	11	936
ESC_NA4926AA	17	191
ESC_NA4925AA	17	191
ESC_NA4924AA	3100	653
ESC_NA4923AA	11	936
ESC_NA4922AA	17	191
ESC_NA4921AA	723	156
ESC_NA4920AA	16	156
ESC_NA4919AA	9343	150
ESC_NA4918AA	9342	175
ESC_NA4917AA	152	257
ESC_NA4916AA	245	261
ESC_NA4915AA	5700	198
ESC_NA4913AA	10	512
ESC_NA4912AA	215	512
ESC_NA4911AA	10	512
ESC_NA4910AA	215	512
ESC_NA4909AA	21	156
ESC_NA4908AA	40	198
ESC_NA4907AA	21	156
ESC_NA4906AA	602	257
ESC_NA4905AA	11	936
ESC_NA4904AA	69	387
ESC_NA4903AA	655	926
ESC_NA4902AA	655	926
ESC_NA4901AA	21	156
ESC_NA4900AA	21	156
ESC_NA4899AA	152	257
ESC_NA4898AA	152	257
ESC_NA4897AA	245	261
ESC_NA4896AA	152	257
ESC_NA4895AA	245	261

ESC_NA4894AA	152	257
ESC_NA4893AA	152	257
ESC_NA4892AA	245	261
ESC_NA4891AA	17	191
ESC_NA4890AA	388	156
ESC_NA4889AA	21	156
ESC_NA4888AA	4577	257
ESC_NA4887AA	4577	257
ESC_NA4886AA	10	512
ESC_NA4885AA	10	512
ESC_NA4884AA	10	512
ESC_NA4883AA	6602	692
ESC_NA4882AA	206	286
ESC_NA4881AA	10	512
ESC_NA4880AA	127	738
ESC_NA4879AA	100	504
ESC_NA4878AA	9867	198
ESC_NA4877AA	100	504
ESC_NA4876AA	9866	512
ESC_NA4875AA	6602	692
ESC_NA4874AA	206	286
ESC_NA4873AA	88	160
ESC_NA4872AA	152	257
ESC_NA4871AA	152	257
ESC_NA4870AA	152	257
ESC_NA4869AA	152	257
ESC_NA4867AA	152	257
ESC_NA4865AA	152	257
ESC_NA4864AA	152	257
ESC_NA4863AA	152	257
ESC_NA4862AA	152	257
ESC_NA4861AA	152	257
ESC_NA4860AA	152	257
ESC_NA4855AA	442	257
ESC_NA4854AA	2144	257
ESC_NA4853AA	215	512
ESC_NA4852AA	206	286
ESC_NA4851AA	10	512
ESC_NA4850AA	342	286
ESC_NA4849AA	215	512
ESC_NA4848AA	152	257
ESC_NA4847AA	73	737
ESC_NA4846AA	245	261
ESC_NA4845AA	21	156
ESC_NA4844AA	17	191
ESC_NA4843AA	11	936
ESC_NA4842AA	17	191
ESC_NA4841AA	205	257
ESC_NA4840AA	10	512
ESC_NA4839AA	10	512

ESC_NA4838AA	205	257
ESC_NA4837AA	205	257
ESC_NA4836AA	205	257
ESC_NA4835AA	205	257
ESC_NA4834AA	10	512
ESC_NA4833AA	342	286
ESC_NA4832AA	205	257
ESC_NA4831AA	17	191
ESC_NA4830AA	17	191
ESC_NA4828AA	17	191
ESC_NA4827AA	16	156
ESC_NA4826AA	205	257
ESC_NA4825AA	205	257
ESC_NA4824AA	205	257
ESC_NA4823AA	17	191
ESC_NA4822AA	205	257
ESC_NA4821AA	34	504
ESC_NA4820AA	11	936
ESC_NA4819AA	11	936
ESC_NA4818AA	32	270
ESC_NA4817AA	4170	87
ESC_NA4816AA	21	156
ESC_NA4815AA	11	936
ESC_NA4814AA	16	156
ESC_NA4813AA	38	669
ESC_NA4812AA	21	156
ESC_NA4811AA	16	156
ESC_NA4810AA	200	198
ESC_NA4809AA	11	936
ESC_NA4808AA	342	286
ESC_NA4807AA	10	512
ESC_NA4806AA	10	512
ESC_NA4805AA	342	286
ESC_NA4804AA	10	512
ESC_NA4803AA	342	286
ESC_NA4802AA	10	512
ESC_NA4801AA	10	512
ESC_NA4800AA	342	286
ESC_NA4799AA	10	512
ESC_NA4798AA	10	512
ESC_NA4797AA	342	286
ESC_NA4796AA	342	286
ESC_NA4795AA	342	286
ESC_NA4794AA	11	936
ESC_NA4793AA	11	936
ESC_NA4792AA	21	156
ESC_NA4791AA	388	156
ESC_NA4790AA	21	156
ESC_NA4789AA	21	156
ESC_NA4788AA	11	936

ESC_NA4787AA	11	936
ESC_NA4786AA	388	156
ESC_NA4785AA	21	156
ESC_NA4784AA	21	156
ESC_NA4783AA	21	156
ESC_NA4782AA	295	160
ESC_NA4781AA	11	936
ESC_NA4780AA	21	156
ESC_NA4779AA	11	936
ESC_NA4778AA	388	156
ESC_NA4777AA	25	234
ESC_NA4776AA	11	936
ESC_NA4775AA	21	156
ESC_NA4774AA	295	160
ESC_NA4773AA	17	191
ESC_NA4772AA	2387	261
ESC_NA4771AA	2387	261
ESC_NA4770AA	2387	261
ESC_NA4769AA	21	156
ESC_NA4768AA	388	156
ESC_NA4767AA	2387	261
ESC_NA4766AA	2387	261
ESC_NA4765AA	388	156
ESC_NA4764AA	58	198
ESC_NA4763AA	342	286
ESC_NA4762AA	295	160
ESC_NA4761AA	17	191
ESC_NA4760AA	388	156
ESC_NA4759AA	17	191
ESC_NA4758AA	21	156
ESC_NA4757AA	388	156
ESC_NA4756AA	21	156
ESC_NA4755AA	117	592
ESC_NA4754AA	388	156
ESC_NA4753AA	164	175
ESC_NA4752AA	46	447
ESC_NA4751AA	46	447
ESC_NA4750AA	46	447
ESC_NA4749AA	131	653
ESC_NA4748AA	167	512
ESC_NA4747AA	131	653
ESC_NA4746AA	44	512
ESC_NA4745AA	9847	512
ESC_NA4744AA	636	653
ESC_NA4743AA	636	653
ESC_NA4742AA	10	512
ESC_NA4741AA	46	447
ESC_NA4740AA	656	789
ESC_NA4739AA	656	789
ESC_NA4738AA	443	257

ESC_NA4737AA	1237	447
ESC_NA4736AA	131	653
ESC_NA4735AA	131	653
ESC_NA4734AA	410	156
ESC_NA4733AA	131	653
ESC_NA4732AA	101	696
ESC_NA4731AA	443	257
ESC_NA4730AA	131	653
ESC_NA4729AA	410	156
ESC_NA4728AA	391	669
ESC_NA4727AA	44	512
ESC_NA4726AA	636	653
ESC_NA4725AA	44	512
ESC_NA4724AA	617	511
ESC_NA4722AA	46	447
ESC_NA4721AA	46	447
ESC_NA4720AA	131	653
ESC_NA4718AA	636	653
ESC_NA4717AA	44	512
ESC_NA4716AA	410	156
ESC_NA4715AA	410	156
ESC_NA4714AA	131	653
ESC_NA4713AA	131	653
ESC_NA4712AA	131	653
ESC_NA4711AA	3489	962
ESC_NA4710AA	4981	473
ESC_NA4709AA	1722	599
ESC_NA4708AA	44	512
ESC_NA4707AA	167	512
ESC_NA4706AA	1722	599
ESC_NA4705AA	410	156
ESC_NA4704AA	167	512
ESC_NA4703AA	656	789
ESC_NA4702AA	3580	257
ESC_NA4700AA	354	15
ESC_NA4699AA	167	512
ESC_NA4697AA	648	359
ESC_NA4696AA	648	359
ESC_NA4695AA	38	669
ESC_NA4694AA	3168	261
ESC_NA4693AA	3168	261
ESC_NA4692AA	636	653
ESC_NA4691AA	44	512
ESC_NA4690AA	2705	504
ESC_NA4689AA	636	653
ESC_NA4688AA	9848	575
ESC_NA4687AA	9848	575
ESC_NA4686AA	9848	575
ESC_NA4685AA	410	156
ESC_NA4684AA	73	737

ESC_NA4683AA	656	789
ESC_NA4682AA	46	447
ESC_NA4681AA	46	447
ESC_NA4680AA	648	359
ESC_NA4679AA	167	512
ESC_NA4678AA	5824	504
ESC_NA4677AA	410	156
ESC_NA4676AA	648	359
ESC_NA4675AA	131	653
ESC_NA4674AA	46	447
ESC_NA4673AA	656	789
ESC_NA4672AA	940	257
ESC_NA4671AA	1722	599
ESC_NA4669AA	131	653
ESC_NA4668AA	167	512
ESC_NA4667AA	648	359
ESC_NA4666AA	34	504
ESC_NA4665AA	44	512
ESC_NA4664AA	405	923
ESC_NA4663AA	405	923
ESC_NA4662AA	636	653
ESC_NA4661AA	656	789
ESC_NA4660AA	940	257
ESC_NA4659AA	131	653
ESC_NA4658AA	131	653
ESC_NA4657AA	656	789
ESC_NA4656AA	517	924
ESC_NA4654AA	648	359
ESC_NA4650AA	167	512
ESC_NA4649AA	131	653
ESC_NA4646AA	424	234
ESC_NA4645AA	1722	599
ESC_NA4644AA	44	512
ESC_NA4643AA	354	15
ESC_NA4642AA	617	511
ESC_NA4640AA	354	15
ESC_NA4639AA	617	511
ESC_NA4638AA	46	447
ESC_NA4636AA	167	512
ESC_NA4635AA	167	512
ESC_NA4634AA	46	447
ESC_NA4633AA	219	877
ESC_NA4632AA	44	512
ESC_NA4631AA	131	653
ESC_NA4630AA	46	447
ESC_NA4629AA	131	653
ESC_NA4628AA	410	156
ESC_NA4627AA	167	512
ESC_NA4626AA	1722	599
ESC_NA4625AA	656	789

ESC_NA4624AA	1722	599
ESC_NA4623AA	1722	599
ESC_NA4622AA	219	877
ESC_NA4621AA	648	359
ESC_NA4620AA	648	359
ESC_NA4619AA	617	511
ESC_NA4618AA	636	653
ESC_NA4617AA	405	923
ESC_NA4616AA	405	923
ESC_NA4615AA	46	447
ESC_NA4614AA	1722	599
ESC_NA4613AA	38	669
ESC_NA4612AA	131	653
ESC_NA4611AA	131	653
ESC_NA4610AA	131	653
ESC_NA4609AA	131	653
ESC_NA4608AA	1722	599
ESC_NA4607AA	191	198
ESC_NA4606AA	410	156
ESC_NA4605AA	410	156
ESC_NA4604AA	1201	512
ESC_NA4603AA	10	512
ESC_NA4602AA	48	76
ESC_NA4601AA	9849	198
ESC_NA4600AA	48	76
ESC_NA4599AA	131	653
ESC_NA4598AA	410	156
ESC_NA4596AA	167	512
ESC_NA4595AA	617	511
ESC_NA4594AA	167	512
ESC_NA4593AA	167	512
ESC_NA4592AA	617	511
ESC_NA4591AA	7237	191
ESC_NA4589AA	652	204
ESC_NA4588AA	1434	512
ESC_NA4587AA	362	234
ESC_NA4586AA	9575	191
ESC_NA4585AA	1491	76
ESC_NA4584AA	21	156
ESC_NA4583AA	21	156
ESC_NA4582AA	21	156
ESC_NA4581AA	16	156
ESC_NA4580AA	342	286
ESC_NA4579AA	16	156
ESC_NA4578AA	21	156
ESC_NA4577AA	32	270
ESC_NA4576AA	17	191
ESC_NA4575AA	6617	519
ESC_NA4574AA	3274	512
ESC_NA4573AA	187	257

ESC_NA4572AA	1079	235
ESC_NA4571AA	9850	257
ESC_NA4570AA	7580	71
ESC_NA4569AA	898	108
ESC_NA4568AA	101	696
ESC_NA4567AA	189	504
ESC_NA4566AA	218	519
ESC_NA4565AA	3188	257
ESC_NA4564AA	69	387
ESC_NA4563AA	77	286
ESC_NA4562AA	1123	257
ESC_NA4561AA	2522	198
ESC_NA4560AA	906	198
ESC_NA4559AA	3580	257
ESC_NA4558AA	8058	160
ESC_NA4557AA	1125	198
ESC_NA4556AA	656	789
ESC_NA4555AA	9851	322
ESC_NA4554AA	9852	311
ESC_NA4553AA	2521	257
ESC_NA4552AA	10	512
ESC_NA4551AA	9853	261
ESC_NA4550AA	23	156
ESC_NA4549AA	655	926
ESC_NA4548AA	10	512
ESC_NA4547AA	2064	261
ESC_NA4546AA	648	359
ESC_NA4545AA	167	512
ESC_NA4544AA	101	696
ESC_NA4543AA	10	512
ESC_NA4542AA	9417	376
ESC_NA4541AA	542	824
ESC_NA4540AA	69	387
ESC_NA4539AA	1485	408
ESC_NA4538AA	155	175
ESC_NA4537AA	131	653
ESC_NA4536AA	21	156
ESC_NA4535AA	152	257
ESC_NA4534AA	245	261
ESC_NA4533AA	152	257
ESC_NA4532AA	152	257
ESC_NA4531AA	152	257
ESC_NA4530AA	152	257
ESC_NA4529AA	152	257
ESC_NA4528AA	245	261
ESC_NA4527AA	152	257
ESC_NA4526AA	152	257
ESC_NA4525AA	152	257
ESC_NA4524AA	152	257
ESC_NA4523AA	152	257

ESC_NA4522AA	152	257
ESC_NA4521AA	152	257
ESC_NA4520AA	1756	257
ESC_NA4519AA	152	257
ESC_NA4518AA	152	257
ESC_NA4517AA	152	257
ESC_NA4516AA	152	257
ESC_NA4515AA	152	257
ESC_NA4514AA	152	257
ESC_NA4513AA	152	257
ESC_NA4512AA	152	257
ESC_NA4511AA	1503	257
ESC_NA4510AA	152	257
ESC_NA4509AA	152	257
ESC_NA4508AA	152	257
ESC_NA4507AA	152	257
ESC_NA4506AA	152	257
ESC_NA4505AA	152	257
ESC_NA4504AA	152	257
ESC_NA4503AA	152	257
ESC_NA4502AA	152	257
ESC_NA4501AA	152	257
ESC_NA4500AA	152	257
ESC_NA4499AA	152	257
ESC_NA4498AA	152	257
ESC_NA4497AA	152	257
ESC_NA4496AA	152	257
ESC_NA4495AA	152	257
ESC_NA4494AA	152	257
ESC_NA4493AA	152	257
ESC_NA4492AA	1756	257
ESC_NA4491AA	152	257
ESC_NA4490AA	152	257
ESC_NA4489AA	152	257
ESC_NA4488AA	1503	257
ESC_NA4487AA	152	257
ESC_NA4486AA	152	257
ESC_NA4485AA	152	257
ESC_NA4484AA	152	257
ESC_NA4483AA	152	257
ESC_NA4482AA	152	257
ESC_NA4481AA	152	257
ESC_NA4480AA	152	257
ESC_NA4479AA	1756	257
ESC_NA4478AA	152	257
ESC_NA4477AA	152	257
ESC_NA4476AA	152	257
ESC_NA4475AA	152	257
ESC_NA4474AA	152	257
ESC_NA4473AA	152	257

ESC_NA4472AA	152	257
ESC_NA4471AA	1503	257
ESC_NA4470AA	152	257
ESC_NA4469AA	152	257
ESC_NA4468AA	152	257
ESC_NA4467AA	152	257
ESC_NA4466AA	152	257
ESC_NA4465AA	152	257
ESC_NA4464AA	152	257
ESC_NA4463AA	152	257
ESC_NA4462AA	152	257
ESC_NA4461AA	152	257
ESC_NA4460AA	152	257
ESC_NA4459AA	9317	257
ESC_NA4458AA	9317	257
ESC_NA4457AA	152	257
ESC_NA4456AA	245	261
ESC_NA4455AA	152	257
ESC_NA4454AA	152	257
ESC_NA4453AA	245	261
ESC_NA4452AA	245	261
ESC_NA4451AA	152	257
ESC_NA4450AA	152	257
ESC_NA4449AA	245	261
ESC_NA4448AA	245	261
ESC_NA4447AA	245	261
ESC_NA4446AA	245	261
ESC_NA4445AA	152	257
ESC_NA4444AA	152	257
ESC_NA4443AA	152	257
ESC_NA4442AA	245	261
ESC_NA4441AA	245	261
ESC_NA4440AA	245	261
ESC_NA4439AA	152	257
ESC_NA4438AA	152	257
ESC_NA4437AA	152	257
ESC_NA4436AA	152	257
ESC_NA4435AA	245	261
ESC_NA4434AA	152	257
ESC_NA4433AA	148	191
ESC_NA4432AA	6917	261
ESC_NA4431AA	152	257
ESC_NA4430AA	11	936
ESC_NA4429AA	630	261
ESC_NA4428AA	442	257
ESC_NA4427AA	17	191
ESC_NA4426AA	1967	191
ESC_NA4425AA	17	191
ESC_NA4424AA	17	191
ESC_NA4423AA	11	936

ESC_NA4422AA	11	936
ESC_NA4421AA	11	936
ESC_NA4420AA	11	936
ESC_NA4419AA	11	936
ESC_NA4418AA	5536	926
ESC_NA4417AA	388	156
ESC_NA4416AA	343	286
ESC_NA4415AA	655	926
ESC_NA4414AA	21	156
ESC_NA4413AA	306	286
ESC_NA4412AA	9855	662
ESC_NA4411AA	10	512
ESC_NA4410AA	11	936
ESC_NA4409AA	17	191
ESC_NA4408AA	11	936
ESC_NA4407AA	11	936
ESC_NA4406AA	16	156
ESC_NA4405AA	9854	156
ESC_NA4404AA	21	156
ESC_NA4403AA	21	156
ESC_NA4402AA	9854	156
ESC_NA4401AA	17	191
ESC_NA4400AA	5560	929
ESC_NA4399AA	678	257
ESC_NA4398AA	16	156
ESC_NA4397AA	10	512
ESC_NA4396AA	130	387
ESC_NA4395AA	69	387
ESC_NA4394AA	21	156
ESC_NA4393AA	11	936
ESC_NA4392AA	17	191
ESC_NA4391AA	56	198
ESC_NA4390AA	56	198
ESC_NA4389AA	11	936
ESC_NA4388AA	21	156
ESC_NA4387AA	56	198
ESC_NA4386AA	11	936
ESC_NA4385AA	7093	868
ESC_NA4384AA	56	198
ESC_NA4383AA	7093	868
ESC_NA4382AA	11	936
ESC_NA4381AA	11	936
ESC_NA4380AA	7093	868
ESC_NA4379AA	11	936
ESC_NA4378AA	21	156
ESC_NA4377AA	21	156
ESC_NA4376AA	21	156
ESC_NA4375AA	7093	868
ESC_NA4374AA	69	387
ESC_NA4373AA	11	936

ESC_NA4372AA	56	198
ESC_NA4371AA	11	936
ESC_NA4370AA	11	936
ESC_NA4369AA	11	936
ESC_NA4368AA	21	156
ESC_NA4367AA	21	156
ESC_NA4366AA	21	156
ESC_NA4365AA	7093	868
ESC_NA4364AA	56	198
ESC_NA4363AA	223	198
ESC_NA4362AA	21	156
ESC_NA4361AA	11	936
ESC_NA4360AA	21	156
ESC_NA4359AA	56	198
ESC_NA4358AA	4198	878
ESC_NA4357AA	1564	512
ESC_NA4356AA	16	156
ESC_NA4355AA	448	257
ESC_NA4354AA	1665	601
ESC_NA4353AA	29	156
ESC_NA4352AA	11	936
ESC_NA4351AA	205	257
ESC_NA4350AA	17	191
ESC_NA4349AA	11	936
ESC_NA4348AA	17	191
ESC_NA4347AA	9856	156
ESC_NA4346AA	829	257
ESC_NA4345AA	11	936
ESC_NA4344AA	21	156
ESC_NA4343AA	718	257
ESC_NA4342AA	38	669
ESC_NA4341AA	11	936
ESC_NA4331AA	93	71
ESC_NA4324AA	4942	36
ESC_NA4323AA	21	156
ESC_NA4322AA	21	156
ESC_NA4321AA	17	191
ESC_NA4320AA	21	156
ESC_NA4319AA	342	286
ESC_NA4318AA	5516	936
ESC_NA4317AA	342	286
ESC_NA4316AA	17	191
ESC_NA4315AA	119	286
ESC_NA4314AA	17	191
ESC_NA4313AA	278	924
ESC_NA4312AA	11	936
ESC_NA4311AA	17	191
ESC_NA4309AA	10	512
ESC_NA4308AA	10	512
ESC_NA4307AA	90	156

ESC_NA4306AA	641	935
ESC_NA4305AA	88	160
ESC_NA4304AA	10	512
ESC_NA4303AA	90	156
ESC_NA4302AA	58	198
ESC_NA4301AA	648	359
ESC_NA4300AA	90	156
ESC_NA4299AA	10	512
ESC_NA4298AA	1079	235
ESC_NA4297AA	669	76
ESC_NA4296AA	8636	935
ESC_NA4295AA	1642	198
ESC_NA4294AA	10	512
ESC_NA4293AA	410	156
ESC_NA4292AA	23	156
ESC_NA4291AA	641	935
ESC_NA4290AA	10	512
ESC_NA4289AA	10	512
ESC_NA4288AA	10	512
ESC_NA4287AA	9857	512
ESC_NA4286AA	655	926
ESC_NA4285AA	11	936
ESC_NA4284AA	11	936
ESC_NA4283AA	16	156
ESC_NA4282AA	17	191
ESC_NA4281AA	1967	191
ESC_NA4280AA	1967	191
ESC_NA4279AA	2836	221
ESC_NA4278AA	21	156
ESC_NA4277AA	21	156
ESC_NA4276AA	17	191
ESC_NA4275AA	16	156
ESC_NA4274AA	1967	191
ESC_NA4273AA	16	156
ESC_NA4272AA	5533	512
ESC_NA4271AA	5533	512
ESC_NA4270AA	5533	512
ESC_NA4269AA	5533	512
ESC_NA4268AA	10	512
ESC_NA4267AA	34	504
ESC_NA4266AA	34	504
ESC_NA4265AA	34	504
ESC_NA4264AA	34	504
ESC_NA4263AA	301	307
ESC_NA4262AA	381	156
ESC_NA4261AA	10	512
ESC_NA4260AA	11	936
ESC_NA4259AA	11	936
ESC_NA4258AA	11	936
ESC_NA4257AA	11	936

ESC_NA4256AA	11	936
ESC_NA4255AA	11	936
ESC_NA4254AA	11	936
ESC_NA4253AA	11	936
ESC_NA4252AA	342	286
ESC_NA4251AA	11	936
ESC_NA4250AA	16	156
ESC_NA4249AA	21	156
ESC_NA4248AA	11	936
ESC_NA4247AA	21	156
ESC_NA4246AA	17	191
ESC_NA4245AA	7431	936
ESC_NA4244AA	32	270
ESC_NA4243AA	17	191
ESC_NA4242AA	11	936
ESC_NA4241AA	1967	191
ESC_NA4240AA	10	512
ESC_NA4239AA	10	512
ESC_NA4237AA	10	512
ESC_NA4236AA	10	512
ESC_NA4235AA	10	512
ESC_NA4234AA	10	512
ESC_NA4233AA	10	512
ESC_NA4232AA	196	257
ESC_NA4231AA	10	512
ESC_NA4230AA	34	504
ESC_NA4229AA	164	175
ESC_NA4228AA	9528	820
ESC_NA4227AA	58	198
ESC_NA4226AA	297	234
ESC_NA4225AA	345	175
ESC_NA4224AA	6031	108
ESC_NA4223AA	906	198
ESC_NA4222AA	794	924
ESC_NA4221AA	2328	257
ESC_NA4220AA	58	198
ESC_NA4219AA	101	696
ESC_NA4218AA	2035	113
ESC_NA4217AA	1056	257
ESC_NA4216AA	9859	257
ESC_NA4215AA	3107	512
ESC_NA4214AA	9858	453
ESC_NA4213AA	1307	257
ESC_NA4212AA	154	257
ESC_NA4211AA	58	198
ESC_NA4210AA	937	198
ESC_NA4209AA	101	696
ESC_NA4208AA	16	156
ESC_NA4207AA	21	156
ESC_NA4206AA	11	936

ESC_NA4205AA	17	191
ESC_NA4204AA	10	512
ESC_NA4203AA	10	512
ESC_NA4202AA	10	512
ESC_NA4201AA	10	512
ESC_NA4200AA	10	512
ESC_NA4199AA	10	512
ESC_NA4198AA	10	512
ESC_NA4197AA	10	512
ESC_NA4196AA	10	512
ESC_NA4195AA	10	512
ESC_NA4194AA	11	936
ESC_NA4193AA	9863	592
ESC_NA4192AA	1167	257
ESC_NA4191AA	1705	156
ESC_NA4190AA	9862	156
ESC_NA4189AA	10	512
ESC_NA4188AA	10	512
ESC_NA4187AA	10	512
ESC_NA4185AA	11	936
ESC_NA4183AA	6	445
ESC_NA4182AA	21	156
ESC_NA4181AA	1967	191
ESC_NA4180AA	11	936
ESC_NA4179AA	9861	924
ESC_NA4178AA	5343	286
ESC_NA4177AA	11	936
ESC_NA4176AA	5419	175
ESC_NA4175AA	2385	198
ESC_NA4174AA	119	286
ESC_NA4173AA	11	936
ESC_NA4172AA	11	936
ESC_NA4171AA	7971	85
ESC_NA4170AA	9860	234
ESC_NA4169AA	11	936
ESC_NA4168AA	17	191
ESC_NA4167AA	343	286
ESC_NA4166AA	2338	257
ESC_NA4165AA	337	257
ESC_NA4164AA	20	198
ESC_NA4163AA	29	156
ESC_NA4162AA	21	156
ESC_NA4161AA	11	936
ESC_NA4160AA	337	257
ESC_NA4159AA	11	936
ESC_NA4158AA	11	936
ESC_NA4157AA	3602	234
ESC_NA4156AA	11	936
ESC_NA4155AA	11	936
ESC_NA4154AA	173	254

ESC_NA4153AA	17	191
ESC_NA4152AA	9865	234
ESC_NA4151AA	17	191
ESC_NA4150AA	3761	117
ESC_NA4148AA	21	156
ESC_NA4147AA	11	936
ESC_NA4146AA	343	286
ESC_NA4145AA	11	936
ESC_NA4144AA	1967	191
ESC_NA4143AA	21	156
ESC_NA4142AA	17	191
ESC_NA4141AA	17	191
ESC_NA4140AA	17	191
ESC_NA4139AA	21	156
ESC_NA4138AA	21	156
ESC_NA4137AA	21	156
ESC_NA4136AA	17	191
ESC_NA4135AA	17	191
ESC_NA4134AA	342	286
ESC_NA4133AA	21	156
ESC_NA4132AA	21	156
ESC_NA4131AA	21	156
ESC_NA4130AA	11	936
ESC_NA4129AA	29	156
ESC_NA4128AA	21	156
ESC_NA4127AA	17	191
ESC_NA4126AA	655	926
ESC_NA4125AA	11	936
ESC_NA4124AA	11	936
ESC_NA4123AA	11	936
ESC_NA4122AA	11	936
ESC_NA4120AA	21	156
ESC_NA4119AA	21	156
ESC_NA4118AA	17	191
ESC_NA4117AA	17	191
ESC_NA4116AA	9864	307
ESC_NA4115AA	16	156
ESC_NA4114AA	17	191
ESC_NA4113AA	675	234
ESC_NA4112AA	5487	198
ESC_NA4111AA	32	270
ESC_NA4110AA	17	191
ESC_NA4109AA	16	156
ESC_NA4108AA	21	156
ESC_NA4107AA	11	936
ESC_NA4106AA	17	191
ESC_NA4105AA	21	156
ESC_NA4104AA	21	156
ESC_NA4103AA	11	936
ESC_NA4102AA	811	234

ESC_NA4101AA	16	156
ESC_NA4100AA	21	156
ESC_NA4099AA	7858	156
ESC_NA4098AA	21	156
ESC_NA4097AA	10	512
ESC_NA4096AA	10	512
ESC_NA4095AA	675	234
ESC_NA4094AA	442	257
ESC_NA4093AA	675	234
ESC_NA4092AA	388	156
ESC_NA4091AA	301	307
ESC_NA4090AA	300	286
ESC_NA4089AA	300	286
ESC_NA4088AA	21	156
ESC_NA4087AA	378	504
ESC_NA4086AA	659	286
ESC_NA4085AA	21	156
ESC_NA4084AA	33	198
ESC_NA4083AA	11	936
ESC_NA4082AA	429	654
ESC_NA4081AA	23	156
ESC_NA4080AA	8874	421
ESC_NA4079AA	93	71
ESC_NA4078AA	2223	76
ESC_NA4077AA	117	592
ESC_NA4076AA	352	744
ESC_NA4075AA	429	654
ESC_NA4072AA	7104	234
ESC_NA4070AA	23	156
ESC_NA4069AA	770	587
ESC_NA4068AA	117	592
ESC_NA4067AA	1249	257
ESC_NA4066AA	2309	669
ESC_NA4065AA	533	198
ESC_NA4064AA	429	654
ESC_NA4063AA	140	738
ESC_NA4062AA	1841	198
ESC_NA4061AA	1157	878
ESC_NA4060AA	354	15
ESC_NA4059AA	23	156
ESC_NA4058AA	746	789
ESC_NA4057AA	95	738
ESC_NA4056AA	23	156
ESC_NA4055AA	162	924
ESC_NA4054AA	23	156
ESC_NA4053AA	1914	956
ESC_NA4052AA	429	654
ESC_NA4051AA	602	257
ESC_NA4050AA	4450	844
ESC_NA4049AA	9844	235

ESC_NA4048AA	152	257
ESC_NA4047AA	182	246
ESC_NA4046AA	215	512
ESC_NA4045AA	10	512
ESC_NA4044AA	1721	311
ESC_NA4043AA	2144	257
ESC_NA4042AA	450	71
ESC_NA4041AA	424	234
ESC_NA4040AA	405	923
ESC_NA4039AA	167	512
ESC_NA4038AA	152	257
ESC_NA4037AA	245	261
ESC_NA4036AA	245	261
ESC_NA4035AA	245	261
ESC_NA4034AA	11	936
ESC_NA4033AA	11	936
ESC_NA4032AA	167	512
ESC_NA4031AA	617	511
ESC_NA4030AA	10	512
ESC_NA4029AA	167	512
ESC_NA4028AA	167	512
ESC_NA4027AA	167	512
ESC_NA4026AA	167	512
ESC_NA4025AA	167	512
ESC_NA4024AA	540	286
ESC_NA4023AA	34	504
ESC_NA4022AA	167	512
ESC_NA4021AA	167	512
ESC_NA4020AA	167	512
ESC_NA4019AA	167	512
ESC_NA4018AA	167	512
ESC_NA4017AA	167	512
ESC_NA4016AA	167	512
ESC_NA4015AA	167	512
ESC_NA4014AA	17	191
ESC_NA4013AA	11	936
ESC_NA4012AA	5292	738
ESC_NA4010AA	131	653
ESC_NA4009AA	131	653
ESC_NA4008AA	131	653
ESC_NA4006AA	131	653
ESC_NA4005AA	9602	504
ESC_NA4004AA	685	311
ESC_NA4003AA	548	512
ESC_NA4002AA	401	142
ESC_NA4001AA	8305	235
ESC_NA4000AA	603	198
ESC_NA3998AA	2064	261
ESC_NA3997AA	10	512
ESC_NA3996AA	155	175

ESC_NA3995AA	1081	198
ESC_NA3994AA	399	198
ESC_NA3993AA	3202	71
ESC_NA3991AA	154	257
ESC_NA3990AA	11	936
ESC_NA3989AA	301	307
ESC_NA3988AA	329	355
ESC_NA3987AA	9829	234
ESC_NA3986AA	131	653
ESC_NA3985AA	131	653
ESC_NA3984AA	9817	787
ESC_NA3983AA	9817	787
ESC_NA3982AA	9817	787
ESC_NA3981AA	9816	286
ESC_NA3979AA	9815	504
ESC_NA3978AA	9813	175
ESC_NA3977AA	4086	842
ESC_NA3976AA	56	198
ESC_NA3975AA	1079	235
ESC_NA3974AA	1434	512
ESC_NA3973AA	1434	512
ESC_NA3972AA	1434	512
ESC_NA3971AA	2111	184
ESC_NA3970AA	1122	307
ESC_NA3969AA	109	257
ESC_NA3968AA	297	234
ESC_NA3967AA	9812	696
ESC_NA3966AA	9811	198
ESC_NA3965AA	16	156
ESC_NA3964AA	329	355
ESC_NA3963AA	388	156
ESC_NA3962AA	9809	198
ESC_NA3960AA	11	936
ESC_NA3959AA	11	936
ESC_NA3958AA	515	1004
ESC_NA3957AA	11	936
ESC_NA3956AA	8811	257
ESC_NA3955AA	152	257
ESC_NA3954AA	73	737
ESC_NA3953AA	73	737
ESC_NA3952AA	73	737
ESC_NA3951AA	73	737
ESC_NA3950AA	73	737
ESC_NA3949AA	73	737
ESC_NA3948AA	73	737
ESC_NA3947AA	73	737
ESC_NA3946AA	73	737
ESC_NA3945AA	73	737
ESC_NA3944AA	73	737
ESC_NA3943AA	73	737

ESC_NA3942AA	73	737
ESC_NA3941AA	73	737
ESC_NA3940AA	73	737
ESC_NA3939AA	73	737
ESC_NA3938AA	73	737
ESC_NA3937AA	73	737
ESC_NA3936AA	73	737
ESC_NA3935AA	73	737
ESC_NA3934AA	73	737
ESC_NA3933AA	73	737
ESC_NA3932AA	73	737
ESC_NA3931AA	73	737
ESC_NA3930AA	73	737
ESC_NA3929AA	73	737
ESC_NA3928AA	73	737
ESC_NA3927AA	73	737
ESC_NA3926AA	73	737
ESC_NA3925AA	9828	626
ESC_NA3924AA	73	737
ESC_NA3923AA	73	737
ESC_NA3922AA	73	737
ESC_NA3921AA	73	737
ESC_NA3920AA	73	737
ESC_NA3919AA	73	737
ESC_NA3918AA	73	737
ESC_NA3917AA	73	737
ESC_NA3916AA	73	737
ESC_NA3915AA	73	737
ESC_NA3914AA	73	737
ESC_NA3913AA	73	737
ESC_NA3912AA	73	737
ESC_NA3911AA	73	737
ESC_NA3910AA	73	737
ESC_NA3909AA	73	737
ESC_NA3908AA	73	737
ESC_NA3907AA	73	737
ESC_NA3906AA	73	737
ESC_NA3905AA	73	737
ESC_NA3904AA	73	737
ESC_NA3903AA	16	156
ESC_NA3902AA	21	156
ESC_NA3901AA	5352	936
ESC_NA3900AA	3594	924
ESC_NA3899AA	162	924
ESC_NA3898AA	5976	924
ESC_NA3897AA	7039	260
ESC_NA3896AA	21	156
ESC_NA3895AA	32	270
ESC_NA3894AA	6879	850
ESC_NA3893AA	11	936

ESC_NA3892AA	25	234
ESC_NA3891AA	6229	156
ESC_NA3890AA	1967	191
ESC_NA3889AA	2217	62
ESC_NA3888AA	21	156
ESC_NA3887AA	655	926
ESC_NA3886AA	306	286
ESC_NA3885AA	342	286
ESC_NA3884AA	40	198
ESC_NA3883AA	34	504
ESC_NA3882AA	245	261
ESC_NA3881AA	245	261
ESC_NA3880AA	245	261
ESC_NA3879AA	245	261
ESC_NA3878AA	11	936
ESC_NA3877AA	1611	198
ESC_NA3876AA	21	156
ESC_NA3875AA	11	936
ESC_NA3874AA	12	626
ESC_NA3873AA	9826	519
ESC_NA3872AA	372	424
ESC_NA3871AA	90	156
ESC_NA3870AA	993	789
ESC_NA3869AA	12	626
ESC_NA3868AA	9826	519
ESC_NA3867AA	131	653
ESC_NA3866AA	152	257
ESC_NA3865AA	152	257
ESC_NA3864AA	16	156
ESC_NA3863AA	90	156
ESC_NA3862AA	9826	519
ESC_NA3861AA	625	756
ESC_NA3860AA	90	156
ESC_NA3859AA	155	175
ESC_NA3858AA	9827	257
ESC_NA3857AA	90	156
ESC_NA3856AA	9826	519
ESC_NA3855AA	9826	519
ESC_NA3854AA	154	257
ESC_NA3853AA	5156	238
ESC_NA3852AA	34	504
ESC_NA3851AA	2015	684
ESC_NA3850AA	101	696
ESC_NA3849AA	17	191
ESC_NA3848AA	1967	191
ESC_NA3847AA	21	156
ESC_NA3846AA	75	257
ESC_NA3845AA	625	756
ESC_NA3844AA	10	512
ESC_NA3843AA	69	387

ESC_NA3842AA	405	923
ESC_NA3841AA	16	156
ESC_NA3840AA	9317	257
ESC_NA3839AA	9317	257
ESC_NA3838AA	245	261
ESC_NA3837AA	245	261
ESC_NA3836AA	17	191
ESC_NA3835AA	11	936
ESC_NA3834AA	11	936
ESC_NA3833AA	11	936
ESC_NA3832AA	21	156
ESC_NA3831AA	17	191
ESC_NA3830AA	5292	738
ESC_NA3829AA	17	191
ESC_NA3828AA	21	156
ESC_NA3827AA	21	156
ESC_NA3826AA	16	156
ESC_NA3825AA	16	156
ESC_NA3824AA	8915	574
ESC_NA3823AA	131	653
ESC_NA3822AA	131	653
ESC_NA3821AA	131	653
ESC_NA3820AA	131	653
ESC_NA3819AA	38	669
ESC_NA3818AA	38	669
ESC_NA3817AA	12	626
ESC_NA3816AA	69	387
ESC_NA3815AA	1193	761
ESC_NA3814AA	1193	761
ESC_NA3813AA	131	653
ESC_NA3812AA	131	653
ESC_NA3811AA	69	387
ESC_NA3810AA	131	653
ESC_NA3809AA	131	653
ESC_NA3808AA	540	286
ESC_NA3807AA	95	738
ESC_NA3806AA	131	653
ESC_NA3805AA	131	653
ESC_NA3804AA	131	653
ESC_NA3803AA	761	512
ESC_NA3802AA	616	156
ESC_NA3801AA	141	644
ESC_NA3800AA	131	653
ESC_NA3799AA	131	653
ESC_NA3798AA	131	653
ESC_NA3797AA	404	761
ESC_NA3796AA	38	669
ESC_NA3795AA	6309	575
ESC_NA3794AA	372	424
ESC_NA3793AA	38	669

ESC_NA3792AA	38	669
ESC_NA3791AA	131	653
ESC_NA3790AA	131	653
ESC_NA3789AA	38	669
ESC_NA3788AA	88	160
ESC_NA3787AA	636	653
ESC_NA3786AA	538	629
ESC_NA3785AA	10	512
ESC_NA3784AA	131	653
ESC_NA3783AA	131	653
ESC_NA3782AA	131	653
ESC_NA3781AA	14	761
ESC_NA3780AA	4363	632
ESC_NA3779AA	95	738
ESC_NA3778AA	73	737
ESC_NA3777AA	73	737
ESC_NA3776AA	131	653
ESC_NA3775AA	69	387
ESC_NA3774AA	117	592
ESC_NA3773AA	1193	761
ESC_NA3772AA	405	923
ESC_NA3771AA	38	669
ESC_NA3770AA	73	737
ESC_NA3769AA	131	653
ESC_NA3768AA	394	400
ESC_NA3767AA	95	738
ESC_NA3766AA	1312	76
ESC_NA3765AA	131	653
ESC_NA3764AA	2020	387
ESC_NA3763AA	131	653
ESC_NA3762AA	117	592
ESC_NA3761AA	131	653
ESC_NA3760AA	73	737
ESC_NA3759AA	95	738
ESC_NA3758AA	38	669
ESC_NA3757AA	405	923
ESC_NA3755AA	131	653
ESC_NA3754AA	405	923
ESC_NA3753AA	38	669
ESC_NA3752AA	1421	447
ESC_NA3751AA	131	653
ESC_NA3750AA	38	669
ESC_NA3749AA	131	653
ESC_NA3748AA	69	387
ESC_NA3747AA	1147	198
ESC_NA3746AA	38	669
ESC_NA3745AA	1722	599
ESC_NA3744AA	58	198
ESC_NA3743AA	131	653
ESC_NA3742AA	2599	257

ESC_NA3741AA	12	626
ESC_NA3740AA	38	669
ESC_NA3739AA	131	653
ESC_NA3738AA	69	387
ESC_NA3737AA	131	653
ESC_NA3736AA	73	737
ESC_NA3735AA	38	669
ESC_NA3734AA	69	387
ESC_NA3733AA	131	653
ESC_NA3732AA	354	15
ESC_NA3731AA	95	738
ESC_NA3730AA	131	653
ESC_NA3729AA	69	387
ESC_NA3728AA	648	359
ESC_NA3726AA	131	653
ESC_NA3725AA	131	653
ESC_NA3724AA	38	669
ESC_NA3723AA	69	387
ESC_NA3722AA	131	653
ESC_NA3721AA	131	653
ESC_NA3720AA	117	592
ESC_NA3719AA	131	653
ESC_NA3718AA	131	653
ESC_NA3717AA	131	653
ESC_NA3716AA	88	160
ESC_NA3715AA	131	653
ESC_NA3714AA	38	669
ESC_NA3713AA	69	387
ESC_NA3712AA	131	653
ESC_NA3711AA	1982	653
ESC_NA3710AA	2020	387
ESC_NA3709AA	131	653
ESC_NA3708AA	12	626
ESC_NA3707AA	6355	625
ESC_NA3706AA	357	654
ESC_NA3705AA	131	653
ESC_NA3704AA	131	653
ESC_NA3703AA	405	923
ESC_NA3702AA	131	653
ESC_NA3701AA	69	387
ESC_NA3700AA	131	653
ESC_NA3699AA	10	512
ESC_NA3698AA	69	387
ESC_NA3696AA	219	877
ESC_NA3695AA	131	653
ESC_NA3694AA	131	653
ESC_NA3693AA	131	653
ESC_NA3692AA	95	738
ESC_NA3691AA	38	669
ESC_NA3690AA	998	644

ESC_NA3689AA	38	669
ESC_NA3688AA	405	923
ESC_NA3687AA	131	653
ESC_NA3686AA	131	653
ESC_NA3685AA	12	626
ESC_NA3684AA	131	653
ESC_NA3683AA	131	653
ESC_NA3682AA	131	653
ESC_NA3681AA	131	653
ESC_NA3680AA	38	669
ESC_NA3679AA	69	387
ESC_NA3678AA	131	653
ESC_NA3677AA	131	653
ESC_NA3676AA	57	50
ESC_NA3675AA	453	935
ESC_NA3674AA	131	653
ESC_NA3673AA	38	669
ESC_NA3672AA	131	653
ESC_NA3671AA	131	653
ESC_NA3670AA	131	653
ESC_NA3669AA	127	738
ESC_NA3668AA	38	669
ESC_NA3667AA	131	653
ESC_NA3666AA	167	512
ESC_NA3665AA	101	696
ESC_NA3664AA	58	198
ESC_NA3663AA	38	669
ESC_NA3662AA	5041	400
ESC_NA3661AA	131	653
ESC_NA3660AA	131	653
ESC_NA3659AA	636	653
ESC_NA3658AA	131	653
ESC_NA3657AA	131	653
ESC_NA3656AA	131	653
ESC_NA3655AA	1193	761
ESC_NA3654AA	131	653
ESC_NA3653AA	131	653
ESC_NA3652AA	38	669
ESC_NA3651AA	131	653
ESC_NA3650AA	69	387
ESC_NA3649AA	131	653
ESC_NA3648AA	131	653
ESC_NA3647AA	69	387
ESC_NA3646AA	14	761
ESC_NA3645AA	131	653
ESC_NA3644AA	131	653
ESC_NA3643AA	73	737
ESC_NA3642AA	131	653
ESC_NA3641AA	75	257
ESC_NA3640AA	73	737

ESC_NA3639AA	131	653
ESC_NA3638AA	131	653
ESC_NA3637AA	127	738
ESC_NA3636AA	131	653
ESC_NA3635AA	131	653
ESC_NA3634AA	131	653
ESC_NA3633AA	131	653
ESC_NA3632AA	95	738
ESC_NA3631AA	131	653
ESC_NA3630AA	131	653
ESC_NA3629AA	131	653
ESC_NA3628AA	131	653
ESC_NA3627AA	73	737
ESC_NA3626AA	131	653
ESC_NA3625AA	1193	761
ESC_NA3624AA	131	653
ESC_NA3623AA	69	387
ESC_NA3622AA	131	653
ESC_NA3621AA	131	653
ESC_NA3620AA	95	738
ESC_NA3619AA	131	653
ESC_NA3618AA	73	737
ESC_NA3617AA	131	653
ESC_NA3616AA	95	738
ESC_NA3615AA	362	234
ESC_NA3614AA	131	653
ESC_NA3613AA	131	653
ESC_NA3612AA	10	512
ESC_NA3611AA	354	15
ESC_NA3610AA	131	653
ESC_NA3609AA	569	738
ESC_NA3608AA	152	257
ESC_NA3607AA	152	257
ESC_NA3606AA	152	257
ESC_NA3605AA	152	257
ESC_NA3604AA	243	197
ESC_NA3603AA	152	257
ESC_NA3602AA	245	261
ESC_NA3601AA	245	261
ESC_NA3600AA	152	257
ESC_NA3599AA	152	257
ESC_NA3598AA	152	257
ESC_NA3597AA	152	257
ESC_NA3593AA	245	261
ESC_NA3592AA	152	257
ESC_NA3591AA	245	261
ESC_NA3590AA	152	257
ESC_NA3589AA	245	261
ESC_NA3588AA	245	261
ESC_NA3586AA	152	257

ESC_NA3585AA	152	257
ESC_NA3584AA	152	257
ESC_NA3583AA	245	261
ESC_NA3582AA	245	261
ESC_NA3581AA	152	257
ESC_NA3580AA	152	257
ESC_NA3579AA	152	257
ESC_NA3578AA	245	261
ESC_NA3577AA	152	257
ESC_NA3576AA	152	257
ESC_NA3575AA	152	257
ESC_NA3574AA	152	257
ESC_NA3573AA	152	257
ESC_NA3572AA	152	257
ESC_NA3570AA	152	257
ESC_NA3569AA	152	257
ESC_NA3568AA	152	257
ESC_NA3567AA	245	261
ESC_NA3566AA	152	257
ESC_NA3565AA	152	257
ESC_NA3564AA	152	257
ESC_NA3563AA	152	257
ESC_NA3562AA	152	257
ESC_NA3561AA	152	257
ESC_NA3560AA	245	261
ESC_NA3559AA	152	257
ESC_NA3558AA	152	257
ESC_NA3557AA	152	257
ESC_NA3556AA	152	257
ESC_NA3555AA	245	261
ESC_NA3554AA	152	257
ESC_NA3553AA	245	261
ESC_NA3552AA	152	257
ESC_NA3551AA	152	257
ESC_NA3550AA	152	257
ESC_NA3549AA	245	261
ESC_NA3548AA	152	257
ESC_NA3547AA	152	257
ESC_NA3546AA	245	261
ESC_NA3545AA	152	257
ESC_NA3544AA	152	257
ESC_NA3543AA	245	261
ESC_NA3542AA	152	257
ESC_NA3541AA	145	256
ESC_NA3540AA	152	257
ESC_NA3539AA	152	257
ESC_NA3538AA	152	257
ESC_NA3537AA	152	257
ESC_NA3536AA	152	257
ESC_NA3535AA	152	257

ESC_NA3534AA	245	261
ESC_NA3533AA	245	261
ESC_NA3532AA	152	257
ESC_NA3531AA	152	257
ESC_NA3530AA	152	257
ESC_NA3529AA	152	257
ESC_NA3528AA	152	257
ESC_NA3527AA	152	257
ESC_NA3526AA	152	257
ESC_NA3525AA	152	257
ESC_NA3524AA	245	261
ESC_NA3523AA	152	257
ESC_NA3522AA	152	257
ESC_NA3521AA	245	261
ESC_NA3520AA	152	257
ESC_NA3519AA	245	261
ESC_NA3518AA	152	257
ESC_NA3517AA	152	257
ESC_NA3516AA	152	257
ESC_NA3515AA	152	257
ESC_NA3514AA	245	261
ESC_NA3513AA	152	257
ESC_NA3512AA	152	257
ESC_NA3511AA	152	257
ESC_NA3510AA	245	261
ESC_NA3509AA	245	261
ESC_NA3508AA	152	257
ESC_NA3507AA	245	261
ESC_NA3506AA	152	257
ESC_NA3505AA	245	261
ESC_NA3504AA	152	257
ESC_NA3503AA	152	257
ESC_NA3502AA	152	257
ESC_NA3501AA	1502	257
ESC_NA3500AA	152	257
ESC_NA3499AA	1503	257
ESC_NA3498AA	152	257
ESC_NA3497AA	152	257
ESC_NA3496AA	145	256
ESC_NA3495AA	245	261
ESC_NA3494AA	245	261
ESC_NA3493AA	152	257
ESC_NA3492AA	152	257
ESC_NA3491AA	152	257
ESC_NA3490AA	152	257
ESC_NA3489AA	152	257
ESC_NA3488AA	152	257
ESC_NA3487AA	245	261
ESC_NA3486AA	243	197
ESC_NA3485AA	152	257

ESC_NA3484AA	152	257
ESC_NA3483AA	630	261
ESC_NA3482AA	152	257
ESC_NA3481AA	245	261
ESC_NA3480AA	152	257
ESC_NA3479AA	5517	257
ESC_NA3478AA	152	257
ESC_NA3477AA	245	261
ESC_NA3476AA	152	257
ESC_NA3475AA	152	257
ESC_NA3474AA	145	256
ESC_NA3473AA	152	257
ESC_NA3472AA	145	256
ESC_NA3471AA	245	261
ESC_NA3470AA	145	256
ESC_NA3469AA	152	257
ESC_NA3468AA	152	257
ESC_NA3467AA	152	257
ESC_NA3466AA	152	257
ESC_NA3465AA	152	257
ESC_NA3464AA	152	257
ESC_NA3463AA	152	257
ESC_NA3462AA	245	261
ESC_NA3461AA	152	257
ESC_NA3460AA	245	261
ESC_NA3459AA	245	261
ESC_NA3458AA	245	261
ESC_NA3457AA	152	257
ESC_NA3456AA	245	261
ESC_NA3455AA	245	261
ESC_NA3454AA	152	257
ESC_NA3453AA	152	257
ESC_NA3452AA	245	261
ESC_NA3451AA	152	257
ESC_NA3450AA	152	257
ESC_NA3449AA	245	261
ESC_NA3448AA	245	261
ESC_NA3447AA	152	257
ESC_NA3446AA	152	257
ESC_NA3445AA	152	257
ESC_NA3444AA	152	257
ESC_NA3443AA	152	257
ESC_NA3442AA	145	256
ESC_NA3441AA	152	257
ESC_NA3440AA	152	257
ESC_NA3439AA	152	257
ESC_NA3438AA	152	257
ESC_NA3437AA	245	261
ESC_NA3436AA	152	257
ESC_NA3435AA	152	257

ESC_NA3434AA	152	257
ESC_NA3433AA	152	257
ESC_NA3432AA	152	257
ESC_NA3431AA	5475	256
ESC_NA3430AA	145	256
ESC_NA3429AA	152	257
ESC_NA3428AA	5517	257
ESC_NA3427AA	152	257
ESC_NA3426AA	245	261
ESC_NA3425AA	245	261
ESC_NA3424AA	245	261
ESC_NA3423AA	245	261
ESC_NA3422AA	245	261
ESC_NA3421AA	152	257
ESC_NA3420AA	152	257
ESC_NA3419AA	152	257
ESC_NA3418AA	245	261
ESC_NA3417AA	152	257
ESC_NA3416AA	152	257
ESC_NA3415AA	245	261
ESC_NA3414AA	245	261
ESC_NA3413AA	245	261
ESC_NA3412AA	5517	257
ESC_NA3411AA	152	257
ESC_NA3410AA	152	257
ESC_NA3409AA	152	257
ESC_NA3408AA	152	257
ESC_NA3407AA	152	257
ESC_NA3405AA	245	261
ESC_NA3404AA	152	257
ESC_NA3403AA	152	257
ESC_NA3402AA	152	257
ESC_NA3401AA	152	257
ESC_NA3400AA	245	261
ESC_NA3399AA	245	261
ESC_NA3398AA	152	257
ESC_NA3397AA	5517	257
ESC_NA3396AA	245	261
ESC_NA3395AA	245	261
ESC_NA3394AA	152	257
ESC_NA3393AA	245	261
ESC_NA3392AA	152	257
ESC_NA3391AA	628	261
ESC_NA3390AA	152	257
ESC_NA3389AA	152	257
ESC_NA3388AA	152	257
ESC_NA3387AA	152	257
ESC_NA3386AA	245	261
ESC_NA3385AA	152	257
ESC_NA3384AA	152	257

ESC_NA3383AA	152	257
ESC_NA3382AA	152	257
ESC_NA3381AA	152	257
ESC_NA3380AA	10	512
ESC_NA3379AA	8878	261
ESC_NA3378AA	245	261
ESC_NA3377AA	152	257
ESC_NA3376AA	245	261
ESC_NA3375AA	152	257
ESC_NA3374AA	152	257
ESC_NA3373AA	6917	261
ESC_NA3372AA	245	261
ESC_NA3371AA	152	257
ESC_NA3370AA	152	257
ESC_NA3369AA	152	257
ESC_NA3368AA	152	257
ESC_NA3367AA	152	257
ESC_NA3366AA	245	261
ESC_NA3365AA	152	257
ESC_NA3364AA	152	257
ESC_NA3363AA	245	261
ESC_NA3362AA	245	261
ESC_NA3361AA	152	257
ESC_NA3360AA	152	257
ESC_NA3359AA	152	257
ESC_NA3358AA	245	261
ESC_NA3357AA	245	261
ESC_NA3356AA	152	257
ESC_NA3355AA	145	256
ESC_NA3354AA	152	257
ESC_NA3353AA	245	261
ESC_NA3352AA	152	257
ESC_NA3351AA	152	257
ESC_NA3350AA	145	256
ESC_NA3349AA	145	256
ESC_NA3348AA	152	257
ESC_NA3347AA	152	257
ESC_NA3346AA	1503	257
ESC_NA3345AA	152	257
ESC_NA3344AA	152	257
ESC_NA3343AA	152	257
ESC_NA3342AA	1025	261
ESC_NA3341AA	152	257
ESC_NA3340AA	152	257
ESC_NA3339AA	145	256
ESC_NA3338AA	1025	261
ESC_NA3337AA	152	257
ESC_NA3336AA	152	257
ESC_NA3335AA	245	261
ESC_NA3334AA	152	257

ESC_NA3333AA	152	257
ESC_NA3332AA	152	257
ESC_NA3331AA	152	257
ESC_NA3330AA	152	257
ESC_NA3329AA	152	257
ESC_NA3328AA	152	257
ESC_NA3327AA	152	257
ESC_NA3326AA	152	257
ESC_NA3325AA	152	257
ESC_NA3324AA	152	257
ESC_NA3323AA	152	257
ESC_NA3322AA	152	257
ESC_NA3320AA	152	257
ESC_NA3319AA	152	257
ESC_NA3317AA	245	261
ESC_NA3316AA	11	936
ESC_NA3315AA	11	936
ESC_NA3314AA	405	923
ESC_NA3313AA	21	156
ESC_NA3312AA	21	156
ESC_NA3311AA	747	640
ESC_NA3309AA	104	737
ESC_NA3308AA	95	738
ESC_NA3307AA	95	738
ESC_NA3306AA	95	738
ESC_NA3303AA	69	387
ESC_NA3302AA	349	670
ESC_NA3300AA	131	653
ESC_NA3299AA	131	653
ESC_NA3298AA	127	738
ESC_NA3297AA	127	738
ESC_NA3293AA	115	669
ESC_NA3292AA	131	653
ESC_NA3291AA	3714	175
ESC_NA3290AA	38	669
ESC_NA3289AA	38	669
ESC_NA3288AA	38	669
ESC_NA3284AA	69	387
ESC_NA3281AA	6453	512
ESC_NA3276AA	1572	533
ESC_NA3274AA	223	198
ESC_NA3273AA	223	198
ESC_NA3272AA	3166	512
ESC_NA3271AA	11	936
ESC_NA3270AA	223	198
ESC_NA3268AA	738	598
ESC_NA3267AA	582	420
ESC_NA3266AA	21	156
ESC_NA3265AA	2952	926
ESC_NA3264AA	29	156

ESC_NA3263AA	738	598
ESC_NA3262AA	25	234
ESC_NA3261AA	11	936
ESC_NA3260AA	11	936
ESC_NA3259AA	6	445
ESC_NA3258AA	9823	400
ESC_NA3256AA	245	261
ESC_NA3254AA	9758	257
ESC_NA3252AA	99	198
ESC_NA3251AA	245	261
ESC_NA3250AA	450	71
ESC_NA3248AA	245	261
ESC_NA3247AA	6	445
ESC_NA3246AA	38	669
ESC_NA3245AA	4267	257
ESC_NA3244AA	4267	257
ESC_NA3243AA	311	204
ESC_NA3241AA	99	198
ESC_NA3240AA	394	400
ESC_NA3239AA	99	198
ESC_NA3238AA	6	445
ESC_NA3237AA	270	204
ESC_NA3236AA	99	198
ESC_NA3235AA	10	512
ESC_NA3234AA	4267	257
ESC_NA3233AA	4267	257
ESC_NA3230AA	200	198
ESC_NA3229AA	6	445
ESC_NA3228AA	270	204
ESC_NA3225AA	6	445
ESC_NA3224AA	311	204
ESC_NA3223AA	6	445
ESC_NA3222AA	99	198
ESC_NA3221AA	2067	257
ESC_NA3219AA	1025	261
ESC_NA3218AA	6	445
ESC_NA3217AA	4267	257
ESC_NA3216AA	152	257
ESC_NA3215AA	6	445
ESC_NA3214AA	9824	204
ESC_NA3213AA	6917	261
ESC_NA3211AA	99	198
ESC_NA3209AA	270	204
ESC_NA3208AA	245	261
ESC_NA3206AA	99	198
ESC_NA3205AA	99	198
ESC_NA3204AA	148	191
ESC_NA3203AA	4267	257
ESC_NA3202AA	245	261
ESC_NA3201AA	245	261

ESC_NA3200AA	245	261
ESC_NA3198AA	628	261
ESC_NA3197AA	3570	156
ESC_NA3196AA	245	261
ESC_NA3195AA	245	261
ESC_NA3194AA	4267	257
ESC_NA3193AA	6	445
ESC_NA3192AA	4267	257
ESC_NA3191AA	245	261
ESC_NA3189AA	131	653
ESC_NA3188AA	131	653
ESC_NA3186AA	131	653
ESC_NA3185AA	131	653
ESC_NA3180AA	131	653
ESC_NA3179AA	131	653
ESC_NA3176AA	131	653
ESC_NA3175AA	131	653
ESC_NA3173AA	131	653
ESC_NA3172AA	131	653
ESC_NA3171AA	131	653
ESC_NA3169AA	245	261
ESC_NA3168AA	301	307
ESC_NA3167AA	301	307
ESC_NA3166AA	21	156
ESC_NA3165AA	21	156
ESC_NA3164AA	21	156
ESC_NA3163AA	38	669
ESC_NA3162AA	38	669
ESC_NA3161AA	38	669
ESC_NA3160AA	38	669
ESC_NA3159AA	69	387
ESC_NA3158AA	38	669
ESC_NA3157AA	38	669
ESC_NA3156AA	38	669
ESC_NA3155AA	38	669
ESC_NA3154AA	2206	222
ESC_NA3153AA	46	447
ESC_NA3152AA	38	669
ESC_NA3151AA	1722	599
ESC_NA3150AA	46	447
ESC_NA3149AA	167	512
ESC_NA3148AA	361	512
ESC_NA3147AA	38	669
ESC_NA3146AA	38	669
ESC_NA3145AA	295	160
ESC_NA3144AA	38	669
ESC_NA3143AA	38	669
ESC_NA3142AA	69	387
ESC_NA3141AA	69	387
ESC_NA3140AA	227	504

ESC_NA3139AA	38	669
ESC_NA3138AA	38	669
ESC_NA3137AA	227	504
ESC_NA3136AA	38	669
ESC_NA3135AA	38	669
ESC_NA3134AA	10	512
ESC_NA3133AA	38	669
ESC_NA3132AA	38	669
ESC_NA3131AA	224	198
ESC_NA3130AA	38	669
ESC_NA3129AA	38	669
ESC_NA3128AA	38	669
ESC_NA3127AA	131	653
ESC_NA3126AA	38	669
ESC_NA3125AA	1722	599
ESC_NA3124AA	38	669
ESC_NA3123AA	38	669
ESC_NA3122AA	10	512
ESC_NA3121AA	38	669
ESC_NA3120AA	648	359
ESC_NA3119AA	38	669
ESC_NA3118AA	131	653
ESC_NA3117AA	38	669
ESC_NA3116AA	38	669
ESC_NA3115AA	38	669
ESC_NA3114AA	3541	842
ESC_NA3113AA	38	669
ESC_NA3112AA	69	387
ESC_NA3111AA	69	387
ESC_NA3110AA	361	512
ESC_NA3109AA	69	387
ESC_NA3108AA	38	669
ESC_NA3107AA	38	669
ESC_NA3106AA	38	669
ESC_NA3105AA	38	669
ESC_NA3104AA	38	669
ESC_NA3103AA	648	359
ESC_NA3102AA	38	669
ESC_NA3101AA	69	387
ESC_NA3100AA	167	512
ESC_NA3099AA	38	669
ESC_NA3098AA	167	512
ESC_NA3097AA	38	669
ESC_NA3096AA	38	669
ESC_NA3095AA	38	669
ESC_NA3094AA	38	669
ESC_NA3093AA	361	512
ESC_NA3092AA	38	669
ESC_NA3091AA	38	669
ESC_NA3090AA	38	669

ESC_NA3089AA	3541	842
ESC_NA3088AA	38	669
ESC_NA3087AA	69	387
ESC_NA3086AA	38	669
ESC_NA3085AA	38	669
ESC_NA3084AA	38	669
ESC_NA3083AA	38	669
ESC_NA3082AA	38	669
ESC_NA3081AA	1193	761
ESC_NA3080AA	9772	595
ESC_NA3079AA	16	156
ESC_NA3078AA	11	936
ESC_NA3077AA	21	156
ESC_NA3076AA	342	286
ESC_NA3075AA	342	286
ESC_NA3074AA	295	160
ESC_NA3073AA	342	286
ESC_NA3072AA	295	160
ESC_NA3071AA	342	286
ESC_NA3070AA	173	254
ESC_NA3069AA	295	160
ESC_NA3068AA	342	286
ESC_NA3067AA	295	160
ESC_NA3066AA	342	286
ESC_NA3065AA	21	156
ESC_NA3064AA	342	286
ESC_NA3063AA	21	156
ESC_NA3062AA	295	160
ESC_NA3061AA	295	160
ESC_NA3060AA	131	653
ESC_NA3059AA	131	653
ESC_NA3058AA	131	653
ESC_NA3057AA	131	653
ESC_NA3056AA	33	198
ESC_NA3055AA	16	156
ESC_NA3054AA	131	653
ESC_NA3053AA	9213	653
ESC_NA3050AA	4684	257
ESC_NA3049AA	16	156
ESC_NA3048AA	659	286
ESC_NA3047AA	9822	156
ESC_NA3045AA	11	936
ESC_NA3044AA	21	156
ESC_NA3043AA	21	156
ESC_NA3042AA	16	156
ESC_NA3041AA	16	156
ESC_NA3040AA	131	653
ESC_NA3039AA	1193	761
ESC_NA3038AA	131	653
ESC_NA3037AA	131	653

ESC_NA3036AA	131	653
ESC_NA3035AA	9317	257
ESC_NA3034AA	245	261
ESC_NA3033AA	245	261
ESC_NA3032AA	9317	257
ESC_NA3031AA	245	261
ESC_NA3030AA	245	261
ESC_NA3029AA	152	257
ESC_NA3028AA	9317	257
ESC_NA3027AA	245	261
ESC_NA3026AA	245	261
ESC_NA3025AA	152	257
ESC_NA3024AA	17	191
ESC_NA3023AA	40	198
ESC_NA3022AA	993	789
ESC_NA3021AA	11	936
ESC_NA3020AA	17	191
ESC_NA3019AA	34	504
ESC_NA3018AA	765	156
ESC_NA3017AA	21	156
ESC_NA3016AA	11	936
ESC_NA3015AA	11	936
ESC_NA3014AA	7713	156
ESC_NA3013AA	11	936
ESC_NA3012AA	21	156
ESC_NA3011AA	1792	156
ESC_NA3010AA	21	156
ESC_NA3009AA	11	936
ESC_NA3008AA	7581	996
ESC_NA3007AA	43	512
ESC_NA3006AA	21	156
ESC_NA3005AA	11	936
ESC_NA3003AA	11	936
ESC_NA3002AA	10	512
ESC_NA3001AA	16	156
ESC_NA3000AA	11	936
ESC_NA2999AA	11	936
ESC_NA2998AA	17	191
ESC_NA2997AA	342	286
ESC_NA2996AA	9771	598
ESC_NA2995AA	245	261
ESC_NA2994AA	152	257
ESC_NA2993AA	245	261
ESC_NA2992AA	245	261
ESC_NA2991AA	17	191
ESC_NA2990AA	17	191
ESC_NA2989AA	11	936
ESC_NA2988AA	21	156
ESC_NA2987AA	21	156
ESC_NA2986AA	17	191

ESC_NA2985AA	21	156
ESC_NA2984AA	655	926
ESC_NA2983AA	11	936
ESC_NA2982AA	29	156
ESC_NA2981AA	2836	221
ESC_NA2980AA	342	286
ESC_NA2979AA	1967	191
ESC_NA2978AA	21	156
ESC_NA2977AA	130	387
ESC_NA2976AA	278	924
ESC_NA2975AA	21	156
ESC_NA2974AA	21	156
ESC_NA2973AA	21	156
ESC_NA2972AA	17	191
ESC_NA2971AA	349	670
ESC_NA2970AA	17	191
ESC_NA2969AA	131	653
ESC_NA2968AA	212	198
ESC_NA2967AA	21	156
ESC_NA2966AA	223	198
ESC_NA2965AA	10	512
ESC_NA2964AA	675	234
ESC_NA2963AA	10	512
ESC_NA2962AA	21	156
ESC_NA2961AA	6412	24
ESC_NA2960AA	442	257
ESC_NA2959AA	223	198
ESC_NA2958AA	223	198
ESC_NA2957AA	442	257
ESC_NA2956AA	25	234
ESC_NA2955AA	33	198
ESC_NA2954AA	223	198
ESC_NA2953AA	11	936
ESC_NA2952AA	21	156
ESC_NA2951AA	21	156
ESC_NA2950AA	11	936
ESC_NA2949AA	21	156
ESC_NA2948AA	38	669
ESC_NA2947AA	38	669
ESC_NA2946AA	38	669
ESC_NA2943AA	131	653
ESC_NA2939AA	131	653
ESC_NA2936AA	131	653
ESC_NA2935AA	9770	575
ESC_NA2934AA	131	653
ESC_NA2933AA	131	653
ESC_NA2929AA	152	257
ESC_NA2928AA	17	191
ESC_NA2927AA	17	191
ESC_NA2926AA	245	261

ESC_NA2925AA	21	156
ESC_NA2924AA	21	156
ESC_NA2923AA	245	261
ESC_NA2922AA	11	936
ESC_NA2921AA	152	257
ESC_NA2920AA	152	257
ESC_NA2919AA	245	261
ESC_NA2918AA	152	257
ESC_NA2917AA	245	261
ESC_NA2916AA	152	257
ESC_NA2915AA	21	156
ESC_NA2914AA	17	191
ESC_NA2913AA	21	156
ESC_NA2912AA	21	156
ESC_NA2911AA	11	936
ESC_NA2910AA	16	156
ESC_NA2909AA	17	191
ESC_NA2908AA	747	640
ESC_NA2907AA	17	191
ESC_NA2906AA	16	156
ESC_NA2905AA	16	156
ESC_NA2904AA	17	191
ESC_NA2903AA	21	156
ESC_NA2902AA	16	156
ESC_NA2901AA	17	191
ESC_NA2900AA	21	156
ESC_NA2899AA	17	191
ESC_NA2898AA	17	191
ESC_NA2897AA	119	286
ESC_NA2896AA	17	191
ESC_NA2895AA	11	936
ESC_NA2894AA	16	156
ESC_NA2893AA	9821	191
ESC_NA2892AA	1859	653
ESC_NA2891AA	21	156
ESC_NA2890AA	17	191
ESC_NA2889AA	17	191
ESC_NA2888AA	17	191
ESC_NA2887AA	17	191
ESC_NA2886AA	21	156
ESC_NA2885AA	17	191
ESC_NA2884AA	223	198
ESC_NA2883AA	300	286
ESC_NA2882AA	21	156
ESC_NA2881AA	9820	924
ESC_NA2877AA	152	257
ESC_NA2876AA	152	257
ESC_NA2875AA	152	257
ESC_NA2874AA	152	257
ESC_NA2873AA	152	257

ESC_NA2872AA	152	257
ESC_NA2871AA	152	257
ESC_NA2870AA	152	257
ESC_NA2869AA	152	257
ESC_NA2868AA	152	257
ESC_NA2867AA	9749	512
ESC_NA2866AA	9747	653
ESC_NA2865AA	9241	738
ESC_NA2864AA	9748	653
ESC_NA2863AA	9746	669
ESC_NA2862AA	9744	560
ESC_NA2861AA	9745	76
ESC_NA2860AA	245	261
ESC_NA2859AA	245	261
ESC_NA2858AA	245	261
ESC_NA2857AA	245	261
ESC_NA2856AA	245	261
ESC_NA2855AA	17	191
ESC_NA2854AA	245	261
ESC_NA2853AA	1193	761
ESC_NA2852AA	29	156
ESC_NA2851AA	11	936
ESC_NA2850AA	9317	257
ESC_NA2849AA	9317	257
ESC_NA2848AA	152	257
ESC_NA2847AA	152	257
ESC_NA2846AA	152	257
ESC_NA2845AA	9818	156
ESC_NA2844AA	7961	592
ESC_NA2843AA	21	156
ESC_NA2842AA	32	270
ESC_NA2840AA	5374	156
ESC_NA2839AA	11	936
ESC_NA2838AA	1147	198
ESC_NA2837AA	11	936
ESC_NA2836AA	11	936
ESC_NA2835AA	69	387
ESC_NA2834AA	17	191
ESC_NA2833AA	32	270
ESC_NA2832AA	32	270
ESC_NA2831AA	16	156
ESC_NA2830AA	17	191
ESC_NA2829AA	21	156
ESC_NA2828AA	1967	191
ESC_NA2827AA	21	156
ESC_NA2826AA	16	156
ESC_NA2825AA	11	936
ESC_NA2824AA	642	924
ESC_NA2823AA	10	512
ESC_NA2822AA	90	156

ESC_NA2821AA	58	198
ESC_NA2820AA	90	156
ESC_NA2819AA	11	936
ESC_NA2818AA	21	156
ESC_NA2817AA	11	936
ESC_NA2816AA	11	936
ESC_NA2815AA	11	936
ESC_NA2814AA	11	936
ESC_NA2813AA	11	936
ESC_NA2812AA	11	936
ESC_NA2811AA	11	936
ESC_NA2810AA	2040	198
ESC_NA2809AA	29	156
ESC_NA2808AA	29	156
ESC_NA2807AA	17	191
ESC_NA2806AA	11	936
ESC_NA2805AA	306	286
ESC_NA2804AA	655	926
ESC_NA2803AA	641	935
ESC_NA2802AA	200	198
ESC_NA2801AA	10	512
ESC_NA2800AA	32	270
ESC_NA2799AA	156	175
ESC_NA2798AA	16	156
ESC_NA2797AA	21	156
ESC_NA2796AA	17	191
ESC_NA2795AA	17	191
ESC_NA2794AA	11	936
ESC_NA2793AA	245	261
ESC_NA2792AA	152	257
ESC_NA2791AA	245	261
ESC_NA2790AA	17	191
ESC_NA2789AA	16	156
ESC_NA2788AA	11	936
ESC_NA2787AA	17	191
ESC_NA2786AA	17	191
ESC_NA2785AA	17	191
ESC_NA2784AA	11	936
ESC_NA2783AA	21	156
ESC_NA2782AA	11	936
ESC_NA2781AA	1967	191
ESC_NA2780AA	21	156
ESC_NA2779AA	398	445
ESC_NA2778AA	10	512
ESC_NA2777AA	17	191
ESC_NA2776AA	17	191
ESC_NA2775AA	6274	257
ESC_NA2774AA	11	936
ESC_NA2773AA	16	156
ESC_NA2772AA	11	936

ESC_NA2771AA	11	936
ESC_NA2770AA	11	936
ESC_NA2769AA	11	936
ESC_NA2768AA	11	936
ESC_NA2767AA	343	286
ESC_NA2766AA	1967	191
ESC_NA2765AA	21	156
ESC_NA2764AA	21	156
ESC_NA2763AA	21	156
ESC_NA2762AA	1967	191
ESC_NA2761AA	567	740
ESC_NA2760AA	69	387
ESC_NA2759AA	131	653
ESC_NA2758AA	567	740
ESC_NA2757AA	131	653
ESC_NA2756AA	131	653
ESC_NA2755AA	362	234
ESC_NA2754AA	95	738
ESC_NA2753AA	297	234
ESC_NA2752AA	567	740
ESC_NA2751AA	154	257
ESC_NA2750AA	642	924
ESC_NA2749AA	154	257
ESC_NA2748AA	297	234
ESC_NA2747AA	1170	629
ESC_NA2746AA	152	257
ESC_NA2745AA	245	261
ESC_NA2744AA	152	257
ESC_NA2743AA	152	257
ESC_NA2742AA	152	257
ESC_NA2741AA	1503	257
ESC_NA2740AA	245	261
ESC_NA2739AA	152	257
ESC_NA2738AA	152	257
ESC_NA2737AA	245	261
ESC_NA2736AA	152	257
ESC_NA2735AA	245	261
ESC_NA2734AA	152	257
ESC_NA2733AA	245	261
ESC_NA2732AA	245	261
ESC_NA2731AA	152	257
ESC_NA2730AA	245	261
ESC_NA2729AA	21	156
ESC_NA2728AA	11	936
ESC_NA2727AA	17	191
ESC_NA2726AA	16	156
ESC_NA2725AA	1967	191
ESC_NA2724AA	7083	640
ESC_NA2723AA	11	936
ESC_NA2722AA	1148	257

ESC_NA2721AA	17	191
ESC_NA2720AA	11	936
ESC_NA2719AA	8345	936
ESC_NA2718AA	11	936
ESC_NA2717AA	11	936
ESC_NA2716AA	11	936
ESC_NA2715AA	11	936
ESC_NA2714AA	11	936
ESC_NA2713AA	5390	83
ESC_NA2712AA	11	936
ESC_NA2711AA	5822	696
ESC_NA2710AA	5390	83
ESC_NA2709AA	11	936
ESC_NA2708AA	10	512
ESC_NA2707AA	17	191
ESC_NA2706AA	11	936
ESC_NA2705AA	442	257
ESC_NA2704AA	32	270
ESC_NA2703AA	3101	160
ESC_NA2702AA	342	286
ESC_NA2701AA	10	512
ESC_NA2700AA	11	936
ESC_NA2698AA	33	198
ESC_NA2697AA	11	936
ESC_NA2696AA	11	936
ESC_NA2695AA	11	936
ESC_NA2694AA	17	191
ESC_NA2693AA	21	156
ESC_NA2692AA	301	307
ESC_NA2691AA	152	257
ESC_NA2690AA	152	257
ESC_NA2689AA	152	257
ESC_NA2688AA	245	261
ESC_NA2687AA	1024	261
ESC_NA2686AA	152	257
ESC_NA2685AA	9802	261
ESC_NA2684AA	11	936
ESC_NA2683AA	11	936
ESC_NA2682AA	25	234
ESC_NA2681AA	29	156
ESC_NA2680AA	33	198
ESC_NA2679AA	17	191
ESC_NA2678AA	11	936
ESC_NA2677AA	11	936
ESC_NA2676AA	442	257
ESC_NA2675AA	295	160
ESC_NA2674AA	642	924
ESC_NA2673AA	202	311
ESC_NA2672AA	6559	234
ESC_NA2671AA	75	257

ESC_NA2670AA	4087	512
ESC_NA2669AA	9804	878
ESC_NA2668AA	9803	257
ESC_NA2667AA	6559	234
ESC_NA2666AA	88	160
ESC_NA2665AA	23	156
ESC_NA2664AA	80	731
ESC_NA2663AA	23	156
ESC_NA2661AA	56	198
ESC_NA2660AA	829	257
ESC_NA2659AA	11	936
ESC_NA2658AA	119	286
ESC_NA2657AA	21	156
ESC_NA2656AA	11	936
ESC_NA2655AA	21	156
ESC_NA2654AA	10	512
ESC_NA2653AA	245	261
ESC_NA2652AA	9575	191
ESC_NA2651AA	33	198
ESC_NA2650AA	11	936
ESC_NA2649AA	16	156
ESC_NA2648AA	659	286
ESC_NA2647AA	16	156
ESC_NA2646AA	9801	198
ESC_NA2645AA	17	191
ESC_NA2644AA	29	156
ESC_NA2643AA	17	191
ESC_NA2642AA	2520	878
ESC_NA2641AA	152	257
ESC_NA2640AA	11	936
ESC_NA2639AA	659	286
ESC_NA2638AA	16	156
ESC_NA2636AA	11	936
ESC_NA2635AA	17	191
ESC_NA2634AA	245	261
ESC_NA2633AA	11	936
ESC_NA2632AA	11	936
ESC_NA2630AA	9721	234
ESC_NA2629AA	9720	156
ESC_NA2628AA	9719	512
ESC_NA2627AA	9718	235
ESC_NA2625AA	152	257
ESC_NA2624AA	300	286
ESC_NA2623AA	21	156
ESC_NA2622AA	17	191
ESC_NA2621AA	21	156
ESC_NA2620AA	21	156
ESC_NA2619AA	29	156
ESC_NA2618AA	21	156
ESC_NA2617AA	21	156

ESC_NA2616AA	9800	135
ESC_NA2615AA	21	156
ESC_NA2614AA	4684	257
ESC_NA2613AA	9018	85
ESC_NA2612AA	17	191
ESC_NA2611AA	16	156
ESC_NA2610AA	245	261
ESC_NA2609AA	1024	261
ESC_NA2608AA	245	261
ESC_NA2607AA	9799	261
ESC_NA2606AA	152	257
ESC_NA2605AA	245	261
ESC_NA2603AA	744	512
ESC_NA2602AA	32	270
ESC_NA2601AA	10	512
ESC_NA2600AA	21	156
ESC_NA2599AA	34	504
ESC_NA2598AA	9814	504
ESC_NA2597AA	109	257
ESC_NA2596AA	155	175
ESC_NA2595AA	58	198
ESC_NA2594AA	156	175
ESC_NA2593AA	155	175
ESC_NA2592AA	1684	504
ESC_NA2591AA	21	156
ESC_NA2590AA	17	191
ESC_NA2589AA	8654	257
ESC_NA2587AA	17	191
ESC_NA2586AA	245	261
ESC_NA2585AA	17	191
ESC_NA2583AA	21	156
ESC_NA2582AA	4607	900
ESC_NA2580AA	1159	381
ESC_NA2579AA	1159	381
ESC_NA2578AA	56	198
ESC_NA2577AA	56	198
ESC_NA2576AA	442	257
ESC_NA2575AA	56	198
ESC_NA2574AA	56	198
ESC_NA2573AA	56	198
ESC_NA2572AA	56	198
ESC_NA2571AA	56	198
ESC_NA2570AA	11	936
ESC_NA2569AA	58	198
ESC_NA2568AA	162	924
ESC_NA2567AA	223	198
ESC_NA2566AA	428	654
ESC_NA2565AA	2253	71
ESC_NA2564AA	218	519
ESC_NA2563AA	57	50

ESC_NA2562AA	155	175
ESC_NA2561AA	5976	924
ESC_NA2560AA	10	512
ESC_NA2559AA	101	696
ESC_NA2558AA	10	512
ESC_NA2557AA	48	76
ESC_NA2556AA	1771	868
ESC_NA2555AA	655	926
ESC_NA2554AA	16	156
ESC_NA2553AA	17	191
ESC_NA2552AA	119	286
ESC_NA2551AA	343	286
ESC_NA2550AA	21	156
ESC_NA2549AA	2165	257
ESC_NA2548AA	2063	198
ESC_NA2547AA	2165	257
ESC_NA2545AA	17	191
ESC_NA2544AA	675	234
ESC_NA2541AA	6024	175
ESC_NA2539AA	155	175
ESC_NA2538AA	155	175
ESC_NA2536AA	155	175
ESC_NA2533AA	10	512
ESC_NA2530AA	38	669
ESC_NA2529AA	10	512
ESC_NA2528AA	10	512
ESC_NA2527AA	10	512
ESC_NA2526AA	58	198
ESC_NA2525AA	10	512
ESC_NA2524AA	58	198
ESC_NA2523AA	131	653
ESC_NA2522AA	101	696
ESC_NA2521AA	101	696
ESC_NA2520AA	46	447
ESC_NA2519AA	46	447
ESC_NA2518AA	46	447
ESC_NA2517AA	46	447
ESC_NA2516AA	297	234
ESC_NA2515AA	3580	257
ESC_NA2514AA	3580	257
ESC_NA2513AA	10	512
ESC_NA2512AA	10	512
ESC_NA2511AA	772	76
ESC_NA2510AA	10	512
ESC_NA2509AA	906	198
ESC_NA2508AA	10	512
ESC_NA2506AA	906	198
ESC_NA2505AA	906	198
ESC_NA2504AA	906	198
ESC_NA2503AA	906	198

ESC_NA2502AA	906	198
ESC_NA2501AA	906	198
ESC_NA2500AA	906	198
ESC_NA2499AA	906	198
ESC_NA2498AA	906	198
ESC_NA2497AA	162	924
ESC_NA2496AA	162	924
ESC_NA2495AA	48	76
ESC_NA2494AA	48	76
ESC_NA2493AA	48	76
ESC_NA2492AA	48	76
ESC_NA2491AA	46	447
ESC_NA2490AA	761	512
ESC_NA2489AA	46	447
ESC_NA2488AA	46	447
ESC_NA2487AA	3580	257
ESC_NA2486AA	3580	257
ESC_NA2483AA	3580	257
ESC_NA2482AA	3580	257
ESC_NA2481AA	3580	257
ESC_NA2480AA	3580	257
ESC_NA2479AA	3580	257
ESC_NA2478AA	3580	257
ESC_NA2477AA	350	50
ESC_NA2475AA	1594	71
ESC_NA2474AA	1594	71
ESC_NA2473AA	349	670
ESC_NA2472AA	23	156
ESC_NA2471AA	9717	160
ESC_NA2470AA	349	670
ESC_NA2469AA	616	156
ESC_NA2468AA	23	156
ESC_NA2467AA	720	900
ESC_NA2466AA	616	156
ESC_NA2463AA	10	512
ESC_NA2462AA	10	512
ESC_NA2461AA	10	512
ESC_NA2460AA	10	512
ESC_NA2459AA	10	512
ESC_NA2458AA	10	512
ESC_NA2456AA	162	924
ESC_NA2455AA	162	924
ESC_NA2454AA	162	924
ESC_NA2452AA	3580	257
ESC_NA2451AA	3580	257
ESC_NA2450AA	3580	257
ESC_NA2449AA	5897	912
ESC_NA2448AA	3489	962
ESC_NA2447AA	58	198
ESC_NA2446AA	117	592

ESC_NA2445AA	602	257
ESC_NA2444AA	117	592
ESC_NA2443AA	155	175
ESC_NA2442AA	155	175
ESC_NA2441AA	118	985
ESC_NA2440AA	752	512
ESC_NA2439AA	752	512
ESC_NA2438AA	676	737
ESC_NA2437AA	706	422
ESC_NA2436AA	4197	856
ESC_NA2435AA	117	592
ESC_NA2434AA	95	738
ESC_NA2433AA	10	512
ESC_NA2432AA	7321	511
ESC_NA2431AA	101	696
ESC_NA2430AA	189	504
ESC_NA2429AA	2500	512
ESC_NA2428AA	2538	900
ESC_NA2427AA	501	900
ESC_NA2426AA	10	512
ESC_NA2425AA	117	592
ESC_NA2424AA	101	696
ESC_NA2423AA	117	592
ESC_NA2422AA	1079	235
ESC_NA2421AA	2171	164
ESC_NA2420AA	349	670
ESC_NA2419AA	457	595
ESC_NA2418AA	362	234
ESC_NA2417AA	501	900
ESC_NA2416AA	501	900
ESC_NA2415AA	117	592
ESC_NA2414AA	165	504
ESC_NA2413AA	117	592
ESC_NA2412AA	192	696
ESC_NA2411AA	118	985
ESC_NA2410AA	2223	76
ESC_NA2409AA	10	512
ESC_NA2408AA	10	512
ESC_NA2407AA	720	900
ESC_NA2406AA	155	175
ESC_NA2405AA	43	512
ESC_NA2404AA	349	670
ESC_NA2403AA	501	900
ESC_NA2402AA	2792	886
ESC_NA2401AA	2001	670
ESC_NA2400AA	46	447
ESC_NA2399AA	2792	886
ESC_NA2398AA	2792	886
ESC_NA2397AA	349	670
ESC_NA2396AA	131	653

ESC_NA2394AA	131	653
ESC_NA2393AA	9714	496
ESC_NA2392AA	602	257
ESC_NA2391AA	131	653
ESC_NA2390AA	602	257
ESC_NA2389AA	501	900
ESC_NA2388AA	117	592
ESC_NA2387AA	38	669
ESC_NA2386AA	23	156
ESC_NA2385AA	57	50
ESC_NA2384AA	38	669
ESC_NA2383AA	1266	210
ESC_NA2382AA	4550	150
ESC_NA2381AA	9715	592
ESC_NA2380AA	134	738
ESC_NA2379AA	1266	210
ESC_NA2378AA	101	696
ESC_NA2377AA	10	512
ESC_NA2376AA	9714	496
ESC_NA2375AA	192	696
ESC_NA2374AA	297	234
ESC_NA2373AA	117	592
ESC_NA2372AA	4197	856
ESC_NA2371AA	4197	856
ESC_NA2370AA	5897	912
ESC_NA2369AA	8582	912
ESC_NA2368AA	58	198
ESC_NA2367AA	224	198
ESC_NA2366AA	3057	672
ESC_NA2365AA	501	900
ESC_NA2364AA	9713	696
ESC_NA2363AA	155	175
ESC_NA2362AA	967	212
ESC_NA2361AA	245	261
ESC_NA2360AA	1503	257
ESC_NA2359AA	245	261
ESC_NA2358AA	152	257
ESC_NA2357AA	152	257
ESC_NA2356AA	245	261
ESC_NA2355AA	152	257
ESC_NA2354AA	11	936
ESC_NA2353AA	32	270
ESC_NA2352AA	21	156
ESC_NA2351AA	4942	36
ESC_NA2350AA	11	936
ESC_NA2349AA	11	936
ESC_NA2348AA	343	286
ESC_NA2347AA	11	936
ESC_NA2346AA	11	936
ESC_NA2345AA	17	191

ESC_NA2344AA	11	936
ESC_NA2343AA	21	156
ESC_NA2342AA	9798	592
ESC_NA2341AA	17	191
ESC_NA2340AA	21	156
ESC_NA2339AA	245	261
ESC_NA2338AA	33	198
ESC_NA2337AA	9793	191
ESC_NA2336AA	655	926
ESC_NA2335AA	21	156
ESC_NA2334AA	152	257
ESC_NA2333AA	21	156
ESC_NA2332AA	32	270
ESC_NA2331AA	349	670
ESC_NA2330AA	131	653
ESC_NA2329AA	23	156
ESC_NA2328AA	117	592
ESC_NA2327AA	369	156
ESC_NA2326AA	428	654
ESC_NA2325AA	131	653
ESC_NA2324AA	978	740
ESC_NA2323AA	69	387
ESC_NA2322AA	23	156
ESC_NA2321AA	23	156
ESC_NA2320AA	10	512
ESC_NA2319AA	369	156
ESC_NA2318AA	1618	736
ESC_NA2317AA	3580	257
ESC_NA2316AA	1852	654
ESC_NA2315AA	9712	912
ESC_NA2314AA	117	592
ESC_NA2313AA	117	592
ESC_NA2312AA	429	654
ESC_NA2311AA	117	592
ESC_NA2310AA	117	592
ESC_NA2309AA	117	592
ESC_NA2308AA	117	592
ESC_NA2307AA	117	592
ESC_NA2306AA	117	592
ESC_NA2305AA	117	592
ESC_NA2303AA	23	156
ESC_NA2301AA	428	654
ESC_NA2300AA	69	387
ESC_NA2299AA	906	198
ESC_NA2298AA	9711	562
ESC_NA2297AA	3580	257
ESC_NA2296AA	355	764
ESC_NA2295AA	1485	408
ESC_NA2294AA	428	654
ESC_NA2292AA	117	592

ESC_NA2291AA	23	156
ESC_NA2289AA	131	653
ESC_NA2288AA	131	653
ESC_NA2287AA	349	670
ESC_NA2286AA	349	670
ESC_NA2285AA	23	156
ESC_NA2284AA	58	198
ESC_NA2283AA	428	654
ESC_NA2282AA	1618	736
ESC_NA2281AA	428	654
ESC_NA2280AA	23	156
ESC_NA2279AA	428	654
ESC_NA2278AA	117	592
ESC_NA2277AA	428	654
ESC_NA2276AA	131	653
ESC_NA2275AA	355	764
ESC_NA2274AA	1852	654
ESC_NA2272AA	117	592
ESC_NA2271AA	23	156
ESC_NA2269AA	3580	257
ESC_NA2268AA	2556	630
ESC_NA2267AA	117	592
ESC_NA2266AA	349	670
ESC_NA2265AA	23	156
ESC_NA2264AA	6949	765
ESC_NA2263AA	3580	257
ESC_NA2262AA	1618	736
ESC_NA2261AA	648	359
ESC_NA2260AA	648	359
ESC_NA2259AA	23	156
ESC_NA2258AA	117	592
ESC_NA2257AA	1485	408
ESC_NA2256AA	23	156
ESC_NA2255AA	8435	198
ESC_NA2254AA	162	924
ESC_NA2253AA	155	175
ESC_NA2252AA	744	512
ESC_NA2251AA	6949	765
ESC_NA2250AA	6949	765
ESC_NA2249AA	6949	765
ESC_NA2248AA	351	268
ESC_NA2247AA	23	156
ESC_NA2246AA	2556	630
ESC_NA2245AA	23	156
ESC_NA2244AA	10	512
ESC_NA2243AA	117	592
ESC_NA2242AA	756	257
ESC_NA2241AA	117	592
ESC_NA2240AA	117	592
ESC_NA2239AA	117	592

ESC_NA2238AA	747	640
ESC_NA2237AA	4392	234
ESC_NA2236AA	117	592
ESC_NA2235AA	2556	630
ESC_NA2234AA	6710	198
ESC_NA2233AA	491	626
ESC_NA2232AA	2556	630
ESC_NA2231AA	6710	198
ESC_NA2230AA	2556	630
ESC_NA2229AA	58	198
ESC_NA2228AA	2556	630
ESC_NA2227AA	23	156
ESC_NA2226AA	2556	630
ESC_NA2225AA	2556	630
ESC_NA2224AA	131	653
ESC_NA2223AA	58	198
ESC_NA2222AA	2556	630
ESC_NA2221AA	1485	408
ESC_NA2219AA	2556	630
ESC_NA2218AA	1851	380
ESC_NA2217AA	2556	630
ESC_NA2216AA	58	198
ESC_NA2215AA	131	653
ESC_NA2214AA	9707	343
ESC_NA2213AA	355	764
ESC_NA2212AA	9769	156
ESC_NA2211AA	2556	630
ESC_NA2210AA	2556	630
ESC_NA2209AA	131	653
ESC_NA2208AA	648	359
ESC_NA2207AA	429	654
ESC_NA2206AA	1582	198
ESC_NA2205AA	131	653
ESC_NA2204AA	1236	380
ESC_NA2203AA	937	198
ESC_NA2202AA	108	257
ESC_NA2201AA	131	653
ESC_NA2200AA	491	626
ESC_NA2199AA	131	653
ESC_NA2198AA	23	156
ESC_NA2197AA	6106	234
ESC_NA2196AA	2556	630
ESC_NA2195AA	9709	582
ESC_NA2194AA	131	653
ESC_NA2193AA	131	653
ESC_NA2192AA	1914	956
ESC_NA2191AA	23	156
ESC_NA2189AA	117	592
ESC_NA2184AA	973	968
ESC_NA2183AA	538	629

ESC_NA2182AA	9708	512
ESC_NA2181AA	23	156
ESC_NA2180AA	428	654
ESC_NA2179AA	162	924
ESC_NA2178AA	23	156
ESC_NA2177AA	23	156
ESC_NA2175AA	2556	630
ESC_NA2174AA	2556	630
ESC_NA2172AA	131	653
ESC_NA2171AA	2556	630
ESC_NA2170AA	428	654
ESC_NA2169AA	428	654
ESC_NA2167AA	5796	592
ESC_NA2166AA	135	653
ESC_NA2165AA	429	654
ESC_NA2164AA	23	156
ESC_NA2163AA	2556	630
ESC_NA2162AA	428	654
ESC_NA2161AA	117	592
ESC_NA2160AA	355	764
ESC_NA2159AA	428	654
ESC_NA2158AA	23	156
ESC_NA2157AA	428	654
ESC_NA2156AA	117	592
ESC_NA2155AA	117	592
ESC_NA2154AA	117	592
ESC_NA2153AA	349	670
ESC_NA2152AA	23	156
ESC_NA2151AA	428	654
ESC_NA2150AA	428	654
ESC_NA2147AA	117	592
ESC_NA2146AA	131	653
ESC_NA2145AA	117	592
ESC_NA2144AA	131	653
ESC_NA2143AA	117	592
ESC_NA2142AA	3580	257
ESC_NA2141AA	428	654
ESC_NA2140AA	117	592
ESC_NA2139AA	428	654
ESC_NA2138AA	131	653
ESC_NA2137AA	117	592
ESC_NA2136AA	117	592
ESC_NA2135AA	428	654
ESC_NA2134AA	974	160
ESC_NA2133AA	428	654
ESC_NA2132AA	117	592
ESC_NA2131AA	355	764
ESC_NA2130AA	428	654
ESC_NA2129AA	355	764
ESC_NA2128AA	616	156

ESC_NA2127AA	355	764
ESC_NA2126AA	117	592
ESC_NA2125AA	355	764
ESC_NA2124AA	355	764
ESC_NA2123AA	1618	736
ESC_NA2122AA	117	592
ESC_NA2121AA	38	669
ESC_NA2120AA	23	156
ESC_NA2119AA	428	654
ESC_NA2118AA	131	653
ESC_NA2117AA	428	654
ESC_NA2116AA	23	156
ESC_NA2115AA	1618	736
ESC_NA2114AA	428	654
ESC_NA2113AA	117	592
ESC_NA2112AA	349	670
ESC_NA2110AA	58	198
ESC_NA2109AA	974	160
ESC_NA2108AA	131	653
ESC_NA2107AA	349	670
ESC_NA2106AA	349	670
ESC_NA2105AA	131	653
ESC_NA2104AA	428	654
ESC_NA2103AA	1618	736
ESC_NA2102AA	117	592
ESC_NA2100AA	349	670
ESC_NA2099AA	428	654
ESC_NA2098AA	117	592
ESC_NA2097AA	1618	736
ESC_NA2096AA	428	654
ESC_NA2095AA	350	50
ESC_NA2094AA	428	654
ESC_NA2093AA	131	653
ESC_NA2092AA	428	654
ESC_NA2091AA	131	653
ESC_NA2090AA	428	654
ESC_NA2089AA	349	670
ESC_NA2088AA	355	764
ESC_NA2087AA	86	935
ESC_NA2086AA	350	50
ESC_NA2085AA	1882	387
ESC_NA2084AA	1882	387
ESC_NA2083AA	349	670
ESC_NA2082AA	297	234
ESC_NA2081AA	349	670
ESC_NA2080AA	297	234
ESC_NA2079AA	350	50
ESC_NA2078AA	297	234
ESC_NA2077AA	369	156
ESC_NA2076AA	117	592

ESC_NA2074AA	69	387
ESC_NA2073AA	428	654
ESC_NA2072AA	1485	408
ESC_NA2071AA	9707	343
ESC_NA2070AA	6949	765
ESC_NA2069AA	1618	736
ESC_NA2068AA	349	670
ESC_NA2067AA	6949	765
ESC_NA2066AA	9707	343
ESC_NA2065AA	355	764
ESC_NA2064AA	974	160
ESC_NA2063AA	117	592
ESC_NA2062AA	428	654
ESC_NA2060AA	69	387
ESC_NA2059AA	1618	736
ESC_NA2058AA	1844	198
ESC_NA2057AA	428	654
ESC_NA2056AA	69	387
ESC_NA2055AA	1618	736
ESC_NA2053AA	1594	71
ESC_NA2051AA	6949	765
ESC_NA2049AA	369	156
ESC_NA2048AA	355	764
ESC_NA2046AA	974	160
ESC_NA2044AA	369	156
ESC_NA2043AA	355	764
ESC_NA2041AA	117	592
ESC_NA2040AA	350	50
ESC_NA2039AA	350	50
ESC_NA2038AA	117	592
ESC_NA2037AA	6949	765
ESC_NA2036AA	23	156
ESC_NA2035AA	351	268
ESC_NA2034AA	428	654
ESC_NA2033AA	1629	924
ESC_NA2032AA	196	257
ESC_NA2031AA	131	653
ESC_NA2030AA	369	156
ESC_NA2029AA	131	653
ESC_NA2028AA	428	654
ESC_NA2027AA	69	387
ESC_NA2026AA	978	740
ESC_NA2025AA	2556	630
ESC_NA2024AA	350	50
ESC_NA2023AA	38	669
ESC_NA2022AA	616	156
ESC_NA2021AA	117	592
ESC_NA2020AA	38	669
ESC_NA2019AA	355	764
ESC_NA2018AA	428	654

ESC_NA2017AA	108	257
ESC_NA2016AA	369	156
ESC_NA2015AA	616	156
ESC_NA2014AA	23	156
ESC_NA2013AA	69	387
ESC_NA2012AA	9704	387
ESC_NA2011AA	349	670
ESC_NA2010AA	131	653
ESC_NA2009AA	9346	286
ESC_NA2008AA	3580	257
ESC_NA2007AA	69	387
ESC_NA2006AA	101	696
ESC_NA2005AA	117	592
ESC_NA2004AA	349	670
ESC_NA2003AA	974	160
ESC_NA2002AA	131	653
ESC_NA2001AA	141	644
ESC_NA2000AA	131	653
ESC_NA1999AA	155	175
ESC_NA1998AA	23	156
ESC_NA1997AA	117	592
ESC_NA1996AA	131	653
ESC_NA1995AA	919	421
ESC_NA1994AA	131	653
ESC_NA1992AA	23	156
ESC_NA1991AA	355	764
ESC_NA1990AA	131	653
ESC_NA1989AA	1618	736
ESC_NA1988AA	428	654
ESC_NA1987AA	1914	956
ESC_NA1986AA	1914	956
ESC_NA1985AA	2792	886
ESC_NA1984AA	428	654
ESC_NA1983AA	355	764
ESC_NA1982AA	355	764
ESC_NA1981AA	131	653
ESC_NA1980AA	428	654
ESC_NA1979AA	131	653
ESC_NA1978AA	349	670
ESC_NA1977AA	117	592
ESC_NA1976AA	428	654
ESC_NA1975AA	1618	736
ESC_NA1974AA	23	156
ESC_NA1973AA	1618	736
ESC_NA1971AA	23	156
ESC_NA1970AA	23	156
ESC_NA1969AA	23	156
ESC_NA1968AA	428	654
ESC_NA1967AA	131	653
ESC_NA1966AA	23	156

ESC_NA1965AA	23	156
ESC_NA1964AA	131	653
ESC_NA1963AA	428	654
ESC_NA1962AA	23	156
ESC_NA1961AA	428	654
ESC_NA1960AA	23	156
ESC_NA1959AA	23	156
ESC_NA1958AA	23	156
ESC_NA1957AA	131	653
ESC_NA1956AA	428	654
ESC_NA1955AA	131	653
ESC_NA1954AA	117	592
ESC_NA1953AA	23	156
ESC_NA1952AA	23	156
ESC_NA1951AA	23	156
ESC_NA1950AA	428	654
ESC_NA1949AA	428	654
ESC_NA1948AA	131	653
ESC_NA1947AA	88	160
ESC_NA1945AA	23	156
ESC_NA1944AA	355	764
ESC_NA1943AA	23	156
ESC_NA1942AA	131	653
ESC_NA1941AA	23	156
ESC_NA1940AA	23	156
ESC_NA1939AA	155	175
ESC_NA1938AA	23	156
ESC_NA1937AA	23	156
ESC_NA1936AA	23	156
ESC_NA1935AA	355	764
ESC_NA1934AA	23	156
ESC_NA1933AA	355	764
ESC_NA1932AA	23	156
ESC_NA1931AA	131	653
ESC_NA1930AA	23	156
ESC_NA1929AA	131	653
ESC_NA1928AA	117	592
ESC_NA1927AA	23	156
ESC_NA1926AA	152	257
ESC_NA1925AA	152	257
ESC_NA1924AA	245	261
ESC_NA1923AA	152	257
ESC_NA1922AA	245	261
ESC_NA1921AA	245	261
ESC_NA1920AA	245	261
ESC_NA1919AA	245	261
ESC_NA1918AA	152	257
ESC_NA1917AA	245	261
ESC_NA1916AA	152	257
ESC_NA1915AA	152	257

ESC_NA1914AA	152	257
ESC_NA1913AA	1273	256
ESC_NA1912AA	152	257
ESC_NA1911AA	5390	83
ESC_NA1910AA	753	842
ESC_NA1909AA	306	286
ESC_NA1908AA	306	286
ESC_NA1907AA	33	198
ESC_NA1906AA	278	924
ESC_NA1905AA	3593	153
ESC_NA1904AA	1041	765
ESC_NA1903AA	10	512
ESC_NA1902AA	38	669
ESC_NA1901AA	301	307
ESC_NA1900AA	404	761
ESC_NA1899AA	17	191
ESC_NA1898AA	301	307
ESC_NA1897AA	3101	160
ESC_NA1896AA	306	286
ESC_NA1895AA	25	234
ESC_NA1894AA	9700	198
ESC_NA1893AA	583	768
ESC_NA1892AA	9701	261
ESC_NA1891AA	25	234
ESC_NA1890AA	9703	99
ESC_NA1889AA	9352	141
ESC_NA1888AA	9702	53
ESC_NA1887AA	162	924
ESC_NA1886AA	355	764
ESC_NA1885AA	131	653
ESC_NA1884AA	117	592
ESC_NA1883AA	428	654
ESC_NA1882AA	355	764
ESC_NA1881AA	847	257
ESC_NA1880AA	355	764
ESC_NA1879AA	428	654
ESC_NA1878AA	428	654
ESC_NA1877AA	428	654
ESC_NA1876AA	349	670
ESC_NA1875AA	117	592
ESC_NA1874AA	355	764
ESC_NA1873AA	349	670
ESC_NA1872AA	8951	943
ESC_NA1870AA	131	653
ESC_NA1869AA	428	654
ESC_NA1868AA	616	156
ESC_NA1867AA	23	156
ESC_NA1866AA	974	160
ESC_NA1865AA	428	654
ESC_NA1864AA	428	654

ESC_NA1863AA	428	654
ESC_NA1862AA	355	764
ESC_NA1861AA	428	654
ESC_NA1860AA	117	592
ESC_NA1859AA	23	156
ESC_NA1856AA	349	670
ESC_NA1854AA	1159	381
ESC_NA1851AA	38	669
ESC_NA1849AA	117	592
ESC_NA1848AA	6949	765
ESC_NA1846AA	962	198
ESC_NA1845AA	131	653
ESC_NA1844AA	1485	408
ESC_NA1842AA	131	653
ESC_NA1841AA	23	156
ESC_NA1840AA	131	653
ESC_NA1839AA	1159	381
ESC_NA1838AA	58	198
ESC_NA1837AA	349	670
ESC_NA1836AA	21	156
ESC_NA1835AA	16	156
ESC_NA1834AA	9792	198
ESC_NA1833AA	746	789
ESC_NA1832AA	21	156
ESC_NA1831AA	10	512
ESC_NA1830AA	9791	512
ESC_NA1829AA	21	156
ESC_NA1828AA	21	156
ESC_NA1827AA	21	156
ESC_NA1826AA	21	156
ESC_NA1825AA	21	156
ESC_NA1824AA	94	257
ESC_NA1823AA	29	156
ESC_NA1822AA	21	156
ESC_NA1821AA	21	156
ESC_NA1820AA	21	156
ESC_NA1819AA	29	156
ESC_NA1818AA	10	512
ESC_NA1817AA	1	667
ESC_NA1816AA	6365	696
ESC_NA1815AA	10	512
ESC_NA1814AA	90	156
ESC_NA1813AA	641	935
ESC_NA1812AA	10	512
ESC_NA1811AA	10	512
ESC_NA1810AA	635	141
ESC_NA1809AA	401	142
ESC_NA1808AA	152	257
ESC_NA1807AA	152	257
ESC_NA1806AA	245	261

ESC_NA1805AA	245	261
ESC_NA1804AA	245	261
ESC_NA1803AA	245	261
ESC_NA1802AA	152	257
ESC_NA1801AA	152	257
ESC_NA1800AA	9317	257
ESC_NA1799AA	152	257
ESC_NA1798AA	17	191
ESC_NA1797AA	5681	286
ESC_NA1796AA	17	191
ESC_NA1795AA	16	156
ESC_NA1794AA	21	156
ESC_NA1793AA	21	156
ESC_NA1792AA	21	156
ESC_NA1791AA	6671	297
ESC_NA1790AA	9790	257
ESC_NA1789AA	11	936
ESC_NA1788AA	1967	191
ESC_NA1787AA	21	156
ESC_NA1786AA	21	156
ESC_NA1785AA	21	156
ESC_NA1784AA	655	926
ESC_NA1783AA	21	156
ESC_NA1782AA	343	286
ESC_NA1781AA	16	156
ESC_NA1780AA	3057	672
ESC_NA1779AA	32	270
ESC_NA1778AA	21	156
ESC_NA1777AA	17	191
ESC_NA1776AA	355	764
ESC_NA1775AA	11	936
ESC_NA1774AA	11	936
ESC_NA1773AA	29	156
ESC_NA1772AA	11	936
ESC_NA1771AA	17	191
ESC_NA1770AA	90	156
ESC_NA1769AA	10	512
ESC_NA1768AA	90	156
ESC_NA1767AA	10	512
ESC_NA1766AA	3546	512
ESC_NA1765AA	1	667
ESC_NA1764AA	10	512
ESC_NA1763AA	90	156
ESC_NA1762AA	245	261
ESC_NA1761AA	152	257
ESC_NA1760AA	245	261
ESC_NA1759AA	152	257
ESC_NA1758AA	152	257
ESC_NA1757AA	152	257
ESC_NA1756AA	152	257

ESC_NA1755AA	243	197
ESC_NA1754AA	245	261
ESC_NA1753AA	152	257
ESC_NA1752AA	1025	261
ESC_NA1751AA	245	261
ESC_NA1750AA	245	261
ESC_NA1749AA	152	257
ESC_NA1748AA	152	257
ESC_NA1747AA	119	286
ESC_NA1746AA	442	257
ESC_NA1744AA	137	270
ESC_NA1743AA	2836	221
ESC_NA1742AA	442	257
ESC_NA1741AA	278	924
ESC_NA1740AA	11	936
ESC_NA1739AA	10	512
ESC_NA1738AA	17	191
ESC_NA1736AA	13	257
ESC_NA1735AA	675	234
ESC_NA1734AA	17	191
ESC_NA1733AA	738	598
ESC_NA1732AA	11	936
ESC_NA1731AA	11	936
ESC_NA1730AA	11	936
ESC_NA1729AA	409	504
ESC_NA1728AA	409	504
ESC_NA1726AA	409	504
ESC_NA1725AA	155	175
ESC_NA1724AA	155	175
ESC_NA1723AA	9697	198
ESC_NA1722AA	9698	738
ESC_NA1721AA	9697	198
ESC_NA1720AA	59	217
ESC_NA1719AA	9698	738
ESC_NA1718AA	9697	198
ESC_NA1717AA	2705	504
ESC_NA1716AA	1431	257
ESC_NA1715AA	2705	504
ESC_NA1714AA	131	653
ESC_NA1713AA	2705	504
ESC_NA1712AA	940	257
ESC_NA1711AA	940	257
ESC_NA1710AA	940	257
ESC_NA1709AA	216	512
ESC_NA1708AA	401	142
ESC_NA1707AA	569	738
ESC_NA1706AA	409	504
ESC_NA1705AA	399	198
ESC_NA1704AA	1316	471
ESC_NA1703AA	21	156

ESC_NA1702AA	29	156
ESC_NA1701AA	21	156
ESC_NA1700AA	21	156
ESC_NA1699AA	21	156
ESC_NA1698AA	29	156
ESC_NA1697AA	21	156
ESC_NA1696AA	29	156
ESC_NA1695AA	29	156
ESC_NA1694AA	21	156
ESC_NA1693AA	21	156
ESC_NA1692AA	152	257
ESC_NA1691AA	1273	256
ESC_NA1690AA	152	257
ESC_NA1689AA	152	257
ESC_NA1688AA	10	512
ESC_NA1687AA	1324	156
ESC_NA1686AA	5493	198
ESC_NA1685AA	728	355
ESC_NA1684AA	9696	968
ESC_NA1683AA	9694	286
ESC_NA1681AA	9693	18
ESC_NA1680AA	9692	234
ESC_NA1679AA	1114	504
ESC_NA1677AA	10	512
ESC_NA1675AA	48	76
ESC_NA1674AA	10	512
ESC_NA1672AA	191	198
ESC_NA1671AA	401	142
ESC_NA1670AA	450	71
ESC_NA1669AA	635	141
ESC_NA1668AA	10	512
ESC_NA1667AA	9665	204
ESC_NA1666AA	8577	445
ESC_NA1664AA	4684	257
ESC_NA1663AA	9666	359
ESC_NA1662AA	9668	512
ESC_NA1661AA	9667	789
ESC_NA1659AA	9661	453
ESC_NA1658AA	9664	257
ESC_NA1657AA	9662	396
ESC_NA1656AA	8395	537
ESC_NA1654AA	9660	447
ESC_NA1653AA	9663	386
ESC_NA1652AA	101	696
ESC_NA1650AA	2659	679
ESC_NA1648AA	410	156
ESC_NA1645AA	152	257
ESC_NA1642AA	11	936
ESC_NA1641AA	11	936
ESC_NA1640AA	4554	738

ESC_NA1639AA	11	936
ESC_NA1638AA	11	936
ESC_NA1637AA	21	156
ESC_NA1636AA	23	156
ESC_NA1635AA	677	257
ESC_NA1634AA	21	156
ESC_NA1633AA	14	761
ESC_NA1632AA	88	160
ESC_NA1631AA	10	512
ESC_NA1630AA	131	653
ESC_NA1629AA	2914	670
ESC_NA1628AA	14	761
ESC_NA1627AA	73	737
ESC_NA1626AA	95	738
ESC_NA1625AA	10	512
ESC_NA1624AA	9778	738
ESC_NA1623AA	127	738
ESC_NA1622AA	541	701
ESC_NA1621AA	541	701
ESC_NA1620AA	9777	355
ESC_NA1618AA	73	737
ESC_NA1617AA	2678	737
ESC_NA1616AA	710	291
ESC_NA1615AA	69	387
ESC_NA1614AA	73	737
ESC_NA1613AA	205	257
ESC_NA1612AA	73	737
ESC_NA1611AA	69	387
ESC_NA1610AA	14	761
ESC_NA1609AA	69	387
ESC_NA1608AA	4088	198
ESC_NA1607AA	939	198
ESC_NA1606AA	106	387
ESC_NA1605AA	906	198
ESC_NA1604AA	83	740
ESC_NA1603AA	635	141
ESC_NA1602AA	95	738
ESC_NA1601AA	95	738
ESC_NA1600AA	2436	179
ESC_NA1599AA	9784	935
ESC_NA1598AA	93	71
ESC_NA1597AA	10	512
ESC_NA1596AA	88	160
ESC_NA1595AA	541	701
ESC_NA1594AA	12	626
ESC_NA1593AA	95	738
ESC_NA1592AA	569	738
ESC_NA1591AA	1618	736
ESC_NA1590AA	5064	198
ESC_NA1589AA	69	387

ESC_NA1588AA	9783	198
ESC_NA1587AA	95	738
ESC_NA1586AA	59	217
ESC_NA1585AA	14	761
ESC_NA1584AA	34	504
ESC_NA1583AA	14	761
ESC_NA1582AA	10	512
ESC_NA1581AA	681	393
ESC_NA1580AA	131	653
ESC_NA1579AA	127	738
ESC_NA1578AA	155	175
ESC_NA1577AA	48	76
ESC_NA1576AA	58	198
ESC_NA1575AA	710	291
ESC_NA1574AA	1072	355
ESC_NA1573AA	607	355
ESC_NA1572AA	141	644
ESC_NA1571AA	7631	268
ESC_NA1570AA	2017	768
ESC_NA1569AA	540	286
ESC_NA1568AA	127	738
ESC_NA1567AA	38	669
ESC_NA1566AA	1049	175
ESC_NA1565AA	58	198
ESC_NA1564AA	10	512
ESC_NA1563AA	404	761
ESC_NA1562AA	2473	257
ESC_NA1560AA	607	355
ESC_NA1559AA	10	512
ESC_NA1558AA	9782	19
ESC_NA1557AA	58	198
ESC_NA1556AA	607	355
ESC_NA1555AA	91	629
ESC_NA1554AA	9788	653
ESC_NA1553AA	1434	512
ESC_NA1552AA	127	738
ESC_NA1551AA	9781	257
ESC_NA1550AA	9780	216
ESC_NA1549AA	131	653
ESC_NA1548AA	69	387
ESC_NA1547AA	120	180
ESC_NA1546AA	73	737
ESC_NA1545AA	9779	761
ESC_NA1544AA	127	738
ESC_NA1543AA	2325	447
ESC_NA1542AA	127	738
ESC_NA1541AA	635	141
ESC_NA1539AA	10	512
ESC_NA1537AA	1727	257
ESC_NA1536AA	713	422

ESC_NA1535AA	867	198
ESC_NA1533AA	9786	879
ESC_NA1532AA	218	519
ESC_NA1530AA	2356	379
ESC_NA1529AA	75	257
ESC_NA1528AA	446	257
ESC_NA1527AA	10	512
ESC_NA1526AA	278	924
ESC_NA1525AA	3	198
ESC_NA1524AA	73	737
ESC_NA1523AA	12	626
ESC_NA1522AA	788	659
ESC_NA1521AA	279	142
ESC_NA1520AA	144	740
ESC_NA1519AA	335	936
ESC_NA1518AA	4287	512
ESC_NA1517AA	399	198
ESC_NA1516AA	4288	900
ESC_NA1515AA	10	512
ESC_NA1514AA	4280	798
ESC_NA1513AA	10	512
ESC_NA1512AA	73	737
ESC_NA1511AA	93	71
ESC_NA1510AA	10	512
ESC_NA1509AA	10	512
ESC_NA1508AA	10	512
ESC_NA1507AA	1972	406
ESC_NA1506AA	1	667
ESC_NA1505AA	350	50
ESC_NA1504AA	1260	512
ESC_NA1503AA	58	198
ESC_NA1502AA	90	156
ESC_NA1501AA	10	512
ESC_NA1500AA	173	254
ESC_NA1499AA	4281	198
ESC_NA1498AA	10	512
ESC_NA1497AA	5675	71
ESC_NA1496AA	10	512
ESC_NA1494AA	5331	512
ESC_NA1493AA	10	512
ESC_NA1492AA	58	198
ESC_NA1491AA	88	160
ESC_NA1490AA	73	737
ESC_NA1489AA	93	71
ESC_NA1488AA	10	512
ESC_NA1487AA	59	217
ESC_NA1486AA	4247	936
ESC_NA1485AA	1402	512
ESC_NA1484AA	372	424
ESC_NA1483AA	2074	858

ESC_NA1482AA	69	387
ESC_NA1481AA	925	387
ESC_NA1480AA	130	387
ESC_NA1479AA	3478	512
ESC_NA1478AA	90	156
ESC_NA1477AA	10	512
ESC_NA1476AA	5786	513
ESC_NA1475AA	4282	191
ESC_NA1474AA	641	935
ESC_NA1473AA	281	355
ESC_NA1472AA	95	738
ESC_NA1471AA	5492	756
ESC_NA1470AA	6446	601
ESC_NA1469AA	398	445
ESC_NA1466AA	6071	817
ESC_NA1465AA	10	512
ESC_NA1464AA	3107	512
ESC_NA1463AA	59	217
ESC_NA1462AA	399	198
ESC_NA1461AA	280	142
ESC_NA1460AA	452	743
ESC_NA1459AA	1439	628
ESC_NA1458AA	10	512
ESC_NA1457AA	12	626
ESC_NA1456AA	3	198
ESC_NA1455AA	3	198
ESC_NA1454AA	94	257
ESC_NA1452AA	93	71
ESC_NA1451AA	737	175
ESC_NA1450AA	2360	537
ESC_NA1449AA	10	512
ESC_NA1448AA	21	156
ESC_NA1447AA	88	160
ESC_NA1446AA	2336	903
ESC_NA1445AA	372	424
ESC_NA1444AA	10	512
ESC_NA1443AA	4293	212
ESC_NA1442AA	641	935
ESC_NA1440AA	10	512
ESC_NA1439AA	43	512
ESC_NA1438AA	648	359
ESC_NA1437AA	131	653
ESC_NA1436AA	648	359
ESC_NA1434AA	3337	512
ESC_NA1433AA	405	923
ESC_NA1432AA	648	359
ESC_NA1431AA	648	359
ESC_NA1430AA	648	359
ESC_NA1429AA	69	387
ESC_NA1428AA	131	653

ESC_NA1427AA	2338	257
ESC_NA1426AA	38	669
ESC_NA1425AA	38	669
ESC_NA1424AA	405	923
ESC_NA1423AA	5150	661
ESC_NA1422AA	38	669
ESC_NA1421AA	131	653
ESC_NA1420AA	131	653
ESC_NA1418AA	156	175
ESC_NA1417AA	372	424
ESC_NA1416AA	131	653
ESC_NA1415AA	69	387
ESC_NA1414AA	5389	615
ESC_NA1413AA	1340	595
ESC_NA1412AA	624	359
ESC_NA1410AA	131	653
ESC_NA1409AA	38	669
ESC_NA1408AA	354	15
ESC_NA1407AA	443	257
ESC_NA1406AA	12	626
ESC_NA1405AA	131	653
ESC_NA1404AA	744	512
ESC_NA1403AA	423	204
ESC_NA1402AA	2334	701
ESC_NA1401AA	1193	761
ESC_NA1400AA	648	359
ESC_NA1399AA	131	653
ESC_NA1398AA	131	653
ESC_NA1397AA	9787	669
ESC_NA1396AA	4288	900
ESC_NA1395AA	1172	234
ESC_NA1394AA	157	387
ESC_NA1393AA	1193	761
ESC_NA1392AA	131	653
ESC_NA1391AA	410	156
ESC_NA1390AA	648	359
ESC_NA1389AA	2076	400
ESC_NA1388AA	624	359
ESC_NA1387AA	2357	76
ESC_NA1386AA	131	653
ESC_NA1385AA	648	359
ESC_NA1384AA	1434	512
ESC_NA1383AA	131	653
ESC_NA1382AA	405	923
ESC_NA1381AA	405	923
ESC_NA1380AA	131	653
ESC_NA1379AA	131	653
ESC_NA1378AA	14	761
ESC_NA1377AA	131	653
ESC_NA1376AA	131	653

ESC_NA1375AA	130	387
ESC_NA1374AA	131	653
ESC_NA1373AA	517	924
ESC_NA1372AA	131	653
ESC_NA1371AA	423	204
ESC_NA1370AA	48	76
ESC_NA1369AA	405	923
ESC_NA1368AA	131	653
ESC_NA1367AA	131	653
ESC_NA1365AA	131	653
ESC_NA1364AA	131	653
ESC_NA1363AA	405	923
ESC_NA1362AA	315	669
ESC_NA1361AA	40	198
ESC_NA1360AA	6438	65
ESC_NA1359AA	131	653
ESC_NA1358AA	48	76
ESC_NA1357AA	131	653
ESC_NA1356AA	3177	362
ESC_NA1355AA	641	935
ESC_NA1354AA	4283	654
ESC_NA1353AA	1146	198
ESC_NA1351AA	6521	842
ESC_NA1350AA	69	387
ESC_NA1349AA	394	400
ESC_NA1348AA	131	653
ESC_NA1347AA	88	160
ESC_NA1346AA	131	653
ESC_NA1345AA	713	422
ESC_NA1344AA	3	198
ESC_NA1343AA	130	387
ESC_NA1342AA	95	738
ESC_NA1341AA	93	71
ESC_NA1340AA	131	653
ESC_NA1338AA	2792	886
ESC_NA1337AA	69	387
ESC_NA1336AA	58	198
ESC_NA1335AA	23	156
ESC_NA1334AA	95	738
ESC_NA1332AA	131	653
ESC_NA1331AA	421	744
ESC_NA1330AA	131	653
ESC_NA1329AA	48	76
ESC_NA1328AA	537	756
ESC_NA1327AA	452	743
ESC_NA1326AA	73	737
ESC_NA1325AA	48	76
ESC_NA1324AA	131	653
ESC_NA1323AA	773	71
ESC_NA1322AA	10	512

ESC_NA1321AA	2139	789
ESC_NA1320AA	23	156
ESC_NA1319AA	457	595
ESC_NA1318AA	538	629
ESC_NA1317AA	657	592
ESC_NA1316AA	131	653
ESC_NA1315AA	127	738
ESC_NA1314AA	635	141
ESC_NA1313AA	1193	761
ESC_NA1312AA	141	644
ESC_NA1311AA	73	737
ESC_NA1310AA	167	512
ESC_NA1309AA	131	653
ESC_NA1308AA	450	71
ESC_NA1307AA	38	669
ESC_NA1306AA	517	924
ESC_NA1305AA	2350	235
ESC_NA1304AA	95	738
ESC_NA1303AA	357	654
ESC_NA1302AA	362	234
ESC_NA1301AA	10	512
ESC_NA1300AA	10	512
ESC_NA1299AA	131	653
ESC_NA1298AA	9777	355
ESC_NA1297AA	1973	291
ESC_NA1296AA	4110	629
ESC_NA1295AA	23	156
ESC_NA1294AA	9789	328
ESC_NA1293AA	12	626
ESC_NA1292AA	224	198
ESC_NA1291AA	73	737
ESC_NA1290AA	708	924
ESC_NA1289AA	538	629
ESC_NA1288AA	501	900
ESC_NA1287AA	405	923
ESC_NA1286AA	205	257
ESC_NA1285AA	1312	76
ESC_NA1284AA	38	669
ESC_NA1283AA	359	696
ESC_NA1282AA	216	512
ESC_NA1281AA	2619	738
ESC_NA1280AA	73	737
ESC_NA1279AA	1071	175
ESC_NA1277AA	2020	387
ESC_NA1276AA	131	653
ESC_NA1275AA	131	653
ESC_NA1274AA	131	653
ESC_NA1273AA	297	234
ESC_NA1272AA	10	512
ESC_NA1271AA	14	761

ESC_NA1270AA	2163	257
ESC_NA1269AA	401	142
ESC_NA1268AA	127	738
ESC_NA1267AA	131	653
ESC_NA1266AA	216	512
ESC_NA1264AA	641	935
ESC_NA1263AA	216	512
ESC_NA1261AA	2308	257
ESC_NA1260AA	648	359
ESC_NA1259AA	607	355
ESC_NA1258AA	1434	512
ESC_NA1257AA	2041	447
ESC_NA1255AA	10	512
ESC_NA1254AA	73	737
ESC_NA1253AA	23	156
ESC_NA1252AA	635	141
ESC_NA1250AA	93	71
ESC_NA1249AA	93	71
ESC_NA1248AA	93	71
ESC_NA1247AA	93	71
ESC_NA1246AA	245	261
ESC_NA1245AA	21	156
ESC_NA1244AA	5681	286
ESC_NA1243AA	5681	286
ESC_NA1242AA	21	156
ESC_NA1241AA	152	257
ESC_NA1240AA	278	924
ESC_NA1239AA	11	936
ESC_NA1238AA	48	76
ESC_NA1237AA	9181	175
ESC_NA1236AA	372	424
ESC_NA1235AA	1193	761
ESC_NA1234AA	16	156
ESC_NA1233AA	21	156
ESC_NA1232AA	300	286
ESC_NA1231AA	11	936
ESC_NA1230AA	11	936
ESC_NA1229AA	58	198
ESC_NA1228AA	131	653
ESC_NA1227AA	11	936
ESC_NA1226AA	11	936
ESC_NA1225AA	9776	297
ESC_NA1224AA	21	156
ESC_NA1223AA	154	257
ESC_NA1222AA	21	156
ESC_NA1221AA	16	156
ESC_NA1220AA	2387	261
ESC_NA1219AA	101	696
ESC_NA1218AA	3234	257
ESC_NA1217AA	73	737

ESC_NA1216AA	73	737
ESC_NA1215AA	73	737
ESC_NA1214AA	372	424
ESC_NA1213AA	73	737
ESC_NA1212AA	73	737
ESC_NA1211AA	16	156
ESC_NA1210AA	21	156
ESC_NA1209AA	6490	847
ESC_NA1208AA	17	191
ESC_NA1207AA	1585	512
ESC_NA1206AA	80	731
ESC_NA1205AA	1585	512
ESC_NA1204AA	58	198
ESC_NA1203AA	372	424
ESC_NA1202AA	372	424
ESC_NA1201AA	297	234
ESC_NA1200AA	1585	512
ESC_NA1199AA	646	738
ESC_NA1198AA	1585	512
ESC_NA1197AA	1257	649
ESC_NA1196AA	73	737
ESC_NA1195AA	9775	924
ESC_NA1194AA	372	424
ESC_NA1193AA	372	424
ESC_NA1192AA	9774	740
ESC_NA1191AA	12	626
ESC_NA1190AA	69	387
ESC_NA1189AA	747	640
ESC_NA1188AA	1585	512
ESC_NA1187AA	167	512
ESC_NA1186AA	5416	445
ESC_NA1185AA	34	504
ESC_NA1184AA	167	512
ESC_NA1183AA	1585	512
ESC_NA1182AA	1585	512
ESC_NA1181AA	5416	445
ESC_NA1179AA	9656	71
ESC_NA1178AA	624	359
ESC_NA1177AA	131	653
ESC_NA1176AA	131	653
ESC_NA1175AA	131	653
ESC_NA1173AA	3570	156
ESC_NA1172AA	401	142
ESC_NA1171AA	635	141
ESC_NA1170AA	10	512
ESC_NA1168AA	9716	356
ESC_NA1167AA	9655	198
ESC_NA1166AA	10	512
ESC_NA1165AA	5295	355
ESC_NA1164AA	10	512

ESC_NA1163AA	3168	261
ESC_NA1162AA	216	512
ESC_NA1161AA	206	286
ESC_NA1160AA	398	445
ESC_NA1159AA	191	198
ESC_NA1158AA	10	512
ESC_NA1157AA	635	141
ESC_NA1156AA	773	71
ESC_NA1155AA	409	504
ESC_NA1154AA	46	447
ESC_NA1152AA	8677	483
ESC_NA1151AA	245	261
ESC_NA1150AA	245	261
ESC_NA1149AA	17	191
ESC_NA1148AA	11	936
ESC_NA1147AA	2514	504
ESC_NA1146AA	657	592
ESC_NA1145AA	4736	87
ESC_NA1144AA	401	142
ESC_NA1143AA	152	257
ESC_NA1142AA	245	261
ESC_NA1141AA	245	261
ESC_NA1140AA	245	261
ESC_NA1139AA	21	156
ESC_NA1138AA	21	156
ESC_NA1137AA	127	738
ESC_NA1136AA	295	160
ESC_NA1135AA	16	156
ESC_NA1134AA	29	156
ESC_NA1133AA	29	156
ESC_NA1132AA	21	156
ESC_NA1131AA	16	156
ESC_NA1130AA	21	156
ESC_NA1129AA	29	156
ESC_NA1128AA	21	156
ESC_NA1127AA	21	156
ESC_NA1126AA	17	191
ESC_NA1125AA	5374	156
ESC_NA1124AA	21	156
ESC_NA1123AA	21	156
ESC_NA1122AA	21	156
ESC_NA1121AA	9734	142
ESC_NA1120AA	9734	142
ESC_NA1119AA	152	257
ESC_NA1118AA	4213	251
ESC_NA1117AA	10	512
ESC_NA1116AA	10	512
ESC_NA1115AA	607	355
ESC_NA1114AA	117	592
ESC_NA1113AA	590	156

ESC_NA1112AA	1792	156
ESC_NA1110AA	245	261
ESC_NA1109AA	17	191
ESC_NA1108AA	655	926
ESC_NA1107AA	21	156
ESC_NA1106AA	17	191
ESC_NA1105AA	17	191
ESC_NA1104AA	21	156
ESC_NA1103AA	69	387
ESC_NA1102AA	16	156
ESC_NA1101AA	25	234
ESC_NA1100AA	21	156
ESC_NA1099AA	16	156
ESC_NA1098AA	11	936
ESC_NA1097AA	11	936
ESC_NA1088AA	678	257
ESC_NA1087AA	442	257
ESC_NA1086AA	442	257
ESC_NA1085AA	659	286
ESC_NA1080AA	1244	291
ESC_NA1079AA	10	512
ESC_NA1078AA	10	512
ESC_NA1077AA	1968	76
ESC_NA1076AA	10	512
ESC_NA1073AA	10	512
ESC_NA1072AA	10	512
ESC_NA1070AA	10	512
ESC_NA1069AA	10	512
ESC_NA1066AA	971	198
ESC_NA1065AA	716	795
ESC_NA1064AA	165	504
ESC_NA1063AA	1244	291
ESC_NA1062AA	641	935
ESC_NA1061AA	165	504
ESC_NA1059AA	117	592
ESC_NA1055AA	3203	76
ESC_NA1053AA	101	696
ESC_NA1052AA	1244	291
ESC_NA1051AA	152	257
ESC_NA1050AA	245	261
ESC_NA1049AA	21	156
ESC_NA1048AA	21	156
ESC_NA1047AA	21	156
ESC_NA1046AA	2175	212
ESC_NA1045AA	38	669
ESC_NA1044AA	297	234
ESC_NA1043AA	297	234
ESC_NA1042AA	297	234
ESC_NA1041AA	297	234
ESC_NA1040AA	297	234

ESC_NA1039AA	297	234
ESC_NA1038AA	297	234
ESC_NA1037AA	297	234
ESC_NA1036AA	297	234
ESC_NA1034AA	2005	175
ESC_NA1031AA	226	512
ESC_NA1030AA	297	234
ESC_NA1029AA	297	234
ESC_NA1028AA	297	234
ESC_NA1027AA	297	234
ESC_NA1026AA	297	234
ESC_NA1025AA	297	234
ESC_NA1024AA	117	592
ESC_NA1023AA	297	234
ESC_NA1022AA	297	234
ESC_NA1021AA	101	696
ESC_NA1020AA	69	387
ESC_NA1019AA	216	512
ESC_NA1018AA	8645	672
ESC_NA1017AA	8645	672
ESC_NA1016AA	245	261
ESC_NA1015AA	152	257
ESC_NA1014AA	152	257
ESC_NA1013AA	245	261
ESC_NA1012AA	245	261
ESC_NA1011AA	245	261
ESC_NA1010AA	152	257
ESC_NA1009AA	245	261
ESC_NA1008AA	16	156
ESC_NA1007AA	17	191
ESC_NA1006AA	131	653
ESC_NA1005AA	17	191
ESC_NA1004AA	17	191
ESC_NA1003AA	17	191
ESC_NA1002AA	21	156
ESC_NA1001AA	11	936
ESC_NA1000AA	11	936
ESC_NA0999AA	16	156
ESC_NA0998AA	8645	672
ESC_NA0997AA	21	156
ESC_NA0996AA	11	936
ESC_NA0995AA	297	234
ESC_NA0994AA	297	234
ESC_NA0993AA	300	286
ESC_NA0992AA	245	261
ESC_NA0991AA	1273	256
ESC_NA0990AA	152	257
ESC_NA0989AA	245	261
ESC_NA0988AA	152	257
ESC_NA0987AA	630	261

ESC_NA0986AA	245	261
ESC_NA0985AA	152	257
ESC_NA0984AA	630	261
ESC_NA0983AA	152	257
ESC_NA0982AA	243	197
ESC_NA0981AA	245	261
ESC_NA0980AA	152	257
ESC_NA0979AA	245	261
ESC_NA0978AA	245	261
ESC_NA0977AA	152	257
ESC_NA0976AA	145	256
ESC_NA0975AA	152	257
ESC_NA0974AA	34	504
ESC_NA0973AA	21	156
ESC_NA0972AA	33	198
ESC_NA0971AA	200	198
ESC_NA0970AA	11	936
ESC_NA0969AA	32	270
ESC_NA0968AA	10	512
ESC_NA0967AA	33	198
ESC_NA0966AA	656	789
ESC_NA0965AA	25	234
ESC_NA0964AA	16	156
ESC_NA0963AA	11	936
ESC_NA0962AA	17	191
ESC_NA0961AA	11	936
ESC_NA0960AA	162	924
ESC_NA0959AA	17	191
ESC_NA0958AA	32	270
ESC_NA0957AA	21	156
ESC_NA0956AA	16	156
ESC_NA0955AA	11	936
ESC_NA0954AA	11	936
ESC_NA0953AA	11	936
ESC_NA0952AA	11	936
ESC_NA0951AA	11	936
ESC_NA0950AA	11	936
ESC_NA0949AA	11	936
ESC_NA0948AA	11	936
ESC_NA0947AA	1434	512
ESC_NA0946AA	197	601
ESC_NA0945AA	17	191
ESC_NA0944AA	21	156
ESC_NA0943AA	11	936
ESC_NA0942AA	152	257
ESC_NA0941AA	9294	191
ESC_NA0940AA	21	156
ESC_NA0939AA	342	286
ESC_NA0938AA	245	261
ESC_NA0937AA	21	156

ESC_NA0935AA	21	156
ESC_NA0934AA	21	156
ESC_NA0933AA	21	156
ESC_NA0932AA	21	156
ESC_NA0931AA	21	156
ESC_NA0930AA	17	191
ESC_NA0929AA	11	936
ESC_NA0928AA	1967	191
ESC_NA0927AA	17	191
ESC_NA0926AA	17	191
ESC_NA0925AA	21	156
ESC_NA0924AA	21	156
ESC_NA0923AA	21	156
ESC_NA0922AA	16	156
ESC_NA0921AA	21	156
ESC_NA0920AA	1967	191
ESC_NA0919AA	17	191
ESC_NA0918AA	21	156
ESC_NA0917AA	17	191
ESC_NA0916AA	11	936
ESC_NA0915AA	16	156
ESC_NA0914AA	21	156
ESC_NA0913AA	11	936
ESC_NA0912AA	17	191
ESC_NA0911AA	16	156
ESC_NA0910AA	11	936
ESC_NA0909AA	8175	286
ESC_NA0908AA	342	286
ESC_NA0907AA	973	968
ESC_NA0906AA	16	156
ESC_NA0905AA	17	191
ESC_NA0904AA	17	191
ESC_NA0903AA	11	936
ESC_NA0902AA	342	286
ESC_NA0901AA	1967	191
ESC_NA0900AA	3570	156
ESC_NA0899AA	5292	738
ESC_NA0898AA	16	156
ESC_NA0897AA	11	936
ESC_NA0896AA	21	156
ESC_NA0895AA	410	156
ESC_NA0894AA	152	257
ESC_NA0893AA	152	257
ESC_NA0892AA	245	261
ESC_NA0891AA	245	261
ESC_NA0890AA	5400	261
ESC_NA0889AA	152	257
ESC_NA0888AA	152	257
ESC_NA0887AA	245	261
ESC_NA0886AA	152	257

ESC_NA0885AA	245	261
ESC_NA0884AA	152	257
ESC_NA0883AA	245	261
ESC_NA0882AA	152	257
ESC_NA0881AA	1503	257
ESC_NA0880AA	152	257
ESC_NA0879AA	245	261
ESC_NA0878AA	270	204
ESC_NA0877AA	1503	257
ESC_NA0876AA	7375	197
ESC_NA0875AA	245	261
ESC_NA0874AA	145	256
ESC_NA0873AA	145	256
ESC_NA0872AA	245	261
ESC_NA0871AA	245	261
ESC_NA0870AA	152	257
ESC_NA0869AA	152	257
ESC_NA0868AA	152	257
ESC_NA0867AA	152	257
ESC_NA0866AA	145	256
ESC_NA0865AA	245	261
ESC_NA0864AA	245	261
ESC_NA0863AA	245	261
ESC_NA0862AA	152	257
ESC_NA0861AA	152	257
ESC_NA0860AA	152	257
ESC_NA0859AA	245	261
ESC_NA0858AA	152	257
ESC_NA0857AA	245	261
ESC_NA0856AA	152	257
ESC_NA0855AA	245	261
ESC_NA0854AA	152	257
ESC_NA0853AA	152	257
ESC_NA0852AA	245	261
ESC_NA0851AA	152	257
ESC_NA0850AA	245	261
ESC_NA0849AA	245	261
ESC_NA0848AA	152	257
ESC_NA0847AA	152	257
ESC_NA0846AA	152	257
ESC_NA0845AA	152	257
ESC_NA0844AA	245	261
ESC_NA0843AA	152	257
ESC_NA0842AA	245	261
ESC_NA0841AA	145	256
ESC_NA0840AA	245	261
ESC_NA0839AA	245	261
ESC_NA0838AA	1503	257
ESC_NA0837AA	5475	256
ESC_NA0836AA	152	257

ESC_NA0835AA	245	261
ESC_NA0834AA	152	257
ESC_NA0833AA	145	256
ESC_NA0832AA	152	257
ESC_NA0831AA	245	261
ESC_NA0830AA	152	257
ESC_NA0829AA	152	257
ESC_NA0828AA	152	257
ESC_NA0827AA	152	257
ESC_NA0826AA	245	261
ESC_NA0825AA	152	257
ESC_NA0824AA	245	261
ESC_NA0823AA	152	257
ESC_NA0822AA	152	257
ESC_NA0821AA	152	257
ESC_NA0820AA	245	261
ESC_NA0819AA	270	204
ESC_NA0818AA	152	257
ESC_NA0817AA	245	261
ESC_NA0816AA	152	257
ESC_NA0815AA	152	257
ESC_NA0814AA	7039	260
ESC_NA0813AA	152	257
ESC_NA0812AA	131	653
ESC_NA0811AA	131	653
ESC_NA0809AA	131	653
ESC_NA0808AA	131	653
ESC_NA0807AA	131	653
ESC_NA0806AA	9657	512
ESC_NA0805AA	10	512
ESC_NA0804AA	10	512
ESC_NA0803AA	10	512
ESC_NA0802AA	10	512
ESC_NA0801AA	10	512
ESC_NA0800AA	10	512
ESC_NA0799AA	10	512
ESC_NA0798AA	10	512
ESC_NA0797AA	10	512
ESC_NA0796AA	10	512
ESC_NA0795AA	10	512
ESC_NA0794AA	10	512
ESC_NA0793AA	10	512
ESC_NA0792AA	10	512
ESC_NA0791AA	10	512
ESC_NA0790AA	10	512
ESC_NA0789AA	10	512
ESC_NA0788AA	10	512
ESC_NA0787AA	10	512
ESC_NA0786AA	10	512
ESC_NA0785AA	10	512

ESC_NA0784AA	10	512
ESC_NA0783AA	10	512
ESC_NA0782AA	10	512
ESC_NA0781AA	10	512
ESC_NA0780AA	10	512
ESC_NA0779AA	10	512
ESC_NA0778AA	10	512
ESC_NA0777AA	10	512
ESC_NA0776AA	10	512
ESC_NA0775AA	10	512
ESC_NA0774AA	10	512
ESC_NA0773AA	10	512
ESC_NA0772AA	10	512
ESC_NA0771AA	10	512
ESC_NA0770AA	10	512
ESC_NA0769AA	10	512
ESC_NA0768AA	10	512
ESC_NA0767AA	10	512
ESC_NA0766AA	10	512
ESC_NA0765AA	10	512
ESC_NA0764AA	10	512
ESC_NA0763AA	10	512
ESC_NA0762AA	10	512
ESC_NA0761AA	10	512
ESC_NA0760AA	10	512
ESC_NA0759AA	10	512
ESC_NA0758AA	10	512
ESC_NA0757AA	10	512
ESC_NA0756AA	10	512
ESC_NA0755AA	10	512
ESC_NA0753AA	10	512
ESC_NA0752AA	10	512
ESC_NA0751AA	10	512
ESC_NA0750AA	10	512
ESC_NA0749AA	10	512
ESC_NA0748AA	10	512
ESC_NA0747AA	10	512
ESC_NA0745AA	10	512
ESC_NA0744AA	10	512
ESC_NA0741AA	10	512
ESC_NA0740AA	10	512
ESC_NA0739AA	10	512
ESC_NA0738AA	10	512
ESC_NA0737AA	10	512
ESC_NA0736AA	10	512
ESC_NA0735AA	10	512
ESC_NA0734AA	10	512
ESC_NA0733AA	10	512
ESC_NA0732AA	10	512
ESC_NA0730AA	10	512

ESC_NA0729AA	10	512
ESC_NA0728AA	10	512
ESC_NA0727AA	10	512
ESC_NA0726AA	10	512
ESC_NA0725AA	10	512
ESC_NA0724AA	10	512
ESC_NA0723AA	10	512
ESC_NA0722AA	10	512
ESC_NA0721AA	10	512
ESC_NA0720AA	10	512
ESC_NA0719AA	10	512
ESC_NA0718AA	10	512
ESC_NA0717AA	10	512
ESC_NA0716AA	10	512
ESC_NA0715AA	10	512
ESC_NA0714AA	10	512
ESC_NA0713AA	10	512
ESC_NA0712AA	10	512
ESC_NA0711AA	10	512
ESC_NA0710AA	10	512
ESC_NA0709AA	10	512
ESC_NA0708AA	10	512
ESC_NA0707AA	10	512
ESC_NA0706AA	10	512
ESC_NA0705AA	10	512
ESC_NA0704AA	10	512
ESC_NA0703AA	10	512
ESC_NA0702AA	10	512
ESC_NA0701AA	10	512
ESC_NA0700AA	10	512
ESC_NA0699AA	117	592
ESC_NA0698AA	59	217
ESC_NA0697AA	9658	387
ESC_NA0696AA	4267	257
ESC_NA0695AA	442	257
ESC_NA0694AA	4267	257
ESC_NA0693AA	738	598
ESC_NA0692AA	5431	0
ESC_NA0691AA	2101	108
ESC_NA0690AA	11	936
ESC_NA0689AA	11	936
ESC_NA0688AA	16	156
ESC_NA0687AA	442	257
ESC_NA0686AA	16	156
ESC_NA0685AA	11	936
ESC_NA0684AA	11	936
ESC_NA0683AA	38	669
ESC_NA0682AA	675	234
ESC_NA0681AA	404	761
ESC_NA0680AA	11	936

ESC_NA0679AA	26	257
ESC_NA0678AA	21	156
ESC_NA0677AA	738	598
ESC_NA0676AA	6	445
ESC_NA0675AA	11	936
ESC_NA0674AA	7641	738
ESC_NA0673AA	11	936
ESC_NA0672AA	9659	257
ESC_NA0671AA	11	936
ESC_NA0670AA	33	198
ESC_NA0669AA	25	234
ESC_NA0668AA	11	936
ESC_NA0667AA	21	156
ESC_NA0666AA	11	936
ESC_NA0665AA	11	936
ESC_NA0664AA	11	936
ESC_NA0663AA	11	936
ESC_NA0662AA	25	234
ESC_NA0661AA	10	512
ESC_NA0660AA	12	626
ESC_NA0659AA	11	936
ESC_NA0658AA	6	445
ESC_NA0657AA	21	156
ESC_NA0656AA	8579	885
ESC_NA0655AA	11	936
ESC_NA0654AA	44	512
ESC_NA0653AA	20	198
ESC_NA0652AA	302	850
ESC_NA0651AA	117	592
ESC_NA0650AA	58	198
ESC_NA0649AA	744	512
ESC_NA0648AA	2705	504
ESC_NA0647AA	1011	198
ESC_NA0646AA	336	878
ESC_NA0645AA	69	387
ESC_NA0644AA	58	198
ESC_NA0643AA	58	198
ESC_NA0642AA	20	198
ESC_NA0641AA	38	669
ESC_NA0640AA	224	198
ESC_NA0639AA	4138	198
ESC_NA0638AA	38	669
ESC_NA0637AA	542	824
ESC_NA0636AA	58	198
ESC_NA0635AA	69	387
ESC_NA0634AA	641	935
ESC_NA0633AA	616	156
ESC_NA0632AA	12	626
ESC_NA0631AA	301	307
ESC_NA0630AA	301	307

ESC_NA0629AA	301	307
ESC_NA0628AA	301	307
ESC_NA0627AA	11	936
ESC_NA0626AA	301	307
ESC_NA0625AA	11	936
ESC_NA0624AA	11	936
ESC_NA0623AA	56	198
ESC_NA0622AA	11	936
ESC_NA0621AA	21	156
ESC_NA0620AA	301	307
ESC_NA0619AA	301	307
ESC_NA0618AA	301	307
ESC_NA0617AA	301	307
ESC_NA0616AA	152	257
ESC_NA0615AA	152	257
ESC_NA0614AA	245	261
ESC_NA0613AA	7039	260
ESC_NA0611AA	11	936
ESC_NA0610AA	33	198
ESC_NA0609AA	162	924
ESC_NA0608AA	11	936
ESC_NA0607AA	155	175
ESC_NA0606AA	11	936
ESC_NA0605AA	212	198
ESC_NA0604AA	11	936
ESC_NA0603AA	278	924
ESC_NA0602AA	21	156
ESC_NA0601AA	11	936
ESC_NA0600AA	300	286
ESC_NA0599AA	6957	221
ESC_NA0598AA	675	234
ESC_NA0597AA	9654	257
ESC_NA0596AA	11	936
ESC_NA0594AA	115	669
ESC_NA0592AA	245	261
ESC_NA0591AA	152	257
ESC_NA0590AA	152	257
ESC_NA0589AA	542	824
ESC_NA0587AA	617	511
ESC_NA0586AA	540	286
ESC_NA0585AA	398	445
ESC_NA0583AA	617	511
ESC_NA0582AA	542	824
ESC_NA0580AA	155	175
ESC_NA0574AA	10597	0
ESC_NA0573AA	16	156
ESC_NA0572AA	16	156
ESC_NA0571AA	409	504
ESC_NA0570AA	58	198
ESC_NA0569AA	8645	672

ESC_NA0568AA	8645	672
ESC_NA0567AA	148	191
ESC_NA0566AA	152	257
ESC_NA0565AA	152	257
ESC_NA0564AA	245	261
ESC_NA0563AA	152	257
ESC_NA0562AA	152	257
ESC_NA0561AA	152	257
ESC_NA0560AA	11	936
ESC_NA0559AA	11	936
ESC_NA0558AA	11	936
ESC_NA0557AA	11	936
ESC_NA0556AA	11	936
ESC_NA0555AA	1804	935
ESC_NA0554AA	21	156
ESC_NA0553AA	88	160
ESC_NA0552AA	9599	935
ESC_NA0551AA	5133	504
ESC_NA0550AA	9598	76
ESC_NA0549AA	162	924
ESC_NA0548AA	224	198
ESC_NA0547AA	17	191
ESC_NA0546AA	9597	268
ESC_NA0545AA	2328	257
ESC_NA0544AA	165	504
ESC_NA0543AA	34	504
ESC_NA0542AA	4198	878
ESC_NA0541AA	1967	191
ESC_NA0540AA	131	653
ESC_NA0537AA	1286	474
ESC_NA0535AA	1286	474
ESC_NA0534AA	617	511
ESC_NA0533AA	1286	474
ESC_NA0531AA	542	824
ESC_NA0528AA	542	824
ESC_NA0527AA	410	156
ESC_NA0526AA	1114	504
ESC_NA0525AA	167	512
ESC_NA0524AA	48	76
ESC_NA0523AA	165	504
ESC_NA0522AA	9461	504
ESC_NA0521AA	48	76
ESC_NA0520AA	4663	257
ESC_NA0518AA	617	511
ESC_NA0516AA	16	156
ESC_NA0515AA	21	156
ESC_NA0514AA	1326	198
ESC_NA0513AA	101	696
ESC_NA0512AA	17	191
ESC_NA0511AA	29	156

ESC_NA0510AA	17	191
ESC_NA0509AA	10	512
ESC_NA0508AA	2040	198
ESC_NA0506AA	617	511
ESC_NA0502AA	9461	504
ESC_NA0501AA	9461	504
ESC_NA0499AA	617	511
ESC_NA0496AA	1286	474
ESC_NA0494AA	1286	474
ESC_NA0487AA	17	191
ESC_NA0486AA	17	191
ESC_NA0485AA	9524	575
ESC_NA0484AA	1193	761
ESC_NA0482AA	11	936
ESC_NA0481AA	21	156
ESC_NA0480AA	21	156
ESC_NA0479AA	21	156
ESC_NA0478AA	301	307
ESC_NA0477AA	301	307
ESC_NA0172AA	21	156
ESC_NA0171AA	847	257
ESC_NA0170AA	16	156
ESC_NA0169AA	17	191
ESC_NA0168AA	152	257
ESC_NA0167AA	152	257
ESC_NA0166AA	152	257
ESC_NA0165AA	21	156
ESC_NA0164AA	17	191
ESC_NA0163AA	21	156
ESC_NA0162AA	398	445
ESC_NA0161AA	117	592
ESC_NA0160AA	9588	258
ESC_NA0159AA	10	512
ESC_NA0158AA	10	512
ESC_NA0157AA	1	667
ESC_NA0156AA	90	156
ESC_NA0155AA	367	156
ESC_NA0154AA	10	512
ESC_NA0153AA	10	512
ESC_NA0152AA	90	156
ESC_NA0151AA	10	512
ESC_NA0150AA	10	512
ESC_NA0149AA	1308	257
ESC_NA0148AA	1	667
ESC_NA0147AA	100	504
ESC_NA0146AA	1	667
ESC_NA0145AA	10	512
ESC_NA0144AA	10	512
ESC_NA0143AA	90	156
ESC_NA0142AA	1	667

ESC_NA0141AA	90	156
ESC_NA0140AA	42	257
ESC_NA0139AA	100	504
ESC_NA0138AA	2496	789
ESC_NA0137AA	23	156
ESC_NA0136AA	100	504
ESC_NA0135AA	9589	850
ESC_NA0134AA	11	936
ESC_NA0133AA	11	936
ESC_NA0132AA	11	936
ESC_NA0131AA	11	936
ESC_NA0130AA	11	936
ESC_NA0129AA	11	936
ESC_NA0128AA	11	936
ESC_NA0127AA	398	445
ESC_NA0126AA	215	512
ESC_NA0125AA	1123	257
ESC_NA0124AA	38	669
ESC_NA0123AA	327	204
ESC_NA0122AA	58	198
ESC_NA0121AA	131	653
ESC_NA0120AA	224	198
ESC_NA0119AA	2025	194
ESC_NA0118AA	9587	387
ESC_NA0117AA	540	286
ESC_NA0116AA	9586	198
ESC_NA0115AA	101	696
ESC_NA0114AA	155	175
ESC_NA0113AA	442	257
ESC_NA0112AA	10	512
ESC_NA0111AA	93	71
ESC_NA0110AA	154	257
ESC_NA0109AA	2600	445
ESC_NA0108AA	93	71
ESC_NA0107AA	1723	900
ESC_NA0106AA	641	935
ESC_NA0105AA	5826	198
ESC_NA0104AA	2425	257
ESC_NA0103AA	752	512
ESC_NA0102AA	131	653
ESC_NA0101AA	34	504
ESC_NA0100AA	6617	519
ESC_NA0099AA	88	160
ESC_NA0098AA	162	924
ESC_NA0097AA	942	198
ESC_NA0096AA	1286	474
ESC_NA0095AA	155	175
ESC_NA0094AA	46	447
ESC_NA0093AA	1623	891
ESC_NA0092AA	8351	140

ESC_NA0091AA	2952	926
ESC_NA0090AA	1172	234
ESC_NA0089AA	336	878
ESC_NA0088AA	2522	198
ESC_NA0086AA	11	936
ESC_NA0085AA	11	936
ESC_NA0084AA	11	936
ESC_NA0082AA	1286	474
ESC_NA0076AA	617	511
ESC_NA0069AA	245	261
ESC_NA0068AA	21	156
ESC_NA0067AA	16	156
ESC_NA0066AA	297	234
ESC_NA0065AA	297	234
ESC_NA0064AA	297	234
ESC_NA0063AA	297	234
ESC_NA0062AA	747	640
ESC_NA0061AA	17	191
ESC_NA0060AA	11	936
ESC_NA0059AA	34	504
ESC_NA0058AA	2732	234
ESC_NA0057AA	16	156
ESC_NA0056AA	21	156
ESC_NA0055AA	16	156
ESC_NA0054AA	2836	221
ESC_NA0053AA	1490	935
ESC_NA0052AA	21	156
ESC_NA0051AA	32	270
ESC_NA0050AA	31	387
ESC_NA0049AA	152	257
ESC_NA0048AA	9584	257
ESC_NA0047AA	410	156
ESC_NA0046AA	1844	198
ESC_NA0045AA	10	512
ESC_NA0044AA	152	257
ESC_NA0043AA	162	924
ESC_NA0042AA	152	257
ESC_NA0041AA	306	286
ESC_NA0040AA	297	234
ESC_NA0039AA	297	234
ESC_NA0038AA	297	234
ESC_NA0037AA	297	234
ESC_NA0036AA	297	234
ESC_NA0035AA	9074	900
ESC_NA0034AA	73	737
ESC_NA0033AA	21	156
ESC_NA0032AA	95	738
ESC_NA0031AA	655	926
ESC_NA0030AA	1193	761
ESC_NA0029AA	297	234

ESC_NA0028AA	21	156
ESC_NA0027AA	297	234
ESC_NA0026AA	17	191
ESC_NA0025AA	73	737
ESC_NA0024AA	998	644
ESC_NA0023AA	16	156
ESC_NA0022AA	297	234
ESC_NA0021AA	297	234
ESC_NA0020AA	16	156
ESC_NA0019AA	21	156
ESC_NA0018AA	127	738
ESC_NA0017AA	2811	233
ESC_NA0016AA	4747	766
ESC_NA0015AA	655	926
ESC_NA0014AA	297	234
ESC_NA0013AA	155	175
ESC_NA0012AA	48	76
ESC_NA0011AA	2973	234
ESC_NA0010AA	4063	156
ESC_NA0003AA	9461	504
ESC_NA0002AA	9497	291
ESC_MA9999AA	152	257
ESC_MA9998AA	245	261
ESC_MA9997AA	152	257
ESC_MA9996AA	243	197
ESC_MA9995AA	152	257
ESC_MA9994AA	152	257
ESC_MA9993AA	152	257
ESC_MA9991AA	152	257
ESC_MA9990AA	245	261
ESC_MA9989AA	152	257
ESC_MA9988AA	7039	260
ESC_MA9987AA	152	257
ESC_MA9986AA	245	261
ESC_MA9985AA	245	261
ESC_MA9984AA	152	257
ESC_MA9983AA	152	257
ESC_MA9982AA	33	198
ESC_MA9981AA	9653	191
ESC_MA9980AA	4684	257
ESC_MA9979AA	10	512
ESC_MA9978AA	342	286
ESC_MA9977AA	34	504
ESC_MA9976AA	295	160
ESC_MA9975AA	11	936
ESC_MA9974AA	11	936
ESC_MA9973AA	11	936
ESC_MA9972AA	718	257
ESC_MA9971AA	718	257
ESC_MA9970AA	515	1004

ESC_MA9969AA	3759	175
ESC_MA9968AA	3759	175
ESC_MA9967AA	11	936
ESC_MA9966AA	11	936
ESC_MA9965AA	11	936
ESC_MA9964AA	11	936
ESC_MA9796AA	8262	234
ESC_MA9790AA	245	261
ESC_MA9789AA	145	256
ESC_MA9788AA	152	257
ESC_MA9787AA	245	261
ESC_MA9786AA	245	261
ESC_MA9785AA	152	257
ESC_MA9784AA	152	257
ESC_MA9783AA	152	257
ESC_MA9782AA	148	191
ESC_MA9781AA	152	257
ESC_MA9780AA	152	257
ESC_MA9779AA	152	257
ESC_MA9778AA	245	261
ESC_MA9777AA	245	261
ESC_MA9776AA	152	257
ESC_MA9775AA	245	261
ESC_MA9774AA	152	257
ESC_MA9773AA	152	257
ESC_MA9772AA	540	286
ESC_MA9771AA	540	286
ESC_MA9770AA	398	445
ESC_MA9769AA	540	286
ESC_MA9767AA	398	445
ESC_MA9765AA	540	286
ESC_MA9764AA	540	286
ESC_MA9762AA	9891	445
ESC_MA9761AA	540	286
ESC_MA9759AA	1727	257
ESC_MA9758AA	540	286
ESC_MA9757AA	398	445
ESC_MA9756AA	1967	191
ESC_MA9755AA	21	156
ESC_MA9754AA	32	270
ESC_MA9753AA	21	156
ESC_MA9752AA	21	156
ESC_MA9751AA	32	270
ESC_MA9750AA	21	156
ESC_MA9749AA	11	936
ESC_MA9748AA	21	156
ESC_MA9747AA	2521	257
ESC_MA9746AA	6467	452
ESC_MA9745AA	135	653
ESC_MA9744AA	8084	801

ESC_MA9743AA	44	512
ESC_MA9742AA	9574	427
ESC_MA9741AA	4049	234
ESC_MA9740AA	9576	331
ESC_MA9739AA	135	653
ESC_MA9738AA	753	842
ESC_MA9737AA	906	198
ESC_MA9736AA	1724	863
ESC_MA9735AA	2966	936
ESC_MA9734AA	17	191
ESC_MA9733AA	11	936
ESC_MA9732AA	33	198
ESC_MA9731AA	3101	160
ESC_MA9730AA	442	257
ESC_MA9729AA	11	936
ESC_MA9728AA	278	924
ESC_MA9727AA	738	598
ESC_MA9726AA	11	936
ESC_MA9725AA	4748	234
ESC_MA9724AA	21	156
ESC_MA9723AA	21	156
ESC_MA9722AA	33	198
ESC_MA9721AA	21	156
ESC_MA9720AA	33	198
ESC_MA9719AA	9575	191
ESC_MA9718AA	655	926
ESC_MA9717AA	21	156
ESC_MA9716AA	4577	257
ESC_MA9715AA	10	512
ESC_MA9714AA	10	512
ESC_MA9713AA	2325	447
ESC_MA9712AA	34	504
ESC_MA9711AA	117	592
ESC_MA9710AA	685	311
ESC_MA9709AA	2425	257
ESC_MA9708AA	9577	824
ESC_MA9707AA	1721	311
ESC_MA9706AA	10	512
ESC_MA9700AA	10	512
ESC_MA9699AA	10	512
ESC_MA9692AA	10	512
ESC_MA9669AA	1081	198
ESC_MA9666AA	156	175
ESC_MA9655AA	4014	696
ESC_MA9651AA	617	511
ESC_MA9635AA	9461	504
ESC_MA9629AA	152	257
ESC_MA9628AA	1967	191
ESC_MA9627AA	11	936
ESC_MA9626AA	11	936

ESC_MA9625AA	11	936
ESC_MA9620AA	245	261
ESC_MA9619AA	245	261
ESC_MA9618AA	245	261
ESC_MA9617AA	245	261
ESC_MA9616AA	152	257
ESC_MA9615AA	288	102
ESC_MA9614AA	245	261
ESC_MA9613AA	617	511
ESC_MA9612AA	617	511
ESC_MA9608AA	226	512
ESC_MA9606AA	226	512
ESC_MA9605AA	617	511
ESC_MA9604AA	617	511
ESC_MA9599AA	617	511
ESC_MA9598AA	9437	923
ESC_MA9597AA	11	936
ESC_MA9596AA	152	257
ESC_MA9595AA	152	257
ESC_MA9594AA	152	257
ESC_MA9593AA	152	257
ESC_MA9592AA	17	191
ESC_MA9591AA	447	198
ESC_MA9590AA	17	191
ESC_MA9589AA	21	156
ESC_MA9588AA	101	696
ESC_MA9587AA	11	936
ESC_MA9586AA	747	640
ESC_MA9585AA	17	191
ESC_MA9584AA	11	936
ESC_MA9583AA	11	936
ESC_MA9582AA	1844	198
ESC_MA9581AA	16	156
ESC_MA9580AA	16	156
ESC_MA9579AA	17	191
ESC_MA9578AA	17	191
ESC_MA9577AA	245	261
ESC_MA9576AA	152	257
ESC_MA9575AA	245	261
ESC_MA9573AA	245	261
ESC_MA9572AA	245	261
ESC_MA9571AA	245	261
ESC_MA9570AA	6200	261
ESC_MA9569AA	152	257
ESC_MA9568AA	10	512
ESC_MA9567AA	10	512
ESC_MA9566AA	120	180
ESC_MA9565AA	10	512
ESC_MA9564AA	117	592
ESC_MA9563AA	11	936

ESC_MA9562AA	448	257
ESC_MA9561AA	10	512
ESC_MA9560AA	1091	512
ESC_MA9559AA	58	198
ESC_MA9558AA	10	512
ESC_MA9557AA	10	512
ESC_MA9556AA	120	180
ESC_MA9555AA	10	512
ESC_MA9554AA	973	968
ESC_MA9553AA	3476	512
ESC_MA9552AA	10	512
ESC_MA9551AA	10	512
ESC_MA9550AA	127	738
ESC_MA9549AA	295	160
ESC_MA9548AA	5785	512
ESC_MA9547AA	56	198
ESC_MA9546AA	1590	982
ESC_MA9545AA	120	180
ESC_MA9544AA	410	156
ESC_MA9543AA	10	512
ESC_MA9542AA	10	512
ESC_MA9541AA	5785	512
ESC_MA9540AA	73	737
ESC_MA9539AA	95	738
ESC_MA9538AA	120	180
ESC_MA9537AA	10	512
ESC_MA9536AA	10	512
ESC_MA9535AA	10	512
ESC_MA9534AA	10	512
ESC_MA9533AA	10	512
ESC_MA9532AA	10	512
ESC_MA9531AA	90	156
ESC_MA9530AA	10	512
ESC_MA9529AA	127	738
ESC_MA9528AA	10	512
ESC_MA9527AA	925	387
ESC_MA9526AA	316	257
ESC_MA9525AA	56	198
ESC_MA9524AA	10	512
ESC_MA9523AA	13	257
ESC_MA9522AA	90	156
ESC_MA9521AA	5785	512
ESC_MA9520AA	10	512
ESC_MA9519AA	100	504
ESC_MA9518AA	88	160
ESC_MA9517AA	58	198
ESC_MA9516AA	10	512
ESC_MA9515AA	155	175
ESC_MA9145AA	3476	512
ESC_MA9144AA	10	512

ESC_MA9143AA	10	512
ESC_MA9142AA	90	156
ESC_MA9141AA	10	512
ESC_MA9140AA	93	71
ESC_MA9139AA	6535	207
ESC_MA9138AA	85	234
ESC_MA9137AA	5785	512
ESC_MA9136AA	90	156
ESC_MA9135AA	10	512
ESC_MA9134AA	1079	235
ESC_MA9133AA	21	156
ESC_MA9132AA	1011	198
ESC_MA9131AA	711	935
ESC_MA9130AA	10	512
ESC_MA9129AA	1638	519
ESC_MA9128AA	69	387
ESC_MA9127AA	10	512
ESC_MA9125AA	1638	519
ESC_MA9124AA	34	504
ESC_MA9123AA	1485	408
ESC_MA9122AA	5871	824
ESC_MA9121AA	101	696
ESC_MA9120AA	162	924
ESC_MA9119AA	10	512
ESC_MA9118AA	8540	76
ESC_MA9117AA	48	76
ESC_MA9116AA	2253	71
ESC_MA9114AA	744	512
ESC_MA9113AA	69	387
ESC_MA9112AA	224	198
ESC_MA9111AA	48	76
ESC_MA9109AA	206	286
ESC_MA9108AA	744	512
ESC_MA9107AA	744	512
ESC_MA9106AA	542	824
ESC_MA9105AA	5995	504
ESC_MA9104AA	7122	519
ESC_MA9102AA	101	696
ESC_MA9101AA	101	696
ESC_MA9100AA	10	512
ESC_MA9099AA	101	696
ESC_MA9097AA	7122	519
ESC_MA9096AA	6756	962
ESC_MA9095AA	58	198
ESC_MA9094AA	648	359
ESC_MA9093AA	1792	156
ESC_MA9092AA	11	936
ESC_MA9091AA	95	738
ESC_MA9090AA	17	191
ESC_MA9089AA	17	191

ESC_MA9088AA	17	191
ESC_MA9087AA	655	926
ESC_MA9086AA	17	191
ESC_MA9085AA	17	191
ESC_MA9084AA	718	257
ESC_MA9083AA	101	696
ESC_MA9082AA	117	592
ESC_MA9081AA	69	387
ESC_MA9080AA	746	789
ESC_MA9079AA	101	696
ESC_MA9078AA	69	387
ESC_MA9077AA	101	696
ESC_MA9076AA	1040	765
ESC_MA9075AA	1193	761
ESC_MA9070AA	9428	291
ESC_MA9069AA	152	257
ESC_MA9068AA	243	197
ESC_MA9067AA	152	257
ESC_MA9066AA	152	257
ESC_MA9065AA	243	197
ESC_MA9064AA	152	257
ESC_MA9063AA	152	257
ESC_MA9062AA	152	257
ESC_MA9061AA	245	261
ESC_MA9060AA	245	261
ESC_MA9058AA	9317	257
ESC_MA9057AA	245	261
ESC_MA9056AA	245	261
ESC_MA9055AA	245	261
ESC_MA9054AA	245	261
ESC_MA9053AA	152	257
ESC_MA9052AA	152	257
ESC_MA9051AA	245	261
ESC_MA9050AA	245	261
ESC_MA9049AA	152	257
ESC_MA9048AA	152	257
ESC_MA9047AA	16	156
ESC_MA9046AA	21	156
ESC_MA9045AA	21	156
ESC_MA9044AA	11	936
ESC_MA9043AA	219	877
ESC_MA9042AA	109	257
ESC_MA9041AA	9621	935
ESC_MA9040AA	9620	198
ESC_MA9039AA	1091	512
ESC_MA9038AA	8253	198
ESC_MA9037AA	131	653
ESC_MA9036AA	949	175
ESC_MA9035AA	8421	261
ESC_MA9034AA	327	204

ESC_MA9033AA	9530	971
ESC_MA9032AA	711	935
ESC_MA9031AA	155	175
ESC_MA9030AA	4151	971
ESC_MA9029AA	446	257
ESC_MA9028AA	58	198
ESC_MA9027AA	457	595
ESC_MA9026AA	515	1004
ESC_MA9025AA	1304	257
ESC_MA9024AA	101	696
ESC_MA9023AA	6046	198
ESC_MA9022AA	8140	878
ESC_MA9021AA	109	257
ESC_MA9020AA	442	257
ESC_MA9019AA	540	286
ESC_MA9018AA	278	924
ESC_MA9017AA	109	257
ESC_MA9016AA	17	191
ESC_MA9015AA	16	156
ESC_MA9014AA	17	191
ESC_MA9013AA	17	191
ESC_MA9012AA	200	198
ESC_MA9011AA	16	156
ESC_MA9010AA	21	156
ESC_MA9009AA	17	191
ESC_MA9008AA	297	234
ESC_MA9007AA	58	198
ESC_MA9006AA	21	156
ESC_MA9005AA	40	198
ESC_MA9004AA	10	512
ESC_MA9003AA	10	512
ESC_MA9002AA	200	198
ESC_MA9001AA	38	669
ESC_MA9000AA	423	204
ESC_MA8999AA	533	198
ESC_MA8998AA	1081	198
ESC_MA8997AA	21	156
ESC_MA8996AA	17	191
ESC_MA8995AA	300	286
ESC_MA8994AA	17	191
ESC_MA8993AA	9287	732
ESC_MA8992AA	1081	198
ESC_MA8991AA	718	257
ESC_MA8990AA	17	191
ESC_MA8989AA	655	926
ESC_MA8988AA	32	270
ESC_MA8987AA	11	936
ESC_MA8986AA	9426	75
ESC_MA8985AA	9425	71
ESC_MA8984AA	9427	506

ESC_MA8983AA	9424	512
ESC_MA8981AA	245	261
ESC_MA8980AA	152	257
ESC_MA8979AA	152	257
ESC_MA8977AA	152	257
ESC_MA8976AA	245	261
ESC_MA8975AA	6666	261
ESC_MA8974AA	245	261
ESC_MA8973AA	245	261
ESC_MA8972AA	245	261
ESC_MA8971AA	152	257
ESC_MA8970AA	152	257
ESC_MA8969AA	152	257
ESC_MA8968AA	152	257
ESC_MA8967AA	245	261
ESC_MA8966AA	152	257
ESC_MA8965AA	152	257
ESC_MA8964AA	152	257
ESC_MA8963AA	11	936
ESC_MA8962AA	21	156
ESC_MA8961AA	21	156
ESC_MA8960AA	245	261
ESC_MA8959AA	11	936
ESC_MA8958AA	17	191
ESC_MA8957AA	152	257
ESC_MA8956AA	245	261
ESC_MA8955AA	152	257
ESC_MA8954AA	245	261
ESC_MA8953AA	245	261
ESC_MA8952AA	245	261
ESC_MA8951AA	152	257
ESC_MA8950AA	245	261
ESC_MA8949AA	245	261
ESC_MA8948AA	152	257
ESC_MA8947AA	245	261
ESC_MA8946AA	245	261
ESC_MA8945AA	245	261
ESC_MA8944AA	145	256
ESC_MA8943AA	152	257
ESC_MA8942AA	245	261
ESC_MA8941AA	152	257
ESC_MA8940AA	245	261
ESC_MA8939AA	152	257
ESC_MA8938AA	152	257
ESC_MA8937AA	152	257
ESC_MA8936AA	152	257
ESC_MA8935AA	245	261
ESC_MA8934AA	21	156
ESC_MA8933AA	10	512
ESC_MA8932AA	16	156

ESC_MA8931AA	937	198
ESC_MA8930AA	11	936
ESC_MA8929AA	33	198
ESC_MA8928AA	33	198
ESC_MA8927AA	11	936
ESC_MA8926AA	2101	108
ESC_MA8925AA	744	512
ESC_MA8924AA	187	257
ESC_MA8923AA	162	924
ESC_MA8922AA	9422	868
ESC_MA8921AA	746	789
ESC_MA8920AA	9421	198
ESC_MA8919AA	224	198
ESC_MA8918AA	6635	71
ESC_MA8917AA	297	234
ESC_MA8916AA	501	900
ESC_MA8915AA	10	512
ESC_MA8914AA	117	592
ESC_MA8913AA	1196	257
ESC_MA8912AA	297	234
ESC_MA8911AA	9420	820
ESC_MA8910AA	155	175
ESC_MA8909AA	2522	198
ESC_MA8908AA	847	257
ESC_MA8907AA	4568	104
ESC_MA8906AA	155	175
ESC_MA8905AA	4568	104
ESC_MA8904AA	155	175
ESC_MA8903AA	10	512
ESC_MA8902AA	155	175
ESC_MA8901AA	1302	817
ESC_MA8900AA	1196	257
ESC_MA8899AA	5766	198
ESC_MA8898AA	155	175
ESC_MA8897AA	6190	198
ESC_MA8896AA	182	246
ESC_MA8895AA	10	512
ESC_MA8894AA	2325	447
ESC_MA8893AA	2690	76
ESC_MA8892AA	13	257
ESC_MA8891AA	48	76
ESC_MA8890AA	40	198
ESC_MA8889AA	1141	512
ESC_MA8888AA	4937	519
ESC_MA8887AA	6636	951
ESC_MA8886AA	398	445
ESC_MA8885AA	9430	1004
ESC_MA8884AA	453	935
ESC_MA8883AA	2175	212
ESC_MA8882AA	448	257

ESC_MA8881AA	446	257
ESC_MA8880AA	540	286
ESC_MA8879AA	641	935
ESC_MA8878AA	2309	669
ESC_MA8877AA	10	512
ESC_MA8876AA	10	512
ESC_MA8875AA	9434	71
ESC_MA8874AA	398	445
ESC_MA8873AA	187	257
ESC_MA8872AA	9429	257
ESC_MA8871AA	9429	257
ESC_MA8870AA	9435	257
ESC_MA8869AA	1298	924
ESC_MA8868AA	2309	669
ESC_MA8867AA	223	198
ESC_MA8866AA	6740	447
ESC_MA8865AA	7020	257
ESC_MA8864AA	9434	71
ESC_MA8863AA	155	175
ESC_MA8862AA	6060	41
ESC_MA8861AA	617	511
ESC_MA8860AA	48	76
ESC_MA8859AA	77	286
ESC_MA8858AA	9433	234
ESC_MA8857AA	4937	519
ESC_MA8856AA	223	198
ESC_MA8855AA	48	76
ESC_MA8854AA	48	76
ESC_MA8853AA	10	512
ESC_MA8852AA	7809	672
ESC_MA8851AA	9432	257
ESC_MA8850AA	746	789
ESC_MA8849AA	58	198
ESC_MA8848AA	9432	257
ESC_MA8847AA	540	286
ESC_MA8846AA	1688	71
ESC_MA8845AA	3856	789
ESC_MA8844AA	9431	286
ESC_MA8843AA	1298	924
ESC_MA8842AA	216	512
ESC_MA8841AA	678	257
ESC_MA8840AA	678	257
ESC_MA8839AA	206	286
ESC_MA8838AA	452	743
ESC_MA8837AA	216	512
ESC_MA8836AA	10	512
ESC_MA8835AA	34	504
ESC_MA8834AA	2936	515
ESC_MA8833AA	9436	76
ESC_MA8832AA	1954	602

ESC_MA8831AA	2728	198
ESC_MA8830AA	5334	512
ESC_MA8829AA	193	795
ESC_MA8828AA	616	156
ESC_MA8827AA	165	504
ESC_MA8826AA	155	175
ESC_MA8825AA	1444	768
ESC_MA8824AA	4623	257
ESC_MA8823AA	2351	234
ESC_MA8822AA	23	156
ESC_MA8821AA	9438	961
ESC_MA8820AA	616	156
ESC_MA8819AA	2351	234
ESC_MA8818AA	2351	234
ESC_MA8817AA	906	198
ESC_MA8816AA	207	512
ESC_MA8815AA	442	257
ESC_MA8814AA	361	512
ESC_MA8813AA	2792	886
ESC_MA8812AA	9443	234
ESC_MA8811AA	297	234
ESC_MA8810AA	110	629
ESC_MA8809AA	361	512
ESC_MA8808AA	48	76
ESC_MA8807AA	4512	71
ESC_MA8806AA	710	291
ESC_MA8805AA	641	935
ESC_MA8804AA	10	512
ESC_MA8803AA	155	175
ESC_MA8802AA	9442	257
ESC_MA8801AA	2522	198
ESC_MA8800AA	6833	445
ESC_MA8799AA	9441	175
ESC_MA8798AA	9440	508
ESC_MA8797AA	58	198
ESC_MA8796AA	10	512
ESC_MA8795AA	10	512
ESC_MA8794AA	4977	71
ESC_MA8793AA	349	670
ESC_MA8792AA	218	519
ESC_MA8791AA	13	257
ESC_MA8790AA	8149	311
ESC_MA8789AA	9439	512
ESC_MA8788AA	10	512
ESC_MA8787AA	10	512
ESC_MA8786AA	708	924
ESC_MA8785AA	540	286
ESC_MA8784AA	3288	286
ESC_MA8783AA	710	291
ESC_MA8782AA	10	512

ESC_MA8781AA	10	512
ESC_MA8780AA	10	512
ESC_MA8779AA	1286	474
ESC_MA8778AA	452	743
ESC_MA8777AA	452	743
ESC_MA8776AA	1726	257
ESC_MA8775AA	6633	764
ESC_MA8774AA	155	175
ESC_MA8773AA	679	257
ESC_MA8772AA	101	696
ESC_MA8771AA	441	198
ESC_MA8770AA	9445	504
ESC_MA8769AA	9444	52
ESC_MA8768AA	3877	512
ESC_MA8767AA	10	512
ESC_MA8766AA	1629	924
ESC_MA8765AA	23	156
ESC_MA8764AA	1011	198
ESC_MA8763AA	6186	868
ESC_MA8762AA	1204	961
ESC_MA8761AA	2521	257
ESC_MA8760AA	1079	235
ESC_MA8759AA	155	175
ESC_MA8758AA	361	512
ESC_MA8757AA	155	175
ESC_MA8756AA	40	198
ESC_MA8755AA	156	175
ESC_MA8754AA	1079	235
ESC_MA8753AA	9448	235
ESC_MA8752AA	10	512
ESC_MA8751AA	207	512
ESC_MA8750AA	156	175
ESC_MA8749AA	88	160
ESC_MA8748AA	101	696
ESC_MA8747AA	4578	286
ESC_MA8746AA	9442	257
ESC_MA8745AA	40	198
ESC_MA8744AA	93	71
ESC_MA8743AA	165	504
ESC_MA8742AA	23	156
ESC_MA8741AA	1079	235
ESC_MA8740AA	7324	257
ESC_MA8739AA	10	512
ESC_MA8738AA	2689	519
ESC_MA8737AA	1638	519
ESC_MA8736AA	372	424
ESC_MA8735AA	297	234
ESC_MA8734AA	9447	789
ESC_MA8733AA	4741	512
ESC_MA8732AA	1288	512

ESC_MA8731AA	101	696
ESC_MA8730AA	8984	257
ESC_MA8729AA	1727	257
ESC_MA8728AA	949	175
ESC_MA8727AA	998	644
ESC_MA8726AA	2614	975
ESC_MA8725AA	2813	756
ESC_MA8724AA	295	160
ESC_MA8723AA	297	234
ESC_MA8722AA	9446	626
ESC_MA8721AA	1851	380
ESC_MA8720AA	939	198
ESC_MA8719AA	201	257
ESC_MA8718AA	210	108
ESC_MA8717AA	3877	512
ESC_MA8716AA	1823	445
ESC_MA8715AA	10	512
ESC_MA8714AA	48	76
ESC_MA8713AA	398	445
ESC_MA8712AA	7285	175
ESC_MA8711AA	9454	198
ESC_MA8710AA	48	76
ESC_MA8709AA	9453	696
ESC_MA8708AA	48	76
ESC_MA8707AA	7285	175
ESC_MA8706AA	616	156
ESC_MA8705AA	2973	234
ESC_MA8704AA	10	512
ESC_MA8703AA	681	393
ESC_MA8702AA	88	160
ESC_MA8701AA	469	257
ESC_MA8700AA	62	574
ESC_MA8699AA	227	504
ESC_MA8698AA	9452	696
ESC_MA8697AA	2175	212
ESC_MA8696AA	542	824
ESC_MA8695AA	398	445
ESC_MA8694AA	409	504
ESC_MA8693AA	9451	160
ESC_MA8692AA	9450	175
ESC_MA8691AA	2008	257
ESC_MA8690AA	216	512
ESC_MA8689AA	793	286
ESC_MA8688AA	9449	291
ESC_MA8687AA	847	257
ESC_MA8686AA	2280	212
ESC_MA8685AA	2522	198
ESC_MA8684AA	641	935
ESC_MA8683AA	48	76
ESC_MA8682AA	9460	233

ESC_MA8681AA	101	696
ESC_MA8680AA	4682	175
ESC_MA8679AA	10	512
ESC_MA8678AA	40	198
ESC_MA8677AA	2522	198
ESC_MA8676AA	4296	844
ESC_MA8675AA	297	234
ESC_MA8674AA	2088	768
ESC_MA8673AA	10	512
ESC_MA8672AA	1431	257
ESC_MA8671AA	4038	257
ESC_MA8670AA	38	669
ESC_MA8669AA	155	175
ESC_MA8668AA	10	512
ESC_MA8667AA	9459	76
ESC_MA8666AA	9458	701
ESC_MA8665AA	694	512
ESC_MA8664AA	164	175
ESC_MA8663AA	1114	504
ESC_MA8662AA	131	653
ESC_MA8661AA	9457	504
ESC_MA8660AA	540	286
ESC_MA8659AA	3274	512
ESC_MA8658AA	1823	445
ESC_MA8657AA	398	445
ESC_MA8656AA	3889	291
ESC_MA8655AA	69	387
ESC_MA8654AA	9456	512
ESC_MA8653AA	10	512
ESC_MA8652AA	2461	76
ESC_MA8651AA	9455	474
ESC_MA8650AA	454	578
ESC_MA8649AA	5122	445
ESC_MA8648AA	4038	257
ESC_MA8647AA	9465	109
ESC_MA8646AA	2165	257
ESC_MA8645AA	3107	512
ESC_MA8644AA	223	198
ESC_MA8643AA	10	512
ESC_MA8642AA	2622	537
ESC_MA8641AA	1304	257
ESC_MA8640AA	517	924
ESC_MA8639AA	541	701
ESC_MA8638AA	155	175
ESC_MA8637AA	9464	165
ESC_MA8636AA	2014	406
ESC_MA8635AA	10	512
ESC_MA8634AA	155	175
ESC_MA8633AA	48	76
ESC_MA8632AA	9463	198

ESC_MA8631AA	1161	764
ESC_MA8630AA	58	198
ESC_MA8629AA	69	387
ESC_MA8628AA	48	76
ESC_MA8627AA	9479	258
ESC_MA8626AA	9481	198
ESC_MA8625AA	9480	445
ESC_MA8624AA	1638	519
ESC_MA8623AA	9479	258
ESC_MA8622AA	196	257
ESC_MA8621AA	1638	519
ESC_MA8620AA	710	291
ESC_MA8619AA	43	512
ESC_MA8618AA	4151	971
ESC_MA8617AA	127	738
ESC_MA8616AA	69	387
ESC_MA8615AA	641	935
ESC_MA8614AA	224	198
ESC_MA8613AA	2005	175
ESC_MA8612AA	2705	504
ESC_MA8611AA	2531	198
ESC_MA8610AA	155	175
ESC_MA8609AA	9478	493
ESC_MA8608AA	10	512
ESC_MA8607AA	6635	71
ESC_MA8606AA	10	512
ESC_MA8605AA	48	76
ESC_MA8604AA	297	234
ESC_MA8603AA	187	257
ESC_MA8602AA	1304	257
ESC_MA8601AA	3270	76
ESC_MA8600AA	9498	257
ESC_MA8599AA	1137	512
ESC_MA8598AA	616	156
ESC_MA8597AA	9499	410
ESC_MA8596AA	9500	789
ESC_MA8595AA	162	924
ESC_MA8594AA	162	924
ESC_MA8593AA	46	447
ESC_MA8592AA	4537	76
ESC_MA8591AA	187	257
ESC_MA8590AA	10	512
ESC_MA8589AA	187	257
ESC_MA8588AA	398	445
ESC_MA8587AA	9498	257
ESC_MA8586AA	399	198
ESC_MA8585AA	187	257
ESC_MA8584AA	1158	387
ESC_MA8583AA	226	512
ESC_MA8582AA	1049	175

ESC_MA8581AA	4481	257
ESC_MA8580AA	1302	817
ESC_MA8579AA	1146	198
ESC_MA8578AA	9505	626
ESC_MA8577AA	1049	175
ESC_MA8576AA	1302	817
ESC_MA8575AA	355	764
ESC_MA8574AA	4977	71
ESC_MA8573AA	155	175
ESC_MA8572AA	9501	156
ESC_MA8571AA	3714	175
ESC_MA8570AA	9443	234
ESC_MA8569AA	48	76
ESC_MA8568AA	10	512
ESC_MA8567AA	9504	175
ESC_MA8566AA	6968	935
ESC_MA8565AA	501	900
ESC_MA8564AA	216	512
ESC_MA8563AA	278	924
ESC_MA8562AA	398	445
ESC_MA8561AA	180	234
ESC_MA8560AA	2539	257
ESC_MA8559AA	1201	512
ESC_MA8558AA	677	257
ESC_MA8557AA	129	257
ESC_MA8556AA	196	257
ESC_MA8555AA	10	512
ESC_MA8554AA	345	175
ESC_MA8552AA	206	286
ESC_MA8551AA	48	76
ESC_MA8550AA	9503	696
ESC_MA8549AA	1196	257
ESC_MA8548AA	9502	519
ESC_MA8547AA	3714	175
ESC_MA8546AA	9507	180
ESC_MA8545AA	4038	257
ESC_MA8544AA	9507	180
ESC_MA8543AA	1298	924
ESC_MA8542AA	10	512
ESC_MA8541AA	10	512
ESC_MA8540AA	297	234
ESC_MA8539AA	10	512
ESC_MA8538AA	9506	682
ESC_MA8537AA	744	512
ESC_MA8536AA	155	175
ESC_MA8535AA	155	175
ESC_MA8534AA	58	198
ESC_MA8533AA	1638	519
ESC_MA8532AA	4038	257
ESC_MA8531AA	3525	504

ESC_MA8530AA	9520	199
ESC_MA8529AA	162	924
ESC_MA8528AA	9519	198
ESC_MA8527AA	1727	257
ESC_MA8526AA	388	156
ESC_MA8525AA	9518	924
ESC_MA8524AA	10	512
ESC_MA8523AA	10	512
ESC_MA8522AA	48	76
ESC_MA8521AA	2178	924
ESC_MA8520AA	616	156
ESC_MA8519AA	297	234
ESC_MA8518AA	165	504
ESC_MA8517AA	9517	198
ESC_MA8516AA	9512	512
ESC_MA8515AA	4038	257
ESC_MA8514AA	7366	824
ESC_MA8513AA	7093	868
ESC_MA8512AA	1727	257
ESC_MA8511AA	602	257
ESC_MA8510AA	201	257
ESC_MA8509AA	10	512
ESC_MA8508AA	10	512
ESC_MA8507AA	38	669
ESC_MA8506AA	9513	824
ESC_MA8505AA	448	257
ESC_MA8504AA	4038	257
ESC_MA8503AA	2325	447
ESC_MA8502AA	196	257
ESC_MA8501AA	4741	512
ESC_MA8500AA	6018	52
ESC_MA8499AA	297	234
ESC_MA8498AA	131	653
ESC_MA8497AA	48	76
ESC_MA8496AA	3288	286
ESC_MA8495AA	6861	789
ESC_MA8494AA	155	175
ESC_MA8493AA	382	481
ESC_MA8492AA	409	504
ESC_MA8491AA	155	175
ESC_MA8490AA	48	76
ESC_MA8489AA	117	592
ESC_MA8488AA	9515	286
ESC_MA8487AA	2178	924
ESC_MA8486AA	131	653
ESC_MA8485AA	9514	504
ESC_MA8484AA	48	76
ESC_MA8483AA	616	156
ESC_MA8482AA	218	519
ESC_MA8481AA	5082	198

ESC_MA8480AA	48	76
ESC_MA8479AA	48	76
ESC_MA8478AA	522	701
ESC_MA8477AA	1844	198
ESC_MA8476AA	155	175
ESC_MA8475AA	297	234
ESC_MA8474AA	1304	257
ESC_MA8473AA	504	738
ESC_MA8472AA	349	670
ESC_MA8471AA	155	175
ESC_MA8470AA	297	234
ESC_MA8469AA	9516	257
ESC_MA8468AA	9441	175
ESC_MA8467AA	156	175
ESC_MA8466AA	1611	198
ESC_MA8465AA	2540	257
ESC_MA8464AA	124	254
ESC_MA8463AA	3057	672
ESC_MA8462AA	155	175
ESC_MA8461AA	5273	682
ESC_MA8460AA	5273	682
ESC_MA8459AA	937	198
ESC_MA8458AA	6178	924
ESC_MA8457AA	939	198
ESC_MA8456AA	9506	682
ESC_MA8455AA	6178	924
ESC_MA8454AA	129	257
ESC_MA8453AA	155	175
ESC_MA8452AA	155	175
ESC_MA8451AA	10	512
ESC_MA8450AA	1303	791
ESC_MA8449AA	295	160
ESC_MA8448AA	9523	825
ESC_MA8447AA	9583	824
ESC_MA8446AA	69	387
ESC_MA8445AA	2541	878
ESC_MA8444AA	3572	512
ESC_MA8443AA	206	286
ESC_MA8442AA	8576	212
ESC_MA8441AA	115	669
ESC_MA8440AA	394	400
ESC_MA8439AA	73	737
ESC_MA8438AA	73	737
ESC_MA8437AA	1170	629
ESC_MA8436AA	73	737
ESC_MA8435AA	675	234
ESC_MA8434AA	297	234
ESC_MA8433AA	2705	504
ESC_MA8432AA	4243	914
ESC_MA8431AA	9580	286

ESC_MA8430AA	681	393
ESC_MA8429AA	129	257
ESC_MA8428AA	9579	71
ESC_MA8427AA	3856	789
ESC_MA8426AA	224	198
ESC_MA8425AA	2772	257
ESC_MA8424AA	345	175
ESC_MA8423AA	1001	257
ESC_MA8422AA	1434	512
ESC_MA8421AA	10	512
ESC_MA8420AA	210	108
ESC_MA8419AA	9578	512
ESC_MA8418AA	3877	512
ESC_MA8417AA	197	601
ESC_MA8416AA	48	76
ESC_MA8415AA	10	512
ESC_MA8414AA	297	234
ESC_MA8413AA	9582	268
ESC_MA8412AA	10	512
ESC_MA8411AA	9582	268
ESC_MA8410AA	206	286
ESC_MA8409AA	46	447
ESC_MA8408AA	9522	204
ESC_MA8407AA	48	76
ESC_MA8406AA	543	842
ESC_MA8405AA	2280	212
ESC_MA8404AA	9581	512
ESC_MA8403AA	3519	101
ESC_MA8402AA	2541	878
ESC_MA8401AA	48	76
ESC_MA8400AA	48	76
ESC_MA8399AA	297	234
ESC_MA8398AA	4360	842
ESC_MA8397AA	1312	76
ESC_MA8396AA	4360	842
ESC_MA8395AA	1237	447
ESC_MA8394AA	1629	924
ESC_MA8393AA	224	198
ESC_MA8392AA	118	985
ESC_MA8391AA	540	286
ESC_MA8390AA	58	198
ESC_MA8389AA	542	824
ESC_MA8388AA	9521	785
ESC_MA8387AA	58	198
ESC_MA8386AA	4434	234
ESC_MA8385AA	9441	175
ESC_MA8384AA	683	175
ESC_MA8383AA	1136	108
ESC_MA8382AA	7020	257
ESC_MA8381AA	641	935

ESC_MA8380AA	9443	234
ESC_MA8379AA	347	286
ESC_MA8378AA	155	175
ESC_MA8377AA	156	175
ESC_MA8376AA	297	234
ESC_MA8375AA	6753	76
ESC_MA8374AA	5834	504
ESC_MA8373AA	10	512
ESC_MA8372AA	48	76
ESC_MA8371AA	401	142
ESC_MA8370AA	3910	672
ESC_MA8369AA	3910	672
ESC_MA8368AA	216	512
ESC_MA8367AA	394	400
ESC_MA8366AA	677	257
ESC_MA8365AA	218	519
ESC_MA8364AA	3107	512
ESC_MA8363AA	5834	504
ESC_MA8362AA	218	519
ESC_MA8361AA	541	701
ESC_MA8360AA	3168	261
ESC_MA8359AA	9443	234
ESC_MA8358AA	141	644
ESC_MA8357AA	69	387
ESC_MA8356AA	59	217
ESC_MA8355AA	23	156
ESC_MA8354AA	609	447
ESC_MA8353AA	1727	257
ESC_MA8352AA	9592	198
ESC_MA8351AA	46	447
ESC_MA8350AA	155	175
ESC_MA8349AA	224	198
ESC_MA8348AA	5625	785
ESC_MA8347AA	9441	175
ESC_MA8346AA	181	311
ESC_MA8345AA	10	512
ESC_MA8344AA	2005	175
ESC_MA8343AA	4977	71
ESC_MA8342AA	88	160
ESC_MA8341AA	224	198
ESC_MA8340AA	48	76
ESC_MA8339AA	206	286
ESC_MA8338AA	2522	198
ESC_MA8337AA	746	789
ESC_MA8336AA	48	76
ESC_MA8335AA	9541	198
ESC_MA8334AA	2522	198
ESC_MA8333AA	10	512
ESC_MA8332AA	181	311
ESC_MA8331AA	216	512

ESC_MA8330AA	351	268
ESC_MA8329AA	10	512
ESC_MA8328AA	569	738
ESC_MA8327AA	2739	76
ESC_MA8326AA	10	512
ESC_MA8325AA	9590	235
ESC_MA8324AA	9591	512
ESC_MA8323AA	155	175
ESC_MA8322AA	69	387
ESC_MA8321AA	2722	234
ESC_MA8320AA	10	512
ESC_MA8319AA	3910	672
ESC_MA8318AA	297	234
ESC_MA8317AA	3640	257
ESC_MA8316AA	10	512
ESC_MA8315AA	4977	71
ESC_MA8314AA	4389	504
ESC_MA8313AA	176	601
ESC_MA8312AA	297	234
ESC_MA8311AA	1408	445
ESC_MA8310AA	162	924
ESC_MA8309AA	10	512
ESC_MA8308AA	3716	410
ESC_MA8307AA	537	756
ESC_MA8306AA	48	76
ESC_MA8305AA	10	512
ESC_MA8304AA	10	512
ESC_MA8303AA	58	198
ESC_MA8302AA	6852	512
ESC_MA8301AA	206	286
ESC_MA8300AA	2622	537
ESC_MA8299AA	10	512
ESC_MA8298AA	2705	504
ESC_MA8297AA	2936	515
ESC_MA8296AA	155	175
ESC_MA8295AA	48	76
ESC_MA8294AA	9432	257
ESC_MA8293AA	155	175
ESC_MA8292AA	34	504
ESC_MA8291AA	9593	386
ESC_MA8290AA	9596	198
ESC_MA8289AA	48	76
ESC_MA8288AA	4096	512
ESC_MA8287AA	154	257
ESC_MA8286AA	9454	198
ESC_MA8285AA	348	175
ESC_MA8284AA	9454	198
ESC_MA8283AA	540	286
ESC_MA8282AA	6018	52
ESC_MA8281AA	6488	198

ESC_MA8280AA	332	257
ESC_MA8279AA	6768	314
ESC_MA8278AA	616	156
ESC_MA8277AA	90	156
ESC_MA8276AA	355	764
ESC_MA8275AA	9525	519
ESC_MA8274AA	1716	512
ESC_MA8273AA	4554	738
ESC_MA8272AA	9595	52
ESC_MA8271AA	398	445
ESC_MA8270AA	155	175
ESC_MA8269AA	48	76
ESC_MA8268AA	9594	519
ESC_MA8267AA	388	156
ESC_MA8266AA	2521	257
ESC_MA8265AA	1706	156
ESC_MA8264AA	224	198
ESC_MA8263AA	155	175
ESC_MA8262AA	193	795
ESC_MA8261AA	2622	537
ESC_MA8260AA	181	311
ESC_MA8259AA	10	512
ESC_MA8258AA	1125	198
ESC_MA8257AA	410	156
ESC_MA8256AA	155	175
ESC_MA8255AA	362	234
ESC_MA8254AA	48	76
ESC_MA8253AA	2622	537
ESC_MA8252AA	1638	519
ESC_MA8251AA	215	512
ESC_MA8250AA	58	198
ESC_MA8249AA	48	76
ESC_MA8248AA	56	198
ESC_MA8247AA	2025	194
ESC_MA8246AA	336	878
ESC_MA8245AA	8273	168
ESC_MA8244AA	10	512
ESC_MA8243AA	3856	789
ESC_MA8242AA	710	291
ESC_MA8241AA	141	644
ESC_MA8240AA	17	191
ESC_MA8239AA	58	198
ESC_MA8238AA	9600	406
ESC_MA8237AA	155	175
ESC_MA8236AA	10	512
ESC_MA8235AA	10	512
ESC_MA8234AA	1463	263
ESC_MA8233AA	206	286
ESC_MA8232AA	7464	76
ESC_MA8231AA	5334	512

ESC_MA8230AA	10	512
ESC_MA8229AA	58	198
ESC_MA8228AA	297	234
ESC_MA8227AA	48	76
ESC_MA8226AA	9526	76
ESC_MA8225AA	93	71
ESC_MA8224AA	480	789
ESC_MA8223AA	1141	512
ESC_MA8222AA	8938	174
ESC_MA8221AA	7428	924
ESC_MA8220AA	2139	789
ESC_MA8219AA	297	234
ESC_MA8218AA	453	935
ESC_MA8217AA	10	512
ESC_MA8216AA	5575	771
ESC_MA8215AA	155	175
ESC_MA8214AA	1237	447
ESC_MA8213AA	155	175
ESC_MA8212AA	58	198
ESC_MA8211AA	297	234
ESC_MA8210AA	4015	512
ESC_MA8209AA	6175	257
ESC_MA8208AA	48	76
ESC_MA8207AA	398	445
ESC_MA8206AA	1727	257
ESC_MA8205AA	69	387
ESC_MA8204AA	1727	257
ESC_MA8203AA	2144	257
ESC_MA8202AA	223	198
ESC_MA8201AA	6096	257
ESC_MA8200AA	10	512
ESC_MA8199AA	9603	649
ESC_MA8198AA	137	270
ESC_MA8197AA	6805	286
ESC_MA8196AA	164	175
ESC_MA8195AA	1485	408
ESC_MA8194AA	2496	789
ESC_MA8193AA	40	198
ESC_MA8192AA	34	504
ESC_MA8191AA	10	512
ESC_MA8190AA	2325	447
ESC_MA8189AA	155	175
ESC_MA8188AA	3188	257
ESC_MA8187AA	1304	257
ESC_MA8186AA	8777	447
ESC_MA8185AA	3057	672
ESC_MA8184AA	3057	672
ESC_MA8183AA	716	795
ESC_MA8181AA	58	198
ESC_MA8180AA	216	512

ESC_MA8179AA	297	234
ESC_MA8178AA	48	76
ESC_MA8177AA	10	512
ESC_MA8176AA	297	234
ESC_MA8175AA	9602	504
ESC_MA8174AA	48	76
ESC_MA8173AA	48	76
ESC_MA8172AA	9601	821
ESC_MA8171AA	48	76
ESC_MA8170AA	2165	257
ESC_MA8169AA	2522	198
ESC_MA8168AA	59	217
ESC_MA8167AA	3234	257
ESC_MA8166AA	131	653
ESC_MA8165AA	1421	447
ESC_MA8164AA	10	512
ESC_MA8163AA	3478	512
ESC_MA8162AA	48	76
ESC_MA8161AA	10	512
ESC_MA8160AA	6096	257
ESC_MA8159AA	2073	198
ESC_MA8158AA	10	512
ESC_MA8157AA	9481	198
ESC_MA8156AA	1727	257
ESC_MA8155AA	5122	445
ESC_MA8154AA	10	512
ESC_MA8153AA	10	512
ESC_MA8152AA	216	512
ESC_MA8151AA	165	504
ESC_MA8150AA	216	512
ESC_MA8149AA	58	198
ESC_MA8148AA	9527	198
ESC_MA8147AA	2144	257
ESC_MA8146AA	906	198
ESC_MA8145AA	162	924
ESC_MA8144AA	162	924
ESC_MA8143AA	641	935
ESC_MA8142AA	155	175
ESC_MA8141AA	4537	76
ESC_MA8140AA	453	935
ESC_MA8139AA	744	512
ESC_MA8138AA	46	447
ESC_MA8137AA	1775	51
ESC_MA8136AA	9605	504
ESC_MA8135AA	48	76
ESC_MA8134AA	10	512
ESC_MA8133AA	2144	257
ESC_MA8132AA	224	198
ESC_MA8131AA	1638	519
ESC_MA8130AA	6190	198

ESC_MA8129AA	2280	212
ESC_MA8128AA	48	76
ESC_MA8127AA	1717	212
ESC_MA8126AA	10	512
ESC_MA8125AA	9604	936
ESC_MA8124AA	7324	257
ESC_MA8123AA	93	71
ESC_MA8122AA	48	76
ESC_MA8121AA	4537	76
ESC_MA8120AA	1141	512
ESC_MA8119AA	2165	257
ESC_MA8118AA	351	268
ESC_MA8117AA	1775	51
ESC_MA8116AA	3856	789
ESC_MA8115AA	398	445
ESC_MA8114AA	2617	217
ESC_MA8113AA	1155	740
ESC_MA8112AA	48	76
ESC_MA8111AA	155	175
ESC_MA8110AA	58	198
ESC_MA8109AA	48	76
ESC_MA8108AA	58	198
ESC_MA8107AA	297	234
ESC_MA8106AA	33	198
ESC_MA8105AA	2175	212
ESC_MA8104AA	4038	257
ESC_MA8103AA	2517	160
ESC_MA8102AA	540	286
ESC_MA8101AA	2175	212
ESC_MA8100AA	398	445
ESC_MA8099AA	1079	235
ESC_MA8098AA	101	696
ESC_MA8097AA	295	160
ESC_MA8096AA	155	175
ESC_MA8095AA	446	257
ESC_MA8094AA	2705	504
ESC_MA8093AA	541	701
ESC_MA8092AA	3576	175
ESC_MA8091AA	602	257
ESC_MA8090AA	7473	789
ESC_MA8089AA	34	504
ESC_MA8088AA	4995	198
ESC_MA8087AA	196	257
ESC_MA8086AA	9441	175
ESC_MA8085AA	9563	515
ESC_MA8084AA	9592	198
ESC_MA8083AA	9592	198
ESC_MA8082AA	448	257
ESC_MA8081AA	5406	156
ESC_MA8080AA	9441	175

ESC_MA8079AA	737	175
ESC_MA8078AA	9441	175
ESC_MA8077AA	9441	175
ESC_MA8076AA	357	654
ESC_MA8075AA	9608	935
ESC_MA8074AA	677	257
ESC_MA8073AA	10	512
ESC_MA8072AA	5078	824
ESC_MA8071AA	155	175
ESC_MA8070AA	1638	519
ESC_MA8069AA	9607	512
ESC_MA8068AA	93	71
ESC_MA8067AA	9528	820
ESC_MA8066AA	206	286
ESC_MA8065AA	9606	76
ESC_MA8064AA	4532	234
ESC_MA8063AA	164	175
ESC_MA8062AA	156	175
ESC_MA8061AA	10	512
ESC_MA8060AA	1056	257
ESC_MA8059AA	3033	737
ESC_MA8058AA	48	76
ESC_MA8057AA	40	198
ESC_MA8056AA	1737	243
ESC_MA8055AA	1717	212
ESC_MA8054AA	156	175
ESC_MA8053AA	4088	198
ESC_MA8052AA	9613	596
ESC_MA8051AA	1125	198
ESC_MA8050AA	9612	76
ESC_MA8049AA	4088	198
ESC_MA8048AA	4088	198
ESC_MA8047AA	1248	156
ESC_MA8046AA	8564	261
ESC_MA8045AA	2705	504
ESC_MA8044AA	9611	167
ESC_MA8043AA	6186	868
ESC_MA8042AA	5762	504
ESC_MA8041AA	1136	108
ESC_MA8040AA	155	175
ESC_MA8039AA	10	512
ESC_MA8038AA	6833	445
ESC_MA8037AA	2614	975
ESC_MA8036AA	13	257
ESC_MA8035AA	23	156
ESC_MA8034AA	8650	257
ESC_MA8033AA	648	359
ESC_MA8032AA	1304	257
ESC_MA8031AA	7116	235
ESC_MA8030AA	641	935

ESC_MA8029AA	9555	701
ESC_MA8028AA	2005	175
ESC_MA8027AA	10	512
ESC_MA8026AA	164	175
ESC_MA8025AA	9536	311
ESC_MA8024AA	10	512
ESC_MA8023AA	9610	165
ESC_MA8022AA	297	234
ESC_MA8021AA	337	257
ESC_MA8020AA	398	445
ESC_MA8019AA	746	789
ESC_MA8018AA	746	789
ESC_MA8017AA	2739	76
ESC_MA8016AA	10	512
ESC_MA8015AA	110	629
ESC_MA8014AA	677	257
ESC_MA8013AA	7798	772
ESC_MA8012AA	48	76
ESC_MA8011AA	9619	765
ESC_MA8010AA	409	504
ESC_MA8009AA	1081	198
ESC_MA8008AA	58	198
ESC_MA8007AA	9609	839
ESC_MA8006AA	58	198
ESC_MA8005AA	372	424
ESC_MA8004AA	5419	175
ESC_MA8003AA	187	257
ESC_MA8002AA	2144	257
ESC_MA8001AA	9529	1002
ESC_MA8000AA	362	234
ESC_MA7999AA	3576	175
ESC_MA7998AA	86	935
ESC_MA7997AA	538	629
ESC_MA7996AA	538	629
ESC_MA7995AA	155	175
ESC_MA7994AA	1136	108
ESC_MA7993AA	11092	512
ESC_MA7992AA	541	701
ESC_MA7991AA	10	512
ESC_MA7990AA	10	512
ESC_MA7989AA	9618	257
ESC_MA7988AA	189	504
ESC_MA7987AA	9617	198
ESC_MA7986AA	10	512
ESC_MA7985AA	2614	975
ESC_MA7984AA	746	789
ESC_MA7983AA	9616	272
ESC_MA7982AA	10	512
ESC_MA7981AA	69	387
ESC_MA7980AA	538	629

ESC_MA7979AA	1121	198
ESC_MA7978AA	9615	175
ESC_MA7977AA	2602	257
ESC_MA7976AA	4080	300
ESC_MA7975AA	5919	699
ESC_MA7974AA	48	76
ESC_MA7973AA	1136	108
ESC_MA7972AA	48	76
ESC_MA7971AA	3877	512
ESC_MA7970AA	10	512
ESC_MA7969AA	10	512
ESC_MA7968AA	517	924
ESC_MA7967AA	224	198
ESC_MA7966AA	6889	76
ESC_MA7965AA	162	924
ESC_MA7964AA	681	393
ESC_MA7963AA	162	924
ESC_MA7962AA	162	924
ESC_MA7961AA	1163	579
ESC_MA7960AA	210	108
ESC_MA7959AA	156	175
ESC_MA7958AA	998	644
ESC_MA7957AA	746	789
ESC_MA7956AA	297	234
ESC_MA7955AA	223	198
ESC_MA7954AA	2074	858
ESC_MA7953AA	2614	975
ESC_MA7952AA	191	198
ESC_MA7951AA	1304	257
ESC_MA7950AA	3877	512
ESC_MA7949AA	5122	445
ESC_MA7948AA	9441	175
ESC_MA7947AA	9441	175
ESC_MA7946AA	2612	821
ESC_MA7945AA	501	900
ESC_MA7944AA	58	198
ESC_MA7943AA	871	447
ESC_MA7942AA	23	156
ESC_MA7941AA	58	198
ESC_MA7940AA	155	175
ESC_MA7939AA	10	512
ESC_MA7938AA	10	512
ESC_MA7937AA	58	198
ESC_MA7936AA	9614	824
ESC_MA7935AA	2387	261
ESC_MA7934AA	1724	863
ESC_MA7933AA	2073	198
ESC_MA7932AA	206	286
ESC_MA7931AA	675	234
ESC_MA7930AA	5797	710

ESC_MA7929AA	9624	263
ESC_MA7928AA	446	257
ESC_MA7927AA	155	175
ESC_MA7926AA	6396	76
ESC_MA7925AA	1844	198
ESC_MA7924AA	746	789
ESC_MA7923AA	4038	257
ESC_MA7922AA	542	824
ESC_MA7921AA	2073	198
ESC_MA7920AA	297	234
ESC_MA7919AA	9531	198
ESC_MA7918AA	9532	198
ESC_MA7917AA	1737	243
ESC_MA7916AA	8650	257
ESC_MA7915AA	201	257
ESC_MA7914AA	9623	810
ESC_MA7913AA	155	175
ESC_MA7912AA	101	696
ESC_MA7911AA	1178	512
ESC_MA7910AA	155	175
ESC_MA7909AA	200	198
ESC_MA7908AA	10	512
ESC_MA7907AA	59	217
ESC_MA7906AA	522	701
ESC_MA7905AA	1163	579
ESC_MA7904AA	9622	76
ESC_MA7903AA	48	76
ESC_MA7902AA	295	160
ESC_MA7901AA	517	924
ESC_MA7900AA	155	175
ESC_MA7899AA	2280	212
ESC_MA7898AA	746	789
ESC_MA7897AA	10	512
ESC_MA7896AA	187	257
ESC_MA7895AA	4568	104
ESC_MA7894AA	345	175
ESC_MA7893AA	156	175
ESC_MA7892AA	155	175
ESC_MA7891AA	1444	768
ESC_MA7890AA	216	512
ESC_MA7889AA	48	76
ESC_MA7888AA	9533	504
ESC_MA7887AA	3910	672
ESC_MA7886AA	2521	257
ESC_MA7885AA	641	935
ESC_MA7884AA	155	175
ESC_MA7883AA	2178	924
ESC_MA7882AA	9534	512
ESC_MA7881AA	2967	289
ESC_MA7880AA	155	175

ESC_MA7879AA	366	198
ESC_MA7878AA	211	257
ESC_MA7877AA	9441	175
ESC_MA7876AA	10	512
ESC_MA7875AA	1196	257
ESC_MA7874AA	9627	903
ESC_MA7873AA	7116	235
ESC_MA7872AA	1196	257
ESC_MA7871AA	6241	900
ESC_MA7870AA	206	286
ESC_MA7869AA	131	653
ESC_MA7868AA	10	512
ESC_MA7867AA	4568	104
ESC_MA7866AA	155	175
ESC_MA7865AA	1730	234
ESC_MA7864AA	155	175
ESC_MA7863AA	210	108
ESC_MA7862AA	2852	257
ESC_MA7861AA	156	175
ESC_MA7860AA	5840	234
ESC_MA7859AA	164	175
ESC_MA7858AA	388	156
ESC_MA7857AA	5273	682
ESC_MA7856AA	9626	257
ESC_MA7855AA	9535	167
ESC_MA7854AA	155	175
ESC_MA7853AA	9625	632
ESC_MA7852AA	906	198
ESC_MA7851AA	582	420
ESC_MA7850AA	10	512
ESC_MA7849AA	187	257
ESC_MA7848AA	48	76
ESC_MA7847AA	1706	156
ESC_MA7846AA	3168	261
ESC_MA7845AA	640	768
ESC_MA7844AA	58	198
ESC_MA7843AA	10	512
ESC_MA7842AA	224	198
ESC_MA7841AA	549	387
ESC_MA7840AA	48	76
ESC_MA7839AA	155	175
ESC_MA7838AA	9536	311
ESC_MA7837AA	2351	234
ESC_MA7836AA	5997	445
ESC_MA7835AA	218	519
ESC_MA7834AA	1308	257
ESC_MA7833AA	297	234
ESC_MA7832AA	164	175
ESC_MA7831AA	56	198
ESC_MA7830AA	155	175

ESC_MA7829AA	121	537
ESC_MA7828AA	5766	198
ESC_MA7827AA	1308	257
ESC_MA7826AA	174	660
ESC_MA7825AA	218	519
ESC_MA7824AA	117	592
ESC_MA7823AA	226	512
ESC_MA7822AA	9630	539
ESC_MA7821AA	442	257
ESC_MA7820AA	2705	504
ESC_MA7819AA	9537	198
ESC_MA7818AA	187	257
ESC_MA7817AA	1146	198
ESC_MA7816AA	187	257
ESC_MA7815AA	101	696
ESC_MA7814AA	196	257
ESC_MA7813AA	10	512
ESC_MA7812AA	7116	235
ESC_MA7811AA	3856	789
ESC_MA7810AA	6701	535
ESC_MA7809AA	216	512
ESC_MA7808AA	2705	504
ESC_MA7807AA	5086	286
ESC_MA7806AA	93	71
ESC_MA7805AA	351	268
ESC_MA7804AA	616	156
ESC_MA7803AA	1727	257
ESC_MA7802AA	1302	817
ESC_MA7801AA	1722	599
ESC_MA7800AA	43	512
ESC_MA7799AA	9629	257
ESC_MA7798AA	58	198
ESC_MA7797AA	641	935
ESC_MA7796AA	9628	512
ESC_MA7795AA	1684	504
ESC_MA7794AA	9441	175
ESC_MA7793AA	48	76
ESC_MA7792AA	3033	737
ESC_MA7791AA	10	512
ESC_MA7790AA	2521	257
ESC_MA7789AA	9638	512
ESC_MA7788AA	3107	512
ESC_MA7787AA	1302	817
ESC_MA7786AA	1302	817
ESC_MA7785AA	8369	696
ESC_MA7784AA	48	76
ESC_MA7783AA	165	504
ESC_MA7782AA	9637	504
ESC_MA7781AA	9539	878
ESC_MA7780AA	161	445

ESC_MA7779AA	297	234
ESC_MA7778AA	9456	512
ESC_MA7777AA	9636	789
ESC_MA7776AA	9635	76
ESC_MA7775AA	295	160
ESC_MA7774AA	1294	512
ESC_MA7773AA	48	76
ESC_MA7772AA	4038	257
ESC_MA7771AA	9634	701
ESC_MA7770AA	746	789
ESC_MA7769AA	746	789
ESC_MA7768AA	155	175
ESC_MA7767AA	678	257
ESC_MA7766AA	202	311
ESC_MA7765AA	224	198
ESC_MA7764AA	48	76
ESC_MA7763AA	7317	519
ESC_MA7762AA	9633	824
ESC_MA7761AA	6071	817
ESC_MA7760AA	1665	601
ESC_MA7759AA	9538	824
ESC_MA7758AA	155	175
ESC_MA7757AA	28	537
ESC_MA7756AA	5869	257
ESC_MA7755AA	7174	512
ESC_MA7754AA	7122	519
ESC_MA7753AA	101	696
ESC_MA7752AA	58	198
ESC_MA7751AA	409	504
ESC_MA7750AA	48	76
ESC_MA7749AA	200	198
ESC_MA7748AA	10	512
ESC_MA7747AA	9434	71
ESC_MA7746AA	4038	257
ESC_MA7745AA	1136	108
ESC_MA7744AA	224	198
ESC_MA7743AA	155	175
ESC_MA7742AA	398	445
ESC_MA7741AA	3107	512
ESC_MA7740AA	48	76
ESC_MA7739AA	2280	212
ESC_MA7738AA	131	653
ESC_MA7737AA	131	653
ESC_MA7736AA	10	512
ESC_MA7734AA	9631	816
ESC_MA7733AA	602	257
ESC_MA7732AA	906	198
ESC_MA7731AA	201	257
ESC_MA7730AA	206	286
ESC_MA7729AA	746	789

ESC_MA7728AA	9433	234
ESC_MA7727AA	8324	257
ESC_MA7726AA	3033	737
ESC_MA7725AA	8184	653
ESC_MA7724AA	127	738
ESC_MA7723AA	2598	198
ESC_MA7722AA	10	512
ESC_MA7721AA	48	76
ESC_MA7720AA	181	311
ESC_MA7719AA	1251	71
ESC_MA7718AA	2165	257
ESC_MA7717AA	93	71
ESC_MA7716AA	9641	55
ESC_MA7715AA	206	286
ESC_MA7714AA	2186	198
ESC_MA7713AA	978	740
ESC_MA7712AA	366	198
ESC_MA7711AA	942	198
ESC_MA7710AA	297	234
ESC_MA7709AA	1056	257
ESC_MA7708AA	541	701
ESC_MA7707AA	223	198
ESC_MA7706AA	9640	156
ESC_MA7705AA	206	286
ESC_MA7704AA	155	175
ESC_MA7703AA	9541	198
ESC_MA7702AA	223	198
ESC_MA7701AA	2389	160
ESC_MA7700AA	1157	878
ESC_MA7699AA	372	424
ESC_MA7698AA	9441	175
ESC_MA7697AA	394	400
ESC_MA7696AA	58	198
ESC_MA7695AA	9639	234
ESC_MA7694AA	48	76
ESC_MA7693AA	589	650
ESC_MA7692AA	1972	406
ESC_MA7691AA	589	650
ESC_MA7690AA	48	76
ESC_MA7689AA	9540	257
ESC_MA7688AA	542	824
ESC_MA7687AA	399	198
ESC_MA7686AA	357	654
ESC_MA7685AA	589	650
ESC_MA7684AA	10	512
ESC_MA7683AA	1433	601
ESC_MA7682AA	447	198
ESC_MA7681AA	13	257
ESC_MA7680AA	2526	257
ESC_MA7679AA	206	286

ESC_MA7678AA	6335	504
ESC_MA7677AA	1861	726
ESC_MA7676AA	4995	198
ESC_MA7675AA	8369	696
ESC_MA7674AA	678	257
ESC_MA7673AA	10	512
ESC_MA7672AA	1720	935
ESC_MA7671AA	3910	672
ESC_MA7670AA	155	175
ESC_MA7669AA	2758	512
ESC_MA7668AA	218	519
ESC_MA7667AA	5277	101
ESC_MA7666AA	9642	198
ESC_MA7665AA	9642	198
ESC_MA7664AA	1730	234
ESC_MA7663AA	2005	175
ESC_MA7662AA	1611	198
ESC_MA7661AA	398	445
ESC_MA7660AA	1727	257
ESC_MA7659AA	2076	400
ESC_MA7658AA	155	175
ESC_MA7657AA	9635	76
ESC_MA7656AA	2758	512
ESC_MA7655AA	155	175
ESC_MA7654AA	7395	923
ESC_MA7653AA	9442	257
ESC_MA7652AA	9542	438
ESC_MA7651AA	6175	257
ESC_MA7650AA	5523	445
ESC_MA7649AA	2800	526
ESC_MA7648AA	388	156
ESC_MA7647AA	1079	235
ESC_MA7646AA	174	660
ESC_MA7645AA	195	76
ESC_MA7644AA	409	504
ESC_MA7643AA	648	359
ESC_MA7642AA	218	519
ESC_MA7641AA	13	257
ESC_MA7640AA	9650	537
ESC_MA7639AA	939	198
ESC_MA7638AA	3889	291
ESC_MA7637AA	1638	519
ESC_MA7636AA	7588	447
ESC_MA7635AA	6726	493
ESC_MA7634AA	2705	504
ESC_MA7633AA	9459	76
ESC_MA7632AA	1716	512
ESC_MA7631AA	2325	447
ESC_MA7630AA	164	175
ESC_MA7629AA	7588	447

ESC_MA7628AA	9648	198
ESC_MA7627AA	58	198
ESC_MA7626AA	9648	198
ESC_MA7625AA	675	234
ESC_MA7624AA	442	257
ESC_MA7623AA	3042	663
ESC_MA7622AA	2073	198
ESC_MA7621AA	9649	445
ESC_MA7620AA	348	175
ESC_MA7619AA	224	198
ESC_MA7618AA	9648	198
ESC_MA7617AA	8165	111
ESC_MA7616AA	1049	175
ESC_MA7615AA	192	696
ESC_MA7614AA	1136	108
ESC_MA7613AA	9652	198
ESC_MA7612AA	48	76
ESC_MA7611AA	2077	198
ESC_MA7610AA	2328	257
ESC_MA7609AA	4038	257
ESC_MA7608AA	297	234
ESC_MA7607AA	58	198
ESC_MA7606AA	10	512
ESC_MA7605AA	101	696
ESC_MA7604AA	58	198
ESC_MA7603AA	129	257
ESC_MA7602AA	101	696
ESC_MA7601AA	9647	198
ESC_MA7600AA	409	504
ESC_MA7599AA	9646	175
ESC_MA7598AA	9645	824
ESC_MA7597AA	10	512
ESC_MA7596AA	450	71
ESC_MA7595AA	10	512
ESC_MA7594AA	616	156
ESC_MA7593AA	191	198
ESC_MA7592AA	2705	504
ESC_MA7591AA	9644	175
ESC_MA7590AA	1136	108
ESC_MA7589AA	6071	817
ESC_MA7588AA	174	660
ESC_MA7587AA	191	198
ESC_MA7586AA	9643	261
ESC_MA7585AA	540	286
ESC_MA7584AA	540	286
ESC_MA7583AA	351	268
ESC_MA7582AA	2280	212
ESC_MA7581AA	906	198
ESC_MA7580AA	1196	257
ESC_MA7579AA	5597	72

ESC_MA7578AA	4995	198
ESC_MA7577AA	636	653
ESC_MA7576AA	9543	221
ESC_MA7575AA	155	175
ESC_MA7574AA	9592	198
ESC_MA7573AA	2165	257
ESC_MA7572AA	372	424
ESC_MA7571AA	8764	694
ESC_MA7570AA	517	924
ESC_MA7569AA	442	257
ESC_MA7568AA	9651	175
ESC_MA7567AA	7615	512
ESC_MA7566AA	2076	400
ESC_MA7565AA	59	217
ESC_MA7564AA	1727	257
ESC_MA7563AA	297	234
ESC_MA7562AA	2598	198
ESC_MA7561AA	297	234
ESC_MA7560AA	48	76
ESC_MA7559AA	10	512
ESC_MA7558AA	10	512
ESC_MA7557AA	155	175
ESC_MA7556AA	58	198
ESC_MA7555AA	48	76
ESC_MA7554AA	1665	601
ESC_MA7553AA	939	198
ESC_MA7552AA	10	512
ESC_MA7551AA	1298	924
ESC_MA7550AA	181	311
ESC_MA7549AA	38	669
ESC_MA7548AA	206	286
ESC_MA7547AA	23	156
ESC_MA7546AA	540	286
ESC_MA7545AA	1298	924
ESC_MA7544AA	1720	935
ESC_MA7543AA	405	923
ESC_MA7542AA	398	445
ESC_MA7541AA	401	142
ESC_MA7540AA	10	512
ESC_MA7539AA	847	257
ESC_MA7538AA	1771	868
ESC_MA7537AA	4398	924
ESC_MA7536AA	48	76
ESC_MA7535AA	206	286
ESC_MA7534AA	165	504
ESC_MA7533AA	3274	512
ESC_MA7532AA	101	696
ESC_MA7531AA	48	76
ESC_MA7530AA	48	76
ESC_MA7529AA	9546	504

ESC_MA7528AA	4398	924
ESC_MA7527AA	46	447
ESC_MA7526AA	155	175
ESC_MA7525AA	206	286
ESC_MA7524AA	10	512
ESC_MA7523AA	48	76
ESC_MA7522AA	394	400
ESC_MA7521AA	7184	36
ESC_MA7520AA	9545	504
ESC_MA7519AA	295	160
ESC_MA7518AA	48	76
ESC_MA7517AA	8823	257
ESC_MA7516AA	206	286
ESC_MA7515AA	48	76
ESC_MA7514AA	9544	795
ESC_MA7513AA	10	512
ESC_MA7512AA	398	445
ESC_MA7511AA	155	175
ESC_MA7510AA	1727	257
ESC_MA7509AA	10	512
ESC_MA7508AA	423	204
ESC_MA7507AA	5909	76
ESC_MA7506AA	206	286
ESC_MA7505AA	206	286
ESC_MA7504AA	641	935
ESC_MA7503AA	746	789
ESC_MA7502AA	1114	504
ESC_MA7501AA	206	286
ESC_MA7500AA	2163	257
ESC_MA7499AA	48	76
ESC_MA7498AA	155	175
ESC_MA7497AA	155	175
ESC_MA7496AA	9541	198
ESC_MA7495AA	2705	504
ESC_MA7494AA	155	175
ESC_MA7493AA	10	512
ESC_MA7492AA	218	519
ESC_MA7491AA	1431	257
ESC_MA7490AA	23	156
ESC_MA7489AA	2076	400
ESC_MA7488AA	10	512
ESC_MA7487AA	10	512
ESC_MA7486AA	594	900
ESC_MA7485AA	8369	696
ESC_MA7484AA	8369	696
ESC_MA7483AA	4682	175
ESC_MA7482AA	206	286
ESC_MA7481AA	2722	234
ESC_MA7480AA	10	512
ESC_MA7479AA	10	512

ESC_MA7478AA	2598	198
ESC_MA7477AA	4598	868
ESC_MA7476AA	206	286
ESC_MA7475AA	48	76
ESC_MA7474AA	442	257
ESC_MA7473AA	2614	975
ESC_MA7472AA	10	512
ESC_MA7471AA	162	924
ESC_MA7470AA	9550	512
ESC_MA7469AA	189	504
ESC_MA7468AA	2622	537
ESC_MA7467AA	192	696
ESC_MA7466AA	224	198
ESC_MA7465AA	93	71
ESC_MA7464AA	223	198
ESC_MA7463AA	189	504
ESC_MA7462AA	2461	76
ESC_MA7461AA	69	387
ESC_MA7460AA	189	504
ESC_MA7459AA	48	76
ESC_MA7458AA	10	512
ESC_MA7457AA	2165	257
ESC_MA7456AA	2914	670
ESC_MA7455AA	7324	257
ESC_MA7454AA	189	504
ESC_MA7453AA	453	935
ESC_MA7452AA	3856	789
ESC_MA7451AA	43	512
ESC_MA7450AA	43	512
ESC_MA7449AA	337	257
ESC_MA7448AA	155	175
ESC_MA7447AA	9549	175
ESC_MA7446AA	48	76
ESC_MA7445AA	58	198
ESC_MA7444AA	223	198
ESC_MA7443AA	218	519
ESC_MA7442AA	746	789
ESC_MA7441AA	10	512
ESC_MA7440AA	1049	175
ESC_MA7439AA	695	519
ESC_MA7438AA	48	76
ESC_MA7437AA	48	76
ESC_MA7436AA	939	198
ESC_MA7435AA	9548	76
ESC_MA7434AA	9547	76
ESC_MA7433AA	205	257
ESC_MA7432AA	10	512
ESC_MA7431AA	616	156
ESC_MA7430AA	5840	234
ESC_MA7429AA	5840	234

ESC_MA7428AA	1196	257
ESC_MA7427AA	9557	234
ESC_MA7426AA	10	512
ESC_MA7425AA	10	512
ESC_MA7424AA	1147	198
ESC_MA7423AA	200	198
ESC_MA7422AA	394	400
ESC_MA7421AA	10	512
ESC_MA7420AA	409	504
ESC_MA7419AA	9556	235
ESC_MA7418AA	174	660
ESC_MA7417AA	38	669
ESC_MA7416AA	10	512
ESC_MA7415AA	2852	257
ESC_MA7414AA	58	198
ESC_MA7413AA	2705	504
ESC_MA7412AA	9555	701
ESC_MA7411AA	58	198
ESC_MA7410AA	48	76
ESC_MA7409AA	2705	504
ESC_MA7408AA	59	217
ESC_MA7407AA	216	512
ESC_MA7406AA	216	512
ESC_MA7405AA	541	701
ESC_MA7404AA	58	198
ESC_MA7403AA	6280	286
ESC_MA7402AA	58	198
ESC_MA7401AA	9554	512
ESC_MA7400AA	9554	512
ESC_MA7399AA	9553	76
ESC_MA7398AA	5909	76
ESC_MA7397AA	9552	76
ESC_MA7396AA	7611	512
ESC_MA7395AA	9551	286
ESC_MA7394AA	6889	76
ESC_MA7393AA	297	234
ESC_MA7392AA	6096	257
ESC_MA7391AA	58	198
ESC_MA7390AA	1304	257
ESC_MA7389AA	6096	257
ESC_MA7388AA	93	71
ESC_MA7387AA	4732	198
ESC_MA7386AA	99	198
ESC_MA7385AA	7687	257
ESC_MA7384AA	48	76
ESC_MA7383AA	998	644
ESC_MA7382AA	69	387
ESC_MA7381AA	1011	198
ESC_MA7380AA	155	175
ESC_MA7379AA	6096	257

ESC_MA7378AA	6736	817
ESC_MA7377AA	224	198
ESC_MA7376AA	48	76
ESC_MA7375AA	10	512
ESC_MA7374AA	616	156
ESC_MA7373AA	10	512
ESC_MA7372AA	226	512
ESC_MA7371AA	746	789
ESC_MA7370AA	216	512
ESC_MA7369AA	219	877
ESC_MA7368AA	295	160
ESC_MA7367AA	226	512
ESC_MA7366AA	9561	730
ESC_MA7365AA	4532	234
ESC_MA7364AA	165	504
ESC_MA7363AA	10	512
ESC_MA7362AA	155	175
ESC_MA7361AA	679	257
ESC_MA7360AA	9560	512
ESC_MA7359AA	602	257
ESC_MA7358AA	607	355
ESC_MA7357AA	1148	257
ESC_MA7356AA	7020	257
ESC_MA7355AA	9479	258
ESC_MA7354AA	1308	257
ESC_MA7353AA	1304	257
ESC_MA7352AA	224	198
ESC_MA7351AA	1304	257
ESC_MA7350AA	1304	257
ESC_MA7349AA	297	234
ESC_MA7348AA	224	198
ESC_MA7347AA	616	156
ESC_MA7346AA	372	424
ESC_MA7345AA	223	198
ESC_MA7344AA	1303	791
ESC_MA7343AA	101	696
ESC_MA7342AA	58	198
ESC_MA7341AA	2178	924
ESC_MA7340AA	616	156
ESC_MA7339AA	3856	789
ESC_MA7338AA	9542	438
ESC_MA7337AA	2351	234
ESC_MA7336AA	641	935
ESC_MA7335AA	48	76
ESC_MA7334AA	127	738
ESC_MA7333AA	4704	504
ESC_MA7332AA	9559	76
ESC_MA7331AA	9559	76
ESC_MA7330AA	2351	234
ESC_MA7329AA	6750	76

ESC_MA7328AA	7329	175
ESC_MA7327AA	9558	76
ESC_MA7326AA	947	257
ESC_MA7325AA	448	257
ESC_MA7324AA	683	175
ESC_MA7323AA	1727	257
ESC_MA7322AA	9566	900
ESC_MA7321AA	58	198
ESC_MA7320AA	4537	76
ESC_MA7319AA	155	175
ESC_MA7318AA	9565	167
ESC_MA7317AA	9441	175
ESC_MA7316AA	4732	198
ESC_MA7315AA	9564	739
ESC_MA7314AA	297	234
ESC_MA7313AA	1727	257
ESC_MA7312AA	297	234
ESC_MA7311AA	447	198
ESC_MA7310AA	2935	512
ESC_MA7309AA	10	512
ESC_MA7308AA	1304	257
ESC_MA7307AA	9441	175
ESC_MA7306AA	642	924
ESC_MA7305AA	398	445
ESC_MA7304AA	58	198
ESC_MA7303AA	348	175
ESC_MA7302AA	2705	504
ESC_MA7301AA	2705	504
ESC_MA7300AA	9563	515
ESC_MA7299AA	9562	198
ESC_MA7298AA	2705	504
ESC_MA7297AA	2351	234
ESC_MA7296AA	48	76
ESC_MA7295AA	657	592
ESC_MA7294AA	8818	789
ESC_MA7293AA	1463	263
ESC_MA7292AA	278	924
ESC_MA7291AA	12	626
ESC_MA7290AA	17	191
ESC_MA7289AA	17	191
ESC_MA7288AA	21	156
ESC_MA7287AA	17	191
ESC_MA7286AA	17	191
ESC_MA7285AA	17	191
ESC_MA7284AA	17	191
ESC_MA7283AA	17	191
ESC_MA7282AA	17	191
ESC_MA7281AA	17	191
ESC_MA7280AA	17	191
ESC_MA7279AA	16	156

ESC_MA7278AA	1967	191
ESC_MA7277AA	117	592
ESC_MA7276AA	11	936
ESC_MA7275AA	211	257
ESC_MA7274AA	345	175
ESC_MA7273AA	104	737
ESC_MA7272AA	155	175
ESC_MA7271AA	1304	257
ESC_MA7270AA	58	198
ESC_MA7269AA	155	175
ESC_MA7268AA	7483	234
ESC_MA7267AA	11	936
ESC_MA7266AA	17	191
ESC_MA7265AA	17	191
ESC_MA7264AA	11	936
ESC_MA7263AA	5292	738
ESC_MA7262AA	21	156
ESC_MA7261AA	3759	175
ESC_MA7260AA	3759	175
ESC_MA7259AA	11	936
ESC_MA7258AA	3759	175
ESC_MA7257AA	3759	175
ESC_MA7256AA	3759	175
ESC_MA7255AA	11	936
ESC_MA7254AA	3759	175
ESC_MA7253AA	3759	175
ESC_MA7252AA	3759	175
ESC_MA7251AA	5172	156
ESC_MA7250AA	8405	696
ESC_MA7249AA	8405	696
ESC_MA7248AA	1079	235
ESC_MA7247AA	3759	175
ESC_MA7246AA	515	1004
ESC_MA7245AA	515	1004
ESC_MA7244AA	11	936
ESC_MA7243AA	515	1004
ESC_MA7242AA	515	1004
ESC_MA7241AA	8405	696
ESC_MA7240AA	335	936
ESC_MA7239AA	5172	156
ESC_MA7238AA	56	198
ESC_MA7237AA	3759	175
ESC_MA7236AA	6546	709
ESC_MA7235AA	3759	175
ESC_MA7234AA	3759	175
ESC_MA7233AA	3759	175
ESC_MA7232AA	56	198
ESC_MA7231AA	3759	175
ESC_MA7230AA	56	198
ESC_MA7228AA	8405	696

ESC_MA7227AA	8405	696
ESC_MA7226AA	3759	175
ESC_MA7225AA	3759	175
ESC_MA7224AA	56	198
ESC_MA7223AA	8405	696
ESC_MA7222AA	56	198
ESC_MA7221AA	206	286
ESC_MA7219AA	56	198
ESC_MA7218AA	56	198
ESC_MA7217AA	718	257
ESC_MA7216AA	5418	257
ESC_MA7215AA	11	936
ESC_MA7214AA	16	156
ESC_MA7213AA	11	936
ESC_MA7212AA	655	926
ESC_MA7211AA	17	191
ESC_MA7210AA	17	191
ESC_MA7209AA	11	936
ESC_MA7208AA	655	926
ESC_MA7207AA	11	936
ESC_MA7206AA	11	936
ESC_MA7205AA	17	191
ESC_MA7204AA	21	156
ESC_MA7203AA	17	191
ESC_MA7202AA	17	191
ESC_MA7201AA	5292	738
ESC_MA7200AA	21	156
ESC_MA7199AA	16	156
ESC_MA7198AA	131	653
ESC_MA7197AA	131	653
ESC_MA7196AA	315	669
ESC_MA7195AA	315	669
ESC_MA7194AA	315	669
ESC_MA7193AA	315	669
ESC_MA7192AA	315	669
ESC_MA7191AA	1140	934
ESC_MA7190AA	7483	234
ESC_MA7189AA	34	504
ESC_MA7188AA	93	71
ESC_MA7187AA	93	71
ESC_MA7186AA	93	71
ESC_MA7185AA	93	71
ESC_MA7184AA	93	71
ESC_MA7183AA	16	156
ESC_MA7182AA	32	270
ESC_MA7181AA	16	156
ESC_MA7180AA	1967	191
ESC_MA7179AA	2836	221
ESC_MA7178AA	17	191
ESC_MA7177AA	747	640

ESC_MA7176AA	40	198
ESC_MA7175AA	306	286
ESC_MA7174AA	9294	191
ESC_MA7173AA	21	156
ESC_MA7172AA	32	270
ESC_MA7171AA	155	175
ESC_MA7170AA	9567	156
ESC_MA7169AA	442	257
ESC_MA7168AA	17	191
ESC_MA7167AA	16	156
ESC_MA7166AA	17	191
ESC_MA7165AA	11	936
ESC_MA7163AA	10	512
ESC_MA7162AA	69	387
ESC_MA7161AA	130	387
ESC_MA7160AA	10	512
ESC_MA7159AA	10	512
ESC_MA7158AA	130	387
ESC_MA7157AA	5289	847
ESC_MA7156AA	362	234
ESC_MA7155AA	949	175
ESC_MA7154AA	448	257
ESC_MA7153AA	162	924
ESC_MA7152AA	57	50
ESC_MA7151AA	10	512
ESC_MA7150AA	10	512
ESC_MA7149AA	21	156
ESC_MA7148AA	167	512
ESC_MA7147AA	10	512
ESC_MA7146AA	10	512
ESC_MA7145AA	10	512
ESC_MA7144AA	925	387
ESC_MA7143AA	10	512
ESC_MA7142AA	88	160
ESC_MA7141AA	10	512
ESC_MA7140AA	10	512
ESC_MA7139AA	23	156
ESC_MA7138AA	130	387
ESC_MA7137AA	783	204
ESC_MA7136AA	10	512
ESC_MA7135AA	117	592
ESC_MA7134AA	23	156
ESC_MA7133AA	783	204
ESC_MA7131AA	167	512
ESC_MA7130AA	540	286
ESC_MA7129AA	925	387
ESC_MA7128AA	88	160
ESC_MA7127AA	88	160
ESC_MA7126AA	925	387
ESC_MA7125AA	58	198

ESC_MA7124AA	10	512
ESC_MA7123AA	58	198
ESC_MA7122AA	1131	936
ESC_MA7121AA	973	968
ESC_MA7120AA	448	257
ESC_MA7119AA	155	175
ESC_MA7118AA	88	160
ESC_MA7117AA	2041	447
ESC_MA7116AA	2449	234
ESC_MA7115AA	117	592
ESC_MA7114AA	58	198
ESC_MA7112AA	88	160
ESC_MA7111AA	949	175
ESC_MA7110AA	69	387
ESC_MA7109AA	69	387
ESC_MA7108AA	106	387
ESC_MA7107AA	69	387
ESC_MA7106AA	69	387
ESC_MA7105AA	362	234
ESC_MA7104AA	106	387
ESC_MA7103AA	537	756
ESC_MA7102AA	2602	257
ESC_MA7101AA	88	160
ESC_MA7100AA	73	737
ESC_MA7098AA	1167	257
ESC_MA7097AA	1091	512
ESC_MA7096AA	224	198
ESC_MA7095AA	9568	387
ESC_MA7094AA	10	512
ESC_MA7093AA	69	387
ESC_MA7091AA	1615	156
ESC_MA7090AA	88	160
ESC_MA7089AA	2041	447
ESC_MA7088AA	10	512
ESC_MA7087AA	10	512
ESC_MA7086AA	1080	198
ESC_MA7085AA	77	286
ESC_MA7084AA	388	156
ESC_MA7083AA	345	175
ESC_MA7082AA	101	696
ESC_MA7081AA	1403	924
ESC_MA7080AA	1081	198
ESC_MA7079AA	297	234
ESC_MA7078AA	1301	270
ESC_MA7077AA	1615	156
ESC_MA7076AA	58	198
ESC_MA7075AA	56	198
ESC_MA7074AA	9569	198
ESC_MA7073AA	10	512
ESC_MA7072AA	88	160

ESC_MA7071AA	88	160
ESC_MA7070AA	4092	198
ESC_MA7069AA	398	445
ESC_MA7068AA	13	257
ESC_MA7067AA	925	387
ESC_MA7066AA	48	76
ESC_MA7065AA	1123	257
ESC_MA7064AA	215	512
ESC_MA7063AA	9570	512
ESC_MA7062AA	1508	261
ESC_MA7061AA	10	512
ESC_MA7060AA	10	512
ESC_MA7059AA	847	257
ESC_MA7058AA	1123	257
ESC_MA7056AA	1508	261
ESC_MA7055AA	4173	234
ESC_MA7054AA	1508	261
ESC_MA7053AA	1122	307
ESC_MA7052AA	10	512
ESC_MA7051AA	10	512
ESC_MA7050AA	101	696
ESC_MA7049AA	3249	156
ESC_MA7048AA	104	737
ESC_MA7047AA	10	512
ESC_MA7046AA	641	935
ESC_MA7045AA	88	160
ESC_MA7044AA	58	198
ESC_MA7043AA	69	387
ESC_MA7042AA	69	387
ESC_MA7041AA	10	512
ESC_MA7040AA	4175	270
ESC_MA7039AA	10	512
ESC_MA7037AA	58	198
ESC_MA7036AA	58	198
ESC_MA7035AA	69	387
ESC_MA7034AA	1139	447
ESC_MA7033AA	10	512
ESC_MA7032AA	58	198
ESC_MA7031AA	1122	307
ESC_MA7030AA	602	257
ESC_MA7029AA	154	257
ESC_MA7028AA	540	286
ESC_MA7027AA	10	512
ESC_MA7026AA	448	257
ESC_MA7025AA	3021	447
ESC_MA7024AA	73	737
ESC_MA7023AA	141	644
ESC_MA7022AA	906	198
ESC_MA7021AA	88	160
ESC_MA7020AA	58	198

ESC_MA7019AA	58	198
ESC_MA7018AA	99	198
ESC_MA7017AA	1303	791
ESC_MA7016AA	155	175
ESC_MA7015AA	2628	878
ESC_MA7014AA	38	669
ESC_MA7013AA	648	359
ESC_MA7012AA	95	738
ESC_MA7011AA	109	257
ESC_MA7010AA	58	198
ESC_MA7009AA	95	738
ESC_MA7008AA	10	512
ESC_MA7007AA	2280	212
ESC_MA7006AA	58	198
ESC_MA7005AA	877	935
ESC_MA7004AA	1304	257
ESC_MA7003AA	2041	447
ESC_MA7002AA	770	587
ESC_MA7001AA	58	198
ESC_MA7000AA	117	592
ESC_MA6999AA	101	696
ESC_MA6998AA	101	696
ESC_MA6997AA	10	512
ESC_MA6996AA	10	512
ESC_MA6995AA	388	156
ESC_MA6994AA	2522	198
ESC_MA6993AA	9585	257
ESC_MA6992AA	58	198
ESC_MA6991AA	117	592
ESC_MA6990AA	11	936
ESC_MA6989AA	11	936
ESC_MA6988AA	8322	156
ESC_MA6987AA	33	198
ESC_MA6986AA	58	198
ESC_MA6985AA	5292	738
ESC_MA6984AA	58	198
ESC_MA6983AA	21	156
ESC_MA6982AA	32	270
ESC_MA6981AA	9571	900
ESC_MA6980AA	167	512
ESC_MA6979AA	617	511
ESC_MA6977AA	1702	512
ESC_MA6976AA	540	286
ESC_MA6975AA	1193	761
ESC_MA6974AA	101	696
ESC_MA6973AA	101	696
ESC_MA6972AA	410	156
ESC_MA6971AA	448	257
ESC_MA6970AA	617	511
ESC_MA6969AA	361	512

ESC_MA6968AA	2659	679
ESC_MA6967AA	405	923
ESC_MA6966AA	1340	595
ESC_MA6965AA	617	511
ESC_MA6964AA	6870	603
ESC_MA6963AA	131	653
ESC_MA6962AA	156	175
ESC_MA6961AA	410	156
ESC_MA6959AA	652	204
ESC_MA6958AA	127	738
ESC_MA6957AA	361	512
ESC_MA6956AA	73	737
ESC_MA6955AA	167	512
ESC_MA6954AA	38	669
ESC_MA6953AA	46	447
ESC_MA6952AA	405	923
ESC_MA6951AA	410	156
ESC_MA6950AA	2914	670
ESC_MA6949AA	38	669
ESC_MA6948AA	167	512
ESC_MA6947AA	38	669
ESC_MA6946AA	38	669
ESC_MA6945AA	372	424
ESC_MA6944AA	68	386
ESC_MA6943AA	9573	424
ESC_MA6942AA	155	175
ESC_MA6941AA	131	653
ESC_MA6940AA	9572	764
ESC_MA6939AA	11	936
ESC_MA6938AA	16	156
ESC_MA6937AA	17	191
ESC_MA6936AA	21	156
ESC_MA6935AA	11	936
ESC_MA6934AA	17	191
ESC_MA6933AA	21	156
ESC_MA6932AA	10	512
ESC_MA6931AA	10	512
ESC_MA6930AA	10	512
ESC_MA6929AA	10	512
ESC_MA6928AA	10	512
ESC_MA6927AA	10	512
ESC_MA6926AA	10	512
ESC_MA6925AA	10	512
ESC_MA6924AA	10	512
ESC_MA6922AA	10	512
ESC_MA6921AA	10	512
ESC_MA6920AA	11	936
ESC_MA6919AA	11	936
ESC_MA6918AA	11	936
ESC_MA6917AA	4	76

ESC_MA6916AA	5419	175
ESC_MA6915AA	21	156
ESC_MA6914AA	16	156
ESC_MA6913AA	1385	653
ESC_MA6912AA	21	156
ESC_MA6911AA	29	156
ESC_MA6910AA	106	387
ESC_MA6909AA	11	936
ESC_MA6908AA	10	512
ESC_MA6907AA	11	936
ESC_MA6906AA	442	257
ESC_MA6905AA	11	936
ESC_MA6904AA	5292	738
ESC_MA6903AA	69	387
ESC_MA6902AA	48	76
ESC_MA6901AA	6015	234
ESC_MA6900AA	23	156
ESC_MA6899AA	345	175
ESC_MA6898AA	348	175
ESC_MA6897AA	350	50
ESC_MA6896AA	706	422
ESC_MA6895AA	2556	630
ESC_MA6894AA	38	669
ESC_MA6893AA	109	257
ESC_MA6892AA	3580	257
ESC_MA6891AA	11	936
ESC_MA6890AA	17	191
ESC_MA6889AA	343	286
ESC_MA6888AA	131	653
ESC_MA6887AA	131	653
ESC_MA6886AA	131	653
ESC_MA6885AA	131	653
ESC_MA6884AA	131	653
ESC_MA6883AA	131	653
ESC_MA6882AA	131	653
ESC_MA6881AA	131	653
ESC_MA6880AA	131	653
ESC_MA6879AA	131	653
ESC_MA6877AA	21	156
ESC_MA6876AA	17	191
ESC_MA6875AA	11	936
ESC_MA6874AA	119	286
ESC_MA6873AA	21	156
ESC_MA6872AA	16	156
ESC_MA6871AA	16	156
ESC_MA6870AA	17	191
ESC_MA6869AA	17	191
ESC_MA6868AA	655	926
ESC_MA6867AA	21	156
ESC_MA6866AA	21	156

ESC_MA6865AA	21	156
ESC_MA6864AA	17	191
ESC_MA6863AA	17	191
ESC_MA6861AA	744	512
ESC_MA6860AA	3076	175
ESC_MA6859AA	93	71
ESC_MA6858AA	354	15
ESC_MA6856AA	154	257
ESC_MA6854AA	189	504
ESC_MA6851AA	617	511
ESC_MA6848AA	101	696
ESC_MA6847AA	1589	156
ESC_MA6845AA	2309	669
ESC_MA6844AA	2144	257
ESC_MA6841AA	69	387
ESC_MA6840AA	7073	257
ESC_MA6839AA	101	696
ESC_MA6838AA	69	387
ESC_MA6837AA	617	511
ESC_MA6835AA	410	156
ESC_MA6833AA	21	156
ESC_MA6832AA	21	156
ESC_MA6831AA	21	156
ESC_MA6830AA	21	156
ESC_MA6829AA	29	156
ESC_MA6828AA	21	156
ESC_MA6824AA	773	71
ESC_MA6822AA	93	71
ESC_MA6820AA	95	738
ESC_MA6819AA	101	696
ESC_MA6818AA	2223	76
ESC_MA6817AA	744	512
ESC_MA6808AA	93	71
ESC_MA6805AA	101	696
ESC_MA6803AA	2705	504
ESC_MA6802AA	101	696
ESC_MA6801AA	101	696
ESC_MA6797AA	48	76
ESC_MA6795AA	162	924
ESC_MA6794AA	69	387
ESC_MA6793AA	10	512
ESC_MA6792AA	744	512
ESC_MA6790AA	1684	504
ESC_MA6789AA	69	387
ESC_MA6788AA	46	447
ESC_MA6786AA	1244	291
ESC_MA6785AA	4623	257
ESC_MA6784AA	25	234
ESC_MA6783AA	1196	257
ESC_MA6781AA	6712	50

ESC_MA6779AA	6712	50
ESC_MA6777AA	453	935
ESC_MA6776AA	4014	696
ESC_MA6775AA	744	512
ESC_MA6774AA	6272	862
ESC_MA6773AA	58	198
ESC_MA6772AA	4014	696
ESC_MA6771AA	69	387
ESC_MA6770AA	10	512
ESC_MA6769AA	2144	257
ESC_MA6767AA	10	512
ESC_MA6766AA	10	512
ESC_MA6765AA	10	512
ESC_MA6764AA	1011	198
ESC_MA6763AA	9159	512
ESC_MA6762AA	410	156
ESC_MA6761AA	25	234
ESC_MA6760AA	95	738
ESC_MA6759AA	206	286
ESC_MA6758AA	1485	408
ESC_MA6757AA	156	175
ESC_MA6756AA	1011	198
ESC_MA6755AA	4038	257
ESC_MA6754AA	4038	257
ESC_MA6753AA	4038	257
ESC_MA6752AA	795	924
ESC_MA6751AA	1193	761
ESC_MA6750AA	453	935
ESC_MA6749AA	131	653
ESC_MA6748AA	345	175
ESC_MA6747AA	95	738
ESC_MA6746AA	58	198
ESC_MA6745AA	207	512
ESC_MA6743AA	95	738
ESC_MA6742AA	398	445
ESC_MA6741AA	48	76
ESC_MA6740AA	226	512
ESC_MA6739AA	2144	257
ESC_MA6738AA	1196	257
ESC_MA6737AA	10	512
ESC_MA6736AA	410	156
ESC_MA6735AA	10	512
ESC_MA6734AA	410	156
ESC_MA6733AA	117	592
ESC_MA6732AA	10	512
ESC_MA6731AA	10	512
ESC_MA6730AA	48	76
ESC_MA6729AA	10	512
ESC_MA6728AA	10	512
ESC_MA6727AA	10	512

ESC_MA6726AA	10	512
ESC_MA6725AA	117	592
ESC_MA6724AA	10	512
ESC_MA6723AA	10	512
ESC_MA6720AA	877	935
ESC_MA6719AA	101	696
ESC_MA6718AA	847	257
ESC_MA6717AA	10	512
ESC_MA6716AA	48	76
ESC_MA6715AA	25	234
ESC_MA6714AA	93	71
ESC_MA6713AA	10	512
ESC_MA6712AA	1249	257
ESC_MA6710AA	69	387
ESC_MA6709AA	871	447
ESC_MA6708AA	1638	519
ESC_MA6707AA	192	696
ESC_MA6706AA	602	257
ESC_MA6705AA	3014	410
ESC_MA6704AA	1716	512
ESC_MA6703AA	410	156
ESC_MA6702AA	1716	512
ESC_MA6701AA	648	359
ESC_MA6700AA	3856	789
ESC_MA6699AA	206	286
ESC_MA6698AA	69	387
ESC_MA6697AA	48	76
ESC_MA6696AA	1196	257
ESC_MA6695AA	226	512
ESC_MA6694AA	6908	519
ESC_MA6693AA	1244	291
ESC_MA6692AA	69	387
ESC_MA6691AA	189	504
ESC_MA6690AA	206	286
ESC_MA6689AA	2935	512
ESC_MA6688AA	2345	795
ESC_MA6687AA	457	595
ESC_MA6686AA	707	257
ESC_MA6685AA	1011	198
ESC_MA6684AA	1136	108
ESC_MA6683AA	10	512
ESC_MA6682AA	206	286
ESC_MA6681AA	189	504
ESC_MA6680AA	744	512
ESC_MA6679AA	971	198
ESC_MA6678AA	410	156
ESC_MA6677AA	189	504
ESC_MA6676AA	1684	504
ESC_MA6675AA	746	789
ESC_MA6674AA	744	512

ESC_MA6673AA	48	76
ESC_MA6672AA	69	387
ESC_MA6671AA	101	696
ESC_MA6670AA	4541	676
ESC_MA6668AA	8539	257
ESC_MA6667AA	58	198
ESC_MA6666AA	1114	504
ESC_MA6665AA	770	587
ESC_MA6664AA	206	286
ESC_MA6663AA	3997	512
ESC_MA6662AA	10	512
ESC_MA6661AA	484	71
ESC_MA6660AA	10	512
ESC_MA6659AA	641	935
ESC_MA6658AA	69	387
ESC_MA6657AA	224	198
ESC_MA6656AA	58	198
ESC_MA6655AA	58	198
ESC_MA6654AA	191	198
ESC_MA6653AA	69	387
ESC_MA6652AA	7652	512
ESC_MA6651AA	167	512
ESC_MA6650AA	7652	512
ESC_MA6649AA	10	512
ESC_MA6648AA	131	653
ESC_MA6646AA	542	824
ESC_MA6645AA	744	512
ESC_MA6644AA	69	387
ESC_MA6643AA	58	198
ESC_MA6642AA	9159	512
ESC_MA6641AA	3489	962
ESC_MA6640AA	152	257
ESC_MA6639AA	152	257
ESC_MA6638AA	10	512
ESC_MA6637AA	398	445
ESC_MA6636AA	13	257
ESC_MA6635AA	3288	286
ESC_MA6634AA	6815	512
ESC_MA6633AA	101	696
ESC_MA6631AA	69	387
ESC_MA6630AA	69	387
ESC_MA6628AA	744	512
ESC_MA6627AA	641	935
ESC_MA6626AA	5487	198
ESC_MA6625AA	5951	406
ESC_MA6624AA	617	511
ESC_MA6623AA	361	512
ESC_MA6622AA	88	160
ESC_MA6621AA	131	653
ESC_MA6620AA	3997	512

ESC_MA6619AA	11	936
ESC_MA6618AA	69	387
ESC_MA6617AA	2708	512
ESC_MA6615AA	21	156
ESC_MA6614AA	218	519
ESC_MA6613AA	101	696
ESC_MA6612AA	542	824
ESC_MA6611AA	871	447
ESC_MA6610AA	10	512
ESC_MA6609AA	542	824
ESC_MA6608AA	10	512
ESC_MA6607AA	345	175
ESC_MA6606AA	3489	962
ESC_MA6605AA	1114	504
ESC_MA6604AA	189	504
ESC_MA6603AA	2179	924
ESC_MA6602AA	10	512
ESC_MA6601AA	2466	307
ESC_MA6598AA	10	512
ESC_MA6597AA	1081	198
ESC_MA6596AA	971	198
ESC_MA6595AA	453	935
ESC_MA6594AA	1684	504
ESC_MA6593AA	642	924
ESC_MA6592AA	10	512
ESC_MA6591AA	3944	512
ESC_MA6590AA	167	512
ESC_MA6589AA	656	789
ESC_MA6588AA	101	696
ESC_MA6587AA	206	286
ESC_MA6586AA	10	512
ESC_MA6585AA	641	935
ESC_MA6584AA	641	935
ESC_MA6583AA	410	156
ESC_MA6582AA	48	76
ESC_MA6581AA	971	198
ESC_MA6580AA	10	512
ESC_MA6579AA	2144	257
ESC_MA6578AA	1258	257
ESC_MA6577AA	5871	824
ESC_MA6576AA	10	512
ESC_MA6575AA	5503	512
ESC_MA6574AA	10	512
ESC_MA6573AA	410	156
ESC_MA6572AA	3941	71
ESC_MA6571AA	2144	257
ESC_MA6570AA	1081	198
ESC_MA6569AA	744	512
ESC_MA6568AA	3203	76
ESC_MA6567AA	1081	198

ESC_MA6566AA	1602	817
ESC_MA6565AA	410	156
ESC_MA6564AA	10	512
ESC_MA6563AA	206	286
ESC_MA6562AA	10	512
ESC_MA6561AA	542	824
ESC_MA6560AA	1716	512
ESC_MA6559AA	101	696
ESC_MA6558AA	3489	962
ESC_MA6557AA	10	512
ESC_MA6556AA	641	935
ESC_MA6555AA	744	512
ESC_MA6554AA	48	76
ESC_MA6553AA	3944	512
ESC_MA6552AA	10	512
ESC_MA6551AA	101	696
ESC_MA6550AA	2705	504
ESC_MA6549AA	716	795
ESC_MA6548AA	1196	257
ESC_MA6547AA	744	512
ESC_MA6546AA	10	512
ESC_MA6544AA	131	653
ESC_MA6543AA	795	924
ESC_MA6542AA	48	76
ESC_MA6541AA	101	696
ESC_MA6540AA	793	286
ESC_MA6539AA	165	504
ESC_MA6538AA	2466	307
ESC_MA6537AA	10	512
ESC_MA6536AA	2935	512
ESC_MA6535AA	10	512
ESC_MA6534AA	10	512
ESC_MA6533AA	101	696
ESC_MA6532AA	1114	504
ESC_MA6531AA	73	737
ESC_MA6530AA	4741	512
ESC_MA6529AA	58	198
ESC_MA6528AA	5215	76
ESC_MA6527AA	117	592
ESC_MA6526AA	10	512
ESC_MA6525AA	93	71
ESC_MA6524AA	10	512
ESC_MA6523AA	10	512
ESC_MA6522AA	871	447
ESC_MA6521AA	746	789
ESC_MA6520AA	69	387
ESC_MA6519AA	3933	318
ESC_MA6518AA	7056	672
ESC_MA6517AA	1244	291
ESC_MA6516AA	744	512

ESC_MA6515AA	1602	817
ESC_MA6514AA	48	76
ESC_MA6513AA	1244	291
ESC_MA6512AA	58	198
ESC_MA6511AA	117	592
ESC_MA6510AA	10	512
ESC_MA6509AA	10	512
ESC_MA6508AA	101	696
ESC_MA6507AA	1244	291
ESC_MA6506AA	7056	672
ESC_MA6505AA	10	512
ESC_MA6504AA	2705	504
ESC_MA6503AA	5503	512
ESC_MA6502AA	156	175
ESC_MA6501AA	744	512
ESC_MA6500AA	8262	234
ESC_MA6499AA	10	512
ESC_MA6497AA	8262	234
ESC_MA6496AA	1244	291
ESC_MA6495AA	5503	512
ESC_MA6494AA	3944	512
ESC_MA6493AA	3944	512
ESC_MA6492AA	1114	504
ESC_MA6491AA	710	291
ESC_MA6490AA	744	512
ESC_MA6488AA	410	156
ESC_MA6487AA	10	512
ESC_MA6486AA	48	76
ESC_MA6485AA	88	160
ESC_MA6484AA	10	512
ESC_MA6483AA	2253	71
ESC_MA6482AA	744	512
ESC_MA6481AA	10	512
ESC_MA6480AA	359	696
ESC_MA6479AA	7056	672
ESC_MA6478AA	101	696
ESC_MA6477AA	10	512
ESC_MA6476AA	359	696
ESC_MA6475AA	9159	512
ESC_MA6474AA	48	76
ESC_MA6473AA	2521	257
ESC_MA6472AA	398	445
ESC_MA6471AA	10	512
ESC_MA6470AA	1244	291
ESC_MA6469AA	101	696
ESC_MA6468AA	1244	291
ESC_MA6467AA	1684	504
ESC_MA6466AA	10	512
ESC_MA6465AA	10	512
ESC_MA6464AA	48	76

ESC_MA6463AA	795	924
ESC_MA6461AA	165	504
ESC_MA6460AA	7054	286
ESC_MA6459AA	410	156
ESC_MA6458AA	10	512
ESC_MA6457AA	101	696
ESC_MA6456AA	101	696
ESC_MA6455AA	4214	824
ESC_MA6454AA	9159	512
ESC_MA6453AA	69	387
ESC_MA6452AA	1602	817
ESC_MA6451AA	10	512
ESC_MA6450AA	4358	71
ESC_MA6449AA	162	924
ESC_MA6448AA	1642	198
ESC_MA6447AA	29	156
ESC_MA6446AA	993	789
ESC_MA6445AA	5995	504
ESC_MA6444AA	8539	257
ESC_MA6443AA	2526	257
ESC_MA6442AA	10	512
ESC_MA6441AA	10	512
ESC_MA6440AA	1081	198
ESC_MA6439AA	101	696
ESC_MA6438AA	34	504
ESC_MA6437AA	398	445
ESC_MA6436AA	2064	261
ESC_MA6435AA	10	512
ESC_MA6434AA	46	447
ESC_MA6433AA	10	512
ESC_MA6432AA	744	512
ESC_MA6431AA	10	512
ESC_MA6430AA	10	512
ESC_MA6428AA	2345	795
ESC_MA6427AA	744	512
ESC_MA6426AA	93	71
ESC_MA6425AA	10	512
ESC_MA6424AA	10	512
ESC_MA6423AA	48	76
ESC_MA6422AA	2345	795
ESC_MA6421AA	10	512
ESC_MA6420AA	410	156
ESC_MA6419AA	48	76
ESC_MA6418AA	871	447
ESC_MA6417AA	48	76
ESC_MA6416AA	2936	515
ESC_MA6415AA	2854	519
ESC_MA6414AA	58	198
ESC_MA6413AA	3274	512
ESC_MA6412AA	7331	512

ESC_MA6411AA	10	512
ESC_MA6410AA	206	286
ESC_MA6409AA	1415	504
ESC_MA6408AA	5086	286
ESC_MA6407AA	8539	257
ESC_MA6406AA	648	359
ESC_MA6405AA	2253	71
ESC_MA6404AA	101	696
ESC_MA6403AA	155	175
ESC_MA6402AA	189	504
ESC_MA6401AA	877	935
ESC_MA6400AA	2253	71
ESC_MA6399AA	4038	257
ESC_MA6398AA	1286	474
ESC_MA6397AA	2253	71
ESC_MA6396AA	2253	71
ESC_MA6395AA	10	512
ESC_MA6394AA	48	76
ESC_MA6393AA	189	504
ESC_MA6391AA	2253	71
ESC_MA6390AA	2253	71
ESC_MA6389AA	101	696
ESC_MA6388AA	744	512
ESC_MA6387AA	1114	504
ESC_MA6386AA	10	512
ESC_MA6385AA	48	76
ESC_MA6384AA	101	696
ESC_MA6383AA	129	257
ESC_MA6382AA	453	935
ESC_MA6381AA	165	504
ESC_MA6380AA	457	595
ESC_MA6379AA	7122	519
ESC_MA6378AA	165	504
ESC_MA6377AA	46	447
ESC_MA6376AA	101	696
ESC_MA6375AA	48	76
ESC_MA6374AA	156	175
ESC_MA6373AA	69	387
ESC_MA6372AA	206	286
ESC_MA6371AA	48	76
ESC_MA6370AA	871	447
ESC_MA6369AA	117	592
ESC_MA6368AA	1684	504
ESC_MA6367AA	1638	519
ESC_MA6366AA	542	824
ESC_MA6365AA	48	76
ESC_MA6364AA	131	653
ESC_MA6363AA	226	512
ESC_MA6362AA	3634	71
ESC_MA6361AA	2509	257

ESC_MA6360AA	453	935
ESC_MA6359AA	117	592
ESC_MA6358AA	2216	592
ESC_MA6357AA	1114	504
ESC_MA6356AA	354	15
ESC_MA6355AA	156	175
ESC_MA6354AA	101	696
ESC_MA6353AA	10	512
ESC_MA6352AA	5542	76
ESC_MA6351AA	4014	696
ESC_MA6350AA	5222	311
ESC_MA6349AA	641	935
ESC_MA6348AA	189	504
ESC_MA6347AA	101	696
ESC_MA6346AA	206	286
ESC_MA6345AA	101	696
ESC_MA6344AA	8539	257
ESC_MA6343AA	9159	512
ESC_MA6342AA	1308	257
ESC_MA6341AA	10	512
ESC_MA6340AA	1244	291
ESC_MA6339AA	165	504
ESC_MA6338AA	1244	291
ESC_MA6337AA	165	504
ESC_MA6336AA	10	512
ESC_MA6335AA	101	696
ESC_MA6334AA	8154	885
ESC_MA6333AA	1249	257
ESC_MA6332AA	1244	291
ESC_MA6331AA	795	924
ESC_MA6330AA	101	696
ESC_MA6328AA	1244	291
ESC_MA6325AA	10	512
ESC_MA6324AA	10	512
ESC_MA6323AA	101	696
ESC_MA6322AA	69	387
ESC_MA6321AA	69	387
ESC_MA6319AA	10	512
ESC_MA6318AA	871	447
ESC_MA6317AA	410	156
ESC_MA6316AA	7056	672
ESC_MA6315AA	1716	512
ESC_MA6314AA	10	512
ESC_MA6304AA	10	512
ESC_MA6303AA	10	512
ESC_MA6300AA	25	234
ESC_MA6299AA	10	512
ESC_MA6298AA	165	504
ESC_MA6297AA	25	234
ESC_MA6296AA	25	234

ESC_MA6294AA	716	795
ESC_MA6293AA	3937	76
ESC_MA6292AA	10	512
ESC_MA6291AA	1244	291
ESC_MA6290AA	1244	291
ESC_MA6289AA	6438	65
ESC_MA6288AA	9159	512
ESC_MA6287AA	795	924
ESC_MA6286AA	9159	512
ESC_MA6284AA	101	696
ESC_MA6283AA	716	795
ESC_MA6281AA	13	257
ESC_MA6279AA	165	504
ESC_MA6278AA	165	504
ESC_MA6276AA	101	696
ESC_MA6275AA	1642	198
ESC_MA6274AA	345	175
ESC_MA6273AA	9159	512
ESC_MA6271AA	165	504
ESC_MA6270AA	10	512
ESC_MA6269AA	542	824
ESC_MA6268AA	1114	504
ESC_MA6267AA	206	286
ESC_MA6266AA	1968	76
ESC_MA6265AA	165	504
ESC_MA6264AA	206	286
ESC_MA6263AA	165	504
ESC_MA6262AA	48	76
ESC_MA6261AA	744	512
ESC_MA6260AA	410	156
ESC_MA6259AA	2179	924
ESC_MA6258AA	57	50
ESC_MA6257AA	69	387
ESC_MA6256AA	57	50
ESC_MA6255AA	117	592
ESC_MA6254AA	1244	291
ESC_MA6253AA	457	595
ESC_MA6252AA	3274	512
ESC_MA6251AA	7122	519
ESC_MA6250AA	117	592
ESC_MA6249AA	95	738
ESC_MA6248AA	6706	76
ESC_MA6247AA	6335	504
ESC_MA6246AA	48	76
ESC_MA6245AA	2913	286
ESC_MA6244AA	799	852
ESC_MA6243AA	410	156
ESC_MA6242AA	10	512
ESC_MA6241AA	2144	257
ESC_MA6239AA	69	387

ESC_MA6237AA	101	696
ESC_MA6236AA	206	286
ESC_MA6235AA	10	512
ESC_MA6234AA	641	935
ESC_MA6233AA	117	592
ESC_MA6229AA	9415	789
ESC_MA6228AA	9414	79
ESC_MA6227AA	7452	924
ESC_MA6226AA	9413	175
ESC_MA6225AA	7452	924
ESC_MA6224AA	9403	512
ESC_MA6223AA	9378	519
ESC_MA6222AA	9412	738
ESC_MA6221AA	9412	738
ESC_MA6220AA	9412	738
ESC_MA6219AA	9409	962
ESC_MA6218AA	9408	923
ESC_MA6217AA	9411	286
ESC_MA6216AA	9405	257
ESC_MA6215AA	9410	387
ESC_MA6214AA	9405	257
ESC_MA6212AA	9407	76
ESC_MA6211AA	9392	936
ESC_MA6209AA	9401	198
ESC_MA6208AA	9403	512
ESC_MA6207AA	9399	785
ESC_MA6206AA	9404	257
ESC_MA6205AA	9402	789
ESC_MA6204AA	9400	810
ESC_MA6203AA	9396	519
ESC_MA6202AA	9397	257
ESC_MA6201AA	9398	76
ESC_MA6200AA	9378	519
ESC_MA6199AA	9395	284
ESC_MA6198AA	9392	936
ESC_MA6197AA	9392	936
ESC_MA6196AA	9394	410
ESC_MA6195AA	9393	404
ESC_MA6194AA	9389	198
ESC_MA6193AA	9391	257
ESC_MA6192AA	9390	696
ESC_MA6191AA	9388	880
ESC_MA6190AA	9386	286
ESC_MA6189AA	9387	495
ESC_MA6188AA	9385	972
ESC_MA6187AA	9384	508
ESC_MA6186AA	9378	519
ESC_MA6185AA	9383	195
ESC_MA6184AA	9382	512
ESC_MA6183AA	9379	795

ESC_MA6182AA	9378	519
ESC_MA6181AA	9380	824
ESC_MA6180AA	9381	204
ESC_MA6179AA	9377	474
ESC_MA6178AA	9375	696
ESC_MA6177AA	3570	156
ESC_MA6176AA	9373	1004
ESC_MA6175AA	131	653
ESC_MA6174AA	21	156
ESC_MA6173AA	21	156
ESC_MA6172AA	32	270
ESC_MA6171AA	3589	156
ESC_MA6170AA	21	156
ESC_MA6169AA	11	936
ESC_MA6168AA	4496	924
ESC_MA6167AA	342	286
ESC_MA6166AA	4496	924
ESC_MA6165AA	4038	257
ESC_MA6164AA	342	286
ESC_MA6163AA	5172	156
ESC_MA6162AA	1642	198
ESC_MA6161AA	1642	198
ESC_MA6160AA	58	198
ESC_MA6159AA	88	160
ESC_MA6158AA	752	512
ESC_MA6157AA	86	935
ESC_MA6156AA	8140	878
ESC_MA6155AA	180	234
ESC_MA6154AA	8253	198
ESC_MA6153AA	4198	878
ESC_MA6152AA	101	696
ESC_MA6151AA	1642	198
ESC_MA6150AA	7504	257
ESC_MA6149AA	329	355
ESC_MA6148AA	7504	257
ESC_MA6147AA	723	156
ESC_MA6146AA	329	355
ESC_MA6145AA	329	355
ESC_MA6144AA	329	355
ESC_MA6143AA	718	257
ESC_MA6142AA	11	936
ESC_MA6141AA	21	156
ESC_MA6140AA	21	156
ESC_MA6139AA	9368	198
ESC_MA6138AA	21	156
ESC_MA6137AA	56	198
ESC_MA6136AA	718	257
ESC_MA6135AA	206	286
ESC_MA6134AA	8405	696
ESC_MA6133AA	718	257

ESC_MA6132AA	515	1004
ESC_MA6131AA	515	1004
ESC_MA6130AA	56	198
ESC_MA6129AA	515	1004
ESC_MA6128AA	56	198
ESC_MA6127AA	329	355
ESC_MA6126AA	7504	257
ESC_MA6125AA	206	286
ESC_MA6124AA	17	191
ESC_MA6123AA	56	198
ESC_MA6122AA	56	198
ESC_MA6121AA	58	198
ESC_MA6120AA	56	198
ESC_MA6119AA	5172	156
ESC_MA6118AA	56	198
ESC_MA6117AA	56	198
ESC_MA6116AA	718	257
ESC_MA6115AA	718	257
ESC_MA6114AA	718	257
ESC_MA6113AA	515	1004
ESC_MA6112AA	1611	198
ESC_MA6111AA	515	1004
ESC_MA6110AA	515	1004
ESC_MA6109AA	515	1004
ESC_MA6108AA	718	257
ESC_MA6107AA	718	257
ESC_MA6106AA	718	257
ESC_MA6105AA	17	191
ESC_MA6104AA	2387	261
ESC_MA6103AA	718	257
ESC_MA6102AA	9368	198
ESC_MA6101AA	718	257
ESC_MA6100AA	718	257
ESC_MA6099AA	56	198
ESC_MA6098AA	56	198
ESC_MA6097AA	21	156
ESC_MA6096AA	21	156
ESC_MA6095AA	21	156
ESC_MA6094AA	1642	198
ESC_MA6093AA	56	198
ESC_MA6092AA	56	198
ESC_MA6091AA	56	198
ESC_MA6090AA	56	198
ESC_MA6089AA	56	198
ESC_MA6088AA	21	156
ESC_MA6087AA	21	156
ESC_MA6086AA	746	789
ESC_MA6085AA	21	156
ESC_MA6084AA	21	156
ESC_MA6083AA	2715	588

ESC_MA6082AA	21	156
ESC_MA6081AA	21	156
ESC_MA6080AA	56	198
ESC_MA6079AA	56	198
ESC_MA6078AA	21	156
ESC_MA6077AA	56	198
ESC_MA6076AA	21	156
ESC_MA6075AA	2217	62
ESC_MA6074AA	21	156
ESC_MA6073AA	1079	235
ESC_MA6072AA	21	156
ESC_MA6071AA	206	286
ESC_MA6070AA	21	156
ESC_MA6069AA	21	156
ESC_MA6068AA	515	1004
ESC_MA6067AA	2217	62
ESC_MA6066AA	718	257
ESC_MA6065AA	21	156
ESC_MA6064AA	515	1004
ESC_MA6063AA	718	257
ESC_MA6062AA	2715	588
ESC_MA6061AA	2715	588
ESC_MA6060AA	329	355
ESC_MA6059AA	515	1004
ESC_MA6058AA	718	257
ESC_MA6057AA	21	156
ESC_MA6056AA	2715	588
ESC_MA6055AA	8405	696
ESC_MA6054AA	56	198
ESC_MA6053AA	718	257
ESC_MA6052AA	56	198
ESC_MA6051AA	152	257
ESC_MA6050AA	6666	261
ESC_MA6049AA	152	257
ESC_MA6048AA	245	261
ESC_MA6047AA	1286	474
ESC_MA6046AA	354	15
ESC_MA6045AA	223	198
ESC_MA6044AA	648	359
ESC_MA6043AA	1665	601
ESC_MA6042AA	131	653
ESC_MA6041AA	9360	504
ESC_MA6040AA	9362	410
ESC_MA6039AA	9361	286
ESC_MA6038AA	10	512
ESC_MA6037AA	10	512
ESC_MA6036AA	12	626
ESC_MA6035AA	58	198
ESC_MA6034AA	1091	512
ESC_MA6033AA	58	198

ESC_MA6032AA	398	445
ESC_MA6031AA	9355	198
ESC_MA6030AA	23	156
ESC_MA6029AA	101	696
ESC_MA6028AA	1421	447
ESC_MA6027AA	58	198
ESC_MA6026AA	1125	198
ESC_MA6025AA	1125	198
ESC_MA6022AA	1495	311
ESC_MA6021AA	56	198
ESC_MA6019AA	1125	198
ESC_MA6018AA	10	512
ESC_MA6017AA	301	307
ESC_MA6016AA	301	307
ESC_MA6015AA	301	307
ESC_MA6014AA	21	156
ESC_MA6013AA	21	156
ESC_MA6012AA	21	156
ESC_MA6011AA	21	156
ESC_MA6010AA	21	156
ESC_MA6009AA	21	156
ESC_MA6008AA	9354	198
ESC_MA5970AA	9353	936
ESC_MA5969AA	301	307
ESC_MA5968AA	301	307
ESC_MA5967AA	21	156
ESC_MA5966AA	21	156
ESC_MA5965AA	5901	234
ESC_MA5964AA	1049	175
ESC_MA5963AA	152	257
ESC_MA5962AA	152	257
ESC_MA5961AA	152	257
ESC_MA5960AA	152	257
ESC_MA5959AA	245	261
ESC_MA5958AA	245	261
ESC_MA5957AA	245	261
ESC_MA5956AA	245	261
ESC_MA5955AA	152	257
ESC_MA5954AA	152	257
ESC_MA5953AA	245	261
ESC_MA5952AA	6880	726
ESC_MA5951AA	6880	726
ESC_MA5950AA	11	936
ESC_MA5949AA	21	156
ESC_MA5948AA	17	191
ESC_MA5947AA	17	191
ESC_MA5946AA	21	156
ESC_MA5945AA	17	191
ESC_MA5944AA	16	156
ESC_MA5943AA	16	156

ESC_MA5942AA	21	156
ESC_MA5941AA	16	156
ESC_MA5940AA	69	387
ESC_MA5939AA	414	387
ESC_MA5938AA	31	387
ESC_MA5937AA	17	191
ESC_MA5936AA	1611	198
ESC_MA5935AA	1380	914
ESC_MA5934AA	7858	156
ESC_MA5933AA	442	257
ESC_MA5932AA	661	92
ESC_MA5931AA	11	936
ESC_MA5930AA	131	653
ESC_MA5929AA	162	924
ESC_MA5927AA	448	257
ESC_MA5926AA	17	191
ESC_MA5925AA	3695	924
ESC_MA5924AA	21	156
ESC_MA5923AA	16	156
ESC_MA5922AA	655	926
ESC_MA5921AA	5292	738
ESC_MA5920AA	1079	235
ESC_MA5919AA	1121	198
ESC_MA5918AA	162	924
ESC_MA5917AA	345	175
ESC_MA5916AA	155	175
ESC_MA5915AA	48	76
ESC_MA5914AA	1125	198
ESC_MA5913AA	155	175
ESC_MA5912AA	109	257
ESC_MA5911AA	88	160
ESC_MA5910AA	95	738
ESC_MA5909AA	731	285
ESC_MA5908AA	21	156
ESC_MA5907AA	21	156
ESC_MA5906AA	21	156
ESC_MA5905AA	16	156
ESC_MA5904AA	17	191
ESC_MA5903AA	17	191
ESC_MA5902AA	173	254
ESC_MA5901AA	5292	738
ESC_MA5900AA	17	191
ESC_MA5899AA	504	738
ESC_MA5898AA	16	156
ESC_MA5897AA	655	926
ESC_MA5896AA	152	257
ESC_MA5895AA	152	257
ESC_MA5894AA	21	156
ESC_MA5893AA	11	936
ESC_MA5892AA	16	156

ESC_MA5891AA	9349	156
ESC_MA5890AA	16	156
ESC_MA5889AA	1892	846
ESC_MA5888AA	300	286
ESC_MA5887AA	1892	846
ESC_MA5886AA	245	261
ESC_MA5885AA	152	257
ESC_MA5884AA	245	261
ESC_MA5883AA	145	256
ESC_MA5882AA	1273	256
ESC_MA5881AA	252	190
ESC_MA5880AA	11	936
ESC_MA5879AA	21	156
ESC_MA5878AA	11	936
ESC_MA5877AA	21	156
ESC_MA5876AA	17	191
ESC_MA5875AA	2144	257
ESC_MA5874AA	1079	235
ESC_MA5873AA	187	257
ESC_MA5872AA	329	355
ESC_MA5871AA	9348	453
ESC_MA5870AA	69	387
ESC_MA5869AA	88	160
ESC_MA5868AA	10	512
ESC_MA5867AA	1727	257
ESC_MA5866AA	329	355
ESC_MA5865AA	11	936
ESC_MA5864AA	245	261
ESC_MA5863AA	145	256
ESC_MA5862AA	245	261
ESC_MA5861AA	1503	257
ESC_MA5860AA	152	257
ESC_MA5859AA	245	261
ESC_MA5858AA	152	257
ESC_MA5857AA	1503	257
ESC_MA5856AA	152	257
ESC_MA5855AA	245	261
ESC_MA5854AA	152	257
ESC_MA5853AA	152	257
ESC_MA5852AA	245	261
ESC_MA5851AA	245	261
ESC_MA5850AA	245	261
ESC_MA5849AA	245	261
ESC_MA5848AA	245	261
ESC_MA5847AA	245	261
ESC_MA5846AA	245	261
ESC_MA5845AA	40	198
ESC_MA5844AA	2966	936
ESC_MA5843AA	11	936
ESC_MA5842AA	11	936

ESC_MA5841AA	11	936
ESC_MA5840AA	101	696
ESC_MA5839AA	21	156
ESC_MA5838AA	306	286
ESC_MA5837AA	9345	155
ESC_MA5836AA	11	936
ESC_MA5835AA	21	156
ESC_MA5834AA	17	191
ESC_MA5833AA	21	156
ESC_MA5832AA	17	191
ESC_MA5831AA	4382	900
ESC_MA5830AA	56	198
ESC_MA5829AA	58	198
ESC_MA5828AA	4086	842
ESC_MA5827AA	937	198
ESC_MA5826AA	10	512
ESC_MA5825AA	131	653
ESC_MA5824AA	162	924
ESC_MA5823AA	187	257
ESC_MA5822AA	93	71
ESC_MA5821AA	1080	198
ESC_MA5820AA	9346	286
ESC_MA5819AA	392	234
ESC_MA5818AA	10	512
ESC_MA5817AA	10	512
ESC_MA5816AA	10	512
ESC_MA5815AA	336	878
ESC_MA5814AA	4198	878
ESC_MA5813AA	155	175
ESC_MA5812AA	58	198
ESC_MA5811AA	8351	140
ESC_MA5810AA	58	198
ESC_MA5809AA	88	160
ESC_MA5808AA	189	504
ESC_MA5807AA	131	653
ESC_MA5806AA	877	935
ESC_MA5805AA	48	76
ESC_MA5804AA	5502	307
ESC_MA5803AA	1679	160
ESC_MA5802AA	1623	891
ESC_MA5801AA	117	592
ESC_MA5800AA	847	257
ESC_MA5799AA	10	512
ESC_MA5798AA	1204	961
ESC_MA5797AA	70	670
ESC_MA5796AA	5906	878
ESC_MA5795AA	58	198
ESC_MA5794AA	10	512
ESC_MA5793AA	1679	160
ESC_MA5792AA	155	175

ESC_MA5791AA	223	198
ESC_MA5790AA	131	653
ESC_MA5789AA	648	359
ESC_MA5788AA	1722	599
ESC_MA5787AA	2015	684
ESC_MA5786AA	131	653
ESC_MA5785AA	12	626
ESC_MA5784AA	648	359
ESC_MA5783AA	127	738
ESC_MA5782AA	12	626
ESC_MA5781AA	131	653
ESC_MA5780AA	73	737
ESC_MA5779AA	131	653
ESC_MA5778AA	131	653
ESC_MA5777AA	1485	408
ESC_MA5776AA	11	936
ESC_MA5775AA	4305	512
ESC_MA5774AA	5342	765
ESC_MA5773AA	737	175
ESC_MA5772AA	200	198
ESC_MA5771AA	443	257
ESC_MA5770AA	11	936
ESC_MA5769AA	99	198
ESC_MA5768AA	1819	808
ESC_MA5767AA	32	270
ESC_MA5766AA	3101	160
ESC_MA5765AA	131	653
ESC_MA5764AA	21	156
ESC_MA5763AA	17	191
ESC_MA5762AA	5390	83
ESC_MA5761AA	737	175
ESC_MA5760AA	17	191
ESC_MA5759AA	11	936
ESC_MA5758AA	9347	170
ESC_MA5757AA	11	936
ESC_MA5756AA	11	936
ESC_MA5755AA	2739	76
ESC_MA5754AA	155	175
ESC_MA5753AA	4450	844
ESC_MA5752AA	10	512
ESC_MA5751AA	2715	588
ESC_MA5750AA	167	512
ESC_MA5749AA	329	355
ESC_MA5748AA	372	424
ESC_MA5727AA	11	936
ESC_MA5726AA	1589	156
ESC_MA5725AA	226	512
ESC_MA5724AA	414	387
ESC_MA5723AA	131	653
ESC_MA5722AA	10	512

ESC_MA5721AA	1177	669
ESC_MA5720AA	167	512
ESC_MA5719AA	6082	789
ESC_MA5718AA	648	359
ESC_MA5717AA	405	923
ESC_MA5716AA	69	387
ESC_MA5715AA	7304	511
ESC_MA5714AA	410	156
ESC_MA5711AA	1193	761
ESC_MA5710AA	1193	761
ESC_MA5709AA	131	653
ESC_MA5707AA	31	387
ESC_MA5706AA	9344	156
ESC_MA5705AA	224	198
ESC_MA5704AA	38	669
ESC_MA5702AA	9167	515
ESC_MA5701AA	362	234
ESC_MA5698AA	44	512
ESC_MA5695AA	69	387
ESC_MA5693AA	1193	761
ESC_MA5691AA	226	512
ESC_MA5689AA	3268	669
ESC_MA5688AA	38	669
ESC_MA5687AA	9166	504
ESC_MA5685AA	9170	445
ESC_MA5684AA	1193	761
ESC_MA5683AA	1193	761
ESC_MA5677AA	1125	198
ESC_MA5676AA	58	198
ESC_MA5675AA	1408	445
ESC_MA5674AA	10	512
ESC_MA5673AA	10	512
ESC_MA5672AA	88	160
ESC_MA5671AA	1121	198
ESC_MA5670AA	1123	257
ESC_MA5669AA	10	512
ESC_MA5668AA	1121	198
ESC_MA5667AA	2598	198
ESC_MA5666AA	9341	108
ESC_MA5665AA	457	595
ESC_MA5664AA	115	669
ESC_MA5663AA	4937	519
ESC_MA5662AA	2485	212
ESC_MA5661AA	457	595
ESC_MA5660AA	69	387
ESC_MA5659AA	453	935
ESC_MA5658AA	457	595
ESC_MA5657AA	2179	924
ESC_MA5656AA	4980	445
ESC_MA5655AA	2179	924

ESC_MA5654AA	429	654
ESC_MA5653AA	373	71
ESC_MA5652AA	38	669
ESC_MA5651AA	38	669
ESC_MA5650AA	38	669
ESC_MA5649AA	1629	924
ESC_MA5648AA	155	175
ESC_MA5647AA	457	595
ESC_MA5646AA	69	387
ESC_MA5645AA	155	175
ESC_MA5644AA	355	764
ESC_MA5643AA	2485	212
ESC_MA5642AA	1629	924
ESC_MA5641AA	2485	212
ESC_MA5640AA	9340	447
ESC_MA5639AA	1629	924
ESC_MA5638AA	457	595
ESC_MA5637AA	429	654
ESC_MA5636AA	457	595
ESC_MA5635AA	155	175
ESC_MA5634AA	457	595
ESC_MA5633AA	155	175
ESC_MA5632AA	388	156
ESC_MA5631AA	753	842
ESC_MA5630AA	155	175
ESC_MA5629AA	695	519
ESC_MA5628AA	1303	791
ESC_MA5627AA	155	175
ESC_MA5626AA	115	669
ESC_MA5625AA	453	935
ESC_MA5624AA	10	512
ESC_MA5623AA	152	257
ESC_MA5622AA	155	175
ESC_MA5621AA	457	595
ESC_MA5620AA	371	50
ESC_MA5619AA	457	595
ESC_MA5618AA	117	592
ESC_MA5617AA	23	156
ESC_MA5616AA	457	595
ESC_MA5615AA	117	592
ESC_MA5614AA	23	156
ESC_MA5613AA	48	76
ESC_MA5612AA	371	50
ESC_MA5611AA	429	654
ESC_MA5610AA	2179	924
ESC_MA5609AA	1163	579
ESC_MA5608AA	117	592
ESC_MA5607AA	4980	445
ESC_MA5606AA	38	669
ESC_MA5605AA	3107	512

ESC_MA5604AA	746	789
ESC_MA5603AA	457	595
ESC_MA5602AA	155	175
ESC_MA5601AA	155	175
ESC_MA5600AA	140	738
ESC_MA5599AA	355	764
ESC_MA5598AA	115	669
ESC_MA5597AA	117	592
ESC_MA5596AA	23	156
ESC_MA5595AA	457	595
ESC_MA5594AA	457	595
ESC_MA5593AA	155	175
ESC_MA5592AA	10	512
ESC_MA5591AA	10	512
ESC_MA5590AA	10	512
ESC_MA5589AA	9334	257
ESC_MA5588AA	10	512
ESC_MA5587AA	1125	198
ESC_MA5586AA	1121	198
ESC_MA5585AA	10	512
ESC_MA5584AA	245	261
ESC_MA5583AA	152	257
ESC_MA5582AA	245	261
ESC_MA5581AA	245	261
ESC_MA5580AA	245	261
ESC_MA5579AA	245	261
ESC_MA5578AA	17	191
ESC_MA5577AA	5292	738
ESC_MA5576AA	32	270
ESC_MA5575AA	17	191
ESC_MA5574AA	1967	191
ESC_MA5573AA	5516	936
ESC_MA5572AA	58	198
ESC_MA5571AA	9333	311
ESC_MA5569AA	88	160
ESC_MA5568AA	101	696
ESC_MA5567AA	603	198
ESC_MA5566AA	4151	971
ESC_MA5565AA	8351	140
ESC_MA5564AA	101	696
ESC_MA5563AA	1564	512
ESC_MA5562AA	6345	199
ESC_MA5561AA	1967	191
ESC_MA5560AA	744	512
ESC_MA5559AA	152	257
ESC_MA5558AA	152	257
ESC_MA5557AA	152	257
ESC_MA5556AA	152	257
ESC_MA5555AA	17	191
ESC_MA5554AA	11	936

ESC_MA5552AA	9329	198
ESC_MA5550AA	93	71
ESC_MA5549AA	300	286
ESC_MA5548AA	25	234
ESC_MA5547AA	101	696
ESC_MA5546AA	9332	257
ESC_MA5545AA	17	191
ESC_MA5544AA	442	257
ESC_MA5543AA	6978	512
ESC_MA5542AA	86	935
ESC_MA5541AA	6981	198
ESC_MA5540AA	1245	270
ESC_MA5539AA	6980	926
ESC_MA5538AA	1245	270
ESC_MA5537AA	10	512
ESC_MA5536AA	6985	915
ESC_MA5535AA	10	512
ESC_MA5534AA	1686	926
ESC_MA5533AA	1686	926
ESC_MA5532AA	10	512
ESC_MA5531AA	1079	235
ESC_MA5530AA	10	512
ESC_MA5529AA	1204	961
ESC_MA5528AA	86	935
ESC_MA5527AA	3211	512
ESC_MA5526AA	641	935
ESC_MA5525AA	10	512
ESC_MA5524AA	162	924
ESC_MA5523AA	1146	198
ESC_MA5522AA	10	512
ESC_MA5521AA	1146	198
ESC_MA5520AA	6982	214
ESC_MA5519AA	641	935
ESC_MA5518AA	10	512
ESC_MA5517AA	6979	198
ESC_MA5516AA	6984	156
ESC_MA5515AA	6983	512
ESC_MA5514AA	10	512
ESC_MA5513AA	1709	936
ESC_MA5512AA	162	924
ESC_MA5511AA	906	198
ESC_MA5510AA	162	924
ESC_MA5509AA	224	198
ESC_MA5508AA	10	512
ESC_MA5507AA	2226	512
ESC_MA5506AA	1250	926
ESC_MA5505AA	4164	880
ESC_MA5504AA	1250	926
ESC_MA5503AA	6986	926
ESC_MA5502AA	10	512

ESC_MA5501AA	162	924
ESC_MA5500AA	603	198
ESC_MA5499AA	641	935
ESC_MA5498AA	641	935
ESC_MA5497AA	21	156
ESC_MA5496AA	21	156
ESC_MA5495AA	21	156
ESC_MA5494AA	11	936
ESC_MA5492AA	9329	198
ESC_MA5491AA	11	936
ESC_MA5490AA	2836	221
ESC_MA5489AA	1786	170
ESC_MA5488AA	342	286
ESC_MA5487AA	7217	156
ESC_MA5486AA	245	261
ESC_MA5485AA	410	156
ESC_MA5484AA	152	257
ESC_MA5483AA	11	936
ESC_MA5482AA	11	936
ESC_MA5481AA	21	156
ESC_MA5480AA	17	191
ESC_MA5479AA	961	623
ESC_MA5478AA	336	878
ESC_MA5477AA	7907	257
ESC_MA5476AA	1844	198
ESC_MA5475AA	372	424
ESC_MA5474AA	33	198
ESC_MA5473AA	297	234
ESC_MA5472AA	555	653
ESC_MA5471AA	9327	198
ESC_MA5470AA	7907	257
ESC_MA5469AA	164	175
ESC_MA5468AA	744	512
ESC_MA5467AA	1262	737
ESC_MA5466AA	10	512
ESC_MA5465AA	4279	900
ESC_MA5464AA	10	512
ESC_MA5463AA	372	424
ESC_MA5462AA	1079	235
ESC_MA5461AA	162	924
ESC_MA5460AA	162	924
ESC_MA5459AA	491	626
ESC_MA5458AA	646	738
ESC_MA5457AA	162	924
ESC_MA5456AA	23	156
ESC_MA5455AA	12	626
ESC_MA5454AA	372	424
ESC_MA5453AA	10	512
ESC_MA5452AA	156	175
ESC_MA5451AA	58	198

ESC_MA5450AA	162	924
ESC_MA5449AA	23	156
ESC_MA5448AA	9326	234
ESC_MA5447AA	73	737
ESC_MA5446AA	372	424
ESC_MA5445AA	196	257
ESC_MA5444AA	58	198
ESC_MA5443AA	660	286
ESC_MA5442AA	127	738
ESC_MA5441AA	8492	234
ESC_MA5438AA	152	257
ESC_MA5437AA	152	257
ESC_MA5436AA	5245	118
ESC_MA5435AA	17	191
ESC_MA5434AA	189	504
ESC_MA5433AA	17	191
ESC_MA5432AA	11	936
ESC_MA5431AA	642	924
ESC_MA5430AA	162	924
ESC_MA5429AA	11	936
ESC_MA5427AA	131	653
ESC_MA5425AA	245	261
ESC_MA5424AA	245	261
ESC_MA5423AA	1503	257
ESC_MA5422AA	245	261
ESC_MA5421AA	245	261
ESC_MA5420AA	5517	257
ESC_MA5419AA	245	261
ESC_MA5418AA	245	261
ESC_MA5417AA	145	256
ESC_MA5416AA	152	257
ESC_MA5415AA	152	257
ESC_MA5414AA	1503	257
ESC_MA5413AA	1753	261
ESC_MA5412AA	245	261
ESC_MA5411AA	152	257
ESC_MA5410AA	152	257
ESC_MA5409AA	152	257
ESC_MA5408AA	152	257
ESC_MA5407AA	737	175
ESC_MA5406AA	8561	936
ESC_MA5405AA	10	512
ESC_MA5404AA	33	198
ESC_MA5403AA	738	598
ESC_MA5402AA	10	512
ESC_MA5401AA	99	198
ESC_MA5400AA	2467	673
ESC_MA5399AA	21	156
ESC_MA5397AA	17	191
ESC_MA5396AA	17	191

ESC_MA5395AA	17	191
ESC_MA5394AA	1967	191
ESC_MA5393AA	17	191
ESC_MA5392AA	17	191
ESC_MA5391AA	17	191
ESC_MA5390AA	17	191
ESC_MA5389AA	17	191
ESC_MA5388AA	17	191
ESC_MA5387AA	1967	191
ESC_MA5386AA	17	191
ESC_MA5385AA	17	191
ESC_MA5384AA	17	191
ESC_MA5383AA	17	191
ESC_MA5382AA	17	191
ESC_MA5381AA	386	191
ESC_MA5380AA	17	191
ESC_MA5379AA	9323	198
ESC_MA5378AA	9317	257
ESC_MA5377AA	3057	672
ESC_MA5376AA	21	156
ESC_MA5375AA	1642	198
ESC_MA5374AA	21	156
ESC_MA5373AA	200	198
ESC_MA5372AA	11	936
ESC_MA5371AA	7858	156
ESC_MA5370AA	747	640
ESC_MA5369AA	16	156
ESC_MA5368AA	7249	257
ESC_MA5367AA	5617	204
ESC_MA5366AA	1049	175
ESC_MA5365AA	349	670
ESC_MA5364AA	206	286
ESC_MA5363AA	21	156
ESC_MA5362AA	9315	504
ESC_MA5361AA	10	512
ESC_MA5360AA	10	512
ESC_MA5359AA	10	512
ESC_MA5358AA	10	512
ESC_MA5357AA	372	424
ESC_MA5356AA	625	756
ESC_MA5355AA	12	626
ESC_MA5354AA	372	424
ESC_MA5353AA	58	198
ESC_MA5352AA	424	234
ESC_MA5351AA	10	512
ESC_MA5350AA	410	156
ESC_MA5349AA	155	175
ESC_MA5348AA	1079	235
ESC_MA5347AA	2280	212
ESC_MA5346AA	9316	861

ESC_MA5345AA	223	198
ESC_MA5344AA	162	924
ESC_MA5343AA	1049	175
ESC_MA5342AA	10	512
ESC_MA5341AA	1079	235
ESC_MA5340AA	4162	180
ESC_MA5339AA	961	623
ESC_MA5338AA	3580	257
ESC_MA5337AA	3576	175
ESC_MA5336AA	12	626
ESC_MA5335AA	152	257
ESC_MA5334AA	11	936
ESC_MA5333AA	11	936
ESC_MA5332AA	11	936
ESC_MA5331AA	11	936
ESC_MA5330AA	10	512
ESC_MA5329AA	10	512
ESC_MA5328AA	10	512
ESC_MA5327AA	10	512
ESC_MA5326AA	10	512
ESC_MA5325AA	10	512
ESC_MA5324AA	10	512
ESC_MA5323AA	10	512
ESC_MA5322AA	10	512
ESC_MA5321AA	10	512
ESC_MA5320AA	10	512
ESC_MA5319AA	48	76
ESC_MA5318AA	301	307
ESC_MA5317AA	21	156
ESC_MA5315AA	21	156
ESC_MA5314AA	21	156
ESC_MA5313AA	21	156
ESC_MA5312AA	21	156
ESC_MA5311AA	21	156
ESC_MA5310AA	21	156
ESC_MA5309AA	21	156
ESC_MA5308AA	21	156
ESC_MA5307AA	746	789
ESC_MA5306AA	245	261
ESC_MA5305AA	152	257
ESC_MA5304AA	288	102
ESC_MA5303AA	1503	257
ESC_MA5302AA	245	261
ESC_MA5301AA	152	257
ESC_MA5300AA	152	257
ESC_MA5299AA	152	257
ESC_MA5298AA	152	257
ESC_MA5297AA	21	156
ESC_MA5296AA	33	198
ESC_MA5295AA	9313	445

ESC_MA5294AA	21	156
ESC_MA5293AA	11	936
ESC_MA5292AA	11	936
ESC_MA5291AA	11	936
ESC_MA5290AA	152	257
ESC_MA5289AA	152	257
ESC_MA5288AA	21	156
ESC_MA5287AA	11	936
ESC_MA5286AA	655	926
ESC_MA5285AA	11	936
ESC_MA5284AA	9312	140
ESC_MA5283AA	245	261
ESC_MA5282AA	245	261
ESC_MA5281AA	245	261
ESC_MA5280AA	245	261
ESC_MA5279AA	152	257
ESC_MA5278AA	21	156
ESC_MA5277AA	21	156
ESC_MA5276AA	21	156
ESC_MA5275AA	21	156
ESC_MA5274AA	971	198
ESC_MA5273AA	2836	221
ESC_MA5272AA	44	512
ESC_MA5271AA	131	653
ESC_MA5270AA	131	653
ESC_MA5269AA	131	653
ESC_MA5268AA	602	257
ESC_MA5267AA	131	653
ESC_MA5266AA	131	653
ESC_MA5265AA	648	359
ESC_MA5264AA	131	653
ESC_MA5263AA	10	512
ESC_MA5260AA	342	286
ESC_MA5259AA	21	156
ESC_MA5258AA	679	257
ESC_MA5257AA	11	936
ESC_MA5251AA	17	191
ESC_MA5250AA	17	191
ESC_MA5249AA	17	191
ESC_MA5248AA	16	156
ESC_MA5247AA	301	307
ESC_MA5246AA	16	156
ESC_MA5245AA	301	307
ESC_MA5244AA	301	307
ESC_MA5242AA	301	307
ESC_MA5241AA	34	504
ESC_MA5240AA	17	191
ESC_MA5239AA	1490	935
ESC_MA5238AA	17	191
ESC_MA5237AA	17	191

ESC_MA5236AA	10	512
ESC_MA5235AA	34	504
ESC_MA5234AA	187	257
ESC_MA5233AA	392	234
ESC_MA5232AA	7187	76
ESC_MA5231AA	58	198
ESC_MA5230AA	1079	235
ESC_MA5229AA	34	504
ESC_MA5228AA	224	198
ESC_MA5227AA	540	286
ESC_MA5226AA	58	198
ESC_MA5225AA	10	512
ESC_MA5224AA	141	644
ESC_MA5223AA	9305	161
ESC_MA5222AA	10	512
ESC_MA5221AA	8271	928
ESC_MA5220AA	297	234
ESC_MA5219AA	58	198
ESC_MA5218AA	1049	175
ESC_MA5217AA	5340	826
ESC_MA5216AA	2175	212
ESC_MA5215AA	5881	555
ESC_MA5214AA	9307	156
ESC_MA5213AA	117	592
ESC_MA5212AA	9308	256
ESC_MA5211AA	9306	160
ESC_MA5210AA	409	504
ESC_MA5209AA	58	198
ESC_MA5208AA	5881	555
ESC_MA5207AA	3580	257
ESC_MA5206AA	2601	785
ESC_MA5205AA	607	355
ESC_MA5204AA	5657	672
ESC_MA5203AA	2522	198
ESC_MA5202AA	58	198
ESC_MA5201AA	453	935
ESC_MA5200AA	10	512
ESC_MA5199AA	58	198
ESC_MA5198AA	2502	235
ESC_MA5197AA	847	257
ESC_MA5196AA	6559	234
ESC_MA5195AA	2144	257
ESC_MA5194AA	428	654
ESC_MA5193AA	4260	737
ESC_MA5192AA	744	512
ESC_MA5191AA	297	234
ESC_MA5190AA	2531	198
ESC_MA5189AA	540	286
ESC_MA5188AA	1434	512
ESC_MA5187AA	602	257

ESC_MA5186AA	117	592
ESC_MA5185AA	7616	257
ESC_MA5184AA	58	198
ESC_MA5183AA	602	257
ESC_MA5182AA	355	764
ESC_MA5181AA	3700	387
ESC_MA5180AA	8768	71
ESC_MA5179AA	109	257
ESC_MA5178AA	46	447
ESC_MA5177AA	355	764
ESC_MA5176AA	109	257
ESC_MA5175AA	117	592
ESC_MA5174AA	86	935
ESC_MA5173AA	75	257
ESC_MA5172AA	540	286
ESC_MA5171AA	8619	924
ESC_MA5170AA	1972	406
ESC_MA5169AA	1125	198
ESC_MA5168AA	88	160
ESC_MA5166AA	117	592
ESC_MA5165AA	428	654
ESC_MA5164AA	5876	847
ESC_MA5163AA	1079	235
ESC_MA5162AA	10	512
ESC_MA5160AA	164	175
ESC_MA5159AA	635	141
ESC_MA5158AA	9309	598
ESC_MA5157AA	155	175
ESC_MA5156AA	706	422
ESC_MA5155AA	657	592
ESC_MA5153AA	10	512
ESC_MA5152AA	56	198
ESC_MA5151AA	8588	257
ESC_MA5150AA	1665	601
ESC_MA5149AA	536	141
ESC_MA5148AA	155	175
ESC_MA5147AA	58	198
ESC_MA5146AA	5881	555
ESC_MA5144AA	9311	504
ESC_MA5143AA	971	198
ESC_MA5142AA	48	76
ESC_MA5141AA	10	512
ESC_MA5140AA	1429	198
ESC_MA5139AA	677	257
ESC_MA5138AA	58	198
ESC_MA5137AA	10	512
ESC_MA5136AA	295	160
ESC_MA5135AA	278	924
ESC_MA5134AA	93	71
ESC_MA5132AA	1463	263

ESC_MA5131AA	38	669
ESC_MA5130AA	681	393
ESC_MA5129AA	1079	235
ESC_MA5128AA	48	76
ESC_MA5127AA	131	653
ESC_MA5126AA	69	387
ESC_MA5125AA	10	512
ESC_MA5124AA	399	198
ESC_MA5123AA	2485	212
ESC_MA5122AA	10	512
ESC_MA5121AA	978	740
ESC_MA5120AA	154	257
ESC_MA5119AA	5308	261
ESC_MA5118AA	641	935
ESC_MA5117AA	117	592
ESC_MA5116AA	10	512
ESC_MA5115AA	48	76
ESC_MA5114AA	164	175
ESC_MA5113AA	117	592
ESC_MA5111AA	2967	289
ESC_MA5110AA	9310	198
ESC_MA5109AA	799	852
ESC_MA5108AA	3580	257
ESC_MA5107AA	3580	257
ESC_MA5106AA	428	654
ESC_MA5105AA	8428	257
ESC_MA5104AA	428	654
ESC_MA5103AA	1081	198
ESC_MA5101AA	11	936
ESC_MA5100AA	301	307
ESC_MA5098AA	21	156
ESC_MA5097AA	21	156
ESC_MA5094AA	16	156
ESC_MA5093AA	152	257
ESC_MA5092AA	7207	960
ESC_MA5091AA	1260	512
ESC_MA5090AA	10	512
ESC_MA5089AA	9296	198
ESC_MA5088AA	206	286
ESC_MA5087AA	10	512
ESC_MA5086AA	2520	878
ESC_MA5085AA	278	924
ESC_MA5084AA	1307	257
ESC_MA5083AA	58	198
ESC_MA5082AA	9304	65
ESC_MA5081AA	21	156
ESC_MA5080AA	718	257
ESC_MA5079AA	1582	198
ESC_MA5078AA	345	175
ESC_MA5077AA	9303	160

ESC_MA5076AA	10	512
ESC_MA5075AA	88	160
ESC_MA5074AA	744	512
ESC_MA5073AA	1632	824
ESC_MA5072AA	540	286
ESC_MA5071AA	9302	257
ESC_MA5070AA	752	512
ESC_MA5069AA	410	156
ESC_MA5068AA	457	595
ESC_MA5067AA	4358	71
ESC_MA5066AA	38	669
ESC_MA5065AA	898	108
ESC_MA5064AA	1665	601
ESC_MA5063AA	88	160
ESC_MA5062AA	88	160
ESC_MA5061AA	4025	234
ESC_MA5060AA	58	198
ESC_MA5059AA	7538	445
ESC_MA5058AA	6707	445
ESC_MA5057AA	8237	504
ESC_MA5056AA	58	198
ESC_MA5055AA	3580	257
ESC_MA5054AA	2522	198
ESC_MA5053AA	10	512
ESC_MA5052AA	2179	924
ESC_MA5051AA	457	595
ESC_MA5050AA	457	595
ESC_MA5049AA	10	512
ESC_MA5048AA	744	512
ESC_MA5047AA	9298	654
ESC_MA5046AA	429	654
ESC_MA5045AA	152	257
ESC_MA5044AA	655	926
ESC_MA5043AA	21	156
ESC_MA5042AA	744	512
ESC_MA5041AA	744	512
ESC_MA5040AA	744	512
ESC_MA5039AA	457	595
ESC_MA5038AA	744	512
ESC_MA5037AA	429	654
ESC_MA5036AA	88	160
ESC_MA5035AA	117	592
ESC_MA5034AA	457	595
ESC_MA5033AA	429	654
ESC_MA5032AA	4512	71
ESC_MA5031AA	457	595
ESC_MA5030AA	457	595
ESC_MA5029AA	10	512
ESC_MA5027AA	457	595
ESC_MA5026AA	744	512

ESC_MA5025AA	457	595
ESC_MA5024AA	744	512
ESC_MA5023AA	106	387
ESC_MA5022AA	17	191
ESC_MA5021AA	43	512
ESC_MA5020AA	17	191
ESC_MA5019AA	16	156
ESC_MA5018AA	21	156
ESC_MA5017AA	17	191
ESC_MA5016AA	4	76
ESC_MA5015AA	17	191
ESC_MA5014AA	2008	257
ESC_MA5013AA	10	512
ESC_MA5012AA	38	669
ESC_MA5011AA	278	924
ESC_MA5010AA	106	387
ESC_MA5009AA	10	512
ESC_MA5008AA	17	191
ESC_MA5007AA	34	504
ESC_MA5006AA	2332	601
ESC_MA5005AA	17	191
ESC_MA5004AA	34	504
ESC_MA5003AA	678	257
ESC_MA5002AA	21	156
ESC_MA5001AA	4	76
ESC_MA5000AA	34	504
ESC_MA4999AA	200	198
ESC_MA4998AA	10	512
ESC_MA4997AA	11	936
ESC_MA4996AA	17	191
ESC_MA4995AA	1049	175
ESC_MA4994AA	295	160
ESC_MA4993AA	58	198
ESC_MA4992AA	11	936
ESC_MA4991AA	69	387
ESC_MA4990AA	4213	251
ESC_MA4989AA	457	595
ESC_MA4988AA	457	595
ESC_MA4987AA	744	512
ESC_MA4986AA	457	595
ESC_MA4985AA	17	191
ESC_MA4984AA	17	191
ESC_MA4983AA	17	191
ESC_MA4982AA	17	191
ESC_MA4981AA	17	191
ESC_MA4980AA	17	191
ESC_MA4979AA	17	191
ESC_MA4978AA	17	191
ESC_MA4977AA	17	191
ESC_MA4976AA	17	191

ESC_MA4975AA	9301	512
ESC_MA4974AA	140	738
ESC_MA4973AA	457	595
ESC_MA4972AA	152	257
ESC_MA4971AA	152	257
ESC_MA4970AA	245	261
ESC_MA4969AA	152	257
ESC_MA4968AA	245	261
ESC_MA4967AA	152	257
ESC_MA4965AA	21	156
ESC_MA4964AA	152	257
ESC_MA4963AA	1193	761
ESC_MA4962AA	21	156
ESC_MA4961AA	300	286
ESC_MA4960AA	2836	221
ESC_MA4959AA	152	257
ESC_MA4958AA	152	257
ESC_MA4957AA	152	257
ESC_MA4956AA	17	191
ESC_MA4955AA	17	191
ESC_MA4954AA	17	191
ESC_MA4953AA	17	191
ESC_MA4952AA	17	191
ESC_MA4951AA	17	191
ESC_MA4950AA	17	191
ESC_MA4949AA	17	191
ESC_MA4948AA	17	191
ESC_MA4947AA	17	191
ESC_MA4946AA	17	191
ESC_MA4945AA	17	191
ESC_MA4944AA	17	191
ESC_MA4943AA	17	191
ESC_MA4942AA	17	191
ESC_MA4941AA	11	936
ESC_MA4940AA	442	257
ESC_MA4939AA	16	156
ESC_MA4938AA	25	234
ESC_MA4937AA	245	261
ESC_MA4936AA	245	261
ESC_MA4935AA	245	261
ESC_MA4934AA	21	156
ESC_MA4933AA	16	156
ESC_MA4917AA	152	257
ESC_MA4916AA	245	261
ESC_MA4915AA	21	156
ESC_MA4914AA	655	926
ESC_MA4913AA	17	191
ESC_MA4912AA	17	191
ESC_MA4911AA	21	156
ESC_MA4910AA	33	198

ESC_MA4909AA	21	156
ESC_MA4908AA	21	156
ESC_MA4907AA	17	191
ESC_MA4906AA	17	191
ESC_MA4905AA	17	191
ESC_MA4904AA	33	198
ESC_MA4903AA	17	191
ESC_MA4902AA	17	191
ESC_MA4901AA	1967	191
ESC_MA4900AA	21	156
ESC_MA4899AA	11	936
ESC_MA4898AA	21	156
ESC_MA4897AA	2836	221
ESC_MA4896AA	16	156
ESC_MA4895AA	17	191
ESC_MA4894AA	655	926
ESC_MA4893AA	17	191
ESC_MA4892AA	131	653
ESC_MA4891AA	115	669
ESC_MA4890AA	315	669
ESC_MA4889AA	10	512
ESC_MA4888AA	131	653
ESC_MA4887AA	131	653
ESC_MA4886AA	93	71
ESC_MA4885AA	405	923
ESC_MA4884AA	131	653
ESC_MA4883AA	131	653
ESC_MA4882AA	131	653
ESC_MA4881AA	131	653
ESC_MA4880AA	38	669
ESC_MA4879AA	131	653
ESC_MA4878AA	131	653
ESC_MA4877AA	131	653
ESC_MA4876AA	10	512
ESC_MA4875AA	131	653
ESC_MA4874AA	131	653
ESC_MA4873AA	131	653
ESC_MA4872AA	131	653
ESC_MA4871AA	131	653
ESC_MA4870AA	131	653
ESC_MA4869AA	131	653
ESC_MA4868AA	131	653
ESC_MA4867AA	131	653
ESC_MA4866AA	2003	669
ESC_MA4865AA	847	257
ESC_MA4864AA	410	156
ESC_MA4863AA	131	653
ESC_MA4860AA	131	653
ESC_MA4859AA	778	638
ESC_MA4858AA	131	653

ESC_MA4857AA	131	653
ESC_MA4856AA	457	595
ESC_MA4855AA	131	653
ESC_MA4854AA	131	653
ESC_MA4853AA	131	653
ESC_MA4852AA	131	653
ESC_MA4851AA	131	653
ESC_MA4850AA	131	653
ESC_MA4849AA	131	653
ESC_MA4848AA	117	592
ESC_MA4847AA	131	653
ESC_MA4846AA	131	653
ESC_MA4845AA	90	156
ESC_MA4844AA	131	653
ESC_MA4843AA	131	653
ESC_MA4842AA	131	653
ESC_MA4841AA	131	653
ESC_MA4840AA	131	653
ESC_MA4839AA	131	653
ESC_MA4838AA	617	511
ESC_MA4837AA	131	653
ESC_MA4836AA	2851	156
ESC_MA4835AA	131	653
ESC_MA4833AA	131	653
ESC_MA4832AA	617	511
ESC_MA4830AA	131	653
ESC_MA4829AA	167	512
ESC_MA4828AA	405	923
ESC_MA4826AA	131	653
ESC_MA4825AA	405	923
ESC_MA4824AA	131	653
ESC_MA4823AA	12	626
ESC_MA4821AA	648	359
ESC_MA4820AA	131	653
ESC_MA4819AA	131	653
ESC_MA4818AA	38	669
ESC_MA4817AA	2142	669
ESC_MA4816AA	131	653
ESC_MA4815AA	405	923
ESC_MA4814AA	648	359
ESC_MA4813AA	131	653
ESC_MA4812AA	131	653
ESC_MA4811AA	131	653
ESC_MA4810AA	131	653
ESC_MA4809AA	405	923
ESC_MA4808AA	131	653
ESC_MA4807AA	131	653
ESC_MA4806AA	38	669
ESC_MA4805AA	131	653
ESC_MA4804AA	131	653

ESC_MA4803AA	9299	900
ESC_MA4802AA	167	512
ESC_MA4801AA	410	156
ESC_MA4800AA	405	923
ESC_MA4799AA	131	653
ESC_MA4798AA	131	653
ESC_MA4795AA	131	653
ESC_MA4794AA	131	653
ESC_MA4793AA	648	359
ESC_MA4792AA	131	653
ESC_MA4791AA	131	653
ESC_MA4790AA	131	653
ESC_MA4789AA	410	156
ESC_MA4787AA	131	653
ESC_MA4786AA	617	511
ESC_MA4785AA	2142	669
ESC_MA4784AA	405	923
ESC_MA4783AA	648	359
ESC_MA4782AA	410	156
ESC_MA4781AA	131	653
ESC_MA4780AA	648	359
ESC_MA4779AA	131	653
ESC_MA4778AA	131	653
ESC_MA4777AA	131	653
ESC_MA4776AA	131	653
ESC_MA4775AA	62	574
ESC_MA4774AA	131	653
ESC_MA4773AA	131	653
ESC_MA4772AA	131	653
ESC_MA4771AA	405	923
ESC_MA4770AA	131	653
ESC_MA4769AA	131	653
ESC_MA4768AA	617	511
ESC_MA4767AA	131	653
ESC_MA4765AA	1177	669
ESC_MA4761AA	648	359
ESC_MA4760AA	131	653
ESC_MA4759AA	1284	604
ESC_MA4758AA	131	653
ESC_MA4757AA	131	653
ESC_MA4754AA	2142	669
ESC_MA4753AA	38	669
ESC_MA4752AA	131	653
ESC_MA4751AA	131	653
ESC_MA4750AA	648	359
ESC_MA4747AA	131	653
ESC_MA4743AA	648	359
ESC_MA4742AA	131	653
ESC_MA4741AA	131	653
ESC_MA4740AA	131	653

ESC_MA4739AA	131	653
ESC_MA4738AA	405	923
ESC_MA4737AA	131	653
ESC_MA4736AA	38	669
ESC_MA4735AA	448	257
ESC_MA4734AA	167	512
ESC_MA4733AA	500	71
ESC_MA4730AA	1284	604
ESC_MA4729AA	410	156
ESC_MA4728AA	167	512
ESC_MA4727AA	648	359
ESC_MA4726AA	648	359
ESC_MA4724AA	602	257
ESC_MA4723AA	405	923
ESC_MA4722AA	131	653
ESC_MA4719AA	245	261
ESC_MA4718AA	152	257
ESC_MA4717AA	152	257
ESC_MA4716AA	152	257
ESC_MA4715AA	152	257
ESC_MA4714AA	1503	257
ESC_MA4713AA	152	257
ESC_MA4712AA	245	261
ESC_MA4711AA	245	261
ESC_MA4710AA	245	261
ESC_MA4709AA	11	936
ESC_MA4708AA	11	936
ESC_MA4707AA	21	156
ESC_MA4706AA	295	160
ESC_MA4705AA	442	257
ESC_MA4704AA	21	156
ESC_MA4703AA	16	156
ESC_MA4702AA	504	738
ESC_MA4701AA	21	156
ESC_MA4700AA	753	842
ESC_MA4699AA	3021	447
ESC_MA4698AA	17	191
ESC_MA4697AA	657	592
ESC_MA4696AA	5431	0
ESC_MA4695AA	429	654
ESC_MA4694AA	9298	654
ESC_MA4693AA	429	654
ESC_MA4692AA	429	654
ESC_MA4691AA	429	654
ESC_MA4690AA	393	343
ESC_MA4689AA	526	745
ESC_MA4688AA	69	387
ESC_MA4687AA	10	512
ESC_MA4686AA	1499	156
ESC_MA4685AA	141	644

ESC_MA4684AA	10	512
ESC_MA4683AA	567	740
ESC_MA4682AA	9297	560
ESC_MA4681AA	10	512
ESC_MA4680AA	73	737
ESC_MA4679AA	59	217
ESC_MA4678AA	69	387
ESC_MA4677AA	95	738
ESC_MA4676AA	131	653
ESC_MA4675AA	95	738
ESC_MA4674AA	69	387
ESC_MA4673AA	1025	261
ESC_MA4672AA	243	197
ESC_MA4671AA	152	257
ESC_MA4670AA	245	261
ESC_MA4669AA	152	257
ESC_MA4668AA	245	261
ESC_MA4667AA	245	261
ESC_MA4666AA	152	257
ESC_MA4665AA	152	257
ESC_MA4664AA	152	257
ESC_MA4663AA	245	261
ESC_MA4662AA	33	198
ESC_MA4661AA	675	234
ESC_MA4660AA	159	789
ESC_MA4659AA	25	234
ESC_MA4658AA	442	257
ESC_MA4657AA	25	234
ESC_MA4656AA	10	512
ESC_MA4655AA	10	512
ESC_MA4654AA	442	257
ESC_MA4653AA	675	234
ESC_MA4652AA	156	175
ESC_MA4651AA	9296	198
ESC_MA4650AA	9296	198
ESC_MA4649AA	9296	198
ESC_MA4648AA	9296	198
ESC_MA4647AA	73	737
ESC_MA4646AA	11	936
ESC_MA4645AA	16	156
ESC_MA4644AA	21	156
ESC_MA4643AA	442	257
ESC_MA4642AA	21	156
ESC_MA4641AA	17	191
ESC_MA4640AA	16	156
ESC_MA4639AA	152	257
ESC_MA4638AA	152	257
ESC_MA4637AA	152	257
ESC_MA4636AA	152	257
ESC_MA4635AA	152	257

ESC_MA4634AA	152	257
ESC_MA4633AA	152	257
ESC_MA4632AA	21	156
ESC_MA4631AA	21	156
ESC_MA4630AA	1727	257
ESC_MA4629AA	17	191
ESC_MA4628AA	17	191
ESC_MA4627AA	655	926
ESC_MA4626AA	11	936
ESC_MA4625AA	11	936
ESC_MA4624AA	11	936
ESC_MA4623AA	11	936
ESC_MA4622AA	95	738
ESC_MA4621AA	58	198
ESC_MA4620AA	421	744
ESC_MA4619AA	95	738
ESC_MA4618AA	69	387
ESC_MA4617AA	141	644
ESC_MA4616AA	301	307
ESC_MA4615AA	404	761
ESC_MA4614AA	12	626
ESC_MA4613AA	1385	653
ESC_MA4612AA	10	512
ESC_MA4611AA	69	387
ESC_MA4610AA	131	653
ESC_MA4609AA	80	731
ESC_MA4608AA	95	738
ESC_MA4607AA	59	217
ESC_MA4606AA	524	270
ESC_MA4605AA	95	738
ESC_MA4604AA	23	156
ESC_MA4601AA	38	669
ESC_MA4600AA	17	191
ESC_MA4599AA	1511	198
ESC_MA4598AA	17	191
ESC_MA4597AA	17	191
ESC_MA4596AA	173	254
ESC_MA4595AA	1415	504
ESC_MA4594AA	10	512
ESC_MA4593AA	8543	92
ESC_MA4592AA	328	924
ESC_MA4591AA	152	257
ESC_MA4590AA	245	261
ESC_MA4589AA	245	261
ESC_MA4588AA	152	257
ESC_MA4587AA	152	257
ESC_MA4586AA	152	257
ESC_MA4585AA	152	257
ESC_MA4584AA	152	257
ESC_MA4583AA	11	936

ESC_MA4582AA	442	257
ESC_MA4581AA	306	286
ESC_MA4580AA	657	592
ESC_MA4579AA	17	191
ESC_MA4578AA	17	191
ESC_MA4577AA	21	156
ESC_MA4576AA	21	156
ESC_MA4575AA	17	191
ESC_MA4574AA	119	286
ESC_MA4573AA	17	191
ESC_MA4572AA	16	156
ESC_MA4571AA	21	156
ESC_MA4570AA	16	156
ESC_MA4569AA	17	191
ESC_MA4568AA	17	191
ESC_MA4567AA	17	191
ESC_MA4566AA	11	936
ESC_MA4565AA	21	156
ESC_MA4564AA	349	670
ESC_MA4563AA	2325	447
ESC_MA4562AA	155	175
ESC_MA4561AA	2325	447
ESC_MA4560AA	540	286
ESC_MA4558AA	4980	445
ESC_MA4557AA	9293	257
ESC_MA4556AA	17	191
ESC_MA4555AA	2325	447
ESC_MA4554AA	11	936
ESC_MA4553AA	9292	504
ESC_MA4552AA	17	191
ESC_MA4551AA	1721	311
ESC_MA4550AA	3107	512
ESC_MA4549AA	1611	198
ESC_MA4548AA	540	286
ESC_MA4547AA	155	175
ESC_MA4546AA	345	175
ESC_MA4545AA	1081	198
ESC_MA4544AA	43	512
ESC_MA4543AA	1125	198
ESC_MA4542AA	10	512
ESC_MA4541AA	1967	191
ESC_MA4540AA	9294	191
ESC_MA4539AA	540	286
ESC_MA4538AA	2325	447
ESC_MA4537AA	2325	447
ESC_MA4536AA	21	156
ESC_MA4535AA	1307	257
ESC_MA4534AA	1389	924
ESC_MA4533AA	2325	447
ESC_MA4532AA	11	936

ESC_MA4531AA	540	286
ESC_MA4530AA	540	286
ESC_MA4529AA	906	198
ESC_MA4528AA	540	286
ESC_MA4527AA	2325	447
ESC_MA4525AA	2144	257
ESC_MA4524AA	5012	24
ESC_MA4523AA	2325	447
ESC_MA4522AA	165	504
ESC_MA4521AA	2325	447
ESC_MA4520AA	48	76
ESC_MA4519AA	2325	447
ESC_MA4518AA	1147	198
ESC_MA4517AA	215	512
ESC_MA4516AA	2325	447
ESC_MA4515AA	345	175
ESC_MA4514AA	5292	738
ESC_MA4513AA	937	198
ESC_MA4512AA	2165	257
ESC_MA4511AA	155	175
ESC_MA4510AA	2614	975
ESC_MA4509AA	9286	857
ESC_MA4508AA	2772	257
ESC_MA4507AA	155	175
ESC_MA4506AA	58	198
ESC_MA4505AA	746	789
ESC_MA4504AA	196	257
ESC_MA4503AA	942	198
ESC_MA4502AA	155	175
ESC_MA4501AA	540	286
ESC_MA4500AA	540	286
ESC_MA4499AA	86	935
ESC_MA4498AA	155	175
ESC_MA4497AA	155	175
ESC_MA4496AA	540	286
ESC_MA4495AA	540	286
ESC_MA4494AA	2067	257
ESC_MA4493AA	602	257
ESC_MA4492AA	155	175
ESC_MA4491AA	540	286
ESC_MA4490AA	155	175
ESC_MA4489AA	48	76
ESC_MA4488AA	2067	257
ESC_MA4487AA	155	175
ESC_MA4486AA	5826	198
ESC_MA4485AA	1423	198
ESC_MA4484AA	155	175
ESC_MA4483AA	155	175
ESC_MA4482AA	443	257
ESC_MA4481AA	2614	975

ESC_MA4480AA	212	198
ESC_MA4479AA	155	175
ESC_MA4478AA	155	175
ESC_MA4477AA	212	198
ESC_MA4476AA	155	175
ESC_MA4475AA	6186	868
ESC_MA4474AA	2614	975
ESC_MA4473AA	155	175
ESC_MA4472AA	155	175
ESC_MA4471AA	2772	257
ESC_MA4470AA	337	257
ESC_MA4469AA	540	286
ESC_MA4468AA	155	175
ESC_MA4467AA	295	160
ESC_MA4466AA	540	286
ESC_MA4465AA	4392	234
ESC_MA4464AA	9285	1008
ESC_MA4463AA	540	286
ESC_MA4462AA	2067	257
ESC_MA4461AA	155	175
ESC_MA4460AA	155	175
ESC_MA4459AA	155	175
ESC_MA4458AA	2461	76
ESC_MA4457AA	540	286
ESC_MA4456AA	542	824
ESC_MA4454AA	2067	257
ESC_MA4453AA	155	175
ESC_MA4452AA	155	175
ESC_MA4451AA	540	286
ESC_MA4450AA	9284	847
ESC_MA4449AA	9284	847
ESC_MA4448AA	6025	233
ESC_MA4447AA	131	653
ESC_MA4446AA	131	653
ESC_MA4444AA	131	653
ESC_MA4443AA	131	653
ESC_MA4442AA	405	923
ESC_MA4441AA	602	257
ESC_MA4440AA	245	261
ESC_MA4439AA	152	257
ESC_MA4438AA	152	257
ESC_MA4433AA	17	191
ESC_MA4432AA	17	191
ESC_MA4431AA	17	191
ESC_MA4430AA	17	191
ESC_MA4429AA	675	234
ESC_MA4428AA	17	191
ESC_MA4427AA	17	191
ESC_MA4426AA	152	257
ESC_MA4425AA	723	156

ESC_MA4422AA	9283	198
ESC_MA4421AA	485	587
ESC_MA4420AA	452	743
ESC_MA4419AA	485	587
ESC_MA4418AA	2705	504
ESC_MA4417AA	603	198
ESC_MA4416AA	5148	963
ESC_MA4415AA	43	512
ESC_MA4414AA	452	743
ESC_MA4413AA	2914	670
ESC_MA4412AA	1727	257
ESC_MA4411AA	469	257
ESC_MA4410AA	410	156
ESC_MA4409AA	394	400
ESC_MA4408AA	9277	743
ESC_MA4407AA	316	257
ESC_MA4406AA	10	512
ESC_MA4405AA	394	400
ESC_MA4404AA	59	217
ESC_MA4403AA	316	257
ESC_MA4402AA	2175	212
ESC_MA4401AA	155	175
ESC_MA4400AA	9274	504
ESC_MA4399AA	58	198
ESC_MA4398AA	9277	743
ESC_MA4397AA	469	257
ESC_MA4396AA	181	311
ESC_MA4395AA	181	311
ESC_MA4394AA	394	400
ESC_MA4393AA	1204	961
ESC_MA4392AA	410	156
ESC_MA4391AA	9274	504
ESC_MA4390AA	59	217
ESC_MA4388AA	101	696
ESC_MA4387AA	9274	504
ESC_MA4386AA	747	640
ESC_MA4384AA	9281	156
ESC_MA4381AA	410	156
ESC_MA4380AA	5148	963
ESC_MA4379AA	603	198
ESC_MA4378AA	485	587
ESC_MA4376AA	5148	963
ESC_MA4375AA	43	512
ESC_MA4374AA	29	156
ESC_MA4373AA	452	743
ESC_MA4372AA	43	512
ESC_MA4371AA	10	512
ESC_MA4370AA	2705	504
ESC_MA4369AA	485	587
ESC_MA4368AA	452	743

ESC_MA4367AA	603	198
ESC_MA4366AA	9279	311
ESC_MA4365AA	394	400
ESC_MA4364AA	38	669
ESC_MA4363AA	398	445
ESC_MA4362AA	58	198
ESC_MA4361AA	58	198
ESC_MA4359AA	59	217
ESC_MA4358AA	316	257
ESC_MA4357AA	101	696
ESC_MA4356AA	2741	696
ESC_MA4355AA	9278	72
ESC_MA4354AA	9274	504
ESC_MA4353AA	181	311
ESC_MA4352AA	469	257
ESC_MA4351AA	9274	504
ESC_MA4350AA	181	311
ESC_MA4349AA	10	512
ESC_MA4348AA	9277	743
ESC_MA4347AA	540	286
ESC_MA4346AA	226	512
ESC_MA4345AA	515	1004
ESC_MA4344AA	337	257
ESC_MA4343AA	316	257
ESC_MA4342AA	223	198
ESC_MA4341AA	38	669
ESC_MA4340AA	59	217
ESC_MA4339AA	9274	504
ESC_MA4338AA	747	640
ESC_MA4337AA	641	935
ESC_MA4336AA	469	257
ESC_MA4335AA	101	696
ESC_MA4334AA	38	669
ESC_MA4333AA	5148	963
ESC_MA4332AA	2705	504
ESC_MA4331AA	5148	963
ESC_MA4330AA	410	156
ESC_MA4329AA	452	743
ESC_MA4328AA	43	512
ESC_MA4327AA	152	257
ESC_MA4326AA	245	261
ESC_MA4325AA	152	257
ESC_MA4324AA	16	156
ESC_MA4323AA	25	234
ESC_MA4322AA	33	198
ESC_MA4321AA	738	598
ESC_MA4319AA	738	598
ESC_MA4318AA	10	512
ESC_MA4317AA	342	286
ESC_MA4316AA	6683	924

ESC_MA4315AA	80	731
ESC_MA4314AA	34	504
ESC_MA4313AA	38	669
ESC_MA4312AA	9289	452
ESC_MA4311AA	9288	684
ESC_MA4310AA	80	731
ESC_MA4309AA	141	644
ESC_MA4308AA	6096	257
ESC_MA4307AA	5702	257
ESC_MA4306AA	5937	257
ESC_MA4304AA	6096	257
ESC_MA4303AA	9290	602
ESC_MA4302AA	1081	198
ESC_MA4288AA	9295	221
ESC_MA4287AA	101	696
ESC_MA4286AA	12	626
ESC_MA4285AA	5295	355
ESC_MA4284AA	533	198
ESC_MA4283AA	5937	257
ESC_MA4282AA	155	175
ESC_MA4281AA	3232	198
ESC_MA4280AA	80	731
ESC_MA4278AA	5840	234
ESC_MA4277AA	5840	234
ESC_MA4276AA	155	175
ESC_MA4275AA	93	71
ESC_MA4274AA	661	92
ESC_MA4273AA	155	175
ESC_MA4272AA	75	257
ESC_MA4271AA	9287	732
ESC_MA4270AA	17	191
ESC_MA4269AA	17	191
ESC_MA4268AA	17	191
ESC_MA4267AA	17	191
ESC_MA4266AA	9273	670
ESC_MA4265AA	245	261
ESC_MA4264AA	21	156
ESC_MA4263AA	29	156
ESC_MA4262AA	655	926
ESC_MA4261AA	11	936
ESC_MA4260AA	173	254
ESC_MA4259AA	17	191
ESC_MA4258AA	131	653
ESC_MA4257AA	9282	597
ESC_MA4256AA	17	191
ESC_MA4255AA	9282	597
ESC_MA4254AA	17	191
ESC_MA4253AA	152	257
ESC_MA4252AA	152	257
ESC_MA4251AA	152	257

ESC_MA4250AA	7375	197
ESC_MA4248AA	243	197
ESC_MA4247AA	245	261
ESC_MA4246AA	152	257
ESC_MA4245AA	152	257
ESC_MA4244AA	137	270
ESC_MA4243AA	5390	83
ESC_MA4242AA	152	257
ESC_MA4241AA	152	257
ESC_MA4240AA	245	261
ESC_MA4239AA	152	257
ESC_MA4238AA	245	261
ESC_MA4237AA	1503	257
ESC_MA4235AA	245	261
ESC_MA4231AA	361	512
ESC_MA4230AA	155	175
ESC_MA4226AA	675	234
ESC_MA4220AA	131	653
ESC_MA4219AA	131	653
ESC_MA4218AA	73	737
ESC_MA4217AA	2020	387
ESC_MA4216AA	38	669
ESC_MA4215AA	58	198
ESC_MA4214AA	131	653
ESC_MA4213AA	1982	653
ESC_MA4212AA	38	669
ESC_MA4211AA	69	387
ESC_MA4210AA	131	653
ESC_MA4209AA	131	653
ESC_MA4208AA	131	653
ESC_MA4207AA	540	286
ESC_MA4206AA	38	669
ESC_MA4204AA	117	592
ESC_MA4203AA	354	15
ESC_MA4202AA	131	653
ESC_MA4200AA	372	424
ESC_MA4199AA	1262	737
ESC_MA4198AA	152	257
ESC_MA4196AA	38	669
ESC_MA4195AA	10	512
ESC_MA4194AA	10	512
ESC_MA4193AA	10	512
ESC_MA4192AA	569	738
ESC_MA4191AA	10	512
ESC_MA4190AA	10	512
ESC_MA4189AA	10	512
ESC_MA4188AA	10	512
ESC_MA4187AA	10	512
ESC_MA4186AA	10	512
ESC_MA4182AA	10	512

ESC_MA4181AA	10	512
ESC_MA4176AA	10	512
ESC_MA4175AA	10	512
ESC_MA4171AA	10	512
ESC_MA4170AA	10	512
ESC_MA4169AA	95	738
ESC_MA4165AA	429	654
ESC_MA4161AA	1638	519
ESC_MA4160AA	117	592
ESC_MA4155AA	10	512
ESC_MA4154AA	10	512
ESC_MA4152AA	10	512
ESC_MA4151AA	95	738
ESC_MA4150AA	95	738
ESC_MA4143AA	117	592
ESC_MA4140AA	95	738
ESC_MA4136AA	10	512
ESC_MA4130AA	95	738
ESC_MA4120AA	95	738
ESC_MA4114AA	95	738
ESC_MA4106AA	162	924
ESC_MA4103AA	10	512
ESC_MA4097AA	95	738
ESC_MA4092AA	101	696
ESC_MA4089AA	245	261
ESC_MA4088AA	152	257
ESC_MA4087AA	245	261
ESC_MA4086AA	1503	257
ESC_MA4085AA	243	197
ESC_MA4084AA	152	257
ESC_MA4083AA	245	261
ESC_MA4082AA	145	256
ESC_MA4081AA	245	261
ESC_MA4080AA	152	257
ESC_MA4079AA	245	261
ESC_MA4078AA	245	261
ESC_MA4077AA	152	257
ESC_MA4076AA	152	257
ESC_MA4075AA	245	261
ESC_MA4074AA	245	261
ESC_MA4073AA	145	256
ESC_MA4072AA	243	197
ESC_MA4071AA	342	286
ESC_MA4070AA	59	217
ESC_MA4069AA	21	156
ESC_MA4068AA	144	740
ESC_MA4067AA	316	257
ESC_MA4066AA	33	198
ESC_MA4065AA	738	598
ESC_MA4064AA	33	198

ESC_MA4063AA	34	504
ESC_MA4062AA	442	257
ESC_MA4061AA	442	257
ESC_MA4060AA	442	257
ESC_MA4059AA	33	198
ESC_MA4058AA	388	156
ESC_MA4057AA	21	156
ESC_MA4056AA	16	156
ESC_MA4055AA	17	191
ESC_MA4054AA	17	191
ESC_MA4053AA	17	191
ESC_MA4052AA	17	191
ESC_MA4051AA	17	191
ESC_MA4050AA	17	191
ESC_MA4049AA	17	191
ESC_MA4048AA	17	191
ESC_MA4047AA	17	191
ESC_MA4046AA	17	191
ESC_MA4045AA	17	191
ESC_MA4044AA	17	191
ESC_MA4043AA	21	156
ESC_MA4042AA	17	191
ESC_MA4040AA	117	592
ESC_MA4037AA	117	592
ESC_MA4034AA	117	592
ESC_MA4027AA	117	592
ESC_MA4025AA	117	592
ESC_MA4024AA	117	592
ESC_MA4015AA	7628	632
ESC_MA4012AA	4619	122
ESC_MA4010AA	13	257
ESC_MA4009AA	10	512
ESC_MA4007AA	9268	878
ESC_MA4006AA	616	156
ESC_MA4005AA	10	512
ESC_MA4004AA	1721	311
ESC_MA4003AA	4566	257
ESC_MA4000AA	10	512
ESC_MA3999AA	4566	257
ESC_MA3998AA	10	512
ESC_MA3997AA	10	512
ESC_MA3996AA	448	257
ESC_MA3995AA	2509	257
ESC_MA3994AA	38	669
ESC_MA3993AA	349	670
ESC_MA3992AA	410	156
ESC_MA3991AA	9269	198
ESC_MA3974AA	95	738
ESC_MA3972AA	95	738
ESC_MA3971AA	10	512

ESC_MA3967AA	410	156
ESC_MA3964AA	617	511
ESC_MA3962AA	617	511
ESC_MA3959AA	410	156
ESC_MA3957AA	95	738
ESC_MA3956AA	10	512
ESC_MA3955AA	10	512
ESC_MA3952AA	167	512
ESC_MA3932AA	131	653
ESC_MA3930AA	131	653
ESC_MA3929AA	1722	599
ESC_MA3928AA	38	669
ESC_MA3927AA	58	198
ESC_MA3926AA	3075	701
ESC_MA3925AA	131	653
ESC_MA3924AA	131	653
ESC_MA3923AA	8881	362
ESC_MA3922AA	2003	669
ESC_MA3921AA	405	923
ESC_MA3920AA	131	653
ESC_MA3919AA	131	653
ESC_MA3917AA	354	15
ESC_MA3916AA	630	261
ESC_MA3915AA	630	261
ESC_MA3914AA	630	261
ESC_MA3913AA	630	261
ESC_MA3912AA	630	261
ESC_MA3911AA	630	261
ESC_MA3910AA	152	257
ESC_MA3909AA	152	257
ESC_MA3908AA	152	257
ESC_MA3907AA	152	257
ESC_MA3906AA	152	257
ESC_MA3905AA	152	257
ESC_MA3904AA	152	257
ESC_MA3903AA	152	257
ESC_MA3901AA	152	257
ESC_MA3900AA	152	257
ESC_MA3899AA	152	257
ESC_MA3898AA	152	257
ESC_MA3897AA	152	257
ESC_MA3896AA	152	257
ESC_MA3895AA	152	257
ESC_MA3894AA	152	257
ESC_MA3893AA	152	257
ESC_MA3892AA	152	257
ESC_MA3891AA	152	257
ESC_MA3890AA	152	257
ESC_MA3889AA	152	257
ESC_MA3888AA	152	257

ESC_MA3887AA	152	257
ESC_MA3886AA	152	257
ESC_MA3885AA	152	257
ESC_MA3884AA	152	257
ESC_MA3883AA	152	257
ESC_MA3882AA	152	257
ESC_MA3881AA	152	257
ESC_MA3880AA	152	257
ESC_MA3841AA	99	198
ESC_MA3840AA	410	156
ESC_MA3839AA	38	669
ESC_MA3838AA	17	191
ESC_MA3837AA	17	191
ESC_MA3836AA	9266	592
ESC_MA3835AA	647	734
ESC_MA3834AA	17	191
ESC_MA3833AA	17	191
ESC_MA3832AA	17	191
ESC_MA3831AA	675	234
ESC_MA3830AA	21	156
ESC_MA3829AA	17	191
ESC_MA3828AA	9256	160
ESC_MA3827AA	2248	257
ESC_MA3826AA	23	156
ESC_MA3825AA	11	936
ESC_MA3824AA	17	191
ESC_MA3823AA	17	191
ESC_MA3822AA	17	191
ESC_MA3821AA	17	191
ESC_MA3820AA	17	191
ESC_MA3819AA	11	936
ESC_MA3818AA	17	191
ESC_MA3817AA	17	191
ESC_MA3816AA	17	191
ESC_MA3815AA	17	191
ESC_MA3814AA	17	191
ESC_MA3813AA	17	191
ESC_MA3812AA	16	156
ESC_MA3811AA	131	653
ESC_MA3810AA	655	926
ESC_MA3809AA	21	156
ESC_MA3808AA	17	191
ESC_MA3807AA	9258	936
ESC_MA3806AA	9257	175
ESC_MA3805AA	88	160
ESC_MA3804AA	6234	257
ESC_MA3801AA	100	504
ESC_MA3799AA	100	504
ESC_MA3798AA	88	160
ESC_MA3795AA	100	504

ESC_MA3790AA	10	512
ESC_MA3788AA	90	156
ESC_MA3785AA	90	156
ESC_MA3782AA	100	504
ESC_MA3778AA	9276	512
ESC_MA3777AA	10	512
ESC_MA3776AA	9275	257
ESC_MA3773AA	100	504
ESC_MA3771AA	100	504
ESC_MA3770AA	10	512
ESC_MA3769AA	6234	257
ESC_MA3768AA	100	504
ESC_MA3767AA	100	504
ESC_MA3766AA	542	824
ESC_MA3764AA	10	512
ESC_MA3763AA	90	156
ESC_MA3762AA	100	504
ESC_MA3753AA	847	257
ESC_MA3752AA	90	156
ESC_MA3750AA	2526	257
ESC_MA3749AA	88	160
ESC_MA3748AA	90	156
ESC_MA3747AA	100	504
ESC_MA3745AA	100	504
ESC_MA3742AA	88	160
ESC_MA3741AA	100	504
ESC_MA3740AA	100	504
ESC_MA3739AA	100	504
ESC_MA3737AA	10	512
ESC_MA3736AA	410	156
ESC_MA3735AA	100	504
ESC_MA3733AA	88	160
ESC_MA3732AA	10	512
ESC_MA3731AA	100	504
ESC_MA3730AA	100	504
ESC_MA3729AA	100	504
ESC_MA3728AA	90	156
ESC_MA3727AA	847	257
ESC_MA3726AA	9261	512
ESC_MA3725AA	100	504
ESC_MA3723AA	410	156
ESC_MA3722AA	88	160
ESC_MA3721AA	156	175
ESC_MA3720AA	752	512
ESC_MA3719AA	9260	198
ESC_MA3718AA	1080	198
ESC_MA3717AA	9263	198
ESC_MA3716AA	10	512
ESC_MA3715AA	1260	512
ESC_MA3714AA	297	234

ESC_MA3713AA	1123	257
ESC_MA3712AA	9259	512
ESC_MA3711AA	10	512
ESC_MA3710AA	1123	257
ESC_MA3709AA	1080	198
ESC_MA3708AA	8618	512
ESC_MA3707AA	6186	868
ESC_MA3706AA	11	936
ESC_MA3705AA	2522	198
ESC_MA3704AA	189	504
ESC_MA3703AA	641	935
ESC_MA3702AA	101	696
ESC_MA3701AA	189	504
ESC_MA3700AA	3006	512
ESC_MA3699AA	38	669
ESC_MA3697AA	648	359
ESC_MA3696AA	131	653
ESC_MA3695AA	131	653
ESC_MA3694AA	131	653
ESC_MA3693AA	69	387
ESC_MA3692AA	10	512
ESC_MA3691AA	1193	761
ESC_MA3690AA	648	359
ESC_MA3689AA	127	738
ESC_MA3688AA	131	653
ESC_MA3687AA	69	387
ESC_MA3686AA	405	923
ESC_MA3685AA	69	387
ESC_MA3684AA	9262	737
ESC_MA3683AA	405	923
ESC_MA3682AA	73	737
ESC_MA3681AA	69	387
ESC_MA3680AA	73	737
ESC_MA3679AA	648	359
ESC_MA3678AA	131	653
ESC_MA3677AA	1193	761
ESC_MA3676AA	1193	761
ESC_MA3675AA	131	653
ESC_MA3674AA	131	653
ESC_MA3673AA	131	653
ESC_MA3672AA	73	737
ESC_MA3671AA	2073	198
ESC_MA3670AA	1193	761
ESC_MA3669AA	12	626
ESC_MA3668AA	95	738
ESC_MA3667AA	405	923
ESC_MA3666AA	131	653
ESC_MA3665AA	73	737
ESC_MA3664AA	131	653
ESC_MA3663AA	95	738

ESC_MA3662AA	131	653
ESC_MA3661AA	131	653
ESC_MA3660AA	69	387
ESC_MA3659AA	131	653
ESC_MA3658AA	73	737
ESC_MA3657AA	617	511
ESC_MA3656AA	131	653
ESC_MA3655AA	1193	761
ESC_MA3654AA	1177	669
ESC_MA3653AA	1193	761
ESC_MA3652AA	648	359
ESC_MA3651AA	38	669
ESC_MA3650AA	361	512
ESC_MA3649AA	73	737
ESC_MA3648AA	69	387
ESC_MA3647AA	648	359
ESC_MA3646AA	131	653
ESC_MA3645AA	404	761
ESC_MA3644AA	404	761
ESC_MA3643AA	12	626
ESC_MA3642AA	410	156
ESC_MA3641AA	131	653
ESC_MA3640AA	131	653
ESC_MA3639AA	131	653
ESC_MA3638AA	1193	761
ESC_MA3637AA	295	160
ESC_MA3636AA	131	653
ESC_MA3635AA	131	653
ESC_MA3634AA	62	574
ESC_MA3633AA	706	422
ESC_MA3632AA	10	512
ESC_MA3631AA	95	738
ESC_MA3630AA	131	653
ESC_MA3629AA	131	653
ESC_MA3628AA	73	737
ESC_MA3627AA	127	738
ESC_MA3626AA	5652	924
ESC_MA3625AA	9264	738
ESC_MA3624AA	90	156
ESC_MA3623AA	167	512
ESC_MA3622AA	73	737
ESC_MA3621AA	131	653
ESC_MA3620AA	131	653
ESC_MA3619AA	131	653
ESC_MA3618AA	135	653
ESC_MA3617AA	6189	175
ESC_MA3616AA	131	653
ESC_MA3615AA	393	343
ESC_MA3614AA	131	653
ESC_MA3613AA	8671	653

ESC_MA3612AA	95	738
ESC_MA3611AA	131	653
ESC_MA3610AA	393	343
ESC_MA3609AA	1702	512
ESC_MA3608AA	9265	387
ESC_MA3607AA	131	653
ESC_MA3606AA	131	653
ESC_MA3605AA	131	653
ESC_MA3604AA	73	737
ESC_MA3603AA	131	653
ESC_MA3602AA	131	653
ESC_MA3601AA	131	653
ESC_MA3600AA	131	653
ESC_MA3599AA	404	761
ESC_MA3598AA	1193	761
ESC_MA3597AA	131	653
ESC_MA3596AA	131	653
ESC_MA3595AA	405	923
ESC_MA3594AA	69	387
ESC_MA3593AA	1193	761
ESC_MA3592AA	131	653
ESC_MA3591AA	1193	761
ESC_MA3590AA	127	738
ESC_MA3589AA	88	160
ESC_MA3588AA	162	924
ESC_MA3587AA	648	359
ESC_MA3586AA	569	738
ESC_MA3585AA	131	653
ESC_MA3581AA	152	257
ESC_MA3580AA	152	257
ESC_MA3579AA	152	257
ESC_MA3578AA	152	257
ESC_MA3577AA	152	257
ESC_MA3576AA	152	257
ESC_MA3575AA	152	257
ESC_MA3574AA	152	257
ESC_MA3573AA	152	257
ESC_MA3572AA	152	257
ESC_MA3571AA	152	257
ESC_MA3570AA	152	257
ESC_MA3545AA	69	387
ESC_MA3544AA	357	654
ESC_MA3543AA	131	653
ESC_MA3542AA	131	653
ESC_MA3541AA	58	198
ESC_MA3540AA	131	653
ESC_MA3539AA	101	696
ESC_MA3538AA	167	512
ESC_MA3537AA	131	653
ESC_MA3536AA	38	669

ESC_MA3535AA	127	738
ESC_MA3534AA	131	653
ESC_MA3531AA	616	156
ESC_MA3528AA	362	234
ESC_MA3527AA	73	737
ESC_MA3526AA	131	653
ESC_MA3525AA	131	653
ESC_MA3523AA	95	738
ESC_MA3522AA	245	261
ESC_MA3521AA	245	261
ESC_MA3520AA	152	257
ESC_MA3519AA	381	156
ESC_MA3518AA	1060	512
ESC_MA3517AA	1060	512
ESC_MA3516AA	21	156
ESC_MA3515AA	21	156
ESC_MA3514AA	21	156
ESC_MA3501AA	152	257
ESC_MA3500AA	11	936
ESC_MA3499AA	33	198
ESC_MA3498AA	16	156
ESC_MA3497AA	343	286
ESC_MA3496AA	17	191
ESC_MA3495AA	343	286
ESC_MA3485AA	38	669
ESC_MA3484AA	405	923
ESC_MA3483AA	38	669
ESC_MA3482AA	405	923
ESC_MA3481AA	405	923
ESC_MA3480AA	405	923
ESC_MA3479AA	1284	604
ESC_MA3478AA	410	156
ESC_MA3477AA	410	156
ESC_MA3476AA	9239	256
ESC_MA3474AA	17	191
ESC_MA3473AA	17	191
ESC_MA3472AA	17	191
ESC_MA3471AA	152	257
ESC_MA3470AA	245	261
ESC_MA3469AA	630	261
ESC_MA3468AA	1503	257
ESC_MA3467AA	1759	102
ESC_MA3466AA	152	257
ESC_MA3465AA	152	257
ESC_MA3464AA	145	256
ESC_MA3463AA	10	512
ESC_MA3462AA	9242	463
ESC_MA3461AA	6	445
ESC_MA3460AA	11	936
ESC_MA3446AA	9234	756

ESC_MA3444AA	11	936
ESC_MA3443AA	93	71
ESC_MA3441AA	93	71
ESC_MA3440AA	93	71
ESC_MA3439AA	4942	36
ESC_MA3438AA	21	156
ESC_MA3437AA	442	257
ESC_MA3436AA	11	936
ESC_MA3434AA	148	191
ESC_MA3433AA	245	261
ESC_MA3432AA	1025	261
ESC_MA3431AA	152	257
ESC_MA3430AA	141	644
ESC_MA3429AA	624	359
ESC_MA3428AA	10	512
ESC_MA3427AA	95	738
ESC_MA3426AA	127	738
ESC_MA3425AA	73	737
ESC_MA3424AA	10	512
ESC_MA3423AA	131	653
ESC_MA3422AA	73	737
ESC_MA3421AA	69	387
ESC_MA3420AA	14	761
ESC_MA3418AA	69	387
ESC_MA3417AA	95	738
ESC_MA3416AA	14	761
ESC_MA3415AA	410	156
ESC_MA3414AA	4705	935
ESC_MA3413AA	1431	257
ESC_MA3412AA	131	653
ESC_MA3411AA	161	445
ESC_MA3410AA	17	191
ESC_MA3409AA	126	740
ESC_MA3408AA	1804	935
ESC_MA3407AA	6880	726
ESC_MA3406AA	504	738
ESC_MA3405AA	11	936
ESC_MA3404AA	738	598
ESC_MA3403AA	9232	400
ESC_MA3402AA	9230	118
ESC_MA3401AA	9227	135
ESC_MA3400AA	7532	118
ESC_MA3399AA	9228	124
ESC_MA3398AA	9229	116
ESC_MA3397AA	152	257
ESC_MA3396AA	152	257
ESC_MA3395AA	152	257
ESC_MA3394AA	152	257
ESC_MA3393AA	152	257
ESC_MA3392AA	152	257

ESC_MA3391AA	152	257
ESC_MA3390AA	152	257
ESC_MA3389AA	152	257
ESC_MA3388AA	442	257
ESC_MA3387AA	17	191
ESC_MA3386AA	21	156
ESC_MA3385AA	17	191
ESC_MA3384AA	11	936
ESC_MA3383AA	21	156
ESC_MA3382AA	11	936
ESC_MA3381AA	388	156
ESC_MA3380AA	21	156
ESC_MA3379AA	655	926
ESC_MA3378AA	21	156
ESC_MA3377AA	16	156
ESC_MA3376AA	21	156
ESC_MA3375AA	17	191
ESC_MA3374AA	21	156
ESC_MA3373AA	21	156
ESC_MA3372AA	32	270
ESC_MA3371AA	11	936
ESC_MA3370AA	59	217
ESC_MA3369AA	354	15
ESC_MA3368AA	131	653
ESC_MA3367AA	73	737
ESC_MA3366AA	963	669
ESC_MA3365AA	38	669
ESC_MA3364AA	429	654
ESC_MA3363AA	361	512
ESC_MA3361AA	95	738
ESC_MA3360AA	1193	761
ESC_MA3359AA	404	761
ESC_MA3358AA	131	653
ESC_MA3355AA	393	343
ESC_MA3353AA	69	387
ESC_MA3351AA	73	737
ESC_MA3350AA	38	669
ESC_MA3349AA	69	387
ESC_MA3348AA	421	744
ESC_MA3347AA	2553	738
ESC_MA3345AA	38	669
ESC_MA3344AA	73	737
ESC_MA3343AA	88	160
ESC_MA3342AA	101	696
ESC_MA3341AA	410	156
ESC_MA3340AA	95	738
ESC_MA3339AA	9241	738
ESC_MA3338AA	393	343
ESC_MA3337AA	131	653
ESC_MA3336AA	555	653

ESC_MA3335AA	69	387
ESC_MA3334AA	2548	387
ESC_MA3333AA	1193	761
ESC_MA3332AA	69	387
ESC_MA3331AA	69	387
ESC_MA3329AA	73	737
ESC_MA3328AA	69	387
ESC_MA3327AA	14	761
ESC_MA3326AA	131	653
ESC_MA3325AA	410	156
ESC_MA3324AA	6889	76
ESC_MA3322AA	410	156
ESC_MA3321AA	38	669
ESC_MA3320AA	73	737
ESC_MA3319AA	453	935
ESC_MA3318AA	69	387
ESC_MA3317AA	394	400
ESC_MA3316AA	404	761
ESC_MA3315AA	101	696
ESC_MA3314AA	648	359
ESC_MA3313AA	6091	738
ESC_MA3312AA	101	696
ESC_MA3311AA	131	653
ESC_MA3310AA	12	626
ESC_MA3309AA	101	696
ESC_MA3308AA	12	626
ESC_MA3307AA	648	359
ESC_MA3306AA	131	653
ESC_MA3305AA	297	234
ESC_MA3304AA	73	737
ESC_MA3303AA	538	629
ESC_MA3302AA	224	198
ESC_MA3301AA	73	737
ESC_MA3300AA	117	592
ESC_MA3299AA	131	653
ESC_MA3298AA	998	644
ESC_MA3297AA	1278	797
ESC_MA3296AA	2581	653
ESC_MA3295AA	539	257
ESC_MA3293AA	648	359
ESC_MA3291AA	101	696
ESC_MA3290AA	354	15
ESC_MA3289AA	1193	761
ESC_MA3288AA	4456	654
ESC_MA3287AA	69	387
ESC_MA3286AA	69	387
ESC_MA3285AA	38	669
ESC_MA3284AA	131	653
ESC_MA3283AA	405	923
ESC_MA3282AA	617	511

ESC_MA3279AA	410	156
ESC_MA3278AA	405	923
ESC_MA3277AA	95	738
ESC_MA3275AA	409	504
ESC_MA3274AA	2003	669
ESC_MA3273AA	69	387
ESC_MA3272AA	224	198
ESC_MA3271AA	648	359
ESC_MA3270AA	73	737
ESC_MA3269AA	12	626
ESC_MA3268AA	73	737
ESC_MA3267AA	10	512
ESC_MA3266AA	538	629
ESC_MA3265AA	69	387
ESC_MA3264AA	73	737
ESC_MA3263AA	88	160
ESC_MA3262AA	131	653
ESC_MA3261AA	393	343
ESC_MA3259AA	10	512
ESC_MA3257AA	410	156
ESC_MA3256AA	641	935
ESC_MA3255AA	59	217
ESC_MA3254AA	131	653
ESC_MA3253AA	1193	761
ESC_MA3252AA	362	234
ESC_MA3251AA	744	512
ESC_MA3250AA	38	669
ESC_MA3248AA	390	738
ESC_MA3247AA	69	387
ESC_MA3246AA	404	761
ESC_MA3245AA	131	653
ESC_MA3244AA	906	198
ESC_MA3243AA	410	156
ESC_MA3240AA	69	387
ESC_MA3239AA	38	669
ESC_MA3237AA	95	738
ESC_MA3236AA	2076	400
ESC_MA3235AA	131	653
ESC_MA3234AA	73	737
ESC_MA3233AA	69	387
ESC_MA3232AA	95	738
ESC_MA3231AA	10	512
ESC_MA3230AA	95	738
ESC_MA3229AA	10	512
ESC_MA3228AA	69	387
ESC_MA3227AA	998	644
ESC_MA3226AA	73	737
ESC_MA3225AA	73	737
ESC_MA3224AA	7676	512
ESC_MA3223AA	420	741

ESC_MA3222AA	1193	761
ESC_MA3221AA	131	653
ESC_MA3220AA	1193	761
ESC_MA3219AA	131	653
ESC_MA3218AA	69	387
ESC_MA3217AA	95	738
ESC_MA3216AA	141	644
ESC_MA3215AA	73	737
ESC_MA3214AA	73	737
ESC_MA3213AA	12	626
ESC_MA3212AA	393	343
ESC_MA3211AA	617	511
ESC_MA3210AA	428	654
ESC_MA3209AA	10	512
ESC_MA3208AA	420	741
ESC_MA3207AA	130	387
ESC_MA3206AA	404	761
ESC_MA3205AA	69	387
ESC_MA3204AA	69	387
ESC_MA3203AA	555	653
ESC_MA3202AA	130	387
ESC_MA3201AA	1177	669
ESC_MA3200AA	550	756
ESC_MA3199AA	101	696
ESC_MA3198AA	69	387
ESC_MA3197AA	550	756
ESC_MA3196AA	73	737
ESC_MA3194AA	95	738
ESC_MA3193AA	73	737
ESC_MA3192AA	73	737
ESC_MA3191AA	69	387
ESC_MA3190AA	38	669
ESC_MA3189AA	73	737
ESC_MA3187AA	69	387
ESC_MA3186AA	69	387
ESC_MA3185AA	69	387
ESC_MA3184AA	73	737
ESC_MA3183AA	354	15
ESC_MA3182AA	393	343
ESC_MA3181AA	95	738
ESC_MA3180AA	224	198
ESC_MA3179AA	95	738
ESC_MA3178AA	405	923
ESC_MA3177AA	95	738
ESC_MA3176AA	73	737
ESC_MA3175AA	38	669
ESC_MA3174AA	420	741
ESC_MA3173AA	141	644
ESC_MA3172AA	117	592
ESC_MA3171AA	69	387

ESC_MA3170AA	362	234
ESC_MA3169AA	429	654
ESC_MA3168AA	195	76
ESC_MA3167AA	10	512
ESC_MA3166AA	297	234
ESC_MA3165AA	665	79
ESC_MA3164AA	224	198
ESC_MA3163AA	162	924
ESC_MA3162AA	354	15
ESC_MA3161AA	131	653
ESC_MA3160AA	14	761
ESC_MA3159AA	95	738
ESC_MA3158AA	131	653
ESC_MA3157AA	127	738
ESC_MA3156AA	131	653
ESC_MA3155AA	69	387
ESC_MA3154AA	131	653
ESC_MA3153AA	73	737
ESC_MA3152AA	131	653
ESC_MA3151AA	59	217
ESC_MA3148AA	69	387
ESC_MA3146AA	1193	761
ESC_MA3145AA	69	387
ESC_MA3144AA	453	935
ESC_MA3143AA	2624	76
ESC_MA3142AA	162	924
ESC_MA3141AA	73	737
ESC_MA3140AA	131	653
ESC_MA3139AA	95	738
ESC_MA3138AA	1851	380
ESC_MA3137AA	117	592
ESC_MA3136AA	131	653
ESC_MA3135AA	1312	76
ESC_MA3133AA	73	737
ESC_MA3132AA	538	629
ESC_MA3131AA	101	696
ESC_MA3130AA	648	359
ESC_MA3129AA	131	653
ESC_MA3128AA	429	654
ESC_MA3127AA	963	669
ESC_MA3126AA	393	343
ESC_MA3125AA	58	198
ESC_MA3124AA	453	935
ESC_MA3122AA	131	653
ESC_MA3121AA	38	669
ESC_MA3120AA	73	737
ESC_MA3119AA	550	756
ESC_MA3118AA	131	653
ESC_MA3117AA	297	234
ESC_MA3116AA	345	175

ESC_MA3114AA	297	234
ESC_MA3113AA	141	644
ESC_MA3112AA	127	738
ESC_MA3110AA	537	756
ESC_MA3109AA	131	653
ESC_MA3108AA	405	923
ESC_MA3107AA	131	653
ESC_MA3106AA	44	512
ESC_MA3105AA	453	935
ESC_MA3104AA	131	653
ESC_MA3103AA	69	387
ESC_MA3102AA	73	737
ESC_MA3101AA	367	156
ESC_MA3100AA	131	653
ESC_MA3099AA	73	737
ESC_MA3098AA	95	738
ESC_MA3097AA	95	738
ESC_MA3096AA	2076	400
ESC_MA3095AA	73	737
ESC_MA3094AA	131	653
ESC_MA3093AA	131	653
ESC_MA3092AA	95	738
ESC_MA3091AA	73	737
ESC_MA3090AA	69	387
ESC_MA3089AA	1262	737
ESC_MA3088AA	127	738
ESC_MA3087AA	354	15
ESC_MA3086AA	69	387
ESC_MA3085AA	12	626
ESC_MA3084AA	58	198
ESC_MA3083AA	69	387
ESC_MA3082AA	224	198
ESC_MA3081AA	694	512
ESC_MA3080AA	131	653
ESC_MA3079AA	117	592
ESC_MA3078AA	93	71
ESC_MA3077AA	69	387
ESC_MA3076AA	95	738
ESC_MA3075AA	69	387
ESC_MA3074AA	73	737
ESC_MA3073AA	58	198
ESC_MA3072AA	69	387
ESC_MA3071AA	131	653
ESC_MA3070AA	405	923
ESC_MA3069AA	453	935
ESC_MA3068AA	354	15
ESC_MA3067AA	405	923
ESC_MA3066AA	405	923
ESC_MA3065AA	73	737
ESC_MA3064AA	359	696

ESC_MA3063AA	69	387
ESC_MA3062AA	101	696
ESC_MA3061AA	95	738
ESC_MA3060AA	457	595
ESC_MA3059AA	38	669
ESC_MA3058AA	131	653
ESC_MA3057AA	131	653
ESC_MA3056AA	131	653
ESC_MA3055AA	135	653
ESC_MA3054AA	95	738
ESC_MA3053AA	69	387
ESC_MA3052AA	405	923
ESC_MA3051AA	405	923
ESC_MA3050AA	95	738
ESC_MA3049AA	10	512
ESC_MA3048AA	1488	511
ESC_MA3046AA	58	198
ESC_MA3045AA	69	387
ESC_MA3044AA	154	257
ESC_MA3043AA	655	926
ESC_MA3042AA	16	156
ESC_MA3041AA	6346	191
ESC_MA3040AA	646	738
ESC_MA3039AA	372	424
ESC_MA3038AA	131	653
ESC_MA3037AA	5597	72
ESC_MA3036AA	127	738
ESC_MA3035AA	58	198
ESC_MA3034AA	69	387
ESC_MA3033AA	12	626
ESC_MA3032AA	2	900
ESC_MA3031AA	372	424
ESC_MA3030AA	245	261
ESC_MA3029AA	245	261
ESC_MA3028AA	152	257
ESC_MA3027AA	152	257
ESC_MA3026AA	200	198
ESC_MA3025AA	1797	512
ESC_MA3024AA	10	512
ESC_MA3023AA	1804	935
ESC_MA3022AA	504	738
ESC_MA3021AA	746	789
ESC_MA3019AA	131	653
ESC_MA3018AA	38	669
ESC_MA3017AA	131	653
ESC_MA3016AA	405	923
ESC_MA3015AA	5041	400
ESC_MA3014AA	131	653
ESC_MA3013AA	131	653
ESC_MA3012AA	131	653

ESC_MA3011AA	131	653
ESC_MA3010AA	301	307
ESC_MA3009AA	301	307
ESC_MA3008AA	301	307
ESC_MA3007AA	301	307
ESC_MA3006AA	100	504
ESC_MA3005AA	12	626
ESC_MA3004AA	12	626
ESC_MA3003AA	847	257
ESC_MA3002AA	100	504
ESC_MA3001AA	224	198
ESC_MA3000AA	12	626
ESC_MA2999AA	100	504
ESC_MA2998AA	2025	194
ESC_MA2997AA	744	512
ESC_MA2996AA	410	156
ESC_MA2995AA	93	71
ESC_MA2994AA	75	257
ESC_MA2993AA	10	512
ESC_MA2992AA	100	504
ESC_MA2991AA	101	696
ESC_MA2990AA	12	626
ESC_MA2989AA	10	512
ESC_MA2988AA	10	512
ESC_MA2987AA	88	160
ESC_MA2986AA	100	504
ESC_MA2985AA	2558	782
ESC_MA2984AA	398	445
ESC_MA2983AA	345	175
ESC_MA2982AA	227	504
ESC_MA2981AA	75	257
ESC_MA2980AA	345	175
ESC_MA2979AA	2521	257
ESC_MA2978AA	100	504
ESC_MA2977AA	100	504
ESC_MA2976AA	5926	50
ESC_MA2975AA	100	504
ESC_MA2974AA	101	696
ESC_MA2973AA	100	504
ESC_MA2972AA	100	504
ESC_MA2971AA	100	504
ESC_MA2970AA	3057	672
ESC_MA2969AA	12	626
ESC_MA2968AA	90	156
ESC_MA2967AA	101	696
ESC_MA2966AA	100	504
ESC_MA2965AA	100	504
ESC_MA2964AA	100	504
ESC_MA2963AA	152	257
ESC_MA2962AA	245	261

ESC_MA2961AA	245	261
ESC_MA2960AA	245	261
ESC_MA2959AA	11	936
ESC_MA2958AA	11	936
ESC_MA2957AA	11	936
ESC_MA2956AA	11	936
ESC_MA2955AA	11	936
ESC_MA2954AA	675	234
ESC_MA2953AA	11	936
ESC_MA2952AA	11	936
ESC_MA2951AA	200	198
ESC_MA2950AA	17	191
ESC_MA2948AA	127	738
ESC_MA2947AA	100	504
ESC_MA2946AA	100	504
ESC_MA2945AA	100	504
ESC_MA2944AA	10	512
ESC_MA2943AA	23	156
ESC_MA2942AA	5759	109
ESC_MA2941AA	101	696
ESC_MA2938AA	73	737
ESC_MA2937AA	69	387
ESC_MA2935AA	131	653
ESC_MA2934AA	131	653
ESC_MA2933AA	131	653
ESC_MA2931AA	69	387
ESC_MA2929AA	11	936
ESC_MA2928AA	21	156
ESC_MA2927AA	21	156
ESC_MA2926AA	21	156
ESC_MA2925AA	9225	669
ESC_MA2924AA	17	191
ESC_MA2923AA	17	191
ESC_MA2922AA	17	191
ESC_MA2921AA	69	387
ESC_MA2920AA	58	198
ESC_MA2918AA	10	512
ESC_MA2917AA	744	512
ESC_MA2916AA	617	511
ESC_MA2915AA	457	595
ESC_MA2914AA	761	512
ESC_MA2913AA	744	512
ESC_MA2912AA	101	696
ESC_MA2911AA	101	696
ESC_MA2910AA	154	257
ESC_MA2909AA	744	512
ESC_MA2908AA	744	512
ESC_MA2907AA	410	156
ESC_MA2906AA	369	156
ESC_MA2905AA	88	160

ESC_MA2904AA	12	626
ESC_MA2903AA	100	504
ESC_MA2902AA	12	626
ESC_MA2901AA	101	696
ESC_MA2900AA	12	626
ESC_MA2899AA	100	504
ESC_MA2898AA	100	504
ESC_MA2897AA	100	504
ESC_MA2896AA	12	626
ESC_MA2895AA	10	512
ESC_MA2894AA	152	257
ESC_MA2893AA	630	261
ESC_MA2892AA	152	257
ESC_MA2891AA	372	424
ESC_MA2890AA	131	653
ESC_MA2889AA	73	737
ESC_MA2888AA	4040	696
ESC_MA2887AA	744	512
ESC_MA2886AA	744	512
ESC_MA2885AA	224	198
ESC_MA2884AA	10	512
ESC_MA2883AA	7941	504
ESC_MA2882AA	744	512
ESC_MA2881AA	744	512
ESC_MA2880AA	167	512
ESC_MA2879AA	617	511
ESC_MA2878AA	10	512
ESC_MA2877AA	744	512
ESC_MA2876AA	744	512
ESC_MA2875AA	457	595
ESC_MA2874AA	744	512
ESC_MA2873AA	398	445
ESC_MA2872AA	767	198
ESC_MA2871AA	10	512
ESC_MA2870AA	641	935
ESC_MA2869AA	90	156
ESC_MA2868AA	9223	512
ESC_MA2867AA	4260	737
ESC_MA2866AA	847	257
ESC_MA2865AA	90	156
ESC_MA2864AA	2628	878
ESC_MA2863AA	641	935
ESC_MA2862AA	90	156
ESC_MA2861AA	12	626
ESC_MA2860AA	537	756
ESC_MA2859AA	847	257
ESC_MA2858AA	10	512
ESC_MA2857AA	847	257
ESC_MA2856AA	847	257
ESC_MA2855AA	9217	935

ESC_MA2854AA	90	156
ESC_MA2853AA	10	512
ESC_MA2851AA	9243	234
ESC_MA2850AA	6833	445
ESC_MA2849AA	641	935
ESC_MA2848AA	86	935
ESC_MA2847AA	58	198
ESC_MA2846AA	641	935
ESC_MA2845AA	9222	512
ESC_MA2844AA	9217	935
ESC_MA2843AA	131	653
ESC_MA2842AA	88	160
ESC_MA2841AA	101	696
ESC_MA2840AA	58	198
ESC_MA2839AA	10	512
ESC_MA2838AA	23	156
ESC_MA2837AA	58	198
ESC_MA2836AA	88	160
ESC_MA2835AA	4015	512
ESC_MA2834AA	58	198
ESC_MA2833AA	88	160
ESC_MA2832AA	58	198
ESC_MA2831AA	12	626
ESC_MA2830AA	12	626
ESC_MA2829AA	12	626
ESC_MA2828AA	12	626
ESC_MA2827AA	10	512
ESC_MA2826AA	12	626
ESC_MA2825AA	12	626
ESC_MA2824AA	12	626
ESC_MA2823AA	101	696
ESC_MA2822AA	90	156
ESC_MA2821AA	73	737
ESC_MA2820AA	12	626
ESC_MA2819AA	58	198
ESC_MA2818AA	6234	257
ESC_MA2817AA	58	198
ESC_MA2816AA	12	626
ESC_MA2815AA	100	504
ESC_MA2814AA	90	156
ESC_MA2813AA	10	512
ESC_MA2812AA	12	626
ESC_MA2811AA	10	512
ESC_MA2810AA	101	696
ESC_MA2809AA	12	626
ESC_MA2808AA	101	696
ESC_MA2807AA	23	156
ESC_MA2806AA	12	626
ESC_MA2805AA	10	512
ESC_MA2804AA	10	512

ESC_MA2803AA	75	257
ESC_MA2802AA	10	512
ESC_MA2801AA	90	156
ESC_MA2800AA	165	504
ESC_MA2799AA	58	198
ESC_MA2798AA	23	156
ESC_MA2797AA	10	512
ESC_MA2795AA	12	626
ESC_MA2794AA	4373	831
ESC_MA2793AA	23	156
ESC_MA2792AA	58	198
ESC_MA2791AA	410	156
ESC_MA2790AA	100	504
ESC_MA2789AA	100	504
ESC_MA2788AA	100	504
ESC_MA2787AA	1112	504
ESC_MA2786AA	1585	512
ESC_MA2785AA	744	512
ESC_MA2784AA	93	71
ESC_MA2783AA	224	198
ESC_MA2782AA	100	504
ESC_MA2781AA	9217	935
ESC_MA2780AA	641	935
ESC_MA2779AA	152	257
ESC_MA2778AA	2966	936
ESC_MA2777AA	1804	935
ESC_MA2776AA	17	191
ESC_MA2775AA	17	191
ESC_MA2774AA	17	191
ESC_MA2773AA	17	191
ESC_MA2772AA	20	198
ESC_MA2771AA	17	191
ESC_MA2770AA	20	198
ESC_MA2769AA	17	191
ESC_MA2768AA	386	191
ESC_MA2767AA	17	191
ESC_MA2766AA	17	191
ESC_MA2765AA	17	191
ESC_MA2764AA	69	387
ESC_MA2763AA	100	504
ESC_MA2762AA	100	504
ESC_MA2761AA	6234	257
ESC_MA2760AA	100	504
ESC_MA2759AA	100	504
ESC_MA2758AA	100	504
ESC_MA2757AA	641	935
ESC_MA2756AA	410	156
ESC_MA2755AA	10	512
ESC_MA2754AA	100	504
ESC_MA2753AA	93	71

ESC_MA2752AA	101	696
ESC_MA2751AA	100	504
ESC_MA2750AA	1112	504
ESC_MA2749AA	1585	512
ESC_MA2748AA	744	512
ESC_MA2747AA	93	71
ESC_MA2746AA	224	198
ESC_MA2745AA	100	504
ESC_MA2744AA	641	935
ESC_MA2743AA	9217	935
ESC_MA2741AA	100	504
ESC_MA2740AA	100	504
ESC_MA2739AA	6234	257
ESC_MA2738AA	100	504
ESC_MA2737AA	100	504
ESC_MA2736AA	4981	473
ESC_MA2735AA	2161	198
ESC_MA2734AA	100	504
ESC_MA2733AA	977	71
ESC_MA2732AA	744	512
ESC_MA2731AA	100	504
ESC_MA2730AA	744	512
ESC_MA2729AA	224	198
ESC_MA2728AA	100	504
ESC_MA2727AA	100	504
ESC_MA2726AA	10	512
ESC_MA2725AA	10	512
ESC_MA2724AA	3901	18
ESC_MA2723AA	6234	257
ESC_MA2722AA	58	198
ESC_MA2721AA	361	512
ESC_MA2720AA	540	286
ESC_MA2713AA	2496	789
ESC_MA2712AA	744	512
ESC_MA2711AA	4981	473
ESC_MA2709AA	744	512
ESC_MA2707AA	10	512
ESC_MA2700AA	58	198
ESC_MA2688AA	1060	512
ESC_MA2687AA	10	512
ESC_MA2686AA	10	512
ESC_MA2685AA	10	512
ESC_MA2684AA	10	512
ESC_MA2683AA	10	512
ESC_MA2682AA	10	512
ESC_MA2681AA	9213	653
ESC_MA2675AA	617	511
ESC_MA2674AA	9203	198
ESC_MA2673AA	245	261
ESC_MA2672AA	245	261

ESC_MA2671AA	52	71
ESC_MA2670AA	245	261
ESC_MA2669AA	245	261
ESC_MA2668AA	245	261
ESC_MA2667AA	245	261
ESC_MA2666AA	127	738
ESC_MA2665AA	5390	83
ESC_MA2664AA	32	270
ESC_MA2663AA	21	156
ESC_MA2662AA	937	198
ESC_MA2661AA	58	198
ESC_MA2660AA	189	504
ESC_MA2659AA	6011	924
ESC_MA2658AA	345	175
ESC_MA2657AA	648	359
ESC_MA2656AA	967	212
ESC_MA2655AA	1423	198
ESC_MA2654AA	906	198
ESC_MA2653AA	2541	878
ESC_MA2652AA	101	696
ESC_MA2651AA	2598	198
ESC_MA2650AA	3574	257
ESC_MA2649AA	189	504
ESC_MA2648AA	2035	113
ESC_MA2647AA	9224	447
ESC_MA2646AA	1011	198
ESC_MA2645AA	278	924
ESC_MA2644AA	11	936
ESC_MA2643AA	101	696
ESC_MA2642AA	32	270
ESC_MA2641AA	683	175
ESC_MA2640AA	245	261
ESC_MA2639AA	245	261
ESC_MA2638AA	152	257
ESC_MA2637AA	3345	445
ESC_MA2636AA	11	936
ESC_MA2635AA	155	175
ESC_MA2634AA	2064	261
ESC_MA2633AA	58	198
ESC_MA2632AA	1084	180
ESC_MA2631AA	648	359
ESC_MA2630AA	1720	935
ESC_MA2629AA	8855	175
ESC_MA2628AA	109	257
ESC_MA2627AA	58	198
ESC_MA2626AA	108	257
ESC_MA2625AA	2064	261
ESC_MA2624AA	109	257
ESC_MA2623AA	641	935
ESC_MA2622AA	752	512

ESC_MA2621AA	6449	504
ESC_MA2620AA	58	198
ESC_MA2619AA	17	191
ESC_MA2618AA	278	924
ESC_MA2617AA	761	512
ESC_MA2616AA	9197	184
ESC_MA2615AA	9196	198
ESC_MA2614AA	9199	234
ESC_MA2613AA	9198	924
ESC_MA2612AA	9195	885
ESC_MA2611AA	9193	567
ESC_MA2610AA	9188	50
ESC_MA2609AA	9190	156
ESC_MA2608AA	9191	512
ESC_MA2607AA	9194	198
ESC_MA2606AA	9192	595
ESC_MA2605AA	9172	160
ESC_MA2603AA	95	738
ESC_MA2602AA	73	737
ESC_MA2601AA	73	737
ESC_MA2600AA	73	737
ESC_MA2599AA	73	737
ESC_MA2597AA	9211	262
ESC_MA2596AA	95	738
ESC_MA2595AA	95	738
ESC_MA2594AA	95	738
ESC_MA2593AA	10	512
ESC_MA2592AA	152	257
ESC_MA2591AA	152	257
ESC_MA2590AA	245	261
ESC_MA2589AA	1273	256
ESC_MA2588AA	152	257
ESC_MA2587AA	245	261
ESC_MA2586AA	245	261
ESC_MA2585AA	245	261
ESC_MA2584AA	152	257
ESC_MA2583AA	245	261
ESC_MA2582AA	152	257
ESC_MA2581AA	245	261
ESC_MA2580AA	152	257
ESC_MA2579AA	11	936
ESC_MA2578AA	442	257
ESC_MA2577AA	33	198
ESC_MA2576AA	11	936
ESC_MA2575AA	1825	936
ESC_MA2574AA	442	257
ESC_MA2573AA	10	512
ESC_MA2572AA	21	156
ESC_MA2571AA	504	738
ESC_MA2570AA	3101	160

ESC_MA2569AA	58	198
ESC_MA2568AA	155	175
ESC_MA2567AA	201	257
ESC_MA2566AA	501	900
ESC_MA2565AA	43	512
ESC_MA2564AA	297	234
ESC_MA2563AA	345	175
ESC_MA2562AA	738	598
ESC_MA2561AA	1395	198
ESC_MA2560AA	1139	447
ESC_MA2559AA	906	198
ESC_MA2558AA	6836	544
ESC_MA2557AA	681	393
ESC_MA2556AA	1286	474
ESC_MA2555AA	8576	212
ESC_MA2554AA	58	198
ESC_MA2553AA	8576	212
ESC_MA2552AA	720	900
ESC_MA2551AA	9210	977
ESC_MA2550AA	9208	843
ESC_MA2549AA	11	936
ESC_MA2546AA	410	156
ESC_MA2543AA	141	644
ESC_MA2542AA	88	160
ESC_MA2541AA	131	653
ESC_MA2540AA	131	653
ESC_MA2539AA	648	359
ESC_MA2538AA	93	71
ESC_MA2537AA	783	204
ESC_MA2536AA	69	387
ESC_MA2535AA	131	653
ESC_MA2533AA	354	15
ESC_MA2531AA	131	653
ESC_MA2530AA	73	737
ESC_MA2529AA	131	653
ESC_MA2528AA	131	653
ESC_MA2526AA	131	653
ESC_MA2525AA	23	156
ESC_MA2524AA	141	644
ESC_MA2523AA	131	653
ESC_MA2522AA	58	198
ESC_MA2520AA	131	653
ESC_MA2519AA	90	156
ESC_MA2518AA	744	512
ESC_MA2517AA	73	737
ESC_MA2515AA	1421	447
ESC_MA2514AA	131	653
ESC_MA2513AA	141	644
ESC_MA2512AA	131	653
ESC_MA2511AA	1434	512

ESC_MA2510AA	131	653
ESC_MA2507AA	361	512
ESC_MA2505AA	10	512
ESC_MA2502AA	131	653
ESC_MA2499AA	648	359
ESC_MA2498AA	744	512
ESC_MA2496AA	448	257
ESC_MA2493AA	410	156
ESC_MA2490AA	131	653
ESC_MA2485AA	405	923
ESC_MA2484AA	226	512
ESC_MA2483AA	131	653
ESC_MA2482AA	38	669
ESC_MA2481AA	90	156
ESC_MA2479AA	648	359
ESC_MA2478AA	156	175
ESC_MA2477AA	9219	447
ESC_MA2472AA	88	160
ESC_MA2470AA	131	653
ESC_MA2468AA	2301	512
ESC_MA2467AA	58	198
ESC_MA2466AA	88	160
ESC_MA2465AA	420	741
ESC_MA2464AA	354	15
ESC_MA2462AA	12	626
ESC_MA2461AA	131	653
ESC_MA2460AA	405	923
ESC_MA2459AA	117	592
ESC_MA2457AA	131	653
ESC_MA2453AA	1177	669
ESC_MA2452AA	131	653
ESC_MA2451AA	205	257
ESC_MA2448AA	69	387
ESC_MA2447AA	349	670
ESC_MA2441AA	88	160
ESC_MA2440AA	131	653
ESC_MA2439AA	224	198
ESC_MA2437AA	88	160
ESC_MA2435AA	91	629
ESC_MA2433AA	354	15
ESC_MA2432AA	38	669
ESC_MA2431AA	162	924
ESC_MA2430AA	617	511
ESC_MA2429AA	10	512
ESC_MA2426AA	90	156
ESC_MA2423AA	69	387
ESC_MA2422AA	648	359
ESC_MA2419AA	73	737
ESC_MA2417AA	1011	198
ESC_MA2414AA	127	738

ESC_MA2413AA	1193	761
ESC_MA2411AA	23	156
ESC_MA2410AA	10	512
ESC_MA2409AA	9220	141
ESC_MA2408AA	131	653
ESC_MA2407AA	131	653
ESC_MA2406AA	73	737
ESC_MA2404AA	131	653
ESC_MA2403AA	744	512
ESC_MA2402AA	131	653
ESC_MA2395AA	131	653
ESC_MA2393AA	617	511
ESC_MA2392AA	173	254
ESC_MA2388AA	9221	387
ESC_MA2386AA	1196	257
ESC_MA2385AA	131	653
ESC_MA2384AA	226	512
ESC_MA2382AA	131	653
ESC_MA2380AA	90	156
ESC_MA2379AA	131	653
ESC_MA2378AA	117	592
ESC_MA2376AA	131	653
ESC_MA2373AA	1193	761
ESC_MA2372AA	641	935
ESC_MA2370AA	2013	725
ESC_MA2368AA	73	737
ESC_MA2367AA	69	387
ESC_MA2366AA	88	160
ESC_MA2364AA	12	626
ESC_MA2363AA	131	653
ESC_MA2362AA	1011	198
ESC_MA2361AA	34	504
ESC_MA2360AA	224	198
ESC_MA2359AA	58	198
ESC_MA2357AA	131	653
ESC_MA2356AA	69	387
ESC_MA2354AA	196	257
ESC_MA2353AA	10	512
ESC_MA2351AA	1193	761
ESC_MA2350AA	69	387
ESC_MA2349AA	131	653
ESC_MA2347AA	131	653
ESC_MA2346AA	1421	447
ESC_MA2344AA	131	653
ESC_MA2342AA	3057	672
ESC_MA2340AA	131	653
ESC_MA2339AA	131	653
ESC_MA2338AA	2083	257
ESC_MA2336AA	224	198
ESC_MA2333AA	648	359

ESC_MA2331AA	131	653
ESC_MA2330AA	131	653
ESC_MA2329AA	410	156
ESC_MA2328AA	2015	684
ESC_MA2327AA	162	924
ESC_MA2325AA	131	653
ESC_MA2323AA	10	512
ESC_MA2320AA	131	653
ESC_MA2319AA	141	644
ESC_MA2318AA	12	626
ESC_MA2317AA	141	644
ESC_MA2316AA	10	512
ESC_MA2314AA	131	653
ESC_MA2313AA	95	738
ESC_MA2312AA	117	592
ESC_MA2311AA	196	257
ESC_MA2310AA	162	924
ESC_MA2309AA	88	160
ESC_MA2307AA	847	257
ESC_MA2306AA	405	923
ESC_MA2305AA	450	71
ESC_MA2304AA	131	653
ESC_MA2303AA	117	592
ESC_MA2301AA	12	626
ESC_MA2299AA	12	626
ESC_MA2298AA	131	653
ESC_MA2297AA	90	156
ESC_MA2295AA	550	756
ESC_MA2294AA	648	359
ESC_MA2293AA	10	512
ESC_MA2292AA	131	653
ESC_MA2291AA	1011	198
ESC_MA2290AA	131	653
ESC_MA2288AA	131	653
ESC_MA2287AA	162	924
ESC_MA2286AA	141	644
ESC_MA2284AA	1434	512
ESC_MA2282AA	1193	761
ESC_MA2281AA	93	71
ESC_MA2280AA	90	156
ESC_MA2276AA	744	512
ESC_MA2272AA	131	653
ESC_MA2271AA	1193	761
ESC_MA2269AA	2083	257
ESC_MA2268AA	1480	160
ESC_MA2266AA	10	512
ESC_MA2265AA	362	234
ESC_MA2263AA	90	156
ESC_MA2262AA	1193	761
ESC_MA2261AA	354	15

ESC_MA2259AA	1431	257
ESC_MA2258AA	131	653
ESC_MA2255AA	9233	629
ESC_MA2252AA	131	653
ESC_MA2248AA	196	257
ESC_MA2246AA	405	923
ESC_MA2242AA	1011	198
ESC_MA2241AA	73	737
ESC_MA2239AA	648	359
ESC_MA2238AA	156	175
ESC_MA2237AA	410	156
ESC_MA2235AA	34	504
ESC_MA2234AA	117	592
ESC_MA2231AA	1193	761
ESC_MA2230AA	131	653
ESC_MA2228AA	10	512
ESC_MA2226AA	101	696
ESC_MA2224AA	131	653
ESC_MA2223AA	354	15
ESC_MA2222AA	127	738
ESC_MA2221AA	405	923
ESC_MA2219AA	69	387
ESC_MA2218AA	362	234
ESC_MA2217AA	354	15
ESC_MA2214AA	38	669
ESC_MA2212AA	131	653
ESC_MA2211AA	69	387
ESC_MA2206AA	167	512
ESC_MA2205AA	127	738
ESC_MA2204AA	10	512
ESC_MA2203AA	95	738
ESC_MA2202AA	196	257
ESC_MA2201AA	131	653
ESC_MA2200AA	10	512
ESC_MA2199AA	131	653
ESC_MA2197AA	58	198
ESC_MA2194AA	744	512
ESC_MA2193AA	162	924
ESC_MA2191AA	88	160
ESC_MA2190AA	131	653
ESC_MA2187AA	2003	669
ESC_MA2186AA	1193	761
ESC_MA2185AA	10	512
ESC_MA2184AA	69	387
ESC_MA2180AA	131	653
ESC_MA2178AA	69	387
ESC_MA2177AA	410	156
ESC_MA2176AA	1011	198
ESC_MA2172AA	131	653
ESC_MA2171AA	744	512

ESC_MA2166AA	45	286
ESC_MA2163AA	45	286
ESC_MA2160AA	2345	795
ESC_MA2159AA	405	923
ESC_MA2157AA	10	512
ESC_MA2156AA	73	737
ESC_MA2154AA	131	653
ESC_MA2153AA	88	160
ESC_MA2151AA	141	644
ESC_MA2150AA	141	644
ESC_MA2149AA	131	653
ESC_MA2148AA	131	653
ESC_MA2147AA	200	198
ESC_MA2145AA	410	156
ESC_MA2144AA	73	737
ESC_MA2142AA	354	15
ESC_MA2141AA	1193	761
ESC_MA2140AA	335	936
ESC_MA2138AA	453	935
ESC_MA2135AA	405	923
ESC_MA2134AA	12	626
ESC_MA2133AA	131	653
ESC_MA2132AA	399	198
ESC_MA2131AA	88	160
ESC_MA2129AA	38	669
ESC_MA2125AA	69	387
ESC_MA2121AA	141	644
ESC_MA2118AA	69	387
ESC_MA2117AA	648	359
ESC_MA2114AA	4456	654
ESC_MA2113AA	141	644
ESC_MA2112AA	12	626
ESC_MA2111AA	131	653
ESC_MA2107AA	162	924
ESC_MA2106AA	95	738
ESC_MA2104AA	141	644
ESC_MA2103AA	648	359
ESC_MA2099AA	1193	761
ESC_MA2097AA	354	15
ESC_MA2096AA	73	737
ESC_MA2095AA	744	512
ESC_MA2093AA	131	653
ESC_MA2092AA	6784	701
ESC_MA2090AA	58	198
ESC_MA2089AA	1193	761
ESC_MA2088AA	131	653
ESC_MA2087AA	167	512
ESC_MA2082AA	88	160
ESC_MA2079AA	2345	795
ESC_MA2078AA	410	156

ESC_MA2077AA	847	257
ESC_MA2073AA	88	160
ESC_MA2072AA	443	257
ESC_MA2069AA	91	629
ESC_MA2068AA	131	653
ESC_MA2067AA	648	359
ESC_MA2064AA	69	387
ESC_MA2063AA	9204	653
ESC_MA2062AA	58	198
ESC_MA2061AA	141	644
ESC_MA2060AA	245	261
ESC_MA2059AA	628	261
ESC_MA2058AA	152	257
ESC_MA2057AA	245	261
ESC_MA2056AA	245	261
ESC_MA2055AA	245	261
ESC_MA2054AA	245	261
ESC_MA2053AA	245	261
ESC_MA2052AA	245	261
ESC_MA2051AA	152	257
ESC_MA2050AA	145	256
ESC_MA2049AA	1767	928
ESC_MA2048AA	245	261
ESC_MA2047AA	152	257
ESC_MA2046AA	152	257
ESC_MA2045AA	10	512
ESC_MA2044AA	10	512
ESC_MA2043AA	10	512
ESC_MA2042AA	9240	512
ESC_MA2041AA	10	512
ESC_MA2040AA	362	234
ESC_MA2039AA	131	653
ESC_MA2036AA	69	387
ESC_MA2035AA	69	387
ESC_MA2034AA	59	217
ESC_MA2033AA	69	387
ESC_MA2032AA	73	737
ESC_MA2031AA	80	731
ESC_MA2030AA	73	737
ESC_MA2029AA	73	737
ESC_MA2028AA	127	738
ESC_MA2027AA	69	387
ESC_MA2026AA	404	761
ESC_MA2025AA	95	738
ESC_MA2024AA	131	653
ESC_MA2023AA	117	592
ESC_MA2022AA	73	737
ESC_MA2021AA	73	737
ESC_MA2020AA	73	737
ESC_MA2019AA	127	738

ESC_MA2018AA	69	387
ESC_MA2017AA	69	387
ESC_MA2016AA	297	234
ESC_MA2015AA	155	175
ESC_MA2014AA	69	387
ESC_MA2013AA	139	900
ESC_MA2012AA	69	387
ESC_MA2011AA	64	978
ESC_MA2010AA	1060	512
ESC_MA2009AA	10	512
ESC_MA2008AA	10	512
ESC_MA2007AA	10	512
ESC_MA2006AA	10	512
ESC_MA2005AA	9238	108
ESC_MA2004AA	12	626
ESC_MA2003AA	73	737
ESC_MA2002AA	131	653
ESC_MA2001AA	131	653
ESC_MA2000AA	10	512
ESC_MA1999AA	355	764
ESC_MA1998AA	12	626
ESC_MA1997AA	95	738
ESC_MA1996AA	10	512
ESC_MA1995AA	17	191
ESC_MA1994AA	442	257
ESC_MA1993AA	7403	304
ESC_MA1992AA	17	191
ESC_MA1991AA	2514	504
ESC_MA1990AA	316	257
ESC_MA1989AA	21	156
ESC_MA1988AA	32	270
ESC_MA1987AA	6	445
ESC_MA1982AA	6	445
ESC_MA1981AA	21	156
ESC_MA1977AA	152	257
ESC_MA1976AA	6271	512
ESC_MA1975AA	245	261
ESC_MA1974AA	131	653
ESC_MA1973AA	362	234
ESC_MA1972AA	648	359
ESC_MA1971AA	38	669
ESC_MA1968AA	11	936
ESC_MA1967AA	4619	122
ESC_MA1966AA	122	768
ESC_MA1953AA	95	738
ESC_MA1952AA	646	738
ESC_MA1951AA	131	653
ESC_MA1950AA	131	653
ESC_MA1949AA	583	768
ESC_MA1948AA	3601	653

ESC_MA1947AA	1304	257
ESC_MA1946AA	6497	221
ESC_MA1945AA	1395	198
ESC_MA1944AA	93	71
ESC_MA1943AA	11	936
ESC_MA1942AA	11	936
ESC_MA1941AA	10	512
ESC_MA1940AA	48	76
ESC_MA1939AA	58	198
ESC_MA1938AA	10	512
ESC_MA1937AA	295	160
ESC_MA1936AA	224	198
ESC_MA1933AA	2705	504
ESC_MA1932AA	1844	198
ESC_MA1931AA	10	512
ESC_MA1930AA	10	512
ESC_MA1913AA	12	626
ESC_MA1912AA	12	626
ESC_MA1910AA	12	626
ESC_MA1909AA	12	626
ESC_MA1908AA	9183	737
ESC_MA1907AA	9182	738
ESC_MA1906AA	28	537
ESC_MA1905AA	12	626
ESC_MA1903AA	245	261
ESC_MA1902AA	245	261
ESC_MA1901AA	245	261
ESC_MA1900AA	245	261
ESC_MA1899AA	245	261
ESC_MA1898AA	245	261
ESC_MA1897AA	245	261
ESC_MA1896AA	245	261
ESC_MA1895AA	245	261
ESC_MA1894AA	145	256
ESC_MA1893AA	145	256
ESC_MA1892AA	145	256
ESC_MA1891AA	145	256
ESC_MA1890AA	245	261
ESC_MA1889AA	245	261
ESC_MA1888AA	245	261
ESC_MA1887AA	245	261
ESC_MA1886AA	245	261
ESC_MA1885AA	245	261
ESC_MA1884AA	152	257
ESC_MA1883AA	245	261
ESC_MA1882AA	5475	256
ESC_MA1881AA	5475	256
ESC_MA1880AA	5475	256
ESC_MA1879AA	152	257
ESC_MA1878AA	9150	257

ESC_MA1877AA	9149	235
ESC_MA1876AA	9146	233
ESC_MA1875AA	9148	288
ESC_MA1874AA	9146	233
ESC_MA1872AA	9147	198
ESC_MA1871AA	8899	198
ESC_MA1870AA	152	257
ESC_MA1869AA	9144	257
ESC_MA1868AA	245	261
ESC_MA1867AA	9145	261
ESC_MA1866AA	152	257
ESC_MA1865AA	245	261
ESC_MA1864AA	152	257
ESC_MA1863AA	56	198
ESC_MA1862AA	58	198
ESC_MA1861AA	101	696
ESC_MA1860AA	10	512
ESC_MA1859AA	603	198
ESC_MA1858AA	189	504
ESC_MA1857AA	4532	234
ESC_MA1856AA	3232	198
ESC_MA1855AA	4559	878
ESC_MA1854AA	10	512
ESC_MA1853AA	648	359
ESC_MA1852AA	164	175
ESC_MA1851AA	655	926
ESC_MA1850AA	641	935
ESC_MA1849AA	906	198
ESC_MA1848AA	155	175
ESC_MA1847AA	4086	842
ESC_MA1846AA	937	198
ESC_MA1845AA	101	696
ESC_MA1844AA	117	592
ESC_MA1843AA	101	696
ESC_MA1842AA	88	160
ESC_MA1841AA	88	160
ESC_MA1840AA	540	286
ESC_MA1839AA	9181	175
ESC_MA1815AA	100	504
ESC_MA1814AA	906	198
ESC_MA1813AA	4607	900
ESC_MA1812AA	90	156
ESC_MA1811AA	345	175
ESC_MA1810AA	547	653
ESC_MA1809AA	2585	953
ESC_MA1808AA	9171	250
ESC_MA1807AA	2852	257
ESC_MA1806AA	90	156
ESC_MA1805AA	3558	234
ESC_MA1804AA	1304	257

ESC_MA1803AA	131	653
ESC_MA1802AA	127	738
ESC_MA1801AA	540	286
ESC_MA1800AA	58	198
ESC_MA1799AA	9173	50
ESC_MA1798AA	2248	257
ESC_MA1797AA	9172	160
ESC_MA1796AA	540	286
ESC_MA1795AA	152	257
ESC_MA1794AA	152	257
ESC_MA1793AA	152	257
ESC_MA1792AA	152	257
ESC_MA1791AA	152	257
ESC_MA1788AA	152	257
ESC_MA1787AA	152	257
ESC_MA1786AA	152	257
ESC_MA1785AA	152	257
ESC_MA1784AA	152	257
ESC_MA1783AA	152	257
ESC_MA1779AA	152	257
ESC_MA1778AA	152	257
ESC_MA1777AA	152	257
ESC_MA1776AA	152	257
ESC_MA1774AA	152	257
ESC_MA1772AA	152	257
ESC_MA1771AA	152	257
ESC_MA1770AA	152	257
ESC_MA1766AA	152	257
ESC_MA1765AA	152	257
ESC_MA1764AA	152	257
ESC_MA1763AA	152	257
ESC_MA1762AA	152	257
ESC_MA1761AA	152	257
ESC_MA1760AA	152	257
ESC_MA1759AA	152	257
ESC_MA1757AA	152	257
ESC_MA1756AA	152	257
ESC_MA1755AA	152	257
ESC_MA1754AA	152	257
ESC_MA1753AA	152	257
ESC_MA1752AA	152	257
ESC_MA1751AA	152	257
ESC_MA1750AA	152	257
ESC_MA1748AA	152	257
ESC_MA1747AA	9164	257
ESC_MA1746AA	152	257
ESC_MA1743AA	152	257
ESC_MA1742AA	152	257
ESC_MA1741AA	152	257
ESC_MA1740AA	152	257

ESC_MA1739AA	152	257
ESC_MA1737AA	152	257
ESC_MA1736AA	152	257
ESC_MA1735AA	152	257
ESC_MA1734AA	152	257
ESC_MA1733AA	152	257
ESC_MA1732AA	152	257
ESC_MA1731AA	152	257
ESC_MA1730AA	152	257
ESC_MA1729AA	152	257
ESC_MA1728AA	152	257
ESC_MA1727AA	152	257
ESC_MA1726AA	152	257
ESC_MA1725AA	152	257
ESC_MA1724AA	152	257
ESC_MA1723AA	152	257
ESC_MA1722AA	152	257
ESC_MA1721AA	152	257
ESC_MA1720AA	152	257
ESC_MA1719AA	152	257
ESC_MA1718AA	152	257
ESC_MA1716AA	152	257
ESC_MA1715AA	152	257
ESC_MA1707AA	648	359
ESC_MA1706AA	405	923
ESC_MA1705AA	69	387
ESC_MA1704AA	410	156
ESC_MA1701AA	1193	761
ESC_MA1700AA	1193	761
ESC_MA1697AA	31	387
ESC_MA1693AA	226	512
ESC_MA1692AA	224	198
ESC_MA1691AA	38	669
ESC_MA1689AA	9167	515
ESC_MA1688AA	362	234
ESC_MA1681AA	69	387
ESC_MA1679AA	1193	761
ESC_MA1677AA	226	512
ESC_MA1675AA	3268	669
ESC_MA1674AA	38	669
ESC_MA1673AA	9166	504
ESC_MA1671AA	9170	445
ESC_MA1670AA	1193	761
ESC_MA1669AA	1193	761
ESC_MA1664AA	131	653
ESC_MA1663AA	1177	669
ESC_MA1660AA	9169	592
ESC_MA1659AA	9168	198
ESC_MA1658AA	226	512
ESC_MA1657AA	3075	701

ESC_MA1656AA	43	512
ESC_MA1655AA	657	592
ESC_MA1654AA	131	653
ESC_MA1653AA	131	653
ESC_MA1652AA	131	653
ESC_MA1650AA	69	387
ESC_MA1649AA	58	198
ESC_MA1648AA	617	511
ESC_MA1645AA	1193	761
ESC_MA1644AA	131	653
ESC_MA1643AA	131	653
ESC_MA1640AA	44	512
ESC_MA1639AA	38	669
ESC_MA1638AA	1193	761
ESC_MA1637AA	131	653
ESC_MA1635AA	93	71
ESC_MA1634AA	405	923
ESC_MA1633AA	62	574
ESC_MA1631AA	10	512
ESC_MA1629AA	69	387
ESC_MA1627AA	131	653
ESC_MA1626AA	12	626
ESC_MA1624AA	405	923
ESC_MA1623AA	636	653
ESC_MA1622AA	648	359
ESC_MA1620AA	10	512
ESC_MA1619AA	38	669
ESC_MA1618AA	69	387
ESC_MA1616AA	88	160
ESC_MA1614AA	38	669
ESC_MA1612AA	131	653
ESC_MA1611AA	131	653
ESC_MA1610AA	5150	661
ESC_MA1609AA	95	738
ESC_MA1608AA	2020	387
ESC_MA1607AA	131	653
ESC_MA1606AA	131	653
ESC_MA1605AA	69	387
ESC_MA1604AA	38	669
ESC_MA1603AA	131	653
ESC_MA1602AA	69	387
ESC_MA1601AA	38	669
ESC_MA1600AA	131	653
ESC_MA1599AA	131	653
ESC_MA1598AA	131	653
ESC_MA1597AA	131	653
ESC_MA1596AA	23	156
ESC_MA1595AA	555	653
ESC_MA1594AA	131	653
ESC_MA1593AA	88	160

ESC_MA1592AA	1564	512
ESC_MA1590AA	73	737
ESC_MA1589AA	1193	761
ESC_MA1587AA	131	653
ESC_MA1586AA	131	653
ESC_MA1585AA	69	387
ESC_MA1584AA	90	156
ESC_MA1583AA	131	653
ESC_MA1582AA	362	234
ESC_MA1581AA	131	653
ESC_MA1580AA	131	653
ESC_MA1578AA	12	626
ESC_MA1577AA	48	76
ESC_MA1576AA	131	653
ESC_MA1575AA	131	653
ESC_MA1574AA	10	512
ESC_MA1573AA	131	653
ESC_MA1572AA	131	653
ESC_MA1571AA	501	900
ESC_MA1570AA	23	156
ESC_MA1569AA	131	653
ESC_MA1568AA	2178	924
ESC_MA1567AA	131	653
ESC_MA1566AA	131	653
ESC_MA1565AA	10	512
ESC_MA1564AA	453	935
ESC_MA1563AA	295	160
ESC_MA1562AA	9174	175
ESC_MA1561AA	10	512
ESC_MA1560AA	58	198
ESC_MA1559AA	641	935
ESC_MA1558AA	6917	261
ESC_MA1557AA	152	257
ESC_MA1556AA	6917	261
ESC_MA1555AA	245	261
ESC_MA1554AA	5475	256
ESC_MA1553AA	1025	261
ESC_MA1552AA	152	257
ESC_MA1551AA	152	257
ESC_MA1550AA	243	197
ESC_MA1549AA	152	257
ESC_MA1548AA	245	261
ESC_MA1547AA	6666	261
ESC_MA1546AA	152	257
ESC_MA1545AA	245	261
ESC_MA1544AA	17	191
ESC_MA1543AA	11	936
ESC_MA1542AA	1804	935
ESC_MA1541AA	17	191
ESC_MA1540AA	4619	122

ESC_MA1539AA	21	156
ESC_MA1538AA	11	936
ESC_MA1537AA	141	644
ESC_MA1536AA	141	644
ESC_MA1535AA	141	644
ESC_MA1534AA	141	644
ESC_MA1533AA	141	644
ESC_MA1532AA	141	644
ESC_MA1531AA	9139	761
ESC_MA1530AA	141	644
ESC_MA1529AA	6467	452
ESC_MA1528AA	9138	878
ESC_MA1527AA	297	234
ESC_MA1526AA	9137	900
ESC_MA1525AA	245	261
ESC_MA1524AA	5517	257
ESC_MA1523AA	152	257
ESC_MA1522AA	152	257
ESC_MA1521AA	245	261
ESC_MA1520AA	152	257
ESC_MA1519AA	152	257
ESC_MA1518AA	11	936
ESC_MA1517AA	5560	929
ESC_MA1516AA	11	936
ESC_MA1515AA	11	936
ESC_MA1514AA	11	936
ESC_MA1513AA	11	936
ESC_MA1512AA	11	936
ESC_MA1511AA	11	936
ESC_MA1510AA	11	936
ESC_MA1509AA	11	936
ESC_MA1508AA	5560	929
ESC_MA1501AA	1804	935
ESC_MA1500AA	16	156
ESC_MA1499AA	5292	738
ESC_MA1498AA	122	768
ESC_MA1497AA	10	512
ESC_MA1496AA	6	445
ESC_MA1495AA	1819	808
ESC_MA1494AA	155	175
ESC_MA1493AA	48	76
ESC_MA1492AA	48	76
ESC_MA1491AA	9124	445
ESC_MA1490AA	9125	257
ESC_MA1488AA	57	50
ESC_MA1487AA	9060	261
ESC_MA1486AA	11	936
ESC_MA1485AA	11	936
ESC_MA1484AA	11	936
ESC_MA1483AA	372	424

ESC_MA1482AA	961	623
ESC_MA1481AA	9143	653
ESC_MA1480AA	127	738
ESC_MA1479AA	73	737
ESC_MA1478AA	1735	737
ESC_MA1477AA	167	512
ESC_MA1476AA	405	923
ESC_MA1475AA	69	387
ESC_MA1474AA	1486	71
ESC_MA1473AA	405	923
ESC_MA1472AA	34	504
ESC_MA1471AA	399	198
ESC_MA1470AA	2851	156
ESC_MA1468AA	141	644
ESC_MA1467AA	141	644
ESC_MA1466AA	141	644
ESC_MA1465AA	141	644
ESC_MA1464AA	141	644
ESC_MA1463AA	141	644
ESC_MA1462AA	141	644
ESC_MA1460AA	141	644
ESC_MA1459AA	17	191
ESC_MA1458AA	17	191
ESC_MA1456AA	17	191
ESC_MA1447AA	11	936
ESC_MA1446AA	11	936
ESC_MA1445AA	11	936
ESC_MA1444AA	11	936
ESC_MA1443AA	11	936
ESC_MA1442AA	11	936
ESC_MA1441AA	11	936
ESC_MA1440AA	11	936
ESC_MA1439AA	11	936
ESC_MA1438AA	68	386
ESC_MA1437AA	46	447
ESC_MA1436AA	167	512
ESC_MA1435AA	68	386
ESC_MA1434AA	8346	234
ESC_MA1433AA	361	512
ESC_MA1431AA	405	923
ESC_MA1429AA	294	156
ESC_MA1428AA	21	156
ESC_MA1427AA	677	257
ESC_MA1426AA	33	198
ESC_MA1425AA	641	935
ESC_MA1424AA	223	198
ESC_MA1423AA	223	198
ESC_MA1422AA	17	191
ESC_MA1421AA	5934	257
ESC_MA1420AA	17	191

ESC_MA1419AA	641	935
ESC_MA1418AA	25	234
ESC_MA1417AA	5977	156
ESC_MA1416AA	17	191
ESC_MA1415AA	21	156
ESC_MA1414AA	332	257
ESC_MA1413AA	21	156
ESC_MA1412AA	21	156
ESC_MA1411AA	655	926
ESC_MA1409AA	21	156
ESC_MA1408AA	223	198
ESC_MA1407AA	25	234
ESC_MA1406AA	21	156
ESC_MA1405AA	294	156
ESC_MA1404AA	21	156
ESC_MA1403AA	442	257
ESC_MA1402AA	17	191
ESC_MA1401AA	21	156
ESC_MA1400AA	205	257
ESC_MA1399AA	25	234
ESC_MA1398AA	21	156
ESC_MA1397AA	25	234
ESC_MA1396AA	294	156
ESC_MA1395AA	25	234
ESC_MA1394AA	294	156
ESC_MA1393AA	21	156
ESC_MA1392AA	442	257
ESC_MA1391AA	223	198
ESC_MA1390AA	25	234
ESC_MA1389AA	245	261
ESC_MA1387AA	95	738
ESC_MA1386AA	95	738
ESC_MA1370AA	145	256
ESC_MA1369AA	648	359
ESC_MA1368AA	5400	261
ESC_MA1367AA	245	261
ESC_MA1366AA	538	629
ESC_MA1365AA	906	198
ESC_MA1364AA	58	198
ESC_MA1363AA	32	270
ESC_MA1362AA	278	924
ESC_MA1361AA	1326	198
ESC_MA1360AA	362	234
ESC_MA1359AA	109	257
ESC_MA1358AA	7894	924
ESC_MA1357AA	906	198
ESC_MA1356AA	56	198
ESC_MA1355AA	5082	198
ESC_MA1354AA	58	198
ESC_MA1353AA	9012	424

ESC_MA1352AA	9013	175
ESC_MA1351AA	3580	257
ESC_MA1350AA	3234	257
ESC_MA1349AA	3580	257
ESC_MA1348AA	8220	447
ESC_MA1347AA	5956	71
ESC_MA1346AA	167	512
ESC_MA1345AA	405	923
ESC_MA1344AA	2079	198
ESC_MA1343AA	1034	270
ESC_MA1342AA	7207	960
ESC_MA1341AA	109	257
ESC_MA1340AA	278	924
ESC_MA1339AA	34	504
ESC_MA1338AA	540	286
ESC_MA1337AA	9142	512
ESC_MA1336AA	10	512
ESC_MA1335AA	101	696
ESC_MA1334AA	252	190
ESC_MA1333AA	152	257
ESC_MA1332AA	152	257
ESC_MA1331AA	152	257
ESC_MA1330AA	152	257
ESC_MA1329AA	245	261
ESC_MA1328AA	152	257
ESC_MA1327AA	243	197
ESC_MA1326AA	245	261
ESC_MA1325AA	288	102
ESC_MA1324AA	278	924
ESC_MA1323AA	69	387
ESC_MA1322AA	398	445
ESC_MA1321AA	3346	592
ESC_MA1320AA	69	387
ESC_MA1319AA	297	234
ESC_MA1318AA	398	445
ESC_MA1317AA	297	234
ESC_MA1316AA	17	191
ESC_MA1315AA	20	198
ESC_MA1314AA	17	191
ESC_MA1313AA	17	191
ESC_MA1312AA	17	191
ESC_MA1311AA	20	198
ESC_MA1310AA	17	191
ESC_MA1309AA	17	191
ESC_MA1308AA	17	191
ESC_MA1307AA	386	191
ESC_MA1306AA	17	191
ESC_MA1305AA	17	191
ESC_MA1304AA	540	286
ESC_MA1303AA	58	198

ESC_MA1302AA	38	669
ESC_MA1301AA	8140	878
ESC_MA1300AA	398	445
ESC_MA1299AA	131	653
ESC_MA1298AA	278	924
ESC_MA1297AA	6	445
ESC_MA1296AA	6	445
ESC_MA1294AA	6	445
ESC_MA1293AA	6	445
ESC_MA1292AA	311	204
ESC_MA1290AA	245	261
ESC_MA1289AA	245	261
ESC_MA1288AA	152	257
ESC_MA1287AA	152	257
ESC_MA1286AA	152	257
ESC_MA1285AA	152	257
ESC_MA1284AA	245	261
ESC_MA1283AA	152	257
ESC_MA1282AA	152	257
ESC_MA1281AA	152	257
ESC_MA1280AA	243	197
ESC_MA1279AA	152	257
ESC_MA1278AA	288	102
ESC_MA1277AA	245	261
ESC_MA1276AA	245	261
ESC_MA1275AA	152	257
ESC_MA1274AA	245	261
ESC_MA1273AA	152	257
ESC_MA1272AA	11	936
ESC_MA1271AA	11	936
ESC_MA1270AA	8291	350
ESC_MA1269AA	11	936
ESC_MA1268AA	10	512
ESC_MA1267AA	675	234
ESC_MA1266AA	17	191
ESC_MA1265AA	33	198
ESC_MA1264AA	738	598
ESC_MA1263AA	404	761
ESC_MA1262AA	152	257
ESC_MA1260AA	56	198
ESC_MA1259AA	9140	160
ESC_MA1258AA	1508	261
ESC_MA1257AA	1101	842
ESC_MA1256AA	767	198
ESC_MA1255AA	11	936
ESC_MA1254AA	117	592
ESC_MA1253AA	9141	877
ESC_MA1252AA	11	936
ESC_MA1251AA	11	936
ESC_MA1250AA	11	936

ESC_MA1249AA	295	160
ESC_MA1248AA	11	936
ESC_MA1247AA	10	512
ESC_MA1246AA	10	512
ESC_MA1245AA	716	795
ESC_MA1244AA	716	795
ESC_MA1243AA	716	795
ESC_MA1242AA	2466	307
ESC_MA1091AA	205	257
ESC_MA1089AA	90	156
ESC_MA1087AA	8346	234
ESC_MA1085AA	448	257
ESC_MA1082AA	156	175
ESC_MA1080AA	101	696
ESC_MA1079AA	405	923
ESC_MA1077AA	8346	234
ESC_MA1074AA	101	696
ESC_MA1073AA	405	923
ESC_MA1071AA	410	156
ESC_MA1070AA	405	923
ESC_MA1068AA	973	968
ESC_MA1067AA	162	924
ESC_MA1066AA	7706	525
ESC_MA1065AA	372	424
ESC_MA1064AA	1148	257
ESC_MA1063AA	372	424
ESC_MA1062AA	73	737
ESC_MA1061AA	372	424
ESC_MA1060AA	372	424
ESC_MA1059AA	127	738
ESC_MA1058AA	162	924
ESC_MA1057AA	127	738
ESC_MA1056AA	646	738
ESC_MA1055AA	131	653
ESC_MA1054AA	4198	878
ESC_MA1053AA	12	626
ESC_MA1052AA	372	424
ESC_MA1051AA	3672	649
ESC_MA1050AA	906	198
ESC_MA1049AA	617	511
ESC_MA1048AA	162	924
ESC_MA1045AA	162	924
ESC_MA1043AA	4748	234
ESC_MA1042AA	200	198
ESC_MA1041AA	200	198
ESC_MA1040AA	33	198
ESC_MA1039AA	442	257
ESC_MA1038AA	335	936
ESC_MA1037AA	7368	511
ESC_MA1036AA	1432	257

ESC_MA1035AA	58	198
ESC_MA1034AA	9136	257
ESC_MA1033AA	7538	445
ESC_MA1032AA	58	198
ESC_MA1031AA	117	592
ESC_MA1030AA	4038	257
ESC_MA1029AA	8161	76
ESC_MA1028AA	453	935
ESC_MA1027AA	88	160
ESC_MA1026AA	16	156
ESC_MA1025AA	1172	234
ESC_MA1024AA	4198	878
ESC_MA1023AA	58	198
ESC_MA1022AA	10	512
ESC_MA1021AA	131	653
ESC_MA1020AA	297	234
ESC_MA1019AA	10	512
ESC_MA1018AA	10	512
ESC_MA1017AA	109	257
ESC_MA1016AA	939	198
ESC_MA1015AA	410	156
ESC_MA1014AA	409	504
ESC_MA1013AA	1060	512
ESC_MA1012AA	1060	512
ESC_MA1011AA	1060	512
ESC_MA1010AA	1060	512
ESC_MA1009AA	1060	512
ESC_MA1008AA	1060	512
ESC_MA1007AA	1060	512
ESC_MA1004AA	405	923
ESC_MA1003AA	245	261
ESC_MA1002AA	245	261
ESC_MA1001AA	245	261
ESC_MA1000AA	245	261
ESC_MA0999AA	152	257
ESC_MA0998AA	152	257
ESC_MA0997AA	152	257
ESC_MA0996AA	9060	261
ESC_MA0995AA	517	924
ESC_MA0994AA	10	512
ESC_MA0993AA	301	307
ESC_MA0992AA	738	598
ESC_MA0991AA	906	198
ESC_MA0990AA	156	175
ESC_MA0989AA	2466	307
ESC_MA0988AA	8993	519
ESC_MA0987AA	2466	307
ESC_MA0986AA	716	795
ESC_MA0985AA	2466	307
ESC_MA0969AA	11	936

ESC_MA0968AA	11	936
ESC_MA0967AA	8986	789
ESC_MA0966AA	152	257
ESC_MA0965AA	152	257
ESC_MA0964AA	145	256
ESC_MA0963AA	152	257
ESC_MA0962AA	245	261
ESC_MA0961AA	152	257
ESC_MA0960AA	152	257
ESC_MA0959AA	245	261
ESC_MA0958AA	245	261
ESC_MA0957AA	9134	159
ESC_MA0956AA	29	156
ESC_MA0955AA	301	307
ESC_MA0954AA	301	307
ESC_MA0953AA	10	512
ESC_MA0952AA	21	156
ESC_MA0951AA	17	191
ESC_MA0950AA	6113	257
ESC_MA0949AA	101	696
ESC_MA0948AA	58	198
ESC_MA0947AA	4429	820
ESC_MA0946AA	9133	198
ESC_MA0945AA	1167	257
ESC_MA0943AA	93	71
ESC_MA0941AA	8930	234
ESC_MA0940AA	44	512
ESC_MA0939AA	131	653
ESC_MA0938AA	167	512
ESC_MA0937AA	3541	842
ESC_MA0936AA	131	653
ESC_MA0935AA	617	511
ESC_MA0934AA	131	653
ESC_MA0933AA	46	447
ESC_MA0932AA	38	669
ESC_MA0931AA	617	511
ESC_MA0930AA	295	160
ESC_MA0929AA	2141	597
ESC_MA0928AA	46	447
ESC_MA0927AA	3018	903
ESC_MA0926AA	443	257
ESC_MA0925AA	773	71
ESC_MA0924AA	3268	669
ESC_MA0923AA	58	198
ESC_MA0922AA	617	511
ESC_MA0921AA	167	512
ESC_MA0920AA	940	257
ESC_MA0919AA	6359	473
ESC_MA0918AA	6359	473
ESC_MA0917AA	1706	156

ESC_MA0916AA	3268	669
ESC_MA0915AA	155	175
ESC_MA0914AA	443	257
ESC_MA0913AA	90	156
ESC_MA0912AA	131	653
ESC_MA0911AA	38	669
ESC_MA0910AA	648	359
ESC_MA0909AA	34	504
ESC_MA0908AA	4450	844
ESC_MA0907AA	167	512
ESC_MA0906AA	10	512
ESC_MA0905AA	10	512
ESC_MA0904AA	10	512
ESC_MA0903AA	10	512
ESC_MA0902AA	10	512
ESC_MA0901AA	10	512
ESC_MA0900AA	10	512
ESC_MA0899AA	10	512
ESC_MA0898AA	10	512
ESC_MA0897AA	10	512
ESC_MA0896AA	3541	842
ESC_MA0895AA	58	198
ESC_MA0894AA	3018	903
ESC_MA0893AA	38	669
ESC_MA0892AA	542	824
ESC_MA0891AA	10	512
ESC_MA0890AA	3541	842
ESC_MA0889AA	215	512
ESC_MA0888AA	38	669
ESC_MA0887AA	410	156
ESC_MA0886AA	10	512
ESC_MA0885AA	10	512
ESC_MA0884AA	10	512
ESC_MA0883AA	10	512
ESC_MA0882AA	10	512
ESC_MA0881AA	10	512
ESC_MA0880AA	10	512
ESC_MA0879AA	10	512
ESC_MA0878AA	10	512
ESC_MA0877AA	10	512
ESC_MA0876AA	10	512
ESC_MA0875AA	10	512
ESC_MA0874AA	202	311
ESC_MA0873AA	10	512
ESC_MA0872AA	10	512
ESC_MA0871AA	10	512
ESC_MA0870AA	10	512
ESC_MA0869AA	10	512
ESC_MA0868AA	10	512
ESC_MA0867AA	10	512

ESC_MA0866AA	10	512
ESC_MA0865AA	2602	257
ESC_MA0864AA	642	924
ESC_MA0863AA	6359	473
ESC_MA0862AA	48	76
ESC_MA0861AA	1060	512
ESC_MA0860AA	1060	512
ESC_MA0859AA	1060	512
ESC_MA0858AA	1060	512
ESC_MA0857AA	1060	512
ESC_MA0856AA	1431	257
ESC_MA0855AA	1060	512
ESC_MA0854AA	405	923
ESC_MA0853AA	131	653
ESC_MA0852AA	1060	512
ESC_MA0851AA	1060	512
ESC_MA0850AA	1060	512
ESC_MA0849AA	1060	512
ESC_MA0848AA	1060	512
ESC_MA0847AA	1060	512
ESC_MA0846AA	1060	512
ESC_MA0845AA	1060	512
ESC_MA0844AA	1060	512
ESC_MA0843AA	1060	512
ESC_MA0842AA	1060	512
ESC_MA0841AA	8356	198
ESC_MA0840AA	6880	726
ESC_MA0839AA	8931	122
ESC_MA0838AA	7403	304
ESC_MA0837AA	657	592
ESC_MA0836AA	6274	257
ESC_MA0835AA	25	234
ESC_MA0834AA	8695	257
ESC_MA0833AA	8695	257
ESC_MA0832AA	8695	257
ESC_MA0831AA	8696	286
ESC_MA0830AA	8694	205
ESC_MA0829AA	8695	257
ESC_MA0828AA	8693	156
ESC_MA0827AA	8929	286
ESC_MA0826AA	8929	286
ESC_MA0825AA	8929	286
ESC_MA0824AA	8930	234
ESC_MA0823AA	8929	286
ESC_MA0822AA	300	286
ESC_MA0821AA	300	286
ESC_MA0820AA	245	261
ESC_MA0819AA	245	261
ESC_MA0818AA	245	261
ESC_MA0817AA	152	257

ESC_MA0816AA	33	198
ESC_MA0767AA	40	198
ESC_MA0766AA	141	644
ESC_MA0765AA	17	191
ESC_MA0764AA	442	257
ESC_MA0763AA	442	257
ESC_MA0762AA	200	198
ESC_MA0761AA	200	198
ESC_MA0760AA	8919	198
ESC_MA0759AA	200	198
ESC_MA0758AA	301	307
ESC_MA0757AA	131	653
ESC_MA0756AA	38	669
ESC_MA0755AA	131	653
ESC_MA0754AA	127	738
ESC_MA0752AA	131	653
ESC_MA0751AA	75	257
ESC_MA0750AA	95	738
ESC_MA0749AA	131	653
ESC_MA0748AA	131	653
ESC_MA0747AA	12	626
ESC_MA0746AA	141	644
ESC_MA0745AA	131	653
ESC_MA0744AA	131	653
ESC_MA0743AA	95	738
ESC_MA0742AA	761	512
ESC_MA0741AA	131	653
ESC_MA0740AA	69	387
ESC_MA0738AA	1147	198
ESC_MA0737AA	131	653
ESC_MA0736AA	219	877
ESC_MA0735AA	131	653
ESC_MA0734AA	131	653
ESC_MA0733AA	131	653
ESC_MA0732AA	131	653
ESC_MA0731AA	38	669
ESC_MA0730AA	131	653
ESC_MA0729AA	131	653
ESC_MA0728AA	95	738
ESC_MA0725AA	131	653
ESC_MA0724AA	131	653
ESC_MA0723AA	69	387
ESC_MA0722AA	88	160
ESC_MA0721AA	38	669
ESC_MA0720AA	131	653
ESC_MA0719AA	141	644
ESC_MA0718AA	73	737
ESC_MA0717AA	1193	761
ESC_MA0716AA	131	653
ESC_MA0715AA	394	400

ESC_MA0714AA	131	653
ESC_MA0712AA	95	738
ESC_MA0711AA	6309	575
ESC_MA0710AA	372	424
ESC_MA0709AA	38	669
ESC_MA0708AA	404	761
ESC_MA0705AA	38	669
ESC_MA0704AA	636	653
ESC_MA0703AA	38	669
ESC_MA0702AA	1193	761
ESC_MA0701AA	38	669
ESC_MA0700AA	69	387
ESC_MA0699AA	117	592
ESC_MA0698AA	131	653
ESC_MA0697AA	131	653
ESC_MA0696AA	131	653
ESC_MA0695AA	131	653
ESC_MA0694AA	131	653
ESC_MA0693AA	16	156
ESC_MA0692AA	58	198
ESC_MA0691AA	8524	398
ESC_MA0690AA	2556	630
ESC_MA0689AA	58	198
ESC_MA0688AA	2728	198
ESC_MA0687AA	973	968
ESC_MA0686AA	2728	198
ESC_MA0685AA	8524	398
ESC_MA0684AA	58	198
ESC_MA0683AA	127	738
ESC_MA0682AA	9132	257
ESC_MA0681AA	2556	630
ESC_MA0680AA	297	234
ESC_MA0679AA	224	198
ESC_MA0678AA	2556	630
ESC_MA0677AA	648	359
ESC_MA0675AA	131	653
ESC_MA0674AA	131	653
ESC_MA0673AA	405	923
ESC_MA0672AA	405	923
ESC_MA0671AA	998	644
ESC_MA0670AA	10	512
ESC_MA0669AA	2599	257
ESC_MA0668AA	12	626
ESC_MA0667AA	6355	625
ESC_MA0666AA	12	626
ESC_MA0664AA	131	653
ESC_MA0663AA	131	653
ESC_MA0662AA	131	653
ESC_MA0661AA	131	653
ESC_MA0659AA	69	387

ESC_MA0658AA	38	669
ESC_MA0657AA	69	387
ESC_MA0656AA	95	738
ESC_MA0654AA	38	669
ESC_MA0653AA	95	738
ESC_MA0652AA	88	160
ESC_MA0651AA	405	923
ESC_MA0650AA	2020	387
ESC_MA0649AA	354	15
ESC_MA0648AA	131	653
ESC_MA0647AA	131	653
ESC_MA0646AA	405	923
ESC_MA0645AA	73	737
ESC_MA0644AA	648	359
ESC_MA0643AA	131	653
ESC_MA0642AA	131	653
ESC_MA0641AA	69	387
ESC_MA0640AA	131	653
ESC_MA0639AA	73	737
ESC_MA0638AA	10	512
ESC_MA0637AA	131	653
ESC_MA0636AA	131	653
ESC_MA0635AA	131	653
ESC_MA0634AA	8915	574
ESC_MA0633AA	131	653
ESC_MA0632AA	38	669
ESC_MA0631AA	1421	447
ESC_MA0630AA	73	737
ESC_MA0629AA	95	738
ESC_MA0628AA	131	653
ESC_MA0627AA	1722	599
ESC_MA0626AA	38	669
ESC_MA0625AA	38	669
ESC_MA0624AA	131	653
ESC_MA0623AA	117	592
ESC_MA0622AA	4363	632
ESC_MA0621AA	152	257
ESC_MA0620AA	245	261
ESC_MA0619AA	245	261
ESC_MA0618AA	1025	261
ESC_MA0617AA	152	257
ESC_MA0616AA	152	257
ESC_MA0615AA	245	261
ESC_MA0614AA	11	936
ESC_MA0613AA	11	936
ESC_MA0612AA	10	512
ESC_MA0611AA	9131	160
ESC_MA0610AA	11	936
ESC_MA0609AA	9130	587
ESC_MA0608AA	11	936

ESC_MA0607AA	1804	935
ESC_MA0606AA	11	936
ESC_MA0605AA	11	936
ESC_MA0604AA	11	936
ESC_MA0603AA	329	355
ESC_MA0602AA	21	156
ESC_MA0601AA	21	156
ESC_MA0600AA	29	156
ESC_MA0599AA	131	653
ESC_MA0598AA	73	737
ESC_MA0597AA	538	629
ESC_MA0596AA	69	387
ESC_MA0595AA	636	653
ESC_MA0594AA	14	761
ESC_MA0593AA	10	512
ESC_MA0592AA	69	387
ESC_MA0591AA	131	653
ESC_MA0590AA	131	653
ESC_MA0589AA	405	923
ESC_MA0588AA	38	669
ESC_MA0586AA	131	653
ESC_MA0585AA	131	653
ESC_MA0584AA	1312	76
ESC_MA0583AA	73	737
ESC_MA0582AA	131	653
ESC_MA0581AA	1193	761
ESC_MA0580AA	131	653
ESC_MA0579AA	131	653
ESC_MA0578AA	131	653
ESC_MA0575AA	95	738
ESC_MA0574AA	131	653
ESC_MA0571AA	1193	761
ESC_MA0569AA	131	653
ESC_MA0568AA	131	653
ESC_MA0567AA	131	653
ESC_MA0566AA	69	387
ESC_MA0565AA	131	653
ESC_MA0563AA	12	626
ESC_MA0562AA	1193	761
ESC_MA0561AA	131	653
ESC_MA0559AA	69	387
ESC_MA0558AA	38	669
ESC_MA0557AA	69	387
ESC_MA0556AA	453	935
ESC_MA0555AA	131	653
ESC_MA0553AA	131	653
ESC_MA0552AA	57	50
ESC_MA0550AA	152	257
ESC_MA0549AA	152	257
ESC_MA0548AA	245	261

ESC_MA0547AA	245	261
ESC_MA0546AA	152	257
ESC_MA0545AA	617	511
ESC_MA0544AA	73	737
ESC_MA0543AA	9111	387
ESC_MA0542AA	73	737
ESC_MA0541AA	127	738
ESC_MA0540AA	131	653
ESC_MA0539AA	491	626
ESC_MA0538AA	59	217
ESC_MA0537AA	95	738
ESC_MA0536AA	131	653
ESC_MA0535AA	144	740
ESC_MA0534AA	59	217
ESC_MA0533AA	428	654
ESC_MA0532AA	405	923
ESC_MA0531AA	73	737
ESC_MA0530AA	141	644
ESC_MA0529AA	73	737
ESC_MA0528AA	131	653
ESC_MA0527AA	80	731
ESC_MA0526AA	69	387
ESC_MA0525AA	95	738
ESC_MA0524AA	1193	761
ESC_MA0523AA	95	738
ESC_MA0522AA	617	511
ESC_MA0521AA	90	156
ESC_MA0520AA	131	653
ESC_MA0519AA	420	741
ESC_MA0518AA	69	387
ESC_MA0517AA	1193	761
ESC_MA0516AA	69	387
ESC_MA0515AA	73	737
ESC_MA0514AA	5022	387
ESC_MA0513AA	131	653
ESC_MA0512AA	131	653
ESC_MA0511AA	73	737
ESC_MA0510AA	69	387
ESC_MA0509AA	127	738
ESC_MA0508AA	73	737
ESC_MA0507AA	7893	198
ESC_MA0506AA	9113	892
ESC_MA0505AA	73	737
ESC_MA0504AA	95	738
ESC_MA0503AA	491	626
ESC_MA0502AA	73	737
ESC_MA0501AA	69	387
ESC_MA0500AA	69	387
ESC_MA0499AA	93	71
ESC_MA0498AA	131	653

ESC_MA0497AA	131	653
ESC_MA0496AA	73	737
ESC_MA0495AA	127	738
ESC_MA0494AA	131	653
ESC_MA0493AA	405	923
ESC_MA0492AA	131	653
ESC_MA0491AA	95	738
ESC_MA0490AA	405	923
ESC_MA0489AA	73	737
ESC_MA0488AA	73	737
ESC_MA0487AA	14	761
ESC_MA0486AA	95	738
ESC_MA0485AA	73	737
ESC_MA0484AA	73	737
ESC_MA0483AA	1086	175
ESC_MA0482AA	131	653
ESC_MA0481AA	1193	761
ESC_MA0480AA	404	761
ESC_MA0479AA	405	923
ESC_MA0478AA	10	512
ESC_MA0477AA	38	669
ESC_MA0476AA	131	653
ESC_MA0475AA	95	738
ESC_MA0474AA	73	737
ESC_MA0473AA	405	923
ESC_MA0472AA	349	670
ESC_MA0471AA	131	653
ESC_MA0470AA	1193	761
ESC_MA0469AA	12	626
ESC_MA0468AA	345	175
ESC_MA0467AA	12	626
ESC_MA0466AA	73	737
ESC_MA0465AA	95	738
ESC_MA0464AA	131	653
ESC_MA0463AA	73	737
ESC_MA0462AA	95	738
ESC_MA0461AA	1193	761
ESC_MA0460AA	131	653
ESC_MA0459AA	127	738
ESC_MA0458AA	131	653
ESC_MA0457AA	1193	761
ESC_MA0456AA	131	653
ESC_MA0455AA	1161	764
ESC_MA0454AA	9116	546
ESC_MA0453AA	131	653
ESC_MA0452AA	95	738
ESC_MA0451AA	73	737
ESC_MA0450AA	1231	738
ESC_MA0449AA	69	387
ESC_MA0448AA	93	71

ESC_MA0447AA	929	742
ESC_MA0446AA	73	737
ESC_MA0445AA	69	387
ESC_MA0444AA	1193	761
ESC_MA0443AA	62	574
ESC_MA0442AA	648	359
ESC_MA0441AA	538	629
ESC_MA0440AA	127	738
ESC_MA0439AA	93	71
ESC_MA0438AA	12	626
ESC_MA0437AA	131	653
ESC_MA0436AA	6975	211
ESC_MA0435AA	547	653
ESC_MA0434AA	73	737
ESC_MA0433AA	95	738
ESC_MA0432AA	73	737
ESC_MA0431AA	131	653
ESC_MA0430AA	648	359
ESC_MA0429AA	297	234
ESC_MA0428AA	131	653
ESC_MA0427AA	95	738
ESC_MA0426AA	95	738
ESC_MA0425AA	93	71
ESC_MA0424AA	131	653
ESC_MA0423AA	69	387
ESC_MA0422AA	62	574
ESC_MA0421AA	73	737
ESC_MA0420AA	131	653
ESC_MA0419AA	9123	626
ESC_MA0418AA	919	421
ESC_MA0417AA	38	669
ESC_MA0416AA	127	738
ESC_MA0415AA	95	738
ESC_MA0414AA	95	738
ESC_MA0413AA	9121	737
ESC_MA0412AA	131	653
ESC_MA0411AA	1193	761
ESC_MA0410AA	73	737
ESC_MA0409AA	363	738
ESC_MA0408AA	73	737
ESC_MA0407AA	58	198
ESC_MA0406AA	95	738
ESC_MA0405AA	131	653
ESC_MA0404AA	73	737
ESC_MA0403AA	9122	28
ESC_MA0402AA	140	738
ESC_MA0401AA	131	653
ESC_MA0400AA	95	738
ESC_MA0399AA	135	653
ESC_MA0398AA	95	738

ESC_MA0397AA	62	574
ESC_MA0396AA	131	653
ESC_MA0395AA	95	738
ESC_MA0394AA	5022	387
ESC_MA0393AA	131	653
ESC_MA0392AA	131	653
ESC_MA0391AA	95	738
ESC_MA0390AA	73	737
ESC_MA0389AA	62	574
ESC_MA0388AA	393	343
ESC_MA0387AA	73	737
ESC_MA0386AA	12	626
ESC_MA0385AA	12	626
ESC_MA0384AA	73	737
ESC_MA0383AA	9126	653
ESC_MA0382AA	95	738
ESC_MA0381AA	131	653
ESC_MA0380AA	131	653
ESC_MA0379AA	95	738
ESC_MA0378AA	73	737
ESC_MA0377AA	38	669
ESC_MA0376AA	393	343
ESC_MA0375AA	131	653
ESC_MA0374AA	28	537
ESC_MA0373AA	73	737
ESC_MA0372AA	69	387
ESC_MA0371AA	69	387
ESC_MA0370AA	349	670
ESC_MA0369AA	69	387
ESC_MA0368AA	607	355
ESC_MA0367AA	38	669
ESC_MA0366AA	73	737
ESC_MA0365AA	393	343
ESC_MA0364AA	73	737
ESC_MA0363AA	135	653
ESC_MA0362AA	1284	604
ESC_MA0361AA	144	740
ESC_MA0360AA	95	738
ESC_MA0359AA	131	653
ESC_MA0358AA	73	737
ESC_MA0357AA	131	653
ESC_MA0356AA	648	359
ESC_MA0355AA	95	738
ESC_MA0354AA	404	761
ESC_MA0353AA	12	626
ESC_MA0352AA	1385	653
ESC_MA0351AA	131	653
ESC_MA0350AA	998	644
ESC_MA0349AA	1598	447
ESC_MA0348AA	491	626

ESC_MA0347AA	73	737
ESC_MA0346AA	131	653
ESC_MA0345AA	38	669
ESC_MA0344AA	73	737
ESC_MA0343AA	399	198
ESC_MA0342AA	59	217
ESC_MA0341AA	1193	761
ESC_MA0340AA	421	744
ESC_MA0339AA	131	653
ESC_MA0338AA	1193	761
ESC_MA0337AA	537	756
ESC_MA0336AA	95	738
ESC_MA0335AA	131	653
ESC_MA0334AA	393	343
ESC_MA0333AA	1844	198
ESC_MA0332AA	131	653
ESC_MA0331AA	10	512
ESC_MA0330AA	73	737
ESC_MA0329AA	95	738
ESC_MA0328AA	1057	761
ESC_MA0327AA	410	156
ESC_MA0326AA	95	738
ESC_MA0325AA	131	653
ESC_MA0324AA	131	653
ESC_MA0323AA	131	653
ESC_MA0322AA	127	738
ESC_MA0321AA	73	737
ESC_MA0320AA	131	653
ESC_MA0319AA	95	738
ESC_MA0318AA	95	738
ESC_MA0317AA	95	738
ESC_MA0316AA	144	740
ESC_MA0315AA	131	653
ESC_MA0314AA	91	629
ESC_MA0313AA	131	653
ESC_MA0312AA	131	653
ESC_MA0311AA	1236	380
ESC_MA0310AA	69	387
ESC_MA0309AA	69	387
ESC_MA0308AA	73	737
ESC_MA0307AA	93	71
ESC_MA0306AA	131	653
ESC_MA0305AA	131	653
ESC_MA0304AA	131	653
ESC_MA0303AA	1284	604
ESC_MA0302AA	95	738
ESC_MA0301AA	73	737
ESC_MA0300AA	131	653
ESC_MA0299AA	10	512
ESC_MA0298AA	95	738

ESC_MA0297AA	131	653
ESC_MA0296AA	131	653
ESC_MA0295AA	95	738
ESC_MA0294AA	131	653
ESC_MA0293AA	73	737
ESC_MA0292AA	131	653
ESC_MA0291AA	131	653
ESC_MA0290AA	1177	669
ESC_MA0289AA	95	738
ESC_MA0288AA	95	738
ESC_MA0287AA	1193	761
ESC_MA0286AA	1623	891
ESC_MA0285AA	131	653
ESC_MA0284AA	135	653
ESC_MA0283AA	46	447
ESC_MA0282AA	357	654
ESC_MA0281AA	131	653
ESC_MA0280AA	12	626
ESC_MA0279AA	131	653
ESC_MA0278AA	95	738
ESC_MA0277AA	73	737
ESC_MA0276AA	226	512
ESC_MA0275AA	131	653
ESC_MA0274AA	131	653
ESC_MA0273AA	131	653
ESC_MA0272AA	52	71
ESC_MA0271AA	10	512
ESC_MA0270AA	73	737
ESC_MA0269AA	131	653
ESC_MA0268AA	95	738
ESC_MA0267AA	95	738
ESC_MA0266AA	73	737
ESC_MA0265AA	131	653
ESC_MA0264AA	73	737
ESC_MA0263AA	141	644
ESC_MA0262AA	1284	604
ESC_MA0261AA	9127	387
ESC_MA0260AA	73	737
ESC_MA0259AA	1193	761
ESC_MA0258AA	409	504
ESC_MA0257AA	131	653
ESC_MA0256AA	73	737
ESC_MA0255AA	131	653
ESC_MA0254AA	73	737
ESC_MA0253AA	676	737
ESC_MA0252AA	131	653
ESC_MA0251AA	131	653
ESC_MA0250AA	93	71
ESC_MA0249AA	131	653
ESC_MA0248AA	95	738

ESC_MA0247AA	95	738
ESC_MA0246AA	131	653
ESC_MA0245AA	602	257
ESC_MA0244AA	144	740
ESC_MA0243AA	68	386
ESC_MA0242AA	131	653
ESC_MA0241AA	131	653
ESC_MA0240AA	9128	900
ESC_MA0239AA	3045	670
ESC_MA0238AA	131	653
ESC_MA0237AA	127	738
ESC_MA0236AA	405	923
ESC_MA0235AA	648	359
ESC_MA0234AA	58	198
ESC_MA0233AA	131	653
ESC_MA0232AA	537	756
ESC_MA0231AA	10	512
ESC_MA0230AA	95	738
ESC_MA0229AA	73	737
ESC_MA0228AA	95	738
ESC_MA0227AA	73	737
ESC_MA0226AA	5969	85
ESC_MA0225AA	127	738
ESC_MA0224AA	428	654
ESC_MA0223AA	69	387
ESC_MA0222AA	73	737
ESC_MA0221AA	3672	649
ESC_MA0220AA	95	738
ESC_MA0219AA	393	343
ESC_MA0218AA	1415	504
ESC_MA0217AA	405	923
ESC_MA0216AA	416	738
ESC_MA0215AA	131	653
ESC_MA0214AA	127	738
ESC_MA0213AA	131	653
ESC_MA0212AA	69	387
ESC_MA0211AA	95	738
ESC_MA0210AA	9129	968
ESC_MA0209AA	14	761
ESC_MA0208AA	131	653
ESC_MA0207AA	38	669
ESC_MA0206AA	127	738
ESC_MA0205AA	1421	447
ESC_MA0204AA	73	737
ESC_MA0203AA	131	653
ESC_MA0202AA	405	923
ESC_MA0201AA	404	761
ESC_MA0200AA	349	670
ESC_MA0199AA	127	738
ESC_MA0198AA	73	737

ESC_MA0197AA	127	738
ESC_MA0196AA	127	738
ESC_MA0195AA	648	359
ESC_MA0194AA	131	653
ESC_MA0193AA	95	738
ESC_MA0192AA	69	387
ESC_MA0191AA	95	738
ESC_MA0190AA	648	359
ESC_MA0189AA	95	738
ESC_MA0188AA	38	669
ESC_MA0187AA	131	653
ESC_MA0186AA	73	737
ESC_MA0185AA	1193	761
ESC_MA0184AA	69	387
ESC_MA0183AA	1262	737
ESC_MA0182AA	95	738
ESC_MA0181AA	95	738
ESC_MA0180AA	131	653
ESC_MA0179AA	127	738
ESC_MA0178AA	4260	737
ESC_MA0177AA	95	738
ESC_MA0176AA	131	653
ESC_MA0175AA	46	447
ESC_MA0174AA	420	741
ESC_MA0173AA	409	504
ESC_MA0172AA	349	670
ESC_MA0171AA	131	653
ESC_MA0170AA	73	737
ESC_MA0169AA	1882	387
ESC_MA0168AA	95	738
ESC_MA0167AA	69	387
ESC_MA0166AA	95	738
ESC_MA0164AA	59	217
ESC_MA0163AA	8590	212
ESC_MA0162AA	998	644
ESC_MA0161AA	73	737
ESC_MA0160AA	95	738
ESC_MA0159AA	59	217
ESC_MA0158AA	69	387
ESC_MA0157AA	95	738
ESC_MA0156AA	10	512
ESC_MA0155AA	2013	725
ESC_MA0154AA	68	386
ESC_MA0153AA	405	923
ESC_MA0152AA	2552	653
ESC_MA0151AA	131	653
ESC_MA0150AA	131	653
ESC_MA0149AA	404	761
ESC_MA0148AA	95	738
ESC_MA0146AA	12	626

ESC_MA0145AA	1431	257
ESC_MA0144AA	95	738
ESC_MA0143AA	131	653
ESC_MA0142AA	4554	738
ESC_MA0141AA	73	737
ESC_MA0140AA	69	387
ESC_MA0139AA	7428	924
ESC_MA0138AA	95	738
ESC_MA0137AA	131	653
ESC_MA0136AA	73	737
ESC_MA0135AA	69	387
ESC_MA0134AA	131	653
ESC_MA0133AA	95	738
ESC_MA0132AA	1193	761
ESC_MA0127AA	361	512
ESC_MA0126AA	46	447
ESC_MA0125AA	48	76
ESC_MA0124AA	361	512
ESC_MA0123AA	46	447
ESC_MA0122AA	8881	362
ESC_MA0121AA	46	447
ESC_MA0120AA	361	512
ESC_MA0117AA	1303	791
ESC_MA0115AA	1585	512
ESC_MA0016AA	131	653
ESC_MA0015AA	58	198
ESC_MA0014AA	206	286
ESC_MA0013AA	1146	198
ESC_MA0012AA	8168	1004
ESC_MA0011AA	48	76
ESC_MA0010AA	9101	512
ESC_MA0009AA	1146	198
ESC_MA0008AA	187	257
ESC_MA0007AA	723	156
ESC_MA0006AA	155	175
ESC_MA0005AA	1122	307
ESC_MA0004AA	3580	257
ESC_MA0003AA	10	512
ESC_MA0002AA	3234	257
ESC_MA0001AA	93	71
ESC_MA0000AA	187	257
ESC_LA9999AA	4481	257
ESC_LA9998AA	117	592
ESC_LA9997AA	540	286
ESC_LA9996AA	3258	592
ESC_LA9995AA	540	286
ESC_LA9994AA	58	198
ESC_LA9993AA	58	198
ESC_LA9992AA	101	696
ESC_LA9991AA	117	592

ESC_LA9990AA	2008	257
ESC_LA9989AA	5082	198
ESC_LA9988AA	10	512
ESC_LA9987AA	46	447
ESC_LA9986AA	752	512
ESC_LA9985AA	58	198
ESC_LA9984AA	793	286
ESC_LA9983AA	58	198
ESC_LA9982AA	88	160
ESC_LA9981AA	58	198
ESC_LA9980AA	189	504
ESC_LA9979AA	501	900
ESC_LA9978AA	1727	257
ESC_LA9977AA	5897	912
ESC_LA9976AA	752	512
ESC_LA9975AA	34	504
ESC_LA9974AA	361	512
ESC_LA9973AA	2025	194
ESC_LA9972AA	58	198
ESC_LA9971AA	46	447
ESC_LA9970AA	9112	160
ESC_LA9969AA	131	653
ESC_LA9968AA	38	669
ESC_LA9967AA	131	653
ESC_LA9966AA	69	387
ESC_LA9965AA	973	968
ESC_LA9964AA	131	653
ESC_LA9963AA	14	761
ESC_LA9962AA	131	653
ESC_LA9961AA	38	669
ESC_LA9960AA	152	257
ESC_LA9959AA	93	71
ESC_LA9958AA	93	71
ESC_LA9913AA	205	257
ESC_LA9912AA	156	175
ESC_LA9911AA	973	968
ESC_LA9910AA	38	669
ESC_LA9909AA	131	653
ESC_LA9908AA	131	653
ESC_LA9907AA	131	653
ESC_LA9906AA	131	653
ESC_LA9905AA	131	653
ESC_LA9904AA	131	653
ESC_LA9902AA	569	738
ESC_LA9901AA	131	653
ESC_LA9900AA	131	653
ESC_LA9899AA	245	261
ESC_LA9898AA	504	738
ESC_LA9897AA	16	156
ESC_LA9896AA	6175	257

ESC_LA9895AA	32	270
ESC_LA9893AA	448	257
ESC_LA9892AA	2450	447
ESC_LA9891AA	90	156
ESC_LA9890AA	405	923
ESC_LA9889AA	1585	512
ESC_LA9888AA	167	512
ESC_LA9887AA	405	923
ESC_LA9886AA	405	923
ESC_LA9885AA	405	923
ESC_LA9884AA	405	923
ESC_LA9883AA	405	923
ESC_LA9882AA	6870	603
ESC_LA9881AA	167	512
ESC_LA9880AA	636	653
ESC_LA9879AA	405	923
ESC_LA9878AA	405	923
ESC_LA9876AA	448	257
ESC_LA9875AA	405	923
ESC_LA9874AA	371	50
ESC_LA9872AA	167	512
ESC_LA9871AA	1060	512
ESC_LA9869AA	1060	512
ESC_LA9868AA	1060	512
ESC_LA9867AA	1060	512
ESC_LA9866AA	1060	512
ESC_LA9865AA	1060	512
ESC_LA9864AA	1060	512
ESC_LA9863AA	1060	512
ESC_LA9862AA	1060	512
ESC_LA9861AA	1060	512
ESC_LA9859AA	1060	512
ESC_LA9857AA	1060	512
ESC_LA9856AA	1060	512
ESC_LA9855AA	1060	512
ESC_LA9854AA	1060	512
ESC_LA9853AA	1060	512
ESC_LA9852AA	1060	512
ESC_LA9851AA	1060	512
ESC_LA9850AA	1060	512
ESC_LA9848AA	1060	512
ESC_LA9847AA	1060	512
ESC_LA9846AA	1060	512
ESC_LA9845AA	1060	512
ESC_LA9843AA	1060	512
ESC_LA9841AA	1060	512
ESC_LA9840AA	1060	512
ESC_LA9839AA	1060	512
ESC_LA9838AA	1060	512
ESC_LA9837AA	1060	512

ESC_LA9836AA	1060	512
ESC_LA9835AA	1060	512
ESC_LA9833AA	1060	512
ESC_LA9827AA	141	644
ESC_LA9826AA	131	653
ESC_LA9825AA	2161	198
ESC_LA9824AA	1967	191
ESC_LA9823AA	16	156
ESC_LA9822AA	349	670
ESC_LA9821AA	2325	447
ESC_LA9820AA	155	175
ESC_LA9819AA	10	512
ESC_LA9818AA	9071	968
ESC_LA9817AA	9070	175
ESC_LA9816AA	1	667
ESC_LA9815AA	939	198
ESC_LA9814AA	9070	175
ESC_LA9813AA	939	198
ESC_LA9812AA	10	512
ESC_LA9811AA	388	156
ESC_LA9810AA	10	512
ESC_LA9808AA	21	156
ESC_LA9807AA	58	198
ESC_LA9806AA	1656	175
ESC_LA9805AA	1	667
ESC_LA9804AA	73	737
ESC_LA9803AA	9073	156
ESC_LA9802AA	29	156
ESC_LA9801AA	21	156
ESC_LA9800AA	21	156
ESC_LA9799AA	21	156
ESC_LA9798AA	129	257
ESC_LA9797AA	1	667
ESC_LA9796AA	10	512
ESC_LA9795AA	961	623
ESC_LA9794AA	1	667
ESC_LA9793AA	2230	126
ESC_LA9792AA	1	667
ESC_LA9791AA	9072	156
ESC_LA9790AA	906	198
ESC_LA9789AA	388	156
ESC_LA9788AA	3246	900
ESC_LA9787AA	540	286
ESC_LA9786AA	155	175
ESC_LA9785AA	10	512
ESC_LA9784AA	95	738
ESC_LA9783AA	10	512
ESC_LA9782AA	1727	257
ESC_LA9781AA	2559	373
ESC_LA9780AA	906	198

ESC_LA9779AA	154	257
ESC_LA9778AA	297	234
ESC_LA9777AA	8184	653
ESC_LA9776AA	937	198
ESC_LA9775AA	2178	924
ESC_LA9774AA	54	256
ESC_LA9773AA	6015	234
ESC_LA9772AA	906	198
ESC_LA9771AA	1125	198
ESC_LA9770AA	9086	198
ESC_LA9769AA	162	924
ESC_LA9768AA	101	696
ESC_LA9767AA	6236	856
ESC_LA9766AA	9084	198
ESC_LA9765AA	2354	257
ESC_LA9764AA	2354	257
ESC_LA9763AA	9089	160
ESC_LA9762AA	154	257
ESC_LA9761AA	297	234
ESC_LA9760AA	28	537
ESC_LA9756AA	245	261
ESC_LA9755AA	152	257
ESC_LA9754AA	152	257
ESC_LA9753AA	152	257
ESC_LA9752AA	152	257
ESC_LA9751AA	245	261
ESC_LA9750AA	152	257
ESC_LA9749AA	152	257
ESC_LA9748AA	245	261
ESC_LA9747AA	245	261
ESC_LA9746AA	245	261
ESC_LA9745AA	245	261
ESC_LA9744AA	152	257
ESC_LA9743AA	3695	924
ESC_LA9742AA	16	156
ESC_LA9741AA	738	598
ESC_LA9740AA	33	198
ESC_LA9739AA	73	737
ESC_LA9704AA	6	445
ESC_LA9701AA	101	696
ESC_LA9700AA	167	512
ESC_LA9699AA	423	204
ESC_LA9698AA	173	254
ESC_LA9697AA	443	257
ESC_LA9696AA	173	254
ESC_LA9695AA	173	254
ESC_LA9694AA	173	254
ESC_LA9693AA	173	254
ESC_LA9692AA	88	160
ESC_LA9691AA	443	257

ESC_LA9690AA	88	160
ESC_LA9689AA	88	160
ESC_LA9687AA	5305	404
ESC_LA9686AA	5344	257
ESC_LA9685AA	5305	404
ESC_LA9684AA	88	160
ESC_LA9683AA	443	257
ESC_LA9682AA	5305	404
ESC_LA9681AA	5305	404
ESC_LA9680AA	449	387
ESC_LA9678AA	5305	404
ESC_LA9677AA	443	257
ESC_LA9676AA	423	204
ESC_LA9675AA	88	160
ESC_LA9674AA	443	257
ESC_LA9673AA	173	254
ESC_LA9672AA	173	254
ESC_LA9671AA	443	257
ESC_LA9670AA	88	160
ESC_LA9668AA	443	257
ESC_LA9667AA	88	160
ESC_LA9666AA	173	254
ESC_LA9665AA	173	254
ESC_LA9664AA	10	512
ESC_LA9663AA	5344	257
ESC_LA9662AA	443	257
ESC_LA9661AA	5344	257
ESC_LA9660AA	423	204
ESC_LA9659AA	443	257
ESC_LA9658AA	3910	672
ESC_LA9657AA	443	257
ESC_LA9656AA	5305	404
ESC_LA9655AA	173	254
ESC_LA9654AA	173	254
ESC_LA9653AA	88	160
ESC_LA9651AA	88	160
ESC_LA9650AA	5305	404
ESC_LA9648AA	5305	404
ESC_LA9647AA	5305	404
ESC_LA9646AA	5305	404
ESC_LA9645AA	443	257
ESC_LA9644AA	127	738
ESC_LA9643AA	131	653
ESC_LA9642AA	73	737
ESC_LA9641AA	372	424
ESC_LA9640AA	3601	653
ESC_LA9639AA	372	424
ESC_LA9638AA	372	424
ESC_LA9637AA	127	738
ESC_LA9636AA	372	424

ESC_LA9635AA	11	936
ESC_LA9634AA	11	936
ESC_LA9633AA	152	257
ESC_LA9632AA	152	257
ESC_LA9631AA	8882	761
ESC_LA9628AA	9765	737
ESC_LA9625AA	2178	924
ESC_LA9624AA	17	191
ESC_LA9623AA	11	936
ESC_LA9622AA	11	936
ESC_LA9621AA	11	936
ESC_LA9620AA	11	936
ESC_LA9619AA	11	936
ESC_LA9618AA	11	936
ESC_LA9617AA	11	936
ESC_LA9616AA	11	936
ESC_LA9615AA	301	307
ESC_LA9614AA	301	307
ESC_LA9613AA	21	156
ESC_LA9612AA	152	257
ESC_LA9611AA	152	257
ESC_LA9609AA	8881	362
ESC_LA9608AA	95	738
ESC_LA9607AA	1992	257
ESC_LA9606AA	515	1004
ESC_LA9605AA	9062	512
ESC_LA9604AA	2111	184
ESC_LA9603AA	8359	156
ESC_LA9602AA	7207	960
ESC_LA9601AA	56	198
ESC_LA9600AA	117	592
ESC_LA9599AA	10	512
ESC_LA9598AA	7093	868
ESC_LA9597AA	117	592
ESC_LA9596AA	410	156
ESC_LA9595AA	1308	257
ESC_LA9594AA	3381	268
ESC_LA9593AA	117	592
ESC_LA9592AA	32	270
ESC_LA9591AA	278	924
ESC_LA9590AA	2111	184
ESC_LA9589AA	2111	184
ESC_LA9588AA	160	257
ESC_LA9587AA	10	512
ESC_LA9586AA	9135	257
ESC_LA9585AA	34	504
ESC_LA9582AA	17	191
ESC_LA9581AA	17	191
ESC_LA9580AA	20	198
ESC_LA9579AA	17	191

ESC_LA9578AA	17	191
ESC_LA9577AA	20	198
ESC_LA9576AA	17	191
ESC_LA9575AA	17	191
ESC_LA9574AA	386	191
ESC_LA9573AA	17	191
ESC_LA9572AA	17	191
ESC_LA9571AA	17	191
ESC_LA9570AA	32	270
ESC_LA9569AA	11	936
ESC_LA9568AA	200	198
ESC_LA9567AA	200	198
ESC_LA9566AA	335	936
ESC_LA9565AA	33	198
ESC_LA9564AA	4748	234
ESC_LA9563AA	442	257
ESC_LA9562AA	8880	900
ESC_LA9561AA	795	924
ESC_LA9560AA	1892	846
ESC_LA9559AA	16	156
ESC_LA9558AA	655	926
ESC_LA9557AA	301	307
ESC_LA9556AA	8879	838
ESC_LA9555AA	58	198
ESC_LA9554AA	349	670
ESC_LA9553AA	73	737
ESC_LA9552AA	73	737
ESC_LA9551AA	681	393
ESC_LA9550AA	245	261
ESC_LA9549AA	245	261
ESC_LA9548AA	245	261
ESC_LA9547AA	245	261
ESC_LA9546AA	245	261
ESC_LA9545AA	10	512
ESC_LA9544AA	245	261
ESC_LA9543AA	628	261
ESC_LA9542AA	245	261
ESC_LA9541AA	245	261
ESC_LA9540AA	6564	45
ESC_LA9539AA	2248	257
ESC_LA9538AA	2728	198
ESC_LA9537AA	127	738
ESC_LA9536AA	428	654
ESC_LA9535AA	127	738
ESC_LA9534AA	345	175
ESC_LA9533AA	2728	198
ESC_LA9532AA	6564	45
ESC_LA9531AA	117	592
ESC_LA9530AA	117	592
ESC_LA9529AA	155	175

ESC_LA9528AA	155	175
ESC_LA9527AA	117	592
ESC_LA9526AA	3580	257
ESC_LA9525AA	348	175
ESC_LA9524AA	3258	592
ESC_LA9523AA	3258	592
ESC_LA9522AA	152	257
ESC_LA9521AA	152	257
ESC_LA9520AA	152	257
ESC_LA9519AA	152	257
ESC_LA9518AA	33	198
ESC_LA9517AA	8878	261
ESC_LA9514AA	8877	842
ESC_LA9513AA	569	738
ESC_LA9512AA	154	257
ESC_LA9511AA	641	935
ESC_LA9510AA	2163	257
ESC_LA9509AA	11	936
ESC_LA9508AA	11	936
ESC_LA9507AA	9061	923
ESC_LA9506AA	648	359
ESC_LA9505AA	28	537
ESC_LA9504AA	38	669
ESC_LA9503AA	38	669
ESC_LA9501AA	152	257
ESC_LA9500AA	152	257
ESC_LA9499AA	245	261
ESC_LA9498AA	245	261
ESC_LA9497AA	152	257
ESC_LA9496AA	245	261
ESC_LA9495AA	152	257
ESC_LA9494AA	245	261
ESC_LA9493AA	152	257
ESC_LA9492AA	152	257
ESC_LA9491AA	245	261
ESC_LA9490AA	442	257
ESC_LA9489AA	4060	744
ESC_LA9488AA	216	512
ESC_LA9487AA	517	924
ESC_LA9486AA	329	355
ESC_LA9484AA	58	198
ESC_LA9480AA	4198	878
ESC_LA9479AA	58	198
ESC_LA9478AA	17	191
ESC_LA9477AA	93	71
ESC_LA9476AA	90	156
ESC_LA9475AA	9057	76
ESC_LA9474AA	117	592
ESC_LA9473AA	708	924
ESC_LA9472AA	10	512

ESC_LA9470AA	90	156
ESC_LA9469AA	10	512
ESC_LA9468AA	501	900
ESC_LA9467AA	350	50
ESC_LA9466AA	9059	519
ESC_LA9465AA	9056	504
ESC_LA9463AA	10	512
ESC_LA9462AA	206	286
ESC_LA9461AA	641	935
ESC_LA9460AA	1718	269
ESC_LA9459AA	973	968
ESC_LA9458AA	13	257
ESC_LA9457AA	95	738
ESC_LA9456AA	90	156
ESC_LA9455AA	23	156
ESC_LA9454AA	46	447
ESC_LA9453AA	1706	156
ESC_LA9452AA	398	445
ESC_LA9451AA	90	156
ESC_LA9450AA	429	654
ESC_LA9449AA	23	156
ESC_LA9448AA	9058	171
ESC_LA9447AA	206	286
ESC_LA9446AA	761	512
ESC_LA9445AA	90	156
ESC_LA9444AA	8433	512
ESC_LA9443AA	90	156
ESC_LA9442AA	763	878
ESC_LA9441AA	57	50
ESC_LA9440AA	90	156
ESC_LA9439AA	5086	286
ESC_LA9438AA	910	929
ESC_LA9437AA	8009	731
ESC_LA9436AA	1684	504
ESC_LA9435AA	206	286
ESC_LA9434AA	10	512
ESC_LA9433AA	11	936
ESC_LA9432AA	665	79
ESC_LA9431AA	648	359
ESC_LA9430AA	10	512
ESC_LA9429AA	542	824
ESC_LA9427AA	665	79
ESC_LA9426AA	9058	171
ESC_LA9425AA	1706	156
ESC_LA9424AA	636	653
ESC_LA9422AA	636	653
ESC_LA9420AA	636	653
ESC_LA9419AA	636	653
ESC_LA9418AA	245	261
ESC_LA9417AA	245	261

ESC_LA9416AA	245	261
ESC_LA9415AA	152	257
ESC_LA9414AA	245	261
ESC_LA9413AA	504	738
ESC_LA9412AA	11	936
ESC_LA9411AA	11	936
ESC_LA9410AA	34	504
ESC_LA9409AA	656	789
ESC_LA9408AA	1249	257
ESC_LA9407AA	1249	257
ESC_LA9406AA	362	234
ESC_LA9404AA	648	359
ESC_LA9403AA	2005	175
ESC_LA9401AA	26	257
ESC_LA9400AA	26	257
ESC_LA9398AA	677	257
ESC_LA9397AA	10	512
ESC_LA9396AA	656	789
ESC_LA9395AA	1161	764
ESC_LA9394AA	847	257
ESC_LA9393AA	3236	212
ESC_LA9392AA	3188	257
ESC_LA9391AA	1249	257
ESC_LA9388AA	245	261
ESC_LA9387AA	152	257
ESC_LA9386AA	6666	261
ESC_LA9385AA	245	261
ESC_LA9384AA	245	261
ESC_LA9383AA	9060	261
ESC_LA9380AA	4554	738
ESC_LA9379AA	3032	912
ESC_LA9378AA	59	217
ESC_LA9377AA	12	626
ESC_LA9376AA	23	156
ESC_LA9373AA	8874	421
ESC_LA9372AA	117	592
ESC_LA9371AA	3695	924
ESC_LA9370AA	11	936
ESC_LA9369AA	11	936
ESC_LA9368AA	11	936
ESC_LA9367AA	11	936
ESC_LA9365AA	8658	156
ESC_LA9364AA	6260	156
ESC_LA9363AA	7931	234
ESC_LA9361AA	1819	808
ESC_LA9360AA	8264	586
ESC_LA9359AA	145	256
ESC_LA9358AA	245	261
ESC_LA9357AA	245	261
ESC_LA9356AA	10	512

ESC_LA9337AA	10	512
ESC_LA9336AA	10	512
ESC_LA9335AA	10	512
ESC_LA9334AA	301	307
ESC_LA9333AA	5822	696
ESC_LA9332AA	101	696
ESC_LA9331AA	11	936
ESC_LA9330AA	4085	791
ESC_LA9329AA	410	156
ESC_LA9328AA	361	512
ESC_LA9327AA	95	738
ESC_LA9326AA	641	935
ESC_LA9325AA	539	257
ESC_LA9324AA	10	512
ESC_LA9323AA	187	257
ESC_LA9322AA	1087	235
ESC_LA9321AA	58	198
ESC_LA9320AA	278	924
ESC_LA9318AA	131	653
ESC_LA9317AA	11	936
ESC_LA9315AA	141	644
ESC_LA9314AA	95	738
ESC_LA9312AA	12	626
ESC_LA9311AA	95	738
ESC_LA9310AA	421	744
ESC_LA9309AA	131	653
ESC_LA9308AA	301	307
ESC_LA9307AA	127	738
ESC_LA9306AA	301	307
ESC_LA9305AA	21	156
ESC_LA9304AA	21	156
ESC_LA9303AA	21	156
ESC_LA9302AA	21	156
ESC_LA9301AA	131	653
ESC_LA9300AA	131	653
ESC_LA9299AA	131	653
ESC_LA9298AA	131	653
ESC_LA9297AA	131	653
ESC_LA9296AA	131	653
ESC_LA9295AA	131	653
ESC_LA9294AA	131	653
ESC_LA9293AA	131	653
ESC_LA9292AA	131	653
ESC_LA9291AA	131	653
ESC_LA9290AA	131	653
ESC_LA9289AA	131	653
ESC_LA9288AA	131	653
ESC_LA9287AA	131	653
ESC_LA9286AA	131	653
ESC_LA9285AA	131	653

ESC_LA9284AA	131	653
ESC_LA9283AA	131	653
ESC_LA9282AA	131	653
ESC_LA9281AA	131	653
ESC_LA9280AA	131	653
ESC_LA9279AA	131	653
ESC_LA9278AA	131	653
ESC_LA9277AA	131	653
ESC_LA9276AA	131	653
ESC_LA9275AA	131	653
ESC_LA9274AA	131	653
ESC_LA9273AA	131	653
ESC_LA9272AA	131	653
ESC_LA9271AA	131	653
ESC_LA9270AA	131	653
ESC_LA9269AA	131	653
ESC_LA9268AA	131	653
ESC_LA9267AA	131	653
ESC_LA9266AA	131	653
ESC_LA9265AA	245	261
ESC_LA9264AA	245	261
ESC_LA9263AA	152	257
ESC_LA9262AA	152	257
ESC_LA9261AA	648	359
ESC_LA9260AA	93	71
ESC_LA9259AA	88	160
ESC_LA9258AA	38	669
ESC_LA9257AA	88	160
ESC_LA9256AA	155	175
ESC_LA9255AA	773	71
ESC_LA9254AA	648	359
ESC_LA9253AA	7470	50
ESC_LA9252AA	46	447
ESC_LA9251AA	773	71
ESC_LA9250AA	10	512
ESC_LA9249AA	68	386
ESC_LA9244AA	405	923
ESC_LA9243AA	131	653
ESC_LA9242AA	3024	696
ESC_LA9241AA	1177	669
ESC_LA9240AA	3024	696
ESC_LA9239AA	602	257
ESC_LA9238AA	131	653
ESC_LA9236AA	131	653
ESC_LA9235AA	10	512
ESC_LA9234AA	56	198
ESC_LA9233AA	23	156
ESC_LA9232AA	405	923
ESC_LA9230AA	38	669
ESC_LA9229AA	131	653

ESC_LA9228AA	131	653
ESC_LA9227AA	58	198
ESC_LA9226AA	56	198
ESC_LA9225AA	9028	696
ESC_LA9224AA	131	653
ESC_LA9222AA	354	15
ESC_LA9221AA	131	653
ESC_LA9220AA	58	198
ESC_LA9219AA	131	653
ESC_LA9218AA	131	653
ESC_LA9217AA	23	156
ESC_LA9214AA	7358	923
ESC_LA9213AA	131	653
ESC_LA9212AA	58	198
ESC_LA9210AA	410	156
ESC_LA9208AA	58	198
ESC_LA9203AA	354	15
ESC_LA9202AA	135	653
ESC_LA9201AA	69	387
ESC_LA9200AA	410	156
ESC_LA9199AA	141	644
ESC_LA9198AA	73	737
ESC_LA9197AA	648	359
ESC_LA9196AA	131	653
ESC_LA9195AA	73	737
ESC_LA9194AA	48	76
ESC_LA9192AA	69	387
ESC_LA9191AA	131	653
ESC_LA9190AA	48	76
ESC_LA9189AA	410	156
ESC_LA9188AA	131	653
ESC_LA9186AA	131	653
ESC_LA9185AA	405	923
ESC_LA9184AA	131	653
ESC_LA9183AA	405	923
ESC_LA9181AA	44	512
ESC_LA9180AA	58	198
ESC_LA9179AA	617	511
ESC_LA9178AA	7470	50
ESC_LA9177AA	34	504
ESC_LA9174AA	23	156
ESC_LA9173AA	648	359
ESC_LA9169AA	162	924
ESC_LA9167AA	648	359
ESC_LA9166AA	131	653
ESC_LA9164AA	90	156
ESC_LA9163AA	131	653
ESC_LA9162AA	131	653
ESC_LA9161AA	69	387
ESC_LA9160AA	131	653

ESC_LA9159AA	410	156
ESC_LA9158AA	167	512
ESC_LA9157AA	131	653
ESC_LA9156AA	131	653
ESC_LA9155AA	744	512
ESC_LA9154AA	744	512
ESC_LA9153AA	10	512
ESC_LA9152AA	10	512
ESC_LA9151AA	8954	198
ESC_LA9150AA	448	257
ESC_LA9149AA	453	935
ESC_LA9148AA	7468	935
ESC_LA9144AA	46	447
ESC_LA9143AA	46	447
ESC_LA9142AA	131	653
ESC_LA9140AA	361	512
ESC_LA9138AA	46	447
ESC_LA9136AA	131	653
ESC_LA9135AA	46	447
ESC_LA9134AA	131	653
ESC_LA9133AA	7469	180
ESC_LA9131AA	90	156
ESC_LA9130AA	88	160
ESC_LA9129AA	155	175
ESC_LA9128AA	744	512
ESC_LA9127AA	88	160
ESC_LA9126AA	10	512
ESC_LA9125AA	58	198
ESC_LA9124AA	131	653
ESC_LA9123AA	683	175
ESC_LA9122AA	155	175
ESC_LA9121AA	131	653
ESC_LA9120AA	131	653
ESC_LA9119AA	131	653
ESC_LA9118AA	3564	160
ESC_LA9117AA	131	653
ESC_LA9116AA	648	359
ESC_LA9115AA	162	924
ESC_LA9114AA	10	512
ESC_LA9113AA	167	512
ESC_LA9111AA	131	653
ESC_LA9110AA	131	653
ESC_LA9108AA	59	217
ESC_LA9107AA	224	198
ESC_LA9106AA	131	653
ESC_LA9105AA	131	653
ESC_LA9104AA	10	512
ESC_LA9103AA	648	359
ESC_LA9102AA	10	512
ESC_LA9101AA	648	359

ESC_LA9100AA	131	653
ESC_LA9099AA	361	512
ESC_LA9098AA	131	653
ESC_LA9097AA	131	653
ESC_LA9096AA	131	653
ESC_LA9095AA	131	653
ESC_LA9094AA	10	512
ESC_LA9093AA	227	504
ESC_LA9092AA	58	198
ESC_LA9090AA	131	653
ESC_LA9089AA	167	512
ESC_LA9088AA	131	653
ESC_LA9086AA	744	512
ESC_LA9085AA	410	156
ESC_LA9084AA	131	653
ESC_LA9083AA	88	160
ESC_LA9081AA	162	924
ESC_LA9080AA	10	512
ESC_LA9079AA	131	653
ESC_LA9078AA	73	737
ESC_LA9077AA	131	653
ESC_LA9074AA	2025	194
ESC_LA9073AA	38	669
ESC_LA9072AA	10	512
ESC_LA9071AA	9030	175
ESC_LA9069AA	131	653
ESC_LA9068AA	131	653
ESC_LA9067AA	3873	175
ESC_LA9066AA	131	653
ESC_LA9065AA	131	653
ESC_LA9064AA	131	653
ESC_LA9063AA	2496	789
ESC_LA9062AA	1421	447
ESC_LA9061AA	58	198
ESC_LA9060AA	131	653
ESC_LA9059AA	131	653
ESC_LA9058AA	227	504
ESC_LA9057AA	162	924
ESC_LA9056AA	1480	160
ESC_LA9055AA	48	76
ESC_LA9054AA	48	76
ESC_LA9053AA	9029	156
ESC_LA9052AA	131	653
ESC_LA9051AA	410	156
ESC_LA9050AA	448	257
ESC_LA9049AA	744	512
ESC_LA9048AA	10	512
ESC_LA9046AA	3873	175
ESC_LA9045AA	410	156
ESC_LA9044AA	131	653

ESC_LA9043AA	167	512
ESC_LA9042AA	224	198
ESC_LA9041AA	167	512
ESC_LA9040AA	131	653
ESC_LA9039AA	1859	653
ESC_LA9038AA	131	653
ESC_LA9037AA	675	234
ESC_LA9034AA	1057	761
ESC_LA9033AA	10	512
ESC_LA9032AA	357	654
ESC_LA9031AA	357	654
ESC_LA9029AA	69	387
ESC_LA9028AA	410	156
ESC_LA9027AA	410	156
ESC_LA9026AA	155	175
ESC_LA9025AA	372	424
ESC_LA9024AA	1444	768
ESC_LA9023AA	5351	731
ESC_LA9022AA	38	669
ESC_LA9021AA	1594	71
ESC_LA9020AA	1594	71
ESC_LA9019AA	1049	175
ESC_LA9018AA	1049	175
ESC_LA9016AA	5778	233
ESC_LA9015AA	5778	233
ESC_LA9014AA	5778	233
ESC_LA9013AA	95	738
ESC_LA9012AA	95	738
ESC_LA9011AA	95	738
ESC_LA9010AA	95	738
ESC_LA9009AA	95	738
ESC_LA9008AA	127	738
ESC_LA9007AA	127	738
ESC_LA9006AA	127	738
ESC_LA9005AA	127	738
ESC_LA9004AA	58	198
ESC_LA9003AA	10	512
ESC_LA9002AA	80	731
ESC_LA9001AA	10	512
ESC_LA9000AA	69	387
ESC_LA8999AA	95	738
ESC_LA8998AA	695	519
ESC_LA8997AA	695	519
ESC_LA8996AA	695	519
ESC_LA8995AA	69	387
ESC_LA8994AA	1231	738
ESC_LA8993AA	1231	738
ESC_LA8992AA	12	626
ESC_LA8990AA	73	737
ESC_LA8989AA	38	669

ESC_LA8988AA	354	15
ESC_LA8987AA	95	738
ESC_LA8986AA	393	343
ESC_LA8985AA	95	738
ESC_LA8984AA	484	71
ESC_LA8983AA	62	574
ESC_LA8982AA	2967	289
ESC_LA8981AA	3312	424
ESC_LA8980AA	69	387
ESC_LA8979AA	69	387
ESC_LA8978AA	131	653
ESC_LA8977AA	95	738
ESC_LA8976AA	131	653
ESC_LA8974AA	73	737
ESC_LA8971AA	624	359
ESC_LA8970AA	624	359
ESC_LA8968AA	88	160
ESC_LA8967AA	69	387
ESC_LA8966AA	2015	684
ESC_LA8965AA	95	738
ESC_LA8963AA	73	737
ESC_LA8962AA	73	737
ESC_LA8961AA	245	261
ESC_LA8960AA	1057	761
ESC_LA8959AA	131	653
ESC_LA8958AA	69	387
ESC_LA8957AA	1652	670
ESC_LA8955AA	2851	156
ESC_LA8954AA	12	626
ESC_LA8953AA	14	761
ESC_LA8951AA	7523	626
ESC_LA8950AA	12	626
ESC_LA8949AA	131	653
ESC_LA8948AA	131	653
ESC_LA8947AA	95	738
ESC_LA8946AA	62	574
ESC_LA8945AA	73	737
ESC_LA8944AA	131	653
ESC_LA8943AA	127	738
ESC_LA8942AA	69	387
ESC_LA8941AA	2175	212
ESC_LA8940AA	219	877
ESC_LA8939AA	131	653
ESC_LA8938AA	69	387
ESC_LA8937AA	69	387
ESC_LA8936AA	69	387
ESC_LA8935AA	131	653
ESC_LA8934AA	14	761
ESC_LA8933AA	69	387
ESC_LA8932AA	636	653

ESC_LA8930AA	131	653
ESC_LA8929AA	73	737
ESC_LA8928AA	73	737
ESC_LA8926AA	80	731
ESC_LA8925AA	73	737
ESC_LA8924AA	617	511
ESC_LA8923AA	38	669
ESC_LA8922AA	131	653
ESC_LA8921AA	131	653
ESC_LA8919AA	12	626
ESC_LA8918AA	141	644
ESC_LA8917AA	421	744
ESC_LA8916AA	131	653
ESC_LA8915AA	3489	962
ESC_LA8914AA	69	387
ESC_LA8913AA	1324	156
ESC_LA8912AA	162	924
ESC_LA8911AA	58	198
ESC_LA8910AA	354	15
ESC_LA8909AA	95	738
ESC_LA8908AA	127	738
ESC_LA8907AA	12	626
ESC_LA8906AA	131	653
ESC_LA8905AA	131	653
ESC_LA8904AA	117	592
ESC_LA8903AA	73	737
ESC_LA8902AA	131	653
ESC_LA8901AA	69	387
ESC_LA8900AA	501	900
ESC_LA8899AA	69	387
ESC_LA8898AA	58	198
ESC_LA8897AA	131	653
ESC_LA8896AA	7519	653
ESC_LA8895AA	131	653
ESC_LA8894AA	73	737
ESC_LA8893AA	93	71
ESC_LA8892AA	7521	512
ESC_LA8891AA	7521	512
ESC_LA8890AA	624	359
ESC_LA8889AA	73	737
ESC_LA8888AA	88	160
ESC_LA8887AA	10	512
ESC_LA8886AA	429	654
ESC_LA8885AA	1057	761
ESC_LA8884AA	1914	956
ESC_LA8883AA	73	737
ESC_LA8882AA	6756	962
ESC_LA8881AA	69	387
ESC_LA8880AA	73	737
ESC_LA8879AA	73	737

ESC_LA8878AA	7525	359
ESC_LA8877AA	69	387
ESC_LA8876AA	131	653
ESC_LA8875AA	131	653
ESC_LA8874AA	69	387
ESC_LA8873AA	95	738
ESC_LA8872AA	73	737
ESC_LA8871AA	69	387
ESC_LA8870AA	95	738
ESC_LA8868AA	12	626
ESC_LA8867AA	131	653
ESC_LA8866AA	544	626
ESC_LA8865AA	131	653
ESC_LA8864AA	38	669
ESC_LA8863AA	127	738
ESC_LA8861AA	131	653
ESC_LA8860AA	73	737
ESC_LA8859AA	428	654
ESC_LA8858AA	80	731
ESC_LA8857AA	12	626
ESC_LA8856AA	73	737
ESC_LA8855AA	681	393
ESC_LA8854AA	131	653
ESC_LA8853AA	1193	761
ESC_LA8852AA	131	653
ESC_LA8851AA	2741	696
ESC_LA8850AA	73	737
ESC_LA8849AA	131	653
ESC_LA8848AA	73	737
ESC_LA8847AA	73	737
ESC_LA8846AA	69	387
ESC_LA8845AA	1859	653
ESC_LA8844AA	69	387
ESC_LA8843AA	58	198
ESC_LA8842AA	73	737
ESC_LA8841AA	12	626
ESC_LA8840AA	550	756
ESC_LA8839AA	95	738
ESC_LA8838AA	69	387
ESC_LA8837AA	12	626
ESC_LA8836AA	38	669
ESC_LA8835AA	73	737
ESC_LA8834AA	73	737
ESC_LA8833AA	95	738
ESC_LA8832AA	95	738
ESC_LA8831AA	7526	626
ESC_LA8830AA	73	737
ESC_LA8829AA	569	738
ESC_LA8828AA	73	737
ESC_LA8827AA	7528	912

ESC_LA8826AA	21	156
ESC_LA8825AA	17	191
ESC_LA8823AA	196	257
ESC_LA8822AA	152	257
ESC_LA8821AA	245	261
ESC_LA8820AA	245	261
ESC_LA8819AA	245	261
ESC_LA8818AA	152	257
ESC_LA8817AA	152	257
ESC_LA8816AA	152	257
ESC_LA8815AA	245	261
ESC_LA8814AA	245	261
ESC_LA8813AA	7379	190
ESC_LA8812AA	145	256
ESC_LA8811AA	245	261
ESC_LA8809AA	152	257
ESC_LA8808AA	8371	898
ESC_LA8807AA	13	257
ESC_LA8806AA	33	198
ESC_LA8805AA	675	234
ESC_LA8803AA	245	261
ESC_LA8802AA	245	261
ESC_LA8801AA	152	257
ESC_LA8800AA	152	257
ESC_LA8799AA	152	257
ESC_LA8797AA	152	257
ESC_LA8796AA	145	256
ESC_LA8795AA	7379	190
ESC_LA8794AA	152	257
ESC_LA8793AA	152	257
ESC_LA8792AA	245	261
ESC_LA8790AA	152	257
ESC_LA8789AA	152	257
ESC_LA8788AA	152	257
ESC_LA8787AA	252	190
ESC_LA8786AA	152	257
ESC_LA8785AA	11	936
ESC_LA8784AA	442	257
ESC_LA8783AA	11	936
ESC_LA8782AA	8925	258
ESC_LA8781AA	117	592
ESC_LA8780AA	11	936
ESC_LA8779AA	6	445
ESC_LA8778AA	297	234
ESC_LA8777AA	17	191
ESC_LA8776AA	11	936
ESC_LA8765AA	298	286
ESC_LA8764AA	21	156
ESC_LA8763AA	32	270
ESC_LA8762AA	21	156

ESC_LA8760AA	21	156
ESC_LA8759AA	21	156
ESC_LA8758AA	21	156
ESC_LA8757AA	21	156
ESC_LA8756AA	21	156
ESC_LA8755AA	29	156
ESC_LA8754AA	21	156
ESC_LA8753AA	21	156
ESC_LA8752AA	21	156
ESC_LA8751AA	21	156
ESC_LA8750AA	21	156
ESC_LA8749AA	21	156
ESC_LA8748AA	21	156
ESC_LA8747AA	21	156
ESC_LA8746AA	152	257
ESC_LA8745AA	245	261
ESC_LA8744AA	152	257
ESC_LA8743AA	152	257
ESC_LA8742AA	245	261
ESC_LA8741AA	152	257
ESC_LA8740AA	1503	257
ESC_LA8739AA	245	261
ESC_LA8738AA	245	261
ESC_LA8737AA	145	256
ESC_LA8735AA	245	261
ESC_LA8734AA	152	257
ESC_LA8733AA	11	936
ESC_LA8732AA	301	307
ESC_LA8731AA	301	307
ESC_LA8730AA	301	307
ESC_LA8729AA	11	936
ESC_LA8728AA	301	307
ESC_LA8727AA	11	936
ESC_LA8726AA	11	936
ESC_LA8725AA	29	156
ESC_LA8724AA	1877	773
ESC_LA8723AA	394	400
ESC_LA8722AA	10	512
ESC_LA8721AA	5295	355
ESC_LA8720AA	1060	512
ESC_LA8719AA	10	512
ESC_LA8718AA	10	512
ESC_LA8717AA	10	512
ESC_LA8716AA	10	512
ESC_LA8715AA	10	512
ESC_LA8714AA	404	761
ESC_LA8713AA	10	512
ESC_LA8712AA	8889	512
ESC_LA8711AA	2617	217
ESC_LA8709AA	10	512

ESC_LA8708AA	10	512
ESC_LA8707AA	10	512
ESC_LA8706AA	10	512
ESC_LA8705AA	10	512
ESC_LA8704AA	11	936
ESC_LA8703AA	131	653
ESC_LA8702AA	167	512
ESC_LA8701AA	224	198
ESC_LA8700AA	224	198
ESC_LA8699AA	224	198
ESC_LA8698AA	7506	198
ESC_LA8697AA	354	15
ESC_LA8696AA	405	923
ESC_LA8695AA	405	923
ESC_LA8694AA	410	156
ESC_LA8693AA	877	935
ESC_LA8692AA	1011	198
ESC_LA8691AA	1193	761
ESC_LA8690AA	1193	761
ESC_LA8689AA	1193	761
ESC_LA8688AA	1193	761
ESC_LA8687AA	1196	257
ESC_LA8686AA	1722	599
ESC_LA8685AA	1193	761
ESC_LA8684AA	6756	962
ESC_LA8683AA	8755	257
ESC_LA8682AA	8891	113
ESC_LA8681AA	8890	789
ESC_LA8680AA	131	653
ESC_LA8679AA	448	257
ESC_LA8678AA	7090	586
ESC_LA8677AA	69	387
ESC_LA8676AA	101	696
ESC_LA8675AA	155	175
ESC_LA8674AA	448	257
ESC_LA8673AA	9175	928
ESC_LA8672AA	2280	212
ESC_LA8671AA	410	156
ESC_LA8670AA	167	512
ESC_LA8669AA	648	359
ESC_LA8668AA	162	924
ESC_LA8667AA	226	512
ESC_LA8666AA	162	924
ESC_LA8665AA	131	653
ESC_LA8664AA	101	696
ESC_LA8663AA	354	15
ESC_LA8661AA	940	257
ESC_LA8660AA	212	198
ESC_LA8659AA	69	387
ESC_LA8658AA	101	696

ESC_LA8657AA	101	696
ESC_LA8656AA	48	76
ESC_LA8655AA	361	512
ESC_LA8654AA	101	696
ESC_LA8653AA	162	924
ESC_LA8652AA	1193	761
ESC_LA8651AA	167	512
ESC_LA8650AA	10	512
ESC_LA8649AA	10	512
ESC_LA8648AA	38	669
ESC_LA8647AA	38	669
ESC_LA8646AA	68	386
ESC_LA8645AA	69	387
ESC_LA8644AA	83	740
ESC_LA8643AA	117	592
ESC_LA8642AA	131	653
ESC_LA8641AA	10	512
ESC_LA8640AA	10	512
ESC_LA8639AA	10	512
ESC_LA8638AA	6389	512
ESC_LA8637AA	10	512
ESC_LA8636AA	10	512
ESC_LA8635AA	9272	512
ESC_LA8634AA	10	512
ESC_LA8633AA	10	512
ESC_LA8632AA	10	512
ESC_LA8631AA	6389	512
ESC_LA8630AA	6389	512
ESC_LA8629AA	10	512
ESC_LA8628AA	10	512
ESC_LA8627AA	9270	512
ESC_LA8626AA	9280	512
ESC_LA8624AA	10	512
ESC_LA8619AA	11	936
ESC_LA8618AA	607	355
ESC_LA8617AA	8895	71
ESC_LA8616AA	93	71
ESC_LA8615AA	11	936
ESC_LA8614AA	5342	765
ESC_LA8613AA	5295	355
ESC_LA8612AA	472	601
ESC_LA8611AA	11	936
ESC_LA8610AA	10	512
ESC_LA8609AA	3570	156
ESC_LA8608AA	8894	512
ESC_LA8607AA	11	936
ESC_LA8606AA	636	653
ESC_LA8605AA	206	286
ESC_LA8604AA	1804	935
ESC_LA8603AA	38	669

ESC_LA8602AA	11	936
ESC_LA8601AA	3014	410
ESC_LA8600AA	34	504
ESC_LA8599AA	372	424
ESC_LA8598AA	10	512
ESC_LA8597AA	11	936
ESC_LA8596AA	117	592
ESC_LA8595AA	117	592
ESC_LA8594AA	117	592
ESC_LA8593AA	117	592
ESC_LA8592AA	95	738
ESC_LA8591AA	117	592
ESC_LA8590AA	117	592
ESC_LA8589AA	1163	579
ESC_LA8588AA	162	924
ESC_LA8587AA	1163	579
ESC_LA8586AA	117	592
ESC_LA8585AA	117	592
ESC_LA8584AA	117	592
ESC_LA8583AA	117	592
ESC_LA8582AA	117	592
ESC_LA8581AA	117	592
ESC_LA8580AA	117	592
ESC_LA8579AA	117	592
ESC_LA8578AA	117	592
ESC_LA8577AA	117	592
ESC_LA8576AA	117	592
ESC_LA8575AA	95	738
ESC_LA8574AA	117	592
ESC_LA8573AA	117	592
ESC_LA8572AA	95	738
ESC_LA8571AA	117	592
ESC_LA8570AA	117	592
ESC_LA8569AA	117	592
ESC_LA8568AA	117	592
ESC_LA8567AA	117	592
ESC_LA8566AA	117	592
ESC_LA8565AA	95	738
ESC_LA8564AA	117	592
ESC_LA8563AA	117	592
ESC_LA8562AA	117	592
ESC_LA8561AA	140	738
ESC_LA8560AA	23	156
ESC_LA8559AA	648	359
ESC_LA8558AA	371	50
ESC_LA8557AA	162	924
ESC_LA8556AA	95	738
ESC_LA8555AA	117	592
ESC_LA8554AA	117	592
ESC_LA8553AA	95	738

ESC_LA8552AA	141	644
ESC_LA8551AA	95	738
ESC_LA8550AA	117	592
ESC_LA8549AA	117	592
ESC_LA8548AA	117	592
ESC_LA8547AA	117	592
ESC_LA8546AA	117	592
ESC_LA8545AA	95	738
ESC_LA8544AA	117	592
ESC_LA8543AA	117	592
ESC_LA8542AA	93	71
ESC_LA8541AA	23	156
ESC_LA8540AA	117	592
ESC_LA8539AA	117	592
ESC_LA8538AA	117	592
ESC_LA8537AA	93	71
ESC_LA8536AA	355	764
ESC_LA8535AA	117	592
ESC_LA8534AA	117	592
ESC_LA8532AA	117	592
ESC_LA8530AA	117	592
ESC_LA8529AA	648	359
ESC_LA8528AA	10	512
ESC_LA8526AA	11	936
ESC_LA8525AA	6178	924
ESC_LA8524AA	131	653
ESC_LA8523AA	131	653
ESC_LA8522AA	131	653
ESC_LA8521AA	131	653
ESC_LA8520AA	131	653
ESC_LA8519AA	131	653
ESC_LA8518AA	131	653
ESC_LA8517AA	131	653
ESC_LA8516AA	131	653
ESC_LA8515AA	131	653
ESC_LA8514AA	131	653
ESC_LA8513AA	131	653
ESC_LA8512AA	131	653
ESC_LA8511AA	131	653
ESC_LA8510AA	131	653
ESC_LA8509AA	131	653
ESC_LA8508AA	131	653
ESC_LA8507AA	131	653
ESC_LA8506AA	131	653
ESC_LA8505AA	131	653
ESC_LA8504AA	131	653
ESC_LA8503AA	131	653
ESC_LA8502AA	131	653
ESC_LA8501AA	131	653
ESC_LA8500AA	131	653

ESC_LA8499AA	131	653
ESC_LA8498AA	131	653
ESC_LA8497AA	131	653
ESC_LA8496AA	131	653
ESC_LA8495AA	131	653
ESC_LA8494AA	131	653
ESC_LA8493AA	131	653
ESC_LA8492AA	131	653
ESC_LA8491AA	131	653
ESC_LA8490AA	131	653
ESC_LA8489AA	131	653
ESC_LA8488AA	131	653
ESC_LA8487AA	131	653
ESC_LA8486AA	131	653
ESC_LA8485AA	131	653
ESC_LA8484AA	131	653
ESC_LA8483AA	131	653
ESC_LA8482AA	131	653
ESC_LA8481AA	131	653
ESC_LA8480AA	131	653
ESC_LA8479AA	131	653
ESC_LA8478AA	131	653
ESC_LA8477AA	131	653
ESC_LA8476AA	131	653
ESC_LA8475AA	131	653
ESC_LA8474AA	131	653
ESC_LA8473AA	131	653
ESC_LA8472AA	131	653
ESC_LA8471AA	131	653
ESC_LA8470AA	131	653
ESC_LA8469AA	131	653
ESC_LA8468AA	131	653
ESC_LA8467AA	131	653
ESC_LA8466AA	131	653
ESC_LA8465AA	131	653
ESC_LA8464AA	131	653
ESC_LA8463AA	131	653
ESC_LA8462AA	131	653
ESC_LA8461AA	131	653
ESC_LA8460AA	131	653
ESC_LA8459AA	131	653
ESC_LA8458AA	131	653
ESC_LA8457AA	131	653
ESC_LA8456AA	131	653
ESC_LA8455AA	131	653
ESC_LA8454AA	131	653
ESC_LA8453AA	131	653
ESC_LA8452AA	131	653
ESC_LA8451AA	131	653
ESC_LA8450AA	131	653

ESC_LA8449AA	131	653
ESC_LA8448AA	131	653
ESC_LA8447AA	131	653
ESC_LA8446AA	131	653
ESC_LA8445AA	131	653
ESC_LA8444AA	131	653
ESC_LA8443AA	131	653
ESC_LA8442AA	131	653
ESC_LA8441AA	131	653
ESC_LA8440AA	131	653
ESC_LA8439AA	131	653
ESC_LA8438AA	131	653
ESC_LA8437AA	131	653
ESC_LA8436AA	131	653
ESC_LA8435AA	131	653
ESC_LA8434AA	131	653
ESC_LA8433AA	131	653
ESC_LA8432AA	131	653
ESC_LA8431AA	131	653
ESC_LA8430AA	131	653
ESC_LA8429AA	131	653
ESC_LA8428AA	131	653
ESC_LA8427AA	131	653
ESC_LA8426AA	131	653
ESC_LA8425AA	131	653
ESC_LA8424AA	131	653
ESC_LA8423AA	131	653
ESC_LA8422AA	131	653
ESC_LA8421AA	131	653
ESC_LA8420AA	131	653
ESC_LA8419AA	131	653
ESC_LA8418AA	131	653
ESC_LA8417AA	131	653
ESC_LA8416AA	131	653
ESC_LA8415AA	131	653
ESC_LA8414AA	131	653
ESC_LA8413AA	131	653
ESC_LA8412AA	131	653
ESC_LA8411AA	131	653
ESC_LA8410AA	131	653
ESC_LA8409AA	131	653
ESC_LA8408AA	131	653
ESC_LA8407AA	131	653
ESC_LA8406AA	131	653
ESC_LA8405AA	131	653
ESC_LA8404AA	131	653
ESC_LA8403AA	131	653
ESC_LA8402AA	131	653
ESC_LA8401AA	131	653
ESC_LA8400AA	131	653

ESC_LA8399AA	131	653
ESC_LA8398AA	131	653
ESC_LA8397AA	131	653
ESC_LA8396AA	131	653
ESC_LA8395AA	131	653
ESC_LA8394AA	131	653
ESC_LA8393AA	131	653
ESC_LA8392AA	131	653
ESC_LA8391AA	131	653
ESC_LA8390AA	131	653
ESC_LA8389AA	131	653
ESC_LA8388AA	131	653
ESC_LA8387AA	131	653
ESC_LA8386AA	131	653
ESC_LA8385AA	131	653
ESC_LA8384AA	131	653
ESC_LA8383AA	131	653
ESC_LA8382AA	131	653
ESC_LA8381AA	131	653
ESC_LA8380AA	131	653
ESC_LA8379AA	131	653
ESC_LA8378AA	131	653
ESC_LA8377AA	131	653
ESC_LA8376AA	131	653
ESC_LA8375AA	131	653
ESC_LA8374AA	131	653
ESC_LA8373AA	131	653
ESC_LA8372AA	131	653
ESC_LA8371AA	131	653
ESC_LA8370AA	131	653
ESC_LA8369AA	131	653
ESC_LA8368AA	131	653
ESC_LA8367AA	131	653
ESC_LA8366AA	131	653
ESC_LA8365AA	131	653
ESC_LA8364AA	131	653
ESC_LA8363AA	131	653
ESC_LA8362AA	131	653
ESC_LA8361AA	131	653
ESC_LA8360AA	131	653
ESC_LA8359AA	131	653
ESC_LA8358AA	131	653
ESC_LA8357AA	131	653
ESC_LA8356AA	131	653
ESC_LA8355AA	131	653
ESC_LA8354AA	131	653
ESC_LA8353AA	131	653
ESC_LA8352AA	131	653
ESC_LA8351AA	131	653
ESC_LA8350AA	131	653

ESC_LA8349AA	131	653
ESC_LA8348AA	131	653
ESC_LA8347AA	131	653
ESC_LA8346AA	131	653
ESC_LA8345AA	131	653
ESC_LA8344AA	131	653
ESC_LA8343AA	131	653
ESC_LA8342AA	131	653
ESC_LA8341AA	131	653
ESC_LA8340AA	131	653
ESC_LA8339AA	131	653
ESC_LA8338AA	131	653
ESC_LA8337AA	131	653
ESC_LA8336AA	131	653
ESC_LA8335AA	131	653
ESC_LA8334AA	131	653
ESC_LA8333AA	131	653
ESC_LA8332AA	131	653
ESC_LA8331AA	131	653
ESC_LA8330AA	131	653
ESC_LA8329AA	131	653
ESC_LA8328AA	131	653
ESC_LA8327AA	131	653
ESC_LA8326AA	131	653
ESC_LA8325AA	131	653
ESC_LA8324AA	131	653
ESC_LA8323AA	131	653
ESC_LA8322AA	131	653
ESC_LA8321AA	131	653
ESC_LA8320AA	131	653
ESC_LA8319AA	131	653
ESC_LA8318AA	131	653
ESC_LA8316AA	372	424
ESC_LA8314AA	1060	512
ESC_LA8252AA	93	71
ESC_LA8251AA	93	71
ESC_LA8241AA	11	936
ESC_LA8237AA	131	653
ESC_LA8236AA	131	653
ESC_LA8235AA	46	447
ESC_LA8234AA	117	592
ESC_LA8233AA	58	198
ESC_LA8232AA	155	175
ESC_LA8231AA	4542	286
ESC_LA8230AA	453	935
ESC_LA8229AA	602	257
ESC_LA8228AA	3902	962
ESC_LA8227AA	127	738
ESC_LA8224AA	10	512
ESC_LA8223AA	10	512

ESC_LA8222AA	10	512
ESC_LA8221AA	10	512
ESC_LA8220AA	10	512
ESC_LA8219AA	10	512
ESC_LA8218AA	10	512
ESC_LA8217AA	10	512
ESC_LA8216AA	10	512
ESC_LA8215AA	10	512
ESC_LA8214AA	10	512
ESC_LA8213AA	93	71
ESC_LA8212AA	93	71
ESC_LA8211AA	93	71
ESC_LA8210AA	93	71
ESC_LA8209AA	93	71
ESC_LA8208AA	93	71
ESC_LA8207AA	93	71
ESC_LA8206AA	93	71
ESC_LA8205AA	93	71
ESC_LA8204AA	93	71
ESC_LA8203AA	93	71
ESC_LA8202AA	93	71
ESC_LA8201AA	93	71
ESC_LA8200AA	93	71
ESC_LA8199AA	93	71
ESC_LA8198AA	93	71
ESC_LA8197AA	93	71
ESC_LA8196AA	93	71
ESC_LA8195AA	93	71
ESC_LA8194AA	93	71
ESC_LA8193AA	93	71
ESC_LA8192AA	93	71
ESC_LA8191AA	93	71
ESC_LA8190AA	93	71
ESC_LA8189AA	93	71
ESC_LA8188AA	93	71
ESC_LA8187AA	93	71
ESC_LA8186AA	93	71
ESC_LA8185AA	93	71
ESC_LA8184AA	93	71
ESC_LA8183AA	93	71
ESC_LA8182AA	93	71
ESC_LA8181AA	93	71
ESC_LA8180AA	93	71
ESC_LA8179AA	93	71
ESC_LA8178AA	93	71
ESC_LA8177AA	93	71
ESC_LA8176AA	93	71
ESC_LA8175AA	93	71
ESC_LA8174AA	93	71
ESC_LA8173AA	93	71

ESC_LA8172AA	93	71
ESC_LA8171AA	93	71
ESC_LA8170AA	93	71
ESC_LA8169AA	93	71
ESC_LA8168AA	93	71
ESC_LA8167AA	93	71
ESC_LA8166AA	93	71
ESC_LA8165AA	93	71
ESC_LA8164AA	93	71
ESC_LA8163AA	93	71
ESC_LA8162AA	93	71
ESC_LA8161AA	93	71
ESC_LA8160AA	93	71
ESC_LA8159AA	93	71
ESC_LA8158AA	93	71
ESC_LA8157AA	93	71
ESC_LA8156AA	93	71
ESC_LA8155AA	93	71
ESC_LA8154AA	93	71
ESC_LA8153AA	93	71
ESC_LA8152AA	93	71
ESC_LA8151AA	93	71
ESC_LA8150AA	93	71
ESC_LA8149AA	93	71
ESC_LA8148AA	93	71
ESC_LA8147AA	93	71
ESC_LA8146AA	93	71
ESC_LA8145AA	93	71
ESC_LA8144AA	93	71
ESC_LA8143AA	93	71
ESC_LA8142AA	93	71
ESC_LA8141AA	93	71
ESC_LA8140AA	93	71
ESC_LA8139AA	93	71
ESC_LA8138AA	93	71
ESC_LA8137AA	93	71
ESC_LA8136AA	93	71
ESC_LA8135AA	93	71
ESC_LA8134AA	93	71
ESC_LA8133AA	93	71
ESC_LA8132AA	93	71
ESC_LA8131AA	93	71
ESC_LA8130AA	93	71
ESC_LA8129AA	93	71
ESC_LA8128AA	93	71
ESC_LA8127AA	93	71
ESC_LA8126AA	93	71
ESC_LA8118AA	10	512
ESC_LA8117AA	10	512
ESC_LA8114AA	10	512

ESC_LA8113AA	10	512
ESC_LA8105AA	10	512
ESC_LA8101AA	117	592
ESC_LA8072AA	11	936
ESC_LA8071AA	11	936
ESC_LA8070AA	11	936
ESC_LA8069AA	448	257
ESC_LA8068AA	3640	257
ESC_LA8067AA	155	175
ESC_LA8065AA	155	175
ESC_LA8064AA	2077	198
ESC_LA8063AA	2077	198
ESC_LA8062AA	196	257
ESC_LA8061AA	1056	257
ESC_LA8060AA	2	900
ESC_LA8059AA	2811	233
ESC_LA8058AA	9237	938
ESC_LA8057AA	9271	900
ESC_LA8056AA	69	387
ESC_LA8055AA	2811	233
ESC_LA8054AA	657	592
ESC_LA8053AA	967	212
ESC_LA8052AA	7581	996
ESC_LA7735AA	405	923
ESC_LA7734AA	68	386
ESC_LA7467AA	9184	512
ESC_LA7466AA	9184	512
ESC_LA7464AA	10	512
ESC_LA7463AA	10	512
ESC_LA7462AA	9184	512
ESC_LA7461AA	10	512
ESC_LA7460AA	10	512
ESC_LA7459AA	10	512
ESC_LA7458AA	10	512
ESC_LA7457AA	10	512
ESC_LA7456AA	10	512
ESC_LA7455AA	10	512
ESC_LA7454AA	10	512
ESC_LA7453AA	10	512
ESC_LA7452AA	10	512
ESC_LA7451AA	10	512
ESC_LA7450AA	10	512
ESC_LA7449AA	10	512
ESC_LA7448AA	10	512
ESC_LA7447AA	10	512
ESC_LA7446AA	10	512
ESC_LA7445AA	8906	512
ESC_LA7444AA	10	512
ESC_LA7443AA	10	512
ESC_LA7442AA	10	512

ESC_LA7441AA	10	512
ESC_LA7439AA	10	512
ESC_LA7438AA	10	512
ESC_LA7437AA	9158	512
ESC_LA7436AA	9158	512
ESC_LA7435AA	10	512
ESC_LA7434AA	21	156
ESC_LA7433AA	21	156
ESC_LA7432AA	131	653
ESC_LA7431AA	14	761
ESC_LA7430AA	69	387
ESC_LA7429AA	131	653
ESC_LA7428AA	73	737
ESC_LA7427AA	69	387
ESC_LA7426AA	131	653
ESC_LA7425AA	131	653
ESC_LA7424AA	744	512
ESC_LA7423AA	127	738
ESC_LA7422AA	372	424
ESC_LA7421AA	398	445
ESC_LA7420AA	131	653
ESC_LA7419AA	69	387
ESC_LA7418AA	127	738
ESC_LA7417AA	648	359
ESC_LA7416AA	127	738
ESC_LA7415AA	95	738
ESC_LA7414AA	224	198
ESC_LA7413AA	131	653
ESC_LA7412AA	69	387
ESC_LA7411AA	4713	653
ESC_LA7410AA	420	741
ESC_LA7409AA	95	738
ESC_LA7408AA	131	653
ESC_LA7407AA	131	653
ESC_LA7406AA	648	359
ESC_LA7405AA	12	626
ESC_LA7404AA	167	512
ESC_LA7403AA	73	737
ESC_LA7402AA	131	653
ESC_LA7401AA	131	653
ESC_LA7400AA	131	653
ESC_LA7399AA	69	387
ESC_LA7398AA	73	737
ESC_LA7397AA	95	738
ESC_LA7396AA	95	738
ESC_LA7395AA	95	738
ESC_LA7394AA	12	626
ESC_LA7393AA	127	738
ESC_LA7392AA	131	653
ESC_LA7391AA	405	923

ESC_LA7390AA	2005	175
ESC_LA7389AA	58	198
ESC_LA7385AA	93	71
ESC_LA7384AA	93	71
ESC_LA7383AA	93	71
ESC_LA7382AA	93	71
ESC_LA7381AA	636	653
ESC_LA7342AA	10	512
ESC_LA7341AA	10	512
ESC_LA7340AA	10	512
ESC_LA7339AA	10	512
ESC_LA7338AA	10	512
ESC_LA7337AA	10	512
ESC_LA7336AA	10	512
ESC_LA7335AA	10	512
ESC_LA7334AA	10	512
ESC_LA7333AA	10	512
ESC_LA7332AA	10	512
ESC_LA7331AA	10	512
ESC_LA7330AA	10	512
ESC_LA7329AA	10	512
ESC_LA7328AA	17	191
ESC_LA7327AA	17	191
ESC_LA7319AA	11	936
ESC_LA7318AA	11	936
ESC_LA7317AA	21	156
ESC_LA7314AA	16	156
ESC_LA7312AA	21	156
ESC_LA7311AA	156	175
ESC_LA7310AA	1611	198
ESC_LA7309AA	8907	234
ESC_LA7308AA	17	191
ESC_LA7303AA	21	156
ESC_LA7302AA	21	156
ESC_LA7301AA	21	156
ESC_LA7300AA	21	156
ESC_LA7299AA	21	156
ESC_LA7298AA	29	156
ESC_LA7297AA	29	156
ESC_LA7296AA	29	156
ESC_LA7295AA	21	156
ESC_LA7294AA	21	156
ESC_LA7293AA	21	156
ESC_LA7292AA	21	156
ESC_LA7291AA	21	156
ESC_LA7290AA	21	156
ESC_LA7289AA	21	156
ESC_LA7288AA	21	156
ESC_LA7287AA	21	156
ESC_LA7286AA	21	156

ESC_LA7285AA	21	156
ESC_LA7284AA	21	156
ESC_LA7283AA	21	156
ESC_LA7282AA	21	156
ESC_LA7281AA	21	156
ESC_LA7280AA	21	156
ESC_LA7279AA	21	156
ESC_LA7278AA	21	156
ESC_LA7277AA	21	156
ESC_LA7276AA	21	156
ESC_LA7275AA	21	156
ESC_LA7274AA	21	156
ESC_LA7273AA	21	156
ESC_LA7272AA	21	156
ESC_LA7271AA	29	156
ESC_LA7270AA	21	156
ESC_LA7269AA	21	156
ESC_LA7268AA	21	156
ESC_LA7267AA	21	156
ESC_LA7266AA	21	156
ESC_LA7265AA	21	156
ESC_LA7264AA	21	156
ESC_LA7263AA	21	156
ESC_LA7262AA	21	156
ESC_LA7261AA	21	156
ESC_LA7260AA	21	156
ESC_LA7259AA	21	156
ESC_LA7258AA	29	156
ESC_LA7257AA	21	156
ESC_LA7256AA	21	156
ESC_LA7255AA	21	156
ESC_LA7254AA	21	156
ESC_LA7253AA	21	156
ESC_LA7252AA	21	156
ESC_LA7251AA	21	156
ESC_LA7250AA	21	156
ESC_LA7249AA	21	156
ESC_LA7248AA	21	156
ESC_LA7247AA	21	156
ESC_LA7246AA	8908	156
ESC_LA7245AA	21	156
ESC_LA7244AA	21	156
ESC_LA7243AA	21	156
ESC_LA7242AA	21	156
ESC_LA7241AA	21	156
ESC_LA7240AA	21	156
ESC_LA7239AA	21	156
ESC_LA7238AA	21	156
ESC_LA7237AA	21	156
ESC_LA7236AA	21	156

ESC_LA7235AA	21	156
ESC_LA7234AA	21	156
ESC_LA7233AA	21	156
ESC_LA7232AA	21	156
ESC_LA7231AA	21	156
ESC_LA7230AA	21	156
ESC_LA7229AA	21	156
ESC_LA7228AA	21	156
ESC_LA7227AA	21	156
ESC_LA7226AA	21	156
ESC_LA7225AA	21	156
ESC_LA7224AA	21	156
ESC_LA7223AA	21	156
ESC_LA7222AA	21	156
ESC_LA7221AA	21	156
ESC_LA7220AA	21	156
ESC_LA7219AA	21	156
ESC_LA7218AA	21	156
ESC_LA7217AA	21	156
ESC_LA7216AA	21	156
ESC_LA7215AA	29	156
ESC_LA7214AA	21	156
ESC_LA7213AA	21	156
ESC_LA7212AA	21	156
ESC_LA7211AA	21	156
ESC_LA7210AA	21	156
ESC_LA7209AA	21	156
ESC_LA7208AA	21	156
ESC_LA7207AA	21	156
ESC_LA7206AA	21	156
ESC_LA7205AA	29	156
ESC_LA7204AA	21	156
ESC_LA7203AA	21	156
ESC_LA7202AA	21	156
ESC_LA7201AA	29	156
ESC_LA7200AA	21	156
ESC_LA7199AA	21	156
ESC_LA7198AA	29	156
ESC_LA7197AA	21	156
ESC_LA7196AA	29	156
ESC_LA7195AA	21	156
ESC_LA7194AA	29	156
ESC_LA7193AA	29	156
ESC_LA7192AA	21	156
ESC_LA7191AA	21	156
ESC_LA7190AA	21	156
ESC_LA7189AA	21	156
ESC_LA7188AA	21	156
ESC_LA7187AA	21	156
ESC_LA7186AA	21	156

ESC_LA7185AA	21	156
ESC_LA7184AA	21	156
ESC_LA7183AA	21	156
ESC_LA7182AA	21	156
ESC_LA7181AA	21	156
ESC_LA7180AA	29	156
ESC_LA7179AA	29	156
ESC_LA7178AA	21	156
ESC_LA7177AA	21	156
ESC_LA7176AA	21	156
ESC_LA7175AA	21	156
ESC_LA7174AA	21	156
ESC_LA7173AA	21	156
ESC_LA7172AA	21	156
ESC_LA7171AA	21	156
ESC_LA7170AA	21	156
ESC_LA7169AA	21	156
ESC_LA7168AA	21	156
ESC_LA7167AA	21	156
ESC_LA7166AA	21	156
ESC_LA7165AA	29	156
ESC_LA7164AA	21	156
ESC_LA7163AA	21	156
ESC_LA7162AA	21	156
ESC_LA7161AA	21	156
ESC_LA7160AA	21	156
ESC_LA7159AA	21	156
ESC_LA7158AA	21	156
ESC_LA7157AA	21	156
ESC_LA7156AA	21	156
ESC_LA7155AA	21	156
ESC_LA7154AA	21	156
ESC_LA7153AA	21	156
ESC_LA7152AA	21	156
ESC_LA7151AA	21	156
ESC_LA7150AA	10	512
ESC_LA7149AA	1	667
ESC_LA7148AA	21	156
ESC_LA7147AA	155	175
ESC_LA7146AA	56	198
ESC_LA7145AA	2165	257
ESC_LA7144AA	993	789
ESC_LA7143AA	1148	257
ESC_LA7142AA	155	175
ESC_LA7141AA	993	789
ESC_LA7140AA	443	257
ESC_LA7139AA	679	257
ESC_LA7138AA	3630	71
ESC_LA7137AA	10	512
ESC_LA7136AA	710	291

ESC_LA7135AA	155	175
ESC_LA7134AA	641	935
ESC_LA7133AA	993	789
ESC_LA7132AA	155	175
ESC_LA7131AA	679	257
ESC_LA7130AA	155	175
ESC_LA7129AA	155	175
ESC_LA7127AA	2514	504
ESC_LA7126AA	155	175
ESC_LA7125AA	155	175
ESC_LA7124AA	155	175
ESC_LA7123AA	5133	504
ESC_LA7122AA	5285	108
ESC_LA7121AA	5285	108
ESC_LA7120AA	9185	900
ESC_LA7119AA	155	175
ESC_LA7118AA	10	512
ESC_LA7116AA	155	175
ESC_LA7114AA	93	71
ESC_LA7113AA	93	71
ESC_LA7106AA	10	512
ESC_LA7105AA	10	512
ESC_LA7104AA	10	512
ESC_LA7103AA	311	204
ESC_LA7102AA	6	445
ESC_LA7101AA	6	445
ESC_LA7100AA	6	445
ESC_LA7098AA	165	504
ESC_LA7097AA	8909	160
ESC_LA7096AA	117	592
ESC_LA7095AA	359	696
ESC_LA7094AA	6756	962
ESC_LA7093AA	359	696
ESC_LA7092AA	359	696
ESC_LA7091AA	648	359
ESC_LA7090AA	156	175
ESC_LA7089AA	167	512
ESC_LA7088AA	4660	519
ESC_LA7086AA	10	512
ESC_LA7085AA	9159	512
ESC_LA7084AA	9159	512
ESC_LA7083AA	117	592
ESC_LA7082AA	88	160
ESC_LA7081AA	162	924
ESC_LA7080AA	351	268
ESC_LA7079AA	399	198
ESC_LA7078AA	5996	512
ESC_LA7077AA	117	592
ESC_LA7076AA	117	592
ESC_LA7075AA	117	592

ESC_LA7074AA	57	50
ESC_LA7073AA	57	50
ESC_LA7072AA	38	669
ESC_LA7071AA	1914	956
ESC_LA7070AA	949	175
ESC_LA7069AA	212	198
ESC_LA7068AA	117	592
ESC_LA7067AA	1914	956
ESC_LA7066AA	155	175
ESC_LA7065AA	23	156
ESC_LA7064AA	43	512
ESC_LA7063AA	429	654
ESC_LA7062AA	48	76
ESC_LA7061AA	3107	512
ESC_LA7060AA	117	592
ESC_LA7059AA	117	592
ESC_LA7058AA	1434	512
ESC_LA7057AA	8911	71
ESC_LA7056AA	641	935
ESC_LA7055AA	355	764
ESC_LA7054AA	117	592
ESC_LA7053AA	117	592
ESC_LA7052AA	3202	71
ESC_LA7051AA	176	601
ESC_LA7050AA	88	160
ESC_LA7049AA	23	156
ESC_LA7048AA	117	592
ESC_LA7047AA	57	50
ESC_LA7046AA	117	592
ESC_LA7045AA	117	592
ESC_LA7044AA	117	592
ESC_LA7043AA	88	160
ESC_LA7042AA	635	141
ESC_LA7041AA	540	286
ESC_LA7040AA	2522	198
ESC_LA7038AA	8910	93
ESC_LA7037AA	2522	198
ESC_LA7036AA	607	355
ESC_LA7035AA	162	924
ESC_LA7034AA	540	286
ESC_LA7033AA	756	257
ESC_LA7032AA	541	701
ESC_LA7031AA	117	592
ESC_LA7030AA	399	198
ESC_LA7029AA	409	504
ESC_LA7028AA	93	71
ESC_LA7027AA	1844	198
ESC_LA7026AA	10	512
ESC_LA7025AA	117	592
ESC_LA7024AA	69	387

ESC_LA7023AA	10	512
ESC_LA7022AA	101	696
ESC_LA7021AA	117	592
ESC_LA7020AA	23	156
ESC_LA7019AA	1141	512
ESC_LA7018AA	117	592
ESC_LA7017AA	117	592
ESC_LA7016AA	973	968
ESC_LA7015AA	295	160
ESC_LA7014AA	538	629
ESC_LA7013AA	58	198
ESC_LA7012AA	351	268
ESC_LA7011AA	101	696
ESC_LA7010AA	1618	736
ESC_LA7009AA	297	234
ESC_LA7008AA	399	198
ESC_LA7007AA	23	156
ESC_LA7005AA	399	198
ESC_LA7004AA	401	142
ESC_LA7003AA	401	142
ESC_LA7002AA	297	234
ESC_LA7001AA	5378	197
ESC_LA7000AA	101	696
ESC_LA6999AA	117	592
ESC_LA6998AA	399	198
ESC_LA6997AA	117	592
ESC_LA6996AA	117	592
ESC_LA6995AA	23	156
ESC_LA6994AA	1079	235
ESC_LA6993AA	117	592
ESC_LA6992AA	3190	221
ESC_LA6991AA	10	512
ESC_LA6990AA	399	198
ESC_LA6989AA	23	156
ESC_LA6988AA	399	198
ESC_LA6987AA	162	924
ESC_LA6986AA	297	234
ESC_LA6985AA	117	592
ESC_LA6984AA	23	156
ESC_LA6983AA	101	696
ESC_LA6982AA	69	387
ESC_LA6981AA	46	447
ESC_LA6980AA	540	286
ESC_LA6979AA	46	447
ESC_LA6978AA	1721	311
ESC_LA6977AA	176	601
ESC_LA6976AA	155	175
ESC_LA6975AA	1056	257
ESC_LA6974AA	5996	512
ESC_LA6973AA	428	654

ESC_LA6972AA	176	601
ESC_LA6971AA	1463	263
ESC_LA6970AA	10	512
ESC_LA6969AA	23	156
ESC_LA6968AA	641	935
ESC_LA6967AA	1721	311
ESC_LA6966AA	3580	257
ESC_LA6964AA	69	387
ESC_LA6963AA	23	156
ESC_LA6962AA	88	160
ESC_LA6961AA	69	387
ESC_LA6960AA	998	644
ESC_LA6959AA	95	738
ESC_LA6958AA	73	737
ESC_LA6957AA	141	644
ESC_LA6956AA	416	738
ESC_LA6955AA	101	696
ESC_LA6954AA	93	71
ESC_LA6953AA	80	731
ESC_LA6952AA	69	387
ESC_LA6951AA	58	198
ESC_LA6950AA	57	50
ESC_LA6949AA	117	592
ESC_LA6948AA	5378	197
ESC_LA6947AA	117	592
ESC_LA6946AA	117	592
ESC_LA6945AA	117	592
ESC_LA6944AA	88	160
ESC_LA6943AA	117	592
ESC_LA6942AA	349	670
ESC_LA6941AA	38	669
ESC_LA6940AA	1721	311
ESC_LA6939AA	1721	311
ESC_LA6938AA	101	696
ESC_LA6937AA	1429	198
ESC_LA6936AA	117	592
ESC_LA6935AA	117	592
ESC_LA6934AA	134	738
ESC_LA6933AA	362	234
ESC_LA6932AA	6016	71
ESC_LA6931AA	95	738
ESC_LA6930AA	23	156
ESC_LA6929AA	34	504
ESC_LA6928AA	181	311
ESC_LA6927AA	88	160
ESC_LA6926AA	2522	198
ESC_LA6925AA	2485	212
ESC_LA6924AA	541	701
ESC_LA6923AA	399	198
ESC_LA6922AA	10	512

ESC_LA6921AA	428	654
ESC_LA6920AA	95	738
ESC_LA6919AA	73	737
ESC_LA6918AA	10	512
ESC_LA6917AA	10	512
ESC_LA6916AA	69	387
ESC_LA6915AA	95	738
ESC_LA6914AA	393	343
ESC_LA6913AA	345	175
ESC_LA6912AA	300	286
ESC_LA6911AA	95	738
ESC_LA6910AA	540	286
ESC_LA6909AA	58	198
ESC_LA6908AA	409	504
ESC_LA6907AA	10	512
ESC_LA6906AA	1163	579
ESC_LA6905AA	117	592
ESC_LA6904AA	162	924
ESC_LA6903AA	1618	736
ESC_LA6902AA	1844	198
ESC_LA6901AA	1582	198
ESC_LA6900AA	540	286
ESC_LA6899AA	43	512
ESC_LA6898AA	5366	355
ESC_LA6897AA	3580	257
ESC_LA6896AA	155	175
ESC_LA6895AA	297	234
ESC_LA6894AA	135	653
ESC_LA6893AA	117	592
ESC_LA6892AA	5995	504
ESC_LA6891AA	3721	93
ESC_LA6890AA	5459	96
ESC_LA6889AA	101	696
ESC_LA6888AA	10	512
ESC_LA6887AA	58	198
ESC_LA6886AA	58	198
ESC_LA6885AA	46	447
ESC_LA6884AA	410	156
ESC_LA6883AA	58	198
ESC_LA6882AA	533	198
ESC_LA6881AA	176	601
ESC_LA6880AA	93	71
ESC_LA6879AA	5940	555
ESC_LA6878AA	295	160
ESC_LA6877AA	101	696
ESC_LA6876AA	536	141
ESC_LA6875AA	46	447
ESC_LA6874AA	48	76
ESC_LA6873AA	135	653
ESC_LA6872AA	345	175

ESC_LA6871AA	10	512
ESC_LA6870AA	23	156
ESC_LA6869AA	355	764
ESC_LA6868AA	349	670
ESC_LA6867AA	410	156
ESC_LA6866AA	155	175
ESC_LA6865AA	410	156
ESC_LA6864AA	88	160
ESC_LA6863AA	75	257
ESC_LA6862AA	69	387
ESC_LA6861AA	2792	886
ESC_LA6860AA	12	626
ESC_LA6859AA	73	737
ESC_LA6858AA	95	738
ESC_LA6857AA	95	738
ESC_LA6856AA	372	424
ESC_LA6854AA	404	761
ESC_LA6853AA	8912	387
ESC_LA6852AA	538	629
ESC_LA6851AA	12	626
ESC_LA6850AA	95	738
ESC_LA6849AA	95	738
ESC_LA6847AA	127	738
ESC_LA6846AA	2015	684
ESC_LA6845AA	73	737
ESC_LA6844AA	127	738
ESC_LA6843AA	12	626
ESC_LA6842AA	73	737
ESC_LA6841AA	69	387
ESC_LA6840AA	1262	737
ESC_LA6839AA	442	257
ESC_LA6838AA	6072	198
ESC_LA6837AA	117	592
ESC_LA6836AA	58	198
ESC_LA6835AA	362	234
ESC_LA6834AA	23	156
ESC_LA6833AA	23	156
ESC_LA6832AA	1163	579
ESC_LA6831AA	23	156
ESC_LA6830AA	6072	198
ESC_LA6829AA	23	156
ESC_LA6828AA	10	512
ESC_LA6827AA	616	156
ESC_LA6826AA	398	445
ESC_LA6825AA	399	198
ESC_LA6824AA	10	512
ESC_LA6823AA	88	160
ESC_LA6822AA	515	1004
ESC_LA6821AA	117	592
ESC_LA6820AA	58	198
[truncated: 161,118 more chars]
